# Site-Selective Copper(I)-Catalyzed Hydrogenation of Amides

**DOI:** 10.1021/jacs.4c14174

**Published:** 2025-01-03

**Authors:** Dimitrios-Ioannis Tzaras, Mahadeb Gorai, Thomas Jacquemin, Thiemo Arndt, Birte M. Zimmermann, Martin Breugst, Johannes F. Teichert

**Affiliations:** Institut für Chemie, Technische Universität Chemnitz, Straße der Nationen 62, 09111 Chemnitz, Germany

## Abstract

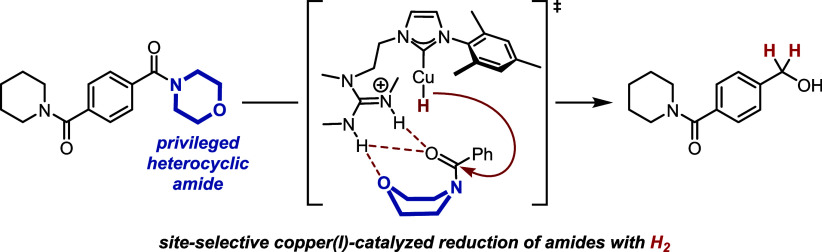

We present a bifunctional catalyst consisting of a copper(I)/N-heterocyclic carbene and an organocatalytic guanidine moiety that enables, for the first time, a copper(I)-catalyzed reduction of amides with H_2_ as the terminal reducing agent. The guanidine allows for reactivity tuning of the originally weakly nucleophilic copper(I) hydrides – formed in situ – to be able to react with difficult-to-reduce amides. Additionally, the guanidine moiety is key for the selective recognition of “privileged” amides based on simple and readily available heterocycles in the presence of other amides within one molecule, giving rise to hitherto unknown site-selective catalytic amide hydrogenation. A substrate scope, mechanistic investigations, and a working hypothesis supported by computational analysis for site-selectivity are presented.

## Introduction

The chemoselective distinction between one of the several (almost) identical functional groups within one molecule, coined site-selectivity, is a key challenge for the development of efficient synthetic methods.^1−4^ Synthetically, several selective reducing agents are available for the reduction of *different* functional groups *in the presence of each other* (e.g., the selective reduction of imines in the presence of ketones).^5,6^ As an example of such a process, the reduction of ketones in the presence of more reactive aldehydes has been reported.^7,8^ However, the distinction between several *structurally closely related* functional groups (e.g., two amides) within the same molecule in a site-selective process is challenging to realize.^9−11^

**Scheme 1:**
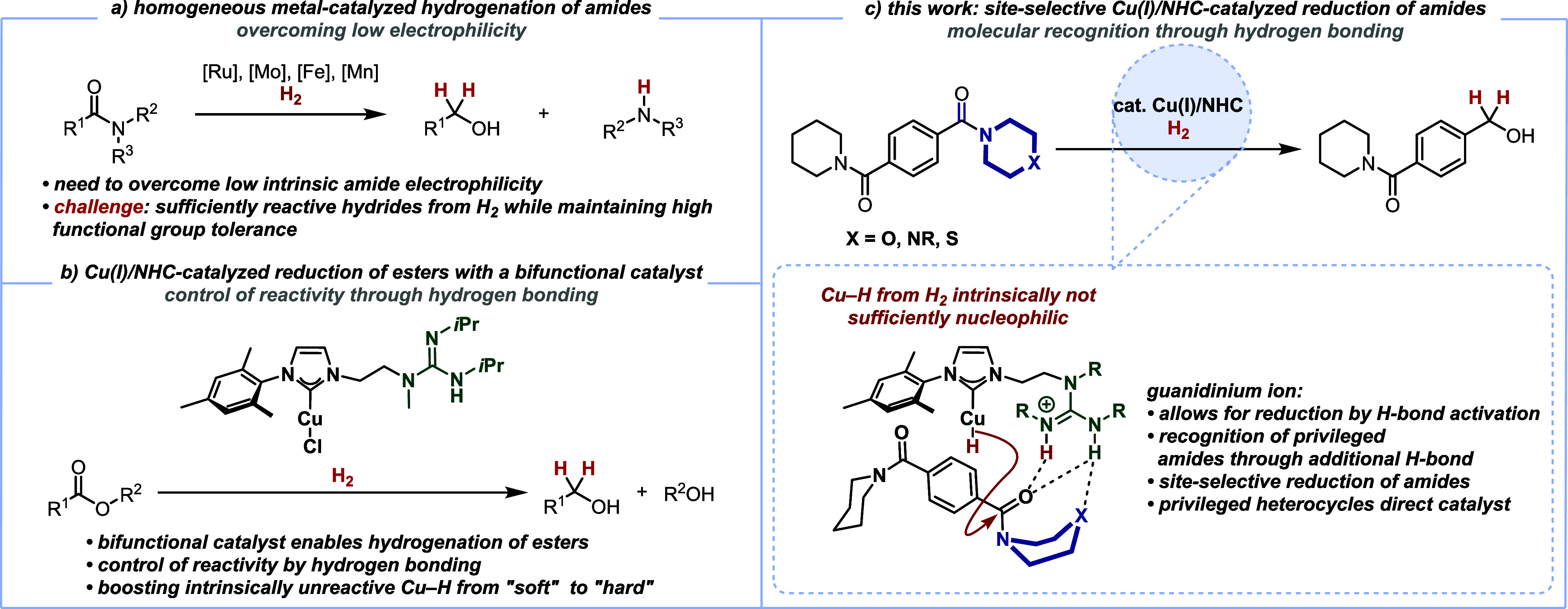
Developing Bifunctional Catalysts for Site-Selective Amide Reduction a) Overview of homogeneous hydrogenation of amides; b) catalytic ester hydrogenation with a bifunctional copper(I) catalyst – control of reactivity; c) concept: the guanidine subunit allows for molecular recognition of a privileged amide in the presence of other amides – control of site-selectivity.

A site-selective reduction of amides poses a formidable challenge for method development, as generally highly nucleophilic hydrides (such as LiAlH_4_) are required. This high reactivity hampers the potential distinction between two (similar) amides. In this vein, in homogeneous catalysis, transition metal-based catalysts for the reduction of amides with H_2_ have gained significant attention in recent years (Scheme 1a).^12−19^ The main focus of these studies was to realize amide reductions with H_2_ in the first place, rather than to achieve any distinction between multiple reactive carboxyl derivatives within one molecule.

We have recently reported on a concept for tuning the reactivity of weakly nucleophilic copper(I) hydride complexes in the presence of a second organocatalytic unit within the same catalyst.^20^ Importantly, copper(I) hydride complexes are not sufficiently nucleophilic by themselves to attack carboxyl groups,^21−23^ and only through the presence of the organocatalyst can an ester reduction with H_2_ as a terminal reducing agent be realized (Scheme 1b). In this design, we have employed a bifunctional copper(I)/N-heterocyclic carbene (NHC) complex^24−27^ bearing a covalently attached guanidine moiety^20,28^ to catalytically reduce esters (“hard” electrophiles) with formally “soft” nucleophilic copper(I) hydrides. We proposed a key activation of the ester substrate through hydrogen bond activation with an intermediately formed guanidinium ion.^20^ Importantly, the key activation of the hydride nucleophiles is brought about only in the proximity of the substrate.

Therefore, this coordination-controlled tuning of *reactivity* bears the potential for broad functional group tolerance. Our approach conceptually contrasts with the control of *regioselectivity* through precoordination by noncovalent interactions in transition metal catalysis.^29,30^

We hypothesized that, in addition to substrate activation (and thus the alteration of *reactivity*), the guanidine moiety in the bifunctional catalysts might also be exploited for the control of *selectivity.*(31) As such, the reactivity of the copper(I)/NHC subunit to generate nucleophilic hydrides from H_2_ would remain,^32^ whereas the guanidine would also affect *substrate recognition* (Scheme 1c).^33−35^ This would allow, for the first time, the distinction of one out of several amides within one molecule, exploiting the hydrogen-bonding abilities of guanidinium ions.^36−40^ Ideally, the proposed recognition mechanism would allow the distinction between several structurally closely related yet synthetically well-accessible amides.

We herein report a site-selective reduction of amides with catalytically generated copper(I) hydrides from H_2_ that allows for the molecular recognition of several privileged amides in the presence of other amides. In addition, in terms of reactivity alone, the reduction of amides lies well beyond any known transformation with nucleophilic copper(I) hydrides.^21−23^

## Results and Discussion

**Scheme 2:**
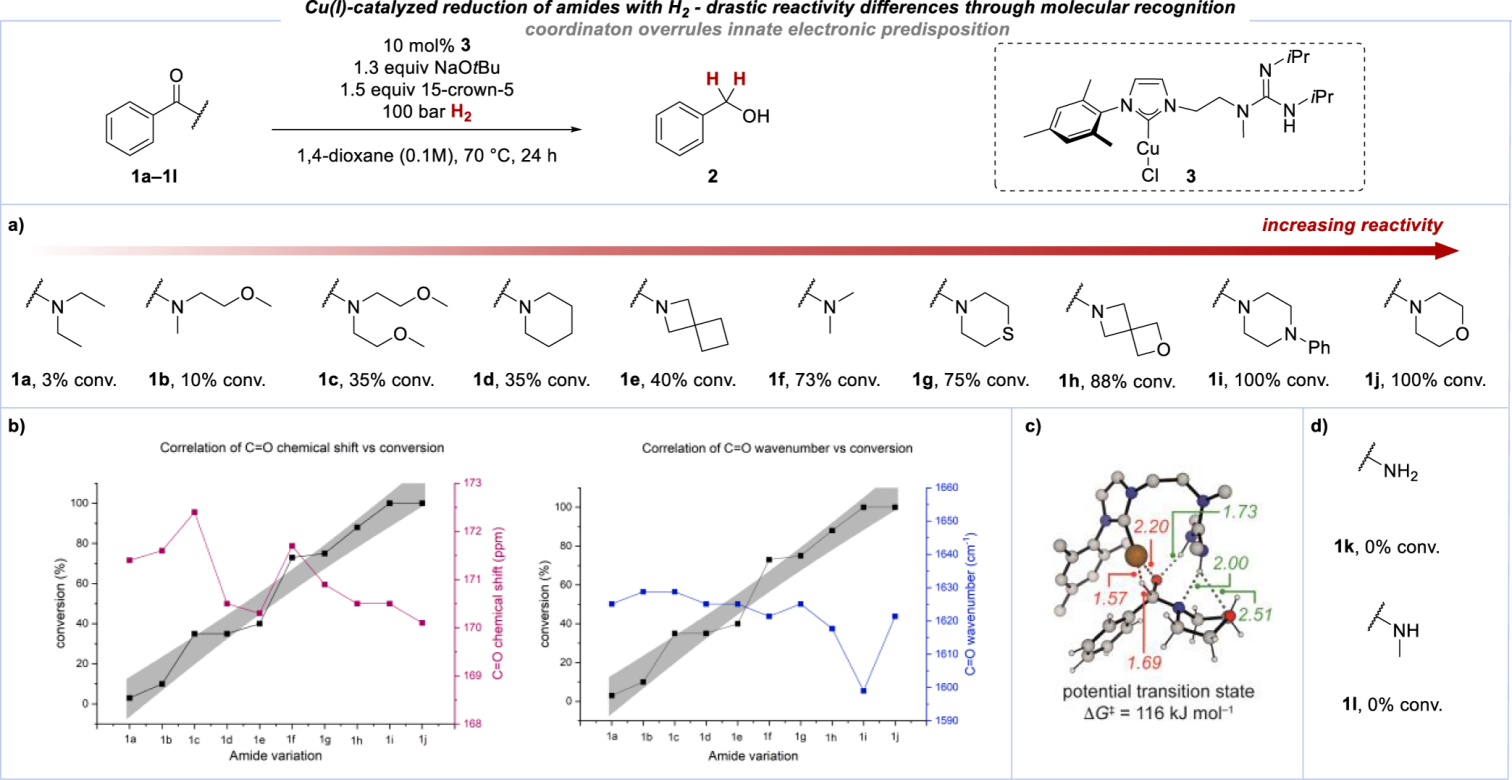
Privileged Amides in Catalytic Hydrogenation:^a,b^ a) Influence of Amine Moieties; b) Correlation of Electronic Predisposition of Amides **1a**–**1j** vs Conversion; c) Hypothesized Transition State with Additional Hydrogen Bonding^c^; d) Failed Substrates All reactions were performed with 0.1 mmol of the corresponding benzamides **1a**–**1l**. Conversion was determined by GC and GC/MS analysis and/or ^1^H NMR analysis. Calculated transition state with selected bond lengths (distances are given in Å, DSD-BLYP-D3(BJ)/CBS/SMD(1,4-dioxane)//M06-L/6-31+G(d,p), SDD for Cu).

As an initial finding, we recognized the strongly varying reactivity of structurally closely related benzamides **1** with the bifunctional copper(I)/NHC-guanidine catalyst **3**(20) (Scheme 2a). We found that benzamides **1a**–**1e**, derived from simple linear or cyclic amines, did not give high conversions of **1** (3–40%). In contrast, we found a stark rise in reactivity (75%–100% conversion) with heterocycle-derived amides **1g**–**1j**. These results are remarkable, as the corresponding structurally closely related heteroatom-devoid piperidine amide **1d** led only to 35% conversion. In order to explain this significant difference in reactivity, we attempted to correlate the electronic nature of the amide carboxyl carbon atoms to the experimental findings (Scheme 2b). More reduction to benzyl alcohol (**2**) could be expected with more electron-poor amides, leading to higher electrophilicity. However, no satisfactory correlation of ^13^C NMR shifts or C=O stretching frequencies (both probes for the electron density at the carboxyl carbon atom^41,42^) of amides **1a**–**1j** with the conversion was found. Furthermore, the ranking of the respective amides by nucleophilicity parameters in the nitrogen atom^43,44^ did not lead to a clear explanation for the observed reactivity.^45^

The key structural difference between the “non-privileged” piperidine derivative **1d** and the “privileged” morpholine or piperazine (**1j** and **1i**, respectively) is the replacement of a methylene unit with a heteroatom. Therefore, we sought another possible explanation for the detected reactivity differences: as a working hypothesis, we put forward an additional H-bonding interaction between the guanidinium ion and the heteroatom of the amide backbone (present after heterolytic cleavage of the H–H bond by the copper(I) catalyst;^32,46−48^ compare Scheme 1c). A similar coordination mode had been proposed for an O···Li^+^ interaction.^49^ Supporting our hypothesis, we carried out NMR titrations with “privileged” vs nonprivileged amides employing the *N*-methyl group of a guanidinium derivative of **3** as a probe. In these experiments, we can observe a change in the ^1^H NMR chemical shift only in the case of the “privileged” morpholine amide **1j** and not with the structurally closely related piperidine derivative **1d**.^45^

To test the feasibility of the proposed dual hydrogen-bond coordination to the carbonyl group and the heteroatom within the amine part of the amide starting materials **1**, orienting DFT calculations have been performed.^50^ The calculations rely on the proposed copper hydride analogue of **3** and the free amide **1j** as a reference. The computational results reveal that a transition state featuring the proposed additional hydrogen bond (see Scheme 2c) is associated with a barrier of 116 kJ mol^–1^ which can be easily overcome under the reaction conditions (70 °C in 1,4-dioxane, 100 bar of H_2_). A natural population analysis further indicated that the N–H···O hydrogen bond is stabilizing the transition state by approximately 5 kJ mol^–1^. Such a dual coordination mode could explain the enhanced reactivity of the formed copper(I) hydride complex with heteroatom-containing amides **1g**–**1j**: the reactivity is boosted in the cases of the privileged amides by effecting both carboxyl activation through hydrogen bonding^20^ and placement of the nucleophilic hydrides in close proximity to the respective privileged amide.

**Scheme 3:**
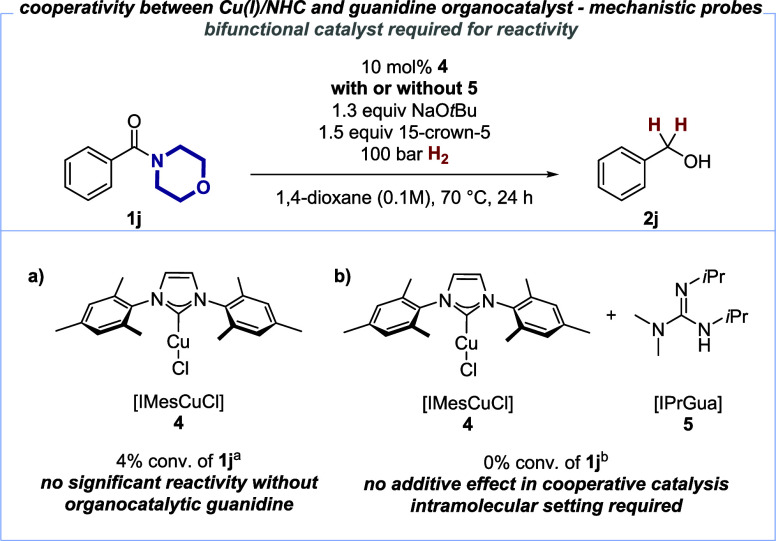
Control Experiments Probing the Necessity for a Bifunctional Catalyst Performed with 0.10 mmol of benzamide **1j**, [IMesCuCl] **4** 10 mol %, NaO*t*Bu 1.3 equiv, 15-crown-5 1.5 equiv in 1,4-dioxane (1.0 mL), 100 bar H_2_, 24 h. Performed with 0.05 mmol of benzamide **1j**, [IMesCuCl] **4** 10 mol %, [IPrGua] **5** 1.1 equiv, NaO*t*Bu 1.3 equiv, 15-crown-5 1.5 equiv in 1,4-dioxane (0.50 mL), 100 bar H_2_, 24 h.

The absence of any reactivity with amides **1k** and **1l** bearing free N–H bonds can be explained by detrimental deprotonation under strongly basic reaction conditions (Scheme 2d). It should be noted that, just as in the previously reported case of ester hydrogenation,^20^ the two catalytic units have to reside within the same catalyst molecule in order to observe any amide reduction with H_2_ at all.^45^ This can be seen from the absence of any significant conversion of **1j** when the two components of the parent copper(I)/NHC complex [IMesCuCl] (**4**), with or without guanidine **5**, were used under otherwise identical conditions (Scheme 3). These two examples underscore the dramatic rate-enhancing effect of the bifunctional catalyst.

The identified privileged amide moieties are based on simple and commercially available heterocycles, such as morpholine and piperazine. Therefore, these privileged amides could be incorporated easily into synthetic routes of more complex molecules, making this approach attractive for selective amide reduction in late-stage functionalization synthesis strategies.

**Scheme 4:**
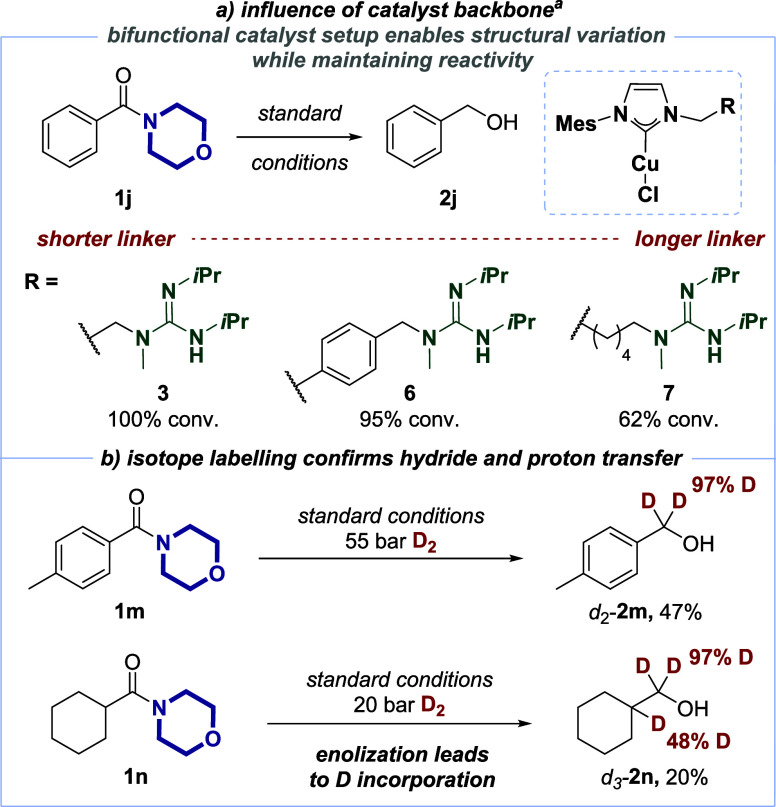
Confounding Experiments on the Influence of the Catalyst Structure (a) and Hydride/Deuteride Transfer Pathway (b) All reactions were performed with 0.1 mmol benzamide **1j**. Conversion was determined by GC, GC/MS, and/or ^1^H NMR analysis.

In order to deepen our understanding of the catalytic amide hydrogenation with copper(I) catalysts, we synthesized bifunctional catalysts **6** and **7** with varied linker lengths between the copper(I)/NHC and the guanidine moieties (Scheme 4a). The introduction of a *para*-disubstituted arene as a linker into **6** should increase the distance between the copper(I)/NHC and the guanidine moiety. Nevertheless, similar reactivity toward the privileged morpholine-derived amide **1j** was found (95% conversion). On the contrary, elongation of the linker length led to a slight decrease in the conversion of **1j** (62%). As can be seen from these results, the catalyst design allows for further functionalization without compromising the overall reactivity in catalytic amide hydrogenations.

Furthermore, to support our working hypothesis of hydride transfer from copper(I) to amides as a key step of the overall reduction, we carried out the catalytic hydrogenation of privileged amides **1m** and **1n** employing D_2_ (Scheme 4b). This gave deuterated benzyl alcohol **2m**-*d*_2_ with very high deuterium incorporation (97% D) in the α-position of the alcohol, supporting the hypothesis of a nucleophilic hydride/deuteride originating from H_2_/D_2_. Privileged alkyl amide **1n** gave rise to similar results in terms of isotope labeling in the α-position of the alcohol *d*_3_-**2n**, along with moderate deuteration (48% D) at the tertiary carbon atom. The latter can be explained by deuteration of an amide enolate under the strongly basic conditions prior to hydride transfer by *t*BuOD.^20,51^ This result also highlights the fate of the proton equivalent after heterolytic cleavage of H_2_/D_2_.^35−37^ The intermediate formation of amide enolates also corroborates the reduced reactivity of alkyl amides in the overall reduction (Table 1, below). Subsequently, we have established that the generally observed chemoselectivity favoring privileged amides remains intact even with unactivated alkyl amides in a series of competition experiments.^45^

**Table 1:**
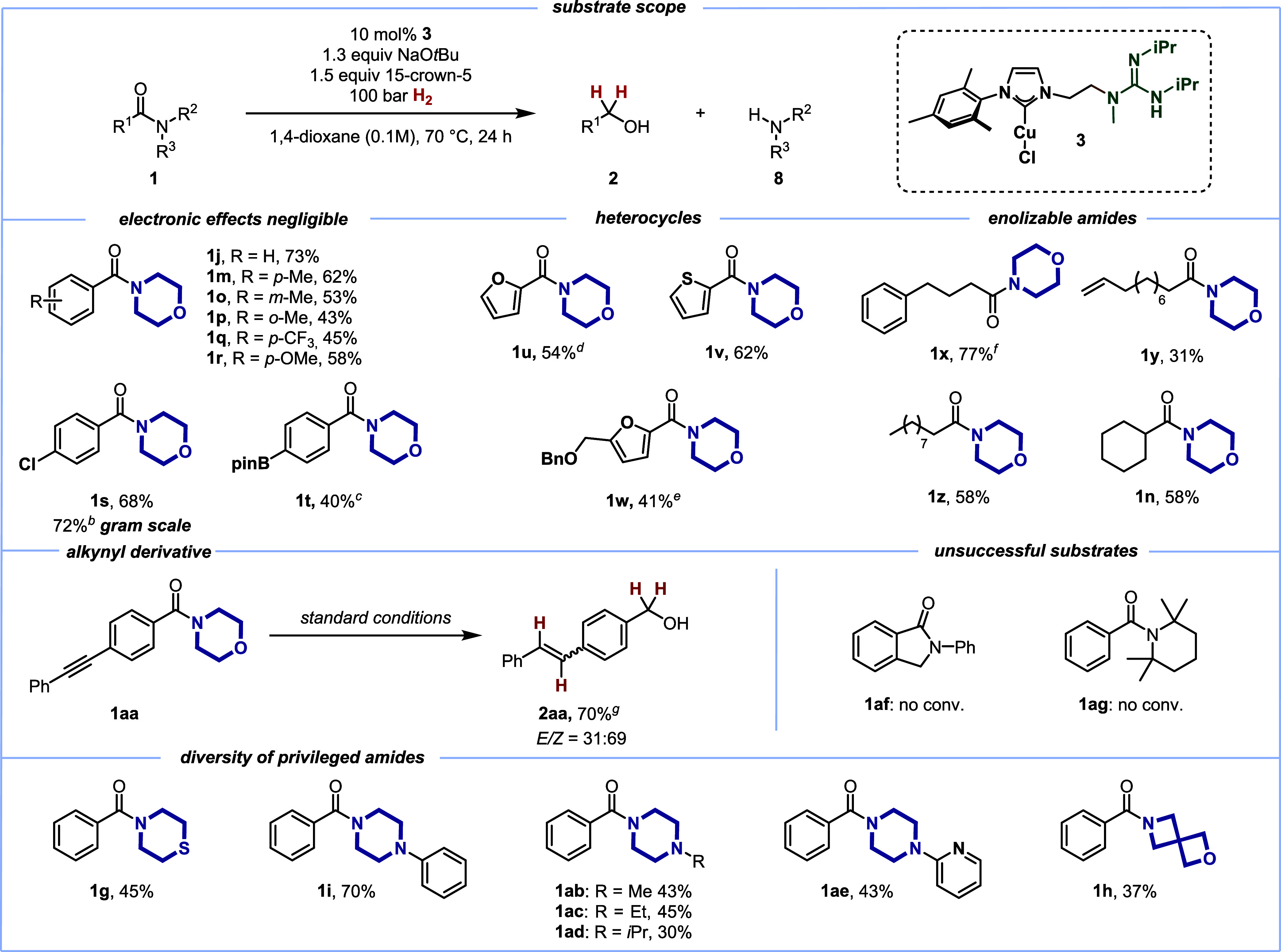
Scope of the Copper(I)-Catalyzed Catalytic Hydrogenation of Amides^a^^b^^c^^d^^e^^f^^g^

a Substrate (0.4 mmol); isolated yields are given.

b Gram scale reaction on a 4.4 mmol scale.

c Isolated as a mixture of **2t**/**2t’** = 60:40; **2t’** corresponds to the protodeborylated benzyl alcohol.

d Volatile product.

e No benzyl deprotection observed.

f With 20 mol % **3**.

g 10% of the corresponding alkane observed.

As of now, no reactivity of copper(I) hydrides with generally unreactive amides has been reported. So, we decided to investigate the scope of the catalytic amide hydrogenation methodology with bifunctional catalyst **3** next (Table 1). For this, we selected the privileged heterocycle-based amides derived from morpholine, piperazine, and thiomorpholine, as identified in Scheme 2.

In this vein, a variety of morpholine-derived benzamides **1j** and **1m**–**1r** could successfully be converted to the corresponding alcohols **2** with H_2_ as the terminal reducing agent, regardless of their electronic nature in the backbone (45% isolated yield for electron-poor trifluoromethylated **1q** vs 58% yield for the electron-rich anisole derivative **1r**). The sterically hindered *ortho*-toluyl derivative **1p** could also be successfully converted. To demonstrate the scalability of the protocol, *para*-chloro benzamide **1s** led to the corresponding alcohol **2s** in good yield (72%) on a gram scale.^52^ The successful reaction of boronic ester derivative **1t** underscores that reactive functional groups are also tolerated by the reaction conditions. Several heterocycles **1u**–**1w**, including the furfural derivative **1w,** could be reduced with moderate overall yields (41–62%).^53^ As alluded to before, potentially enolizable amides **1n** and **1x**–**1z** generally gave somewhat lower yields.

Generally, alkenes are challenging secondary functional groups in catalytic amide reductions^54^ (and carbonyl/carboxyl hydrogenations in general) due to their high propensity for olefin hydrogenation.^55^ In this vein, the tolerance of the terminal alkene in **1y** is particularly noteworthy, as this result underscores that our approach with a bifunctional catalyst allows for a wide substrate scope. At the same time, it features the advantage of NHC-derived copper(I) hydride intermediates in catalytic hydrogenations, as they generally do not react with alkenes.^56−60^ When tolane derivative **1aa**, bearing an internal alkyne, was subjected to the reaction conditions, stilbenyl alcohol **2aa** was found as the major product. This result demonstrates that the commonly known alkyne semihydrogenation^32,58,59,61,62^ occurs at a higher rate than the catalytic amide reduction. In a similar vein, the prototypical reaction of copper hydride complexes is the conjugate reduction of enones or enoates;^21−23^ we have established that this reactivity mode exceeds the amide reduction in terms of reaction rate.^45^

A variety of heterocycle-based amides **1g**, **1i**, **1ab**–**1ae**, and **1h** could successfully be reduced, displaying the potential of the present method for selective hydrogenation of amides in more complex substrates. As a limitation of the present protocol, lactams such as **1af** did not show any reactivity, mirroring lactones in their reluctance to react, as found earlier.^20^ Interestingly, the so-called twisted amide **1ag** did not show any reactivity. This serves as a negative control, as twisted amides have been shown to be highly reactive in catalytic cross-coupling reactions, relating back to the reduced resonance stabilization.^63^ This rules out a possible activation mode of the privileged amides by forced twisting out of conjugation, as effected by the bifunctional catalyst.

**Scheme 5:**
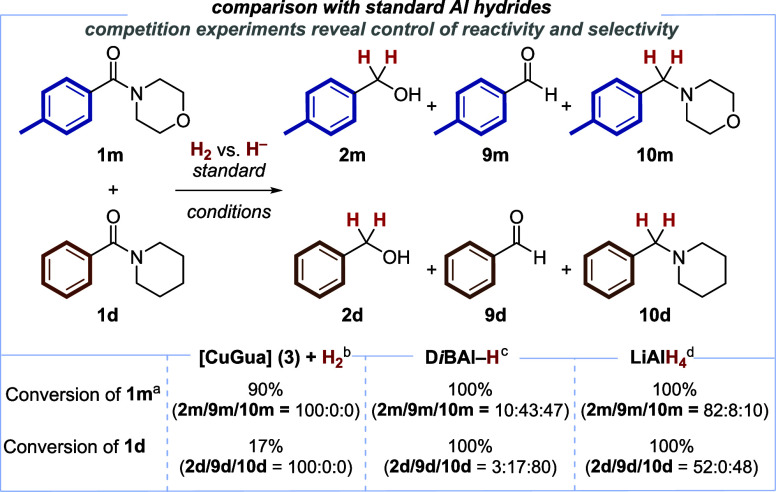
Comparison of Chemoselectivity in Competition Experiments:, Bifunctional Catalyst vs Commonly Employed Stoichiometric Aluminum Hydrides Conversion was determined by GC and GC/MS analysis and/or ^1^H NMR analysis. Substrates 0.20 mmol, [CuGua] **3** 10 mol %, NaO*t*Bu 1.3 equiv, tridecane 10 mol %, 15-crown-5 1.5 equiv in 1,4-dioxane (2.0 mL) for 4 h at 70°C. Substrates 0.30 mmol, D*i*BAl–H (1.0 M in toluene, 4.0 equiv) in dry THF (3.0 mL) for 15 min at rt. Substrates 0.30 mmol, LiAlH_4_ 4.0 equiv in dry THF (3.0 mL) for 15 min at rt.

Typically, stoichiometric metal-based reducing agents such as aluminum hydrides are employed for the reduction of amides to the corresponding amines in a deoxygenative pathway. The present protocol offers a catalytic and highly selective alternative protocol based on H_2_ as the terminal reducing agent with orthogonal reactivity, leading to the corresponding alcohols in a deaminative pathway.^64^ In order to further illustrate this point, a comparison between our catalytic protocol and aluminum hydride reagents was carried out (Scheme 5). In an intermolecular competition experiment, morpholine-derived amide **1m** was reduced in the presence of an equimolar amount of a structurally related piperidine derivative **1d**. The bifunctional catalyst **3** allowed for a selective conversion only to the corresponding alcohols **2**, displaying a remarkable reactivity difference favoring the privileged morpholine **1m** over piperidine **1d** (90% conversion of **1m** vs 17% conversion of **1d**). With D*i*BAl-H and LiAlH_4_, neither chemoselective reductions to alcohols **2m**/**2d** nor amines **10m**/**10d,** nor a significant preference for the reduction of morpholine derivative **1m** over piperidine **1d** was found. These combined experiments demonstrate a remarkably chemoselective catalytic amide reduction with H_2_ based on our protocol.

**Scheme 6:**
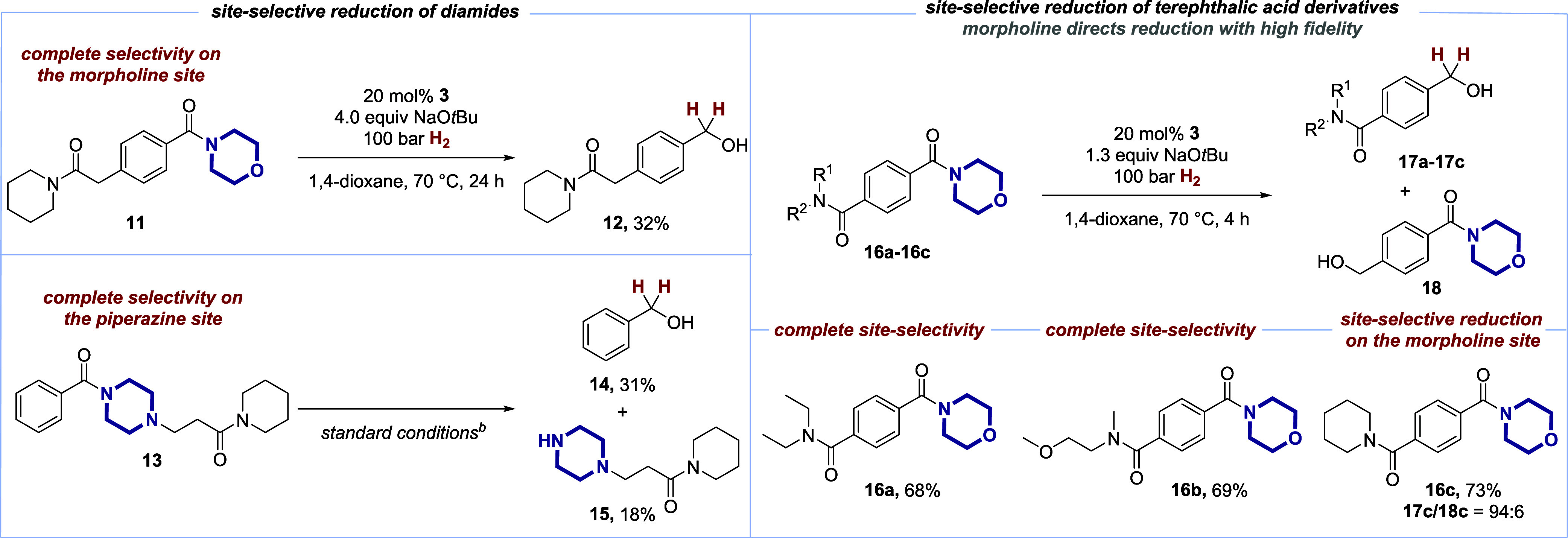
Site-Selective Amide Hydrogenations Substrate (0.2 mmol); isolated yields are given. Substrate (0.3 mmol), [CuGua] **3** 20 mol%, NaO*t*Bu 2.5 equiv, in 1,4-dioxane (3.0 mL), 100 bar H_2_, 24 h.

Emanating from the remarkable chemoselectivity in the reduction of privileged amides based on heterocyclic amines, as depicted in Scheme 2, we wanted to further explore this unique feature of our catalyst **3** by investigating whether this selectivity could be translated into a site-selective catalytic hydrogenation of diamides. Remarkably, when the intramolecular competition between a piperidine-derived and a morpholine-derived amide in **11** was probed, the site-selective reduction of only the privileged morpholine site to give alcohol **12** was observed (Scheme 6). To the best of our knowledge, no such site selectivity has been reported in any other amide reduction methods. Similarly, good results in terms of site-selectivity were found with piperazine derivative **13** as an alternative privileged amide. This could be reduced selectively to give the corresponding free piperazine **15**, while keeping the structurally closely related piperidine amide in **15** intact.^65^

The intramolecular competition between two benzoic acid-derived amides was investigated next when terephthalic acid derivatives **16** were subjected to the site-selective amide hydrogenation protocol. Indeed, also in these cases, the privileged morpholine-derived amides could be reduced to the corresponding alcohols **17**. These reductions took place in the presence of structurally closely related amides based on diethylamine (as in **16a**), an aminoethanol (in **16b**, which could be regarded as the “ring-opened” morpholine), and piperidine **16c** with very high site-selectivity. Only in the latter case, the undesired alcohol **18c** was detected (6%). For all shown examples of site-selective amide reductions in Scheme 6, we also carried out the reductions with both LiAlH_4_ and D*i*BAl–H, which established that neither of these reducing agents could reach either chemo- or site-selectivity as displayed by the bifunctional catalyst.^45^

These results underscore the remarkable capability of the bifunctional catalyst to detect only the privileged heterocyclic amides within a molecule and, subsequently direct the reactivity toward these privileged sites. We believe that the results with the terephthalic acid-derived amides **16** could bear special potential for the selective hydrogenative cleavage of polymeric materials.

## Conclusion

We have demonstrated that the use of a bifunctional catalyst consisting of a copper(I)/NHC moiety to activate H_2_ and generate nucleophilic hydrides, in combination with an organocatalytic guanidine unit, can bring about hitherto unknown reactivity and selectivity in the realm of “copper hydride catalysis”. On the one hand, we could show that, for the first time, poorly electrophilic and thus hard-to-reduce amides could be catalytically converted with H_2_ to the corresponding alcohols by virtue of copper(I) hydrides, generally considered mildly nucleophilic. This reactivity boosting is enabled by the action of the guanidine moiety in the catalyst and, on the whole, gives rise to the first copper(I)-catalyzed amide hydrogenation. Additionally, the guanidine subunit could be exploited for site-selective amide reductions with H_2_ as the terminal reducing agent through molecular recognition via a proposed hydrogen bonding network. In this manner, privileged amides based on heterocycles could be selectively hydrogenated in the presence of structurally closely related amides with unprecedented selectivity.

## Supporting Information Available

Detailed starting material synthesis, general procedures, characterization data, NMR and IR spectra for all compounds, and computational methods (PDF)
NMR, COSY, HMBC, HSQC, HRMS, and IR spectral data for compounds (PDF)
