# Supplementary material for: Site-Selective Copper(I)-Catalyzed Hydrogenation of Amides
Source: J Am Chem Soc. 2025 Jan 3;147(2):1867–74. doi: 10.1021/jacs.4c14174 (PMC11744755; doi:10.1021/jacs.4c14174)
Supplement: Supplementary file 1 — ja4c14174_si_001.pdf [file ja4c14174_si_001.pdf]

Site-Selective Copper(I)-Catalyzed Hydrogenation of Amides

Dimitrios-Ioannis Tzaras, Mahadeb Gorai, Thomas Jacquemin, Thiemo Arndt, Birte M. Zimmermann, Martin Breugst, Johannes F. Teichert*

Institut für Chemie, Technische Universität Chemnitz, Straße der Nationen 62, 09111 Chemnitz, Germany

Supporting Information

Contents

1 General Information ... 4
1.1 Solvents ... 5
1.2 Reactions under H_2 pressure ... 5
1.3 Chemicals ... 5
2 Additional data ... 6
2.1 Additional optimization data ... 6
3 General procedures ... 11
3.1 General procedure 1 – synthesis of amides *via* EDC-mediated amidation (GP1) ... 11
3.2 General procedure 2 – synthesis of amides *via* acyl chlorides (GP2) ... 11
3.3 General procedure 3 – H_2-mediated 1,2-reduction of amides (GP3) ... 12
3.4 General procedure 4 – site-selective 1,2-reduction of diamides (GP4) ... 13
3.5 General procedure 5 – Amide reduction with $LiAlH_4$ (GP5) ... 14
3.6 General procedure 6 – Amide reduction with D*i*BAl–H (GP6) ... 14
4 Experimental details ... 15
4.1 Syntheses of amides ... 15
4.2 Products of Cu(I)-catalyzed reduction of amides with H_2 ... 54
4.3 Additional substrates for the Cu(I) catalyzed reduction of amides with H_2 ... 64
4.4 Site-selective reduction of diamides ... 65
4.5 Additional substrates for the Cu(I) catalyzed reduction of diamides with H_2 part I ... 94
4.6 Additional substrates for the Cu(I) catalyzed reduction of diamides with H_2 part II ... 96
4.7 Gram-scale reaction ... 97
4.8 Isotope labelling experiments ... 98
4.9 Cu(I) catalyzed reduction of amides with H_2 – Mass balance ... 100
4.10 Linker variation and reactivity in Cu(I)-catalyzed reduction of amides with H_2 ... 101
5 Competition experiments ... 116
5.1 Competition experiments / Cu(I)-catalyzed reduction of amides with H_2 ... 116
5.2 Competition experiments reduction of amides using stoichiometric reducing reagents 124
5.3 Potential formation of an aldehyde as intermediate ... 128

5.4 Correlation of conversion to electronic and kinetic parameters of amides 131

6 Computational methods 133

7 NMR titration experiments 143

8 References 147

1 General Information

The numbering of the respective molecules in the main manuscript and supporting information do not necessarily follow IUPAC recommendations and were done at our own discretion to guide the reader.

All reactions were carried out in flame dried glassware under a nitrogen atmosphere using standard Schlenk techniques. Glassware and stir bars contaminated with transition metals were treated with *aqua regia* (conc. HCl/conc. HNO_3 3:1) prior to cleaning. For cleaning, glassware and stir bars were kept in an *iso*-PrOH/KOH bath overnight, rinsed with H_2O, kept in a citric acid/H_2O bath overnight and finally rinsed with deionized H_2O and dried at 120 °C. Solutions and reagents were added with nitrogen-flushed disposable syringes/needles. Solvents were added using glass syringes and stainless-steel needles (stored at 120 °C). Analytical thin layer chromatography (TLC) was performed on silica gel 60 G/UV_{254} polyester sheets (*Macherey-Nagel*). Flash column chromatography was performed on silica gel Davisil LC60A (40-63 µm, pore size 60 Å, *Grace*) using the indicated solvents. NMR spectra were recorded on Avance III 600 (*Bruker*) at the Institute for Chemistry of *Technische Universität Chemnitz.* Chemical shifts (δ) are reported in parts per million (ppm) and are referenced to the residual solvent resonance as the internal standard according to the standard literature.[1,2] Data are reported as follows: chemical shift, multiplicity (br s = broad singlet, s = singlet, d = doublet, t = triplet, q = quartet, quint = quintet, m = multiplet, m_c = centrosymmetric multiplet), coupling constants (Hz), integration and – if possible – atom assignment. The assignment refers to the atom number shown in the corresponding molecule figure and was achieved *via* analysis of 2D NMR spectra (COSY, HMQC, HSQC, HMBC, NOESY). If a distinct assignment was not possible, atoms were marked with "*" and are interchangeable. Melting points (m.p.) were determined using a Melting Point System MP70 (*Mettler Toledo*). Infrared (IR) spectra were recorded on a Cary 630 FT-IR spectrometer equipped with an ATR unit (*Agilent Technologies).* Mass spectra (HRMS) were obtained from the Analytical Facility at the Institute for Chemistry at *Technische Universität Chemnitz* (Bruker timsTOF MS System). The parameters for flash column chromatography are given as „(d × h, A/B = a:b, C, #n–m)", with "d" = column diameter; "h" = filling height; "A/B" = eluent solvents; "a:b" = solvent ratio; "C" = fraction volume and #n–m" = fraction number. Analytical gas chromatography (GC) of reaction mixtures was performed using a gas chromatograph *Agilent* 8890 GC System. The instrument was equipped with an *Agilent* J&W HP-5 GC column (length: 30 m, inner diameter: 0.32 mm, film thickness of the stationary phase: 0.25 µm). The following temperature program was used for the analysis: carrier gas N_2, detector temperature 320 ^{o}C, flow rate 6.0 mL/min, temperature program: 40 ^{o}C start temperature, 20 ^{o}C/min heating rate to 250 ^{o}C and remain for 10 min at 250 ^{o}C. The data was taken with the program OpenLab CDS (*Agilent softwares*).

1.1 Solvents

THF and Et_2O were dried over sodium/benzophenone and distilled under N_2 atmosphere prior to use. Et_3N, CH_2Cl_2 and MeOH were dried over CaH_2 and distilled under N_2 atmosphere prior to use. Solvents (technical grade) for extraction/chromatography (*n*-pentane, cyclohexane, CH_2Cl_2, Et_2O, EtOAc) were distilled under reduced pressure prior to use.

1.2 Reactions under H_2 pressure

All reactions under H_2 pressure were carried out in microwave vials (*Biotage* Microwave), equipped with a magnetic stir bar and a rubber septum in autoclaves BR-100 or BR-300 (including the appropriate heating blocks, *Berghof*). The autoclave was purged with N_2 (3 x 10 bar) before the vials were placed in the autoclave and the septum was pierced under a counter flow of N_2. The autoclave was purged with N_2 (1 x 1 bar, 3 x 5 bar) and H_2 (3 x 20 bar) or D_2 (2 x 5 bar) before the appropriate H_2 or D_2 pressure was applied (pressure is given as initial pressure before heating). The heating block was pre-heated or a cooling bath (EtOH, cryostat) was pre-cooled before the autoclave was placed inside. After the respective reaction time the autoclave was allowed to cool or warm to room temperature and H_2 or D_2 was released. The autoclave was purged with N_2 (3 x 10 bar) before the vials were taken out.

1.3 Chemicals

All reagents were purchased from established commercial suppliers (*Sigma-Aldrich, AlfaAesar, TCI, Acros, Strem, Merck, ABCR, Fluka, Fisher Scientific, BLD Pharm*) and used without further purification. NaO*t*Bu was sublimed and stored in an Ar-filled glovebox. 15-Crown-5 was dried over 3 Å MS, distilled under N_2 atmosphere and stored under N_2 over 3 Å MS. H_2 (99.999%) was purchased from *Air Liquide* and D_2 (99.8%) from *Sigma-Aldrich*. [CuGua] (**3**),[3] dibenzyloxymethane,[4] [IMesCuCl] (**4**),[5] 2,3-diisopropyl-1,1-dimethylguanidine (**5**),[3] (4-bromophenyl)(morpholino)methanone (**S38**),[6] *N,N*-diisopropylbenzamide (**S39**),[7] 2-phenylisoindolin-1-one (**1af**),[8] phenyl(2,2,6,6-tetramethylpiperidin-1-yl)methanone (**1ag**),[9] 1-(2,4,6-trimethylphenyl)-1-*H* imidazole (**S78**),[3] 2-[4-(2-methylpropyl)phenyl]-1-morpholin-4-ylpropan-1-one (**S88**),[10] 1-morpholinobut-3-en-1-one (**S95**),[11] cyclohexyl-1-piperidinylmethanone (**S105**)[12] were synthesized according to literature known procedures.

2 Additional data

2.1 Additional optimization data

All reactions were performed following general procedure **GP3**.

2.1.1 Influence of NaO*t*Bu, pressure, solvent and catalyst loading

Table S1: Influence of NaOtBu, pressure, solvent and catalyst loading[a]

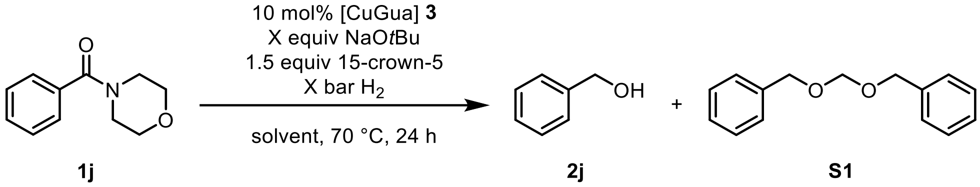

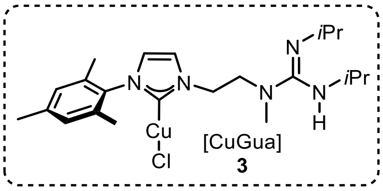

entry	pressure (bar)	NaO*t*Bu (equiv)	solvent	conversion of 1j[b]	2j/S1[b]
1	100	1.3	1,4-dioxane	100%	100:0
2	100	1.3	THF	86%	9:91
3	100	1.3	toluene	50%	76:24
4	100	0.5	1,4-dioxane	80%	96:4
5	50	1.3	1,4-dioxane	58%	71:29

[a] All reactions were performed with 0.1 mmol benzamide **1j** in 1 mL of solvent; [b] Conversion was determined by GC and GC/MS analysis and/or ^{1}H NMR analysis.

In order to identify the structure of acetal **S1**, purification by flash column chromatography on silica gel (cyclohexane/ethyl acetate = 8:2) of the reaction with THF as a solvent (**Table** , entry 2) was performed. The data is in accordance with literature.[4]

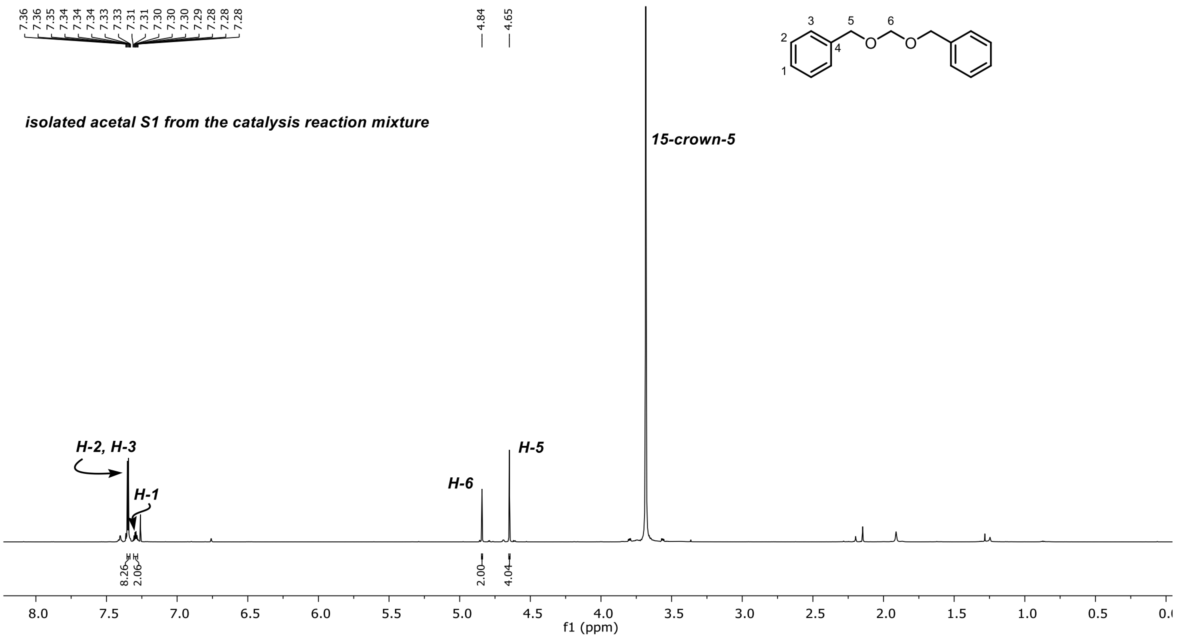

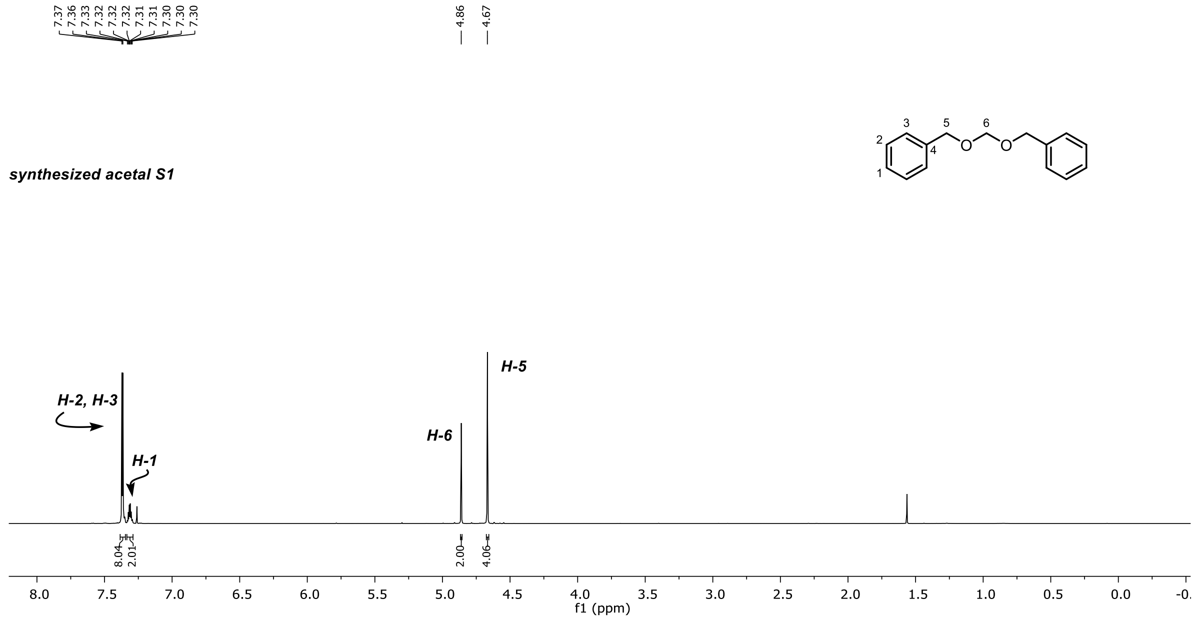

Figure S1: ^{1}H NMR of the acetal **S1** and comparison with synthesized acetal **S1**.

2.1.2 Influence of catalyst, catalyst loading and additive

Table S2: Influence of catalyst and catalyst loading[a]

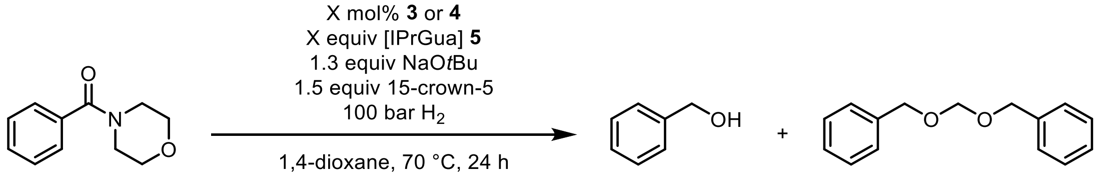

1j **2j** **S1**

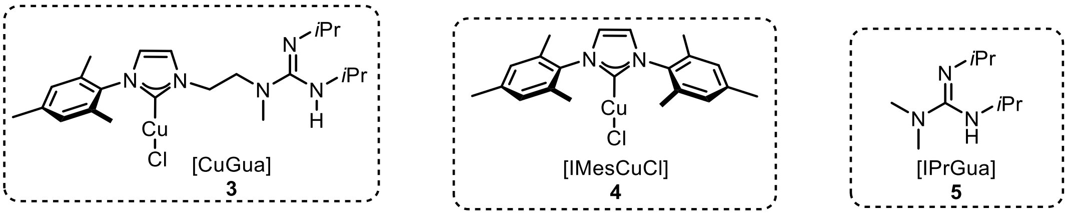

entry	catalyst	catalyst loading (mol%)	IPrGua (equiv)	conversion of 1j[b]	2j/S1[b]
1	[CuGua]	10	-	100%	100:0
2	[CuGua]	7.5	-	66%	95:5
3	[CuGua]	5	-	62%	87:13
4	[IMesCuCl]	10	-	4%	100:0
5	[IMesCuCl]	10	0.15	0%	-
6[c]	[IMesCuCl]	10	1.1	0%	-

[a] All reactions were performed with 0.1 mmol benzamide **1j** in 1 mL of 1,4-dioxane; [b] Conversion was determined by GC and GC/MS analysis and/or ^{1}H NMR analysis; [c] 0.05 mmol benzamide **1j** in 0.5 mL of 1,4-dioxane.

2.1.3 Negative controls

Table S3: Control experiments[a]

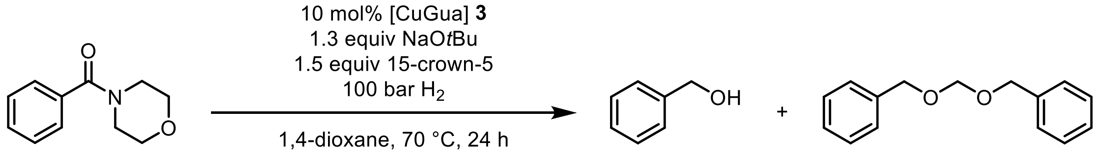

1j **2j** **S1**

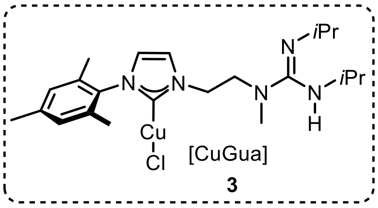

entry	deviation from standard conditions[b]	conversion of 1j[c]	1j/S1[c]
1	none	100%	100:0
2	No 15-crown-5	94%	100:0
3	No catalyst	0%	-
4	No H_2	0%	-
5	No base	0%	-

[a] All reactions were performed with 0.1 mmol benzamide **1j** in 1 mL of 1,4-dioxane; [b] Standard conditions refer to the conditions on Table , entry 1; [c] Conversion was determined by GC and GC/MS analysis and/or ^{1}H NMR analysis.

In order to gain more insight into the mechanism of this transformation, the above-mentioned control experiments were conducted. A key result shown in this table (Table , entry 4) provided no formation of the corresponding *tert*-butyl ester, whereas after the reaction only the presence of the starting amide **1j** could be detected. This experiment supports the conclusion that the copper-hydride complex reduced directly the amide, excluding the *in situ* formation of a *tert*-butyl ester, which could possibly serve as an intermediate for this transformation.

2.1.4 Influence of the *N*-substitution on the reactivity

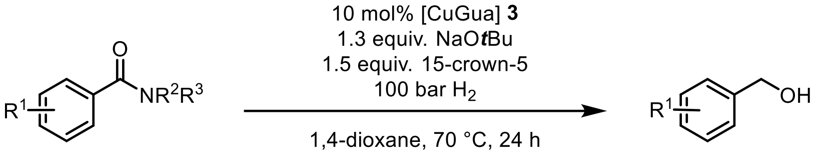

1 **2**

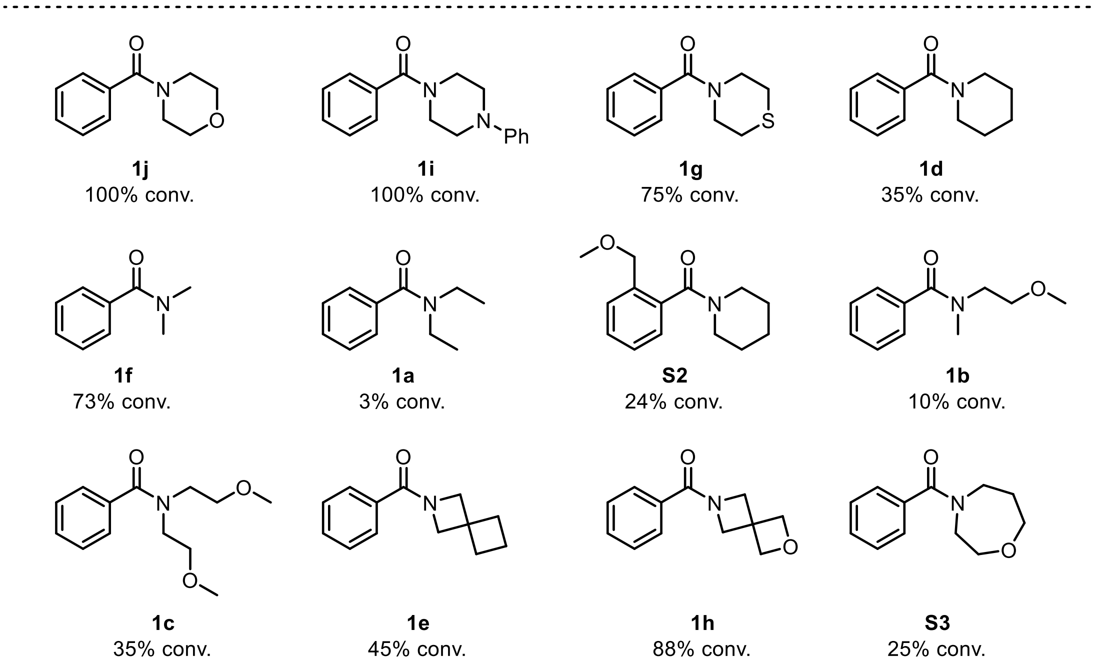

Figure S2: Influence of a second heteroatom on the molecule's skeleton.

3 General procedures

3.1 General procedure 1 – synthesis of amides *via* EDC-mediated amidation (GP1)

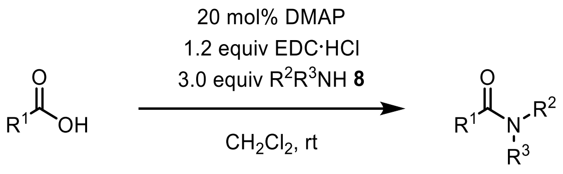

S4 **1**

According to a literature procedure,[13] a Schlenk flask is charged with the corresponding carboxylic acid (**S4**, 5.00 mmol, 1.00 equiv), DMAP (122 mg, 1.00 mmol, 20 mol%), the corresponding secondary amine (**8**, 15.0 mmol, 3.00 equiv) and CH_2Cl_2 (2 mL/mmol carboxylic acid). EDC·HCl (1.20 g, 6.00 mmol, 1.20 equiv) is added to the reaction mixture. The reaction mixture is stirred at room temperature until full conversion of the carboxylic acid **S4** is detected (conversion monitored *via* TLC). After quenching the reaction by addition of H_2O (2 mL/mmol carboxylic acid), the organic phase is washed with aq. HCl 2M (2 mL/mmol), brine (2 x 2 mL/mmol) and sat. aq. $NaHCO_3$ (2 mL/mmol). The organic layers are dried over $MgSO_4$ and filtered. All volatiles are removed under reduced pressure. The obtained crude product **1** is purified by flash column chromatography on silica gel.

3.2 General procedure 2 – synthesis of amides *via* acyl chlorides (GP2)

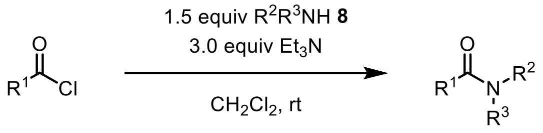

S5 **1**

According to a literature procedure,[14] a Schlenk flask is charged with the corresponding secondary amine **8** (7.50 mmol, 1.50 equiv), Et_3N (4.18 mL, 30.0 mmol, 3.00 equiv) and CH_2Cl_2 (2 mL/mmol acyl chloride). The corresponding acyl chloride (**S5**, 5.00 mmol, 1.00 equiv) is added dropwise to the reaction mixture. The reaction mixture is stirred at room temperature until full conversion of **S5** is detected (conversion monitored *via* TLC). The organic phase is washed with aq. HCl 2M (2 x 2 mL/mmol) and brine (2 x 2 mL/mmol). The organic layer is dried over $MgSO_4$ and filtered. All volatiles are removed under reduced pressure. The crude product **1** is purified by flash column chromatography on silica gel.

3.3 General procedure 3 – H_2-mediated 1,2-reduction of amides (GP3)

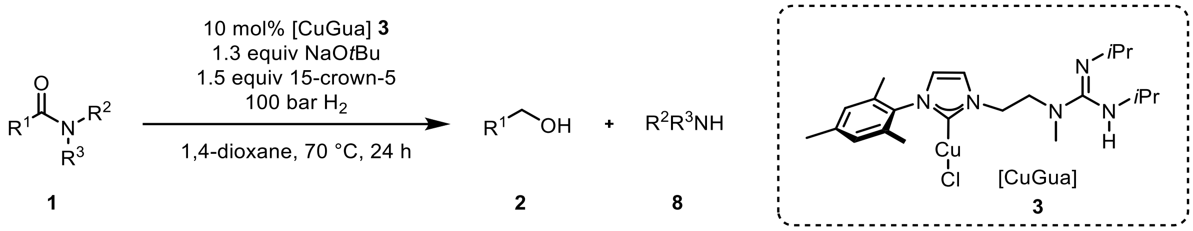

In an Ar-filled glovebox, [CuGua] (**3**, 18.7 mg, 40.0 µmol, 10 mol%) and NaO*t*Bu (49.9 mg, 0.520 mmol, 1.30 equiv) are placed in a 20 mL microwave vial with a stirring bar. The vial is capped inside the glovebox and then transferred outside. 1,4-Dioxane (3.0 mL) is added to the microwave vial and all solids are dissolved. The mixture is stirred in an oil bath for 5 min at 40 °C. Dried 15-crown-5 (0.120 mL, 0.600 mmol, 1.50 equiv) is added to the reaction mixture at rt. In a second 5 mL glass vial with a septum, the corresponding amide (**1**, 0.400 mmol, 1.00 equiv, for liquid amides the vial is purged with N_2, for solid amides three cycles of vacuum and backfilling with N_2 is followed) is dissolved in 1,4-dioxane (1.0 mL) and transferred to the reaction microwave vial. The vial is placed in an autoclave and the septum is pierced with a needle under N_2-counterflow. The autoclave is purged with H_2 (3 x 10 bar). The reaction mixture is stirred for 24 h at 70 °C under H_2-atmosphere (100 bar). The crude reaction mixture is filtered over a plug of silica (eluent: CH_2Cl_2/MeOH, 50:1, 1 x 5 cm, 60 mL) and all volatiles are removed under reduced pressure. The crude product **2** is purified by flash column chromatography on silica gel.

Notes: Before transferring the reaction mixture to the autoclave, it was necessary to have a tight closed reaction vial and to work under inert conditions. NaO*t*Bu had to be sublimed and stored in a glovebox. 15-Crown-5 had to be dried as described before.

3.4 General procedure 4 – site-selective 1,2-reduction of diamides (GP4)

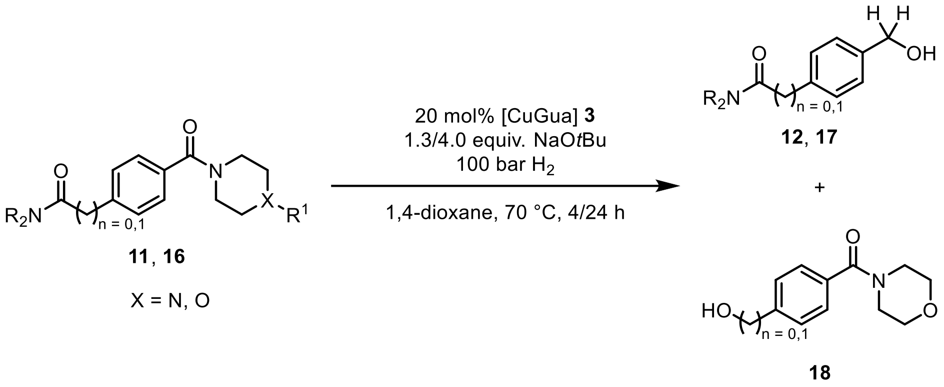

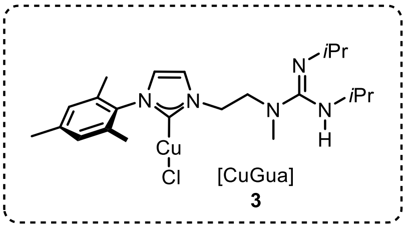

In an Ar-filled glovebox, [CuGua] (**3,** 18.7 mg, 40.0 µmol, 20 mol%) and NaO*t*Bu (25.0 mg, 0.260 mmol, 1.30 equiv or 76.9 mg, 0.800 mmol, 4.00 equiv) are placed in a 20 mL microwave vial with a stirring bar. The vial is capped inside the glovebox and then transferred outside. 1,4-Dioxane (1.0 mL) is added to the microwave vial and all solids are dissolved. The mixture is stirred in an oil bath for 5 min at 40 °C. In a second 5 mL glass vial with a septum, the corresponding amide (**11**, **16,** 0.200 mmol, 1.00 equiv, for liquid amides the vial is purged with N_2, for solid amides three cycles of vacuum and backfilling with N_2 is followed) is dissolved in 1,4-dioxane (1.0 mL) and transferred to the reaction microwave vial. The vial is placed in an autoclave and the septum is pierced with a needle under N_2-counterflow. The autoclave is purged with H_2 (3 x 10 bar). The reaction mixture is stirred for 4 or 24 h (4 h refers to the terephthalic acid derivatives **16a–16c**) at 70 °C under H_2-atmosphere (100 bar). The crude reaction mixture is filtered over a plug of silica (eluent: CH_2Cl_2/MeOH, 50:1, 1 x 5 cm, 60 mL, refers to the terephthalic acid derivatives **16a–16c**) or a PTFE filter 0.45 µm (refers to diamide **11**) and all volatiles are removed under reduced pressure. The crude product **12, 17** is purified by flash column chromatography on silica gel.

Notes: Before transferring the reaction mixture to the autoclave, it was necessary to have a tight closed reaction vial and to work under inert conditions. NaO*t*Bu had to be sublimed and stored in a glovebox.

3.5 General procedure 5 – Amide reduction with $LiAlH_4$ (GP5)

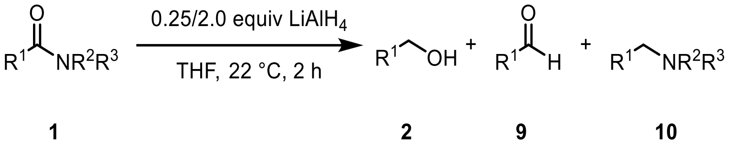

In deference to a literature procedure,[15] a Schlenk flask is charged with the corresponding amide (1.00 equiv) in dry THF (10 mL/1.00 mmol of **1**). $LiAlH_4$ (0.250 equiv for competition experiments, see section 5.2.2 or 2.00 equiv for reduction of diamides) is added to the reaction mixture. The reaction mixture is stirred at room temperature for 2 h. The reaction mixture is quenched with dropwise addition of H_2O (1.0 mL/1.00 mmol of **1**), diluted in EtOAc (1.0 mL/1.00 mmol of **1**) and the solution was filtered through a PTFE filter (0.45 µm) and all volatiles are removed under reduced pressure. The crude reaction mixture is analysed with GC, GCMS and/or ^{1}H NMR.

3.6 General procedure 6 – Amide reduction with D*i*BAl–H (GP6)

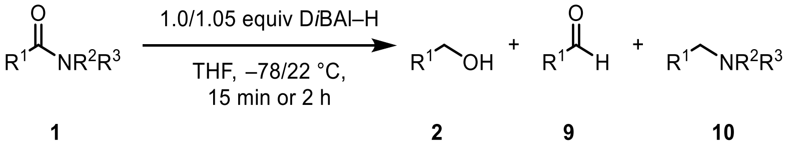

In deference to a literature procedure,[16] a Schlenk flask is charged with the corresponding amide (1.00 equiv) in dry THF (10 mL/1.00 mmol of **1**). D*i*BAl–H 1.2M in toluene (1.00 equiv for competition experiments, see section 5.2.1 or 1.05 equiv for reduction of diamides **11, 13, 16**) is added dropwise to the reaction mixture. The reaction mixture is stirred at room temperature for 15 min/2 h. The reaction mixture is quenched with H_2O (1.0 mL/1.00 mmol of **1**), diluted in EtOAc (1.0 mL/1.00 mmol of **1**) and the solution was filtered through a PTFE filter (0.45 µm) and all volatiles are removed under reduced pressure. The crude reaction mixture is analysed with GC, GCMS and/or ^{1}H NMR.

4 Experimental details

4.1 Syntheses of amides

4.1.1 *N,N*-Diethylbenzamide (1a)

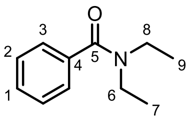

1a

$C_{11}H_{15}NO$

Mw = 177.25 g mol^{-1}

Prepared according to **GP2** from benzoyl chloride (**S5a**, 1.16 mL, 10.0 mmol, 1.00 equiv), diethylamine (**8a**, 1.55 mL, 15.0 mmol, 1.50 equiv) and Et_3N (4.18 mL, 30.0 mmol, 3.00 equiv) in CH_2Cl_2 (20 mL). The reaction mixture was stirred for 16 h at rt. Purification by flash column chromatography on silica gel (CH_2Cl_2/MeOH = 19:1) yielded **1a** as a white solid (1.23 g, 4.60 mmol, 92%).

R*f* = 0.40 (CH_2Cl_2/MeOH = 19:1).

^{1}H NMR (600 MHz, $CDCl_3$): δ = 1.09 (s, 3H, H-7)*, 1.24 (s, 3H, H-9)*, 3.24 (s, 2H, H-6)**, 3.55 (s, 2H, H-8)**, 7.37 (s, 5H, H-1, H-2, H-3) ppm.

^{13}C NMR (151 MHz, $CDCl_3$): δ = 13.0 (C-7)*, 14.3 (C-9)*, 39.3 (C-8)**, 43.4 (C-6)**, 126.4 (C-2)***, 128.5 (C-3)***, 129.2 (C-1), 137.4 (C-4), 171.4 (C-5) ppm.

HRMS (ESI) for $C_{11}H_{16}NO^+$ [$(M+H)^+$] calculated: 178.1232, found 178.1229.

The data is in accordance with literature.[17]

4.1.2 *N*-(2-Methoxyethyl)-*N*-methylbenzamide (1b)

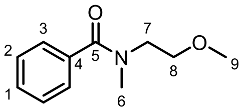

1b

$C_{11}H_{15}NO_2$

Mw = 193.25 g mol^{-1}

Prepared according to **GP2** from benzoyl chloride (**S5a**, 0.580 mL, 5.00 mmol, 1.00 equiv), 2-methoxy-*N*-methylethan-1-amine (**8b**, 0.815 mL, 7.50 mmol, 1.50 equiv) and Et_3N (2.09 mL, 15.0 mmol, 3.00 equiv) in CH_2Cl_2 (10 mL). The reaction mixture was stirred for 16 h at rt. Amide **1b** was afforded as a pale yellow oil (920 mg, 4.76 mmol, 95%) and was used without further purification.

R*f* = 0.40 (CH_2Cl_2/MeOH = 19:1).

^{1}H NMR (600 MHz, $CDCl_3$): δ = 2.92–3.21 (m, 3H, H-6), 3.21–3.49 (m, 5H, H-9, H-7*), 3.60–3.77 (m, 2H, H-8*), 7.28–7.66 (m, 5H, H-1, H-2, H-3) ppm.

^{13}C NMR (151 MHz, $CDCl_3$): δ = 33.5 (C-6), 39.3 (C-6), 47.6 (C-8*, C-7*), 50.8 (C-7*, C-8*), 59.0 (C-9), 70.3 (C-7*, C-8*), 71.1 (C-7*, C-8*), 127.0 (C-2)**, 128.4 (C-3)**, 129.4 (C-1)**, 129.5 (C-1)**, 136.7 (C-4), 171.6 (C-5), 172.4 (C-5) ppm. The product was obtained as a mixture of *E* and *Z* isomers.

IR (ATR): ṽ = 2929 (w), 2884 (w), 2817 (w), 1628 (s), 1397 (m), 1118 (m), 1073 (m), 708 (m) cm^{-1}.

HRMS (ESI) for $C_{11}H_{15}NO_2Na^+$ [$(M+Na)^+$] calculated: 216.0995, found 216.0999.

4.1.3 *N,N*-bis(2-Methoxyethyl)benzamide (1c)

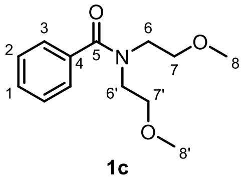

1c
$C_{13}H_{19}NO_3$
Mw 237.30 g mol^{-1}

Prepared according to **GP2** from benzoyl chloride (**S5a**, 0.580 mL, 5.00 mmol, 1.00 equiv), bis(2-methoxyethyl)amine (**8c**, 1.11 mL, 7.50 mmol, 1.50 equiv) and Et_3N (2.09 mL, 15.0 mmol, 3.00 equiv) in CH_2Cl_2 (10 mL). The reaction mixture was stirred for 16 h at rt. Amide **1c** was afforded as a pale yellow oil (1.18 g, 4.97 mmol, 99%) and was used without further purification.

R*f* = 0.30 (CH_2Cl_2/MeOH = 19:1).
^{1}H NMR (600 MHz, $CDCl_3$): δ = 3.25 (s, 3H, H-8')*, 3.34–3.45 (m, 5H, H-8*, H-7*), 3.51 (s, 2H, H-6)**, 3.66 (s, 2H, H-7')*, 3.75 (s, 2H, H-6')**, 7.30–7.53 (m, 5H, H-1, H-2, H-3) ppm.
^{13}C NMR (151 MHz, $CDCl_3$): δ = 45.6 (C-6')*, 49.8 (C-6)*, 59.0 (C-8, C-8'), 70.8 (C-7)**, 71.1 (C-7')**, 127.0 (C-2)***, 128.5 (C-3)***, 129.3 (C-1)***, 136.9 (C-4), 172.4 (C-5) ppm.
IR (ATR): ṽ = 2929 (w), 2881 (w), 2817 (w), 1628 (s), 1461 (m), 1416 (m), 1114 (s), 1073 (m), 1017 (m), 704 (m) cm^{-1}.
HRMS (ESI) for $C_{13}H_{20}NO_3^+$ [$(M+H)^+$] calculated: 238.1438, found 238.1437.

4.1.4 Phenyl(piperidin-1-yl)methanone (1d)

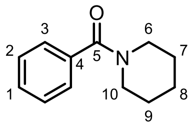

1d
$C_{12}H_{15}NO$
Mw = 189.26 g mol^{-1}

Prepared according to **GP2** from benzoyl chloride (**S5a**, 0.460 mL, 4.00 mmol, 1.00 equiv), piperidine (**8d**, 0.590 mL, 6.00 mmol, 1.50 equiv) and Et_3N (1.67 mL, 12.0 mmol, 3.00 equiv) in CH_2Cl_2 (8 mL). The reaction mixture was stirred for 16 h at rt. Purification by flash column chromatography on silica gel (cyclohexane/ ethyl acetate = 8:2) yielded **1d** (747 mg, 3.90 mmol, 98%) as a white solid.

R*f* = 0.20 (cyclohexane/ethyl acetate = 8:2).
^{1}H NMR (600 MHz, $CDCl_3$): δ = 1.52 (br s, 2H, H-7)*, 1.63–1.74 (m, 4H, H-8*, H-9*), 3.34 (br s, 2H, H-10)**, 3.71 (br s, 2H, H-6)**, 7.16–7.67 (m, 5H, H-1, H-2, H-3) ppm.
^{13}C NMR (151 MHz, $CDCl_3$): δ = 24.8 (C-8)*, 25.8 (C-7)*, 26.7 (C-9)*, 43.3 (C-6)**, 48.9 (C-10)**, 126.9 (C-1)***, 128.5 (C-2)***, 129.5 (C-3)***, 136.7 (C-4), 170.5 (C-5) ppm.
HRMS (ESI) for $C_{12}H_{16}NO^+$ [$(M+H)^+$] calculated: 190.1232, found: 190.1228.
The data is in accordance with literature.[14]

4.1.5 2-Azaspiro[3.3]heptan-2-yl(phenyl)methanone (1e)

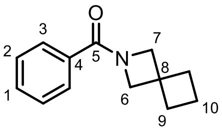

1e
$C_{13}H_{15}NO$
Mw = 201.27 g mol^{-1}

Prepared according to **GP2** from benzoyl chloride (**S5a**, 0.145 mL, 1.25 mmol, 1.00 equiv), 2-azaspiro[3.3]heptane-hydrochloride (**8e**, 250 mg, 1.87 mmol, 1.50 equiv) and Et_3N (0.697 mL, 5.00 mmol, 4.00 equiv) in CH_2Cl_2 (4 mL). Purification by flash column chromatography

on silica gel (cyclohexane/*tert*-butyl methyl ether = 1:1) yielded **1e** as a white solid (250 mg, 1.24 mmol, 99%).

R*f* = 0.50 (cyclohexane/ethyl acetate = 1:1).
m.p.: 48–50 °C.
^{1}H NMR (600 MHz, $CDCl_3$): δ = 1.76–1.95 (m, 2H, H-10), 2.12–2.27 (m, 4H, H-9), 4.16 (s, 2H, H-6)*, 4.22 (s, 2H, H-7)*, 7.37–7.42 (m, 2H, H-3), 7.41–7.51 (m, 1H, H-1), 7.59–7.68 (m, 2H, H-3) ppm.
^{13}C NMR (151 MHz, $CDCl_3$): 16.2 (C-10), 33.3 (C-9), 38.5 (C-8), 61.3 (C-6)*, 65.9 (C-7), 128.0 (C-3), 128.4 (C-2), 130.9 (C-1), 133.6 (C-4), 170.3 (C-5) ppm.
IR (ATR): ṽ = 2941 (m), 2870 (w), 1625 (s), 1573 (m), 1401 (s), 1308 (m), 798 (m), 716 (s) cm^{-1}.
HRMS (ESI) for $C_{13}H_{16}NO^+$ $[(M+H)^+]$ calculated: 202.1226, found: 202.1228.

4.1.6 Phenyl-4-thiomorpholinylmethanone (1g)

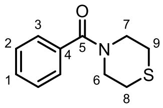

1g
$C_{11}H_{13}NOS$
Mw = 207.29 g mol^{-1}

Prepared according to **GP2** from benzoyl chloride (**S5a**, 0.600 mL, 5.00 mmol, 1.00 equiv), thiomorpholine (**8g**, 0.750 mL, 7.50 mmol, 1.50 equiv) and Et_3N (1.33 mL, 15.0 mmol, 3.00 equiv) in CH_2Cl_2 (10 mL). The reaction mixture was stirred for 16 h at rt. Amide **1g** was afforded as a white solid (869 mg, 4.50 mmol, 91%) and was used without further purification.

R*f* = 0.25 (cyclohexane/ethyl acetate = 7:3).
^{1}H NMR (600 MHz, $CDCl_3$): δ = 2.56 (s, 2H, H-8)*, 2.73 (s, 2H, H-9)*, 3.67 (s, 2H, H-6)**, 4.03 (s, 2H, H-7)**, 7.34–7.39 (m, 2H, H-3), 7.39–7.45 (m, 3H, H-1, H-2) ppm.
^{13}C NMR (151 MHz, $CDCl_3$): δ = 27.6 (C-8)*, 28.2 (C-9)*, 44.7 (C-7), 50.3 (C-6), 126.9 (C-3), 128.8 (C-2), 129.9 (C-1), 135.9 (C-4), 170.9 (C-5) ppm.
HRMS (ESI) for $C_{11}H_{14}NOS^+$ $[(M+H)^+]$ calculated: 208.0791, found: 208.0801.
The data is in accordance with literature.[18]

4.1.7 2-Oxa-6-azaspiro[3.3]hept-6-ylphenylmethanone (1h)

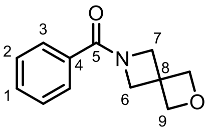

1h
$C_{12}H_{13}NO_2$
Mw = 203.24 g mol^{-1}

Prepared according to **GP2** from benzoyl chloride (**S5a**, 0.232 mL, 2.00 mmol, 1.00 equiv), 2-oxa-6-azaspiro[3.3]heptane (**8h**, 300 mg, 3.00 mmol, 1.50 equiv) and Et_3N (0.836 mL, 6.00 mmol, 3.00 equiv) in CH_2Cl_2 (4 mL). The reaction mixture was stirred for 16 h at rt. Amide **1h** was afforded as a white solid (400 mg, 1.97 mmol, 98%) and was used without further purification.

R*f* = 0.20 (cyclohexane/ethyl acetate = 8:2).
^{1}H NMR (600 MHz, $CDCl_3$): δ = 7.65–7.54 (m, 2H, H-3), 7.51–7.43 (m, 1H, H-2), 7.45–7.30 (m, 2H, H-4), 4.83 (s, 2H, H-9), 4.78 (s, 2H, H-9), 4.43 (s, 2H, H-6)*, 4.34 (s, 2H, H-7)* ppm.
^{13}C NMR (151 MHz, $CDCl_3$): 170.5 (C-5), 132.9 (C-4), 131.4 (C-1), 128.6 (C-2), 128.0 (C-3), 81.0 (C-9), 63.0 (C-6)*, 58.3 (C-7)*, 38.5 (C-8) ppm.
HRMS (ESI) for $C_{12}H_{14}NO_2^+$ [(M+H)$^+$] calculated: 204.1019, found: 204.1026.
The data is in accordance with literature.[19]

4.1.8 Phenyl(4-phenylpiperazin-1-yl)methanone (1i)

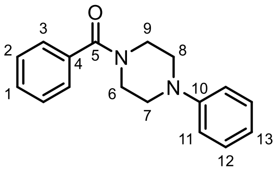

1i
$C_{17}H_{18}N_2O$
Mw = 266.34 g mol^{-1}

Prepared according to **GP2** from benzoyl chloride (**S5a**, 1.16 mL, 10.0 mmol, 1.00 equiv), 1-phenylpiperazine (**8i**, 2.29 mL, 15.0 mmol, 1.50 equiv) and Et_3N (4.18 mL, 30.0 mmol, 3.00 equiv) in CH_2Cl_2 (20 mL). The reaction mixture was stirred for 16 h at rt. Purification by flash column chromatography on silica gel (cyclohexane/*tert*-butyl methyl ether = 1:1) yielded **1i** as a white solid (1.19 g, 4.47 mmol, 45%).

R*f* = 0.15 (cyclohexane/*tert*-butyl methyl ether = 1:1).
^{1}H NMR (600 MHz, $CDCl_3$): δ = 3.14 (br s, 2H, H-6)*, 3.28 (br s, 2H, H-9)*, 3.62 (br s, 2H, H-8)**, 3.97 (br s, 2H, H-7)**, 6.86–7.07 (m, 3H, H-11 H-13), 7.30 (t, $^3J_{13,12}$ = 7.8 Hz, 2H, H-12), 7.41–7.47 (m, 5H, H-1, H-2, H-3) ppm.
^{13}C NMR (151 MHz, $CDCl_3$): δ = 42.2 (C-7)*, 47.6 (C-8)*, 49.9 (C-6)**, 50.3 (C-9)**, 117.0 (C-11)***, 121.0 (C-13)***, 127.3 (C-2)****, 128.7 (C-3)****, 129.4 (C-12), 130.0 (C-1), 135.7 (C-4), 150.9 (C-10), 170.5 (C-5) ppm.
HRMS (APCI) for $C_{17}H_{19}ON_2^+$ [(M+H)$^+$] calculated: 267.1492, found 267.1485.
The data is in accordance with literature.[20]

4.1.9 Morpholino(phenyl)methanone (1j)

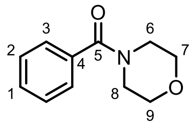

1j
$C_{11}H_{13}NO_2$
Mw = 191.23 g mol^{-1}

Prepared according to **GP2** from benzoyl chloride (**S5a**, 1.16 mL, 10.0 mmol, 1.00 equiv), morpholine (**8j**, 1.30 mL, 15.0 mmol, 1.50 equiv) and Et_3N (4.18 mL, 30.0 mmol, 3.00 equiv) in CH_2Cl_2 (20 mL). The reaction mixture was stirred for 16 h at rt. Amide **1j** was afforded as a white solid (1.59 g, 8.30 mmol, 83%) and was used without further purification.

R*f* = 0.15 (cyclohexane/ethyl acetate = 8:2).
^{1}H NMR (600 MHz, $CDCl_3$): δ = 3.44 (br s, 2H, H-6*), 3.56–3.85 (m, 6H, H-7*, H-8, H-9), 7.37–7.47 (m, 5H, H-1, H-2, H-3) ppm.
^{13}C NMR (151 MHz, $CDCl_3$): δ = 42.7 (C-6)*, 48.4 (C-8)*, 67.0 (C-7, C-9), 127.2 (C-1)**, 128.7 (C-2)**, 130.0 (C-3)**, 135.4 (C-4), 170.6 (C-5) ppm.
HRMS (ESI) for $C_{11}H_{13}NO_2Na^+$ [(M+Na)$^+$] calculated: 214.0844, found: 214.0841.
The data is in accordance with literature.[21]

4.1.10 Morpholino(*p*-tolyl)methanone (1m)

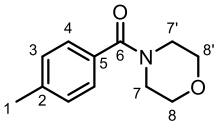

1m
$C_{12}H_{15}NO_2$
Mw = 205.26 g mol^{-1}

Prepared according to **GP2** from 4-methylbenzoyl chloride (**S5b**, 0.650 mL, 5.00 mmol, 1.00 equiv), morpholine (**8j**, 0.650 mL, 7.50 mmol, 1.50 equiv) and Et_3N (2.09 mL, 15.0 mmol, 3.00 equiv) in CH_2Cl_2 (10 mL). The reaction mixture was stirred for 16 h at rt. Amide **1m** was afforded as a white solid (1.02 g, 4.90 mmol, 99%) and was used without further purification.

R*f* = 0.20 (cyclohexane/*tert*-butyl methyl ether = 1:1).
^{1}H NMR (400 MHz, $CDCl_3$): δ = 2.38 (s, 3H, H-1), 3.69 (s, 8H, H-7, H-7', H-8, H-8'), 7.21 (d, $^3J_{3,4}$ = 8.2 Hz, 2H, H-3), 7.31 (d, $^3J_{4,3}$ = 8.0 Hz, 2H, H-4) ppm.
^{13}C NMR (101 MHz, $CDCl_3$): δ = 21.5 (C-1), 42.8 (C-7)*, 48.7 (C-7')*, 67.1 (C-8, C-8'), 127.4 (C-4), 129.3 (C-3), 132.5 (C-5), 140.2 (C-2), 170.7 (C-6) ppm.
HRMS (APCI) for $C_{12}H_{16}NO_2^+$ [(M+H)$^+$] calculated: 206.1181, found: 206.1174.
The data is in accordance with literature.[21]

4.1.11 Cyclohexyl(morpholino)methanone (1n)

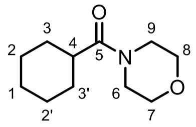

1n
$C_{11}H_{19}NO_2$
Mw = 197.28 g mol^{-1}

Prepared according to **GP1** from cyclohexanecarboxylic acid (**S4a**, 0.620 mL, 5.00 mmol, 1.00 equiv), morpholine (**8j**, 1.29 mL, 15.0 mmol, 3.00 equiv), EDC·HCl (1.15 g, 6.00 mmol, 1.20 equiv) and DMAP (122 mg, 1.00 mmol, 20 mol%) in CH_2Cl_2 (10 mL). The reaction mixture was stirred for 16 h at rt. Amide **1n** was afforded as a white solid (905 mg, 4.59 mmol, 92%) and was used without further purification.

R*f* = 0.40 (cyclohexane/*tert*-butyl methyl ether = 6:4).

^{1}H NMR (600 MHz, $CDCl_3$): δ = 1.16–1.34 (m, 3H, H-3*,H-1a**), 1.53 (qd, ^3J = 11.9 Hz, ^3J = 3.9 Hz, 2H, H-3')*, 1.64–1.74 (m, 3H, H-2'***, H-1b**), 1.76–1.86 (m, 2H, H-2)***, 2.43 (tt, $^3J_{4,3ax}$ = 11.6 Hz, $^3J_{4,3eq}$ = 3.4 Hz, 1H, H-4), 3.49 (s, 2H, H-9)****, 3.61 (s, 2H, H-6)****, 3.66 (s, 4H, H-7, H-8) ppm.

^{13}C NMR (151 MHz, $CDCl_3$): δ = 26.0 (C-1)*, 26.0 (C-2, C-2')*, 29.5 (C-3, C-3')*, 40.4 (C-4), 42.1 (C-6)**, 46.1 (C-9)**, 67.1 (C-7)***, 67.2 (C-8)***, 174.9 (C-5) ppm.

HRMS (APCI) for $C_{11}H_{20}O_2N^+$ [$(M+H)^+$] calculated: 198.1489, found 198.1488.

The data is in accordance with literature.[22]

4.1.12 Morpholino(*m*-tolyl)methanone (1o)

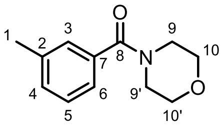

1o
$C_{12}H_{15}NO_2$
Mw = 205.26 g mol^{-1}

Prepared according to **GP2** from 3-methylbenzoyl chloride (**S5c**, 0.650 mL, 5.00 mmol, 1.00 equiv), morpholine (**8j**, 0.650 mL, 7.50 mmol, 1.50 equiv) and Et_3N (2.09 mL, 15.0 mmol, 3.00 equiv) in CH_2Cl_2 (10 mL). The reaction mixture was stirred for 16 h at rt. Purification by flash column chromatography on silica gel (cyclohexane/*tert*-butyl methyl ether = 6:4) yielded **1o** as a colourless oil (816 mg, 4.00 mmol, 80%).

R_f = 0.15 (cyclohexane/*tert*-butyl methyl ether = 6:4).

^{1}H NMR (400 MHz, $CDCl_3$): δ = 2.37 (s, 3H, H-1), 3.44 (br s, 2H, H-9)*, 3.62 (br s, 2H, H-10)*, 3.76 (br s, 4H, H-9', H-10'), 7.17 (d, $^3J_{6,5}$ = 7.5 Hz, 1H, H-6), 7.20–7.24 (m, 2H, H-3, H-4), 7.28 (t, $^3J_{5,4}$ = 7.3 Hz, 1H, H-5) ppm.

^{13}C NMR (101 MHz, $CDCl_3$): δ = 21.5 (C-1), 42.6 (C-9)*, 48.4 (C-9')*, 67.1 (C-10, C-10'), 124.1 (C-6), 127.8 (C-3)**, 128.5 (C-5), 130.7 (C-4)**, 135.4 (C-7), 138.7 (C-2), 170.8 (C-8) ppm.

HRMS (APCI) for $C_{12}H_{16}NO_2^+$ [$(M+H)^+$] calculated: 206.1181, found: 206.1172.

The data is in accordance with literature.[21]

4.1.13 Morpholino(*o*-tolyl)methanone (1p)

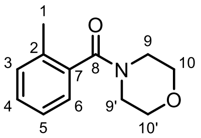

1p
$C_{12}H_{15}NO_2$
Mw = 205.26 g mol^{-1}

Prepared according to **GP2** from 2-methylbenzoyl chloride (**S5d**, 0.650 mL, 5.00 mmol, 1.00 equiv), morpholine (**8j**, 0.650 mL, 7.50 mmol, 1.50 equiv) and Et_3N (2.09 mL, 15.0 mmol, 3.00 equiv) in CH_2Cl_2 (10 mL). The reaction mixture was stirred for 16 h at rt. Purification by flash column chromatography on silica gel (cyclohexane/*tert*-butyl methyl ether = 6:4) yielded **1p** as a white solid (805 mg, 3.90 mmol, 79%).

R_f = 0.15 (cyclohexane/*tert*-butyl methyl ether = 6:4).

^{1}H NMR (400 MHz, $CDCl_3$): δ = 2.31 (s, 3H, H-1), 3.24 (s, 2H, H-9)*, 3.57 (s, 2H, H-10)**, 3.77 (s, 2H, H-10')**, 3.83 (s, 2H, H-9')*, 7.15 (d, $^3J_{6,5}$ = 8.2 Hz, 1H, H-6), 7.18–7.25 (m, 2H, H-3, H-4), 7.28 (t, $^3J_{5,4}$ = 8.0 Hz, 1H, H-5) ppm.

^{13}**C NMR** (101 MHz, $CDCl_3$): δ = 19.2 (C-1), 42.0 (C-9')*, 47.4 (C-9)*, 67.0 (C-10)**, 67.1 (C-10')**, 126.0 (C-6), 126.1 (C-3)***, 129.2 (C-5), 130.6 (C-4)***, 134.3 (C-7), 135.8 (C-2), 170.2 (C-8) ppm.
HRMS (APCI) for $C_{12}H_{16}NO_2^+$ $[(M+H)^+]$ calculated: 206.1181, found: 206.1174.
The data is in accordance with literature.[21]

4.1.14 Morpholino(4-(trifluoromethyl)phenyl)methanone (1q)

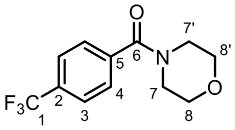

1q
$C_{12}H_{12}F_3NO_2$
Mw = 259.23 g mol^{-1}

Prepared according to **GP1** from 4-(trifluoromethyl)benzoic acid (**S4b**, 1.00 g, 5.26 mmol, 1.00 equiv), morpholine (**8j**, 1.36 mL, 15.8 mmol, 3.00 equiv), EDC·HCl (1.21 g, 6.31 mmol, 1.20 equiv) and DMAP (129 mg, 1.05 mmol, 20 mol%) in CH_2Cl_2 (10 mL). The reaction mixture was stirred for 16 h at rt. Amide **1q** was afforded as a white solid (980 mg, 3.78 mmol, 72%) and was used without further purification.

$\mathbf{R_{\mathit{f}}}$ = 0.15 (cyclohexane/*tert*-butyl methyl ether = 1:1).
^{1}**H NMR** (600 MHz, $CDCl_3$): δ = 3.40 (s, 2H, H-7)*, 3.62 (s, 2H, H-8)**, 3.79 (s, 4H, H-7'*, H-8'**), 7.52 (d, $^3J_{4,3}$ = 8.0 Hz, 2H, H-4), 7.69 (d, $^3J_{3,4}$ = 8.0 Hz, 2H, H-3) ppm.
^{13}**C NMR** (151 MHz, $CDCl_3$): δ = 42.7 (C-7)*, 48.2 (C-7')*, 66.9 (C-8, C-8'), 123.9. (q, $^1J_{C\text{-}1,F}$ = 272.4 Hz, C-1), 125.8 (q, $^3J_{C\text{-}3,F}$ = 3.8 Hz, C-3), 127.6 (C-4), 132.1 (q, $^2J_{C\text{-}2,F}$ = 32.7 Hz, C-2), 139.0 (C-5), 169.1 (C-6) ppm.
^{19}**F NMR** (564 MHz, $CDCl_3$): δ = – 62.9 ppm.
HRMS (APCI) for $C_{12}H_{13}O_2NF_3^+$ $[(M+H)^+]$ calculated: 260.0893, found: 260.0888.
The data is in accordance with literature.[22]

4.1.15 (4-Methoxyphenyl)(morpholino)methanone (1r)

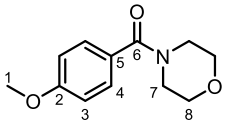

1r
$C_{12}H_{15}NO_3$
Mw = 221.26 g mol^{-1}

Prepared according to **GP1** from 4-methoxybenzoic acid (**S4c**, 1.07 g, 7.00 mmol, 1.00 equiv), morpholine (**8j**, 1.81 mL, 21.0 mmol, 3.00 equiv), EDC·HCl (1.61 g, 8.40 mmol, 1.20 equiv) and DMAP (171 mg, 1.40 mmol, 20 mol%) in CH_2Cl_2 (15 mL). The reaction mixture was stirred for 16 h at rt. Purification by flash column chromatography on silica gel (CH_2Cl_2/MeOH = 19:1) yielded **1r** as a colourless oil (1.22 g, 5.52 mmol, 79%).

$\mathbf{R_{\mathit{f}}}$ = 0.35 (CH_2Cl_2/MeOH = 19:1).
^{1}**H NMR** (600 MHz, $CDCl_3$): δ = 3.61–3.79 (m, 8H, H-7, H-8), 3.82 (s, 3H, H-1), 6.91 (d, $^3J_{3,4}$ = 8.7 Hz, 2H, H-3), 7.38 (d, $^3J_{4,3}$ = 8.7 Hz, 2H, H-4) ppm.
Traces of unidentified side products could be detected in ^{1}H NMR at 3.46 ppm.
^{13}**C NMR** (151 MHz, $CDCl_3$): δ = 55.5 (C-1), 67.0 (C-7, C-8), 113.9 (C-3), 127.4 (C-5), 129.3 (C-4), 161.0 (C-2), 170.6 (C-6) ppm.
HRMS (APCI) for $C_{12}H_{16}O_3N^+$ $[(M+H)^+]$ calculated: 222.1125, found: 222.1132.

The data is in accordance with literature.[21]

4.1.16 (4-Chlorophenyl)(morpholino)methanone (1s)

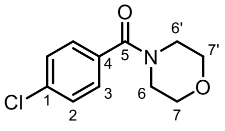

1s
$C_{11}H_{12}ClNO_2$
Mw = 225.67 g mol^{-1}

Prepared according to **GP1** from 4-chlorobenzoic acid (**S4d**, 783 mg, 5.00 mmol, 1.00 equiv), morpholine (**8j**, 1.29 mL, 15.0 mmol, 3.00 equiv), EDC·HCl (1.15 g, 6.00 mmol, 1.20 equiv) and DMAP (122 mg, 1.00 mmol, 20 mol%) in CH_2Cl_2 (10 mL). The reaction mixture was stirred for 16 h at rt. Amide **1s** was afforded as a white solid (907 mg, 4.02 mmol, 80%) and was used without further purification.

R*f* = 0.15 (cyclohexane/*tert*-butyl methyl ether = 1:1).
^{1}H NMR (600 MHz, $CDCl_3$): δ = 3.43 (br s, 2H, H-6)*, 3.64–3.77 (m, 6H, H-6', H-7, H-7')*, 7.35 (d, $^3J_{3,2}$ = 8.5 Hz, 2H, H-3), 7.39 (d, $^3J_{2,3}$ = 8.6 Hz, 2H, H-2) ppm.
^{13}C NMR (151MHz, $CDCl_3$): δ = 42.8 (C-6)*, 48.4 (C-6')*, 67.0 (C-7, C-7'), 128.8 (C-3), 129.0 (C-2), 133.7 (C-4), 136.1 (C-1), 169.5 (C-5) ppm.
HRMS (APCI) for $C_{11}H_{13}O_2N^{35}Cl^+$ [(M+H)$^+$] calculated: 226.0629, found: 226.0624.
The data is in accordance with literature.[21]

4.1.17 Morpholino(4-(4,4,5,5-tetramethyl-1,3,2-dioxaborolan-2-yl)phenyl)methanone (1t)

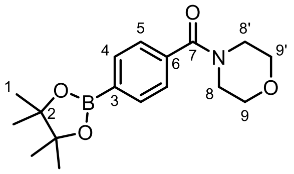

1t
$C_{17}H_{24}BNO_4$
Mw = 317.19 g mol^{-1}

Prepared according to **GP1** from 4-(4,4,5,5-tetramethyl-1,3,2-dioxaborolan-2-yl)benzoic acid (**S4e**, 1.24 g, 5.00 mmol, 1.00 equiv), morpholine (**8j**, 1.30 mL, 15.0 mmol, 3.00 equiv), EDC·HCl (1.10 g, 6.00 mmol, 1.20 equiv) and DMAP (122 mg, 1.00 mmol, 20 mol%) in CH_2Cl_2 (10 mL). The reaction mixture was stirred for 16 h at rt. Purification by flash column chromatography on silica gel (cyclohexane/*tert*-butyl methyl ether = 1:1) yielded **1t** as a white solid (736 mg, 3.90 mmol, 77%).

R*f* = 0.15 (cyclohexane/*tert*-butyl methyl ether = 1:1).
^{1}H NMR (600MHz, $CDCl_3$): δ = 1.35 (s, 12H, H-1), 3.40 (s, 2H, H-8)*, 3.59 (s, 2H, H-9)*, 3.77 (s, 4H, H-8', H-9')*, 7.39 (d, $^3J_{5,4}$ = 8.1 Hz, 2H, H-5), 7.84 (d, $^3J_{4,5}$ = 7.9 Hz, 2H, H-4) ppm.
^{13}C NMR (151 MHz, $CDCl_3$): δ = 25.0 (C-1), 42.7 (C-8'), 48.3 (C-8), 67.0 (C-9, C-9´), 84.2 (C-2), 126.3 (C-5), 135.0 (C-4), 138.0 (C-6), 170.5 (C-7) ppm. C-3 could not be detected in ^{13}C NMR.
^{11}B NMR (193 MHz, $CDCl_3$): δ = 30.1 ppm.
HRMS (APCI) for $C_{17}H_{25}O_4NB^+$ [(M+H)$^+$] calculated: 318.1874, found: 318.1875.
The data is in accordance with literature.[23]

4.1.18 Furan-2-yl(morpholino)methanone (1u)

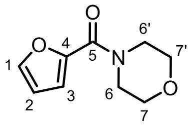

1u

$C_9H_{11}NO_3$

Mw = 181.19 g mol^{-1}

Prepared according to **GP2** from furan-2-carbonyl chloride (**S5e**, 0.500 mL, 5.00 mmol, 1.00 equiv), morpholine (**8j**, 0.650 mL, 7.50 mmol, 1.50 equiv) and Et_3N (2.09 mL, 15.0 mmol, 3.00 equiv) in CH_2Cl_2 (10 mL). The reaction mixture was stirred for 16 h at rt. Purification by flash column chromatography on silica gel (cyclohexane/*tert*-butyl methyl ether = 6:4) yielded **1u** as a white solid (780 mg, 4.30 mmol, 86%).

R*f* = 0.10 (cyclohexane/*tert*-butyl methyl ether = 6:4).

^{1}H NMR (600 MHz, $CDCl_3$): δ = 3.61–3.99 (m, 8H, H-6, H-6´, H-7, H-7'), 6.38–6.62 (m, 1H, H-2), 6.92–7.11 (m, 1H, H-3), 7.34–7.53 (m, 1H, H-1) ppm.

^{13}C NMR (151 MHz, $CDCl_3$): δ = 43.5 (C-6)*, 47.1 (C-6')*, 67.1 (C-7, C-7'), 111.5 (C-2), 116.9 (C-3), 143.9 (C-1), 147.9 (C-4), 159.2 (C-5) ppm.

HRMS (APCI) for $C_9H_{12}O_3N^+$ [$(M+H)^+$] calculated: 182.0817, found: 182.0815.

The data is in accordance with literature.[24]

4.1.19 Morpholino(thiophen-2-yl)methanone (1v)

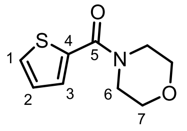

1v

$C_9H_{11}NO_2S$

Mw = 197.25 g mol^{-1}

Prepared according to **GP2** from thiophene-2-carbonyl chloride (**S5f**, 0.550 mL, 5.00 mmol, 1.00 equiv), morpholine (**8j**, 0.650 mL, 7.50 mmol, 1.50 equiv) and Et_3N (2.09 mL, 15.0 mmol, 3.00 equiv) in CH_2Cl_2 (10 mL). The reaction mixture was stirred for 16 h at rt. Purification by flash column chromatography on silica gel (cyclohexane/*tert*-butyl methyl ether = 1:1) yielded **1v** as a white solid (832 mg, 4.20 mmol, 84%).

R*f* = 0.15 (cyclohexane/*tert*-butyl methyl ether = 1:1).

^{1}H NMR (600 MHz, $CDCl_3$): δ = 3.70–3.74 (m, 4H, H-6), 3.75–3.78 (m, 4H, H-7), 7.05 (dd, $^3J_{2,1}$ = 5.0 Hz, $^3J_{2,3}$ = 3.7 Hz, 1H, H-2), 7.29 (d, $^3J_{3,2}$ = 3.7 Hz, 1H, H-3), 7.46 (d, $^3J_{1,2}$ = 4.9 Hz, 1H, H-1) ppm.

^{13}C NMR (151 MHz, $CDCl_3$): δ = 45.9 (C-6), 67.0 (C-7), 126.9 (C-2), 129.0 (C-1), 129.1 (C-3), 136.7 (C-4), 163.8 (C-5) ppm.

HRMS (APCI) for $C_9H_{12}O_2NS^+$ [$(M+H)^+$] calculated: 198.0589, found: 198.0580.

The data is in accordance with literature.[21]

4.1.20 (5-((Benzyloxy)methyl)furan-2-yl)(morpholino)methanone (1w)

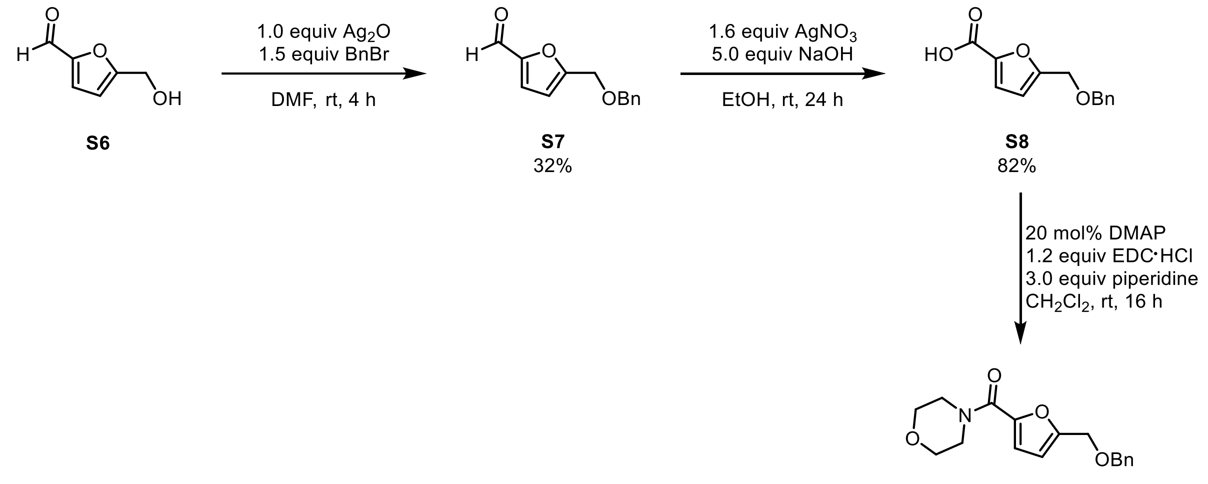

Scheme S1: Synthesis of (5-((benzyloxy)methyl)furan-2-yl)(morpholino)methanone (**1w**).

According to a literature procedure,[25] a 100 mL Schlenk tube equipped with a magnetic stir bar was charged with 5-(hydroxymethyl)furan-2-carbaldehyde (**S6**, 2.03 mL, 20.0 mmol, 1.00 equiv) and anhydrous DMF (15 mL). Benzyl bromide (3.57 mL, 30.0 mmol, 1.50 equiv) and Ag_2O (4.63 g, 20.0 mmol, 1.00 equiv) were added subsequently. The Schlenk tube was covered with aluminum foil to exclude light and the reaction mixture was stirred at room temperature for 4 h. After full conversion of **S6** (monitored *via* TLC), the suspension was diluted with EtOAc (30 mL), filtered through a pad of Celite© (3 cm), washed with EtOAc (20 mL) and all volatiles were removed under reduced pressure. Purification by flash column chromatography on silica gel (cyclohexane/ethyl acetate = 9:1) yielded, 5-((benzyloxy)methyl)furan-2-carbaldehyde (**S7**, 1.39 g, 6.43 mmol, 32%) as a yellow oil.

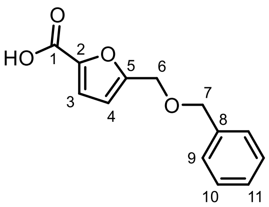

S8
$C_{13}H_{12}O_4$
Mw = 232.24 g mol^{-1}

Based on a modified literature procedure,[26] a 100 mL Schlenk tube equipped with a magnetic stir bar was charged with aldehyde **S7** (500 mg, 2.31 mmol, 1.00 equiv) and EtOH (17 mL). A solution of $AgNO_3$ (625 mg, 3.68 mmol, 1.6 equiv in 0.5 mL H_2O and 8.0 mL EtOH) was added to the reaction mixture. A solution of NaOH (460 mg, 11.5 mmol, 5.0 equiv in 1.0 mL H_2O and 8.0 mL EtOH) was added dropwise and the reaction mixture was stirred at room temperature for 24 h. The reaction mixture was filtered through a pad of Celite© (3 cm), washed with EtOH (20 mL) and all volatiles were removed under reduced pressure. The crude product was dissolved with H_2O (10 mL) and acidified with conc. HCl until pH = 1 was reached. The corresponding solid was filtered through a glass frit (P4), washed with H_2O (20 mL) and all volatiles were removed under reduced pressure, yielding 5-((benzyloxy)methyl)furan-2-carboxylic acid (**S8**, 440 mg, 1.89 mmol, 82%) as a pale yellow solid.

R_f = 0.15 (ethyl acetate/diethylether = 2:8).
m.p: 99–100 °C.
^{1}H NMR (600 MHz, $CDCl_3$): δ = 4.58 (s, 2H, H-6), 4.61 (s, 2H, H-7), 6.50 (d, $^3J_{4,3}$ = 3.4 Hz, 1H, H-4), 7.28–7.34 (m, 2H, H-4, H-11), 7.35–7.40 (m, 4H, H-9, H-10), 11.53 (s, 1H, *COO–H*) ppm.
^{13}C NMR (151 MHz, $CDCl_3$): δ = 64.1 (C-6), 72.8 (C-7), 111.3 (C-4), 121.1 (C-3), 128.1 (C-10, C-11)*, 128.6 (C-9)*, 137.4 (C-8), 143.5 (C-2), 157.6 (C-5), 163.6 (C-1) ppm.
IR (ATR): ṽ = 3123 (w), 3060 (w), 2892 (w), 2858 (w), 2799 (w), 1673 (s), 1531 (m), 1423 (m), 1349 (m), 1207 (m), 1162 (m), 697 (m) cm^{-1}.
HRMS (APCI) for $C_{13}H_{13}O_3^+$ [$(M-O+H^+)^+$] calculated: 217.0859, found 217.0855.

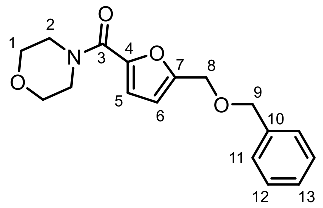

1w
$C_{17}H_{19}NO_4$
Mw = 301.34 g mol^{-1}

Following general procedure **GP1**, a 25 mL Schlenk tube equipped with a magnetic stir bar, was charged with 5-((benzyloxy)methyl)furan-2-carboxylic acid (**S8**, 400 mg, 1.72 mmol, 1.00 equiv) and CH_2Cl_2 (10 mL). EDC·HCl (395 mg, 2.06 mmol, 1.20 equiv), morpholine (**8j**, 407 µL, 5.16 mmol, 3.00 equiv) and DMAP (43.0 mg, 350 µmol, 20 mol%) were added to the reaction mixture. The reaction mixture was stirred at rt until full conversion of **S8** was detected (monitored *via* TLC). Purification by flash column chromatography on silica gel (ethyl acetate/diethylether = 2:8) yielded (5-((benzyloxy)methyl)furan-2-yl)(morpholino)methanone (**1w**, 220 mg, 0.730 mmol, 42%) as a pale yellow solid.

R_f = 0.25 (ethyl acetate/diethylether = 2:8).
m.p: 51–54 °C.
^{1}H NMR (600 MHz, $CDCl_3$): 3.73 (t, $^3J_{1,2}$ = 4.6 Hz, 4H, H-1), 3.82 (br s, 4H, H-2), 4.51 (s, 2H, H-8), 4.56 (s, 2H, H-9), 6.42 (d, $^3J_{6,5}$ = 3.4 Hz, 1H, H-6), 6.99 (d, $^3J_{5,6}$ = 3.1 Hz, 1H, H-5), 7.27–7.40 (m, 5H, H-11, H-12, H-13) ppm.
^{13}C NMR (151 MHz, $CDCl_3$): δ = 64.1 (C-8), 67.1 (C-1), 72.4 (C-9), 110.9 (C-6), 117.7 (C-5), 128.0 (C-11)*, 128.1 (C-13)*, 128.7 (C-12)*, 137.6 (C-10), 147.8 (C-4), 153.6 (C-7), 159.1 (C-3) ppm.
C-1 and C-2 could not be detected by ^{13}C NMR. The appropriate data was extracted from the ^{1}H, ^{1}H COSY, ^{1}H, ^{13}C HMBC and ^{1}H, ^{13}C HSQC experiments.
IR (ATR): ṽ = 2847 (w), 1625 (s), 1427 (m), 1278 (m), 1110 (m), 1069 (m), 1028 (m), 745 (m) cm^{-1}.
HRMS (ESI) for $C_{17}H_{20}NO_4^+$ [$(M+H)^+$] calculated: 302.1387, found 302.1387.

4.1.21 1-Morpholino-4-phenylbutan-1-one (1x)

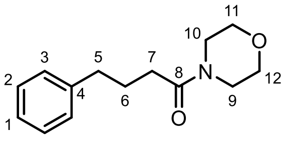

1x
$C_{14}H_{19}NO_2$
Mw = 233.31 g mol^{-1}

Prepared according to **GP1** from 4-phenylbutanoic acid (**S4f**, 851 mg, 5.00 mmol, 1.00 equiv), morpholine (**8j**, 1.29 mL, 15.0 mmol, 3.00 equiv), EDC·HCl (1.15 g, 6.00 mmol, 1.20 equiv) and DMAP (122 mg, 1.00 mmol, 20 mol%) in CH_2Cl_2 (10 mL). The reaction mixture was stirred for 16 h at rt. Amide **1x** was afforded as a colourless oil (801 mg, 3.43 mmol, 69%) and was used without further purification.

R*f* = 0.30 (cyclohexane/ethyl acetate = 7:3).
^{1}H NMR (600 MHz, $CDCl_3$): δ = 1.99 (p, $^3J_{6,5/7}$ = 7.5 Hz, 2H, H-6), 2.31 (t, $^3J_{7,6}$ = 7.5 Hz, 2H, H-7), 2.68 (t, $^3J_{5,6}$ = 7.5 Hz, 2H, H-5), 3.37 (t, $^3J_{8,11}$ = 4.8 Hz, 2H, H-9)*, 3.51–3.76 (m, 6H, H-10*, H-11, H-12), 7.16–7.23 (m, 3H, H-1, H-3), 7.28 (t, $^3J_{2,1/3}$ = 7.5 Hz, 2H, H-2) ppm.
^{13}C NMR (151 MHz, $CDCl_3$): δ = 26.7 (C-6), 32.3 (C-7), 35.4 (C-5), 42.0 (C-10)*, 46.1 (C-9)*, 66.8 (C-11)**, 67.1 (C-12)**, 126.1 (C-1), 128.5 (C-2), 128.6 (C-3), 141.7 (C-4), 171.5 (C-8) ppm.
HRMS (APCI) for $C_{14}H_{20}NO_2^+$ [(M+H)$^+$] calculated: 234.1489, found 234.1485.
The data is in accordance with literature.[27]

4.1.22 1-Morpholinoundec-10-en-1-one (1y)

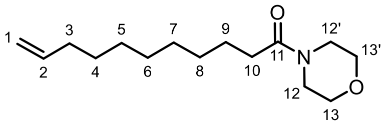

1y
$C_{15}H_{27}NO_2$
Mw = 253.39 g mol^{-1}

Prepared according to **GP1** from undec-10-enoic acid (**S4g**, 1.41 mL, 7.00 mmol, 1.00 equiv), morpholine (**8j**, 1.81 mL, 21.0 mmol, 3.00 equiv), EDC·HCl (1.61 g, 8.40 mmol, 1.20 equiv) and DMAP (171 mg, 1.40 mmol, 20 mol%) in CH_2Cl_2 (15 mL). The reaction mixture was stirred for 16 h at rt. Amide **1y** was afforded as a colourless oil (1.48 g, 5.84 mmol, 83%) and was used without further purification.

R*f* = 0.35 (cyclohexane/*tert*-butyl methyl ether = 1:1).
^{1}H NMR (600 MHz, $CDCl_3$): δ = 1.19–1.42 (m, 10H, H-4, H-5, H-6, H-7, H-8), 1.56–1.70 (m, 2H, H-9), 2.03 (td, $^3J_{3,4}$ = 7.7 Hz, $^3J_{3,2}$ = 7.1 Hz, 2H, H-3), 2.25–2.36 (m, 2H, H-10), 3.46 (t, $^3J_{12,13}$ = 4.8 Hz, 2H, H-12)*, 3.61 (t, $^3J_{12',13'}$ = 5.0 Hz, 2H, H-12')*, 3.66 (t, $^3J_{13/13',12/12'}$ = 4.8 Hz, 4H, H-13, H-13'), 4.90–5.01 (m, 2H, H-1), 5.75–5.85 (m, 1H, H-2) ppm.
^{13}C NMR (151 MHz, $CDCl_3$): δ = 25.4 (C-9), 29.0 (C-4*), 29.2 (C-5)*, 29.5 (C-6)*, 29.5 (C-7)*, 29.6 (C-8)*, 33.3 (C-10), 33.9 (C-3), 42.0 (C-12')*, 46.2 (C-12)*, 66.8 (C-13')*, 67.1 (C-13)*, 114.3 (C-1), 139.3 (C-2), 172.1 (C-11) ppm.
IR (ATR): ṽ = 2925 (m), 2855 (m), 1644 (s), 1427 (m), 1118 (m) cm^{-1}.
HRMS (APCI) for $C_{15}H_{28}O_2N^+$ [(M+H)$^+$] calculated: 254.2115, found: 254.2109.

4.1.23 1-Morpholinodecan-1-one (1z)

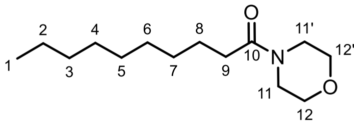

1z
$C_{14}H_{27}NO_2$
Mw = 241.38 g mol^{-1}

Prepared according to **GP1** from decanoic acid (**S4h**, 0.568 mL, 3.00 mmol, 1.00 equiv), morpholine (**8j**, 0.776 mL, 9.00 mmol, 3.00 equiv), EDC·HCl (690 mg, 3.60 mmol, 1.20 equiv) and DMAP (73.3 mg, 0.600 mmol, 20 mol%) in CH_2Cl_2 (5 mL). The reaction mixture was stirred for 16 h at rt. Purification by flash column chromatography on silica gel (CH_2Cl_2/MeOH = 19:1) yielded **1z** as a colourless oil (599 mg, 2.48 mmol, 83%).

R_f = 0.50 (CH_2Cl_2/MeOH = 19:1).
^{1}H NMR (600 MHz, $CDCl_3$): δ = 0.87 (t, $^3J_{1,2}$ = 6.9 Hz, 3H, H-1), 1.17–1.38 (m, 12H, H-2, H-3, H-4, H-5, H-6, H-7), 1.54–1.70 (m, 2H, H-8), 2.30 (t, $^3J_{9,8}$ = 7.7 Hz, 2H, H-9), 3.41–3.63 (m, 4H, H-11, H-11'), 3.65 (s, 4H, H-12, H-12') ppm.
^{13}C NMR (151 MHz, $CDCl_3$): δ = 14.2 (C-1), 22.8 (C-2)**, 25.4 (C-8), 29.2 (C-7)**, 29.4 (C-3)**, 29.5 (C-4)**, 29.6 (C-5)**, 32.0 (C-6)**, 33.3 (C-9), 42.0 (C-11)*, 46.2 (C-11')*, 67.0 (C-12, C-12'), 172.1 (C-10) ppm.
HRMS (APCI) for $C_{14}H_{28}O_2N^+$ [$(M+H)^+$] calculated: 242.2115, found: 242.2114.
The data is in accordance with literature.[28]

4.1.24 Morpholino(4-(phenylethynyl)phenyl)methanone (1aa)

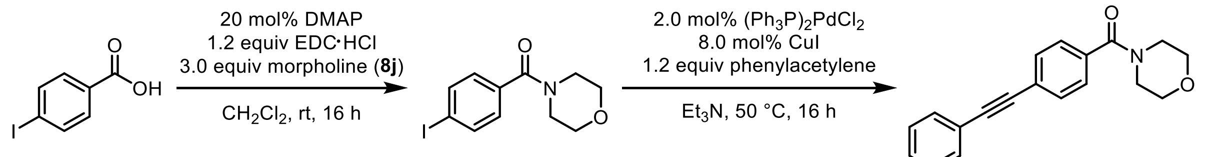

S9 S10 66% 1aa 68%

Scheme S2: Synthesis of morpholino(4-(phenylethynyl)phenyl)methanone (**1aa**).

Following general procedure **GP1**, a 100 mL Schlenk tube equipped with a magnetic stir bar, was charged with 4-iodobenzoic acid (**S9**, 2.48 g, 10.0 mmol, 1.00 equiv) and CH_2Cl_2 (20 mL). EDC·HCl (2.30 g, 12.0 mmol, 1.20 equiv), morpholine (**8j**, 2.59 mL, 30.0 mmol, 3.00 equiv) and 4-(dimethylamino)pyridine (244 mg, 2.00 mmol, 0.200 equiv) were added to the reaction mixture. The reaction mixture was stirred at rt until full conversion of **S9** was detected (monitored *via* TLC). After quenching the reaction by addition of H_2O (20 mL), the organic phase was washed with aq. HCl 1M (20 mL), sat. aq. NaCl (2 x 20 mL) and sat. aq. $NaHCO_3$ (20 mL). The organic layers were dried over $MgSO_4$, filtered and all volatiles were removed under reduced pressure. The obtained product, (4-iodophenyl)-morpholin-4-ylmethanone, (**S10**, 2.10 g, 6.62 mmol, 66%) was used in the next step without further purification.[29]

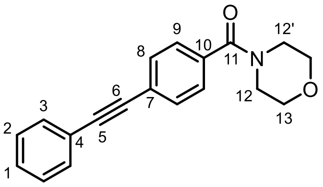

1aa
$C_{19}H_{17}NO_2$
Mw = 291.35 g mol^{-1}

In deference to a literature procedure,[30] a 25 mL Schlenk tube equipped with a magnetic stir bar, was charged with (4-iodophenyl)-morpholin-4-ylmethanone (**S10**, 634 mg, 2.00 mmol, 1.00 equiv) and Et_3N (10 mL). $(Ph_3P)_2PdCl_2$ (56.2 mg, 80.0 µmol, 2.0 mol%), CuI (30.5 mg, 160 µmol, 4.0 mol%) and phenylacetylene (264 µL, 2.40 mmol, 1.20 equiv) were added to the reaction mixture. The reaction mixture was stirred at rt until full conversion of **S10** was detected (monitored *via* TLC). Purification by flash column chromatography on silica gel (cyclohexane/ethyl acetate = 7:3) yielded morpholino(4-(phenylethynyl)phenyl)methanone (**1aa**, 396 mg, 1.36 mmol, 68%) as a white solid.

R_f = 0.30 (cyclohexane/ethyl acetate = 1:1).
m.p.: 122–124 °C.
^{1}H NMR (600 MHz, $CDCl_3$): 3.37–3.87 (m, 8H, H-12, H-13), 7.34–7.38 (m, 3H, H-1, H-3*), 7.40 (d, $^3J_{9,8}$ = 8.2 Hz, 2H, H-9), 7.51–7.56 (m, 2H, H-2*), 7.57 (d, $^3J_{8,9}$ = 8.3 Hz, 2H, H-8) ppm.
^{13}C NMR (151 MHz, $CDCl_3$): δ = 42.8 (C-12), 48.4 (C-12'), 67.0 (C-13), 88.6 (C-6), 91.2 (C-5), 122.9 (C-4), 125.3 (C-7), 127.4 (C-9), 128.6 (C-3)*, 128.8 (C-1), 131.8 (C-2)*, 131.9 (C-8), 134.9 (C-10), 169.9 (C-11) ppm.
IR (ATR): ṽ = 2855 (w), 1632 (m), 1423 (m), 1066 (w), 1010 (m), 834 (m), 760 (m), 693 (m) cm^{-1}.
HRMS (ESI) for $C_{19}H_{18}NO_2^+$ [(M+H)$^+$] calculated: 292.1332, found 292.1332.

4.1.25 (4-Methylpiperazin-1-yl)(phenyl)methanone (1ab)

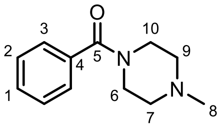

1ab
$C_{12}H_{16}N_2O$
Mw = 204.27 g mol^{-1}

Prepared according to **GP2** from benzoyl chloride (**S5a**, 0.580 mL, 5.00 mmol, 1.00 equiv), 1-methyl piperazine (**8ab**, 0.832 mL, 7.50 mmol, 1.50 equiv) and Et_3N (2.09 mL, 15.0 mmol, 3.00 equiv) in CH_2Cl_2 (10 mL). The reaction mixture was stirred for 16 h at rt. Purification by flash column chromatography on silica gel (cyclohexane/ethyl acetate = 8:2) yielded **1ab** as a colourless oil (300 mg, 1.47 mmol, 29%).

R_f = 0.25 (cyclohexane/ethyl acetate = 8:2).
^{1}H NMR (600 MHz, $CDCl_3$): δ = 2.25–2.39 (m, 5H, H-8, H-7)*, 2.48 (br s, 2H, H-9)*, 3.43 (s, 2H, H-6)**, 3.80 (br s, 2H, H-10)**, 7.40 (s, 5H, H-1, H-2, H-3) ppm.
^{13}C NMR (151 MHz, $CDCl_3$): δ = 42.2 (C-10)*, 46.2 (C-8), 47.8 (C-6)*, 54.9 (C-7)**, 55.5 (C-9)**, 127.2 (C-2)***, 128.6 (C-3)***, 129.8 (C-1), 136.0 (C-4), 170.4 (C-5) ppm.
HRMS (APCI) for $C_{12}H_{17}ON_2^+$ [(M+H)$^+$] calculated: 205.1335, found 205.1332.
The data is in accordance with literature.[20]

4.1.26 (4-Ethylpiperazin-1-yl)(phenyl)methanone (1ac)

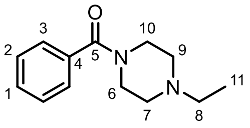

1ac
$C_{13}H_{18}N_2O$
Mw = 218.30 g mol^{-1}

Prepared according to **GP2** from benzoyl chloride (**S5a**, 0.580 mL, 5.00 mmol, 1.00 equiv), 1-ethyl piperazine (**8ac**, 0.953 mL, 7.50 mmol, 1.50 equiv) and Et_3N (2.09 mL, 15.0 mmol, 3.00 equiv) in CH_2Cl_2 (10 mL). The reaction mixture was stirred for 16 h at rt. Purification by flash column chromatography on silica gel (CH_2Cl_2/MeOH = 98:2) yielded **1ac** as a brown oil (1.07 g, 4.90 mmol, 98%).

$\mathbf{R_f}$ = 0.50 (CH_2Cl_2/MeOH = 19:1).
^{1}H NMR (600 MHz, $CDCl_3$): δ = 1.08 (t, $^3J_{11,8}$ = 7.2 Hz, 3H, H-11), 2.37 (s, 2H, H-9)*, 2.44 (q, $^3J_{8,11}$ = 7.2 Hz, 2H, H-8), 2.51 (s, 2H, H-7)*, 3.43 (br s, 2H, H-6)**, 3.80 (br s, 2H, H-10)**, 7.39 (s, 5H, H-1, H-2, H-3) ppm.
^{13}C NMR (151 MHz, $CDCl_3$): δ = 12.0 (C-11), 42.2 (C-10)*, 47.8 (C-6)*, 52.4 (C-8), 52.6 (C-7)**, 53.3 (C-9)**, 127.2 (C-2)***, 128.6 (C-3)***, 129.7 (C-1), 136.0 (C-4), 170.4 (C-5) ppm.
IR (ATR): ṽ = 2971 (w), 2810 (m), 2773 (m), 1632 (s), 1427 (s), 1289 (m), 1013 (s) cm^{-1}.
HRMS (APCI) for $C_{13}H_{19}ON_2^+$ [(M+H)$^+$] calculated: 219.1492, found 219.1489.
The data is in accordance with literature.[31]

4.1.27 (4-Isopropylpiperazin-1-yl)(phenyl)methanone (1ad)

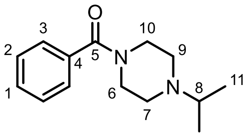

1ad
$C_{14}H_{20}N_2O$
Mw = 232.33 g mol^{-1}

Prepared according to **GP2** from benzoyl chloride (**S5a**, 0.580 mL, 5.00 mmol, 1.00 equiv), 1-isopropyl piperazine (**8ad**, 1.07 mL, 7.50 mmol, 1.50 equiv) and Et_3N (2.09 mL, 15.0 mmol, 3.00 equiv) in CH_2Cl_2 (10 mL). The reaction mixture was stirred for 16 h at rt. Purification by flash column chromatography on silica gel (CH_2Cl_2/MeOH = 98:2) yielded **1ad** as a yellow oil (700 mg, 3.01 mmol, 60%).

$\mathbf{R_f}$ = 0.55 (CH_2Cl_2/MeOH = 19:1).
^{1}H NMR (600 MHz, $CDCl_3$): δ = 1.04 (d, $^3J_{11,8}$ = 6.6 Hz, 6H, H-11), 2.44 (s, 2H, H-9)*, 2.59 (s, 2H, H-7)*, 2.72 (sept, $^3J_{8,11}$ = 6.4 Hz, 1H, H-8), 3.42 (s, 2H, H-6)**, 3.79 (s, 2H, H-10)**, 7.39 (s, 5H, H-1, H-2, H-3) ppm.
^{13}C NMR (151 MHz, $CDCl_3$): δ = 18.5 (C-11), 42.6 (C-10)*, 48.2 (C-6)*, 48.5 (C-7)**, 49.2 (C-9)**, 54.7 (C-8), 127.2 (C-2)***, 128.6 (C-3)***, 129.7 (C-1), 136.1 (C-4), 170.3 (C-5) ppm.
HRMS (APCI) for $C_{14}H_{21}ON_2^+$ [(M+H)$^+$] calculated: 233.1648, found 233.1644.
The data is in accordance with literature.[31]

4.1.28 Phenyl(4-(pyridin-2-yl)piperazin-1-yl)methanone (1ae)

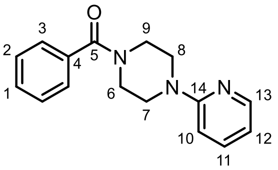

1ae
$C_{16}H_{17}N_3O$
Mw = 267.33 g mol^{-1}

Prepared according to **GP2** from benzoyl chloride (**S5a**, 0.580 mL, 5.00 mmol, 1.00 equiv), 1-(pyridin-2-yl)piperazine (**8ae**, 1.14 mL, 7.50 mmol, 1.50 equiv) and Et_3N (2.09 mL, 15.0 mmol, 3.00 equiv) in CH_2Cl_2 (10 mL). The reaction mixture was stirred for 16 h at rt. Purification by flash column chromatography on silica gel (CH_2Cl_2/MeOH = 98:2) yielded **1ae** as a white solid (1.23 g, 4.60 mmol, 92%).

R_f = 0.45 (CH_2Cl_2/MeOH = 19:1).
^{1}H NMR (600 MHz, $CDCl_3$): δ = 3.58 (br s, 6H, H-6, H-7, H-8)*, 3.90 (s, 2H, H-9)*, 6.63–6.71 (m, 2H, H-12 H-10), 7.43 (s, 5H, H-1, H-2, H-3), 7.52 (m_c, 1H, H-11), 8.16–8.23 (m, 1H, H-13) ppm.
^{13}C NMR (151 MHz, $CDCl_3$): δ = 42.1 (C-9)*, 45.6 (C-6, C-7)*, 47.5 (C-8)*, 107.5 (C-12)**, 114.2 (C-10)**, 127.2 (C-2)***, 128.7 (C-3)***, 130.0 (C-1), 135.8 (C-4), 137.8 (C-11), 148.2 (C-13), 159.3 (C-14), 170.7 (C-5) ppm.
HRMS (APCI) for $C_{16}H_{18}ON_3^+$ [$(M+H)^+$] calculated: 268.1444, found 268.1440.
The data is in accordance with literature.[24]

4.1.29 (2-(Methoxymethyl)phenyl)(piperidin-1-yl)methanone (S2)

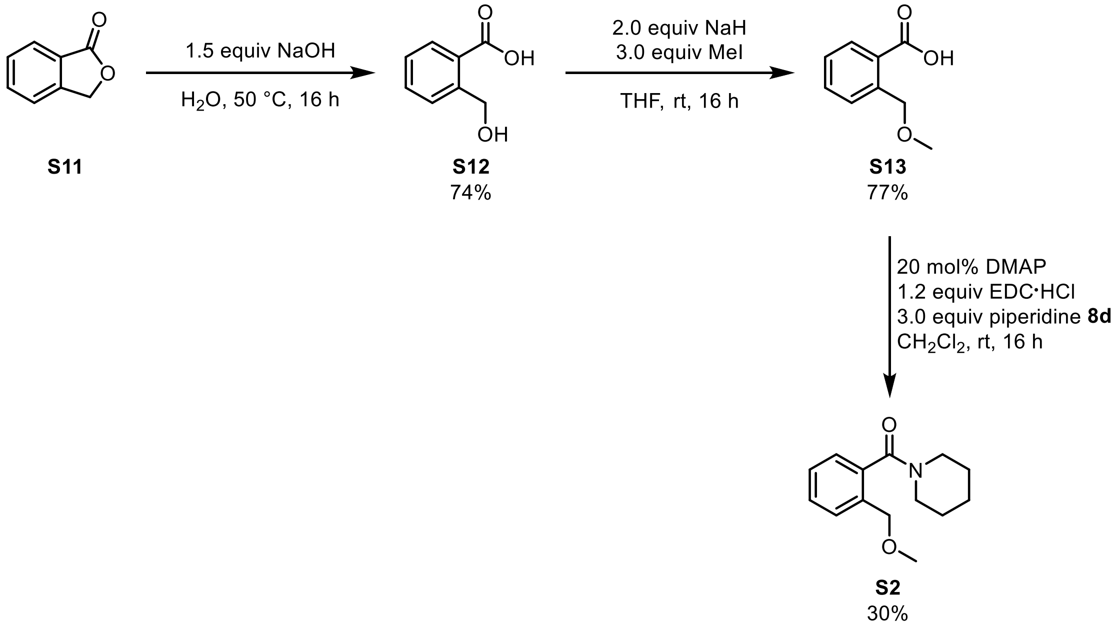

Scheme S3: Synthesis of (2-(methoxymethyl)phenyl)(piperidin-1-yl)methanone (**S2**).

According to a literature procedure,[32] a 100 mL Schlenk tube equipped with a magnetic stir bar was charged under N_2 atmosphere with a solution of NaOH (2.40 g, 60.0 mmol, 1.50 equiv) in H_2O (20 mL). Phthalide (**S11**, 5.45 g, 40.0 mmol, 1.00 equiv) was added and the reaction mixture was stirred at 50 °C for 16 h. After full conversion of **S11** (monitored *via* TLC), *conc.* HCl was added to the reaction mixture until pH = 1 was reached. The resulted white precipitate was filtered off with a glass frit (P4) and dried under reduced pressure. The product 2-(hydroxymethyl)benzoic acid (**S12**, 4.50 g, 29.6 mmol, 74%) was obtained as a white solid and was used in the next step without further purification.

According to a literature procedure,[33] a 50 mL Schlenk tube equipped with a magnetic stir bar, was charged under N_2 atmosphere with NaH (60% in mineral oil, 400 mg, 10.0 mmol, 2.00 equiv),dry THF (5 mL) and MeI (934 µL, 15.0 mmol, 3.00 equiv). A solution of 2-(hydroxymethyl)benzoic acid (**S12**, 761 mg, 5.00 mmol, 1.00 equiv) in dry THF (5 mL) was added and the reaction mixture was stirred at room temperature for 16 h. After full conversion of **S12** was reached (monitored *via* TLC), all insoluble salts were removed by filtration a glass frit (P4) and washed with EtOAc (5 mL) and H_2O (2 x 5 mL). The filtrate was acidified with *conc.* HCl until pH = 1 was reached. The formed precipitate was filtered off with a glass frit (P4), washed with H_2O (2 x 5 mL), dried under reduced pressure. 2-(Methoxymethyl)benzoic acid (**S13**, 640 mg, 3.85 mmol, 77%) was obtained as a white solid and used in the next step without further purification.

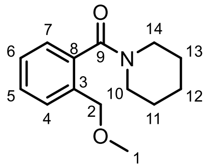

S2
$C_{14}H_{19}NO_2$
Mw = 233.31 g mol^{-1}

Following general procedure **GP1**, a 25 mL Schlenk tube equipped with a magnetic stir bar, was charged with 2-(methoxymethyl)benzoic acid (**S13**, 332 mg, 2.00 mmol, 1.00 equiv) and CH_2Cl_2 (4 mL). EDC·HCl (460 mg, 2.40 mmol, 1.20 equiv), piperidine (**8d**, 593 µL, 6.00 mmol, 3.00 equiv) and 4-(dimethylamino)pyridine (48.9 mg, 400 µmol, 20 mol%) were added to the reaction mixture. The reaction mixture was stirred at room temperature for 16 h. Purification by flash column chromatography on silica gel (cyclohexane/ethyl acetate = 7:3) yielded (2-(methoxymethyl)phenyl)(piperidin-1-yl)methanone (**S2**, 140 mg, 600 µmol, 30%) as a yellow oil.

$\mathbf{R}_f$ = 0.20 (cyclohexane/ethyl acetate = 7:3).
^{1}H NMR (600 MHz, $CDCl_3$): 1.43–1.50 (m, 2H, H-11)*, 1.66 (s, 4H, H-12, H-13*), 3.16–3.19 (m, 2H, H-10)**, 3.39 (s, 3H, H-1), 3.73 (s, 2H, H-14)**, 4.39 (d, $^2J_{2a,2b}$ = 11.8 Hz, 1H, H-2a), 4.55 (d, $^2J_{2b,2a}$ = 11.8 Hz, 1H, H-2b), 7.18 (dd, $^3J_{7,6}$ = 7.5 Hz, $^4J_{7,5}$ = 1.3 Hz, 1H, H-7), 7.30 (td, $^3J_{6,5/7}$ = 7.5 Hz, $^4J_{6,4}$ = 1.3 Hz, 1H, H-6), 7.35 (td, $^3J_{5,4/6}$ = 7.6 Hz, $^4J_{5,7}$ = 1.4 Hz, 1H, H-5), 7.43 (d, $^3J_{4,5}$ = 7.6 Hz, 1H, H-4) ppm.

^{13}**C NMR** (151 MHz, $CDCl_3$): δ = 24.7 (C-12)*, 25.8 (C-13)*, 26.4 (C-11), 42.6 (C-14), 48.5 (C-10), 58.8 (C-1) 72.3 (C-2), 125.9 (C-7), 127.8 (C-6), 128.9 (C-5), 128.9 (C-4), 135.2 (C-3), 136.3 (C-8), 169.5 (C-9) ppm.

IR (ATR): ṽ = 2933 (w), 2859 (w), 1625 (s), 1431 (s), 1278 (m), 1230 (m), 1103 (m), 999 (m), 749 (m) cm^{-1}.

HRMS (ESI) for $C_{14}H_{20}NO_2^+$ $[(M+H)^+]$ calculated: 234.1489, found 234.1489.

The data is in accordance with literature.[34]

4.1.30 Phenyl(tetrahydro-1,4-oxazepin-4(5*H*)-yl)methanone (S3)

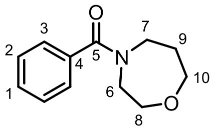

S3

$C_{12}H_{15}NO_2$

Mw = 205.26 g mol^{-1}

Prepared according to **GP2** from benzoyl chloride (**S5a**, 0.232 mL, 2.00 mmol, 1.00 equiv), 1,4-oxazepane (**S14**, 0.337 mL, 3.00 mmol, 1.50 equiv) and Et_3N (0.836 mL, 6.00 mmol, 3.00 equiv) in CH_2Cl_2 (4 mL). The reaction mixture was stirred for 16 h at rt. Amide **S3** was afforded as a colourless oil (388 mg, 1.89 mmol, 95%) and was used without further purification.

R_f = 0.30 (cyclohexane/ethyl acetate = 7:3).

^{1}**H NMR** (600 MHz, $CDCl_3$): δ = 1.81 (quint., $^3J_{9,7/10}$ = 5.9 Hz, 1H, H-9a), 2.04 (quint., $^3J_{9,7/10}$ = 5.9 Hz, 1H, H-9b), 3.42–3.57 (m, 2H, H-6a, H-7a), 3.63 (t, $^3J_{8a,6}$ = 4.9 Hz, 1H, H-8a), 3.77 (t, $^3J_{10a,9}$ = 5.4 Hz, 1H, H-10a), 3.79–3.90 (m, 4H, H-6b, H-7b, H-8b, H9b), 6.64–7.80 (m, 5H, H-1, H-2, H-3) ppm.

^{13}**C NMR** (151 MHz, $CDCl_3$): δ = 29.2 (C-9b), 31.1 (C-9a), 44.7 (C-6b), 48.4 (C-7a), 48.8 (C-7b), 52.5 (C-6a), 69.4(C-10b), 69.8 (C-10a), 71.0 (C-8b), 71.5 (C-8a), 126.6 (C-1a)*, 126.7 (C-1b)*, 128.6 (C-2)*, 129.5 (C-3)*, 136.7 (C-4), 171.7 (C-5a), 171.8 (C-5b) ppm.

The product was obtained as a mixture of amide *E* and *Z* isomers.

IR (ATR): ṽ = 2944 (w), 2862 (w), 1625 (s), 1420 (m), 1118 (m), 1073 (m), 704 (m) cm^{-1}.

HRMS (ESI) for $C_{12}H_{16}NO_2^+$ $[(M+H)^+]$ calculated: 206.1176, found: 206.1182.

4.1.31 2-[4-(Morpholine-4-carbonyl)phenyl]-1-(1-piperidyl)ethanone (11)

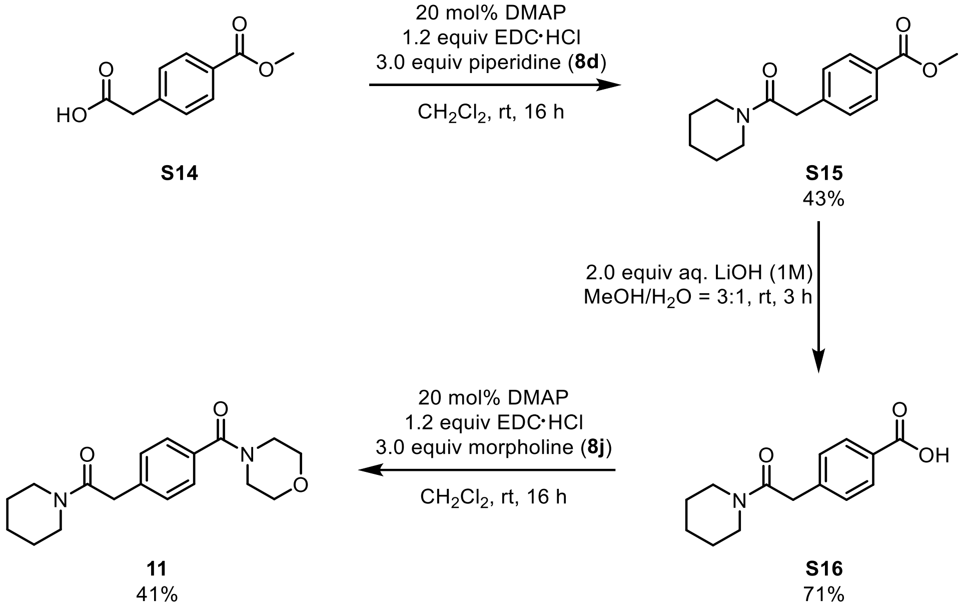

Scheme S4: Synthesis of 2-[4-(morpholine-4-carbonyl)phenyl]-1-(1-piperidyl)ethanone (**11**).

Following general procedure **GP1**, a 50 mL Schlenk tube equipped with a magnetic stir bar, was charged with 2-(4-methoxycarbonylphenyl)acetic acid (**S14**, 800 mg, 4.12 mmol, 1.00 equiv) and CH_2Cl_2 (8.0 mL). EDC·HCl (948 mg, 4.94 mmol, 1.20 equiv), piperidine (**8d**, 1.22 mL, 12.4 mmol, 3.00 equiv) and DMAP (101 mg, 0.824 mmol, 20 mol%) were added to the reaction mixture. The reaction mixture was stirred at rt until full conversion of **S14** was detected (monitored *via* TLC). After quenching the reaction by addition of H_2O (8.0 mL), the organic phase was washed with aq. HCl 1M (8.0 mL), sat. aq. NaCl (2 x 8.0 mL) and sat. aq. $NaHCO_3$ (8.0 mL). The combined organic layers were dried over $MgSO_4$, filtered and all volatiles were removed under reduced pressure. The obtained pale yellow solid product, methyl 4-(2-oxo-2-piperidin-1-ylethyl)benzoate, (**S15**, 458 g, 1.75 mmol, 43%) was used in the next step without further purification.[35]

A 10 mL Schlenk tube equipped with a magnetic stir bar was charged with methyl 4-(2-oxo-2-piperidin-1-ylethyl)benzoate, (**S15**, 458 mg, 1.75 mmol, 1.00 equiv) and MeOH (1.0 mL). An aq. LiOH solution (1M, 84.0 mg in 3.50 mL H_2O, 3.50 mmol, 2.00 equiv) was added dropwise and the reaction mixture was stirred for 3 h at rt. All volatiles were removed under reduced pressure and the residue was acidified with aq. HCl 2M until pH = 6 was reached. The product 4-[2-oxo-2-(1-piperidyl)ethyl]benzoic acid (**S16**, 308 mg, 1.25 mmol, 71%) was isolated after filtration through a glass frit (P4) as a white solid and was used in the next step without further purification.

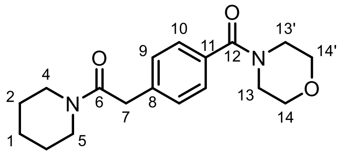

11
$C_{18}H_{24}N_2O_3$
Mw = 316.40 g mol^{-1}

Prepared according to **GP1** from methyl 4-(2-oxo-2-piperidin-1-ylethyl)benzoate, (**S16**, 308 g, 1.25 mmol, 1.00 equiv), morpholine (**8j**, 0.322 mL, 3.74 mmol, 3.00 equiv), EDC·HCl (287 g, 1.50 mmol, 1.20 equiv) and DMAP (30.4 mg, 0.25 mmol, 20 mol%) in CH_2Cl_2 (5 mL). The reaction mixture was stirred for 16 h at rt. Purification by flash column chromatography on silica gel (CH_2Cl_2/MeOH = 98:2) yielded **11** as a colourless oil (163 mg, 0.515 mmol, 41%).

R_f = 0.35 (CH_2Cl_2/MeOH = 95:5).
^{1}H NMR (600 MHz, $CDCl_3$): δ = 1.38–1.44 (m, 2H, H-2)**, 1.48–1.55 (m, 2H, H-3)**, 1.55–1.63 (m, 2H, H-1), 3.36–3.39 (m, 2H, H-4)*, 3.40–3.48 (m, 2H, H-13)**, 3.51–3.60 (m, 2H, H-5)*, 3.58–3.81 (m, 8H, H-7, H-14, H-14', H-13'**), 7.28 (d, $^3J_{9,10}$ = 7.9 Hz, 2H, H-9), 7.35 (d, $^3J_{10,9}$ = 8.1 Hz, 2H, H-10) ppm.
^{13}C NMR (151 MHz, $CDCl_3$): δ = 24.5 (C-1), 25.6 (C-3)**, 26.4 (C-2)**, 40.7 (C-7), 43.0 (C-5)*, 46.6 (C-13)***, 47.3 (C-4)*, 48.4 (C-13')***, 67.0 (C-14, C-14'), 127.6 (C-10), 129.0 (C-9), 133.7 (C-11), 137.6 (C-8), 168.7 (C-6), 170.4 (C-12) ppm.
IR (ATR): ṽ = 2855 (w), 1625 (s), 1427 (s), 1278 (m), 1110 (s), 1010 (s), 834 (m) cm^{-1}.
HRMS (APCI) for $C_{18}H_{25}N_2O_3^+$ [$(M+H)^+$] calculated: 317.1860, found: 317.1862.

4.1.32 3-(4-Benzoylpiperazin-1-yl)-1-(1-piperidyl)propan-1-one (13)

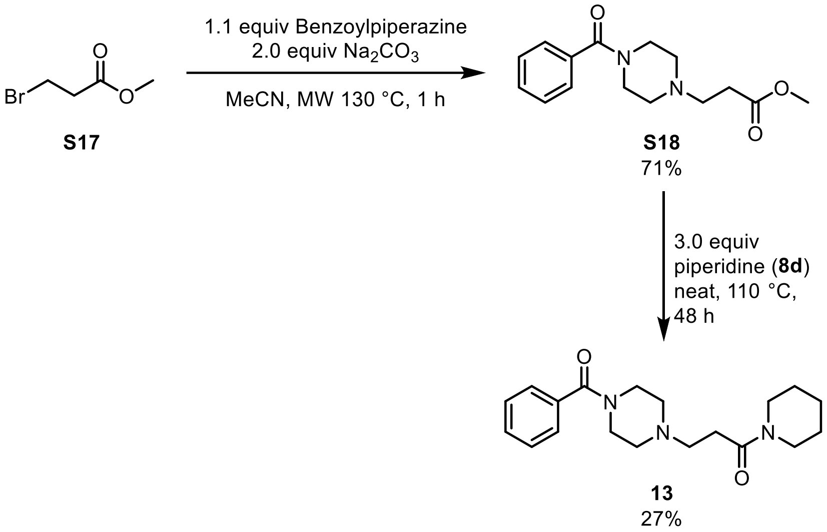

Scheme S5: Synthesis of 3-(4-benzoylpiperazin-1-yl)-1-(1-piperidyl)propan-1-one (**13**).

In deference to a literature procedure,[36] a 20 mL microwave vial was charged with methyl 3-bromopropanoate (**S17**, 1.00 g, 5.99 mmol, 1.00 equiv), phenyl(piperazin-1-yl)methanone (1.25 g, 6.59 mmol, 1.10 equiv), Na_2CO_3 (1.27 g, 12.0 mmol, 2.00 equiv) and MeCN (5.0 mL). The resulting suspension was stirred at 130 °C in a microwave reactor for 1 h. The reaction mixture allowed to cool to room temperature and diluted with EtOAc (10 mL). The suspension was washed with H_2O (2 x 10 mL), the organic phase was dried over $MgSO_4$ and all volatiles were removed under reduced pressure. The product methyl 3-(4-benzoylpiperazin-1-yl)propanoate (**S18**, 1.18 g, 4.27 mmol, 71%) was used in the next step without further purification.

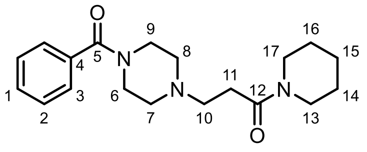

13
$C_{19}H_{27}N_3O_2$
Mw = 329.44 g mol^{-1}

A 10 mL pressure tube was charged with 3-(4-benzoylpiperazin-1-yl)propanoate (**S18**, 500 mg, 1.81 mmol, 1.00 equiv) and piperidine (**8d**, 0.536 mL, 5.43 mmol, 3.00 equiv) and the resulting solution was stirred at 110 °C for 48 h. The reaction mixture allowed to cool to room temperature and the obtained crude product was purified by flash-chromatography on silica gel (CH_2Cl_2/MeOH = 100:0 to 98:2) yielded 3-(4-benzoylpiperazin-1-yl)-1-(1-piperidyl)propan-1-one (**13**, 161 mg, 0.489 mmol, 27% yield) as an orange solid.

R*f* = 0.30 (CH_2Cl_2/MeOH = 95:05).

m.p.: 104–107 °C.

^{1}H NMR (600 MHz, $CDCl_3$): δ = 1.50–1.60 (m, 4H, H-14, H-16), 1.61–1.69 (m, 2H, H-15), 2.44 (br s, 2H, H-6)**, 2.49–2.67 (m, 4H, H-11, H-7**), 2.68–2.84 (m, 2H, H-10), 3.37–3.47 (m, 4H,

H-13***, H-8**), 3.55 (t, $^3J_{17,16}$ = 5.6 Hz, 2H, H-17)***, 3.79 (br s, 2H, H-9)**, 7.36–7.44 (m, 5H, H-1, H-2, H-3) ppm.
The integration for H-14, and H-16 is slightly higher due to overlapping resonances with the residual H_2O of $CDCl_3$.
^{13}C NMR (151 MHz, $CDCl_3$): δ = 24.7 (C-15), 25.7 (C-14)*, 26.7 (C-16)*, 31.0 (C-11), 42.2 (C-9)***, 42.8 (C-17)**, 46.8 (C-13)**, 47.8 (C-8)***, 52.9 (C-6)***, 53.8 (C-7)***, 54.2 (C-10), 127.2 (C-2)**, 128.6 (C-3)**, 129.8 (C-1), 135.9 (C-4), 169.7 (C-12), 170.4 (C-5) ppm.
IR (ATR): ṽ = 2930 (m), 2855 (m), 2807 (w), 1625 (s), 1435 (s), 1264 (m), 1129 (m), 999 (m), 704 (m) cm^{-1}.
HRMS (ESI) for $C_{19}H_{28}N_3O_2^+$ [$(M+H)^+$] calculated: 330.2176, found 330.2178.

4.1.33 *N,N*-diethyl-4-(morpholine-4-carbonyl)benzamide (16a)

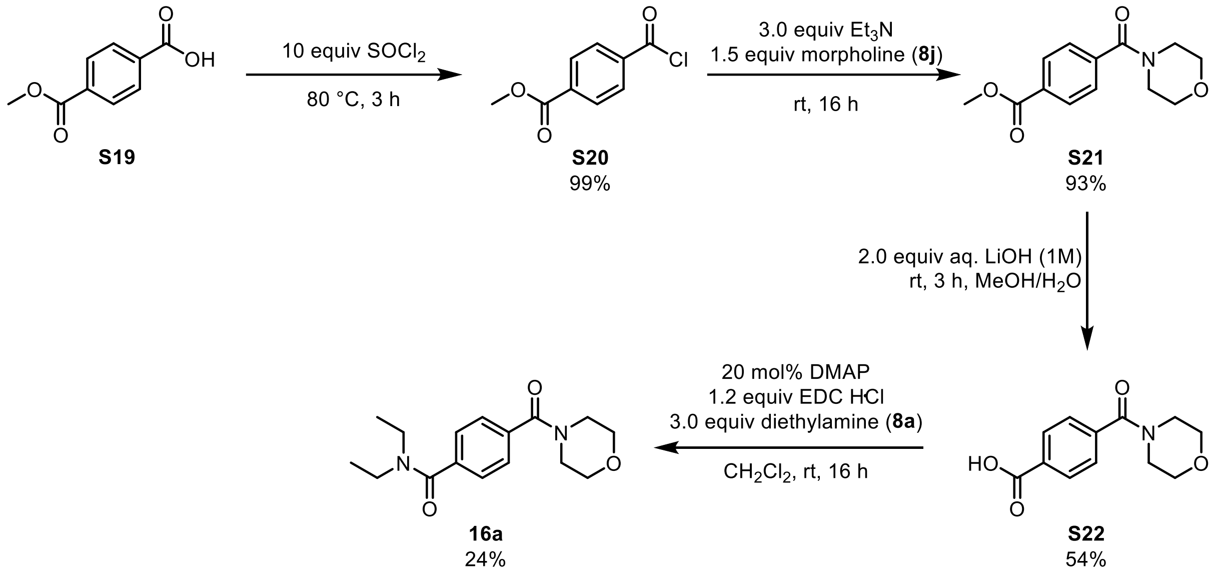

Scheme S6: Synthesis of *N,N*-diethyl-4-(morpholine-4-carbonyl)benzamide (**16a**).

A 100 mL Schlenk tube equipped with a magnetic stir bar, was charged with 4-methoxycarbonylbenzoic acid (**S19**, 5.04 g, 28.0 mmol, 1.00 equiv) and thionyl chloride (20.4 mL, 280 mmol, 10.0 equiv). The reaction mixture was stirred for 3 h at 80 °C. After full conversion of **S19** (monitored *via* TLC), excess of thionyl chloride was removed under reduced pressure and the crude product methyl 4-carbonochloridoylbenzoate (**S20**, 5.50 g, 27.7 mmol, 99%), was afforded as a white solid and was used in the next step without further purification.[37]

Following general procedure **GP2** and in deference to a literature procedure,[37] a 100 mL Schlenk tube equipped with a magnetic stir bar was charged with morpholine (**8j**, 3.62 mL, 42.0 mmol, 1.50 equiv), triethylamine (11.7 mL, 84.0 mmol, 3.00 equiv) and CH_2Cl_2 (10 mL). The resulting mixture was cooled down to 0 °C and a solution of methyl 4-

(chlorocarbonyl)benzoate (**S20**, 5.50 g, 28.0 mmol) in CH_2Cl_2 (10 mL) was added dropwise. After complete addition, the reaction mixture was stirred for 16 h at rt. Purification by flash column chromatography on silica gel (CH_2Cl_2/MeOH = 98:2), yielded methyl 4-(morpholine-4-carbonyl)benzoate (**S21**, 6.50 g, 26.1 mmol, 93%) as a white solid.

A 100 mL Schlenk tube equipped with a magnetic stir bar was charged with methyl 4-(morpholine-4-carbonyl)benzoate (**S21**, 6.50 g, 26.1 mmol, 1.00 equiv) and MeOH (20 mL). An aq. 1M LiOH solution (1.25 g in 52.2 mL H_2O, 52.2 mmol, 2.00 equiv) was added dropwise and the reaction mixture was stirred for 3 h at rt. All volatiles were removed under reduced pressure and the residue was acidified with aq. HCl 2M until pH = 6 was reached. The product 4-(morpholine-4-carbonyl)benzoic acid (**S22**, 3.30 g, 14.0 mmol, 54%) was isolated by filtration through a glass frit (P4) as a white solid. The product was used in the next step without further purification.[37] *N,N*-Diethyl-4-(morpholine-4-carbonyl)benzamide (16a)

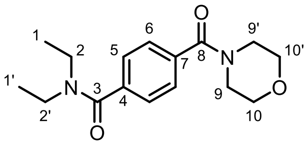

16a
$C_{16}H_{22}N_2O_3$
Mw = 290.36 g mol^{-1}

Following general procedure **GP1**, a 25 mL Schlenk tube equipped with a magnetic stir bar, was charged with 4-(morpholine-4-carbonyl)benzoic acid (**S22**, 706 mg, 3.00 mmol, 1.00 equiv) and CH_2Cl_2 (6.0 mL). EDC·HCl (690 mg, 3.60 mmol, 1.20 equiv), *N,N*-diethylamine (**8a**, 658 mg, 931 µL, 9.00 mmol, 3.00 equiv) and DMAP (73.3 mg, 600 µmol, 20 mol%) were added to the reaction mixture. The reaction mixture was stirred at rt until full conversion of **S22** was detected (monitored *via* TLC). After quenching the reaction by addition of H_2O (6 mL), the organic phase was washed with aq. HCl 1M (6 mL), sat. aq. NaCl (2 x 6 mL) and sat. aq. $NaHCO_3$ (6 mL). The organic layers were dried over $MgSO_4$ and filtered. All volatiles were removed under reduced pressure, yielding *N,N*-diethyl-4-(morpholine-4-carbonyl)benzamide (**16a**, 207 mg, 0.713 mmol, 24%) as an orange solid.

R*f* = 0.35 (CH_2Cl_2/MeOH = 98:2).

m.p.: 111–114 °C.

^{1}H NMR (600 MHz, $CDCl_3$): δ = 1.08 (s, 3H, H-1)*, 1.24 (s, 3H, H-1')*, 3.22 (s, 2H, H-2)**, 3.41 (s, 2H, H-9)***, 3.49–3.85 (m, 8H, H-2'**, H-9'***, H-10, H-10'), 7.33–7.68 (m, 4H, H-5, H-6) ppm.

^{13}C NMR (151 MHz, $CDCl_3$): δ = 13.0 (C-1'), 14.3 (C-1), 39.4 (C-2'), 42.7 (C-9), 43.3 (C-2), 48.3 (C-9'), 66.9 (C-10, C-10´), 126.7 (C-5)*, 127.3 (C-6)*, 136.1 (C-4)**, 138.9 (C-7)**, 169.9 (C-3)***, 170.5 (C-8)*** ppm.

IR (ATR): ṽ = 2981 (w), 2851 (w), 1621 (s), 1423 (m), 1274 (m), 1110 (s), 1010 (m), 834 (m), 730 (m) cm^{-1}.

HRMS (APCI) for $C_{16}H_{23}N_2O_3^+$ [(M+H)$^+$] calculated: 291.1703, found 291.1698.

4.1.34 *N*-(2-methoxyethyl)-*N*-methyl-4-(morpholine-4-carbonyl)benzamide (16b)

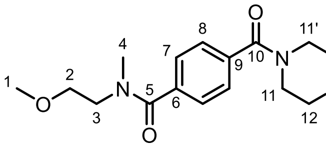

16b
$C_{16}H_{22}N_2O_4$
Mw = 306.36 g mol^{-1}

Following general procedure **GP1**, a 25 mL Schlenk tube equipped with a magnetic stir bar, was charged with 4-(morpholine-4-carbonyl)benzoic acid (**S22**, 261 mg, 1.11 mmol, 1.00 equiv) and CH_2Cl_2 (2.0 mL). EDC·HCl (255 mg, 1.33 mmol, 1.20 equiv), 2-methoxy-*N*-methylethan-1-amine (**8b**, 362 µL, 3.33 mmol, 3.00 equiv) and DMAP (27.1 mg, 222 µmol, 20 mol%) were added to the reaction mixture. The reaction mixture was stirred at rt until full conversion of **S22** was detected (monitored *via* TLC). Purification by flash column chromatography on silica gel (CH_2Cl_2/MeOH = 9.8:0.2) yielded *N*-(2-methoxyethyl)-*N*-methyl-4-(morpholine-4-carbonyl)benzamide (**16b**, 190 mg, 0.620 mmol, 56%) as a white solid.

R*f* = 0.60 (CH_2Cl_2/MeOH = 19:1).

m.p.: 85–83 °C.

^{1}H NMR (600 MHz, $CDCl_3$): δ = 2.89–3.21 (m, 3H, H-4), 3.21–3.49 (m, 7H, H-1, 1 x H-2a**, 1 x H-3***, H-11'*), 3.53–3.88 (m, 8H, H-12, H-11*, 1 x H-2b**, 1 x H-3***), 7.41–7.51 (m, 4H, H-7, H-8) ppm.

^{13}C NMR (151 MHz, $CDCl_3$): δ = 33.4 (C-4), 39.2 (C-4), 42.7 (C-3)*, 47.7 (C-11)*, 48.3 (C-3)*, 50.9 (C-11)*, 59.0 (C-1), 67.0 (C-12)**, 70.0 (C-2a), 71.0 (C-2b)**, 127.2 (C-7)***, 127.3 (C-7)***, 127.4 (C-8)***, 127.5 (C-8)***, 136.3 (C-6)****, 136.5 (C-6) ****, 138.2 (C-9) ****, 138.3 (C-9) ****, 169.9 (C-5), 170.7 (C-5), 171.6 (C-10) ppm. The product was isolated as a mixture of *E* and *Z* isomers.

IR (ATR): ṽ = 2862 (w), 1621 (s), 1431 (m), 1278 (m), 1110 (s), 1066 (m), 1010 (m), 835 (m) cm^{-1}.

HRMS (APCI) for $C_{16}H_{23}N_2O_4^+$ [$(M+H)^+$] calculated: 307.1652, found 307.1655.

4.1.35 (4-(Morpholine-4-carbonyl)phenyl)(piperidin-1-yl)methanone (16c)

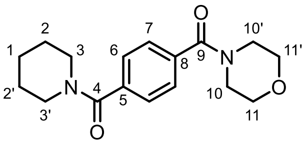

16c
$C_{17}H_{22}N_2O_3$
Mw = 302.37 g mol-1

Following general procedure **GP1**, a 25 mL Schlenk tube equipped with a magnetic stir bar was charged with 4-(morpholine-4-carbonyl)benzoic acid (**S22**, 706 mg, 3.00 mmol, 1.00 equiv) and CH_2Cl_2 (6.0 mL). EDC·HCl (690 mg, 3.60 mmol, 1.20 equiv), piperidine (**8d**, 889 µL, 9.00 mmol, 3.00 equiv) and DMAP (73.3 mg, 600 µmol, 20 mol%) were added to the reaction mixture. The reaction mixture was stirred at rt until full conversion of **S22** was detected (monitored *via* TLC). Purification by flash column chromatography on silica gel (CH_2Cl_2/MeOH = 98:2) yielded (4-(morpholine-4-carbonyl)phenyl)-piperidin-1-ylmethanone (**16c**, 381 mg, 1.26 mmol, 42%) as a white solid.

R*f* = 0.20 (CH_2Cl_2/MeOH = 19:1).

m.p.: 179–182 °C.

^{1}H NMR (600 MHz, $CDCl_3$): δ = 1.50 (s, 2H, H-2)*, 1.68 (s, 4H, H-1, H-2'*), 3.31 (s, 2H, H-3)**, 3.42 (s, 2H, H-11)***, 3.51–3.92 (m, 8H, H-3'**, H-11'***, H-10, H-10'), 7.43 (s, 4H, H-6, H-7) ppm.

^{13}C NMR (151 MHz, $CDCl_3$): δ = 24.6 (C-1), 25.7 (C-2)*, 26.7 (C-2)*, 42.7 (C-10), 43.3 (C-3)**, 48.3 (C-10')***, 48.9 (C-3')**, 67.0 (C-11)***, 127.2 (C-6)****, 127.4 (C-7)****, 136.4 (C-5)*****, 138.3 (C-8)*****, 169.5 (C-4)******, 169.8 (C-9)****** ppm.

IR (ATR): ṽ = 2925 (m), 2855 (m), 1617 (s), 1423 (s), 1274 (s), 1114 (s), 1002 (m), 842 (m), 730 (m) cm^{-1}.

HRMS (APCI) for $C_{17}H_{23}N_2O_3^+$ $[(M+H)^+]$ calculated: 303.1703, found 303.1697.

4.1.36 Ethyl methyl(2-morpholino-2-oxoethyl)carbamate (S25)

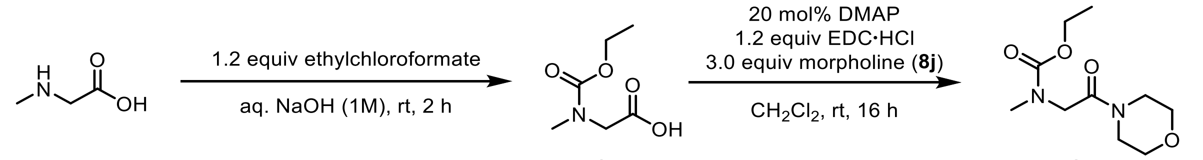

Scheme S7: Synthesis of ethyl methyl(2-morpholino-2-oxoethyl)carbamate (**S25**).

In deference to a literature procedure,[38] a 100 mL Schlenk tube equipped with a magnetic stir bar, was charged with *N*-methyl glycine (**S23**, 1.78 g, 20.0 mmol, 1.00 equiv) and aq. 1M NaOH-solution (20 mL). Ethyl chloroformate (2.28 mL, 24.0 mmol, 1.20 equiv) was added dropwise to the solution over 1 h at 0 °C. The pH of the mixture was kept at 9–10 by adding additional NaOH. The reaction mixture was stirred at room temperature for 1 h. After completion of the reaction (monitored *via* TLC), the reaction mixture was extracted with CH_2Cl_2 (20 mL). The aqueous layer was cooled to 0 °C, and acidified to pH = 1 by careful addition of conc. HCl. The mixture was extracted with CH_2Cl_2 (3 × 20 mL), the combined organic layers were dried over $MgSO_4$, filtered, and all volatiles were removed under reduced pressure to afford 2-[ethoxycarbonyl(methyl)amino]acetic acid (**S24**, 2.45 g, 15.2 mmol, 76%) as a colorless oil.

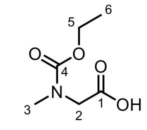

S24
$C_6H_{11}NO_4$
Mw = 161.16 g mol^{-1}

R_f = 0.20 (cyclohexane/ethyl acetate = 2:8).

^{1}H NMR (600 MHz, $CDCl_3$): 1.17–1.31 (m, 3H, H-6), 2.98 (s, 3H, H-3a, H-3b), 3.99–4.09 (m, 2H, H-2), 4.11–4.24 (m, 2H, H-5), 9.40 (s, 1H, COO–*H*) ppm.

The ^{1}H NMR spectra indicates the presence of two conformers, which can be seen with the peaks of H-6 at 1.17–1.31 ppm and of H-2 at 3.99–4.09 ppm.

^{13}C NMR (151 MHz, $CDCl_3$): δ = 14.7 (C-6), 35.5 (C-3a), 36.0 (C-3b), 50.3 (C-2a), 50.6 (C-2b), 62.1 (C-5a), 62.3 (C-5b), 156.5 (C-4a), 157.4 (C-4b), 174.8 (C-1a), 175.0 (C-1b) ppm.
IR (ATR): ṽ = 2985 (w), 2944 (w), 1658 (s), 1405 (m), 1226 (s), 1151 (s), 775 (m) cm^{-1}.
HRMS (APCI) $C_6H_{10}O_4N^-$ $[(M-H^+)^-]$ calculated: 160.0604, found 106.0608.

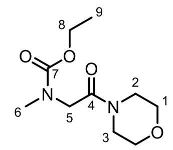

S25
$C_{10}H_{18}N_2O_4$
Mw = 230.2640 g mol^{-1}

Following general procedure **GP1**, a 100 mL Schlenk tube equipped with a magnetic stir bar, was charged with 2-[ethoxycarbonyl(methyl)amino]acetic acid (**S24**, 806 mg, 5.00 mmol, 1.00 equiv) and CH_2Cl_2 (10 mL). EDC·HCl (1.15 g, 6.00 mmol, 1.20 equiv), morpholine (**8j**, 1.29 mL, 15.0 mmol, 3.00 equiv) and DMAP (122 mg, 1.00 mmol, 20 mol%) were added to the reaction mixture. The reaction mixture was stirred at rt until full conversion of **S24** was detected (monitored *via* TLC). After quenching the reaction by addition of H_2O (10 mL), the organic phase was washed with aq. HCl 1M (10 mL), sat. aq. NaCl (2 x 10 mL) and sat. aq. $NaHCO_3$ (10 mL). The combined organic layers were dried over $MgSO_4$, filtered and all volatiles were removed under reduced pressure. The product, ethyl methyl(2-morpholino-2-oxoethyl)carbamate, (**S25**, 645 mg, 2.80 mmol, 56%) was obtained as a white solid and used in the next step without further purification.

R_f = 0.25 (cyclohexane/ethyl acetate = 1:1).
m.p: 39–42 °C.
^{1}H NMR (600 MHz, $CDCl_3$): 1.18–1.35 (m, 3H, H-9), 2.98 (s, 3H, H-6), 3.39–3.50 (m, 2H, H-2)*, 3.57–3.66 (m, 2H, H-3)*, 3.65–3.72 (m, 4H, H-1), 3.99–4.10 (m, 2H, H-5), 4.15 (q, $^3J_{8,9}$ = 7.3 Hz, 2H, H-8) ppm.
The ^{1}H NMR spectra indicates the presence of two conformers, which can be seen with the peaks of H-9 at 1.18–1.35 ppm and of H-5 at 3.99–4.10 ppm.
^{13}C NMR (151 MHz, $CDCl_3$): δ = 14.8 (C-9a), 14.9 (C-9b), 35.4 (C-6a), 36.1 (C-6b), 42.3 (C-3), 45.4 (C-2), 50.2 (C-5a), 50.5 (C-5b), 61.8 (C-8a), 61.9 (C-8b), 66.6 (C-1a), 67.0 (C-1b), 156.5 (C-7a), 157.2 (C-7b), 167.0 (C-4a), 167.2 (C-4b) ppm.
IR (ATR): ṽ = 2966 (w), 2862 (w), 1688 (s), 1654 (s), 1461 (m), 1401 (m), 1226 (s), 1151 (m), 1110 (m), 1021 (m), 846 (w) cm^{-1}.
HRMS (APCI) $C_{10}H_{18}N_2O_4^+$ $[(M+H)^+]$ calculated: 231.1339, found 231.1335.

4.1.37 Morpholino(phenyl-d5)methanone (S26)

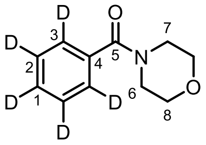

S26
$C_{11}H_8D_5NO_2$
Mw = 196.26 g mol^{-1}

Prepared according to **GP1** from benzoic-2,3,4,5,6-*d*$_5$ acid (**S4i**, 254 mg, 2.00 mmol, 1.00 equiv), morpholine (**8j**, 0.518 mL, 15.0 mmol, 3.00 equiv), EDC·HCl (460 g, 6.00 mmol, 1.20 equiv) and DMAP (48.9 mg, 1.00 mmol, 20 mol%) in CH_2Cl_2 (5.0 mL). The reaction mixture was stirred for 16 h at rt. Amide **S26** was afforded as a white solid (230 mg, 1.17 mmol, 59%) and was used without further purification.

R*f* = 0.15 (cyclohexane/ethyl acetate = 8:2).
m.p: 63–65 °C.
^{1}H NMR (600 MHz, $CDCl_3$): δ = 3.45 (s, 2H, H-6)*, 3.53–3.90 (m, 6H, H-7*, H-8) ppm.
^{2}H NMR (92 MHz, $CHCl_3$): δ = 7.23–7.60 (m, 5H, H-1, H-2, H-3) ppm.
^{13}C NMR (151 MHz, $CDCl_3$): δ = 42.7 (C-6)*, 48.4 (C-7)*, 67.1 (C-8), 126.3 (t, $^1J_{3,D}$ = 24.8 Hz, C-3), 128.3 (t, $^1J_{2,D}$ = 24.8 Hz, C-2), 129.5 (t, $^1J_{1,D}$ = 24.8 Hz, C-1), 135.3 (C-4), 170.6 (C-5) ppm.
IR (ATR): ṽ = 2922 (w), 2851 (w), 1617 (s), 1435 (s), 1267 (s), 1107 (s), 1006 (s), 842 (m) cm^{-1}.
HRMS (ESI) for $C_{11}H_9D_5NO_2^+$ [$(M+H)^+$] calculated: 197.1333, found 197.1336.

4.1.38 2-Methylene-4-morpholino-1-(1-piperidyl)butane-1,4-dione (S30)

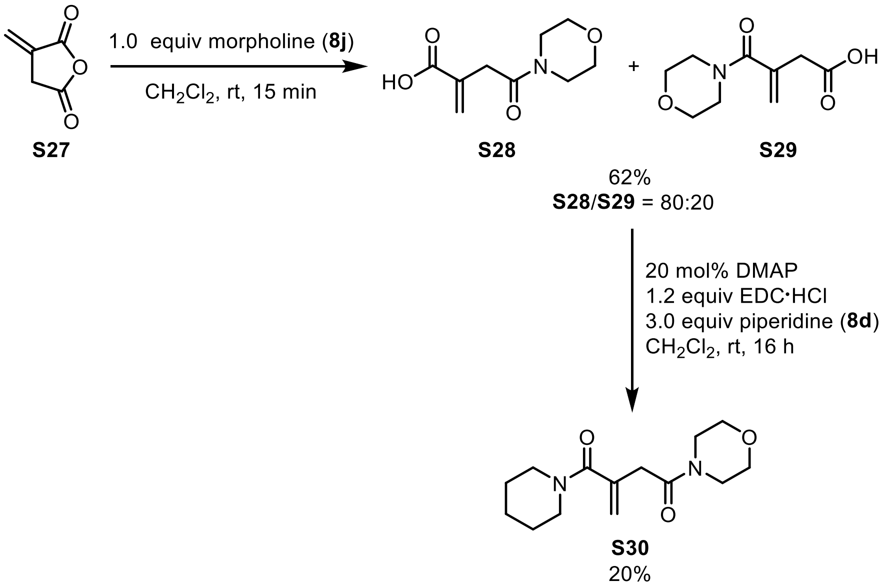

Scheme S8: Synthesis of 2-methylene-4-morpholino-1-(1-piperidyl)butane-1,4-dione (**S30**).

In deference to a literature procedure,[39] in a 100 mL Schlenk tube equipped with a magnetic stir bar, itaconic anhydride (**S27**, 1.00 g, 8.92 mmol, 1.00 equiv) was dissolved in CH_2Cl_2 (20 mL). After addition of morpholine (**8j**, 770 µL, 8.92 mmol, 1.00 equiv) in 10 min, the reaction mixture was stirred for additional 15 minutes. The reaction mixture was diluted with

CH_2Cl_2 (10 mL) and was washed with aq. HCl (1M, 20 mL) and sat. aq. NaCl (10 mL). The organic phase was dried over $MgSO_4$, filtered and all volatiles were removed under reduced pressure. Purification by flash column chromatography on silica gel (cyclohexane/ethyl acetate = 4:6), yielded 2-methylidene-4-morpholin-4-yl-4-oxobutanoic acid (**S28**) as a mixture with **S29** in a **S28**/**S29** = 80:20 ratio (1.11 g, 5.57 mmol, 62%).

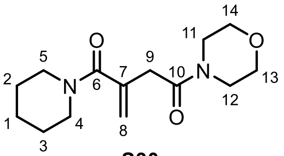

S30
$C_{14}H_{22}N_2O_3$
Mw = 266.34 g mol^{-1}

Prepared according to **GP1** from 2,4-dioxo-4-(1-piperidyl)butanoic acid (**S28**, 1.05 g, 5.27 mmol, 1.00 equiv), piperidine (**8d**, 1.56 mL, 15.8 mmol, 3.00 equiv), EDC·HCl (1.21 g, 6.33 mmol, 1.20 equiv) and DMAP (129 mg, 1.05 mmol, 20 mol%) in CH_2Cl_2 (10 mL). The reaction mixture was stirred for 16 h at rt. Purification by flash column chromatography on silica gel (CH_2Cl_2/MeOH = 98:2) yielded **S30** as a pale yellow solid (276 mg, 1.04 mmol, 20%).

R*f* = 0.27 (CH_2Cl_2/MeOH, 98:2).
m.p.: 54–57 °C.
^{1}H NMR (600MHz, $CDCl_3$): δ = 1.47–1.63 (m, 4H, H-2, H-3), 1.63–1.74 (m, 2H, H-1), 3.42 (s, 2H, H-9), 3.45–3.55 (m, 2H, H-12)*, 3.55–3.61 (m, 4H, H-4, H-5), 3.62–3.70 (m, 6H, H-11*, H-13, H-14), 5.20 (d, $^2J_{8,8'}$ = 0.9 Hz, 1H, H-8), 5.28 (q, $^4J_{8',9}$ = 1.2 Hz, $^2J_{8',8}$ = 1.2 Hz, 1H, H 8') ppm.
^{13}C NMR (151 MHz, $CDCl_3$): δ = 24.8 (C-1), 25.7 (C-2)*, 26.6 (C-3)*, 39.1 (C-9), 42.1 (C-4)***, 43.0 (C-5)***, 46.3 (C-12)*, 48.8 (C-11)*, 66.8 (C-13)**, 66.9 (C-14)**, 117.2 (C-8), 138.3 (C-7), 169.0 (C-10), 169.9 (C-6) ppm.
IR (ATR): ṽ = 2930 (w), 2855 (w), 1640 (s), 1606 (s), 1431 (s), 1215 (s), 1110 (s) cm^{-1}.
HRMS (ESI) for $C_{14}H_{23}N_2O_3^+$ [$(M+H)^+$] calculated: 267.1703, found: 267.1699.

4.1.39 2-Methylene-1-morpholino-4-(1-piperidyl)butane-1,4-dione (S33)

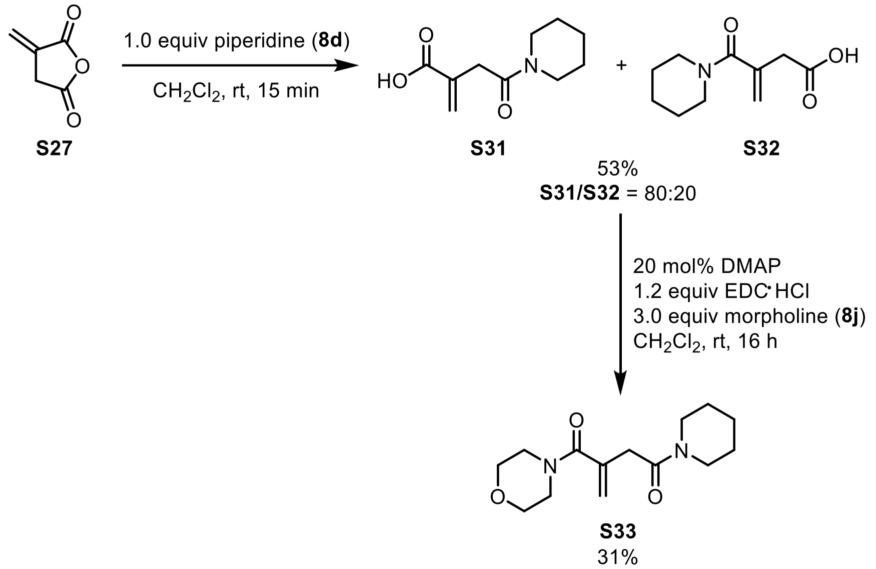

Scheme S9: Synthesis of 2-methylene-1-morpholino-4-(1-piperidyl)butane-1,4-dione (**S33**).

In deference to a literature procedure,[39] in a 100 mL Schlenk tube equipped with a magnetic stir bar, itaconic anhydride (**S27**, 1.00 g, 8.92 mmol, 1.00 equiv) was dissolved in CH_2Cl_2 (20 mL). After addition of piperidine (881 µL, 8.92 mmol, 1.00 equiv) in 10 min, the reaction mixture was stirred for additional 15 minutes. The reaction mixture was diluted with CH_2Cl_2 (10 mL) and was washed with aq. HCl (1M, 20 mL) and sat. aq. NaCl (10 mL). The organic phase was dried over $MgSO_4$, filtered and all volatiles were removed under reduced pressure. Purification by flash column chromatography on silica gel (cyclohexane/ethyl acetate = 4:6), yielded 4-(diethylamino)-2-methylene-4-oxo-butanoic acid (**S31**) as a mixture with **S32** in a **S31**/**S32** = 80:20 ratio (934 mg, 4.74 mmol, 53%).

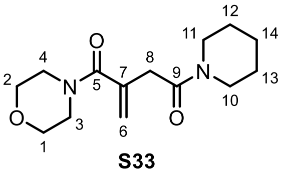

S33
$C_{15}H_{24}N_2O_2$
Mw = 264.37 g mol^{-1}

Prepared according to **GP1** from 4-(diethylamino)-2-methylene-4-oxo-butanoic acid (**S31**, 895 g, 4.54 mmol, 1.00 equiv), morpholine (**8j**, 1.17 mL, 13.6 mmol, 3.00 equiv), EDC·HCl (1.04 g, 5.44 mmol, 1.20 equiv) and DMAP (111 mg, 0.908 mmol, 20 mol%) in CH_2Cl_2 (10 mL). The reaction mixture was stirred for 16 h at rt. Purification by flash column chromatography on silica gel (CH_2Cl_2/MeOH = 98:2) yielded **S33** as a yellow oil (380 mg, 1.43 mmol, 31%).

R_f = 0.30 (CH_2Cl_2/MeOH = 98:2).
^{1}H NMR (600 MHz, $CDCl_3$): δ = 1.45–1.52 (m, 2H, H-12)*, 1.52–1.58 (m, 2H, H-13)*, 1.56–1.65 (m, 2H, H-14), 3.34–3.37 (m, 2H, H-10)**, 3.42 (s, 2H, H-8), 3.45–3.48 (m, 2H, H-11)**, 3.58–3.69 (m, 6H, H-1, H-2, H-3***), 3.77 (s, 2H, H-4)***, 5.14 (s, 1H, H-6), 5.26 (s, 1H, H-6') ppm.

^{13}**C NMR** (151 MHz, $CDCl_3$): δ = 24.5 (C-14), 25.5 (C-12)*, 26.4 (C-13)*, 39.2 (C-8), 42.3 (C-3)**, 42.7 (C-11)***, 46.8 (C-10)***, 48.4 (C-4)**, 66.8 (C-1)****, 67.1 (C-2)****, 117.9 (C-6), 138.3 (C-7), 168.4 (C-9), 170.5 (C-5) ppm.
IR (ATR): ṽ = 2933 (w), 2855 (w), 1614 (s), 1439 (s), 1219 (m), 1111 (s), 1032 (m) cm^{-1}.
HRMS (ESI) for $C_{14}H_{23}N_2O_3^+$ [$(M+H)^+$] calculated: 267.1703, found: 267.1700.

4.1.40 1-Morpholino-10-(1-piperidyl)decane-1,10-dione (S35)

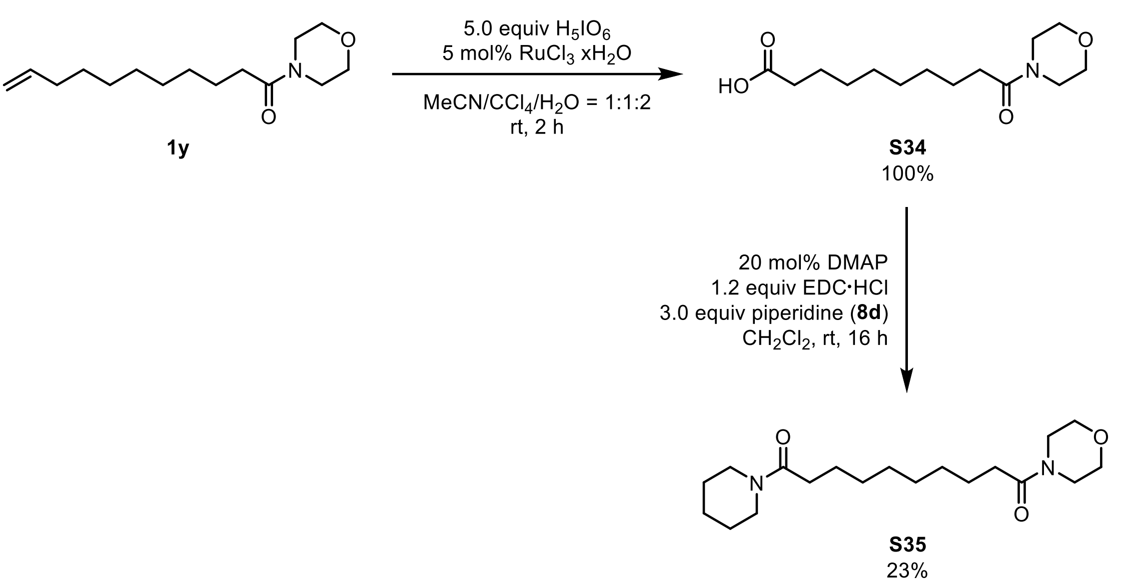

Scheme S10: Synthesis of 1-morpholino-10-(1-piperidyl)decane-1,10-dione (**S35**).

In deference to a literature procedure,[40] a 100 mL round bottom flask equipped with a magnetic stir bar was charged with 1-morpholinoundec-10-en-1-one (**1y**, 1.00 g, 3.95 mmol, 1.00 equiv) and a biphasic mixture of MeCN (14 mL), CCl_4 (14 mL) and H_2O (28 mL). In the biphasic mixture, $RuCl_3$ xH_2O (40.9 mg, 197 µmol, 5 mol%) and H_5IO_6 (4.50 g, 19.7 mmol, 5.00 equiv) were added and the reaction mixture was stirred at room temperature until full conversion of **1y** was observed (monitored *via* TLC, during the reaction, the reaction mixture turned form black to a yellow color). The reaction mixture was extracted with CH_2Cl_2 (3 x 20 mL) and the combined organic phases were dried over $MgSO_4$ and filtered through a pad of Celite© (3 cm). All volatiles were removed under reduced pressure to afford 10-morpholino-10-oxo-decanoic acid (**S34**, 1.05 g, 3.87 mmol, 98% yield) as a purple solid, which was used on the next step without further purification.

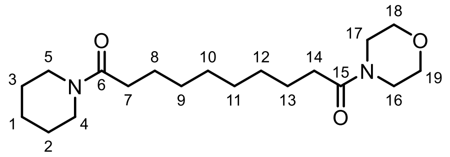

S35

$C_{19}H_{34}N_2O_3$

Mw = 338.49 g mol^{-1}

Prepared according to **GP1** from 10-morpholino-10-oxo-decanoic acid (**S34**, 1.00 g, 3.67 mmol, 1.00 equiv), piperidine (**8d**, 1.09 mL, 11.1 mmol, 3.00 equiv), EDC·HCl (848 mg, 4.42 mmol, 1.20 equiv) and DMAP (90.0 mg, 0.747 mmol, 20 mol%) in CH_2Cl_2 (10 mL). The reaction mixture was stirred for 16 h at rt. Purification by flash column chromatography on silica gel (CH_2Cl_2/MeOH = 98: 2) yielded **S35** as a purple solid (288 mg, 0.851 mmol, 23%).

R*f* = 0.45 (CH_2Cl_2/MeOH = 95:05).

m.p.: 65-68 °C.

^{1}H NMR (600MHz, $CDCl_3$): δ = 1.18–1.41 (m, 8H, H-1***, H-10***, H-11***, H-12***), 1.45–1.69 (m, 10H, H-2, H-3, H-8, H-13, H-9***), 2.19–2.39 (m, 4H, H-7, H-14), 3.33–3.41 (m, 2H, H-5)*, 3.43–3.48 (m, 2H, H-16)**, 3.50–3.56 (m, 2H, H-4)*, 3.61 (t, $^3J_{19,20}$ = 4.4 Hz, 2H, H-17)**, 3.63–3.71 (m, 4H, H-18, H-19) ppm.

^{13}C NMR (151 MHz, $CDCl_3$): δ = 24.7 (C-2)*****, 25.3 (C-3) *****, 25.6 (C-8) *****, 25.7 (C-13) *****, 26.7 (C-9) *****, 29.4 (C-1) *****, 29.4 (C-10) *****, 29.5 (C-11) *****, 29.6 (C-12) *****, 33.2 (C-7)****, 33.6 (C-14)****, 42.0 (C-17)**, 42.7 (C-4)***, 46.2 (C-16)**, 46.8 (C-5)***, 66.8 (C-18)*, 67.1 (C-19)*, 171.5 (C-6), 172.0 (C-15) ppm.

IR (ATR): ṽ = 2911 (m), 2851 (m), 1629 (s), 1424 (s, 1230 (s), 1118 (s), 1014 (m), 854 (m) cm^{-1}.

HRMS (ESI) for $C_{19}H_{34}N_2O_3^+$ [(M+H)$^+$] calculated: 339.2642, found: 339.2637.

4.1.41 [4-(Hydroxymethyl)phenyl]-(1-piperidyl)methanone (S36)

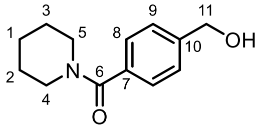

S36
$C_{13}H_{17}NO_2$
Mw = 219.28 g mol^{-1}

Prepared according to **GP1** from 4-(hydroxymethyl)benzoic acid (**S4j**, 304 mg, 2.00 mmol, 1.00 equiv), piperidine (**8d**, 0.593 mL, 6.00 mmol, 3.00 equiv), EDC·HCl (460 mg, 2.40 mmol, 1.20 equiv) and DMAP (48.9 mg, 0.400 mmol, 20 mol%) in CH_2Cl_2 (5.0 mL). The reaction mixture was stirred for 16 h at rt. Amide **S36** was afforded as a white solid (140 mg, 0.639 mmol, 32%) and was used without further purification.

R*f* = 0.30 (CH_2Cl_2/MeOH = 98:2).
m.p.: 112–115 °C.
^{1}H NMR (600 MHz, $CDCl_3$): δ = 1.49 (s, 2H, H-1), 1.61–1.72 (m, 4H, H-2, H-3), 2.57 (br s, 1H, O–*H*), 3.32 (s, 2H, H-4)*, 3.69 (s, 2H, H-5)*, 4.67 (s, 2H, H-11), 7.32 (s, 4H, H-8, H-9) ppm.
^{13}C NMR (151 MHz, $CDCl_3$): δ = 24.7 (C-2)*, 25.7 (C-3)*, 26.7 (C-1), 43.3 (C-5)**, 48.9 (C-4)**, 64.7 (C-11), 126.8 (C-8)***, 127.1 (C-9)***, 135.5 (C-7), 142.7 (C-10), 170.4 (C-6) ppm.
IR (ATR): ṽ = 3336 (s), 2937 (w), 2826 (w), 1595 (s), 1275 (m), 1047 (m), 1003 (m), 757 (m) cm^{-1}.
HRMS (APCI) for $C_{13}H_{18}NO_2^+$ [(M+H)$^+$] calculated: 220.1332, found 220.1333.

4.1.42 [4-(Hydroxymethyl)phenyl]-morpholino-methanone (S37)

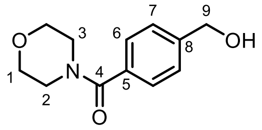

S37
$C_{12}H_{15}NO_3$
Mw = 221.26 g mol^{-1}

Prepared according to **GP1** from 4-(hydroxymethyl)benzoic acid (**S4j**, 761 mg, 5.00 mmol, 1.00 equiv), morpholine (**8j**, 1.29 mL, 15.0 mmol, 3.00 equiv), EDC·HCl (1.15 g, 6.00 mmol, 1.20 equiv) and DMAP (122 mg, 1.00 mmol, 20 mol%) in CH_2Cl_2 (10 mL). The reaction mixture was stirred for 16 h at rt. Amide **S37** was afforded as a white solid (140 mg, 0.633 mmol, 13%) and was used without further purification.

R*f* = 0.30 (CH_2Cl_2/MeOH = 98:2).
m.p.: 90–93 °C.
^{1}H NMR (600 MHz, $CDCl_3$): δ = 2.29 (s, 1H, O–*H*), 3.27–3.94 (m, 8H, H-1, H-2, H-3), 4.70 (d, $^3J_{9,OH}$ = 5.3 Hz, 2H, H-9), 7.37 (s, 4H, H-6, H-7) ppm.
^{13}C NMR (151 MHz, $CDCl_3$): δ = 42.8 (C-2)*, 48.4 (C-3)*, 64.7 (C-9), 67.0 (C-1), 126.9 (C-7), 127.4 (C-8), 134.4 (C-5), 143.1 (C-8), 170.5 (C-4) ppm.
IR (ATR): ṽ = 3455 (w), 2863 (w), 1617 (m), 1435 (m), 1275 (m), 1103 (m), 1010 (m), 839 (m), 757 (m) cm^{-1}.
HRMS (ESI) for $C_{12}H_{16}NO_3^+$ [(M+H)$^+$] calculated: 222.1125, found 222.1124.

4.1.43 Morpholino-2-[2-(piperidine-1-carbonyl)phenyl]ethanone (S94)

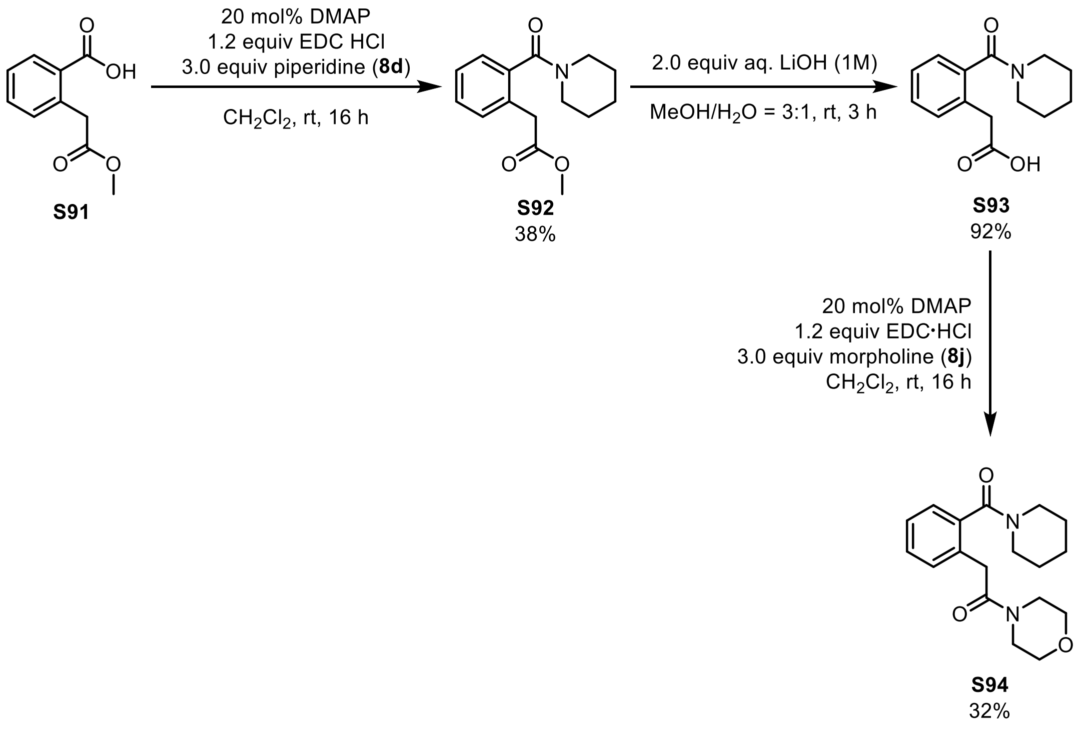

Scheme S11: Synthesis of 1-morpholino-2-[2-(piperidine-1-carbonyl)phenyl]ethanone (**S94**).

Following general procedure **GP1**, a 50 mL Schlenk tube equipped with a magnetic stir bar, was charged with 2-(2-methoxy-2-oxoethyl)benzoic acid (**S91**, 1.49 g, 7.62 mmol, 1,00 equiv.) and CH_2Cl_2 (20 mL). EDC·HCl (1.75 g, 9.15 mmol, 1.20 equiv), piperidine (**8d**, 2.26 mL, 22.9 mmol, 3.00 equiv) and DMAP (186 mg, 1.52 mmol, 20 mol%) were added to the reaction mixture. The reaction mixture was stirred at rt until full conversion of **S91** was detected (monitored *via* TLC). After quenching the reaction by addition of H_2O (15 mL), the organic phase was washed with aq. HCl 1M (15 mL), sat. aq. NaCl (2 x 15 mL) and sat. aq. $NaHCO_3$ (15 mL). The combined organic layers were dried over $MgSO_4$, filtered and all volatiles were removed under reduced pressure. The obtained brown oil product, methyl 2-[2-(piperidine-1-carbonyl)phenyl]acetate (**S92**, 760 mg, 2.91 mmol, 38%) was used in the next step without further purification.

A 10 mL Schlenk tube equipped with a magnetic stir bar was charged with, methyl 2-[2-(piperidine-1-carbonyl)phenyl]acetate (**S92**, 760 mg, 2.91 mmol, 1.00 equiv) and MeOH (5.0 mL). An aq. LiOH solution (1M, 139 mg in 5.82 mL H_2O, 5.82 mmol, 2.00 equiv) was added dropwise and the reaction mixture was stirred for 3 h at rt. All volatiles were removed under reduced pressure and the residue was acidified with aq. HCl 2M until pH = 2 was reached. The crude reaction mixture was extracted with EtOAc (10 mL) and the organic phase was dried with $MgSO_4$, filtered and all volatiles were removed under reduced pressure. The

product 2-[2-(piperidine-1-carbonyl)phenyl]acetic acid (**S93**, 665 mg, 2.69 mmol, 92%), as a yellow oil, was used in the next step without further purification.

R*f* = 0.30 (CH_2Cl_2/MeOH = 95:5).

^{1}H NMR (600 MHz, $CDCl_3$): δ = 1.48 (s, 1H, H-11a)*, 1.62 (s, 1H, H-11b)*, 1.72 (s, 4H, H-12, H-13*), 3.29–3.48 (m, 2H, H-10)**, 3.63 (s, 2H, H-7), 3.72–3.85 (m, 2H, H-14)**, 7.24–7.27 (m, 1H, H-4), 7.32 (t, $^3J_{2,3/1}$ = 7.3 Hz, 1H, H-2)***, 7.42 (t, $^3J_{3,2/4}$ = 7.5 Hz, 1H, H-3)***, 7.48 (d, $^3J_{1,2}$ = 7.7 Hz, 1H, H-1) ppm.

COO–*H* could not be detected by ^{1}H NMR. Traces of acetic acid at 2.08 ppm could be detected.

^{13}C NMR (151 MHz, $CDCl_3$): δ = 24.4 (C-12), 25.9 (C-13)*, 26.9 (C-11)*, 41.9 (C-7), 43.9 (C-14)**, 49.4 (C-10)**, 127.1 (C-4), 127.4 (C-2)***, 130.8 (C-3)***, 131.5 (C-1), 133.1 (C-5)****, 134.0 (C-6)****, 171.0 (C-9), 171.5 (C-8) ppm.

IR (ATR): ṽ = 2937 (w), 2859 (w), 1726 (m), 1584 (m), 1446 (m), 1279 (m) cm^{-1}.

HRMS (ESI) for $C_{14}H_{18}NO_3^+$ [$(M+H)^+$] calculated: 248.1281, found: 248.1289.

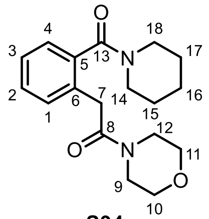

S94

$C_{18}H_{24}N_2O_3$
Mw = 316.40 g mol^{-1}

Prepared according to **GP1** from 2-[2-(piperidine-1-carbonyl)phenyl]acetic acid (**S93**, 665 mg, 2.69 mmol, 1.00 equiv), morpholine (**8j**, 0.696 mL, 8.07 mmol, 3.00 equiv), EDC·HCl (619 g, 3.23 mmol, 1.20 equiv) and DMAP (65.7 mg, 0.538 mmol, 20 mol%) in CH_2Cl_2 (6 mL). The reaction mixture was stirred for 16 h at rt. Purification by flash column chromatography on silica gel (CH_2Cl_2/MeOH = 99:1) yielded **S94** as a yellow solid (269 mg, 0.850 mmol, 32%).

R*f* = 0.30 (CH_2Cl_2/MeOH = 95:5).

m.p.: 78–80 °C.

^{1}H NMR (600 MHz, $CDCl_3$): δ = 1.46 (s, 2H, H-16), 1.65 (s, 4H, H-15, H-17), 3.10–3.28 (m, 2H, H-18)**, 3.37–3.52 (m, 2H, H-12***), 3.51–3.72 (m, 7H, H-9***, H-10, H-11, H-14a**), 3.71–3.81 (m, 3H, H-7, H-14b**), 7.17 (d, $^3J_{4,3}$ = 7.6 Hz, 1H, H-4), 7.24–7.28 (m, 1H, H-1), 7.28–7.31 (m, 1H, H-2), 7.30–7.36 (m, 1H, H-3) ppm.

N-Acetylmorpholine as inseparable sideproduct can be detected at 2.08 ppm. The integration for H-12 at 3.37–3.52 ppm is slightly higher due to overlapping resonances with *N*-Acetylmorpholine.

^{13}C NMR (151 MHz, $CDCl_3$): δ = 24.6 (C-15)*, 25.9 (C-17)*, 26.5 (C-16), 37.5 (C-7), 42.4 (C-9)***, 42.7 (C-14)**, 46.5 (C-12)***, 48.4 (C-18)**, 66.8 (C-11)****, 66.9 (C-10)****, 126.1 (C-4), 127.0 (C-1), 129.2 (C-3), 129.8 (C-2), 132.1 (C-5), 136.5 (C-6), 169.5 (C-13), 169.6 (C-8) ppm.

N-Acetylmorpholine as sideproduct can be detected at 21.3, 41.9, 46.8 and 169.2 ppm.

IR (ATR): ṽ = 2937 (w), 2848 (w), 1618 (s), 1424 (s), 1275 (m), 1230 (m), 1115 (s), 1003 (m), 842 (w), 775 (w), 746 (m) cm^{-1}.

HRMS (ESI) for $C_{18}H_{25}N_2O_3^+$ [$(M+H)^+$] calculated: 317.1860, found: 317.1866.

4.1.44 1-Morpholino-4-[4-(piperidine-1-carbonyl)phenyl]butan-1-one (S97)

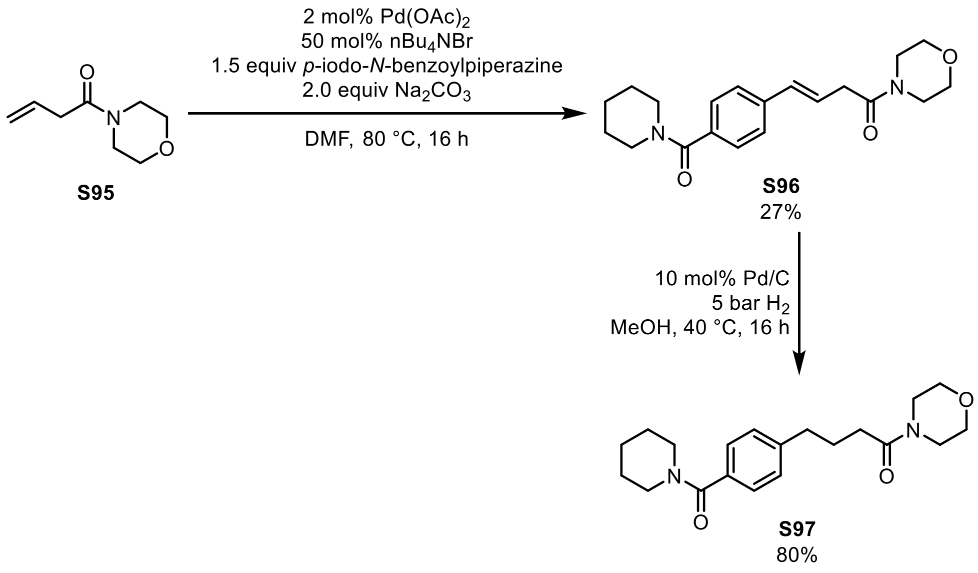

Scheme S12: Synthesis of 1-morpholino-4-[4-(piperidine-1-carbonyl)phenyl]butan-1-one (**S97**).

In deference to a literature procedure,[41] a 10 mL Schlenk tube equipped with a magnetic stir bar was charged under N_2 atmosphere with 1-morpholinobut-3-en-1-one (**S95**, 400 mg, 2.58 mmol, 1.00 equiv), $Pd(OAc)_2$ (11.6 mg, 51.5 µmol, 2 mol%), *n*-tetrabutylammoniumbromide (415 mg, 1.29 mmol, 50 mol%), *p*-iodo-*N*-benzoylpiperazine (1.22 g, 3.87 mmol, 1.50 equiv), Na_2CO_3 (546 mg, 5.16 mmol, 2.00 equiv) and DMF (2.0 mL). The reaction mixture was stirred at 80 °C for 16 h. After completion of the reaction, the reaction mixture was filtered through a pad of Celite© (3 cm), all volatiles were removed under reduced pressure. The obtained brown oil product, (*E*)-1-morpholino-4-[4-(piperidine-1-carbonyl)phenyl]but-3-en-1-one (**S96**, 237 mg, 0.692 mmol, 27%) was used in the next step without further purification.

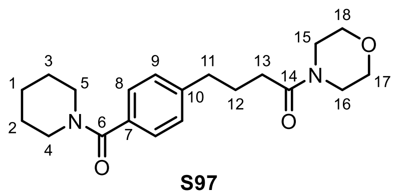

S97

$C_{20}H_{28}N_2O_3$

Mw = 344.46 g mol^{-1}

In deference to a literature procedure,[42] a 20 mL glass vial equipped with a magnetic stir bar was charged with (*E*)-1-morpholino-4-[4-(piperidine-1-carbonyl)phenyl]but-3-en-1-one (**S96**, 150 mg, 0.438 mmol, 1.00 equiv), Pd/C (10%, 46.4 mg, 43.8 µmmol, 10 mol%) and MeOH (4.0 mL). The vial is placed in an autoclave and the septum is pierced with a needle under N_2-counterflow. The autoclave is purged with H_2 (3 x 5 bar). The reaction mixture is stirred for 16 h at 40 °C under H_2-atmosphere (5 bar). The crude reaction mixture is filtered

over a plug of silica (eluent: CH_2Cl_2, 1 x 5 cm, 20 mL) and all volatiles are removed under reduced pressure. The product 1-morpholino-4-[4-(piperidine-1-carbonyl)phenyl]butan-1-one (**S97**, 120 mg, 0.349 mmol, 80%) was isolated as a yellow oil.

R*f* = 0.20 (CH_2Cl_2/MeOH = 95:5).
^{1}H NMR (600 MHz, $CDCl_3$): δ = 1.51 (s, 2H, H-2)*, 1.67 (s, 4H, H-1, H-3*), 1.98 (q, $^2J_{12,11/13}$ = 7.5 Hz, 2H, H-12), 2.29 (t, $^3J_{13,12}$ = 7.5 Hz, 2H, H-13), 2.70 (t, $^3J_{11,12}$ = 7.4 Hz, 2H, H-11), 3.30–3.45 (m, 4H, H-4**, H-15***), 3.59–3.77 (m, 8H, H-17, H-18, H-5**, H-16***), 7.20 (d, $^3J_{9,8}$ = 7.8 Hz, 2H, H-9), 7.31 (d, $^3J_{8,9}$ = 7.8 Hz, 2H, H-8) ppm.
^{13}C NMR (151 MHz, $CDCl_3$): δ = 24.7 (C-1), 25.8 (C-3)*, 26.4 (C-12), 26.7 (C-2)*, 32.0 (C-13), 35.2 (C-11), 42.0 (C-16)***, 43.3 (C-5)****, 46.0 (C-15)***, 48.9 (C-4)****, 66.8 (C-17)**, 67.0 (C-18)**, 127.2 (C-8), 128.6 (C-9), 134.3 (C-7), 143.2 (C-10), 170.5 (C-6), 171.3 (C-14) ppm.
IR (ATR): ṽ = 2937.1 (w), 2858.9 (w), 1617.7 (s), 1435.0 (s), 1274.7 (m), 1114.5 (m), 913.2 (m), 726.8 (s) cm^{-1}.
GC-MS (EI) for $C_{20}H_{28}N_2O_3^+$ [M^+] calculated: 344.2, found: 344.2.

4.1.45 1-Cyclopropyl-6-fluoro-7-(4-methylpiperazin-1-yl)-3-(morpholine-4-carbonyl)quinolin-4-one (S100)

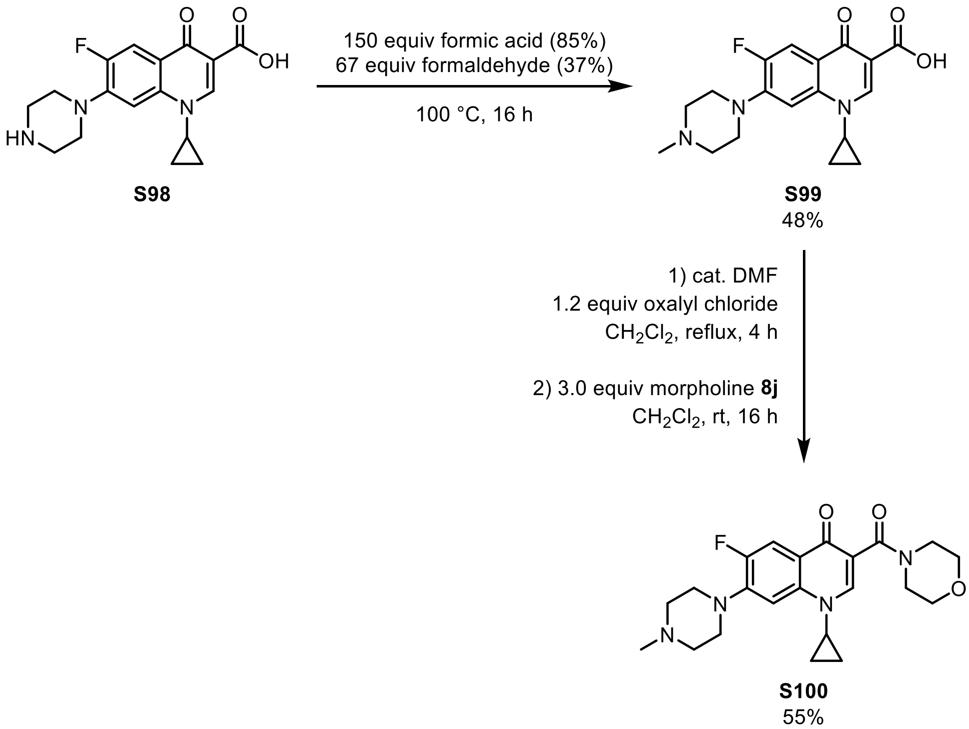

Scheme S13: Synthesis of 1-cyclopropyl-6-fluoro-7-(4-methylpiperazin-1-yl)-3-(morpholine-4-carbonyl)quinolin-4-one (**S100**)

In deference to a literature procedure,[43] a 100 mL round bottom flask equipped with a magnetic stir bar was charged with ciprofloxacin (**S98**, 1.00 g, 3.02 mmol, 1.00 equiv), formic acid (85%, 10.0 mL, 450 mmol, 150 equiv) and formaldehyde (37%, 20.0 mL, 200 mmol, 67 equiv) and the reaction mixture was stirred at 100 °C for 16 h. The reaction solution was then cooled and all volatiles were removed under reduced pressure. The residue was dissolved in H_2O (5.0 mL), neutralised with aq. NaOH (1M) and extracted with CH_2Cl_2 (3 x 20 ml). The combined organic layers were washed with brine (1 x 10 mL), dried over $MgSO_4$ and filtered. All volatiles were removed under reduced pressure yielding 1-cyclopropyl-6-fluoro-7-(4-methylpiperazin-1-yl)-4-oxo-1,4-dihydroquinoline-3-carboxylic acid (**S99**, 500 mg, 1.45 mmol, 48%) as a white solid, which was used in the next step without further purification.

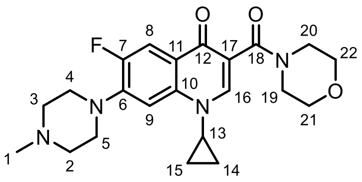

S100
$C_{22}H_{27}FN_4O_3$
Mw = 414.48 g mol^{-1}

In deference to a literature procedure,[44] a 25 mL Schlenk tube equipped with a magnetic stir bar, was charged with 1-cyclopropyl-6-fluoro-7-(4-methylpiperazin-1-yl)-4-oxo-1,4-dihydroquinoline-3-carboxylic acid (**S99**, 200 mg, 0.579 mmol, 1.00 equiv), 1 drop DMF and CH_2Cl_2 (5.0 mL). Oxalylchloride (60.0 µL, 700 µmol, 1.20 equiv) was added dropwise and the reaction mixture was stirred at reflux for 4 h. The reaction solution was then cooled and all volatiles were removed under reduced pressure. The residue was dissolved in CH_2Cl_2 (10 mL) and morpholine (**8j**, 0.15 mL, 1.74 mmol, 3.00 equiv) was added dropwise and the reaction mixture was stirred at rt for 16 h. Purification by flash column chromatography on silica gel (CH_2Cl_2/MeOH = 98:2) yielded **S100** as a pale yellow solid (132 mg, 0.319 mmol, 55%).

R*f* = 0.15 (CH_2Cl_2/MeOH = 98:2).
m.p: 79–82 °C.
^{1}H NMR (600 MHz, $CDCl_3$): δ = 1.07–1.20 (m, 2H, H-15)*, 1.25–1.36 (m, 2H, H-14)*, 2.39 (s, 3H, H-1), 2.54–2.74 (m, 4H, H-2, H-3), 3.30 (t, $^3J_{4,3}$ = 4.9 Hz, 4H, H-4, H-5), 3.36–3.46 (m, 3H, H-13, H-19**), 3.60–3.90 (m, 6H, H-20**, H-21, H-22), 7.28 (d, $^4J_{9,F}$ = 7.1 Hz, 1H, H-9), 7.99 (d, $^3J_{8,F}$ = 13.3 Hz, 1H, H-8), 8.08 (s, 1H, H-16) ppm.
^{13}C NMR (151 MHz, $CDCl_3$): δ = 8.2 (C-14, C-15), 34.5 (C-13), 43.2 (C-20)*, 46.3 (C-1), 48.2 (C-19)*, 50.1 (C-4, C-5), 55.0 (C-2, C-3), 67.0 (C-21)**, 67.5 (C-22)**, 104.7 (d, $^3J_{C,F}$ = 2.9 Hz, C-9), 112.9 (d, $^2J_{C,F}$ = 23.0 Hz, C-8), 117.2 (C-17), 121.8 (d, $^3J_{C,F}$ = 7.0 Hz, C-11)***, 138.5 (C-17), 144.8 (d, $^3J_{C,F}$ = 10.8 Hz, C-6)***, 145.2 (C-16), 153.5 (d, $^1J_{C,F}$ = 248.9 Hz, C-7), 166.2 (C-18), 172.4 (C-12) ppm.
C-6 could not be detected by ^{13}C NMR.
^{19}F NMR (564 MHz, $CDCl_3$): δ = – 123.9 ppm.
IR (ATR): ṽ = 2843.9 (w), 2795.5 (w), 1625.1 (m), 1584.1 (m), 1479.7 (m), 1256.1 (m), 1110.7 (m), 1006.4 (m) cm^{-1}.

HRMS (ESI) for $C_{22}H_{28}N_4O_3F^+$ [$(M+H)^+$] calculated: 415.2140, found: 415.2142.

4.1.46 7-[4-(4-Chlorobenzoyl)piperazin-1-yl]-1-cyclopropyl-6-fluoro-3-(piperidine-1-carbonyl)quinolin-4-one (S102)

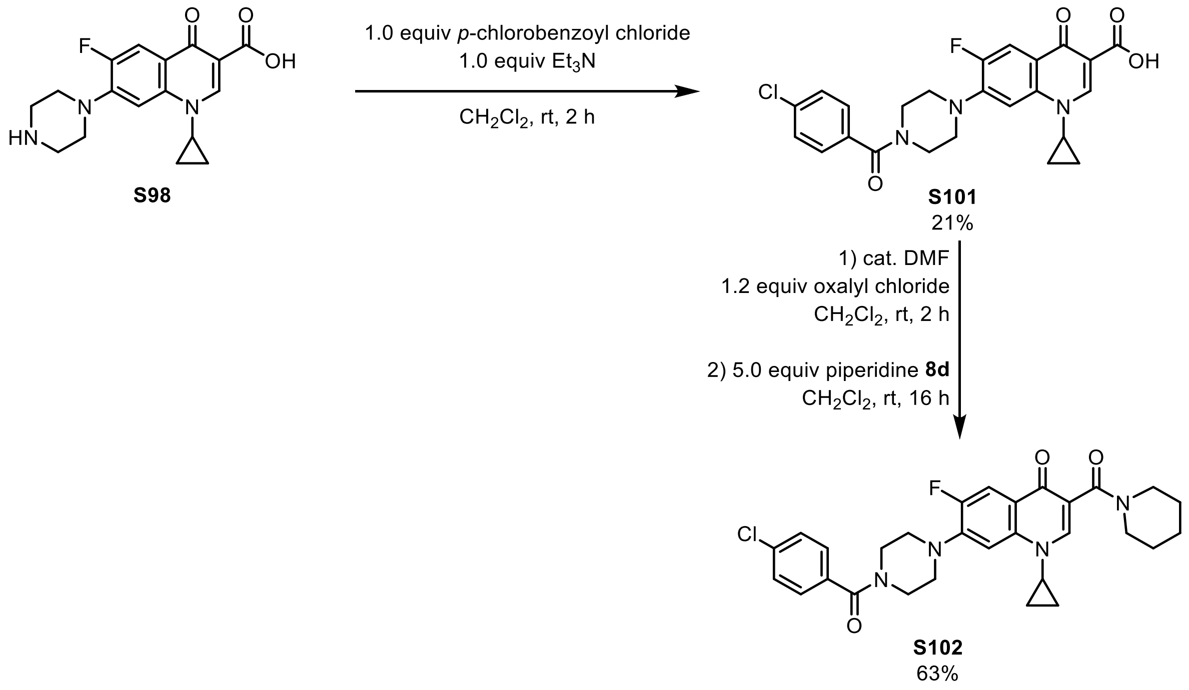

Scheme S14: Synthesis of 7-[4-(4-chlorobenzoyl)piperazin-1-yl]-1-cyclopropyl-6-fluoro-3-(piperidine-1-carbonyl)quinolin-4-one (**S102**).

In deference to a literature procedure,[45] a Schlenk tube equipped with a magnetic stir bar was charged with ciprofloxacin (**S98**, 5.00 g, 15.1 mmol, 1.00 equiv), and CH_2Cl_2 (60 mL). *p*-Chlorobenzoyl chloride (2.61 g, 15.0 mmol, 1.00 equiv) and Et_3N (2.00 mL, 15.0 mmol, 1.00 equiv) were added and the reaction mixture was stirred at room temperature for 2 h. After complete consumption of *p*-chlorobenzoyl chloride (monitored *via* TLC, CH_2Cl_2/MeOH = 97:3), the reaction mixture was quenched by addition of sat. aq. NH_4Cl solution (10 mL) and diluted with CH_2Cl_2 (40 mL). The organic layer was separated and was washed with sat. aq. NH_4Cl solution (3 × 20 mL) and dried over $MgSO_4$. After filtration all volatiles were removed under reduced pressure. The crude product was purified by flash column chromatography on silica gel (CH_2Cl_2/MeOH = 97:3) to afford **S101** as colorless solid (1.51 g, 3.21 mmol, 21% yield). The purified acid **S101** was directly used for the next step.

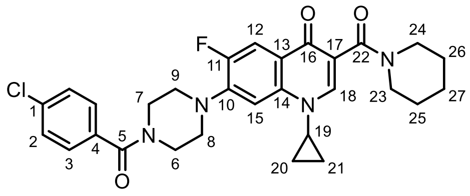

S102
$C_{29}H_{30}ClFN_4O_3$
Mw = 537.03 g mol^{-1}

In deference to a literature procedure,[44] a Schlenk tube equipped with a magnetic stir bar was charged with 7-(4-(4-chlorobenzoyl)piperazin-1-yl)-1-cyclopropyl-6-fluoro-4-oxo-1,4-dihydroquinoline-3-carboxylic acid (**S101**, 1.50 g, 3.20 mmol, 1.00 equiv), 1 drop DMF and CH_2Cl_2 (20 mL). Oxalyl chloride (0.33 mL, 3.84 mmol, 1.20 equiv) was added dropwise over 5 min and the reaction mixture was stirred at room temperature for 2 h. All volatiles were removed under reduced pressure. The crude mixture was dissolved in CH_2Cl_2 (20 mL). Piperidine (**8d**, 1.60 mL, 16.0 mmol, 1.20 equiv) was added and the reaction mixture was stirred at room temperature for 16 h. All volatiles were removed under reduced pressure. The crude product was purified by flash column chromatography on silica gel (CH_2Cl_2/MeOH = 97:3) to afford **S102** as yellow solid (1.08 g, 2.06 mmol, 63% yield).

R_f = 0.15 (CH_2Cl_2/MeOH = 98:2).
m.p: 137–140 °C.
^{1}H NMR (600 MHz, $CDCl_3$): δ = 1.09–1.18 (m, 2H, H-20)*, 1.25–1.33 (m, 2H, H-21)*, 1.53–1.63 (m, 2H, H-25)**, 1.65 (s, 4H, H-26**, H-27), 3.00–3.91 (m, 13H, H-6, H-7, H-8, H-9, H-19, H-23, H-24), 7.28 (d, $^3J_{15,F}$ = 7.0 Hz, 1H, H-15), 7.36–7.48 (m, 4H, H-2, H-3), 7.98–8.08 (m, 2H, H-12, H-18) ppm.
^{13}C NMR (151 MHz, $CDCl_3$): δ = 8.3 (C-20, C-21), 24.7 (C-26)*, 25.8 (C-27)*, 26.8 (C-25)*, 34.3 (C-19), 43.5 (C-6, C-7, C-8, C-9)**, 48.8 (C-23, C-24)**, 105.0 (C-15), 113.3 (d, $^2J_{C,F}$ = 22.8 Hz, C-12), 118.7 (C-17), 122.4 (d, $^3J_{C,F}$ = 22.8 Hz, C-13), 128.9 (C-2)***, 129.1 (C-3)***, 133.7 (C-1)****, 136.4 (C-4)****, 138.5 (C-10)*****, 144.0 (C-11)*****, 144.4 (C-18), 152.5 (C-14), 165.6 (C-22), 169.6 (C-5), 172.4 (C-16) ppm.
^{19}F NMR (564 MHz, $CDCl_3$): δ = – 125.0 ppm.
IR (ATR): ṽ = 2929.7 (w), 2855.1 (w), 1625.1 (m), 1587.8 (m), 1431.3 (m), 1252.4 (m), 1006.4 (m), 834.9 (m), 752.9 (m) cm^{-1}.

4.2 Products of Cu(I)-catalyzed reduction of amides with H_2

4.2.1 Benzyl alcohol from phenyl(thiomorpholino)methanone (2g)

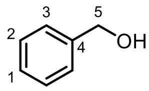

2g

C_7H_8O

Mw = 108.14 g mol^{-1}

Prepared according to **GP3** from phenyl(thiomorpholino)methanone (**1g,** 83 mg, 0.40 mmol, 1.0 equiv), [CuGua] **3** (19 mg, 40 µmol, 10 mol%), NaO*t*Bu (50 mg, 0.52 mmol, 1.3 equiv) and 15-crown-5 (0.12 mL, 0.60 mmol, 1.5 equiv) in 1,4-dioxane (4.0 mL). The reaction mixture was stirred for 24 h at 70 °C. Purification by flash column chromatography on silica gel (cyclohexane/ethyl acetate = 90:10) yielded **2g** as colourless oil (75% conversion of **1g**, 20 mg, 0.18 mmol, 45%).

R*f* = 0.30 (cyclohexane/ethyl acetate = 80:20).

^{1}H NMR (600 MHz, $CDCl_3$): δ = 1.66 (s, 1H, O–*H*), 4.70 (s, 2H, H-5), 7.28–7.33 (m, 1H, H-1), 7.34–7.40 (m, 4H, H-2, H-3) ppm.

^{13}C NMR (151 MHz, $CDCl_3$): δ = 65.6 (C-5), 127.1 (C-3), 127.8 (C-2), 128.7 (C-1), 141.0 (C-4) ppm.

HRMS (APCI) for $C_7H_7^+$ [$(M–OH)^+$] calculated: 91.0542, found 91.0534.

The data is in accordance with literature.[3]

4.2.2 Benzyl alcohol from 2-oxa-6-azaspiro[3.3]hept-6-ylphenylmethanone (2h)

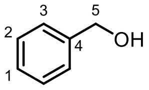

2h

C_7H_8O

Mw = 108.14 g mol^{-1}

Prepared according to **GP3** from 2-oxa-6-azaspiro[3.3]hept-6-ylphenylmethanone (**1h,** 81 mg, 0.40 mmol, 1.0 equiv), [CuGua] **3** (19 mg, 40 µmol, 10 mol%), NaO*t*Bu (50 mg, 0.52 mmol, 1.3 equiv) and 15-crown-5 (0.12 mL, 0.60 mmol, 1.5 equiv) in 1,4-dioxane (4.0 mL). The reaction mixture was stirred for 24 h at 70 °C. Purification by flash column chromatography on silica gel (cyclohexane/ethyl acetate = 90:10) yielded **2h** as colourless oil (88% conversion of **1h**, 16 mg, 0.15 mmol, 38%).

The data is in accordance with the data of section **4.2.1**.

4.2.3 Benzyl alcohol from phenyl(4-phenylpiperazin-1-yl)methanone (2i)

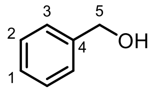

2i

C_7H_8O

Mw = 108.14 g mol^{-1}

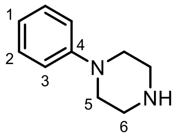

8i

$C_{10}H_{14}N_2$

Mw = 162.24 g mol^{-1}

Prepared according to **GP3** from phenyl(4-phenylpiperazin-1-yl)methanone (**1i**, 0.11 g, 0.40 mmol, 1.0 equiv), [CuGua] **3** (19 mg, 40 µmol, 10 mol%), NaO*t*Bu (50 mg, 0.52 mmol, 1.3 equiv) and 15-crown-5 (0.12 mL, 0.60 mmol, 1.5 equiv) in 1,4-dioxane (4.0 mL). The reaction mixture was stirred for 24 h at 70 °C. The reaction mixture was diluted with EtOAc (10 mL) and washed with aq. HCl (2M) solution. The organic phase was dried over $MgSO_4$, filtered and all volatiles were removed under reduced pressure.

Purification by flash column chromatography on silica gel (cyclohexane/ethyl acetate = 90:10) yielded **2i** as colourless oil (26 mg, 0.24 mmol, 60%). The aqueous phase was basified with aq. NaOH (2M) solution until pH = 10, extracted with EtOAc (2 x 10 mL) and the combined organic layers were dried over $MgSO_4$, filtered and all volatiles were removed under reduced pressure, yielding **8i** as a pale yellow solid (45 mg, 0.28 mmol, 70%).

Benzyl alcohol (2i):
R*f* = 0.30 (cyclohexane/ethyl acetate = 8:2).
^{1}H NMR (600 MHz, $CDCl_3$): δ = 1.63 (s, 1H, O–*H*), 4.70 (s, 2H, H-5), 7.27–7.34 (m, 1H, H-1), 7.34–7.41 (m, 4H, H-2, H-3) ppm.
^{13}C NMR (151 MHz, $CDCl_3$): δ = 65.6 (C-5), 127.1 (C-3), 127.8 (C-2), 128.7 (C-1), 141.0 (C-4) ppm.
The data is in accordance with the data of section **4.2.1**.

1-Phenylpiperazine (8i):
^{1}H NMR (600 MHz, $CDCl_3$): δ = 3.04–3.09 (m, 4H, H-6), 3.15–3.20 (m, 4H, H-5), 6.83–6.91 (m, 1H, H-1), 6.90–6.99 (m, 2H, H-3), 7.23–7.31 (m, 3H, H-2) ppm.
The integration for H-2 at 7.23–7.31 ppm is slightly higher due to overlapping resonances with $CDCl_3$. Traces of unidentified sideproducts at 1.33, 2.16 and 2.22 ppm could be detected in ^{1}H NMR. The N–*H* peak could not be detected ^{1}H NMR.
^{13}C NMR (151 MHz, $CDCl_3$): δ = 46.1 (C-6), 50.4 (C-5), 116.4 (C-3), 120.0 (C-1), 129.3 (C-2), 151.9 (C-4) ppm.
HRMS (APCI) for $C_{10}H_{15}N_2^+$ [$(M+H)^+$] calculated: 163.1230, found 163.1229.
The data is in accordance with literature.[46]

4.2.4 Benzyl alcohol from morpholino(phenyl)methanone (2j)

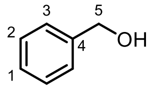

2j
C_7H_8O
Mw = 108.14 g mol^{-1}

Prepared according to **GP3** from morpholino(phenyl)methanone (**1j,** 77 mg, 0.40 mmol, 1.0 equiv), [CuGua] **3** (19 mg, 40 µmol, 10 mol%), NaO*t*Bu (50 mg, 0.52 mmol, 1.3 equiv) and 15-crown-5 (0.12 mL, 0.60 mmol, 1.5 equiv) in 1,4-dioxane (4.0 mL). The reaction mixture was stirred for 24 h at 70 °C. Purification by flash column chromatography on silica gel (cyclohexane/ethyl acetate = 90:10) yielded **2j** as colourless oil (30 mg, 0.28 mmol, 70%).

R*f* = 0.30 (cyclohexane/ethyl acetate = 80:20).
^{1}H NMR (600 MHz, $CDCl_3$): δ = 4.67 (s, 2H, H-5), 7.26–7.32 (m, 1H, H-1), 7.35–7.38 (m, 4H, H-2, H-3) ppm.
^{13}C NMR (151 MHz, $CDCl_3$): δ = 65.4 (C-5), 127.1 (C-3), 127.7 (C-2), 128.6 (C-1), 141.0 (C-4) ppm.

HRMS (APCI) for $C_7H_7^+$ [(M–OH)$^+$] calculated: 91.0542, found 91.0541.
The data is in accordance with literature.[3]

4.2.5 *p*-Tolylmethanol (2m)

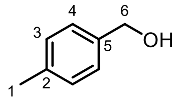

2m
$C_8H_{10}O$
Mw = 122.17 g mol^{-1}

Prepared according to **GP3** from morpholino(*p*-tolyl)methanone (**1m,** 82 mg, 0.40 mmol, 1.0 equiv), [CuGua] **3** (19 mg, 40 µmol, 10 mol%), NaO*t*Bu (50 mg, 0.52 mmol, 1.3 equiv) and 15-crown-5 (0.12 mL, 0.60 mmol, 1.5 equiv) in 1,4-dioxane (4.0 mL). The reaction mixture was stirred for 24 h at 70 °C. Purification by flash column chromatography on silica gel (*n*-pentane/ethyl acetate = 80:20) yielded **2m** as an off white solid (83% conversion of **1m**, 30 mg, 0.25 mmol, 62%).

R*f* = 0.20 (*n*-pentane/ethyl acetate = 80:20).
^{1}H NMR (600 MHz, $CDCl_3$): δ = 1.80 (br s, 1H, O–*H*), 2.36 (s, 3H, H-1), 4.64 (s, 2H, H-6), 7.18 (d, $^3J_{3,4}$ = 7.8 Hz, 2H, H-3), 7.26 (d, $^3J_{4,3}$ = 7.9 Hz, 2H, H-4) ppm.
^{13}C NMR (151 MHz, $CDCl_3$): δ = 21.3 (C-1), 65.4 (C-6), 127.2 (C-4), 129.4 (C-3), 137.5 (C-2), 138.0 (C-5) ppm.
HRMS (APCI) for $C_8H_9^+$ [(M–OH)$^+$] calculated: 105.0699, found 105.0699.
The data is in accordance with literature.[3]

4.2.6 Cyclohexylmethanol (2n)

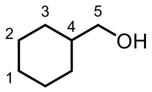

2n
$C_7H_{14}O$
Mw = 114.19 g mol^{-1}

Prepared according to **GP3** from cyclohexyl(morpholino)methanone (**1n,** 79 mg, 0.40 mmol, 1.0 equiv), [CuGua] **3** (19 mg, 40 µmol, 10 mol%), NaO*t*Bu (50 mg, 0.52 mmol, 1.3 equiv) and 15-crown-5 (0.12 mL, 0.60 mmol, 1.5 equiv) in 1,4-dioxane (4.0 mL). The reaction mixture was stirred for 24 h at 70 °C. Purification by flash column chromatography on silica gel (cyclohexane/ethyl acetate = 90:10) yielded **2n** as a pale yellow oil (85% conversion of **1n**, 26 mg, 0.23 mmol, 58%).

R*f* = 0.60 (cyclohexane/ethyl acetate = 80:20).
^{1}H NMR (600 MHz, $CDCl_3$): δ = 0.84–1.05 (m, 2H, H-3), 1.10–1.39 (m, 4H, H-1, H-2, O–*H*)*, 1.42–1.53 (m, 1H, H-4), 1.63–1.87 (m, 5H, H-1, H-2', H-3')*, 3.44 (d, $^3J_{5,4}$ = 6.4 Hz, 2H, H-5) ppm.
^{13}C NMR (151 MHz, $CDCl_3$): δ = 26.0 (C-2)*, 26.7 (C-1)*, 29.7 (C-3)*, 40.6 (C-4), 68.9 (C-5) ppm.
HRMS (APCI) for $C_7H_{13}^+$ [(M–OH)$^+$] calculated: 97.1012, found 97.1008.
The data is in accordance with literature.[3]

4.2.7 *m*-Tolylmethanol (2o)

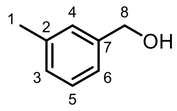

2o

$C_8H_{10}O$

Mw = 122.17 g mol^{-1}

Prepared according to **GP3** from morpholino(*m*-tolyl)methanone (**1o,** 81 mg, 0.40 mmol, 1.0 equiv), [CuGua] **3** (19 mg, 40 µmol, 10 mol%), NaO*t*Bu (50 mg, 0.52 mmol, 1.3 equiv) and 15-crown-5 (0.12 mL, 0.60 mmol, 1.5 equiv) in 1,4-dioxane (4.0 mL). The reaction mixture was stirred for 24 h at 70 °C. Purification by flash column chromatography on silica gel (*n*-pentane/ethyl acetate = 80:20) yielded **2o** as a colourless oil (77% conversion of **1o**, 26 mg, 0.21 mmol, 53%).

R*f* = 0.25 (*n*-pentane /ethyl acetate = 80:20).

^{1}H NMR (600 MHz, $CDCl_3$): δ = 1.85 (br s, 1H, O–*H*), 2.35 (s, 3H, H-1), 4.63 (s, 2H, H-8), 7.10 (d, $^3J_{3,5}$ = 7.5 Hz, 1H, H-3), 7.14 (d, $^3J_{6,5}$ = 7.6 Hz, 1H, H-6), 7.17 (s, 1H, H-4), 7.24 (t, $^3J_{5,3/6}$ = 7.5 Hz, 1H, H-5) ppm.

^{13}C NMR (151 MHz, $CDCl_3$): δ = 21.5 (C-1), 65.5 (C-8), 124.2 (C-6), 127.9 (C-4), 128.5 (C-3), 128.6 (C-5), 138.4 (C-2), 140.9 (C-7) ppm.

HRMS (APCI) for $C_8H_9^+$ [(M–OH)$^+$] calculated: 105.0699, found 105.0698.

The data is in accordance with literature.[48]

4.2.8 *o*-Tolylmethanol (2p)

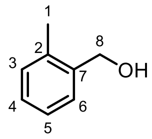

2p

$C_8H_{10}O$

Mw = 122.17 g mol^{-1}

Prepared according to **GP3** from morpholino(*o*-tolyl)methanone (**1p,** 81 mg, 0.40 mmol, 1.0 equiv), [CuGua] **3** (19 mg, 40 µmol, 10 mol%), NaO*t*Bu (50 mg, 0.52 mmol, 1.3 equiv) and 15-crown-5 (0.12 mL, 0.60 mmol, 1.5 equiv) in 1,4-dioxane (4.0 mL). The reaction mixture was stirred for 24 h at 70 °C. Purification by flash column chromatography on silica gel (cyclohexane/ethyl acetate = 80:20) yielded **2p** as a pale yellow solid (58% conversion of **1p**, 21 mg, 0.17 mmol, 43%).

R*f* = 0.30 (cyclohexane/ethyl acetate = 80:20).

^{1}H NMR (600 MHz, $CDCl_3$): δ = 1.59 (s, 1H, O–*H*), 2.37 (s, 3H, H-1), 4.71 (s, 2H, H-8), 7.13–7.25 (m, 3H, H-3, H-4*, H-5*), 7.33–7.40 (m, 1H, H-6)* ppm.

^{13}C NMR (151 MHz, $CDCl_3$): δ = 18.8 (C-1), 63.7 (C-8), 126.2 (C-4)*, 127.7 (C-6)*, 128.0 (C-5)*, 130.5 (C-3), 136.3 (C-2), 138.8 (C-7) ppm.

HRMS (APCI) for $C_8H_9^+$ [(M–OH)$^+$] calculated: 105.0699, found 105.0698.

The data is in accordance with literature.[48]

4.2.9 4-(Trifluoromethyl)phenyl)methanol (2q)

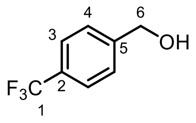

2q

$C_8H_7F_3O$

Mw = 176.14 g mol^{-1}

Prepared according to **GP3** from morpholino(4-(trifluoromethyl)phenyl) methanone (**1q,** 0.10 g, 0.40 mmol, 1.0 equiv), [CuGua] **3** (19 mg, 40 μmol, 10 mol%), NaO*t*Bu (50 mg, 0.52 mmol, 1.3 equiv) and 15-crown-5 (0.12 mL, 0.60 mmol, 1.5 equiv) in 1,4-dioxane (4.0 mL). The reaction mixture was stirred for 24 h at 70 °C. Purification by flash column chromatography on silica gel (cyclohexane/ethyl acetate = 90:10) yielded **2q** as a white solid (32 mg, 0.18 mmol, 45%).

R$_f$ = 0.20 (cyclohexane/ethyl acetate = 80:20).

^{1}H NMR (600 MHz, $CDCl_3$): δ = 1.78 (s, 1H, O–*H*), 4.78 (s, 2H, H-6), 7.49 (d, $^3J_{4,3}$ = 8.1 Hz, 2H, H-4), 7.62 (d, $^3J_{3,4}$ = 8.0 Hz, 2H, H-3) ppm.

^{13}C NMR (151 MHz, $CDCl_3$): δ = 64.7 (C-6), 124.3 (q, $^1J_{1,F}$ = 271.9 Hz, C-1), 125.6 (q, $^3J_{3,F}$ = 3.8 Hz, C-3), 127.1 (C-4), 129.9 (q, $^2J_{2,F}$ = 32.4 Hz, C-2), 144.9 (C-5) ppm.

^{19}F NMR (564 MHz, $CDCl_3$): δ = – 62.5 ppm.

HRMS (APCI) for $C_8H_6F_3^+$ [(M–OH)$^+$] calculated: 159.0416, found 159.0414.

The data is in accordance with literature.[3]

4.2.10 (4-Methoxyphenyl)methanol (2r)

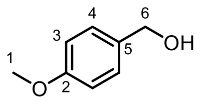

2r

$C_8H_{10}O_2$

Mw = 138.17 g mol^{-1}

Prepared according to **GP3** from (4-methoxyphenyl)(morpholino) methanone (**1r,** 89 mg, 0.40 mmol, 1.0 equiv), [CuGua] **3** (19 mg, 40 μmol, 10 mol%), NaO*t*Bu (50 mg, 0.52 mmol, 1.3 equiv) and 15-crown-5 (0.12 mL, 0.60 mmol, 1.5 equiv) in 1,4-dioxane (4.0 mL). The reaction mixture was stirred for 24 h at 70 °C. Purification by flash column chromatography on silica gel (cyclohexane/ethyl acetate = 9:1) yielded **2r** as a white solid (74% conversion of **1r**, 32 mg, 0.23 mmol, 58%).

R$_f$ = 0.30 (cyclohexane/ethyl acetate = 80:20).

^{1}H NMR (600 MHz, $CDCl_3$): δ = 1.91 (s, 1H, O–*H*), 3.81 (s, 3H, H-1), 4.60 (s, 2H, H-6), 6.89 (d, $^3J_{3,4}$ = 8.7 Hz, 2H, H-3), 7.29 (d, $^3J_{4,3}$ = 8.4 Hz, 2H, H-4) ppm.

^{13}C NMR (151 MHz, $CDCl_3$): δ = 55.4 (C-1), 65.1 (C-6), 114.0 (C-3), 128.7 (C-4), 133.2 (C-5), 159.3 (C-2) ppm.

HRMS (APCI) for $C_8H_9O^+$ [(M–OH)$^+$] calculated: 121.0648, found 121.0643.

The data is in accordance with literature.[3]

4.2.11 (4-Chlorophenyl)methanol (2s)

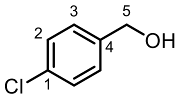

2s
C_7H_7ClO
Mw = 142.58 g mol^{-1}

Prepared according to **GP3** from (4-chlorophenyl)(morpholino) methanone (**1s,** 90 mg, 0.40 mmol, 1.0 equiv), [CuGua] **3** (19 mg, 40 µmol, 10 mol%), NaO*t*Bu (50 mg, 0.52 mmol, 1.3 equiv) and 15-crown-5 (0.12 mL, 0.60 mmol, 1.5 equiv) in 1,4-dioxane (4.0 mL). The reaction mixture was stirred for 4 h at 70 °C. Purification by flash column chromatography on silica gel (cyclohexane/ethyl acetate = 90:10) yielded **2s** as a white solid (39 mg, 0.27 mmol, 68%).

R*f* = 0.45 (cyclohexane/ethyl acetate = 80:20).

^{1}H NMR (600 MHz, $CDCl_3$): δ = 1.74 (s, 1H, O–*H*), 4.67 (s, 2H, H-5), 7.30 (d, $^3J_{3,2}$ = 8.5 Hz, 2H, H-3), 7.33 (d, $^3J_{2,3}$ = 8.5 Hz, 2H, H-2) ppm.

^{13}C NMR (151 MHz, $CDCl_3$): δ = 64.7 (C-5), 128.4 (C-3), 128.8 (C-2), 133.5 (C-1), 139.4 (C-4) ppm.

HRMS (APCI) for $C_7H_6{}^{35}Cl^+$ [M–OH)$^+$] calculated: 125.0153, found 125.0150.

The data is in accordance with literature.[48]

4.2.12 (4-(4,4,5,5-Tetramethyl-1,3,2-dioxaborolan-2-yl)phenyl)methanol (2t)

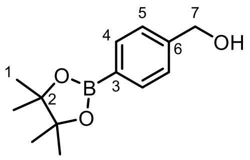

2t
$C_{13}H_{19}BO_3$
Mw = 234.10 g mol^{-1}

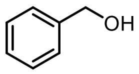

2t'
C_7H_8O
Mw = 108.14 g mol^{-1}

Prepared according to **GP3** from morpholino (4(4,4,5,5-tetramethyl-1,3,2-dioxaborolan-2-yl)phenyl)methanone (**1t,** 0.13 g, 0.40 mmol, 1.0 equiv), [CuGua] **3** (19 mg, 40 µmol, 10 mol%), NaO*t*Bu (50 mg, 0.52 mmol, 1.3 equiv) and 15-crown-5 (0.12 mL, 0.60 mmol, 1.5 equiv) in 1,4-dioxane (4.0 mL). The reaction mixture was stirred for 24 h at 70 °C. Purification by flash column chromatography on silica gel (*n*-pentane/ethyl acetate = 70:30) yielded **2t**/**2t'** as a viscous oil (78% conversion of **1t**, **2t**/**2t'** = 74:36, isolated as a mixture of **2t**/**2t'** = 60:40, 37 mg, 0.16 mmol, 40% of **2t**).

R*f* = 0.15 (*n*-pentane/ethyl acetate = 80:20).

^{1}H NMR (600 MHz, $CDCl_3$): δ = 1.35 (s, 12H, H-1), 1.68 (s, 1.6H, O–*H*), 4.72 (s, 2H, H-7), 7.37 (d, $^3J_{5,4}$ = 7.8 Hz, 2H, H-5), 7.81 (d, $^3J_{4,5}$ = 5.5 Hz, 2H, H-4) ppm.

The integration for O–*H* at 1.68 ppm is slightly higher due to overlapping resonances with benzyl alcohol (**2t'**).

^{13}C NMR (151 MHz, $CDCl_3$): δ = 25.0 (C-1), 65.5 (C-7), 84.0 (C-2), 126.2 (C-5), 127.1 (C-3), 135.2 (C-4), 144.1 (C-6) ppm.

^{11}B NMR (193 MHz, $CDCl_3$): δ = 30.8 ppm.

HRMS (APCI) for $C_{13}H_{18}O_2B^+$ [(M–OH)$^+$] calculated: 217.1395, found 217.1396.

The data is in accordance with literature.[49]

4.2.13 Furan-2-ylmethanol (2u)

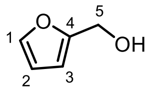

2u
$C_5H_6O_2$
Mw = 98.10 g mol^{-1}

Prepared according to **GP3** from furan-2-yl(morpholino)methanone (**1u,** 72 mg, 0.40 mmol, 1.0 equiv), [CuGua] **3** (19 mg, 40 µmol, 10 mol%), NaO*t*Bu (50 mg, 0.52 mmol, 1.3 equiv) and 15-crown-5 (0.12 mL, 0.60 mmol, 1.5 equiv) in 1,4-dioxane (4.0 mL). The reaction mixture was stirred for 24 h at 70 °C. Purification by flash column chromatography on silica gel (*n*-pentane/diethylether = 90:10) yielded **2u** as a colourless oil (21 mg, 0.21 mmol, 54%).

R_f = 0.30 (*n*-pentane/diethylether = 70:30).
^{1}H NMR (600 MHz, $CDCl_3$): δ = 1.74 (t, $^3J_{O–H,5}$ = 5.9 Hz, 1H, O–*H*), 4.61 (d, $^3J_{5,O–H}$ = 5.2 Hz, 2H, H-5), 6.30 (d, $^3J_{3,2}$ = 3.2 Hz, 1H, H-3), 6.34 (dd, $^3J_{2,3}$= 3.2 Hz, $^3J_{2,1}$ = 1.9 Hz, 1H, H-2), 7.40 (d, $^3J_{1,2}$ = 1.7 Hz, 1H, H-1) ppm.
Traces of 1,4-dioxane at 3.70 ppm could be detected in ^{1}H NMR.
^{13}C NMR (151 MHz, $CDCl_3$): δ = 57.7 (C-5), 107.9 (C-3), 110.5 (C-2), 142.7 (C-1), 154.1 (C-4) ppm.
HRMS (APCI) for $C_5H_5O^+$ [(M–OH)$^+$] calculated: 81.0335, found 81.0338.
The data is in accordance with literature.[3]

4.2.14 Thiophen-2-ylmethanol (2v)

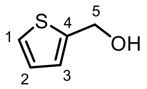

2v
C_5H_6OS
Mw = 114.16 g mol^{-1}

Prepared according to **GP3** from morpholino(thiophen-2-yl)methanone (**1v,** 79 mg, 0.40 mmol, 1.0 equiv), [CuGua] **3** (19 mg, 40 µmol, 10 mol%), NaO*t*Bu (50 mg, 0.52 mmol, 1.3 equiv) and 15-crown-5 (0.12 mL, 0.60 mmol, 1.5 equiv) in 1,4-dioxane (4.0 mL). The reaction mixture was stirred for 24 h at 70 °C. Purification by flash column chromatography on silica gel (cyclohexane/ethyl acetate = 90:10) yielded **2v** as a yellow oil (28 mg, 0.25 mmol, 62%).

R_f = 0.40 (cyclohexane/ethyl acetate = 90:10).
^{1}H NMR (600 MHz, $CDCl_3$): δ = 1.94 (s, 1H, O–*H*), 4.83 (s, 2H, H-5), 6.97–6.99 (m, 1H, H-2), 7.02 (d, $^3J_{3,2}$ = 3.4 Hz, 1H, H-3), 7.28 (dd, $^3J_{1,2}$ = 5.1 Hz, $^4J_{1,3}$ = 1.2 Hz, 1H, H-1) ppm.
Traces of 1,4-dioxane at 3.70 ppm could be detected in ^{1}H NMR.
^{13}C NMR (151 MHz, $CDCl_3$): δ = 60.1 (C-5), 125.6 (C-3), 125.7 (C-1), 127.0 (C-2), 144.1 (C-4) ppm.
HRMS (APCI) for $C_5H_5S^+$ [(M–OH)$^+$] calculated: 97.0107, found 97.0109.
The data is in accordance with literature.[3]

4.2.15 (5-((Benzyloxy)methyl)furan-2-yl)methanol (2w)

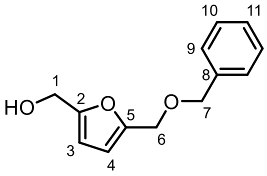

2w
$C_{13}H_{14}O_3$
Mw = 218.25 g mol^{-1}

Prepared according to **GP3** from (5-((benzyloxy)methyl)furan-2-yl)(morpholino)methanone (**1w,** 115 mg, 0.400 mmol, 1.00 equiv), [CuGua] **3** (19 mg, 40 µmol, 10 mol%), NaO*t*Bu (50 mg, 0.52 mmol, 1.3 equiv) and 15-crown-5 (0.12 mL, 0.60 mmol, 1.5 equiv) in 1,4-dioxane (4.0 mL). The reaction mixture was stirred for 24 h at 70 °C. Purification by flash column chromatography on silica gel (*n*-pentane/ethyl acetate = 80:20) yielded **2w** as yellow oil (60% conversion of **1w**, 36 mg, 0.16 mmol, 41%).

R*f* = 0.15 (cyclohexane/ethyl acetate = 80:20).
^{1}H NMR (600 MHz, $CDCl_3$): δ = 1.76 (br s, 1H, O–*H*), 4.47 (s, 2H, H-6), 4.57 (s, 2H, H-7), 4.60 (s, 2H, H-1), 6.25 (d, $^3J_{3,4}$ = 3.2 Hz, 1H, H-3), 6.28 (d, $^3J_{4,3}$ = 3.2 Hz, 1H, H-4), 7.27–7.33 (m, 1H, H-11), 7.32–7.39 (m, 4H, H-9, H-10) ppm.
^{13}C NMR (151 MHz, $CDCl_3$): δ = 57.8 (C-1), 64.1 (C-6), 72.2 (C-7), 108.6 (C-3), 110.4 (C-4), 127.9 (C-11), 128.1 (C-10)*, 128.6 (C-9)*, 137.9 (C-8), 151.9 (C-5), 154.5 (C-2) ppm.
HRMS (APCI) for $C_{13}H_{13}O_2^+$ [(M-OH)$^+$] calculated: 201.0911, found 201.0911.
The data is in accordance with literature.[50]

4.2.16 4-Phenylbutan-1-ol (2x)

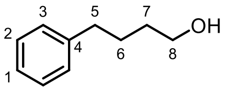

2x
$C_{10}H_{14}O$
Mw = 150.22 g mol^{-1}

Prepared according to **GP3** from 1-morpholino-4-phenylbutan-1-one (**1x,** 47 mg, 0.20 mmol, 1.0 equiv), [CuGua] **3** (19 mg, 40 µmol, 20 mol%), NaO*t*Bu (25 mg, 0.26 mmol, 1.3 equiv) and 15-crown-5 (0.06 mL, 0.30 mmol, 1.5 equiv) in 1,4-dioxane (4.0 mL). The reaction mixture was stirred for 24 h at 70 °C. Purification by flash column chromatography on silica gel (cyclohexane/ethyl acetate = 90:10) yielded **2x** as a colourless oil (91% conversion of **1x**, 23 mg, 0.15 mmol, 77%).

R*f* = 0.25 (cyclohexane/ethyl acetate = 80:20).
^{1}H NMR (600 MHz, $CDCl_3$): δ = 1.23 (br s, 1H, *O–H*), 1.58–1.69 (m, 2H, H-7), 1.66–1.81 (m, 2H, H-6), 2.65 (t, $^3J_{5,6}$ = 7.6 Hz, 2H, H-5), 3.67 (t, $^3J_{8,7}$ = 6.5 Hz, 2H, H-8), 7.15–7.22 (m, 3H, H-1, H-3), 7.28 (dd, $^3J_{2,1/3}$ = 8.2 Hz, $^3J_{2,3/1}$ = 6.9 Hz, 2H, H-2) ppm.
^{13}C NMR (151 MHz, $CDCl_3$): δ = 27.7 (C-6), 32.5 (C-7), 35.8 (C-5), 63.0 (C-8), 125.9 (C-1), 128.4 (C-2), 128.5 (C-3), 142.5 (C-4) ppm.
HRMS (APCI) for $C_{10}H_{13}^+$ [(M–OH)$^+$] calculated: 133.1012, found 133.1005.
The data is in accordance with literature.[23]

4.2.17 Undec-10-en-1-ol (2y)

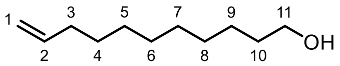

2y

$C_{11}H_{22}O$

Mw = 170.30 g mol^{-1}

Prepared according to **GP3** from 1-morpholinoundec-10-en-1-one (**1y,** 0.10 g, 0.40 mmol, 1.0 equiv), [CuGua] **3** (19 mg, 40 µmol, 10 mol%), NaO*t*Bu (50 mg, 0.52 mmol, 1.3 equiv) and 15-crown-5 (0.12 mL, 0.60 mmol, 1.5 equiv) in 1,4-dioxane (4.0 mL). The reaction mixture was stirred for 24 h at 70 °C. Purification by flash column chromatography on silica gel (cyclohexane/ethyl acetate = 90:10) yielded **2y** as a colourless oil (60% conversion of **1y**, 21 mg, 0.12 mmol, 31%).

R_f = 0.25 (cyclohexane/ethyl acetate = 90:10).
^{1}H NMR (600 MHz, $CDCl_3$): δ = 1.24–1.43 (m, 13H, H-4, H-5, H-6, H-7, H-8, H-9, O–*H*), 1.48–1.64 (m, 2H, H-10), 1.93–2.13 (m, 2H, H-3), 3.64 (t, $^3J_{11,10}$ = 6.7 Hz, 2H, H-11), 4.88–5.03 (m, 2H, H-1), 5.81 (ddt, $^3J_{2,1a}$ = 16.9 Hz, $^3J_{2,1b}$ = 10.2 Hz, $^3J_{2,3}$ = 6.7 Hz, 1H, H-2) ppm.
^{13}C NMR (151 MHz, $CDCl_3$): δ = 25.9 (C-4)*, 29.1 (C-5)*, 29.3 (C-6)*, 29.6 (C-7)*, 29.6 (C-8)*, 29.7 (C-9)*, 33.0 (C-10), 34.0 (C-3), 63.3 (C-11), 114.3 (C-1), 139.4 (C-2) ppm.
HRMS (APCI) for $C_{11}H_{23}O^+$ [$(M+H)^+$] calculated: 171.1744, found 171.1742.
The data is in accordance with literature.[47]

4.2.18 Decan-1-ol (2z)

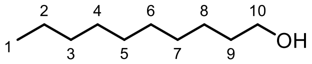

2z

$C_{10}H_{22}O$

Mw = 158.28 g mol^{-1}

Prepared according to **GP3** from 1-morpholinodecan-1-one (**1z,** 97 mg, 0.40 mmol, 1.0 equiv), [CuGua] **3** (19 mg, 40 µmol, 10 mol%), NaO*t*Bu (50 mg, 0.52 mmol, 1.3 equiv) and 15-crown-5 (0.12 mL, 0.60 mmol, 1.5 equiv) in 1,4-dioxane (4.0 mL). The reaction mixture was stirred for 24 h at 70 °C. Purification by flash column chromatography on silica gel (cyclohexane/ethyl acetate = 90:10) yielded **2z** as a yellow oil (88% conversion of **1z**, 37 mg, 0.23 mmol, 58%).

R_f = 0.30 (cyclohexane/ethyl acetate = 80:20).
^{1}H NMR (600 MHz, $CDCl_3$): δ = 0.87 (t, $^3J_{1,2}$ = 7.0 Hz, 3H, H-1), 1.20–1.41 (m, 15H, H-2, H-3, H-4, H-5, H-6, H-7, H-8, O–*H*), 1.53–1.63 (m, 2H, H-9), 3.63 (t, $^3J_{10,9}$ = 6.7 Hz, 2H, H-10) ppm.
^{13}C NMR (151 MHz, $CDCl_3$): δ = 14.2 (C-1), 22.8 (C-2)*, 25.9 (C-3)*, 29.5 (C-4)*, 29.6 (C-5)*, 29.7 (C-6)*, 29.8 (C-7)*, 32.0 (C-8)*, 32.9 (C-9), 63.2 (C-10) ppm.
HRMS (APCI) for $C_{10}H_{21}^+$ [$(M–OH)^+$] calculated: 141.1638, found 141.1630.
The data is in accordance with literature.[21]

4.2.19 (4-Styrylphenyl)methanol (2aa)

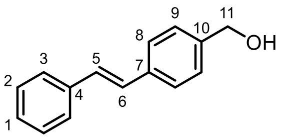

***E*-2aa**
$C_{15}H_{14}O$
Mw = 210.28 g mol^{-1}

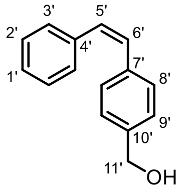

***Z*-2aa**
$C_{15}H_{14}O$
Mw = 210.28 g mol^{-1}

Prepared according to **GP3** from (5 morpholino(4-(phenylethynyl)phenyl)methanone (**1aa,** 117 mg, 0.400 mmol, 1.00 equiv), [CuGua] **3** (19 mg, 40 µmol, 10 mol%), NaO*t*Bu (50 mg, 0.52 mmol, 1.3 equiv) and 15-crown-5 (0.12 mL, 0.60 mmol, 1.5 equiv) in 1,4-dioxane (4.0 mL). The reaction mixture was stirred for 24 h at 70 °C. Purification by flash column chromatography on silica gel (*n*-pentane/ethyl acetate = 90:10) yielded a mixture of ***E*-2aa/*Z*-2aa** = 31:69 as a pale yellow solid (10% of the corresponding alkane detected, 59 mg, 0.28 mmol, 70%).

$\mathbf{R}_f$ = 0.20 (*n*-pentane/ethyl acetate = 90:10).

***Z* isomer**

^{1}H NMR (600 MHz, $CDCl_3$): δ = 1.58 (t, $^3J_{O–H,11'}$ = 5.9 Hz, 1H, O–*H*), 4.66 (d, $^3J_{11', O–H}$ = 5.8 Hz, 2H, H-11'), 6.54–6.65 (m, 2H, H-5', H-6'), 7.16–7.25 (m, 14H, H-1', H-2', H-3', H-8', H-9', H-1*) ppm.

The integration at 7.16–7.25 ppm is slightly higher due to overlapping resonances with $CDCl_3$.

***E* isomer**

^{1}H NMR (600 MHz, $CDCl_3$): δ = 1.61 (t, $^3J_{O–H,11}$ = 6.0 Hz, 1H, O–*H*), 4.71 (d, $^3J_{11, O–H}$ = 5.9 Hz, 0.89H, H-11), 7.12 (s, 0.87H, H-5, H-6), 7.16–7.25 (m, 14H, H-1', H-2', H-3', H-8', H-9', H-1*), 7.32–7.41 (m, 1.85H, H-2, H-3)*, 7.48–7.56 (m, 1.71H, H-8, H-9)* ppm.

The integration for H-1 at 7.16–7.25 ppm higher due to overlapping resonances with the aromatic H atoms of *Z* isomer and with $CDCl_3$.

***E* and *Z* isomer**

^{13}C NMR (151 MHz, $CDCl_3$): δ = 65.3 (C-11, C-11'), 126.7 (C-8)**, 126.8 (C-9)**, 127.0 (C-1)****, 127.3 (C-2)****, 127.3 (C-3)****, 127.5 (C-1')****, 127.8 (C-4)****, 128.4 (C-7)****, 128.5 (C-8')****, 128.8 (C-9')****, 128.9 (C-2')****, 129.0 (C-3')****, 129.2 (C-5, C-6)****, 130.0 (C-5')*, 130.5 (C-6')*, 136.8 (C-4')***, 137.4 (C-7')***, 139.8 (C-10, C-10') ppm.

HRMS (APCI) for $C_{15}H_{13}^+$ [(M-OH)$^+$] calculated: 193.1012, found 193.1012.

The data is in accordance with literature.[51]

4.2.20 Benzyl alcohol from (4-methylpiperazin-1-yl)(phenyl)methanone (2ab)

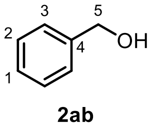

2ab
C_7H_8O
Mw = 108.14 g mol^{-1}

Prepared according to **GP3** from (4-methylpiperazin-1-yl)(phenyl)methanone (**1ab,** 82 mg, 0.40 mmol, 1.0 equiv), [CuGua] **3** (19 mg, 40 µmol, 10 mol%), NaO*t*Bu (50 mg, 0.52 mmol, 1.3 equiv) and 15-crown-5 (0.12 mL, 0.60 mmol, 1.5 equiv) in 1,4-dioxane (4.0 mL). The

reaction mixture was stirred for 24 h at 70 °C. Purification by flash column chromatography on silica gel (cyclohexane/ethyl acetate = 90:10) yielded **2ab** as colourless oil (70% conversion of **1ab**, 18 mg, 0.17 mmol, 43%).

The data is in accordance with the data of section **4.2.1**.

4.2.21 Benzyl alcohol from (4-ethylpiperazin-1-yl)(phenyl)methanone (2ac)

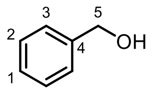

2ac
C_7H_8O
Mw = 108.14 g mol^{-1}

Prepared according to **GP3** from (4-ethylpiperazin-1-yl)(phenyl)methanone (**1ac,** 87 mg, 0.40 mmol, 1.0 equiv), [CuGua] **3** (19 mg, 40 µmol, 10 mol%), NaO*t*Bu (50 mg, 0.52 mmol, 1.3 equiv) and 15-crown-5 (0.12 mL, 0.60 mmol, 1.5 equiv) in 1,4-dioxane (4.0 mL). The reaction mixture was stirred for 24 h at 70 °C. Purification by flash column chromatography on silica gel (cyclohexane/ethyl acetate = 90:10) yielded **2ac** as colourless oil (67% conversion of **1ac**, 20 mg, 0.18 mmol, 45%).

The data is in accordance with the data of section **4.2.1**.

4.2.22 Benzyl alcohol from (4-isopropylpiperazin-1-yl)(phenyl)methanone (2ad)

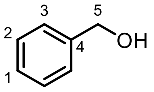

2ad
C_7H_8O
Mw = 108.14 g mol^{-1}

Prepared according to **GP3** from (4-isopropylpiperazin-1-yl)(phenyl)methanone (**1ad,** 93 mg, 0.40 mmol, 1.0 equiv), [CuGua] **3** (19 mg, 40 µmol, 10 mol%), NaO*t*Bu (50 mg, 0.52 mmol, 1.3 equiv) and 15-crown-5 (0.12 mL, 0.60 mmol, 1.5 equiv) in 1,4-dioxane (4.0 mL). The reaction mixture was stirred for 24 h at 70 °C. Purification by flash column chromatography on silica gel (cyclohexane/ethyl acetate = 90:10) yielded **2ad** as colourless oil (44% conversion of **1ad**, 13 mg, 0.12 mmol, 30%).

The data is in accordance with the data of section **4.2.1**.

4.2.23 Benzyl alcohol from phenyl(4-(pyridin-2-yl)piperazin-1-yl)methanone (2ae)

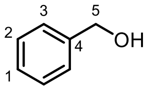

2ae
C_7H_8O
Mw = 108.14 g mol^{-1}

Prepared according to **GP3** from phenyl(4-(pyridin-2-yl)piperazin-1-yl)methanone (**1ae,** 0.11 g, 0.40 mmol, 1.0 equiv), [CuGua] **3** (19 mg, 40 µmol, 10 mol%), NaO*t*Bu (50 mg, 0.52 mmol, 1.3 equiv) and 15-crown-5 (0.12 mL, 0.60 mmol, 1.5 equiv) in 1,4-dioxane (4.0 mL). The reaction mixture was stirred for 24 h at 70 °C. Purification by flash column chromatography on silica gel (cyclohexane/ethyl acetate = 90:10) yielded **2ae** as colourless oil (45% conversion of **1ae**, 19 mg, 0.17 mmol, 43%).

The data is in accordance with the data of section **4.2.1**.

4.3 Additional substrates for the Cu(I) catalyzed reduction of amides with H_2

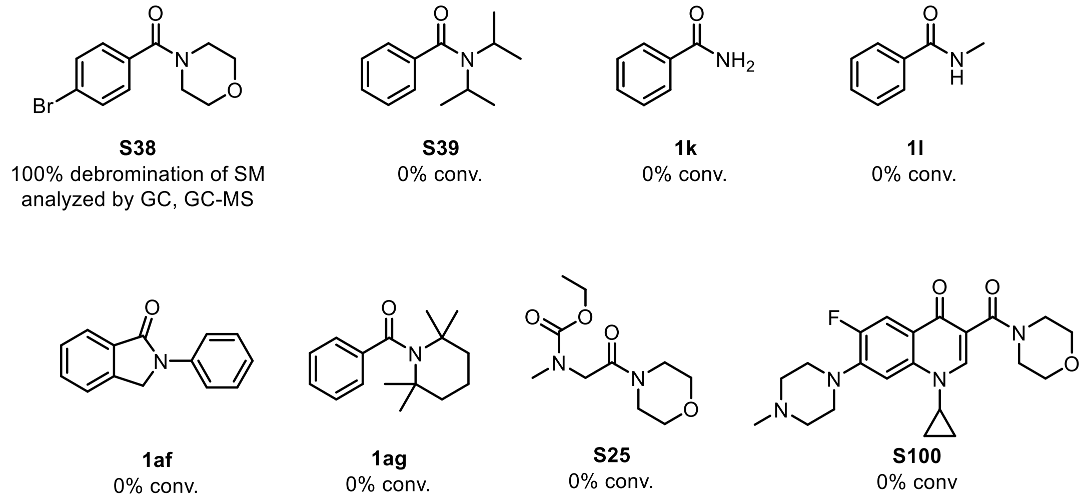

Figure S3: Unsuccessful substrates for the Cu(I) catalyzed reduction of amides with H_2.

4.4 Site-selective reduction of diamides

4.4.1 Reduction of 2-[4-(morpholine-4-carbonyl)phenyl]-1-(1-piperidyl)ethanone (11)

4.4.1.1 Cu(I)-catalyzed reduction of 2-[4-(morpholine-4-carbonyl)phenyl]-1-(1-piperidyl)ethanone (11) with H_2

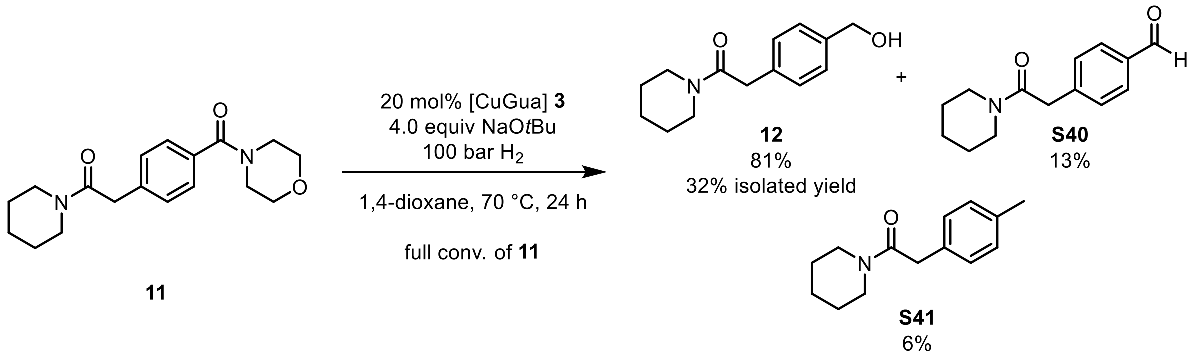

Scheme S15: Site-selective Cu(I)-catalyzed reduction of 2-[4-(morpholine-4-carbonyl)phenyl]-1-(1-piperidyl)ethanone (**11**) with H_2.

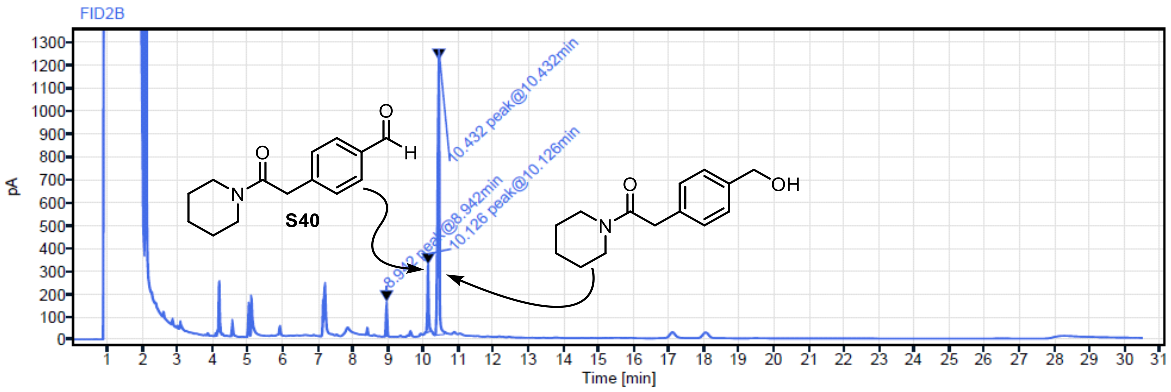

Signal: FID2B

Name	RT [min]	RF	Area	Peak Area Percent
peak@8.942min	8.94		290.946	6.14
peak@10.126min	10.13		609.844	12.86
peak@10.432min	10.43		3841.308	81.00

Figure S4: GC analysis of the crude reaction mixture of the Cu(I)-catalyzed reduction of 2-[4-(morpholine-4-carbonyl)phenyl]-1-(1-piperidyl)ethanone (**11**) with H_2 (GC method: 40_20_250_20).

4.4.1.1.1 2-[4-(Hydroxymethyl)phenyl]-1-(1-piperidyl)ethanone (12)

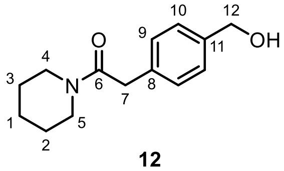

12
$C_{14}H_{19}NO_2$
Mw = 233.31 g mol-1

Prepared according to **GP4** from 2-[4-(morpholine-4-carbonyl)phenyl]-1-(1-piperidyl)ethanone (**11**, 63 mg, 0.20 mol, 1.0 equiv), [CuGua] **3** (19 mg, 40 µmol, 20 mol%), NaO*t*Bu (77 mg, 0.80 mmol, 4.0 equiv) in 1,4-dioxane (2.0 mL). The reaction mixture was stirred for 24 h at 70 °C. Purification by flash column chromatography on silica gel (CH_2Cl_2/MeOH = 95:5) yielded **12** as a white solid (15 mg, 0.064 mmol, 32%).

R_f = 0.15 (CH_2Cl_2/MeOH = 95:5).

m.p.: 68–71 °C.

^{1}H NMR (600 MHz, $CDCl_3$): δ = 1.32–1.42 (m, 2H, H-3)**, 1.48–1.54 (m, 2H, H-2)**, 1.54–1.65 (m, 2H, H-1), 1.92 (br s, 1H, O–*H*), 3.33–3.38 (m, 2H, H-4)*, 3.53–3.59 (m, 2H, H-5)*, 3.71 (s, 2H, H-7), 4.66 (s, 2H, H-12), 7.23 (d, $^{3}J_{9,10}$ = 7.9 Hz, 2H, H-9), 7.31 (d, $^{3}J_{10,9}$ = 8.0 Hz, 2H, H-10) ppm.

^{13}C NMR (151 MHz, $CDCl_3$): δ = 24.6 (C-1), 25.6 (C-2)*, 26.4 (C-3)*, 40.9 (C-7), 43.0 (C-5)**, 47.4 (C-4)**, 65.2 (C-12), 127.5 (C-10), 128.9 (C-9), 134.9 (C-8), 139.5 (C-11), 169.3 (C-6) ppm.

IR (ATR): ṽ = 3325 (w), 2941 (w), 2855 (w), 1618 (s), 1442 (s), 1047 (m), 1018 (s), 768 (m) cm^{-1}.

HRMS (ESI) for $C_{14}H_{20}NO_2^+$ [$(M+H)^+$] calculated: 234.1489, found 234.1496.

4.4.1.2 Reduction of 2-[4-(morpholine-4-carbonyl)phenyl]-1-(1-piperidyl)ethanone (11) with stoichiometric reducing reagents – Comparison of selectivity

4.4.1.2.1 Reduction of 2-[4-(morpholine-4-carbonyl)phenyl]-1-(1-piperidyl)ethanone (11) with D*I*BAl–H

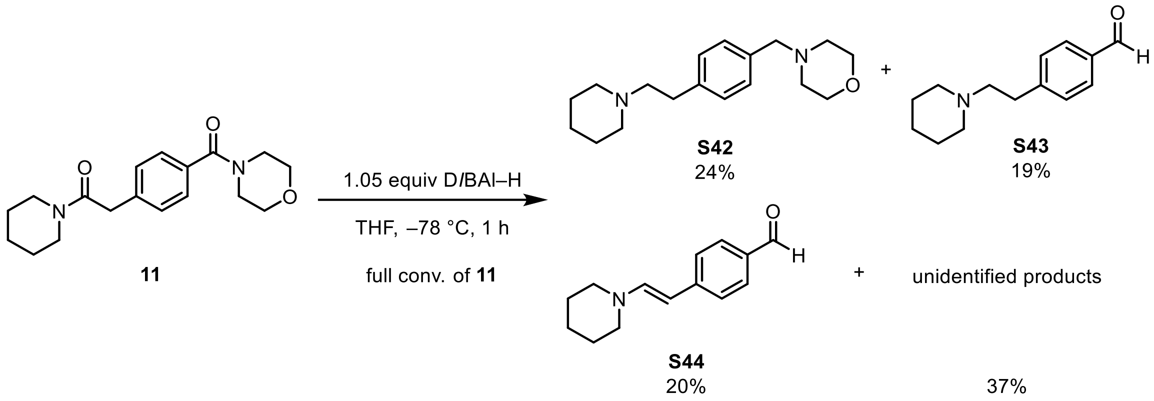

Scheme S16: Reduction of 2-[4-(morpholine-4-carbonyl)phenyl]-1-(1-piperidyl)ethanone (**11**) with D*i*BAl–H. Conversion and selectivity were determined with GC, GC-MS and ^{1}H NMR.

Prepared according to **GP6** from 2-[4-(morpholine-4-carbonyl)phenyl]-1-(1-piperidyl)ethanone (**11**, 32 mg, 0.10 mmol, 1.0 equiv), D*i*BAl–H (1.2 M in toluene, 0.088 mL, 0.11 mmol, 1.05 equiv) in dry THF (1.0 mL). The reaction mixture was stirred for 1 h at –78 °C. Analysis of the crude mixture was carried out by GC, GCMS and/or ^{1}H NMR.

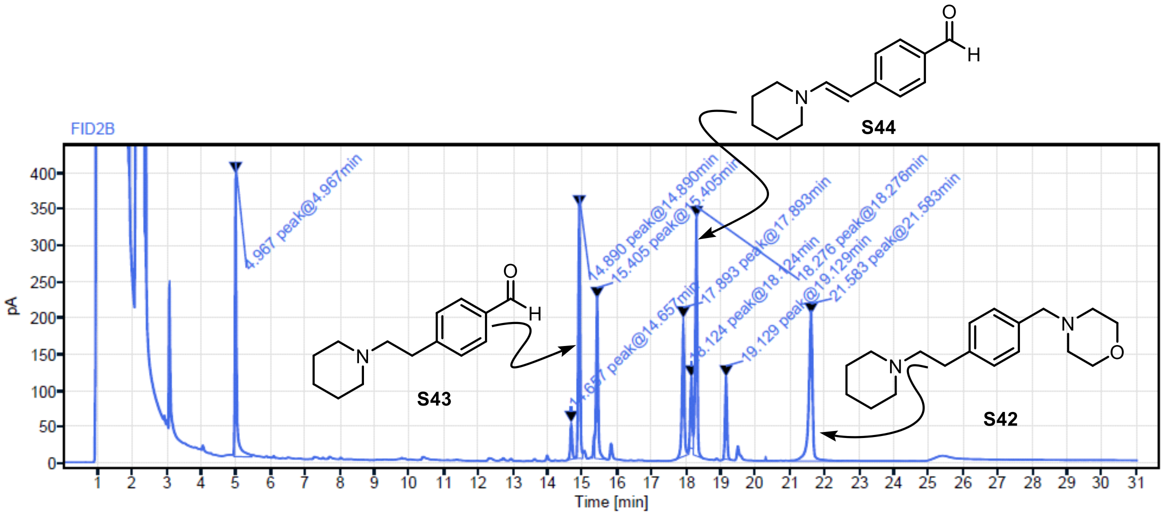

Signal: FID2B

Name	RT [min]	RF	Area	Peak Area Percent
peak@4.967min	4.97		1179.423	14.93
peak@14.657min	14.66		184.716	2.34
peak@14.890min	14.89		1211.134	15.33
peak@15.405min	15.40		864.771	10.95
peak@17.893min	17.89		825.269	10.45
peak@18.124min	18.12		374.021	4.73
peak@18.276min	18.28		1283.888	16.25
peak@19.129min	19.13		427.045	5.41
peak@21.583min	21.58		1549.025	19.61

Figure S5: GC analysis of the reaction mixture of 2-[4-(morpholine-4-carbonyl)phenyl]-1-(1-piperidyl)ethanone (**11**) with D*i*BAl–H (GC method: 40_10_250_10).

4.4.1.2.2 Reduction of 2-[4-(morpholine-4-carbonyl)phenyl]-1-(1-piperidyl)ethanone (11) with $LiAlH_4$

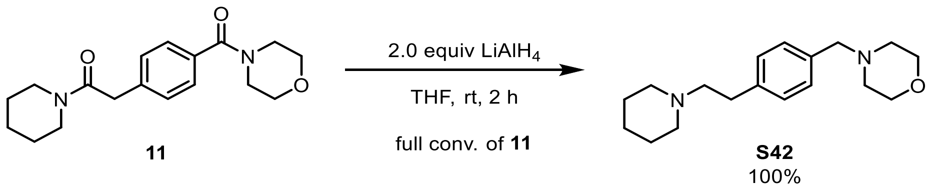

Scheme S17: Reduction of 2-[4-(morpholine-4-carbonyl)phenyl]-1-(1-piperidyl)ethanone (**11**) with $LiAlH_4$. Conversion and selectivity were determined with GC, GC-MS and ^{1}H NMR.

Prepared according to **GP5** from 2-[4-(morpholine-4-carbonyl)phenyl]-1-(1-piperidyl)ethanone (**11**, 16 mg, 0.10 mmol, 1.0 equiv), $LiAlH_4$ (3.8 mg, 0.20 mmol, 2.00 equiv) in dry THF (0.5 mL). The reaction mixture was stirred for 2 h at rt. Analysis of the crude mixture was carried out by GC, GCMS and/or ^{1}H NMR.

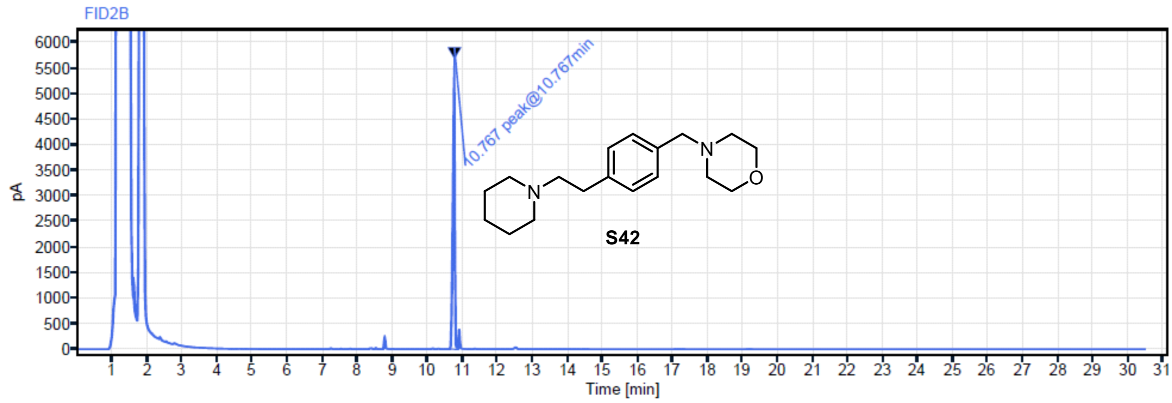

Signal:	FID2B			
**Name**	**RT [min]**	**RF**	**Area**	**Peak Area Percent**
peak@10.767min	10.77		20117.688	100.00

Figure S6: GC analysis of the reaction mixture of 2-[4-(morpholine-4-carbonyl)phenyl]-1-(1-piperidyl)ethanone (**11**) with $LiAlH_4$ (GC method: 40_20_250_20).

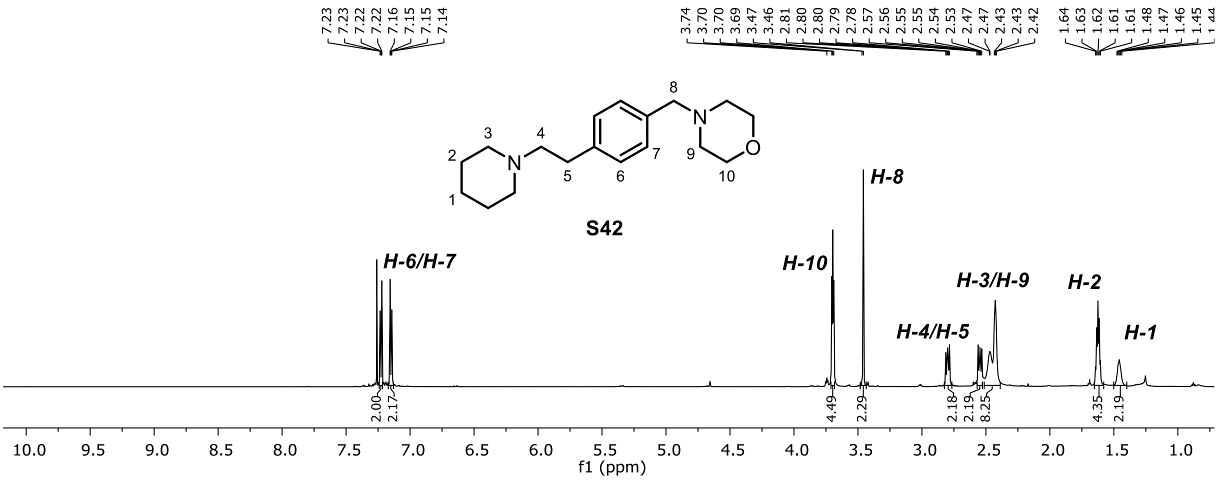

Figure S7: Crude ^{1}H-NMR spectrum of the reaction mixture of 2-[4-(morpholine-4-carbonyl)phenyl]-1-(1-piperidyl)ethanone (**11**) with $LiAlH_4$ (600 MHz, $CDCl_3$).

4.4.2 Reduction of 3-(4-benzoylpiperazin-1-yl)-1-(1-piperidyl)propan-1-one (13)

4.4.2.1 Cu(I)-catalyzed reduction of 3-(4-benzoylpiperazin-1-yl)-1-(1-piperidyl)propan-1-one (13) with H_2

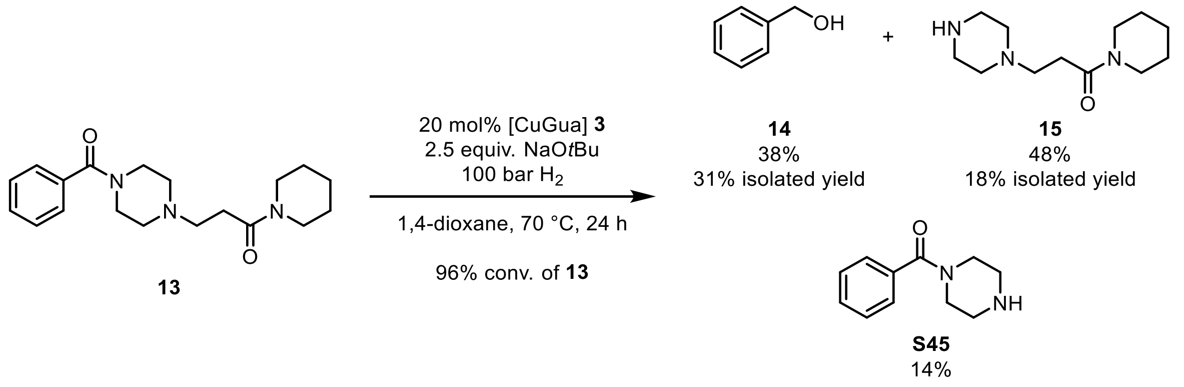

Scheme S18: Site-selective Cu(I)-catalyzed reduction of 3-(4-benzoylpiperazin-1-yl)-1-(1-piperidyl)propan-1-one (**13**) with H_2.

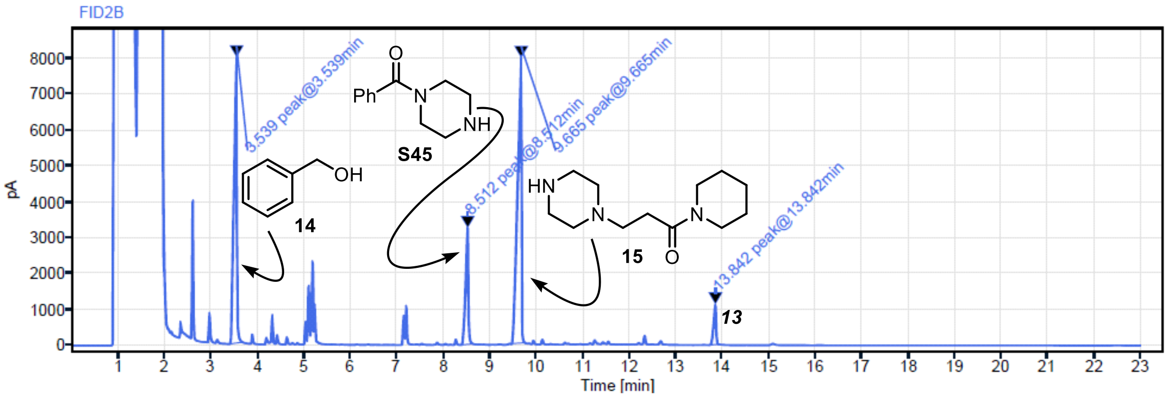

Signal: FID2B

Name	RT [min]	RF	Area	Peak Area Percent
peak@3.539min	3.54		34346.469	35.82
peak@8.512min	8.51		13057.485	13.62
peak@9.665min	9.66		44503.379	46.41
peak@13.842min	13.84		3978.965	4.15

Figure S8: GC analysis of the reaction mixture of the Cu(I)-catalyzed reduction of 3-(4-benzoylpiperazin-1-yl)-1-(1-piperidyl)propan-1-one (**13**) with H_2. No peak at 10.775 min is observed which is attributed to the alcohol after the reduction of the piperidine site (GC method: 40_20_300_10_inlet_305 °C).

4.4.2.1.1 Benzyl alcohol from 3-(4-benzoylpiperazin-1-yl)-1-(1-piperidyl)propan-1-one (14)

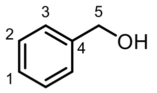

14
C_7H_8O
Mw = 108.14 g mol^{-1}

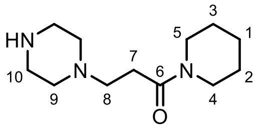

15
$C_{12}H_{23}N_3O$
Mw = 225.34 g mol^{-1}

Prepared according to **GP4** from 3-(4-benzoylpiperazin-1-yl)-1-(1-piperidyl)propan-1-one (**13**, 99 mg, 0.30 mol, 1.0 equiv), [CuGua] **3** (28 mg, 60 µmol, 20 mol%), NaO*t*Bu (72 mg, 0.75 mmol, 2.5 equiv) in 1,4-dioxane (3.0 mL). The reaction mixture was stirred for 24 h at 70 °C. Purification by flash column chromatography on alumina 90 basic (CH_2Cl_2/MeOH = 95:5) yielded amine **15** as a white solid (12 mg, 53 µmol, 18%). A second purification by flash column chromatography on silica gel (CH_2Cl_2) yielded **14** as a colourless oil (10 mg, 0.092 mmol, 31%).

Benzyl alcohol from 3-(4-benzoylpiperazin-1-yl)-1-(1-piperidyl)propan-1-one (14)
The data is in accordance with the data of section **4.2.1**.

3-Piperazin-1-yl-1-(1-piperidyl)propan-1-one (**15**):

R*f* = 0.15 (CH_2Cl_2/MeOH = 9.5:0.5) on alumina 90 basic.
m.p.: 87–90 °C

^{1}H NMR (600 MHz, $CDCl_3$): δ = 1.41–1.60 (m, 5H, H-2, H-3, N–*H*), 1.58–1.69 (m, 2H, H-1), 2.45 (br s, 4H, H-10), 2.49–2.58 (m, 2H, H-8)*, 2.64–2.75 (m, 2H, H-7)*, 2.88 (t, $^{3}J_{9,10}$ = 4.9 Hz, 4H, H-9), 3.36–3.45 (m, 2H, H-4)**, 3.47–3.57 (m, 2H, H-5)** ppm.
^{13}C NMR (151 MHz, $CDCl_3$): δ = 24.7 (C-1), 25.7 (C-2)*, 26.7 (C-3)*, 31.0 (C-8)**, 42.8 (C-5)***, 46.2 (C-9), 46.8 (C-4)***, 54.8 (C-10), 55.1 (C-7)**, 170.0 (C-6) ppm.
IR (ATR): ṽ = 2933 (w), 2855 (w), 1625 (s), 1435 (m), 1219 (m), 1129 (m), 999 (m) cm^{-1}.
HRMS (APCI) $C_{12}H_{24}N_3O^+$ [$(M+H)^+$] calculated: 226.1914, found 226.1905.

4.4.2.2 Reduction of 3-(4-benzoylpiperazin-1-yl)-1-(1-piperidyl)propan-1-one (13) with stoichiometric reducing reagents – Comparison of selectivity

4.4.2.2.1 Reduction of 3-(4-benzoylpiperazin-1-yl)-1-(1-piperidyl)propan-1-one (13) with D*i*BAl–H

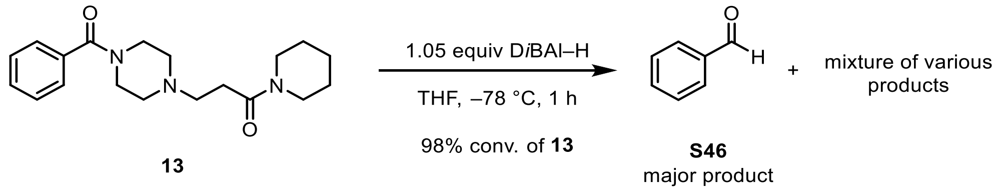

Scheme S19: Reduction of 3-(4-benzoylpiperazin-1-yl)-1-(1-piperidyl)propan-1-one (**13**) with DIBAL–H. Conversion and selectivity were determined with GC, GC-MS and ^{1}H NMR.

Prepared according to **GP6** from 3-(4-benzoylpiperazin-1-yl)-1-(1-piperidyl)propan-1-one (**13**, 16 mg, 0.050 mmol, 1.0 equiv), D*i*BAl–H (1.2M in toluene, 0.042 mL, 0.050 mmol, 1.00 equiv) in dry THF (0.5 mL). The reaction mixture was stirred for 1 h at –78 °C. Analysis of the crude mixture was carried out by GC, GCMS and/or ^{1}H NMR.

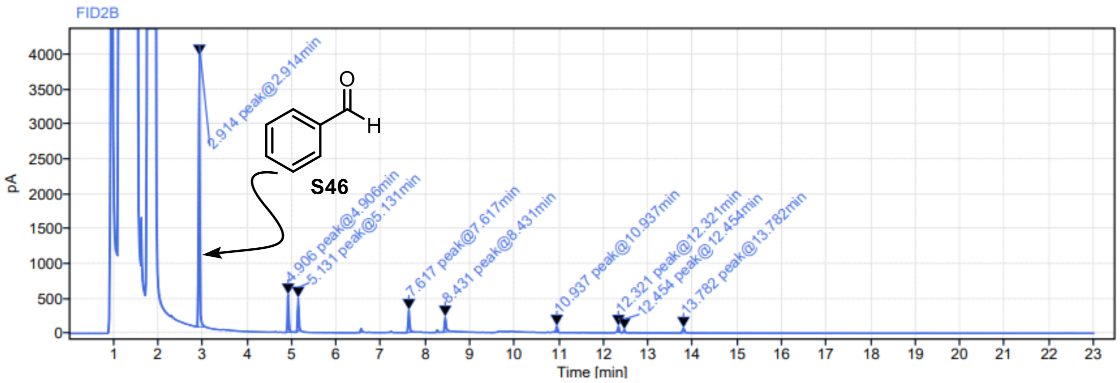

Signal: FID2B

Name	RT [min]	RF	Area	Peak Area Percent
peak@2.914min	2.91		6313.687	64.59
peak@4.906min	4.91		936.685	9.58
peak@5.131min	5.13		711.184	7.28
peak@7.617min	7.62		674.918	6.90
peak@8.431min	8.43		513.635	5.25
**Name**	**RT [min]**	**RF**	**Area**	**Peak Area Percent**
peak@10.937min	10.94		190.721	1.95
peak@12.321min	12.32		173.572	1.78
peak@12.454min	12.45		60.845	0.62
peak@13.782min	13.78		200.064	2.05

Figure S9: GC analysis of the reaction mixture of 3-(4-benzoylpiperazin-1-yl)-1-(1-piperidyl)propan-1-one (**13**) with D*i*BAl–H (GC method: 40_20_300_10_inlet_305 °C).

4.4.2.2.2 Reduction of 3-(4-benzoylpiperazin-1-yl)-1-(1-piperidyl)propan-1-one (13) with $LiAlH_4$

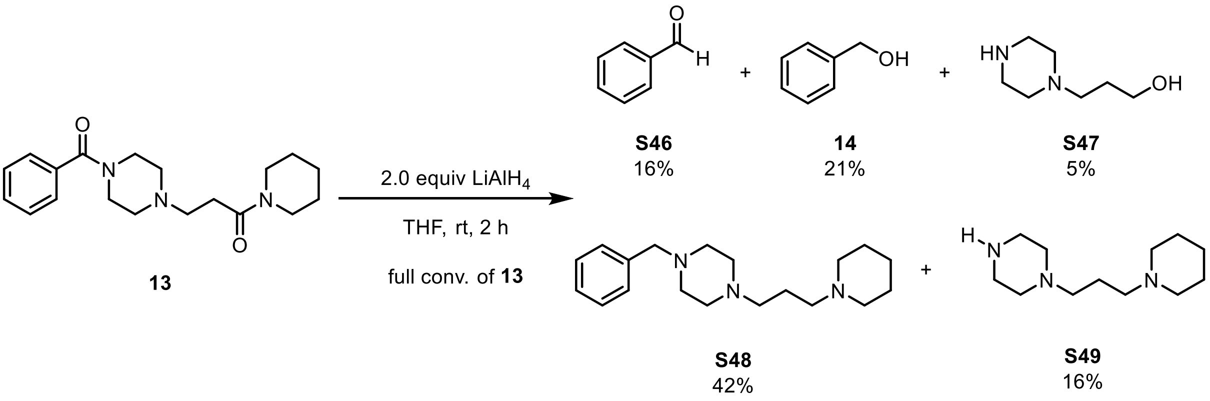

Scheme S20: Reduction of 3-(4-benzoylpiperazin-1-yl)-1-(1-piperidyl)propan-1-one (**13**) with $LiAlH_4$. Conversion and selectivity were determined with GC, GC-MS and ^{1}H NMR.

Prepared according to **GP5** from 3-(4-benzoylpiperazin-1-yl)-1-(1-piperidyl)propan-1-one (**13**, 16 mg, 0.050 mmol, 1.0 equiv), $LiAlH_4$ (3.8 mg, 0.20 mmol, 2.00 equiv) in dry THF (0.5 mL). The reaction mixture was stirred for 2 h at rt. Analysis of the crude mixture was carried out by GC, GCMS and/or ^{1}H NMR.

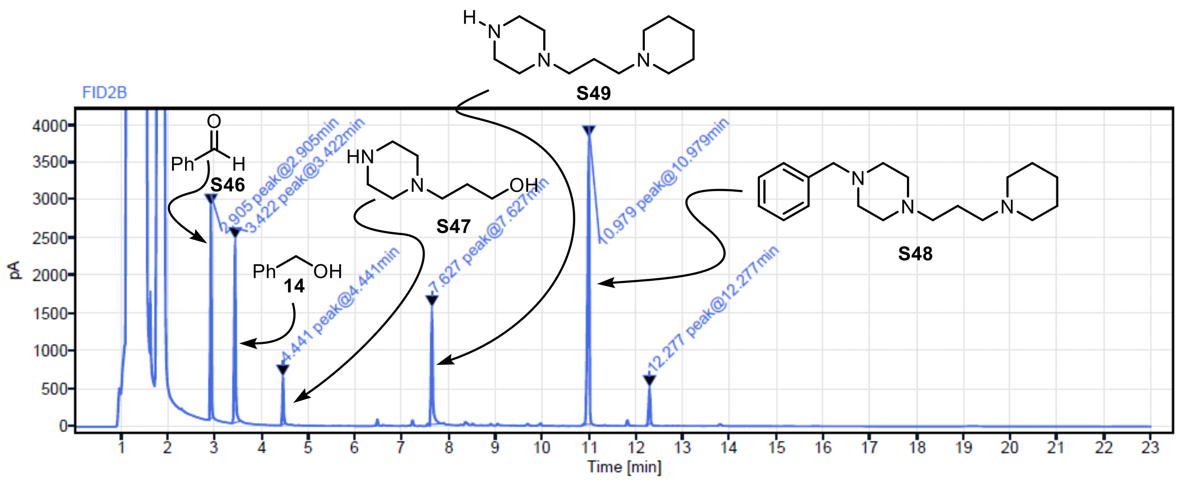

Signal:	FID2B			
**Name**	**RT [min]**	**RF**	**Area**	**Peak Area Percent**
peak@2.905min	2.90		4037.217	16.35
peak@3.422min	3.42		4920.180	19.93
peak@4.441min	4.44		1069.571	4.33
peak@7.627min	7.63		3758.515	15.22
peak@10.979min	10.98		9803.597	39.71

Figure S10: GC analysis of the reaction mixture of 3-(4-benzoylpiperazin-1-yl)-1-(1-piperidyl)propan-1-one (**13**) with $LiAlH_4$ (GC method: 40_20_300_10_inlet_305 °C).

4.4.3 Reduction of *N,N*-diethyl-4-(morpholine-4-carbonyl)benzamide (16a)

4.4.3.1 Cu(I)-catalyzed reduction of *N,N*-diethyl-4-(morpholine-4-carbonyl)benzamide (16a) with H_2

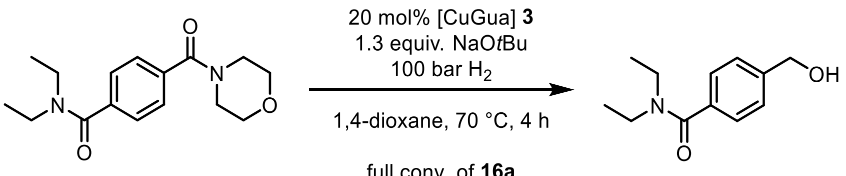

Scheme S21: Site-selective Cu(I)-catalyzed reduction reduction of *N,N*-diethyl-4-(morpholine-4-carbonyl)benzamide (**16a**) with H_2.

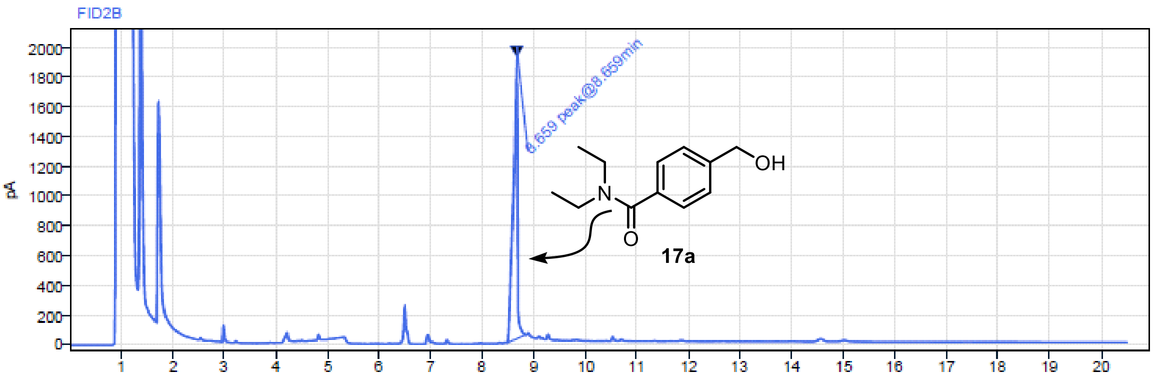

Signal:	FID2B				
Name	RT [min]	RF	Area	Peak Area Percent
peak@8.659min	8.66		10281.991	100.00

Figure S11: GC analysis of the crude reaction mixture of the Cu(I)-catalyzed reduction of *N,N*-diethyl-4-(morpholine-4-carbonyl)benzamide (**16a**) with H_2 (GC method: 40_20_250_10).

4.4.3.1.1 *N,N*-Diethyl-4-(hydroxymethyl)benzamide (17a)

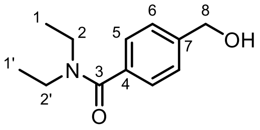

17a

$C_{12}H_{17}NO_2$

Mw = 207.27 g mol^{-1}

Prepared according to **GP4** from *N,N*-diethyl-4-(morpholine-4-carbonyl)benzamide (**16a**, 58 mg, 0.20 mol, 1.0 equiv), [CuGua] **3** (19 mg, 40 µmol, 10 mol%), NaO*t*Bu (25 mg, 0.26 mmol, 1.3 equiv) in 1,4-dioxane (2.0 mL). The reaction mixture was stirred for 4 h at 70 °C. Purification by flash column chromatography on silica gel (CH_2Cl_2/MeOH = 98: 2) yielded **17a** as a pale yellow solid (28 mg, 0.14 mmol, 70%)

R*f* = 0.45 (CH_2Cl_2/MeOH = 95:5).

^{1}H NMR (600 MHz, $CDCl_3$): δ = 1.11 (s, 3H, H-1)*, 1.25 (s, 3H, H-1')*, 1.72 (t, $^3J_{O\text{–}H,8}$ = 5.9 Hz, 1H, O–*H*), 3.25 (s, 2H, H-2)**, 3.55 (s, 2H, H-2')**, 4.73 (d, $^3J_{8,\,O\text{–}H}$ = 5.8 Hz, 2H, H-8), 7.37 (d, $^3J_{6,5}$ = 8.4 Hz, 2H, H-6), 7.39 (d, $^3J_{5,6}$ = 8.2 Hz, 2H, H-5) ppm.

^{13}C NMR (151 MHz, $CDCl_3$): δ = 13.0 (C-1)*, 14.3 (C-1')*, 39.4 (C-2')**, 43.4 (C-2)**, 64.4 (C-8), 126.3 (C-5), 126.6 (C-6), 135.9 (C-4), 142.7 (C-7), 171.6 (C-3) ppm.

HRMS (APCI) for $C_{12}H_{18}O_2N^+$ [$(M+H)^+$] calculated: 208.1332, found 208.1336.

The data is in accordance with literature.[52]

4.4.3.2 Reduction of *N,N*-diethyl-4-(morpholine-4-carbonyl)benzamide (16a) with stoichiometric reducing reagents – Comparison of selectivity

4.4.3.2.1 Reduction of *N,N*-diethyl-4-(morpholine-4-carbonyl)benzamide (16a) with D*i*BAl–H

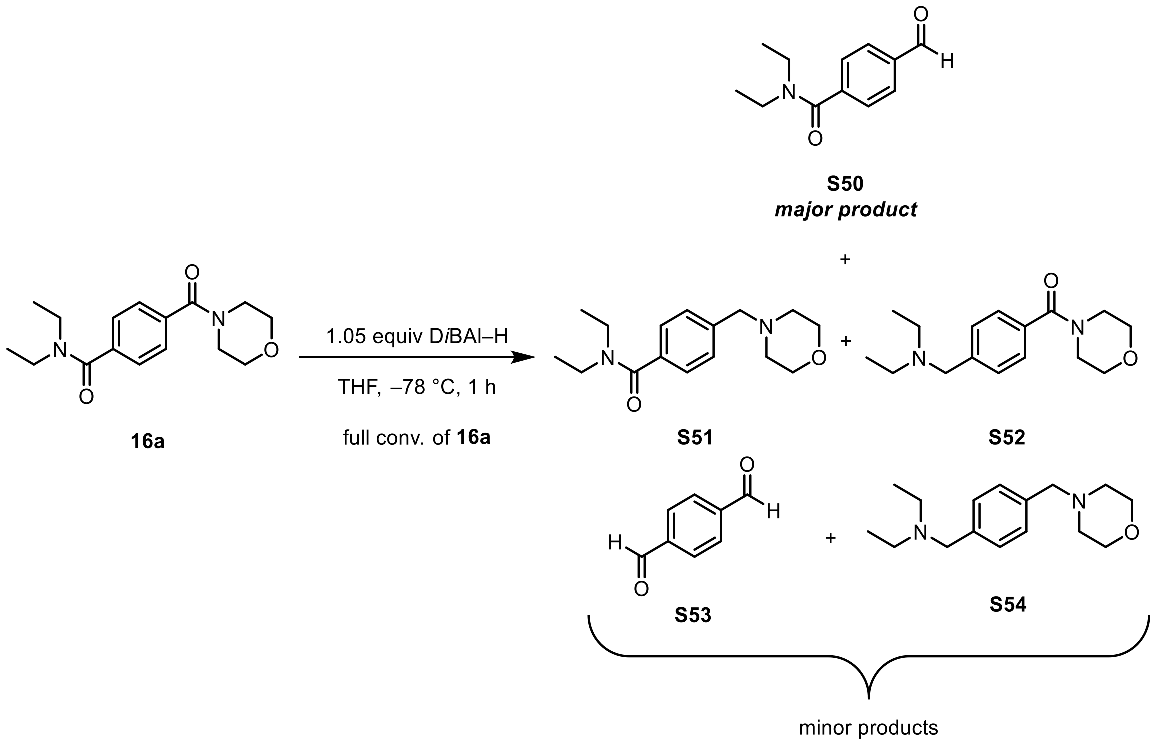

Scheme S22: Reduction of *N,N*-diethyl-4-(morpholine-4-carbonyl)benzamide (**16a**) with D*i*BAl–H. Conversion and selectivity were determined with GC, GC-MS and ^{1}H NMR.

Prepared according to **GP6** from *N,N*-diethyl-4-(morpholine-4-carbonyl)benzamide (**16a**, 29 mg, 0.10 mmol, 1.0 equiv), D*i*BAl–H (1.2 M in toluene, 0.088 mL, 0.11 mmol, 1.05 equiv) in dry THF (1.0 mL). The reaction mixture was stirred for 1 h at –78 °C. Analysis of the crude mixture was carried out by GC, GCMS and/or ^{1}H NMR.

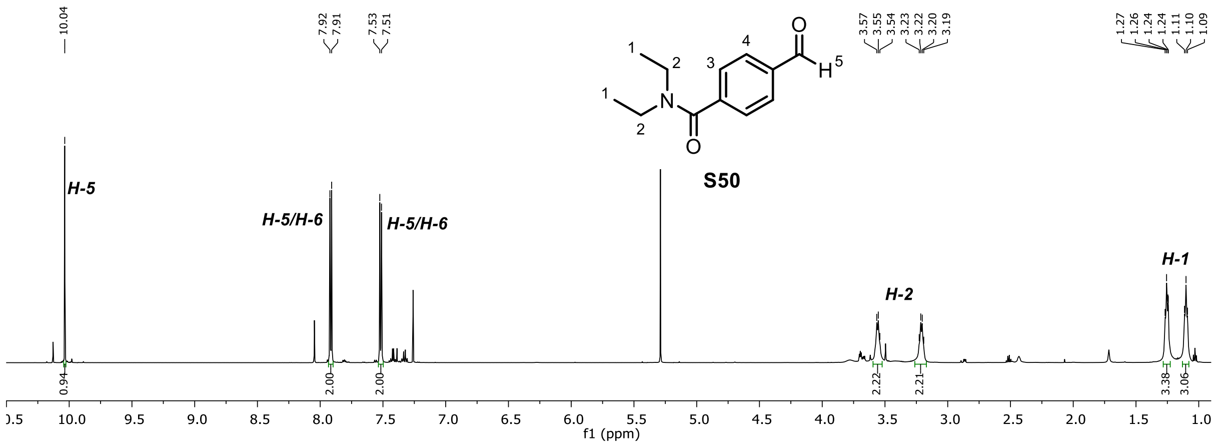

Figure S12: ^{1}H NMR spectrum of the reaction mixture of *N,N*-diethyl-4-(morpholine-4-carbonyl)benzamide (**16a**) with D*i*BAl–H (600 MHz, $CDCl_3$).

4.4.3.2.2 Reduction of *N,N*-diethyl-4-(morpholine-4-carbonyl)benzamide (16a) with $LiAlH_4$

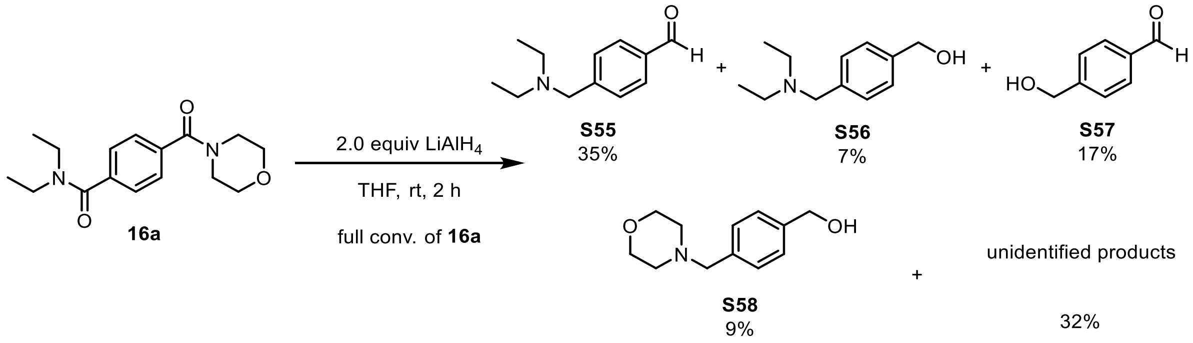

Scheme S23: Reduction of *N,N*-diethyl-4-(morpholine-4-carbonyl)benzamide (**16a**) with $LiAlH_4$. Conversion and selectivity were determined with GC, GC-MS and ^1H NMR.

Prepared according to **GP5** from *N,N*-diethyl-4-(morpholine-4-carbonyl)benzamide (**16a**, 29 mg, 0.10 mmol, 1.0 equiv), $LiAlH_4$ (7.6 mg, 0.20 mmol, 2.00 equiv) in dry THF (1.0 mL). The reaction mixture was stirred for 2 h at rt. Analysis of the crude mixture was carried out by GC, GCMS and/or ^1H NMR.

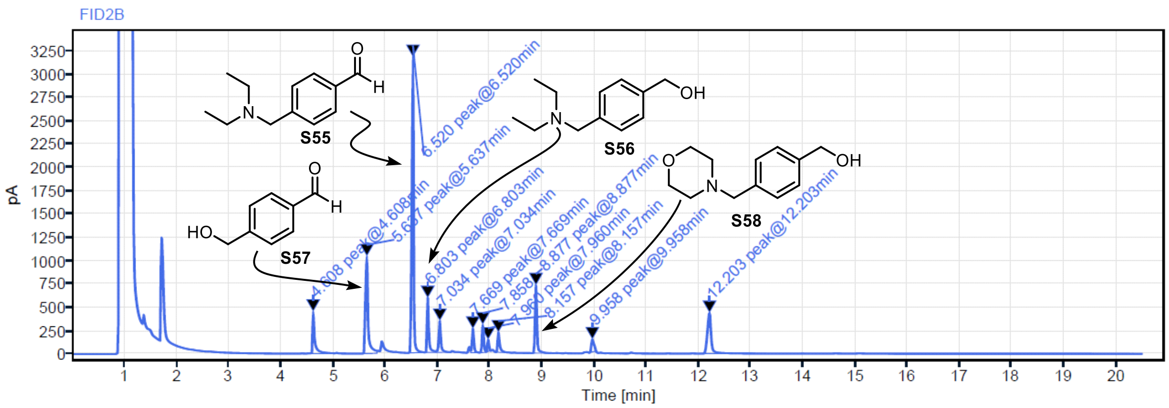

Name	RT [min]	RF	Area	Peak Area Percent
peak@4.608min	4.61		1349.434	6.22
peak@5.637min	5.64		3697.312	17.05
peak@6.520min	6.52		7525.015	34.70
peak@6.803min	6.80		1407.789	6.49
peak@7.034min	7.03		921.655	4.25
peak@7.669min	7.67		615.303	2.84

Name	RT [min]	RF	Area	Peak Area Percent
peak@7.960min	7.96		283.814	1.31
peak@8.157min	8.16		606.380	2.80
peak@8.877min	8.88		1840.669	8.49
peak@9.958min	9.96		462.260	2.13
peak@12.203min	12.20		2305.943	10.63

Figure S13: GC analysis of the crude reaction mixture of *N,N*-diethyl-4-(morpholine-4-carbonyl)benzamide (**16a**) with $LiAlH_4$ (GC method: 40_20_250_10).

4.4.4 Reduction of *N*-(2-methoxyethyl)-*N*-methyl-4-(morpholine-4-carbonyl)benzamide (16b)

4.4.4.1 Cu(I)-catalyzed reduction of *N*-(2-methoxyethyl)-*N*-methyl-4-(morpholine-4-carbonyl)benzamide (16b) with H_2

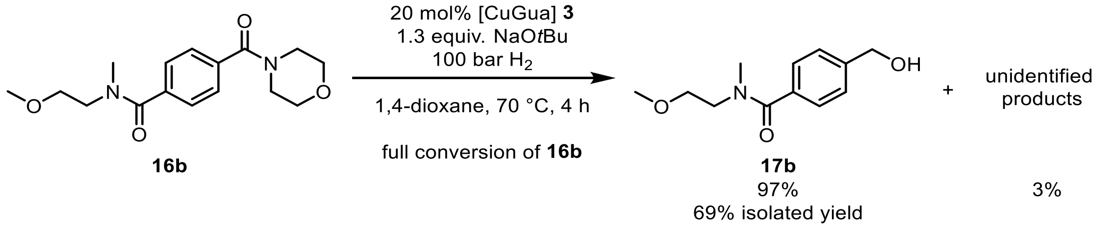

Scheme S24: Site-selective Cu(I)-catalyzed reduction of *N*-(2-methoxyethyl)-*N*-methyl-4-(morpholine-4-carbonyl)benzamide (**16b**) with H_2.

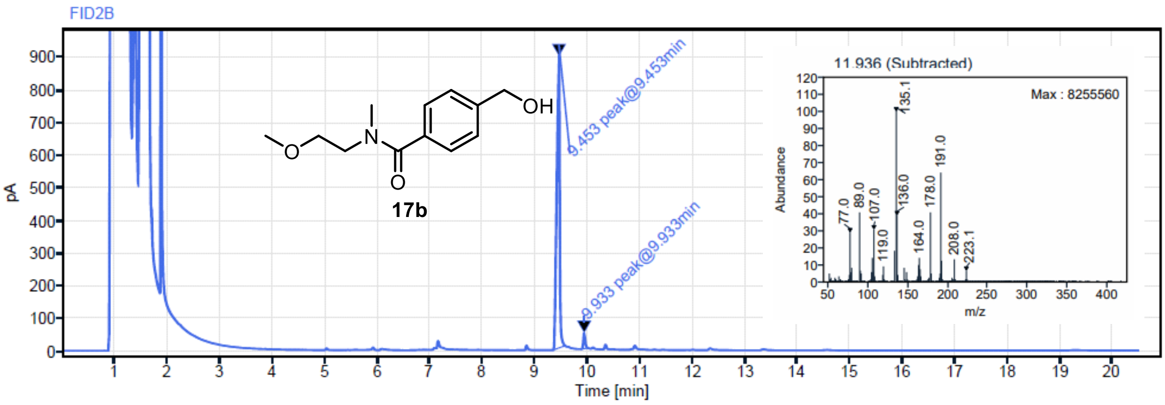

Signal: FID2B

Name	RT [min]	RF	Area	Peak Area Percent	Concentration []
peak@9.453min	9.45		3412.484	96.88	
peak@9.933min	9.93		109.917	3.12	

Figure S14: GC data of the crude reaction mixture of the Cu(I)-catalyzed reduction of *N*-(2-methoxyethyl)-*N*-methyl-4-(morpholine-4-carbonyl)benzamide (**16b**) with H_2 (GC method: 40_20_250_10).

4.4.4.1.1 4-(Hydroxymethyl)-*N*-(2-methoxyethyl)-*N*-methyl-benzamide (17b)

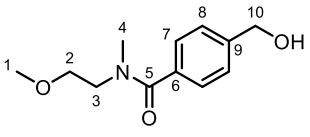

17b
$C_{12}H_{17}NO_3$
Mw = 223.27 g mol-1

Prepared according to **GP4** from *N*-(2-methoxyethyl)-*N*-methyl-4-(morpholine-4-carbonyl)benzamide (**16b**, 61 mg, 0.20 mol, 1.0 equiv), [CuGua] **3** (19 mg, 40 µmol, 10 mol%), NaO*t*Bu (25 mg, 0.26 mmol, 1.3 equiv) in 1,4-dioxane (2.0 mL). The reaction mixture was stirred for 4 h at 70 °C. Purification by flash column chromatography on silica gel (CH_2Cl_2/MeOH = 98:2) yielded **17b** as a colourless oil (31 mg, 0.14 mmol, 69%).

The product was obtained as a mixture of amide *E* and *Z* isomers. The corresponding H and C atoms have been assigned by letters a and b. There is no correlation between the letters and the isomers.

R_f = 0.15 (CH_2Cl_2/MeOH = 98:2).

^{1}**H NMR** (600 MHz, $CDCl_3$): δ = 1.86 (s, 1H, O–*H*), 2.88–3.25 (m, 3H, H-4), 3.19–3.51 (m, 5H, H-1, 1 x H-2a, 1 x H-3a), 3.56–3.88 (m, 2H, 1 x H-2b, 1 x H-3b), 4.72 (d, $^3J_{10,O-H}$ = 5.7 Hz, 2H, H-10), 7.38 (d, $^3J_{8,7}$ = 8.0 Hz, 2H, H-8), 7.41 (d, $^3J_{7,8}$ = 7.7 Hz, 2H, H-7) ppm.
^{13}**C NMR** (151 MHz, $CDCl_3$): δ = 33.5 (C-4), 39.3 (C-4), 47.7 (C-3a), 50.9 (C-3b), 59.0 (C-1), 64.6 (C-10), 70.3 (C-2a), 71.0 (C-2b), 126.6 (C-8), 127.2 (C-7), 135.4 (C-6), 142.7 (C-9), 142.9 (C-9), 171.7 (C-5), 172.6 (C-5) ppm.
IR (ATR): ṽ = 3388 (w), 2930 (w), 2877 (w), 1610 (s), 1401 (m), 1114 (m), 1014 (m), 831 (m) cm^{-1}.
HRMS (ESI) for $C_{12}H_{18}NO_3{}^+$ [$(M+H)^+$] calculated: 224.1281, found 224.1273.

4.4.4.2 Reduction of (*N*-(2-methoxyethyl)-*N*-methyl-4-(morpholine-4-carbonyl) benzamide (16b) with stoichiometric reducing reagents – Comparison of selectivity

4.4.4.2.1 Reduction of (*N*-(2-methoxyethyl)-*N*-methyl-4-(morpholine-4-carbonyl) benzamide (16b) with D*i*BAl–H

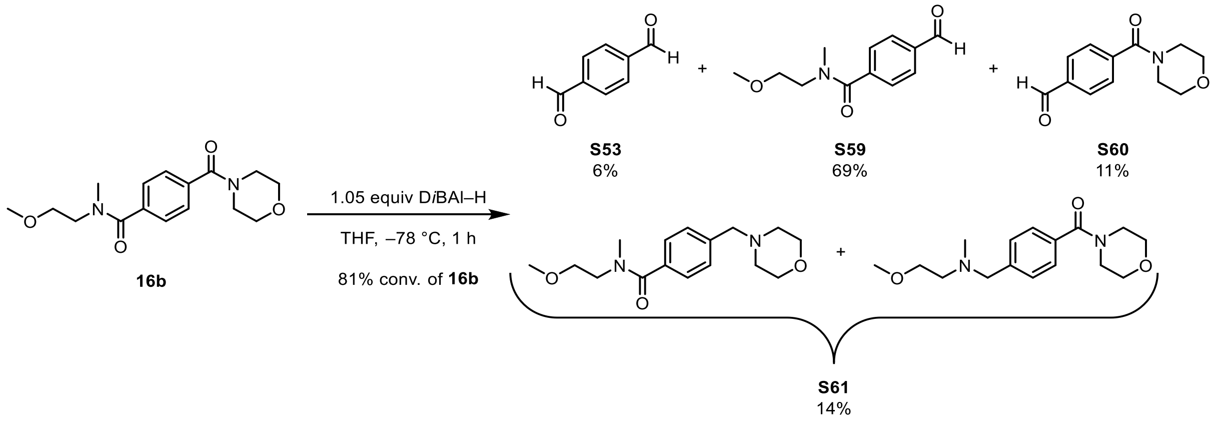

Scheme S25: Reduction of (*N*-(2-methoxyethyl)-*N*-methyl-4-(morpholine-4 carbonyl) (**16b**) with D*i*BAl–H. Conversion and selectivity were determined with GC, GC-MS and ^{1}H NMR.

Prepared according to **GP6** from (*N*-(2-methoxyethyl)-*N*-methyl-4-(morpholine-4 carbonyl) (**16b**, 31 mg, 0.10 mmol, 1.0 equiv), D*i*BAl–H (1.2 M in toluene, 0.088 mL, 0.11 mmol, 1.05 equiv) in dry THF (1.0 mL). The reaction mixture was stirred for 1 h at –78 °C. Analysis of the crude mixture was carried out by GC, GCMS and/or ^{1}H NMR.

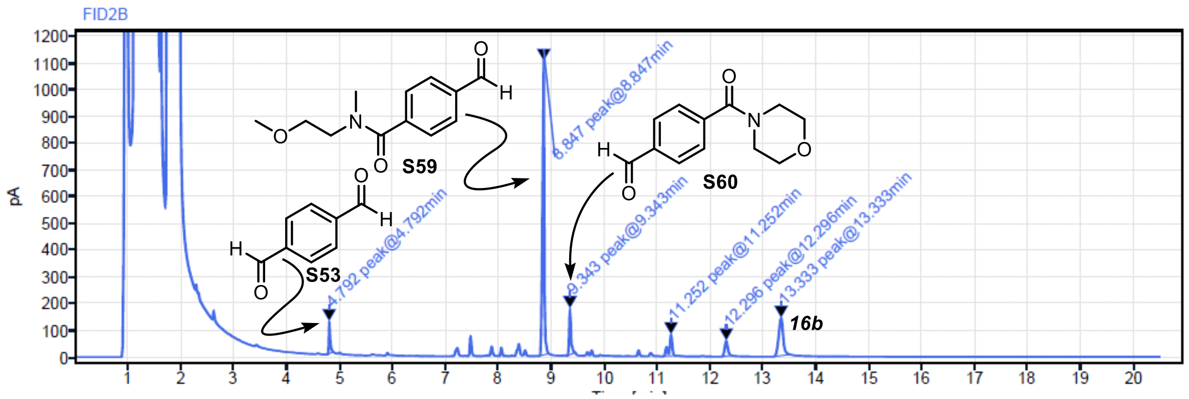

Signal: FID2B

Name	RT [min]	RF	Area	Peak Area Percent
peak@4.792min	4.79		216.022	4.80
peak@8.847min	8.85		2537.600	56.38
peak@9.343min	9.34		392.315	8.72
peak@11.252min	11.25		313.950	6.98
peak@12.296min	12.30		225.434	5.01
peak@13.333min	13.33		815.326	18.12

Figure S15: GC analysis of the reaction mixture of (*N*-(2-methoxyethyl)-*N*-methyl-4-(morpholine-4 carbonyl) (**16b**) with D*i*BAl–H (GC method: 40_20_250_10).

4.4.4.2.2 Reduction of (*N*-(2-methoxyethyl)-*N*-methyl-4-(morpholine-4-carbonyl) benzamide (16b) with $LiAlH_4$

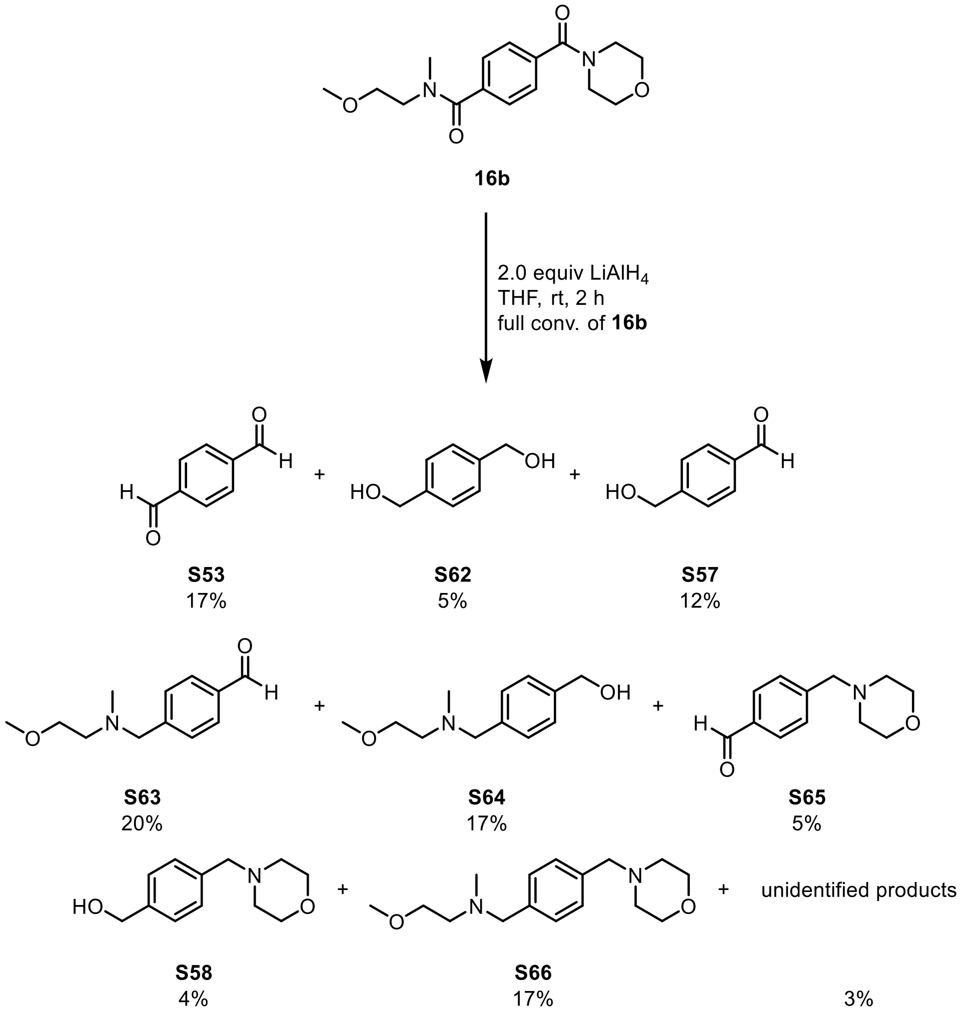

Scheme S26: Reduction of (N-(2-methoxyethyl)-N-methyl-4-(morpholine-4 carbonyl) (**16b**) with $LiAlH_4$. Conversion and selectivity were determined with GC, GC-MS and ^1H NMR.

Prepared according to **GP5** from (N-(2-methoxyethyl)-N-methyl-4-(morpholine-4 carbonyl) (**16b**, 31 mg, 0.10 mmol, 1.0 equiv), $LiAlH_4$ (7.6 mg, 0.20 mmol, 2.00 equiv) in dry THF (1.0 mL). The reaction mixture was stirred for 2 h at rt. Analysis of the crude mixture was carried out by GC, GCMS and/or ^1H NMR.

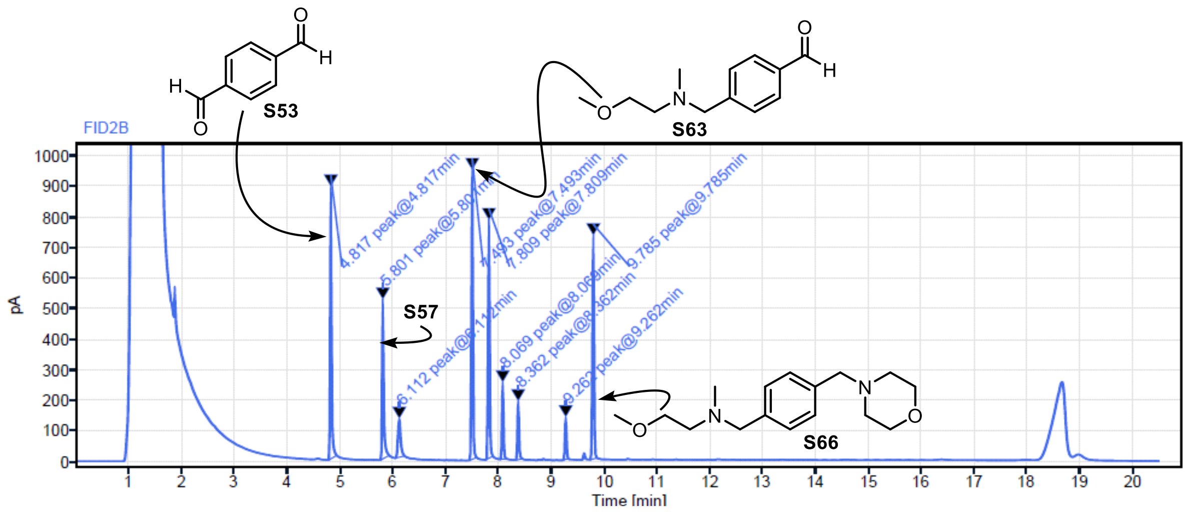

Signal: FID2B

Name	RT [min]	RF	Area	Peak Area Percent
peak@4.817min	4.82		1563.414	17.30
peak@5.801min	5.80		1063.495	11.77
peak@6.112min	6.11		435.038	4.81
peak@7.493min	7.49		1816.644	20.10
peak@7.809min	7.81		1523.184	16.85
peak@8.069min	8.07		471.118	5.21
peak@8.362min	8.36		377.698	4.18
peak@9.262min	9.26		273.122	3.02
peak@9.785min	9.78		1514.787	16.76

Figure S16: GC analysis of the reaction mixture of (N-(2-methoxyethyl)-N-methyl-4-(morpholine-4 carbonyl) (**16b**) with $LiAlH_4$ (GC method: 40_20_250_10).

4.4.5 Reduction of (4-(morpholine-4-carbonyl)phenyl)(piperidin-1-yl)methanone (16c)

4.4.5.1 Cu(I)-catalyzed reduction of (4-(morpholine-4-carbonyl)phenyl)(piperidin-1-yl)methanone (16c) with H_2

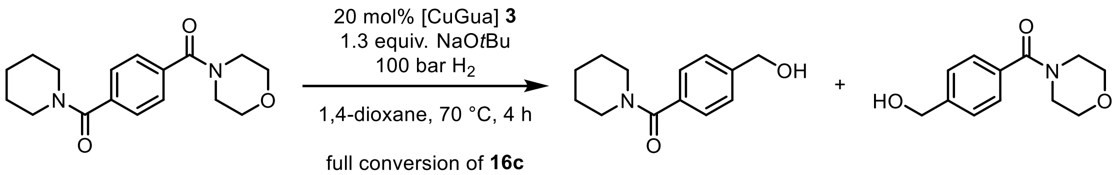

Scheme S27: Site-selective Cu(I)-catalyzed reduction of (4-(morpholine-4-carbonyl)phenyl)(piperidin-1-yl)methanone (**16c**) with H_2.

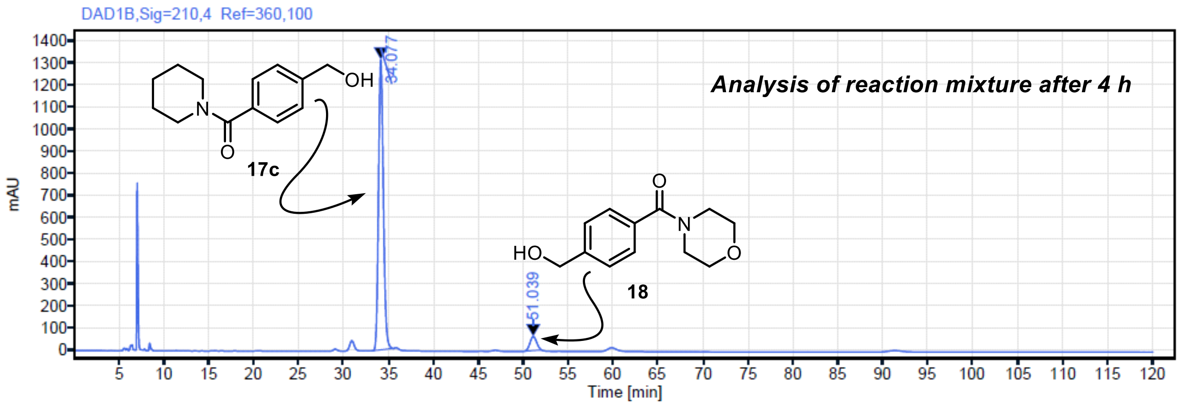

Signal: DAD1B,Sig=210,4 Ref=360,100

RT [min]	Type	Width [min]	Area	Height	Area%	Name
34.077	MM m	2.61	49196.01	1314.07	93.72	
51.039	MM m	1.89	3296.83	63.81	6.28	
		**Sum**	**52492.84**			

Figure S17: HPLC analysis of the reaction mixture of the Cu(I)-catalyzed reduction of 4-(morpholine-4-carbonyl)phenyl)(piperidin-1-yl)methanone (**16c**) with H_2.

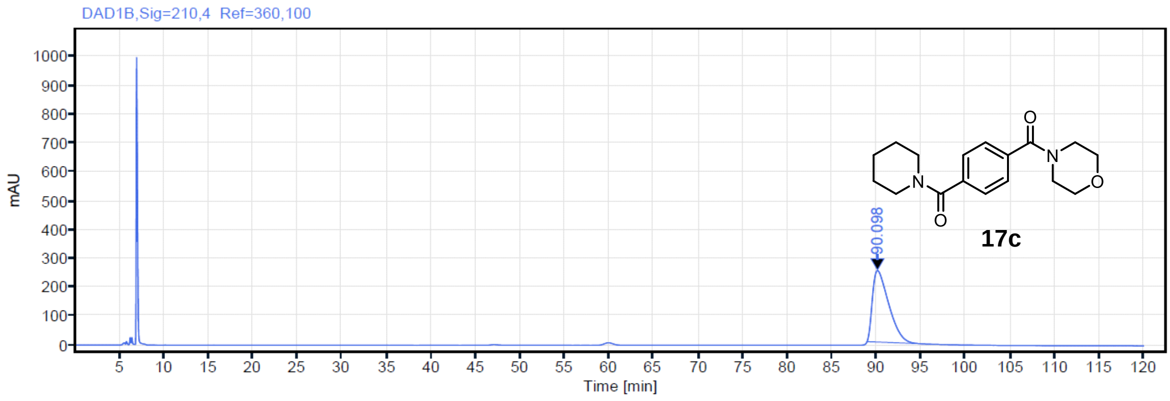

Signal:	DAD1B,Sig=210,4 Ref=360,100					
**RT [min]**	**Type**	**Width [min]**	**Area**	**Height**	**Area%**	**Name**
90.098	BV	5.62	31579.71	248.07	100.00	peak@90.098min
		**Sum**	**31579.71**			

Figure S18: HPLC trace of pure (4-(morpholine-4-carbonyl)phenyl)(piperidin-1-yl)methanone (**16c**).

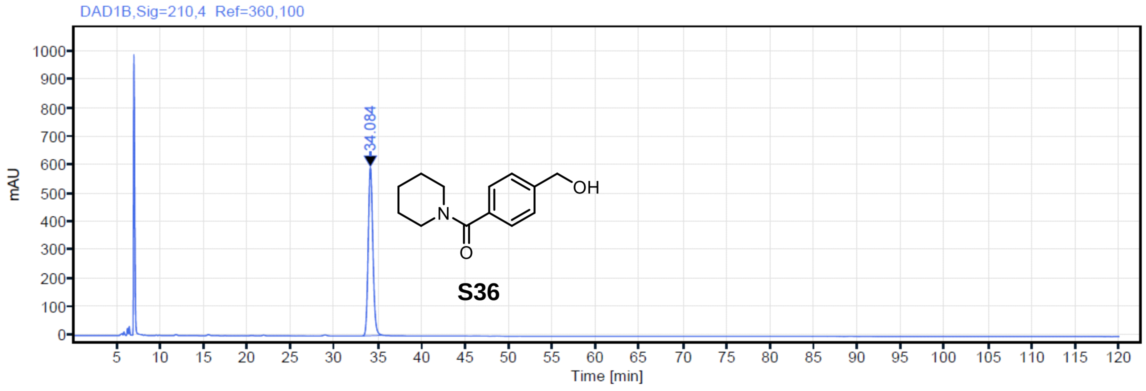

Signal:	DAD1B,Sig=210,4 Ref=360,100					
**RT [min]**	**Type**	**Width [min]**	**Area**	**Height**	**Area%**	**Name**
34.084	MM m	2.41	21984.02	592.79	100.00	peak@34.077min
		**Sum**	**21984.02**			

Figure S19: HPLC trace of individually synthesized [4-(hydroxymethyl)phenyl]-(1-piperidyl)methanone (**S36**).

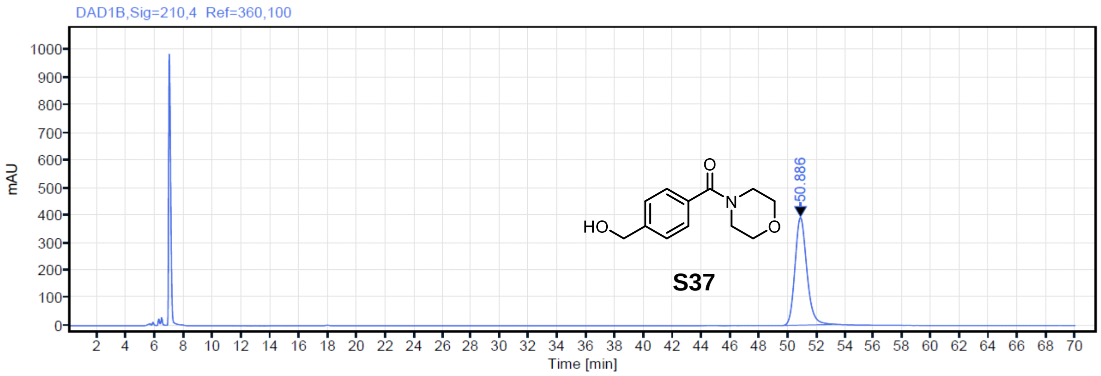

Signal: DAD1B,Sig=210,4 Ref=360,100

RT [min]	Type	Width [min]	Area	Height	Area%	Name
50.886	VV	3.43	22310.51	390.21	100.00	peak@50.886min
		**Sum**	**22310.51**			

Figure S20: HPLC trace of individually synthesized [4-(hydroxymethyl)phenyl]-morpholino-methanone (**S37**).

4.4.5.1.1 (4-(Hydroxymethyl)phenyl)(piperidin-1-yl)methanone (17c)

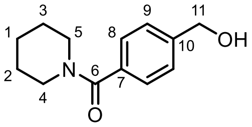

17c
$C_{13}H_{17}NO_2$
Mw = 219.28 g mol^{-1}

Prepared according to **GP4** from (4-(morpholine-4-carbonyl)phenyl)(piperidin-1-yl)methanone (**16c**, 61 mg, 0.20 mol, 1.0 equiv), [CuGua] **3** (19 mg, 40 µmol, 20 mol%), NaO*t*Bu (25 mg, 0.26 mmol, 1.3 equiv) in 1,4-dioxane (2.0 mL). The reaction mixture was stirred for 4 h at 70 °C. At this stage a sample for HPLC analysis (0.5 mL) was taken and analyzed by the following method: (AD-H, *n*heptane/*i*PrOH, = 90:10, flow rate 0.5 mL/min, 25 bar). Purification by flash column chromatography on silica gel (CH_2Cl_2/MeOH = 98:2) yielded **17c** as a pale yellow solid (32 mg, 0.15 mmol, 73%).

R*f* = 0.30 (CH_2Cl_2/MeOH = 98: 2).

m.p.: 112–115 °C.

^{1}H NMR (600 MHz, $CDCl_3$): δ = 1.50 (s, 2H, H-1), 1.67 (s, 4H, H-2, H-3), 3.19 (s, 1H, O–*H*), 3.32 (s, 2H, H-4)*, 3.69 (s, 2H, H-5)*, 4.65 (s, 2H, H-11), 7.30 (s, 4H, H-8, H-9) ppm.

^{13}C NMR (151 MHz, $CDCl_3$): δ = 24.6 (C-2)*, 25.7 (C-3)*, 26.6 (C-1), 43.3 (C-4)**, 48.9 (C-5)**, 64.5 (C-11), 126.6 (C-8), 126.9 (C-9), 135.2 (C-7), 142.9 (C-10), 170.5 (C-6) ppm.
IR (ATR): ṽ = 3336 (w), 2937 (w), 2859 (w), 1595 (m), 1446 (m), 1275 (m), 757 (m) cm^{-1}.
HRMS (APCI) for $C_{13}H_{18}NO_2^+$ $[(M+H)^+]$ calculated: 220.1332, found 220.1331.

4.4.5.2 Reduction of (4-(morpholine-4-carbonyl)phenyl)(piperidin-1-yl)methanone (16c) with stoichiometric reducing reagents – Comparison of selectivity

4.4.5.2.1 Reduction of (4-(morpholine-4-carbonyl)phenyl)(piperidin-1-yl)methanone (16c) with D*i*BAl–H

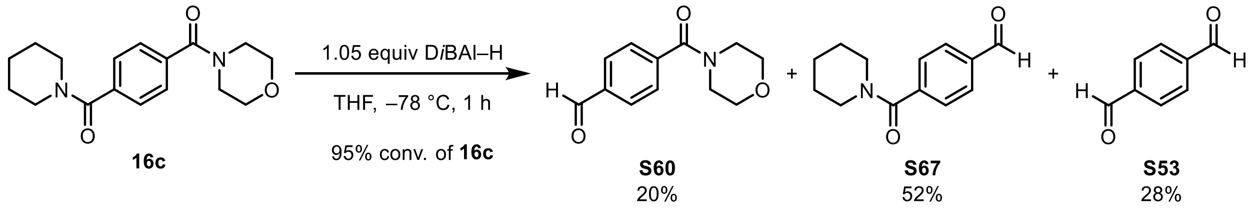

Scheme S28: Reduction of (4-(morpholine-4-carbonyl)phenyl)(piperidin-1-yl)methanone (**16c**) with D*i*BAl–H. Conversion and selectivity were determined with GC, GC-MS and ^{1}H NMR.

Prepared according to **GP6** from (4-(morpholine-4-carbonyl)phenyl)(piperidin-1-yl)methanone (**16c**, 30 mg, 0.10 mmol, 1.0 equiv), D*i*BAl–H (1.2 M in toluene, 0.088 mL, 0.11 mmol, 1.05 equiv) in dry THF (1.0 mL). The reaction mixture was stirred for 1 h at –78 °C. Analysis of the crude mixture was carried out by GC, GCMS and/or ^{1}H NMR.

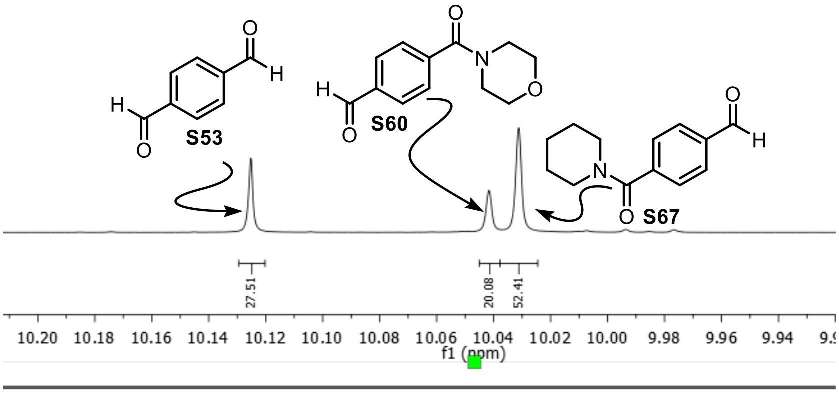

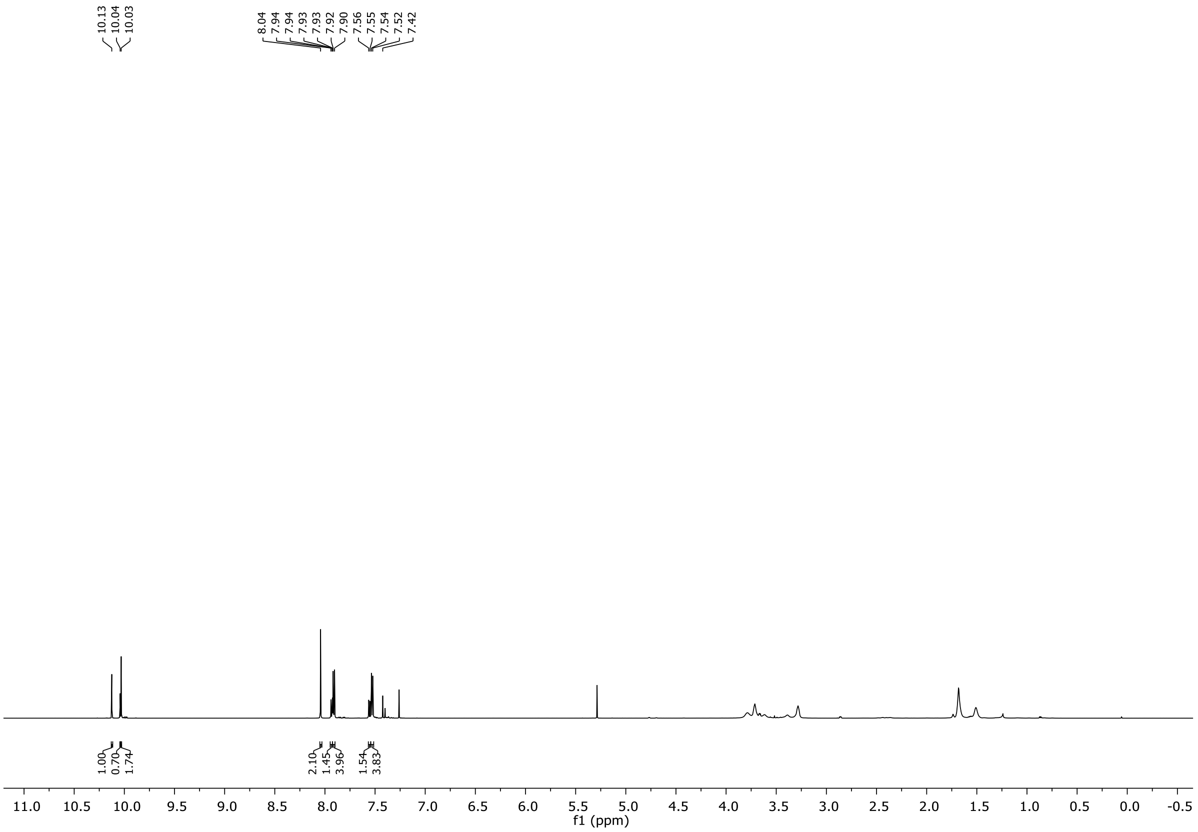

Figure S21: Analysis by ^{1}H NMR of the reaction mixture of (4-(morpholine-4-carbonyl)phenyl)(piperidin-1-yl)methanone (**16c**) with D*i*BAl–H (600 MHz, $CDCl_3$).

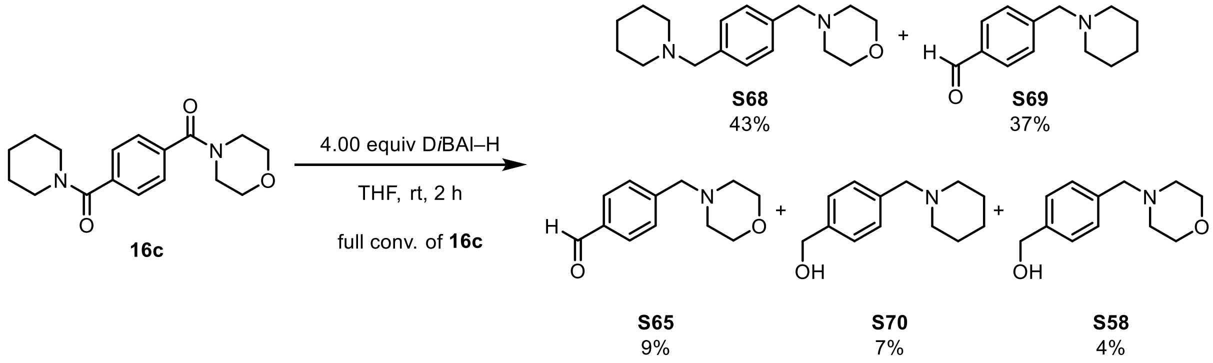

Scheme S29: Reduction of (4-(morpholine-4-carbonyl)phenyl)(piperidin-1-yl)methanone (**16c**) with excess amount of D*i*BAl–H. Conversion and selectivity were determined with GC and GC-MS.

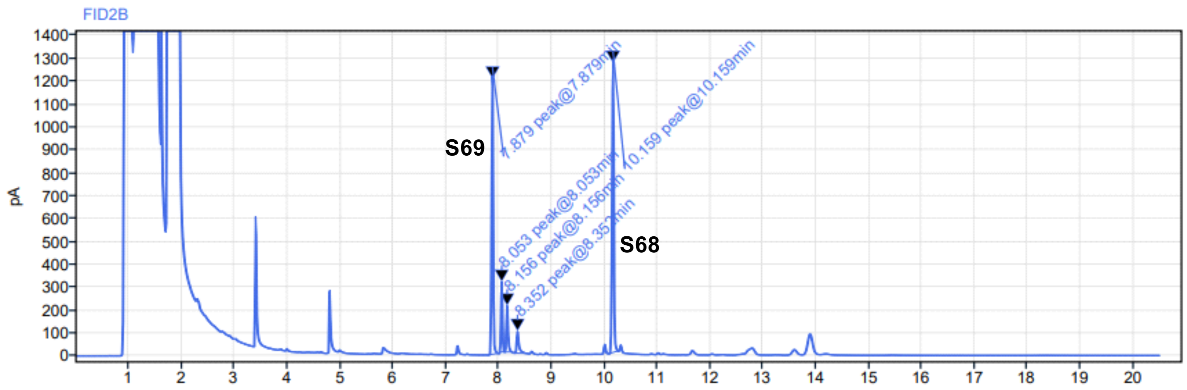

Signal: FID2B

Name	RT [min]	RF	Area	Peak Area Percent
peak@7.879min	7.88		2397.657	37.08
peak@8.053min	8.05		563.829	8.72
peak@8.156min	8.16		443.259	6.86
peak@8.352min	8.35		273.545	4.23
peak@10.159min	10.16		2787.501	43.11

Figure S22: GC analysis of the reaction mixture of (4-(morpholine-4-carbonyl)phenyl)(piperidin-1-yl)methanone (**16c**) with excess amount of D*i*BAl–H (GC method: 40_20_250_10).

4.4.5.2.2 Reduction of (4-(morpholine-4-carbonyl)phenyl)(piperidin-1-yl)methanone (16c) with $LiAlH_4$

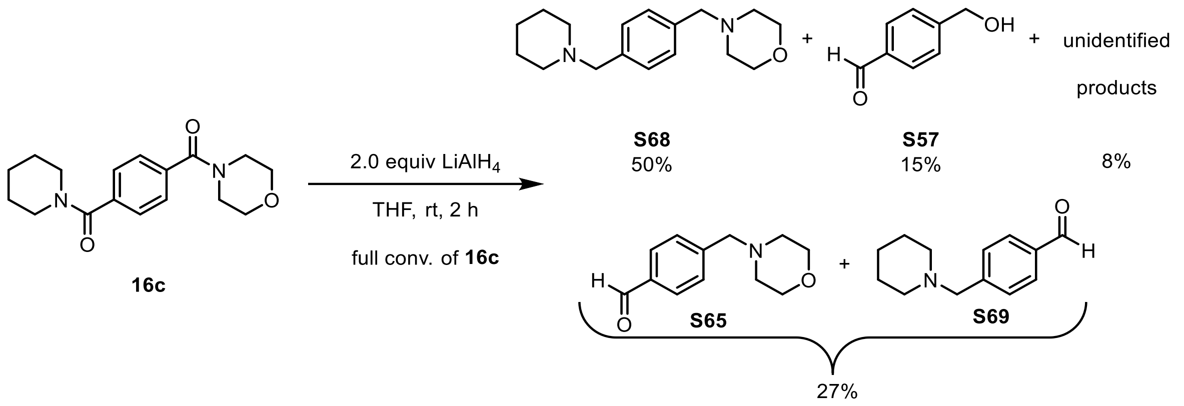

Scheme S30: Reduction of (4-(morpholine-4-carbonyl)phenyl)(piperidin-1-yl)methanone (**16c**) with $LiAlH_4$. Conversion and selectivity were determined with GC, GC-MS and ^{1}H NMR.

Prepared according to **GP5** from (4-(morpholine-4-carbonyl)phenyl)(piperidin-1-yl)methanone (**16c**, 30 mg, 0.10 mmol, 1.0 equiv), $LiAlH_4$ (7.6 mg, 0.20 mmol, 2.00 equiv) in dry THF (1.0 mL). The reaction mixture was stirred for 2 h at rt. Analysis of the crude mixture was carried out by GC, GCMS and/or ^{1}H NMR.

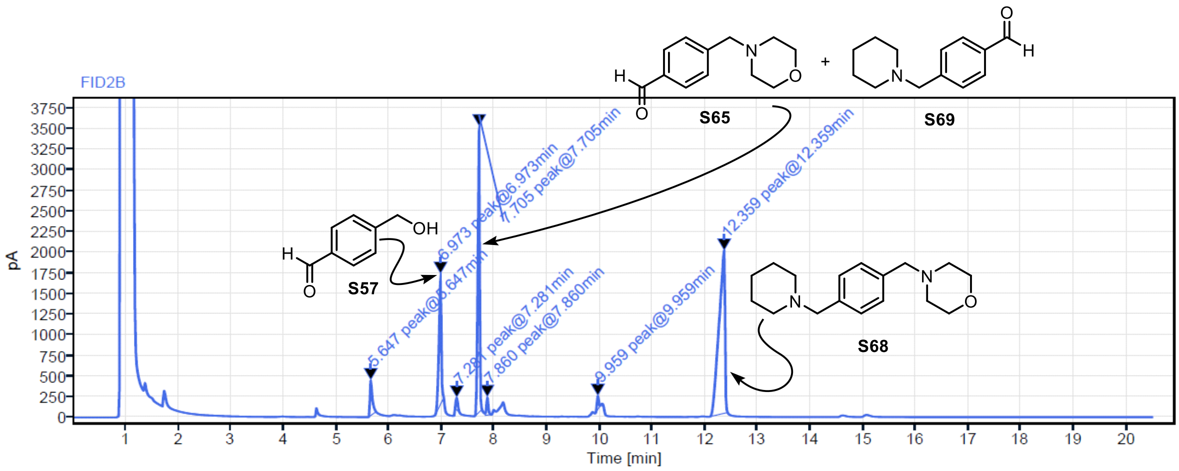

Name	RT [min]	RF	Area	Peak Area Percent
peak@5.647min	5.65		1301.842	4.15
peak@6.973min	6.97		4821.785	15.36
peak@7.281min	7.28		394.507	1.26
peak@7.705min	7.71		8387.948	26.72
peak@7.860min	7.86		511.714	1.63
peak@9.959min	9.96		365.906	1.17

Name	RT [min]	RF	Area	Peak Area Percent
peak@12.359min	12.36		15614.052	49.73

Figure S23: GC analysis of the reaction mixture of (4-(morpholine-4-carbonyl)phenyl)(piperidin-1-yl)methanone (**16c**) with $LiAlH_4$ (GC method: 40_20_250_10).

4.4.6 Reduction of 1-morpholino-2-[2-(piperidine-1-carbonyl)phenyl]ethanone (S94)

4.4.6.1 Cu(I)-catalyzed reduction of 1-morpholino-2-[2-(piperidine-1-carbonyl)phenyl]ethanone (S94) with H_2

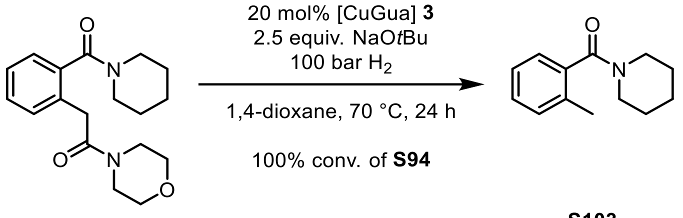

S94

S103
49% isolated yield

Scheme S31: Site-selective Cu(I)-catalyzed reduction of 1-morpholino-2-[2-(piperidine-1-carbonyl)phenyl]ethanone (**S94**) with H_2.

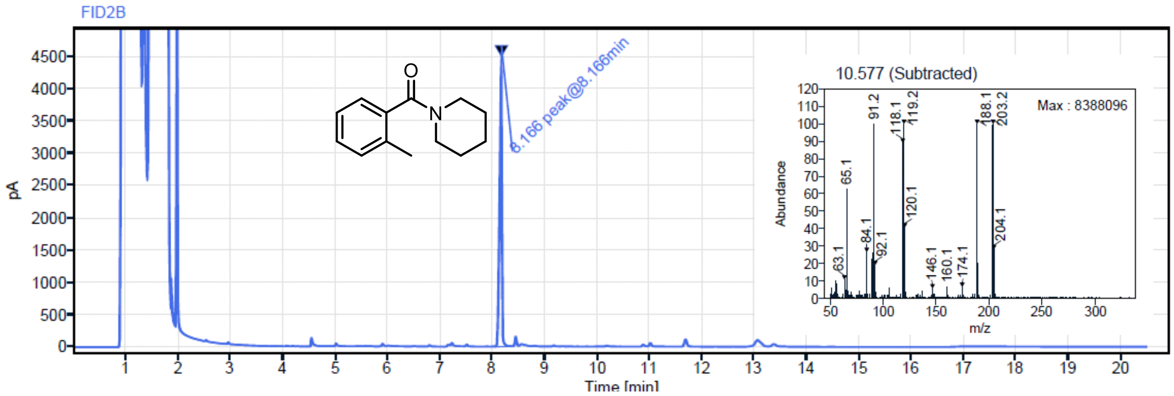

Signal:	FID2B			
Name	RT [min]	RF	Area	Peak Area Percent
peak@8.166min	8.17		12986.621	100.00

Figure S24: GC analysis of the reaction mixture of the Cu(I)-catalyzed reduction of 1-morpholino-2-[2-(piperidine-1-carbonyl)phenyl]ethanone (**S94**) with H_2 (GC method: 40_20_250_10_inlet_300 °C).

From these results we could conclude that, activated alkyl morpholine amides have been decomposed in our catalytic system. We hypothesize that a possible decomposition pathway could be a dehydroxymethylation of the primary alcohol to the corresponding toluene derivative **S94** under highly basic and hydrogenative conditions.[53,54] It should be noted that in absence of H_2 the starting material **S94** remained intact, excluding a base mediated transformation.

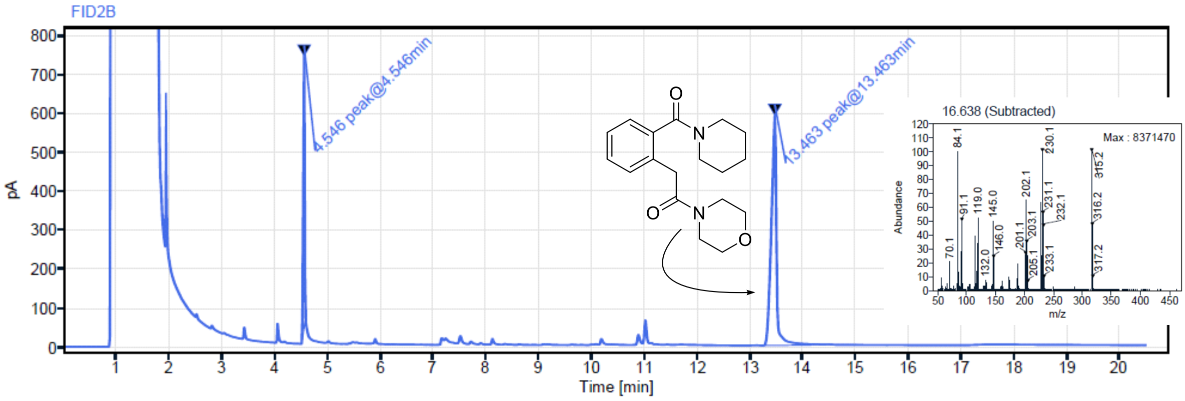

Figure S25: GC analysis of the reaction mixture of the Cu(I)-catalyzed reduction of 1-morpholino-2-[2-(piperidine-1-carbonyl)phenyl]ethanone (**S94**) without H_2 (GC method: 40_20_250_10_inlet_300 °C).

4.4.6.1.1 (2-Methylphenyl)-1-piperidinylmethanone (S103)

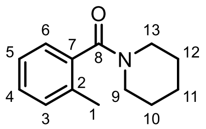

S103

$C_{13}H_{17}NO$

Mw = 203.29 g mol^{-1}

Prepared according to **GP4** from 1-morpholino-2-[2-(piperidine-1-carbonyl)phenyl]ethanone (**S94**, 16 mg, 0.050 mmol, 1.0 equiv), [CuGua] **3** (4.7 mg, 10 µmol, 20 mol%), NaO*t*Bu (12 mg, 0.125 mmol, 2.5 equiv) in 1,4-dioxane (0.50 mL). The reaction mixture was stirred for 24 h at 70 °C. Purification by flash column chromatography on silica gel (cyclohexane/ethyl acetate = 80:20) yielded **S103** as a yellow oil (5.0 mg, 0.0246 mmol, 49%).

R_f = 0.25 (cyclohexane/ethyl acetate = 80:20).

^{1}H NMR (600 MHz, $CDCl_3$): δ =1.45 (s, 2H, H-10)*, 1.66 (s, 4H, H-11, H-10*), 2.31 (s, 3H, H-1), 3.17 (q, $^3J_{9,10}$ = 4.9 Hz, 2H, H-9**), 3.64–3.72 (m, 1H, H-13a)**, 3.78–3.85 (m, 1H, H-13b)**, 7.12–7.17 (m, 1H, H-6), 7.16–7.23 (m, 2H, H-3, H-5***), 7.23–7.26 (m, 1H, H-4***) ppm.

The letters a and b are assigned to axial and equatorial H atoms. There is no correlation between the letters and the position of H atom.

^{13}C NMR (151 MHz, $CDCl_3$): δ = 19.1 (C-1), 24.7 (C-11), 25.9 (C-12)*, 26.7 (C-10)*, 42.5 (C-13)**, 48.0 (C-9)**, 125.8 (C-6), 126.0 (C-5)****, 128.7 (C-4)****, 130.5 (C-3)****, 134.2 (C-7)***, 136.9 (C-2)***, 170.0 (C-8) ppm.

GC-MS (EI) for $C_{13}H_{17}NO^+$ [(M)$^+$], calculated: 203.1, found 203.2.

The data is in accordance with literature.[55]

4.5 Additional substrates for the Cu(I) catalyzed reduction of diamides with H_2 – part I

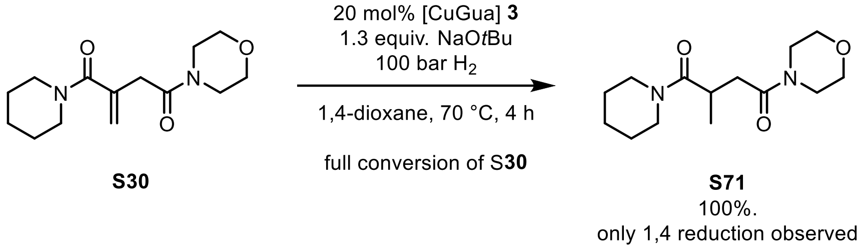

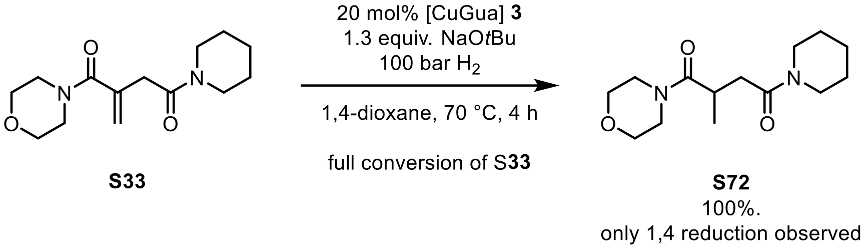

4.5.1 Cu(I)-catalyzed reduction of 7-[4-(4-chlorobenzoyl)piperazin-1-yl]-1-cyclopropyl-6-fluoro-3-(piperidine-1-carbonyl)quinolin-4-one (S102) with H_2

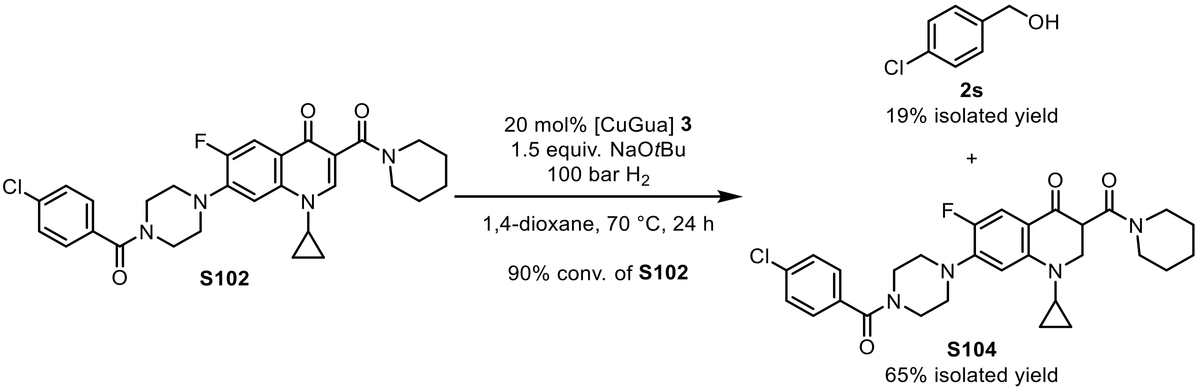

Scheme S32: Site-selective Cu(I)-catalyzed reduction of 7-[4-(4-chlorobenzoyl)piperazin-1-yl]-1-cyclopropyl-6-fluoro-3-(piperidine-1-carbonyl)quinolin-4-one (**S102**) with H_2.

Prepared according to **GP3** from 7-[4-(4-chlorobenzoyl)piperazin-1-yl]-1-cyclopropyl-6-fluoro-3-(piperidine-1-carbonyl)quinolin-4-one (**S102**, 107 mg, 0.20 mmol, 1.0 equiv), [CuGua] **3** (19 mg, 40 µmol, 10 mol%), NaO*t*Bu (29 mg, 0.26 mmol, 1.5 equiv) in 1,4-dioxane (2.0 mL). The reaction mixture was stirred for 4 h at 70 °C. Purification by flash column chromatography on silica gel (cyclohexane/ethyl acetate = 70:30 to CH_2Cl_2/MeOH = 98:2) yielded **2s** as a white solid (5.4 mg, 0.038 mmol, 19%). Product **S104** (70 mg, 0.13 mmol, 65%) was isolated as a yellow solid.

4.5.1.1 (4-Chlorophenyl)methanol (2s) from 7-[4-(4-chlorobenzoyl)piperazin-1-yl]-1-cyclopropyl-6-fluoro-3-(piperidine-1-carbonyl)quinolin-4-one (2s)

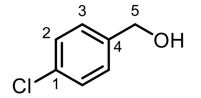

2s
C_7H_7ClO
Mw = 142.58 g mol^{-1}

R*f* = 0.45 (cyclohexane/ethyl acetate = 80:20).

^{1}H NMR (600 MHz, $CDCl_3$): δ = 1.72 (s, 1H, O–*H*), 4.67 (s, 2H, H-5), 7.30 (d, $^3J_{3,2}$ = 8.6 Hz, 2H, H-3), 7.33 (d, $^3J_{2,3}$ = 8.5 Hz, 2H, H-2) ppm.

^{13}C NMR (151 MHz, $CDCl_3$): δ = 64.7 (C-5), 128.4 (C-3), 128.8 (C-2), 133.5 (C-1), 139.3 (C-4) ppm.

The data is in accordance with literature.[48]

4.5.1.2 7-[4-(4-Chlorobenzoyl)piperazin-1-yl]-1-cyclopropyl-6-fluoro-3-(piperidine-1-carbonyl)-2,3-dihydroquinolin-4-one (S104)

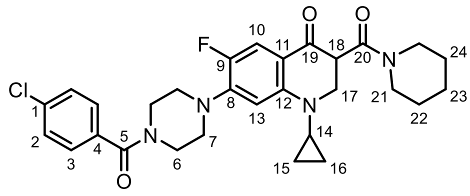

S104
$C_{29}H_{32}ClFN_4O_3$
Mw = 539.05 g mol^{-1}

R*f* = 0.10 (CH_2Cl_2/MeOH = 98:2).

m.p: 77–80 °C.

^{1}H NMR (600 MHz, $CDCl_3$): δ = 0.66–0.80 (m, 2H, H-15a, H-16a)*, 0.81–0.91 (m, 1H, H16b)*, 0.92–1.05 (m, 1H, H-15b)*, 1.51–1.86 (m, 6H, H-22, H-23, H-24), 2.39 (tt, $^3J_{14,15}$ = 6.7, $^3J_{14,16}$ = 3.7 Hz, 1H, H-14), 2.99–4.06 (m, 15H, H-6, H-7, H-17, H-18, H-21), 6.71 (d, $^3J_{13,F}$ = 7.2 Hz, 1H, H-13), 7.34–7.47 (m, 4H, H-2, H-3), 7.53 (d, J = 13.3 Hz, 1H, H-10) ppm.

Traces of cyclohexane could be detected in ^{1}H NMR at 1.42 ppm.

^{13}C NMR (151 MHz, $CDCl_3$): δ = 7.3 (C-16)*, 10.1 (C-15)*, 24.7 (C-22)**, 25.7 (C-23)**, 26.6 (C-24)**, 32.6 (C-14), 43.1 (C-21)***, 47.5 (C-6)***, 50.4 (C-18), 52.7 (C-7, C-17)***, 103.1 (C-13), 113.9 (d, $^3J_{C,F}$ = 6.0 Hz, C-11)******, 114.6 (d, $^2J_{C,F}$ = 22.1 Hz, C-10), 128.9 (C-2)****, 129.0 (C-3)****, 133.8 (C-1)*****, 136.3 (C-4)*****, 145.9 (d, $^2J_{C,F}$ = 9.8 Hz, C-8)******, 148.8 (d, $^1J_{C,F}$ = 239.8 Hz, C-9), 150.9 (C-12), 166.2 (C-20), 169.5 (C-5), 188.0 (C-19) ppm.

Traces of cyclohexane could be detected in ^{13}C NMR at 27.1 ppm.

^{19}F NMR (564 MHz, $CDCl_3$): δ = – 134.8 ppm.

IR (ATR): ṽ = 2925.9 (w), 2855.1 (w), 1632.6 (m), 1435.0 (m), 1401.5 (m), 1244.9 (m), 1151.7 (m), 1006.4 (m) cm^{-1}.

4.6 Additional substrates for the Cu(I) catalyzed reduction of diamides with H_2 – part II

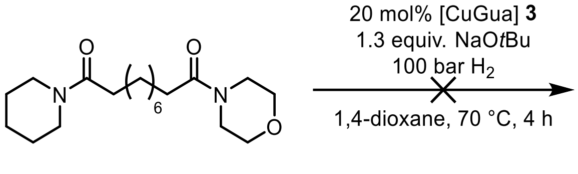

S35

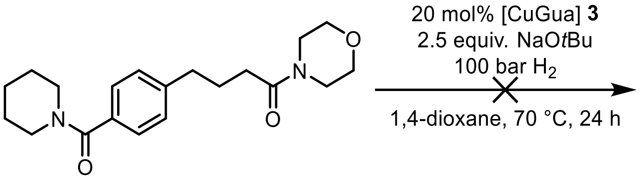

S97

Scheme S33: Unsuccessful substrates for the Cu(I) catalyzed reduction of diamides with H_2.

4.7 Gram-scale reaction

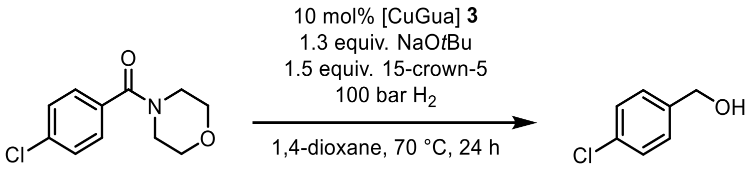

1s 2s

(4-Chlorophenyl)methanol (2s)

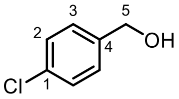

2s

C_7H_7ClO

Mw = 142.58 g mol^{-1}

Prepared according to **GP3** from (4-chlorophenyl)(morpholino) methanone (**1s,** 1.0 g, 4.4 mmol, 1.0 equiv), [CuGua] **3** (0.21 g, 0.44 mol, 10 mol%), NaO*t*Bu (0.55 g, 5.8 mmol, 1.3 equiv) and 15-crown-5 (1.3 mL, 6.6 mmol, 1.5 equiv) in 1,4-dioxane (40 mL). The reaction mixture was stirred for 4 h at 70 °C. Purification by flash column chromatography on silica gel (cyclohexane/ethyl acetate = 90:10) yielded **2s** as a white solid (450 mg, 3.16 mmol, 72%).

$\mathbf{R}_f$ = 0.45 (cyclohexane/ethyl acetate = 80:20).
^{1}H NMR (600 MHz, $CDCl_3$): δ = 2.40 (s, 1H, O–*H*), 4.67 (s, 2H, H-5), 7.32 (d, $^3J_{3,2}$ = 8.2 Hz, 2H, H-3), 7.37 (d, $^3J_{2,3}$ = 7.5 Hz, 2H, H-2) ppm.
^{13}C NMR (151 MHz, $CDCl_3$): δ = 64.5 (C-5), 128.4 (C-3), 128.7 (C-2), 133.4 (C-1), 139.3 (C-4) ppm.
HRMS (APCI) for $C_7H_6{}^{35}Cl^+$ [$(M–OH)^+$] calculated: 125.0153, found 125.0151.
The data is in accordance with literature.[48]

4.8 Isotope labelling experiments

Deuteration experiments were carried out following **GP3** using D_2 (99.8 %D) instead of H_2. Deuterium incorporation was determined *via* quantitative ^{1}H NMR and comparison of two selected ^{1}H NMR signals (relaxation delay, d_1 = 40s), and selected ^{1}H NMR and ^{2}H NMR signals with the respective substrates).

4.8.1 *p*-Tolylmethan-*d*$_2$-ol (*d*$_2$-2m)

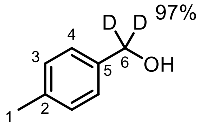

***d*$_2$-2m**
$C_8H_8D_2O$
Mw = 124.18 g mol^{-1}

Prepared according to **GP3** from morpholino(*p*-tolyl)methanone (**1m,** 81 mg, 0.40 mmol, 1.0 equiv), [CuGua] **3** (19 mg, 40 µmol, 10 mol%), NaO*t*Bu (50 mg, 0.52 mmol, 1.3 equiv) and 15-crown-5 (0.12 mL, 0.60 mmol, 1.5 equiv) in 1,4-dioxane (4.0 mL). The reaction mixture was stirred for 24 h at 70 °C with 55 bar D_2. Purification by flash column chromatography on silica gel (*n*-pentane/ethyl acetate = 80:20) yielded ***d*$_2$-2m** as an off white solid (77% conversion of **1m**, 23 mg, 0.19 mmol, 47%).

R_f = 0.20 (*n*-pentane /ethyl acetate = 80:20).
^{1}H NMR (600 MHz, $CDCl_3$): δ = 1.51 (s, 1H, O–*H*), 2.35 (s, 3H, H-1), 4.64 (s, 0.07H, H-6), 7.18 (d, $^3J_{3,4}$ = 7.8 Hz, 2H, H-3), 7.24–7.33 (m, 2H, H-4) ppm.
^{2}H NMR (92 MHz, CH_2Cl_2): δ = 4.59 (s, D-6) ppm.
^{13}C NMR (151 MHz, $CDCl_3$): δ = 21.3 (C-1), 64.8 (C-6), 127.3 (C-4), 129.4 (C-3), 137.6 (C-2), 137.9 (C-5) ppm.
HRMS (APCI) for $C_8H_7D_2^+$ [(M-OH)$^+$] calculated: 107.0824, found 107.0819.
The deuterium incorporation was determined by comparing the integrals of the corresponding ^{1}H NMR signal H-6 (δ = 4.64 ppm) with H-3 (δ = 7.18 ppm) [^{1}H NMR (500 MHz, $CDCl_3$), d_1 = 40 s].
The data is in accordance with literature.[56]

4.8.2 Cyclohexylmethan-d_3-ol (d_3-2n)

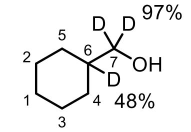

***d*₃-2n**

$C_7H_{11}D_3O$

Mw = 117.21 g mol^{-1}

Prepared according to **GP3** from cyclohexyl(morpholino)methanone (**1n,** 79 mg, 0.40 mmol, 1.0 equiv), [CuGua] **3** (19 mg, 40 µmol, 10 mol%), NaO*t*Bu (50 mg, 0.52 mmol, 1.3 equiv) and 15-crown-5 (0.12 mL, 0.60 mmol, 1.5 equiv) in 1,4-dioxane (4.0 mL). The reaction mixture was stirred for 24 h at 70 °C with 20 bar D_2. Purification by flash column chromatography on silica gel (cyclohexane/ethyl acetate = 90:10) yielded ***d*₃-2n** as a pale yellow oil (55% conversion of **1n**, 9.0 mg, 0.08 mmol, 20%).

R*f* = 0.35 (cyclohexane/ethyl acetate = 90:10).

^{1}H NMR (600 MHz, $CDCl_3$): δ = 0.89–1.01 (m, 2H, H-3)*, 1.10–1.38 (m, 4H, H-1*, H-2*, O–*H*), 1.41–1.53 (m, 0.52H, H-6), 1.63–1.80 (m, 5H, H-1', H-2', H-3')*, 3.39–3.44 (m, 0.07H, H-7) ppm.

^{2}H NMR (92 MHz, CH_2Cl_2): δ = 1.38 (s, D-4), 3.32 (s, D-5) ppm.

^{13}C NMR (151 MHz, $CDCl_3$): δ = 26.0 (C-1)*, 26.8 (C-2)*, 29.6 (C-3)*, 29.7 (C-4)*, 29.9 (C-5)*, 40.5 (C-6), 68.1 (C-7) ppm.

HRMS (APCI) for $C_7H_{10}D_3$ [$(M+H)^+$] calculated: 107.1200, found 107.1197. Mol peak with very low intensity (baseline noise) was detected. This compound may be unstable in the source at the measurement's conditions and/or difficult ionizable.

The deuterium incorporation was determined by comparing the integrals of the corresponding ^{1}H NMR signal H-3 (δ = 0.89–1.01 ppm) with H-4 (δ = 1.41–1.53 ppm) and H-5 (δ = 3.32 ppm) [^{1}H NMR (500 MHz, $CDCl_3$), d_1 = 40 s].

The data is in accordance with literature.[43]

4.9 Cu(I) catalyzed reduction of amides with H_2 – Mass balance

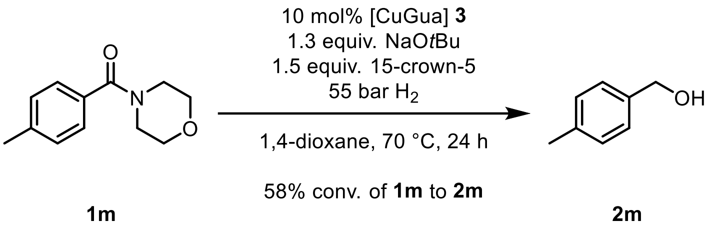

mass before catalysis: 70.7 mg (66 mg without catalyst 3)
mass after catalysis: 64 mg (after filtration)

This reaction was conducted according to **GP3** from morpholino(*p*-tolyl)methanone (**1m,** 21 mg, 0.10 mmol, 1.0 equiv), [CuGua] **3** (4.7 mg, 10 µmol, 10 mol%), NaO*t*Bu (12 mg, 0.13 mmol, 1.3 equiv) and 15-crown-5 (0.030 mL, 0.15 mmol, 1.5 equiv) in 1,4-dioxane (1.0 mL). The reaction mixture was stirred for 24 h at 70 °C. The crude reaction mixture is filtered over a plug of silica (eluent: CH_2Cl_2/MeOH, 50:1, 1 x 5 cm, 5 mL) and all volatiles were removed under reduced pressure.

The mass of the crude reaction mixture was found to be m = 64 mg, a result which shows 91% of mass recovery in this catalytic transformation.

4.10 Linker variation and reactivity in Cu(I)-catalyzed reduction of amides with H_2

4.10.1 Synthesis of benzene linker based Cu-NHC/Guanidine bifunctional catalyst 6

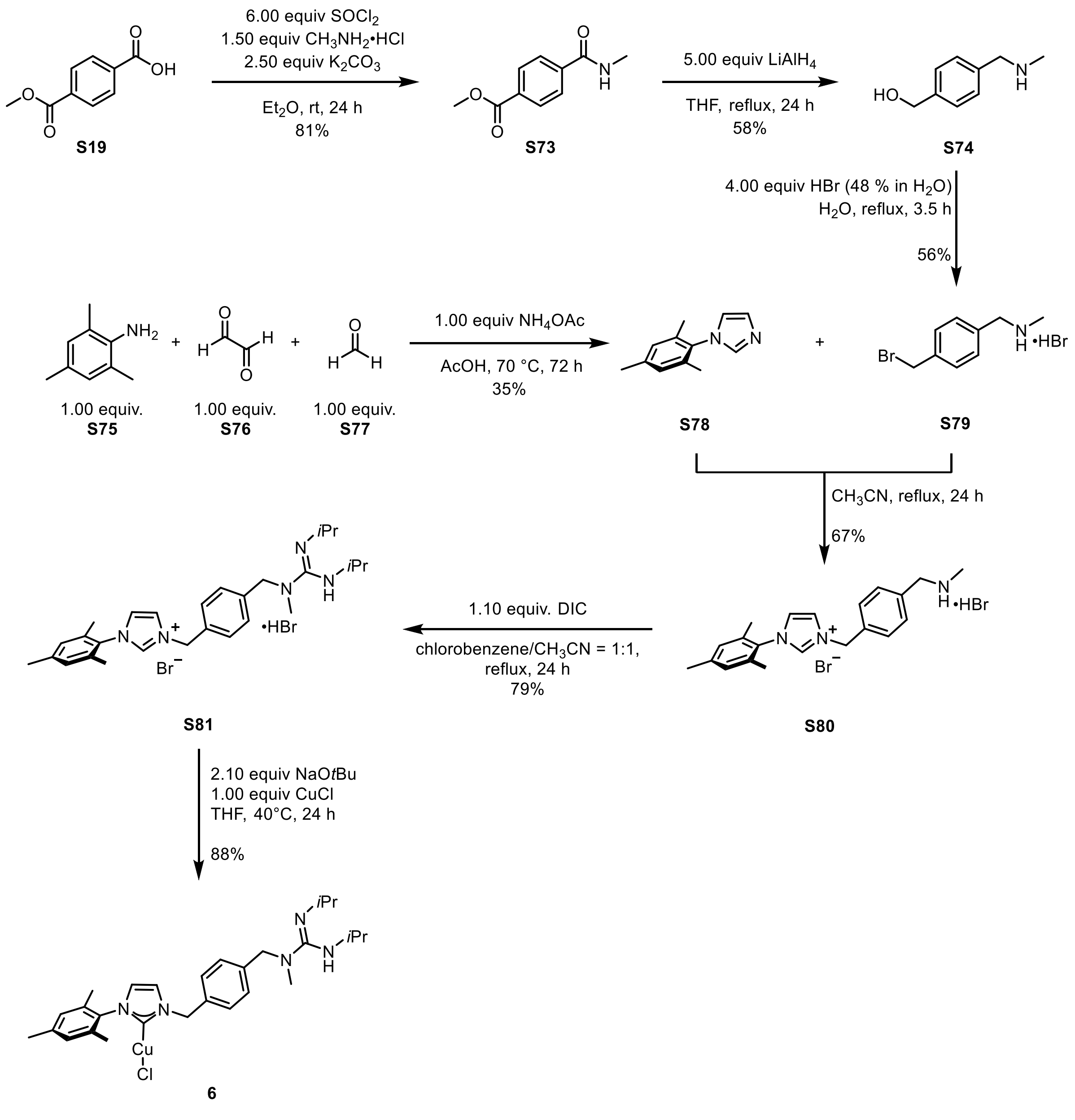

Scheme S34: Synthesis of benzene linker based Cu-NHC/Guanididne bifunctional catalyst **6**. (DIC stands for diisopropylcarbodiimide)

4.10.1.1 Methyl 4-(methylcarbamoyl)benzoate (S73)

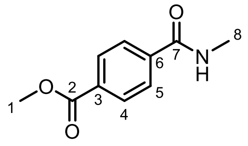

S73
$C_{10}H_{11}NO_3$
Mw = 193.20 g mol^{-1}

Following a literature procedure,[57] in a 50 mL Schenk flask charged with a magnetic stirring bar, a mixture of 4-methoxycarbonylbenzoic acid (**S19**, 2.79 g, 15.5 mmol, 1.00 equiv), thionyl dichloride (11.1 g, 6.82 mL, 92.9 mmol, 6.00 equiv) and DMF (2 drops) was heated to 80 °C and stirred for 1 h. The excess of $SOCl_2$ was removed under reduced pressure, and the crude acid chloride was dissolved in anhydrous Et_2O (15 mL). K_2CO_3 (5.35 g, 38.7 mmol, 2.50 equiv) was added followed by methylamine hydrochloride (1.57 g, 23.2 mmol, 1.50 equiv). The reaction mixture was stirred at room temperature for 16 h, then diluted with EtOAc (50 mL). The reaction mixture was filtered through a glass frit (P4) and washed with EtOAc (10 mL). The filtrate was concentrated under reduced pressure yielding **S73** (2.42 g, 12.5 mmol, 81%) as white solid, which was used without any additional purification to the next step.

m.p. : 131 °C.
R*f* = 0.25 (cyclohexane/EtOAc = 2:1).
^{1}H NMR (600 MHz, $CDCl_3$): δ = 3.02 (s, 3H, H-1), 3.91 (d, $^3J_{8,\,N\text{–}H}$ = 4.8 Hz, 3H, H-8), 6.30 (br s, 1H, N–*H*), 7.81 (d, $^3J_{4,5}$ = 8.4 Hz, 2H, H-4), 8.07 (d, $^3J_{5,4}$ = 8.4 Hz, 2H, H-5) ppm.
^{13}C NMR (151 MHz, $CDCl_3$): δ = 27.0 (C-1), 52.4 (C-8), 126.9 (C-4), 129.8 (C-5), 132.2 (C-3), 138.5 (C-6), 166.3 (C-2), 167.4 (C-7) ppm.
IR (ATR): ṽ = 3366 (m), 3045 (w), 2998 (w), 2948 (w), 2840 (w), 2806 (w), 1640 (s), 1543, 1435 (s), 1278 (s), 1189 (s), 1114 (s), 875 (s), 816 (s), 726 (s), 689 (s) cm^{-1}.
HMRS (APCI) for $C_{10}H_{12}NO_3^+$ [(M+H$^+$)$^+$] calculated: 194.0812, found: 194.0819.

4.10.1.2 (4-((Methylamino)methyl)phenyl)methanol (S74)

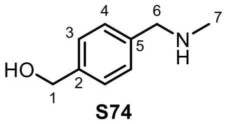

S74
$C_9H_{13}NO$
Mw = 151.21 g mol^{-1}

In deference to a literature procedure,[15] In a 250 mL Schlenk flask charged with a magnetic stirring bar, $LiAlH_4$ (4.16 g, 110 mmol, 5.00 equiv) was added in THF (20 mL) at 0 °C. To this reaction mixture, a solution of methyl 4-(methylcarbamoyl)benzoate (**S73**, 4.24 g, 21.9 mmol, 1.00 equiv) in THF (50 mL) was added dropwise at 0 °C over a period of 30 min. The reaction mixture was stirred at reflux for 24 h. After cooling down to 0 °C, the mixture was quenched with sat. aq. NH_4Cl (5 mL) and diluted with EtOAc (100 mL). The mixture was then filtered over a pad of Celite (4 × 3 cm) The filtrate was concentrated under reduced pressure to afford product **S74** (1.92 g, 12.7 mmol, 58%) as colorless oil, which was used without any additional purification to the next step.

^{1}H NMR (600 MHz, $CDCl_3$): δ = 2.42 (s, 3H, H-7), 3.72 (s, 2H, H-6), 4.65 (s, 2H, H-1), 7.29 (m, 4H, H-3, H-4) ppm.

^{13}C NMR (151 MHz, $CDCl_3$): δ = 36.0 (C-7), 55.8 (C-6), 65.1 (C-1), 127.2 (C-3)*, 128.5 (C-4)*, 139.2 (C-5), 140.0 (C-2) ppm.

IR (ATR): ṽ = 3362 (br), 3216 (br), 3030 (m), 2851 (m), 1714 (w), 1550 (s), 1408 (s), 1334 1289 (s), 1010 (s), 820 (s), 730 (s), 689 (s) cm^{-1}.

HMRS (APCI) for $C_9H_{14}NO^+$ [(M+H$^+$)] calculated: 151.1070, found: 151.1073.

4.10.1.3 1-(4-(Bromomethyl)phenyl)-*N*-methylmethanamine hydrobromide (S79)

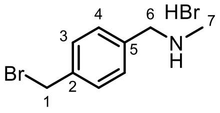

S79

$C_9H_{13}Br_2N$

Mw = 295.02 g ml^{-1}

In deference to a literature procedure,[58] In a 25 mL-round bottom flask, aq. HBr (48% w/w in H_2O, 3.50 mL, 13.2 mmol, 2.00 equiv) was cooled to 0 °C. 4-((Methylamino)methyl)phenyl)methanol (**S74**, 1.00 g, 6.61 mmol, 1.00 equiv) was added over 30 min at 0 °C. The reaction mixture was stirred under reflux for 3.5 h. Residual H_2O and unreacted HBr were distilled off. After cooling down to room temperature, EtOAc (15 mL) was added, the precipitate was collected on a glass frit (P4) and washed with EtOAc (3 x 25 mL). The solid was dried under reduced pressure. The product **S79** (1.10 g, 3.73 mmol, 56%) was obtained as a brown solid.

m.p.: >300 °C (decomp.).

^{1}H NMR (600 MHz, DMSO-d_6): δ = 2.56 (t, $^3J_{7,N\text{–}H}$ = 5.4 Hz, 3H, H-7), 4.12 (t, $^3J_{6,N\text{–}H}$ = 6.0 Hz, 2H, H-6), 4.72 (s, 2H, H-1), 7.47 (d, $^3J_{3,4}$ = 8.2 Hz, 2H, H-3), 7.52 (d, $^3J_{4,3}$ = 8.2 Hz, 2H, H-4), 8.75 (brs, 2H, N–*H*a, *H*b) ppm.

^{13}C NMR (151 MHz, DMSO-d_6): δ = 32.2 (C-7), 33.8 (C-6), 50.9 (C-1), 129.6 (C-3)*, 130.2 (C-4)*, 132.0 (C-5), 139.0 (C-2) ppm.

IR (ATR): ṽ = 3023 (w), 2981 (m), 2929 (s), 2791 (s), 2702 (s), 2545 (m), 2396 (m), 1513 (w), 1476 (m), 1420 (s), 1230 (s), 1121 (w), 1013 (s), 969 (s), 928 (s), 853 (s), 767 (s),726 (m) cm^{-1}.

HMRS (APCI) for $C_9H_{13}N^{79}Br$ [(M-H^{79}Br+H$^+$)]$^+$ calculated: 214.0226 found: 214.0228.

4.10.1.4 1-Mesityl-3-(4-((methylamino)methyl)benzyl)-1*H*-imidazol-3-ium bromide hydrobromide (S80)

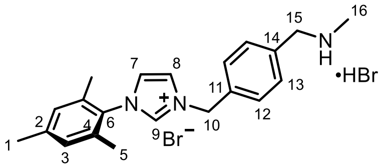

S80

$C_{21}H_{27}Br_2N_3$
Mw = 481.28

In deference to a literature procedure,[3] In a 10 mL pressure tube, 1-(2, 4, 6-trimethylphenyl)imidazole (**S78**, 575 mg, 3.08 mmol, 1.30 equiv) and 1-(4-(bromomethyl)phenyl)-*N*-methylmethanamine hydrobromide (**S79**, 700 mg, 2.37 mmol, 1.00 equiv) were dissolved in CH_3CN (3 mL) and the reaction mixture was stirred at reflux for 48 h. After cooling the reaction mixture to room temperature, all volatiles were removed under reduced pressure. The resulting solid was washed with EtOAc (3 x 20 mL) and dried under reduced pressure. The product **S80** (760 mg, 1.58 mmol, 67%) was obtained as a light-yellow solid.

m.p.: 246.2 °C.

^{1}H NMR (600 MHz, DMSO-*d*$_6$): δ = 2.01 (s, 6H, H-5), 2.33 (s, 3H, H-1), 2.56 (t, $^3J_{16,N–H}$ = 5.0 Hz, 3H, H-16), 4.16 (t, $^3J_{15,N–H}$ = 5.5 Hz, 2H, H-15), 5.58 (s, 2H, H-10), 7.15 (s, 2H, H-3), 7.54 (d, $^3J_{12,13}$ = 8.2 Hz, 2H, H-12), 7.58 (d, $^3J_{13,12}$ = 8.2 Hz, 2H, H-13), 7.96 (*app* t, *J* = 1.7 Hz, 1H, H-7)*, 8.09 (*app* t, *J* = 1.7 Hz, 1H, H-8)*, 8.87 (brs, 2H, N–*H*a, Hb), 9.68 (s, 1H, H-9) ppm.

^{13}C NMR (151 MHz, DMSO-*d*$_6$): δ = 16.9 (C-5), 20.6 (C-1), 32.1 (C-16), 50.8 (C-15), 51.9 (C-10), 123.2 (C-8)*, 124.4 (C-7)*, 128.4 (C-12)**, 129.3 (C-3), 130.6 (C-13)**, 131.1 (C-14), 132.6 (C-6), 134.2 (C-4), 135.5 (C-11), 137.7 (C-9), 140.3 (C-2) ppm.

IR (ATR): ṽ = 3302 (br), 2952 (br), 2873 (w), 1736 (s), 1643 (s), 1550 (s), 1438 (s), 1367 (m), 1256 (s), 1200 (s), 1159 (s), 745 (w) cm^{-1}.

HMRS (ESI) for $C_{21}H_{26}N_3^+$ [(M–H^{79}Br–^{79}Br$^-$)]$^+$: calculated: 321.2121, found: 321.2115.

4.10.1.5 (*E*)-3-(4-((2,3-Diisopropyl-1-methylguanidino)methyl)benzyl)-1-mesityl-1*H*-imidazol-3-ium bromide hydrobromide (S81)

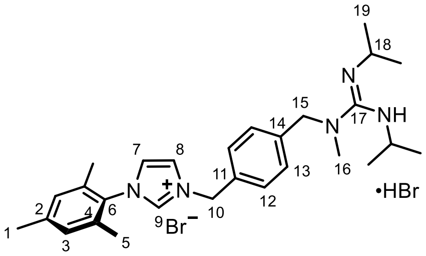

S81
$C_{28}H_{41}Br_2N_5$
Mw = 607.48

In deference to a literature procedure,[3] In a 25 mL 3-necked flask with a reflux condenser, 1-mesityl-3-(4-((methylamino)methyl)benzyl)-1*H*-imidazol-3-ium bromide hydrobromide (**S80**, 1.10 g, 2.29 mmol, 1.00 equiv) and *N*,*N*'-diisopropylcarbodiimide (217 mg, 394 µL, 1.59 mmol, 1.10 equiv) were dissolved in a mixture of chlorobenzene and acetonitrile (10 mL, 1:1) and heated at 120 °C for 18 h. After cooling the reaction mixture to room temperature, all volatiles were removed under reduced pressure. The resulting solid was washed with EtOAc (3 x 20 mL) and dried under reduced pressure. The product **S81** (1.10 g, 1.81 mmol, 79%) was obtained as a light-yellow solid.

m.p.: >300 °C (decomp.).

^{1}H NMR (600 MHz, DMSO-*d*$_6$): δ = 1.13 (d, $^3J_{19,18}$ = 6.4 Hz, 12H, H-19), 1.99 (s, 6H, H-5), 2.33 (s, 3H, H-1), 2.90 (s, 3H, H-16), 3.69 (m, 2H, H-18), 4.54 (s, 2H, H-15), 5.56 (s, 2H, H-10), 7.15 (s, 2H, H-3), 7.36 (d, $^3J_{12,13}$ = 8.1 Hz, 2H, H-12), 7.48 (br s, 2H, N-*H*$_2$), 7.52 (d, $^3J_{13,12}$ = 8.1 Hz, 2H, H-13), 7.95 (s, 1H, H-7), 8.07 (s, 1H, H-8), 9.68 (s, 1H, H-9) ppm.

^{13}C NMR (151 MHz, DMSO-*d*$_6$): δ = 16.8 (C-5), 20.7 (C-1), 22.7 (C-19), 38.4 (C-16), 46.8 (C-18), 52.0 (C-10), 54.1 (C-15), 123.2 (C-8), 124.4 (C-7), 128.4 (C-12), 128.6 (C-13), 129.3 (C-3), 130.3 (C-6), 131.1 (C-14), 134.2 (C-4), 134.4 (C-11), 137.6 (C-9), 140.3 (C-2), 157.7 (C-17) ppm.

HMRS (ESI) for $C_{28}H_{40}N_5^+$ [(M–H^{79}Br–^{79}Br$^-$)]$^+$ calculated: 446.3278, found: 446.3277.

4.10.1.6 (*E*)-(1-(4-((2,3-Diisopropyl-1-methylguanidino)methyl)benzyl)-3-mesityl-1,3-dihydro-2*H*-imidazol-2-ylidene)copper(I) chloride (6)

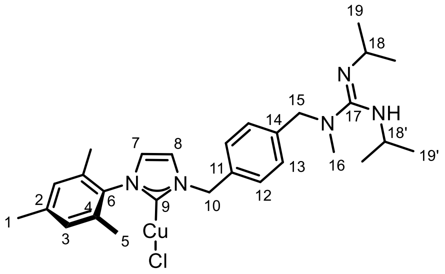

6

$C_{28}H_{39}ClCuN_5$

Mw = 544.66 g mol^{-1}

In deference to a literature procedure,[59]] In a Schlenk flask charged with a magnetic stirring bar (*E*)-3-(4-((2, 3 diisopropyl-1-methylguanidino)methyl)benzyl)-1-mesityl-1*H*-imidazol-3-ium bromide hydrobromide (**S81**, 100 mg, 0.164 mmol, 1.00 equiv), CuCl (16.3 mg, 0.164 mmol, 1.00 equiv), NaO*t*Bu (30.2 mg, 0.34 mmol, 2.10 equiv) were dissolved in THF (2 mL). The reaction mixture was stirred for 3 h at 40 °C. The resulting yellow mixture was then filtered under N_2 atmosphere with a Schlenk frit (P4). The filtrate was dried under reduced pressure, yielding copper(I) complex **6** as pale-yellow solid (85.0 mg, 0.14 mmol, 88%).

^{1}H NMR (600 MHz, $CDCl_3$): δ = 1.14 (m, 12H, H-19, H-19'), 1.98 (s, 6H, H-5), 2.02 (s, 1H, N–*H*), 2.15 (s, 3H, H-1), 2.63 (s, 3H, H-16), 3.36 (br s, 1H, H-18), 3.52 (m, 1H, H-18'), 4.31 (s, 2H, H-15), 5.00 (s, 2H, H-10), 6.68 (s, 1H, H-7)*, 6.73 (s, 2H, H-3), 6.76 (s, 1H, H-8)*, 7.04 (d, $^3J_{12,13}$ = 8.1 Hz, 2H, H-12), 7.22 (d, $^3J_{13,12}$ = 7.8 Hz, 2H, H-13) ppm. The compound contains impurities from DIC at 1.11 ppm (^{1}H NMR).

^{13}C NMR (151 MHz, $CDCl_3$): δ = 18.0 (C-5), 21.2 (C-1), 23.9 (C-19)***, 25.2 (C-19')***, 37.1 (C-16), 46.2 (C-18)**, 47.7 (C-18')**, 54.3 (C-10), 54.8 (C-15), 121.2 (C-8)*, 128.0 (C-12)*, 128.2 (C-13)*, 129.0 (C-7)*, 129.2 (C-3), 129.8 (C-4), 134.8 (C-6), 135.5 (C-11), 138.4 (C-9), 139.5 (C-2), 155.8 (C-17) ppm.

HMRS (APCI) for $C_{28}H_{40}{}^{35}ClCuN_5^+$ [M+H$^+$]: calculated: 544.2263, found: 544.2262.

4.10.2 Cu(I)-catalyzed reduction of morpholino(phenyl)methanone (1j) with Cu(I)-complex 6 and H_2

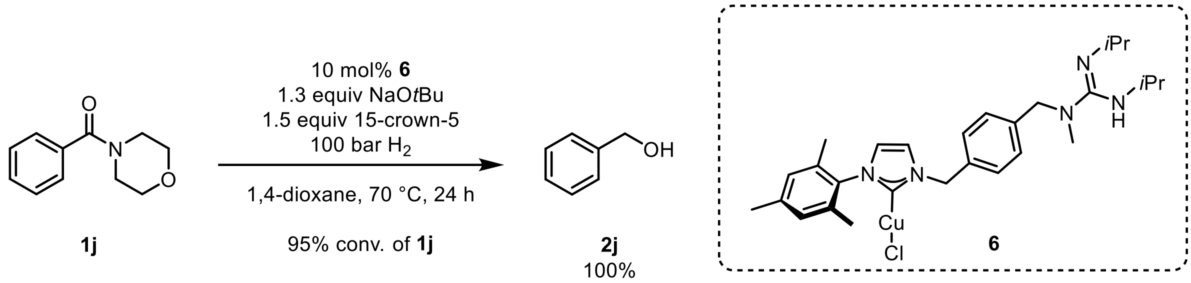

Scheme S35: Cu(I)-catalyzed reduction of morpholino(phenyl)methanone (**1j**) with Cu(I)-complex **6** and H_2.

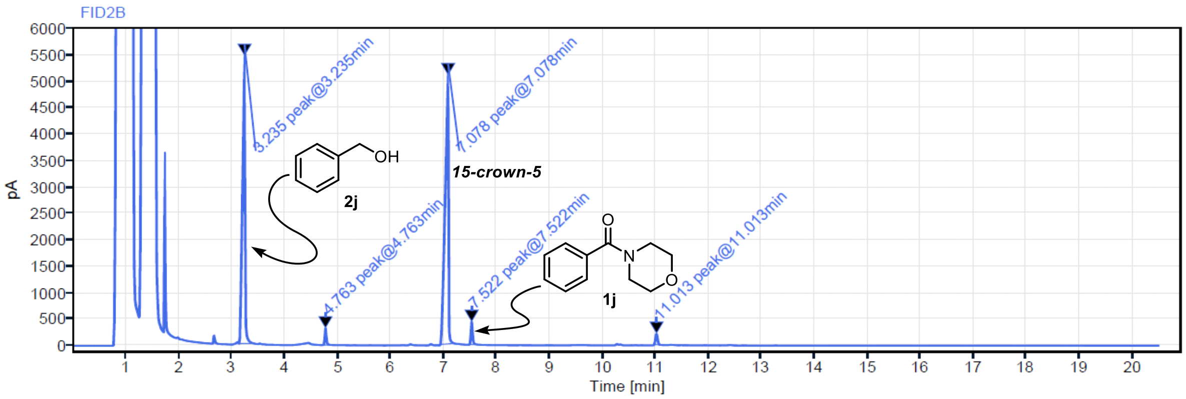

Name	RT [min]	RF	Area	Peak Area Percent
peak@3.235min	3.24		17922.785	42.93
peak@4.763min	4.76		609.871	1.46
peak@7.078min	7.08		21768.028	52.14
peak@7.522min	7.52		898.481	2.15
peak@11.013min	11.01		550.486	1.32

Figure S26: GC analysis of the reaction mixture of the Cu(I)-catalyzed reduction of morpholino(phenyl)methanone (**1j**) with Cu(I)-complex **6** and H_2.

4.10.3 Synthesis of 6-carbon chain linker based Cu-NHC/guanidine bifunctional catalyst 7

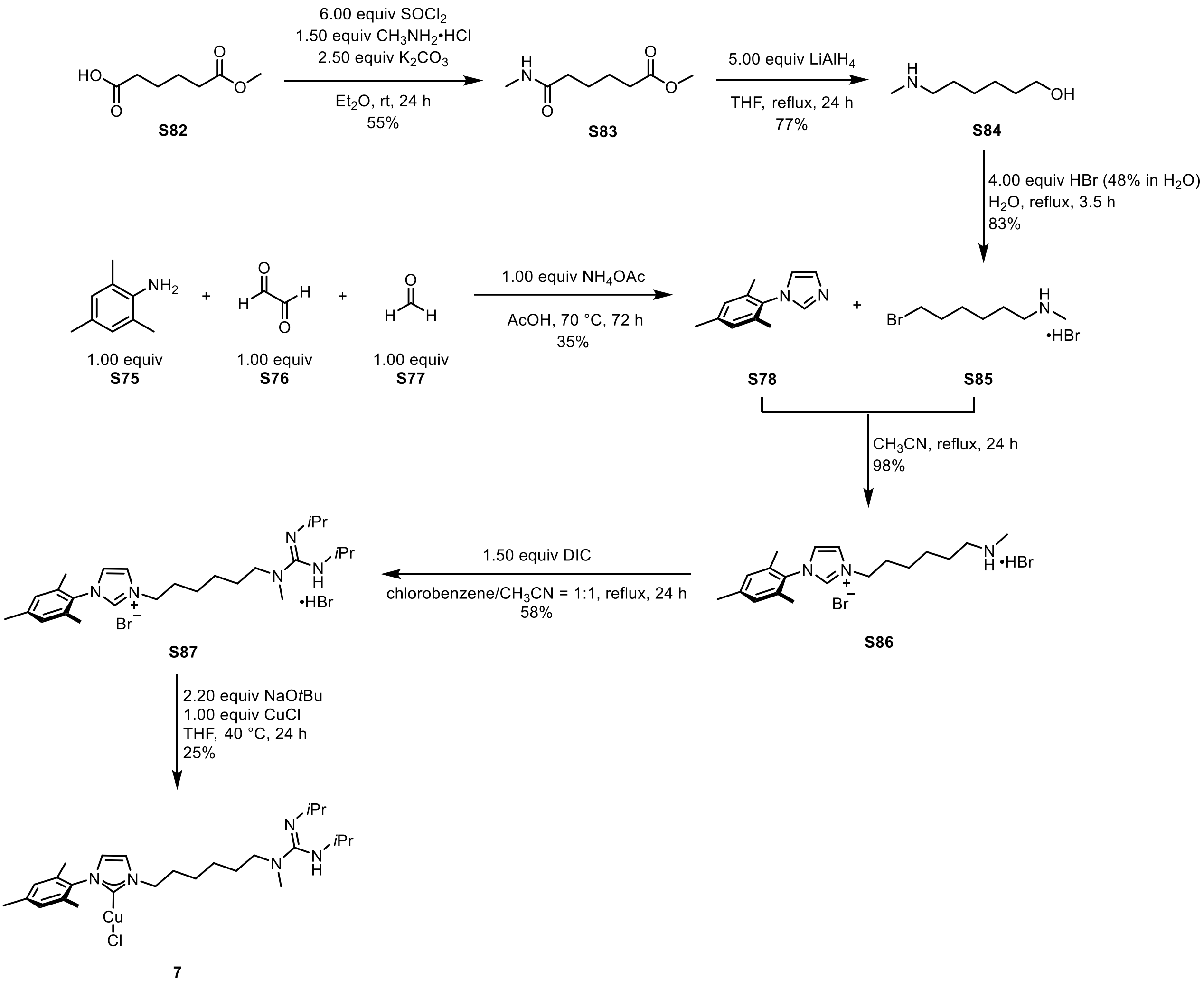

Scheme S35: Synthesis of 6-carbon chain linker based Cu-NHC/guanidine bifunctional catalyst **7** (DIC stands for diisopropylcarbodiimide).

4.10.3.1 Methyl 6-(methylamino)-6-oxohexanoate (S83)

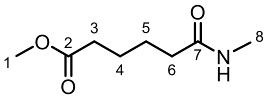

S83
$C_8H_{15}NO_3$
Mw = 173.21 g mol^{-1}

Following a literature procedure,[57] In a 50 mL Schlenk flask charged with a magnetic stirring bar, a mixture of 6-methoxy-6-oxohexanoic acid (**S82**, 4.00 g, 3.70 mL, 25.0 mmol, 1.00 equiv) and $SOCl_2$ (13.8 mL, 187 mmol, 7.50 equiv) was heated to 85 °C and stirred for 2 h. The excess $SOCl_2$ was removed under reduced pressure and the crude acid chloride was dissolved in THF (75 mL). K_2CO_3 (10.4 g, 74.9 mmol, 3.00 equiv) and methyl amine hydrochloride (2.04 g, 30.0 mmol, 1.20 equiv) were added to the reaction mixture and stirred at room temperature for 24 h. After full conversion of **S82** (monitored *via* TLC) the reaction mixture was diluted with EtOAc (20 mL) and filtered with a frit (P4). The filtrate was concentrated under reduced pressure, yielding product **S83** (2.40 g, 13.9 mmol, 55%) as colourless oil, which was used for the next step without further purification.

^{1}H NMR (600 MHz, $CDCl_3$): δ = 1.66 (m, 4H, H-4, H-5), 2.19 (t, $^3J_{6,5}$ = 6.9 Hz, 2H, H-6), 2.33 (t, $^3J_{3,4}$ = 5.6 Hz, 2H, H-3), 2.80 (d, $^3J_{8,N\text{–}H}$ = 4.9 Hz, 3H, H-8), 3.66 (s, 3H, H-1), 5.57 (brs, 1H, N–*H*) ppm.
^{13}C NMR (151 MHz, $CDCl_3$): δ = 24.5 (C-4), 25.1 (C-5), 26.3 (C-8), 32.6 (C-6), 33.7 (C-3), 51.7 (C-1), 173.3 (C-7), 174.1 (C-2) ppm.
IR (ATR): ṽ = 3302 (br), 2952 (br), 2873 (w), 1736 (s), 1643 (s), 1550 (s), 1438 (s), 1367 (m), 1256 (s), 1200 (s), 1159 (s) cm^{-1}.
HRMS (APCI) for $C_8H_{16}NO_3^+$ [(M+H$^+$)]: calculated: 174.1125, found: 174.1127.

4.10.3.2 6-(Methylamino)hexan-1-ol (S84)

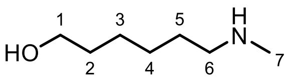

S84
$C_7H_{17}NO$
Mw = 131.21 g mol^{-1}

In deference to a literature procedure,[15] in a 250 mL Schlenk flask charged with a magnetic stirring bar, $LiAlH_4$ (2.63 g, 69.3 mmol, 5.00 equiv) was added in THF (20 mL) at 0 °C. A mixture of methyl 6-(methylamino)-6-oxohexanoate (**S83**, 2.40 g, 13.9 mmol, 1.00 equiv) in THF (50 mL) was added dropwise at 0 °C over 30 min. The reaction mixture was stirred under reflux for 24 h. After cooling down to 0 °C, the reaction mixture was quenched with sat. aq. NH_4Cl and diluted with EtOAc (100 mL). The mixture was then filtered over Celite (4 × 3 cm). The filtrate was dried under reduced pressure to afford product **S84** (1.40 g, 10.7 mmol, 77%) as colourless oil, which was used without further purification.

^{1}H NMR (600 MHz, $CDCl_3$): δ = 1.38 (m, 4H, H-3, H-4), 1.50 (m, 2H, H-5), 1.57 (m, 2H, H-2), 2.43 (s, 3H, H-7), 2.57 (t, $^3J_{6,5}$ = 7.1 Hz, 2H, H-6), 3.67 (t, $^3J_{1,2}$ = 6.5 Hz, 2H, H-1), 4.57 (brs, 1H, N–*H)* ppm.
^{13}C NMR (151 MHz, $CDCl_3$): δ = 25.8 (C-3), 27.2 (C-4), 29.9 (C-5), 32.3 (C-2), 36.7 (C-7), 52.1 (C-6), 62.9 (C-1) ppm.
IR (ATR): ṽ = 3294 (br), 3078 (br), 2929 (s), 2855 (s), 2799 (m), 1457 (m), 1375 (m), 1200 (w), 1118 (m), 1058 (s), 857 (m) cm^{-1}.
HMRS (APCI) for $C_7H_{18}NO^+$: $[(M+H^+)]^+$: calculated: 132.1383, found: 132.1385.

4.10.3.3 6-Bromo-*N*-methylhexan-1-amine hydrobromide (S85)

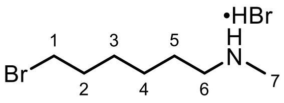

S85

$C_7H_{17}Br_2N$

Mw = 275.03 g mol^{-1}

In deference to a literature procedure,[58] in a 25 mL round bottom flask, aq. HBr (48% w/w in H_2O, 5.00 mL, 2.40 mmol, 2.00 equiv) was cooled to 0 °C. Then, 6-(methylamino)hexan-1-ol (**S84**, 1.10 g, 8.38 mmol, 1.00 equiv) was added over 30 min at 0 °C. The reaction mixture was stirred under reflux for 3.5 h. Residual H_2O and unreacted HBr were distilled off. After cooling down to room temperature, EtOAc (15 mL) was added, the precipitate was collected on a glass frit (P4) and washed with EtOAc (3 x 25 mL). The solid was dried under reduced pressure, yielded product **S85** (1.92 g, 6.98 mmol, 83%) as a brown solid.

mp: 144 °C.

^{1}H NMR (600 MHz, DMSO-d_6): δ = 1.32 (m, 2H, H-3), 1.40 (p, $^3J_{4,3/5}$ = 7.6 Hz, 2H, H-4), 1.55 (p, $^3J_{5,4/6}$ = 7.6 Hz, 2H, H-5), 1.80 (p, $^3J_{2,1/3}$ = 6.9 Hz, 2H, H-2), 2.55 (t, $^3J_{7,\text{N-}H}$ = 5.5 Hz, 3H, H-7), 2.86 (m, 2H, H-6), 3.53 (t, $^3J_{1,2/3}$ = 6.2 Hz, 2H, H-1), 8.32 (br s, 2H, N–*H*) ppm.

^{13}C NMR (151 MHz, DMSO-d_6): δ = 24.9 (C-3), 25.1 (C-5), 27.0 (C-4), 32.0 (C-2), 32.4 (C-7), 35.0 (C-1), 48.0 (C-6) ppm.

IR (ATR): ṽ = 3295 (br), 3078 (w), 2929 (m), 2855 (w), 2799 (w), 1457 (m), 1375 (w), 1200 (w), 1118 (w), 1058 (m), 857 (w) cm^{-1}.

HMRS (ESI): for $C_7H_{18}{}^{79}BrN^+$: [(M–H^{79}Br+H$^+$)]$^+$: calculated: 194.0539, found: 194.0542.

4.10.3.4 1-Mesityl-3-(6-(methylamino)hexyl)-1*H*-imidazol-3-ium bromide hydrobromide (S86)

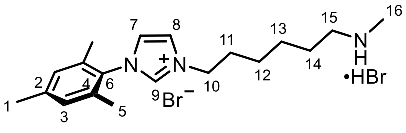

S86

$C_{19}H_{31}Br_2N_3$

Mw = 461.29 g mol^{-1}

In deference to a literature procedure,[3] in a 20 mL pressure tube, 1-(2,4,6-trimethylphenyl)imidazole (**S78**, 1.81 g, 9.73 mmol, 1.40 equiv) and 6-bromo-*N*-methylhexan-1-amine hydrobromide (**S85**, 1.91 g, 6.95 mmol, 1.00 equiv.) were dissolved in CH_3CN (3 mL) and heated at 95 °C for 48 h. After allowing the reaction mixture to cool to rt, all volatiles were removed under reduced pressure. The resulting solid was washed with EtOAc (3 x 30 mL) and dried under reduced pressure. The product **S86** (3.13 g, 6.79 mmol, 98%) was obtained as a light-brown solid.

^{1}H NMR (600 MHz, DMSO-*d*$_6$): δ = 1.27 (p, $^3J_{12,11/13}$ = 7.7 Hz, 2H, H-12), 1.35 (p, $^3J_{13,12/14}$ = 7.7 Hz, 2H, H-13), 1.56 (p, $^3J_{14,13/15}$ = 7.7 Hz, 2H, H-14), 1.89 (p, $^3J_{11,10/12}$ = 7.3 Hz, 2H, H-11), 2.01 (s, 6H, H-5), 2.33 (s, 3H, H-1), 2.54 (t, $^3J_{16,N\text{-}H}$ = 5.5 Hz, 3H, H-16), 2.85 (m, 2H, H-15), 4.28 (t, $^3J_{10,11}$ = 7.1 Hz, 2H, H-10), 7.15 (s, 2H, H-3), 7.95 (app t, *J* = 1.8 Hz, 1H, H-7)*, 8.11 (app t, *J* = 1.8 Hz, 1H, H-8)*, 8.31 (br s, 2H, N–*H*$_2$), 9.46 (s, 1H, H-9) ppm.

^{13}C NMR (151 MHz, DMSO-*d*$_6$): δ = 16.9 (C-5), 20.6 (C-1), 25.0 (C-12), 25.1 (C-14), 28.8 (C-11), 32.4 (C-16), 48.0 (C-15), 49.1 (C-10), 123.1 (C-8)*, 124.0 (C-7)*, 129.2 (C-3), 131.1 (C-6), 134.4 (C-4), 137.2 (C-9), 140.3 (C-2) ppm. C-13 could not be detected.

IR (ATR): ṽ = 2937 (br), 2862 (s), 2743 (br), 1610 (m), 1546 (s), 1461 (s), 1379 (s), 1244 (w), 1203 (s), 1162 (m), 1107 (w), 1069 (m), 1039 (m), 935 (w), 857 (m), 764 (m) cm^{-1}.

HMRS (**ESI**): [$C_{19}H_{30}N_3^+$ [(M-2H^{79}Br+H$^+$)]$^+$: calculated: 300.2434, found: 300.2426.

4.10.3.5 (*E*)-3-(6-(2,3-Diisopropyl-1-methylguanidino)hexyl)-1-mesityl-*1H*-imidazol-3-ium bromide hydrobromide (S87)

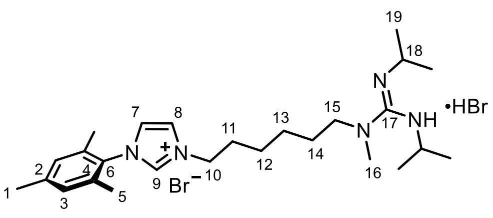

S87
$C_{26}H_{45}Br_2N_5$
Mw = 587.49 g mol^{-1}

In deference to a literature procedure,[3] in a 20 mL pressure tube, 1-mesityl-3-(6-(methylamino)hexyl)-1*H*-imidazol-3-ium bromide hydrobromide (**S86**, 2.00 g, 4.34 mmol, 1.00 equiv) and *N*,*N'*-diisopropylcarbodiimide (1.34 mL, 6.51 mmol, 1.50 equiv) were dissolved in a mixture of chlorobenzene and acetonitrile (10 mL, 1:1) and heated to 120 °C for 18 h. After allowing the reaction mixture to cool to rt, all volatiles were removed under reduced pressure. The resulting solid was washed with EtOAc (3 x 20 mL) and dried under reduced pressure. The product **S87** (1.90 g, 3.23 mmol, 75%) was obtained as a brown solid.

^{1}H NMR (600 MHz, DMSO-d_6): δ = 1.19 (d, $^3J_{19,18}$ = 6.4 Hz, 12H, H-19), 1.26 (m, 4H, H-12, H-13), 1.53 (m, 2H, H-14), 1.89 (m, 2H, H-11), 2.01 (s, 6H, H-5), 2.33 (s, 3H, H-1), 2.91 (s, 3H, H-16), 3.26 (t, $^3J_{15,14}$ = 6.9 Hz, 2H, H-15), 3.66 (m, 2H, H-18), 4.28 (t, $^3J_{10,11}$ = 7.2 Hz, 2H, H-10), 7.15 (s, 2H, H-3), 7.28 (br s, 2H, N–Ha, *H*b), 7.95 (s, 1H, H-7)*, 8.12 (s, 1H, H-8)*, 9.48 (s, 1H, H-9) ppm.

^{13}C NMR (151 MHz, DMSO-d_6): δ = 16.9 (C-5), 20.6 (C-1), 22.8 (C-19), 25.1 (C-12), 25.2 (C-13), 26.5 (C-14), 29.0 (C-11), 37.3 (C-16), 47.0 (C-18), 49.3 (C-10), 51.0 (C-15), 123.3 (C-8)*, 124.0 (C-7)*, 129.3 (C-3), 131.1 (C-6), 134.3 (C-4), 137.4 (C-9), 140.3 (C-2), 157.8 (C-17) ppm.

IR (ATR): ṽ 3198 (br), 3160 (br), 3041 (br), 2974 (s), 2937 (s), 2870 (br), 1610 (s), 1565 (s), 1449 (m), 1330 (m), 1271 (m), 1203 (m), 1159 (m), 1133 (m), 1066 (m), 857 (m), 730 (s), 697 (m) cm^{-1}.

HMRS (ESI): [$C_{26}H_{44}N_5^+$ [M-2H^{79}Br+H$^+$)]: calculated: 426.3591, found: 385.2958.

The possible fragment could be

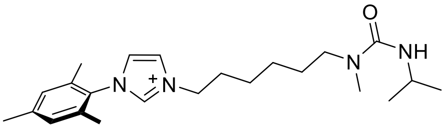

$C_{23}H_{37}N_4O^+$
Exact Mass: 385,2962

4.10.3.6 (*E*)-(1-(6-(2,3-Diisopropyl-1-methylguanidino)hexyl)-3-mesityl-1, 3-dihydro-2*H*-imidazol-2-ylidene)copper(I) chloride (7)

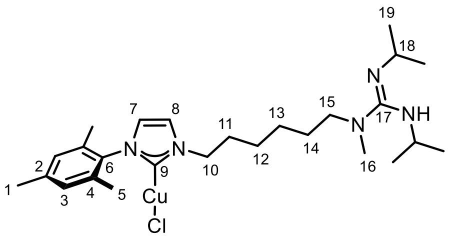

7
$C_{26}H_{43}ClCuN_5$
Mw = 524.26

In deference to a literature procedure,[59] in a 25 mL Schlenk flask charged with magnetic stirring bar, (*E*)-3-(6-(2,3-diisopropyl-1-methylguanidino)hexyl)-1-mesityl-1*H*-imidazol-3-ium bromide hydrobromide (**S87**, 85.0 mg, 0.145 mmol, 1.00 equiv), CuCl (14.3 mg, 0.145 mmol , 1.00 equiv) and NaO*t*Bu (30.5 mg, 0.320 mmol, 2.20 equiv) were added and dissolved in THF (4 mL). The reaction mixture was then stirred for 3 h at 40 °C. The resulting brown mixture was then filtered under N_2 atmosphere using a Schlenk frit (P4). The filtrate was then dried under reduced pressure, resulting the copper(I) complex **7** as brown solid (22.2 mg, 0.0370 mmol, 25%).

^{1}H NMR (600 MHz, $CDCl_3$): δ = 1.19 (d, $^3J_{19,18}$ = 6.1 Hz, 12H, H-19), 1.33 (m, 4H, H-12, H-13), 1.56 (p, $^3J_{14,13/15}$ = 7.1 Hz, 2H, H-14), 1.91 (p, $^3J_{11,10/12}$ = 7.2 Hz, 2H, H-11), 1.97 (s, 6H, H-5), 2.30 (s, 3H, H-1), 2.90 (s, 3H, H-16), 3.23 (m, 2H, H-15), 3.48 (m, 2H, H-18), 4.24 (t, $^3J_{10,11}$ = 6.9 Hz, 2H, H-10), 6.86 (s, 1H, H-7)*, 6.89 (s, 2H, H-3), 7.28 (s, 1H, H-8)* ppm.
The compound contains impurities; signals at 1.10 and 1.12 ppm.
^{13}C NMR (151 MHz, $CDCl_3$): δ = 17.7 (C-5), 21.0 (C-1), 22.7 (C-12)*, 24.6 (C-19), 25.6 (C-13)*, 27.3 (C-14), 31.2 (C-11), 34.3 (C-16), 48.4 (C-15), 49.6 (C-18), 50.9 (C-10), 120.9 (C-8)*, 122.4 (C-7)*, 129.3 (C-3), 134.6 (C-4), 135.2 (C-6), 139.3 (C-2), 163.3 (C-17), 177.2 (C-9) ppm.
HRMS (ACPI) for $C_{26}H_{43}CuN_5^+$ [(M–$^{35}Cl^-$)$^+$]: calculated: 488.2823, found: 488.2810.

4.10.4 Cu(I)-catalyzed reduction of morpholino(phenyl)methanone (1j) with Cu(I)-complex 7 and H_2

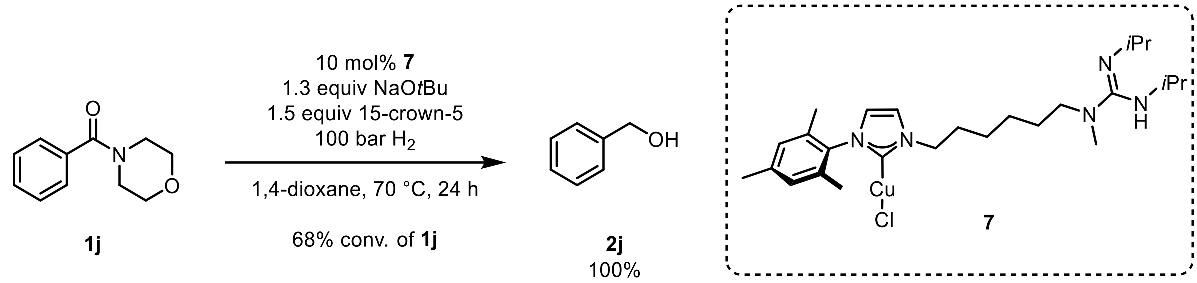

Scheme S36: Cu(I)-catalyzed reduction of morpholino(phenyl)methanone (**1j**) with Cu(I)-complex **7** and H_2.

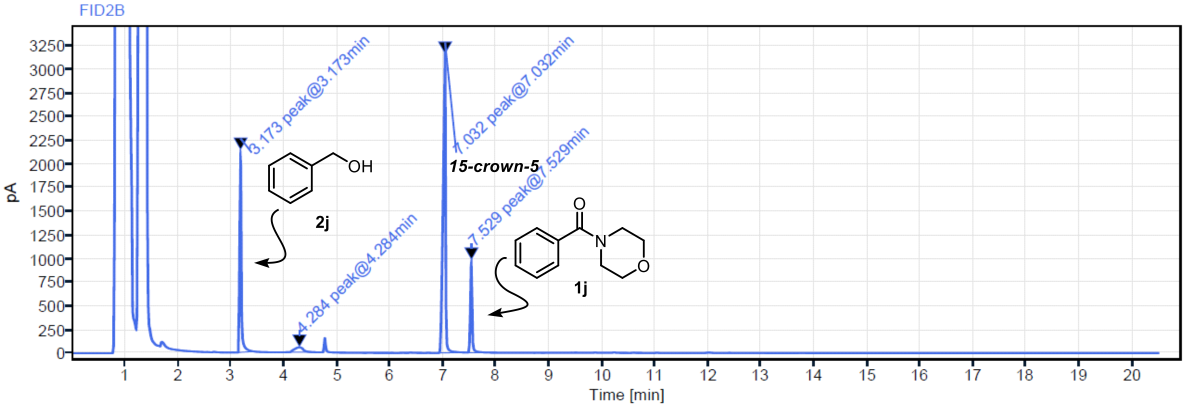

Signal: FID2B

Name	RT [min]	RF	Area	Peak Area Percent
peak@3.173min	3.17		4696.427	26.03
peak@4.284min	4.28		610.875	3.39
peak@7.032min	7.03		10488.577	58.14
peak@7.529min	7.53		2245.782	12.45

Figure S27: GC analysis of the reaction micture of the Cu(I)-catalyzed reduction of morpholino(phenyl)methanone (**1j**) with Cu(I)-complex **7** and H_2.

5 Competition experiments

5.1 Competition experiments / Cu(I)-catalyzed reduction of amides with H_2

All competitions experiments have been carried out according to **GP3** (10 mo% catalyst **3**, 1.3 equiv NaO*t*Bu, 1.5 equiv 15-crown-5, 100 bar H_2, 1,4-dioxane, 70 °C, called *standard conditions* in Scheme S37 below) except for a shorter reaction time of 4 h in order to identify subtle reactivity differences. As part of this study, we have established that generally, aryl amides more readily than alkyl amides (coined *reactive* vs. *unreactive* in this study). This can be traced back to the fact that alkyl amides are enolizable and therefore could undergo detrimental deprotonation under the strongly basic reaction conditions.

With the following competition experiments, it becomes clear that *privileged* amides based on morpholine can overrule the *reactive/unreactive* imperative.

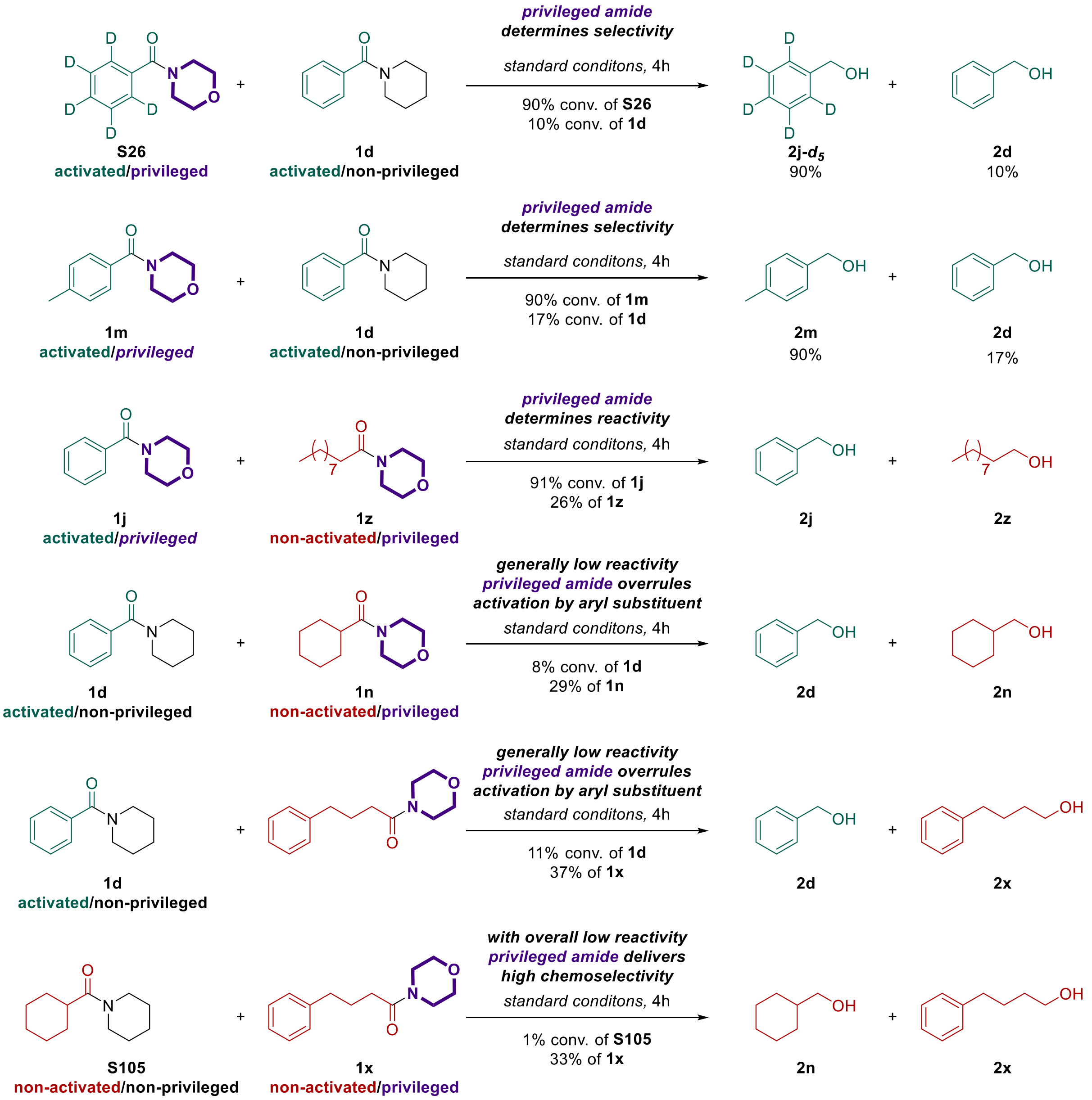

Scheme S37: Overall results of competition experiments of Cu(I)-catalyzed reduction of amides with H_2. (all values are conversions of the respective starting materials as confirmed by GC and/or ^{1}H NMR analysis)

5.1.1 Morpholino(phenyl-d5)methanone (S26) Vs phenyl(piperidin-1-yl)methanone (1d)

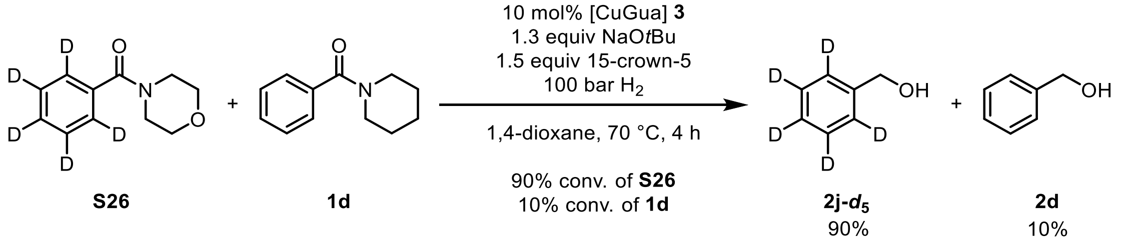

Scheme S38: Competition experiment between morpholino(phenyl-*d*5)methanone (**S26**) and phenyl(piperidin-1-yl)methanone (**1d**).

Carried out according to **GP3** morpholino(phenyl-*d*5)methanone (**S26**, 39 mg, 0.20 mmol, 1.0 equiv), phenyl(piperidin-1-yl)methanone (**1d**, 38 mg, 0.20 mmol, 1.0 equiv), [CuGua] **3** (9.4 mg, 20 µmol, 10 mol%), NaO*t*Bu (25 mg, 0.26 mmol, 1.3 equiv) and 15-crown-5 (60 µL, 0.30 mmol, 1.5 equiv) in 1,4-dioxane (4.0 mL). The reaction mixture was stirred for 4 h at 70 °C. Purification by flash column chromatography on silica gel (cyclohexane/ethyl acetate = 90:10) gave **2j-*d*5** and **2d** as yellow oil.

No overall yield determined.

With ^{1}H NMR and ^{2}H NMR experiments the ratio of (phenyl-*d*5)methanol (**2j-*d*5**) and benzyl alcohol (**2d**) was determined to be **2j-*d*5/2d** = 90:10.

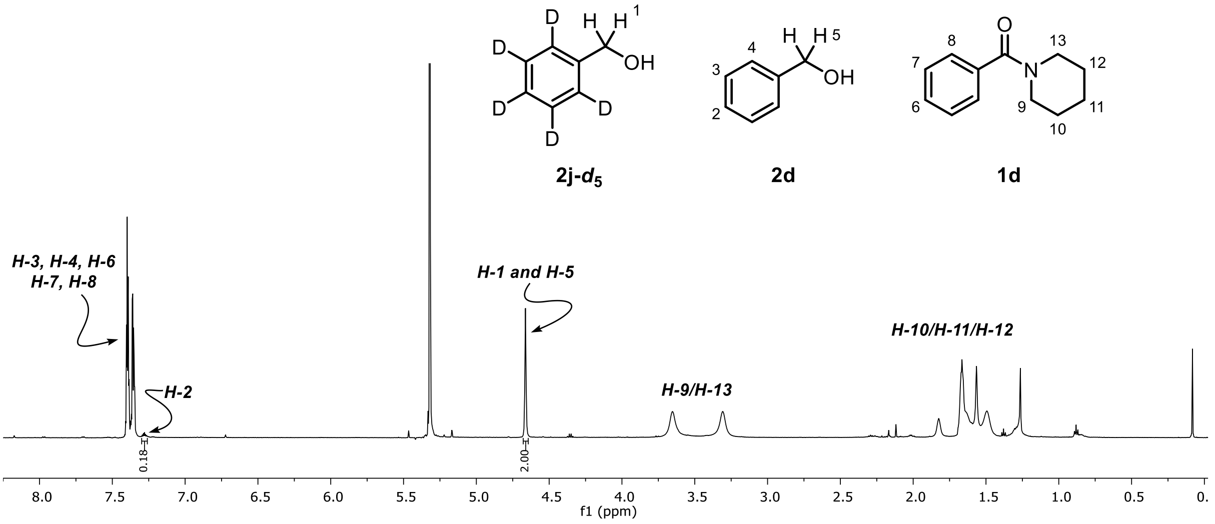

Figure S28: ^{1}H NMR of the isolated mixture after competition experiment (600 MHz, $CDCl_3$).

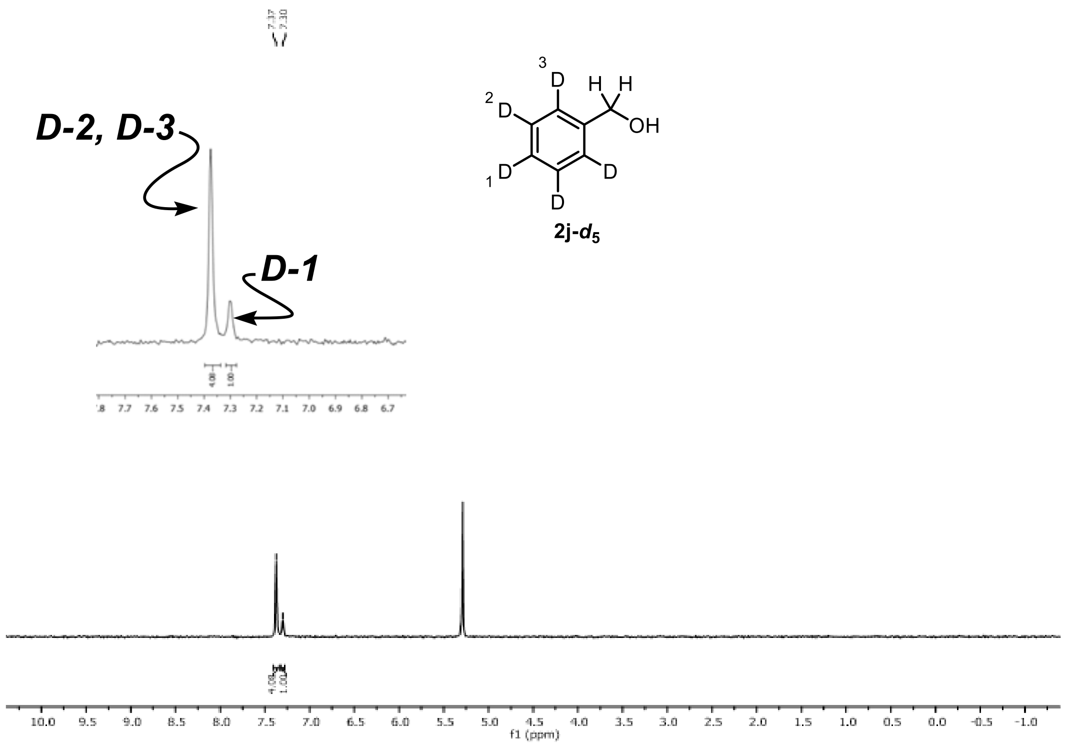

Figure S29: ^{2}H NMR of the isolated mixture after competition experiment (92 MHz, $CHCl_3$).

5.1.2 Morpholino(*p*-tolyl)methanone (1m) Vs phenyl(piperidin-1-yl)methanone (1d)

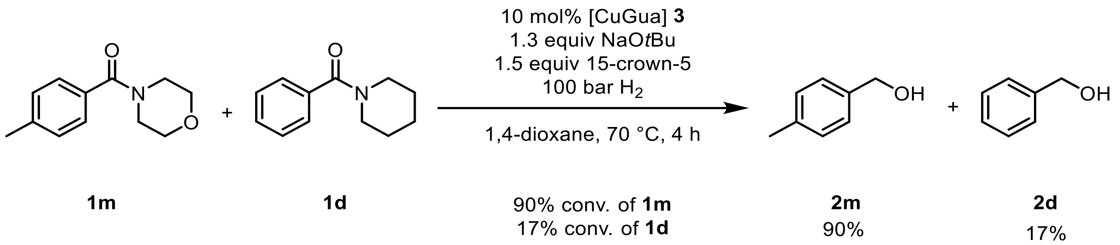

Scheme S39: Competition experiment between morpholino(*p*-tolyl)methanone (**1m**) and phenyl(piperidin-1-yl)methanone (**2d**).

Prepared according to **GP3** from morpholino(*p*-tolyl)methanone (**1m,** 41 mg, 0.20 mmol, 1.0 equiv), phenyl(piperidin-1-yl)methanone (**1d,** 38 mg, 0.20 mmol, 1.0 equiv), [CuGua] **3** (9.4 mg, 20 µmol, 10 mol%), NaO*t*Bu (25 mg, 0.52 mmol, 1.3 equiv), tridecane (3.67 mg, 0.020 mmol, 10 mol%) and 15-crown-5 (0.06 mL, 0.30 mmol, 1.5 equiv) in 1,4-dioxane (2.0 mL). The reaction mixture was stirred for 4 h at 70 °C. Analysis of the crude mixture was carried out by GC and GC-MS.

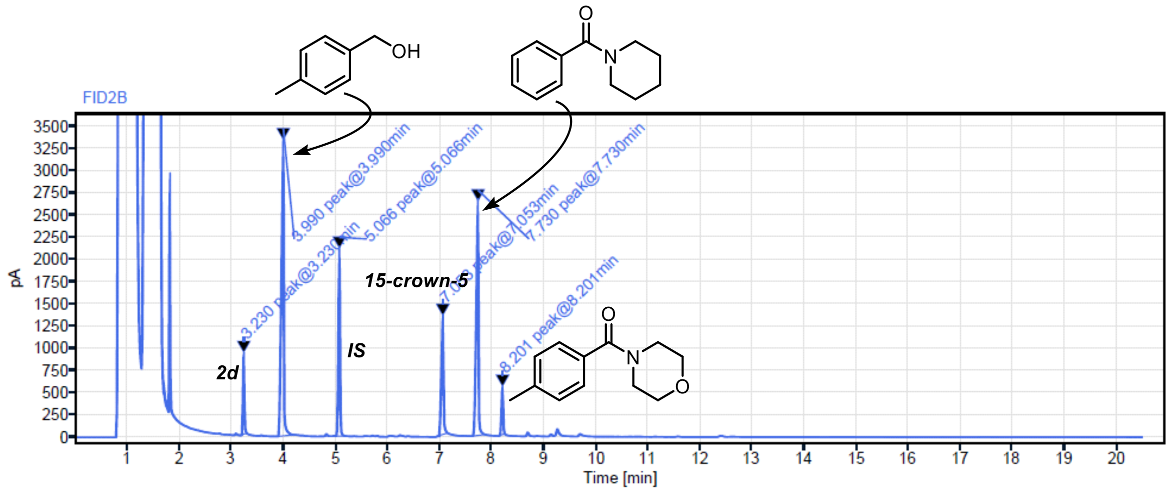

Signal: FID2B

Name	RT [min]	RF	Area	Peak Area Percent
peak@3.230min	3.23		1562.624	5.47
peak@3.990min	3.99		10237.321	35.84
peak@5.066min	5.07		4410.825	15.44
peak@7.053min	7.05		3602.195	12.61
peak@7.730min	7.73		7626.078	26.70
peak@8.201min	8.20		1121.748	3.93

Figure S30: GC analysis of the reaction mixture of the competition experiment between morpholino(*p*-tolyl)methanone (**1m**) and phenyl(piperidin-1-yl)methanone (**1d**) (GC method: 40_20_250_10).

5.1.3 Morpholino(phenyl)methanone (1j) Vs 1-Morpholinodecan-1-one (1z)

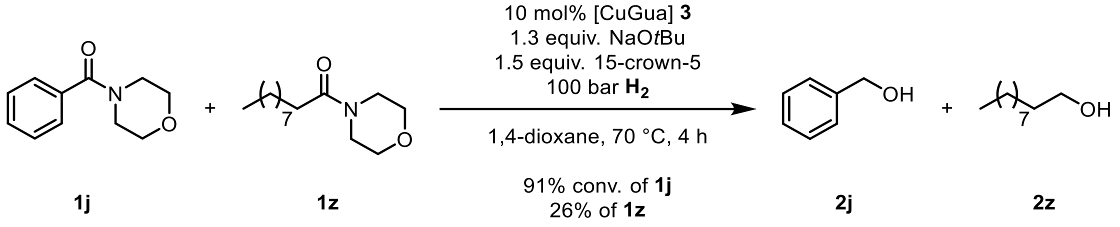

Scheme S40: Competition experiment between morpholino(phenyl)methanone (**1j**) and 1-morpholinodecan-1-one (**1z**).

Prepared according to **GP3** from morpholino(phenyl)methanone (**1j,** 38 mg, 0.20 mmol, 1.0 equiv), 1-morpholinodecan-1-one (**1z,** 48 mg, 0.20 mmol, 1.0 equiv), [CuGua] **3** (9.4 mg, 20 µmol, 10 mol%), NaO*t*Bu (25 mg, 0.26 mmol, 1.3 equiv), and 15-crown-5 (0.06 mL, 0.30 mmol, 1.5 equiv) in 1,4-dioxane (2.0 mL). The reaction mixture was stirred for 4 h at 70 °C. Analysis of the crude mixture was carried out by GC, GC-MS and ^{1}H NMR.

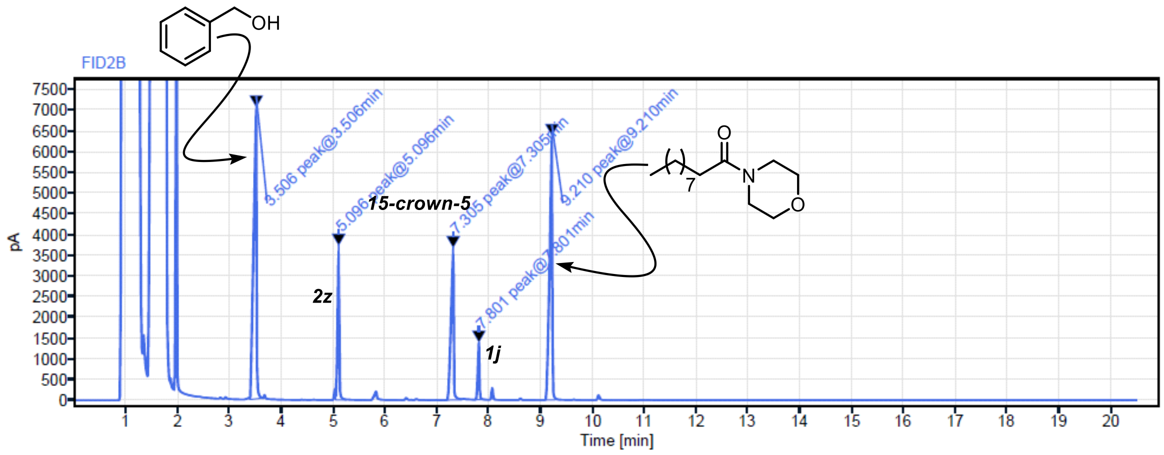

Signal: FID2B

Name	RT [min]	RF	Area	Peak Area Percent
peak@3.506min	3.51		28325.973	37.59
peak@5.096min	5.10		8233.289	10.93
peak@7.305min	7.30		12982.813	17.23
peak@7.801min	7.80		2829.527	3.75
peak@9.210min	9.21		22989.934	30.51

Figure S31: GC analysis of the reaction mixture of the competition experiments between morpholino(phenyl)methanone (**1j**) and 1-morpholinodecan-1-one (**1z**) (GC method: 40_20_250_10).

5.1.4 Phenyl(piperidin-1-yl)methanone (1d) vs Cyclohexyl(morpholino)methanone (1n)

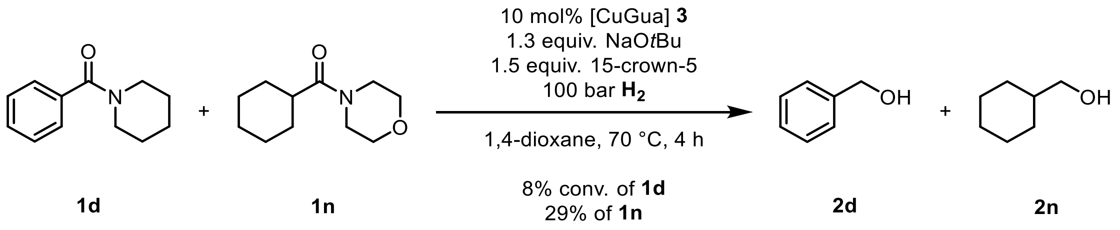

Scheme S41: Competition experiment between phenyl(piperidin-1-yl)methanone (**1d**) and cyclohexyl(morpholino)methanone (**1n**).

Prepared according to **GP3** from phenyl(piperidin-1-yl)methanone (**1d,** 19 mg, 0.10 mmol, 1.0 equiv), cyclohexyl(morpholino)methanone (**1n,** 20 mg, 0.10 mmol, 1.0 equiv), [CuGua] **3** (4.7 mg, 10 µmol, 10 mol%), NaO*t*Bu (12 mg, 0.13 mmol, 1.3 equiv), and 15-crown-5 (0.03 mL, 0.15 mmol, 1.5 equiv) in 1,4-dioxane (1.0 mL). The reaction mixture was stirred for 4 h at 70 °C. Analysis of the crude mixture was carried out by GC, GC-MS and ^{1}H NMR.

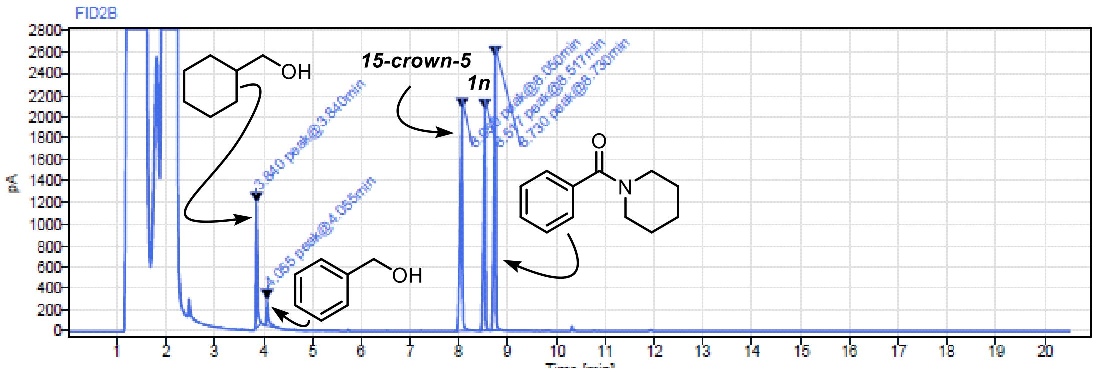

Signal: FID2B

Name	RT [min]	RF	Area	Peak Area Percent
peak@3.840min	3.84		1929.346	9.81
peak@4.055min	4.06		536.066	2.73
peak@8.050min	8.05		6336.458	32.22
peak@8.517min	8.52		4758.483	24.20
peak@8.730min	8.73		6105.267	31.05

Figure S32: GC analysis of the reaction mixture of the competition experiments between phenyl(piperidin-1-yl)methanone (**1d**) and cyclohexyl(morpholino)methanone (**1n**) (GC method: 40_20_250_10).

5.1.5 Phenyl(piperidin-1-yl)methanone (1d) vs 1-Morpholino-4-phenylbutan-1-one (1x)

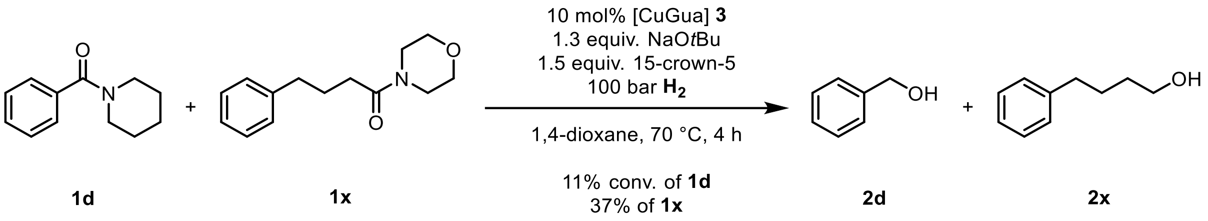

Scheme 42: Competition experiment between phenyl(piperidin-1-yl)methanone (**1d**) and 1-morpholino-4-phenylbutan-1-one (**1x**).

Prepared according to **GP3** from phenyl(piperidin-1-yl)methanone (**1d,** 19 mg, 0.10 mmol, 1.0 equiv), 1-morpholino-4-phenylbutan-1-one (**1x,** 23 mg, 0.10 mmol, 1.0 equiv), [CuGua] **3** (4.7 mg, 10 µmol, 10 mol%), NaO*t*Bu (12 mg, 0.13 mmol, 1.3 equiv), and 15-crown-5 (0.03 mL, 0.15 mmol, 1.5 equiv) in 1,4-dioxane (1.0 mL). The reaction mixture was stirred for 4 h at 70 °C. Analysis of the crude mixture was carried out by GC, GC-MS and ^{1}H NMR.

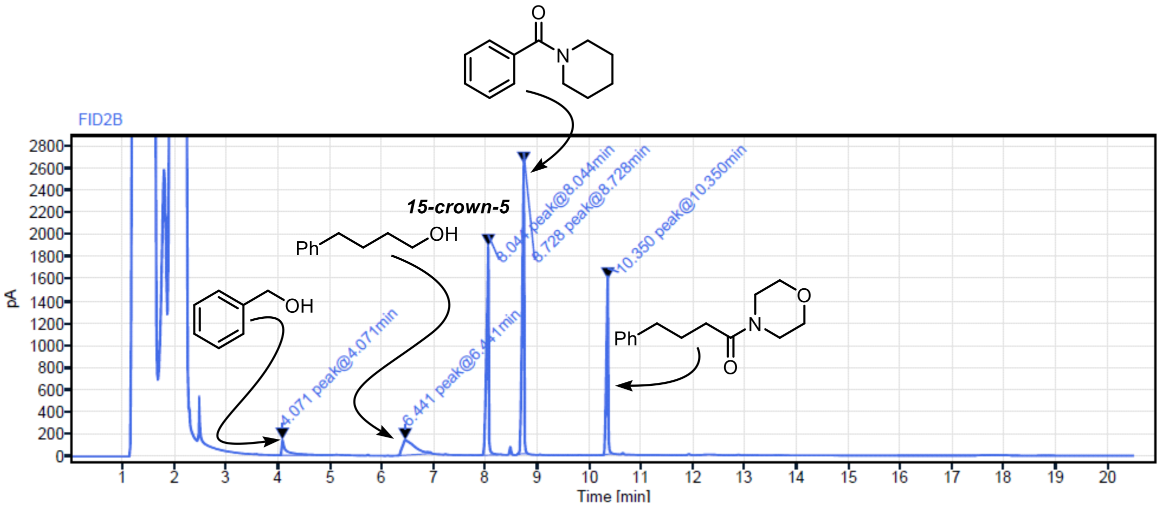

Signal: FID2B

Name	RT [min]	RF	Area	Peak Area Percent
peak@4.071min	4.07		885.943	4.64
peak@6.441min	6.44		2249.878	11.78
peak@8.044min	8.04		5482.403	28.70
peak@8.728min	8.73		6701.500	35.08
peak@10.350min	10.35		3781.209	19.80

Figure S33: GC analysis of the reaction mixture of the competition experiments between phenyl(piperidin-1-yl)methanone (**1d**) and 1-morpholino-4-phenylbutan-1-one (**1x**) (GC method: 40_20_250_10).

5.1.6 Cyclohexyl(piperidin-1-yl)methanone (S105) vs 1-Morpholino-4-phenylbutan-1-one (1x)

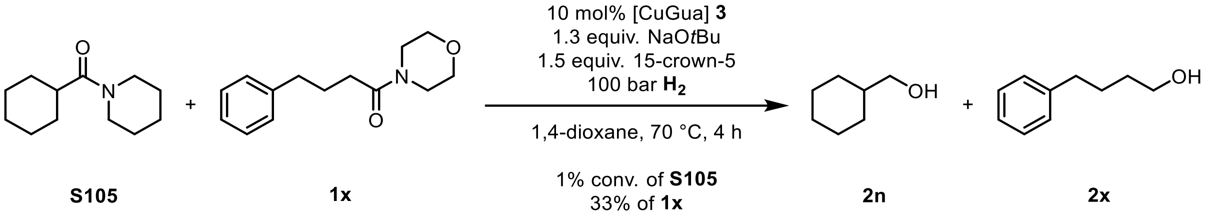

Scheme S43: Competition experiment between cyclohexyl(piperidin-1-yl)methanone (**S105**) and 1-morpholino-4-phenylbutan-1-one (**1x**).

Prepared according to **GP3** from cyclohexyl(piperidin-1-yl)methanone (**S105,** 20 mg, 0.10 mmol, 1.0 equiv), 1-morpholino-4-phenylbutan-1-one (**1x,** 23 mg, 0.10 mmol, 1.0 equiv), [CuGua] **3** (4.7 mg, 10 µmol, 10 mol%), NaO*t*Bu (12 mg, 0.13 mmol, 1.3 equiv), and 15-crown-5 (0.03 mL, 0.15 mmol, 1.5 equiv) in 1,4-dioxane (1.0 mL). The reaction mixture was stirred for 4 h at 70 °C. Analysis of the crude mixture was carried out by GC, GC-MS and ^{1}H NMR.

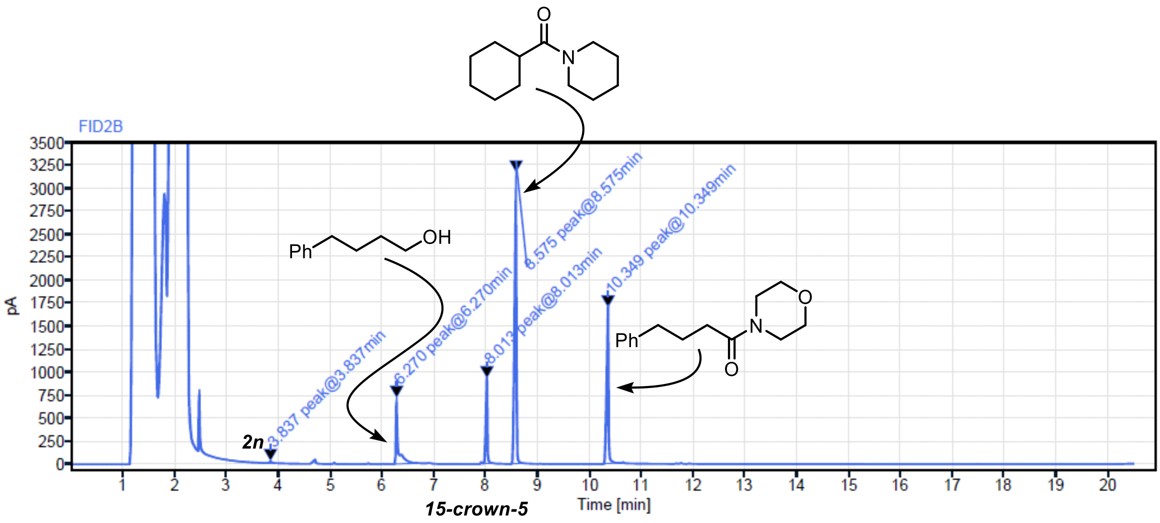

Signal: FID2B

Name	RT [min]	RF	Area	Peak Area Percent
peak@3.837min	3.84		35.943	0.23
peak@6.270min	6.27		2093.338	13.41
peak@8.013min	8.01		1817.311	11.64
peak@8.575min	8.58		7471.558	47.87
peak@10.349min	10.35		4189.047	26.84

Figure S34: GC analysis of the reaction mixture of the competition experiments between cyclohexyl(piperidin-1-yl)methanone (**S105**) and 1-morpholino-4-phenylbutan-1-one (**1x**) (GC method: 40_20_250_10).

5.2 Competition experiments reduction of amides using stoichiometric reducing reagents

5.2.1 Morpholino(*p*-tolyl)methanone (1m) Vs phenyl(piperidin-1-yl)methanone (1d)-Reduction with D*i*BAl–H

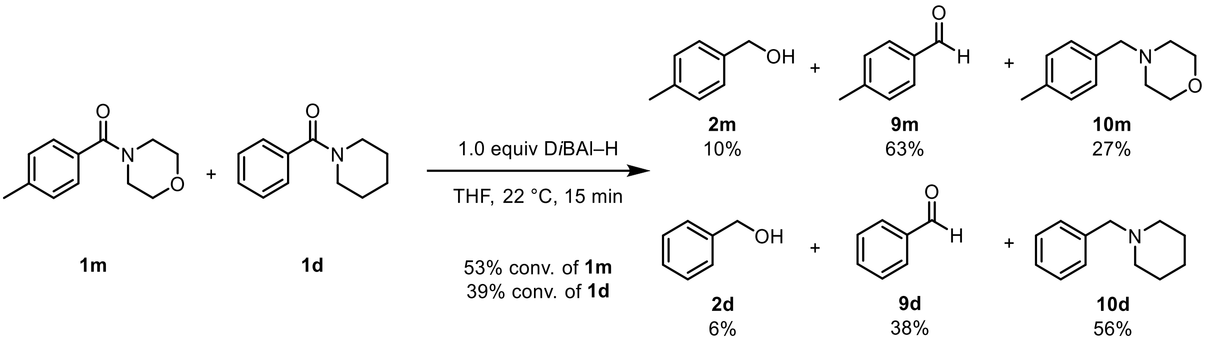

Scheme S44: Competition experiment between morpholino(*p*-tolyl)methanone (**1m**) and phenyl(piperidin-1-yl)methanone (**1d**) with D*i*BAl–H as the reducing agent.

Prepared according to **GP6** from morpholino(*p*-tolyl)methanone (**1m,** 62 mg, 0.30 mmol, 1.0 equiv), phenyl(piperidin-1-yl)methanone (**1d,** 57 mg, 0.30 mmol, 1.0 equiv), D*i*BAl–H (1.2 M in toluene, 0.25 mL, 0.30 mmol, 1.0 equiv) in dry THF (3.0 mL). The reaction mixture was stirred for 15 min at rt. Analysis of the crude mixture was carried out by GC, GCMS and/or ^{1}H NMR.

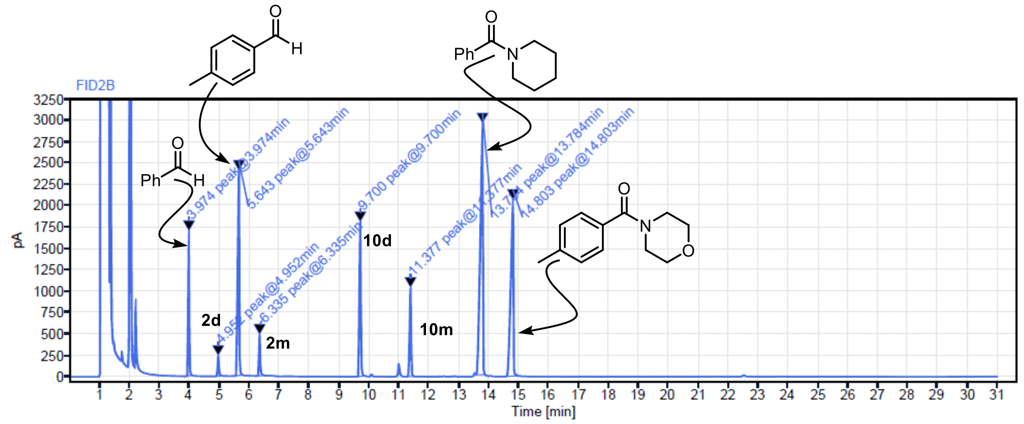

Signal: FID2B

**Name**	**RT [min]**	**RF**	**Area**	**Peak Area Percent**
peak@3.974min	3.97		4109.545	7.69
peak@4.952min	4.95		656.383	1.23
peak@5.643min	5.64		8516.162	15.93
peak@6.335min	6.33		1362.748	2.55
peak@9.700min	9.70		6072.674	11.36
peak@11.377min	11.38		3448.342	6.45
peak@13.784min	13.78		17258.978	32.28
peak@14.803min	14.80		12049.554	22.53

Figure S35: GC analysis of the reaction mixture of the competition experiment between morpholino(*p*-tolyl)methanone (**1m**) and phenyl(piperidin-1-yl)methanone (**1d**) with D*i*BAl–H as the reducing agent (GC method: 40_10_250_10).

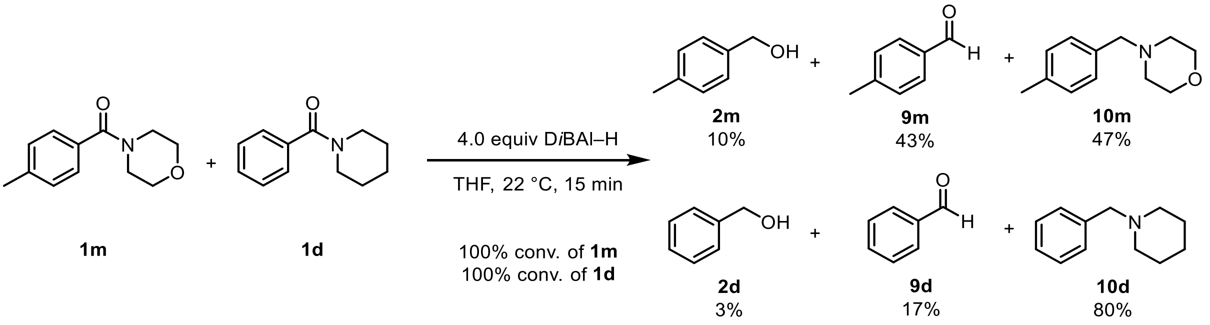

Scheme S45: Competition experiment between morpholino(*p*-tolyl)methanone (**1m**) and phenyl(piperidin-1-yl)methanone (**1d**) with excess of D*i*BAl–H as the reducing agent.

Prepared according to **GP6** from morpholino(*p*-tolyl)methanone (**1m,** 62 mg, 0.30 mmol, 1.0 equiv), phenyl(piperidin-1-yl)methanone (**1d,** 57 mg, 0.30 mmol, 1.0 equiv), D*i*BAl–H (1.0 M in toluene, 1.2 mL, 1.2 mmol, 1.0 equiv) in dry THF (3.0 mL). The reaction mixture was stirred for 15 min at rt. Analysis of the crude mixture was carried out by GC, GCMS and/or ^{1}H NMR.

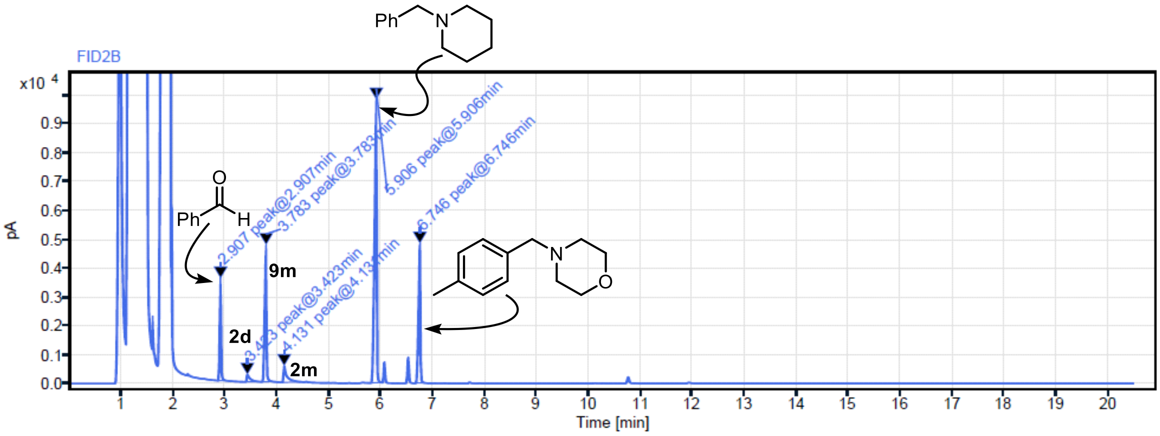

Signal: FID2B

Name	RT [min]	RF	Area	Peak Area Percent
peak@2.907min	2.91		5825.601	9.99
peak@3.423min	3.42		1170.200	2.01
peak@3.783min	3.78		10630.411	18.24
peak@4.131min	4.13		2551.859	4.38
peak@5.906min	5.91		26705.565	45.81
peak@6.746min	6.75		11410.649	19.57

Figure S36: GC analysis of the reaction mixture of the competition experiment between morpholino(*p*-tolyl)methanone (**1m**) and phenyl(piperidin-1-yl)methanone (**1d**) with excess of D*i*BAl–H as the reducing agent (GC method: 40_20_250_10).

5.2.2 Morpholino(*p*-tolyl)methanone (1m) Vs phenyl(piperidin-1-yl)methanone (1d)-Reduction with $LiAlH_4$

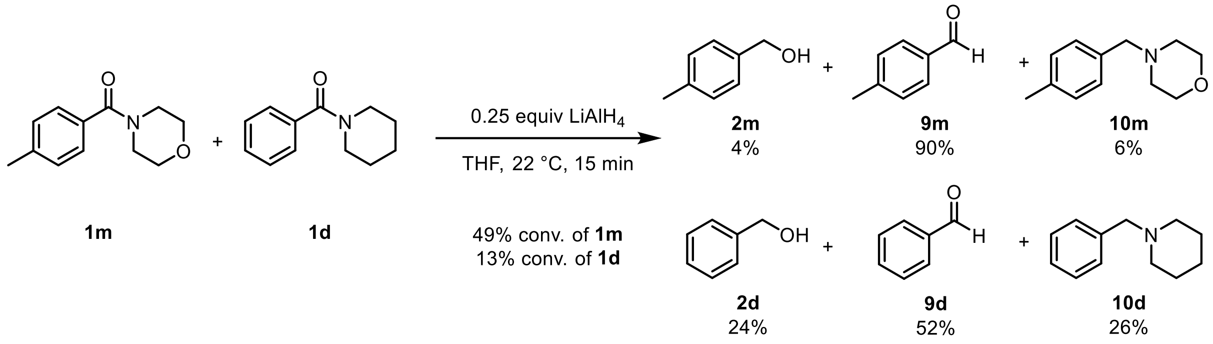

Scheme S46: Competition experiment between morpholino(*p*-tolyl)methanone (**1m**) and phenyl(piperidin-1-yl)methanone (**1d**) with $LiAlH_4$ as the reducing agent.

Prepared according to **GP5** from morpholino(*p*-tolyl)methanone (**1m,** 62 mg, 0.30 mmol, 1.0 equiv), phenyl(piperidin-1-yl)methanone (**1d,** 57 mg, 0.30 mmol, 1.0 equiv), $LiAlH_4$ (2.8 mg, 0.075 mmol, 0.25 equiv) in dry THF (3.0 mL). The reaction mixture was stirred for 15 min at rt. Analysis of the crude mixture was carried out by GC, GCMS and/or ^{1}H NMR.

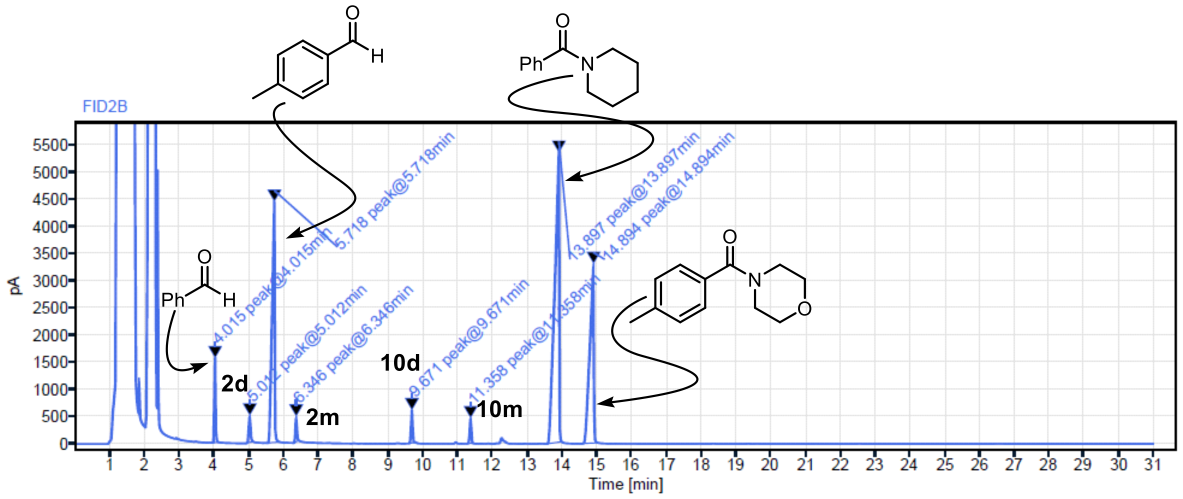

Signal: FID2B

Name	RT [min]	RF	Area	Peak Area Percent
peak@4.015min	4.02		3621.779	3.45
peak@5.012min	5.01		1708.190	1.63
peak@5.718min	5.72		22576.577	21.49
peak@6.346min	6.35		1242.895	1.18
peak@9.671min	9.67		1792.243	1.71
peak@11.358min	11.36		1413.355	1.35
peak@13.897min	13.90		46853.954	44.59
peak@14.894min	14.89		25863.319	24.61

Figure S37: GC analysis of the reaction mixture of the competition experiment between morpholino(*p*-tolyl)methanone (**1m**) and phenyl(piperidin-1-yl)methanone (**1d**) with $LiAlH_4$ as the reducing agent (GC method: 40_10_250_10).

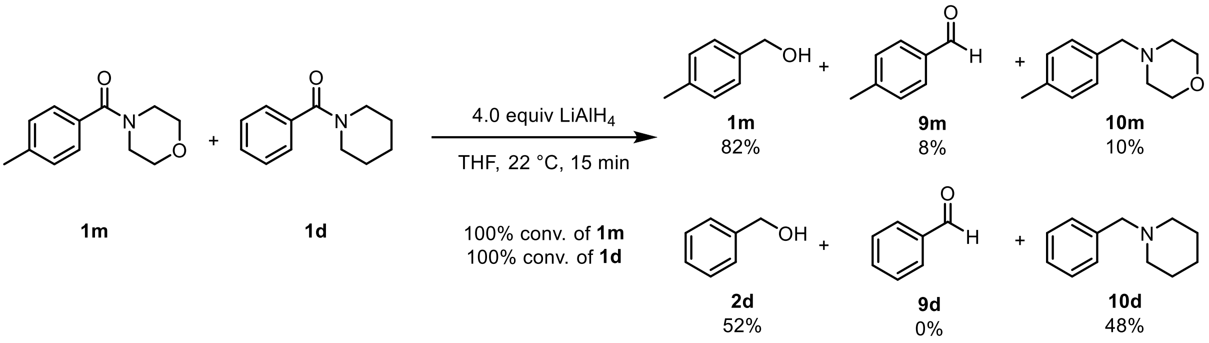

Scheme S47: Competition experiment between morpholino(*p*-tolyl)methanone (**1m**) and phenyl(piperidin-1-yl)methanone (**1d**) with excess of $LiAlH_4$ as the reducing agent.

Prepared according to **GP5** from morpholino(*p*-tolyl)methanone (**1m,** 62 mg, 0.30 mmol, 1.0 equiv), phenyl(piperidin-1-yl)methanone (**1d,** 57 mg, 0.30 mmol, 1.0 equiv), $LiAlH_4$ (46 mg, 1.2 mmol, 4.0 equiv) in dry THF (3.0 mL). The reaction mixture was stirred for 15 min at rt. Analysis of the crude mixture was carried out by GC, GCMS and/or ^{1}H NMR.

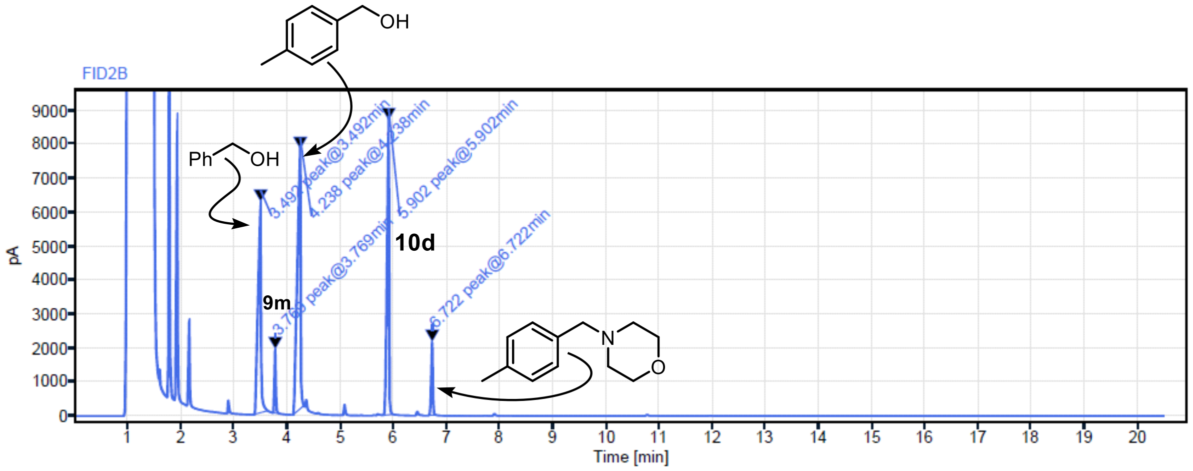

Signal: FID2B

Name	RT [min]	RF	Area	Peak Area Percent
peak@3.492min	3.49		24258.915	27.91
peak@3.769min	3.77		3213.480	3.70
peak@4.238min	4.24		32614.404	37.53
peak@5.902min	5.90		22534.508	25.93
peak@6.722min	6.72		4285.032	4.93

Figure S38: GC analysis of the reaction mixture of the competition experiment between morpholino(*p*-tolyl)methanone (**1m**) and phenyl(piperidin-1-yl)methanone (**1d**) with excess of $LiAlH_4$ as the reducing agent (GC method: 40_20_250_10).

5.3 Potential formation of an aldehyde as intermediate

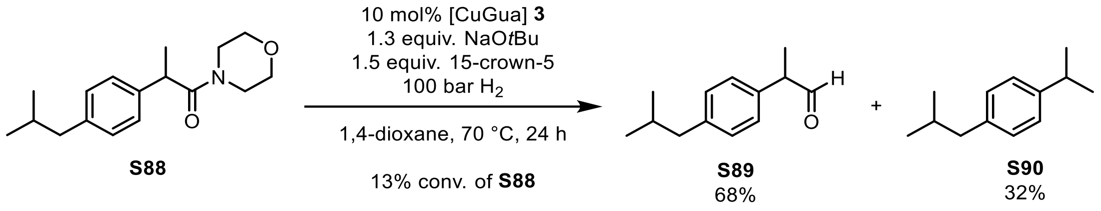

Scheme S48: Cu(I)-catalyzed reduction of 2-(4-isobutylphenyl)-1-morpholino-propan-1-one (**S88**).

Carried out according to **GP3** from 2-[4-(2-methylpropyl)phenyl]-1-morpholin-4-ylpropan-1-one (**S88,** 28 mg, 0.10 mmol, 1.0 equiv), [CuGua] **3** (4.7 mg, 10 µmol, 10 mol%), NaO*t*Bu (12 mg, 0.13 mmol, 1.3 equiv) and 15-crown-5 (30 µL, 0.15 mmol, 1.5 equiv) in 1,4-dioxane (1.0 mL). The reaction mixture was stirred for 24 h at 70 °C. Crude reaction mixture was analysed by ^{1}H NMR, GC, GC/MS. Due to the steric hindrance in the *α*-position of the amide reacting center, 13% of conversion was observed. Analysis of the mixture showed the presence of the aldehyde **S89**, as a possible reaction intermediate.

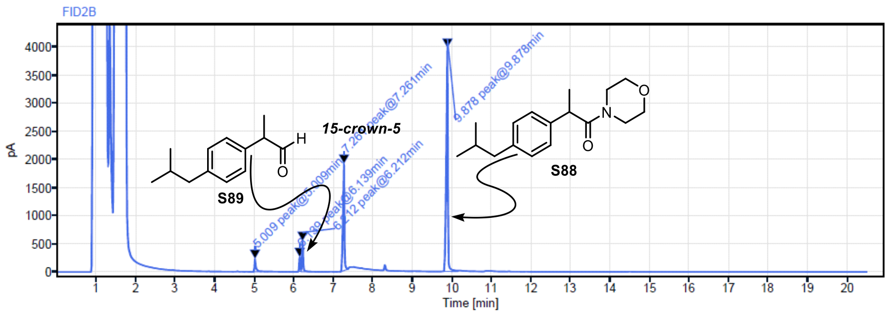

Signal: FID2B

Name	RT [min]	RF	Area	Peak Area Percent
peak@5.009min	5.01		603.641	3.25
peak@6.139min	6.14		488.894	2.63
peak@6.212min	6.21		1152.297	6.21
peak@7.261min	7.26		4868.498	26.23
peak@9.878min	9.88		11448.421	61.68

Figure S39: GC analysis of the crude reaction mixture of the Cu(I)-catalyzed reduction of ibuprofen derivative **S88** with H_2 (GC method: 40_20_250_10).

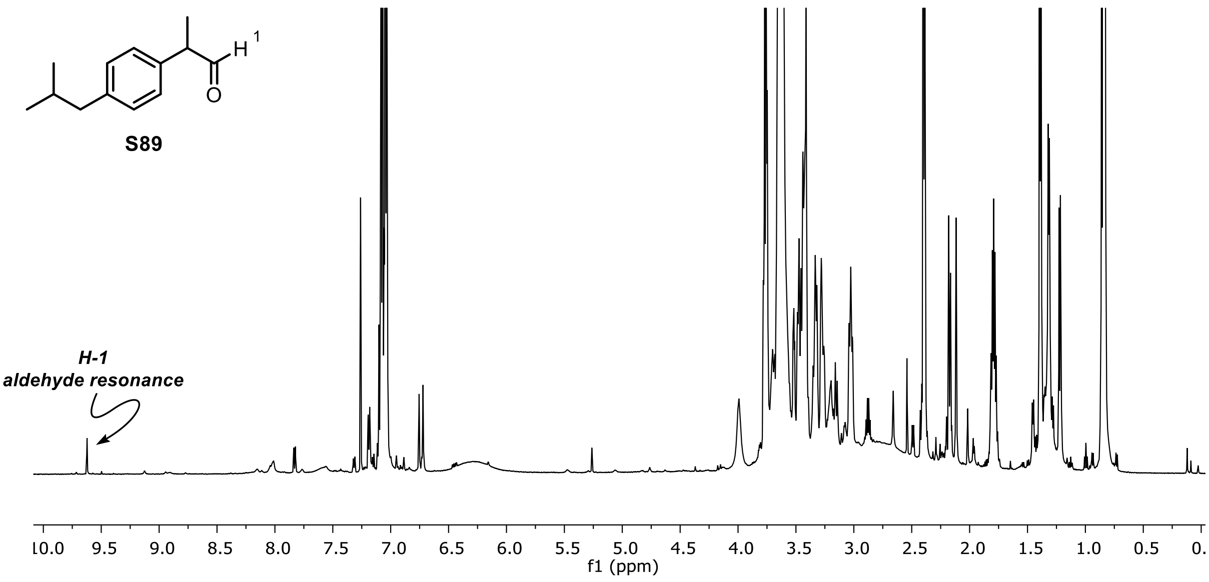

Figure S40: ^{1}H NMR analysis of the reaction mixture of the Cu(I)-catalyzed reduction of ibuprofen derivative **S88** with H_2 (600 MHz, $CDCl_3$).

5.4 Correlation of conversion to electronic and kinetic parameters of amides

In our efforts to explain the source of the site-selectivity observed in our catalytic protocol, we correlated the conversion of different benzamides with experimental data, which could indicate the influence of an electronic effect. We chose those experimental data to be, the NMR chemical shifts of the C=O bond in ^{13}C NMR and the wavenumber of the C=O group via IR Spectroscopy. From both diagrams there is no linear correlation between the conversion of the Cu(I)-catalyzed reduction of amide with H_2 and the electronic parameters of the carbonyl group in a variety of benzamides (Figure S41).

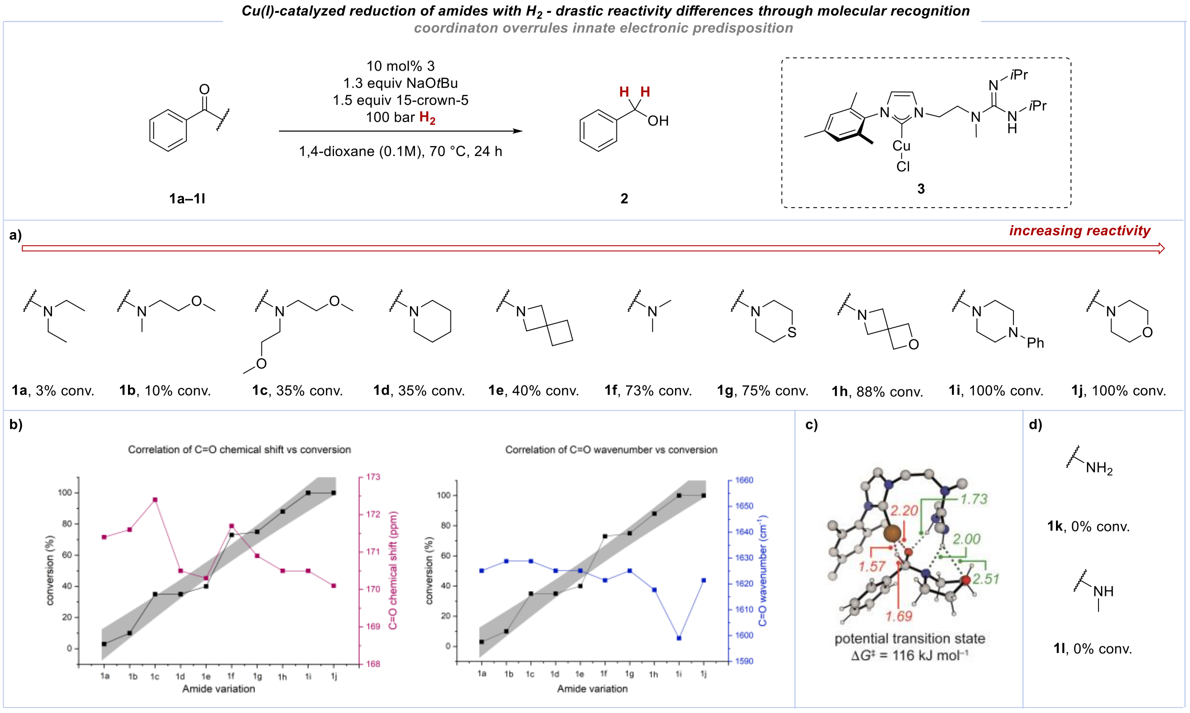

Figure S41: Correlation of conversion of different benzamides with chemical shift in ^{13}C NMR of C=O bond and C=O bond wavenumber in IR.

Moreover, we investigated the correlation of the conversion not only with the electronic parameters of the amides, but also with the kinetic parameters of the nucleophiles.[60,61] Investigating Mayr's database of reactivity parameters,[62] we found four amines **1a, 1c, 1d** and **1j** that were relevant for our study. Thus, the conversion of the catalytic reaction was correlated first with the *N* parameter of the different amines (all measured in MeCN as solvent, Figure S42).[63] Furthermore, in order to expand our investigation, we also utilized the literature known rate constants of the reaction between the amines and $jul_2CH^+BF_4^-$ (1-azatricyclo[7.3.1.0^{5,13}]trideca-5(13),6,8-triene;7-ethyl-1-azatricyclo[7.3.1.0^{5,13}]trideca-5(13),6,8-triene tetrafluoroborate salt) as the electrophile (Figure S42).[63] From both diagrams there is no linear correlation between the conversion of the Cu(I)-catalyzed reduction of amide with H_2 and the kinetic parameters of the amines.

From these experiments, we conclude that the presence of the site-selectivity relies on more sophisticated interaction rather than on simple electronic or kinetic parameters of the substrates.

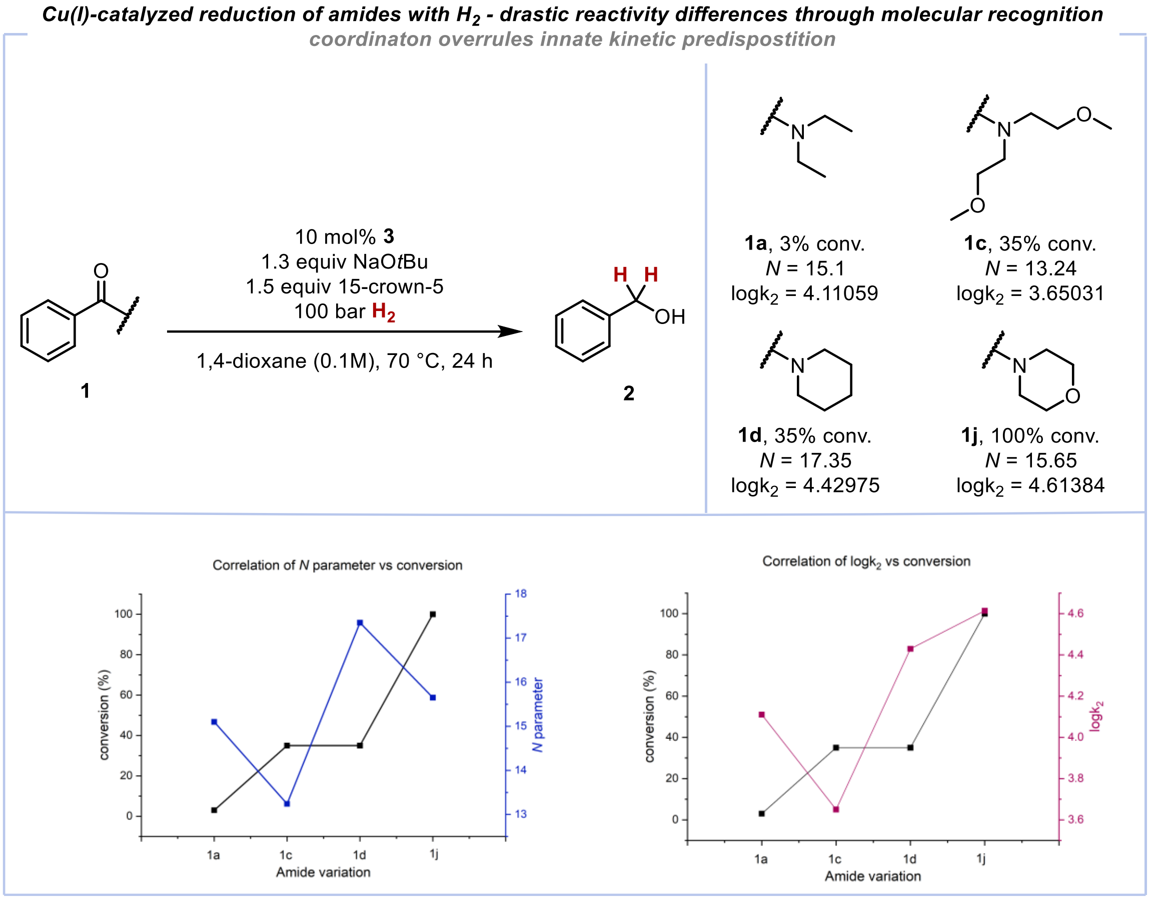

[a]All reactions were performed with 0.1 mmol of the corresponding benzamide **1**. [b]Conversion was determined by GC and GC/MS analysis and/or ^1H NMR analysis.

Figure S42: Correlation of conversion of different benzamides with kinetic parameters of the corresponding amine nucleophiles[a,b].

6 Computational methods

The computational investigations were performed on a slightly truncated ligand system, where the two isopropyl groups of the guanidine substructure were replaced by methyl groups. The conformational space for the intermediately formed copper hydride complex, the free amide **1j**, and the corresponding transition state was explored using the meta-dynamics package Conformer Rotamer Ensemble Sampling Tool (CREST).[64] The default parameters were used in combination with the GFN-FF force-field method.[65] The obtained conformers were subsequently optimized with the meta-hybrid M06-L functional,[66] the double-□ 6-31+G(d,p) basis set for all atoms except Cu, and the SDD pseudopotential for Cu.[67] The superfine grid was used for the numerical integration of the density. Vibrational analysis verified that each structure was a minimum or transition state. Following the intrinsic reaction coordinates (IRC) confirmed that all transition states connected the corresponding minima on the potential energy surface. Thermal corrections were calculated from unscaled harmonic vibrational frequencies at the same level of theory and refer to a standard state of 298.15 K and 1 mol L^{-1}. Entropic contributions to free energies were obtained from partition functions evaluated with Grimme's quasi-harmonic approximation.[68] This method employs the free-rotor approximation for all frequencies below 100 cm^{-1}, the rigid-rotor-harmonic-oscillator(RRHO) approximation for all frequencies above 100 cm^{-1}, and a damping function to interpolate between the two expressions. Energies were subsequently calculated with single-point calculations employing the DSD-BLYP functional,[69] Grimme's D3 correction with Becke-Johnson damping,[70] extrapolation to the basis set limit with def2-QZVPP basis set,[71–73] and the SMD solvation model for 1,4-dioxane.[74] All calculations involving M06L were performed with Gaussian 16,[75] while all DSD-BLYP calculations were performed with ORCA 5.[76]

6.2 Cartesian Coordinates and Calculated Energies

Amide 2j

SCF energy:	–631.816985 hartree
Zero-point correction:	+0.225970 hartree
Enthalpy correction:	+0.238526 hartree
Free energy correction:	+0.187264 hartree
Grimme's Delta G correction:	+0.188847 hartree

Cartesian Coordinates

O	-0.20094	2.17687	-0.23719
C	-0.05154	0.95780	-0.31010
N	-1.09143	0.11272	-0.63871
C	-2.39967	0.70468	-0.90427
C	-3.30999	0.51929	0.29431
O	-3.42796	-0.85658	0.63531
C	-2.15966	-1.39994	0.94896
C	-1.19051	-1.28259	-0.21819
C	1.30134	0.35290	-0.11619
C	2.12997	0.90054	0.86824
C	3.42509	0.42356	1.04207
C	3.91576	-0.58491	0.21307
C	3.10614	-1.11462	-0.79079
C	1.80227	-0.65355	-0.95022
H	-2.83094	0.19847	-1.77763
H	-2.25450	1.76163	-1.13210
H	-4.32326	0.87113	0.08443
H	-2.91115	1.08494	1.15351
H	-2.32667	-2.44795	1.21322
H	-1.73521	-0.88030	1.82711
H	-1.55090	-1.88276	-1.06509
H	-0.20894	-1.65863	0.07378
H	1.74343	1.70949	1.48173
H	4.05760	0.84598	1.81778
H	4.93087	-0.95015	0.34181
H	3.49205	-1.88472	-1.45294
H	1.17373	-1.05741	-1.74094

Copper Hydride Catalyst

SCF energy:	–2616.574095 hartree
Zero-point correction:	+0.453978 hartree
Enthalpy correction:	+0.481758 hartree

Free energy correction: +0.396667 hartree

Grimme's Delta G correction: +0.401534 hartree

Cartesian Coordinates

C	0.63767	1.70493	2.00507
N	0.90977	0.62533	1.18181
C	-0.67646	1.62503	2.33758
N	-1.16657	0.48895	1.71347
C	-0.19729	-0.15050	0.99124
C	2.17786	0.35734	0.55643
C	2.83183	-0.85500	0.83860
C	2.71283	1.30723	-0.32606
C	4.04348	-1.09840	0.19371
C	3.92985	1.00629	-0.94363
C	4.61020	-0.18699	-0.70236
H	4.56433	-2.03029	0.40560
H	4.35471	1.73081	-1.63605
Cu	-0.43034	-1.68920	-0.13215
H	1.39990	2.41215	2.29285
H	-1.28182	2.24662	2.97980
C	-2.54153	0.02521	1.77208
C	-3.50350	0.86025	0.93477
H	-2.88804	0.04191	2.81044
H	-2.52535	-1.02043	1.44738
N	-3.16458	0.94780	-0.49251
H	-3.50134	1.89177	1.29877
H	-4.52247	0.48525	1.08684
C	-2.80827	-0.13649	-1.21689
C	-3.12487	2.29130	-1.06479
H	-3.04290	2.23104	-2.14911
H	-2.28516	2.87549	-0.67098
H	-4.05611	2.80861	-0.82008
N	-3.32191	-1.35193	-0.98349
N	-1.93422	-0.01913	-2.23463
C	-0.76323	0.84942	-2.21837

H	-0.66508	1.31244	-1.23569
H	-0.82767	1.62720	-2.98484
H	0.13233	0.24512	-2.38806
C	-4.61078	-1.63529	-0.38736
H	-4.99516	-2.55953	-0.81955
H	-4.55788	-1.76766	0.70071
H	-5.31379	-0.83364	-0.61885
H	-1.85049	-0.85555	-2.79805
H	-2.65586	-2.12311	-1.13247
H	-0.84283	-2.78422	-1.13430
C	2.27965	-1.86416	1.79924
H	3.07458	-2.51497	2.16910
H	1.79277	-1.39656	2.65986
H	1.52875	-2.50982	1.32333
C	2.04676	2.62030	-0.61790
H	0.95975	2.58427	-0.50199
H	2.40537	3.41294	0.04904
H	2.26905	2.94688	-1.63707
C	5.91531	-0.48577	-1.37162
H	6.20054	0.30134	-2.07257
H	6.72187	-0.58721	-0.63865
H	5.87328	-1.42946	-1.92393

Reactant Complex

SCF energy:	–3248.411924 hartree
Zero-point correction:	+0.680748 hartree
Enthalpy correction:	+0.722471 hartree
Free energy correction:	+0.602332 hartree
Grimme's Delta G correction:	+0.613430 hartree

Cartesian Coordinates

C	-3.23302	-2.82753	1.07376
N	-2.93286	-1.57851	0.55834

C	-2.06910	-3.52783	1.10234
N	-1.09403	-2.67788	0.60968
C	-1.61034	-1.46178	0.25913
C	-3.88417	-0.52026	0.34494
C	-4.35534	0.19187	1.45433
C	-4.28320	-0.22947	-0.96691
C	-5.27141	1.21864	1.21951
C	-5.19971	0.80692	-1.14441
C	-5.70935	1.53878	-0.06770
H	-5.64562	1.78920	2.06750
H	-5.52352	1.04943	-2.15492
Cu	-0.71041	0.00641	-0.58762
H	-4.23491	-3.09989	1.36751
H	-1.85249	-4.53169	1.43377
C	0.30135	-3.00200	0.38374
C	0.58362	-3.24607	-1.10417
H	0.57098	-3.86224	1.00440
H	0.89847	-2.14186	0.71647
N	2.01281	-3.18642	-1.39206
H	0.04798	-2.47694	-1.67041
H	0.20509	-4.21869	-1.43429
C	2.61420	-1.96044	-1.43978
C	2.79017	-4.39797	-1.16559
H	2.81564	-4.71149	-0.11493
H	3.81302	-4.24535	-1.50997
H	2.34991	-5.20493	-1.75709
N	3.74529	-1.71081	-0.76328
N	2.09347	-0.98951	-2.18952
C	1.20140	-1.15817	-3.31690
H	1.21380	-2.19688	-3.65057
H	1.54248	-0.51948	-4.13389
H	0.17905	-0.85564	-3.05200
C	4.07713	-2.32560	0.51466
H	2.42913	-0.04669	-1.98610
H	4.10935	-0.76574	-0.91285
H	0.03853	1.12161	-1.31208
C	1.91340	-0.32170	2.91519

C	3.10124	-0.34630	3.64578
C	1.84941	0.37238	1.70860
C	2.98245	1.02500	1.21087
C	4.22891	0.31549	3.15889
C	4.17692	0.98516	1.93753
C	2.95955	1.65859	-0.14434
N	2.22439	2.76528	-0.33978
C	1.31670	3.35441	0.64501
C	2.06545	3.31128	-1.68926
C	1.39661	4.86886	0.57036
H	1.58510	3.00849	1.64495
H	0.29713	3.01105	0.41368
C	2.10589	4.82554	-1.63795
H	1.09704	2.96164	-2.07607
H	2.86427	2.91039	-2.31499
O	1.13852	5.33670	-0.73795
H	2.39295	5.20922	0.90185
H	0.64354	5.31890	1.22107
H	1.87440	5.24611	-2.61880
H	3.11117	5.16865	-1.33980
O	3.63472	1.13496	-1.06319
H	1.02663	-0.82767	3.29192
H	0.91774	0.41438	1.13646
H	3.14699	-0.87077	4.59573
H	5.15247	0.30827	3.73048
H	5.05898	1.48715	1.54734
C	-3.87425	-0.11752	2.83891
H	-2.78011	-0.16415	2.88271
H	-4.20840	0.64305	3.54672
H	-4.24368	-1.08377	3.20019
C	-3.73336	-0.98196	-2.13995
H	-2.69913	-0.67660	-2.35487
H	-3.71353	-2.06288	-1.96458
H	-4.32523	-0.79309	-3.03763
C	-6.71167	2.62831	-0.29177
H	-7.71241	2.21423	-0.45595
H	-6.77566	3.30138	0.56608

H	-6.46728	3.22484	-1.17483
H	4.55376	-1.57128	1.14213
H	4.76518	-3.16776	0.39922
H	3.17681	-2.66649	1.03397

Transition State

SCF energy:	–3248.366884 hartree
Zero-point correction:	+0.678785 hartree
Enthalpy correction:	+0.719557 hartree
Free energy correction:	+0.604798 hartree
Grimme's Delta G correction:	+0.613543 hartree
Imaginary Frequency:	515.9 *i*cm^{-1}

Cartesian Coordinates

C	-0.44345	-4.21876	0.01021
N	-0.72349	-2.87905	-0.18383
C	0.86245	-4.39238	-0.31946
N	1.34111	-3.15056	-0.71264
C	0.36910	-2.19883	-0.63014
C	-1.98760	-2.23375	0.07086
C	-2.32474	-1.92921	1.39608
C	-2.80686	-1.89505	-1.01443
C	-3.55280	-1.30970	1.62317
C	-4.02054	-1.26641	-0.73053
C	-4.42020	-0.98268	0.57734
H	-3.83256	-1.06283	2.64604
H	-4.67151	-0.99198	-1.55887
Cu	0.26289	-0.31888	-0.79910
H	-1.19359	-4.91877	0.34441
H	1.47766	-5.27897	-0.33519
C	2.71184	-2.90833	-1.13640
C	3.68709	-2.62487	0.02343

H	2.69171	-2.06439	-1.83445
H	3.03801	-3.78855	-1.69772
N	4.08854	-1.22159	0.14503
H	4.61273	-3.18568	-0.12643
H	3.25368	-2.99408	0.95713
C	3.17797	-0.26567	0.49097
C	5.34452	-0.85544	-0.50118
H	6.14207	-1.47794	-0.08698
H	5.57945	0.18606	-0.28411
H	5.32500	-0.99558	-1.58872
N	2.36774	-0.43944	1.53977
N	3.10667	0.89031	-0.18130
C	3.38277	1.04389	-1.60136
H	2.58856	1.65869	-2.02906
H	3.36209	0.07884	-2.11305
H	4.34409	1.53485	-1.77956
C	2.58103	-1.36537	2.62774
H	2.23780	-0.90253	3.55506
H	2.02082	-2.30099	2.49253
H	3.64334	-1.59645	2.72739
H	2.45725	1.60437	0.17951
H	1.47892	0.09863	1.51864
H	0.23683	1.12505	-1.41433
C	2.31209	3.91094	1.49627
O	2.84246	4.08204	0.18554
C	0.88274	3.35304	1.44285
N	0.74987	2.64585	0.16446
C	1.85763	4.63061	-0.67604
C	0.73784	3.60763	-0.93246
C	-0.23449	1.58220	0.14632
C	-1.65997	1.95138	-0.19832
C	-2.53572	2.14921	0.87660
C	-3.44085	2.54027	-1.72851
C	-3.84669	2.55563	0.64810
C	-4.30325	2.75186	-0.65458
O	-0.08587	0.72453	1.10814
C	-1.36967	-2.19506	2.51696

H	-1.81491	-1.94067	3.48053
H	-1.04610	-3.24060	2.56162
H	-0.47111	-1.57914	2.39041
C	-2.38981	-2.16371	-2.42737
H	-2.00758	-3.18095	-2.56047
H	-3.22607	-2.02232	-3.11448
H	-1.58596	-1.48297	-2.73777
C	-5.75495	-0.36387	0.85313
H	-6.53092	-1.13231	0.94274
H	-5.75267	0.20049	1.78942
H	-6.06079	0.30968	0.04806
H	-2.17880	1.96744	1.88759
H	-4.51798	2.70859	1.48908
H	0.69966	2.65160	2.26012
H	0.13237	4.15811	1.52174
H	2.36853	4.90508	-1.60138
H	1.45042	5.55453	-0.23709
H	0.89121	3.05274	-1.86794
H	-0.23563	4.11965	-1.02243
H	2.33793	4.86348	2.04642
H	2.99520	3.21414	1.99517
H	-5.32956	3.06160	-0.83187
C	-2.12439	2.14573	-1.49994
H	-3.79305	2.67918	-2.74676
H	-1.45275	1.96149	-2.33685

6.3 Preliminary Investigations into Catalyst 6

To get a preliminary understanding of the behavior of catalyst **6**, we have optimized the proposed copper-hydride intermediate obtained from **6**. The lowest-energy conformer is shown below and reveals that the Cu–H motive and the guanidinium substructure are fairly close in space. We have furthermore performed an optimization with loose convergence criteria for the corresponding amide complex. Again, the crude optimization shows that the reduction through hydride transfer should be feasible.

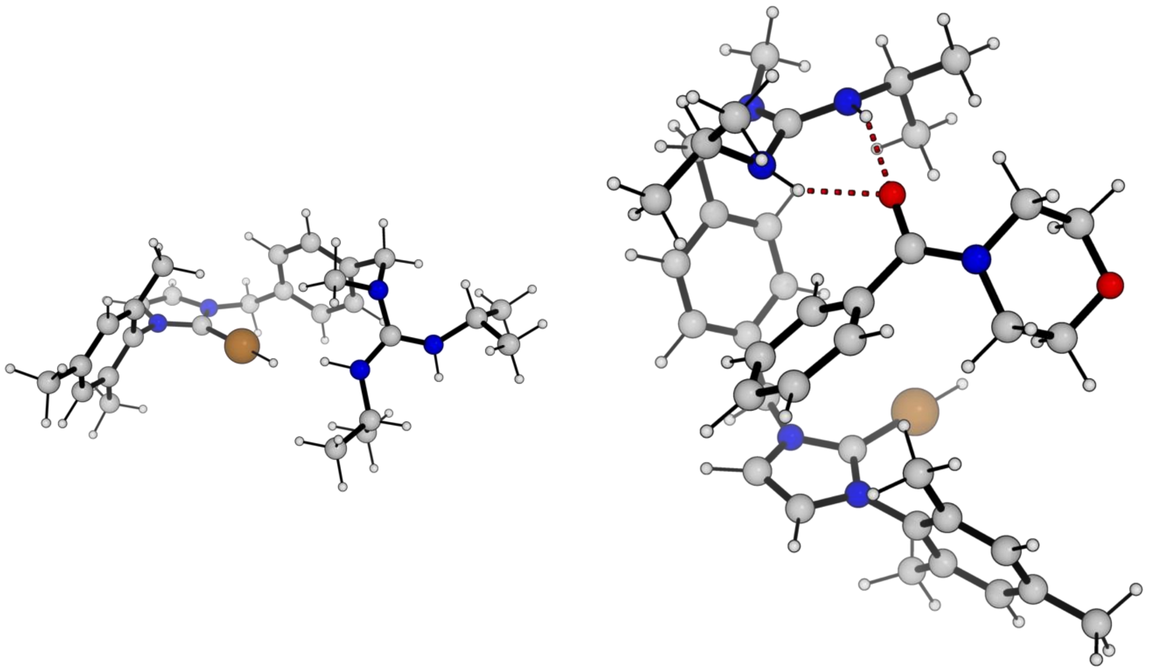

Figure S43: Lowest-energy structure for the copper-hydride intermediate derived from catalyst **6** (left) and a representative amide complex (right).

6.4 Guanidinium-Amide Interactions

We also looked into the potential hydrogen-bond interactions between a model tetramethyl guanidinium cation and the amide **2j**. The following structures and relative energies show representative low-energy conformers for different non-covalent interactions.

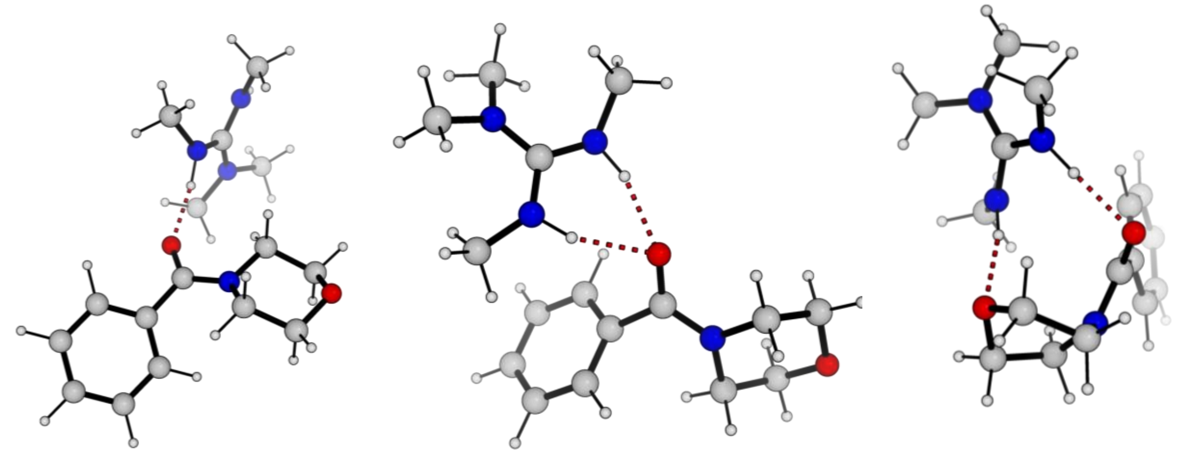

+5 kJ mol^{-1} 0 kJ mol^{-1} +34 kJ mol^{-1}

Figure S44: Computed structures and relative free energies for different interactions between the tetramethyl guanidinium ion and the amide **2j**.

7 NMR titration experiments

In order to gain more insight for the presence of a possible hydrogen bonding interaction between the protonated catalyst and privileged and non-privileged amide substrates, NMR titration experiments were carried out. For this, the copper(I) chloride precatalyst **3** was employed in the absence of H_2 but instead protonated by PTSA in order to deliver the proton equivalent to form the proposed guanidinium ion. After this, the respective amide was added. To follow the binding event, the *N*-methyl group of the guanidine was employed as a reporter unit, as this methyl group is not directly affected by the binding event, yet is directly influenced by the changing of the electron density of the guanidinium ion.

Following literature procedures and techniques used in the field of supramolecular chemistry,[77,78] a J. Young NMR tube was charged with [CuGua] **3** (10 mg, 21 μmol, 1.0 equiv), *p*-toluolsulfonic acid (PTSA, 1.0 mg, 5.8 μmol, 27 mol% for morpholine benzamide **1j** and 1.5 mg, 8.7 μmol, 41 mol% for piperidine benzamide **1d**) in CD_2Cl_2 (0.8 mL) which was used as the host and in which various amounts of morpholine **1j** or piperidine **1d** benzamides were added as guest (Figure S45). (It should be noted that a larger amount of PTSA led to decomposition of **3** due to elimination of the guanidinium.)

Expectably, the binding constants are small, which is why in neither case a plateau was reached even after addition of 14 equivalents of the respective amide.

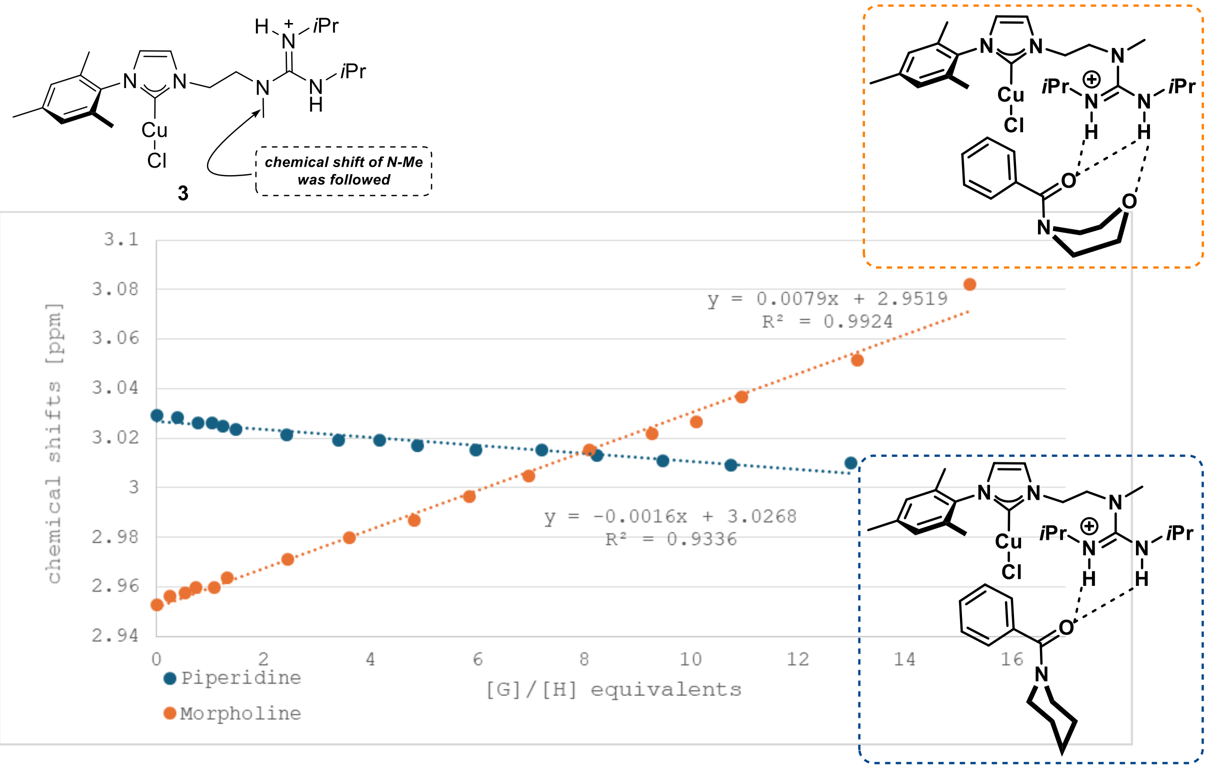

Figure S45: NMR titration experiments.

As can be seen from Figure S45, the chemical shift of the methyl probe is changing in a linear fashion towards lower field when the “privileged” morpholine amide is employed. Such a shift is in line with a generally lower electron density within the guanidinium moiety as a result of an additional hydrogen bond (orange frame). In stark contrast, the non-privileged yet structurally closely related piperidine-derived amide leads to a significantly smaller shift, if it all in an orthogonal fashion to the privileged amide leading to higher field of the methyl probe. This could be explained by the expected double hydrogen bonding of the guanidinium ion to the amide.

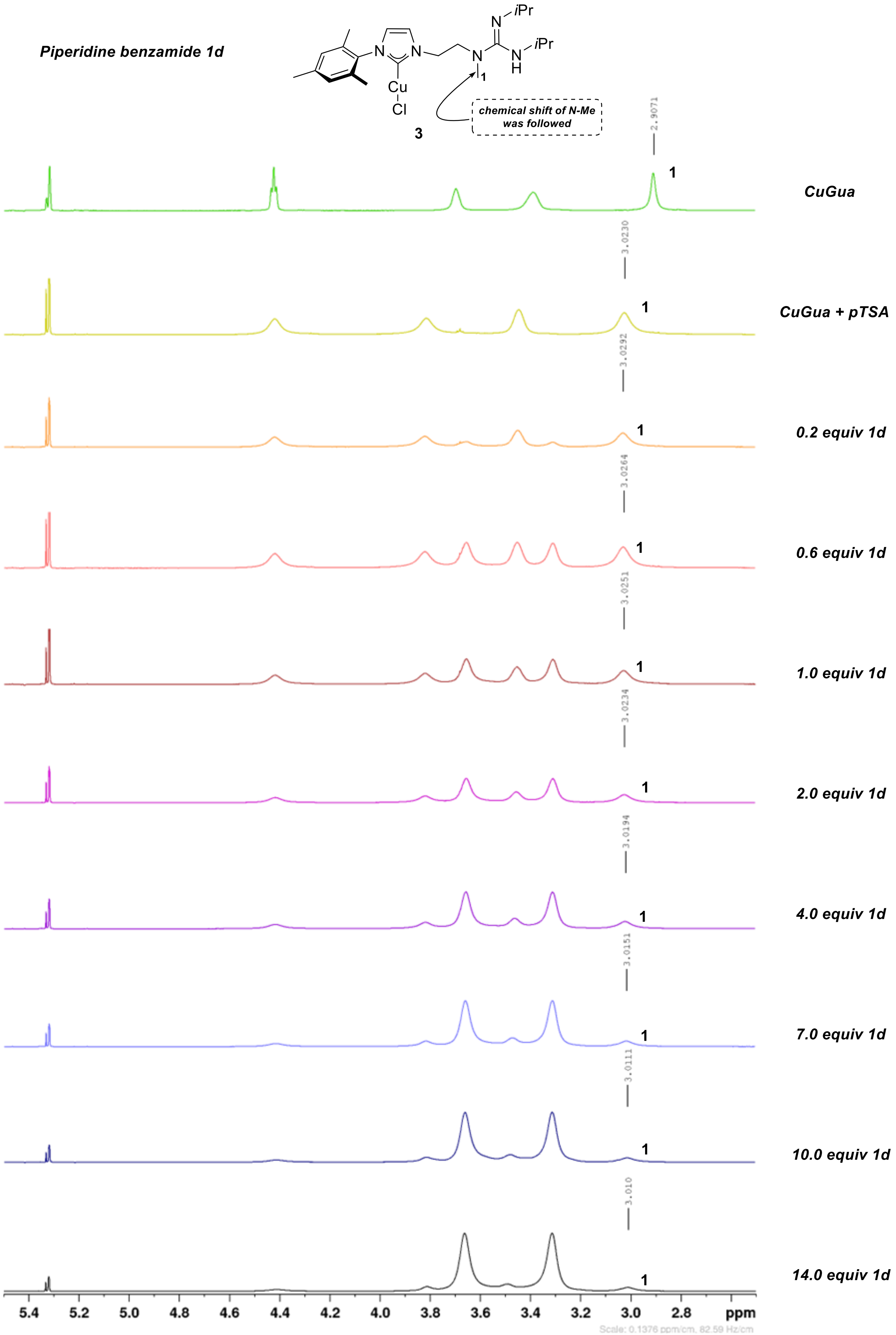

Figure S46: NMR titration of pipreidine benzamide **1d**.

Morpholine benzamide 1j

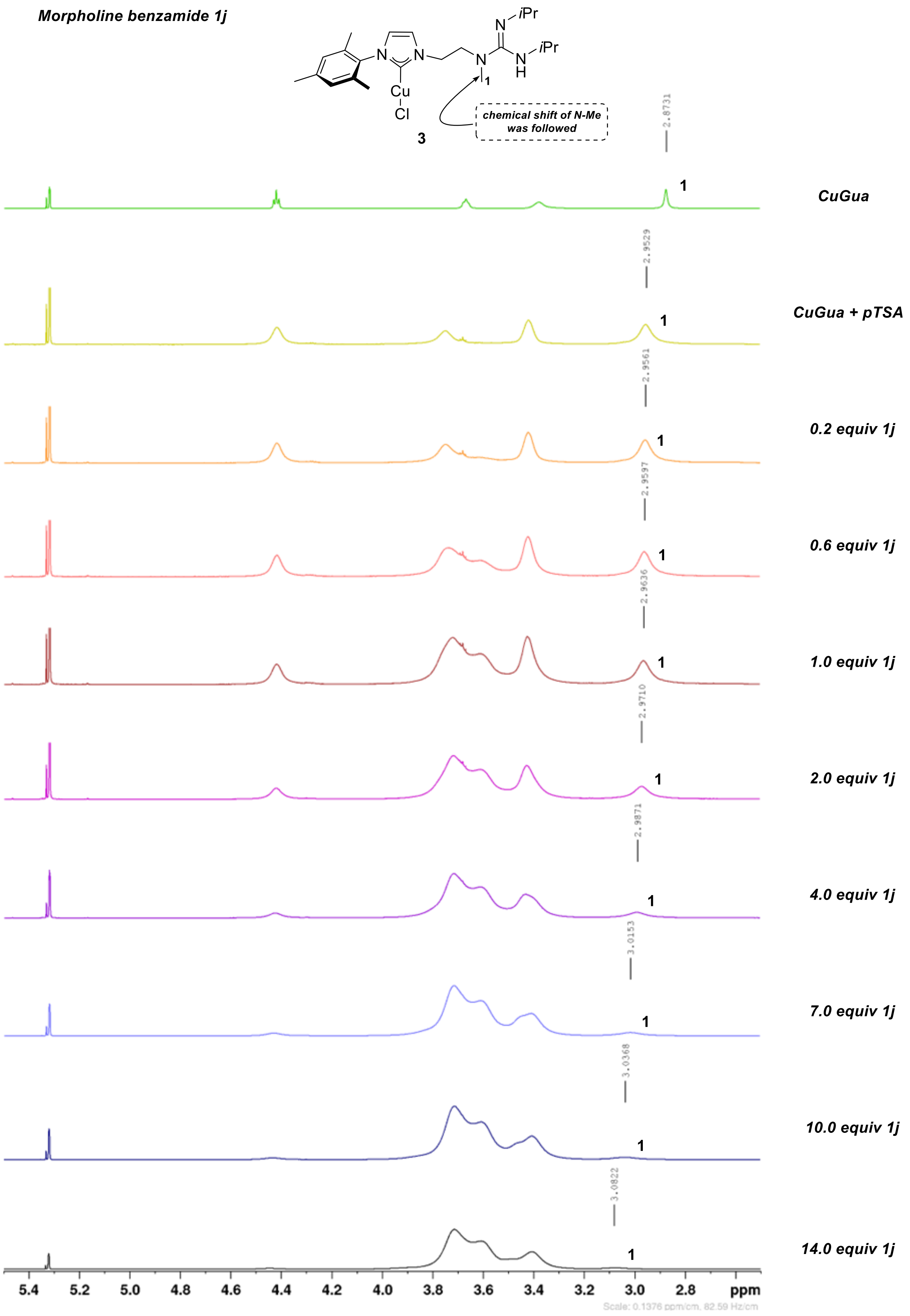

Figure S47: NMR titration of morpholine benzamide **1j**.

8 References

[1] G. R. Fulmer, A. J. M. Miller, N. H. Sherden, H. E. Gottlieb, A. Nudelman, B. M. Stoltz, J. E. Bercaw, K. I. Goldberg, *Organometallics* **2010**, *29*, 2176–2179.
[2] H. E. Gottlieb, V. Kotlyar, A. Nudelman, *J. Org. Chem.* **1997**, *62*, 7512–7515.
[3] B. M. Zimmermann, T. T. Ngoc, D.-I. Tzaras, T. Kaicharla, J. F. Teichert, *J. Am. Chem. Soc.* **2021**, *143*, 16865–16873.
[4] R. Cacciapaglia, S. Di Stefano, L. Mandolini, *J. Am. Chem. Soc.* **2005**, *127*, 13666–13671.
[5] O. Santoro, A. Collado, A. M. Z. Slawin, S. P. Nolan, C. S. J. Cazin, *Chem. Commun.* **2013**, *49*, 10483–10485.
[6] A. Takemiya, J. F. Hartwig, *J. Am. Chem. Soc.* **2006**, *128*, 14800–14801.
[7] S. Ghinato, D. Territo, A. Maranzana, V. Capriati, M. Blangetti, C. Prandi, *Chem. Eur. J.* **2021**, *27*, 2868–2874.
[8] Y. Zhou, P. Chen, X. Lv, J. Niu, Y. Wang, M. Lei, L. Hu, *Tetrahedron Lett.* **2017**, *58*, 2232–2235.
[9] G. Meng, M. Szostak, *Org. Biomol. Chem.* **2016**, *14*, 5690–5707.
[10] M. Mart, I. Karakaya, J. Jurczak, *ChemistrySelect* **2022**, *7*, e202202436.
[11] T. Fujii, S. Gallarati, C. Corminboeuf, Q. Wang, J. Zhu, *J. Am. Chem. Soc.* **2022**, *144*, 8920–8926.
[12] N. A. Sitte, M. Bursch, S. Grimme, J. Paradies, *J. Am. Chem. Soc.* **2019**, *141*, 159–162.
[13] J.-Y. Gu, W. Zhang, S. R. Jackson, Y.-H. He, Z. Guan, *Chem. Commun.* **2020**, *56*, 13441–13444.
[14] S. Das, D. Addis, S. Zhou, K. Junge, M. Beller, *J. Am. Chem. Soc.* **2010**, *132*, 1770–1771.
[15] Y. Ma, C. E. Stivala, A. M. Wright, T. Hayton, J. Liang, I. Keresztes, E. Lobkovsky, D. B. Collum, A. Zakarian, *J. Am. Chem. Soc.* **2013**, *135*, 16853–16864.
[16] S. Azeez, P. Sureshbabu, S. Sabiah, J. Kandasamy, *Org. Biomol. Chem.* **2022**, *20*, 2048–2053.
[17] G. Barbe, A. B. Charette, *J. Am. Chem. Soc.* **2008**, *130*, 18–19.
[18] M.-Z. Zhang, Q.-H. Guo, W.-B. Sheng, C.-C. Guo, *Adv. Synth. Catal.* **2015**, *357*, 2855–2861.
[19] M. Martinelli, C. Giorgiutti, T. Fessard, Q. Lefebvre, *Org. Biomol. Chem.* **2023**, *21*, 9230–9235.
[20] G. Pandey, S. Koley, R. Talukdar, P. K. Sahani, *Org. Lett.* **2018**, *20*, 5861–5865.
[21] T. T. Dang, Y. Zhu, J. S. Y. Ngiam, S. C. Ghosh, A. Chen, A. M. Seayad, *ACS Catal.* **2013**, *3*, 1406–1410.
[22] Z. Wu, K. L. Hull, *Chem. Sci.* **2016**, *7*, 969–975.
[23] L. Yang, K. Semba, Y. Nakao, *Angew. Chem. Int. Ed.* **2017**, *56*, 4853–4857.
[24] T. Ben Halima, J. Masson-Makdissi, S. G. Newman, *Angew. Chem. Int. Ed.* **2018**, *57*, 12925–12929.
[25] P.-F. Koh, T.-P. Loh, *Green Chem.* **2015**, *17*, 3746–3750.
[26] D. Chakraborty, R. R. Gowda, P. Malik, *Tetrahedron Lett.* **2009**, *50*, 6553–6556.
[27] M. K. Gupta, Z. Li, T. S. Snowden, *Org. Lett.* **2014**, *16*, 1602–1605.
[28] C. Henry, D. Bolien, B. Ibanescu, S. Bloodworth, D. C. Harrowven, X. Zhang, A. Craven, H. F. Sneddon, R. J. Whitby, *Eur. J. Org. Chem.* **2015**, 1491–1499.
[29] W. Li, X.-F. Wu, *Org. Lett.* **2015**, *17*, 1910–1913.
[30] Y. Wang, Z. Huang, Z. Huang, *Nat Catal* **2019**, *2*, 529–536.
[31] C.-P. Xu, Z.-H. Xiao, B.-Q. Zhuo, Y.-H. Wang, P.-Q. Huang, *Chem. Commun.* **2010**, *46*, 7834.
[32] F. La Spisa, A. Feo, R. Mossetti, G. C. Tron, *Org. Lett.* **2012**, *14*, 6044–6047.
[33] L. L. McCoy, D. Mal, *J. Org. Chem.* **1984**, *49*, 939–942.
[34] P. Wójcik, A. M. Trzeciak, *Appl. Catal. A* **2018**, *560*, 73–83.
[35] W. Shen, A. Kunzer, *Org. Lett.* **2002**, *4*, 1315–1317.
[36] J. Reiter, P. Trinka, F. L. Bartha, L. Pongó, B. Volk, G. Simig, *Org. Proc. Res. Dev.* **2012**, *16*, 1279–1282.
[37] P. Wang, S. M. Batt, B. Wang, L. Fu, R. Qin, Y. Lu, G. Li, G. S. Besra, H. Huang, *J. Med. Chem.* **2021**, *64*, 6241–6261.

[38] N. Iwamoto, N. Oka, T. Sato, T. Wada, *Angew. Chem. Int. Ed.* **2009**, *48*, 496–499.
[39] M. Graupe, J. T. Palmer, J. W. Patterson, S. D. Pickett, D. J. Aldous, S. Thurairatnam, A. P. Timm, F. Halley, J. Lai, J. Link, J. Li, *Novel Compounds and Compositions as Cathepsin Inhibitors*, **2003**, US 2003/0105099 A l.
[40] W. P. Griffith, A. G. Shoair, M. Suriaatmaja, *Synth. Commun.* **2000**, *30*, 3091–3095.
[41] T. Jeffery, *Tetrahedron* **1996**, *52*, 10113–10130.
[42] D. J. Mason, Y. G. Timofeyenko, B. Jagadish, E. A. Mash, *Synth. Commun.* **2022**, *52*, 1825–1833.
[43] M. D. Wallace, N. F. Waraich, A. W. Debowski, M. G. Corral, A. Maxwell, J. S. Mylne, K. A. Stubbs, *Chem. Commun.* **2018**, *54*, 1869–1872.
[44] N. Aravindan, V. Vinayagam, M. Jeganmohan, *Org. Lett.* **2022**, *24*, 5260–5265.
[45] R. Cormier, W. N. Burda, L. Harrington, J. Edlinger, K. M. Kodigepalli, J. Thomas, R. Kapolka, G. Roma, B. E. Anderson, E. Turos, L. N. Shaw, *Bioorg. Med. Chem. Lett.* **2012**, *22*, 6513–6520.
[46] C. Desmarets, R. Schneider, Y. Fort, *J. Org. Chem.* **2002**, *67*, 3029–3036.
[47] M. Szostak, M. Spain, A. J. Eberhart, D. J. Procter, *J. Am. Chem. Soc.* **2014**, *136*, 2268–2271.
[48] T. Osako, K. Torii, S. Hirata, Y. Uozumi, *ACS Catal.* **2017**, *7*, 7371–7377.
[49] W. M. J. Ma, T. D. James, J. M. J. Williams, *Org. Lett.* **2013**, *15*, 4850–4853.
[50] G. Huang, B. Yin, *Adv. Synth. Catal.* **2019**, *361*, 5576–5586.
[51] C. Belger, B. Plietker, *Chem. Commun.* **2012**, *48*, 5419.
[52] D. Peng, M. Zhang, Z. Huang, *Chem. Eur. J.* **2015**, *21*, 14737–14741.
[53] A. Modak, T. Naveen, D. Maiti, *Chem. Commun.* **2013**, *49*, 252–254.
[54] S. Yamaguchi, H. Kondo, K. Uesugi, K. Sakoda, K. Jitsukawa, T. Mitsudome, T. Mizugaki, *ChemCatChem* **2021**, *13*, 1135–1139.
[55] S. L. Zultanski, J. Zhao, S. S. Stahl, *J. Am. Chem. Soc.* **2016**, *138*, 6416–6419.
[56] C. Sandford, L. R. Fries, T. E. Ball, S. D. Minteer, M. S. Sigman, *J. Am. Chem. Soc.* **2019**, *141*, 18877–18889.
[57] L. Ackermann, A. V. Lygin, N. Hofmann, *Angew. Chem. Int. Ed.* **2011**, *50*, 6379–6382.
[58] K. Kai, H. Fujii, R. Ikenaka, M. Akagawa, H. Hayashi, *Chem. Commun.* **2014**, *50*, 8586–8589.
[59] S. Okamoto, S. Tominaga, N. Saino, K. Kase, K. Shimoda, *J. Organomet. Chem.* **2005**, *690*, 6001–6007.
[60] C. D. Ritchie, R. J. Minasz, A. A. Kamego, M. Sawada, *J. Am. Chem. Soc.* **1977**, *99*, 3747–3753.
[61] L. A. P. Kane-Maguire, E. D. Honig, D. A. Sweigart, *Chem. Rev.* **1984**, *84*, 525–543.
[62] H. Mayr, M. Patz, *Angew. Chem. Int. Ed. Engl.* **1994**, *33*, 938–957.
[63] T. Kanzian, T. A. Nigst, A. Maier, S. Pichl, H. Mayr, *Eur. J. Org. Chem.* **2009**, *2009*, 6379–6385.
[64] S. Grimme, *J. Chem. Theory Comput.* **2019**, *15*, 2847–2862.
[65] S. Spicher, S. Grimme, *Angew. Chem. Int. Ed.* **2020**, *59*, 15665–15673.
[66] Y. Zhao, D. G. Truhlar, *J. Chem. Phys.* **2006**, *125*, 194101.
[67] A. Bergner, M. Dolg, W. Küchle, H. Stoll, H. Preuß, *Mol. Phys.* **1993**, *80*, 1431–1441.
[68] S. Grimme, *Chem. Eur. J.* **2012**, *18*, 9955–9964.
[69] S. Kozuch, D. Gruzman, J. M. L. Martin, *J. Phys. Chem. C* **2010**, *114*, 20801–20808.
[70] S. Grimme, S. Ehrlich, L. Goerigk, *J. Comput. Chem.* **2011**, *32*, 1456–1465.
[71] F. Weigend, *J. Comput. Chem.* **2008**, *29*, 167–175.
[72] F. Weigend, R. Ahlrichs, *Phys. Chem. Chem. Phys.* **2005**, *7*, 3297.
[73] A. Hellweg, C. Hättig, S. Höfener, W. Klopper, *Theor. Chem. Acc.* **2007**, *117*, 587–597.
[74] A. V. Marenich, C. J. Cramer, D. G. Truhlar, *J. Phys. Chem. B* **2009**, *113*, 6378–6396.
[75] Gaussian 16, Revision C.01, M. J. Frisch, G. W. Trucks, H. B. Schlegel, G. E. Scuseria, M. A. Robb, J. R. Cheeseman, G. Scalmani, V. Barone, G. A. Petersson, H. Nakatsuji, X. Li, M. Caricato, A. V. Marenich, J. Bloino, B. G. Janesko, R. Gomperts, B. Mennucci, H. P. Hratchian, J. V. Ortiz, A. F. Izmaylov, J. L. Sonnenberg, D. Williams-Young, F. Ding, F. Lipparini, F. Egidi, J. Goings, B. Peng, A. Petrone, T. Henderson, D. Ranasinghe, V. G. Zakrzewski, J. Gao, N. Rega, G. Zheng, W. Liang, M. Hada, M. Ehara, K. Toyota, R.

Fukuda, J. Hasegawa, M. Ishida, T. Nakajima, Y. Honda, O. Kitao, H. Nakai, T. Vreven, K. Throssell, J. A. Montgomery, Jr., J. E. Peralta, F. Ogliaro, M. J. Bearpark, J. J. Heyd, E. N. Brothers, K. N. Kudin, V. N. Staroverov, T. A. Keith, R. Kobayashi, J. Normand, K. Raghavachari, A. P. Rendell, J. C. Burant, S. S. Iyengar, J. Tomasi, M. Cossi, J. M. Millam, M. Klene, C. Adamo, R. Cammi, J. W. Ochterski, R. L. Martin, K. Morokuma, O. Farkas, J. B. Foresman, and D. J. Fox, Gaussian, Inc., Wallingford CT, 2016., **n.d.**

[76] F. Neese, *Wiley Interdiscip. Rev. Comput. Mol. Sci.* **2022**, *12*, e1606.

[77] P. Thordarson, *Chem. Soc. Rev.* **2011**, *40*, 1305–1323.

[78] D. Brynn Hibbert, P. Thordarson, *Chem. Commun.* **2016**, *52*, 12792–12805.
