# Supplementary material for: Site-Selective Copper(I)-Catalyzed Hydrogenation of Amides
Source: J Am Chem Soc. 2025 Jan 3;147(2):1867–74. doi: 10.1021/jacs.4c14174 (PMC11744755; doi:10.1021/jacs.4c14174)
Supplement: Supplementary file 2 — ja4c14174_si_002.pdf [file ja4c14174_si_002.pdf]

Site-Selective Copper(I)-Catalyzed Hydrogenation of Amides

Dimitrios-Ioannis Tzaras, Mahadeb Gorai, Thomas Jacquemin, Thiemo Arndt, Birte M. Zimmermann, Martin Breugst, Johannes F. Teichert*

Institut für Chemie, Technische Universität Chemnitz, Straße der Nationen 62, 09111 Chemnitz, Germany

Supporting Information

1 *N,N*-Diethylbenzamide (1a)

^{1}H NMR

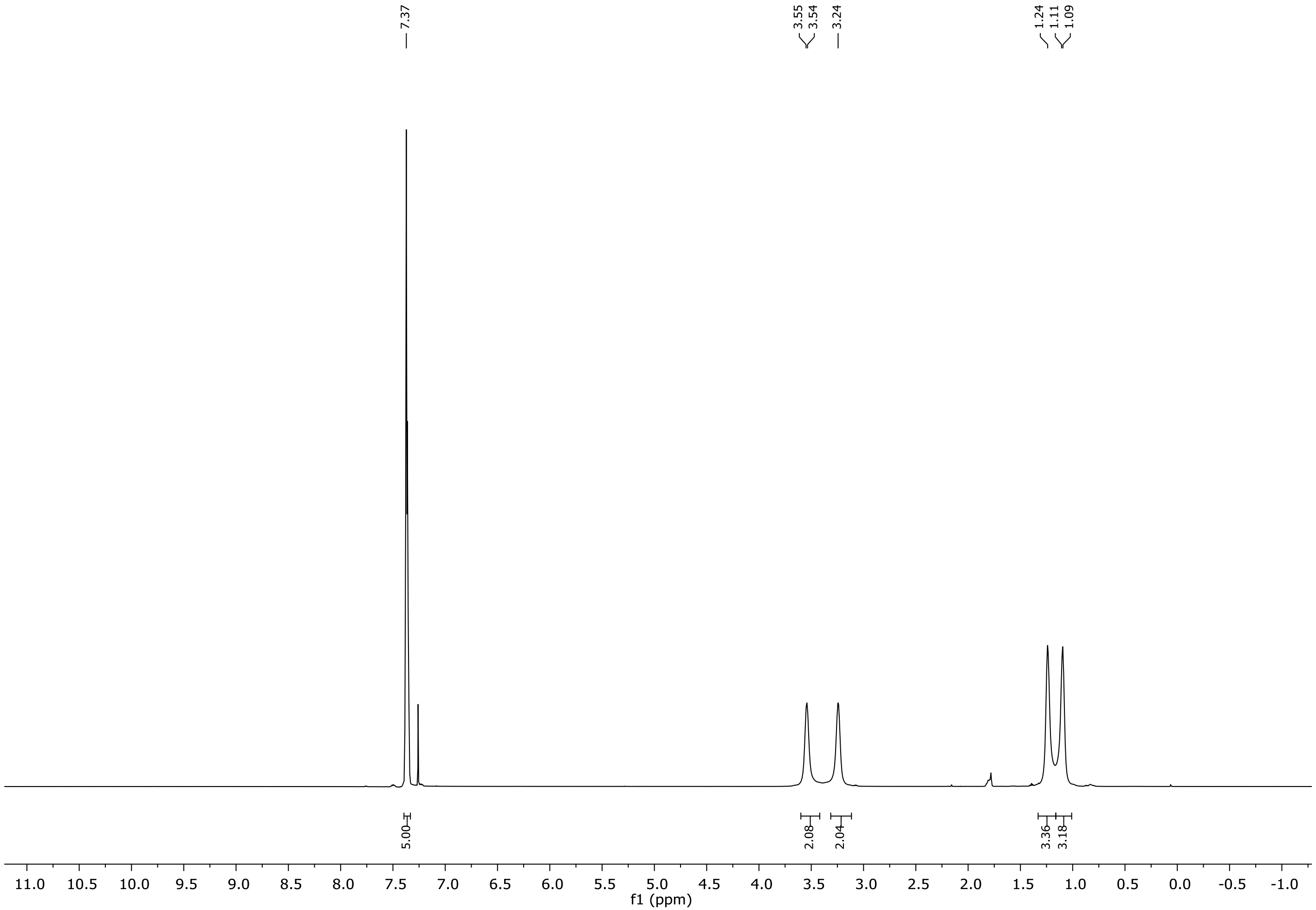

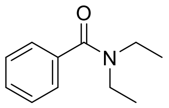

1a

^{13}C NMR

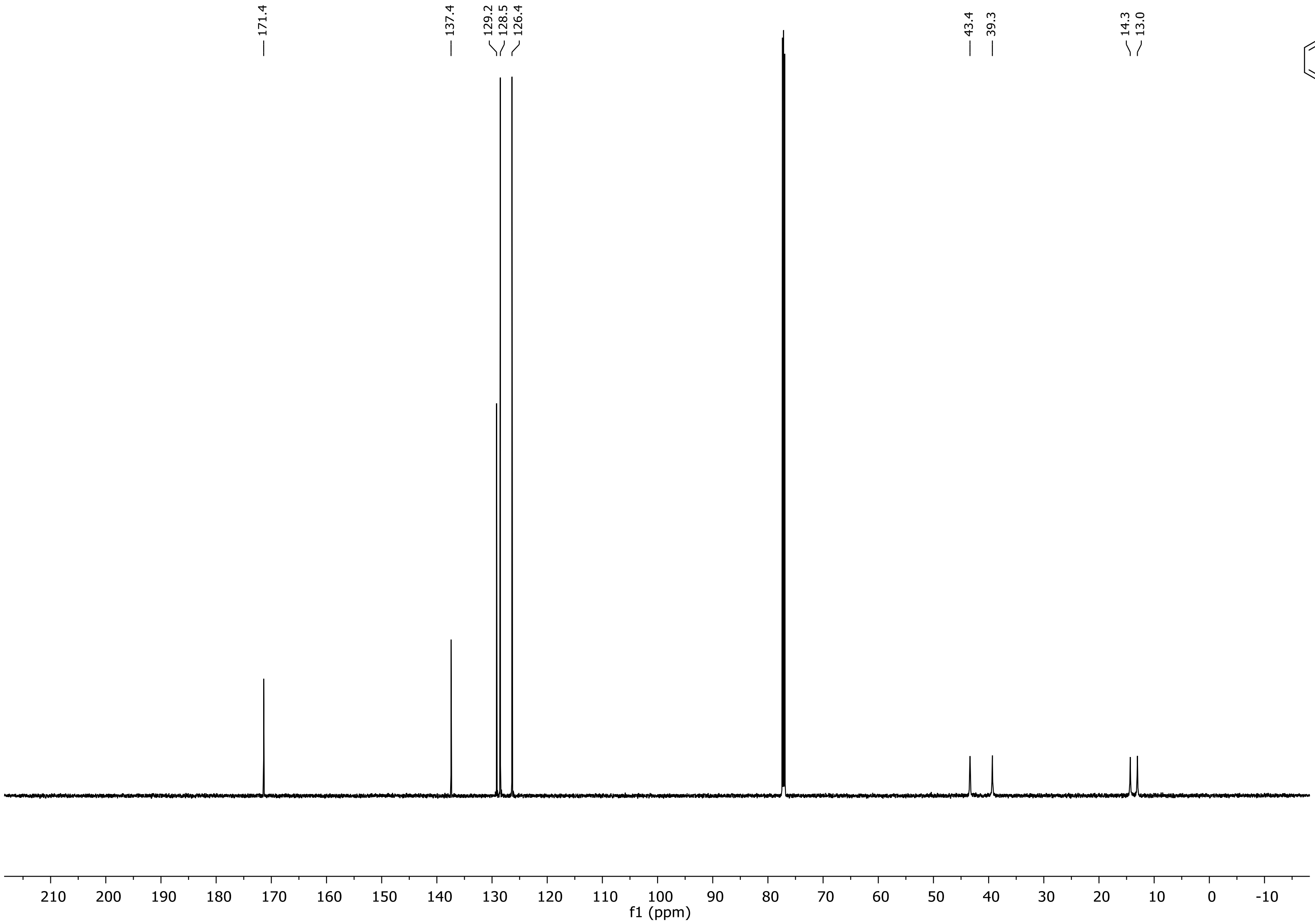
171.4
137.4
129.2
128.5
126.4
43.4
39.3
14.3
13.0
210 200 190 180 170 160 150 140 130 120 110 100 90 80 70 60 50 40 30 20 10 0 -10
f1 (ppm)

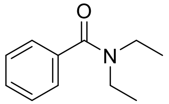
O
N

1a

^1H, ^1H COSY

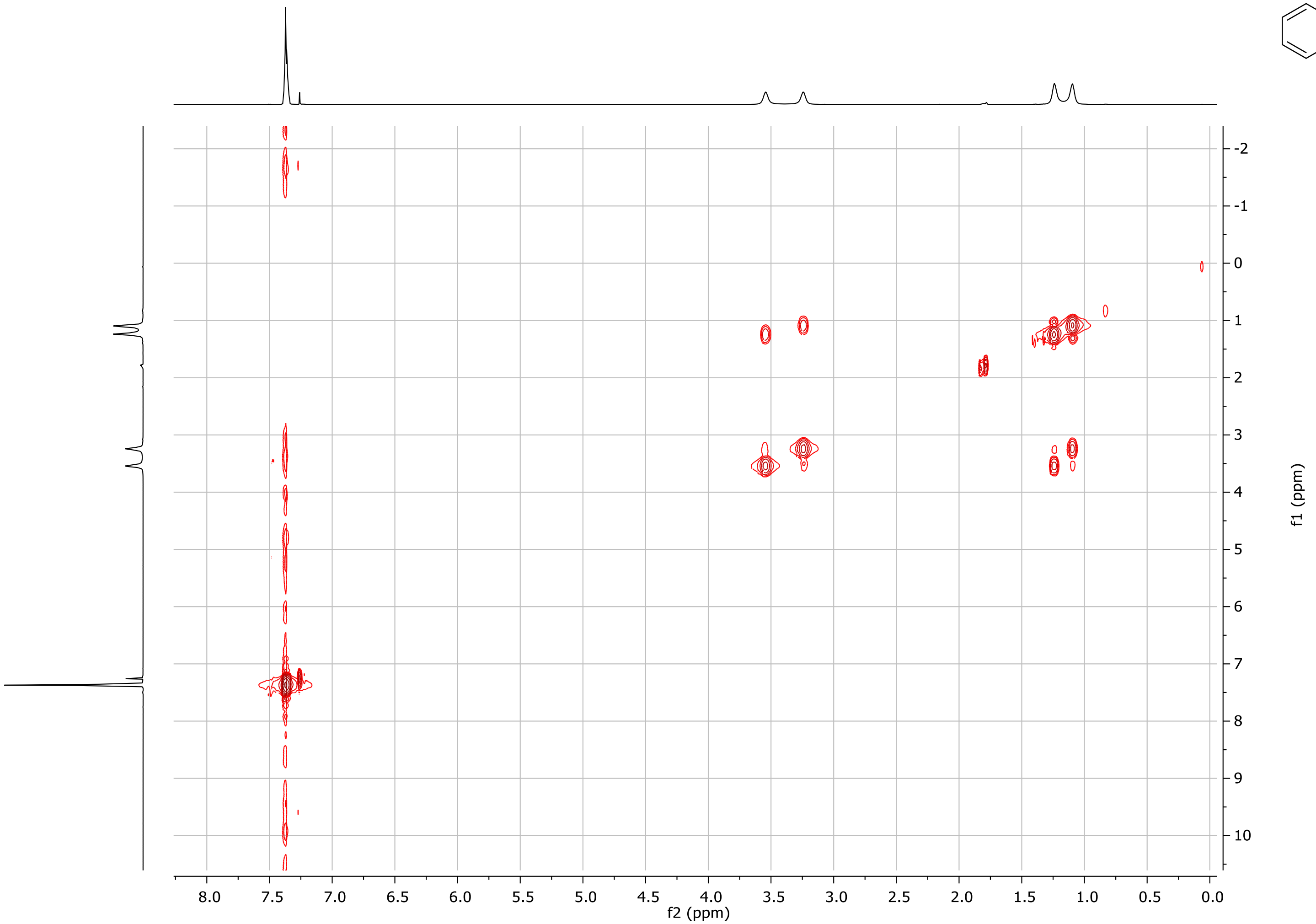

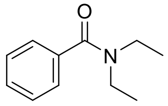

1a

^{1}H, ^{13}C HMBC

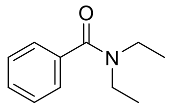

1a

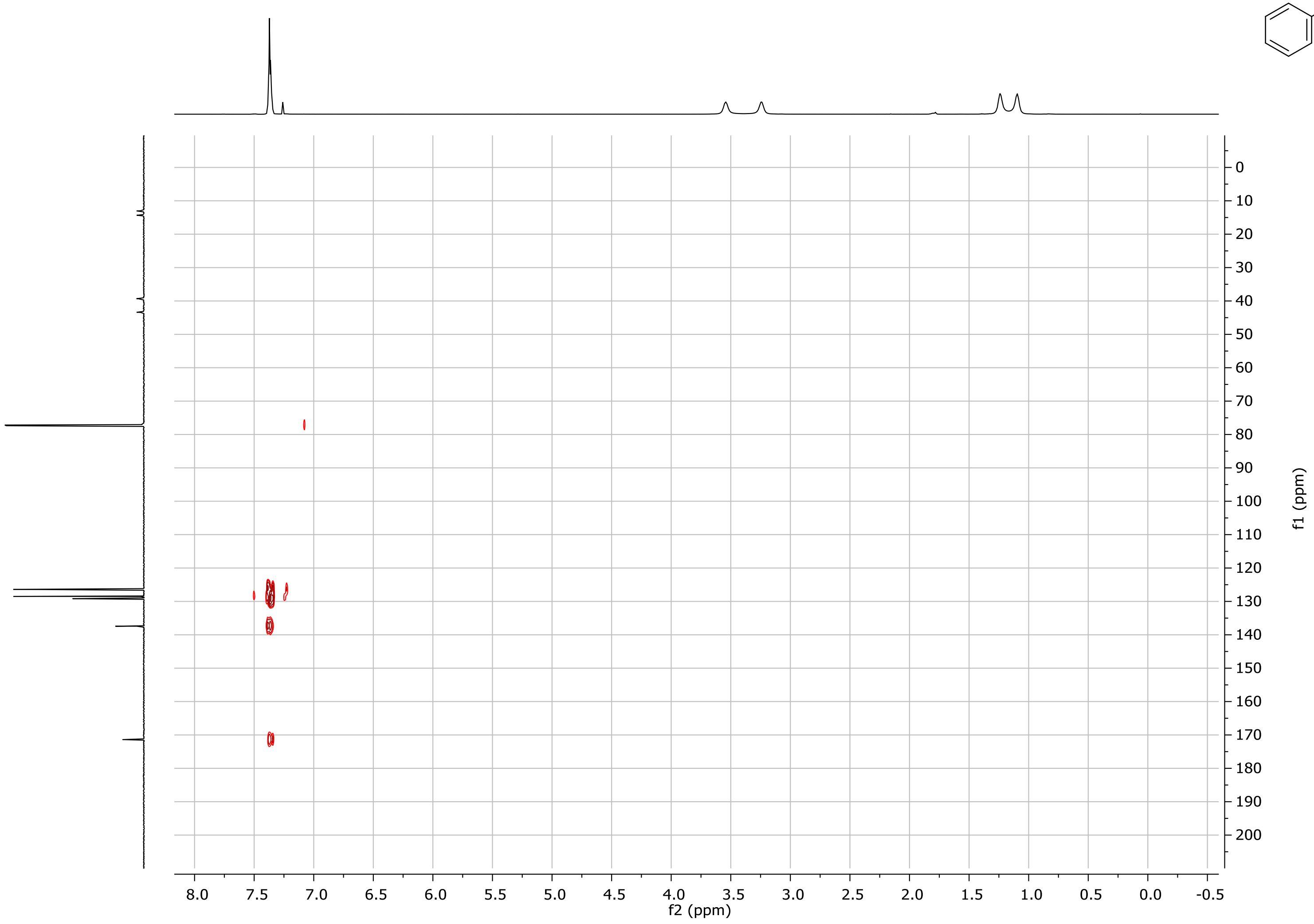

^{1}H, ^{13}C HSQC

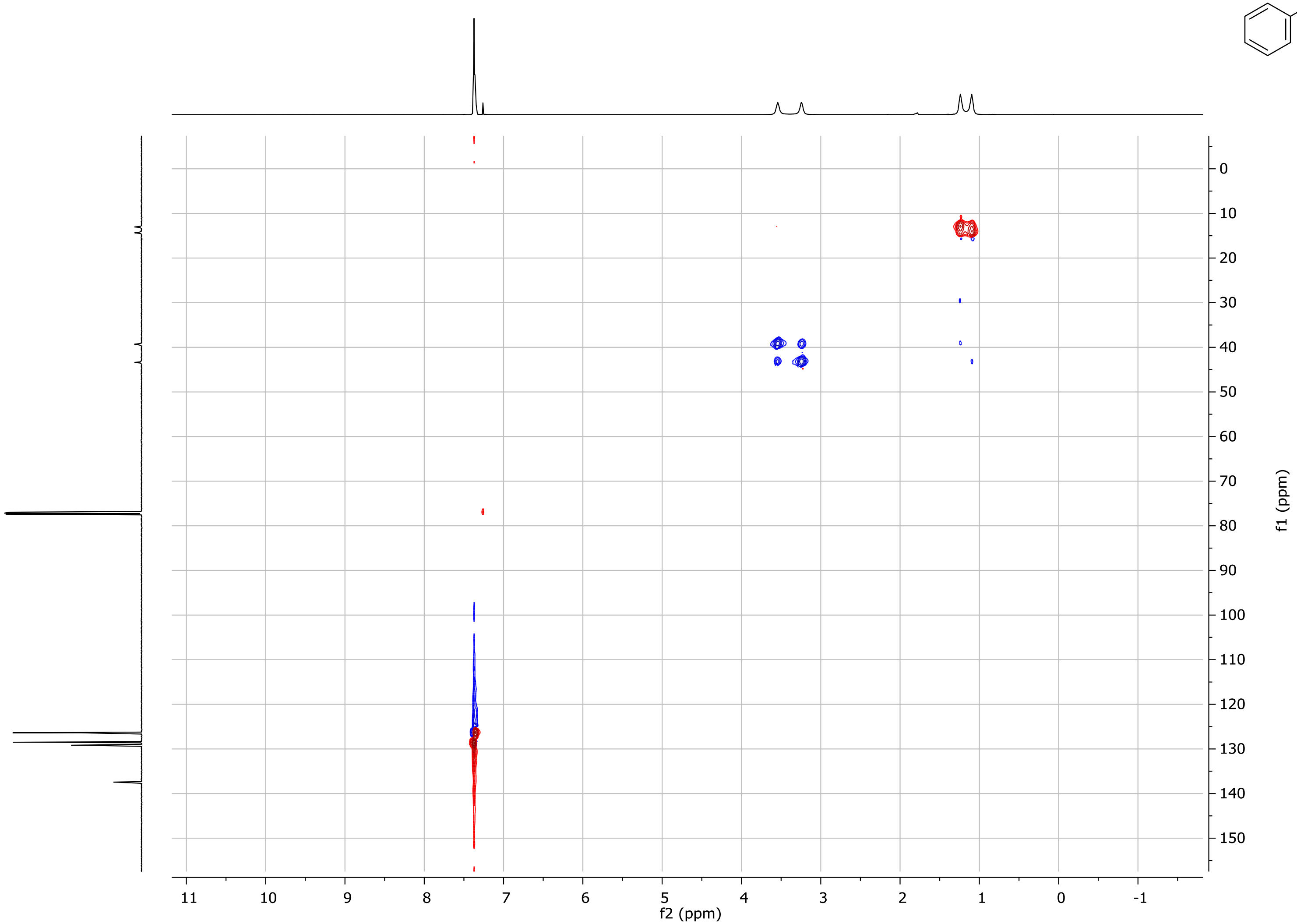

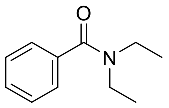

1a

HRMS

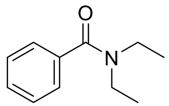

1a

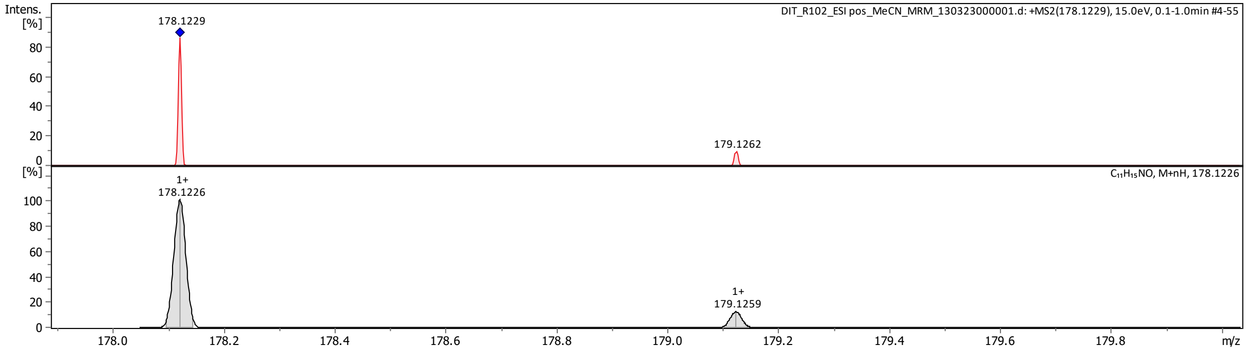
Intens. [%]
DIT_R102_ESI pos_MeCN_MRM_130323000001.d: +MS2(178.1229), 15.0eV, 0.1-1.0min #4-55
178.1229
179.1262
$C_{11}H_{15}NO$, M+nH, 178.1226
1+ 178.1226
1+ 179.1259
m/z

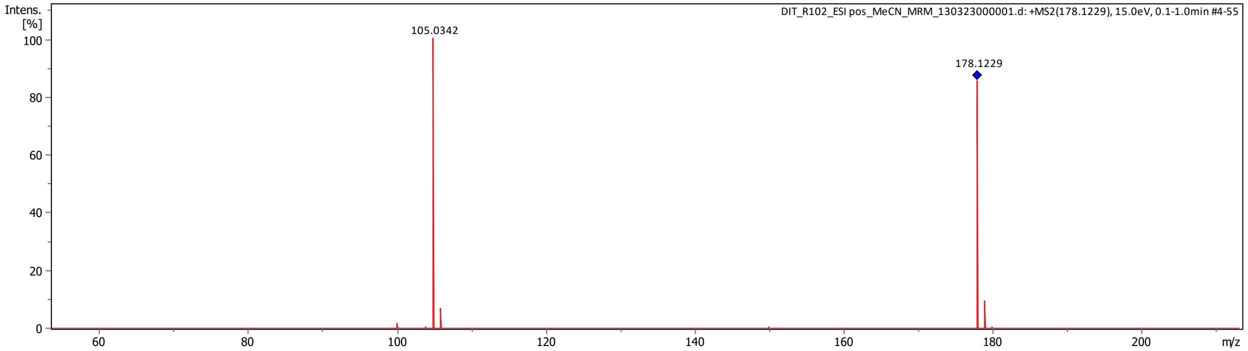
Intens. [%]
DIT_R102_ESI pos_MeCN_MRM_130323000001.d: +MS2(178.1229), 15.0eV, 0.1-1.0min #4-55
105.0342
178.1229
m/z

IR

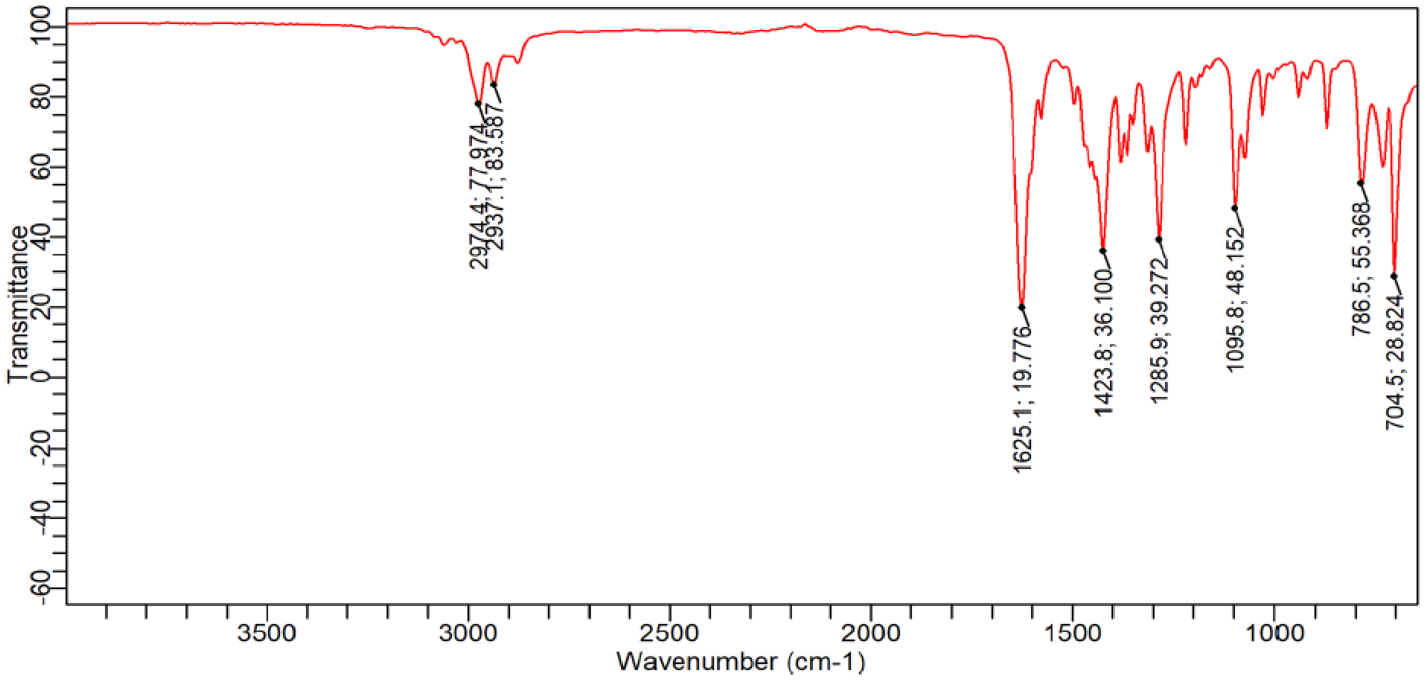
Transmittance
Wavenumber (cm-1)
100
80
60
40
20
0
-20
-40
-60
3500
3000
2500
2000
1500
1000
2974.4; 77.974
2937.1; 83.587
1625.1; 19.776
1423.8; 36.100
1285.9; 39.272
1095.8; 48.152
786.5; 55.368
704.5; 28.824

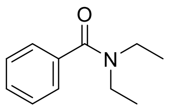
O
N

1a

2 *N*-(2-Methoxyethyl)-*N*-methylbenzamide (1b)

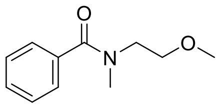

1b

^{1}H NMR

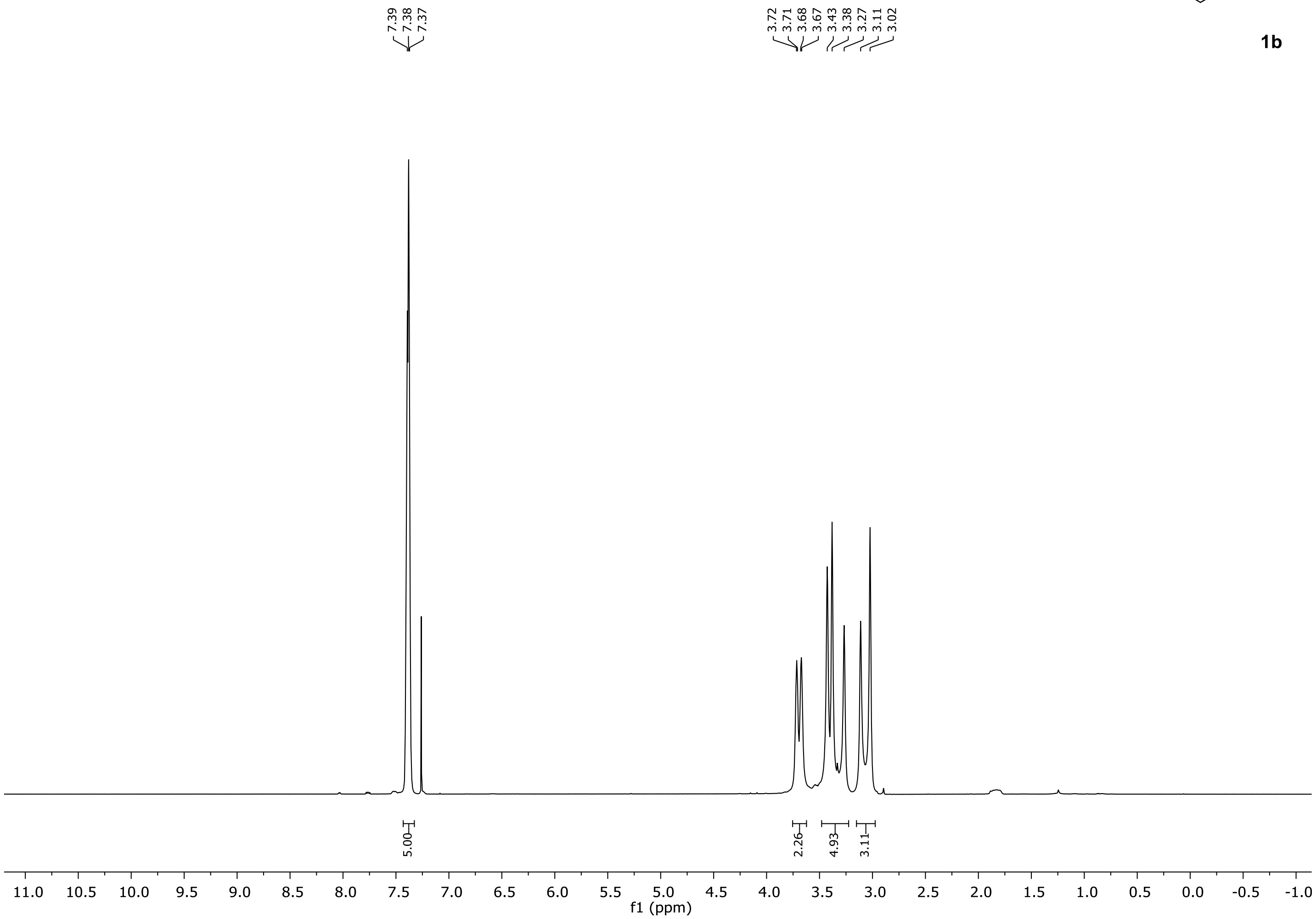

^{13}C NMR

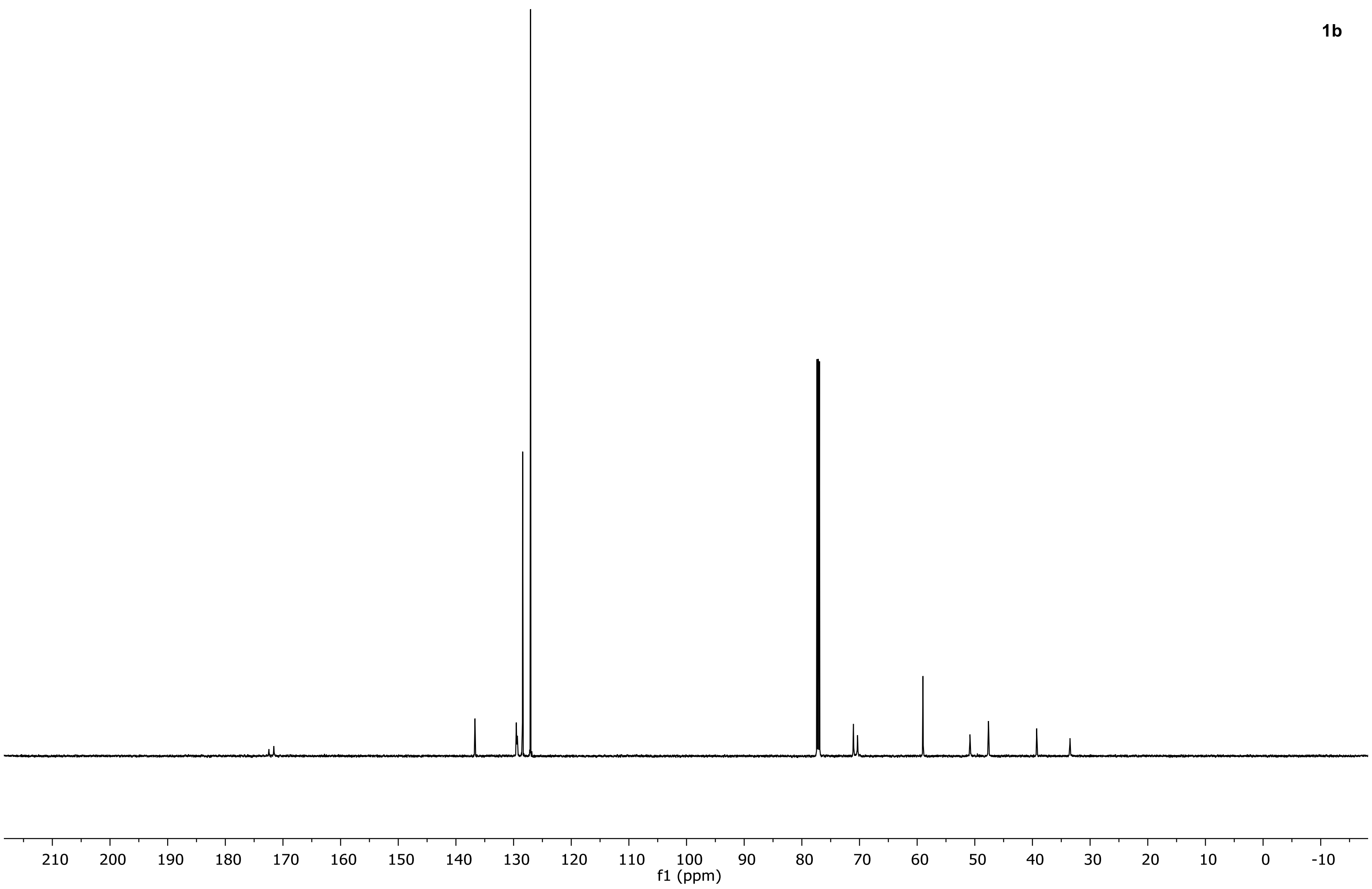
172.4
171.6
136.7
129.5
129.4
128.4
127.0
71.1
70.3
59.0
50.8
47.6
39.3
33.5
210 200 190 180 170 160 150 140 130 120 110 100 90 80 70 60 50 40 30 20 10 0 -10
f1 (ppm)

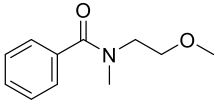
O
N
O

1b

^{1}H, ^{1}H COSY

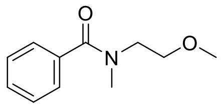

1b

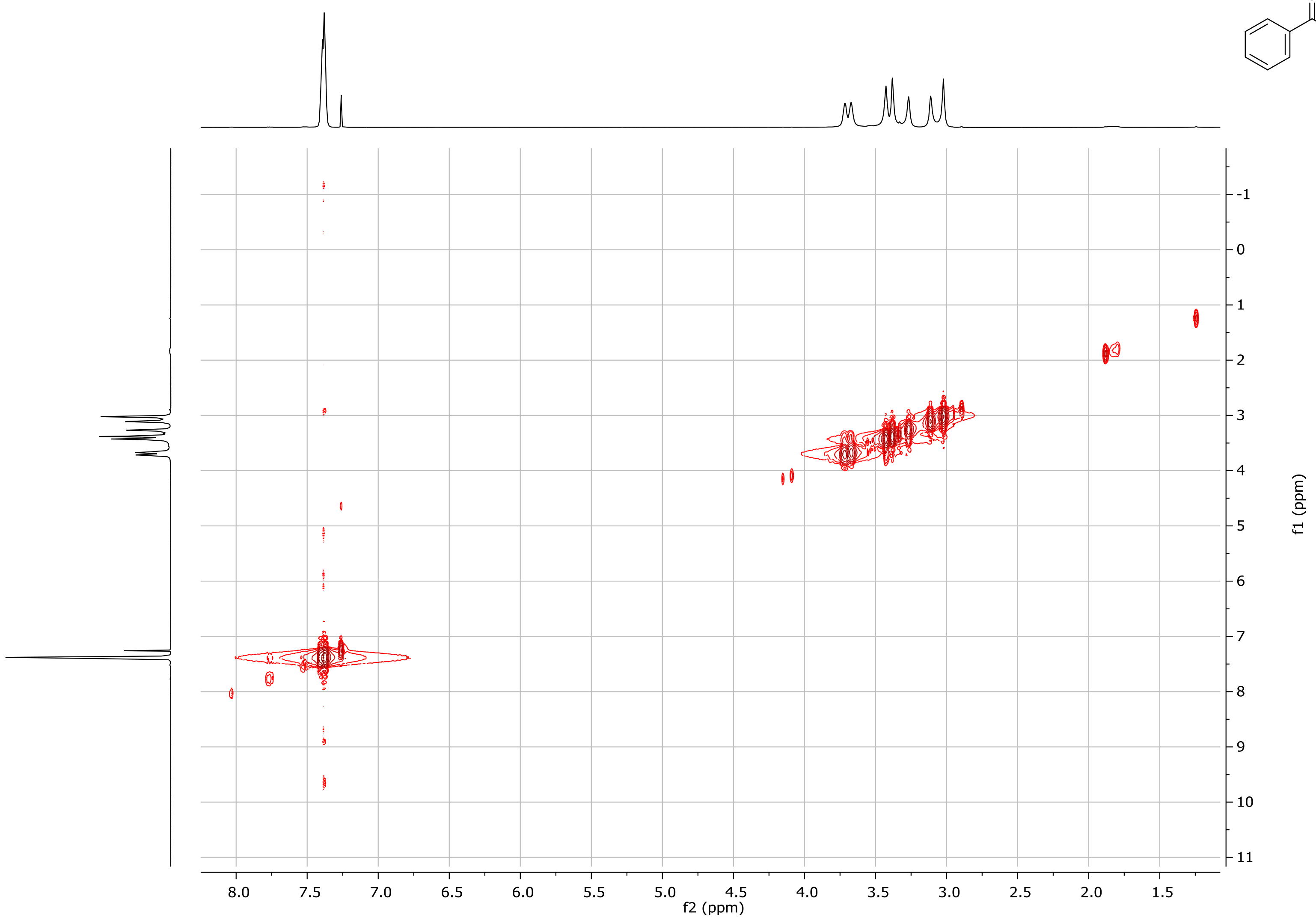
f2 (ppm)
f1 (ppm)
8.0 7.5 7.0 6.5 6.0 5.5 5.0 4.5 4.0 3.5 3.0 2.5 2.0 1.5
-1 0 1 2 3 4 5 6 7 8 9 10 11

^{1}H, ^{13}C HMBC

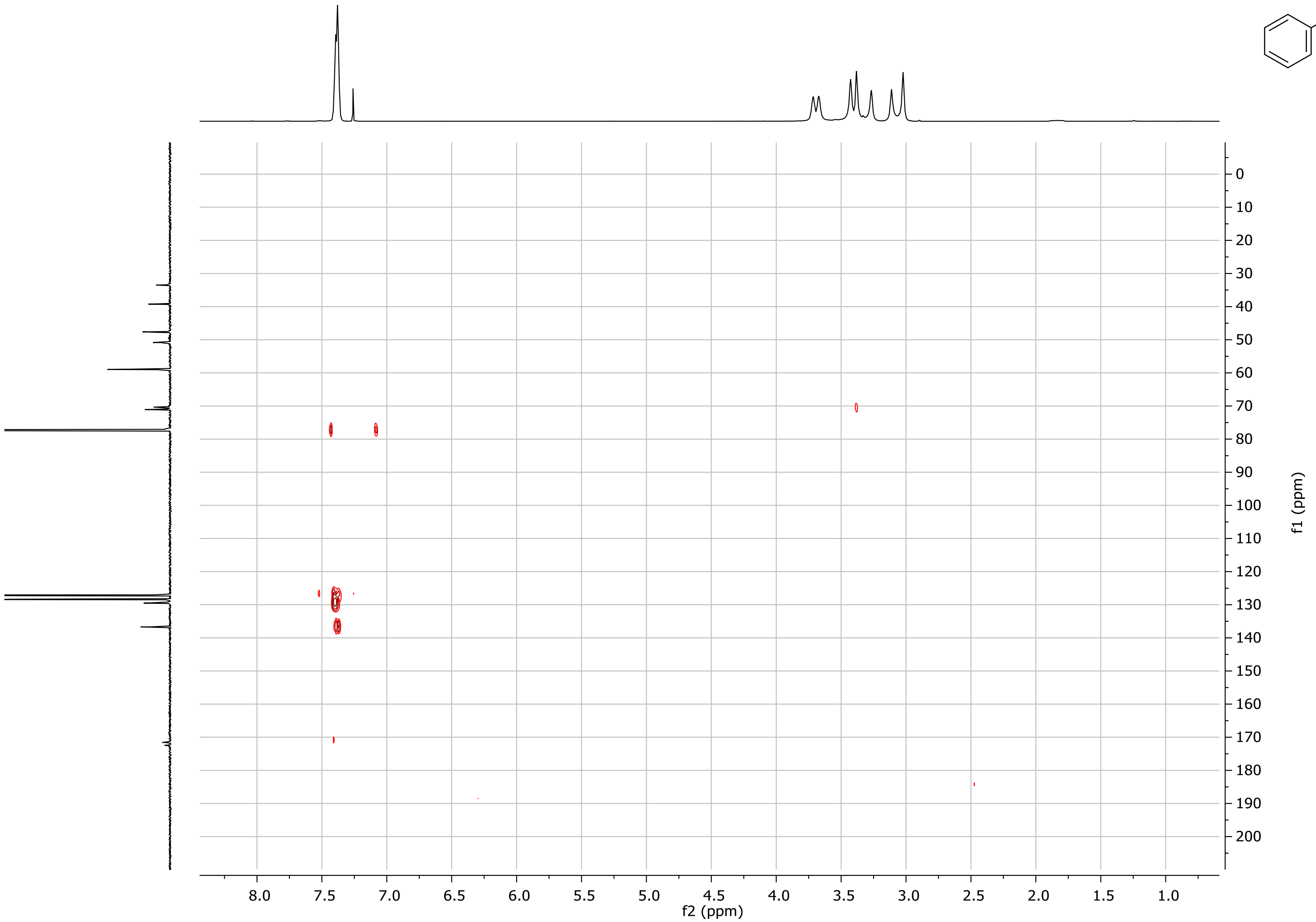

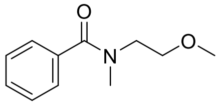

1b

^{1}H, ^{13}C HSQC

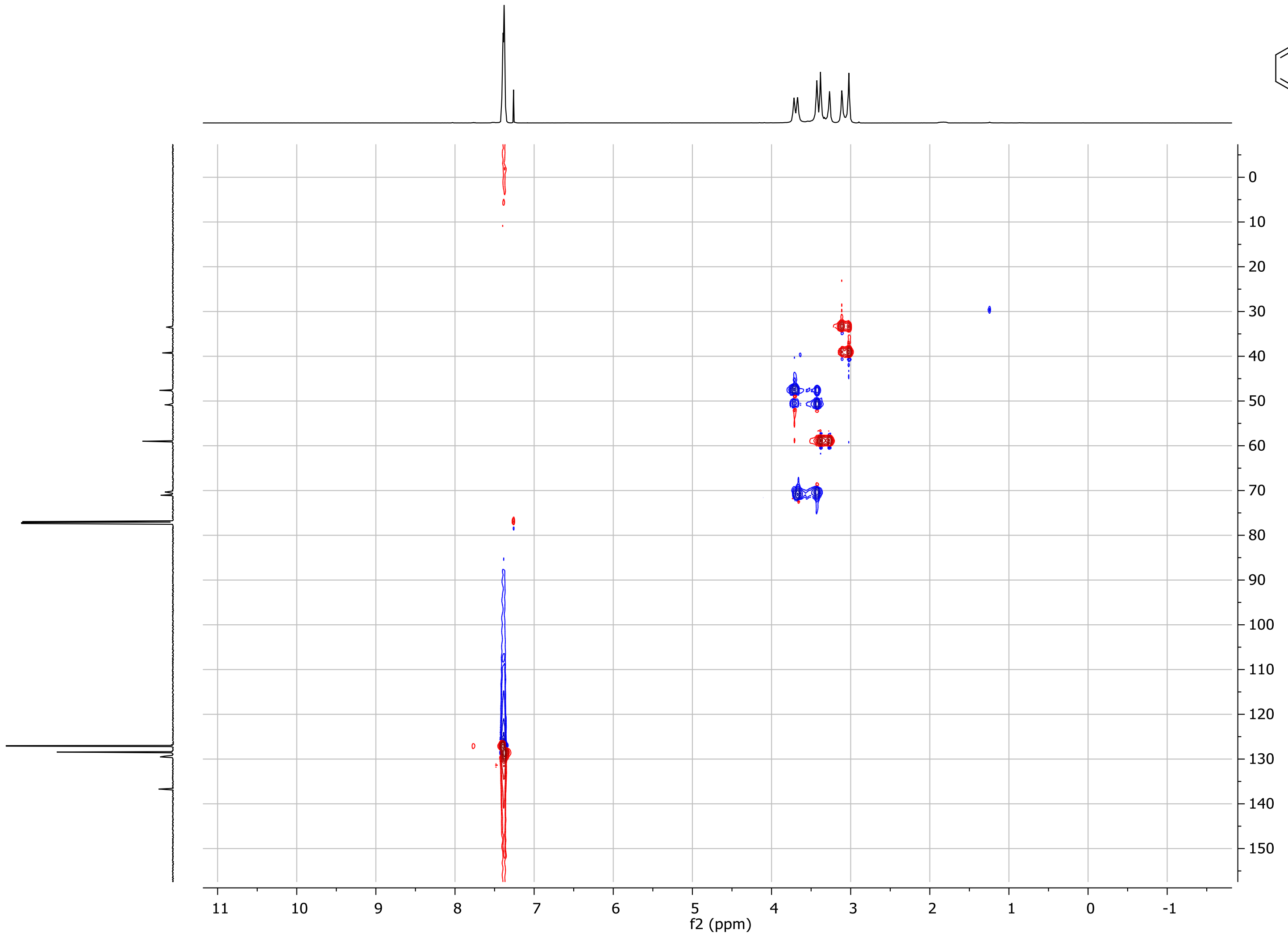

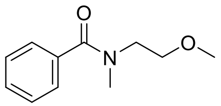

1b

HRMS

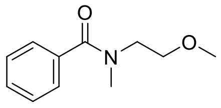

1b

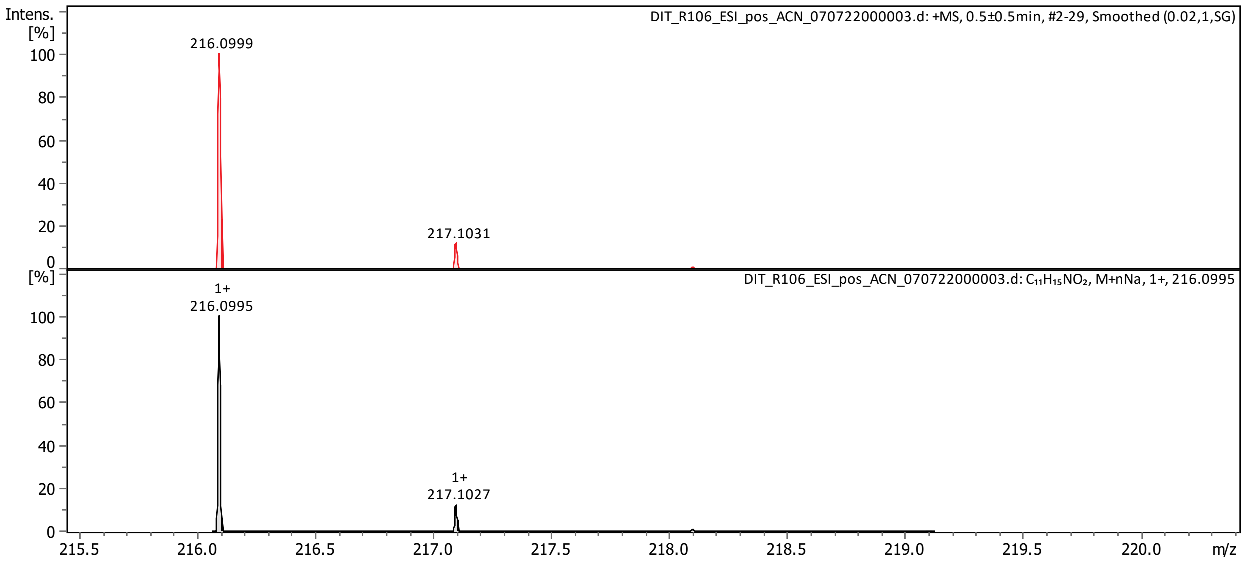

Intens.
[%]
DIT_R106_ESI_pos_ACN_070722000003.d: +MS, 0.5±0.5min, #2-29, Smoothed (0.02,1,SG)
216.0999
217.1031
DIT_R106_ESI_pos_ACN_070722000003.d: C11H15NO2, M+nNa, 1+, 216.0995
1+
216.0995
1+
217.1027
m/z

IR

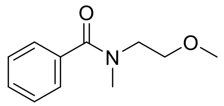
O
N
O

1b

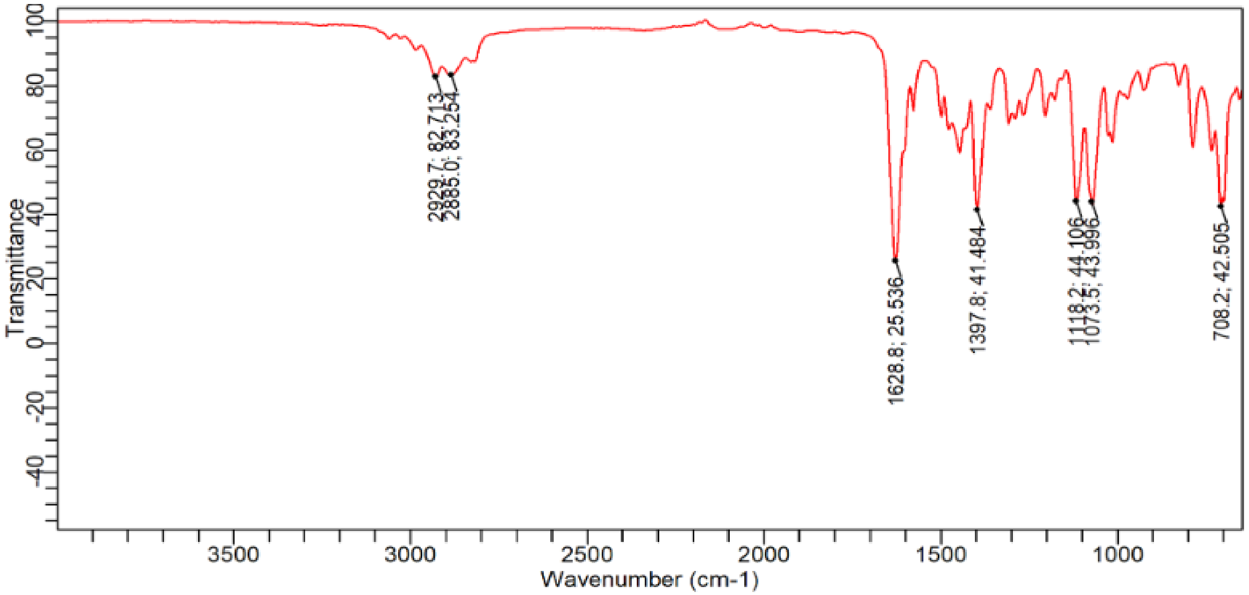
Transmittance
Wavenumber (cm-1)
100
80
60
40
20
0
-20
-40
3500
3000
2500
2000
1500
1000
2929.7; 82.713
2885.0; 83.254
1628.8; 25.536
1397.8; 41.484
1118.2; 44.106
1073.5; 43.996
708.2; 42.505

3 *N,N*-bis(2-Methoxyethyl)benzamide (1c)

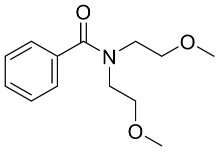

1c

^{1}H NMR

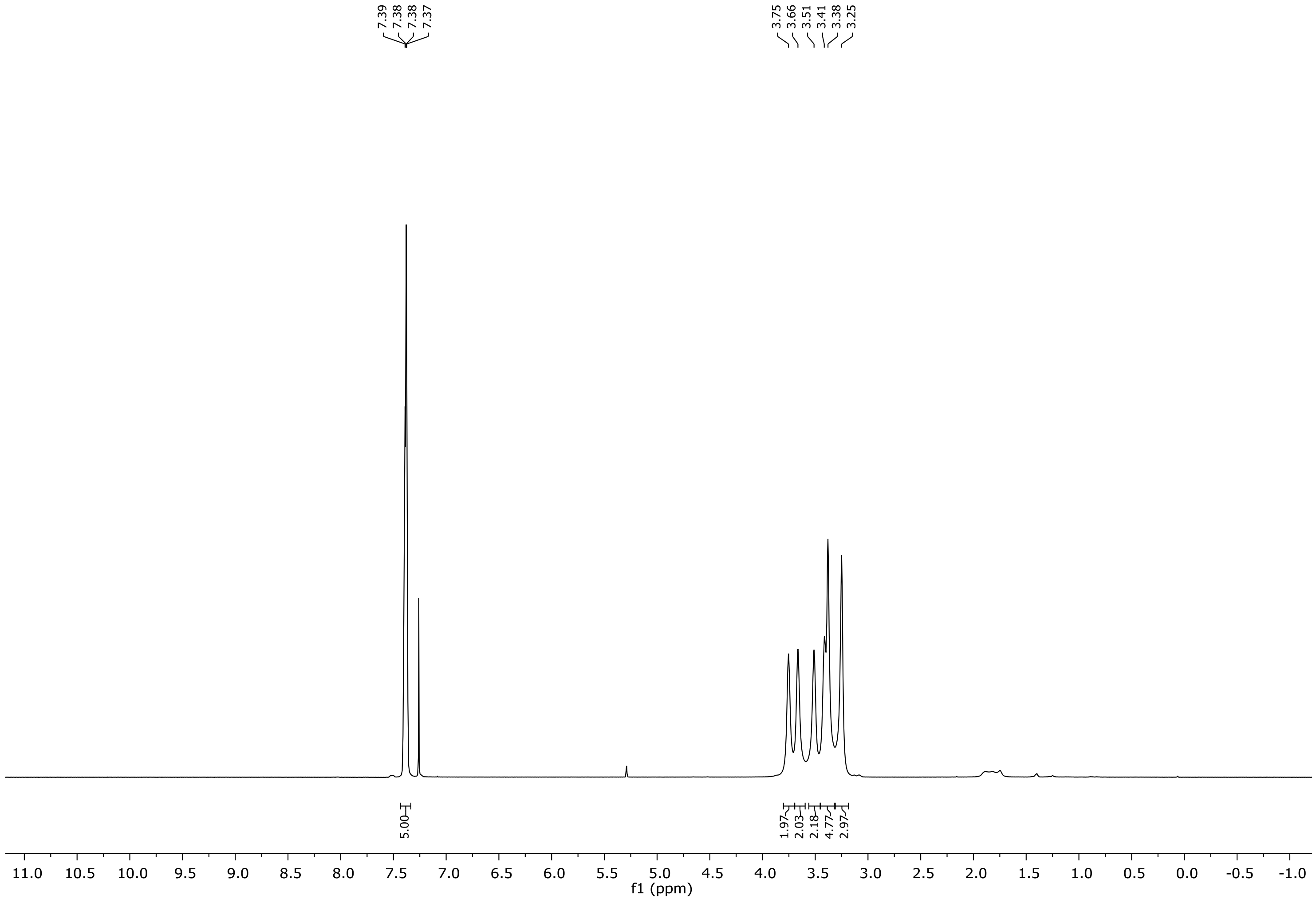

^{13}C NMR

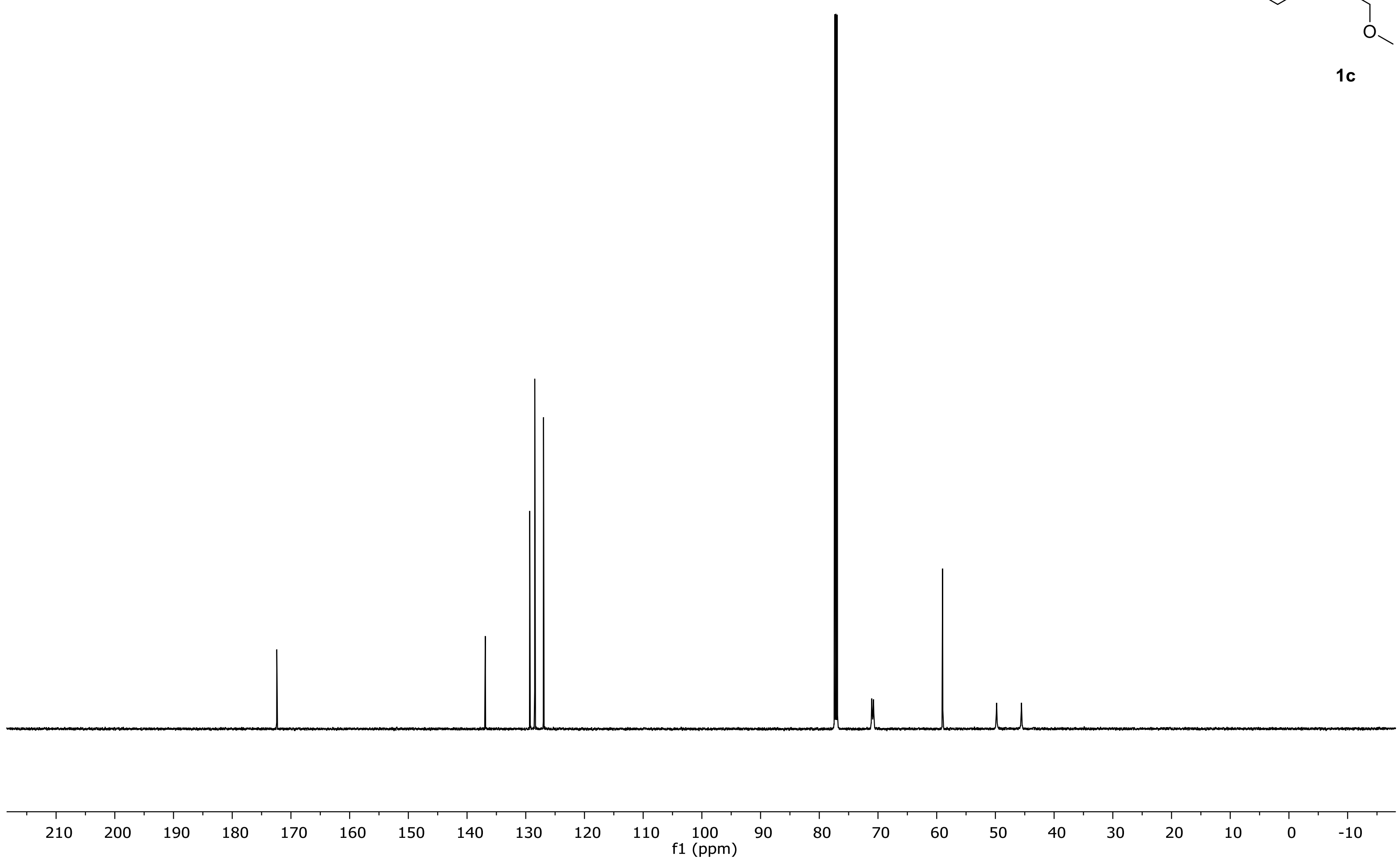
172.4
136.9
129.3
128.5
127.0
71.1
70.8
59.0
49.8
45.6
210 200 190 180 170 160 150 140 130 120 110 100 90 80 70 60 50 40 30 20 10 0 -10
f1 (ppm)

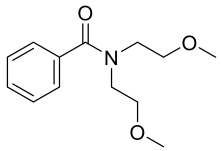
O
N
O
O

1c

^1H, ^1H COSY

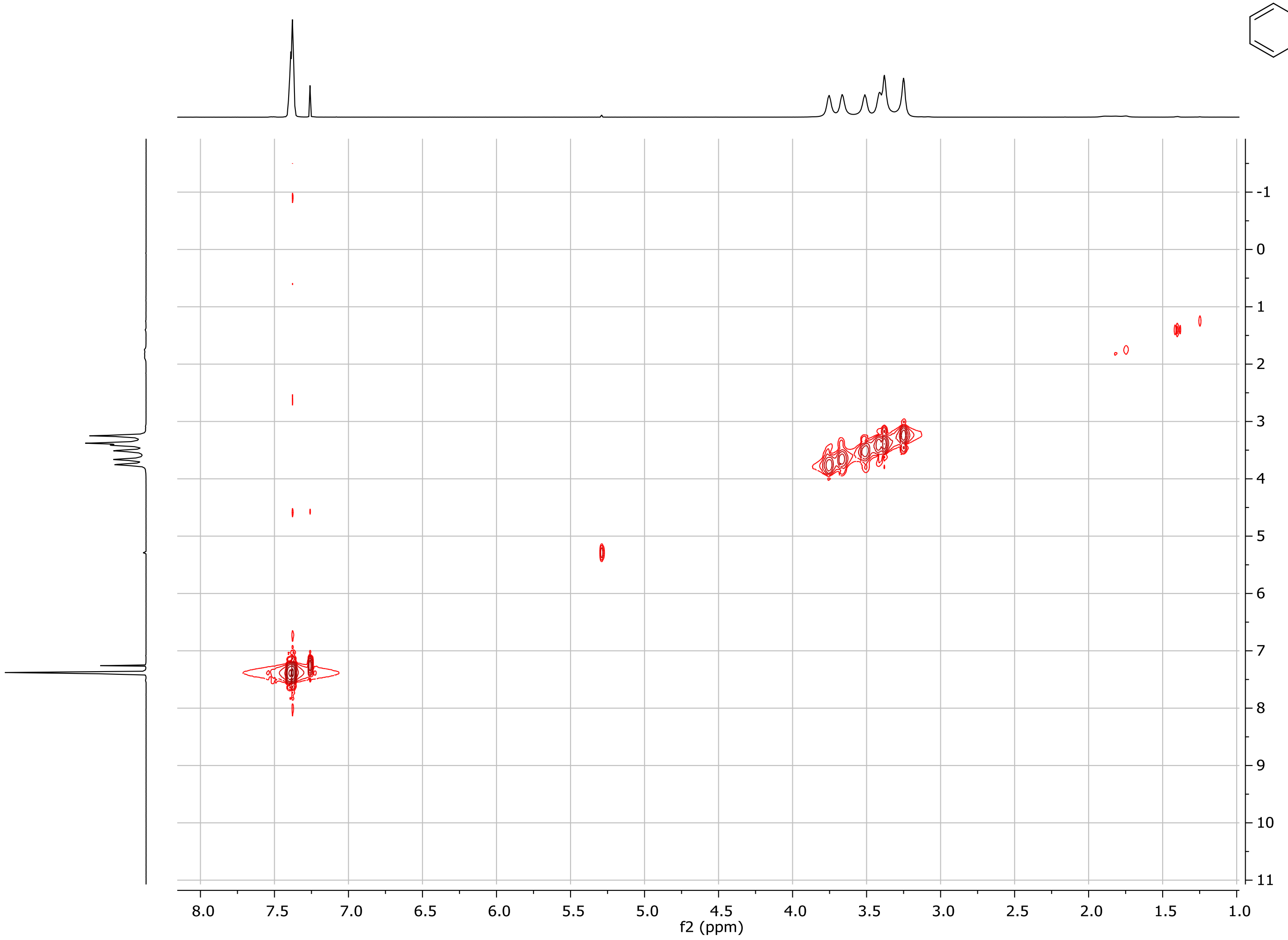

f2 (ppm)
f1 (ppm)
8.0 7.5 7.0 6.5 6.0 5.5 5.0 4.5 4.0 3.5 3.0 2.5 2.0 1.5 1.0
-1 0 1 2 3 4 5 6 7 8 9 10 11

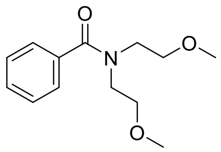

O
N
O
O

1c

^{1}H, ^{13}C HMBC

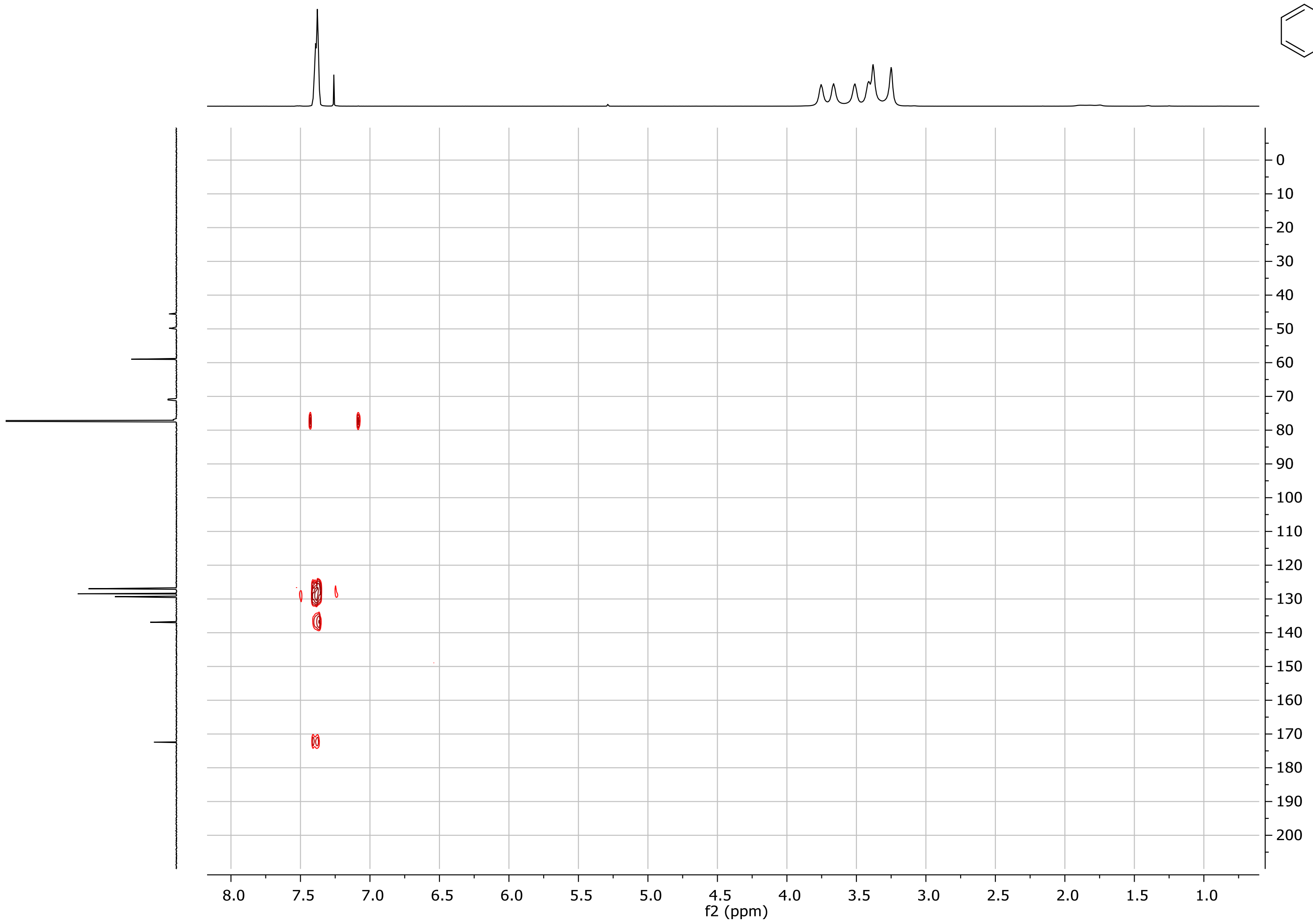

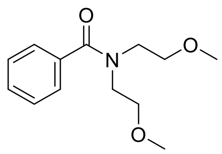

1c

^1H, ^{13}C HSQC

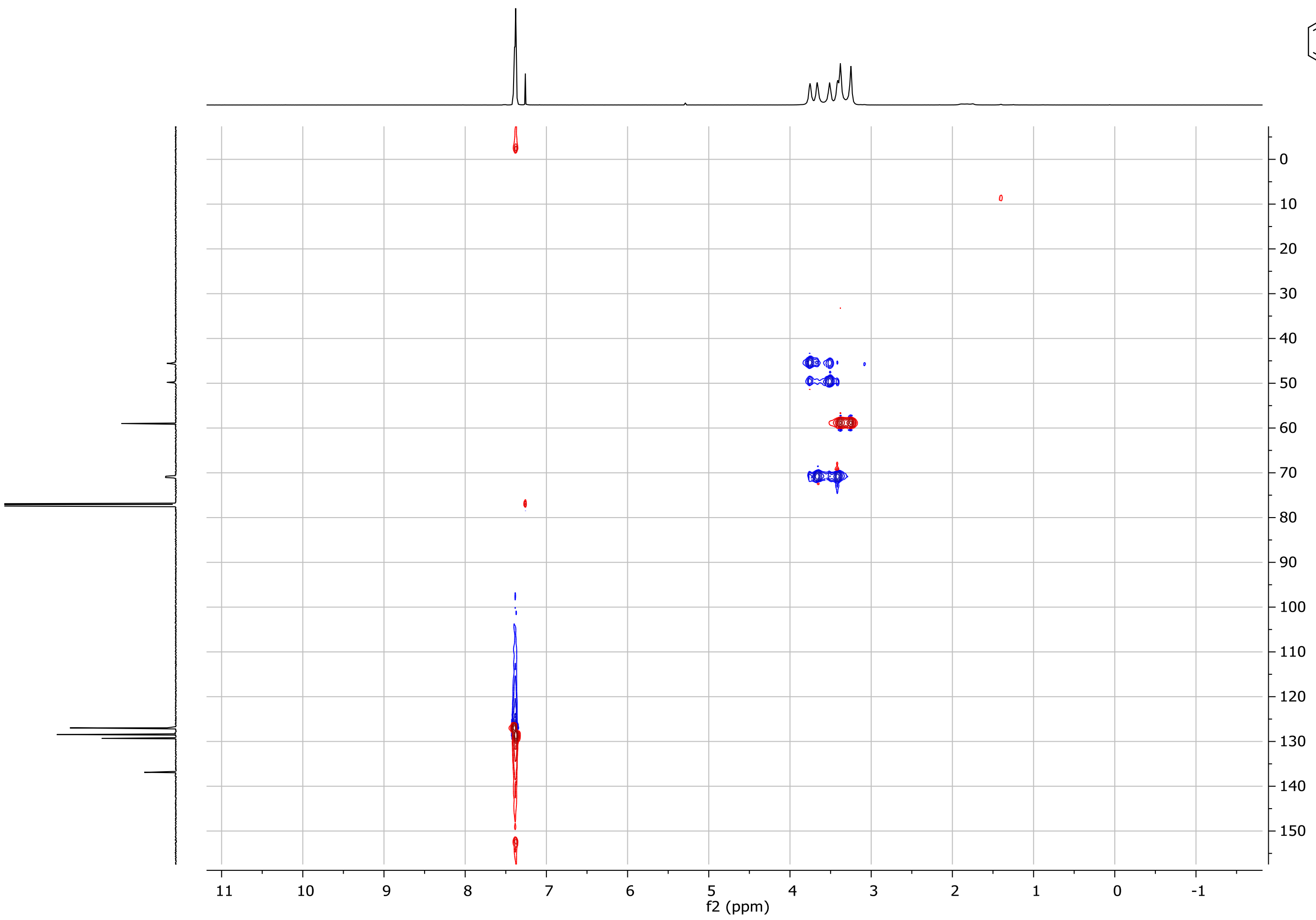

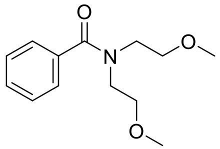

1c

HRMS

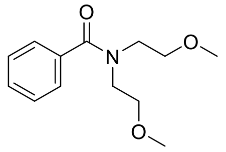

1c

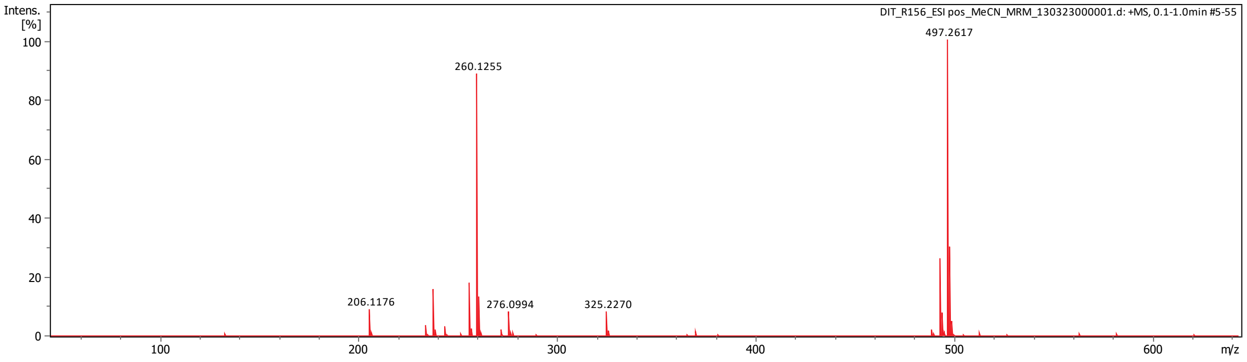

Intens. [%]
DIT_R156_ESI pos_MeCN_MRM_130323000001.d: +MS, 0.1-1.0min #5-55
497.2617
260.1255
206.1176
276.0994
325.2270
m/z

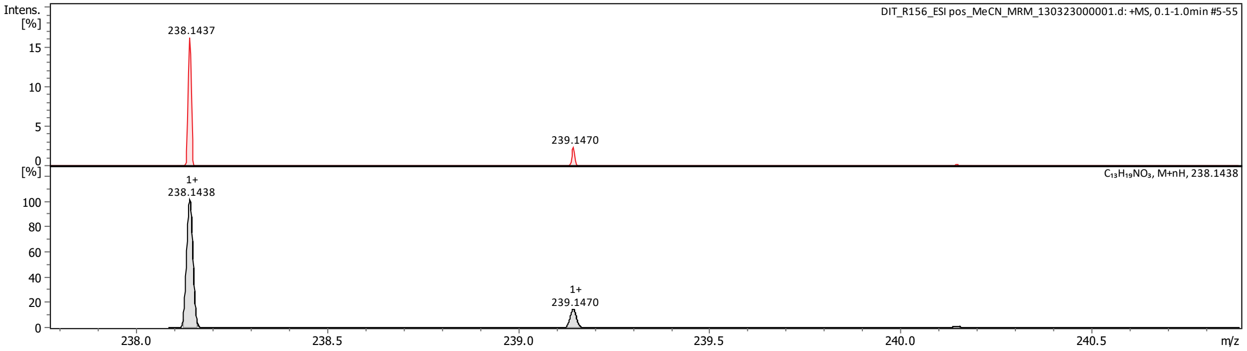

Intens. [%]
DIT_R156_ESI pos_MeCN_MRM_130323000001.d: +MS, 0.1-1.0min #5-55
238.1437
239.1470
C13H19NO3, M+nH, 238.1438
1+
238.1438
1+
239.1470
m/z

IR

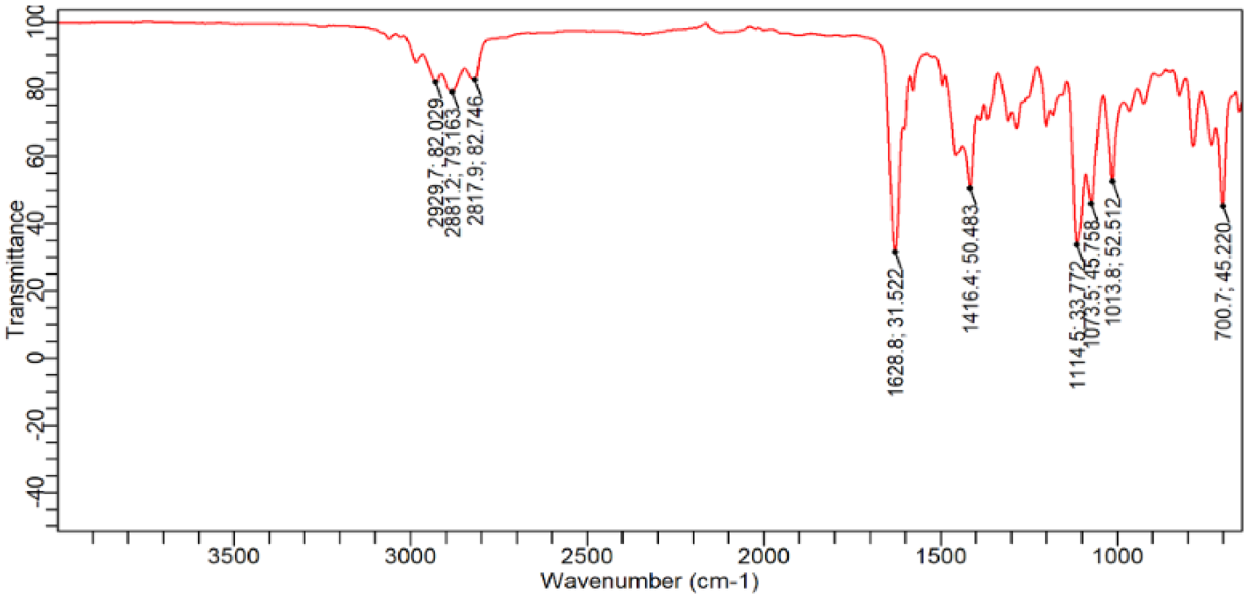

Transmittance
Wavenumber (cm-1)
100
80
60
40
20
0
-20
-40
3500
3000
2500
2000
1500
1000
2929.7; 82.029
2881.2; 79.163
2817.9; 82.746
1628.8; 31.522
1416.4; 50.483
1114.5; 33.772
1073.5; 45.758
1013.8; 52.512
700.7; 45.220

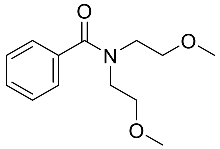

1c

4 Phenyl(piperidin-1-yl)methanone (1d)

^{1}H NMR

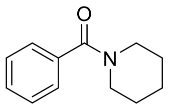

1d

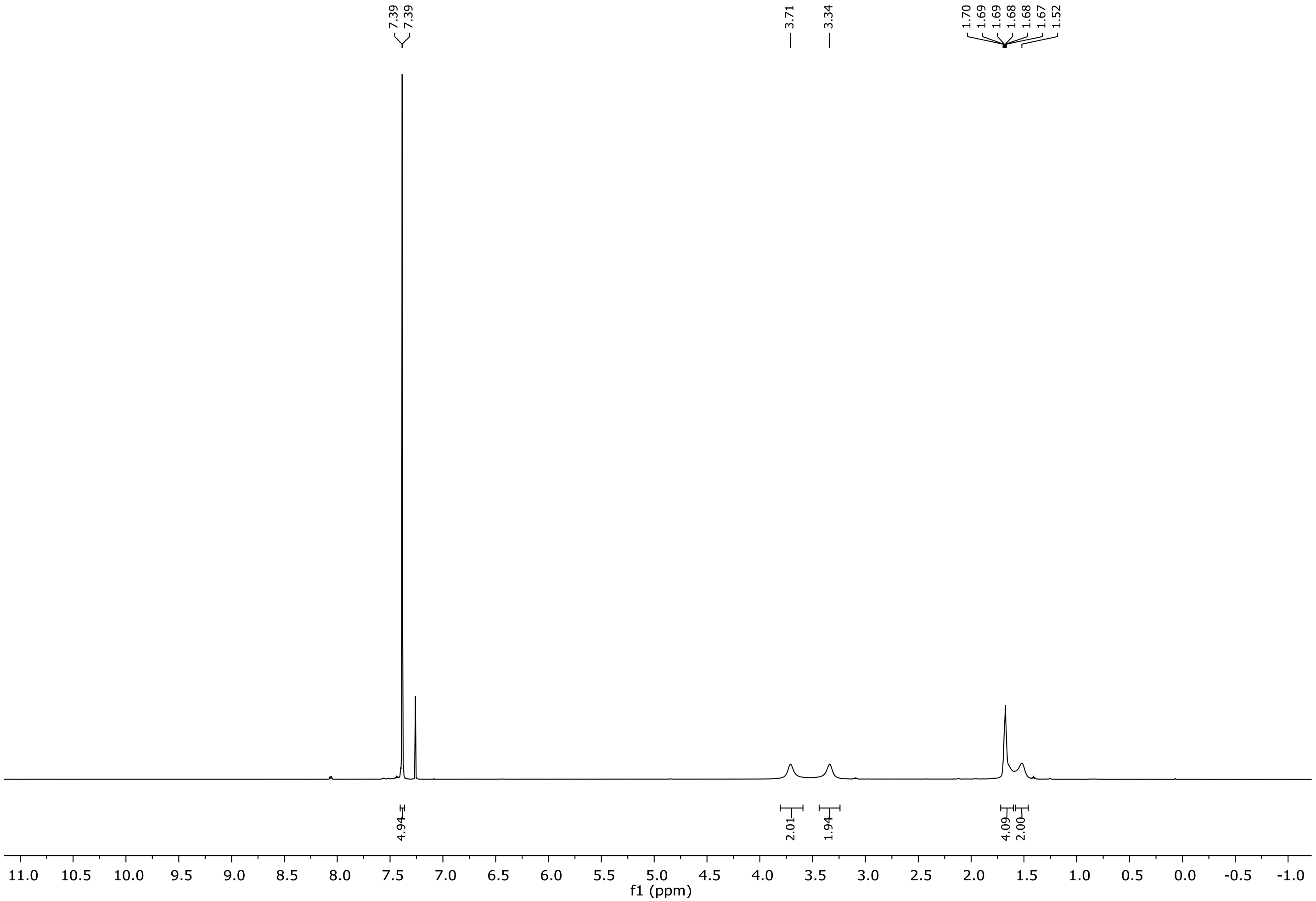

^{13}C NMR

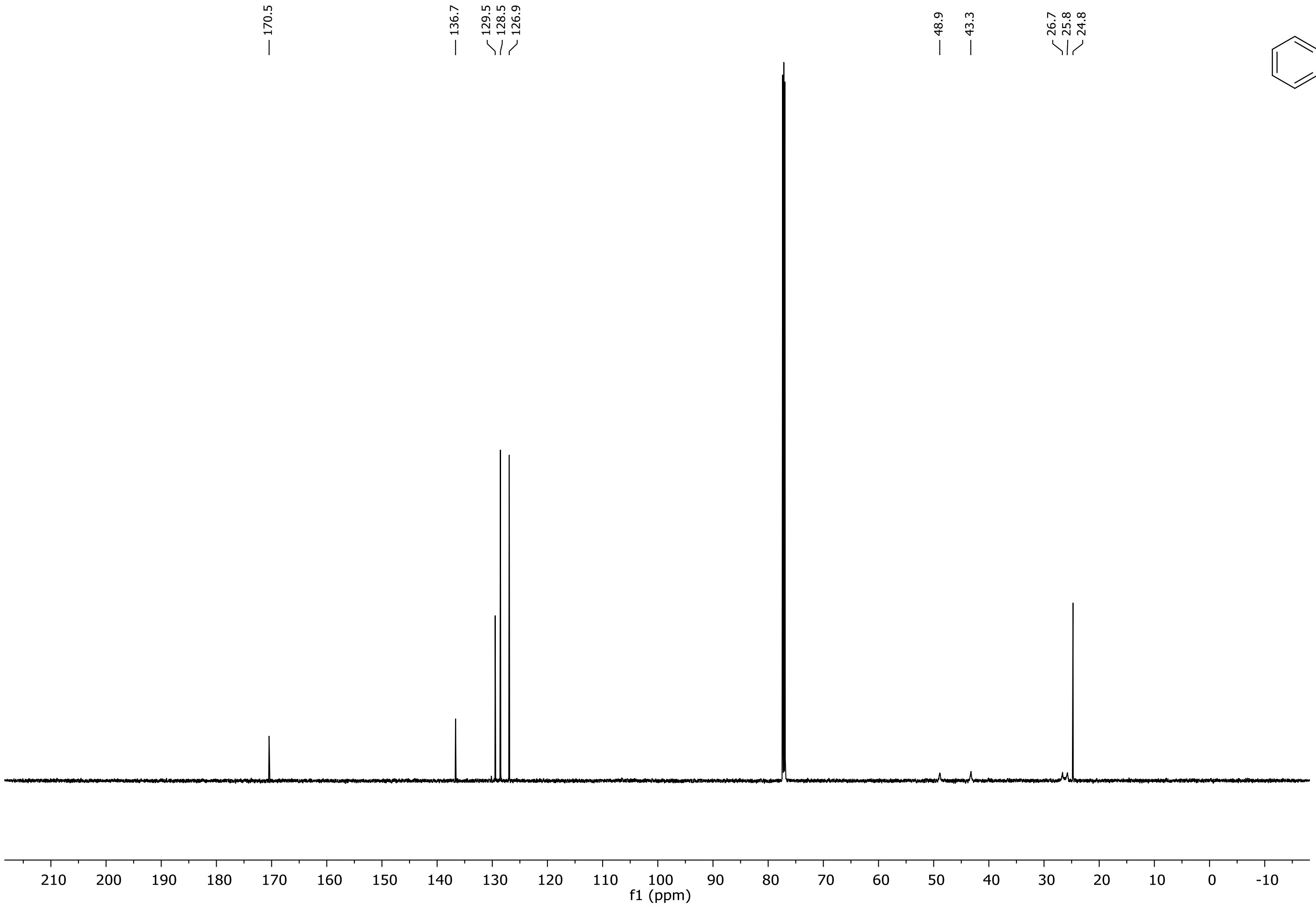
170.5
136.7
129.5
128.5
126.9
48.9
43.3
26.7
25.8
24.8
210
200
190
180
170
160
150
140
130
120
110
100
90
80
70
60
50
40
30
20
10
0
-10
f1 (ppm)

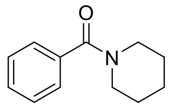
O
N

1d

^{1}H, ^{1}H COSY

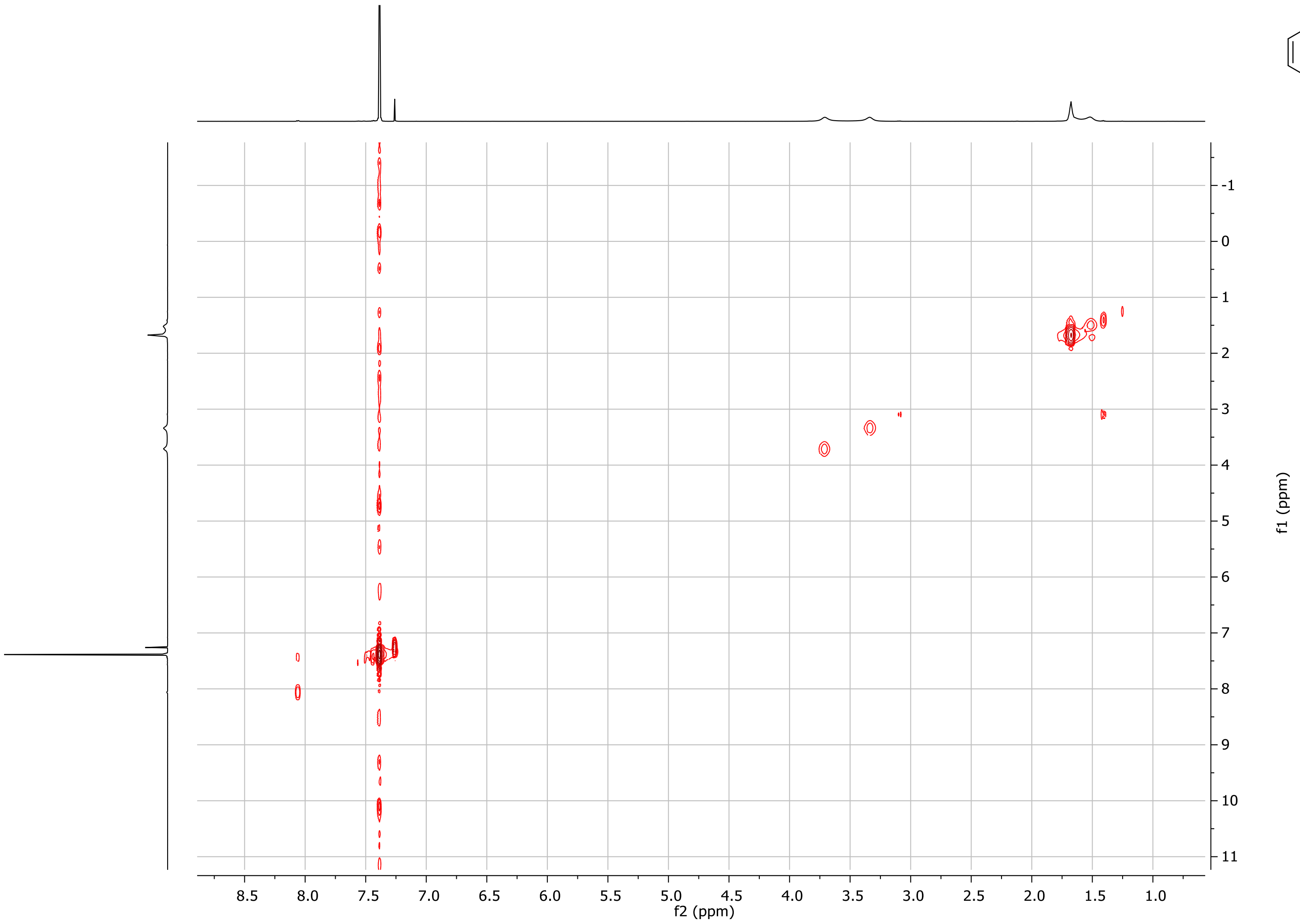

8.5
8.0
7.5
7.0
6.5
6.0
5.5
5.0
4.5
4.0
3.5
3.0
2.5
2.0
1.5
1.0
f2 (ppm)
-1
0
1
2
3
4
5
6
7
8
9
10
11
f1 (ppm)

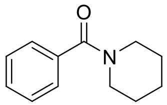

O
N

1d

^1H, ^{13}C HMBC

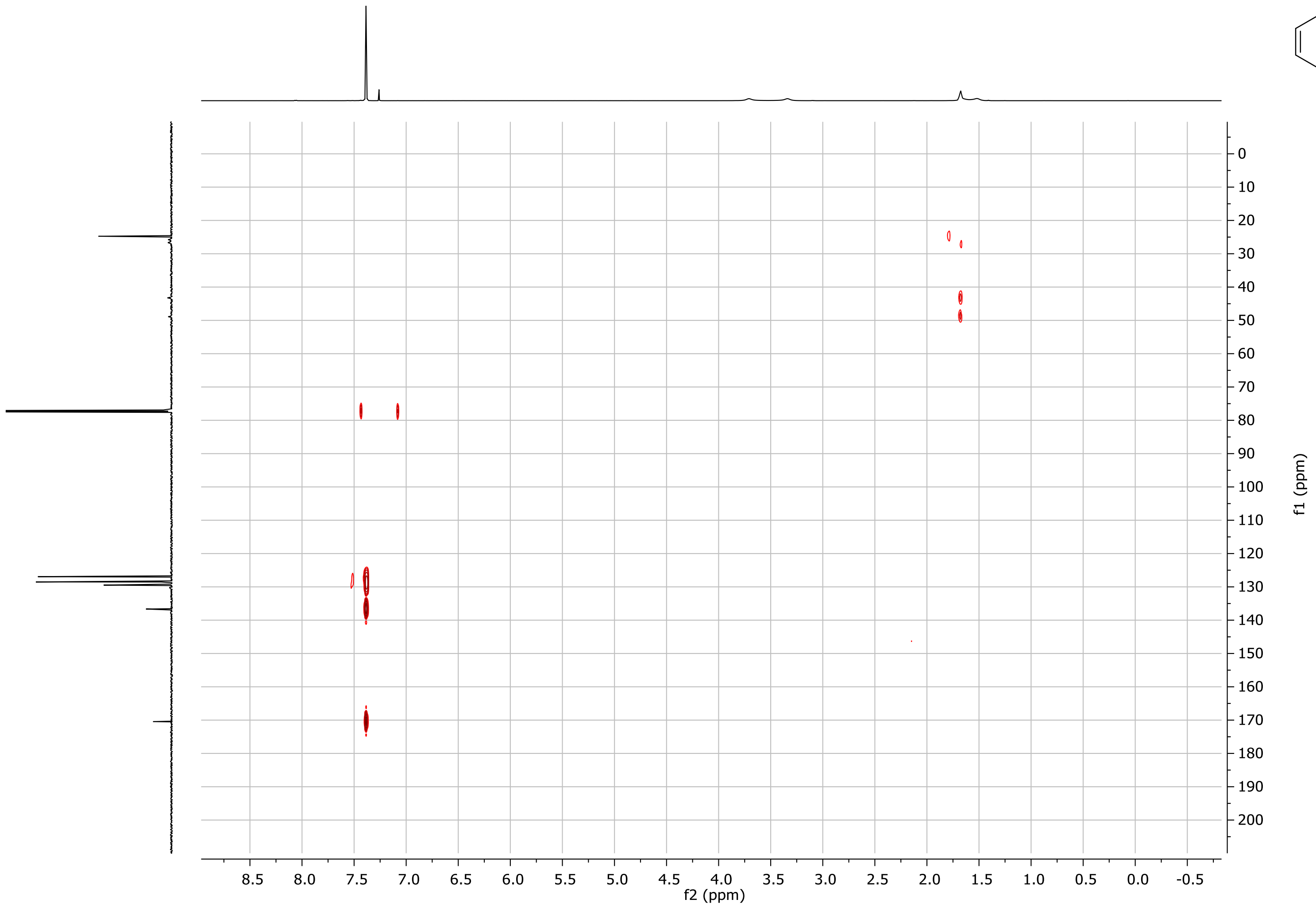

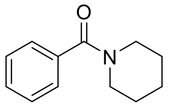

1d

^{1}H, ^{13}C HSQC

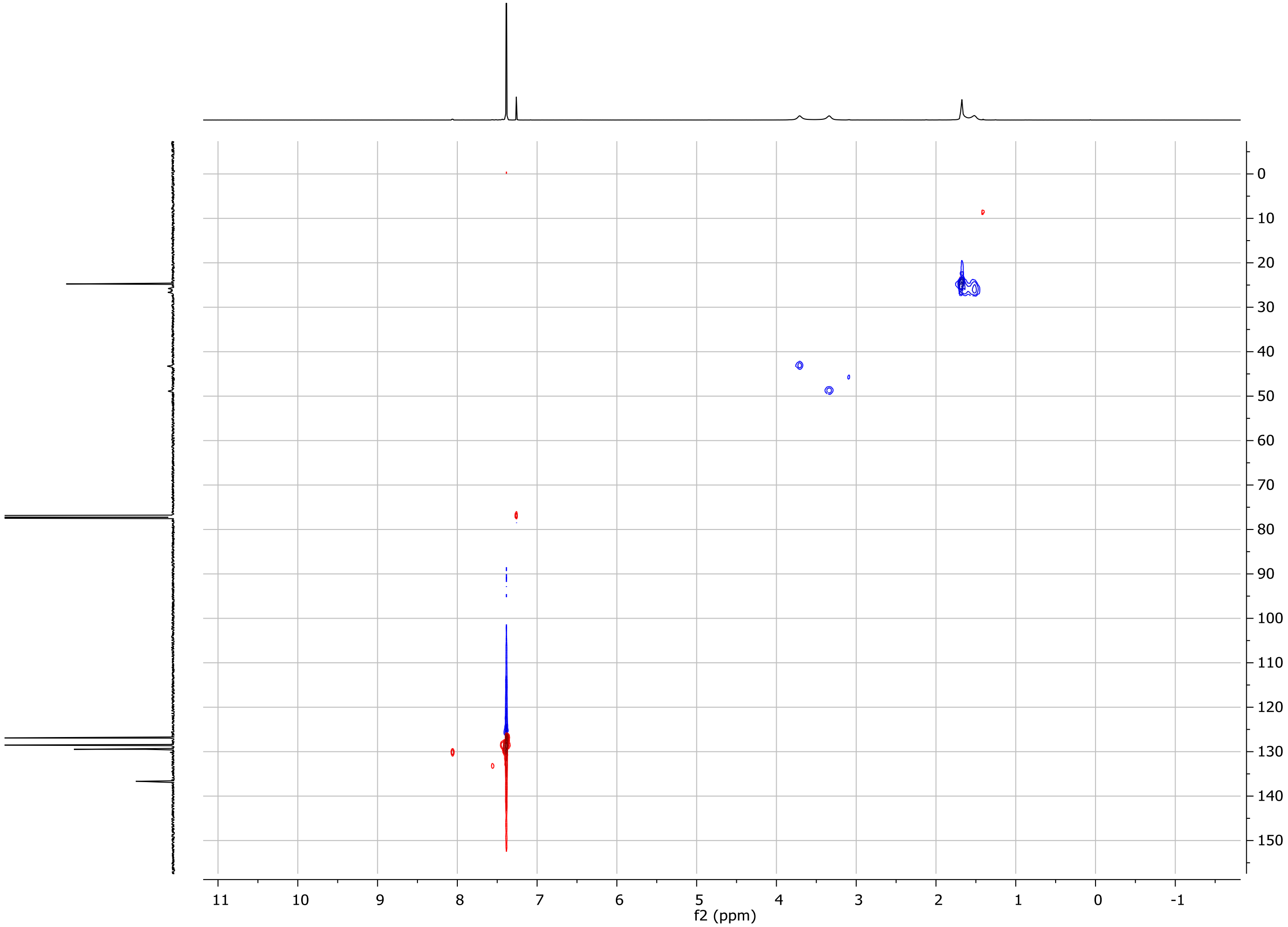

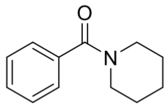

1d

HRMS

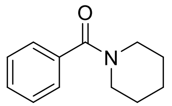

1d

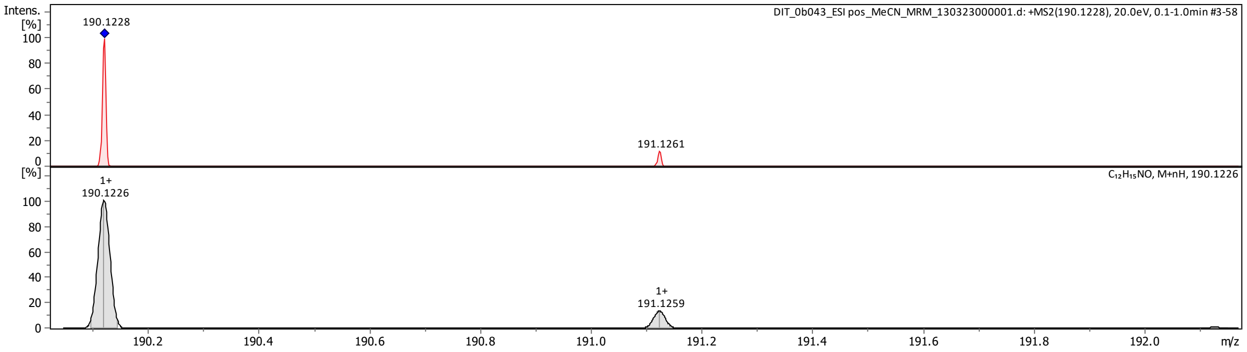
Intens. [%]
DIT_0b043_ESI pos_MeCN_MRM_130323000001.d: +MS2(190.1228), 20.0eV, 0.1-1.0min #3-58
190.1228
191.1261
C12H15NO, M+nH, 190.1226
1+
190.1226
1+
191.1259
m/z

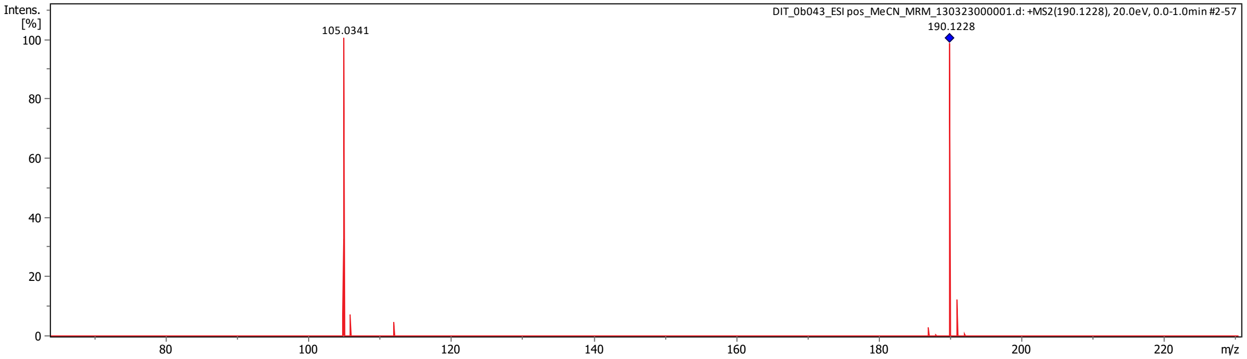
Intens. [%]
DIT_0b043_ESI pos_MeCN_MRM_130323000001.d: +MS2(190.1228), 20.0eV, 0.0-1.0min #2-57
105.0341
190.1228
m/z

IR

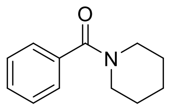

1d

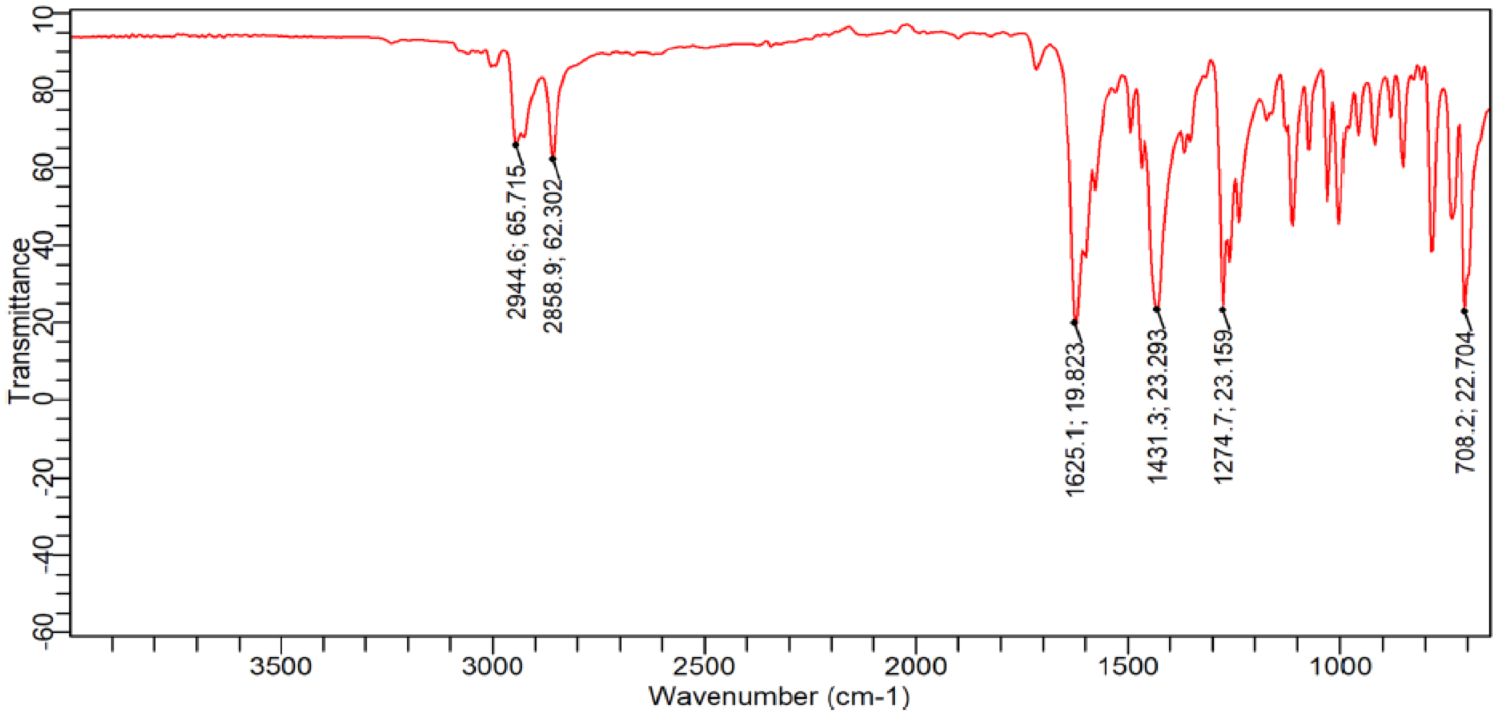

Transmittance
Wavenumber (cm-1)
2944.6; 65.715
2858.9; 62.302
1625.1; 19.823
1431.3; 23.293
1274.7; 23.159
708.2; 22.704

5 2-Azaspiro[3.3]heptan-2-yl(phenyl)methano (1e)

^{1}H NMR

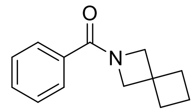

1e

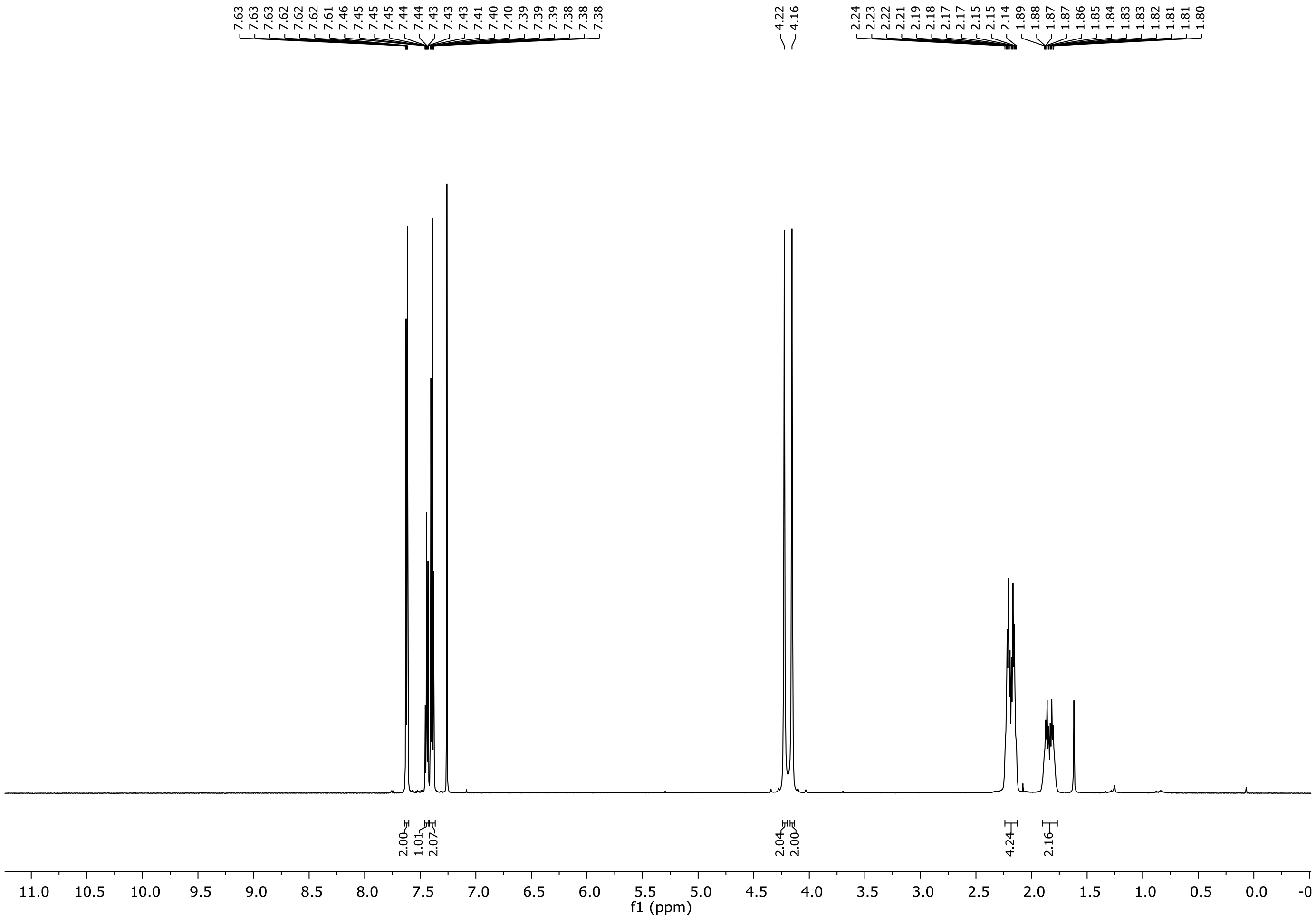

^{13}C NMR

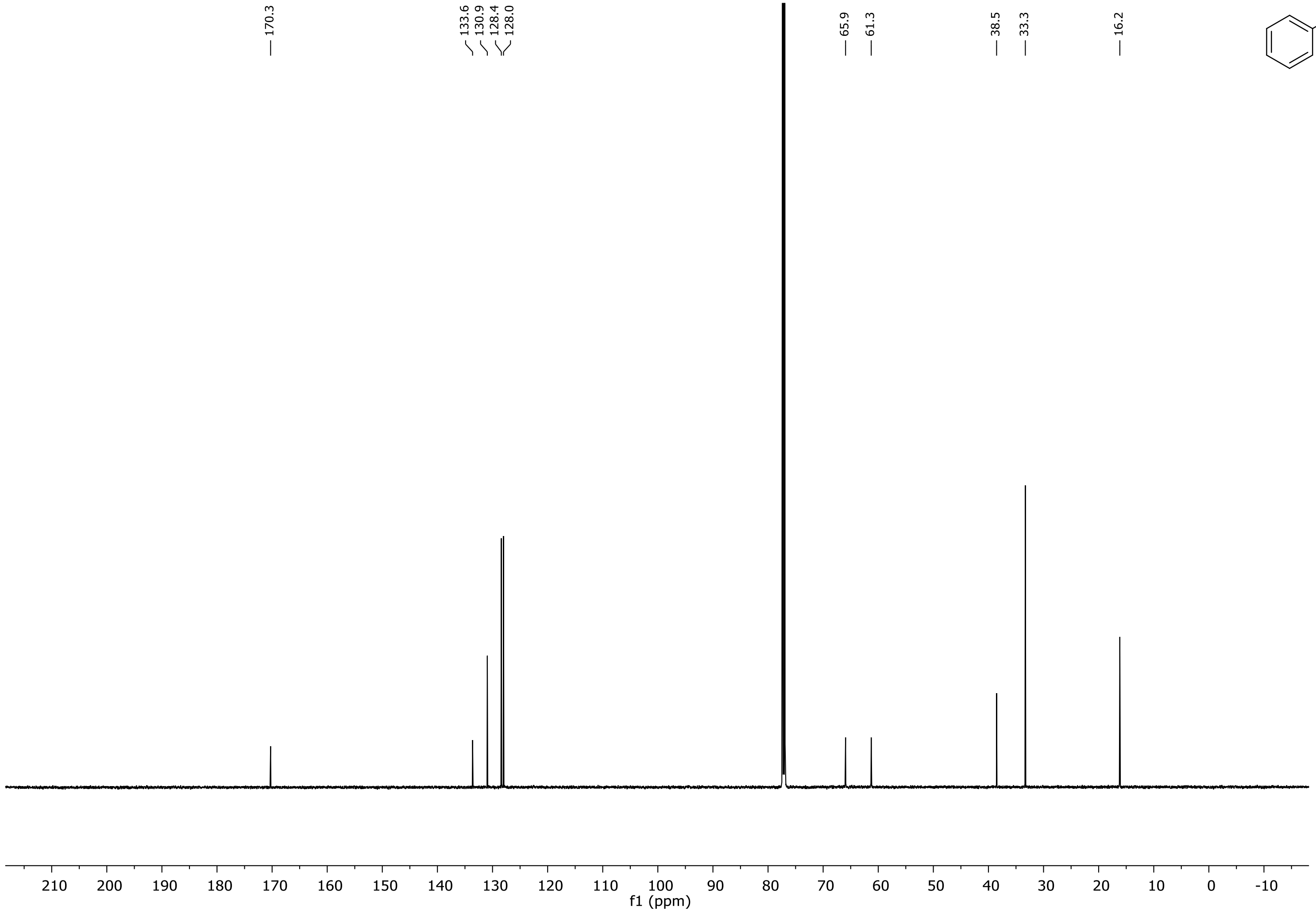
170.3
133.6
130.9
128.4
128.0
65.9
61.3
38.5
33.3
16.2
210
200
190
180
170
160
150
140
130
120
110
100
90
80
70
60
50
40
30
20
10
0
-10
f1 (ppm)

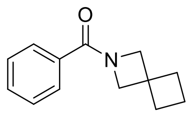
O
N

1e

^{1}H, ^{1}H COSY

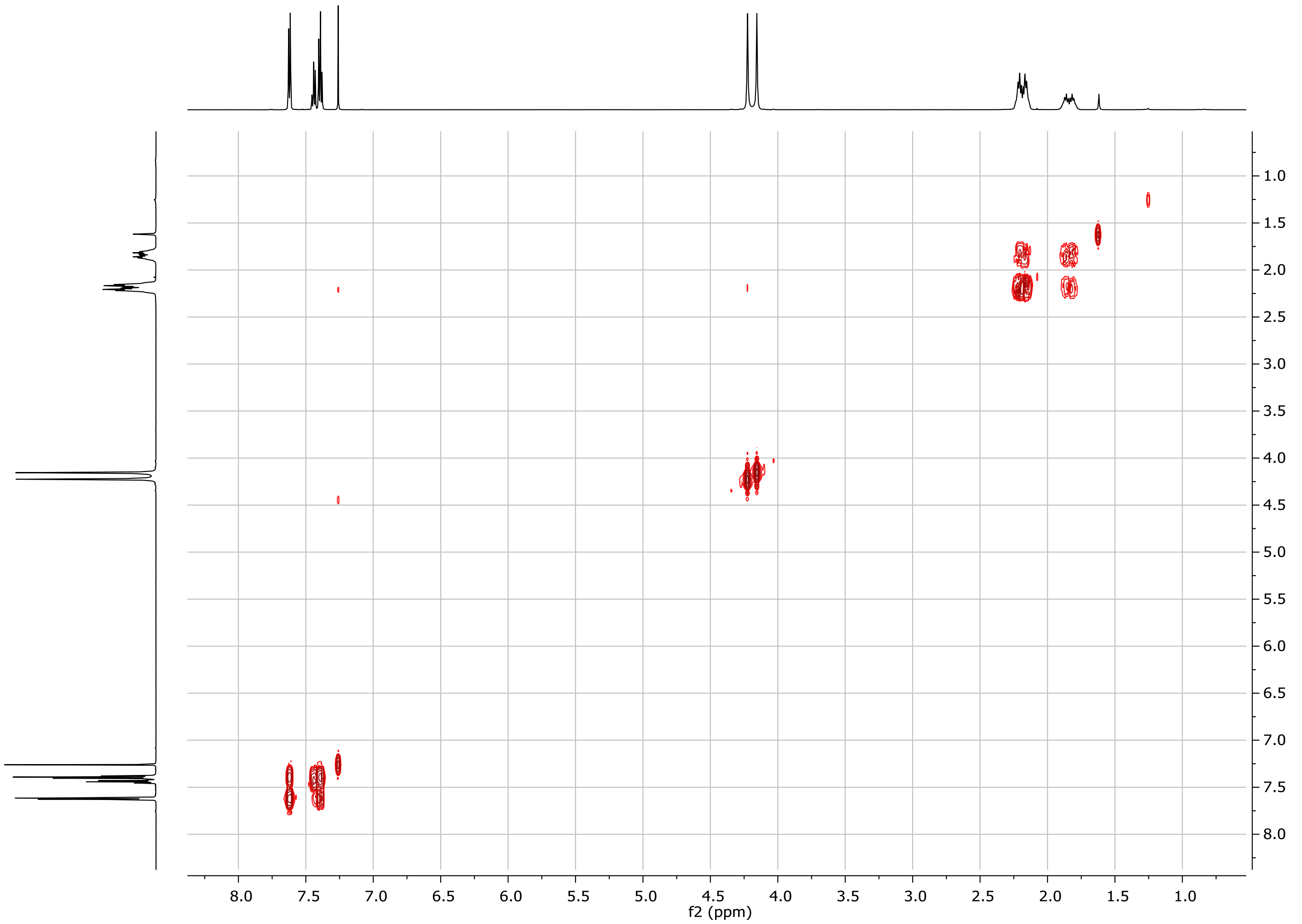

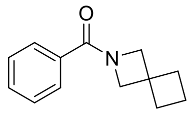

1e

^{1}H, ^{13}C HMBC

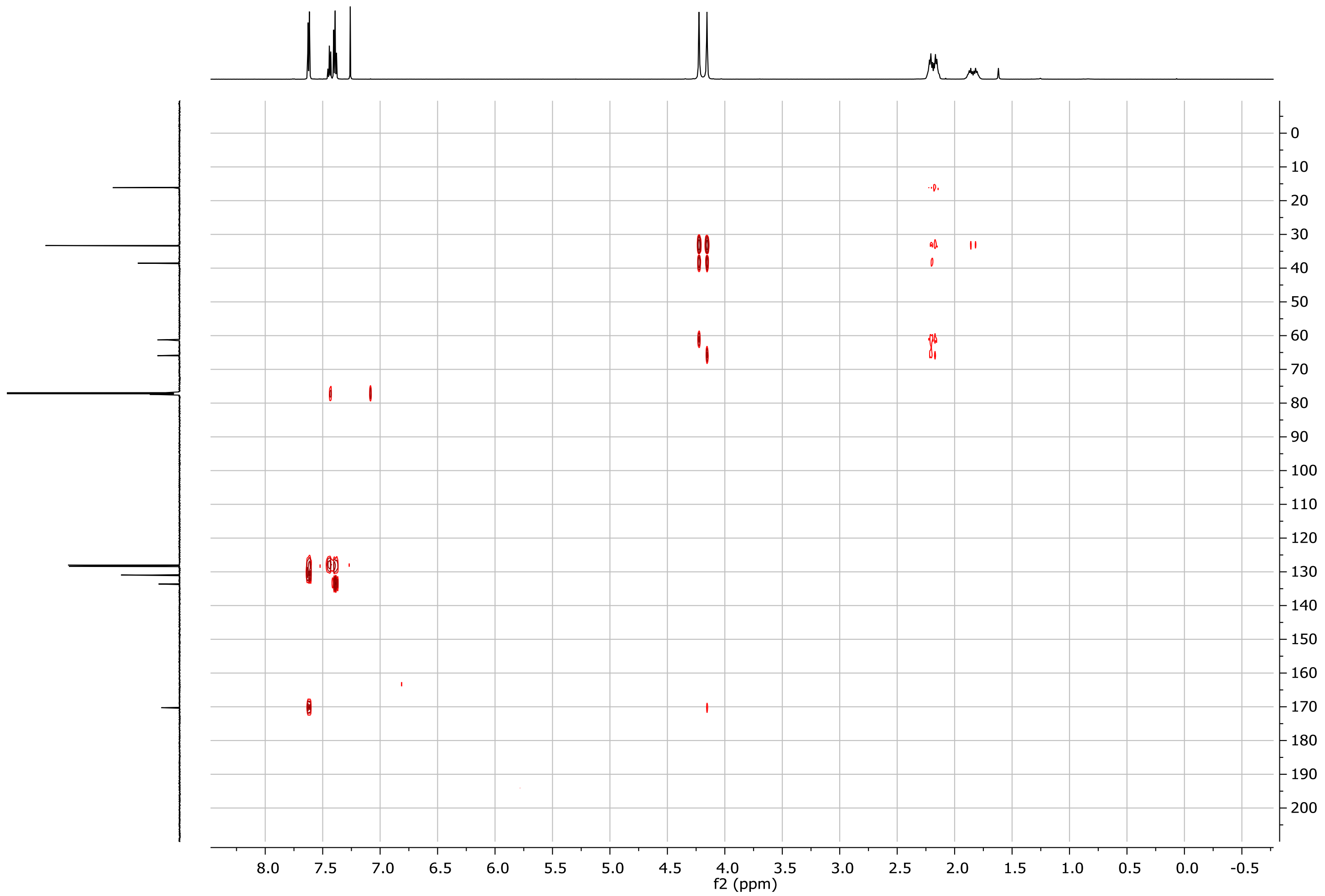

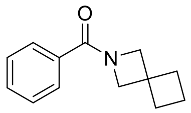

1e

^{1}H, ^{13}C HSQC

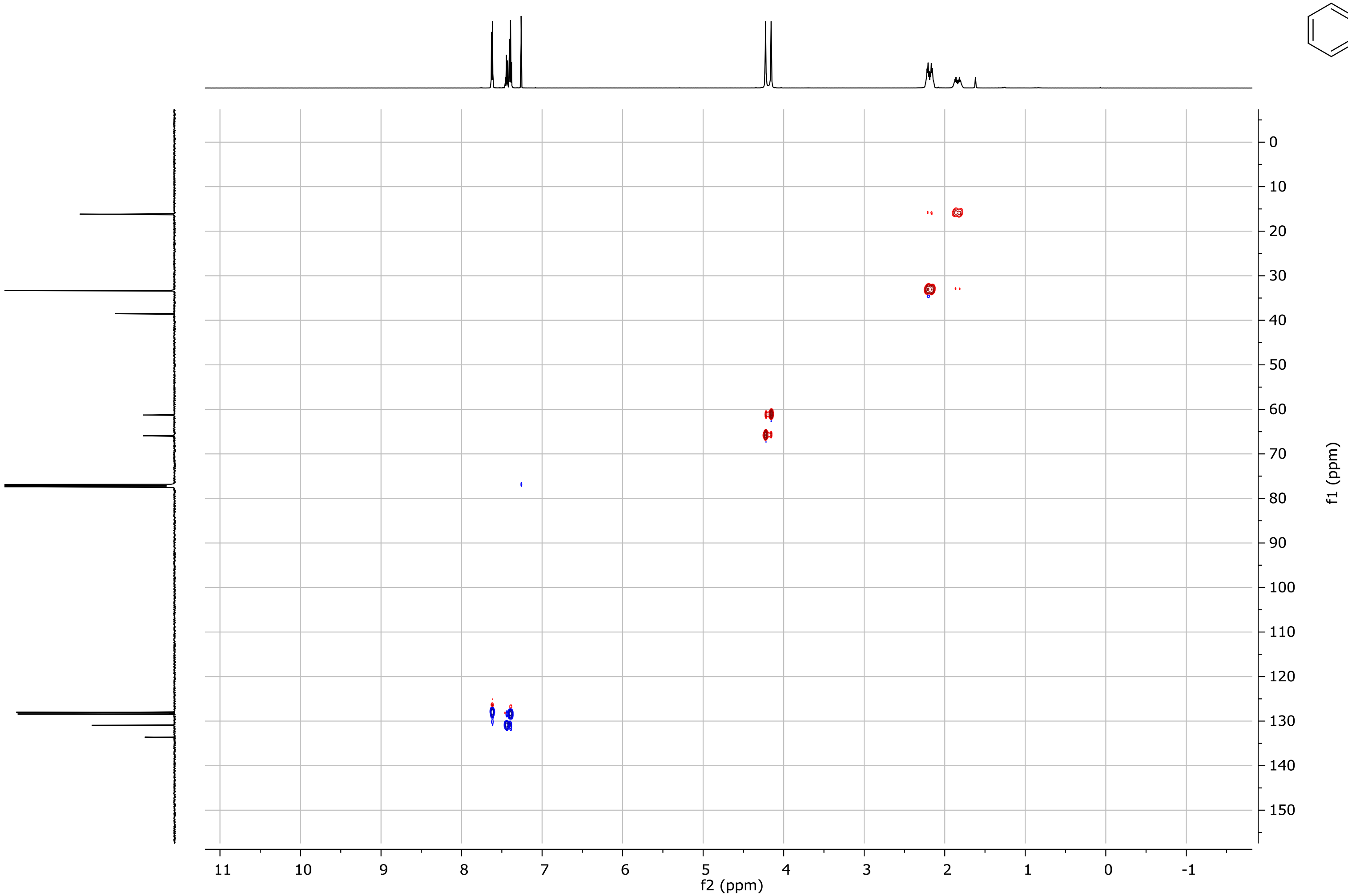

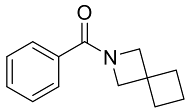

1e

HRMS

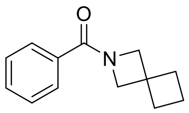
O
N

1e

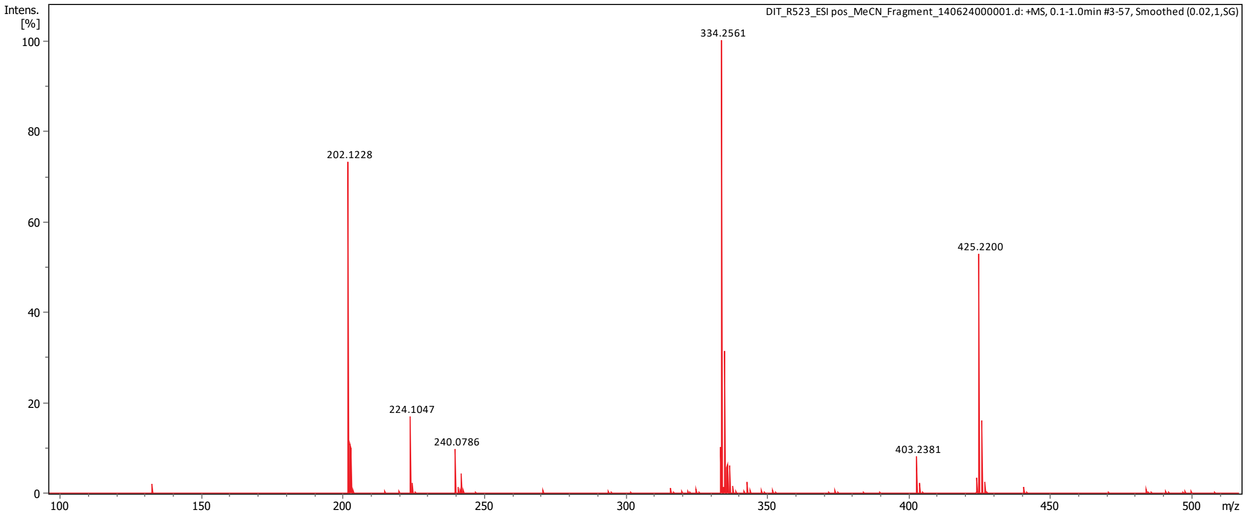
Intens.
[%]
DIT_R523_ESI pos_MeCN_Fragment_140624000001.d: +MS, 0.1-1.0min #3-57, Smoothed (0.02,1,SG)
334.2561
202.1228
425.2200
224.1047
240.0786
403.2381
100
80
60
40
20
0
100
150
200
250
300
350
400
450
500
m/z

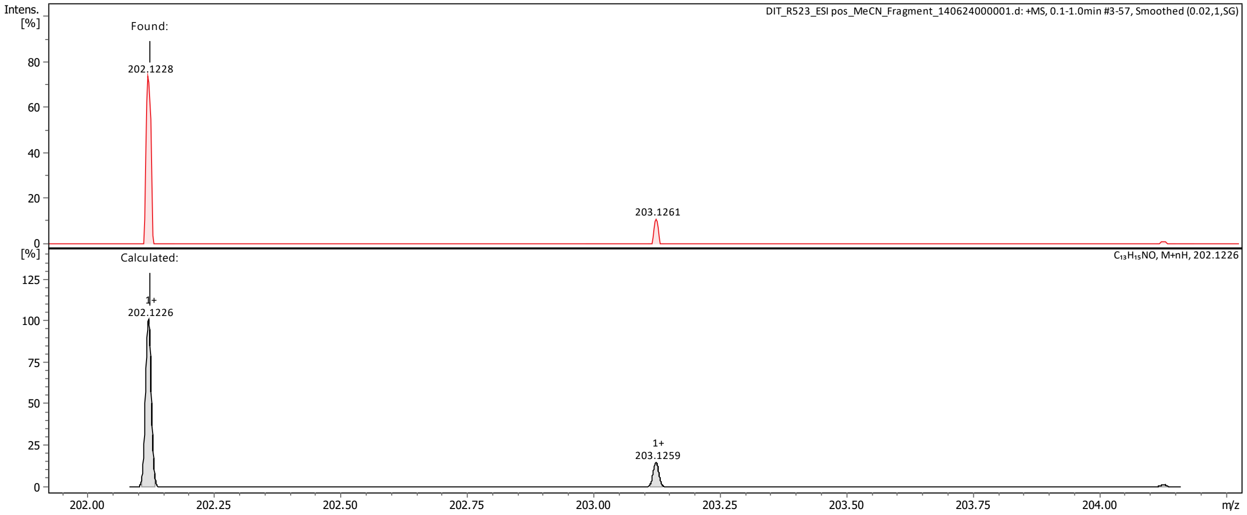
Intens.
[%]
DIT_R523_ESI pos_MeCN_Fragment_140624000001.d: +MS, 0.1-1.0min #3-57, Smoothed (0.02,1,SG)
Found:
202.1228
203.1261
80
60
40
20
0
[%]
Calculated:
$C_{13}H_{15}NO$, M+nH, 202.1226
1+
202.1226
1+
203.1259
125
100
75
50
25
0
202.00
202.25
202.50
202.75
203.00
203.25
203.50
203.75
204.00
m/z

IR

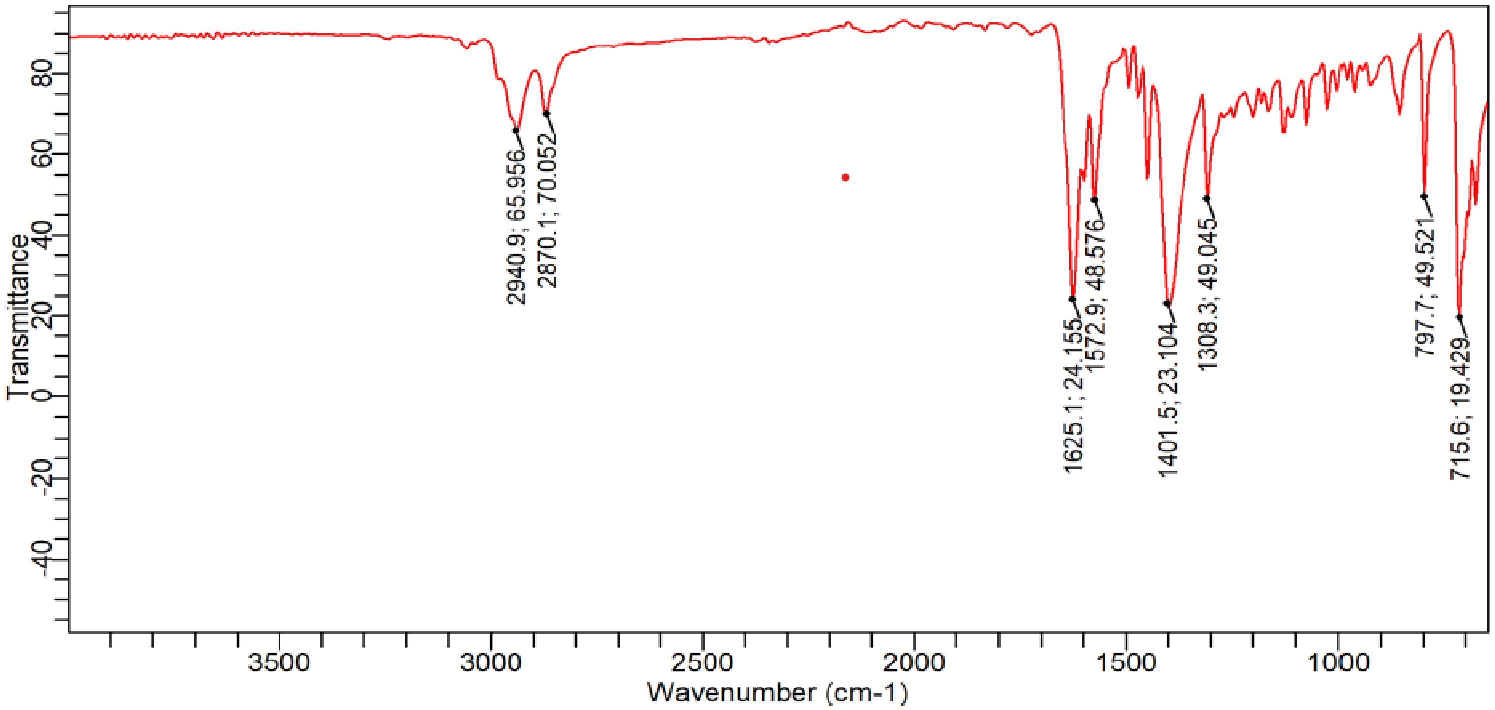

Transmittance
80
60
40
20
0
-20
-40
3500
3000
2500
2000
1500
1000
Wavenumber (cm-1)
2940.9; 65.956
2870.1; 70.052
1625.1; 24.155
1572.9; 48.576
1401.5; 23.104
1308.3; 49.045
797.7; 49.521
715.6; 19.429

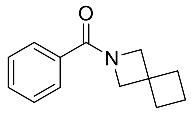

O
N

1e

6 Phenyl(thiomorpholino)methanone (1g)

^{1}H NMR

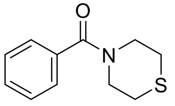

1g

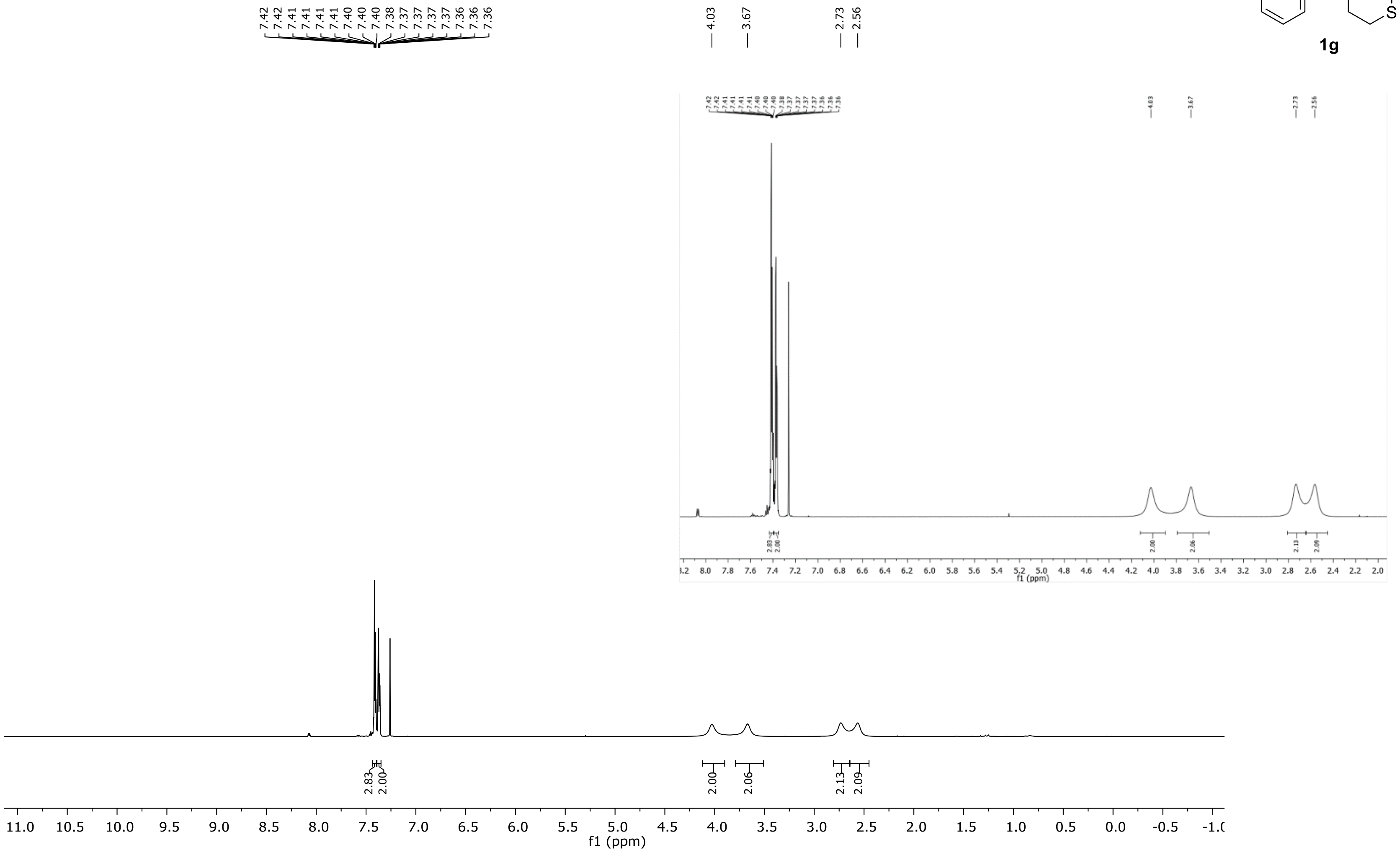

^{13}C NMR

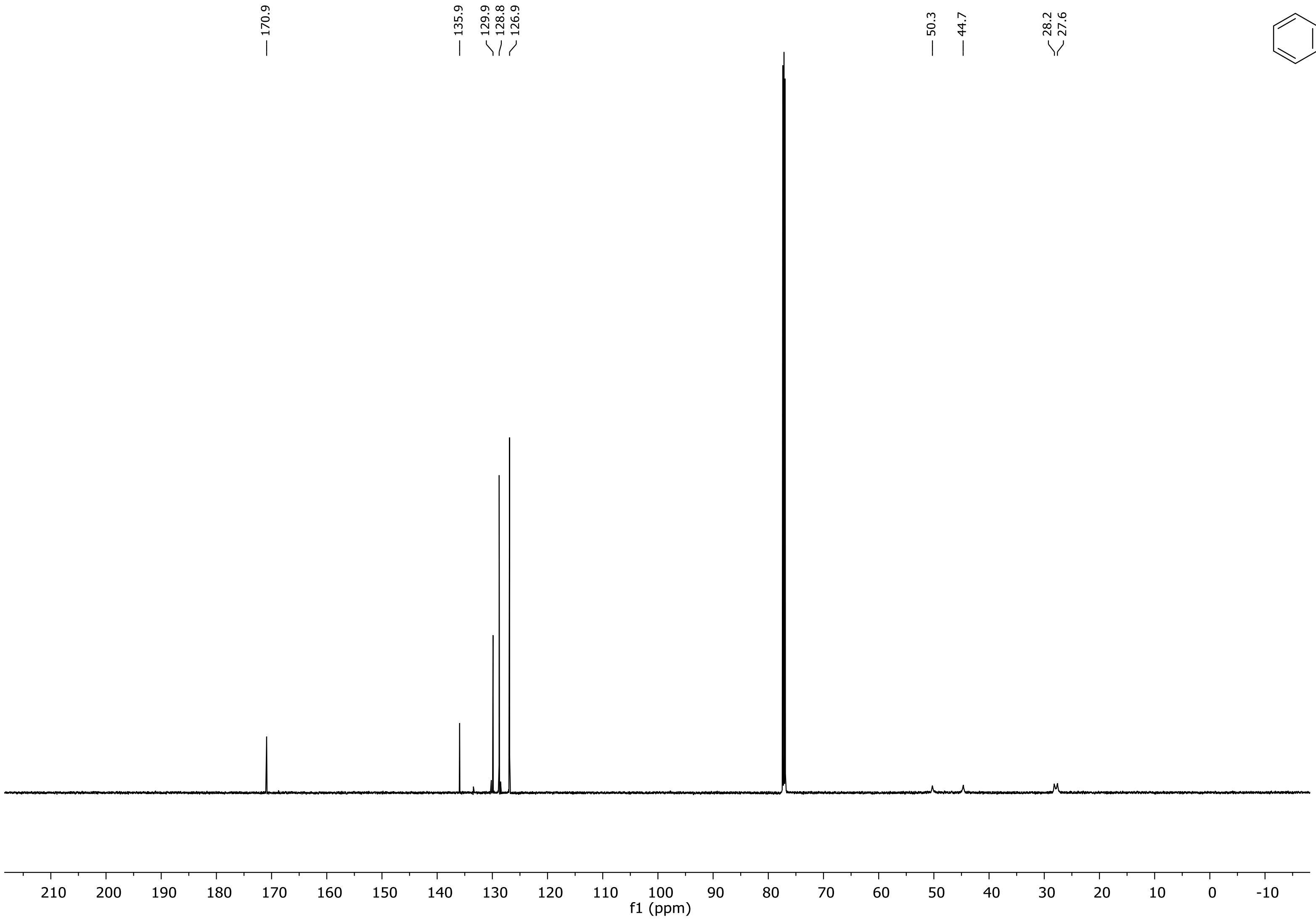

170.9
135.9
129.9
128.8
126.9
50.3
44.7
28.2
27.6
210 200 190 180 170 160 150 140 130 120 110 100 90 80 70 60 50 40 30 20 10 0 -10
f1 (ppm)

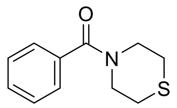

O
N
S

1g

^{1}H, ^{1}H COSY

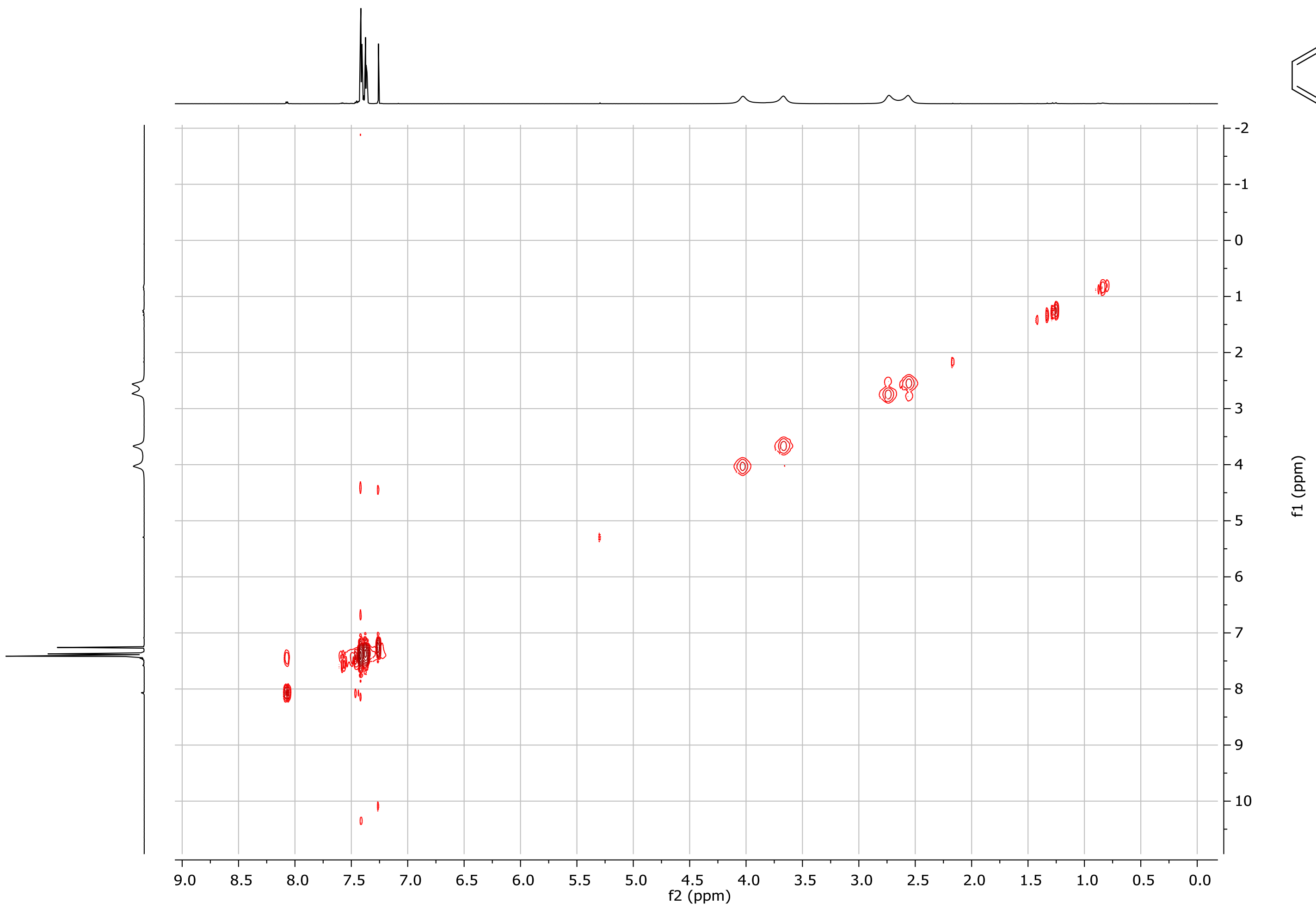
f2 (ppm)
f1 (ppm)
9.0
8.5
8.0
7.5
7.0
6.5
6.0
5.5
5.0
4.5
4.0
3.5
3.0
2.5
2.0
1.5
1.0
0.5
0.0
-2
-1
0
1
2
3
4
5
6
7
8
9
10

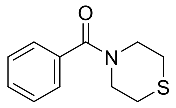
O
N
S

1g

^{1}H, ^{13}C HMBC

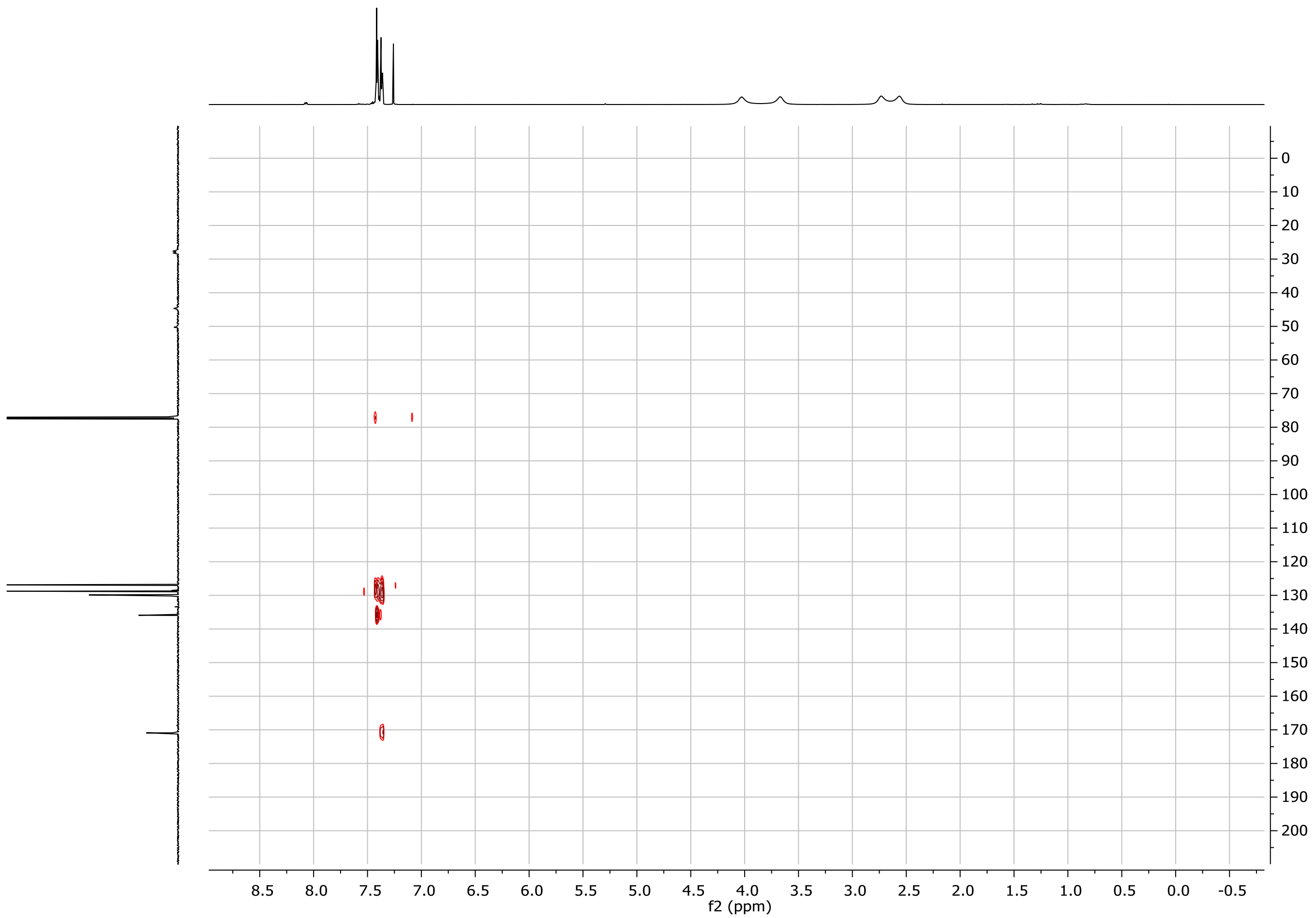

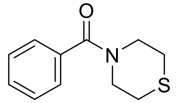

1g

^{1}H, ^{13}C HSQC

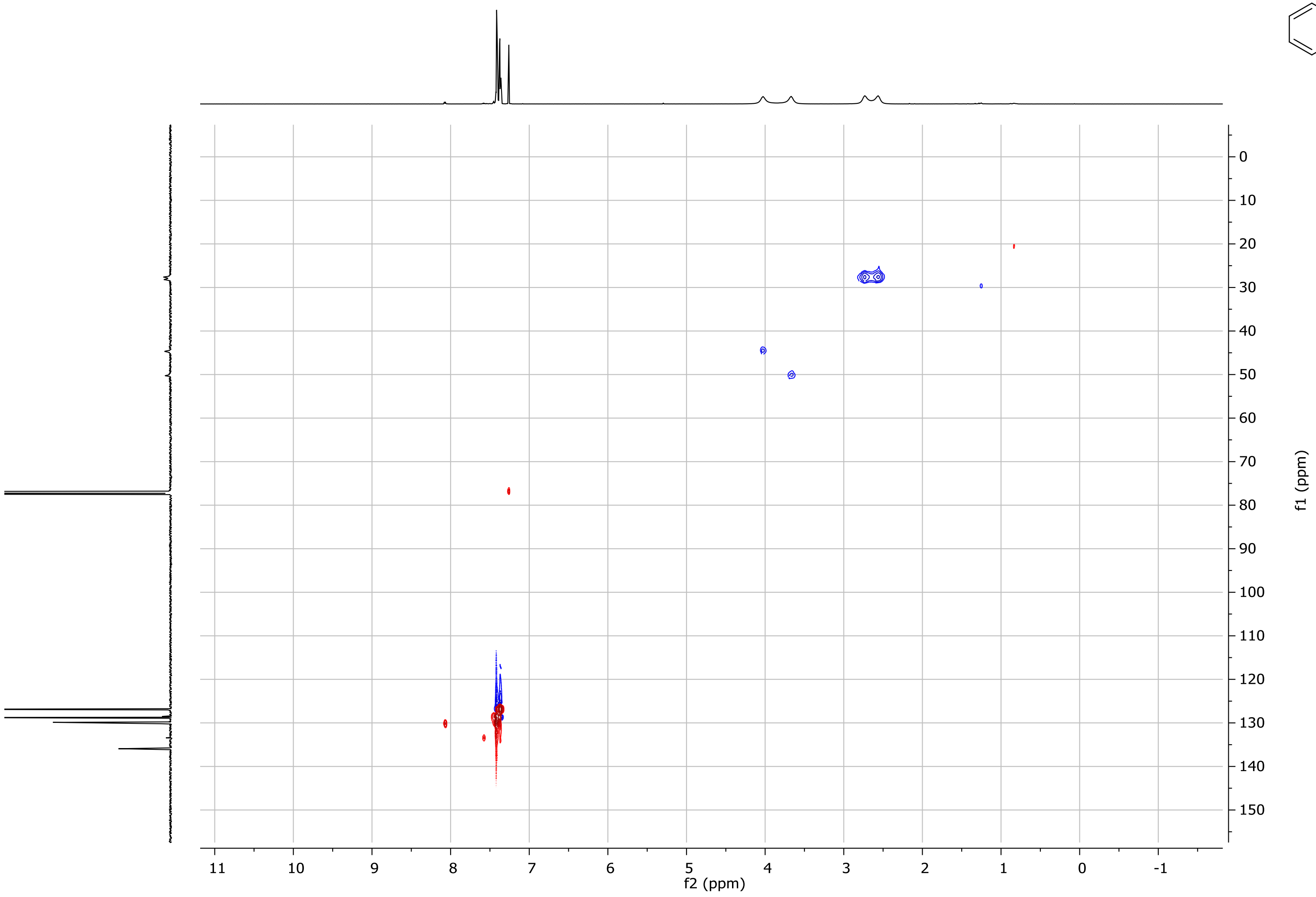

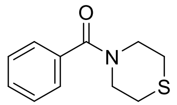

1g

HRMS

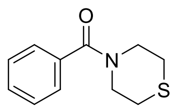

1g

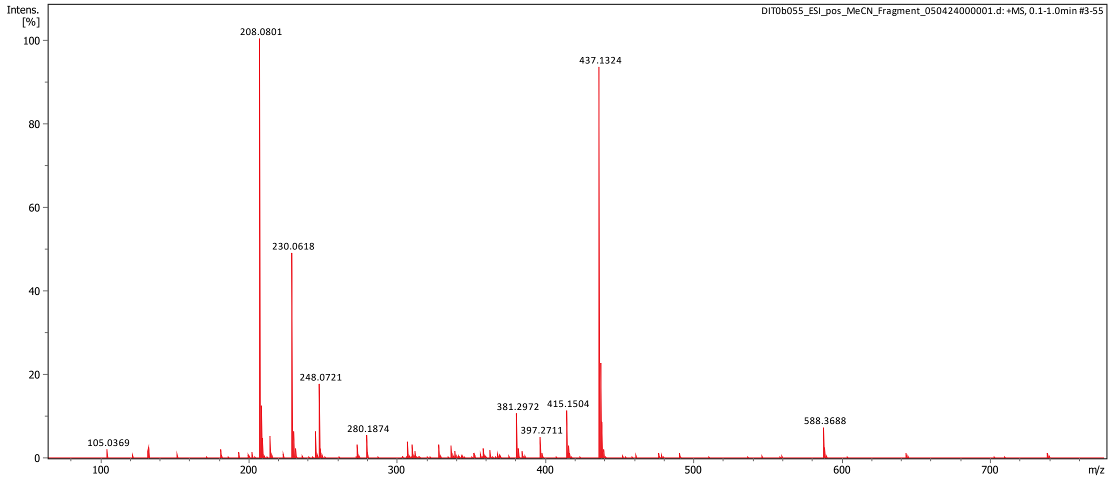
DIT0b055_ESI_pos_MeCN_Fragment_050424000001.d: +MS, 0.1-1.0min #3-55
Intens. [%]
208.0801
437.1324
230.0618
248.0721
280.1874
381.2972
415.1504
397.2711
588.3688
105.0369
m/z

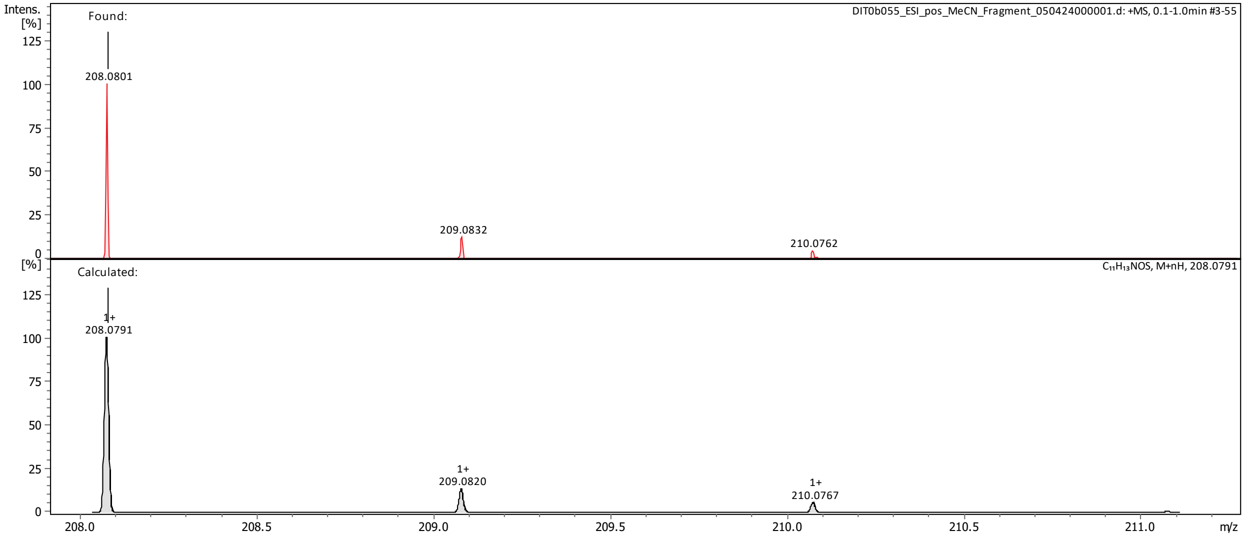
Found:
DIT0b055_ESI_pos_MeCN_Fragment_050424000001.d: +MS, 0.1-1.0min #3-55
208.0801
209.0832
210.0762
Calculated:
C11H13NOS, M+nH, 208.0791
1+
208.0791
1+
209.0820
1+
210.0767
m/z

IR

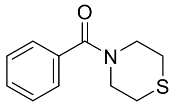

1g

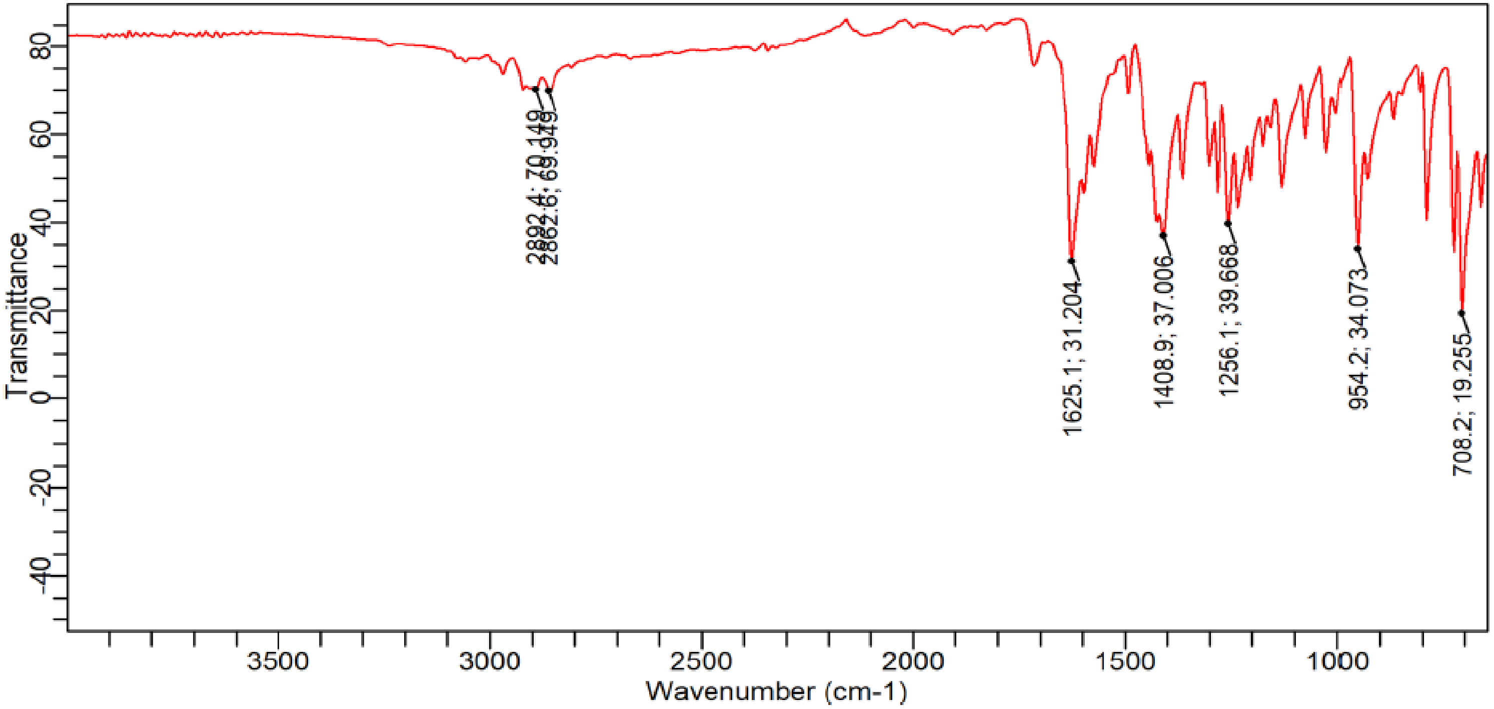
Transmittance
80
60
40
20
0
-20
-40
3500
3000
2500
2000
1500
1000
Wavenumber (cm-1)
2892.4; 70.149
2862.6; 69.949
1625.1; 31.204
1408.9; 37.006
1256.1; 39.668
954.2; 34.073
708.2; 19.255

7 2-Oxa-6-azaspiro[3.3]hept-6-ylphenylmethanone (1h)

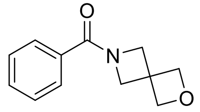

1h

^{1}H NMR

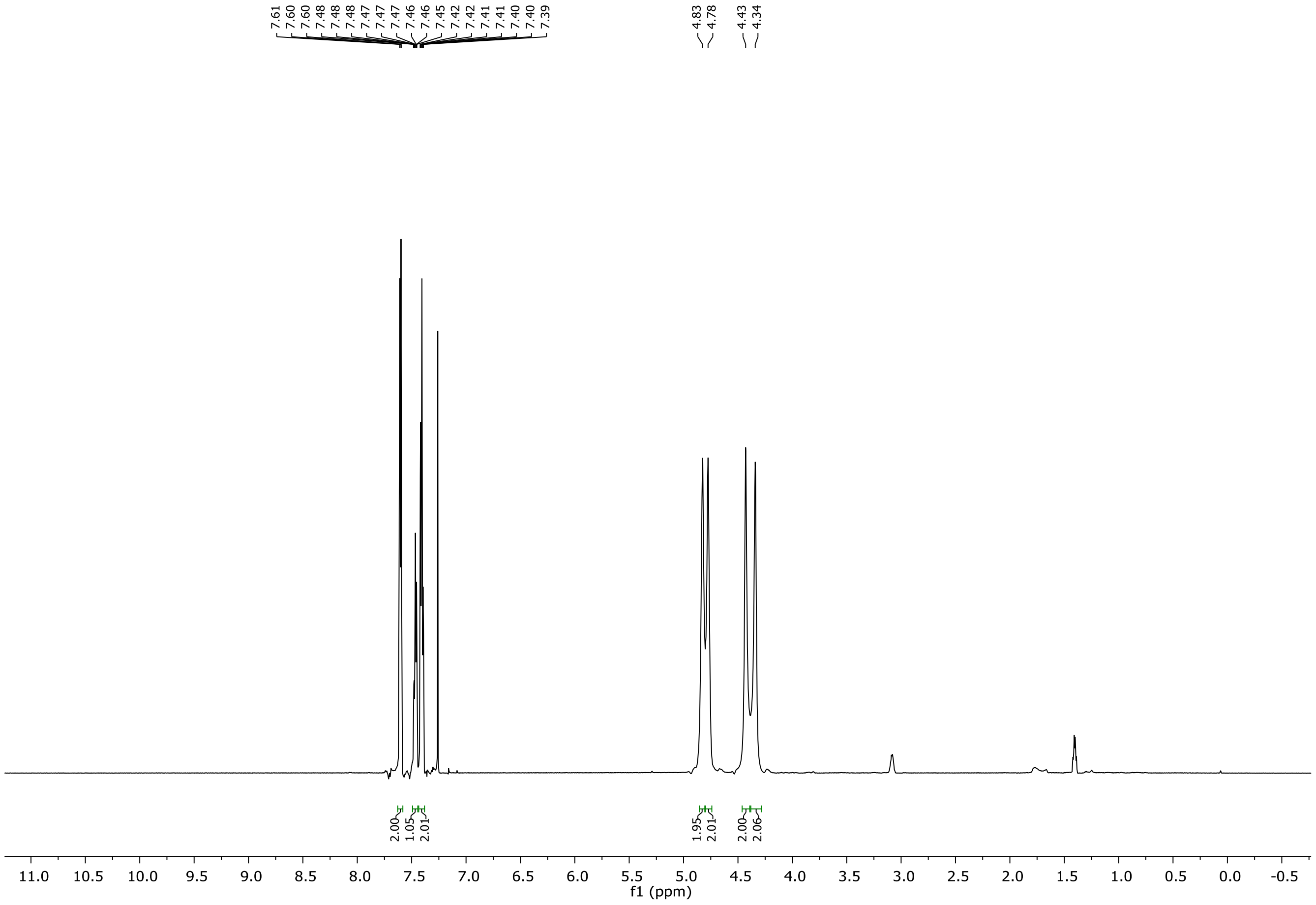

^{13}C NMR

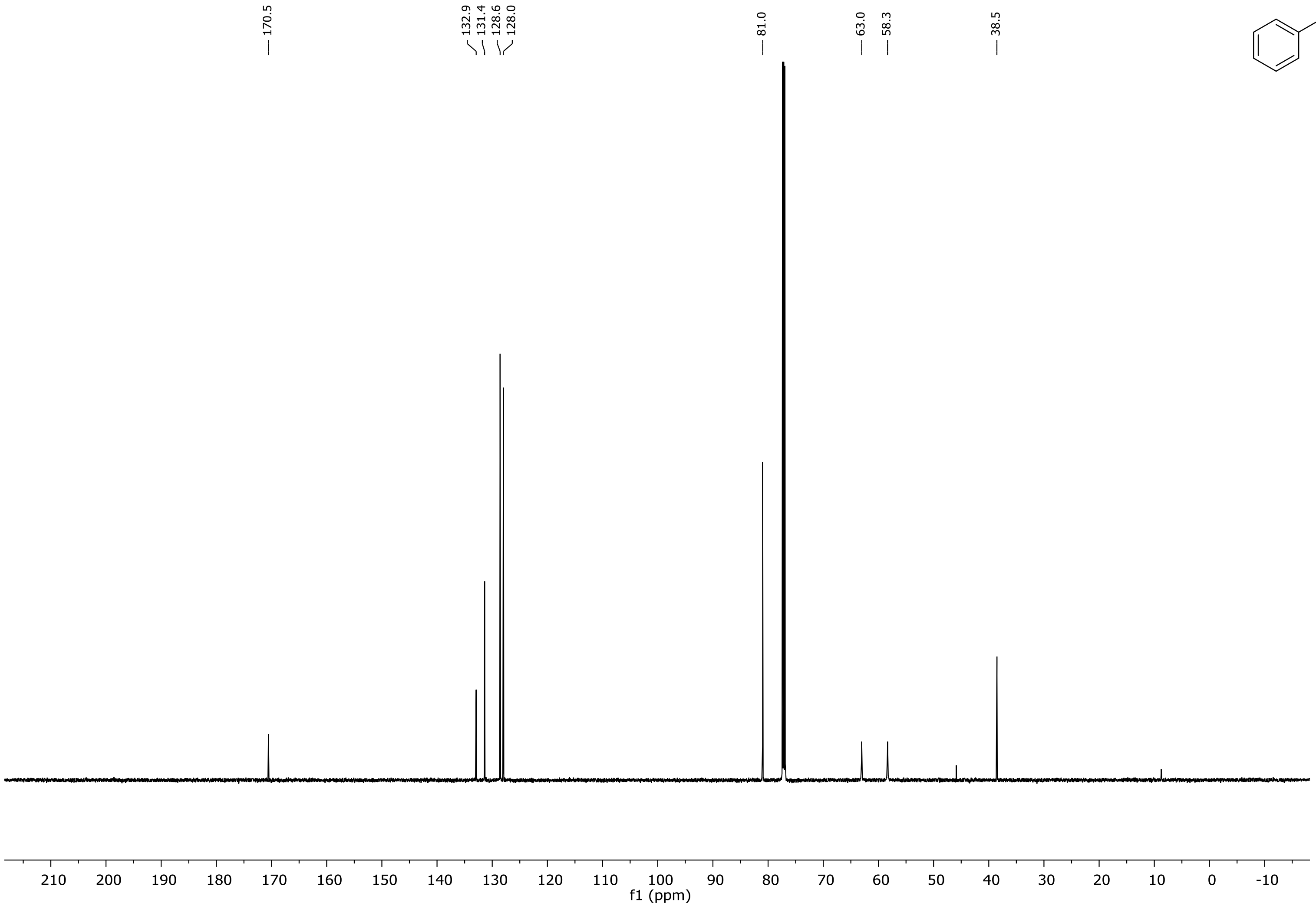
170.5
132.9
131.4
128.6
128.0
81.0
63.0
58.3
38.5
210 200 190 180 170 160 150 140 130 120 110 100 90 80 70 60 50 40 30 20 10 0 -10
f1 (ppm)

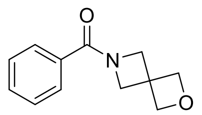
O
N
O

1h

^{1}H, ^{1}H COSY

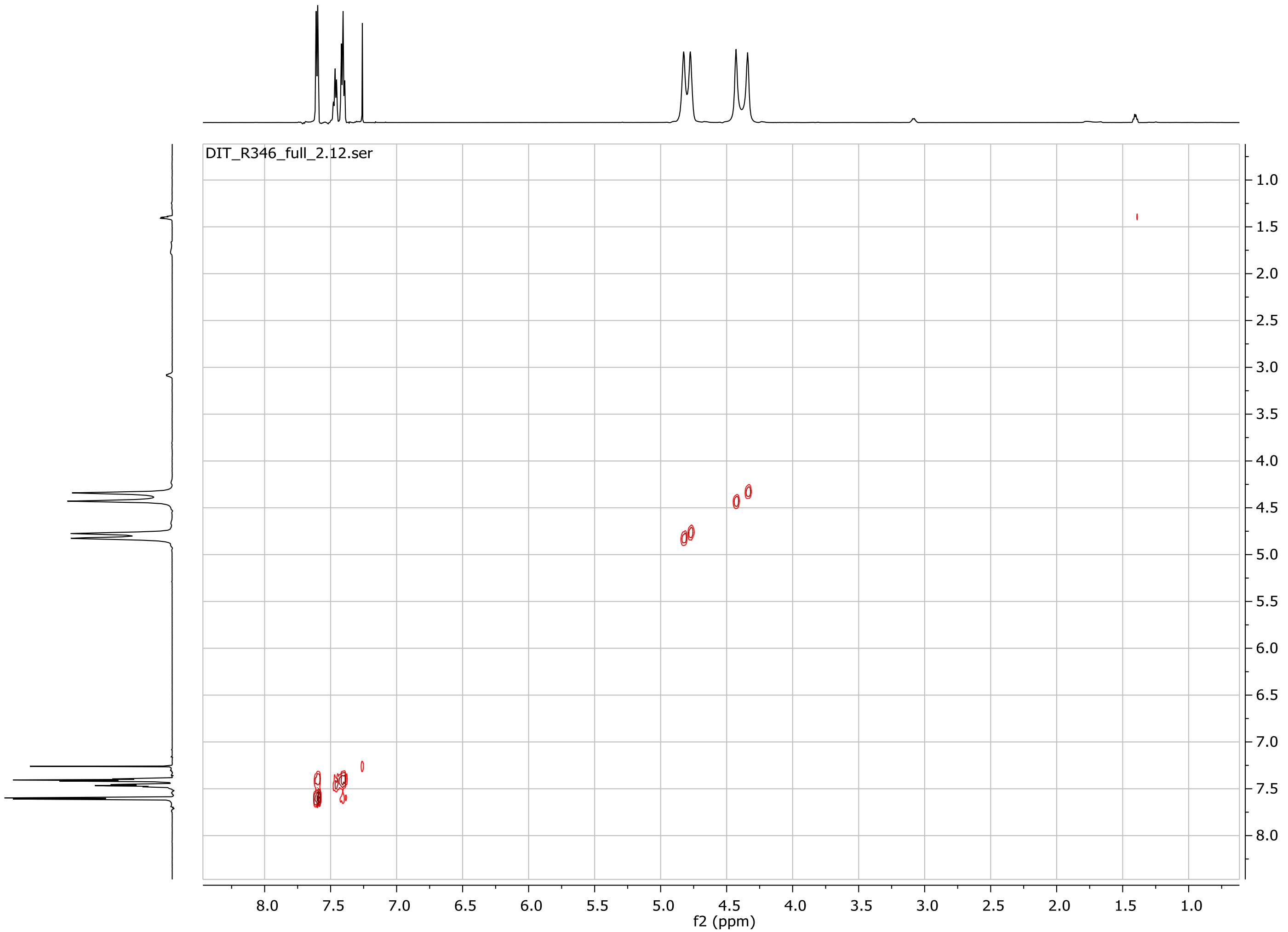

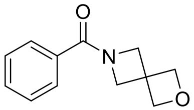

1h

^{1}H, ^{13}C HMBC

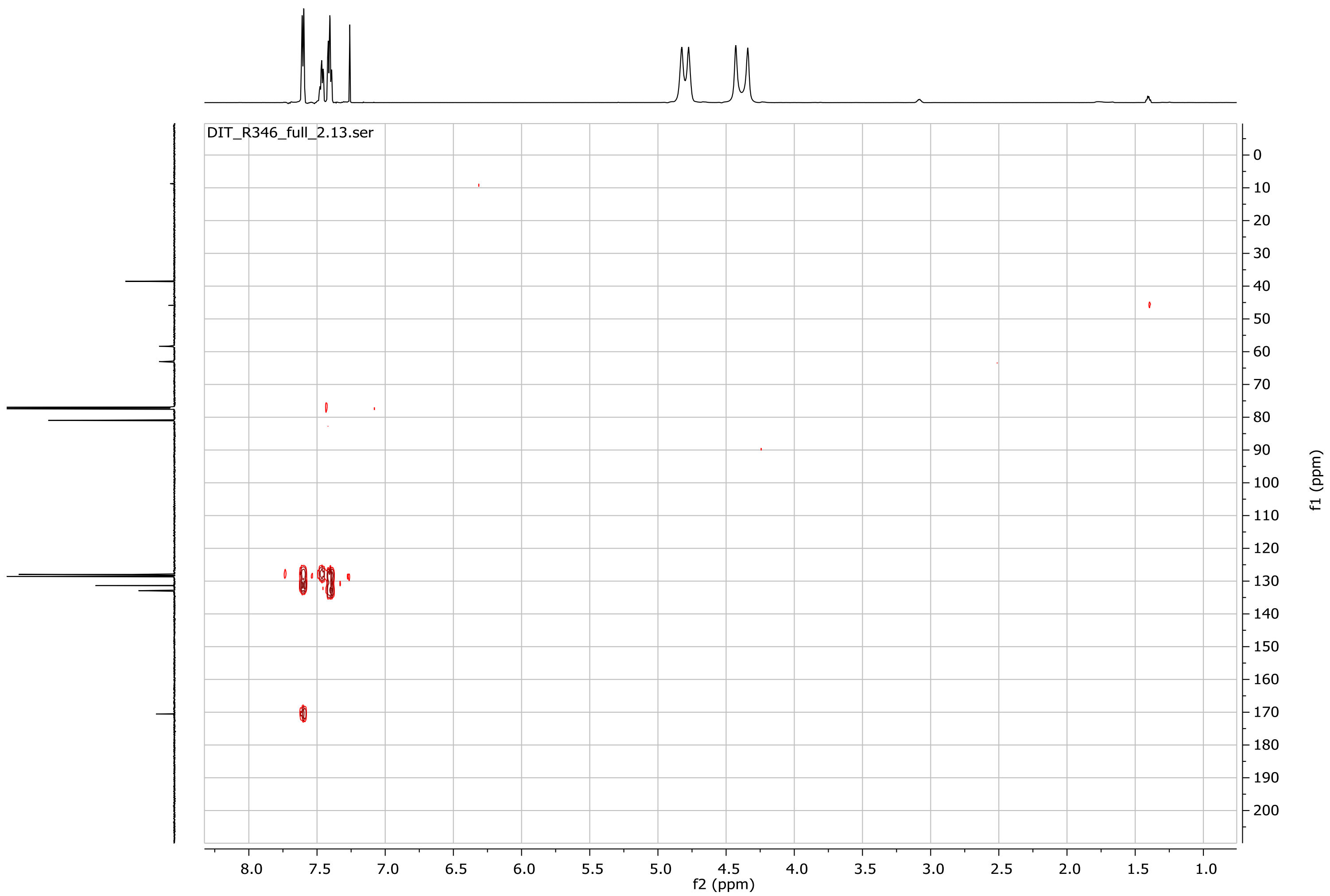

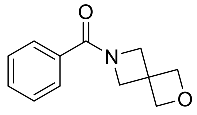

1h

^{1}H, ^{13}C HSQC

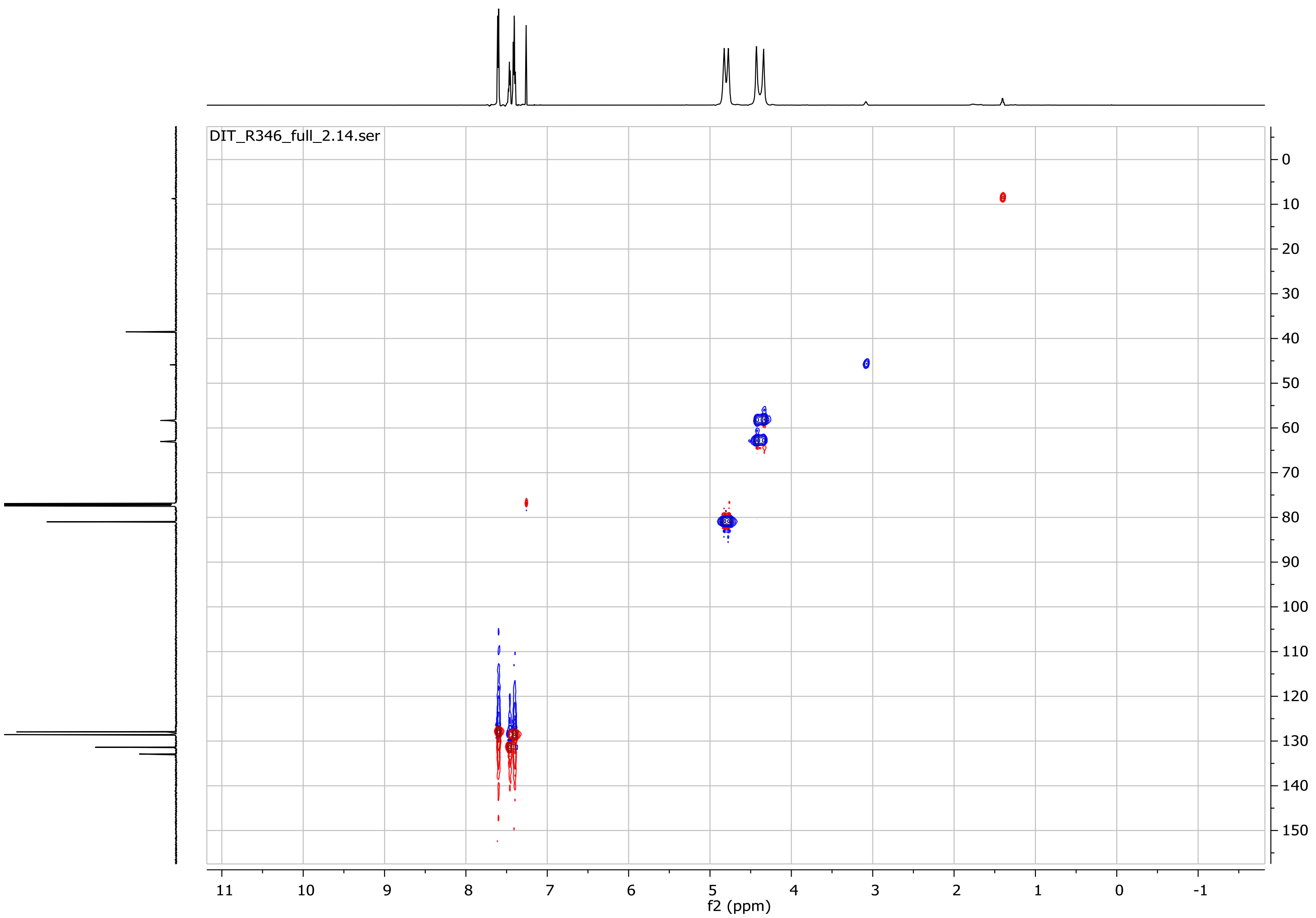

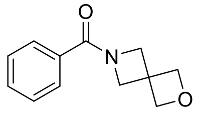

1h

HRMS

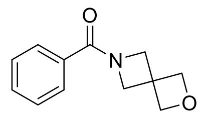

1h

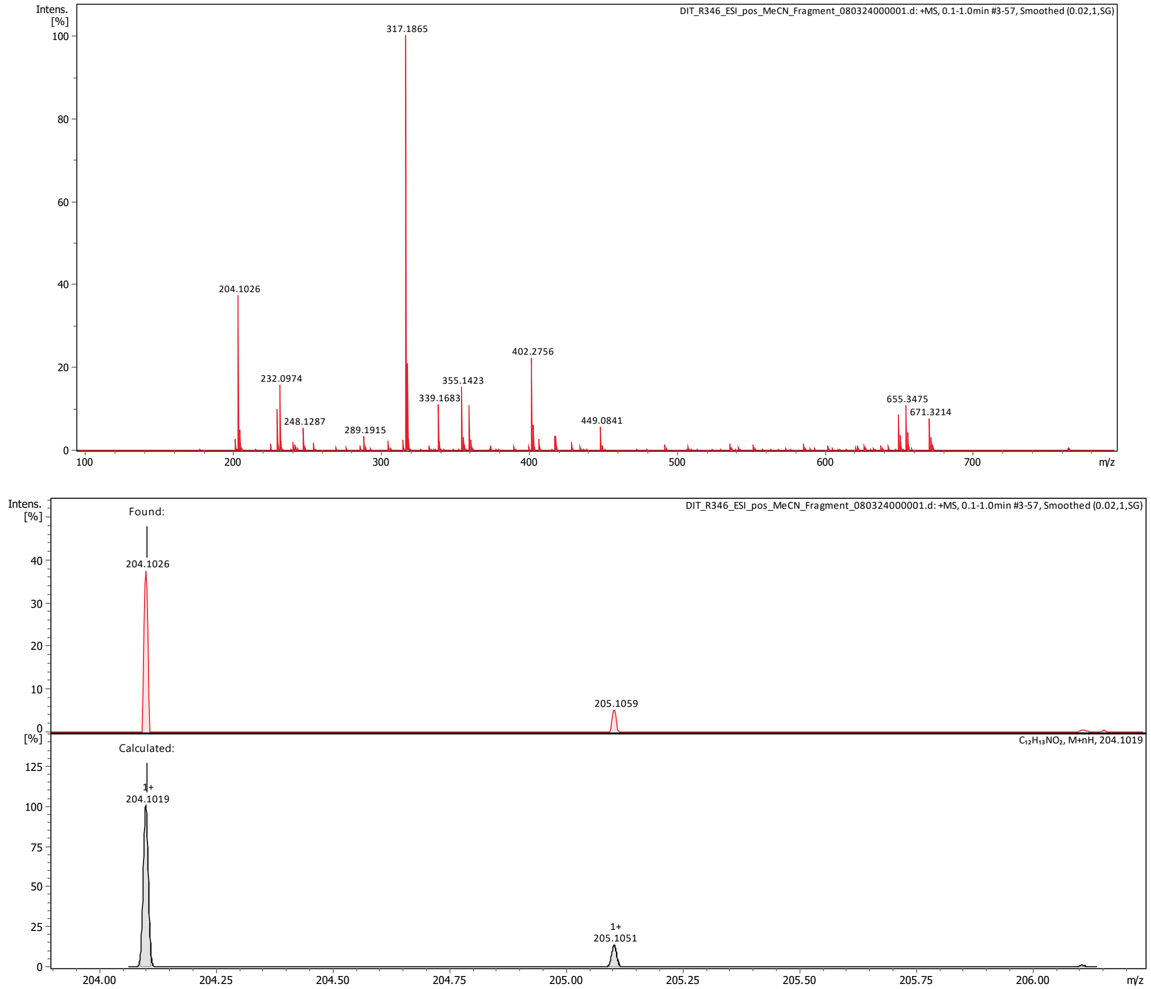
Intens. [%]
DIT_R346_ESI_pos_MeCN_Fragment_080324000001.d: +MS, 0.1-1.0min #3-57, Smoothed (0.02,1,SG)
317.1865
204.1026
232.0974
248.1287
289.1915
339.1683
355.1423
402.2756
449.0841
655.3475
671.3214
m/z
Found:
204.1026
205.1059
Calculated:
$C_{12}H_{13}NO_2$, M+nH, 204.1019
1+ 204.1019
1+ 205.1051

IR

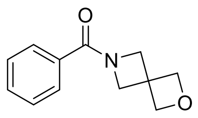

O
N
O

1h

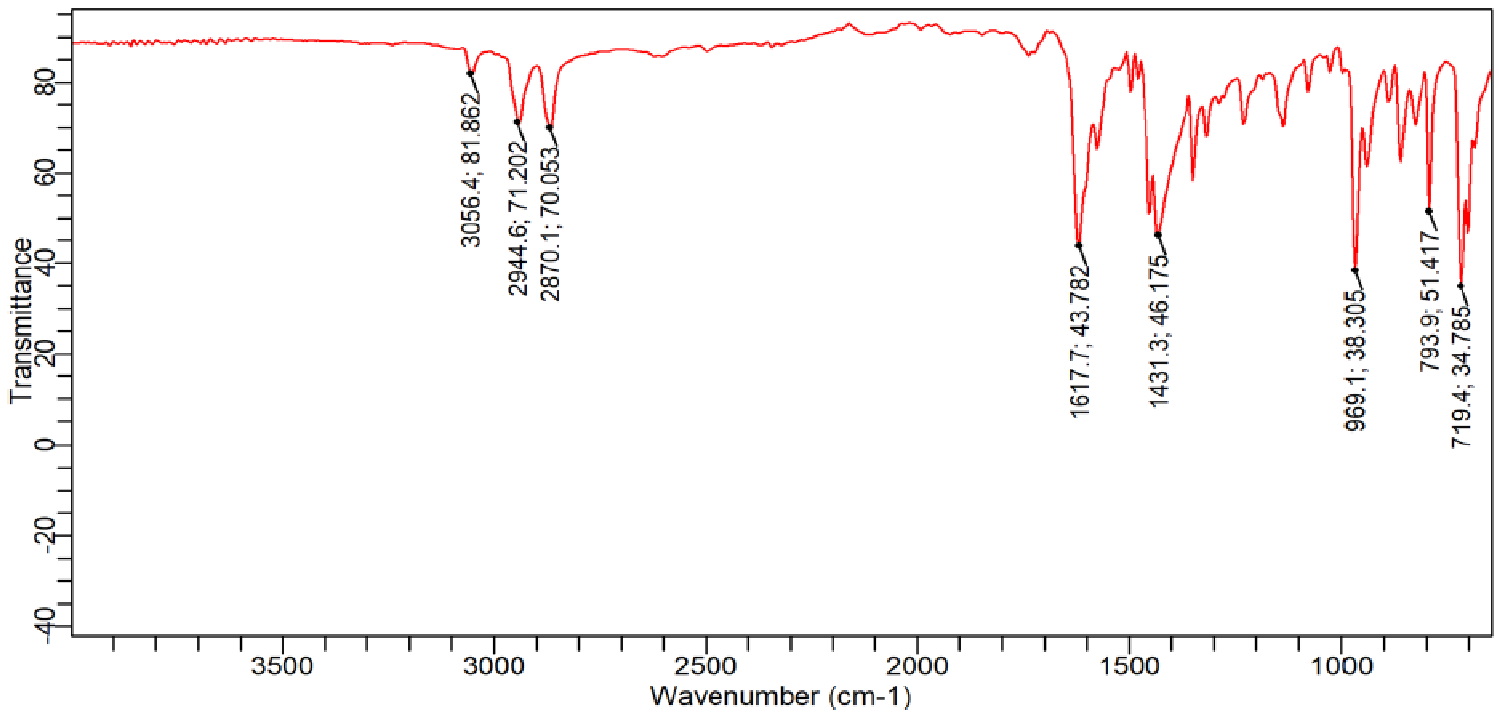

3056.4; 81.862
2944.6; 71.202
2870.1; 70.053
1617.7; 43.782
1431.3; 46.175
969.1; 38.305
793.9; 51.417
719.4; 34.785
Transmittance
Wavenumber (cm-1)

8 Phenyl(4-phenylpiperazin-1-yl)methanone (1i)

¹H NMR

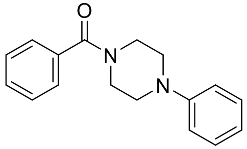

1i

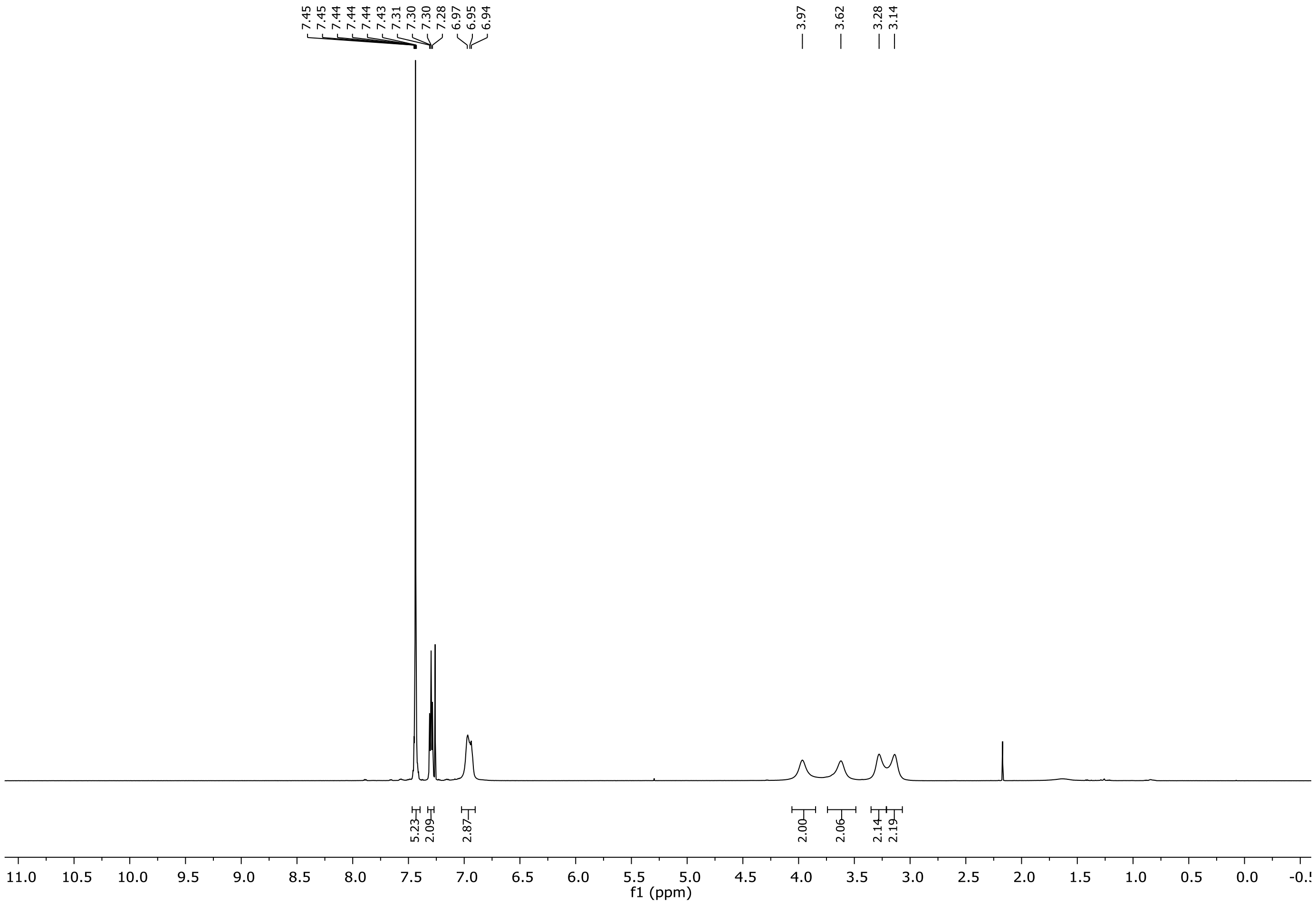

^{13}C NMR

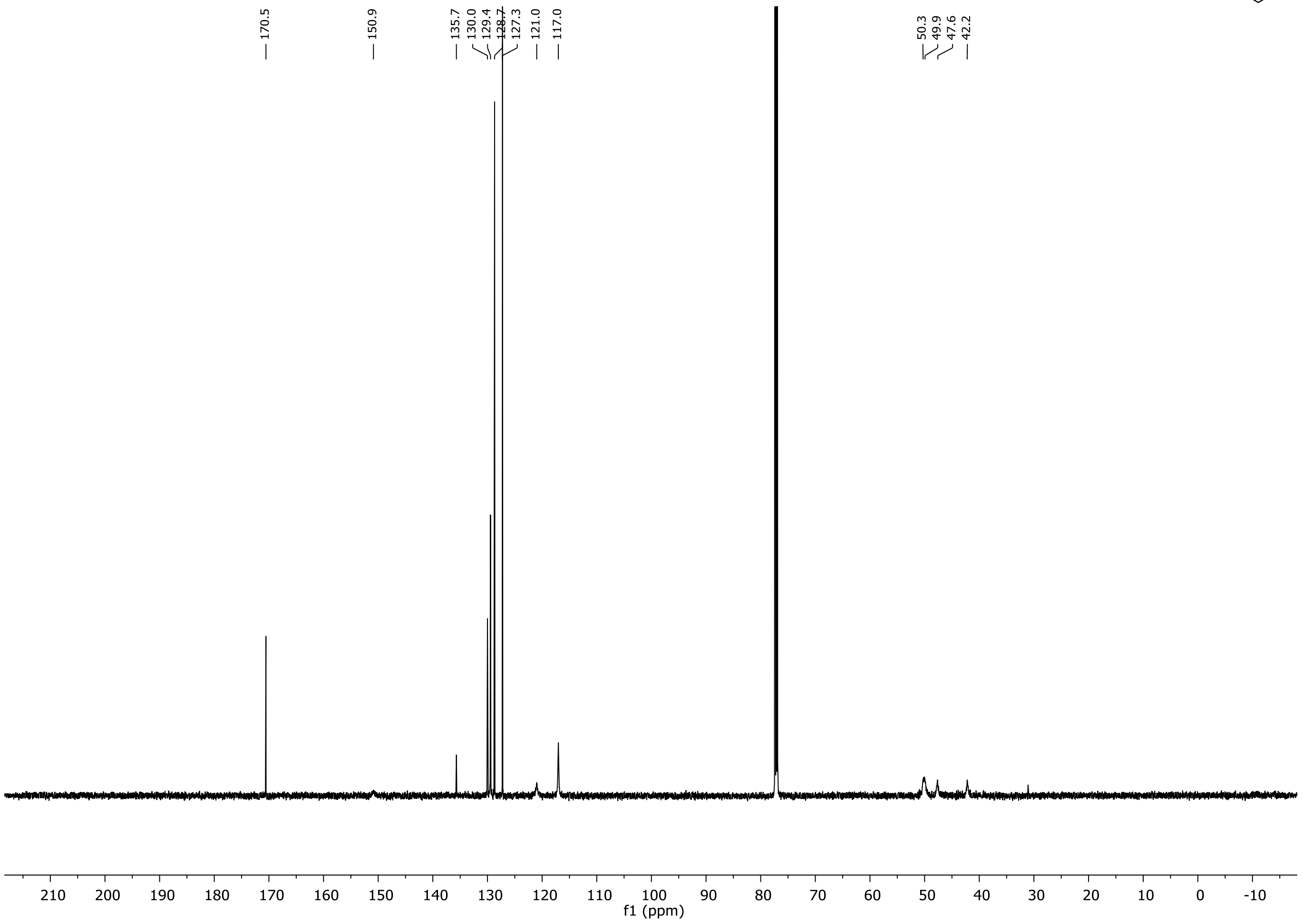
170.5
150.9
135.7
130.0
129.4
128.7
127.3
121.0
117.0
50.3
49.9
47.6
42.2
210 200 190 180 170 160 150 140 130 120 110 100 90 80 70 60 50 40 30 20 10 0 -10
f1 (ppm)

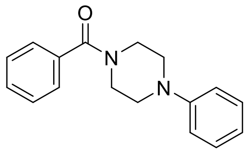
O
N
N

1i

^1H, ^1H COSY

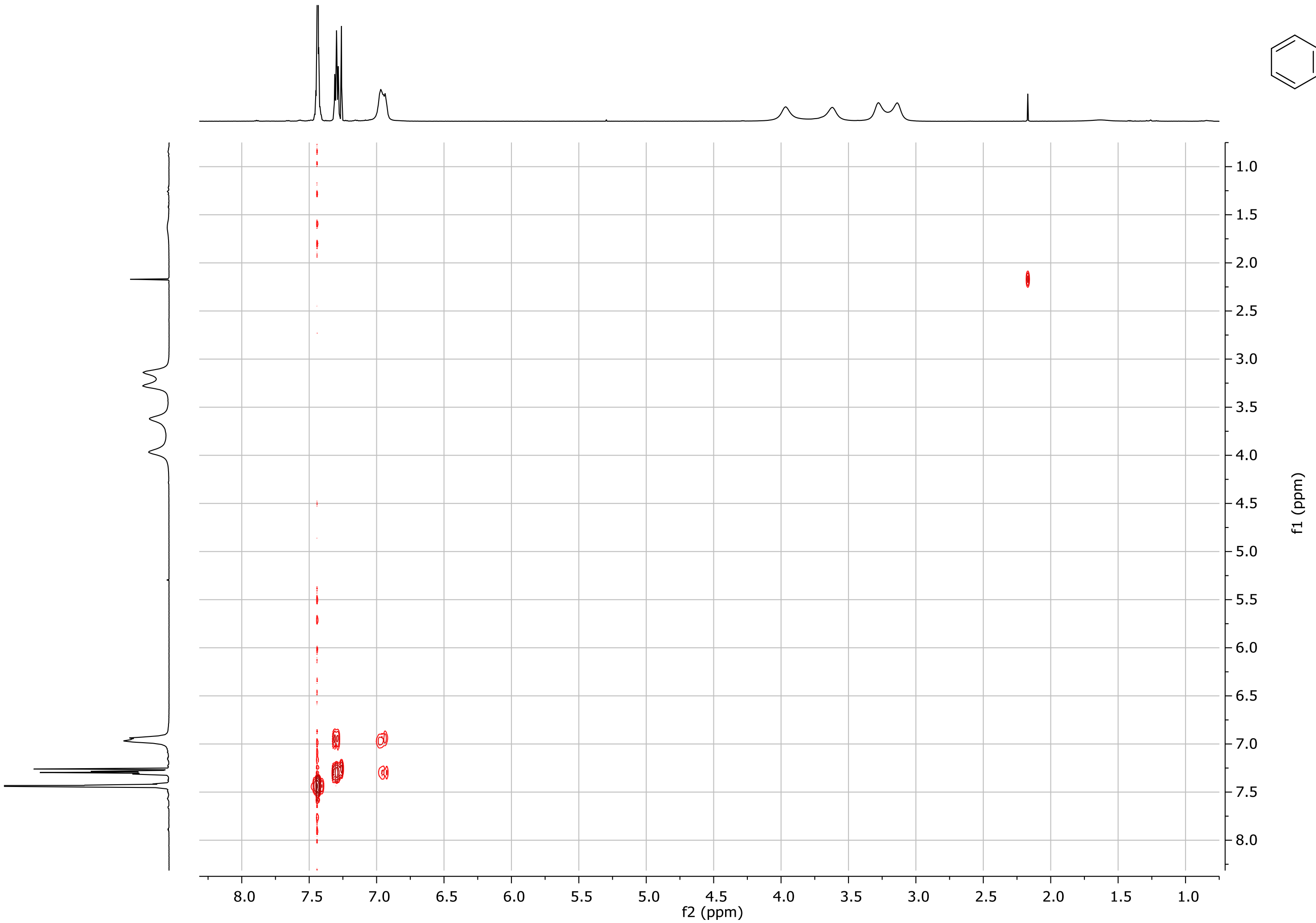

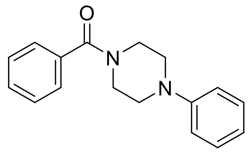

1i

^{1}H, ^{13}C HMBC

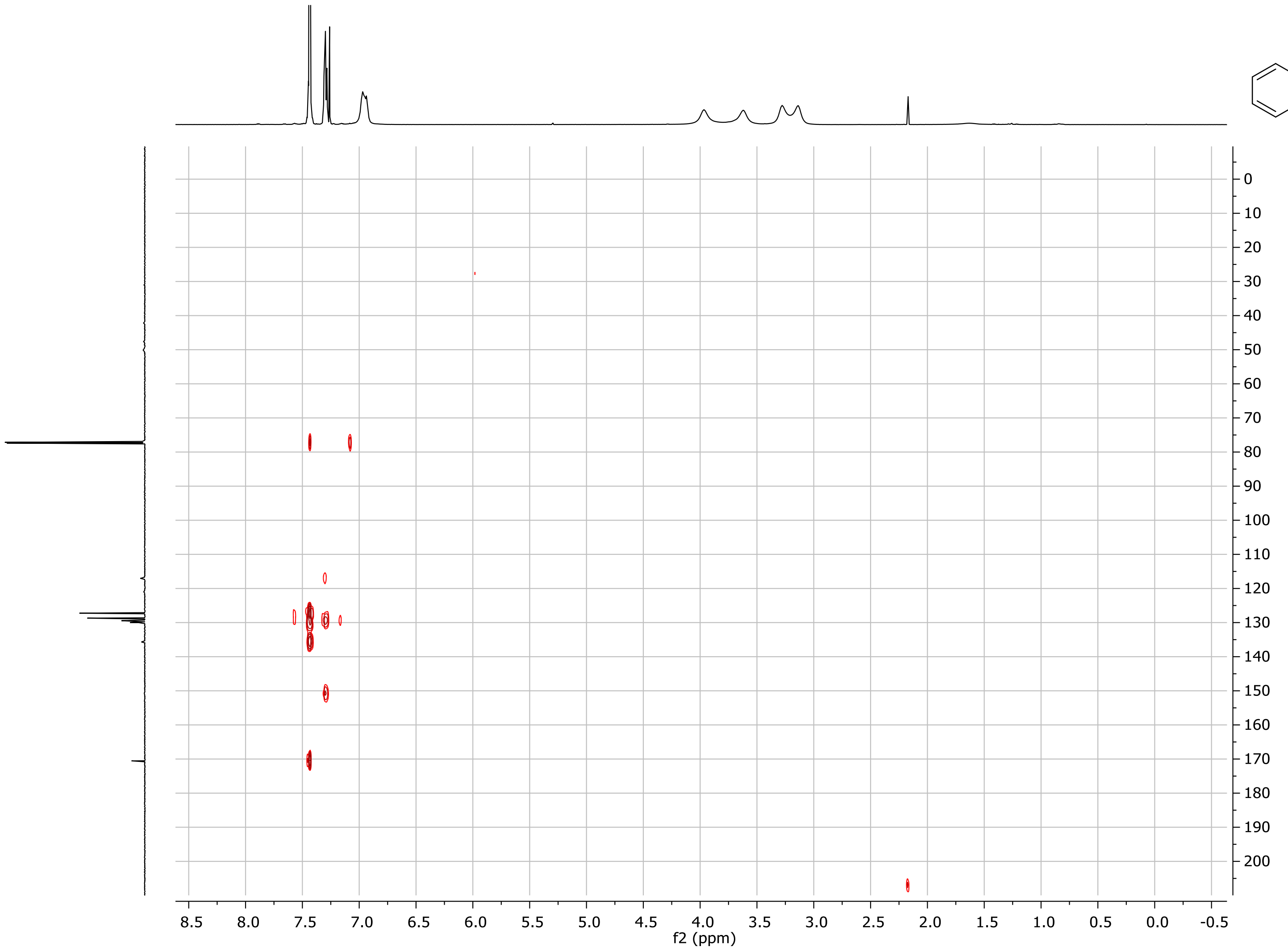

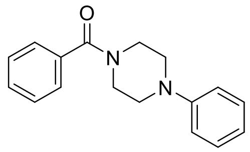

1i

^{1}H, ^{13}C HSQC

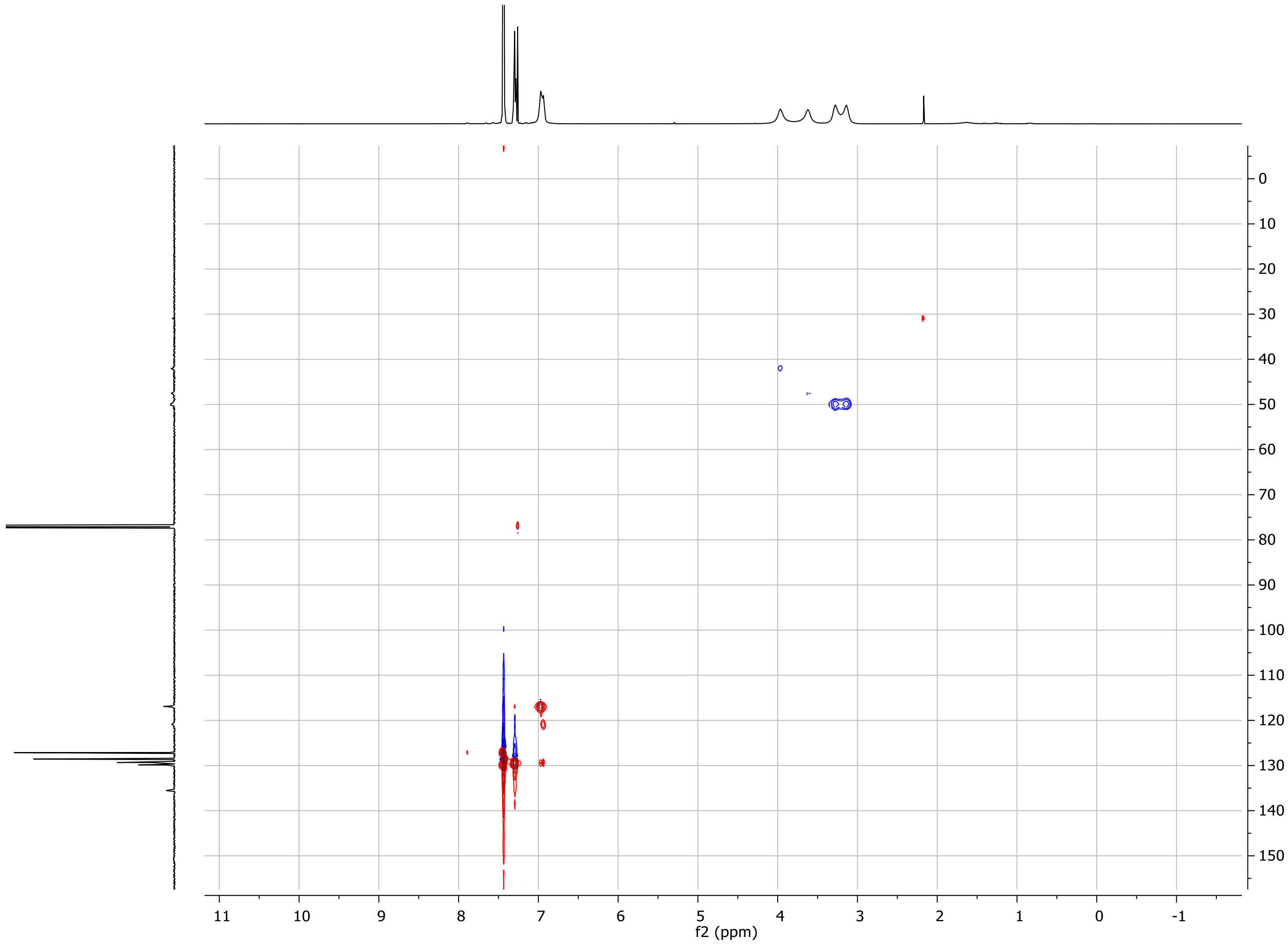

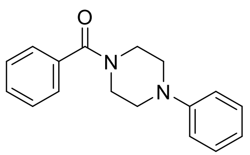

1i

HRMS

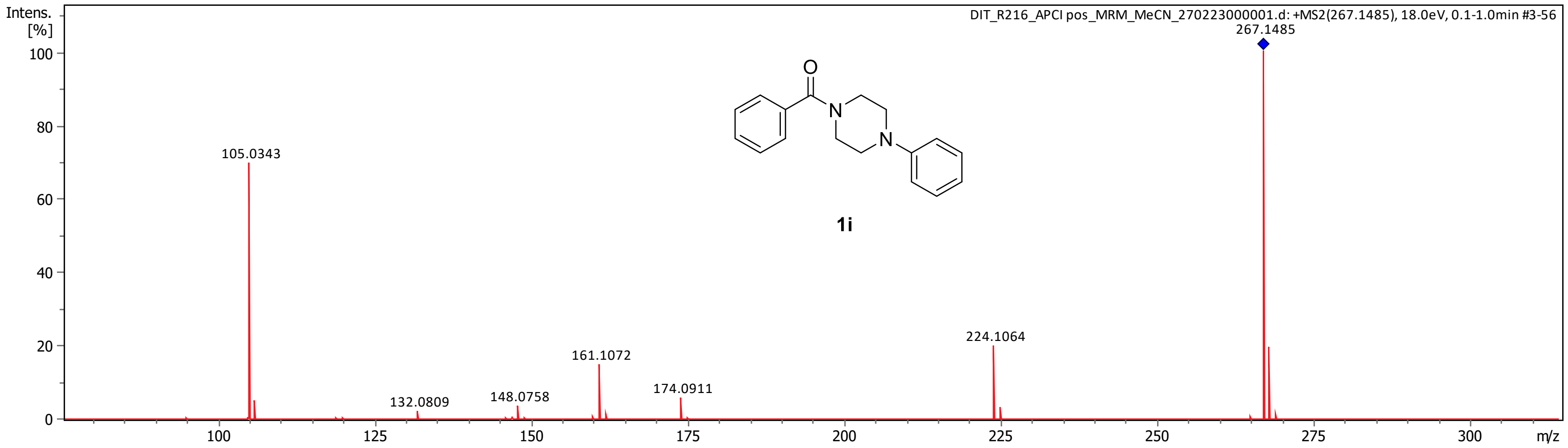

Intens. [%]
DIT_R216_APCI pos_MRM_MeCN_270223000001.d: +MS2(267.1485), 18.0eV, 0.1-1.0min #3-56
267.1485
105.0343
132.0809
148.0758
161.1072
174.0911
224.1064
1i
m/z

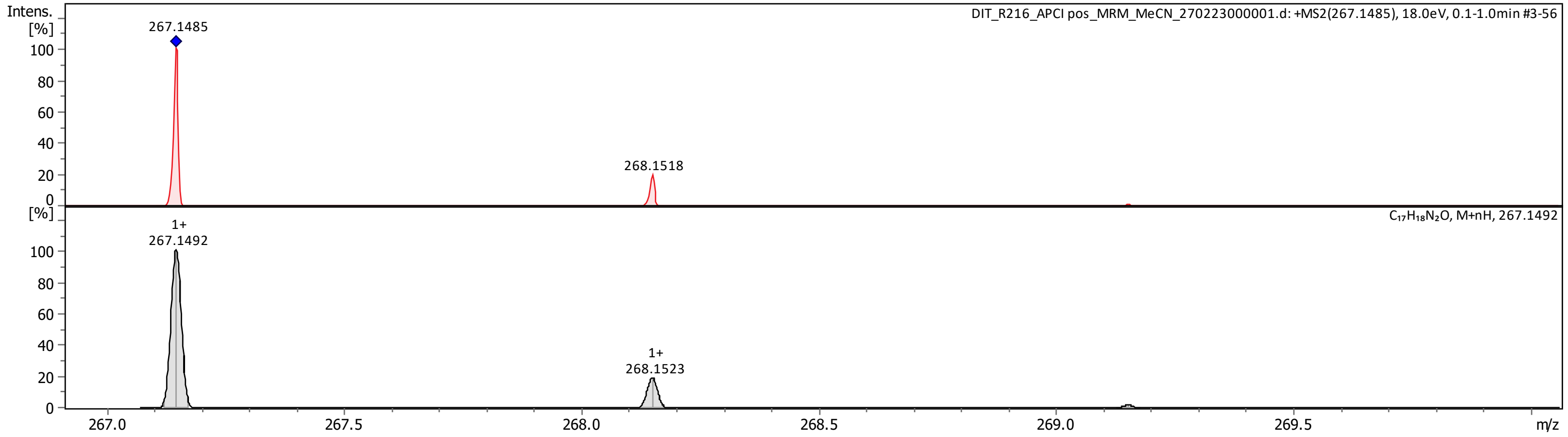

Intens. [%]
DIT_R216_APCI pos_MRM_MeCN_270223000001.d: +MS2(267.1485), 18.0eV, 0.1-1.0min #3-56
267.1485
268.1518
[%]
C17H18N2O, M+nH, 267.1492
1+
267.1492
1+
268.1523
m/z

IR

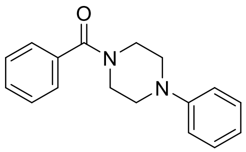
O
N
N

1i

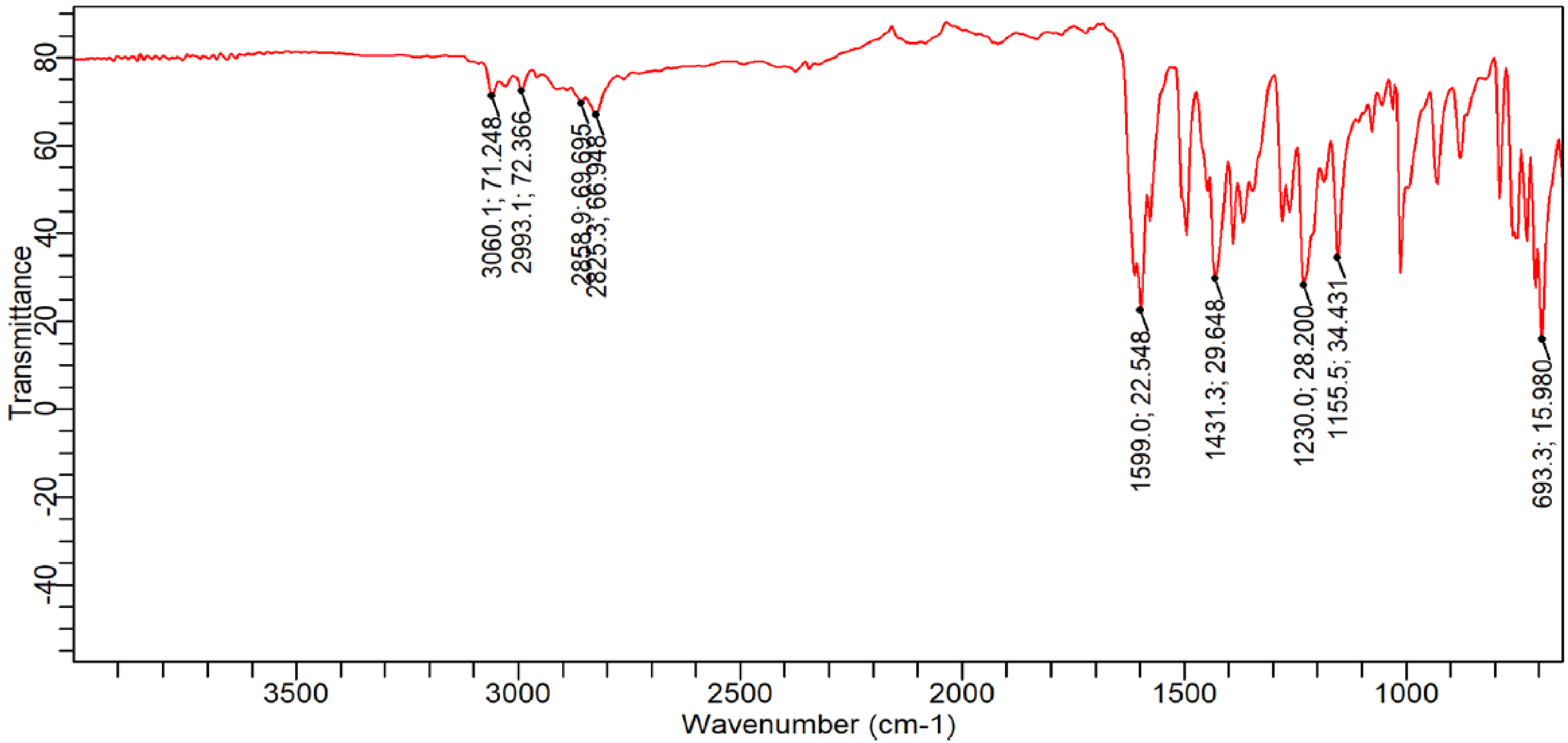
3060.1; 71.248
2993.1; 72.366
2858.9; 69.695
2825.3; 66.948
1599.0; 22.548
1431.3; 29.648
1230.0; 28.200
1155.5; 34.431
693.3; 15.980
Transmittance
80
60
40
20
0
-20
-40
3500
3000
2500
2000
1500
1000
Wavenumber (cm-1)

9 Morpholino(phenyl)methanone (1j)

¹H NMR

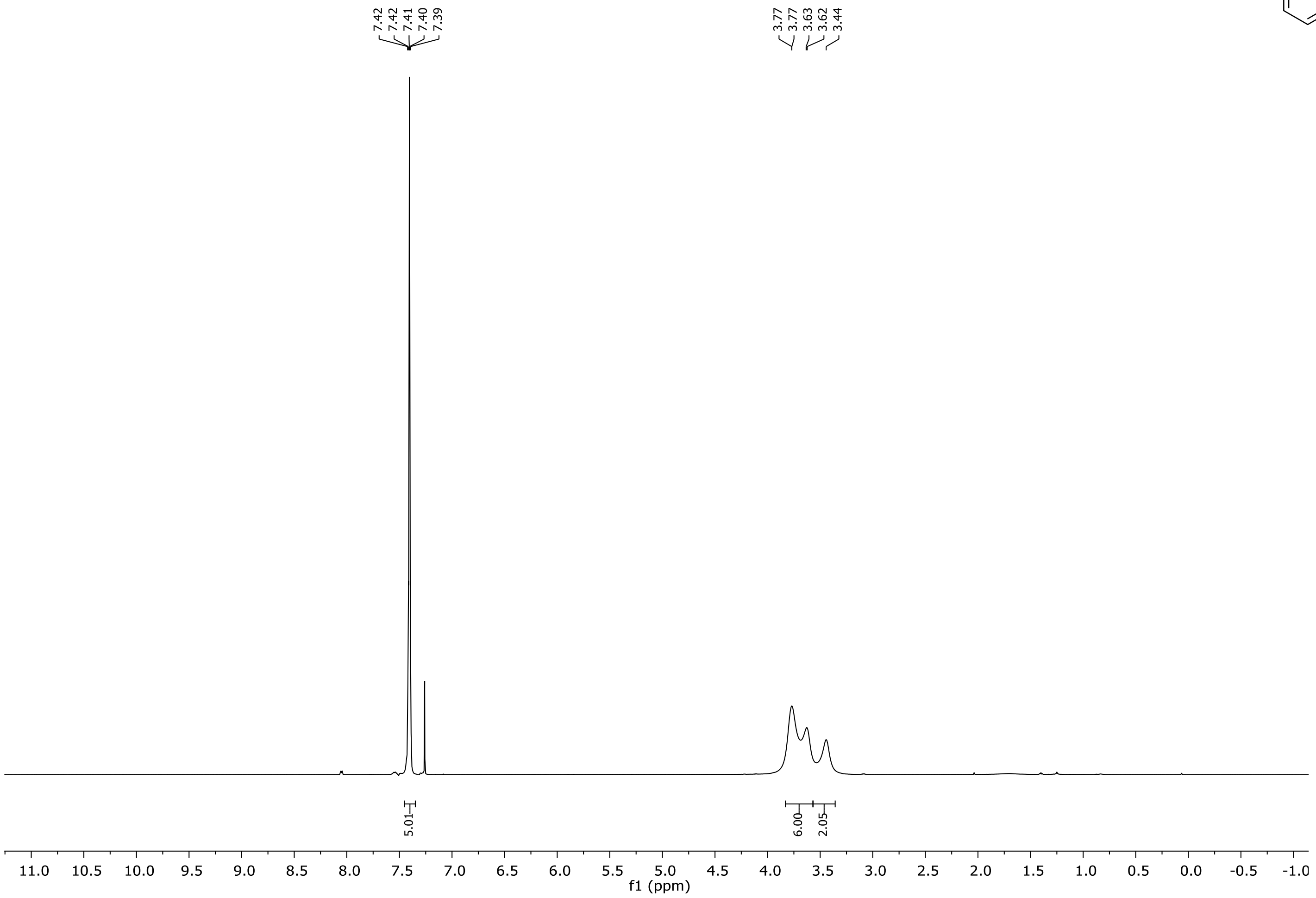

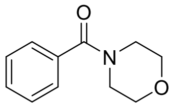

1j

^{13}C NMR

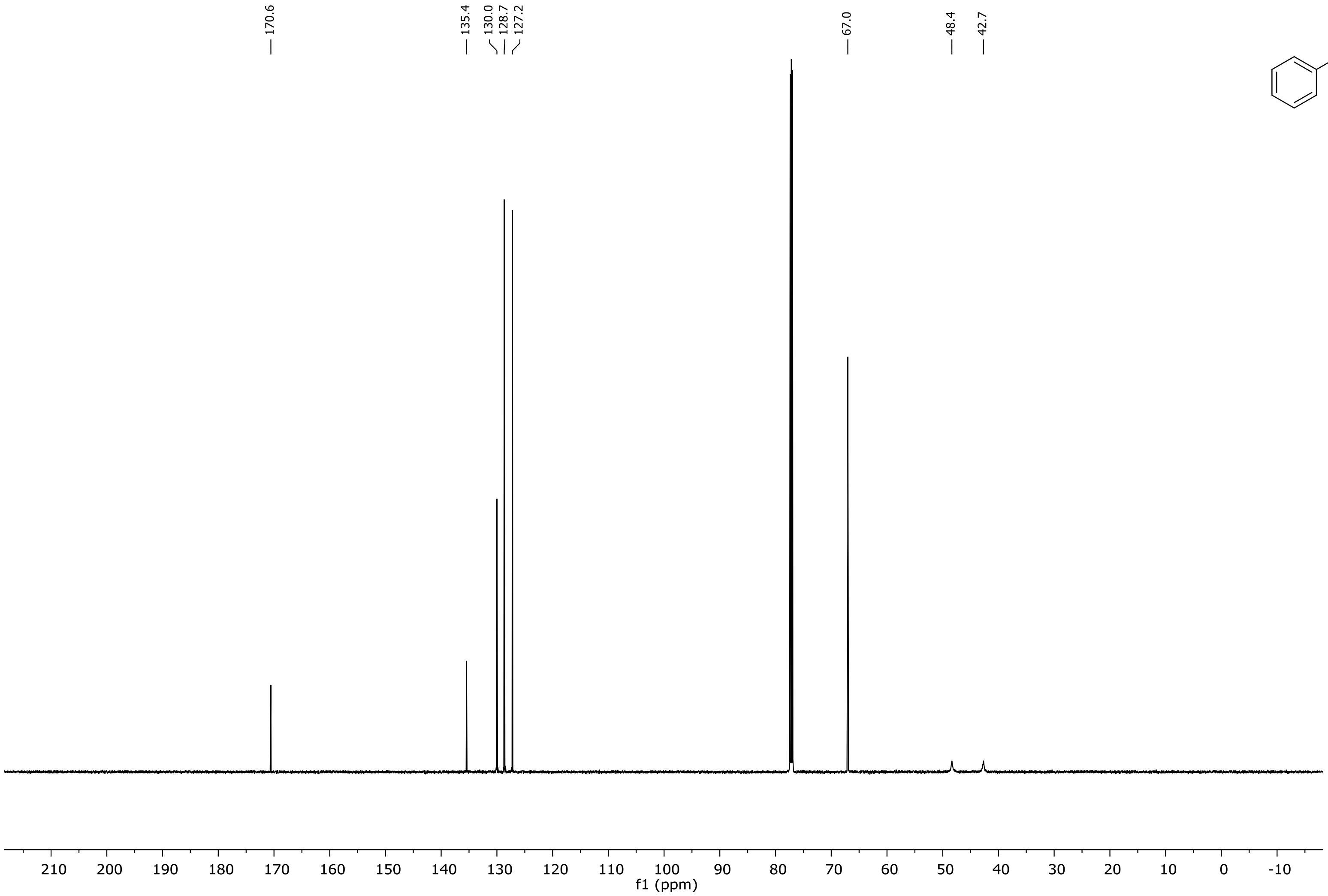
170.6
135.4
130.0
128.7
127.2
67.0
48.4
42.7
210
200
190
180
170
160
150
140
130
120
110
100
90
80
70
60
50
40
30
20
10
0
-10
f1 (ppm)

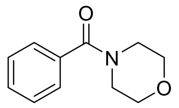
O
N
O

1j

^1H, ^1H COSY

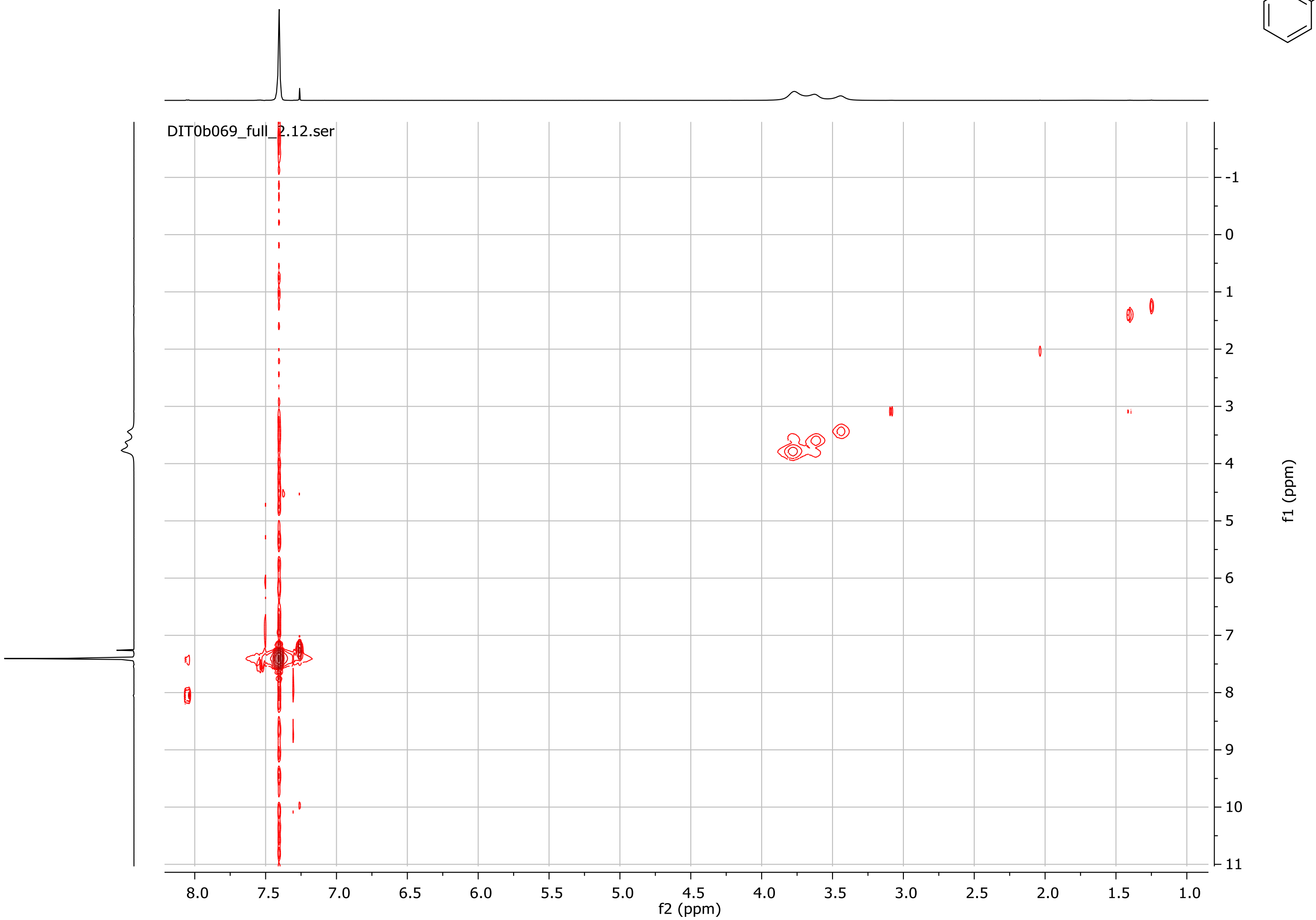

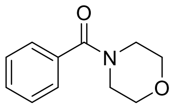

1j

^{1}H, ^{13}C HMBC

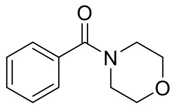

1j

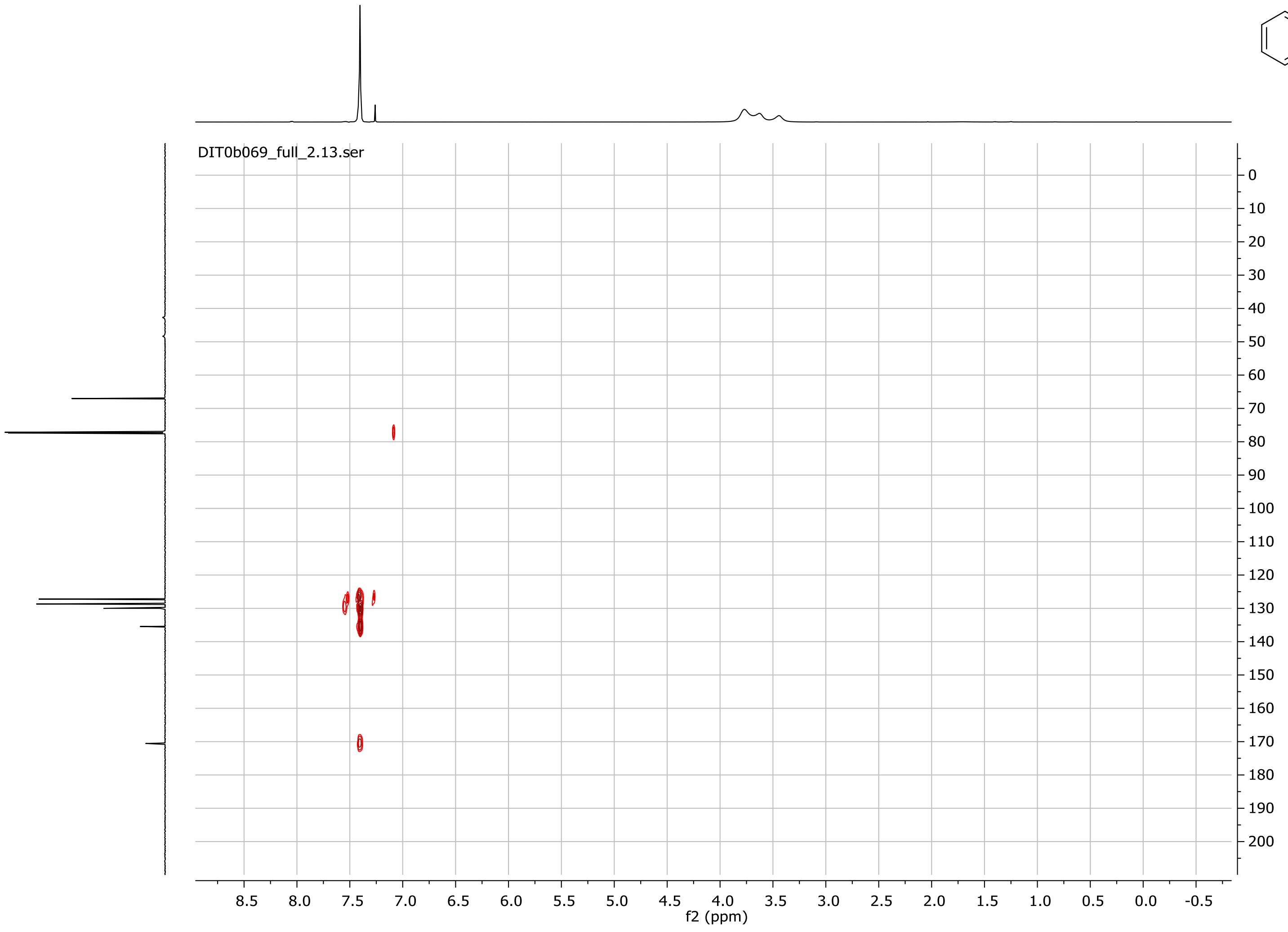

^{1}H, ^{13}C HSQC

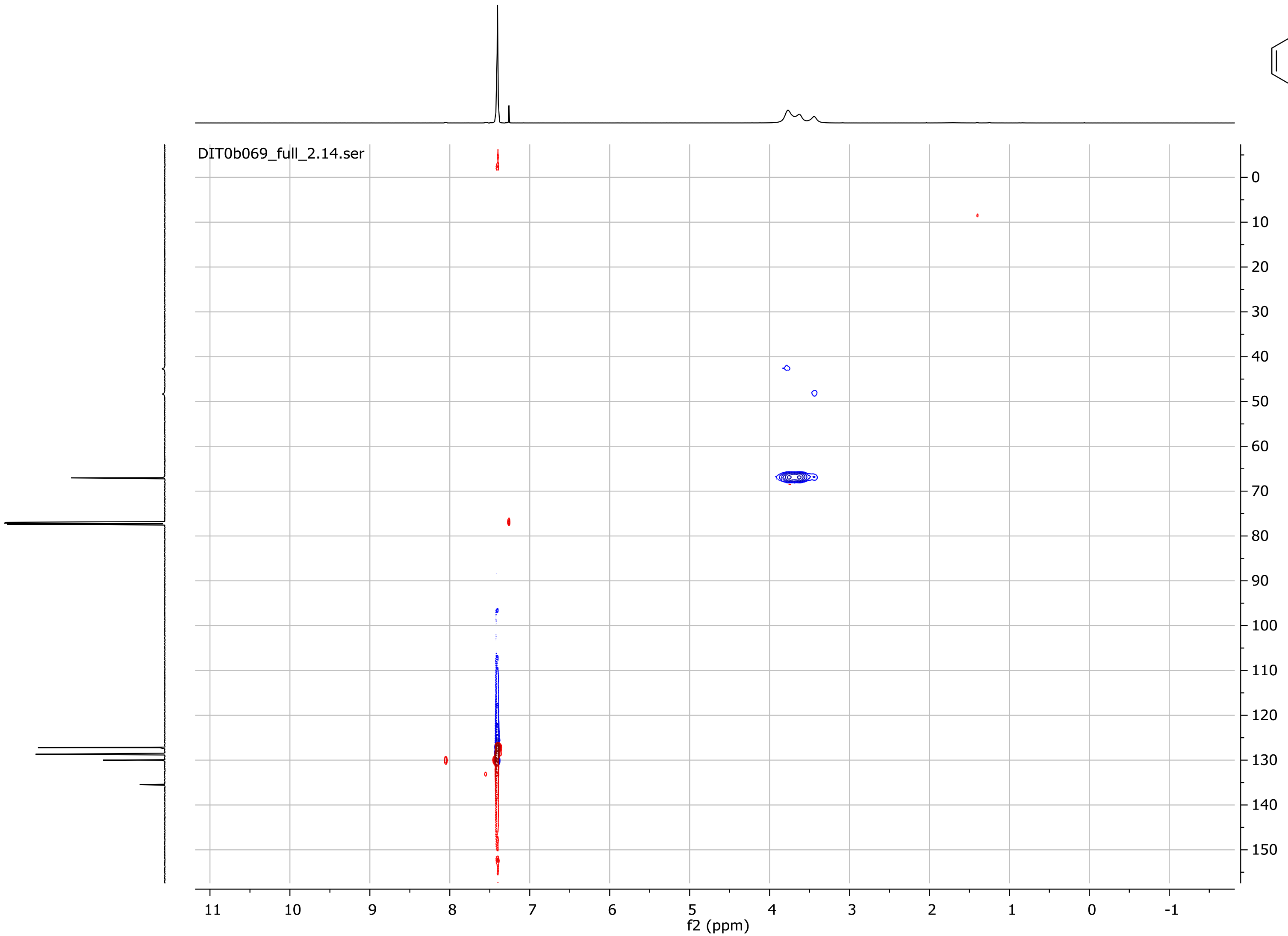

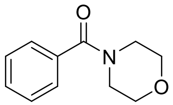

1j

HRMS

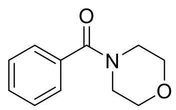

1j

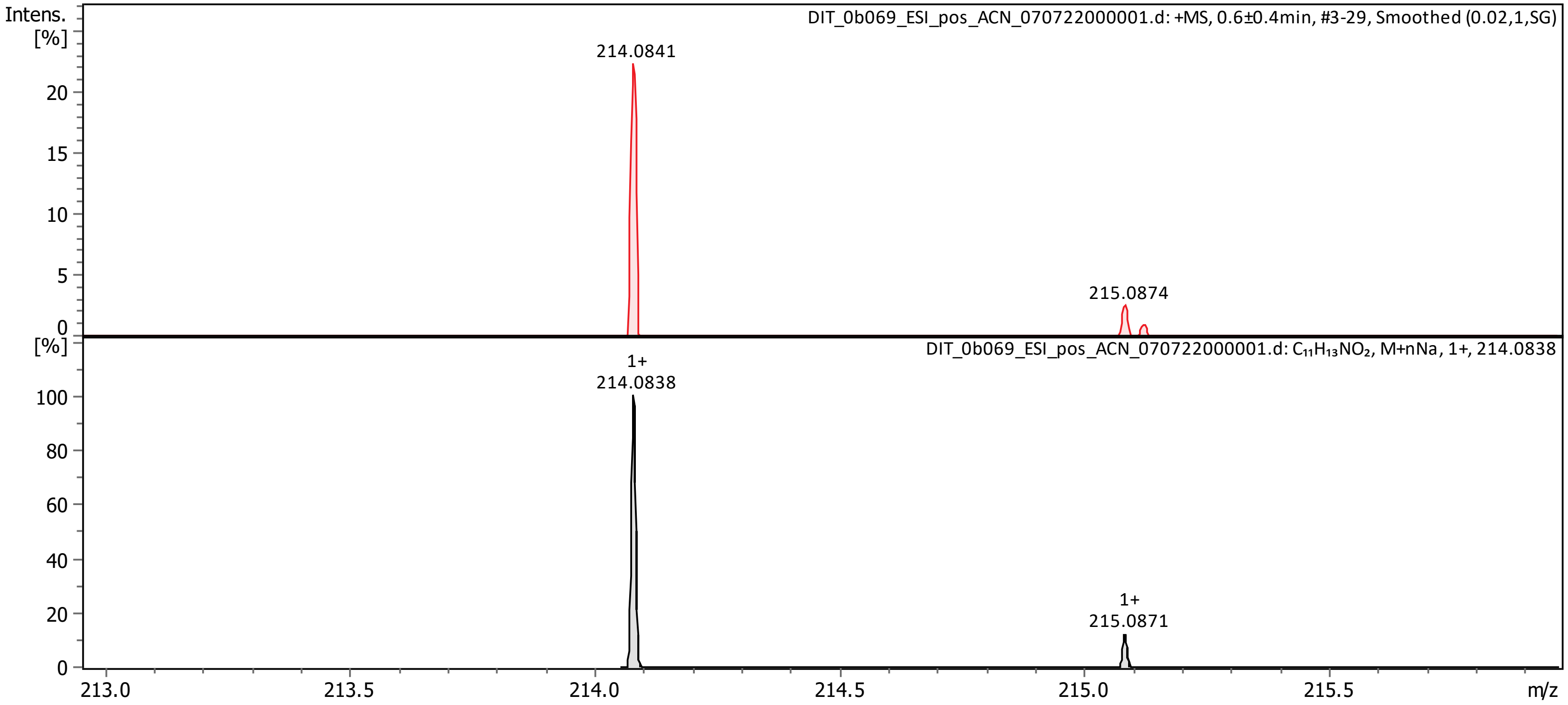
Intens. [%]
DIT_0b069_ESI_pos_ACN_070722000001.d: +MS, 0.6±0.4min, #3-29, Smoothed (0.02,1,SG)
214.0841
215.0874
20
15
10
5
0
[%]
DIT_0b069_ESI_pos_ACN_070722000001.d: C₁₁H₁₃NO₂, M+nNa, 1+, 214.0838
1+
214.0838
1+
215.0871
100
80
60
40
20
0
213.0
213.5
214.0
214.5
215.0
215.5
m/z

IR

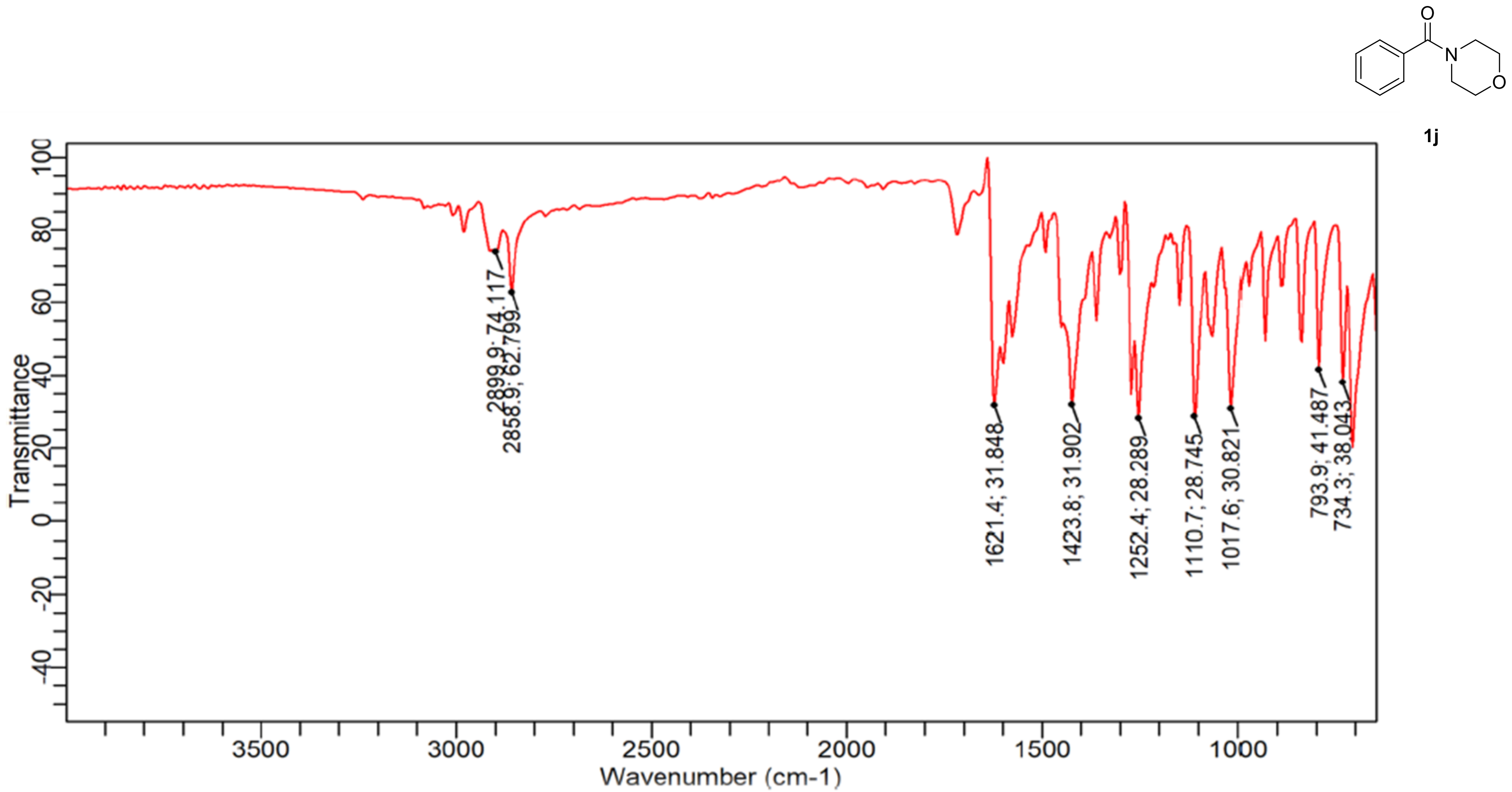
Transmittance
Wavenumber (cm-1)
2899.9; 74.117
2858.9; 62.799
1621.4; 31.848
1423.8; 31.902
1252.4; 28.289
1110.7; 28.745
1017.6; 30.821
793.9; 41.487
734.3; 38.043
O
N
O

1j

10 Morpholino(*p*-tolyl)methanone (1m)

^{1}H NMR

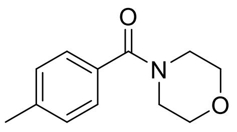

1m

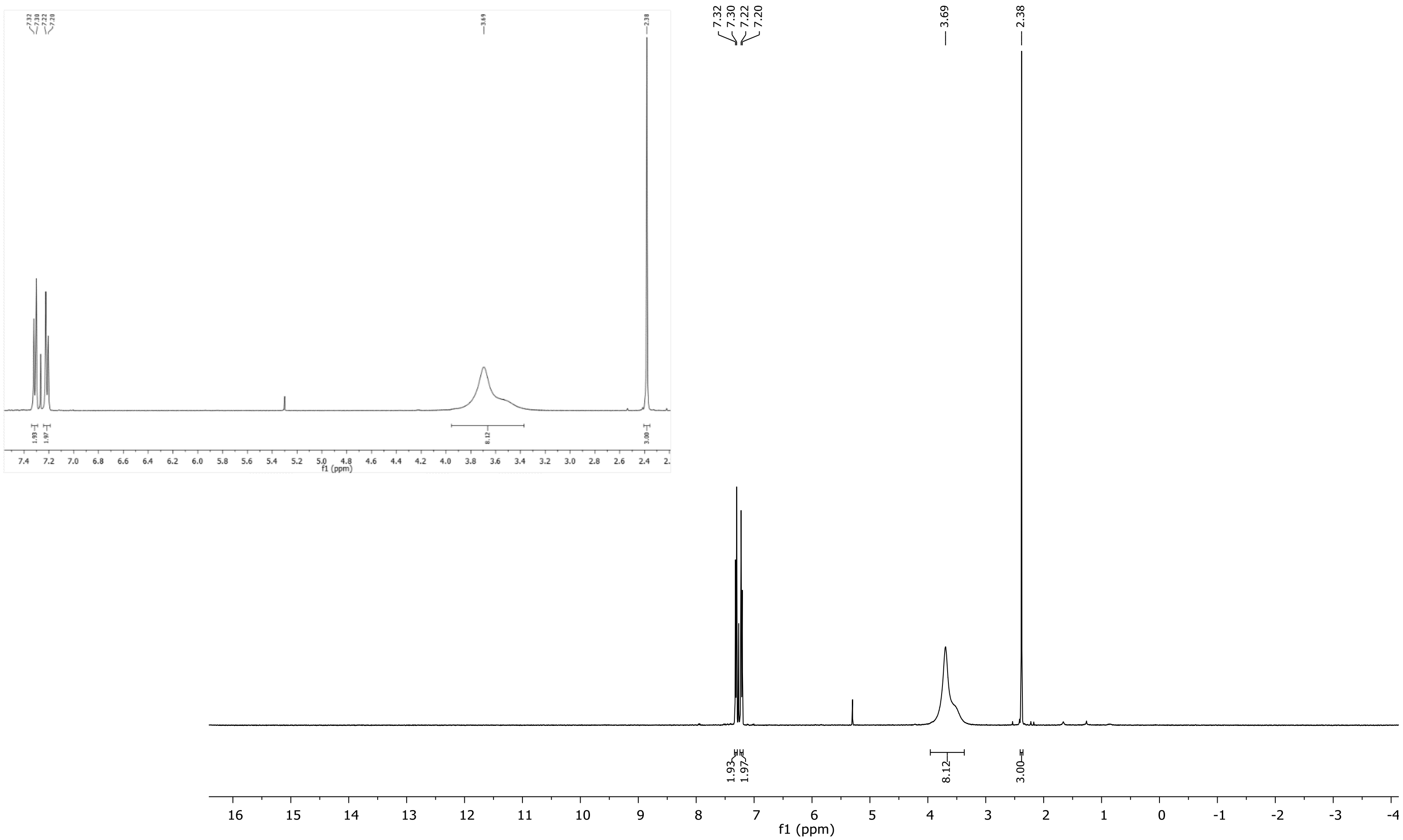

^{13}C NMR

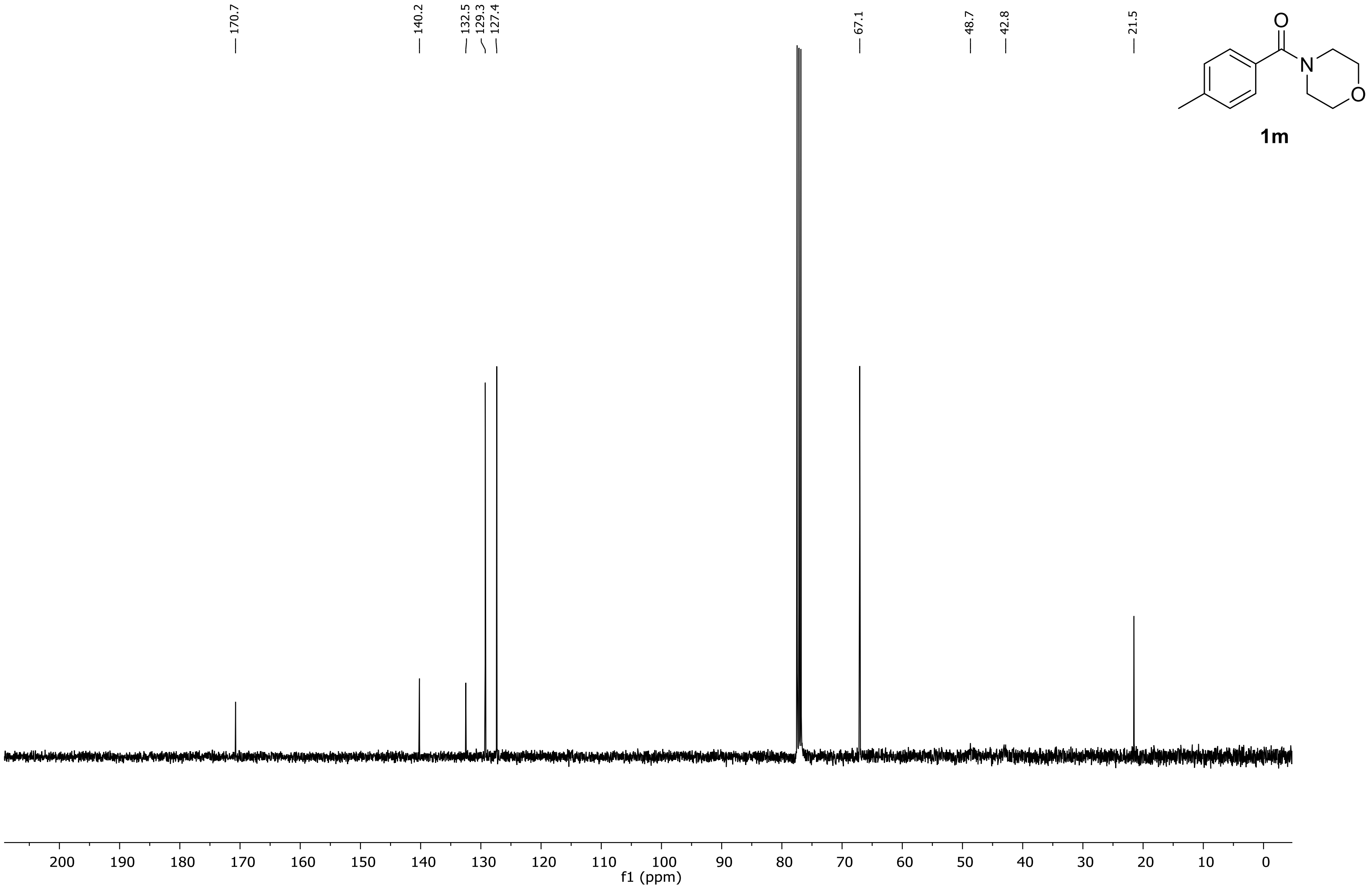
170.7
140.2
132.5
129.3
127.4
67.1
48.7
42.8
21.5
200 190 180 170 160 150 140 130 120 110 100 90 80 70 60 50 40 30 20 10 0
f1 (ppm)
O
N
O

1m

^{1}H, ^{1}H COSY

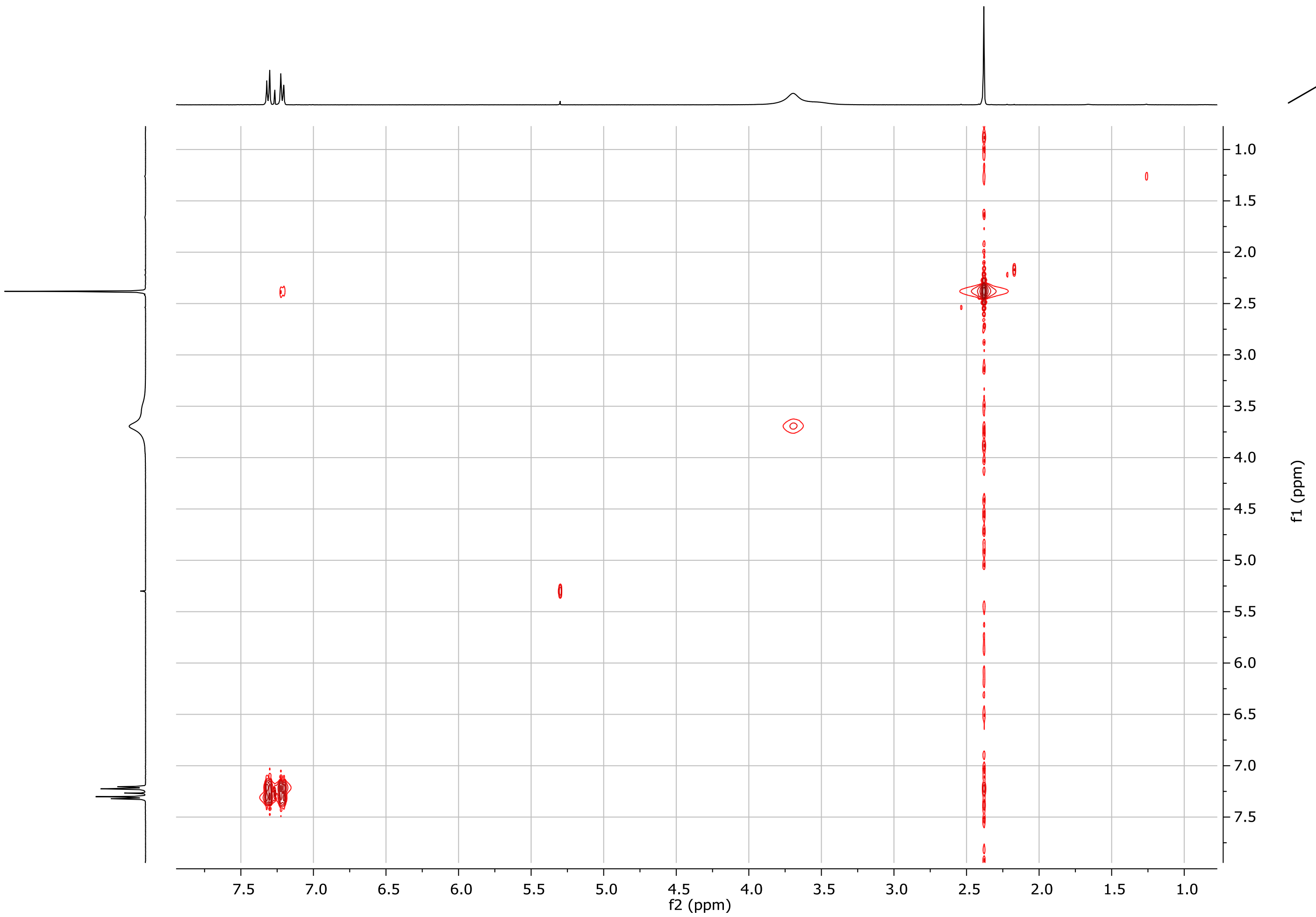

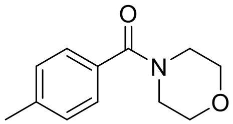

1m

^{1}H, ^{13}C HMBC

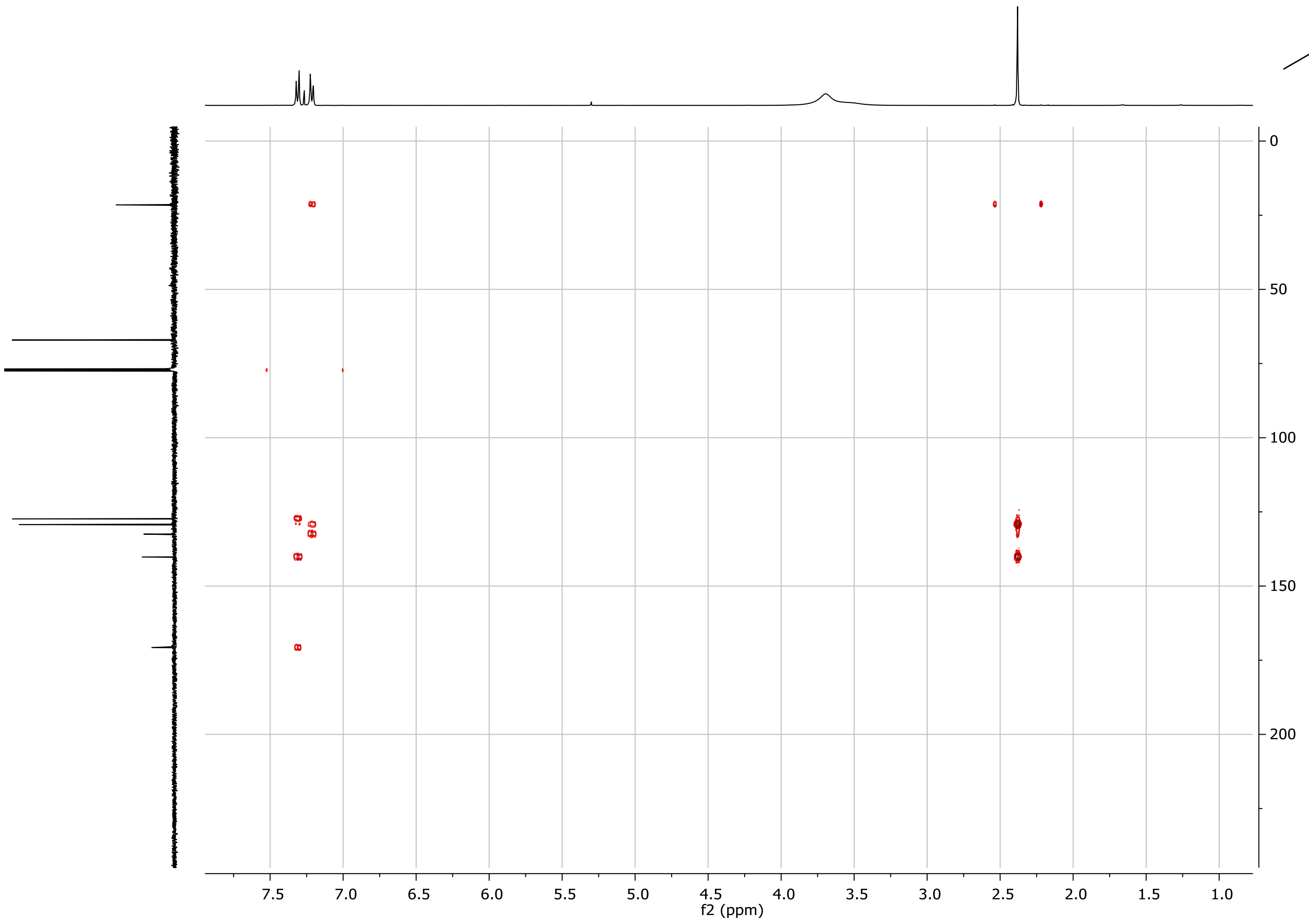

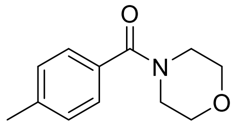

1m

^{1}H, ^{13}C HSQC

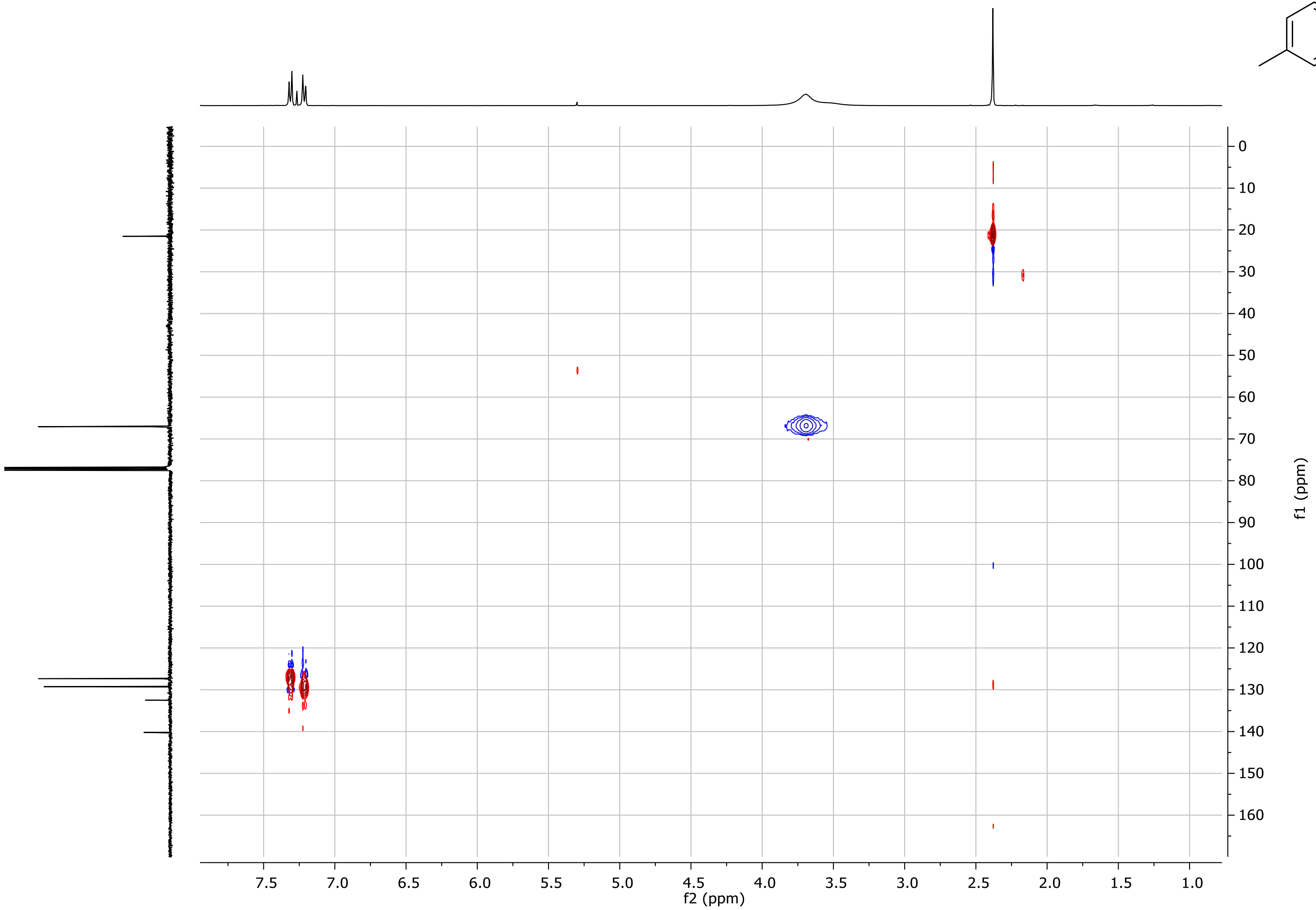

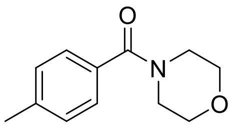

1m

HRMS

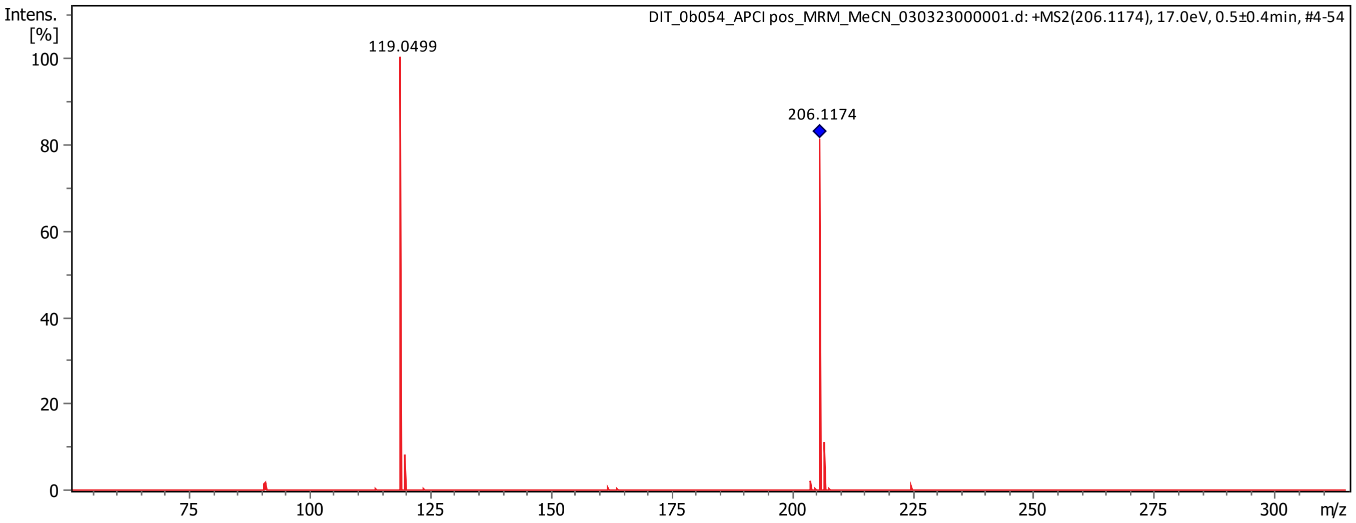
Intens.
[%]
DIT_0b054_APCI pos_MRM_MeCN_030323000001.d: +MS2(206.1174), 17.0eV, 0.5±0.4min, #4-54
119.0499
206.1174
m/z

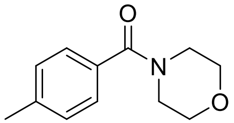

1m

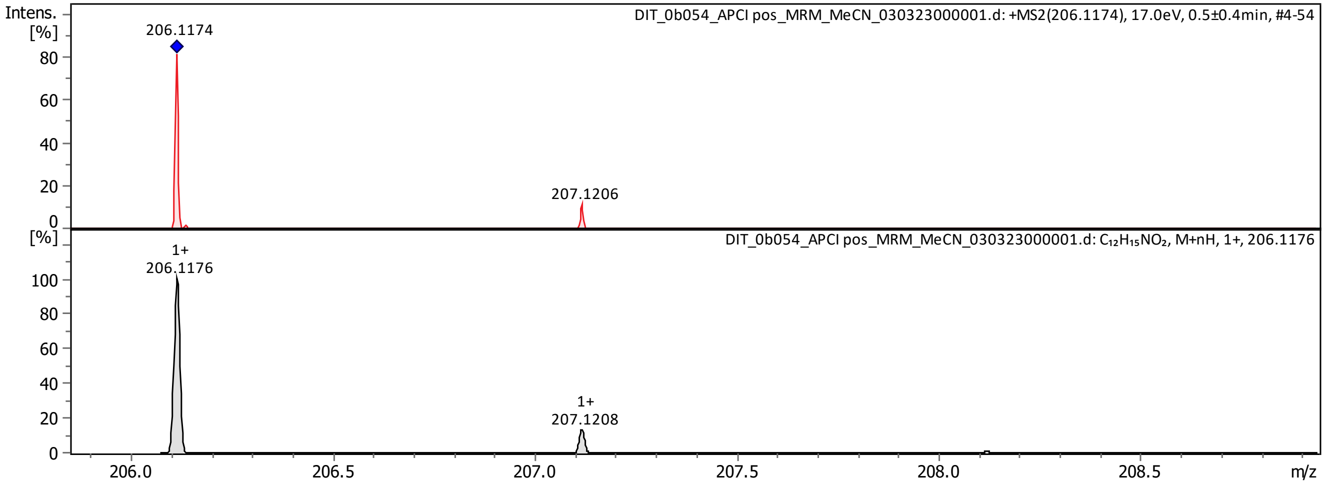
Intens.
[%]
DIT_0b054_APCI pos_MRM_MeCN_030323000001.d: +MS2(206.1174), 17.0eV, 0.5±0.4min, #4-54
206.1174
207.1206
DIT_0b054_APCI pos_MRM_MeCN_030323000001.d: C₁₂H₁₅NO₂, M+nH, 1+, 206.1176
1+
206.1176
1+
207.1208
m/z

11 Cyclohexyl(morpholino)methanone (1n)

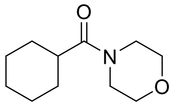

1n

^{1}H NMR

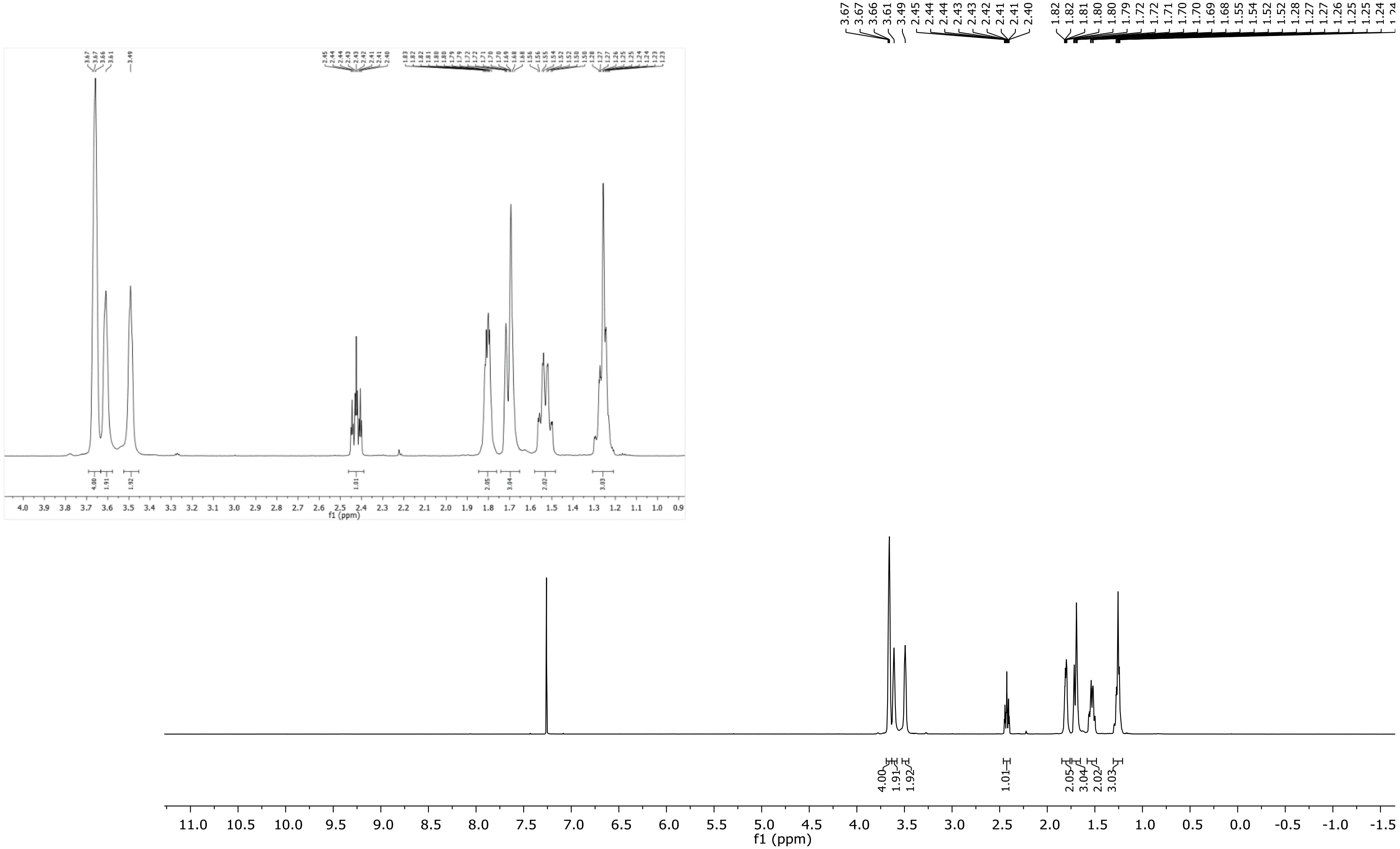

^{13}C NMR

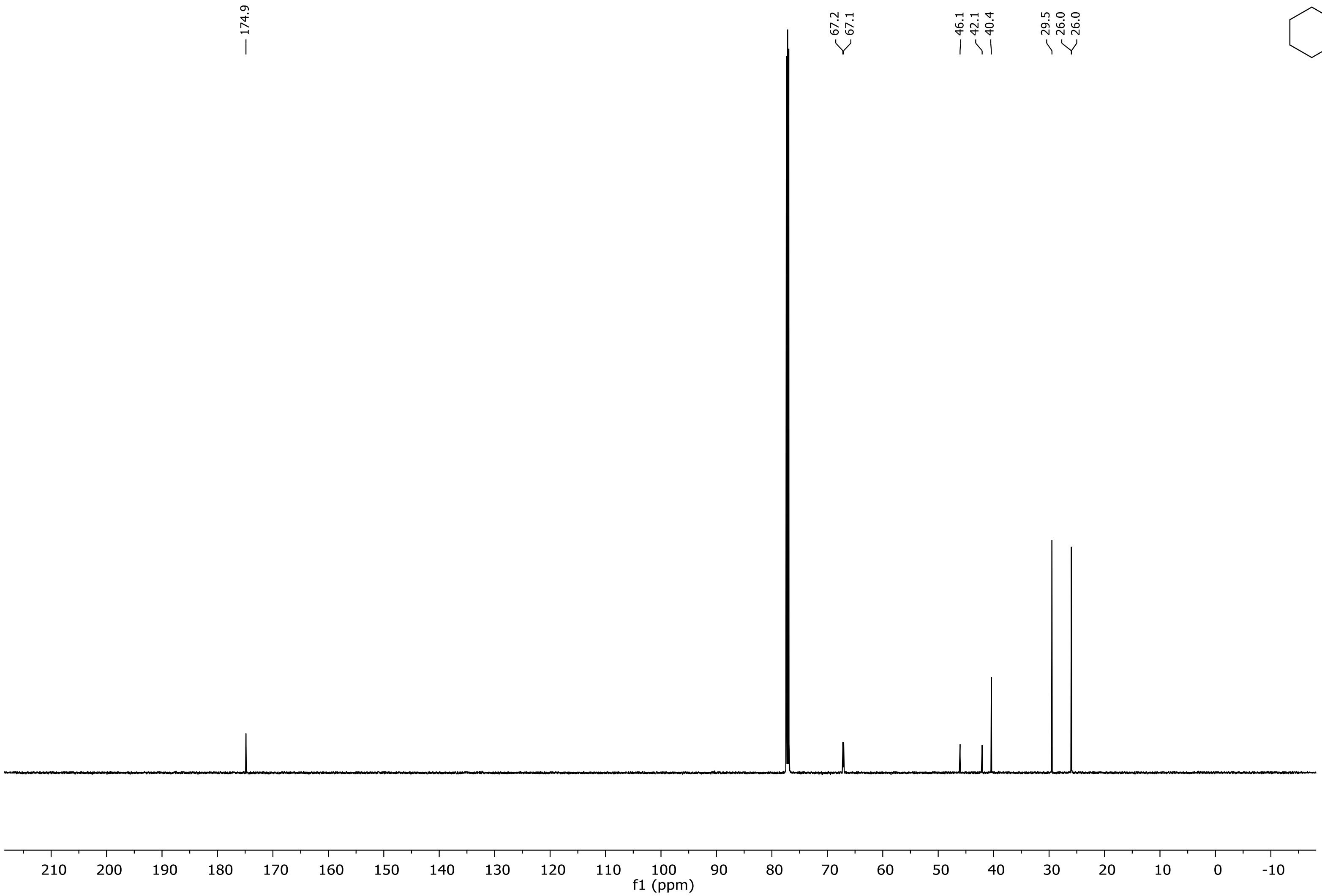

174.9
67.2
67.1
46.1
42.1
40.4
29.5
26.0
26.0
210 200 190 180 170 160 150 140 130 120 110 100 90 80 70 60 50 40 30 20 10 0 -10
f1 (ppm)

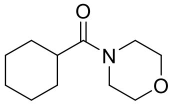

O
N
O

1n

^1H, ^1H COSY

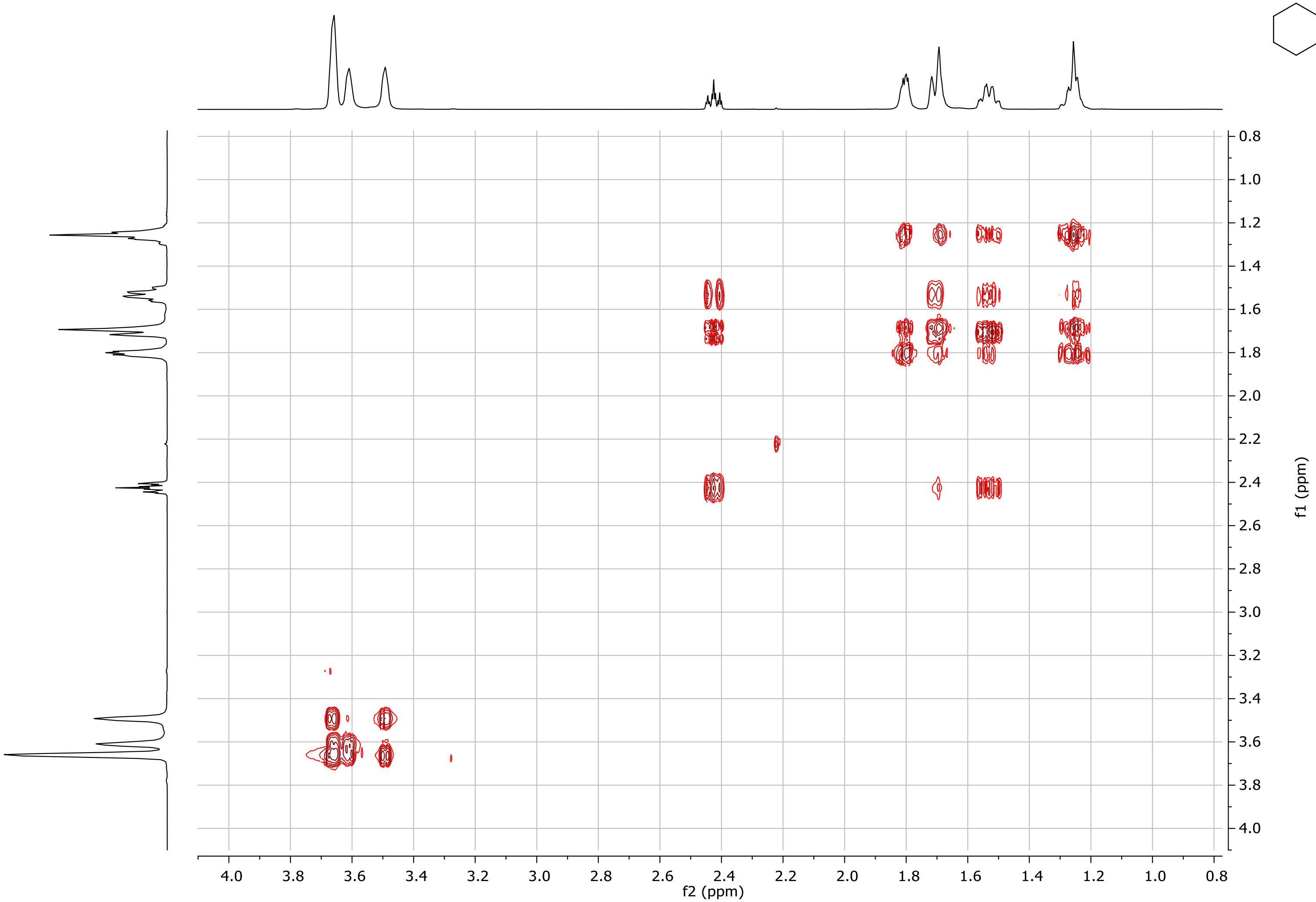

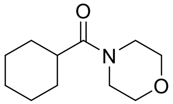

1n

^{1}H, ^{13}C HMBC

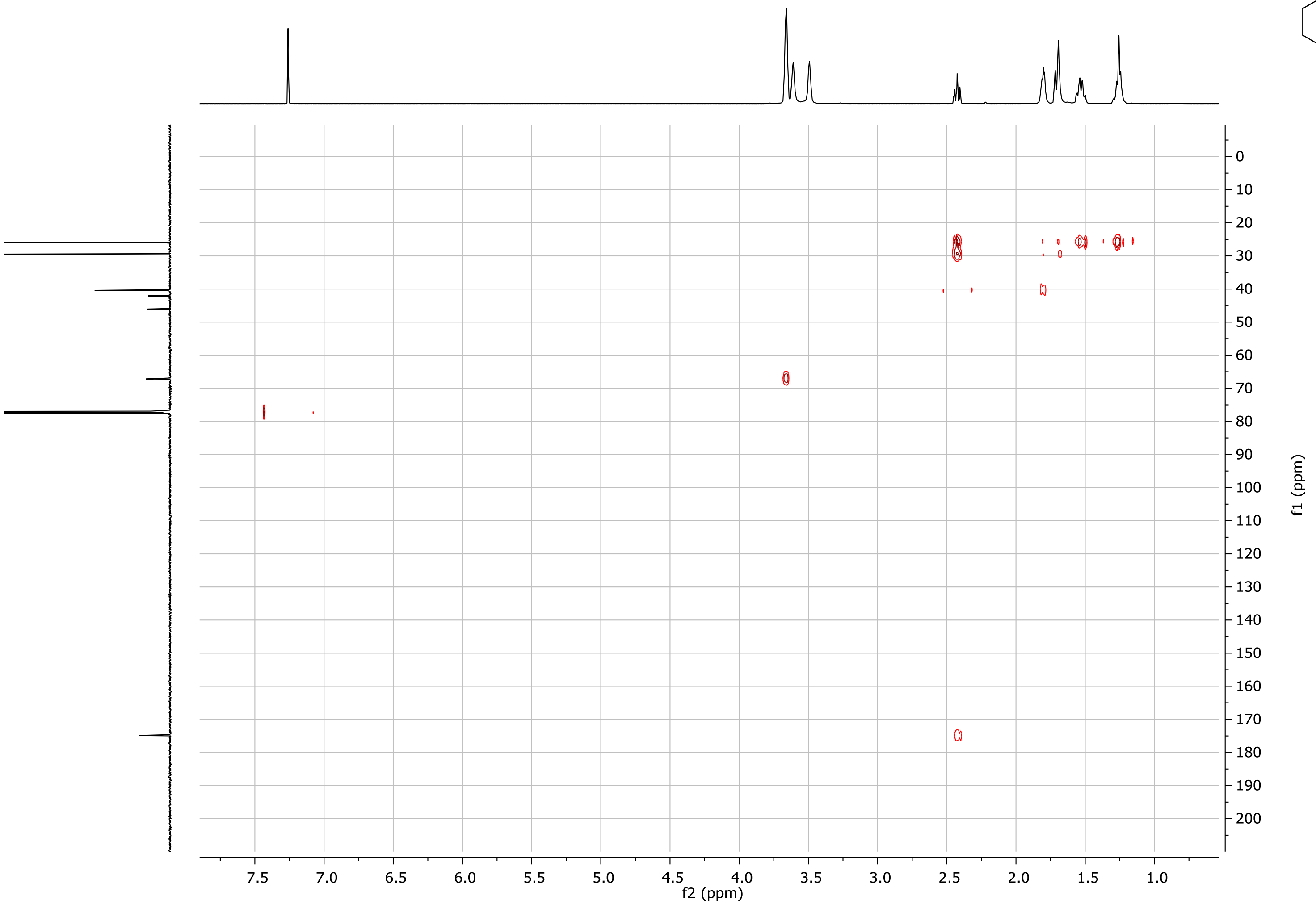

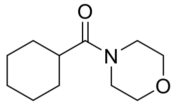

1n

^{1}H, ^{13}C HSQC

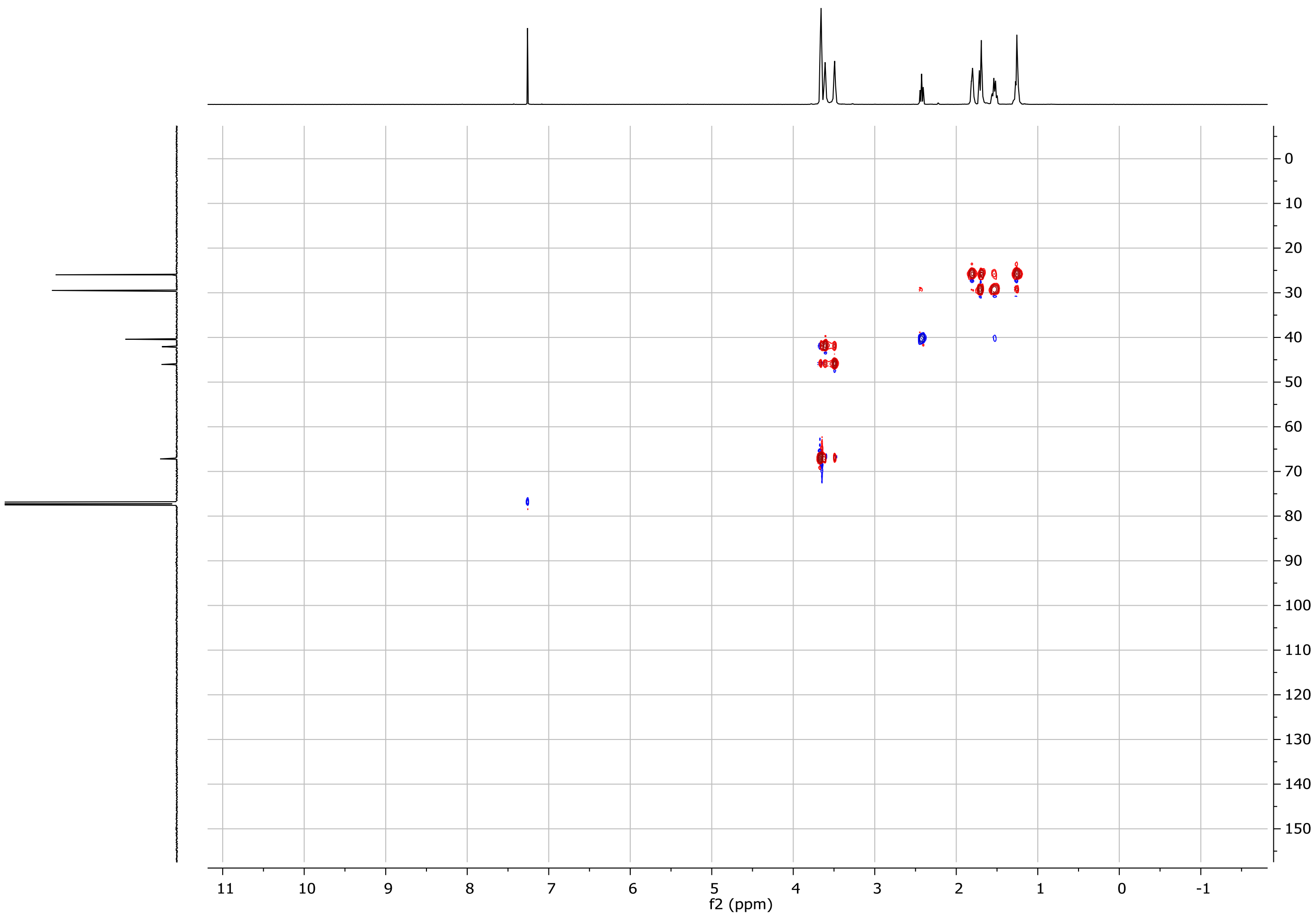

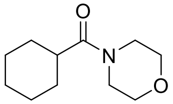

1n

HRMS

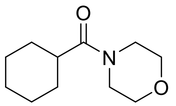

1n

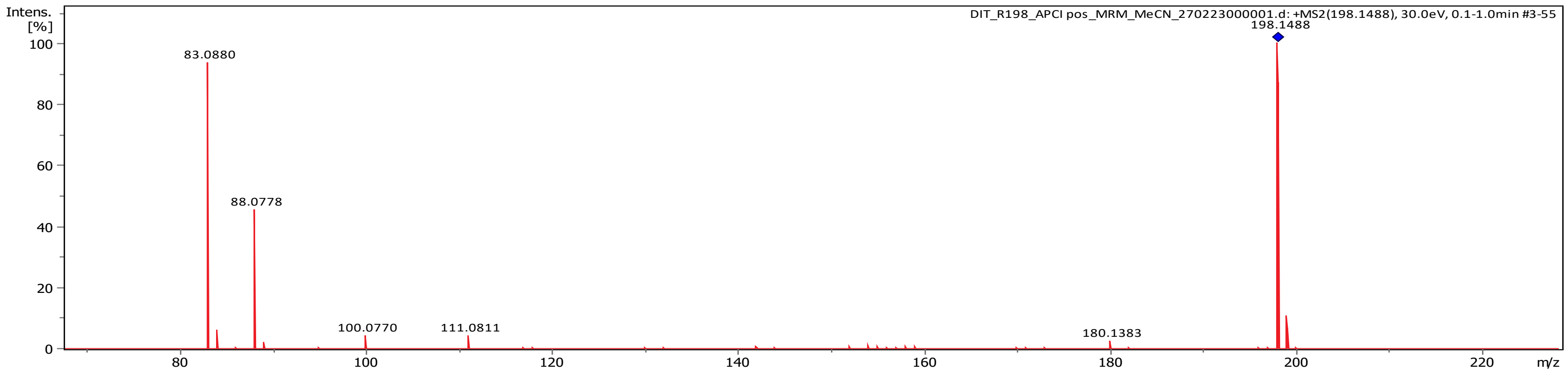
Intens.
[%]
DIT_R198_APCI pos_MRM_MeCN_270223000001.d: +MS2(198.1488), 30.0eV, 0.1-1.0min #3-55
198.1488
83.0880
88.0778
100.0770
111.0811
180.1383
m/z

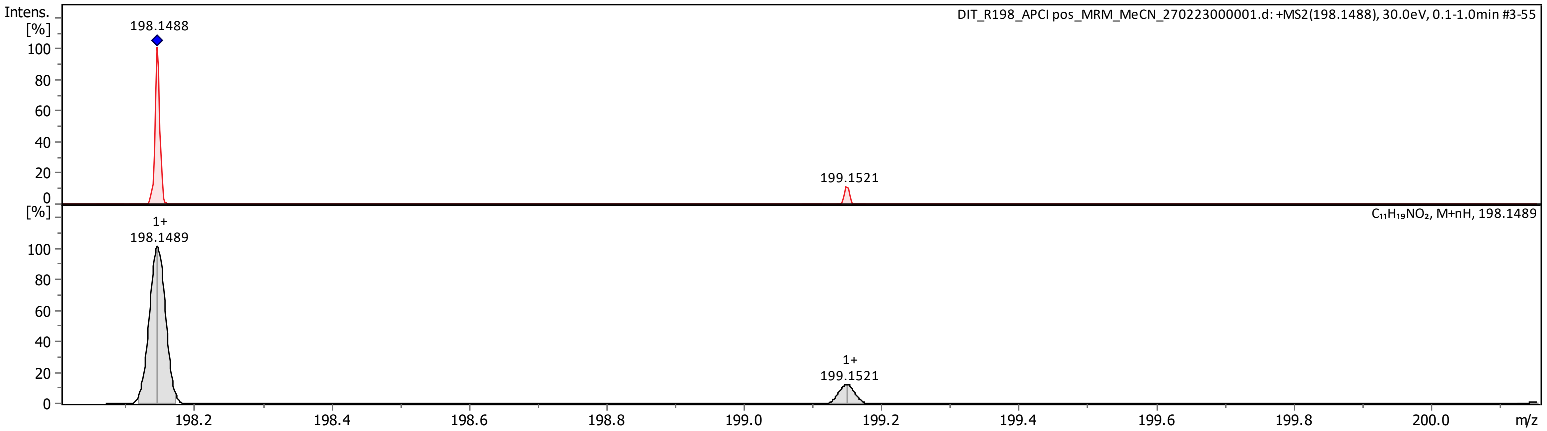
Intens.
[%]
DIT_R198_APCI pos_MRM_MeCN_270223000001.d: +MS2(198.1488), 30.0eV, 0.1-1.0min #3-55
198.1488
199.1521
C11H19NO2, M+nH, 198.1489
1+
198.1489
1+
199.1521
m/z

12 Morpholino(*m*-tolyl)methanone (1o)

^{1}H NMR

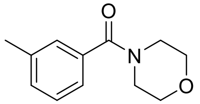

1o

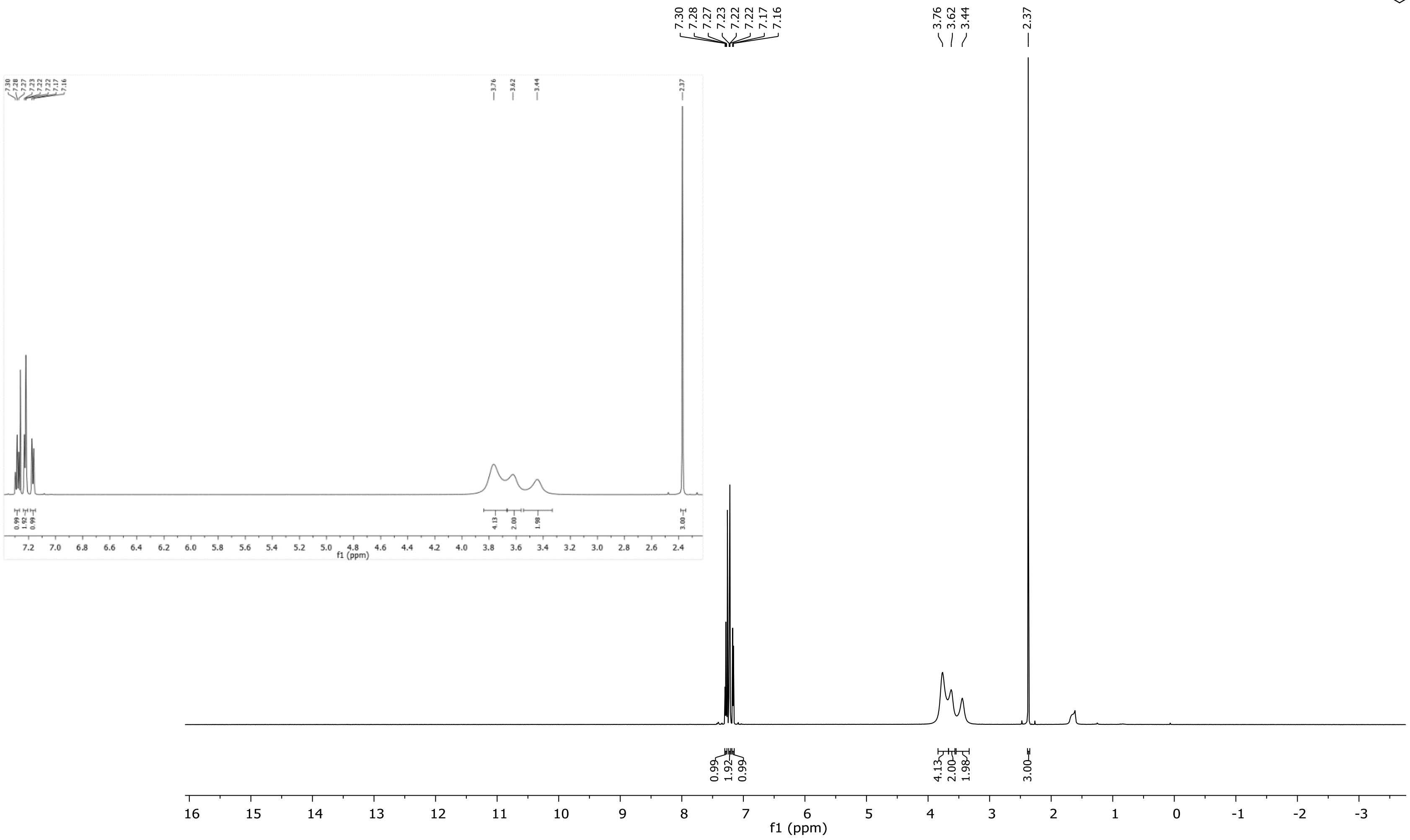

^{13}C NMR

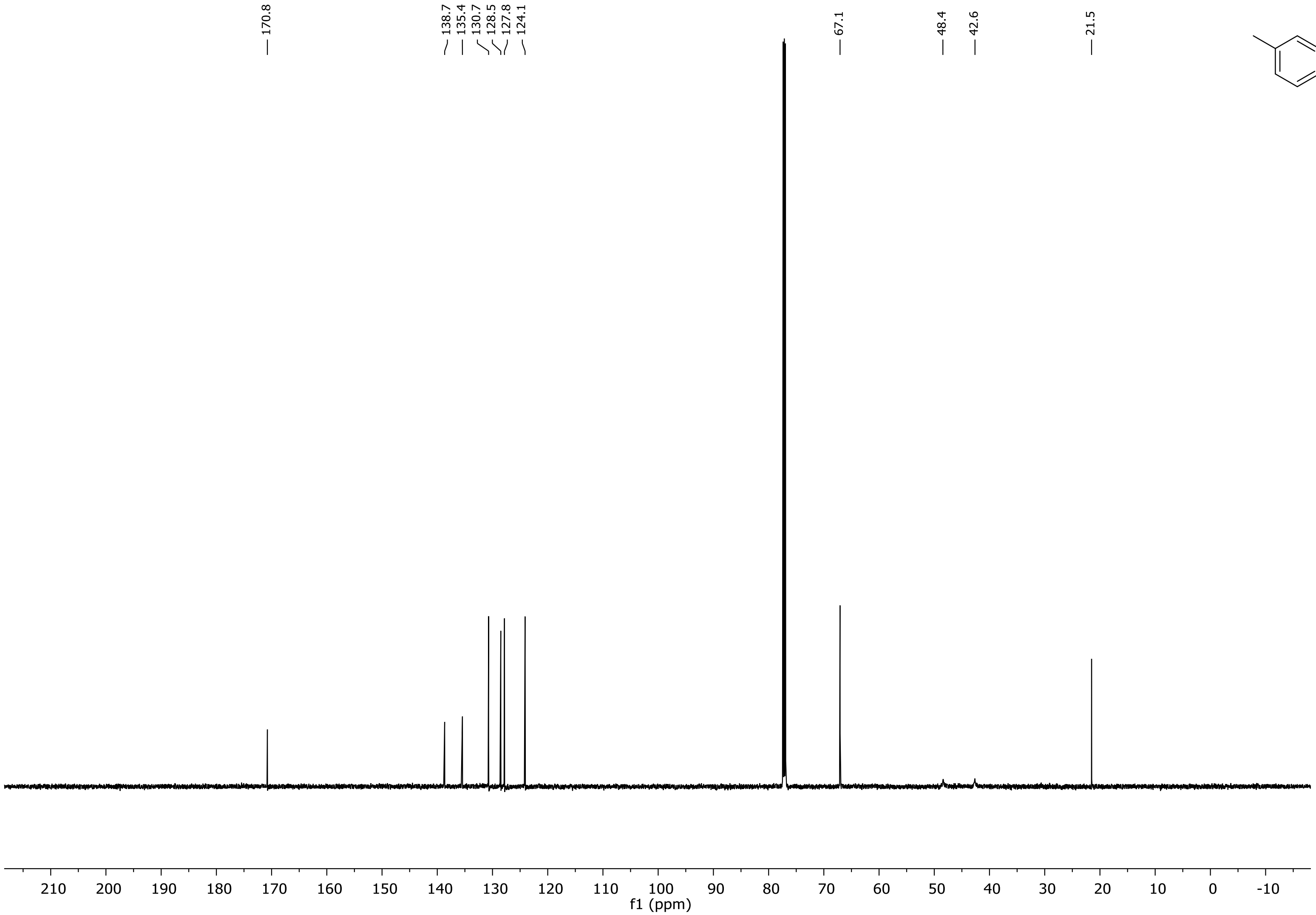

170.8
138.7
135.4
130.7
128.5
127.8
124.1
67.1
48.4
42.6
21.5
210 200 190 180 170 160 150 140 130 120 110 100 90 80 70 60 50 40 30 20 10 0 -10
f1 (ppm)

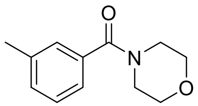

O
N
O

1o

^{1}H, ^{1}H COSY

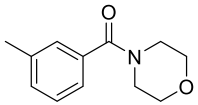

1o

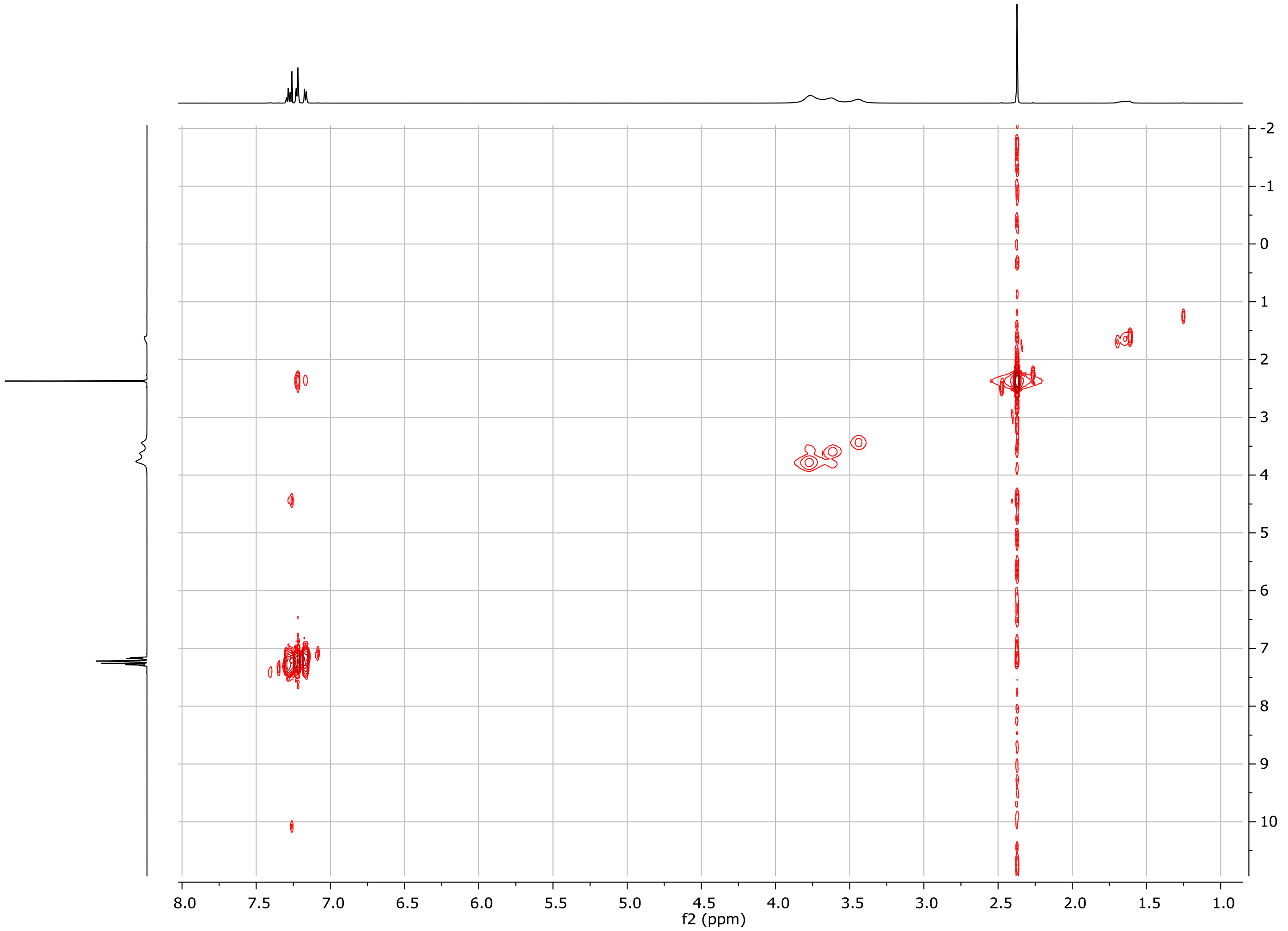

8.0
7.5
7.0
6.5
6.0
5.5
5.0
4.5
4.0
3.5
3.0
2.5
2.0
1.5
1.0
f2 (ppm)
-2
-1
0
1
2
3
4
5
6
7
8
9
10
f1 (ppm)

^{1}H, ^{13}C HMBC

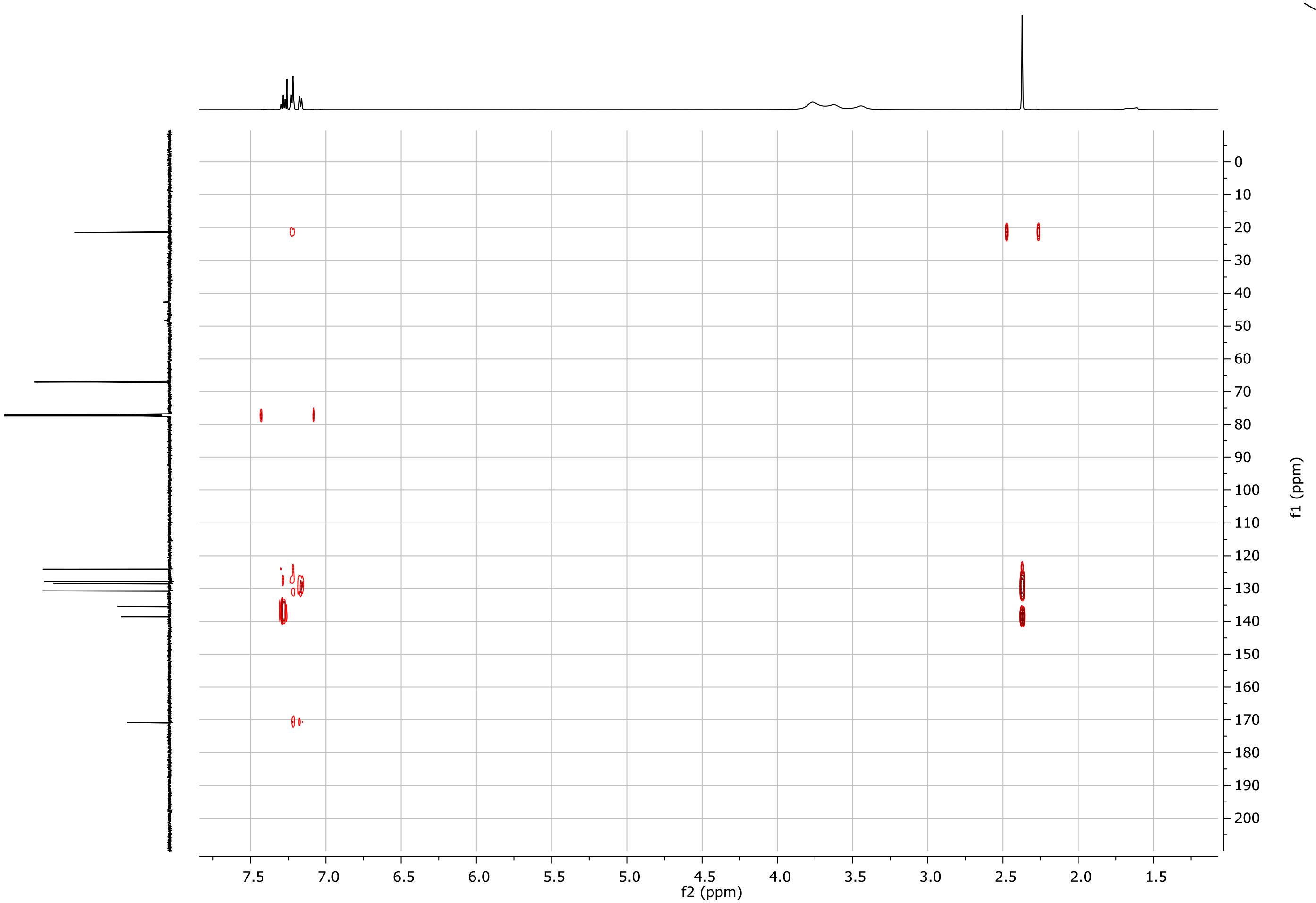

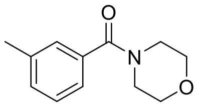

1o

^{1}H, ^{13}C HSQC

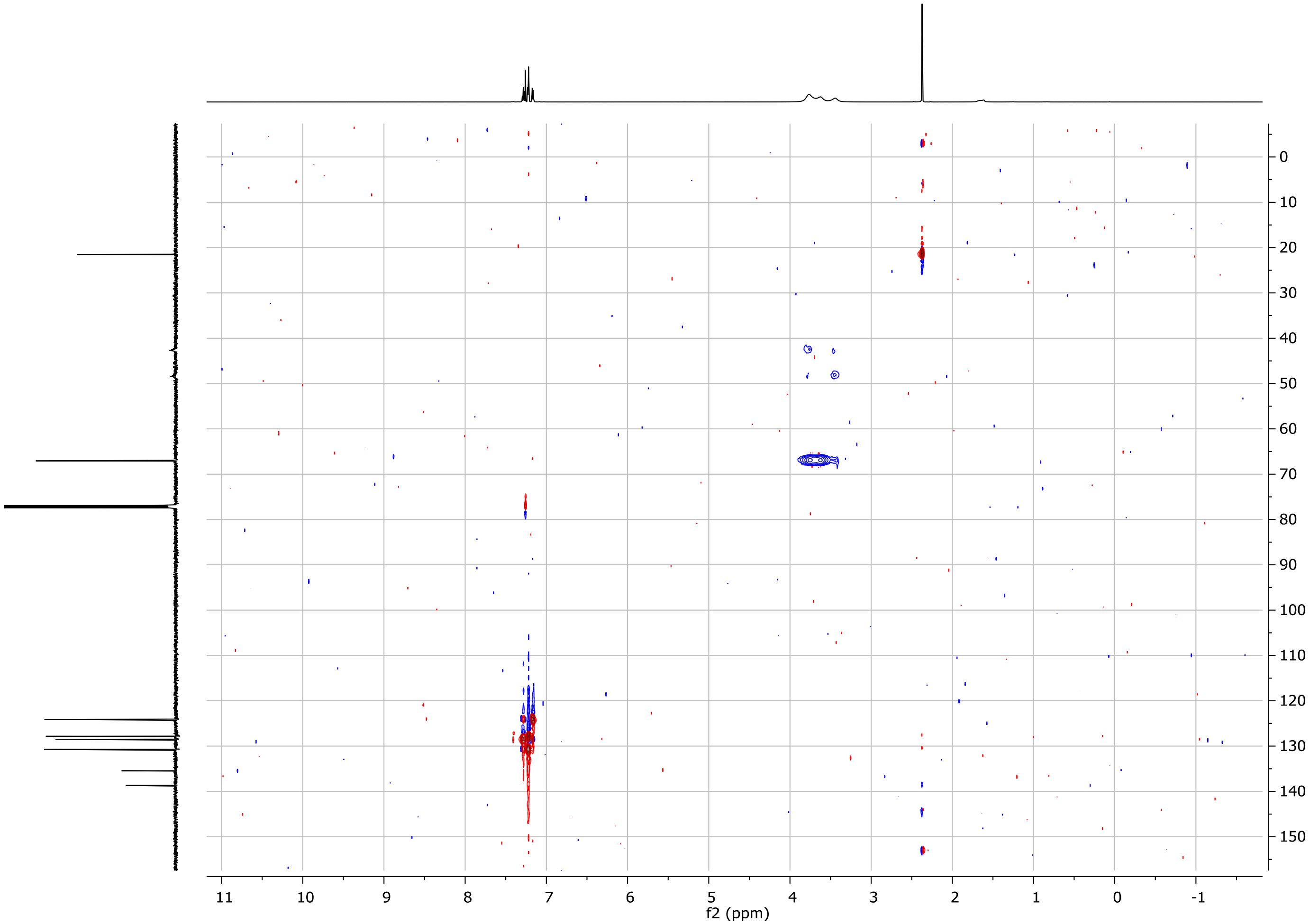

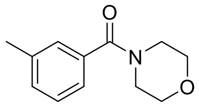

1o

HRMS

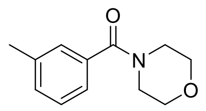

1o

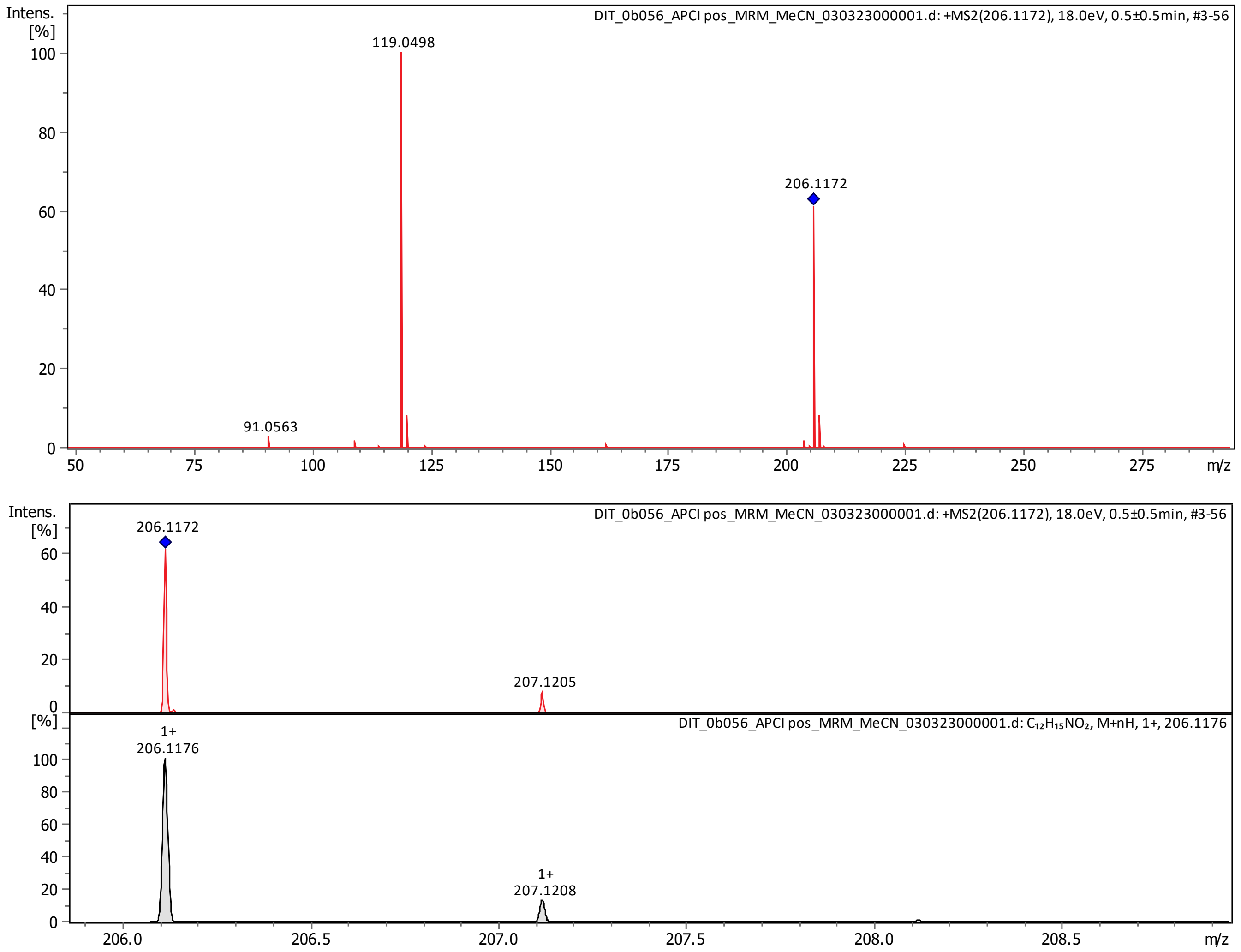

Intens. [%]
DIT_0b056_APCI pos_MRM_MeCN_030323000001.d: +MS2(206.1172), 18.0eV, 0.5±0.5min, #3-56
119.0498
206.1172
91.0563
m/z
Intens. [%]
DIT_0b056_APCI pos_MRM_MeCN_030323000001.d: +MS2(206.1172), 18.0eV, 0.5±0.5min, #3-56
206.1172
207.1205
[%]
DIT_0b056_APCI pos_MRM_MeCN_030323000001.d: C₁₂H₁₅NO₂, M+nH, 1+, 206.1176
1+
206.1176
1+
207.1208
m/z

13 Morpholino(*o*-tolyl)methanone (1p)

^{1}H NMR

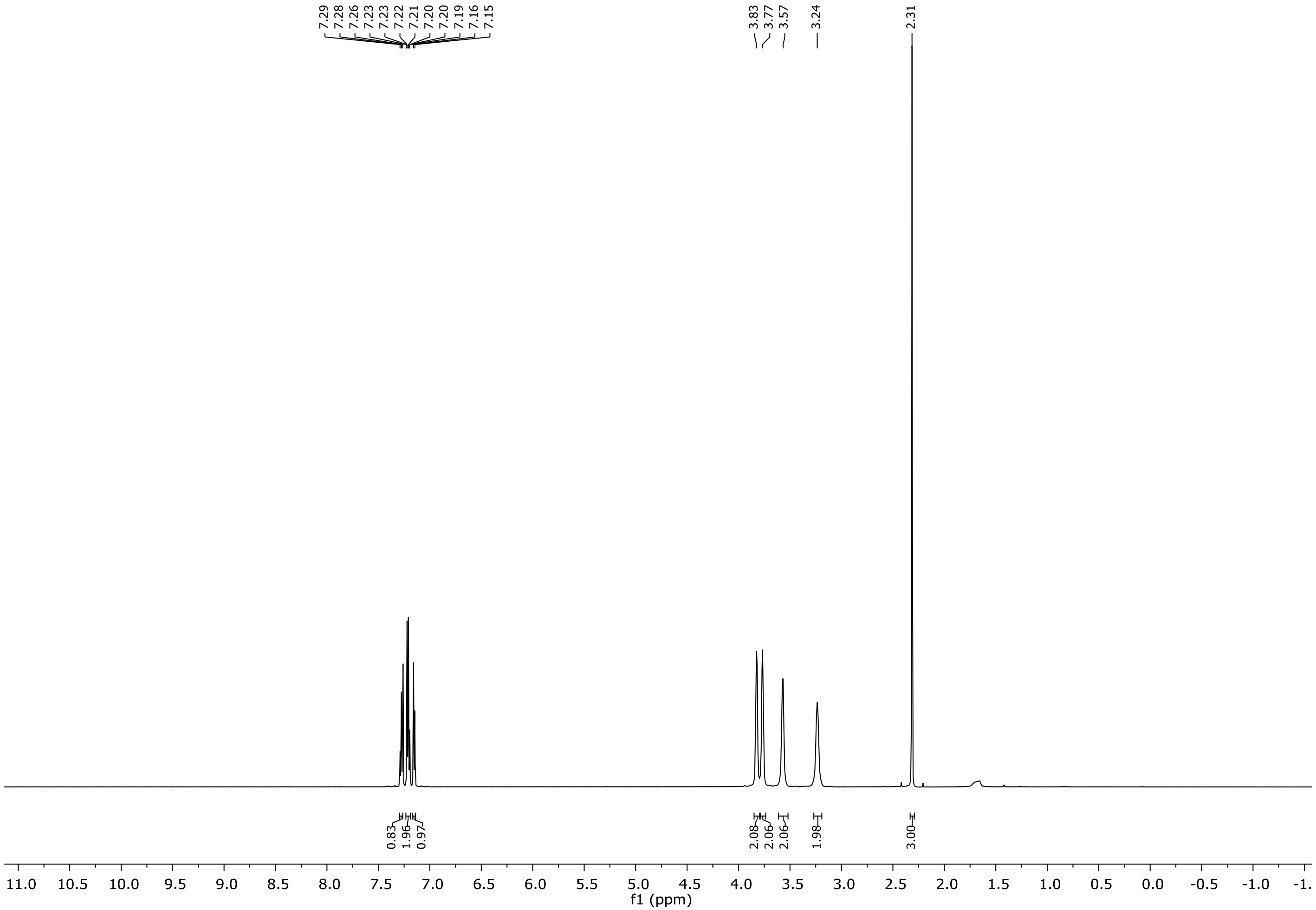

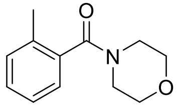

1p

^{13}C NMR

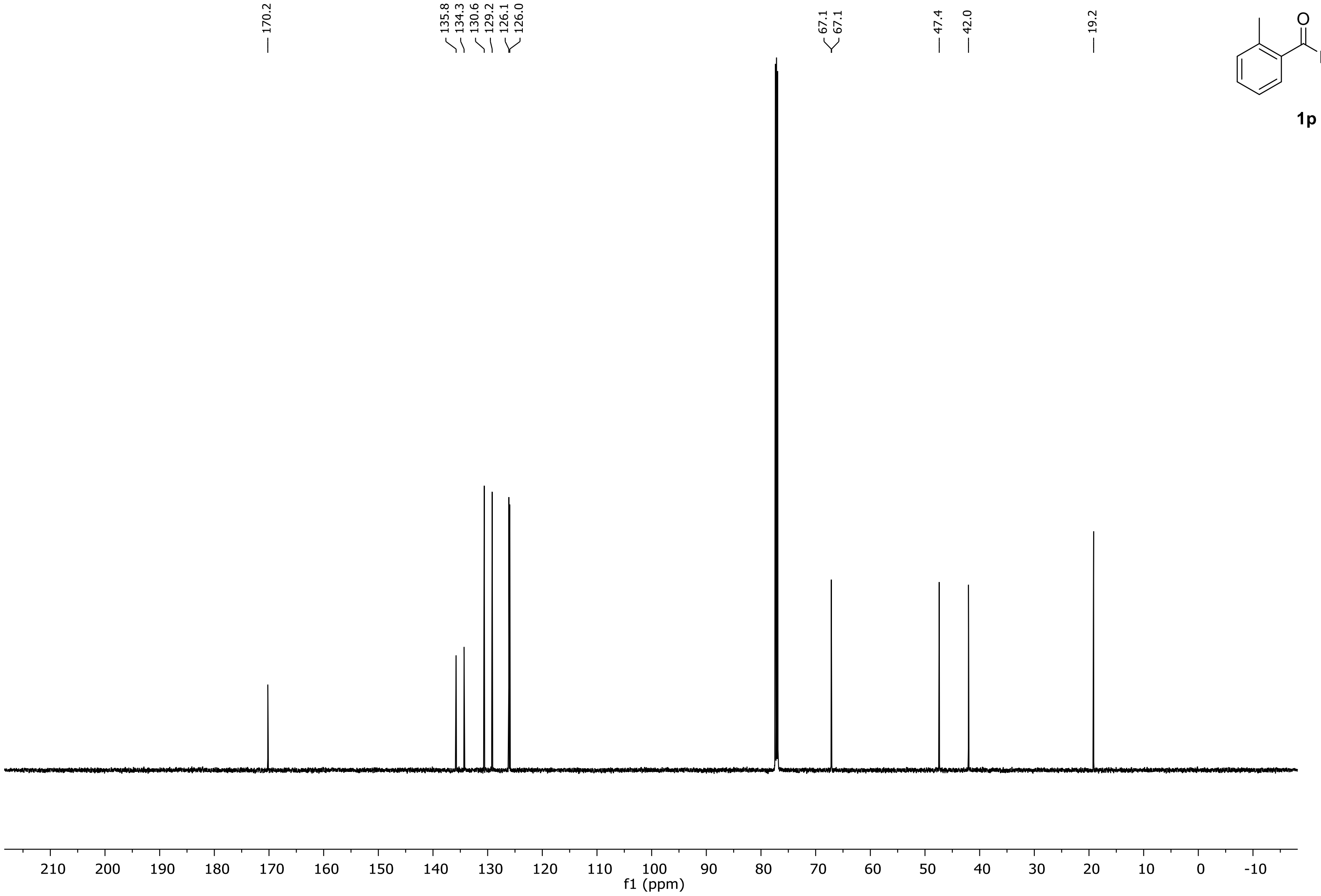

170.2
135.8
134.3
130.6
129.2
126.1
126.0
67.1
67.1
47.4
42.0
19.2
210 200 190 180 170 160 150 140 130 120 110 100 90 80 70 60 50 40 30 20 10 0 -10
f1 (ppm)

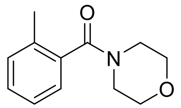

O
N
O

1p

^1H, ^1H COSY

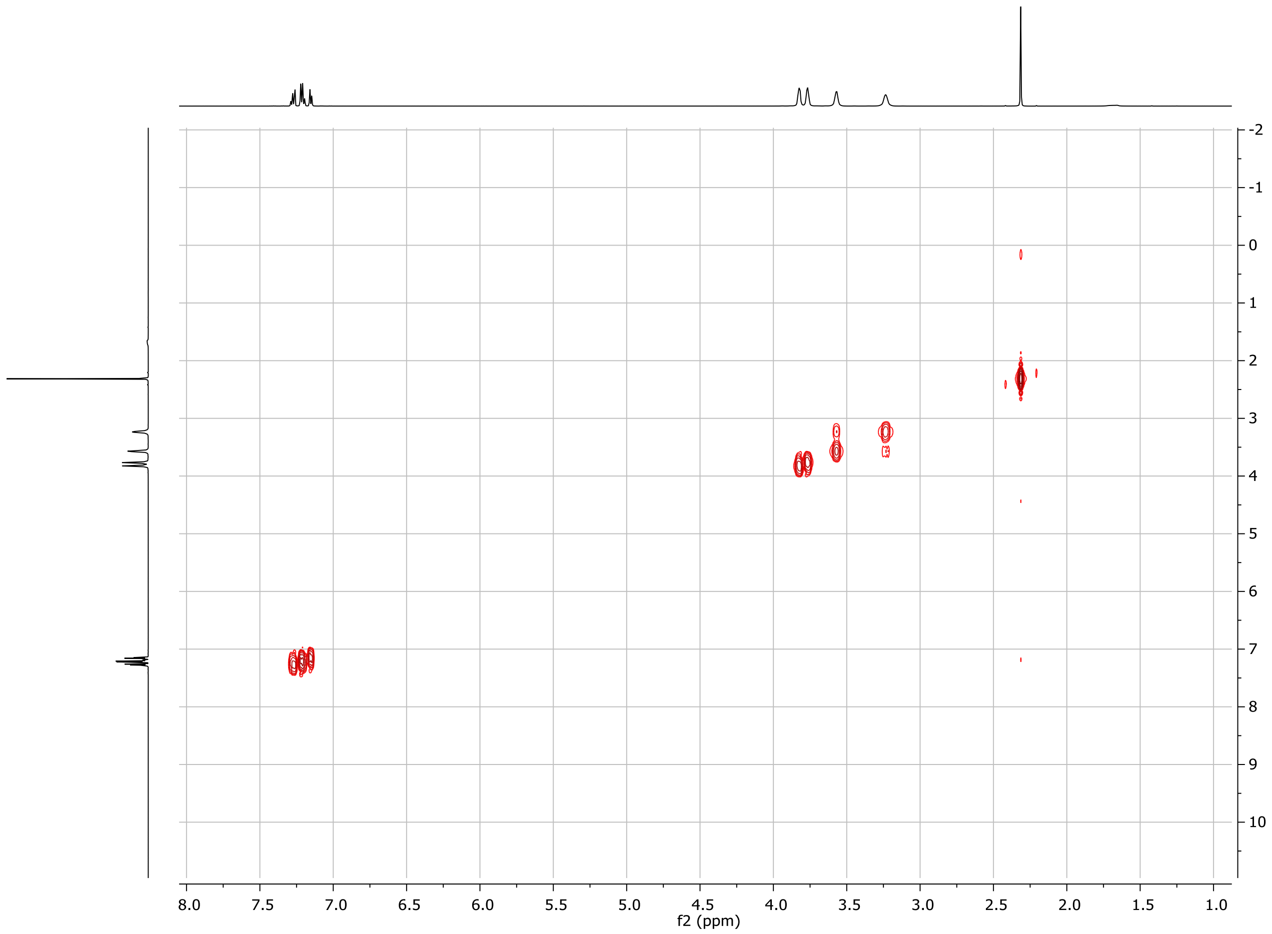

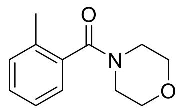

1p

^{1}H, ^{13}C HMBC

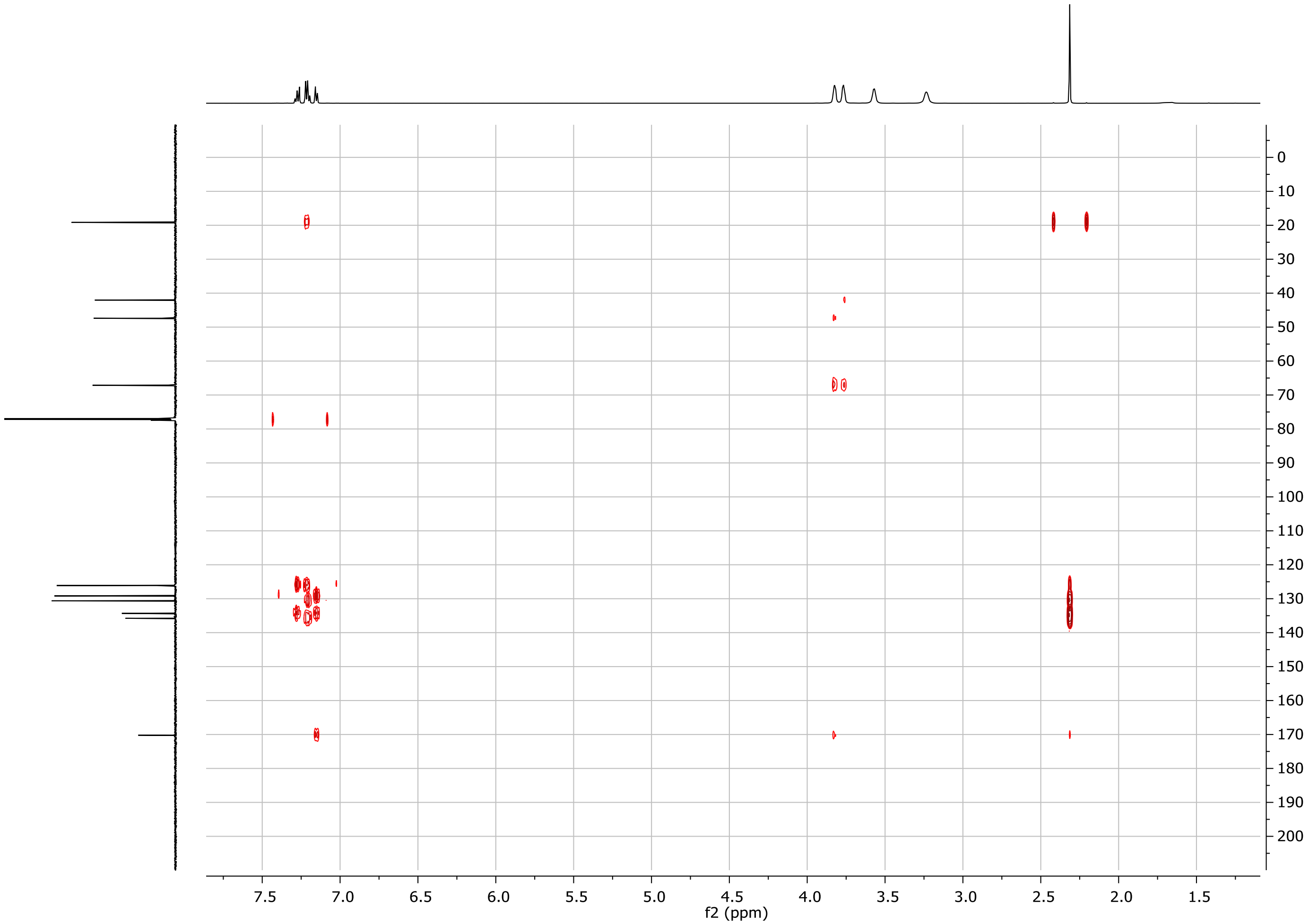

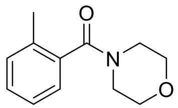

1p

^{1}H, ^{13}C HSQC

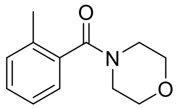

1p

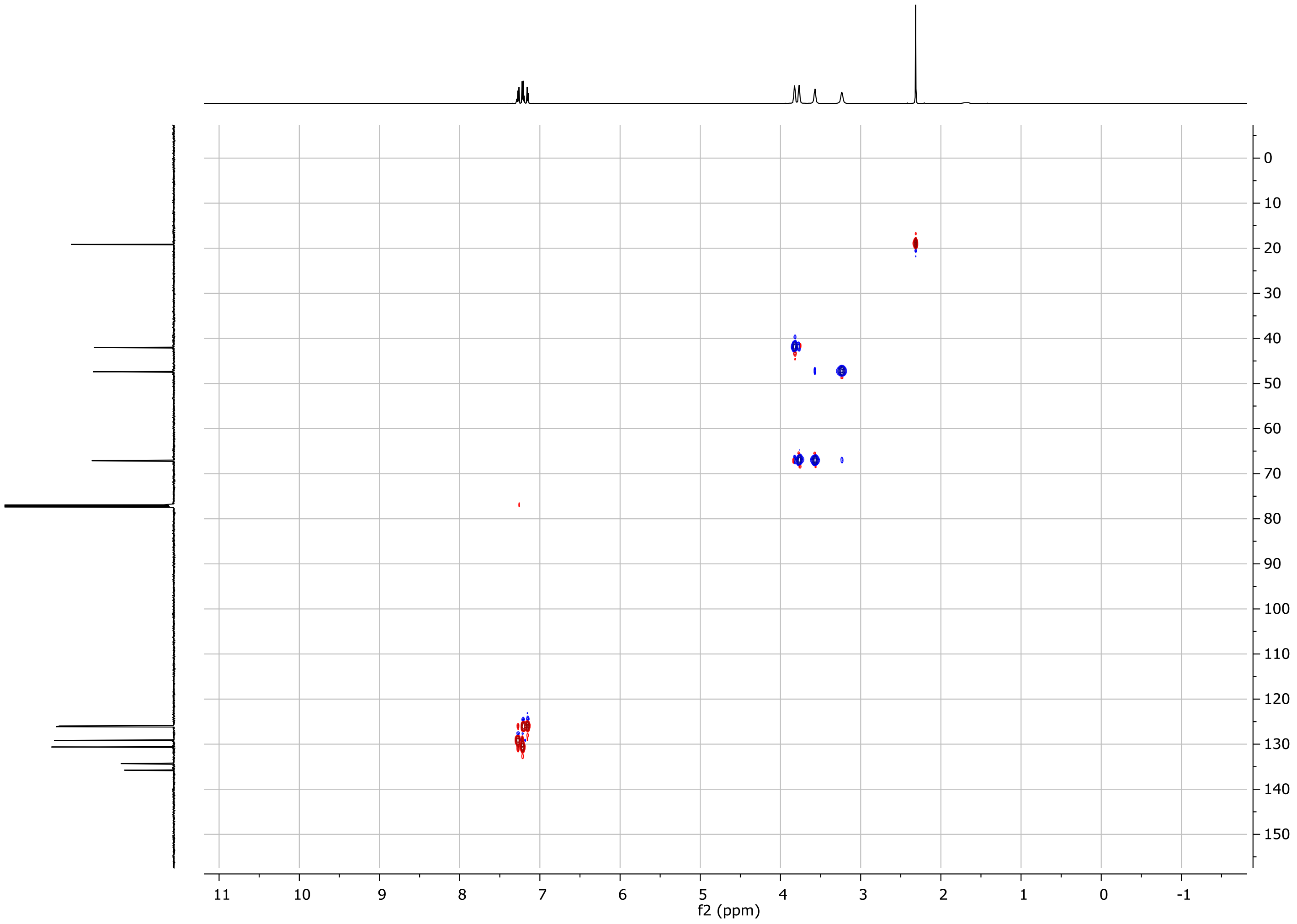

HRMS

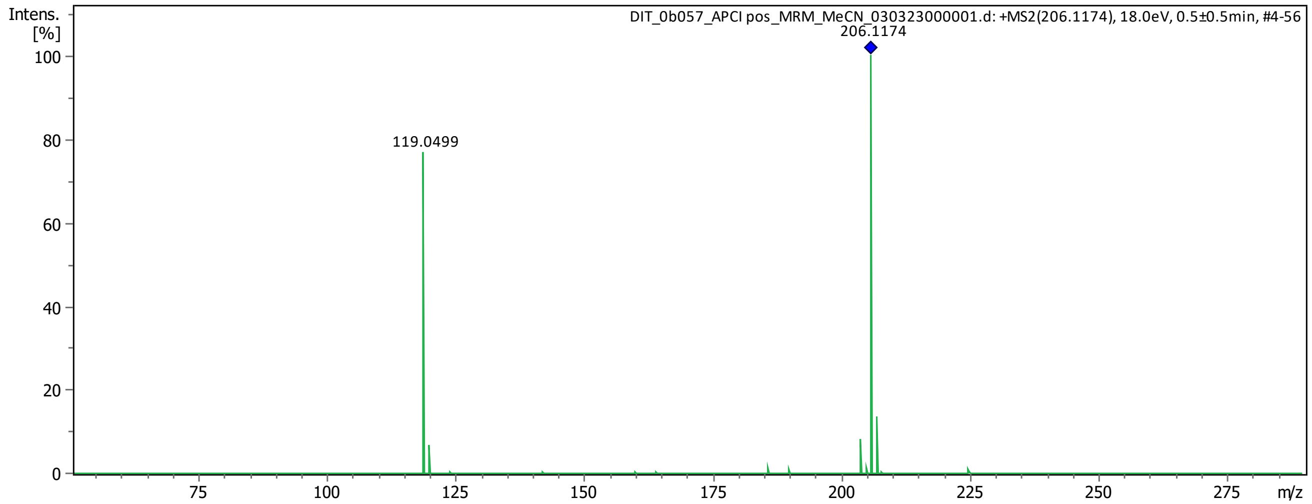

Intens.
[%]
DIT_0b057_APCI pos_MRM_MeCN_030323000001.d: +MS2(206.1174), 18.0eV, 0.5±0.5min, #4-56
206.1174
119.0499
m/z

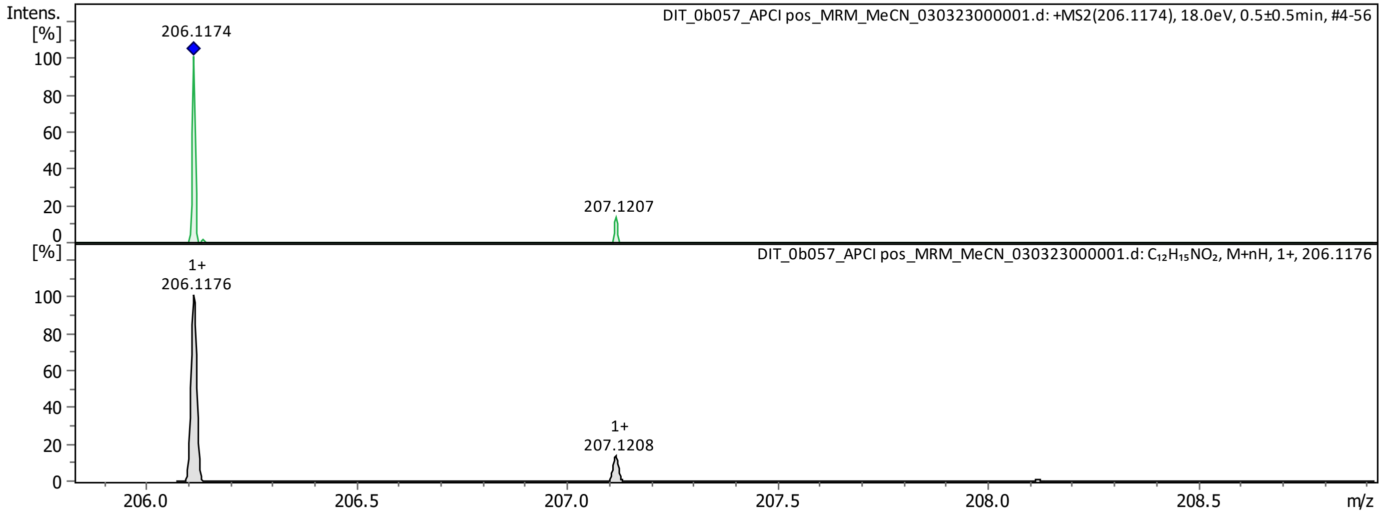

Intens.
[%]
DIT_0b057_APCI pos_MRM_MeCN_030323000001.d: +MS2(206.1174), 18.0eV, 0.5±0.5min, #4-56
206.1174
207.1207
DIT_0b057_APCI pos_MRM_MeCN_030323000001.d: C₁₂H₁₅NO₂, M+nH, 1+, 206.1176
1+
206.1176
1+
207.1208
m/z

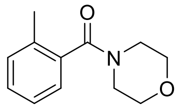

1p

14 Morpholino(4-(trifluoromethyl)phenyl)methanone (1q)

^{1}H NMR

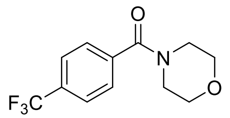

1q

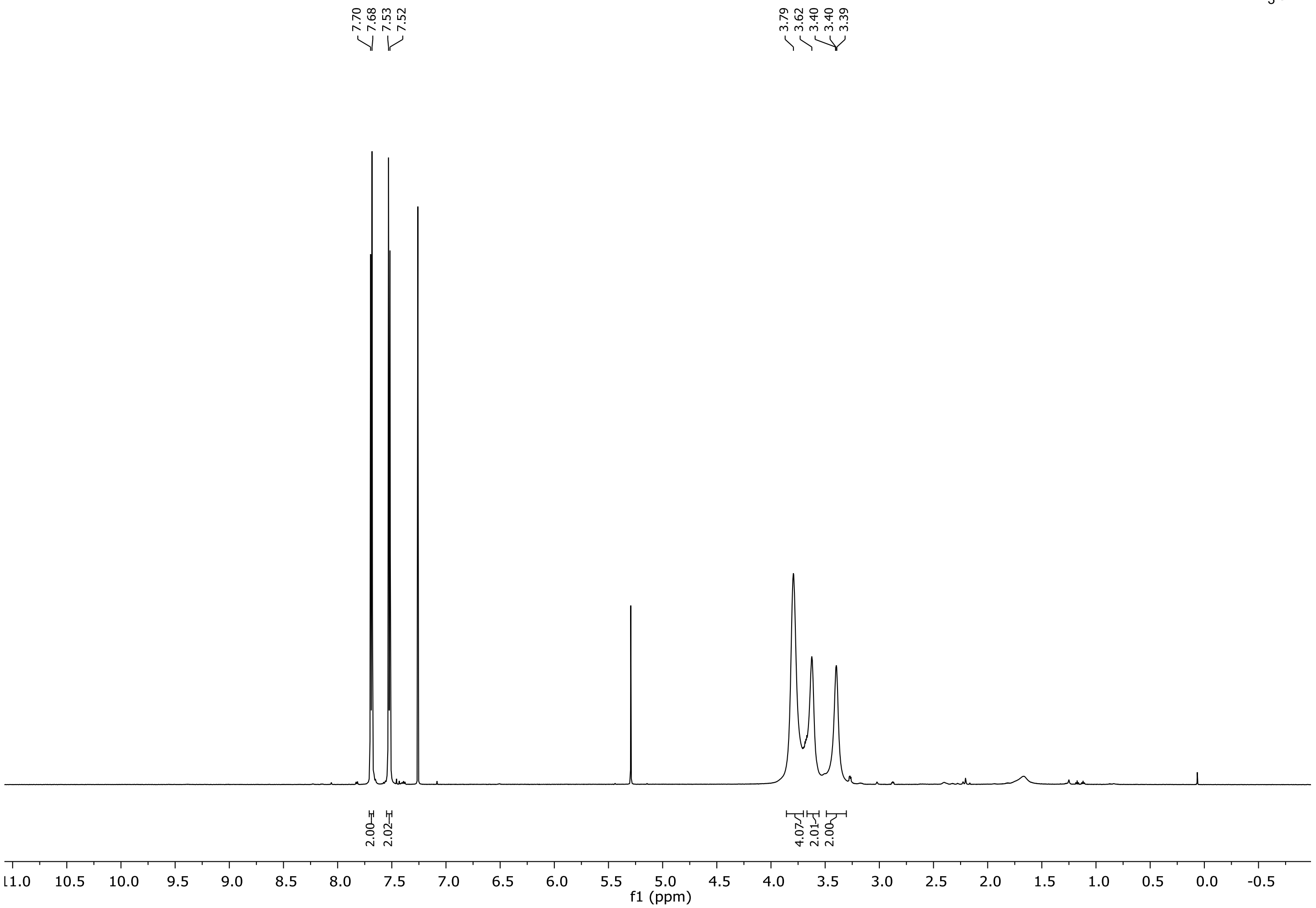

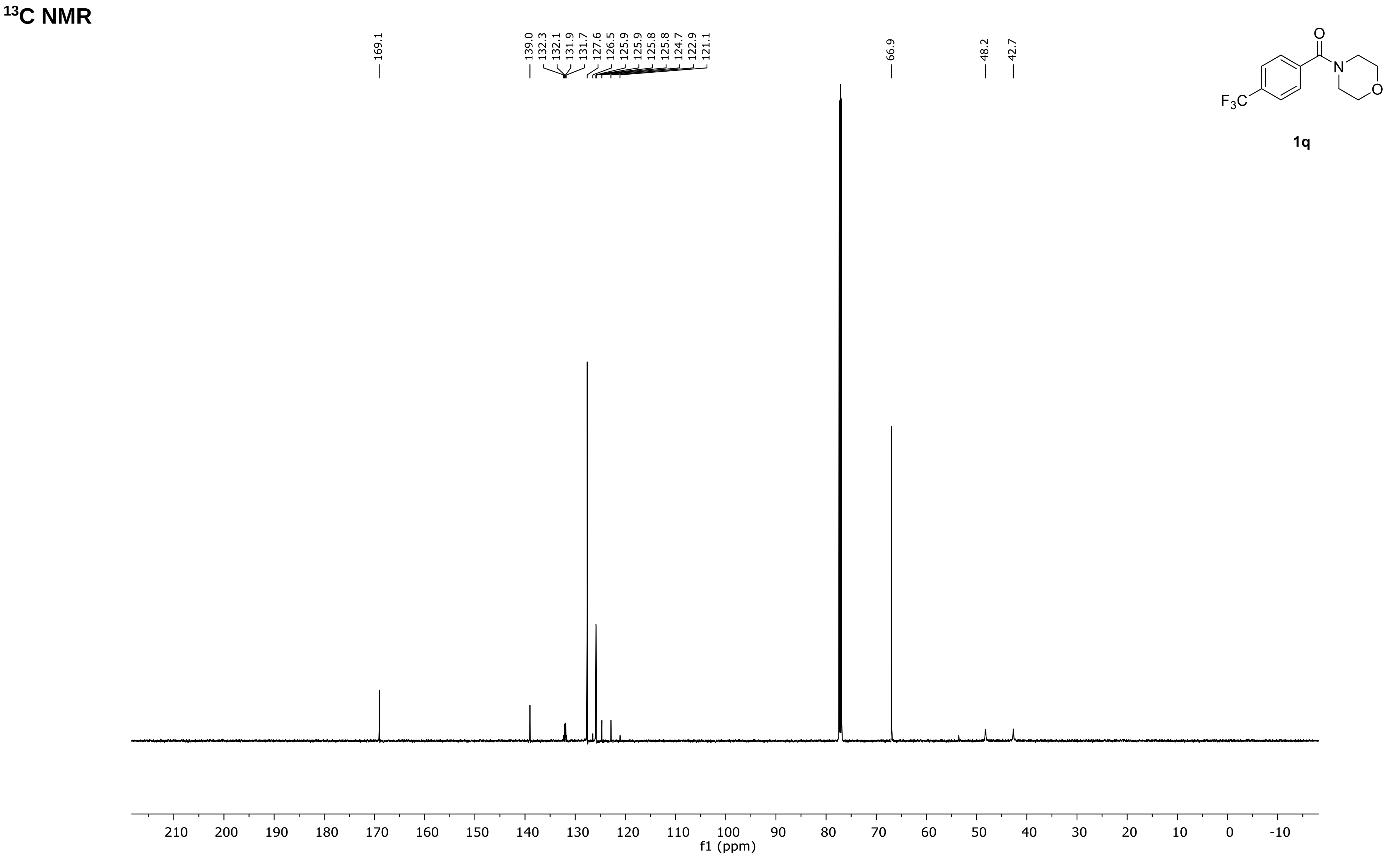
13C NMR
169.1
139.0
132.3
132.1
131.9
131.7
127.6
126.5
125.9
125.9
125.8
125.8
124.7
122.9
121.1
66.9
48.2
42.7
210
200
190
180
170
160
150
140
130
120
110
100
90
80
70
60
50
40
30
20
10
0
-10
f1 (ppm)
O
N
O
F3C

1q

^{19}F NMR

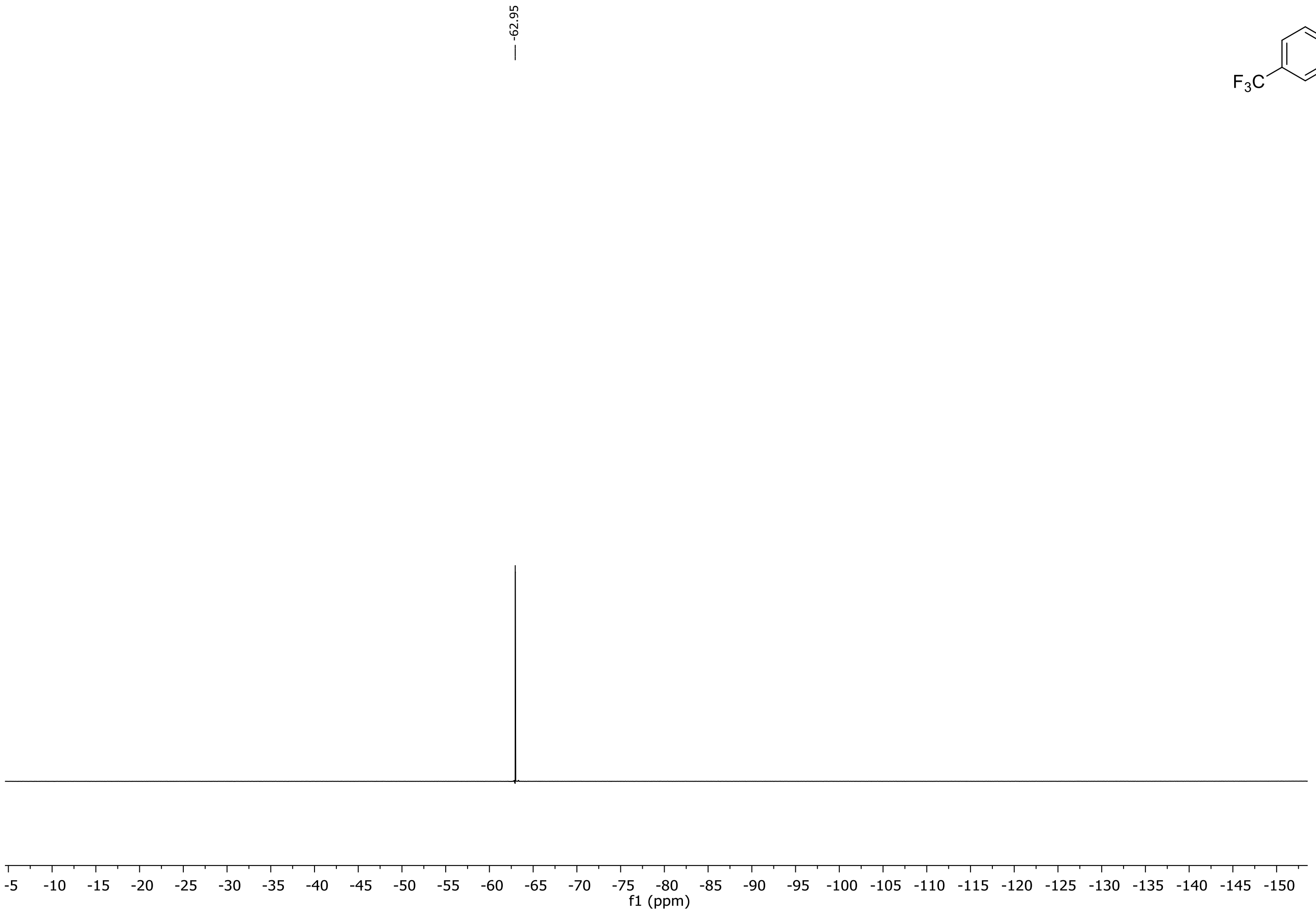

-62.95
-5 -10 -15 -20 -25 -30 -35 -40 -45 -50 -55 -60 -65 -70 -75 -80 -85 -90 -95 -100 -105 -110 -115 -120 -125 -130 -135 -140 -145 -150
f1 (ppm)

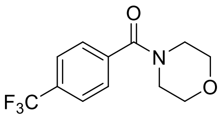

O
N
O
F3C

1q

^1H, ^1H COSY

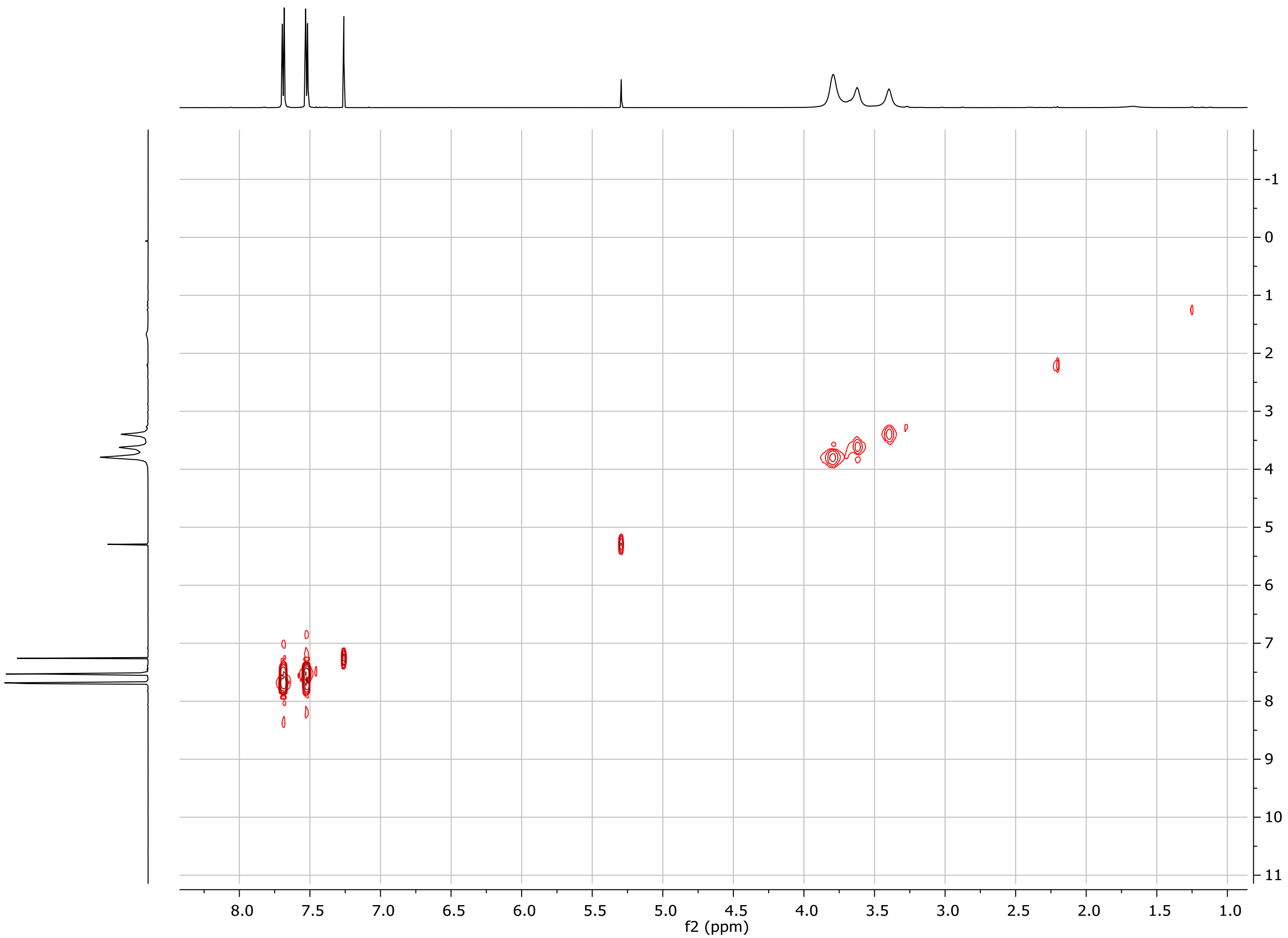

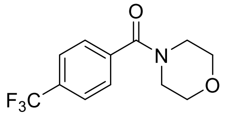

1q

^1H, ^{13}C HMBC

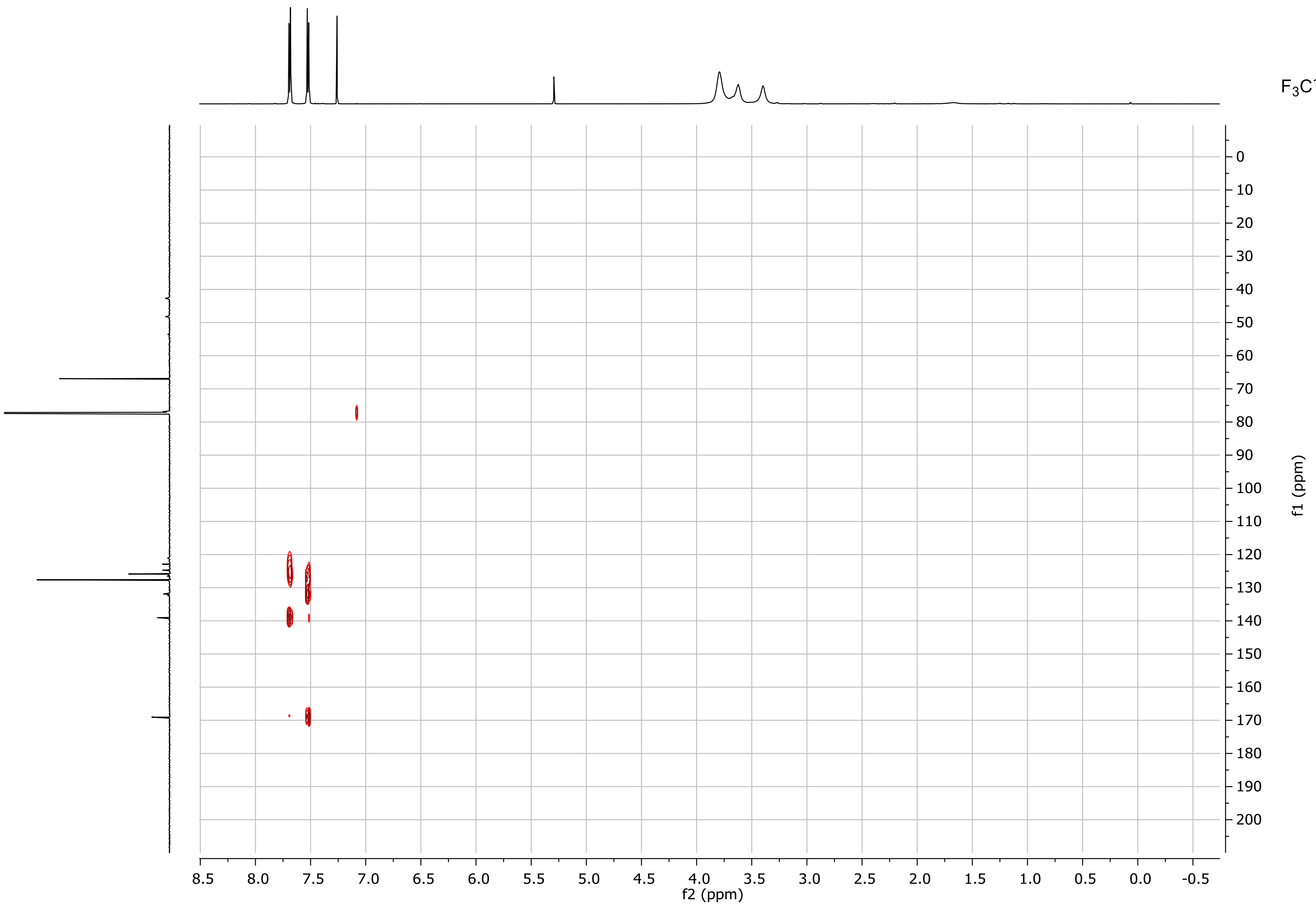

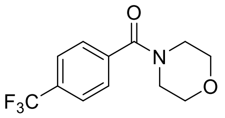

1q

^1H, ^{13}C HSQC

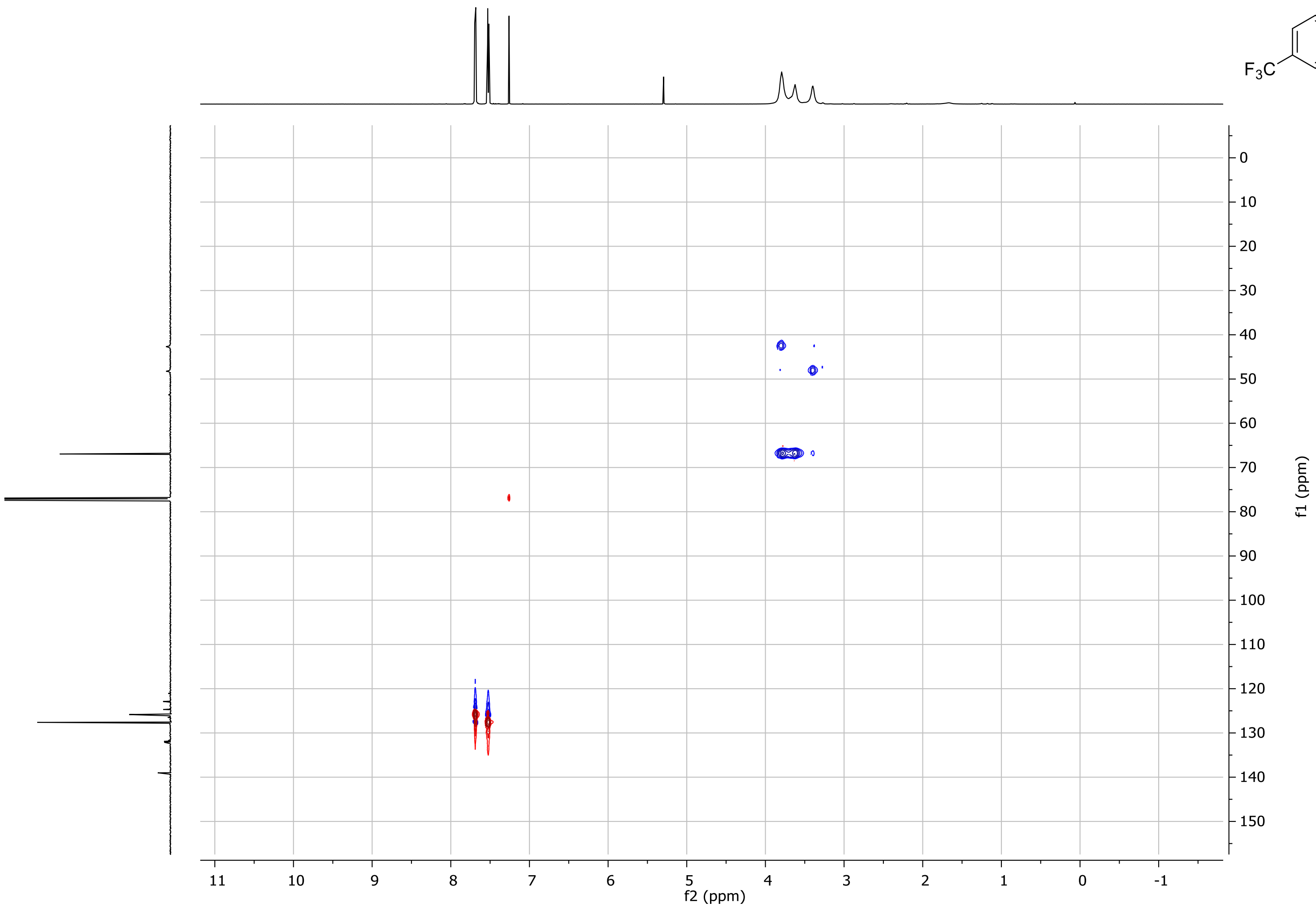

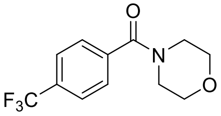

1q

HRMS

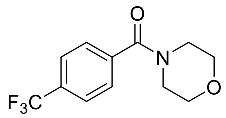

1q

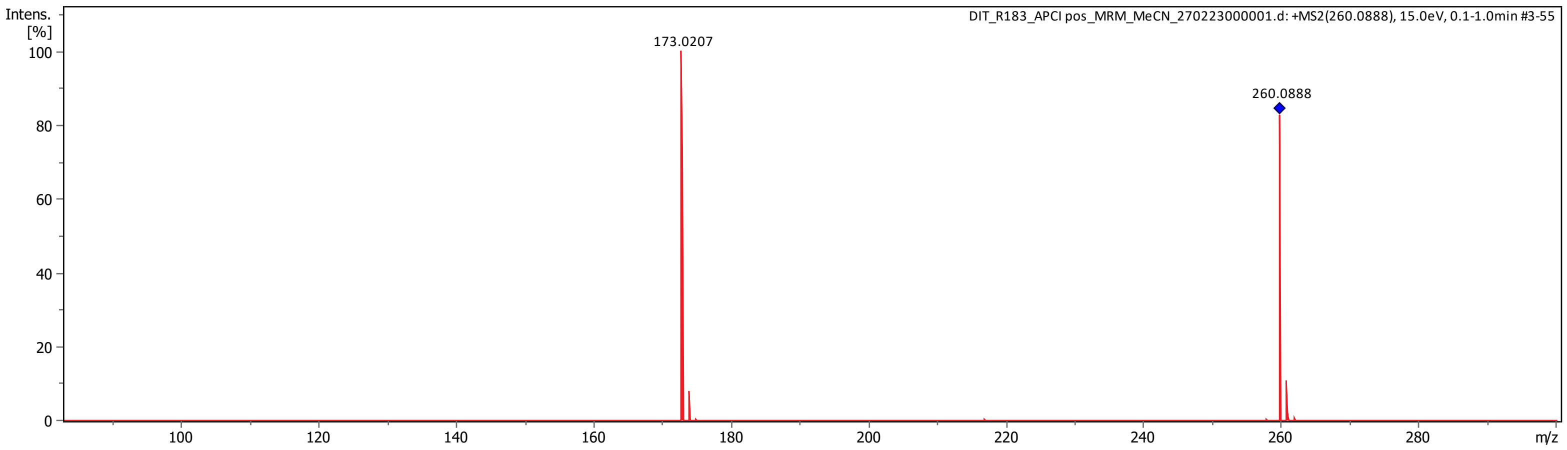

Intens. [%]
DIT_R183_APCI pos_MRM_MeCN_270223000001.d: +MS2(260.0888), 15.0eV, 0.1-1.0min #3-55
173.0207
260.0888
m/z

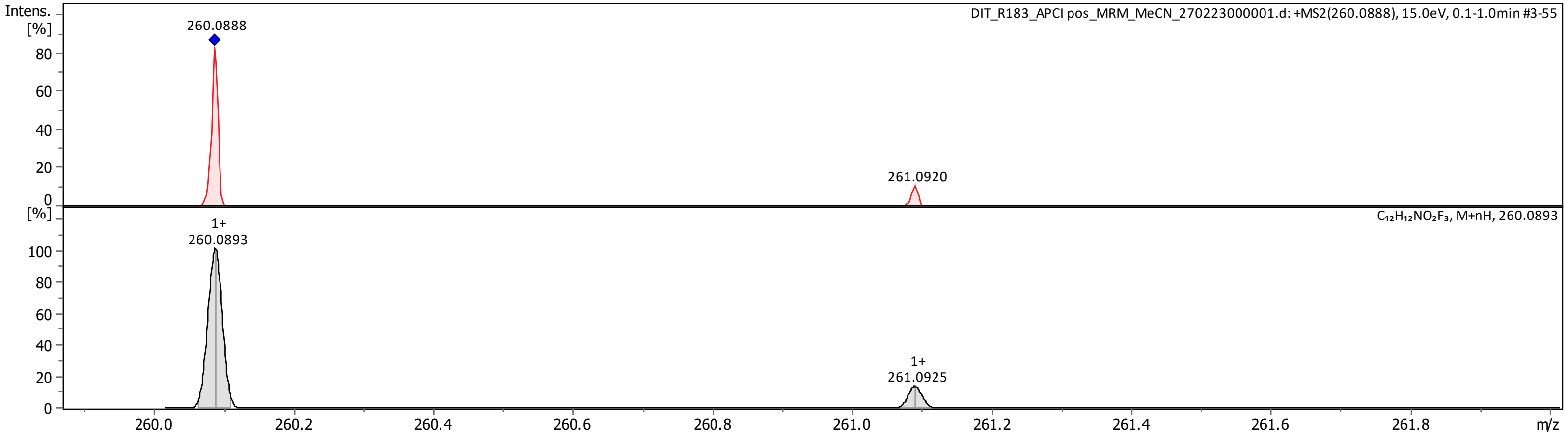

Intens. [%]
DIT_R183_APCI pos_MRM_MeCN_270223000001.d: +MS2(260.0888), 15.0eV, 0.1-1.0min #3-55
260.0888
261.0920
C₁₂H₁₂NO₂F₃, M+nH, 260.0893
1+ 260.0893
1+ 261.0925
m/z

15 (4-Methoxyphenyl)(morpholino)methanone (1r)

^{1}H NMR

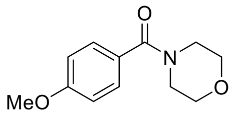

1r

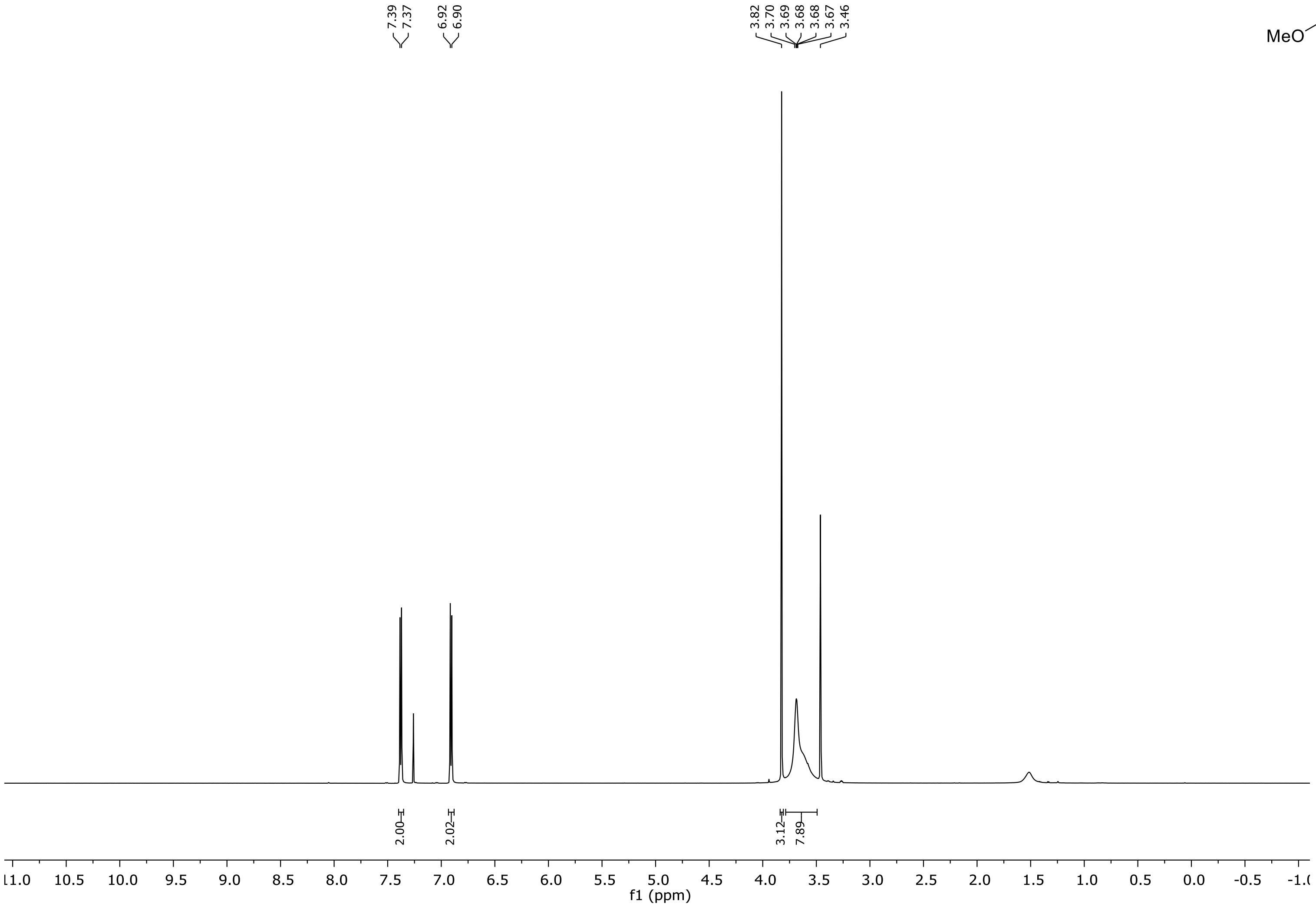

^{13}C NMR

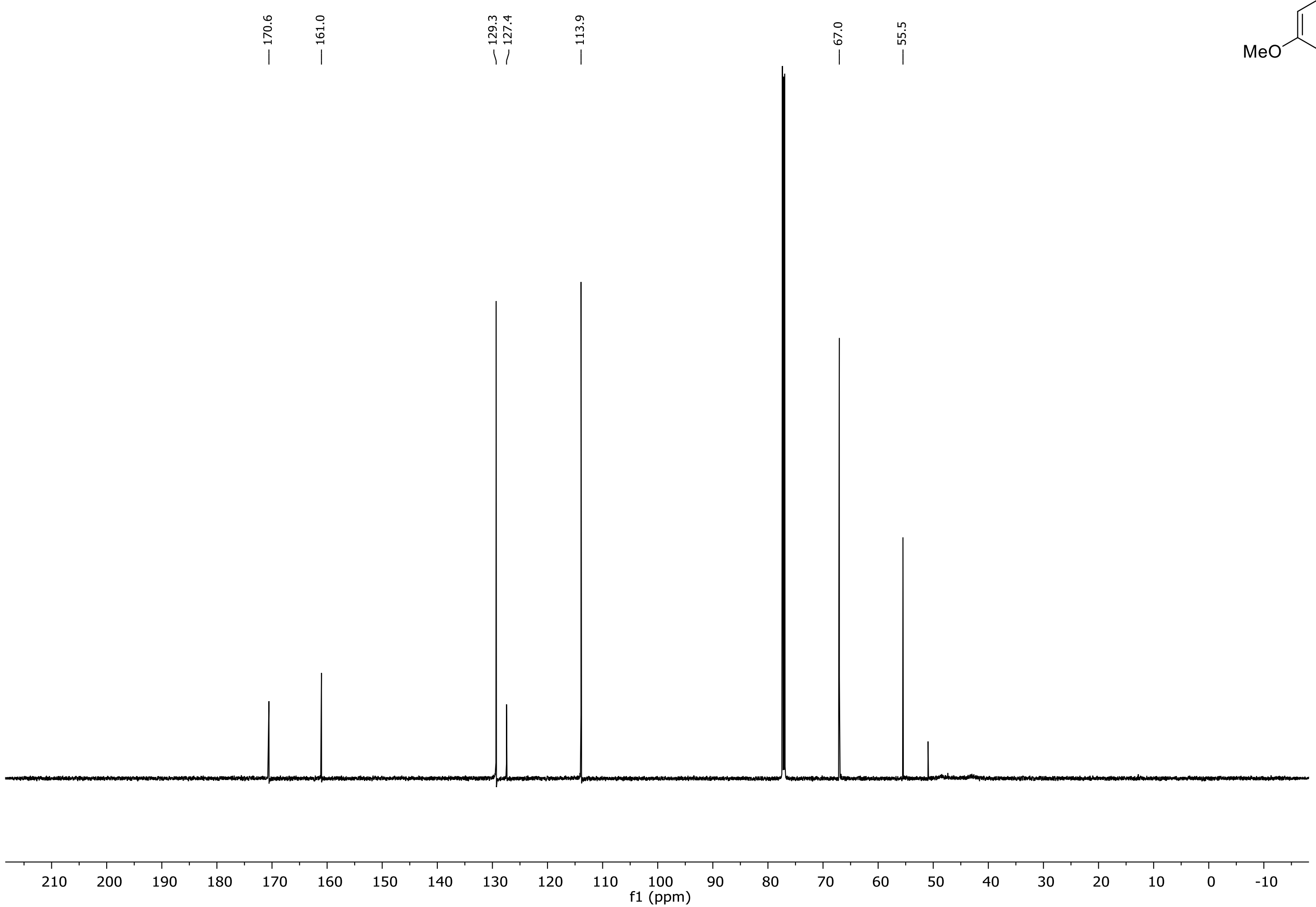
170.6
161.0
129.3
127.4
113.9
67.0
55.5
210
200
190
180
170
160
150
140
130
120
110
100
90
80
70
60
50
40
30
20
10
0
-10
f1 (ppm)

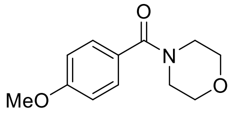
O
N
O
MeO

1r

^1H, ^1H COSY

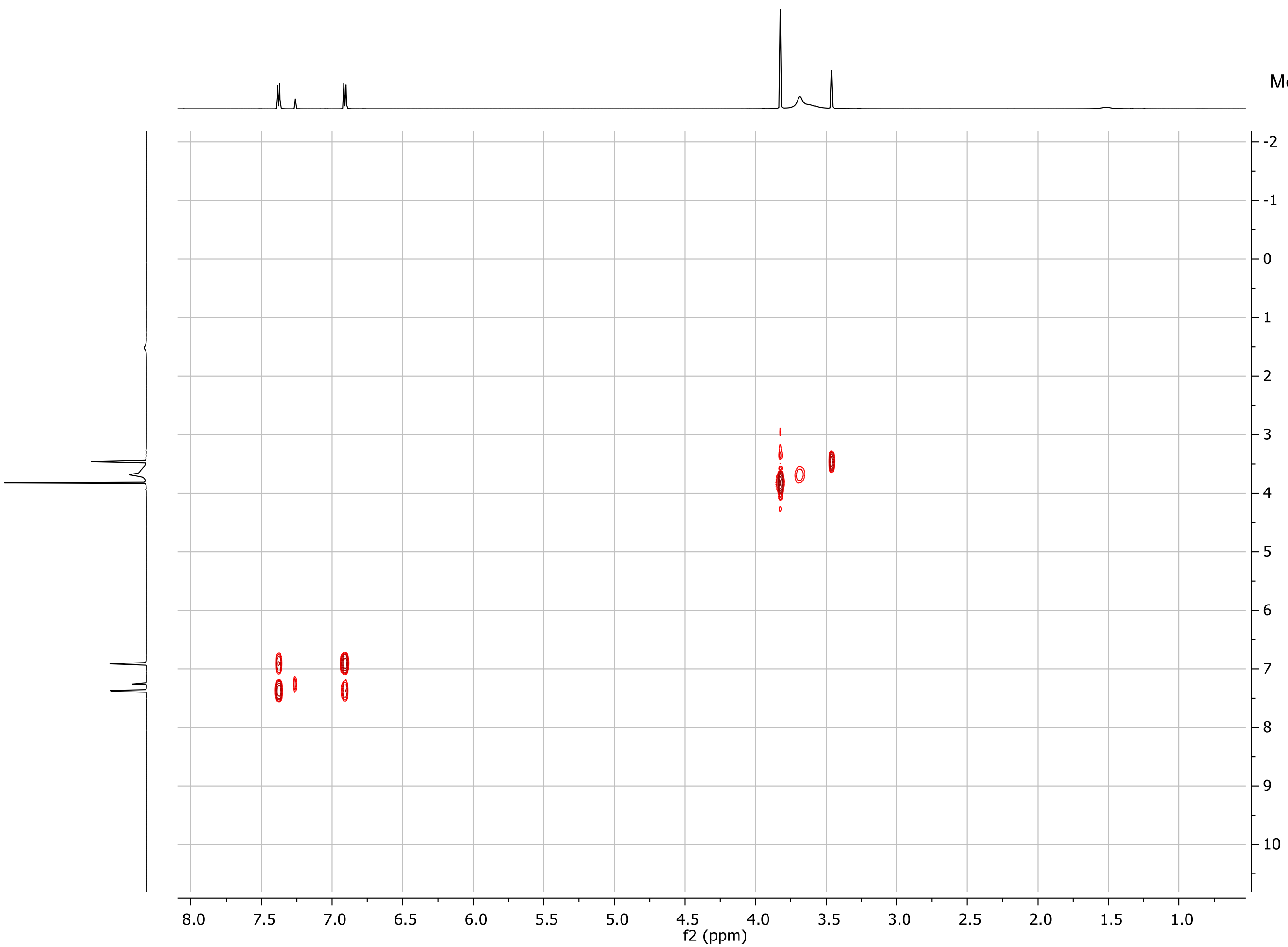

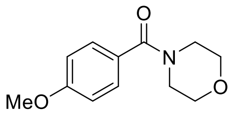

1r

^{1}H, ^{13}C HMBC

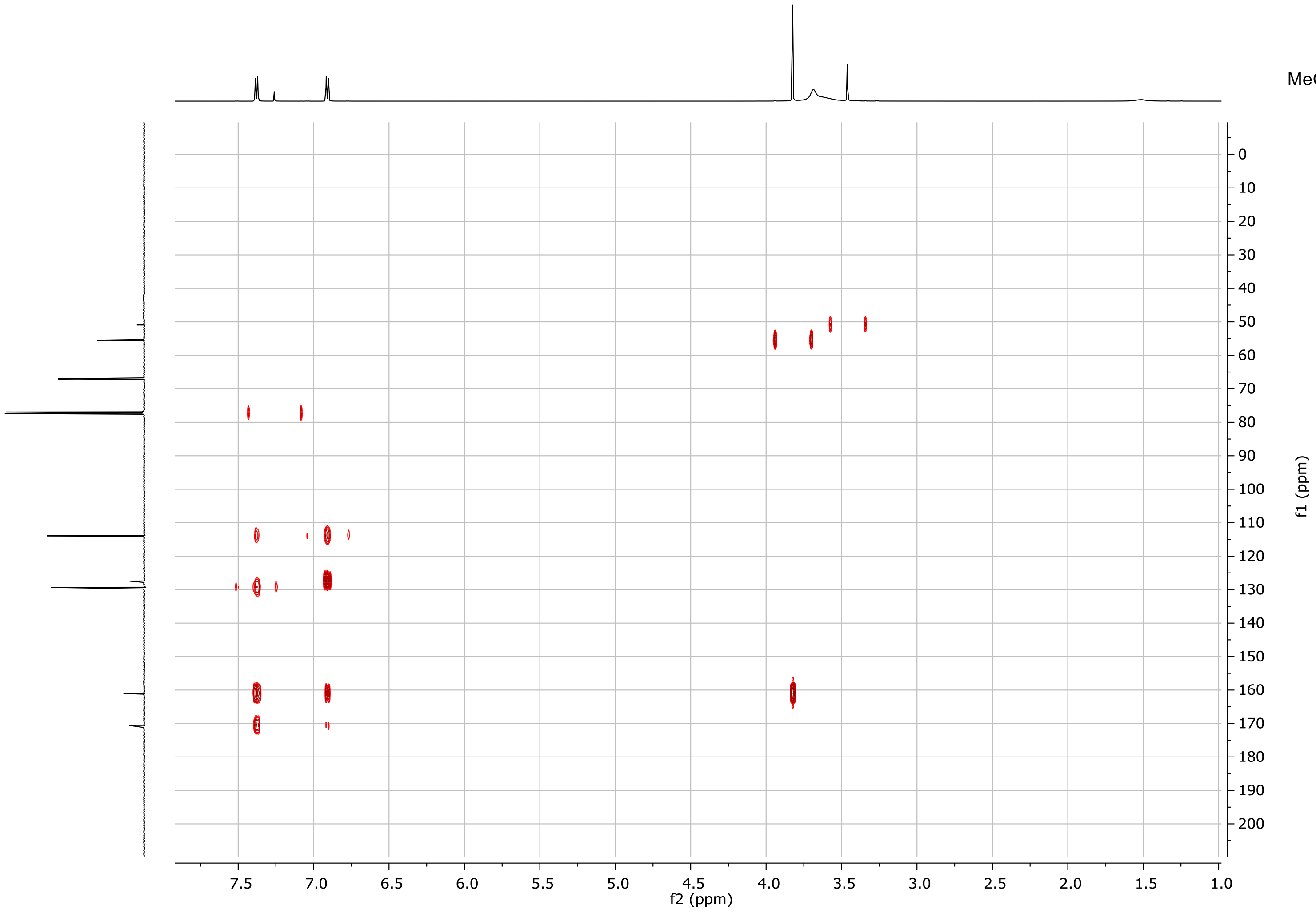

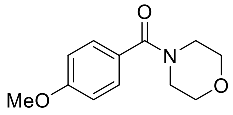

1r

^{1}H, ^{13}C HSQC

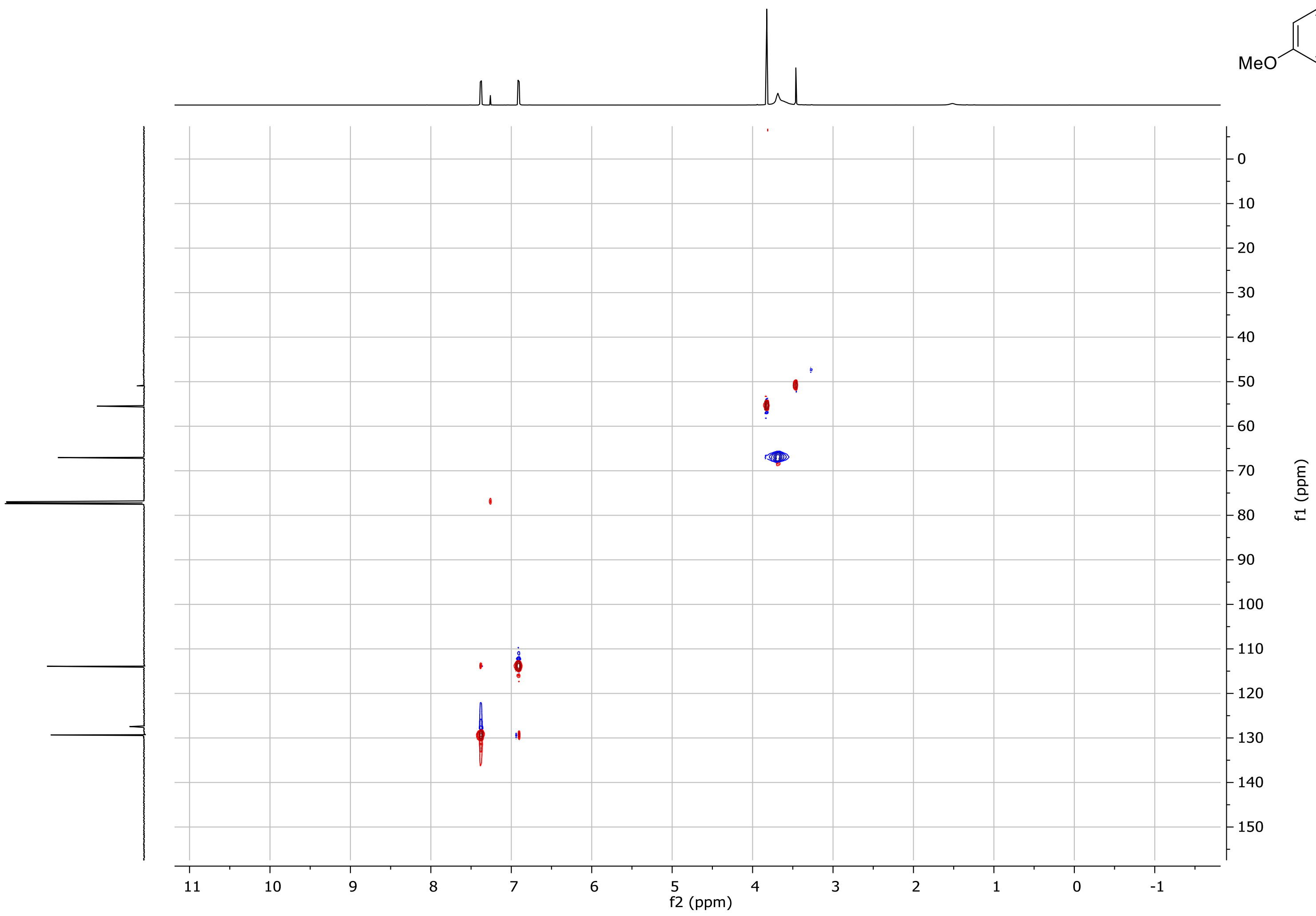

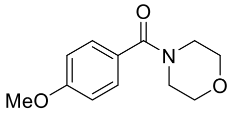

1r

HRMS

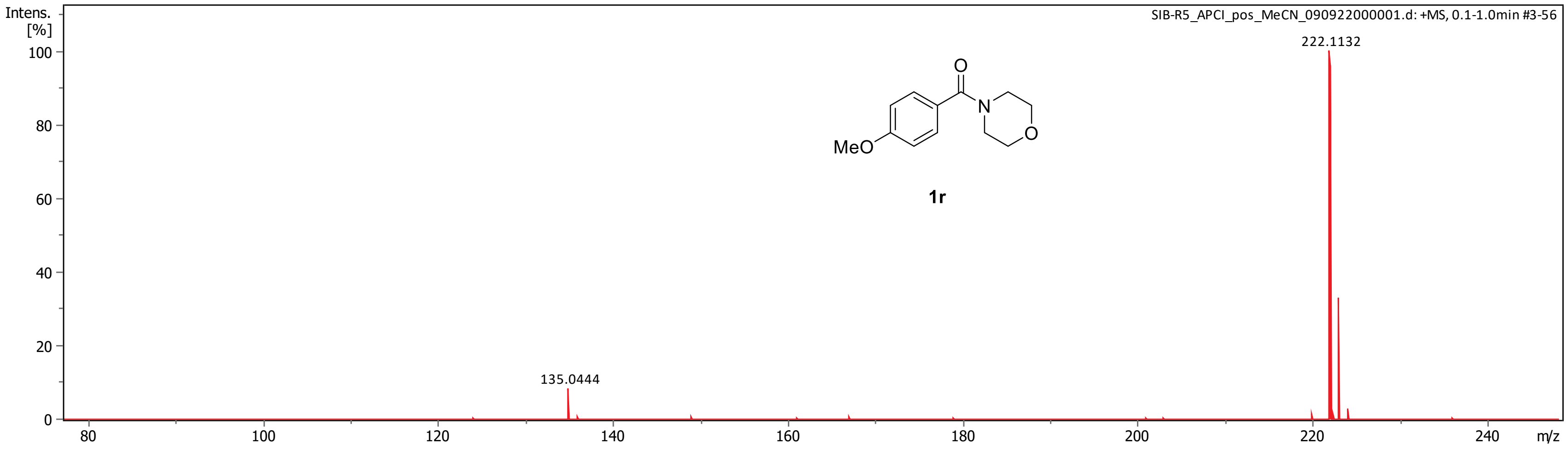
Intens.
[%]
100
80
60
40
20
0
SIB-R5_APCI_pos_MeCN_090922000001.d: +MS, 0.1-1.0min #3-56
222.1132
135.0444
MeO
O
N
O
1r
80
100
120
140
160
180
200
220
240
m/z

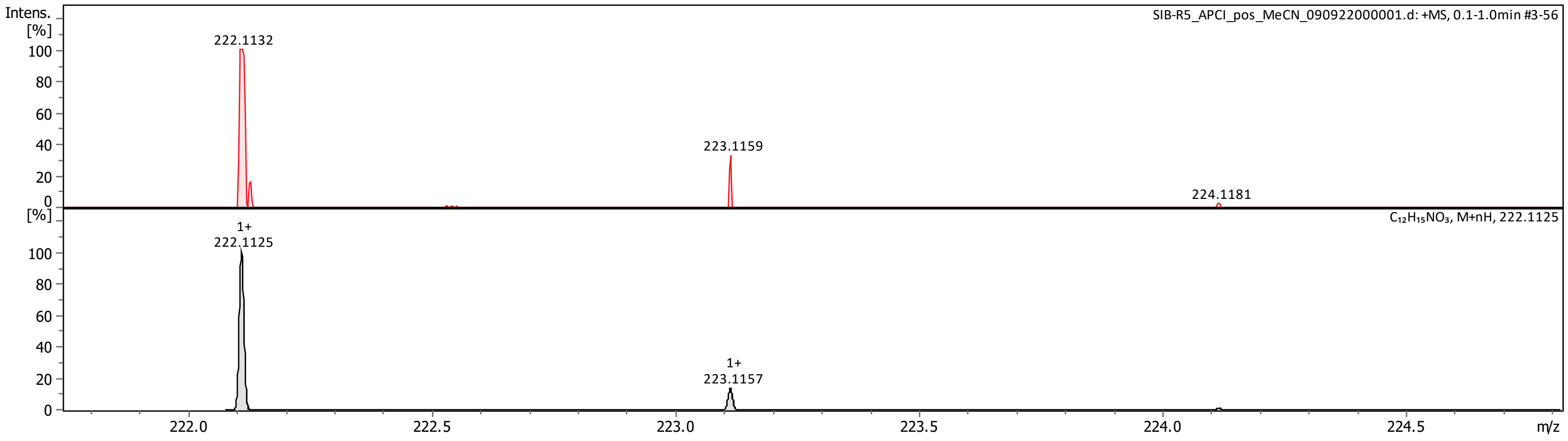
Intens.
[%]
SIB-R5_APCI_pos_MeCN_090922000001.d: +MS, 0.1-1.0min #3-56
222.1132
223.1159
224.1181
C₁₂H₁₅NO₃, M+nH, 222.1125
1+
222.1125
1+
223.1157
222.0
222.5
223.0
223.5
224.0
224.5
m/z

16 (4-Chlorophenyl)(morpholino)methanone (1s)

^{1}H NMR

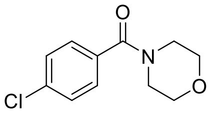

1s

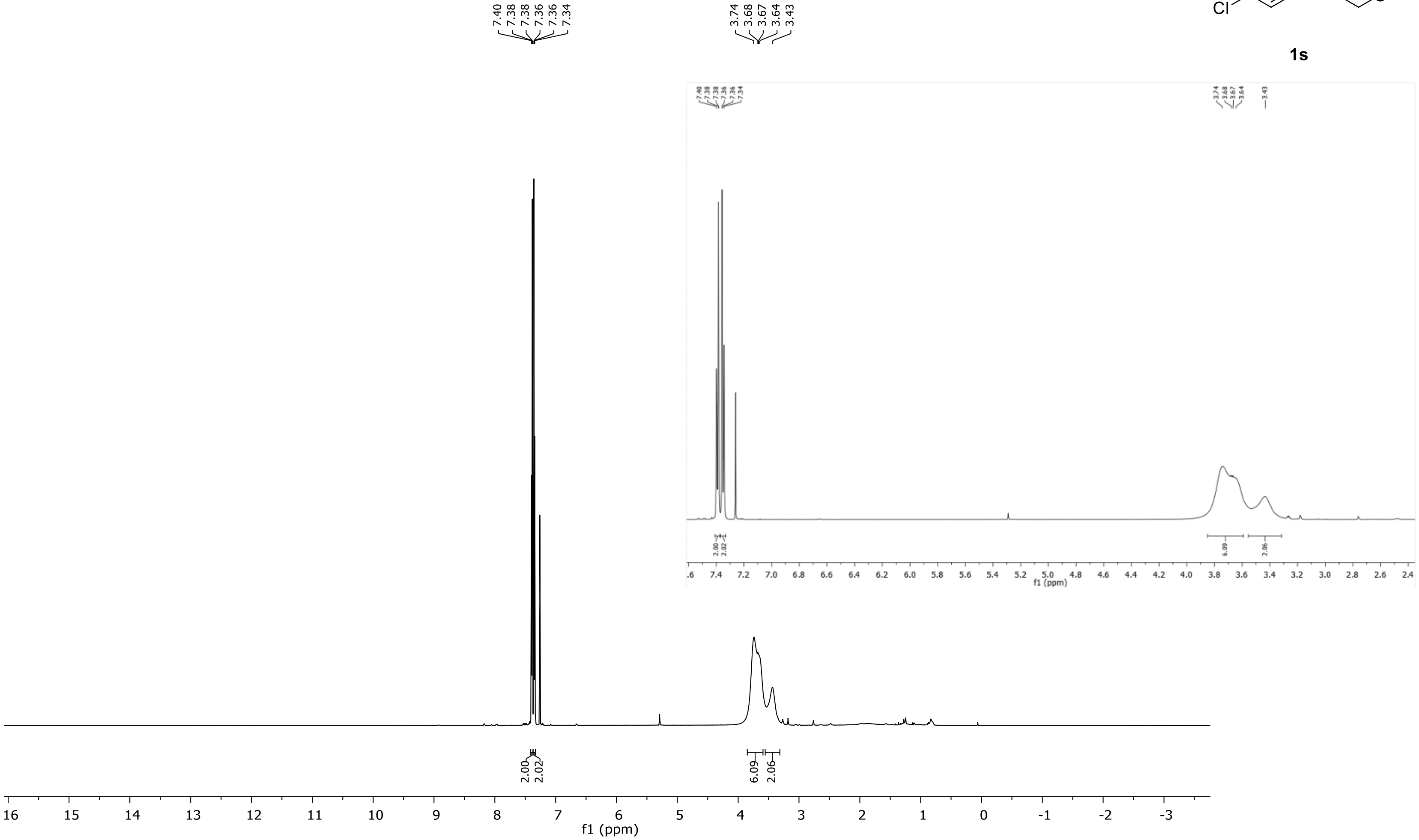

^{13}C NMR

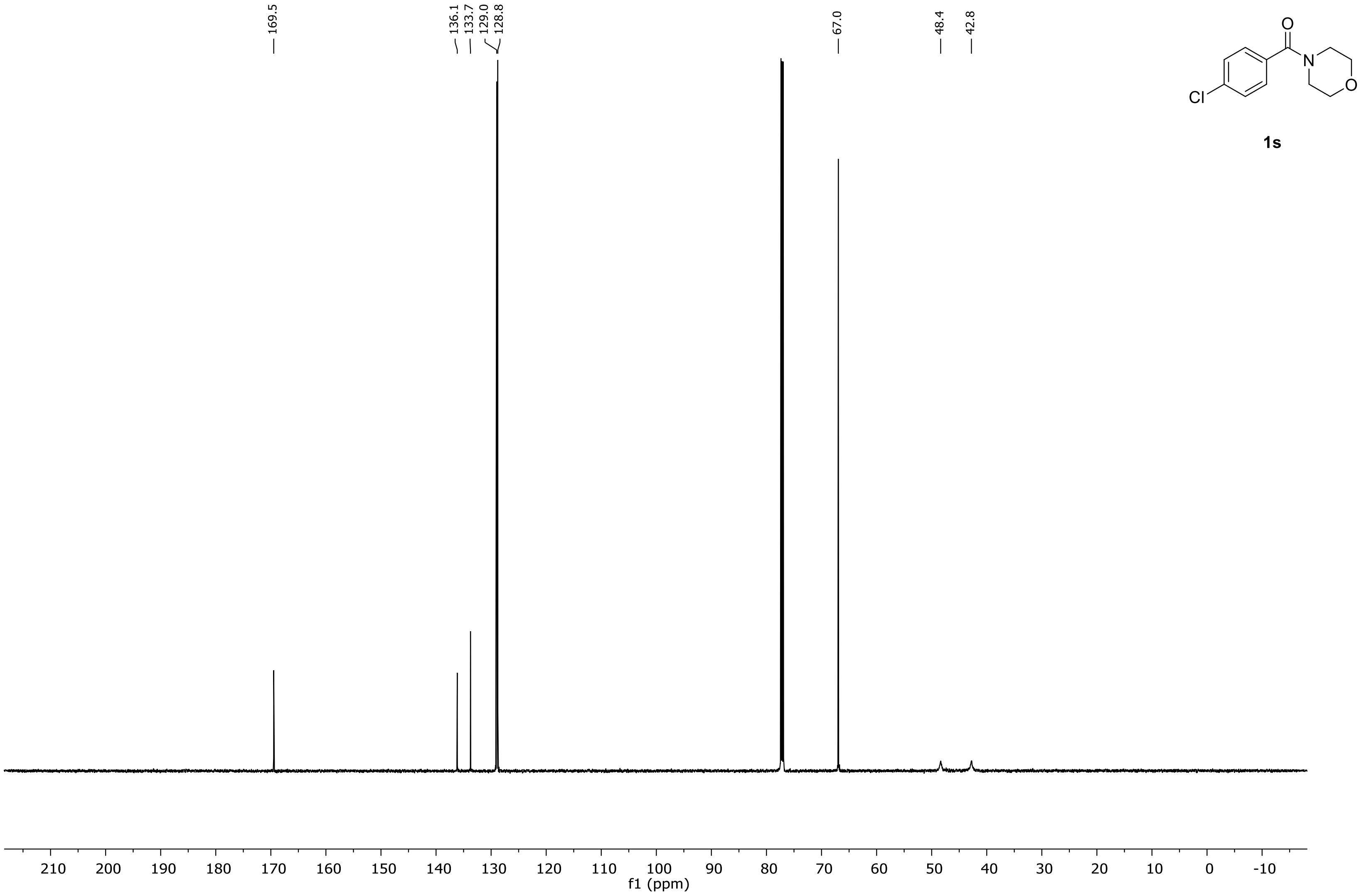
169.5
136.1
133.7
129.0
128.8
67.0
48.4
42.8
210 200 190 180 170 160 150 140 130 120 110 100 90 80 70 60 50 40 30 20 10 0 -10
f1 (ppm)
O
N
O
Cl

1s

^{1}H, ^{1}H COSY

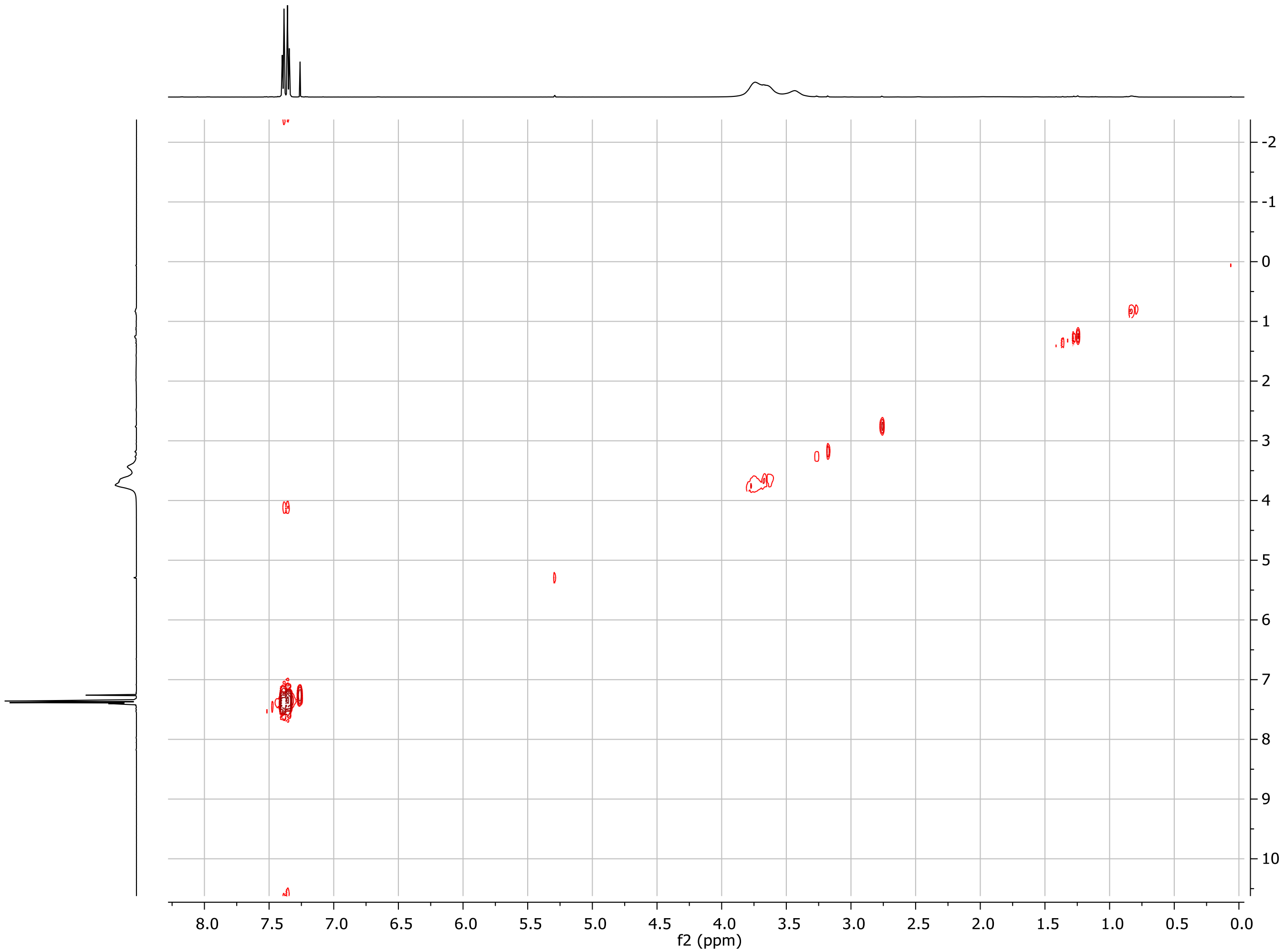

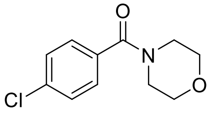

1s

^{1}H, ^{13}C HMBC

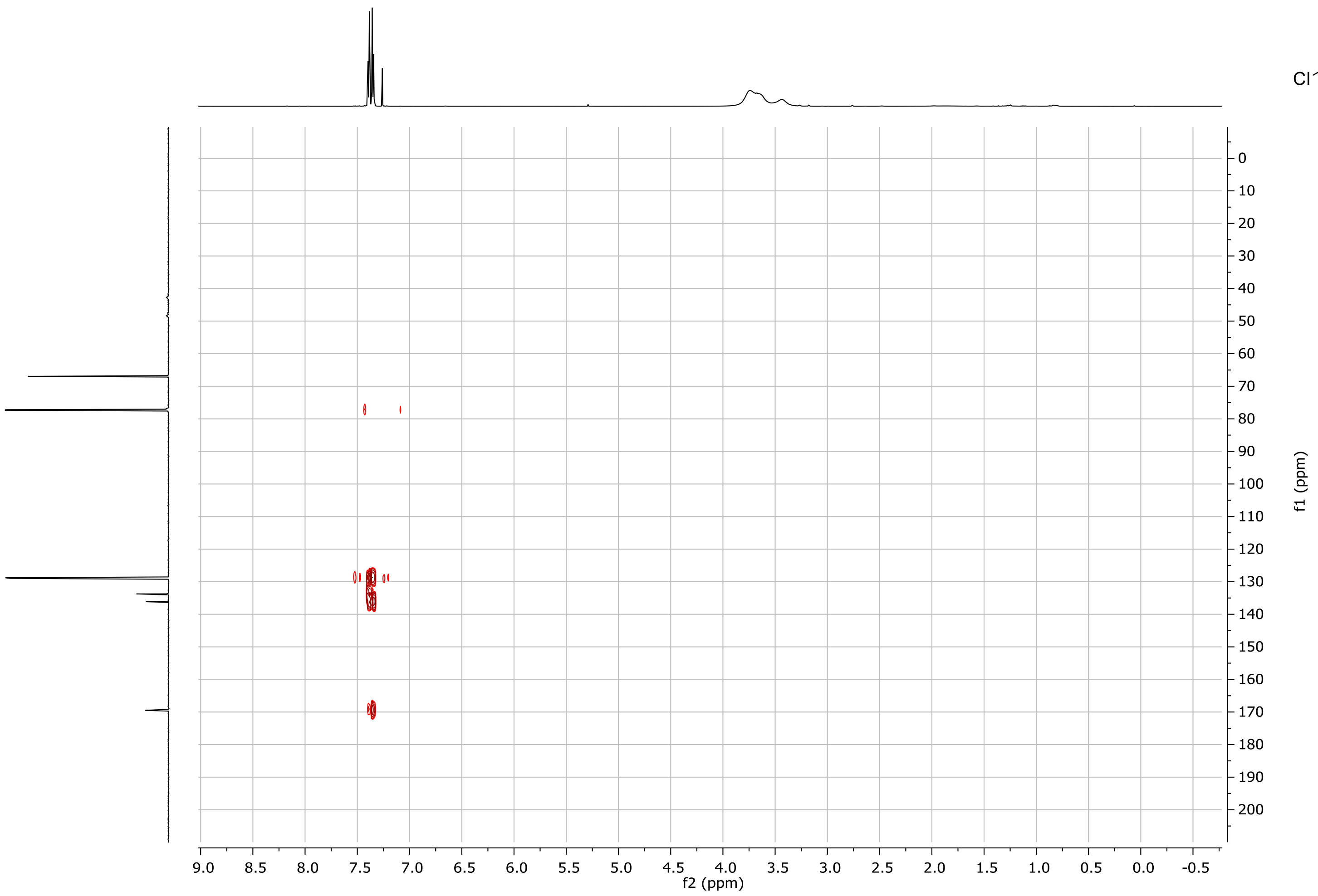

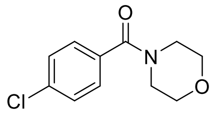

1s

^1H, ^{13}C HSQC

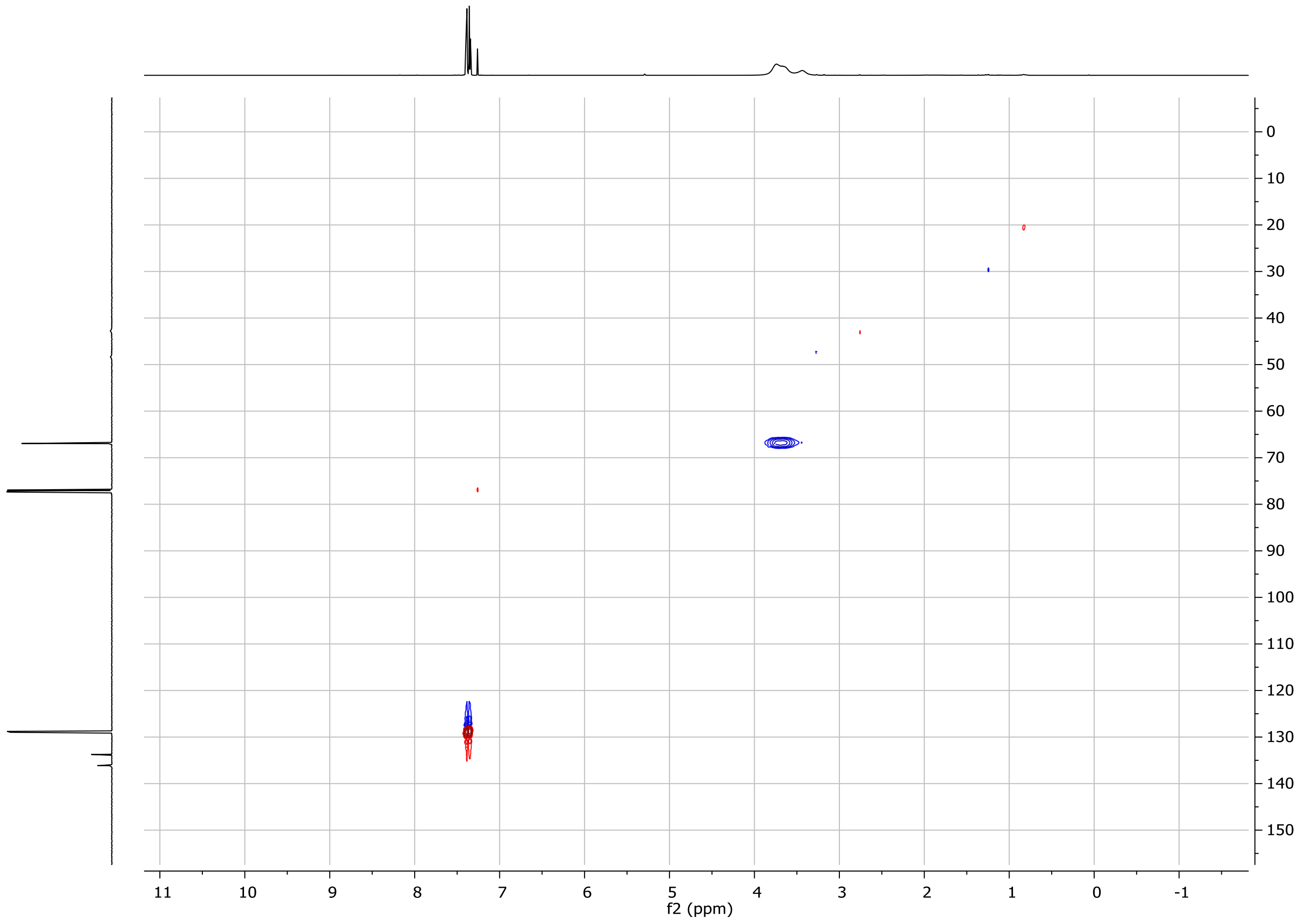

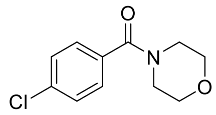

1s

HRMS

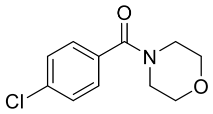

O
N
O
Cl

1s

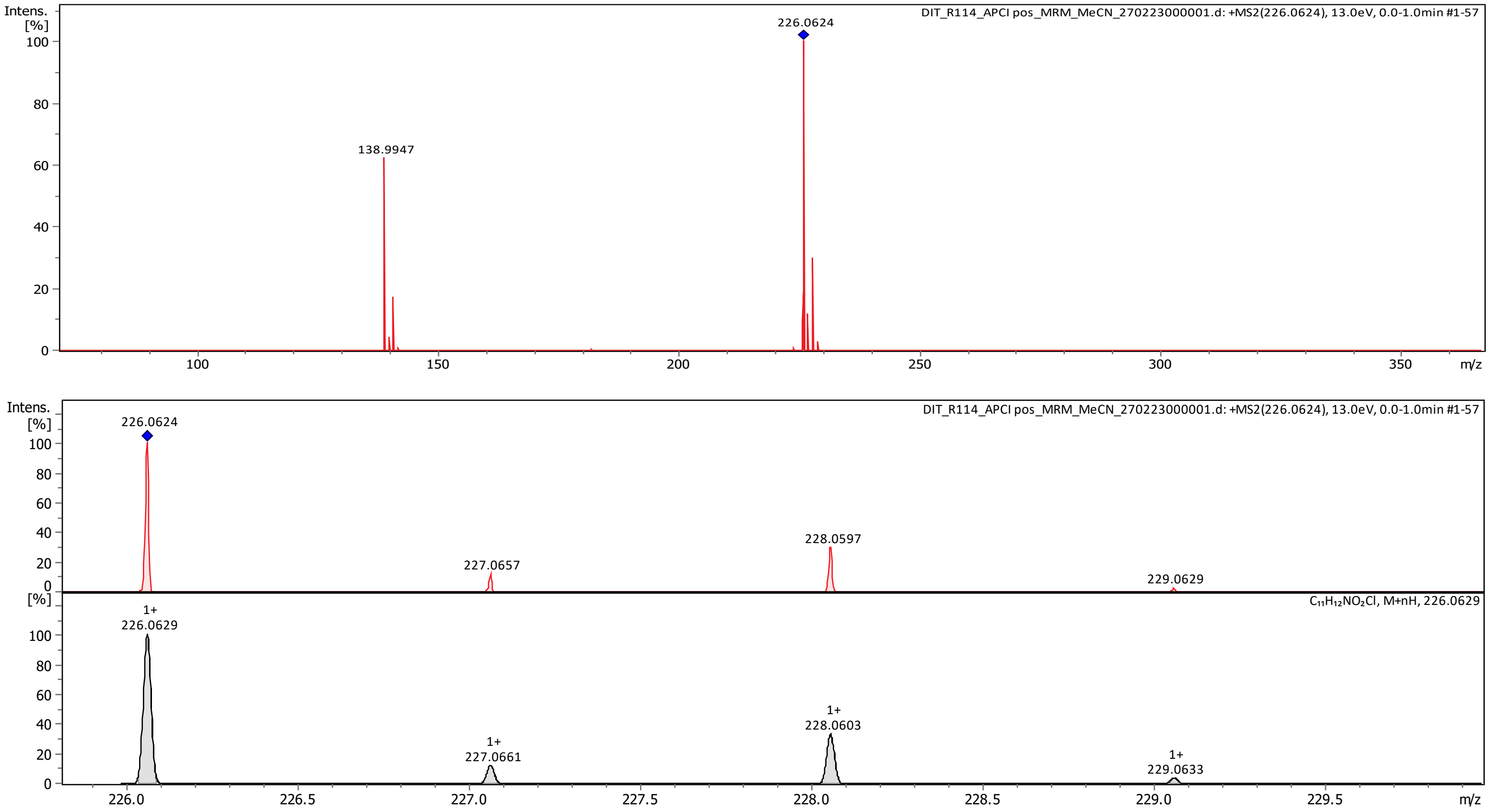

Intens.
[%]
226.0624
138.9947
DIT_R114_APCI pos_MRM_MeCN_270223000001.d: +MS2(226.0624), 13.0eV, 0.0-1.0min #1-57
m/z
226.0624
227.0657
228.0597
229.0629
C11H12NO2Cl, M+nH, 226.0629
1+
226.0629
1+
227.0661
1+
228.0603
1+
229.0633

17 Morpholino(4-(4,4,5,5-tetramethyl-1,3,2-dioxaborolan-2-yl)phenyl)methanone (1t)

^{1}H NMR

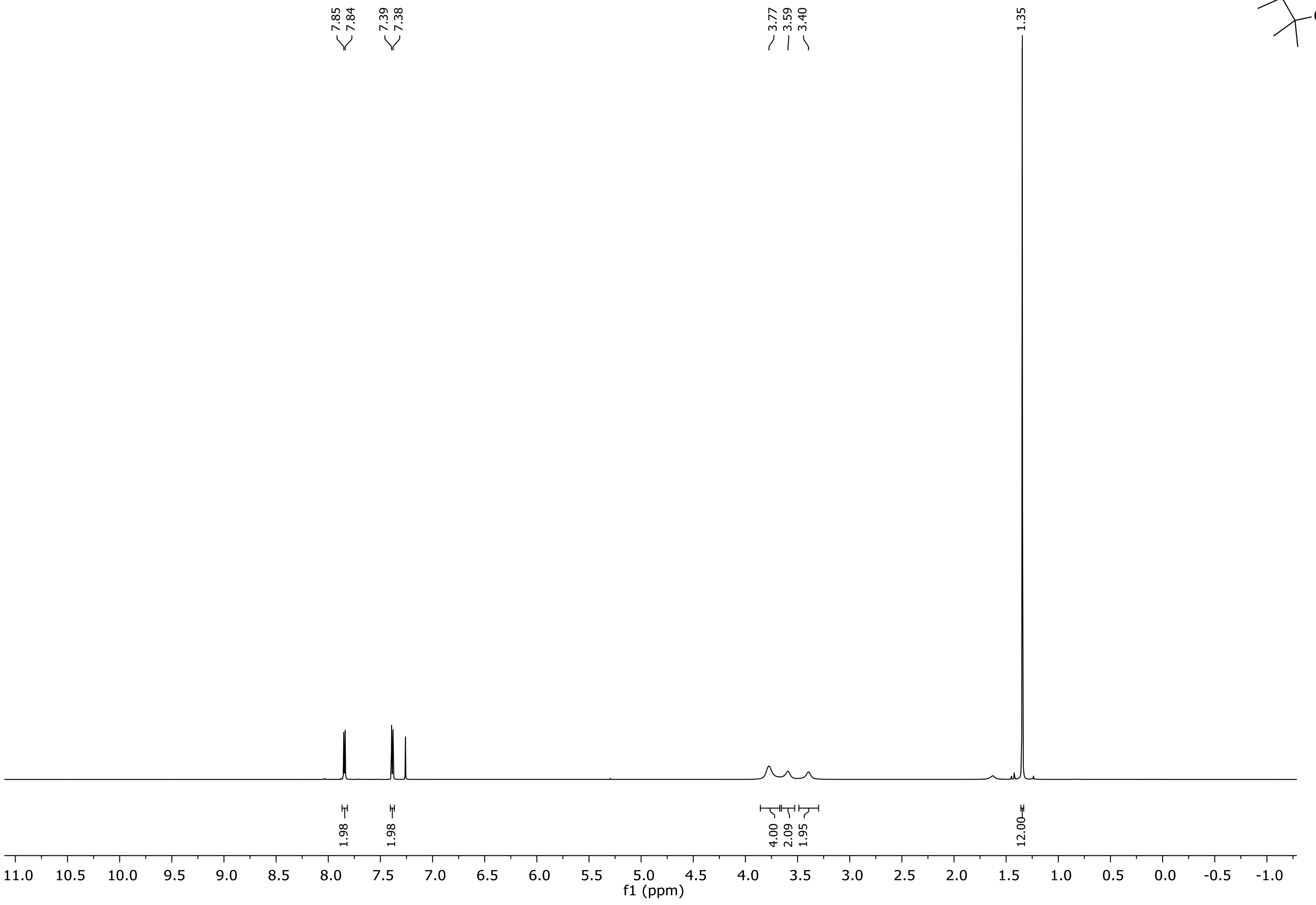

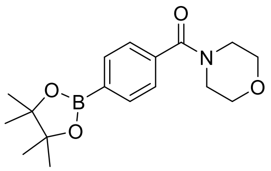

1t

^{13}C NMR

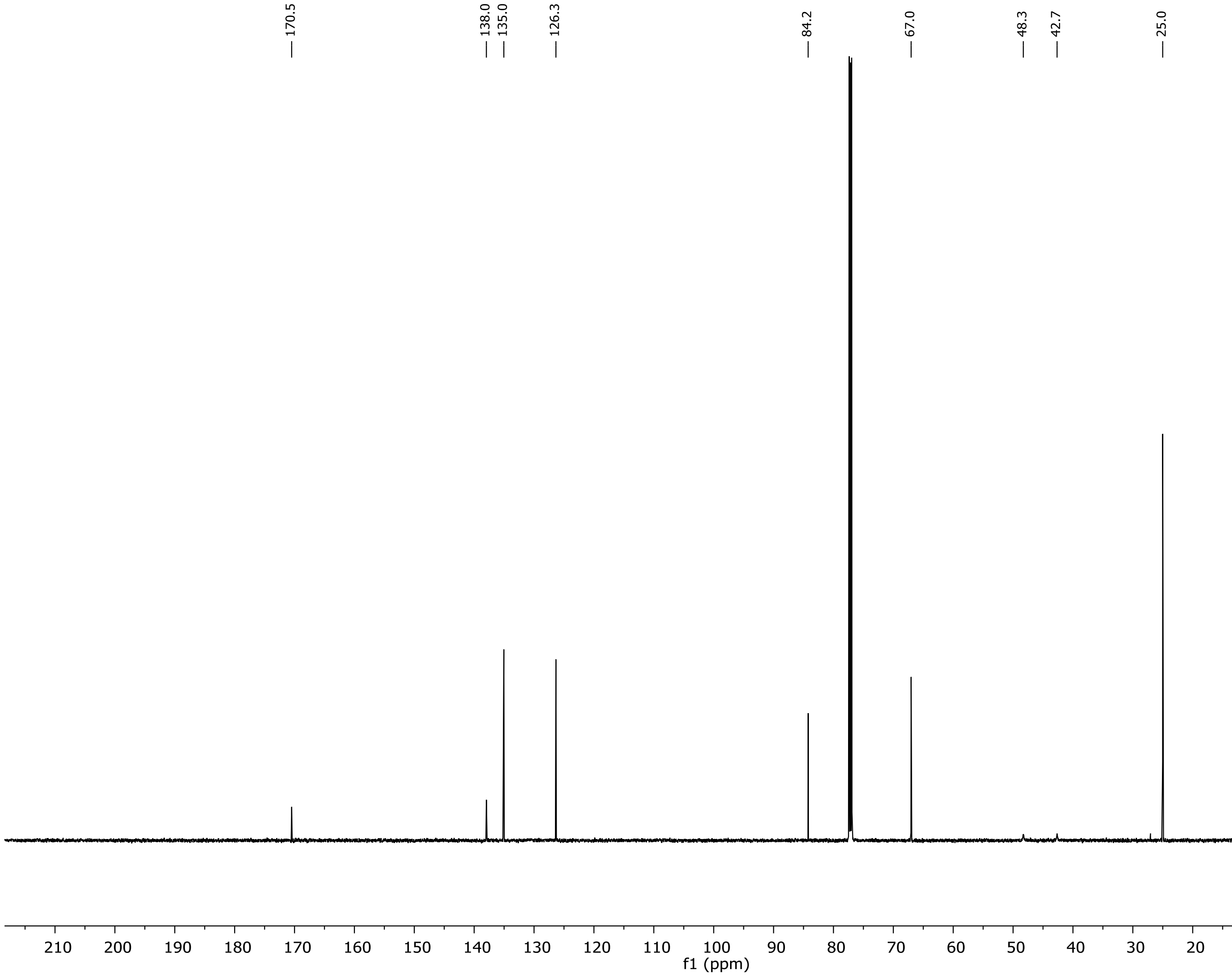

170.5
138.0
135.0
126.3
84.2
67.0
48.3
42.7
25.0
210 200 190 180 170 160 150 140 130 120 110 100 90 80 70 60 50 40 30 20 10 0 -10
f1 (ppm)

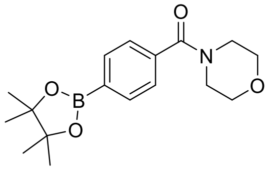

1t

^{11}B NMR

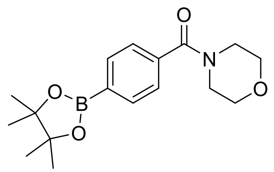

1t

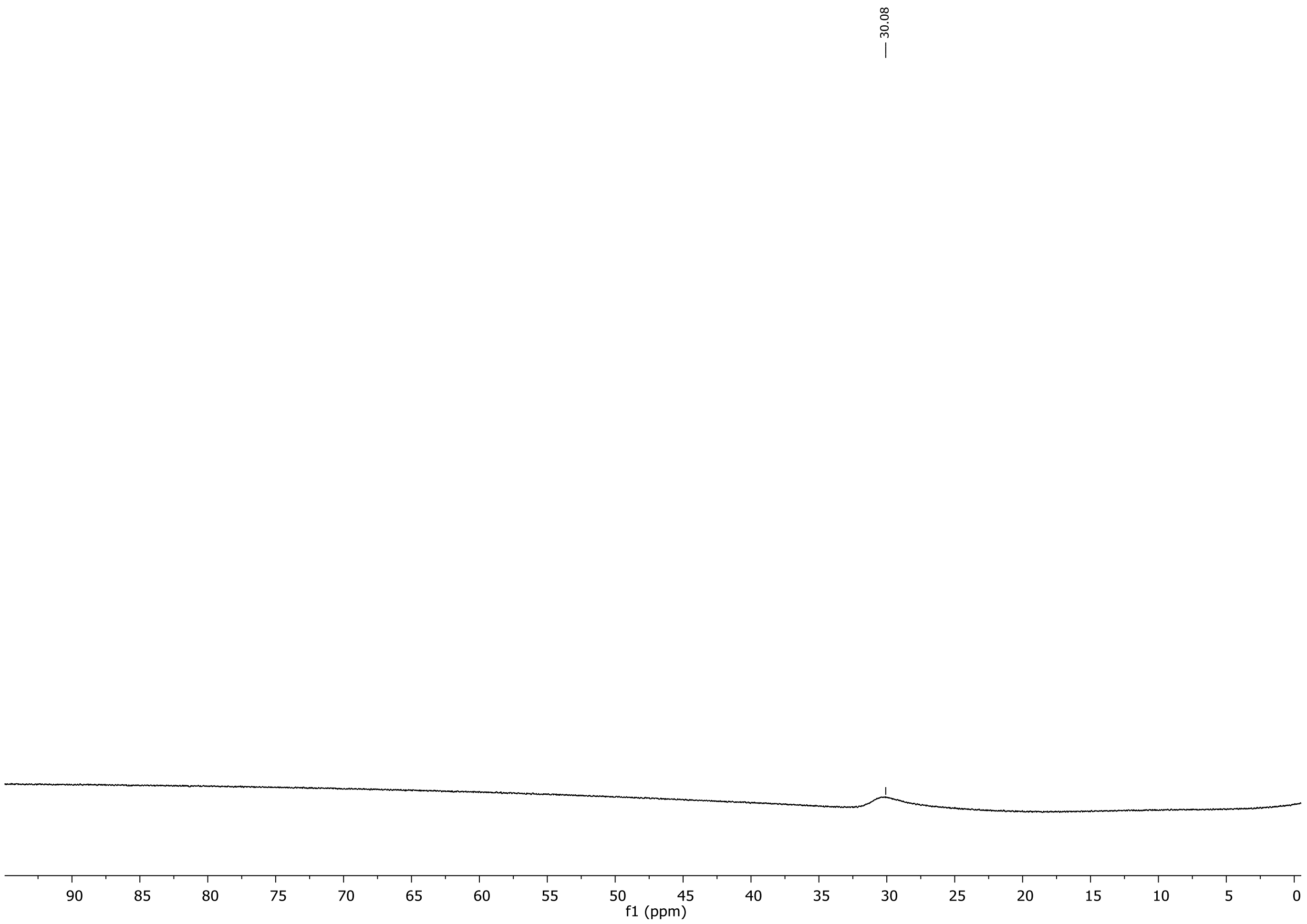

^1H, ^1H COSY

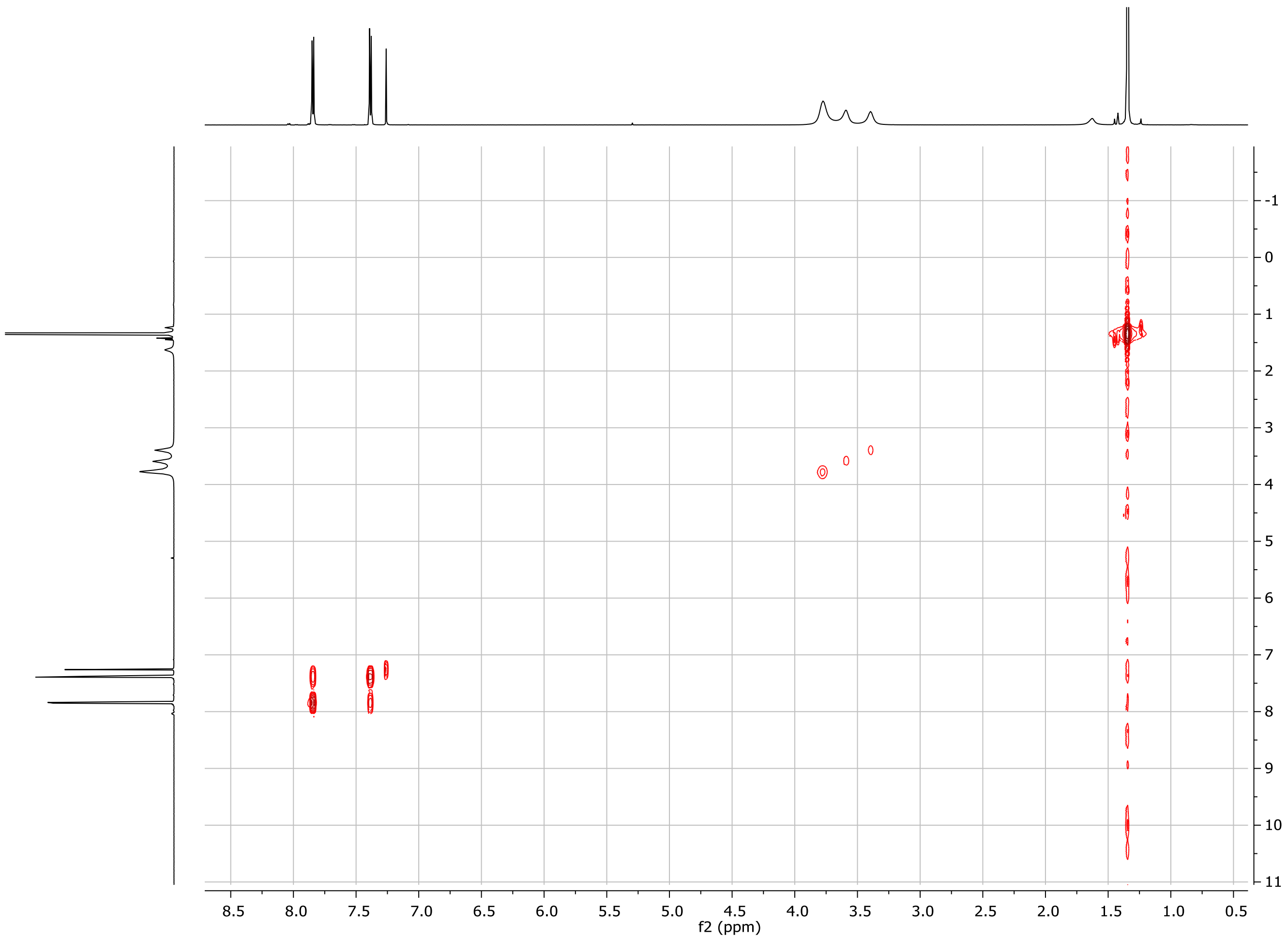

8.5
8.0
7.5
7.0
6.5
6.0
5.5
5.0
4.5
4.0
3.5
3.0
2.5
2.0
1.5
1.0
0.5
f2 (ppm)
-1
0
1
2
3
4
5
6
7
8
9
10
11
f1 (ppm)

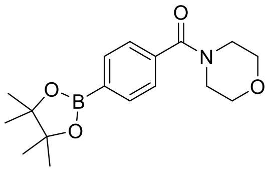

O
N
O
O
B
O

1t

^{1}H, ^{13}C HMBC

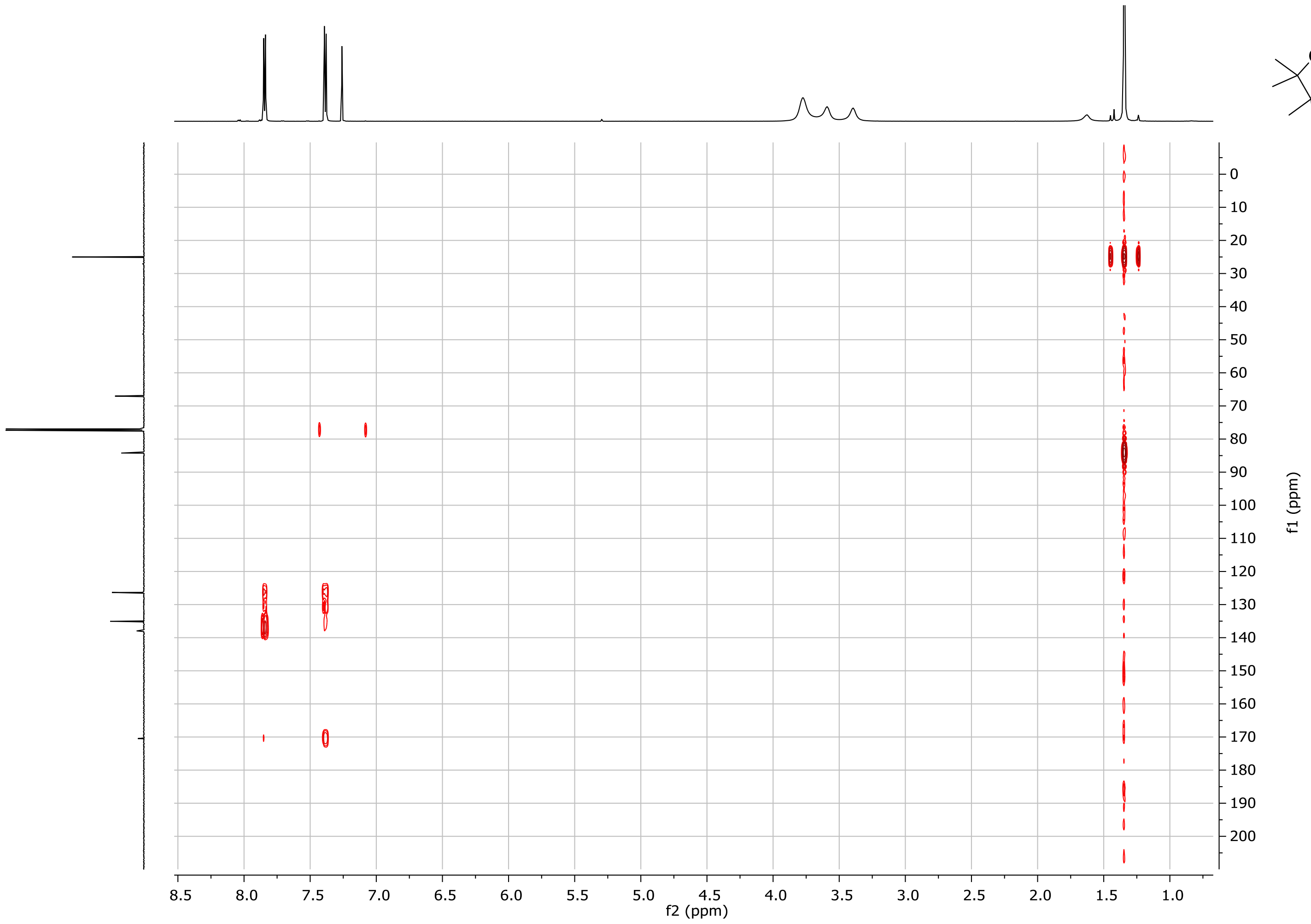

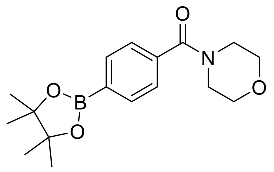

1t

^{1}H, ^{13}C HSQC

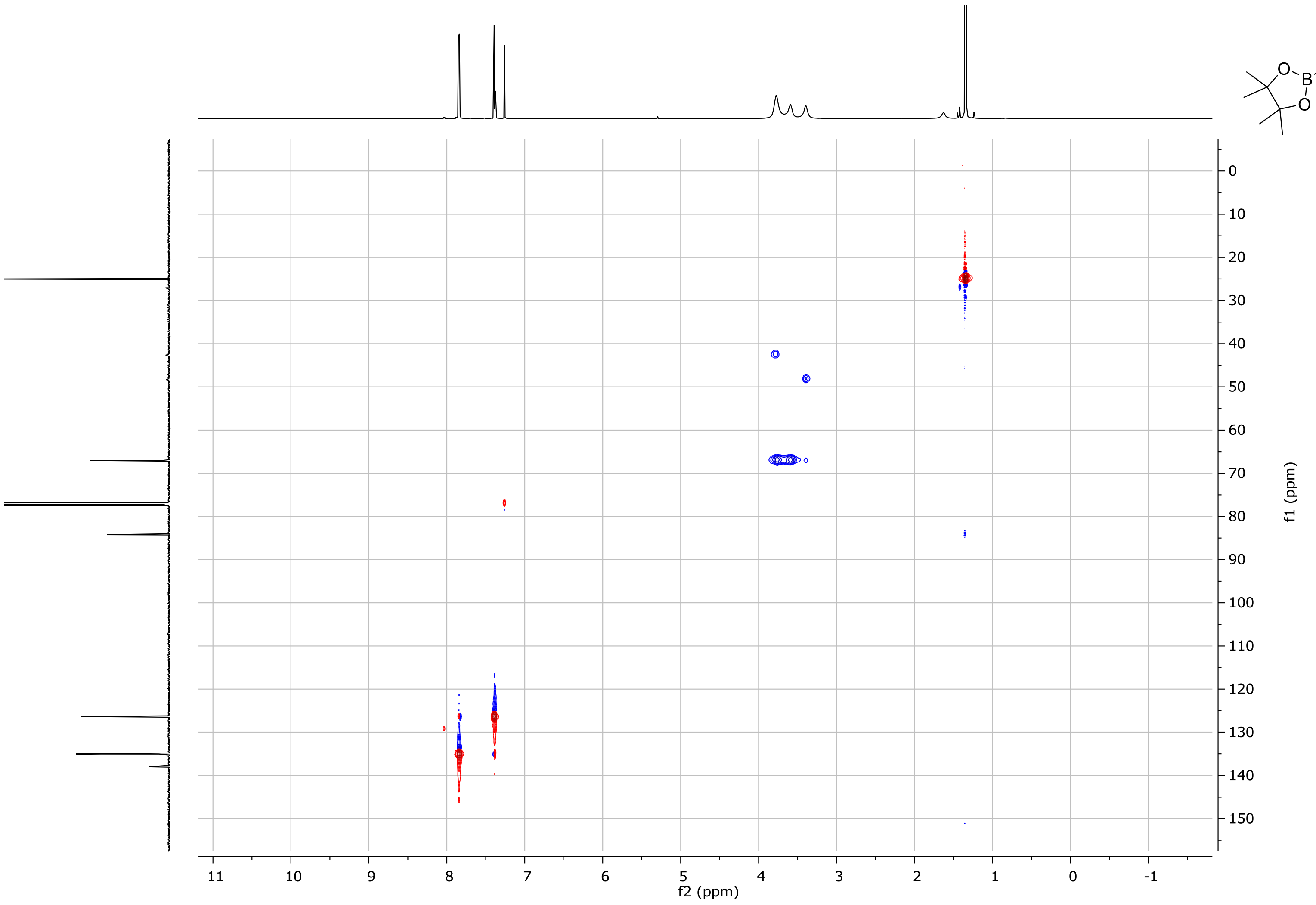

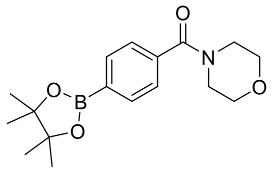

1t

HRMS

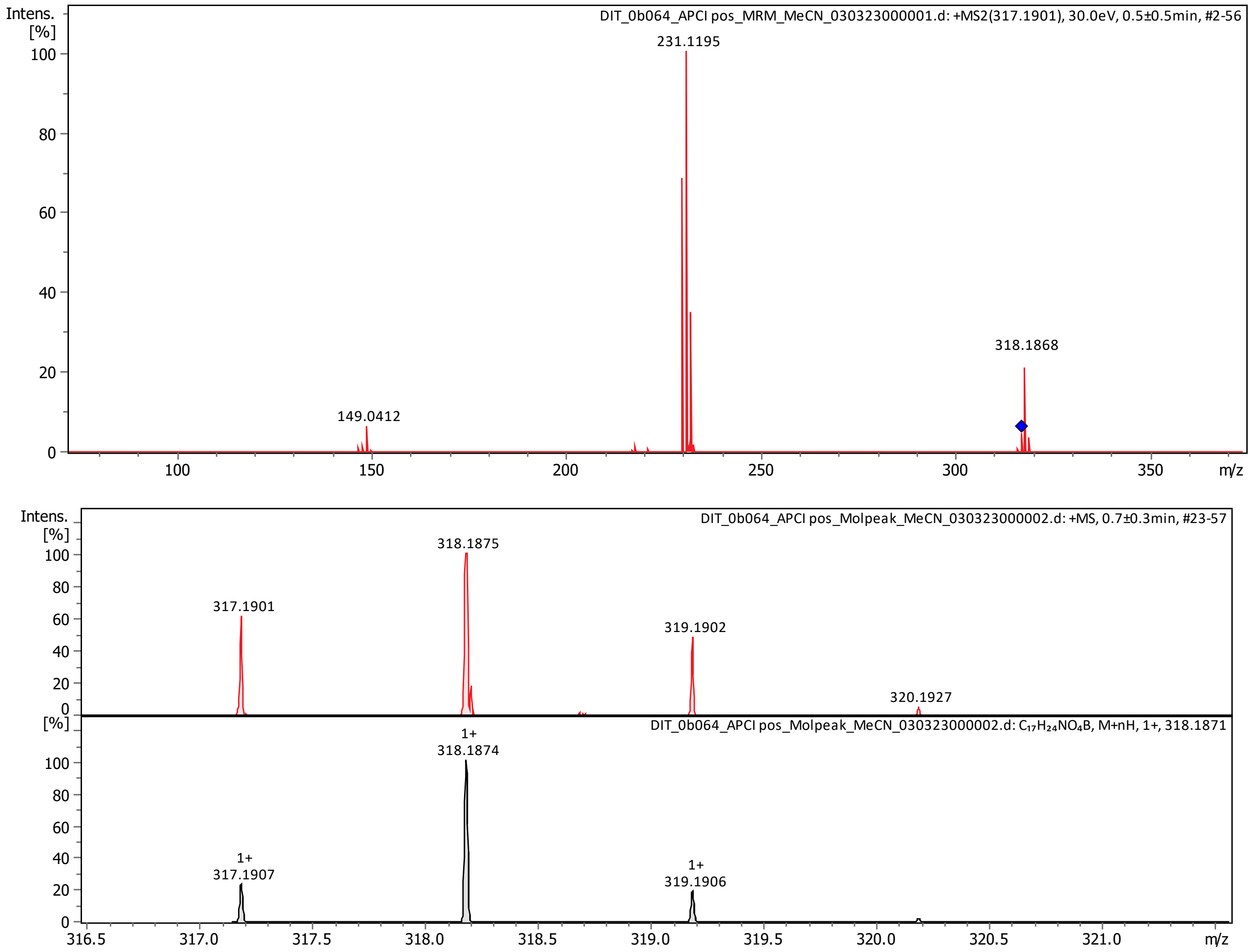

Intens. [%]
DIT_0b064_APCI pos_MRM_MeCN_030323000001.d: +MS2(317.1901), 30.0eV, 0.5±0.5min, #2-56
231.1195
318.1868
149.0412
m/z
DIT_0b064_APCI pos_Molpeak_MeCN_030323000002.d: +MS, 0.7±0.3min, #23-57
317.1901
318.1875
319.1902
320.1927
DIT_0b064_APCI pos_Molpeak_MeCN_030323000002.d: C17H24NO4B, M+nH, 1+, 318.1871
1+ 317.1907
1+ 318.1874
1+ 319.1906

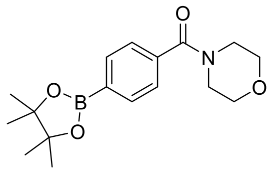

1t

18 Furan-2-yl(morpholino)methanone (1u)

^{1}H NMR

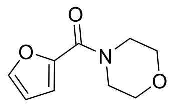

1u

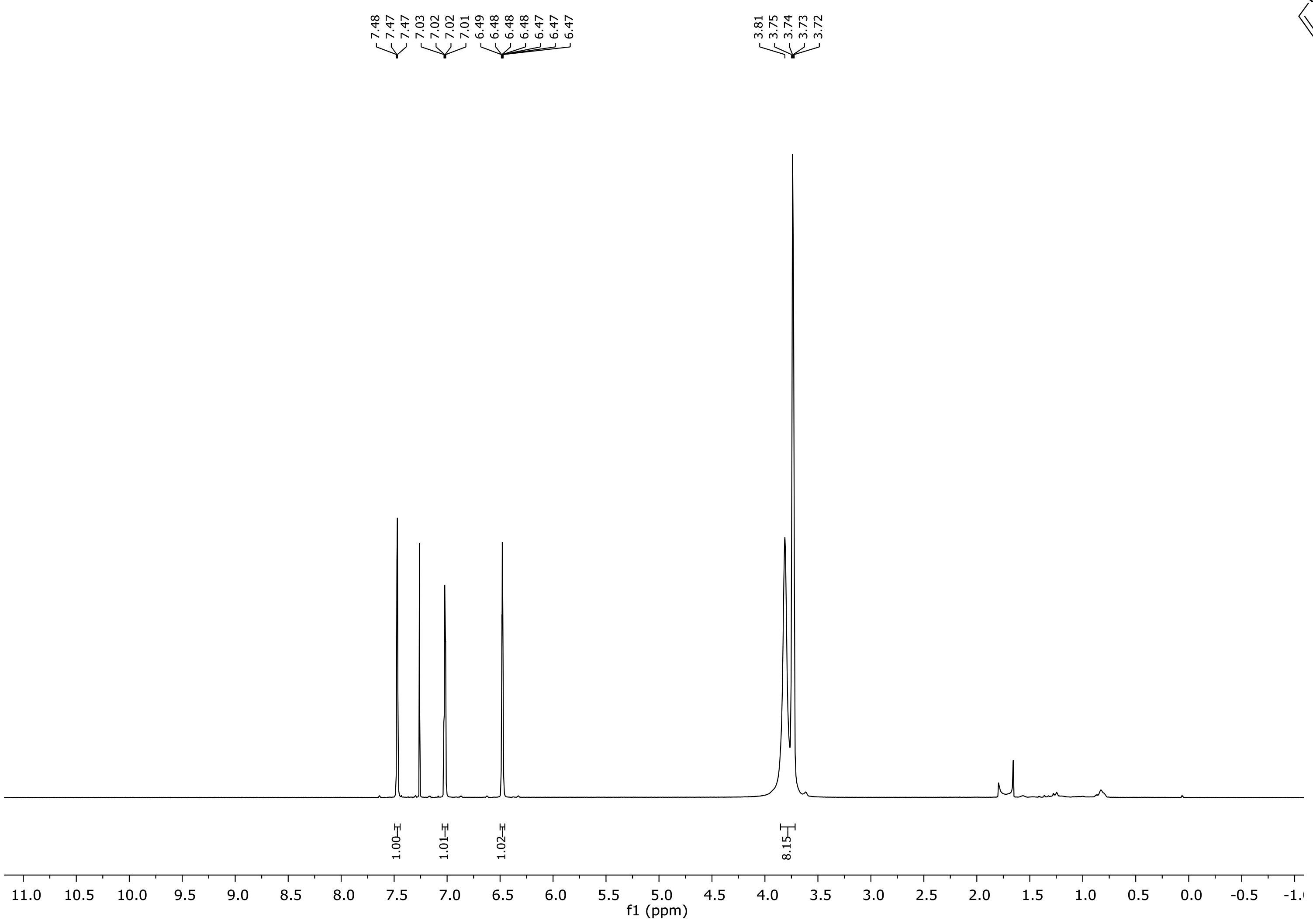

^{13}C NMR

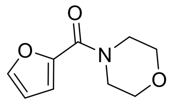

1u

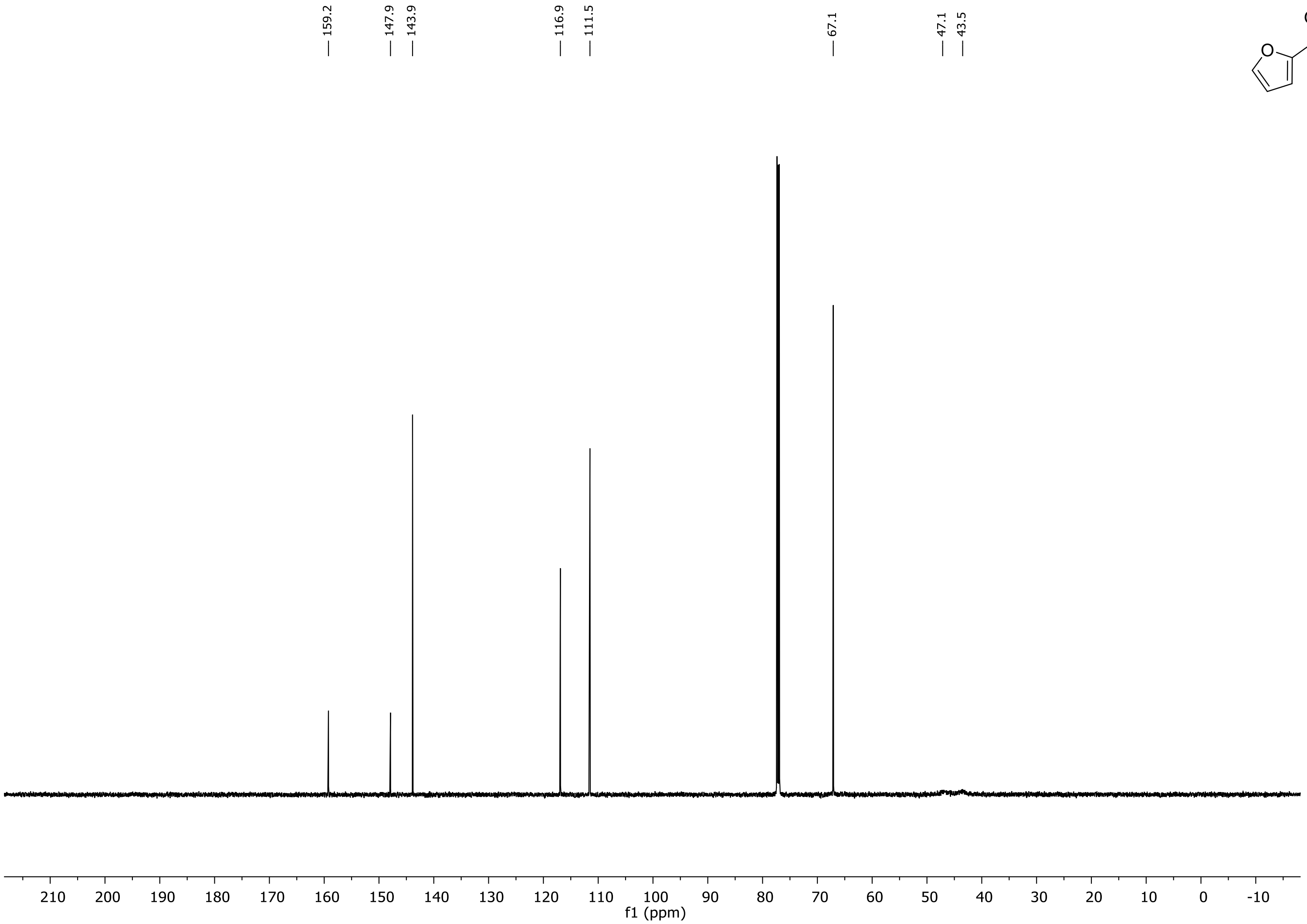
159.2
147.9
143.9
116.9
111.5
67.1
47.1
43.5
210
200
190
180
170
160
150
140
130
120
110
100
90
80
70
60
50
40
30
20
10
0
-10
f1 (ppm)

^{1}H, ^{1}H COSY

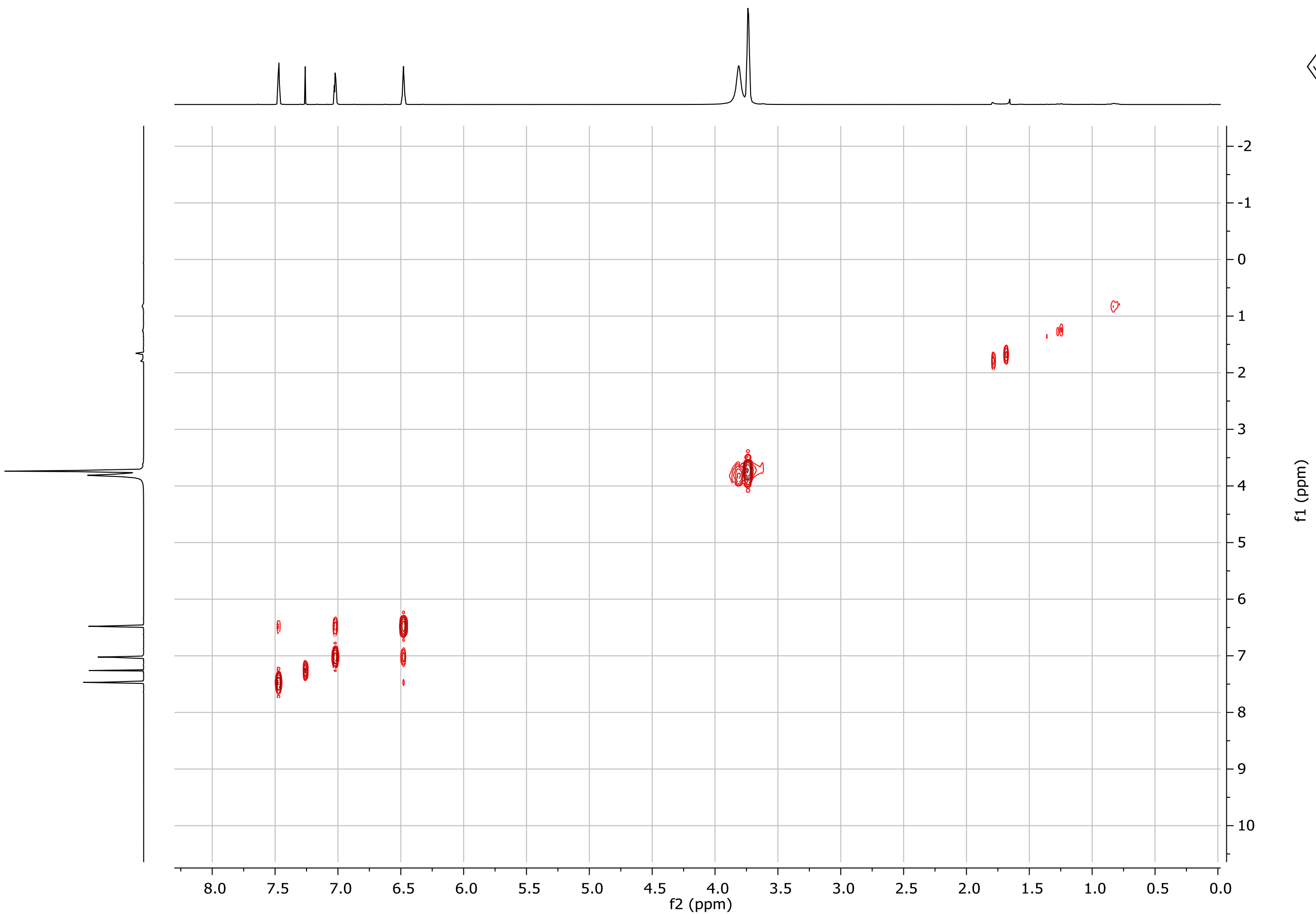

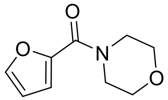

1u

^{1}H, ^{13}C HMBC

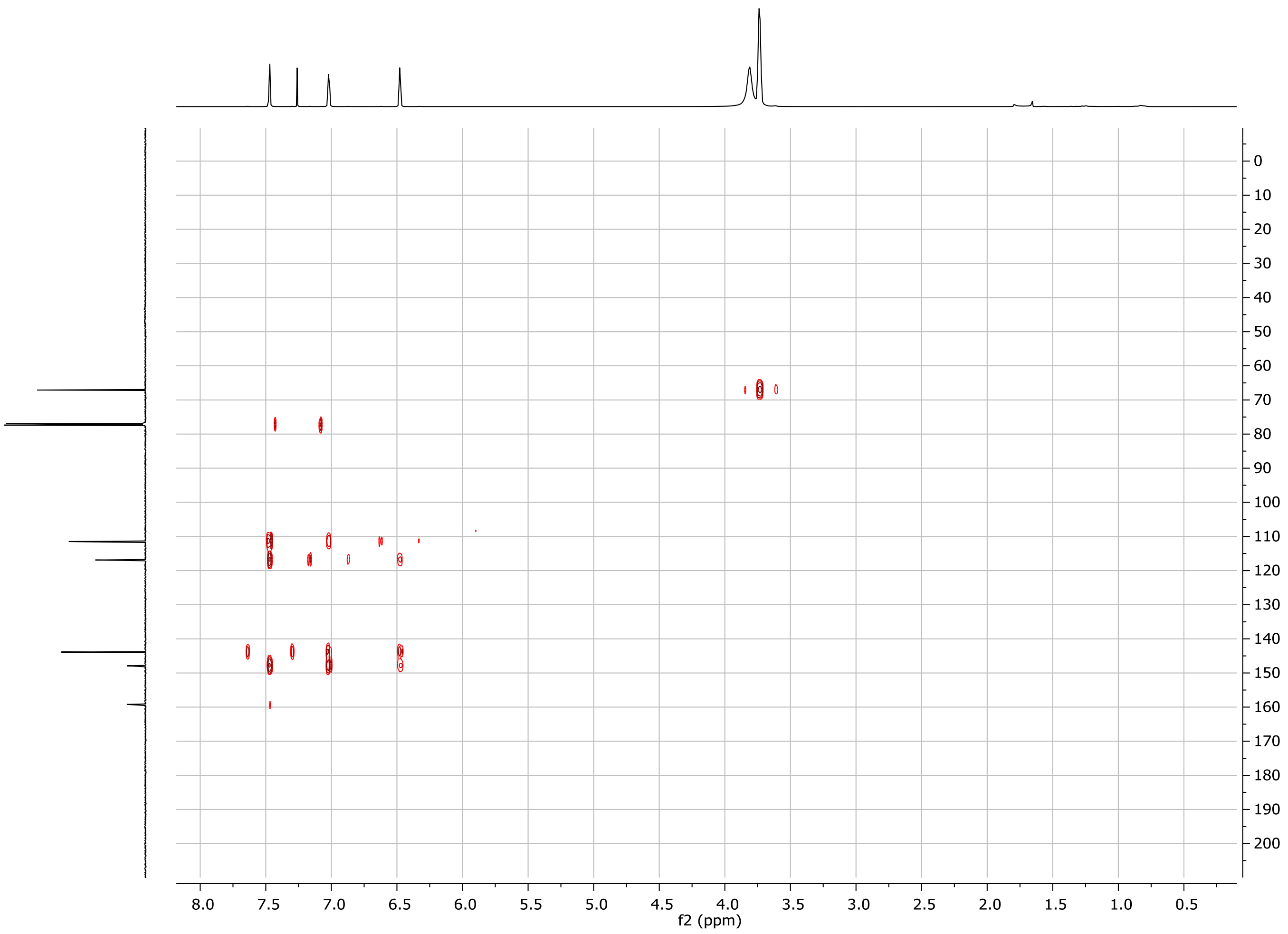

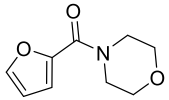

1u

^{1}H, ^{13}C HSQC

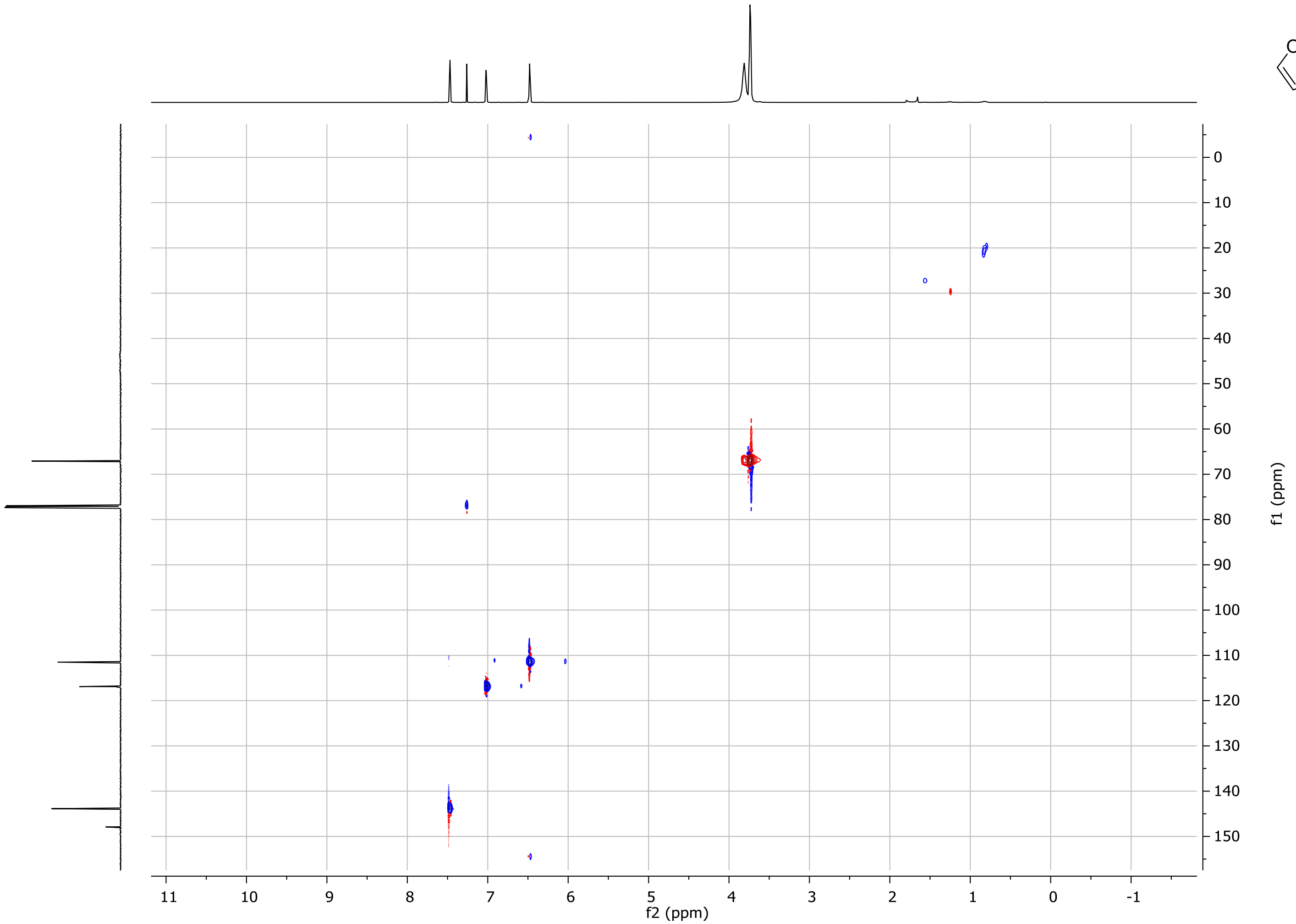

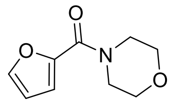

1u

HRMS

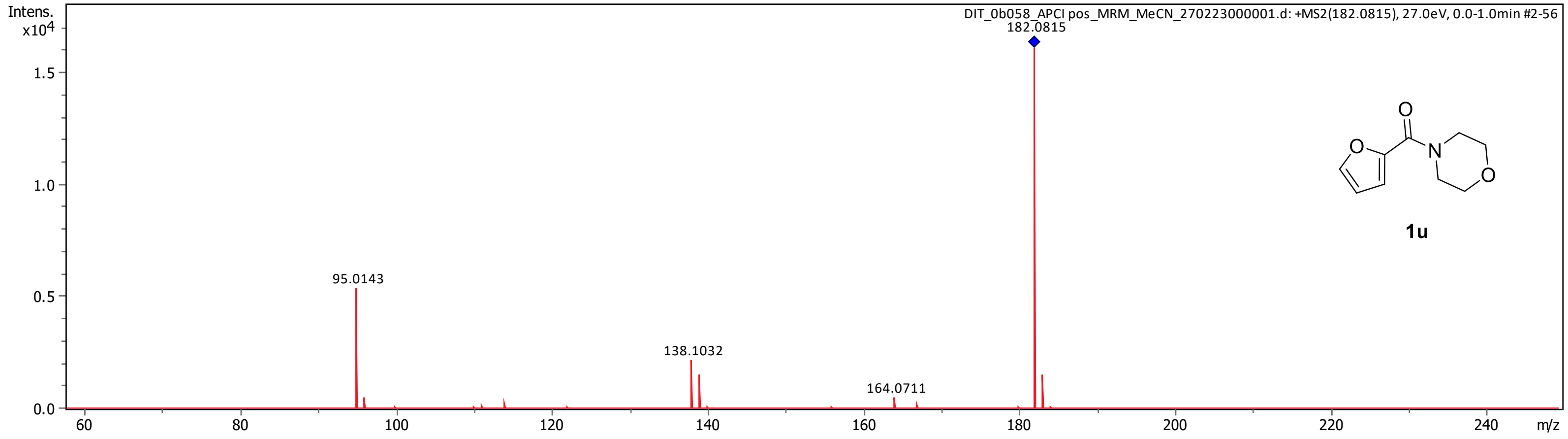

Intens.
x10^4
DIT_0b058_APCI pos_MRM_MeCN_270223000001.d: +MS2(182.0815), 27.0eV, 0.0-1.0min #2-56
182.0815
95.0143
138.1032
164.0711
m/z
1u

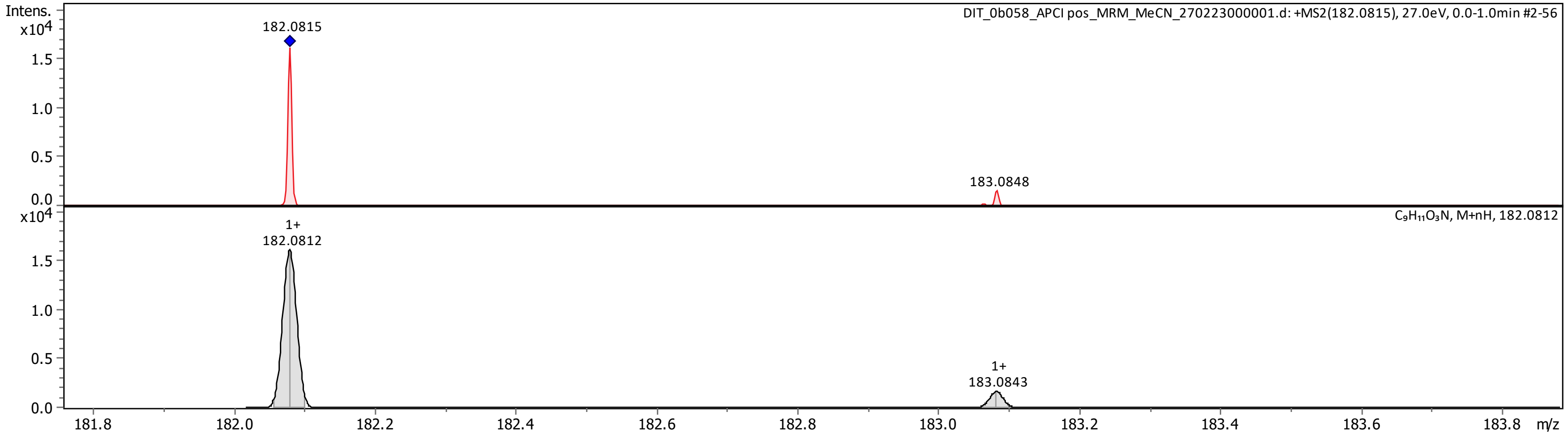

Intens.
x10^4
DIT_0b058_APCI pos_MRM_MeCN_270223000001.d: +MS2(182.0815), 27.0eV, 0.0-1.0min #2-56
182.0815
183.0848
$C_9H_{11}O_3N$, M+nH, 182.0812
1+
182.0812
1+
183.0843
m/z

19 Morpholino(thiophen-2-yl)methanone (1v)

^{1}H NMR

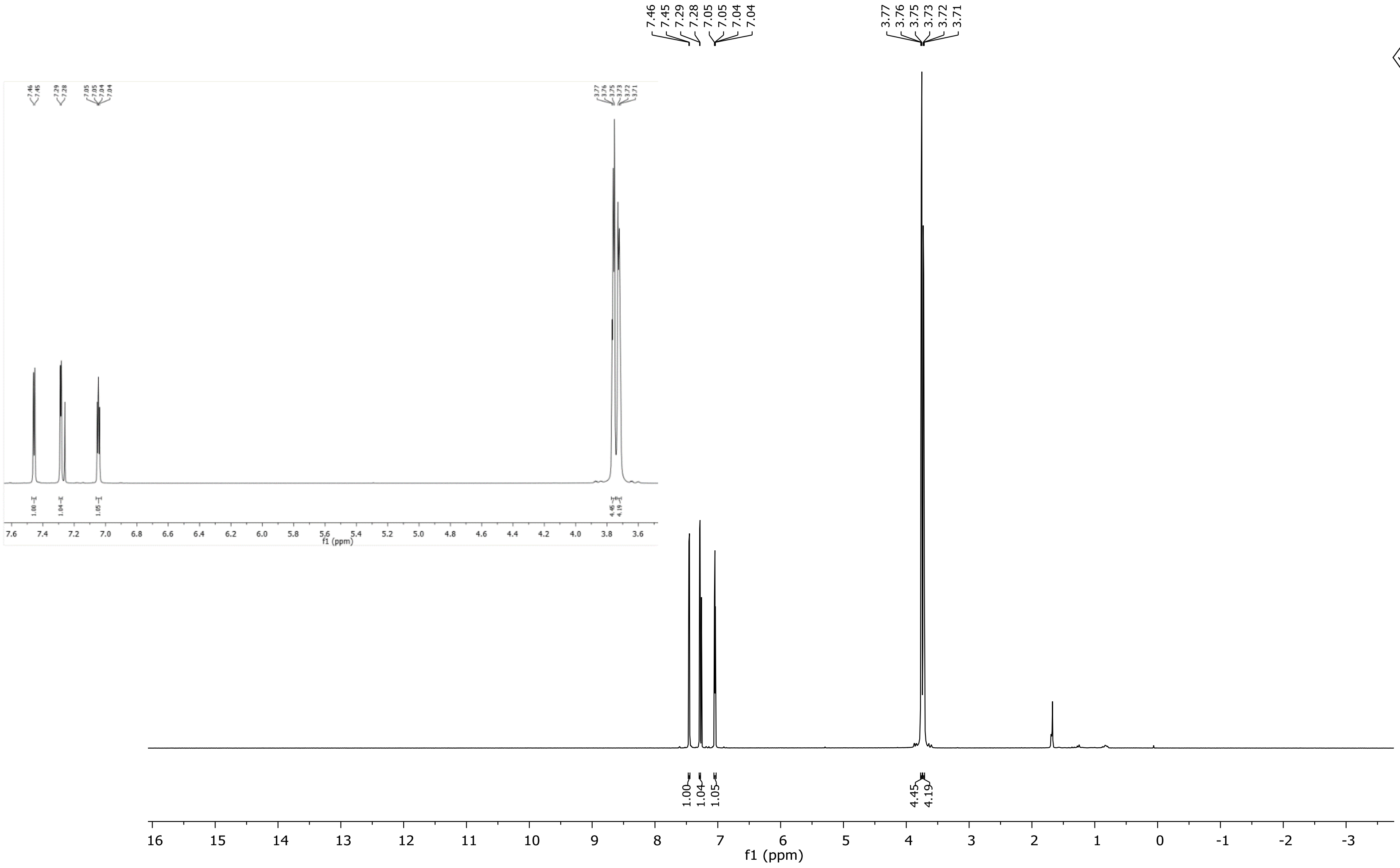

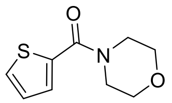

1v

^{13}C NMR

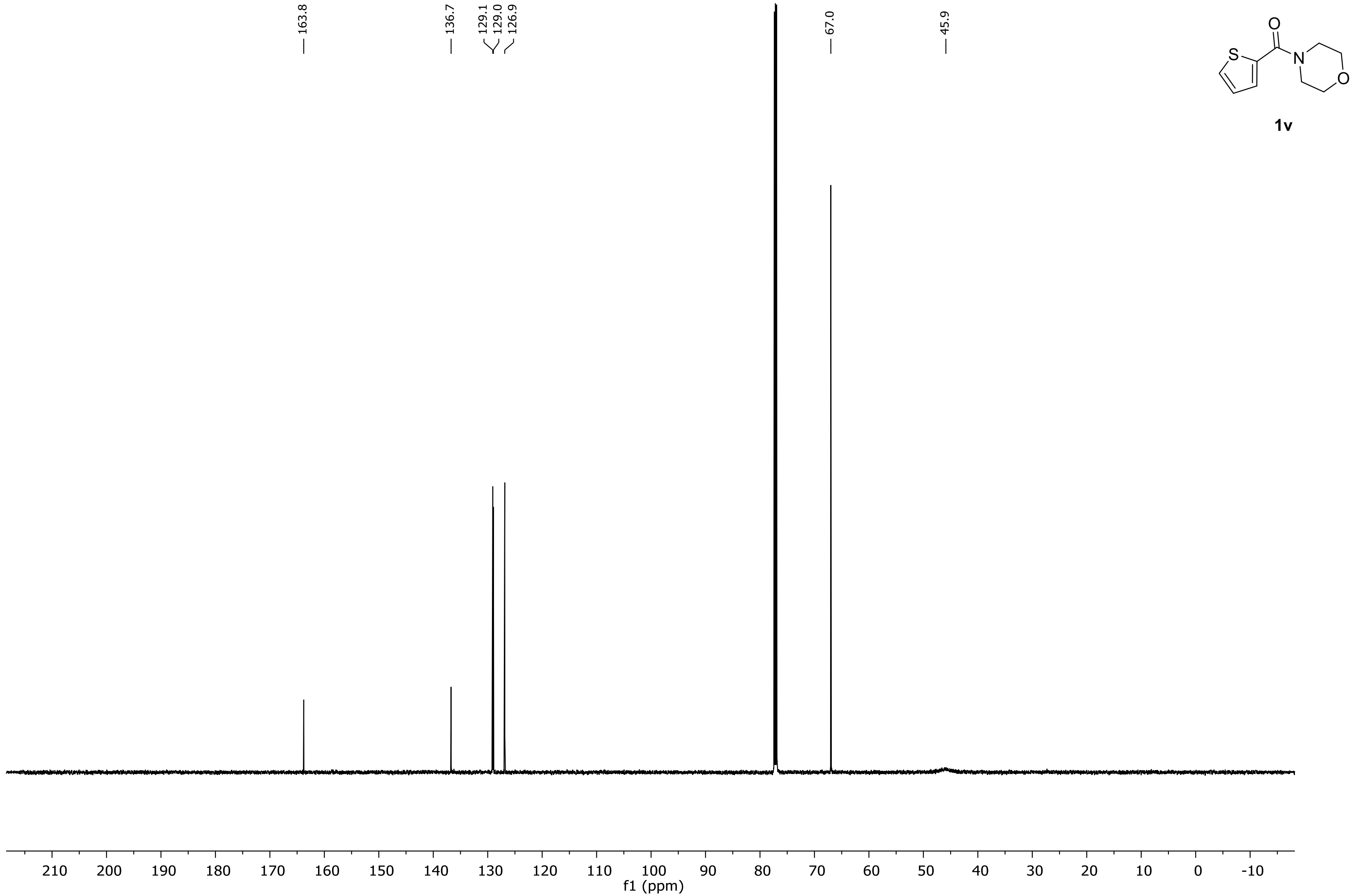

163.8
136.7
129.1
129.0
126.9
67.0
45.9
210
200
190
180
170
160
150
140
130
120
110
100
90
80
70
60
50
40
30
20
10
0
-10
f1 (ppm)
O
S
N
O

1v

^{1}H, ^{1}H COSY

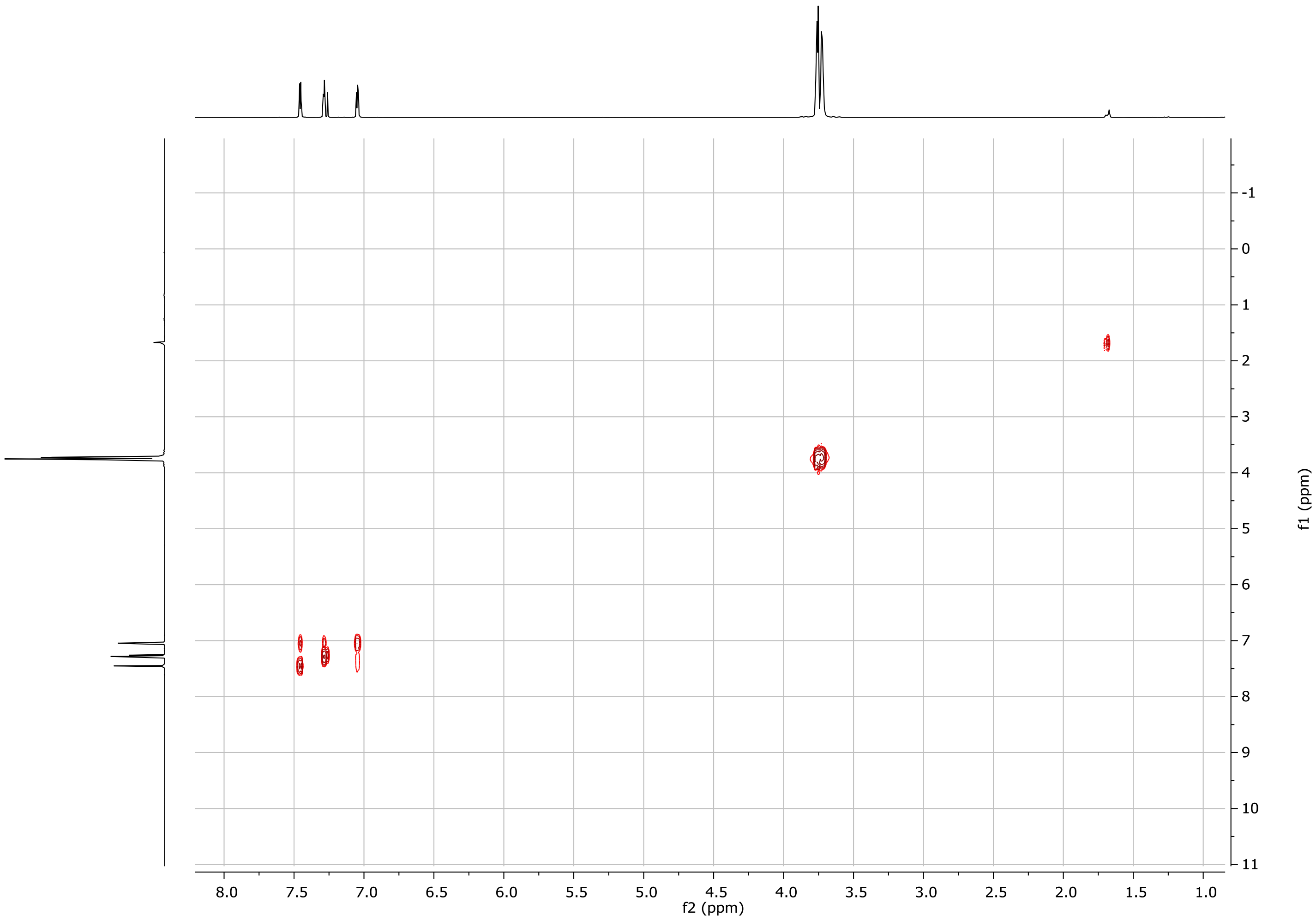

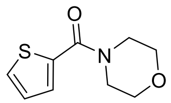

1v

^1H, ^{13}C HMBC

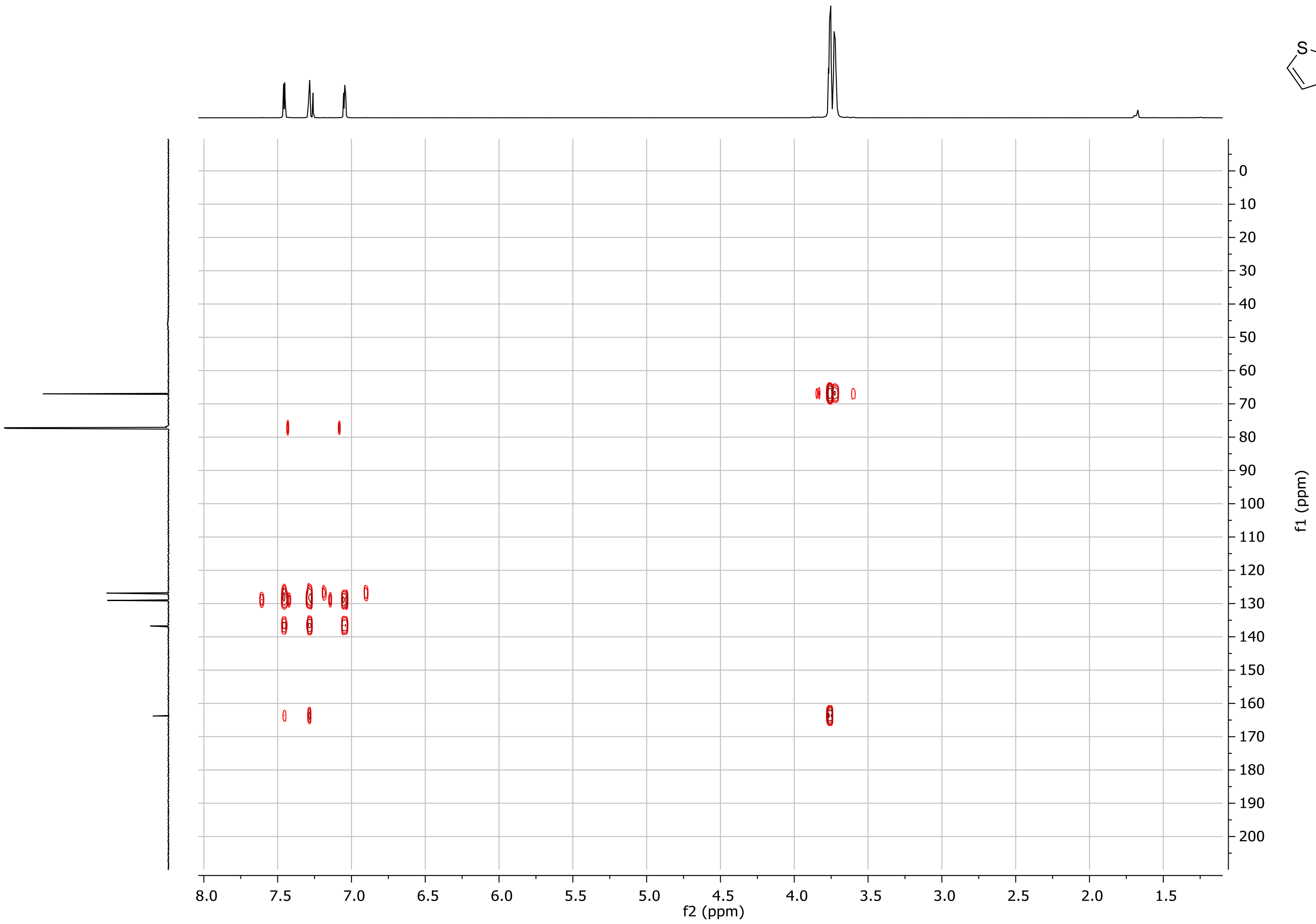

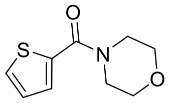

1v

^{1}H, ^{13}C HSQC

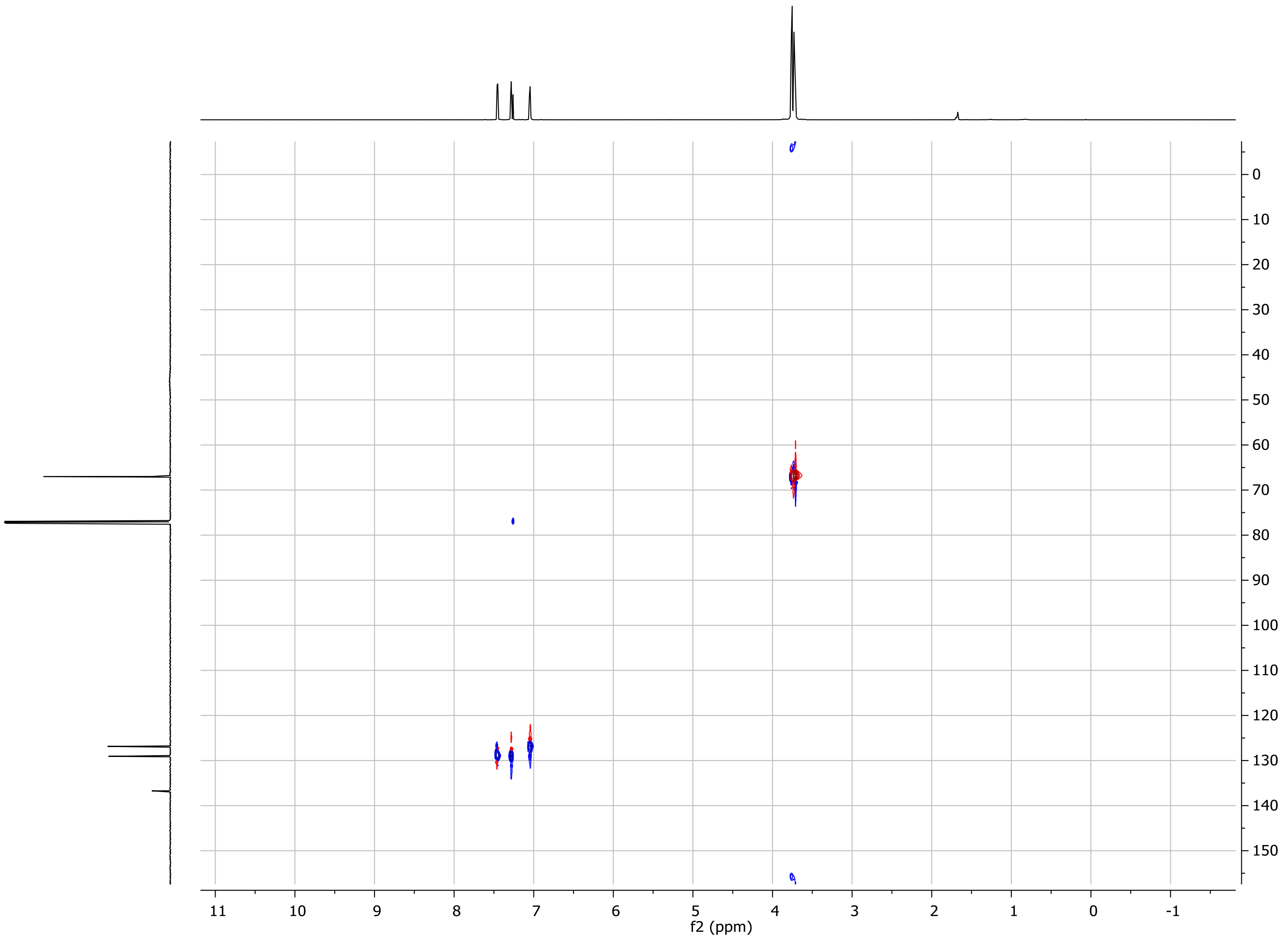

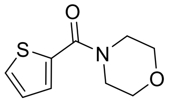

1v

HRMS

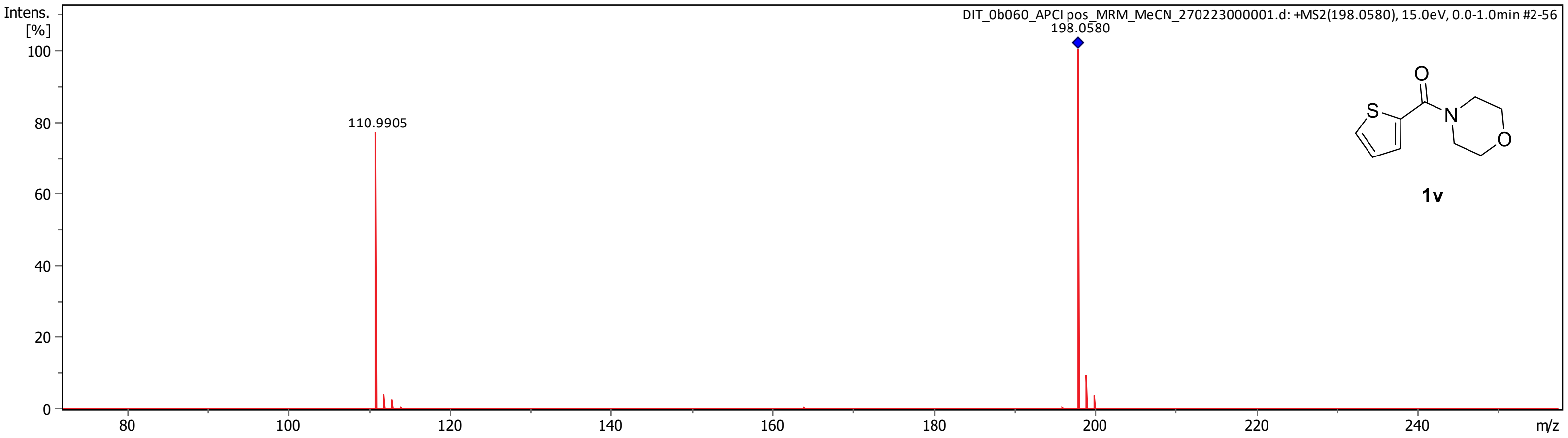
Intens.
[%]
DIT_0b060_APCI pos_MRM_MeCN_270223000001.d: +MS2(198.0580), 15.0eV, 0.0-1.0min #2-56
198.0580
110.9905
S
O
N
O
m/z

1v

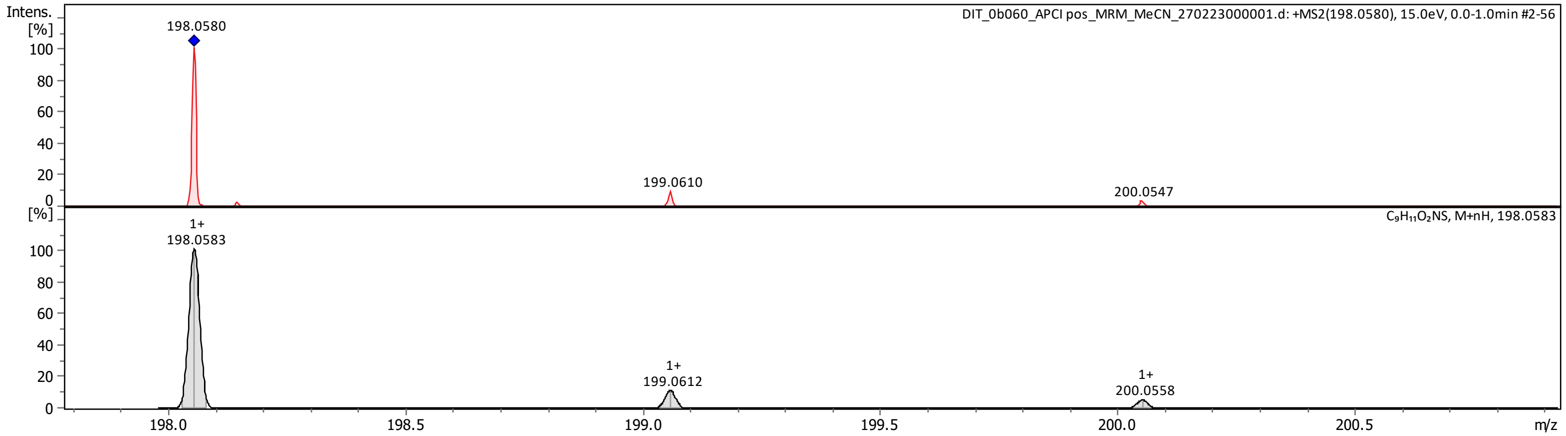
Intens.
[%]
DIT_0b060_APCI pos_MRM_MeCN_270223000001.d: +MS2(198.0580), 15.0eV, 0.0-1.0min #2-56
198.0580
199.0610
200.0547
C9H11O2NS, M+nH, 198.0583
1+
198.0583
1+
199.0612
1+
200.0558
m/z

20 5-((Benzyloxy)methyl)furan-2-carboxylic acid (S8)

^{1}H NMR

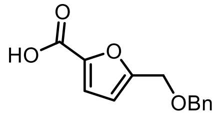

S8

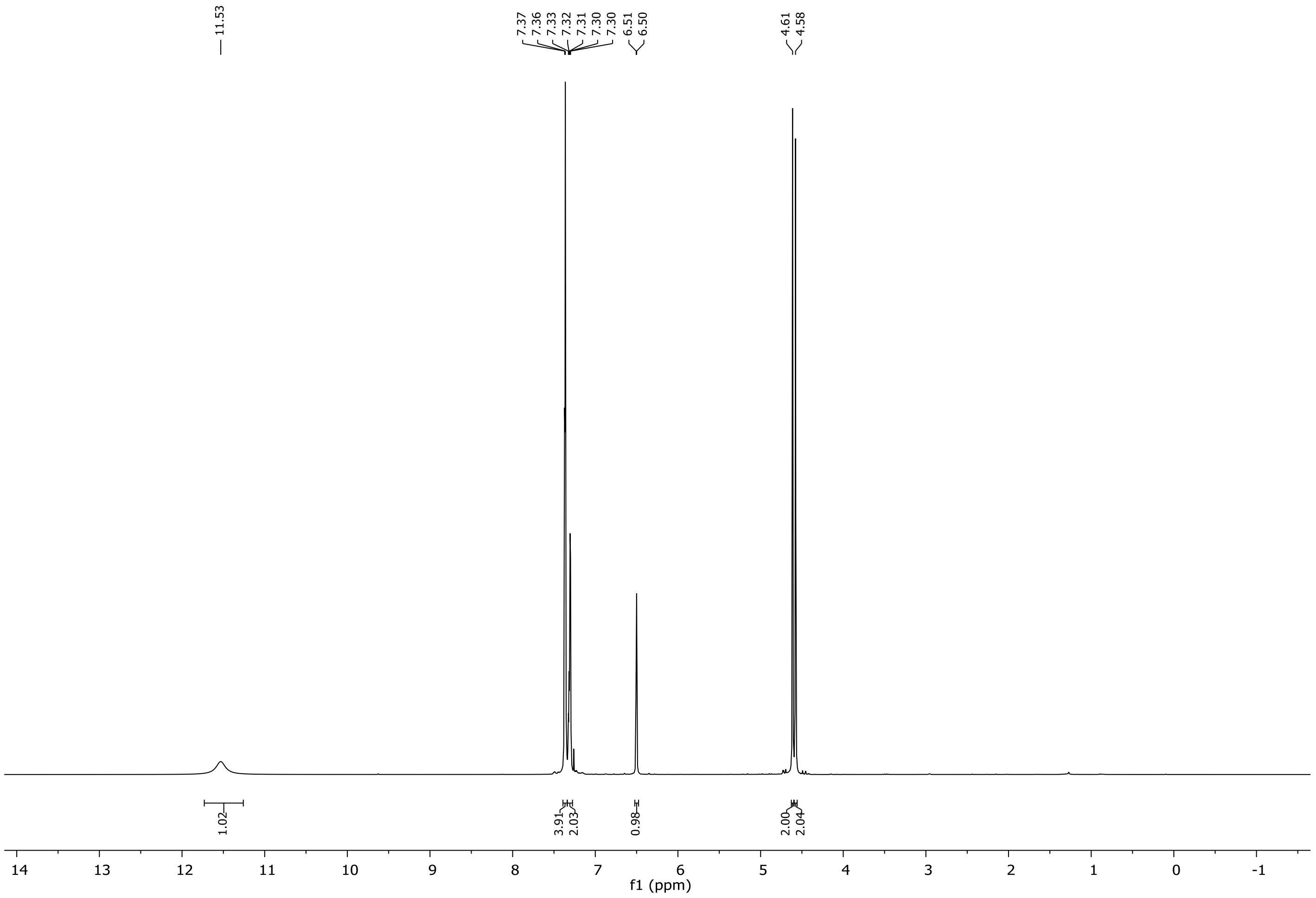

^{13}C NMR

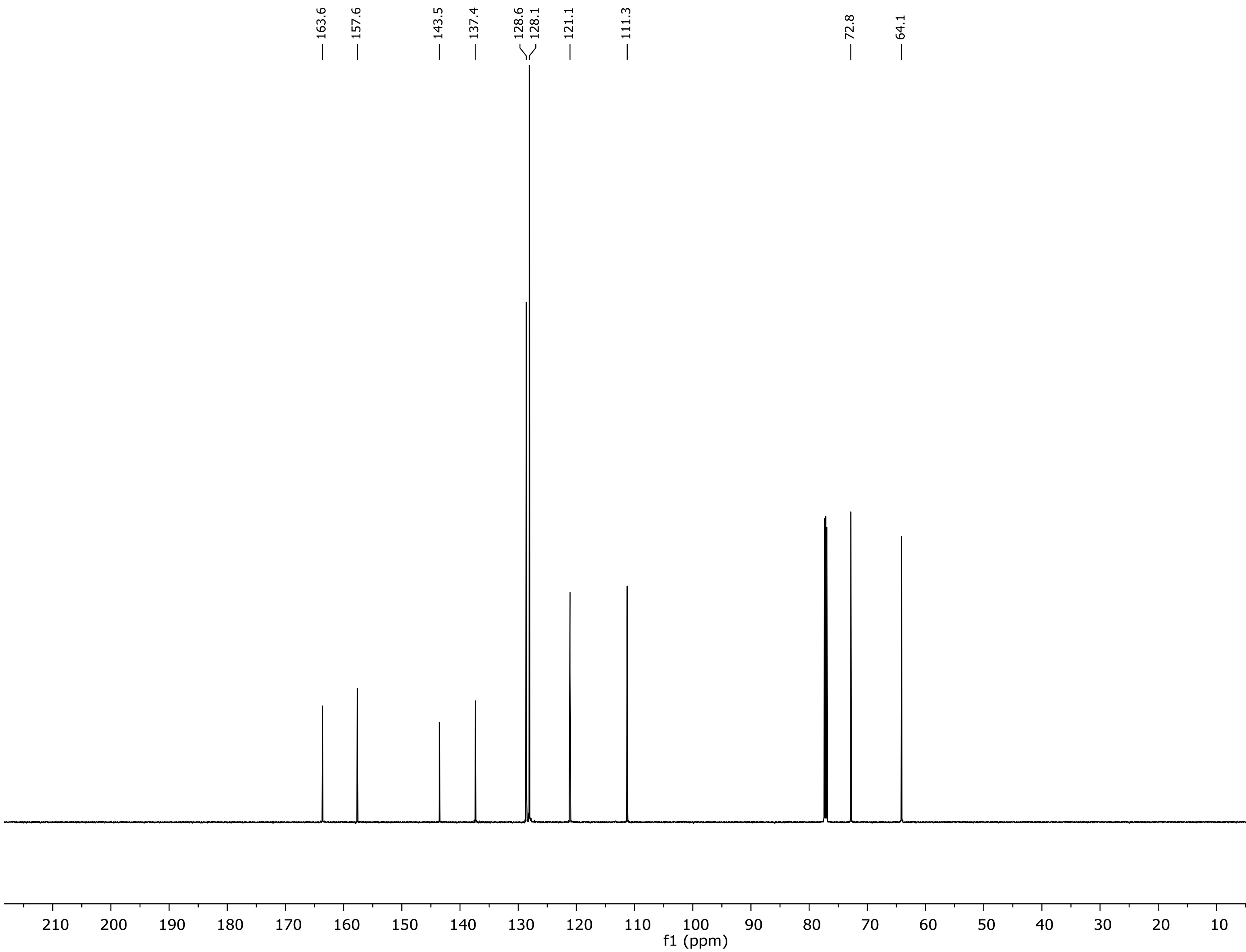
163.6
157.6
143.5
137.4
128.6
128.1
121.1
111.3
72.8
64.1
210 200 190 180 170 160 150 140 130 120 110 100 90 80 70 60 50 40 30 20 10 0 -10
f1 (ppm)

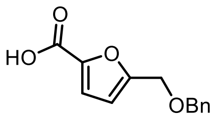
O
HO
O
OBn

S8

^{1}H, ^{1}H COSY

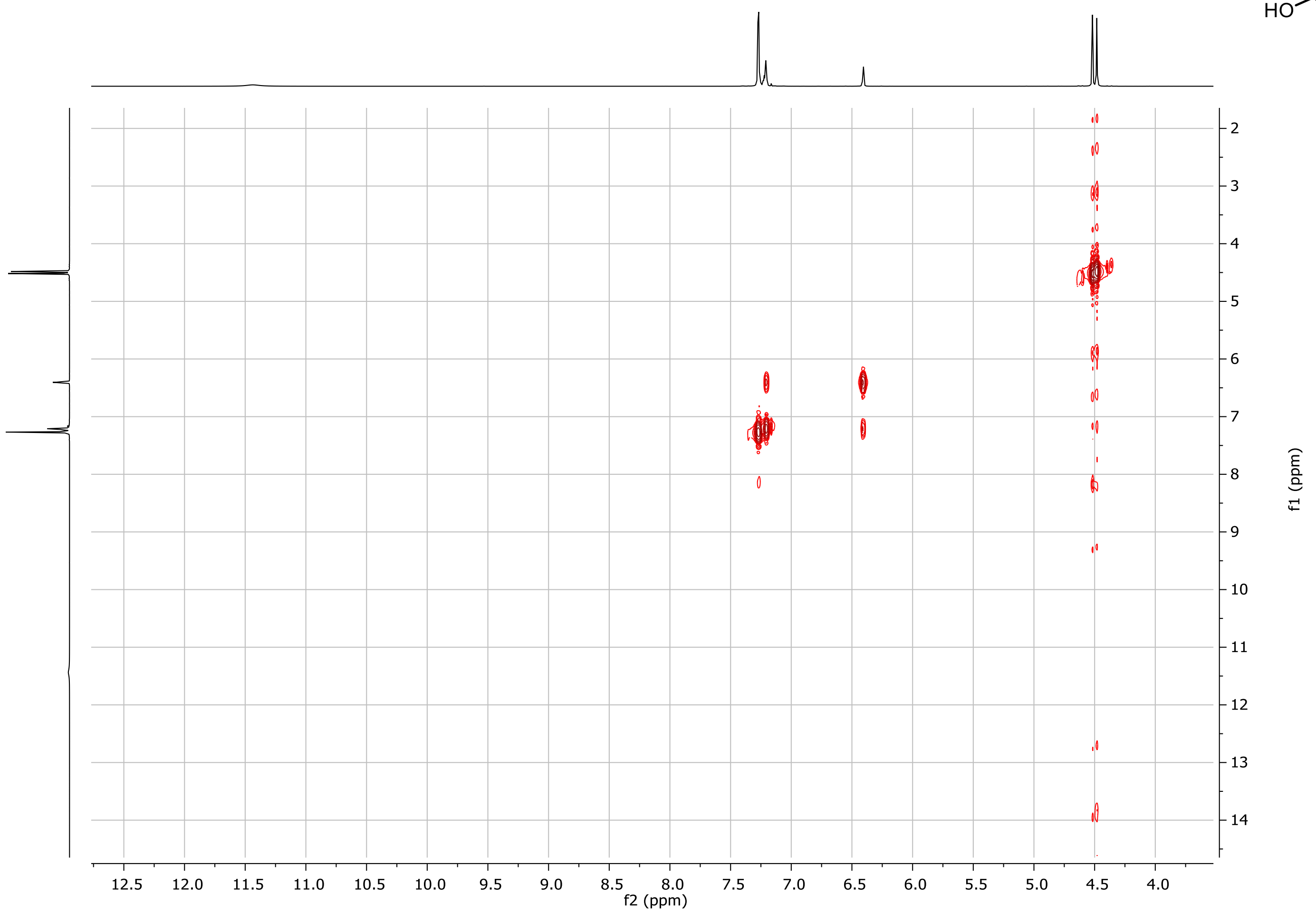

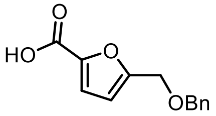

S8

^{1}H, ^{13}C HMBC

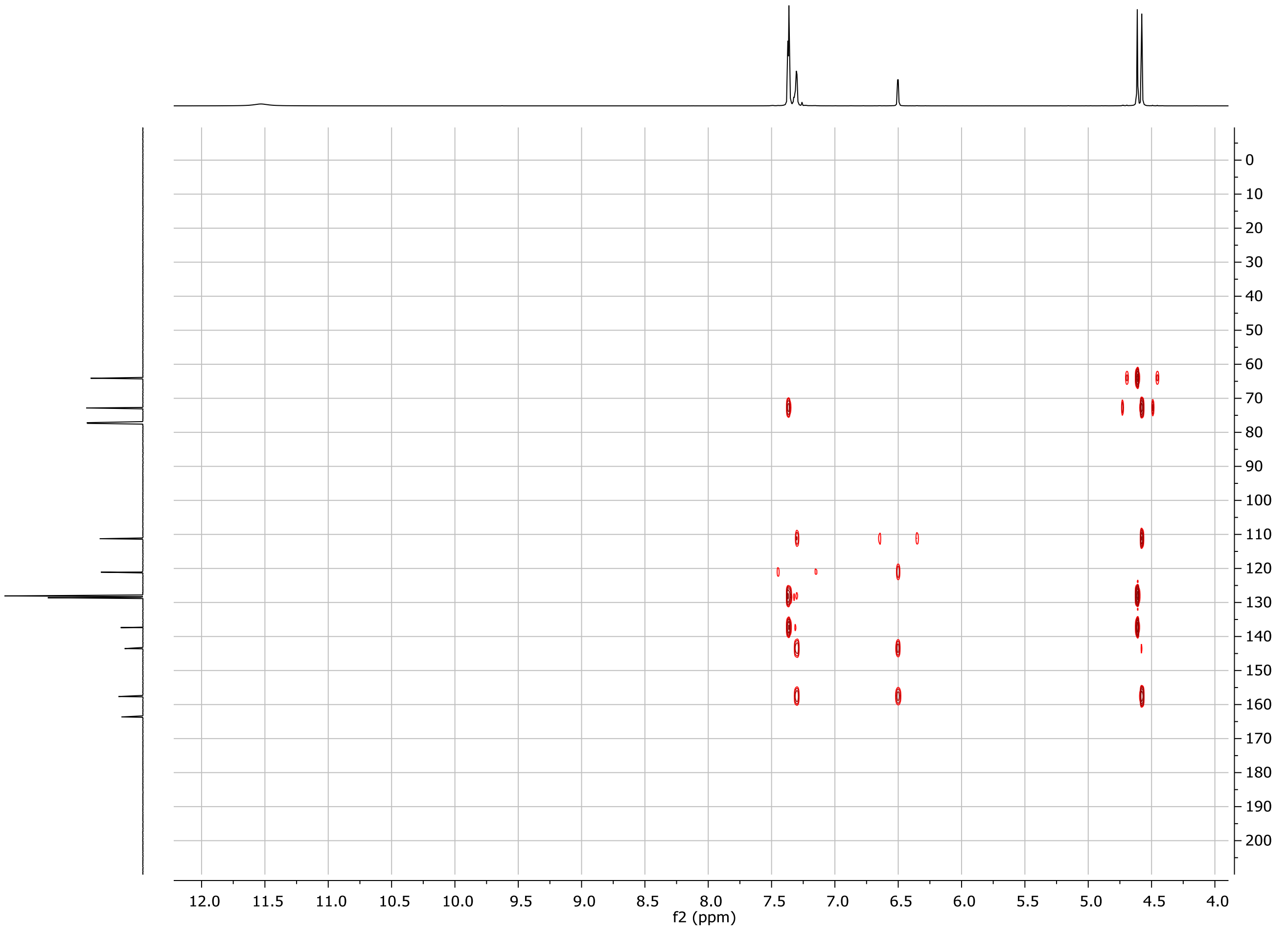

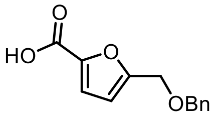

S8

^{1}H, ^{13}C HSQC

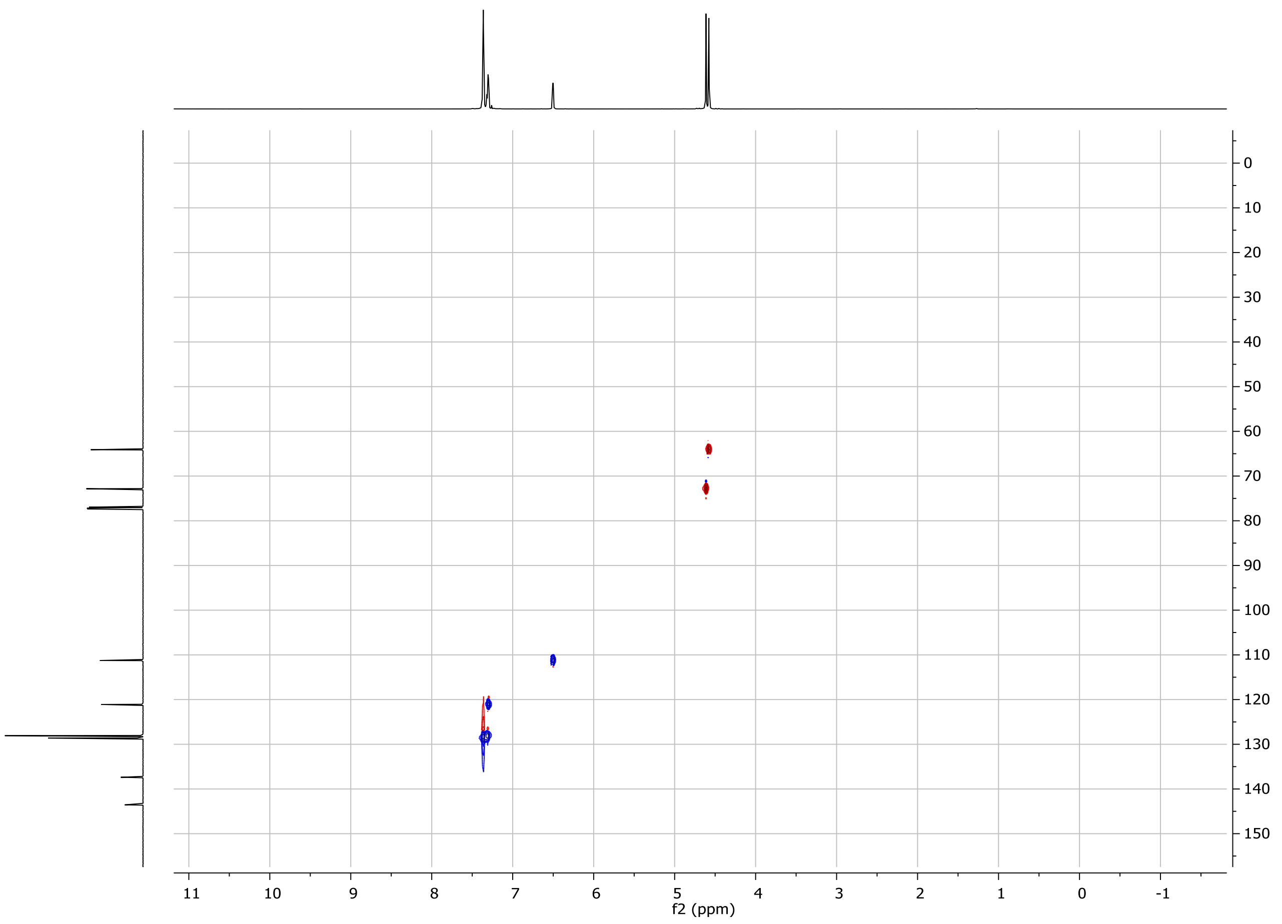

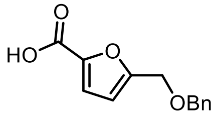

S8

HRMS

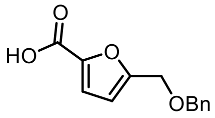

HO
O
O
OBn

S8

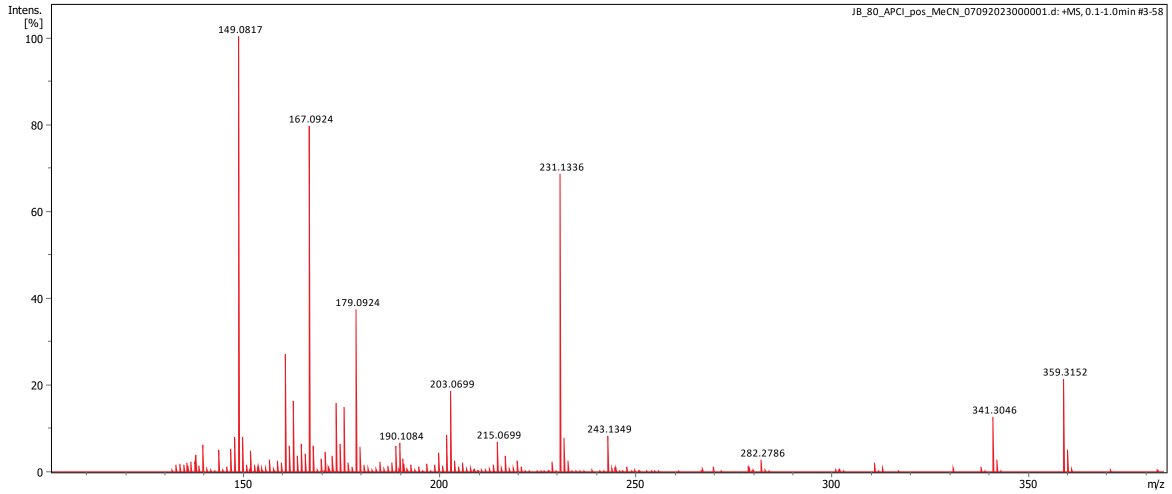

Intens. [%]
JB_80_APCI_pos_MeCN_07092023000001.d: +MS, 0.1-1.0min #3-58
149.0817
167.0924
179.0924
190.1084
203.0699
215.0699
231.1336
243.1349
282.2786
341.3046
359.3152
m/z

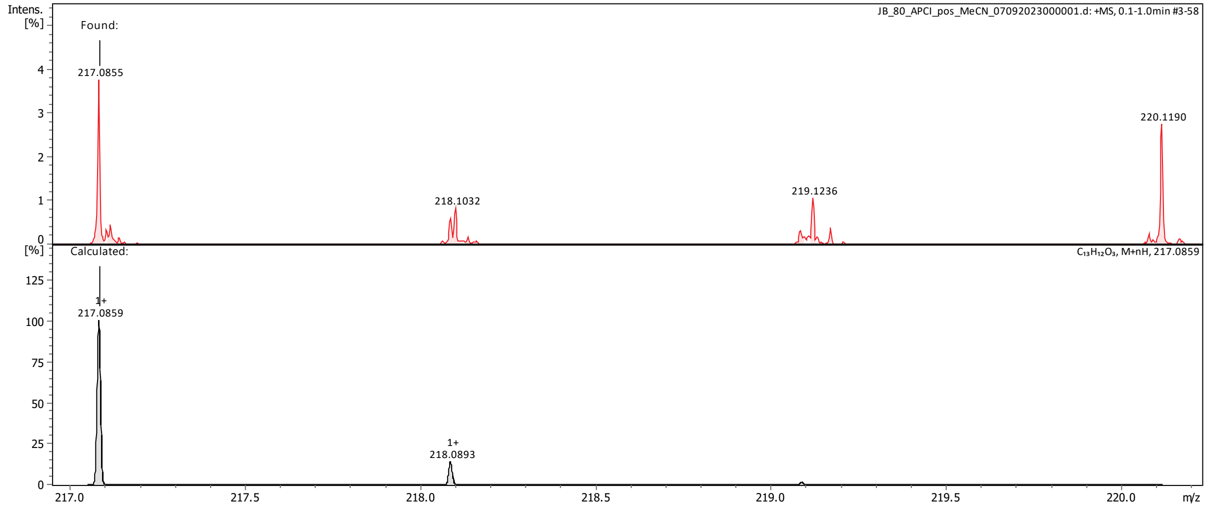

Intens. [%]
Found:
JB_80_APCI_pos_MeCN_07092023000001.d: +MS, 0.1-1.0min #3-58
217.0855
218.1032
219.1236
220.1190
Calculated:
C13H12O3, M+nH, 217.0859
1+ 217.0859
1+ 218.0893
m/z

IR

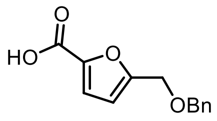

O
HO
O
OBn

S8

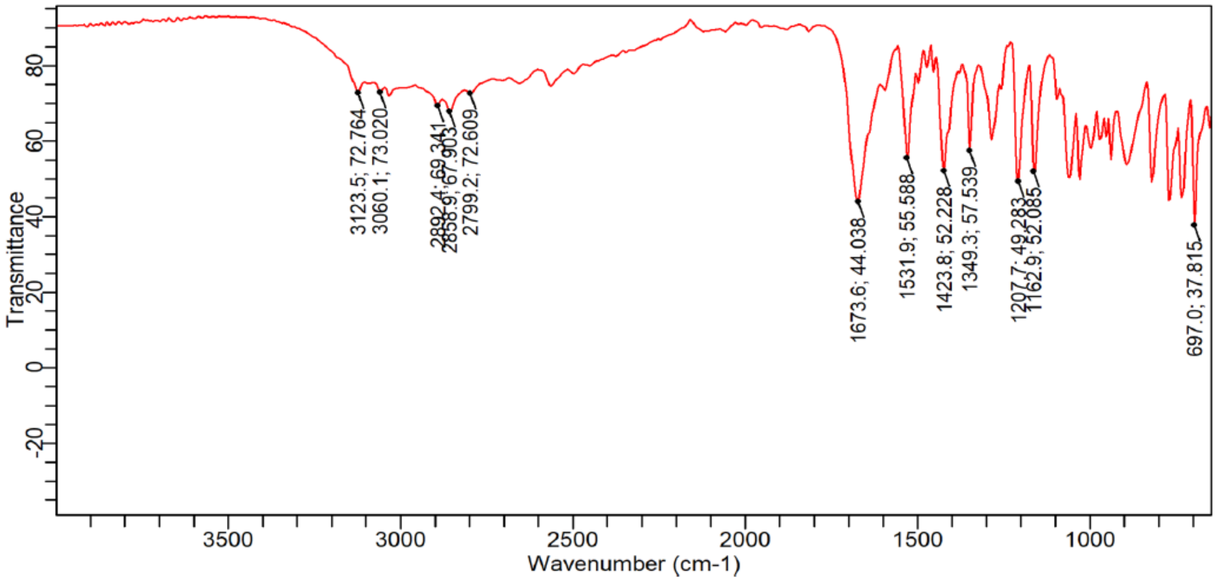

Transmittance
80
60
40
20
0
-20
3500
3000
2500
2000
1500
1000
Wavenumber (cm-1)
3123.5; 72.764
3060.1; 73.020
2892.4; 69.341
2858.9; 67.903
2799.2; 72.609
1673.6; 44.038
1531.9; 55.588
1423.8; 52.228
1349.3; 57.539
1207.7; 49.283
1162.9; 52.085
697.0; 37.815

21 (5-((Benzyloxy)methyl)furan-2-yl)(morpholino)methanone (1w)

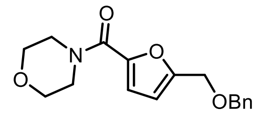

1w

^{1}H NMR

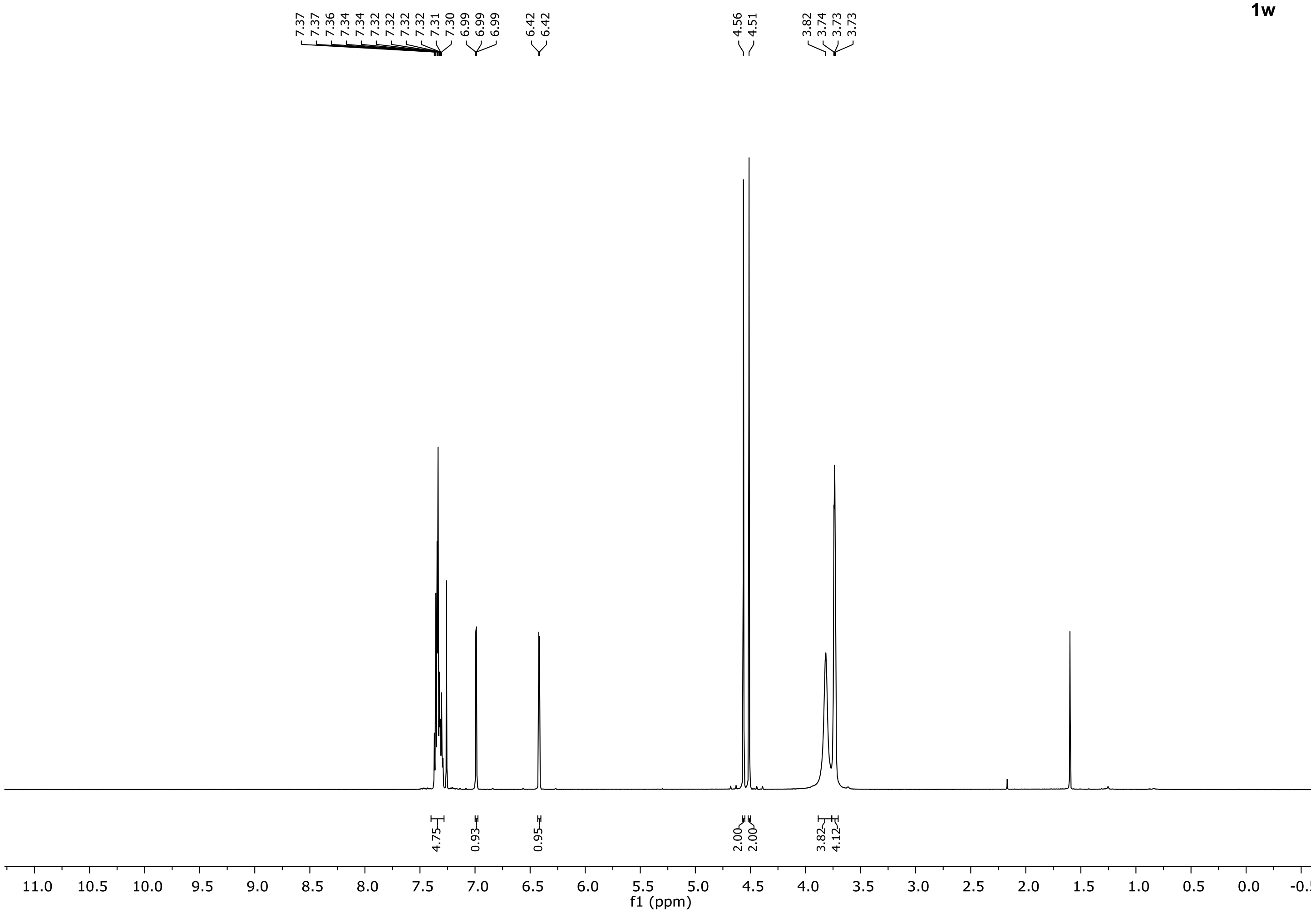

^{13}C NMR

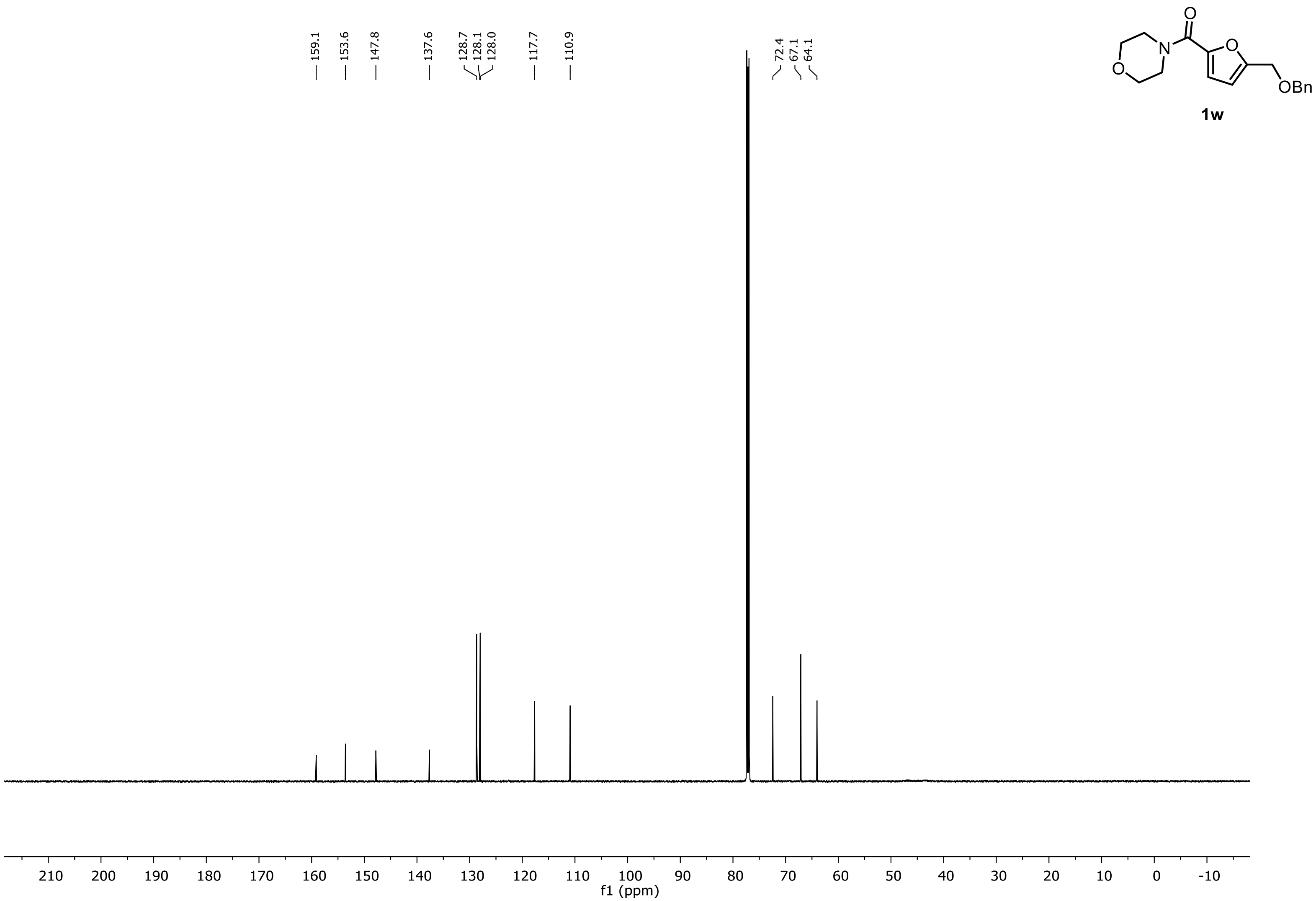
159.1
153.6
147.8
137.6
128.7
128.1
128.0
117.7
110.9
72.4
67.1
64.1
O
N
O
O
OBn
210 200 190 180 170 160 150 140 130 120 110 100 90 80 70 60 50 40 30 20 10 0 -10
f1 (ppm)

1w

^{1}H, ^{1}H COSY

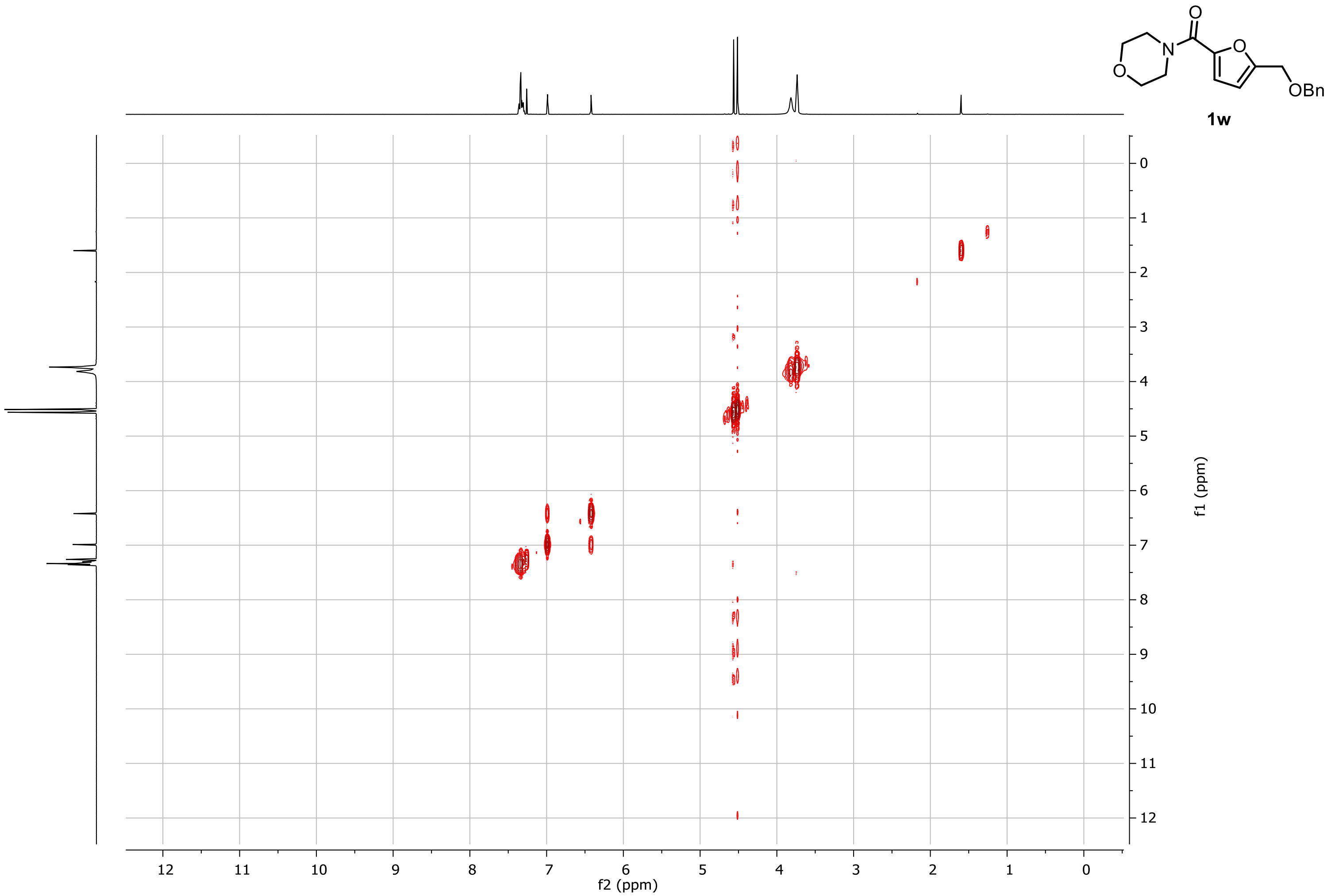

1w

^{1}H, ^{13}C HMBC

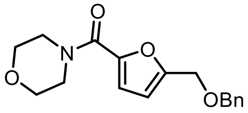

1w

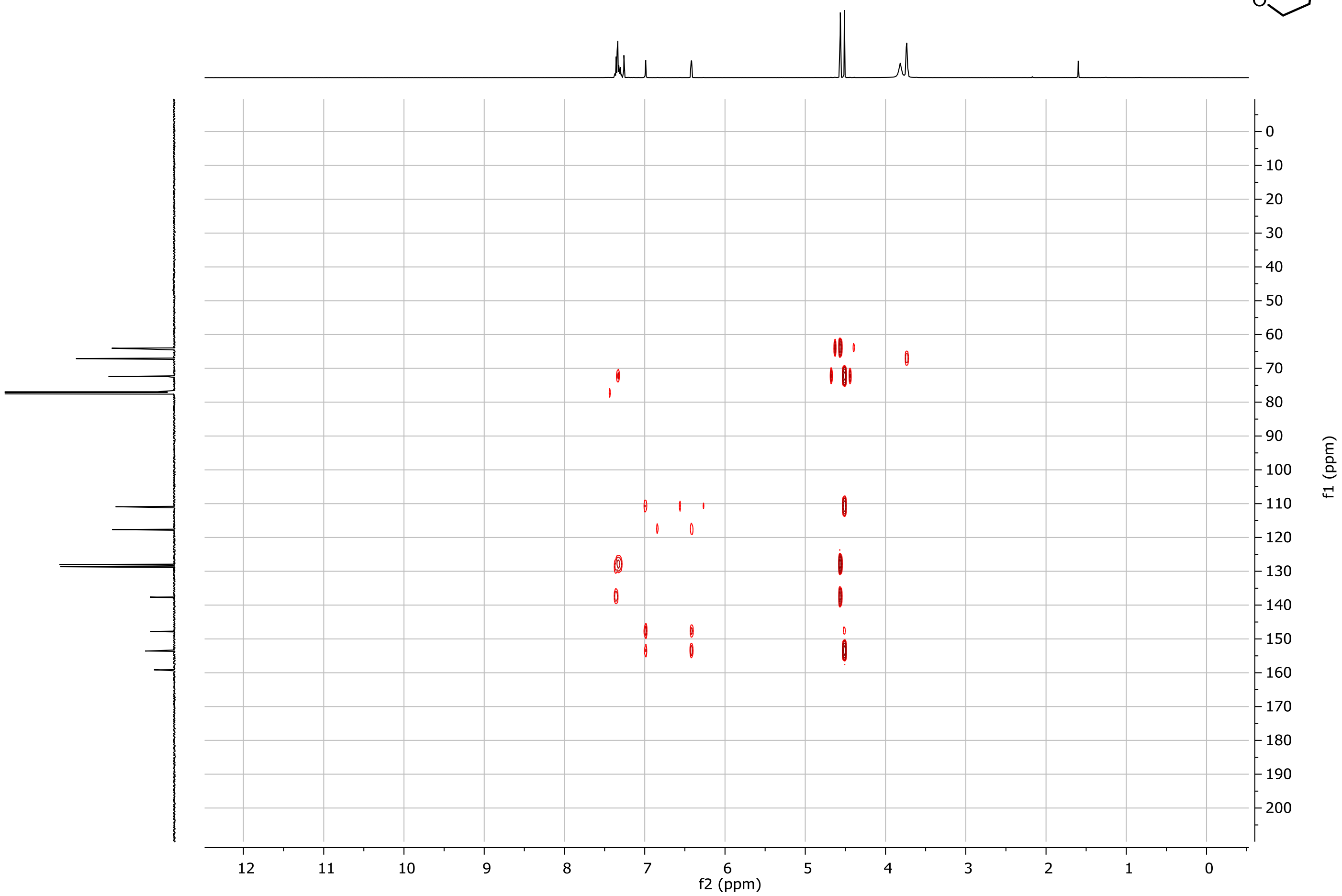

^{1}H, ^{13}C HSQC

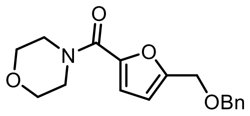

1w

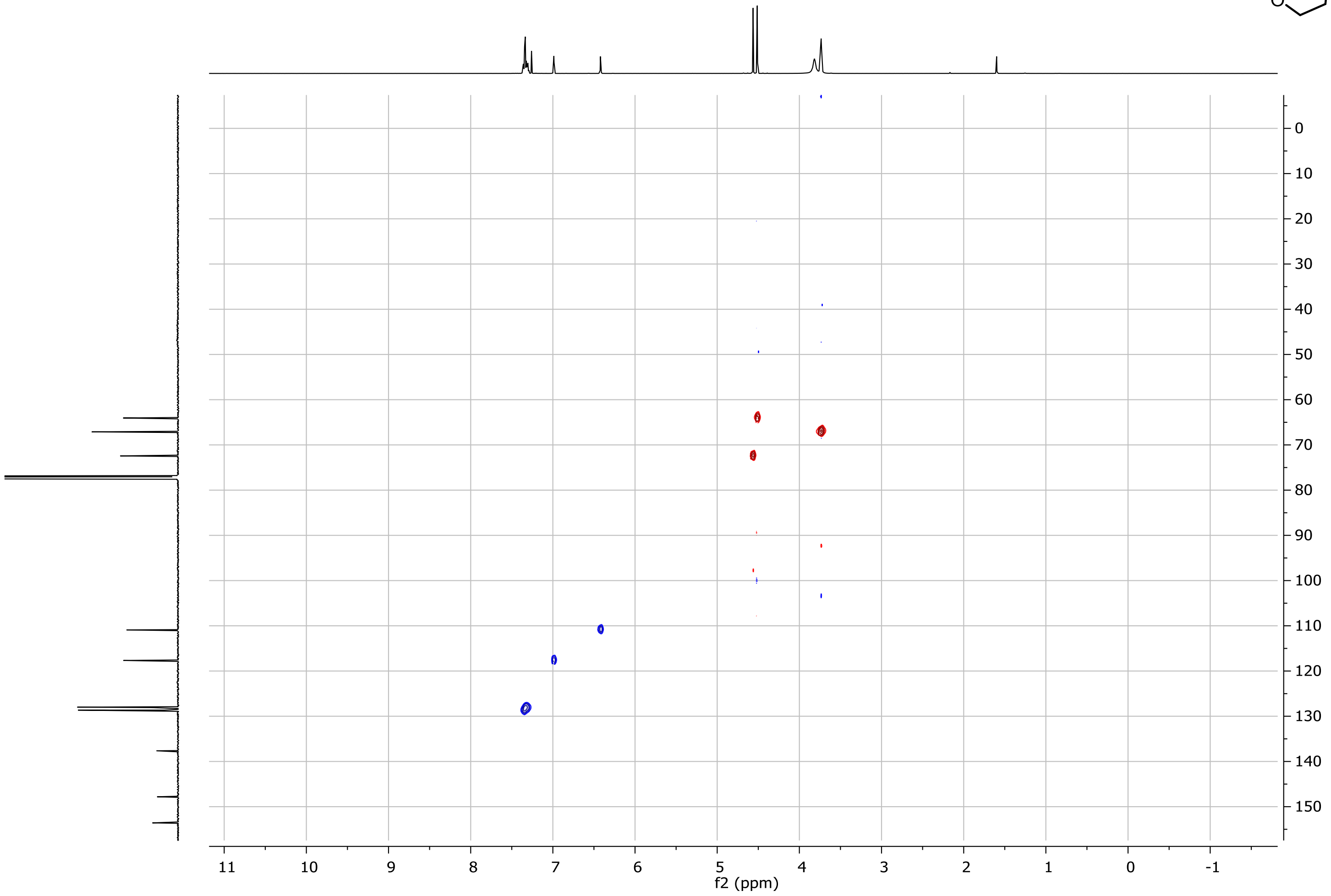

HRMS

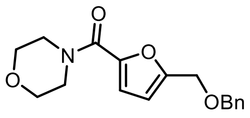

O
N
O
O
OBn

1w

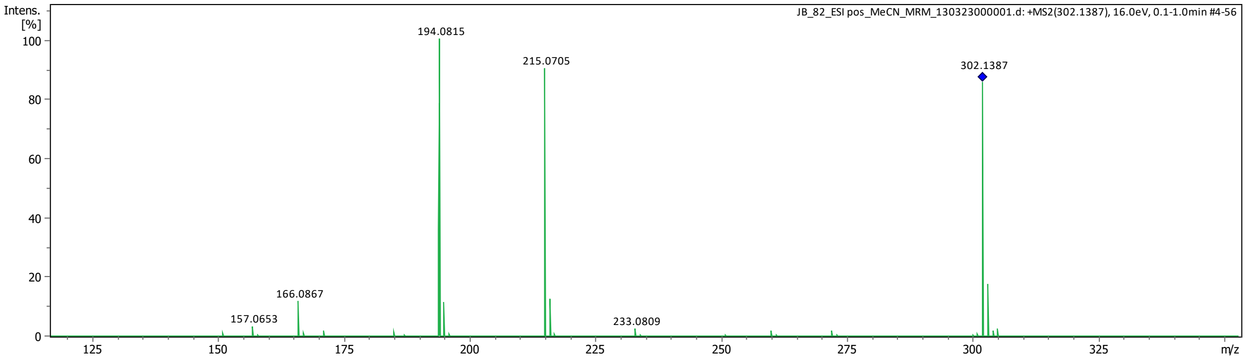

Intens. [%]
JB_82_ESI pos_MeCN_MRM_130323000001.d: +MS2(302.1387), 16.0eV, 0.1-1.0min #4-56
194.0815
215.0705
302.1387
166.0867
157.0653
233.0809
m/z

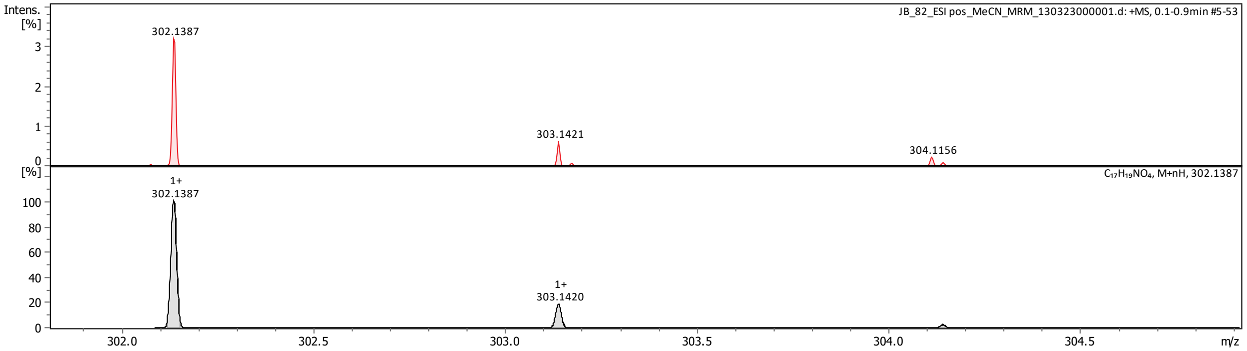

Intens. [%]
JB_82_ESI pos_MeCN_MRM_130323000001.d: +MS, 0.1-0.9min #5-53
302.1387
303.1421
304.1156
C17H19NO4, M+nH, 302.1387
1+
302.1387
1+
303.1420
m/z

IR

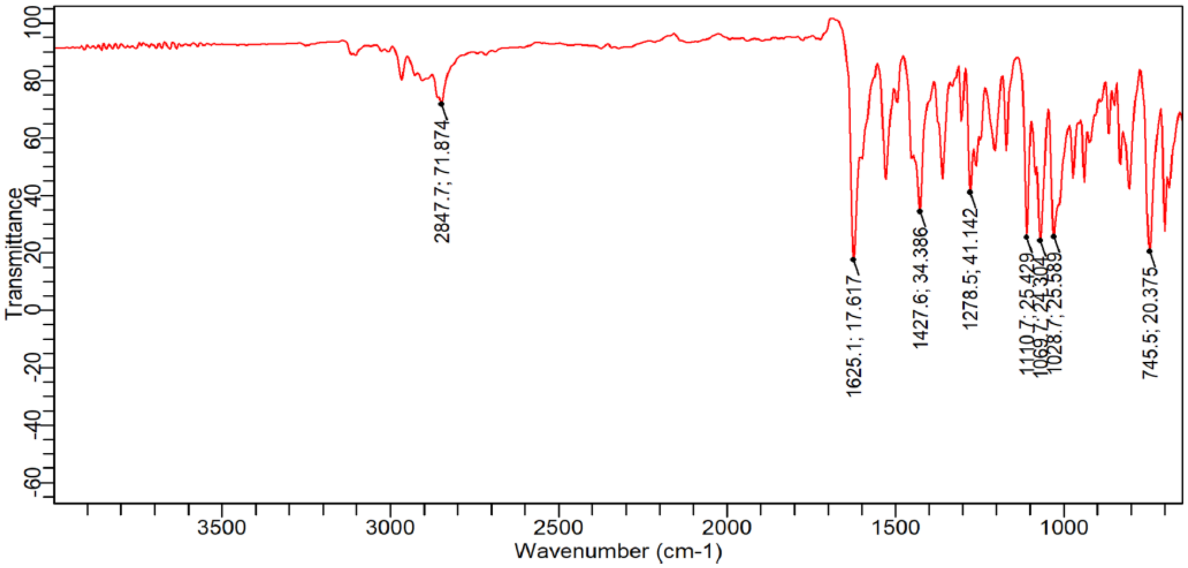

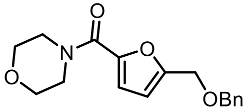

1w

22 1-Morpholino-4-phenylbutan-1-one (1x)

^{1}H NMR

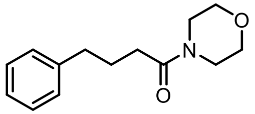

1x

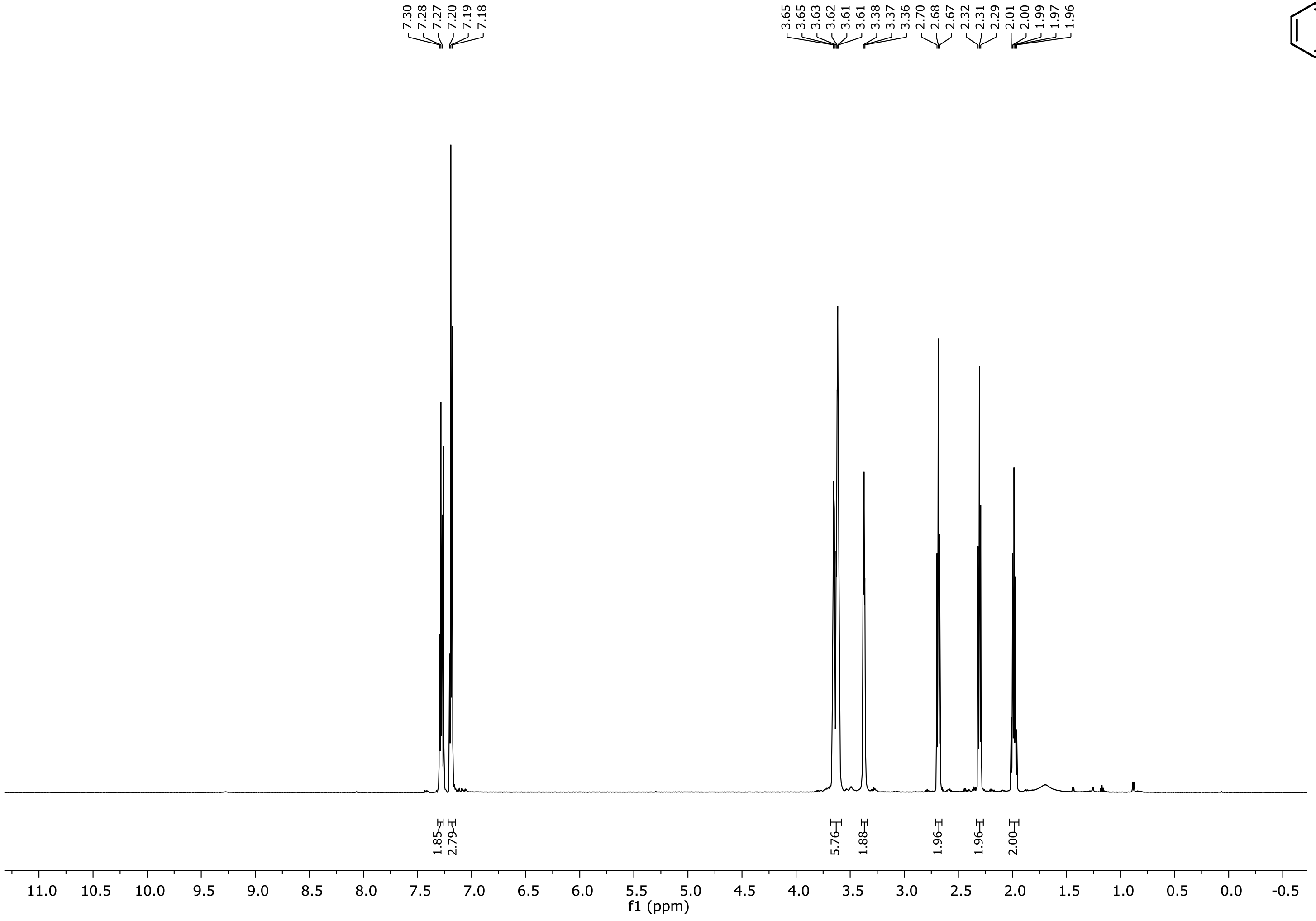

^{13}C NMR

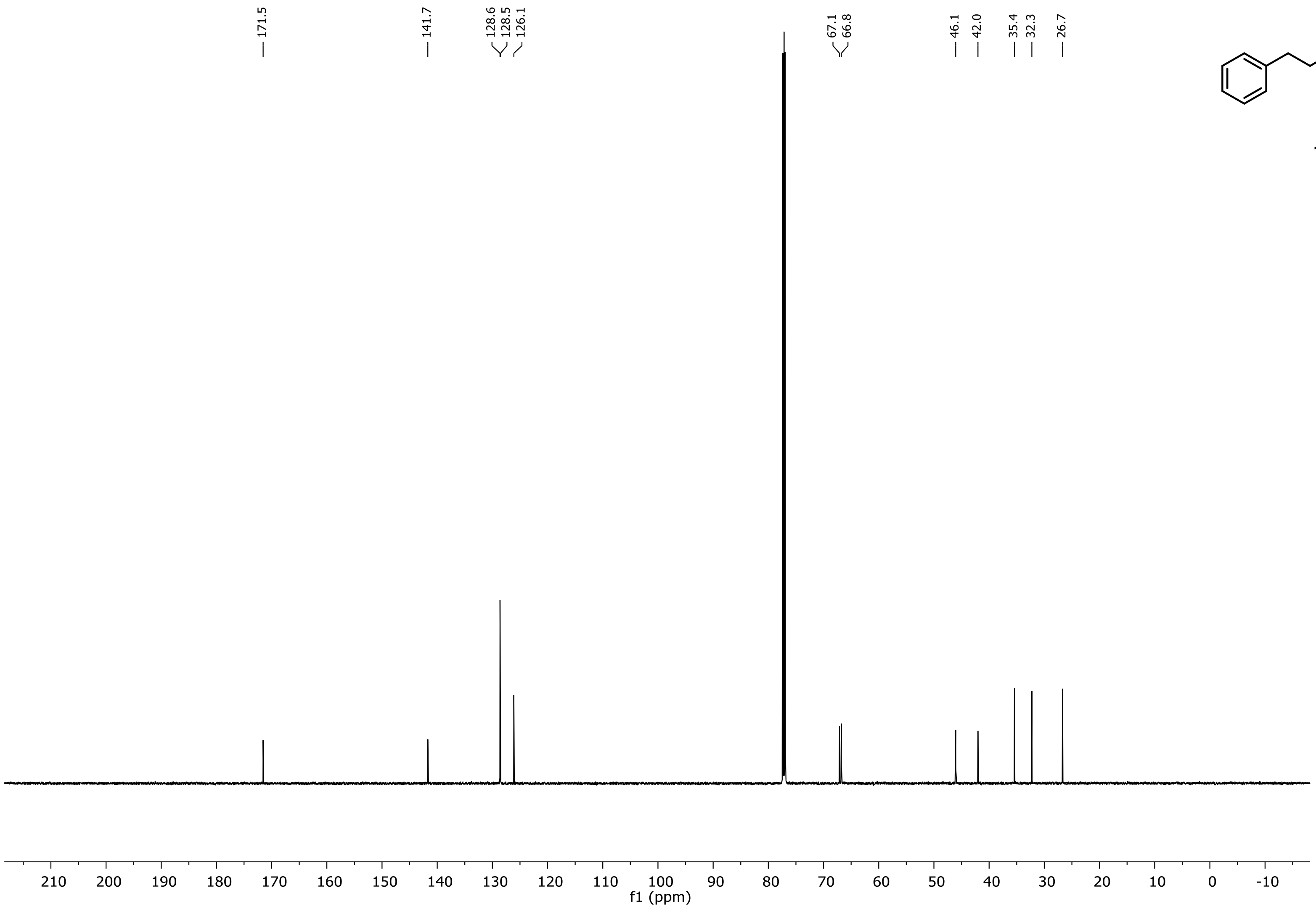

171.5
141.7
128.6
128.5
126.1
67.1
66.8
46.1
42.0
35.4
32.3
26.7
210 200 190 180 170 160 150 140 130 120 110 100 90 80 70 60 50 40 30 20 10 0 -10
f1 (ppm)

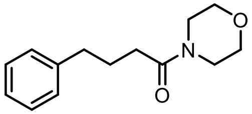

N
O
O

1x

^{1}H, ^{1}H COSY

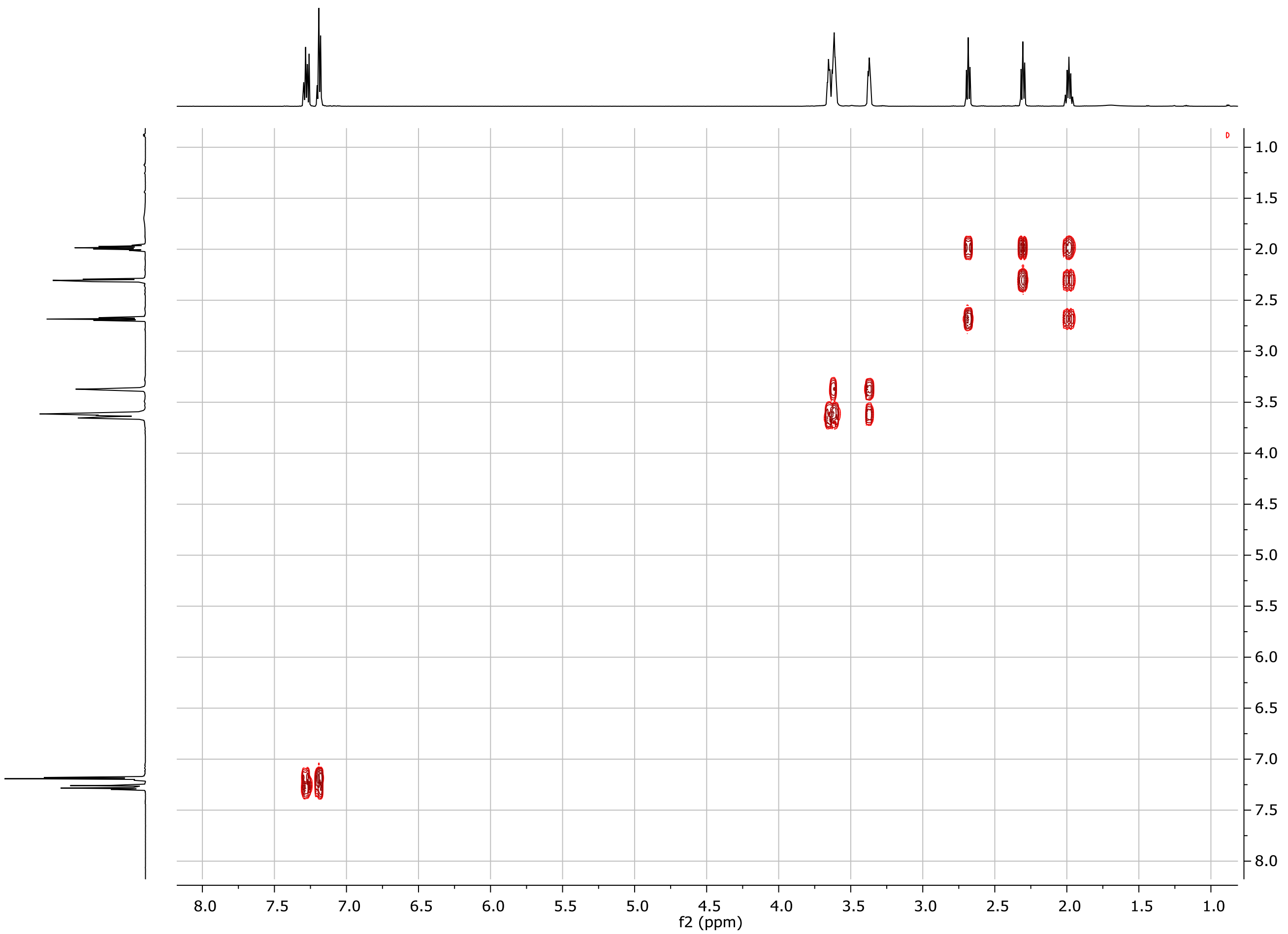

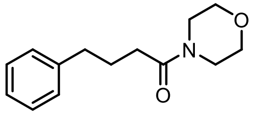

1x

^{1}H, ^{13}C HMBC

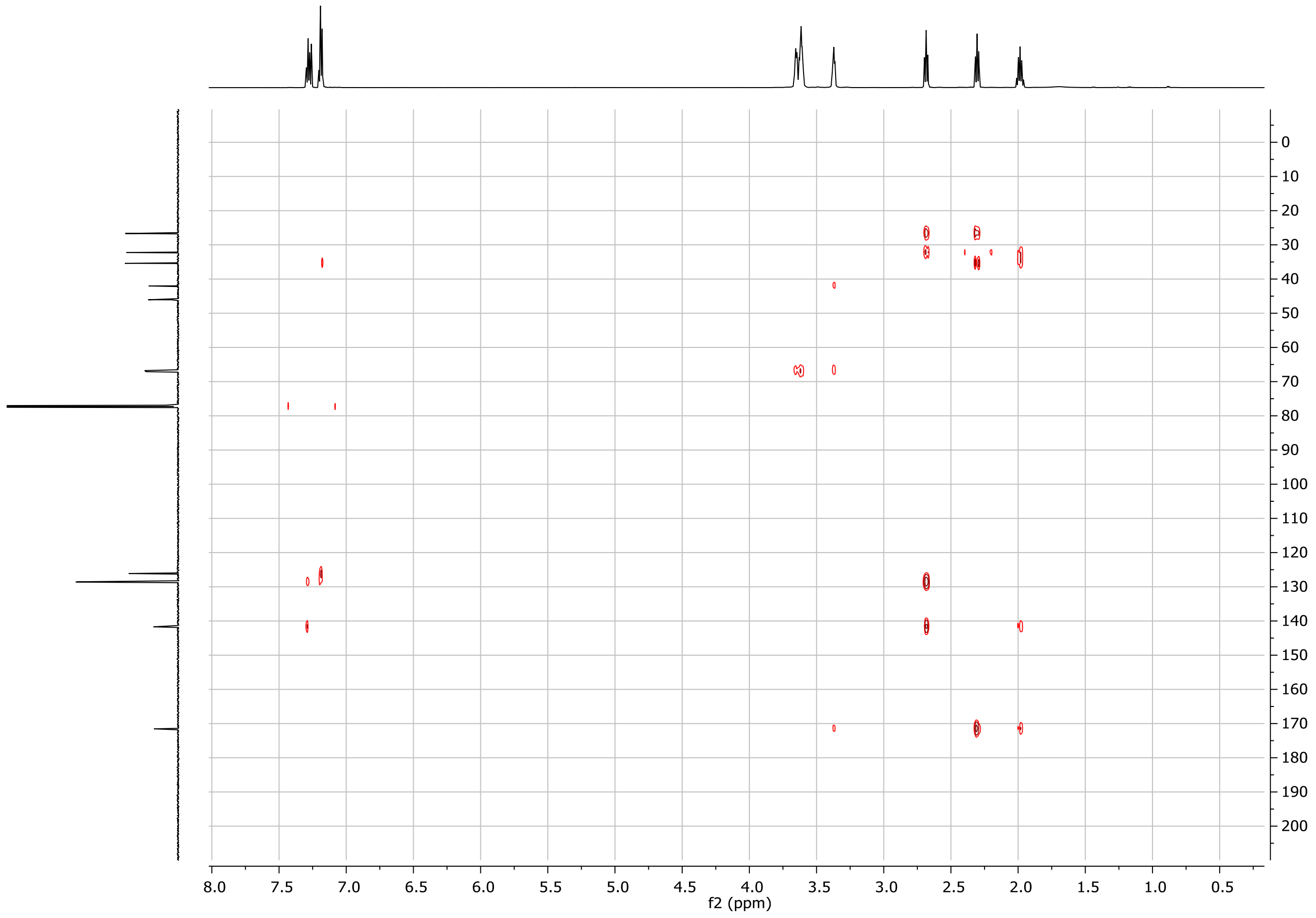

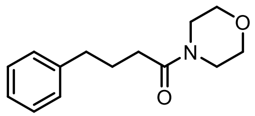

1x

^{1}H, ^{13}C HSQC

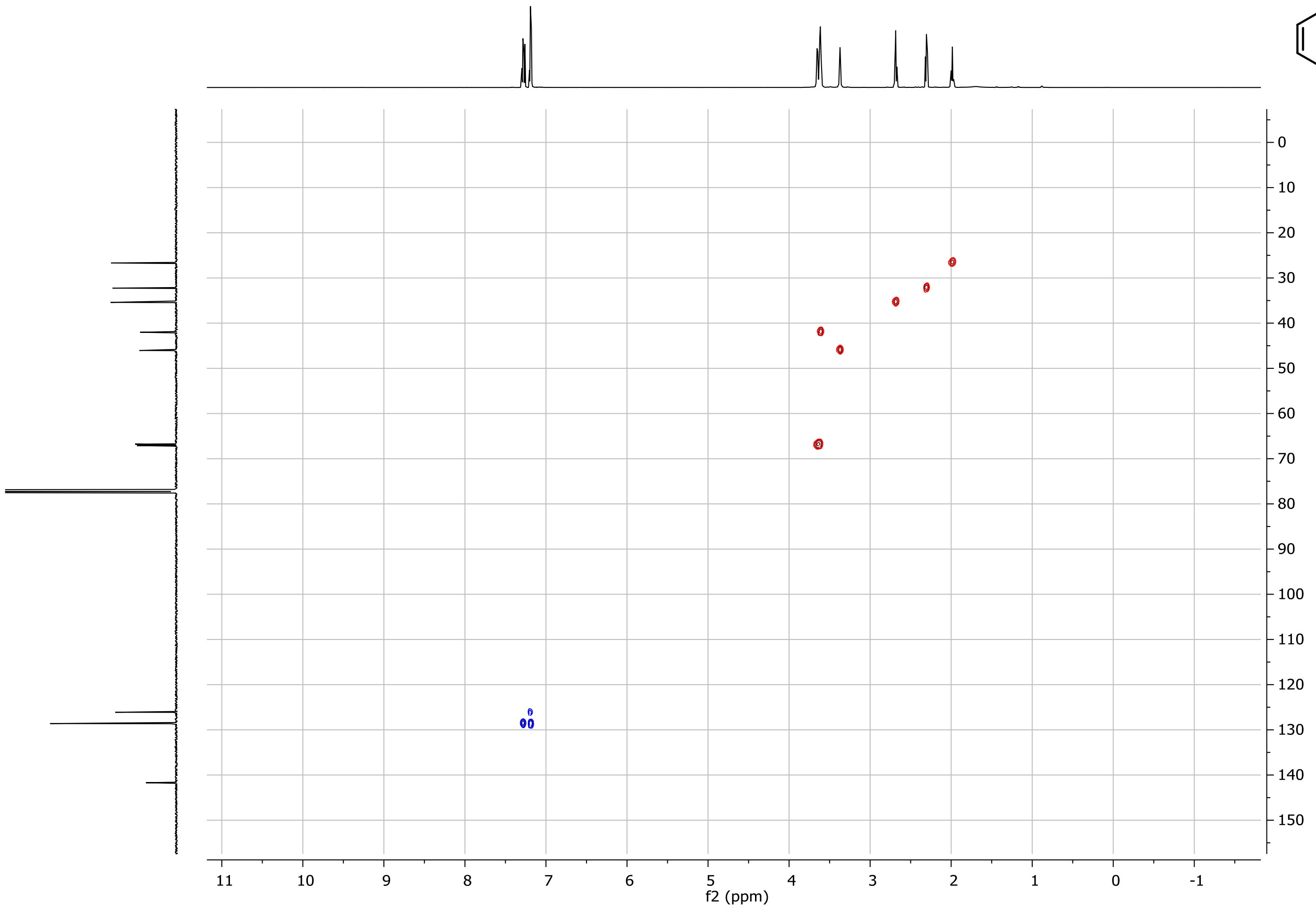

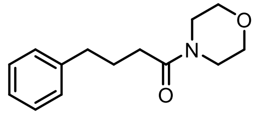

1x

HRMS

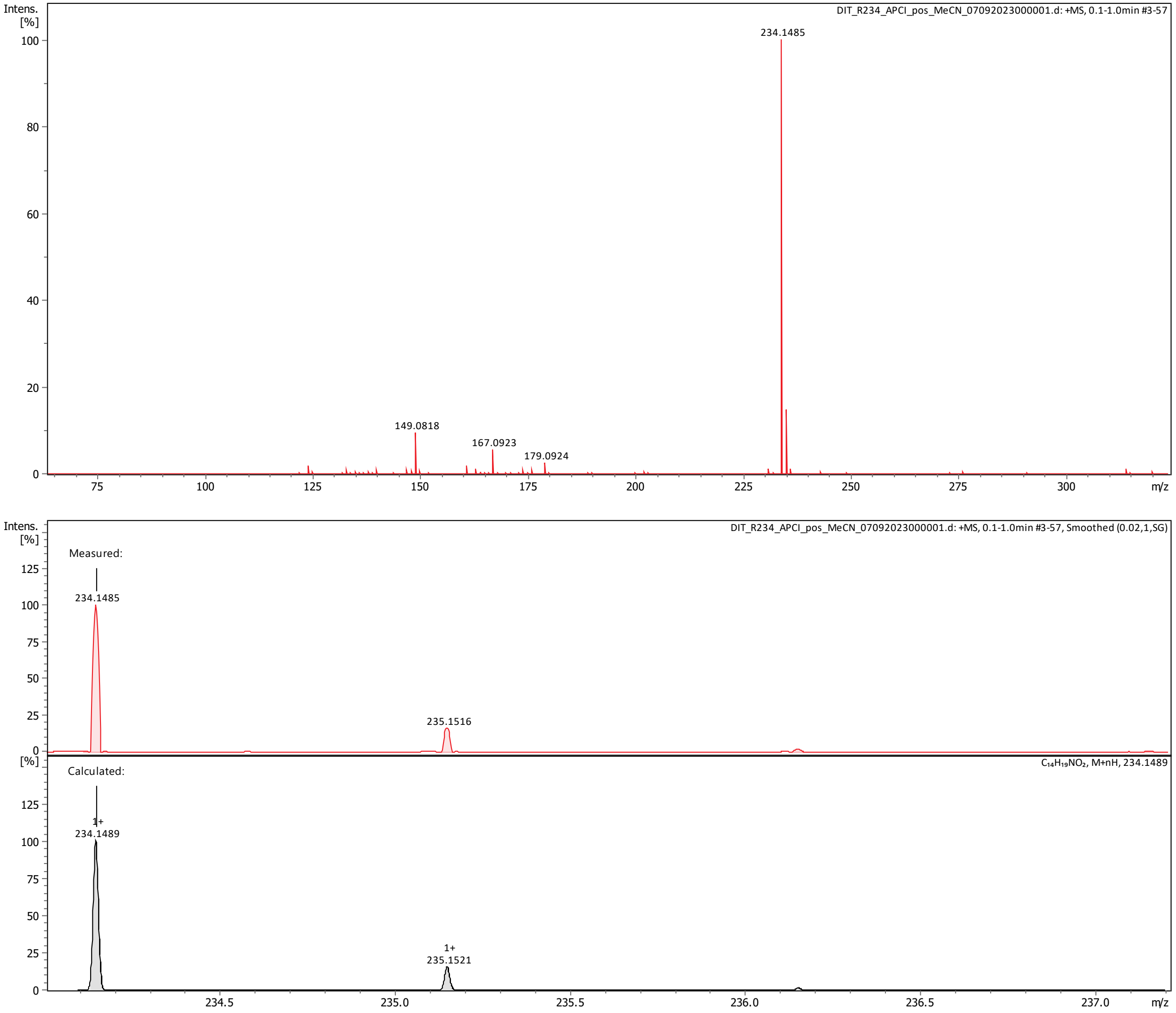
DIT_R234_APCI_pos_MeCN_07092023000001.d: +MS, 0.1-1.0min #3-57
234.1485
149.0818
167.0923
179.0924
DIT_R234_APCI_pos_MeCN_07092023000001.d: +MS, 0.1-1.0min #3-57, Smoothed (0.02,1,SG)
Measured:
234.1485
235.1516
Calculated:
C14H19NO2, M+nH, 234.1489
1+
234.1489
1+
235.1521
Intens. [%]
m/z

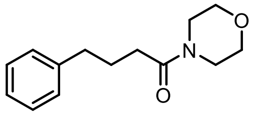

1x

23 1-Morpholinoundec-10-en-1-one (1y)

^{1}H NMR

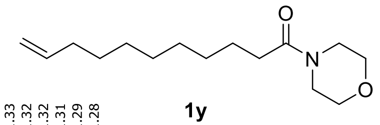

1y

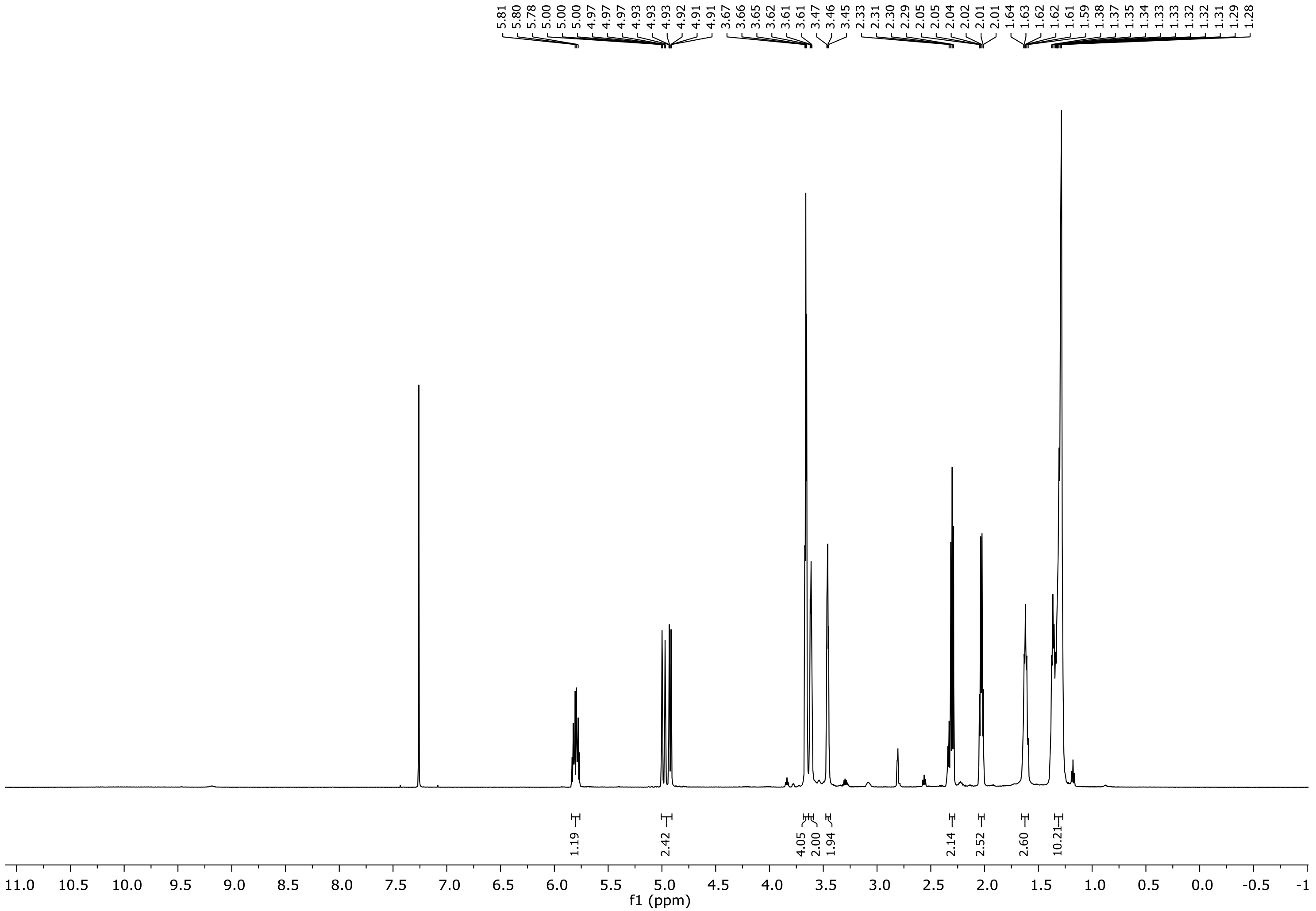

^{13}C NMR

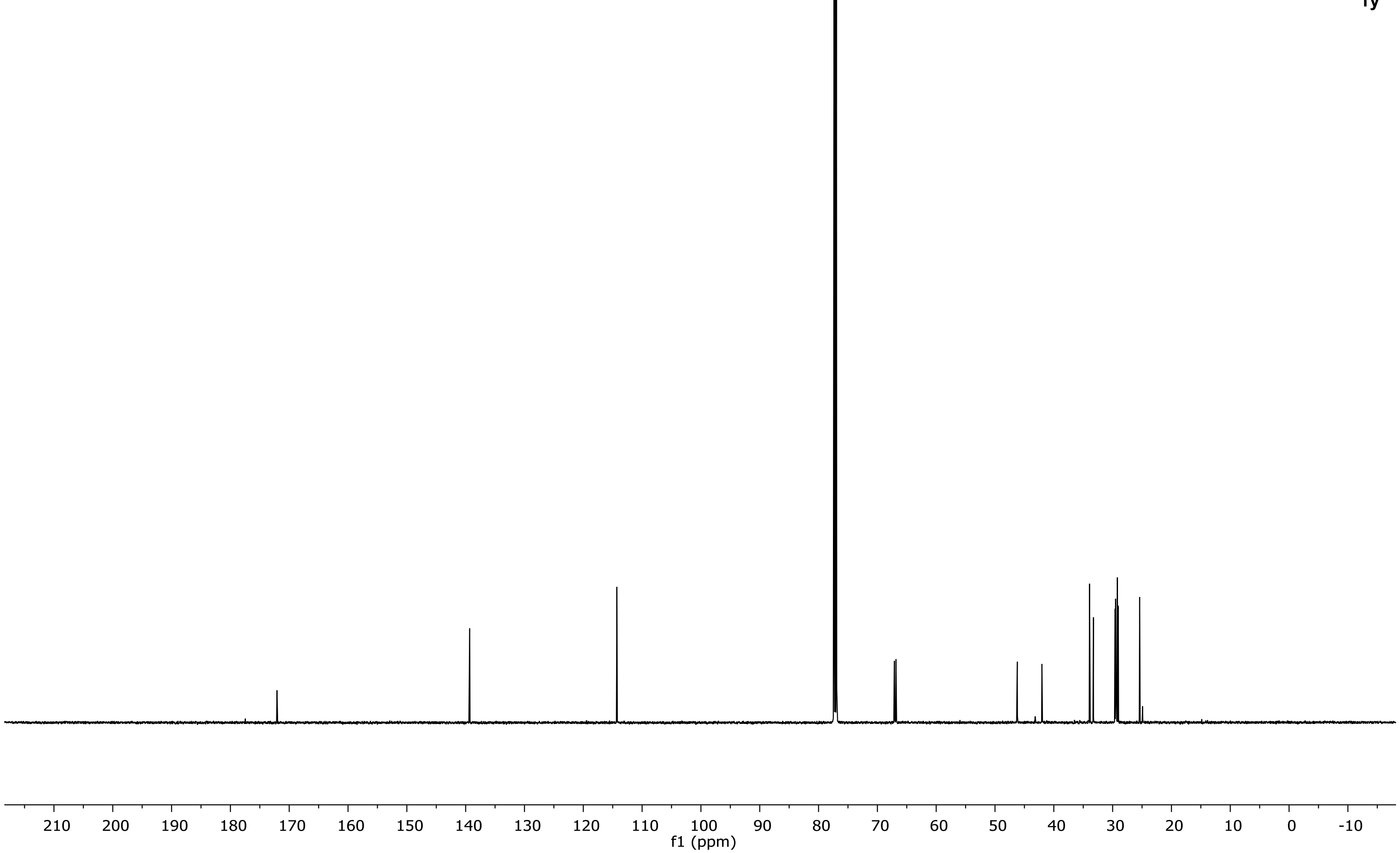

172.1
139.3
114.3
67.1
66.8
46.2
42.0
33.9
33.3
29.6
29.5
29.5
29.2
29.0
25.4
210
200
190
180
170
160
150
140
130
120
110
100
90
80
70
60
50
40
30
20
10
0
-10
f1 (ppm)

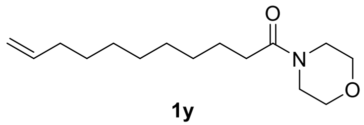

O
N
O

1y

^{1}H, ^{1}H COSY

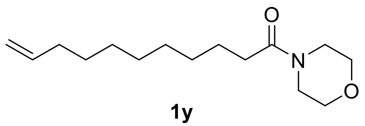

1y

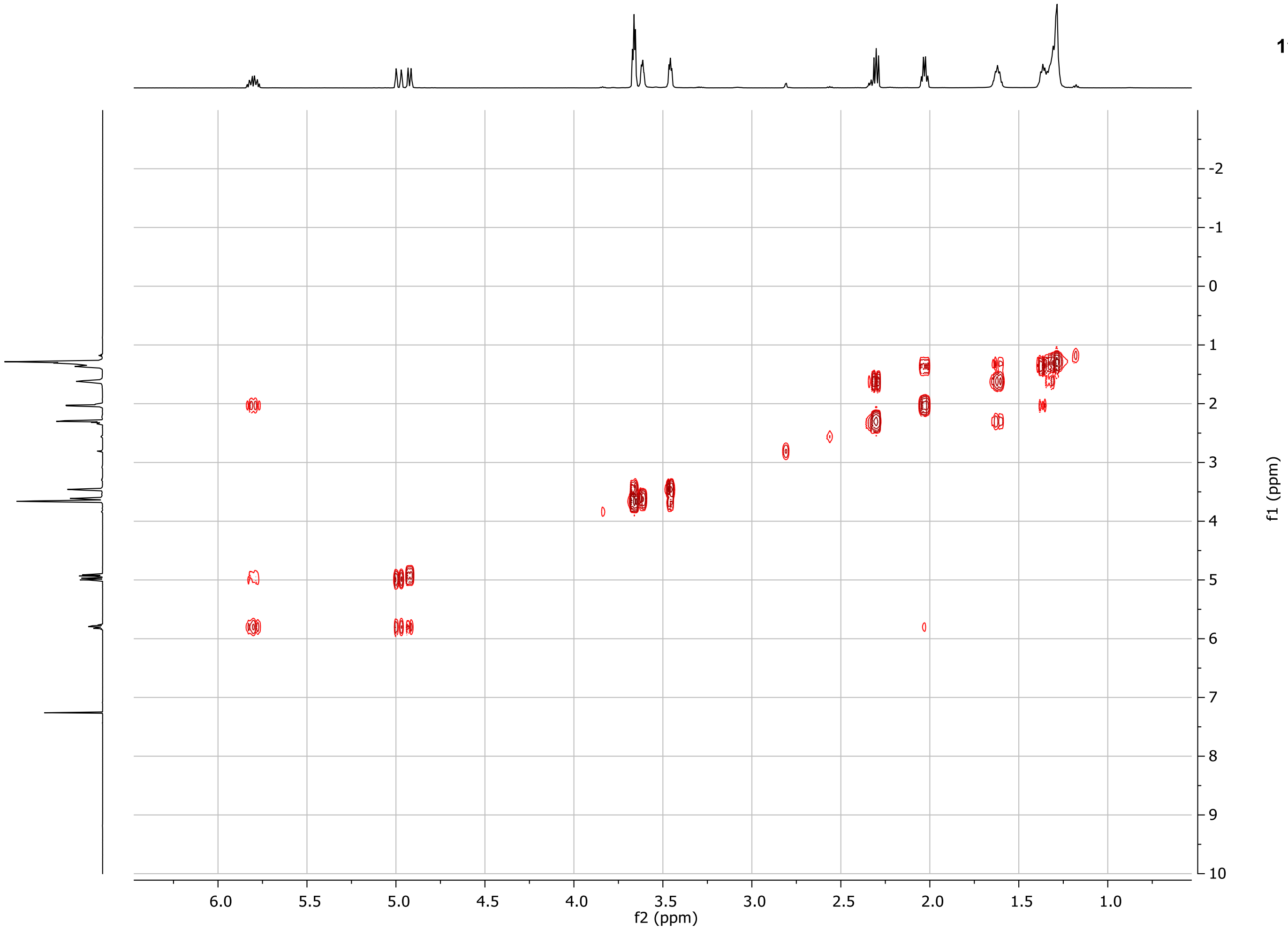

^{1}H, ^{13}C HMBC

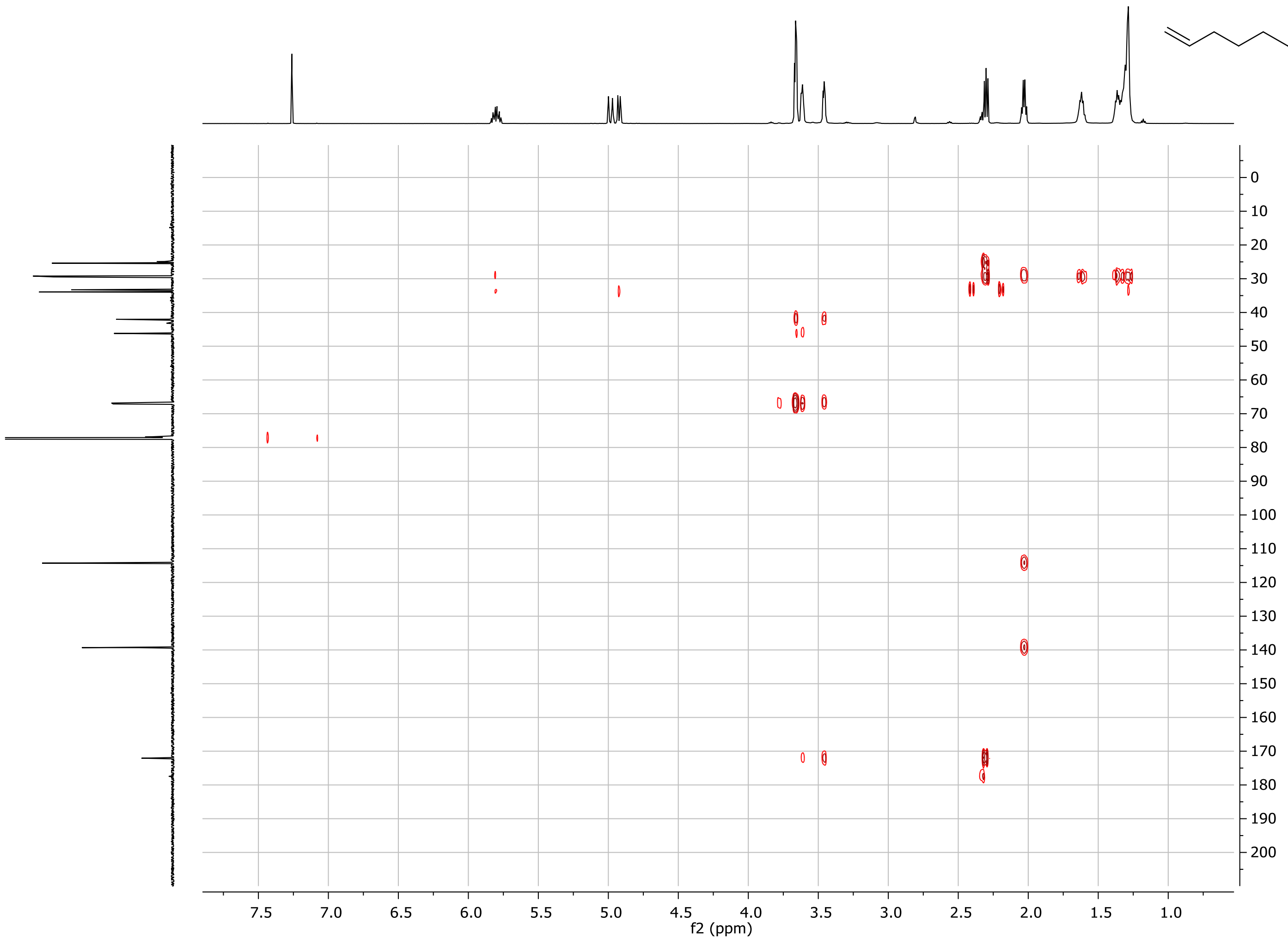

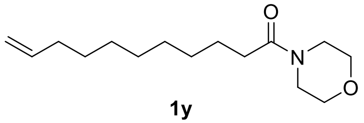

1y

^{1}H, ^{13}C HSQC

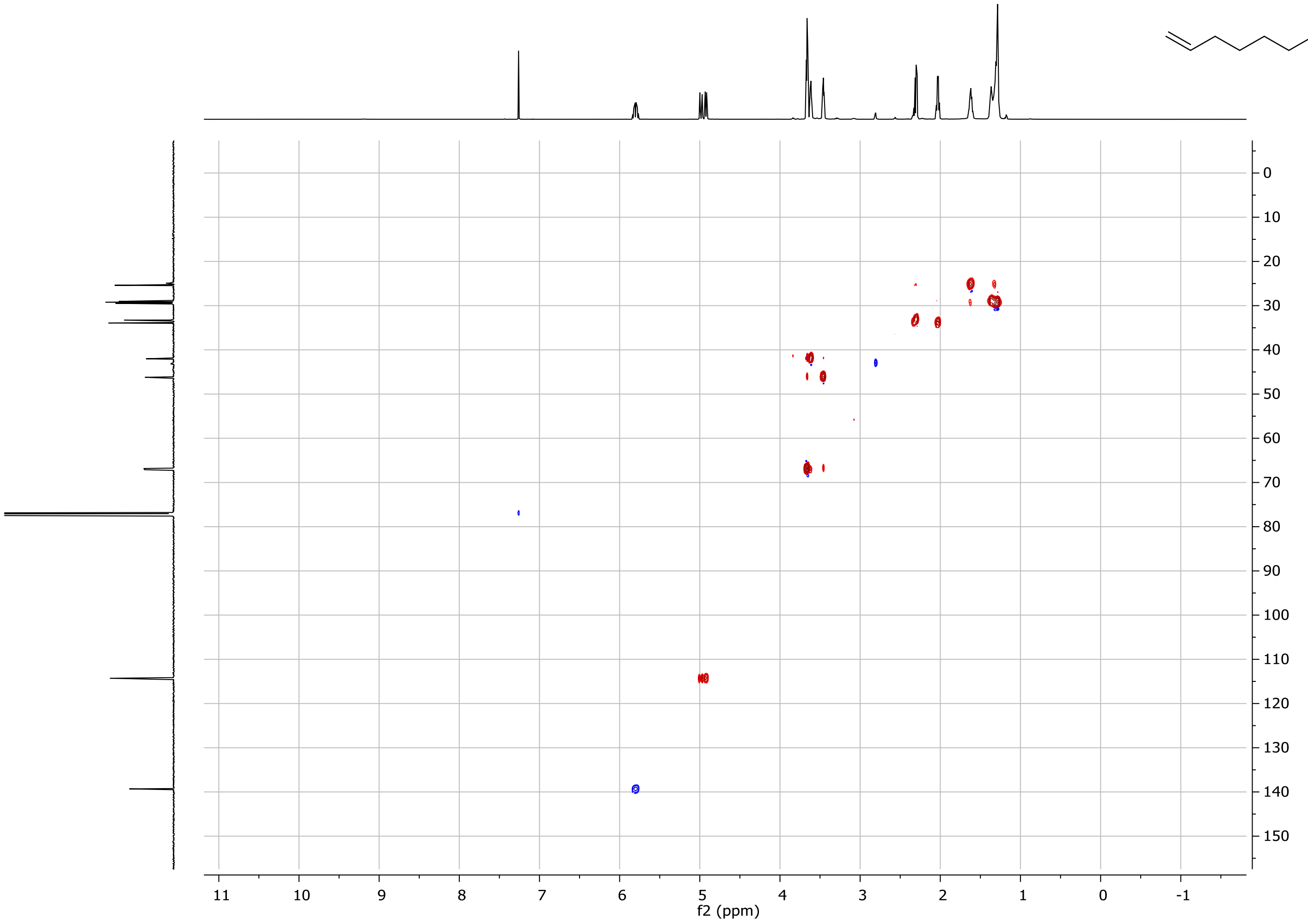

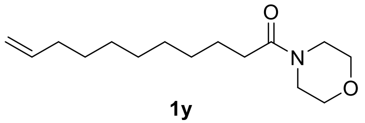

1y

HRMS

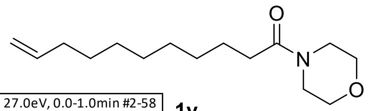

1y

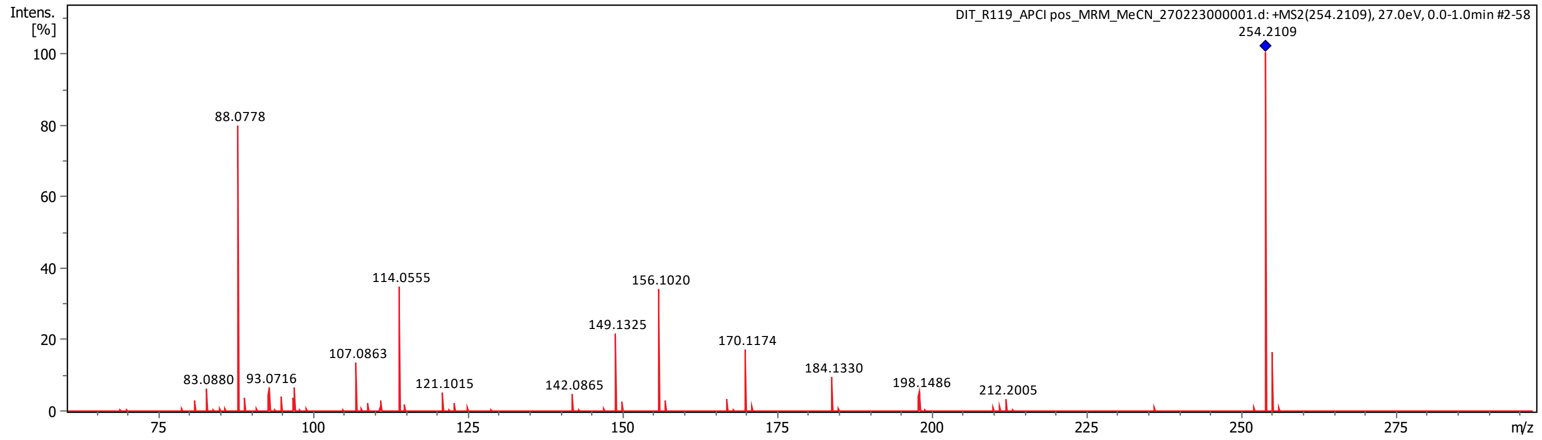
Intens. [%]
DIT_R119_APCI pos_MRM_MeCN_270223000001.d: +MS2(254.2109), 27.0eV, 0.0-1.0min #2-58
254.2109
88.0778
114.0555
156.1020
149.1325
170.1174
107.0863
184.1330
83.0880
93.0716
121.1015
142.0865
198.1486
212.2005
m/z

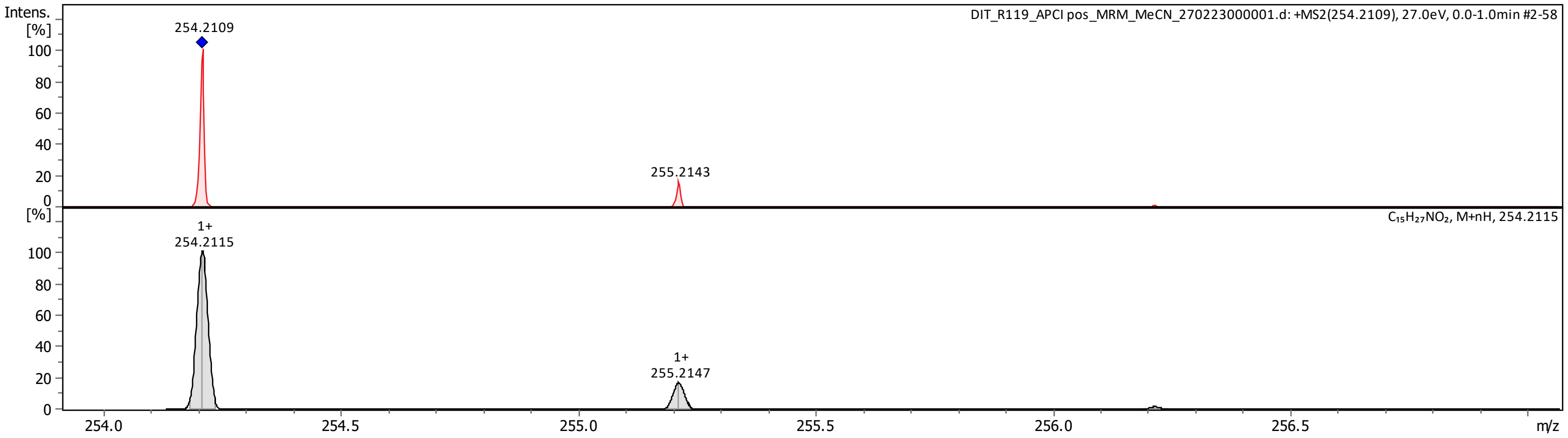
Intens. [%]
DIT_R119_APCI pos_MRM_MeCN_270223000001.d: +MS2(254.2109), 27.0eV, 0.0-1.0min #2-58
254.2109
255.2143
C₁₅H₂₇NO₂, M+nH, 254.2115
1+
254.2115
1+
255.2147
m/z

IR

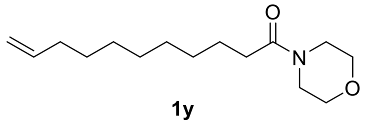

1y

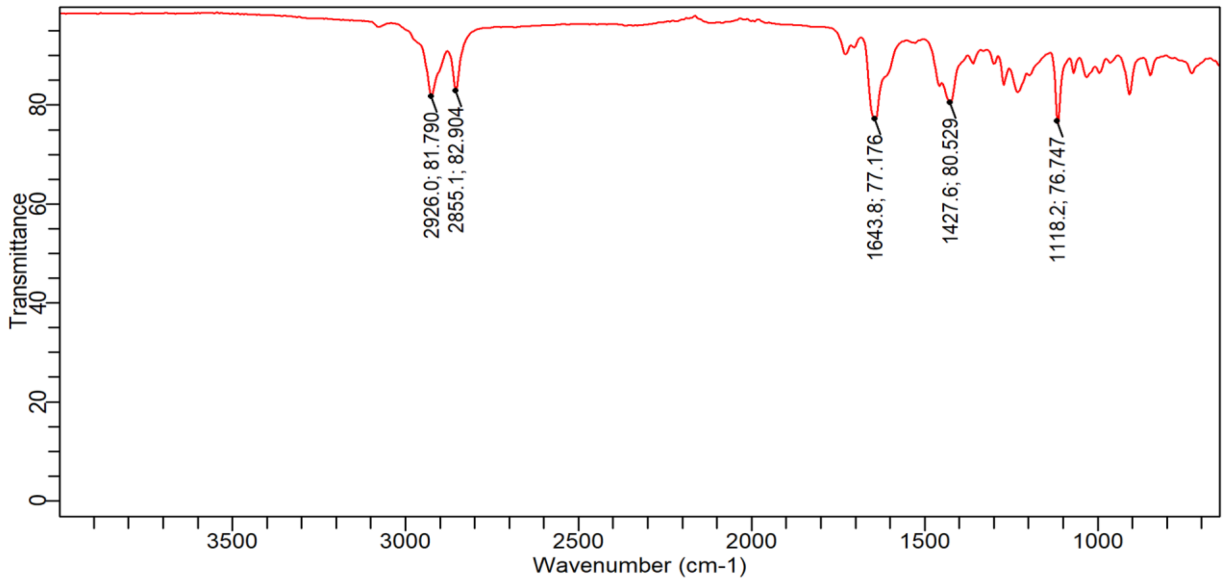

2926.0; 81.790
2855.1; 82.904
1643.8; 77.176
1427.6; 80.529
1118.2; 76.747
Transmittance
0
20
40
60
80
3500
3000
2500
2000
1500
1000
Wavenumber (cm-1)

24 1-Morpholinodecan-1-one (1z)

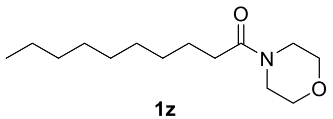

1z

^{1}H NMR

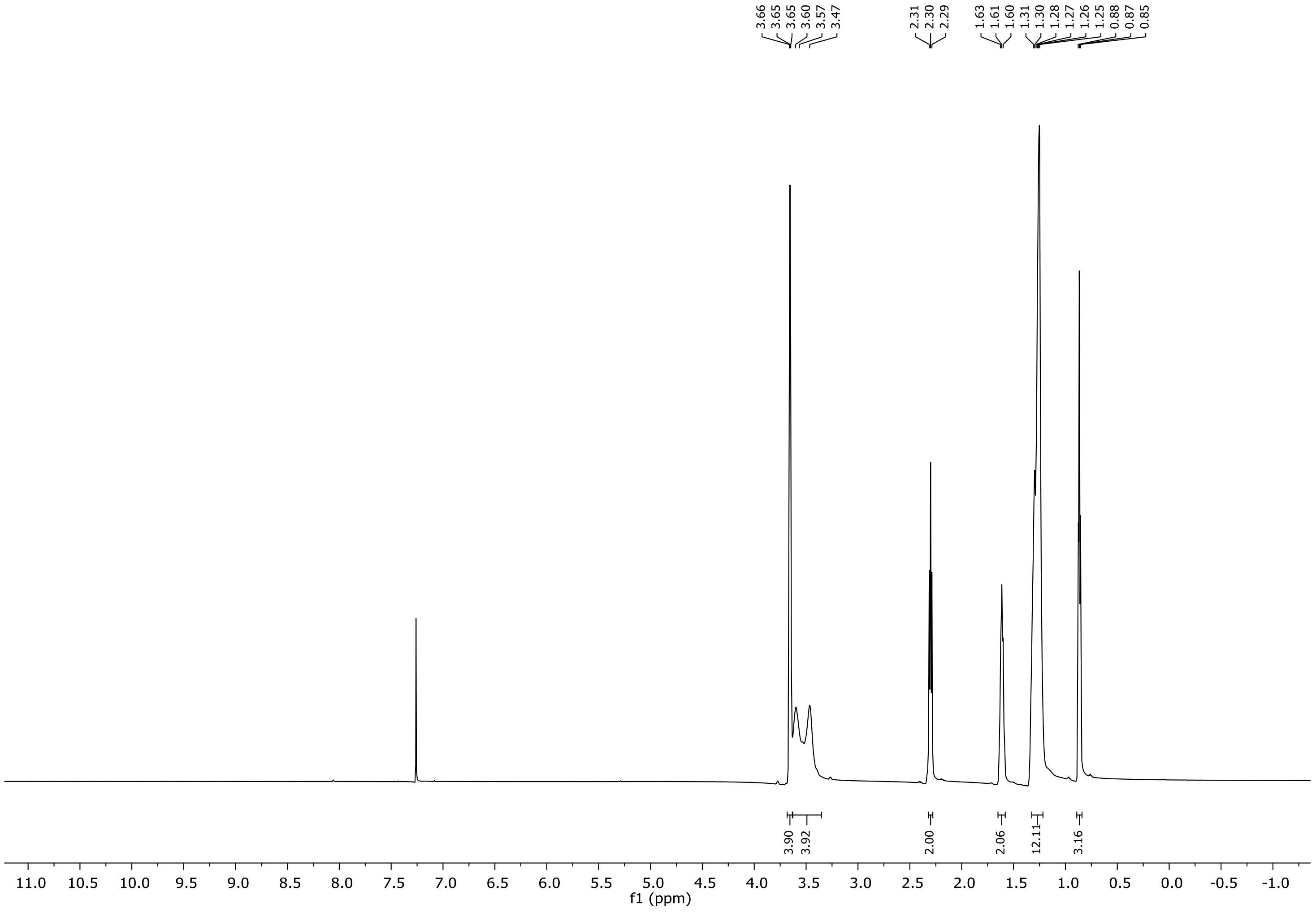

^{13}C NMR

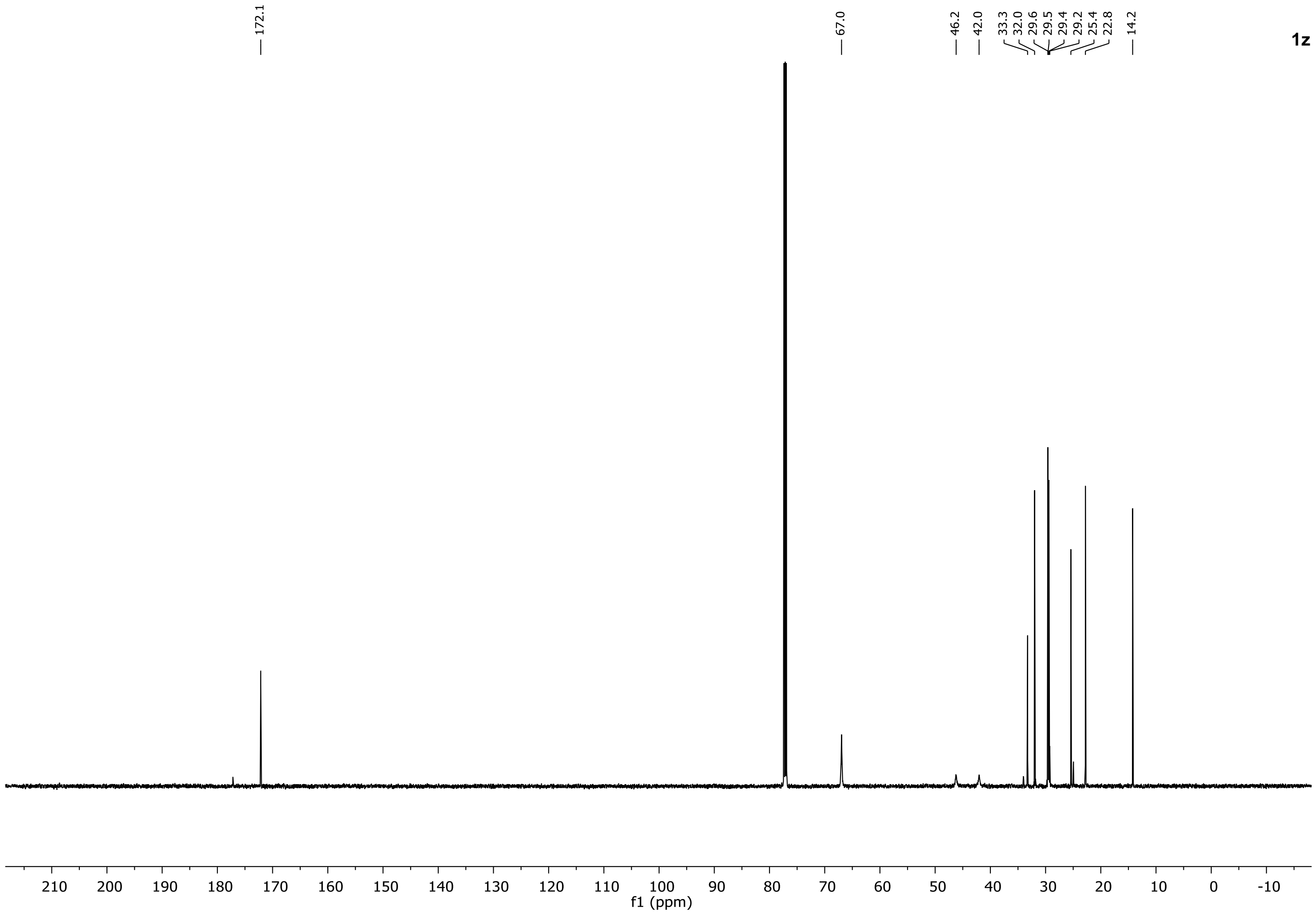

172.1
67.0
46.2
42.0
33.3
32.0
29.6
29.5
29.4
29.2
25.4
22.8
14.2
210 200 190 180 170 160 150 140 130 120 110 100 90 80 70 60 50 40 30 20 10 0 -10
f1 (ppm)

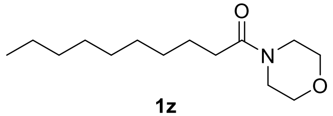

O
N
O

1z

^{1}H, ^{1}H COSY

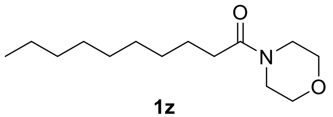

1z

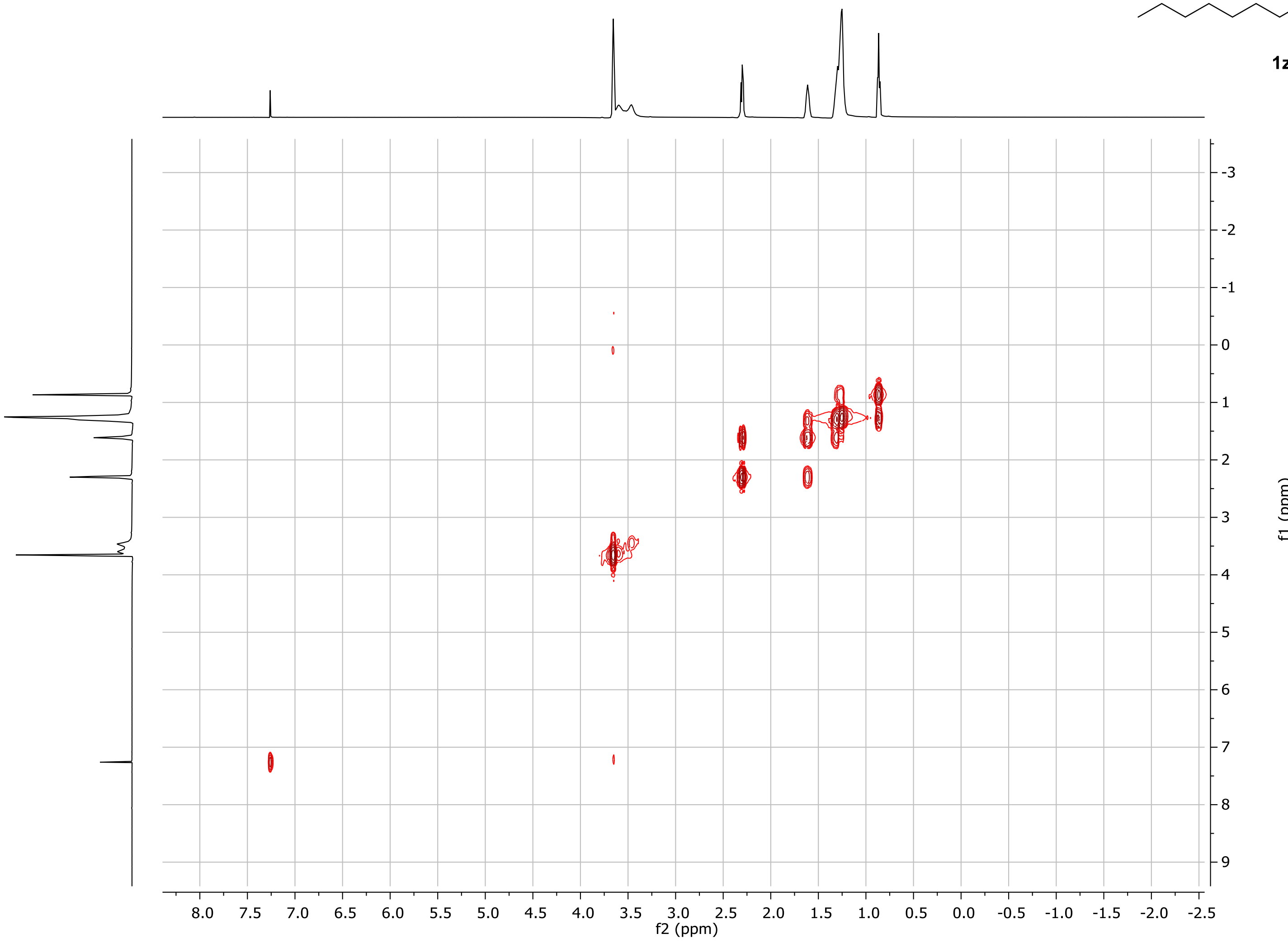
f1 (ppm)
f2 (ppm)
8.0 7.5 7.0 6.5 6.0 5.5 5.0 4.5 4.0 3.5 3.0 2.5 2.0 1.5 1.0 0.5 0.0 -0.5 -1.0 -1.5 -2.0 -2.5
-3 -2 -1 0 1 2 3 4 5 6 7 8 9

^{1}H, ^{13}C HMBC

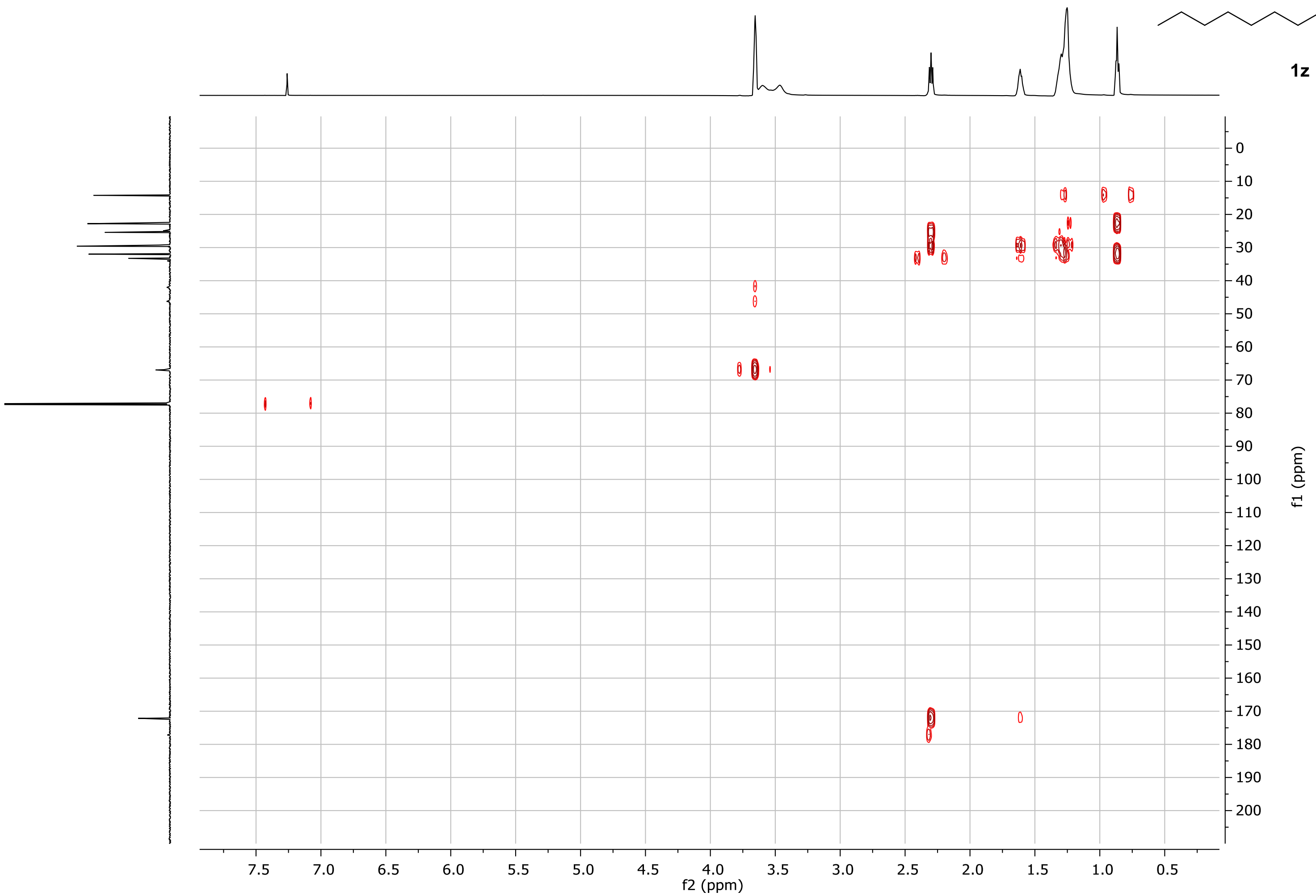

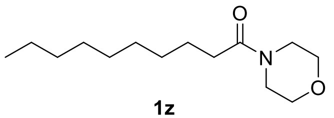

1z

^{1}H, ^{13}C HSQC

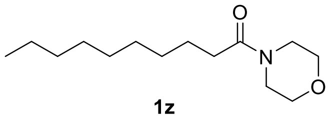

1z

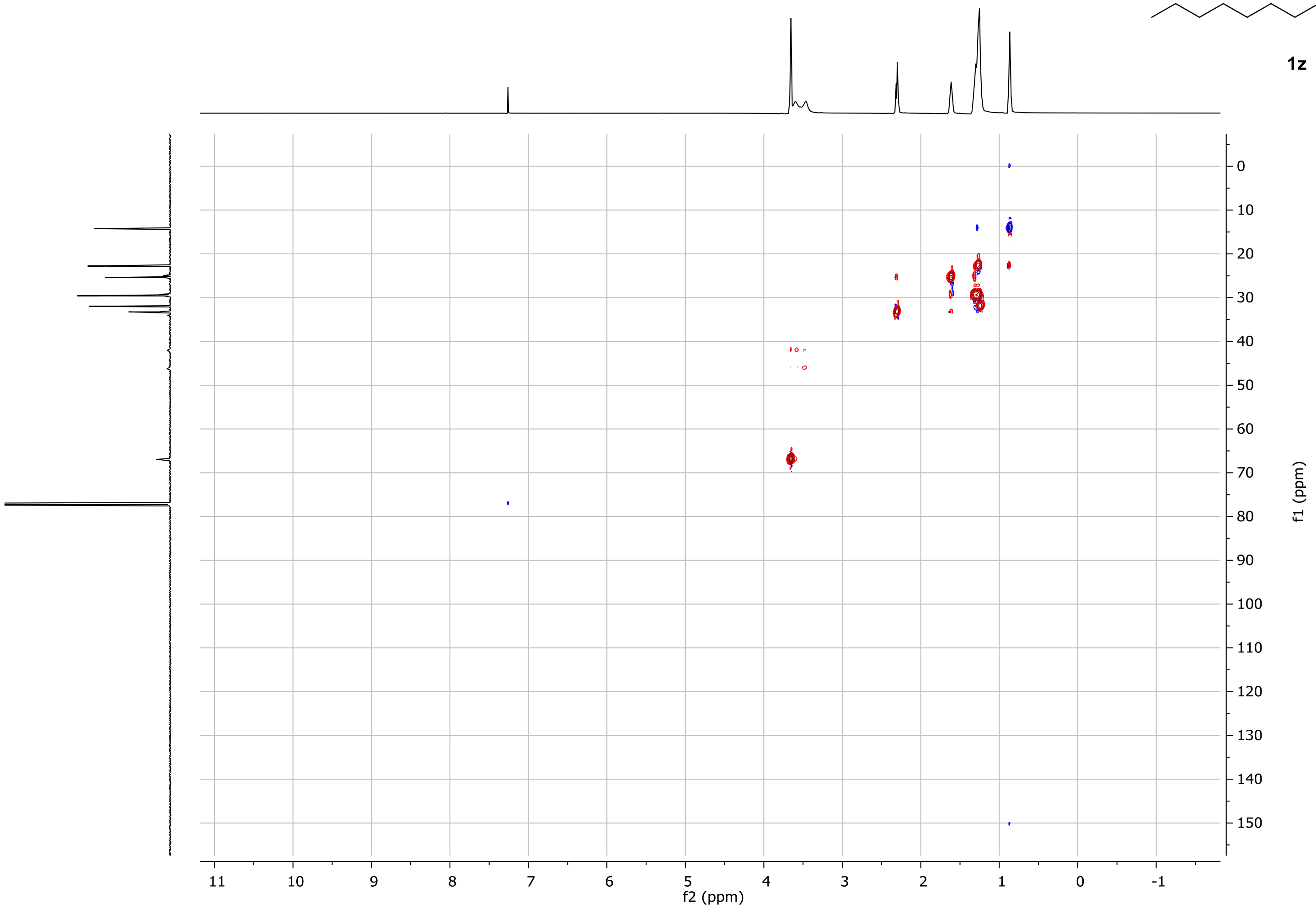

HRMS

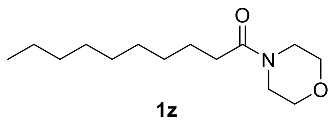
O
N
O

1z

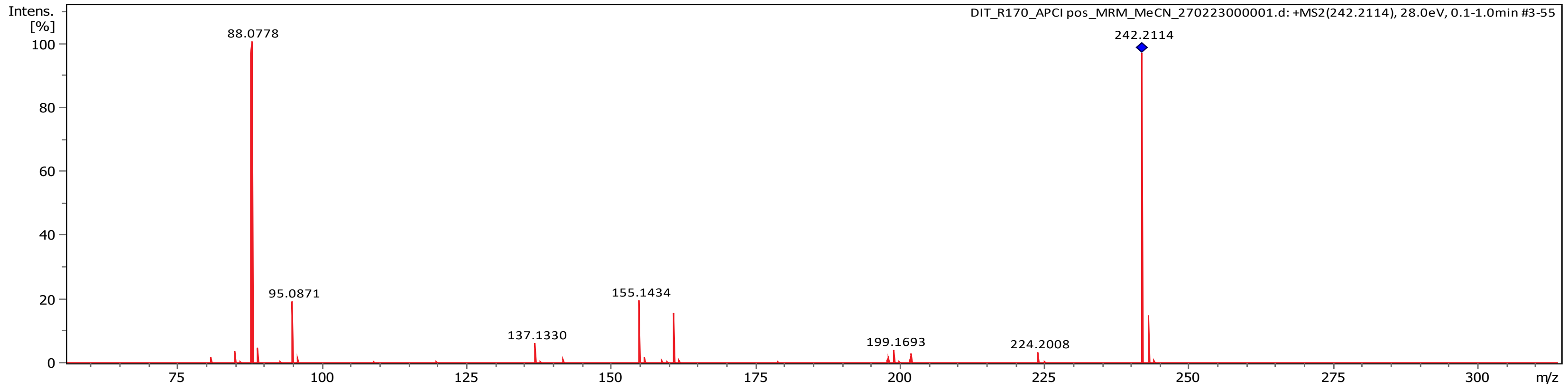
Intens. [%]
DIT_R170_APCI pos_MRM_MeCN_270223000001.d: +MS2(242.2114), 28.0eV, 0.1-1.0min #3-55
88.0778
242.2114
95.0871
155.1434
137.1330
199.1693
224.2008
m/z

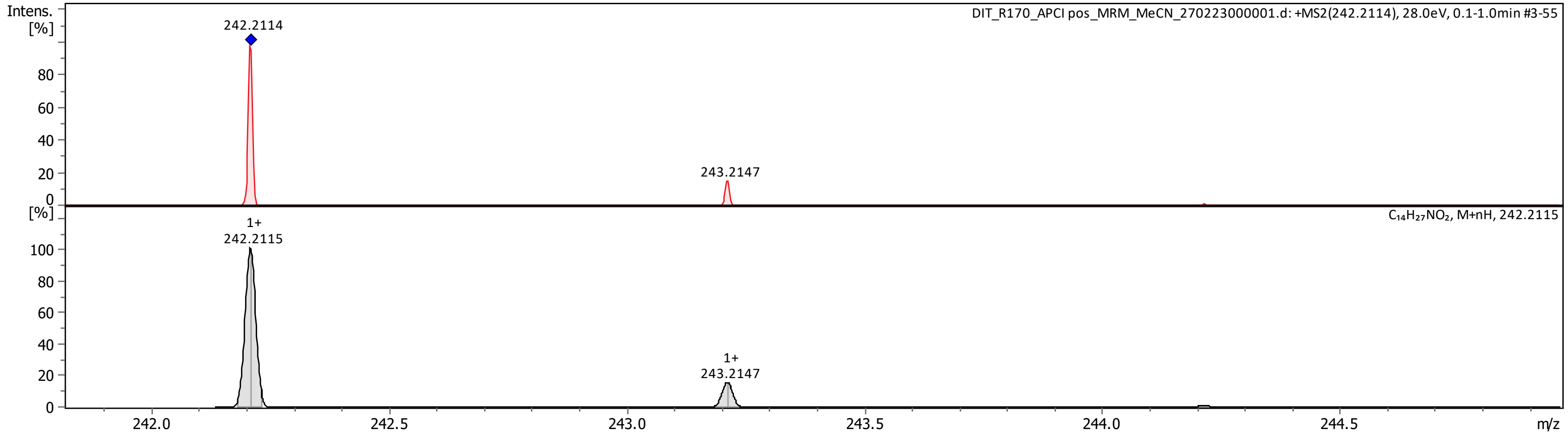
Intens. [%]
DIT_R170_APCI pos_MRM_MeCN_270223000001.d: +MS2(242.2114), 28.0eV, 0.1-1.0min #3-55
242.2114
243.2147
[%]
C14H27NO2, M+nH, 242.2115
1+
242.2115
1+
243.2147
m/z

25 Morpholino(4-(phenylethynyl)phenyl)methanone (1aa)

^{1}H NMR

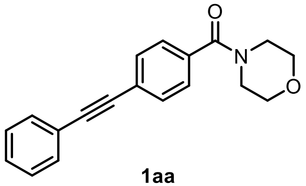

1aa

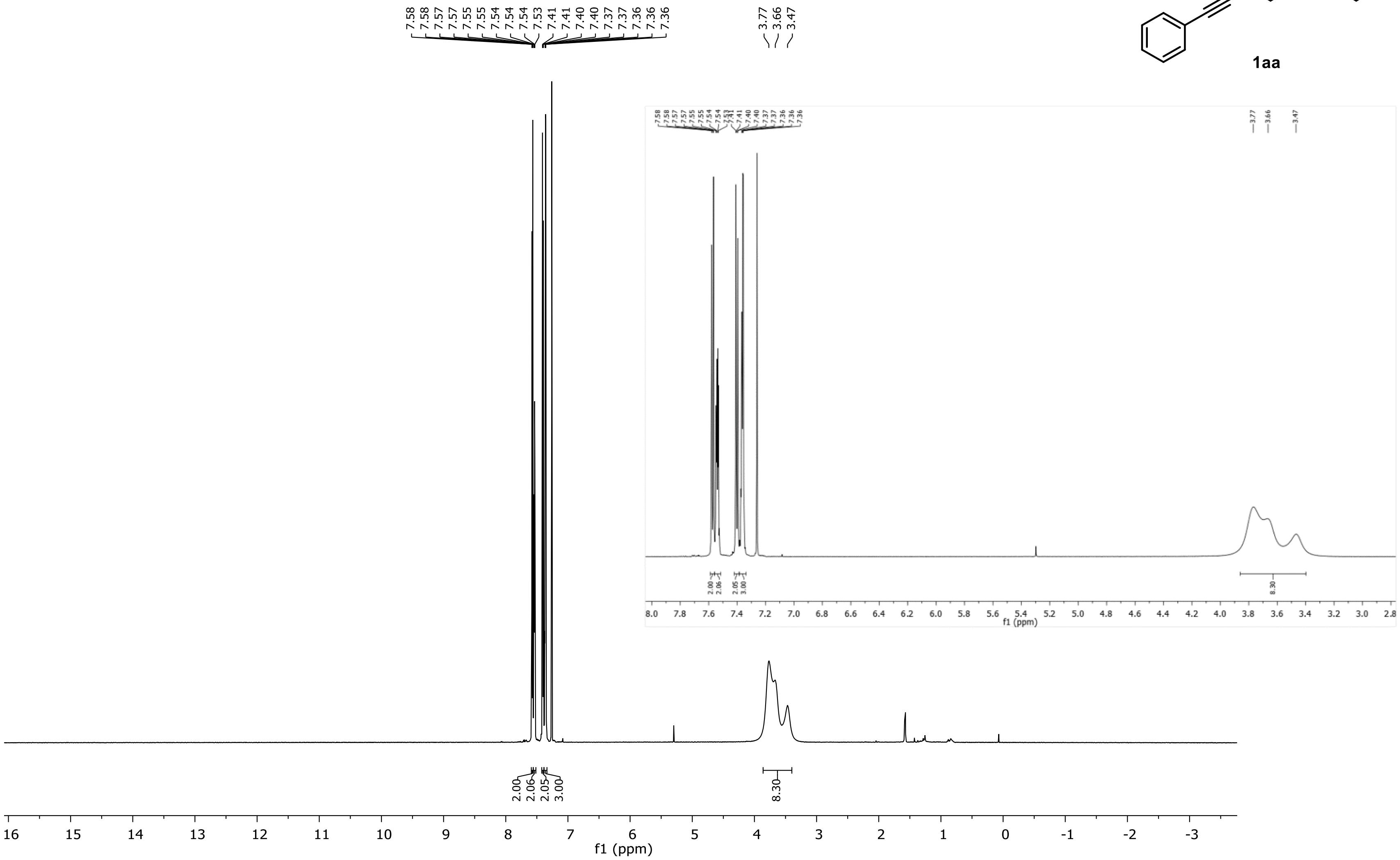

^{13}C NMR

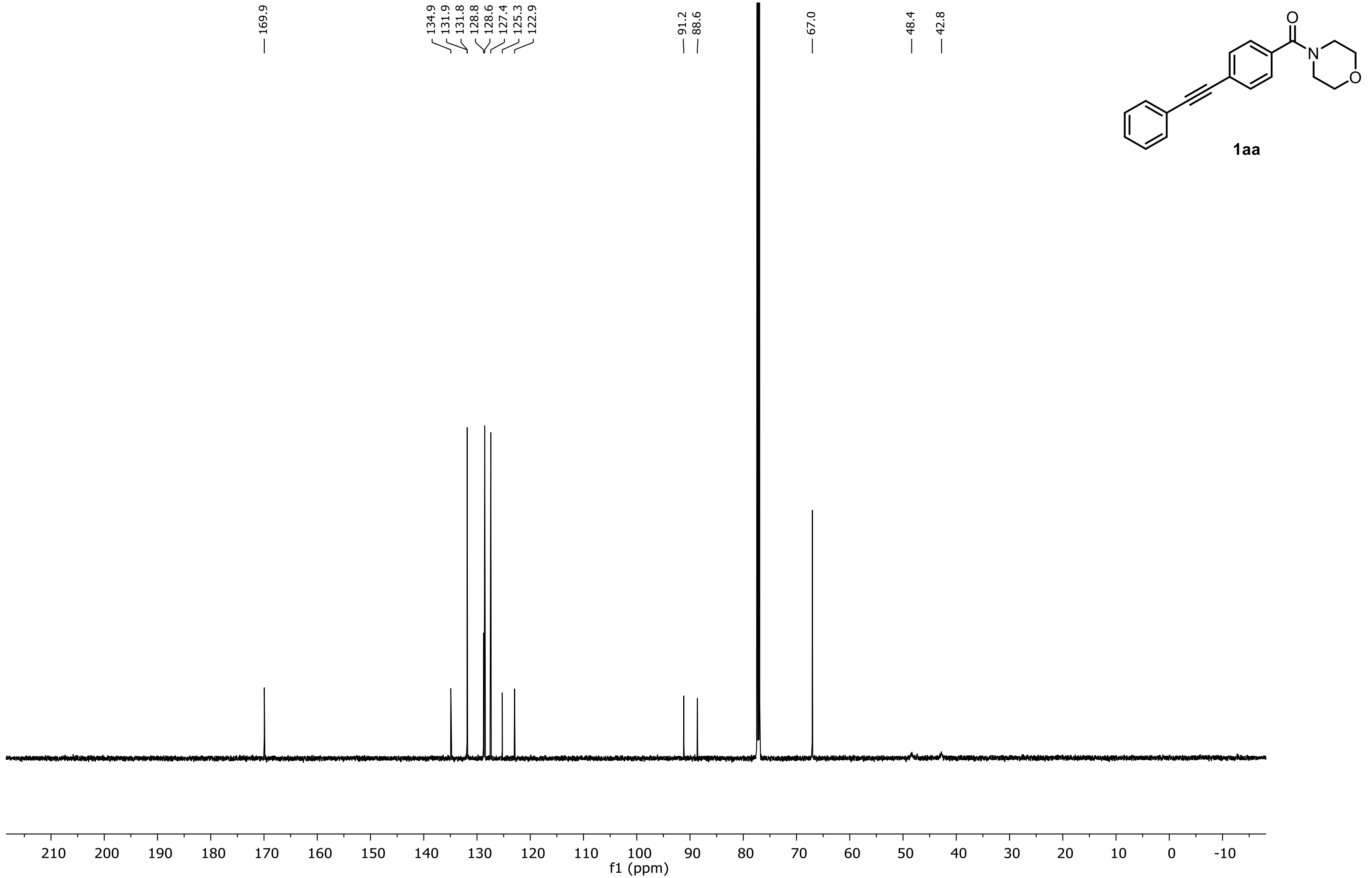
169.9
134.9
131.9
131.8
128.8
128.6
127.4
125.3
122.9
91.2
88.6
67.0
48.4
42.8
210
200
190
180
170
160
150
140
130
120
110
100
90
80
70
60
50
40
30
20
10
0
-10
f1 (ppm)
O
N
O

1aa

^{1}H, ^{1}H COSY

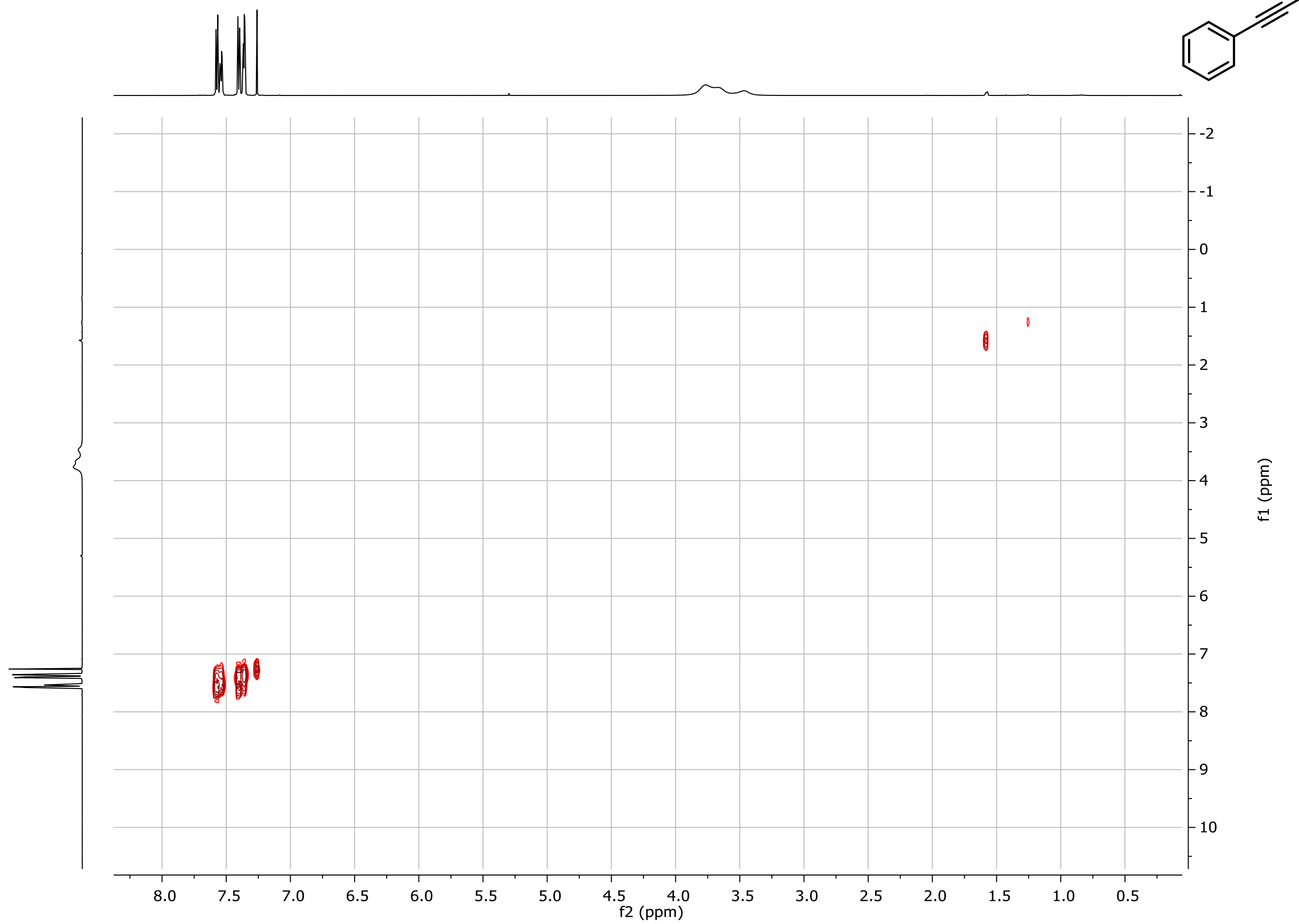

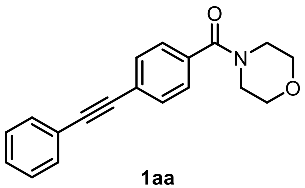

1aa

^{1}H, ^{13}C HMBC

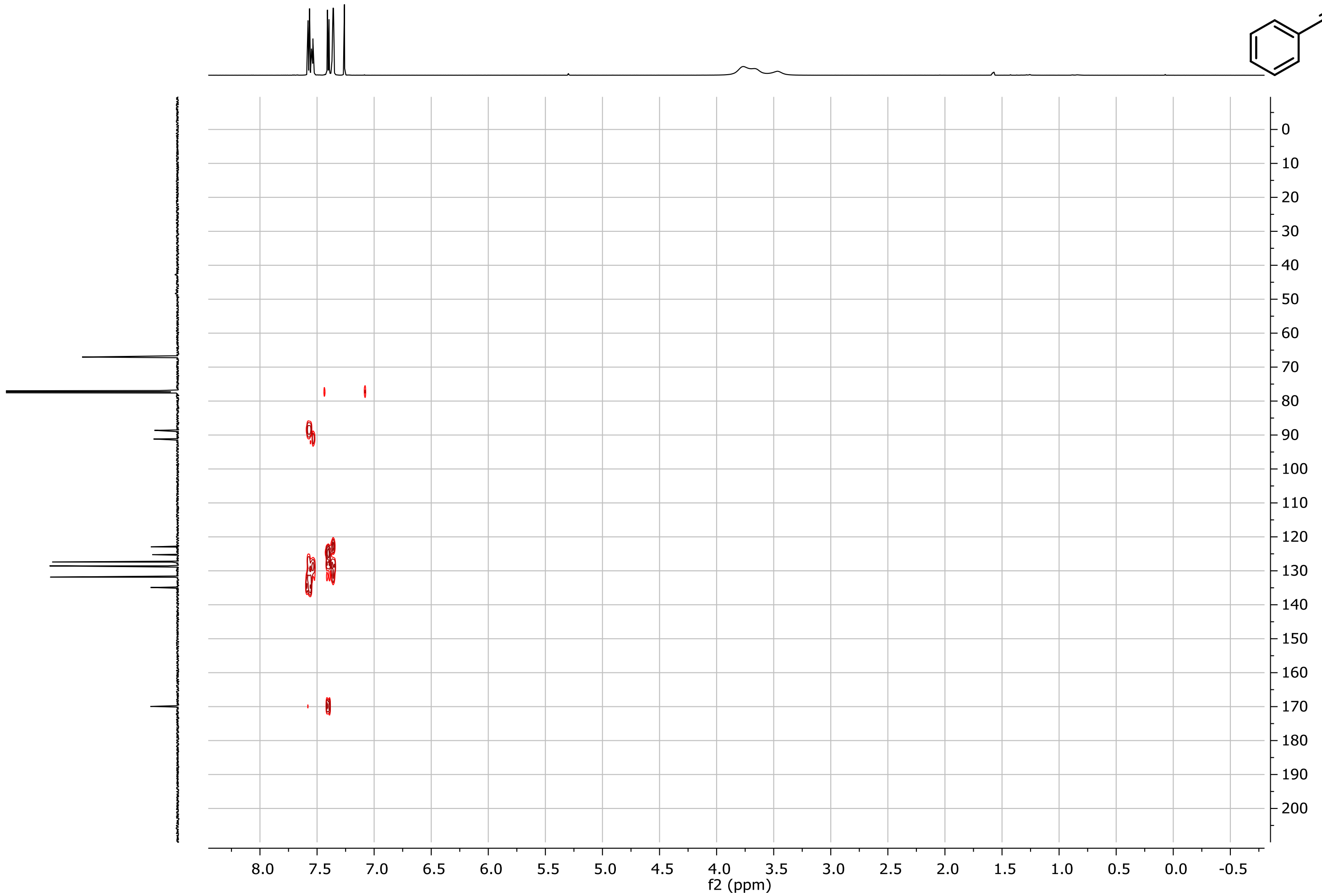

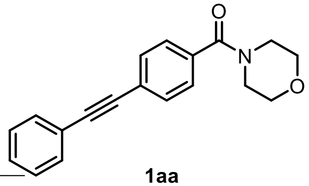

1aa

^1H, ^{13}C HSQC

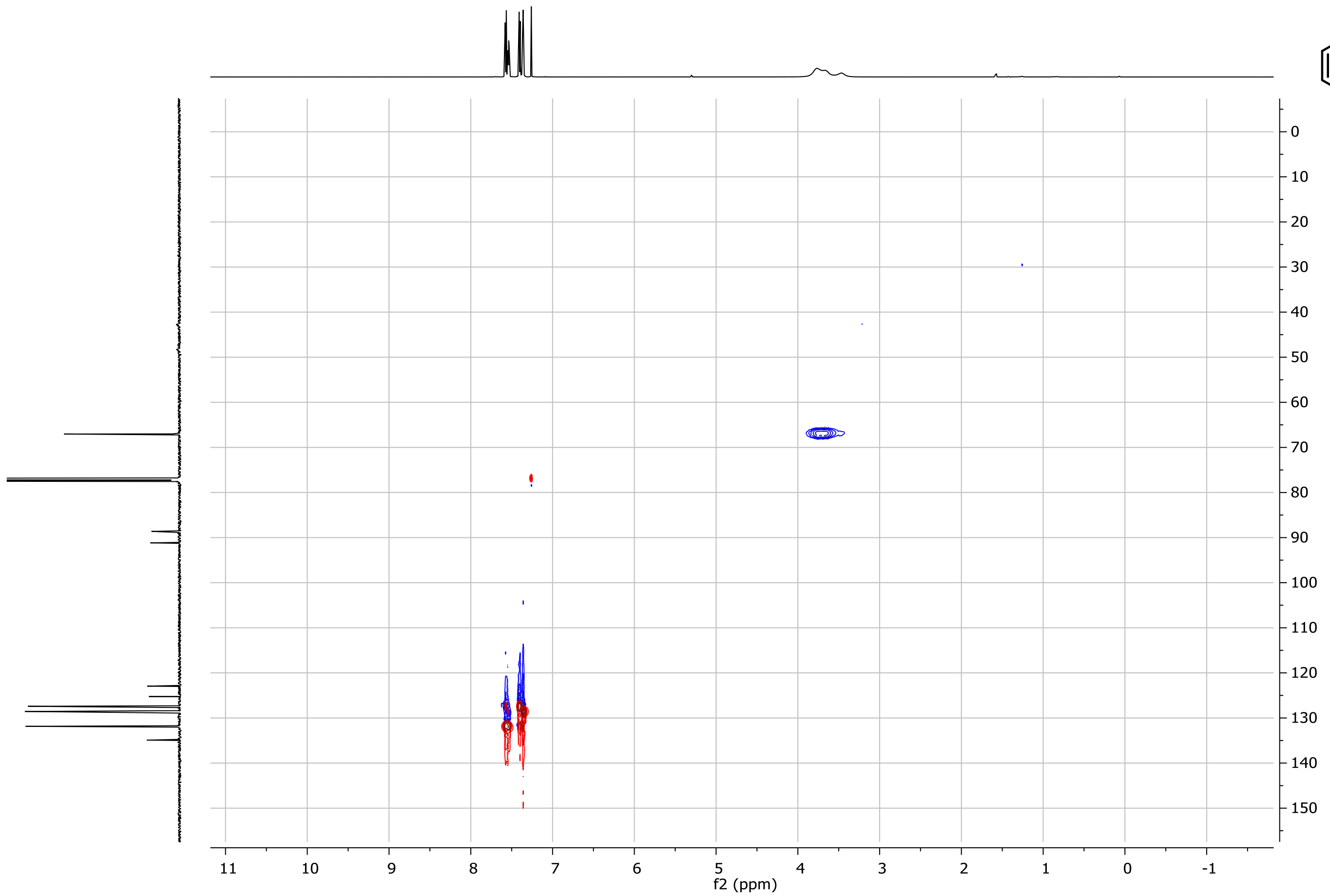

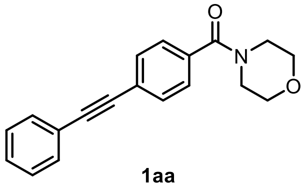

1aa

HRMS

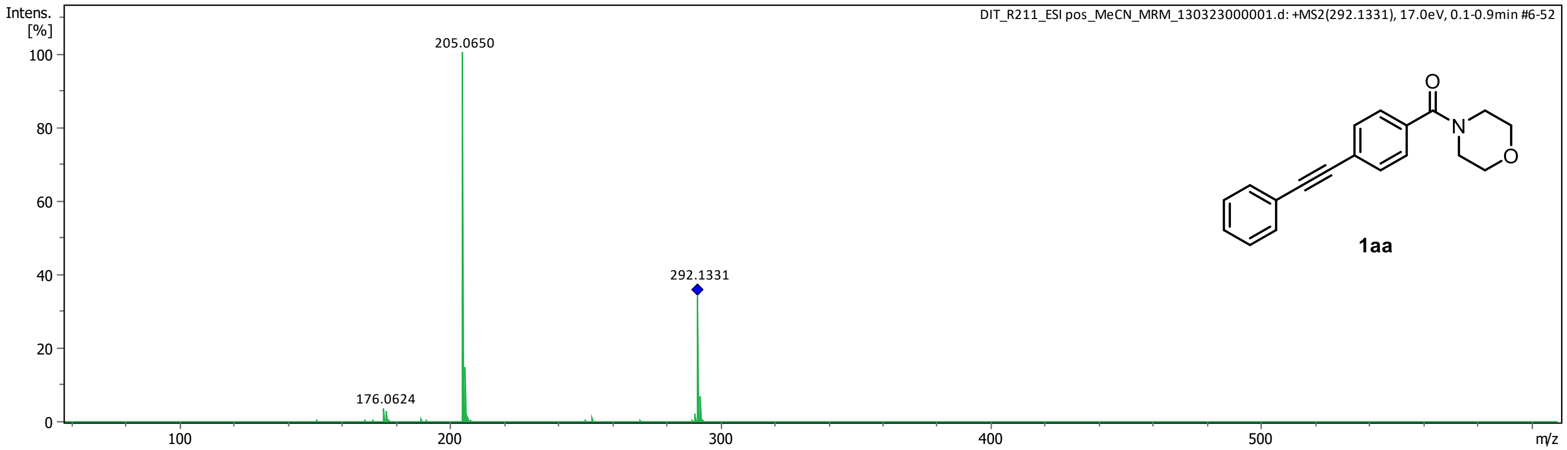

Intens. [%]
DIT_R211_ESI pos_MeCN_MRM_130323000001.d: +MS2(292.1331), 17.0eV, 0.1-0.9min #6-52
205.0650
292.1331
176.0624
1aa
m/z

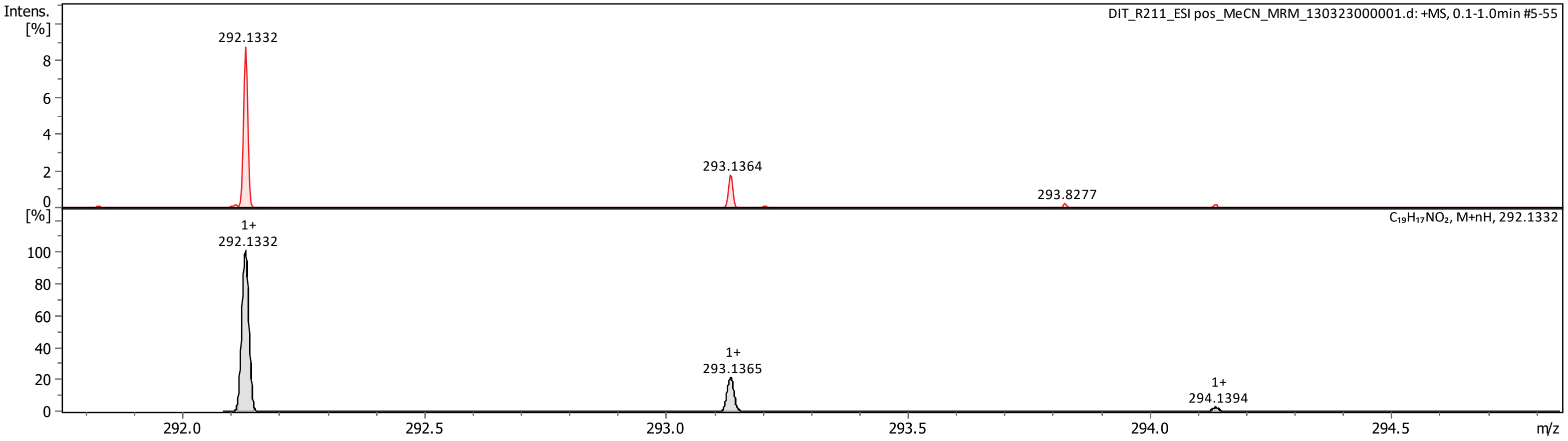

Intens. [%]
DIT_R211_ESI pos_MeCN_MRM_130323000001.d: +MS, 0.1-1.0min #5-55
292.1332
293.1364
293.8277
$C_{19}H_{17}NO_2$, M+nH, 292.1332
1+ 292.1332
1+ 293.1365
1+ 294.1394
m/z

IR

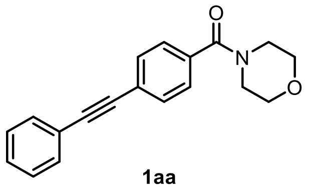

1aa

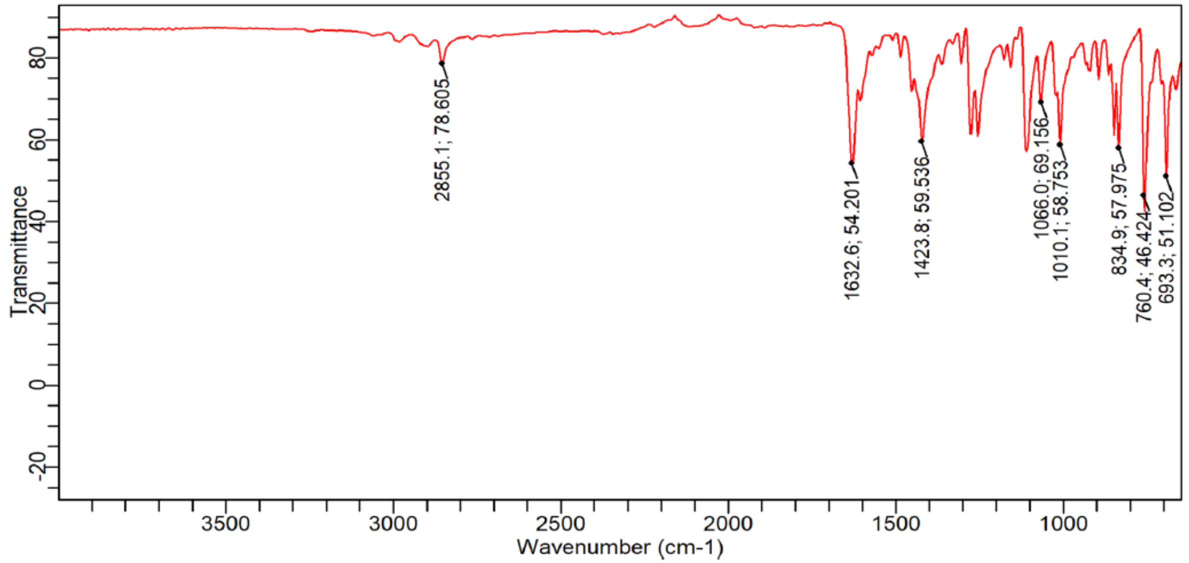

Transmittance
Wavenumber (cm-1)
2855.1; 78.605
1632.6; 54.201
1423.8; 59.536
1066.0; 69.156
1010.1; 58.753
834.9; 57.975
760.4; 46.424
693.3; 51.102

26 (4-Methylpiperazin-1-yl)(phenyl)methanone (1ab)

^{1}H NMR

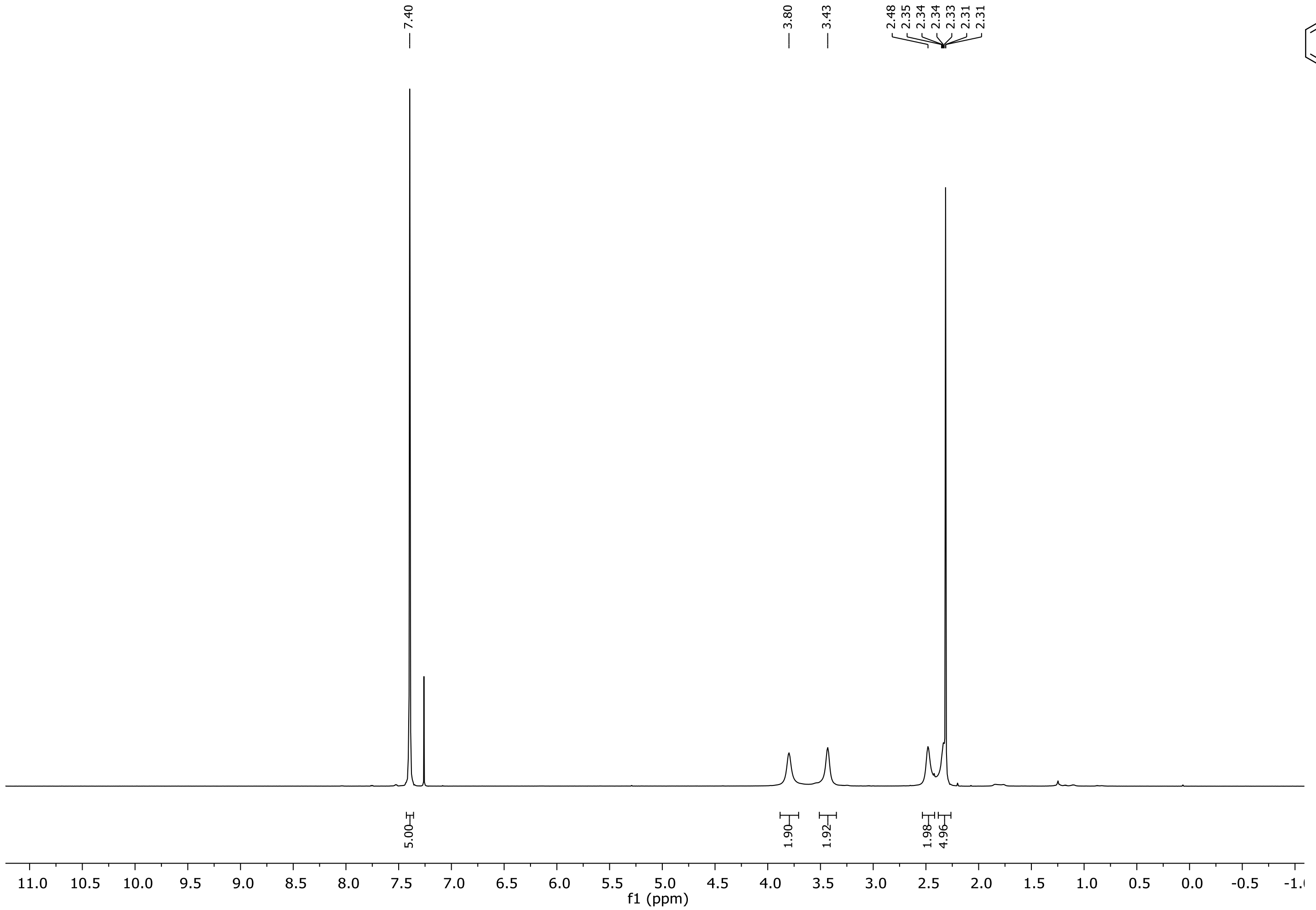

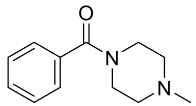

1ab

^{13}C NMR

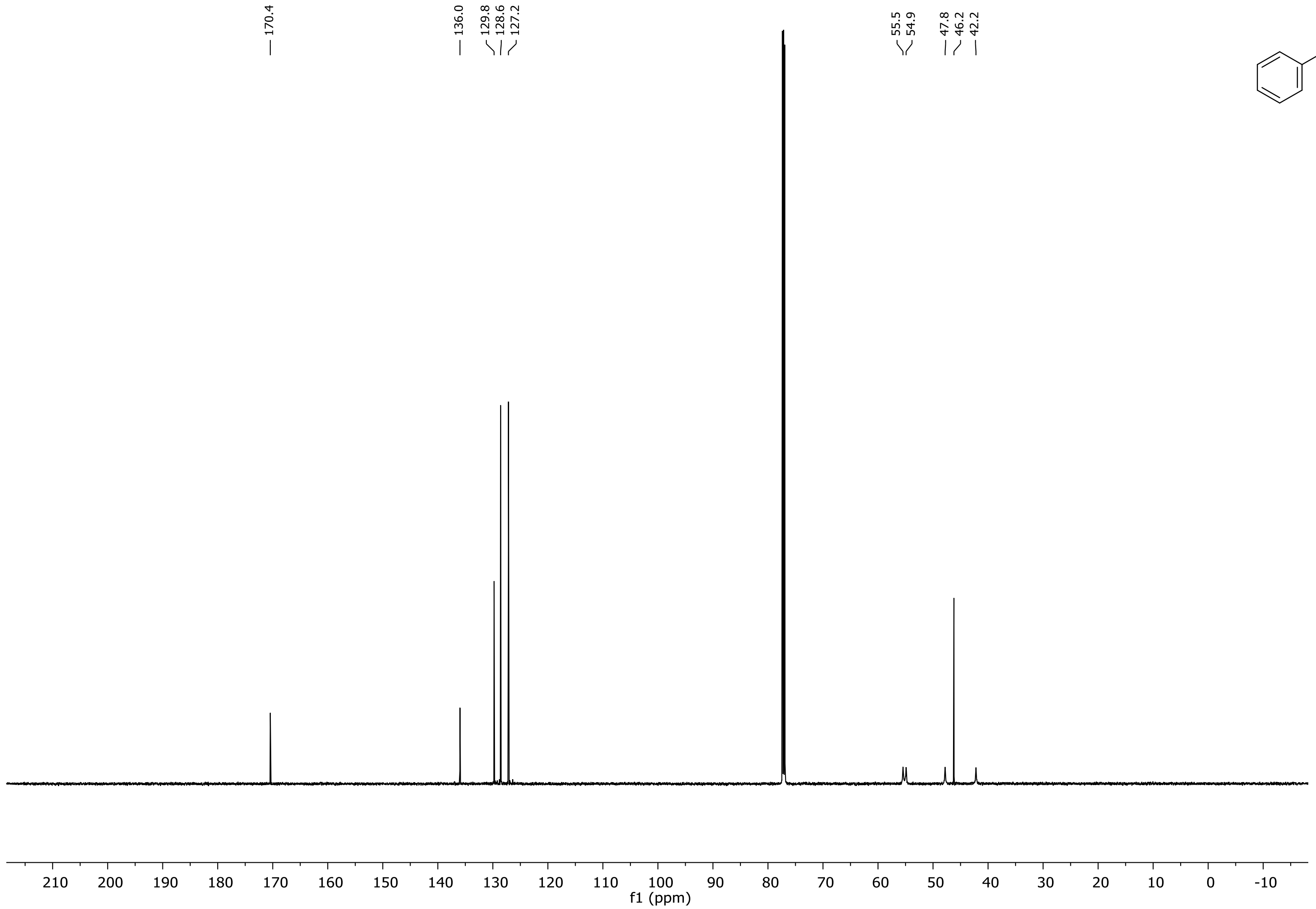
170.4
136.0
129.8
128.6
127.2
55.5
54.9
47.8
46.2
42.2
210
200
190
180
170
160
150
140
130
120
110
100
90
80
70
60
50
40
30
20
10
0
-10
f1 (ppm)

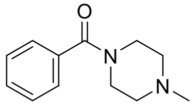
O
N
N

1ab

^1H, ^1H COSY

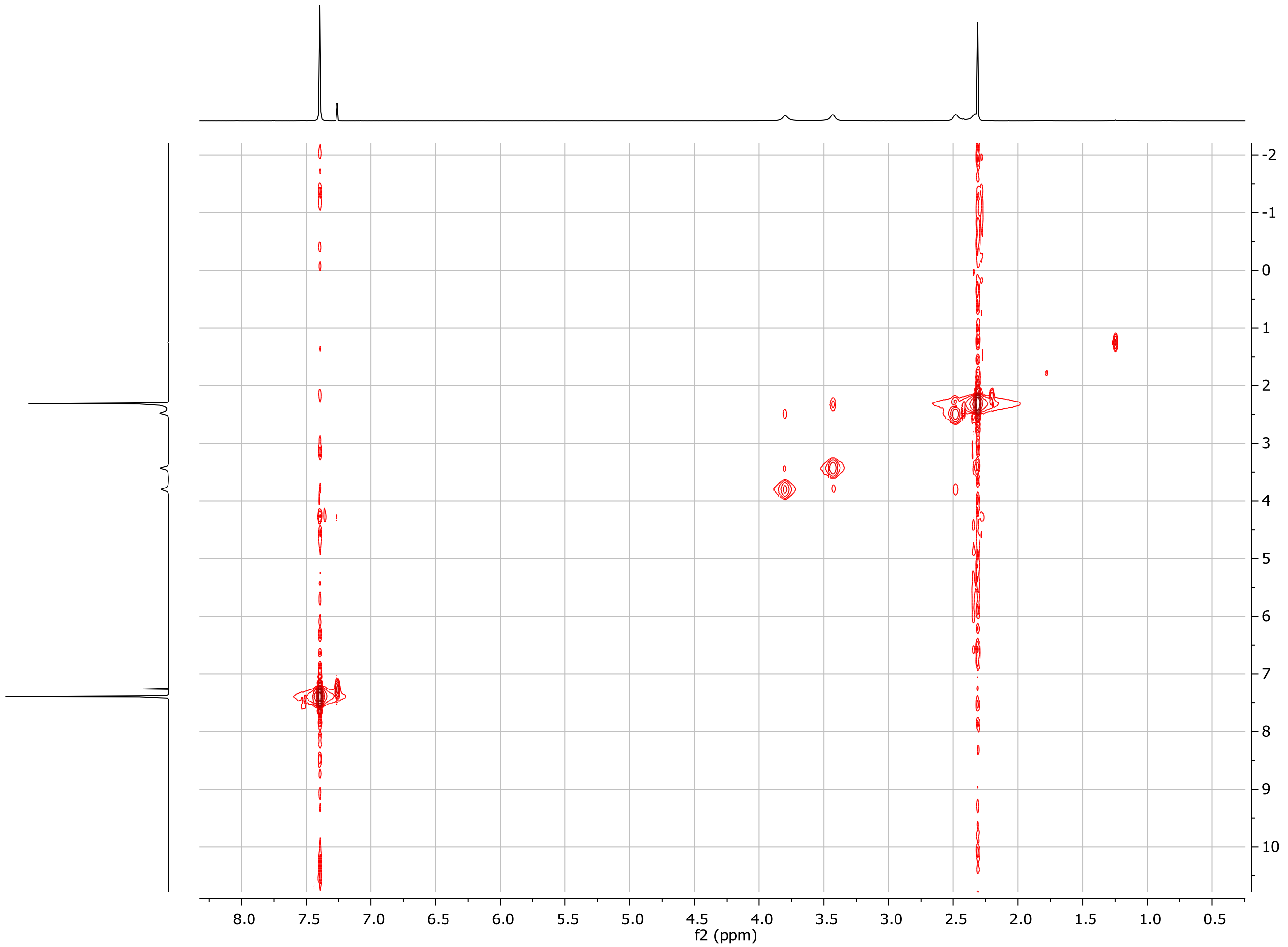

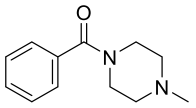

1ab

^{1}H, ^{13}C HMBC

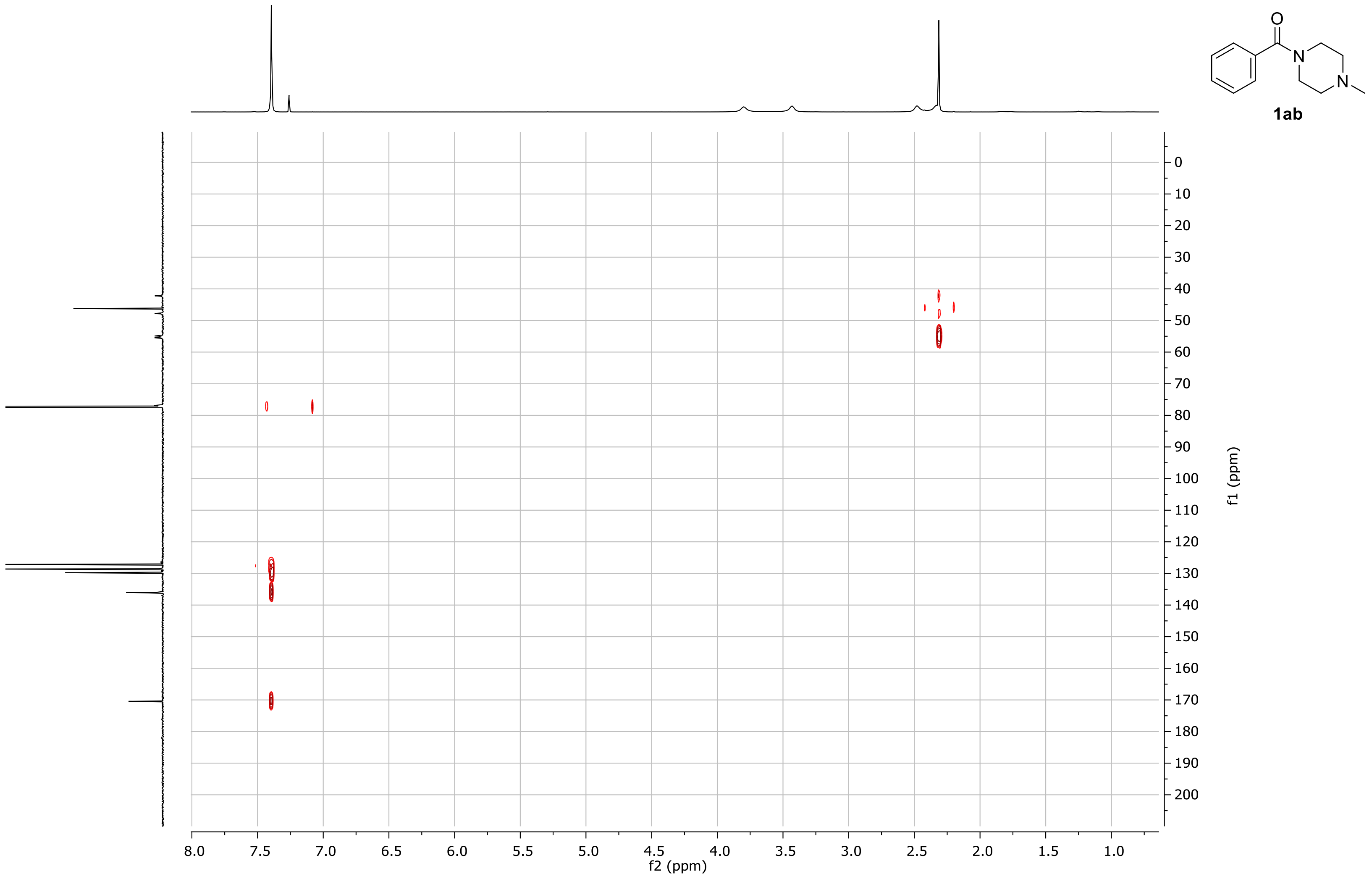

^{1}H, ^{13}C HSQC

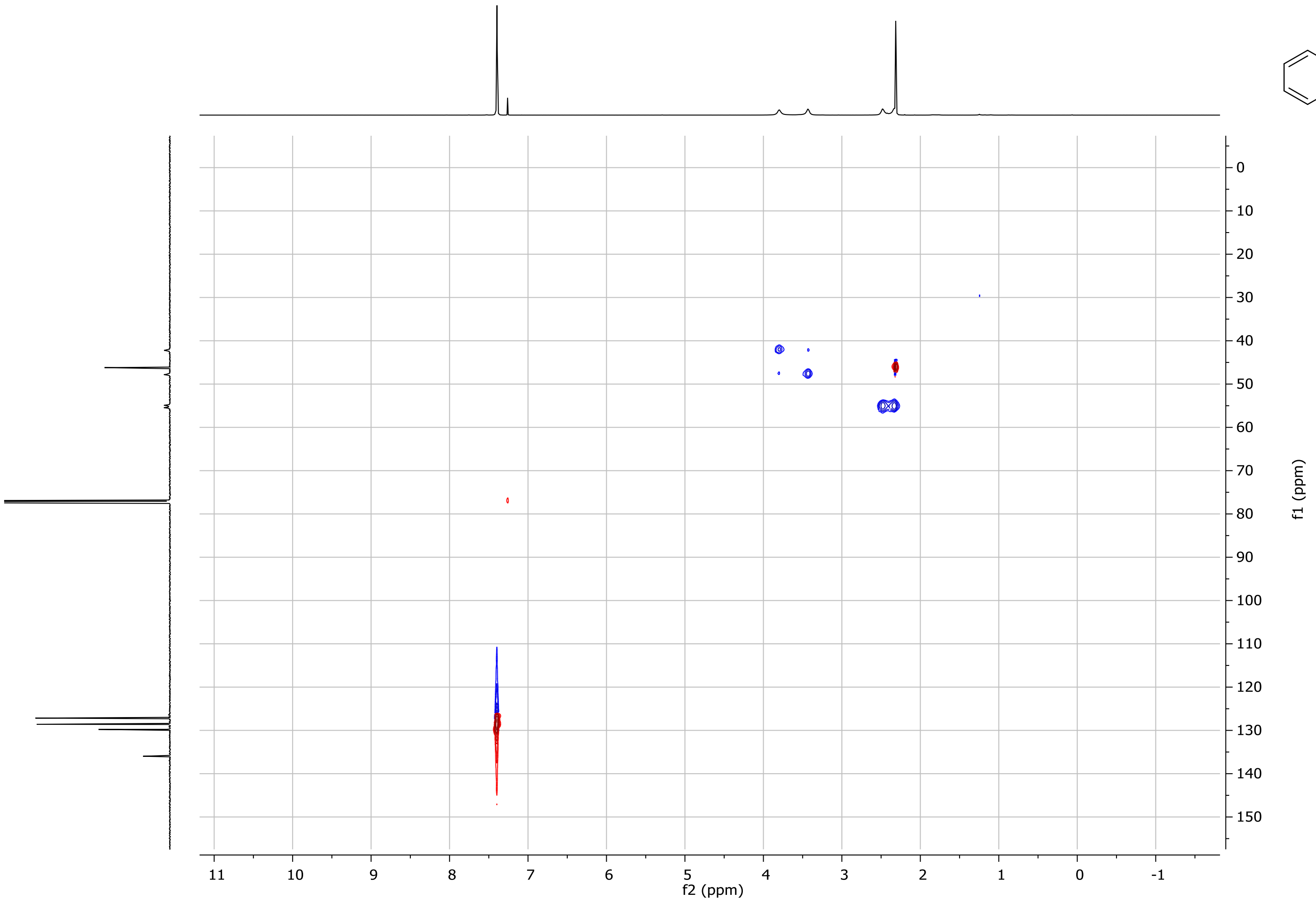

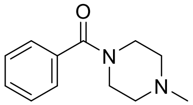

1ab

HRMS

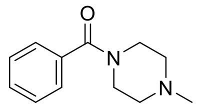

1ab

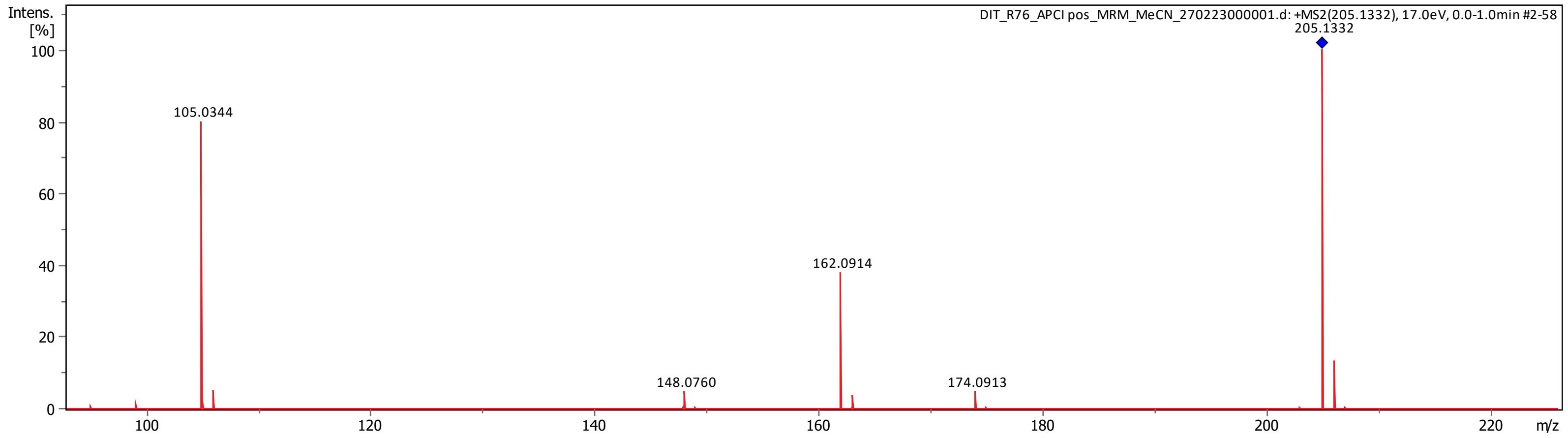

Intens. [%]
DIT_R76_APCI pos_MRM_MeCN_270223000001.d: +MS2(205.1332), 17.0eV, 0.0-1.0min #2-58
205.1332
105.0344
162.0914
148.0760
174.0913
m/z

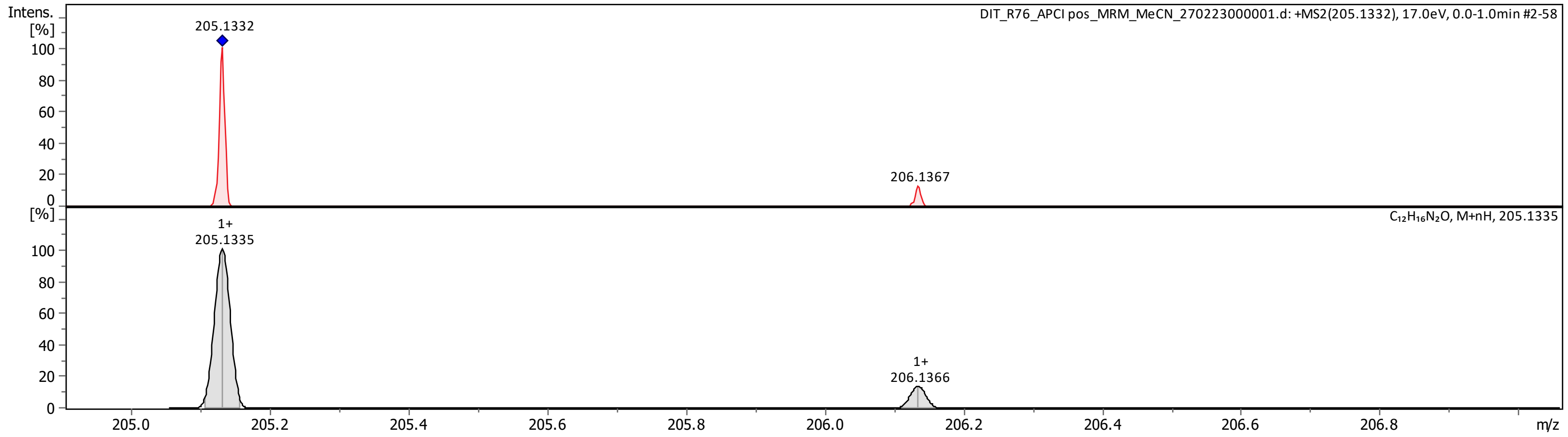

Intens. [%]
DIT_R76_APCI pos_MRM_MeCN_270223000001.d: +MS2(205.1332), 17.0eV, 0.0-1.0min #2-58
205.1332
206.1367
C12H16N2O, M+nH, 205.1335
1+
205.1335
1+
206.1366
m/z

27 (4-Ethylpiperazin-1-yl)(phenyl)methanone (1ac)

^{1}H NMR

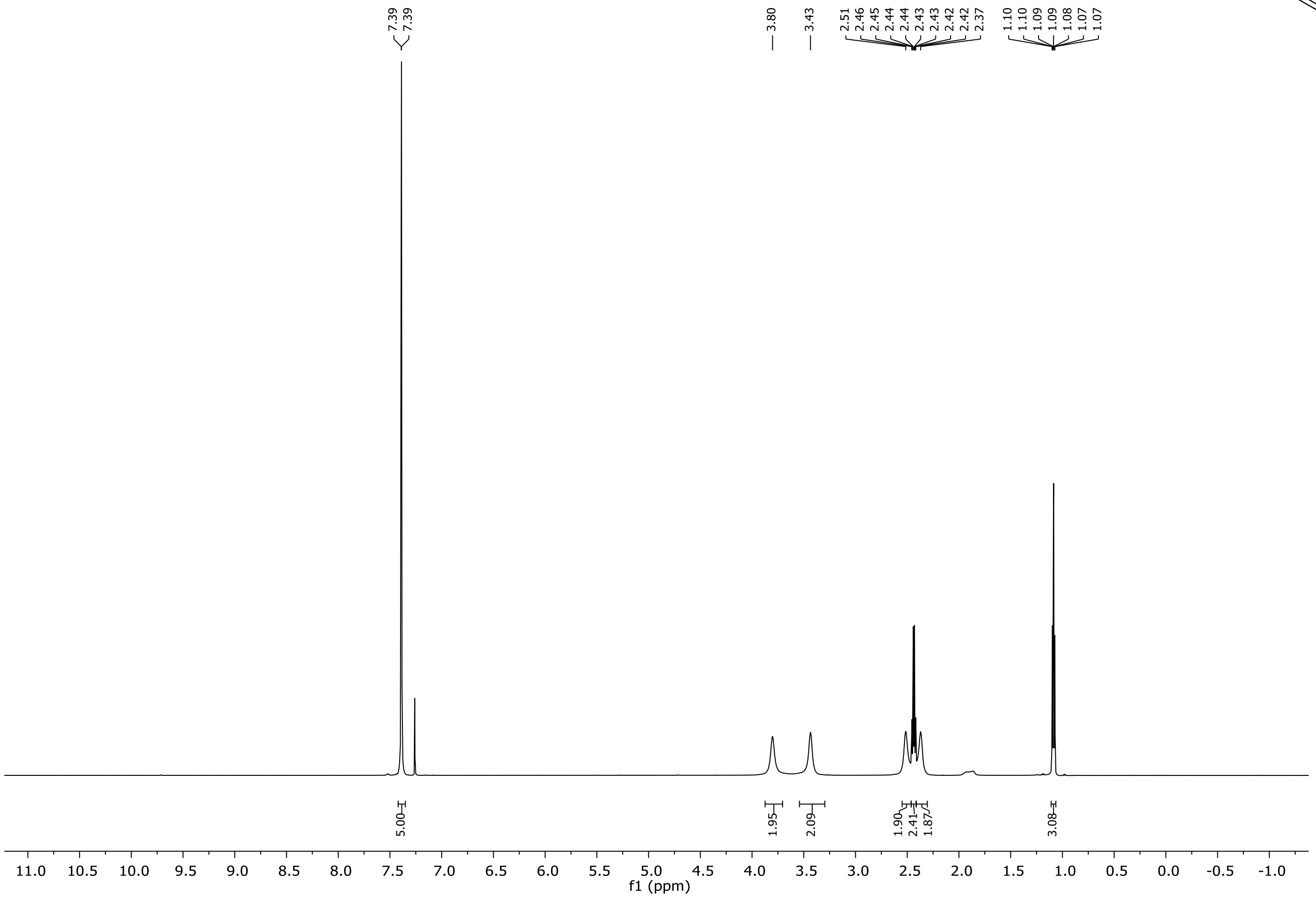

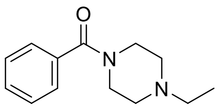

1ac

^{13}C NMR

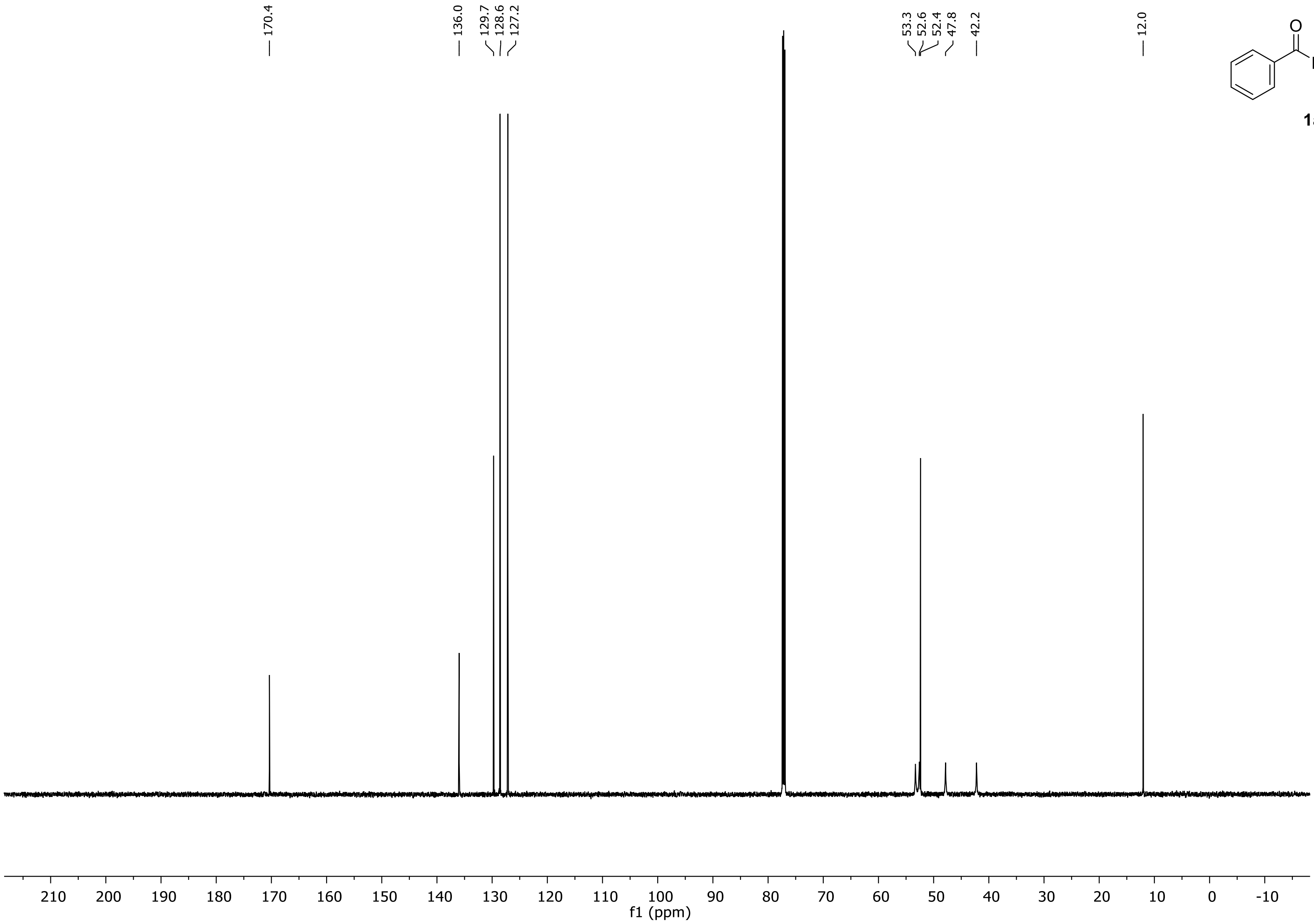
170.4
136.0
129.7
128.6
127.2
53.3
52.6
52.4
47.8
42.2
12.0
210 200 190 180 170 160 150 140 130 120 110 100 90 80 70 60 50 40 30 20 10 0 -10
f1 (ppm)

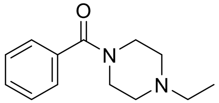
O
N
N

1ac

^{1}H, ^{1}H COSY

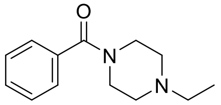

1ac

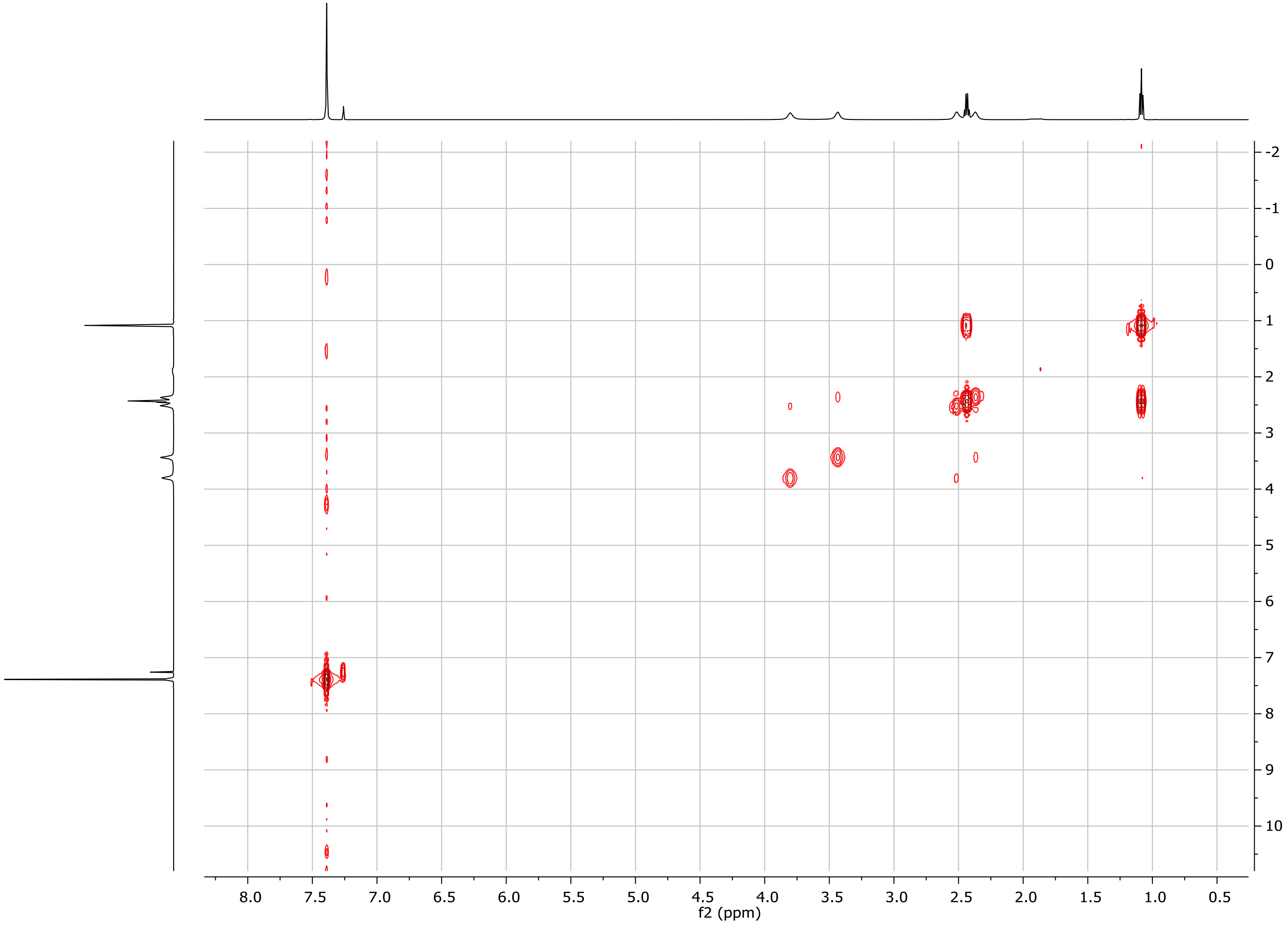

^{1}H, ^{13}C HMBC

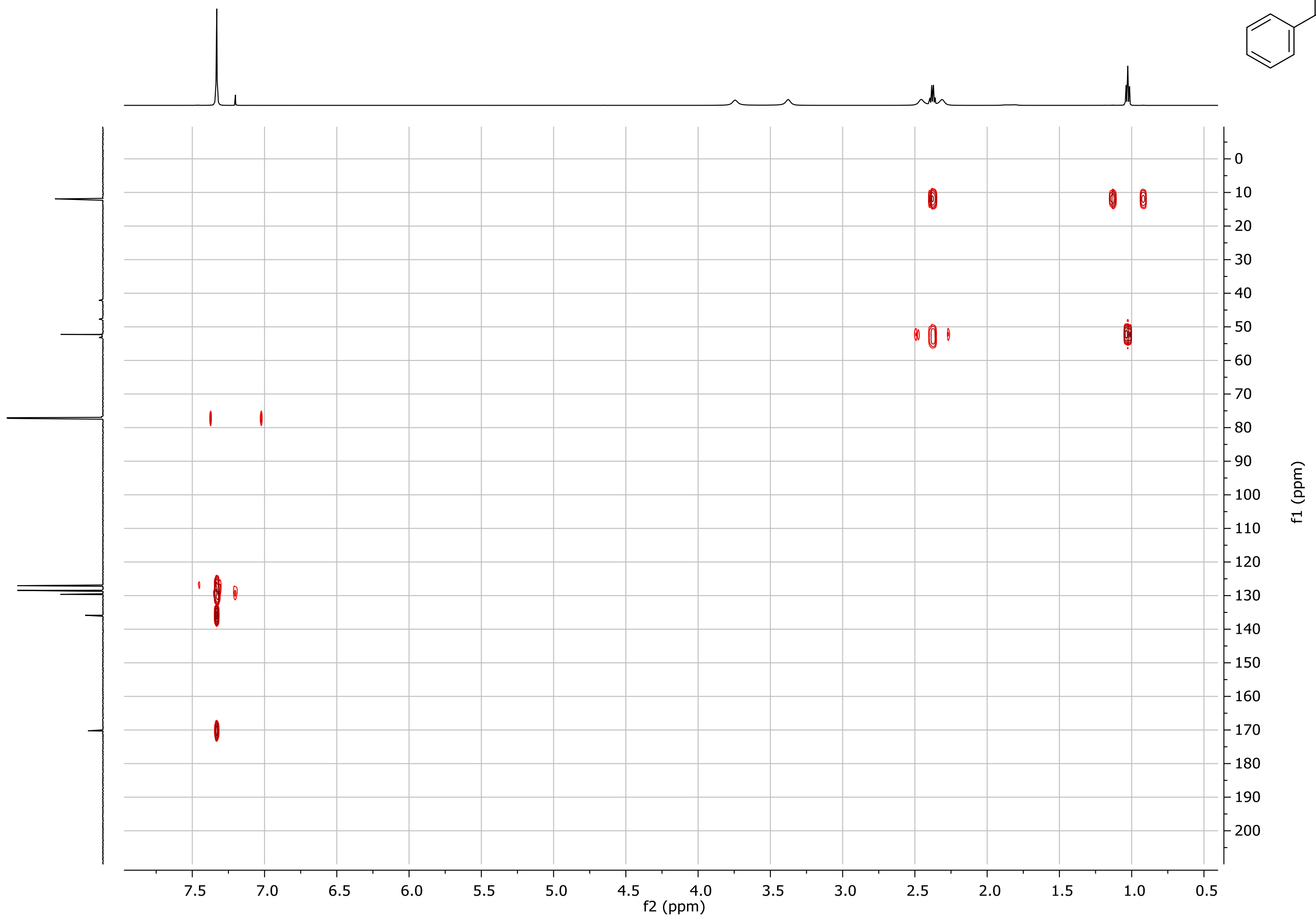

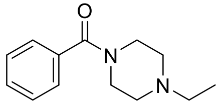

1ac

^{1}H, ^{13}C HSQC

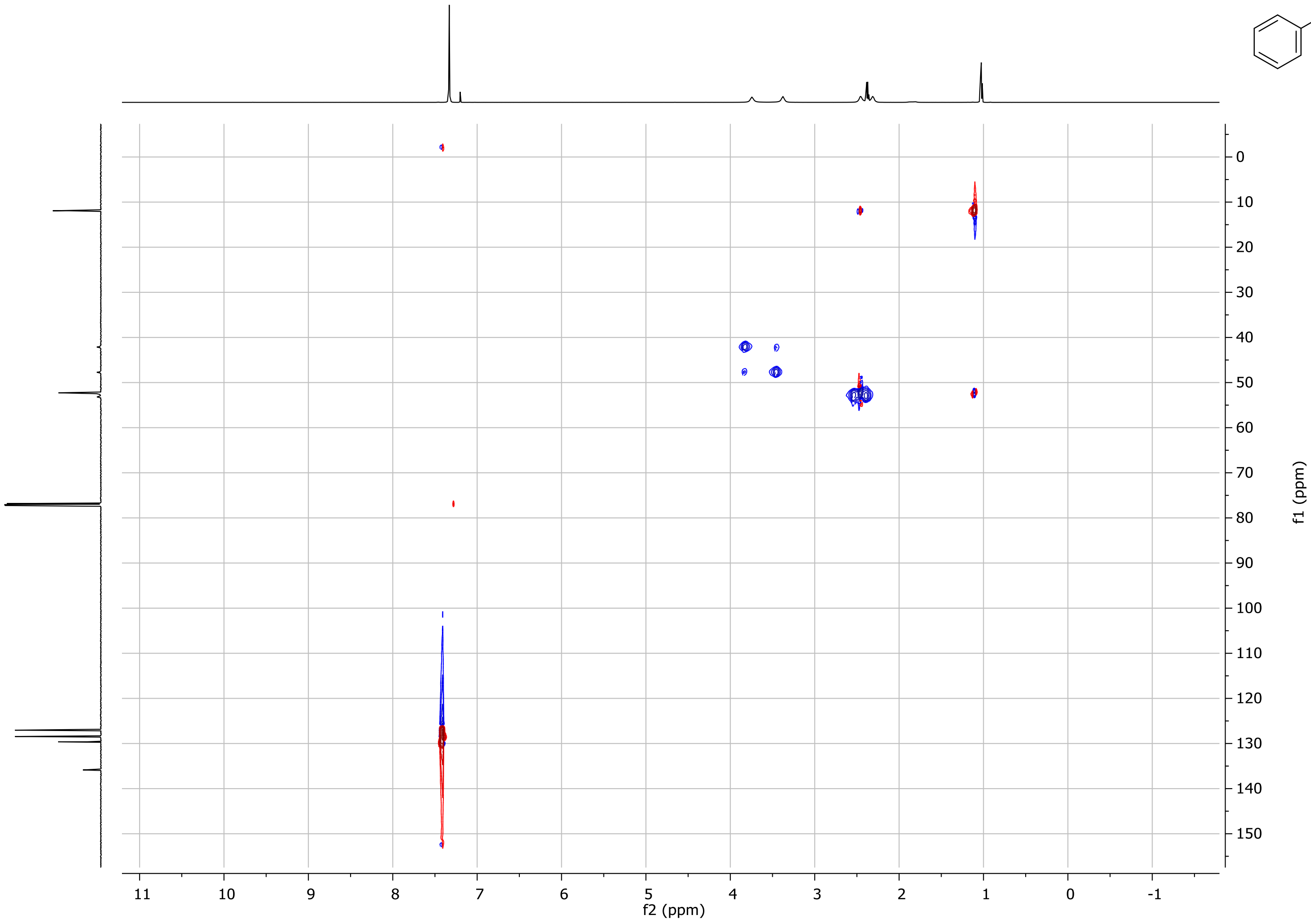

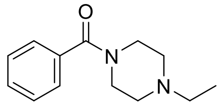

1ac

HRMS

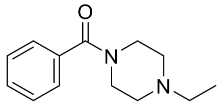
O
N
N

1ac

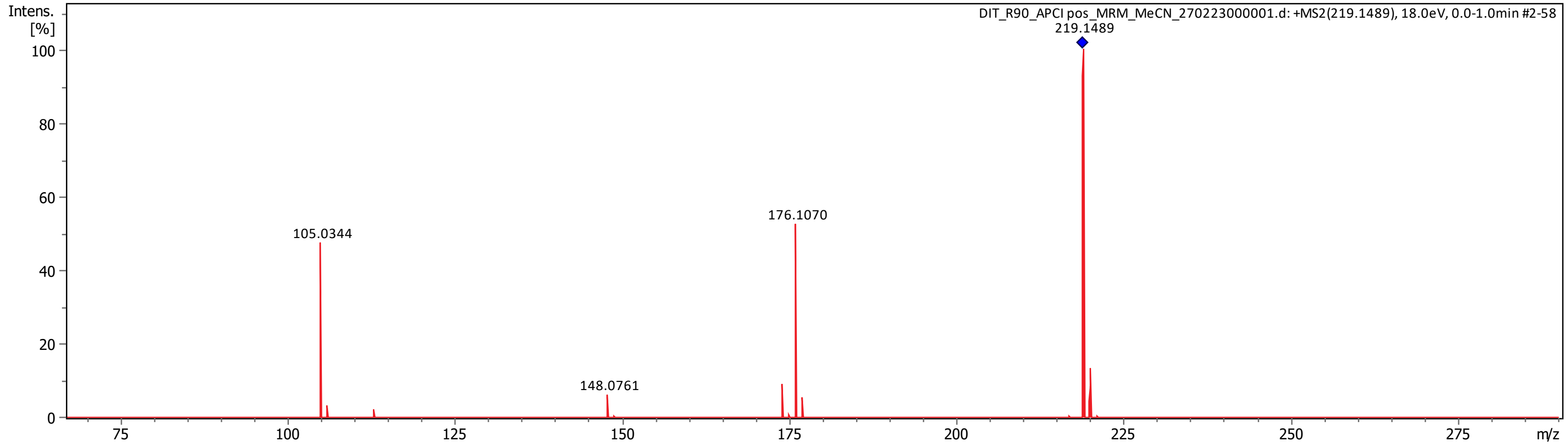
Intens.
[%]
DIT_R90_APCI pos_MRM_MeCN_270223000001.d: +MS2(219.1489), 18.0eV, 0.0-1.0min #2-58
219.1489
176.1070
105.0344
148.0761
m/z

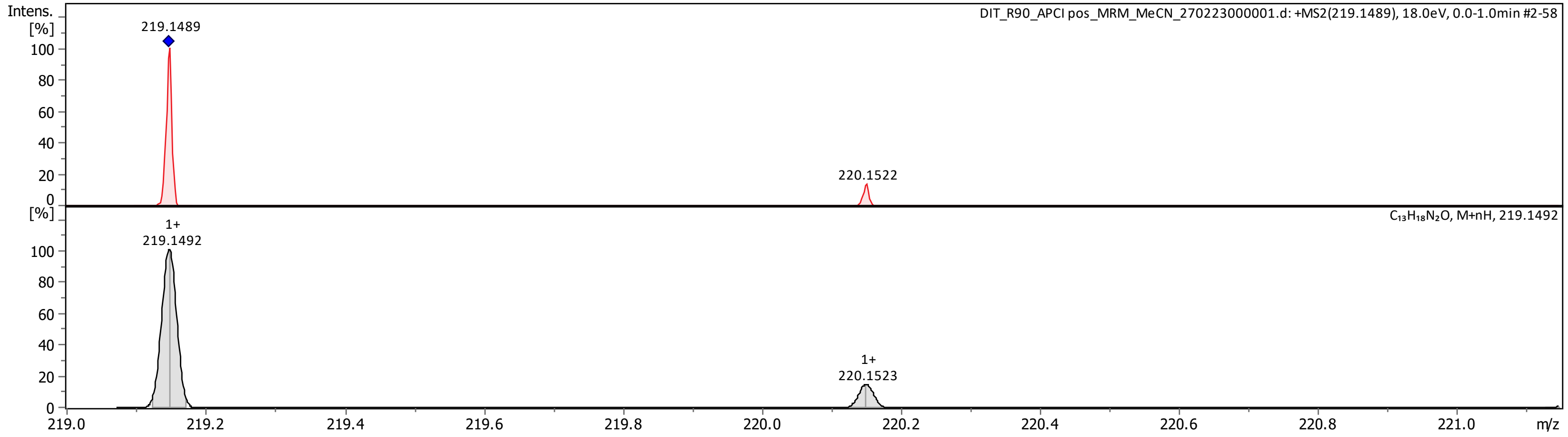
Intens.
[%]
DIT_R90_APCI pos_MRM_MeCN_270223000001.d: +MS2(219.1489), 18.0eV, 0.0-1.0min #2-58
219.1489
220.1522
$C_{13}H_{18}N_2O$, M+nH, 219.1492
1+
219.1492
1+
220.1523
m/z

IR

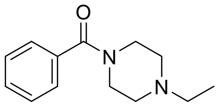

1ac

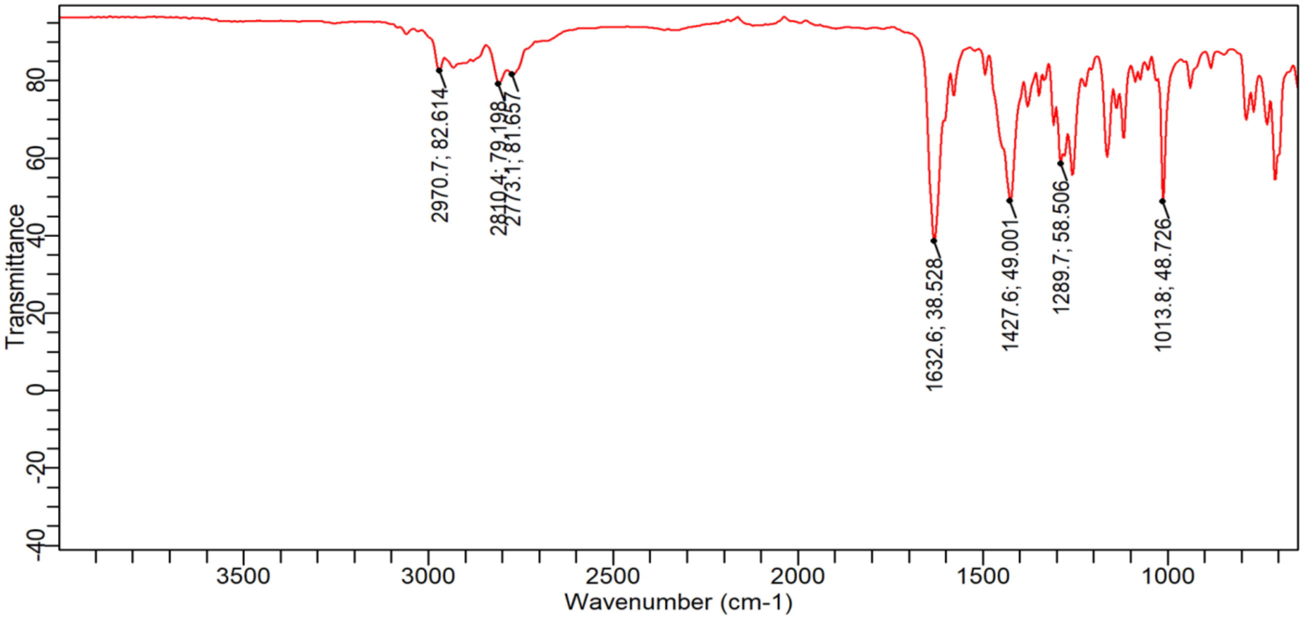
Transmittance
Wavenumber (cm-1)
2970.7; 82.614
2810.4; 79.198
2773.1; 81.657
1632.6; 38.528
1427.6; 49.001
1289.7; 58.506
1013.8; 48.726

28 (4-Isopropylpiperazin-1-yl)(phenyl)methanone (1ad)

^{1}H NMR

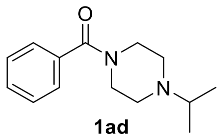

1ad

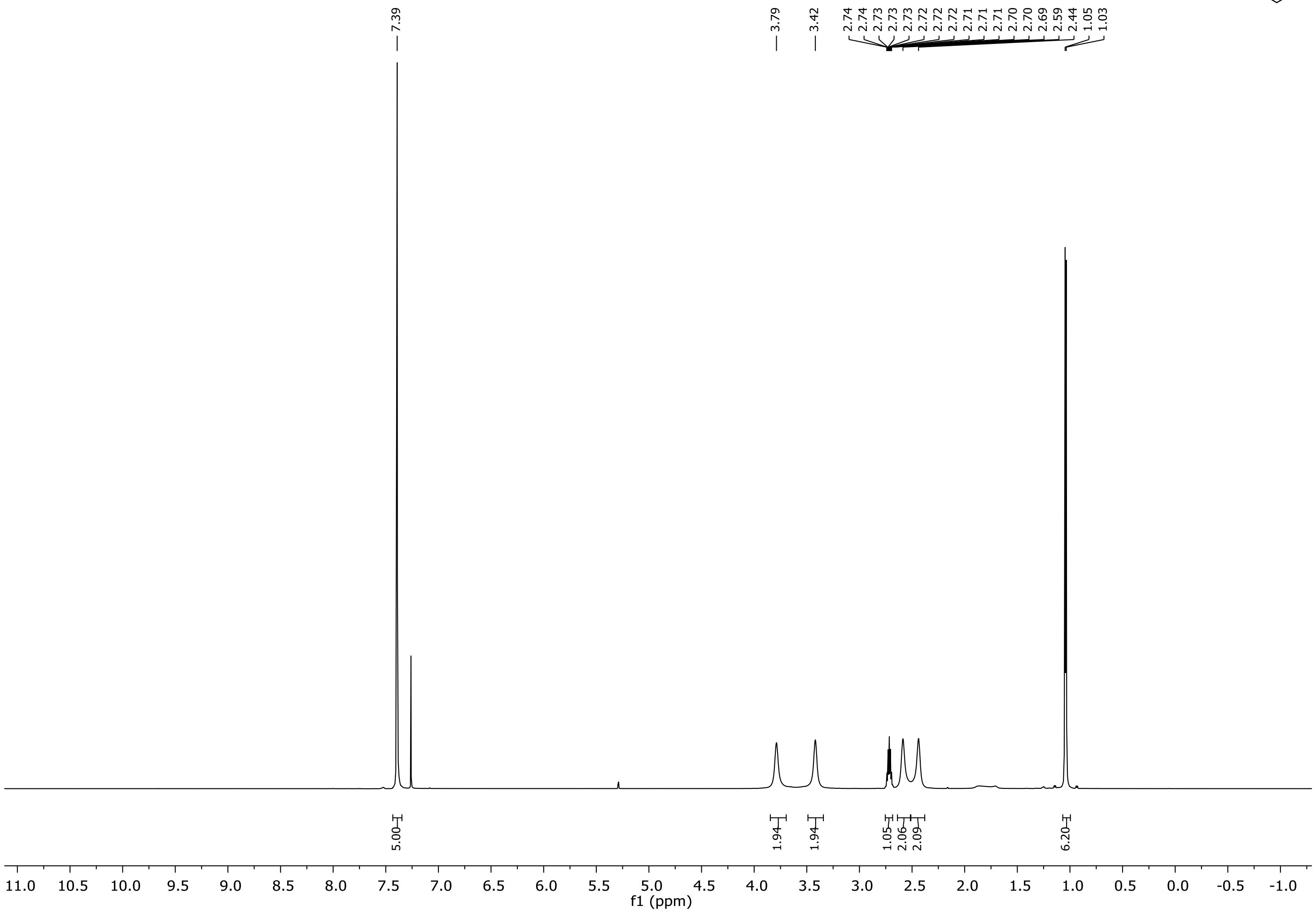

^{13}C NMR

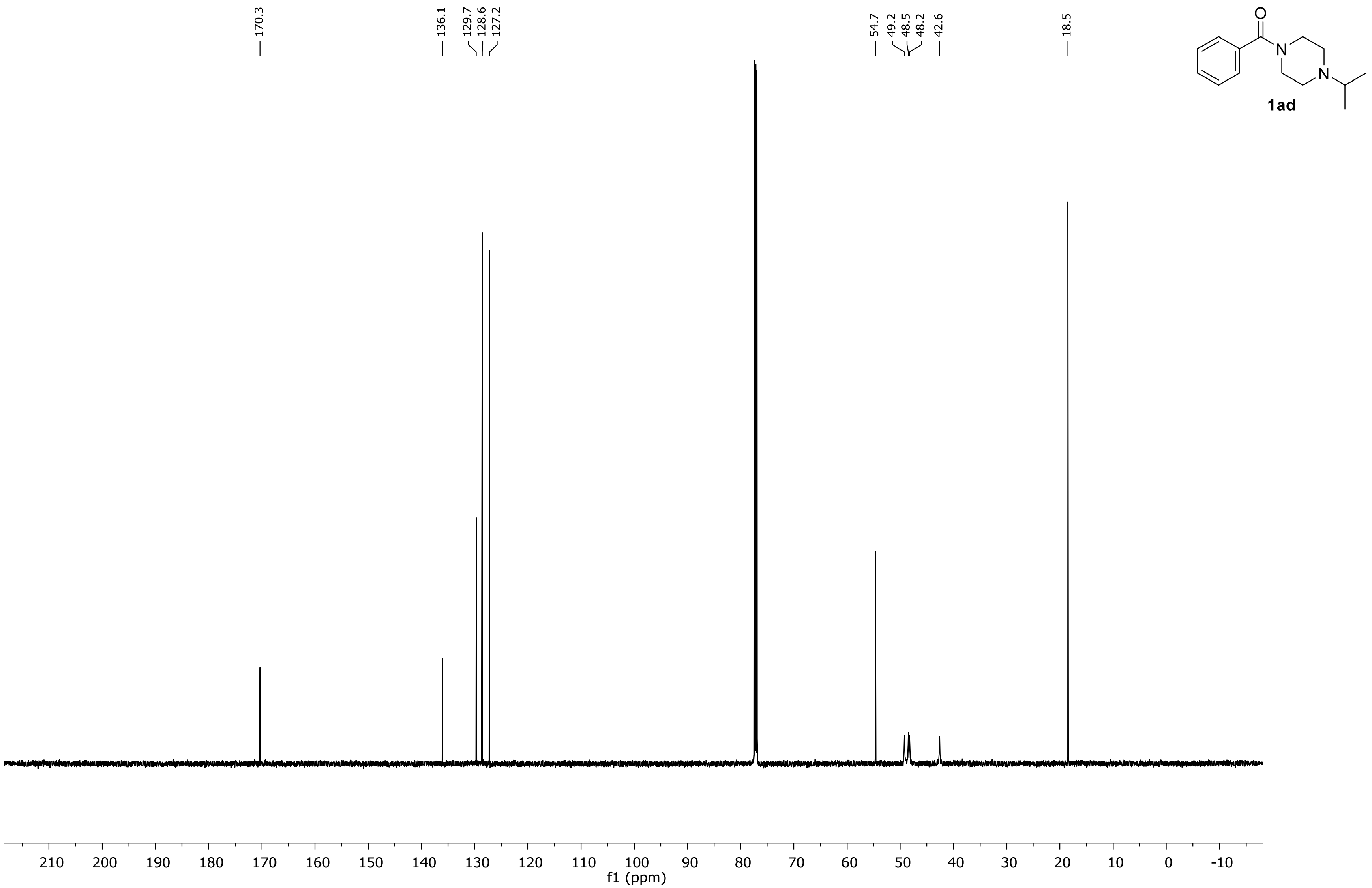
170.3
136.1
129.7
128.6
127.2
54.7
49.2
48.5
48.2
42.6
18.5
O
N
N
210 200 190 180 170 160 150 140 130 120 110 100 90 80 70 60 50 40 30 20 10 0 -10
f1 (ppm)

1ad

^{1}H, ^{1}H COSY

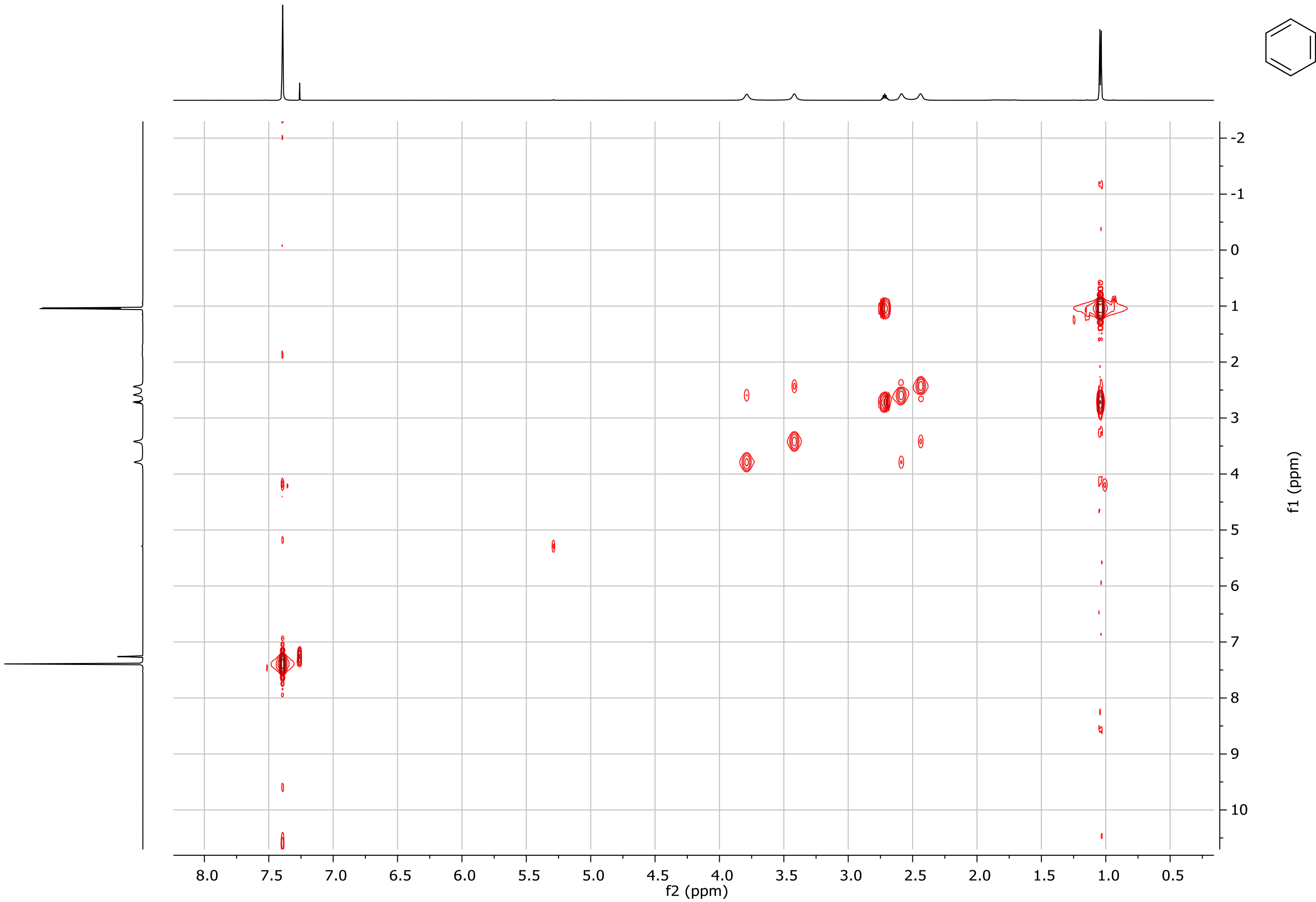

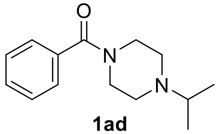

1ad

^{1}H, ^{13}C HMBC

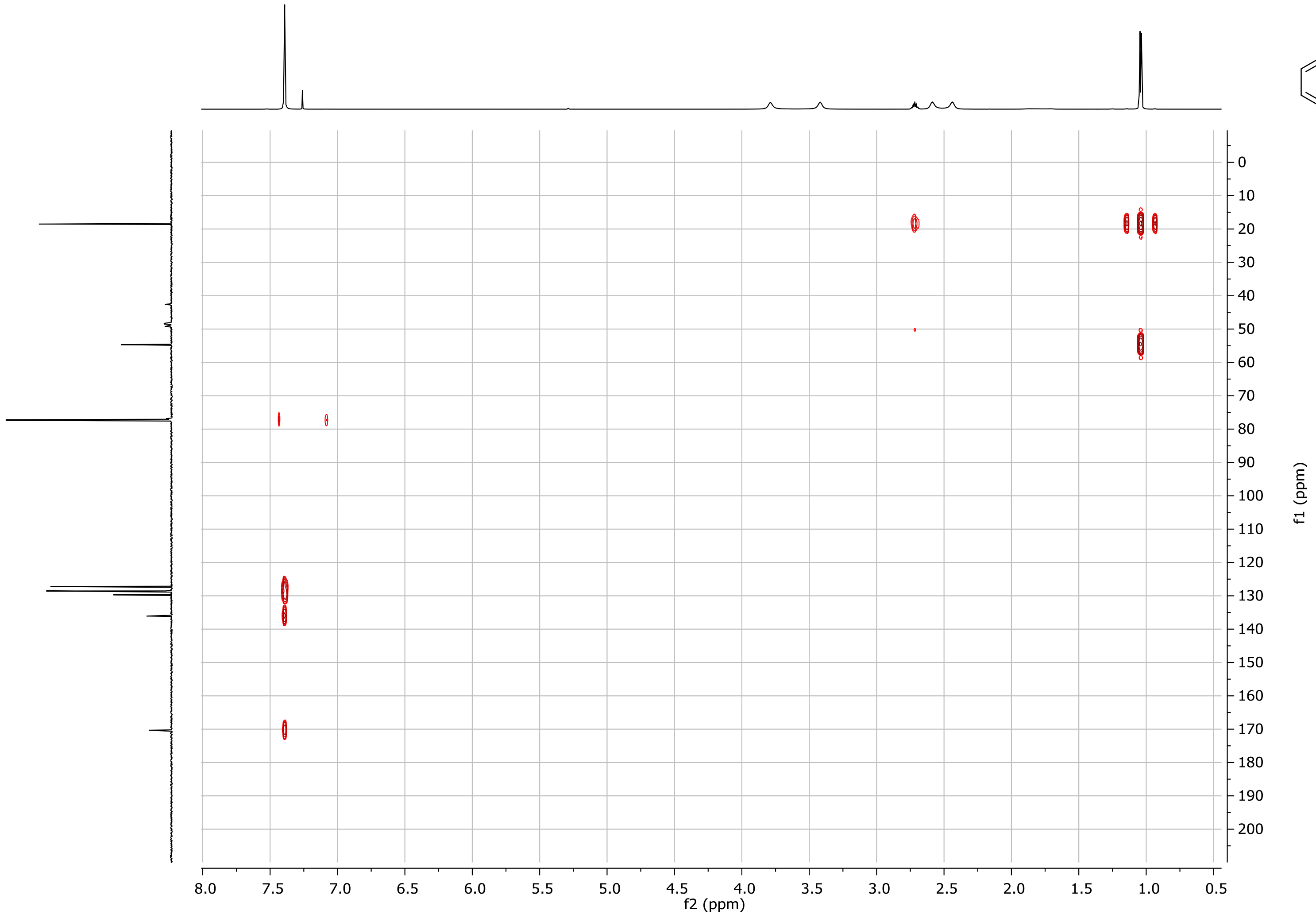

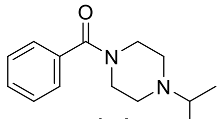

1ad

^{1}H, ^{13}C HSQC

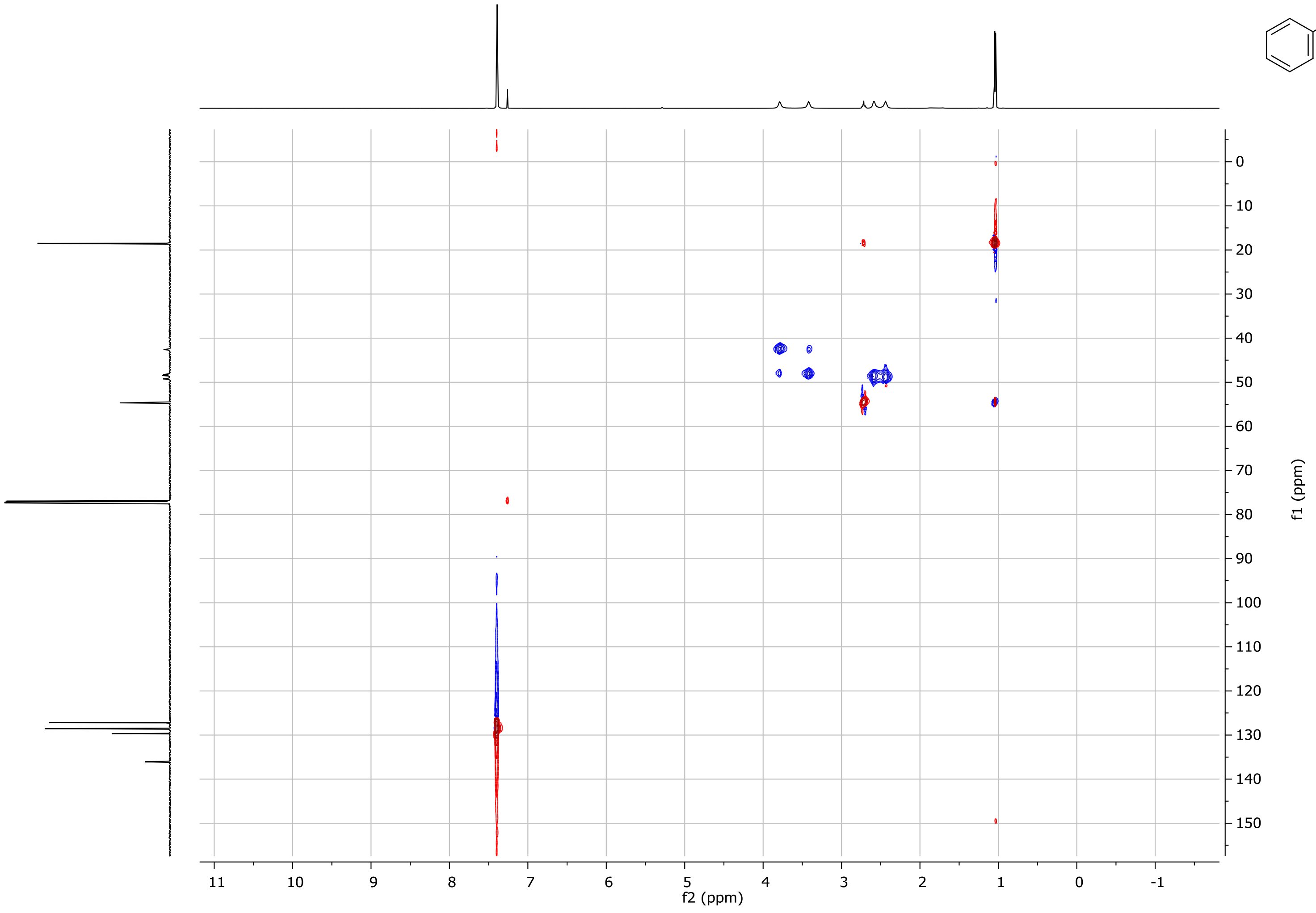

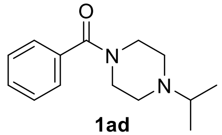

1ad

HRMS

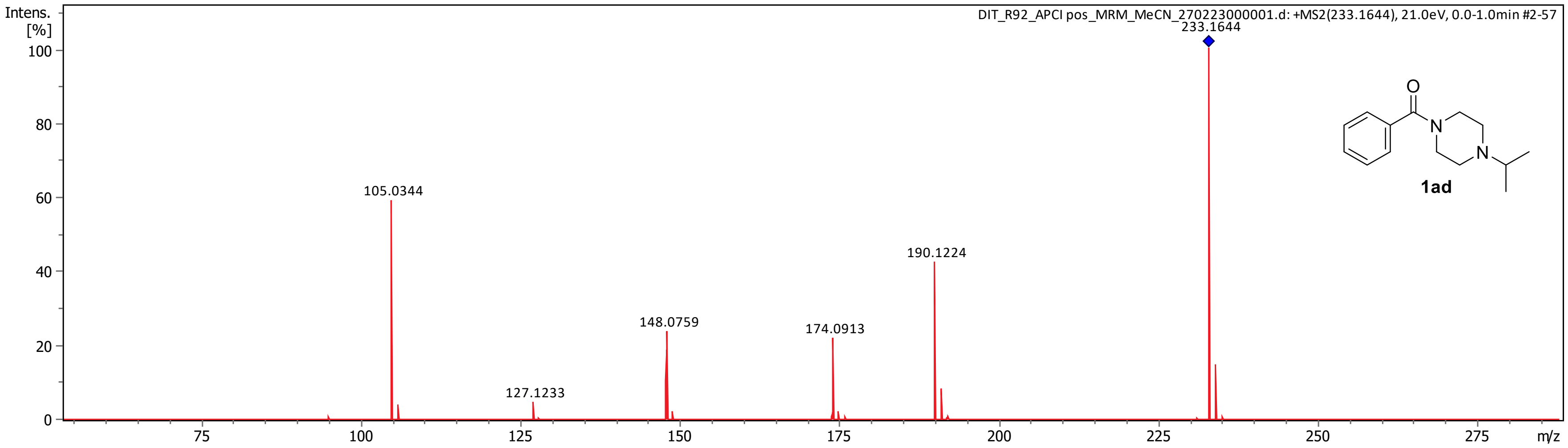

Intens.
[%]
DIT_R92_APCI pos_MRM_MeCN_270223000001.d: +MS2(233.1644), 21.0eV, 0.0-1.0min #2-57
233.1644
105.0344
127.1233
148.0759
174.0913
190.1224
1ad
m/z

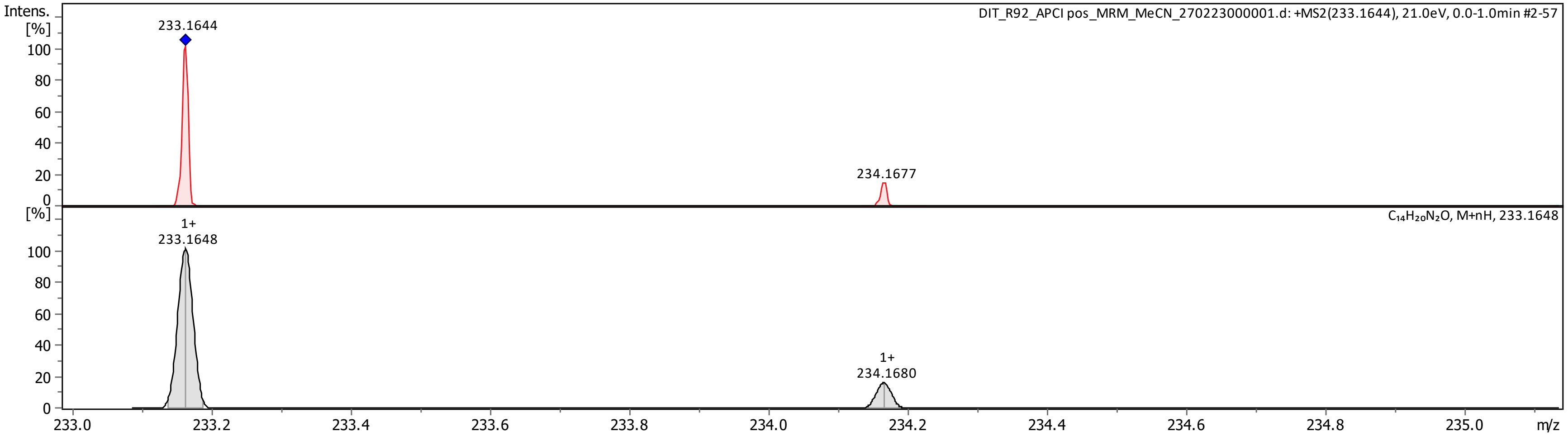

Intens.
[%]
DIT_R92_APCI pos_MRM_MeCN_270223000001.d: +MS2(233.1644), 21.0eV, 0.0-1.0min #2-57
233.1644
234.1677
$C_{14}H_{20}N_2O$, M+nH, 233.1648
1+
233.1648
1+
234.1680
m/z

29 Phenyl(4-(pyridin-2-yl)piperazin-1-yl)methanone (1ae)

^{1}H NMR

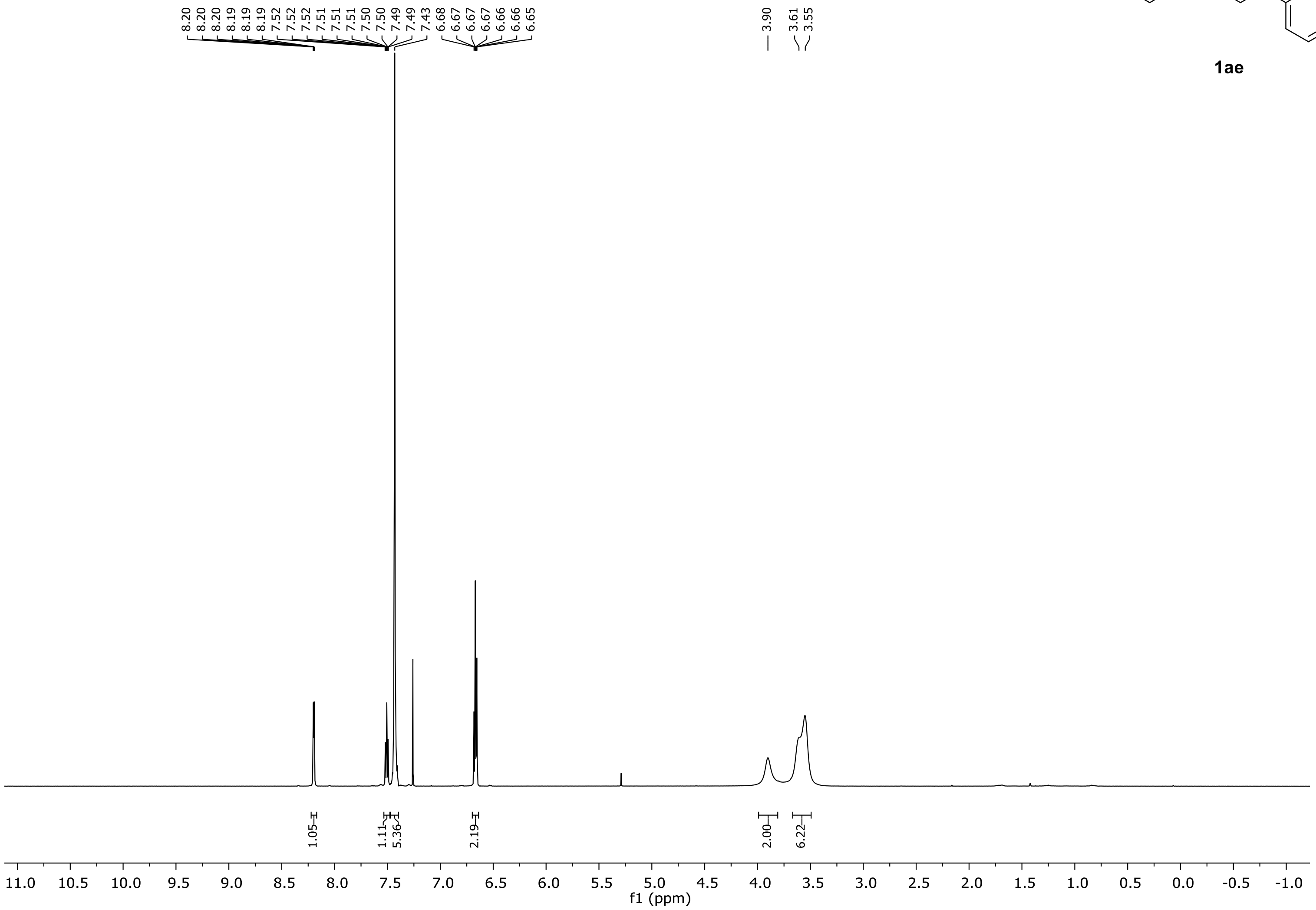

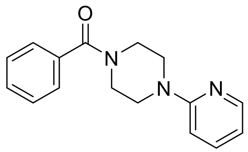

1ae

^{13}C NMR

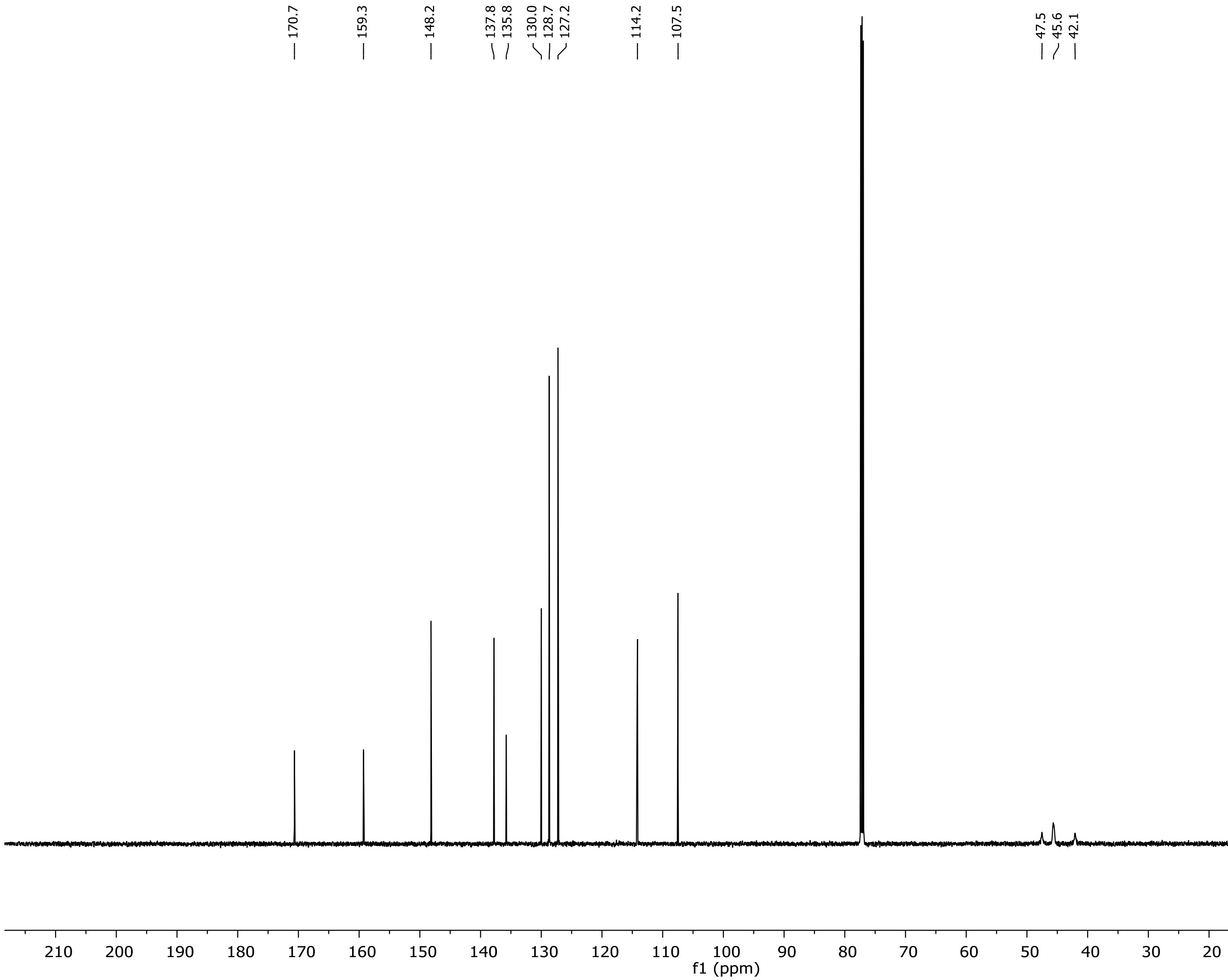
170.7
159.3
148.2
137.8
135.8
130.0
128.7
127.2
114.2
107.5
47.5
45.6
42.1
210 200 190 180 170 160 150 140 130 120 110 100 90 80 70 60 50 40 30 20 10 0 -10
f1 (ppm)

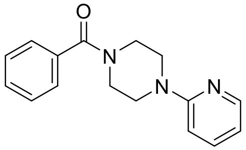
O
N
N
N

1ae

^{1}H, ^{1}H COSY

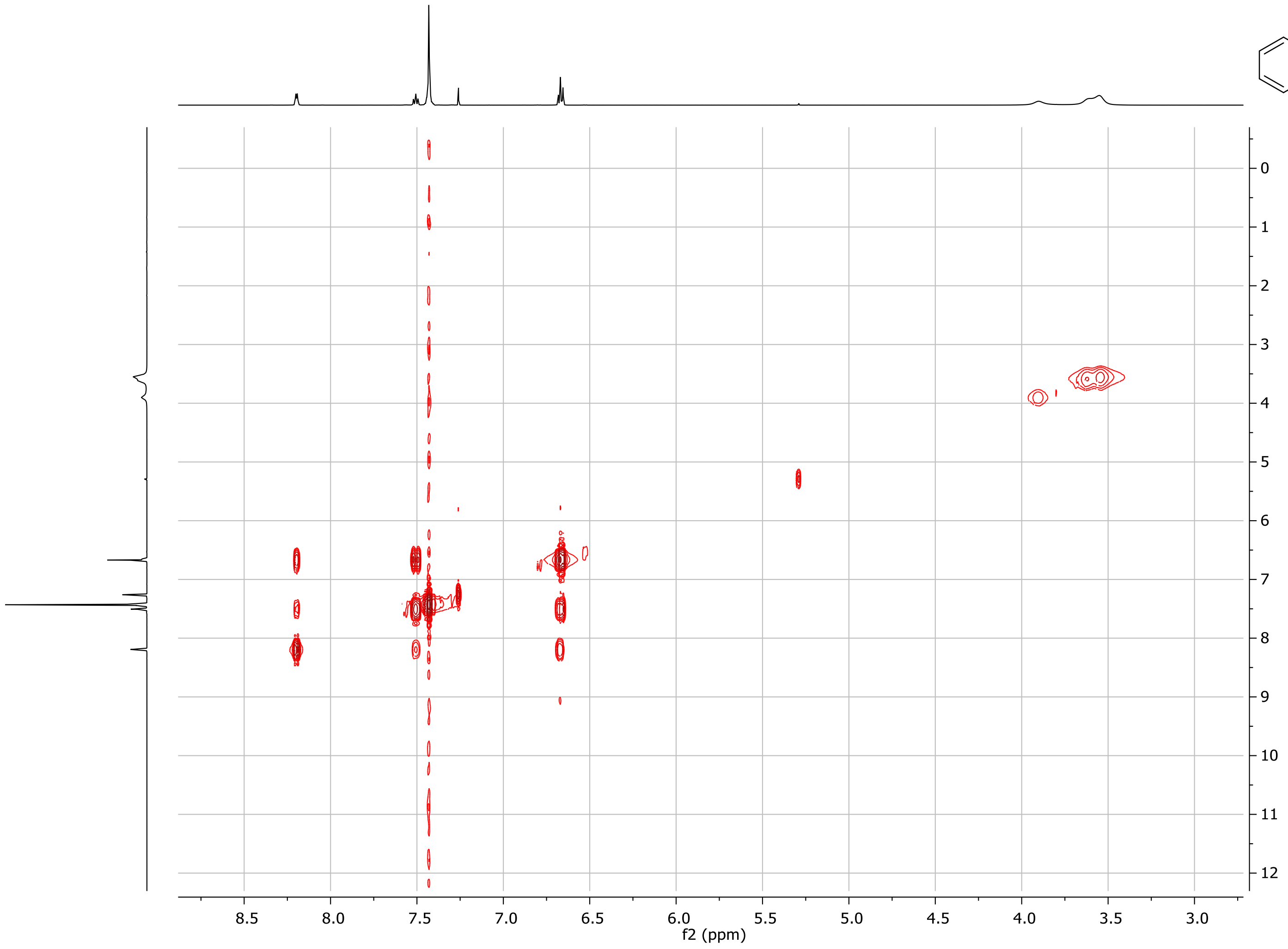

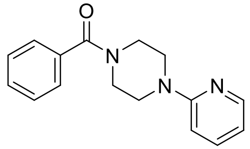

1ae

^{1}H, ^{13}C HMBC

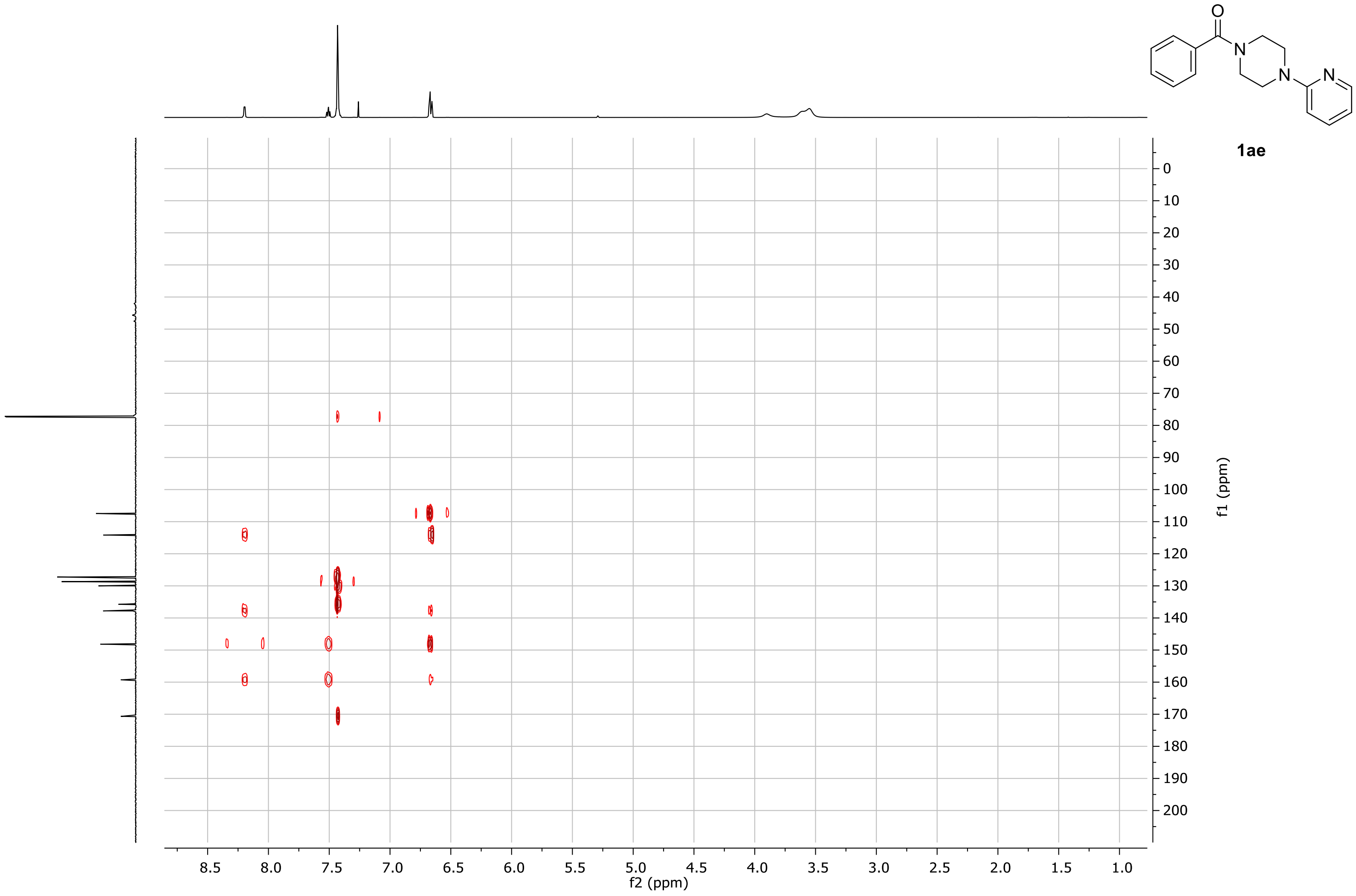

^{1}H, ^{13}C HSQC

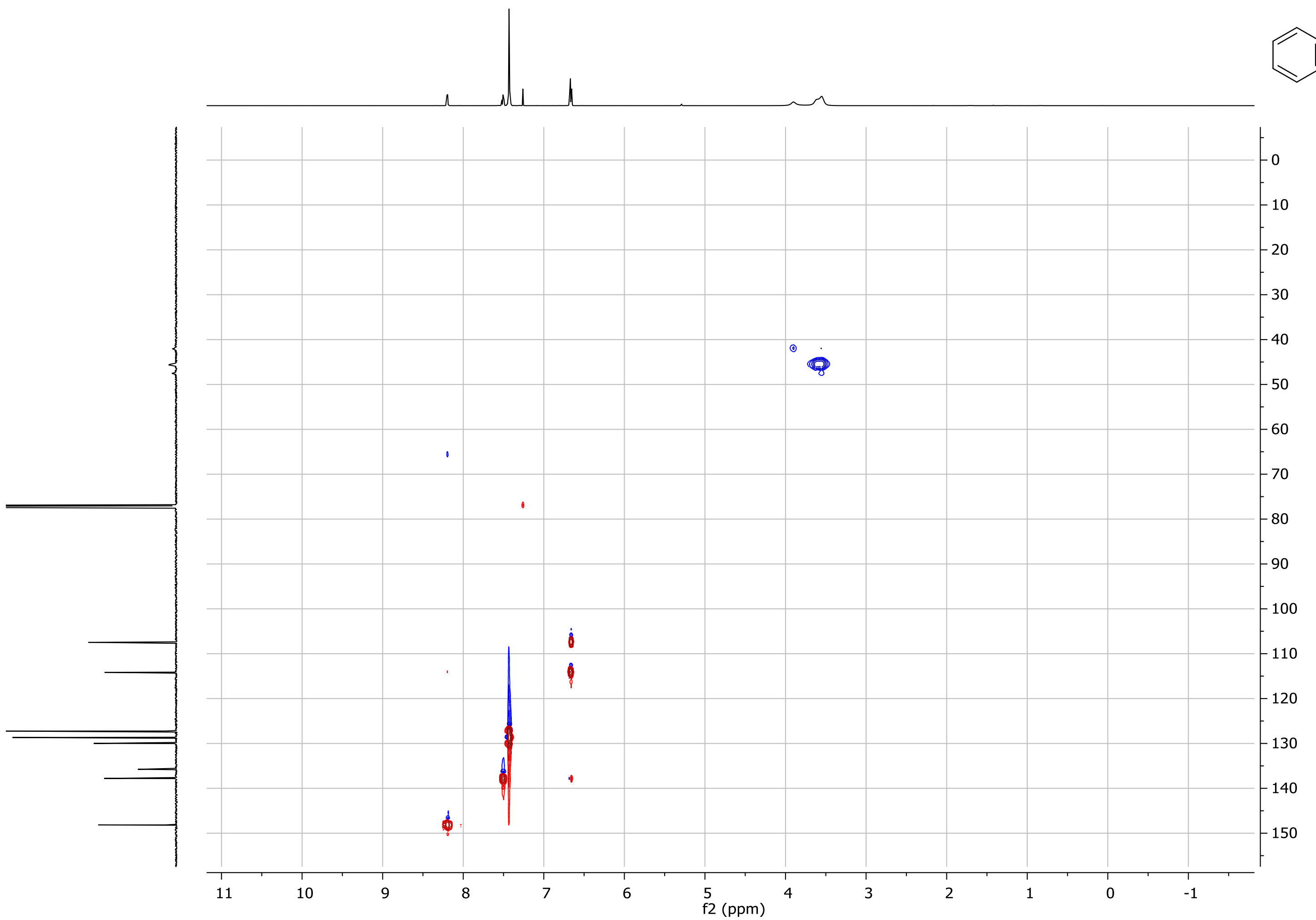

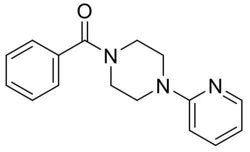

1ae

HRMS

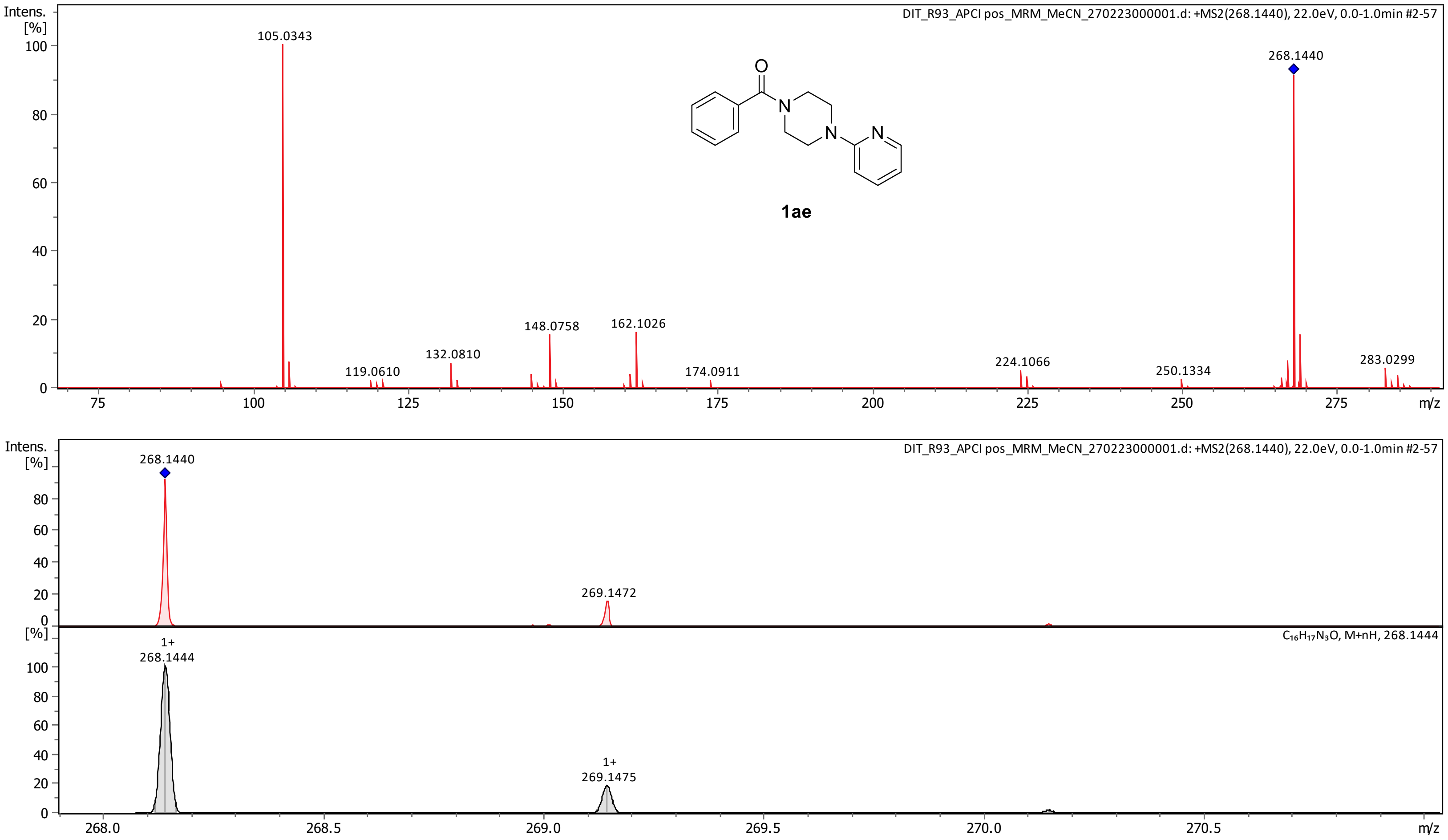

Intens. [%]
DIT_R93_APCI pos_MRM_MeCN_270223000001.d: +MS2(268.1440), 22.0eV, 0.0-1.0min #2-57
105.0343
268.1440
119.0610
132.0810
148.0758
162.1026
174.0911
224.1066
250.1334
283.0299
1ae
m/z
269.1472
1+
268.1444
269.1475
C₁₆H₁₇N₃O, M+nH, 268.1444

30 (2-(Methoxymethyl)phenyl)(piperidin-1-yl)methanone (S2)

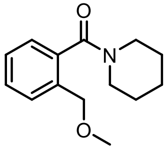

S2

^{1}H NMR

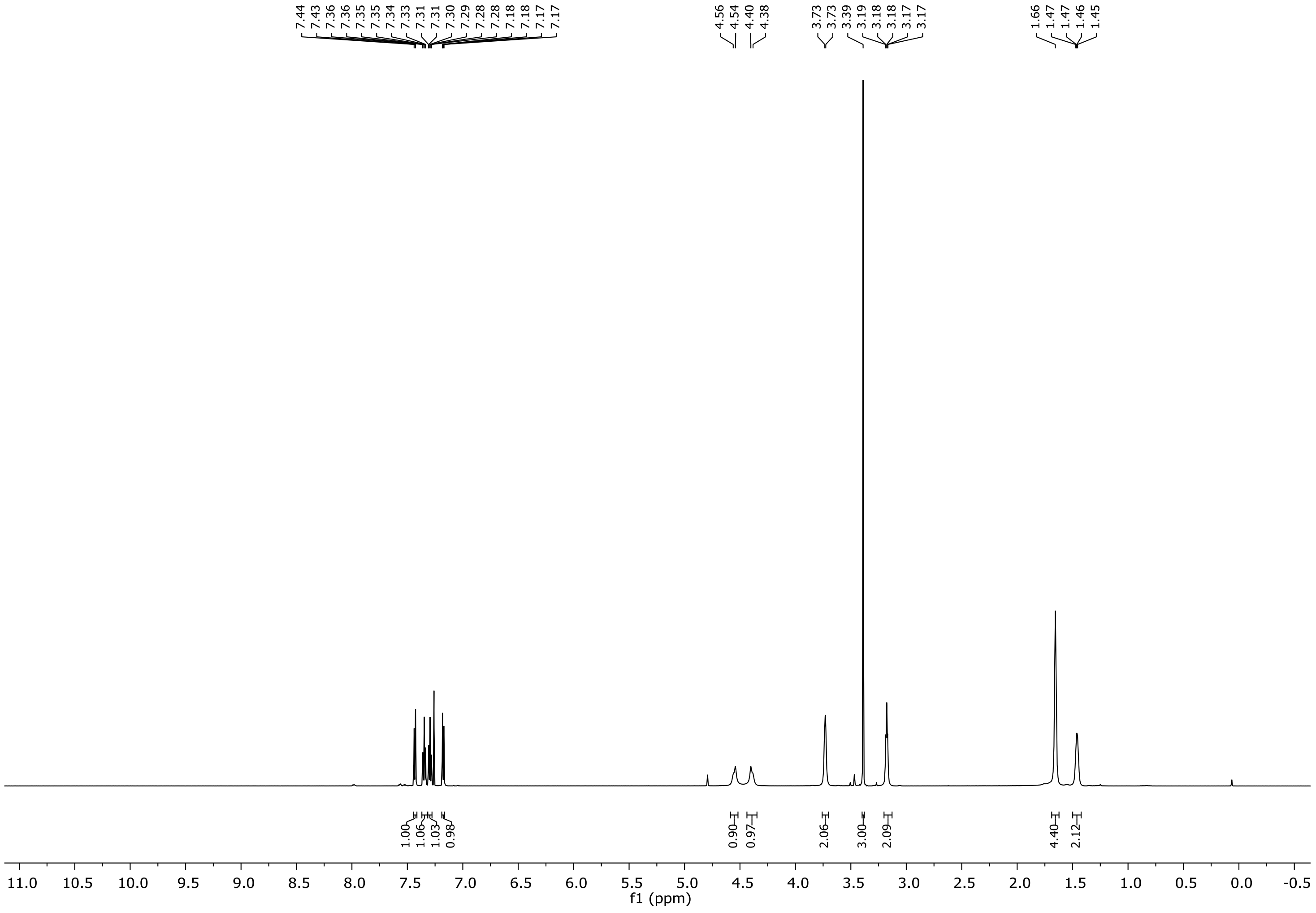

^{13}C NMR

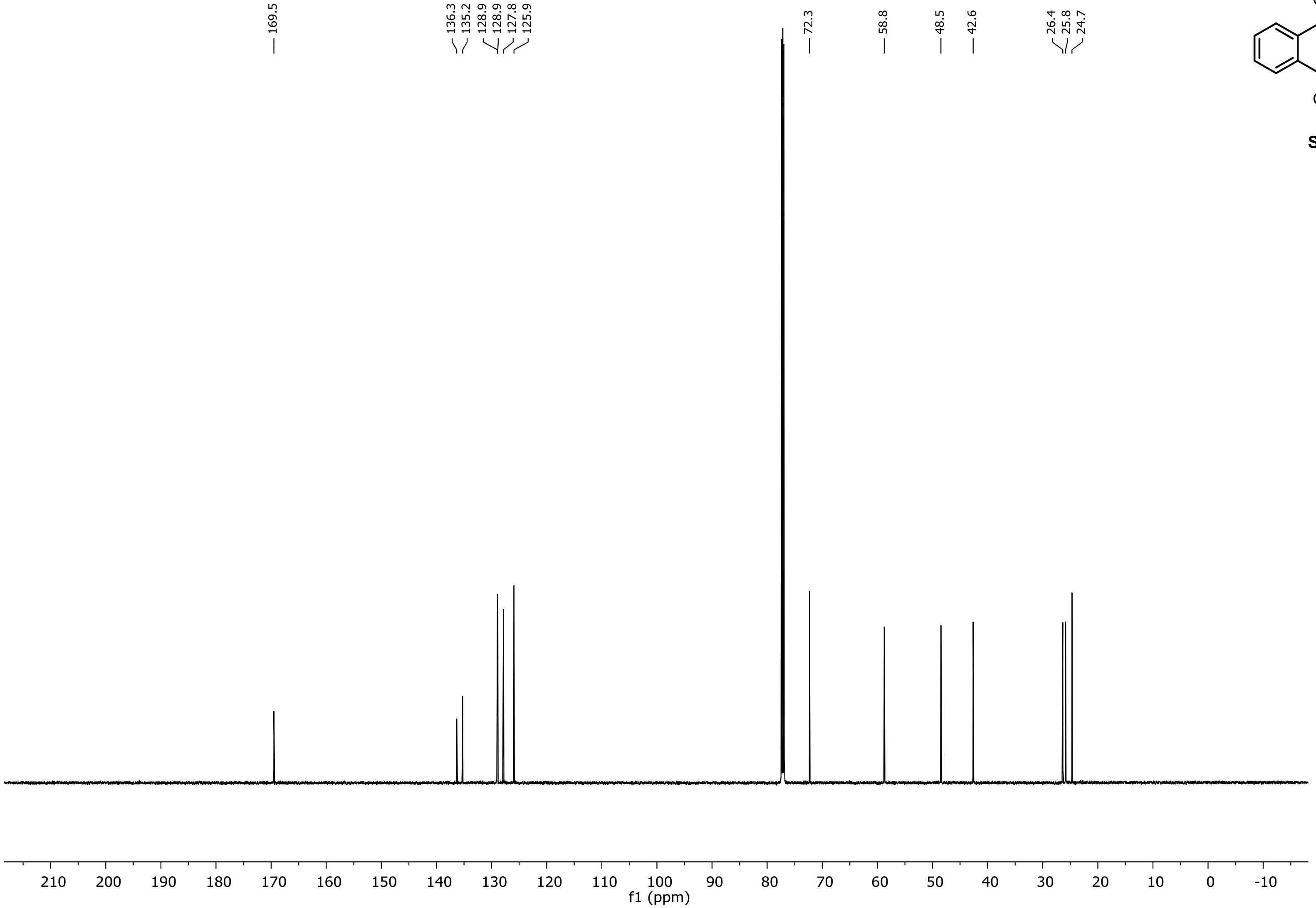

169.5
136.3
135.2
128.9
128.9
127.8
125.9
72.3
58.8
48.5
42.6
26.4
25.8
24.7
210 200 190 180 170 160 150 140 130 120 110 100 90 80 70 60 50 40 30 20 10 0 -10
f1 (ppm)

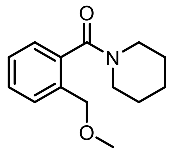

O
N
O

S2

^{1}H, ^{1}H COSY

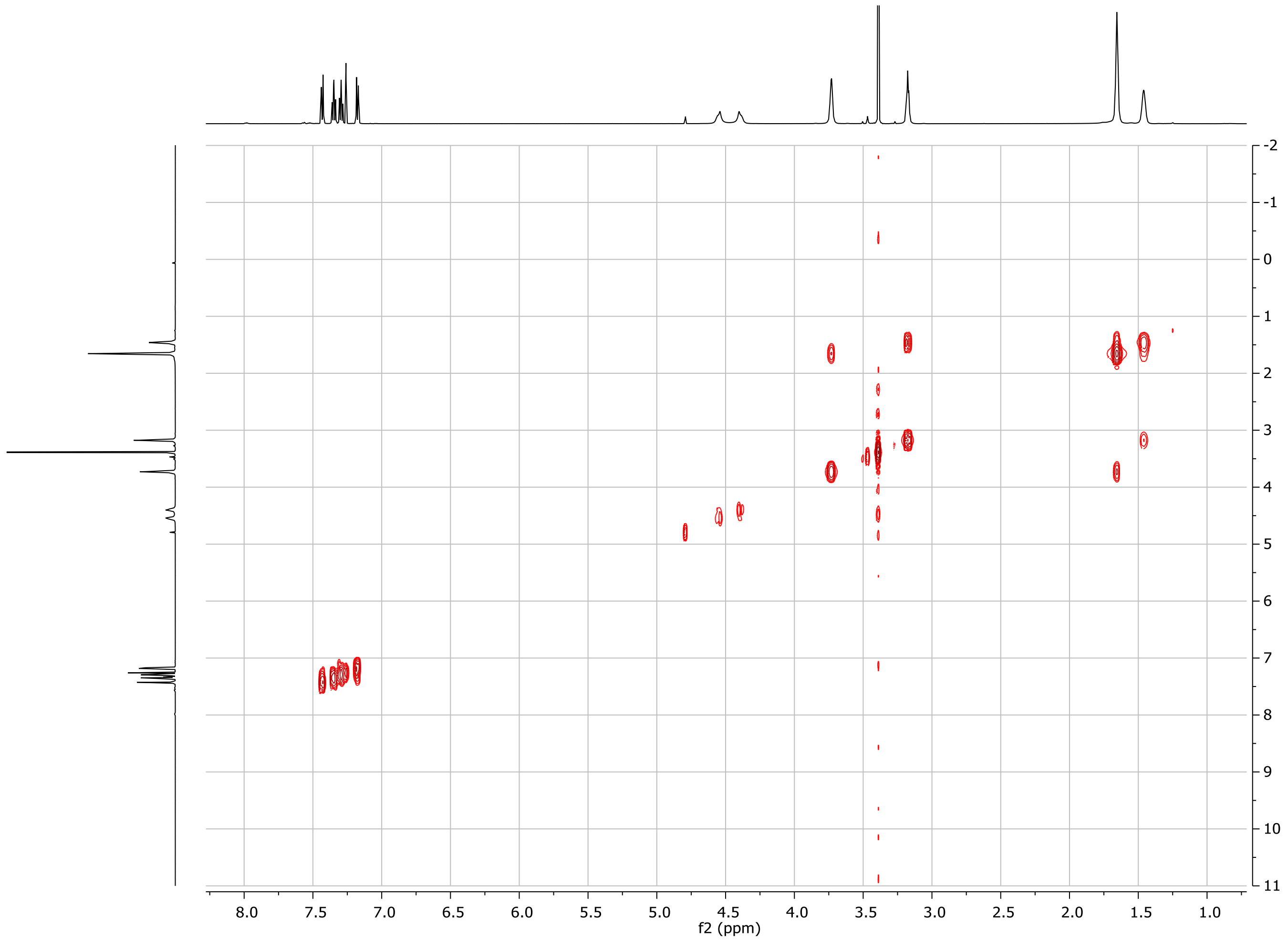

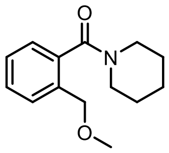

S2

^{1}H, ^{13}C HMBC

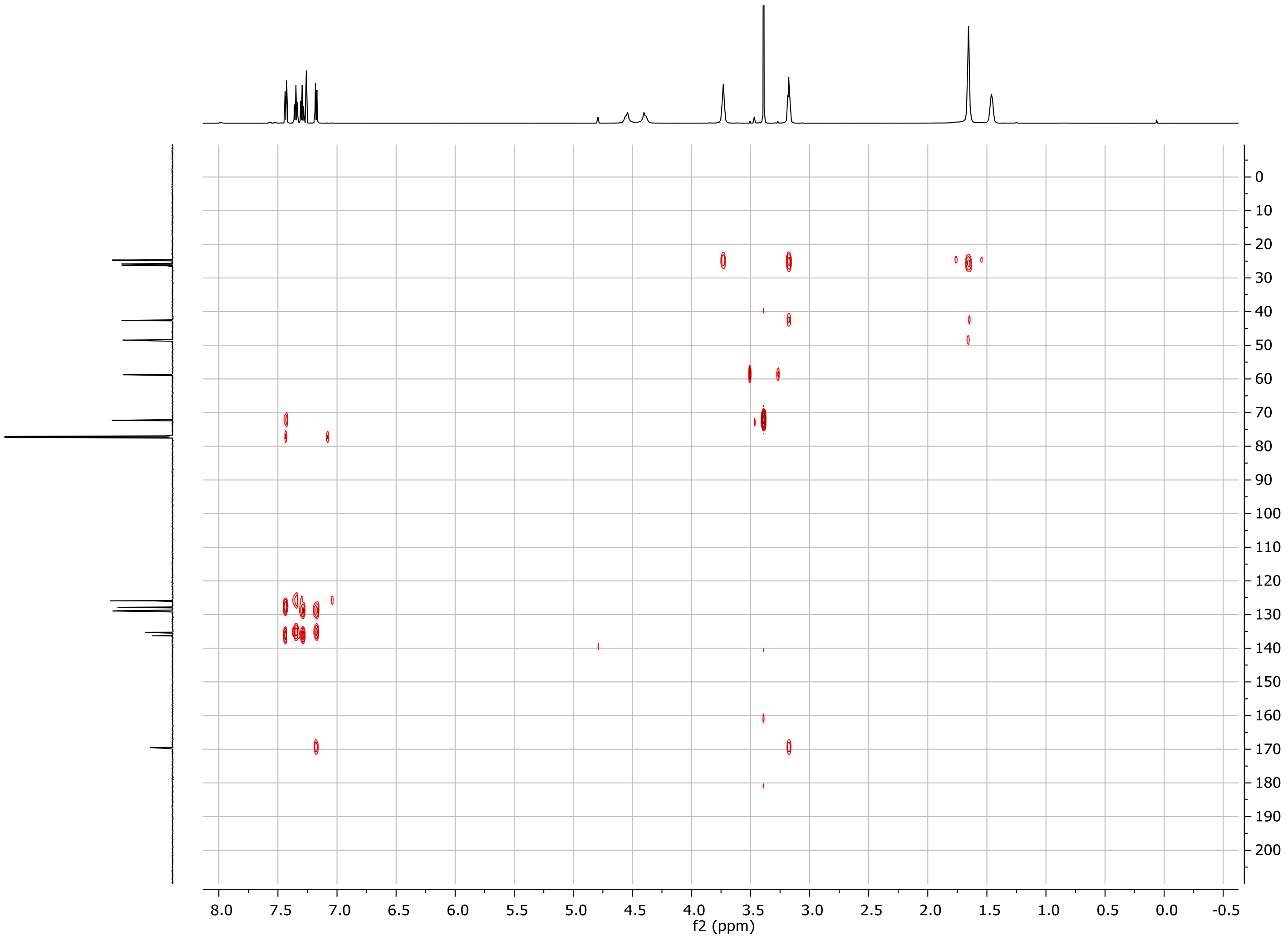

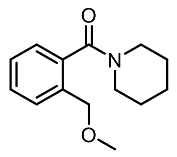

S2

^{1}H, ^{13}C HSQC

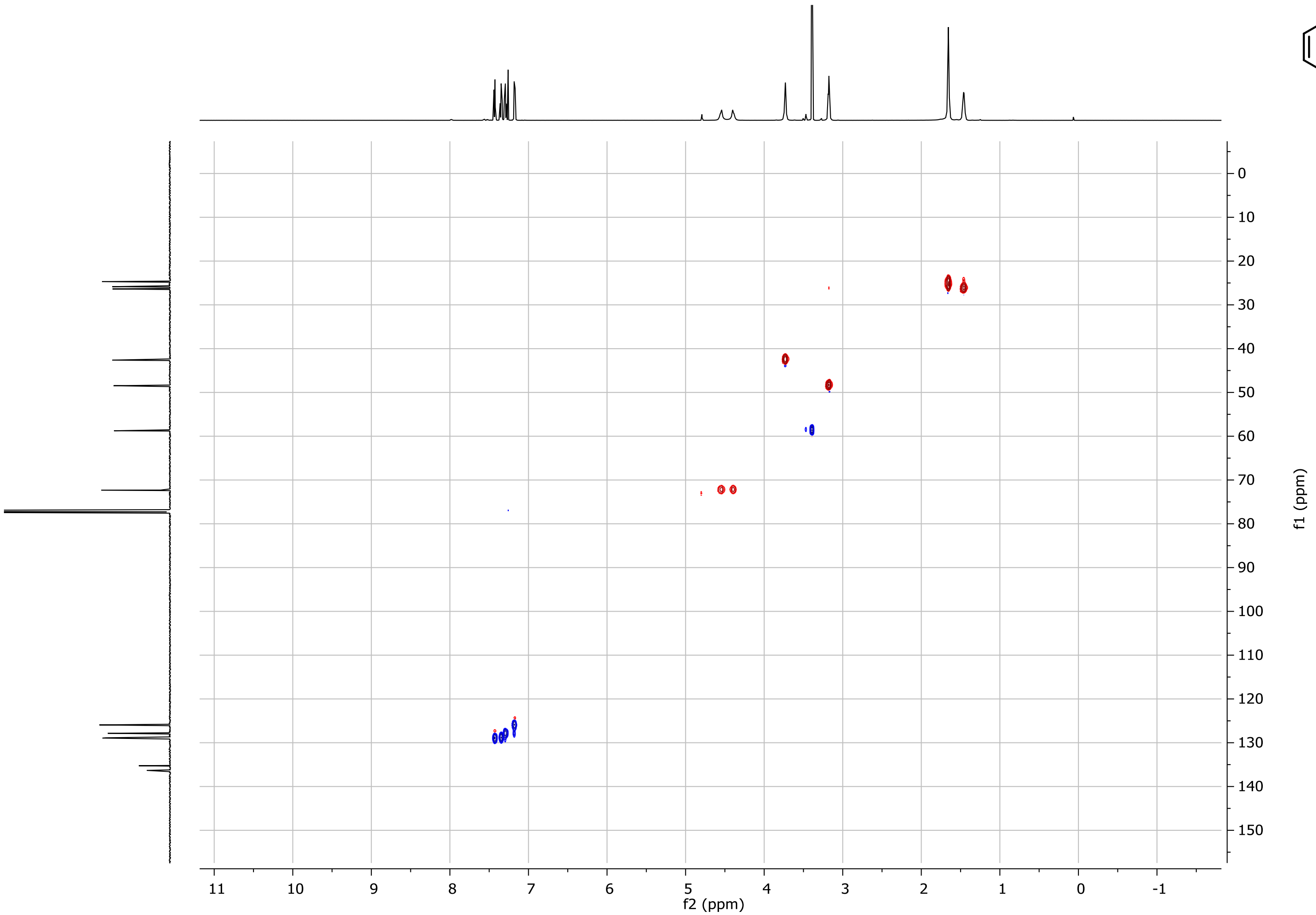

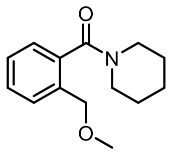

S2

HRMS

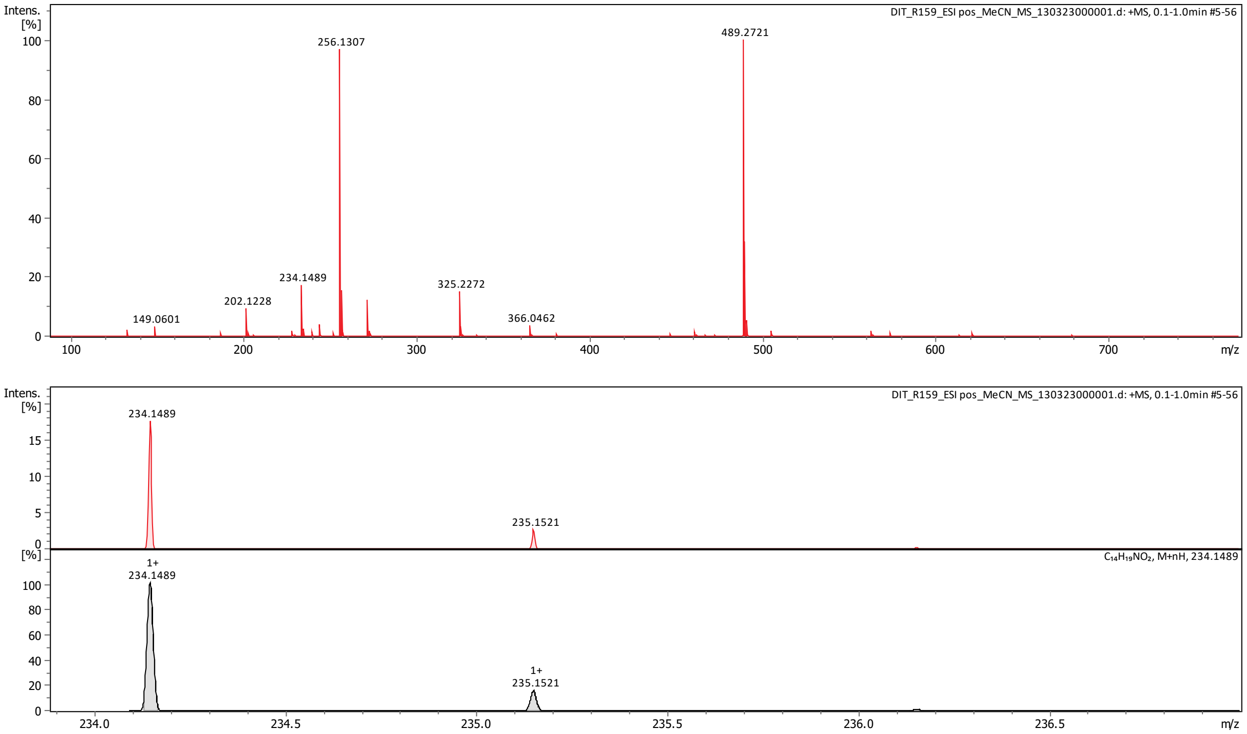

Intens. [%]
DIT_R159_ESI pos_MeCN_MS_130323000001.d: +MS, 0.1-1.0min #5-56
256.1307
489.2721
234.1489
325.2272
202.1228
149.0601
366.0462
m/z
234.1489
235.1521
$C_{14}H_{19}NO_2$, M+nH, 234.1489
1+
234.1489
1+
235.1521
m/z

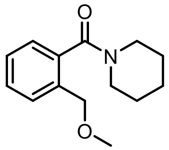

S2

IR

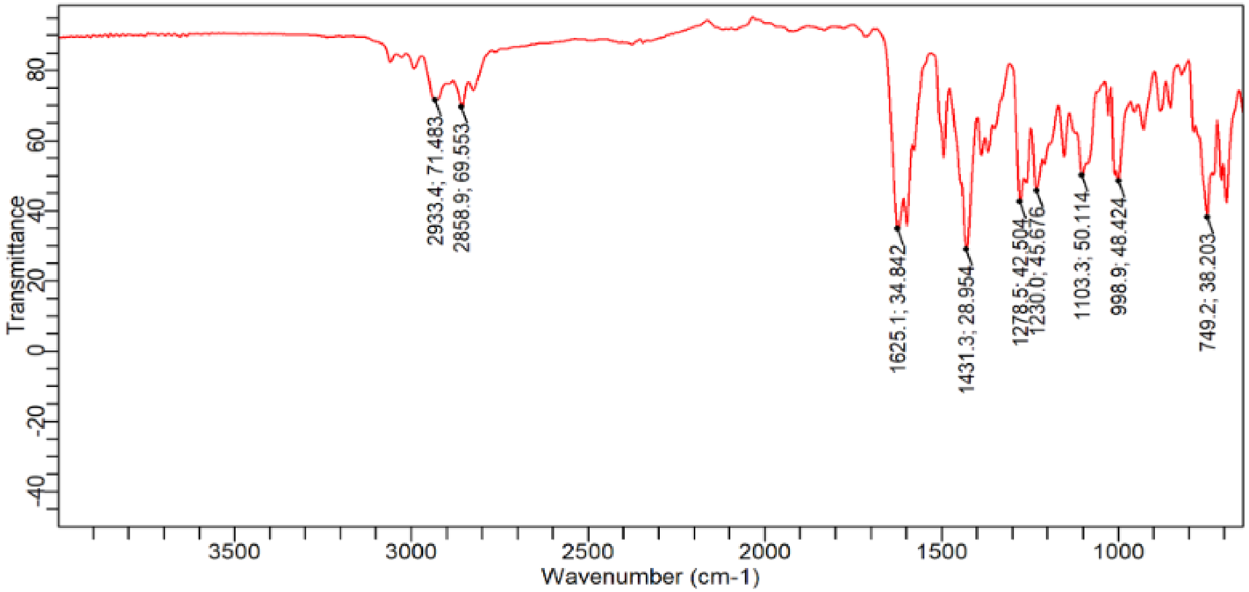

Transmittance
80
60
40
20
0
-20
-40
3500
3000
2500
2000
1500
1000
Wavenumber (cm-1)
2933.4; 71.483
2858.9; 69.553
1625.1; 34.842
1431.3; 28.954
1278.5; 42.504
1230.0; 45.676
1103.3; 50.114
998.9; 48.424
749.2; 38.203

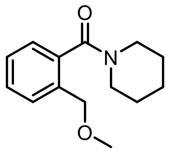

O
N
O

S2

31 Phenyl(tetrahydro-1,4-oxazepin-4(5*H*)-yl)methanone (S3)

^{1}H NMR

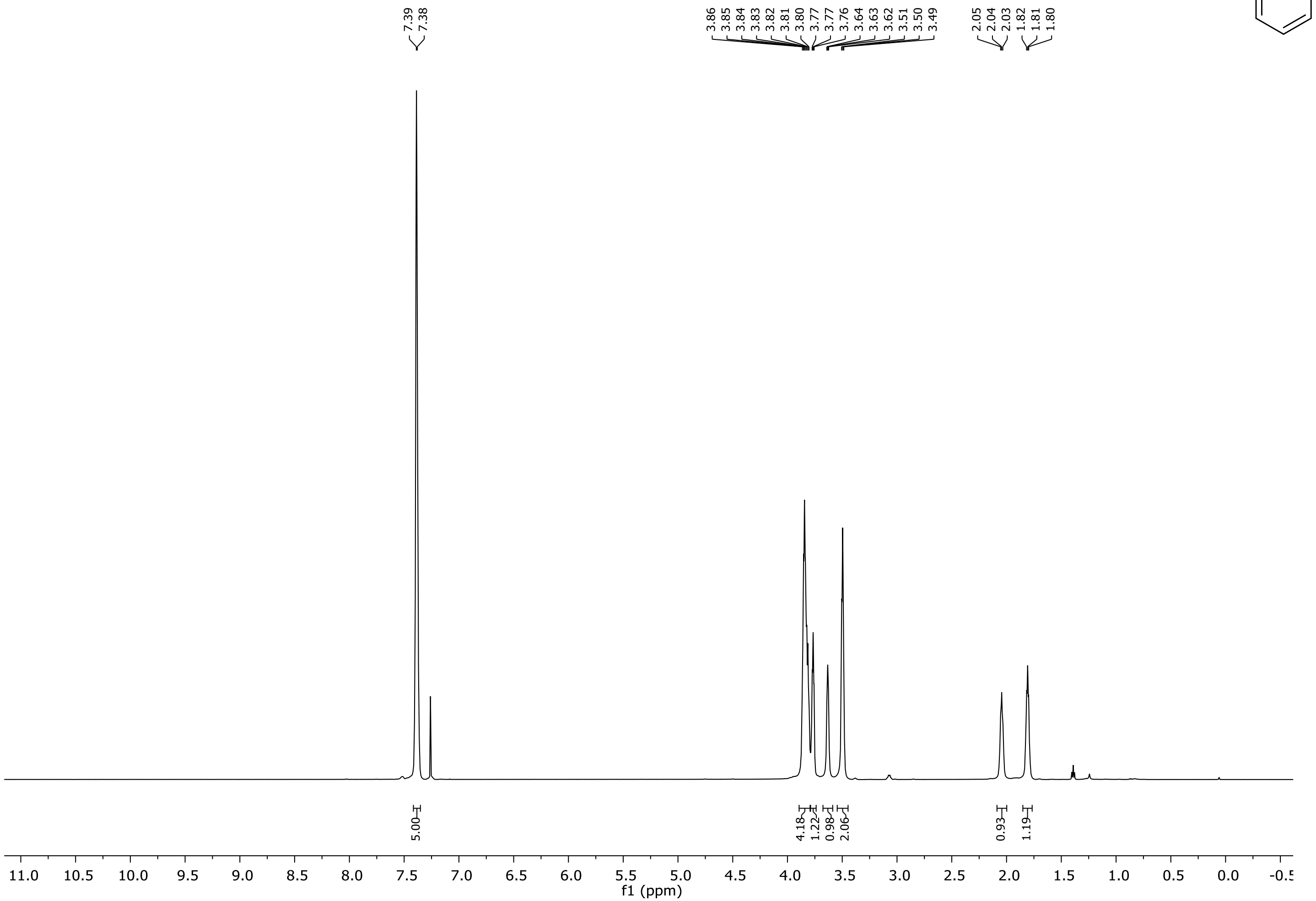

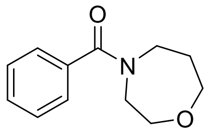

S3

^{13}C NMR

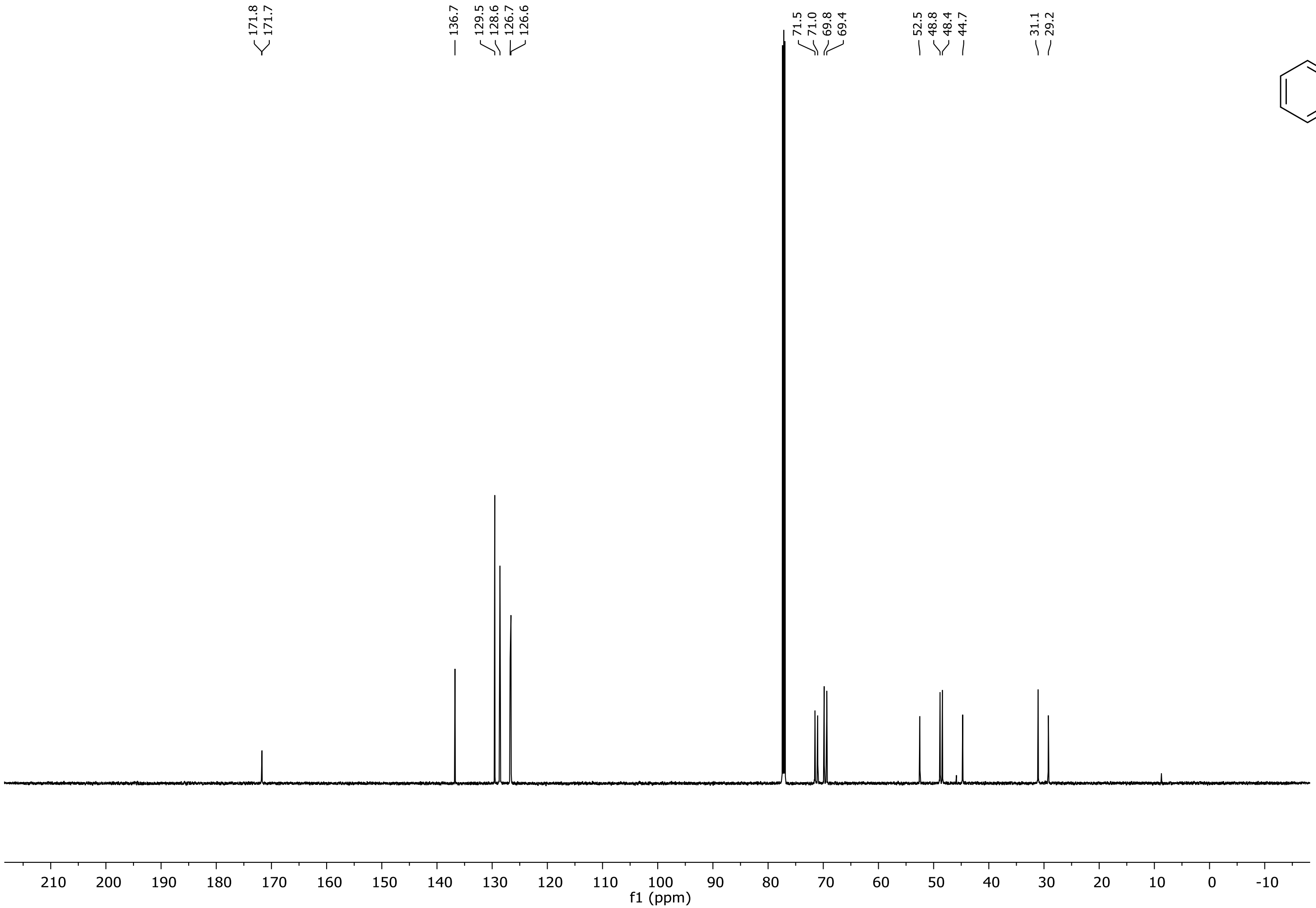
171.8
171.7
136.7
129.5
128.6
126.7
126.6
71.5
71.0
69.8
69.4
52.5
48.8
48.4
44.7
31.1
29.2
210 200 190 180 170 160 150 140 130 120 110 100 90 80 70 60 50 40 30 20 10 0 -10
f1 (ppm)

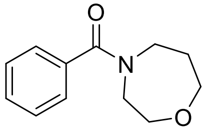
O
N
O

S3

^{1}H, ^{1}H COSY

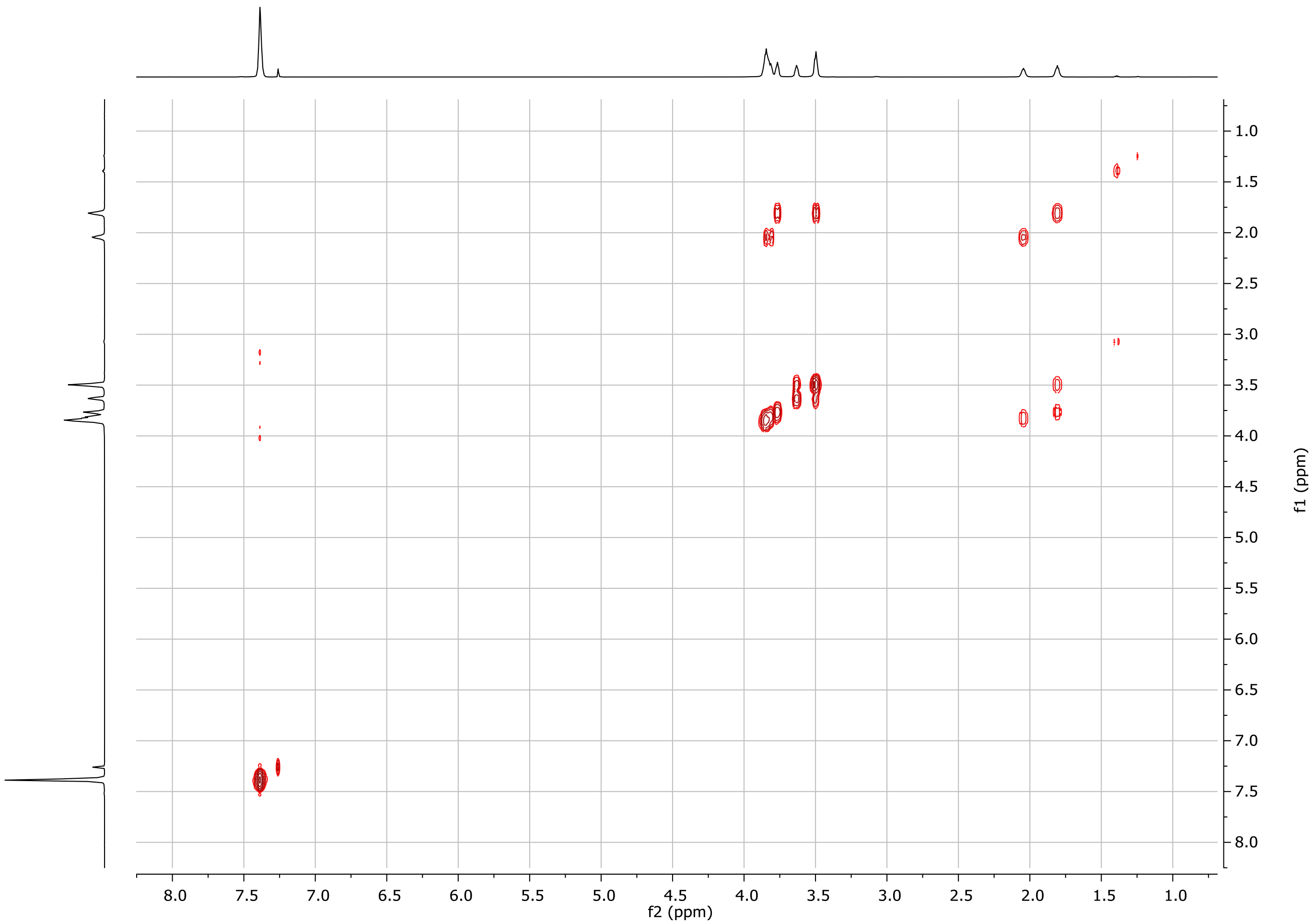

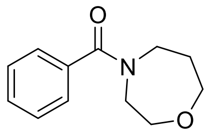

S3

^{1}H, ^{13}C HMBC

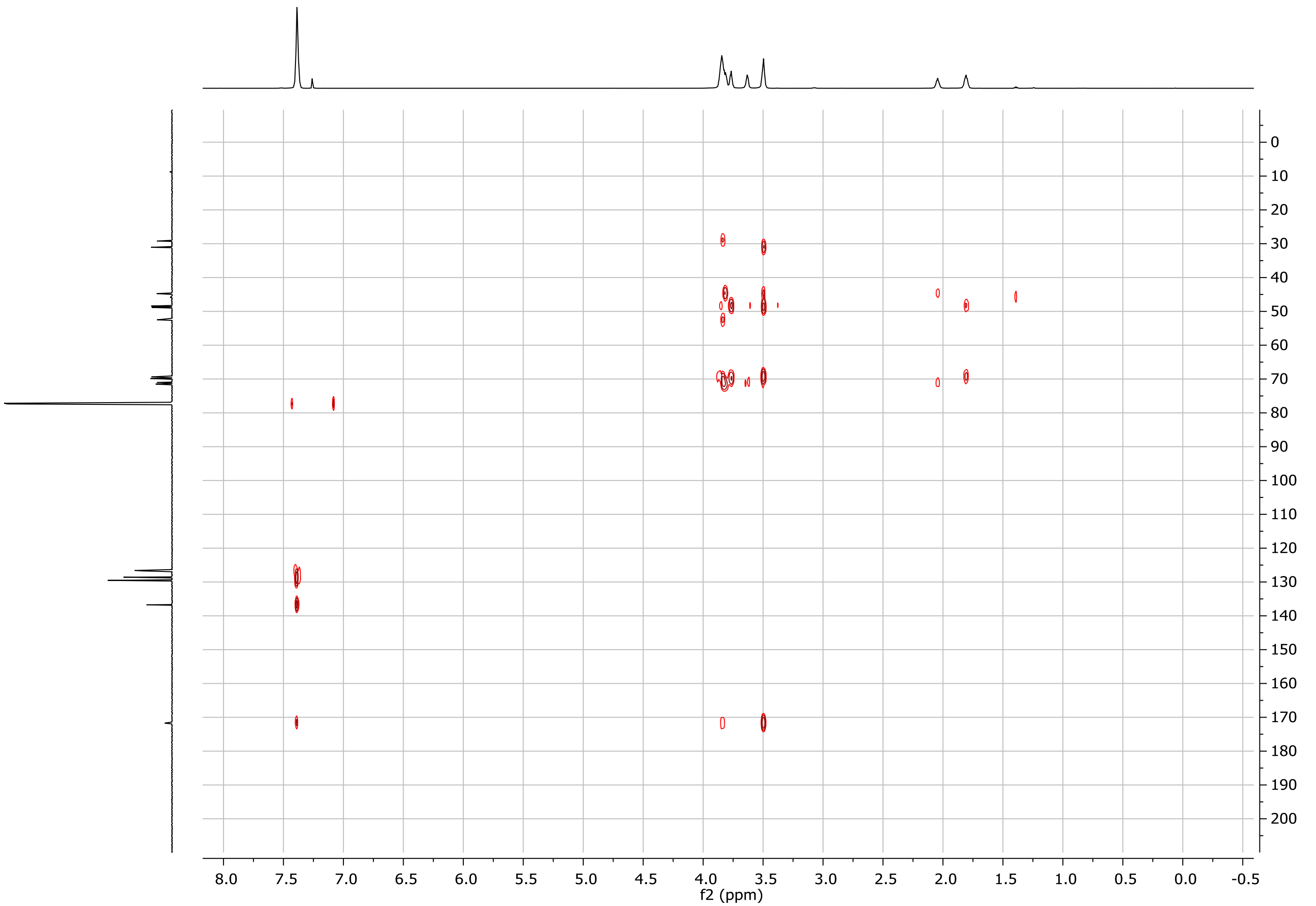

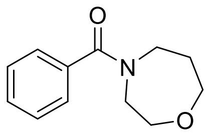

S3

^{1}H, ^{13}C HSQC

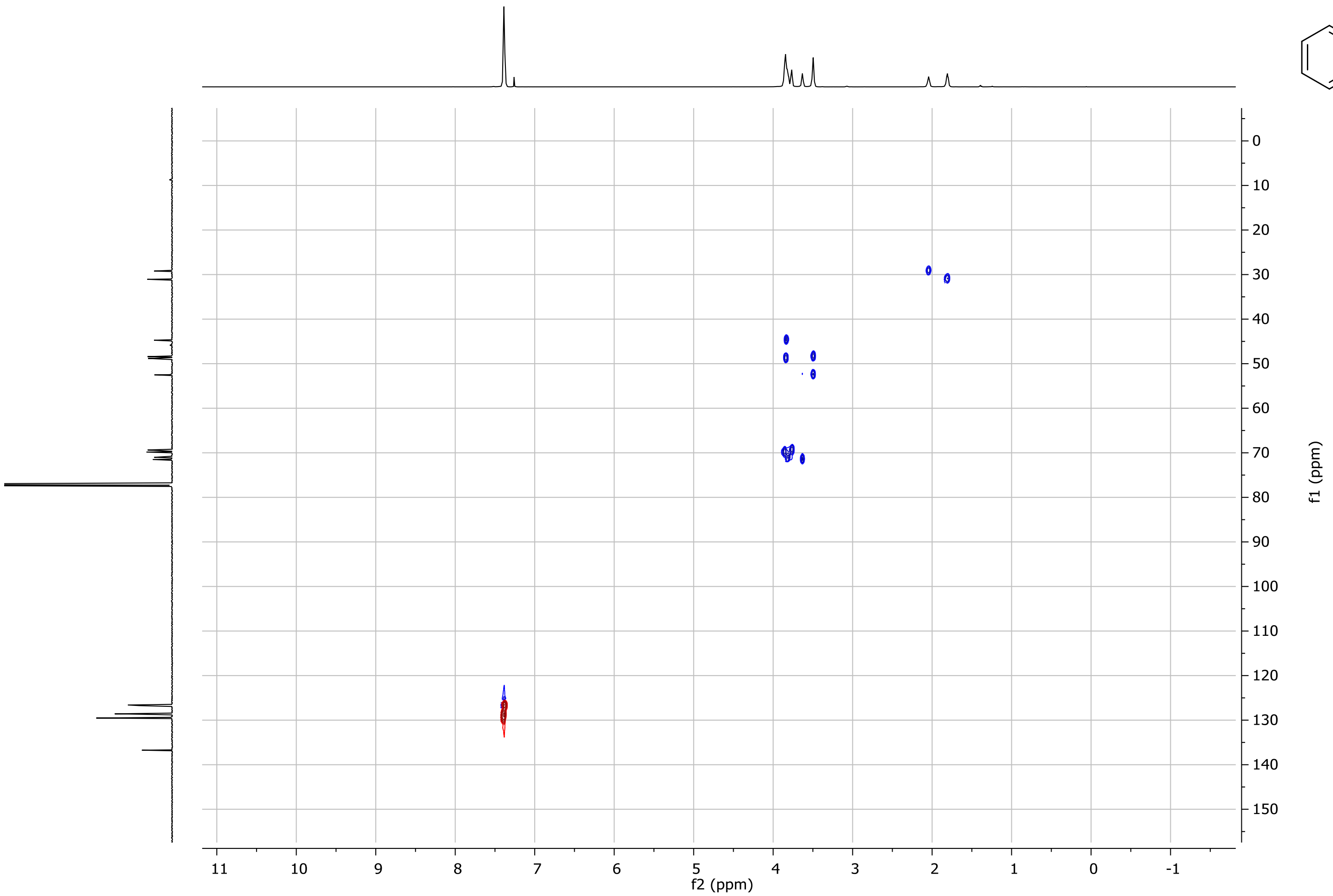

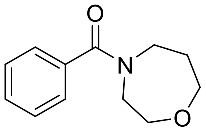

S3

HRMS

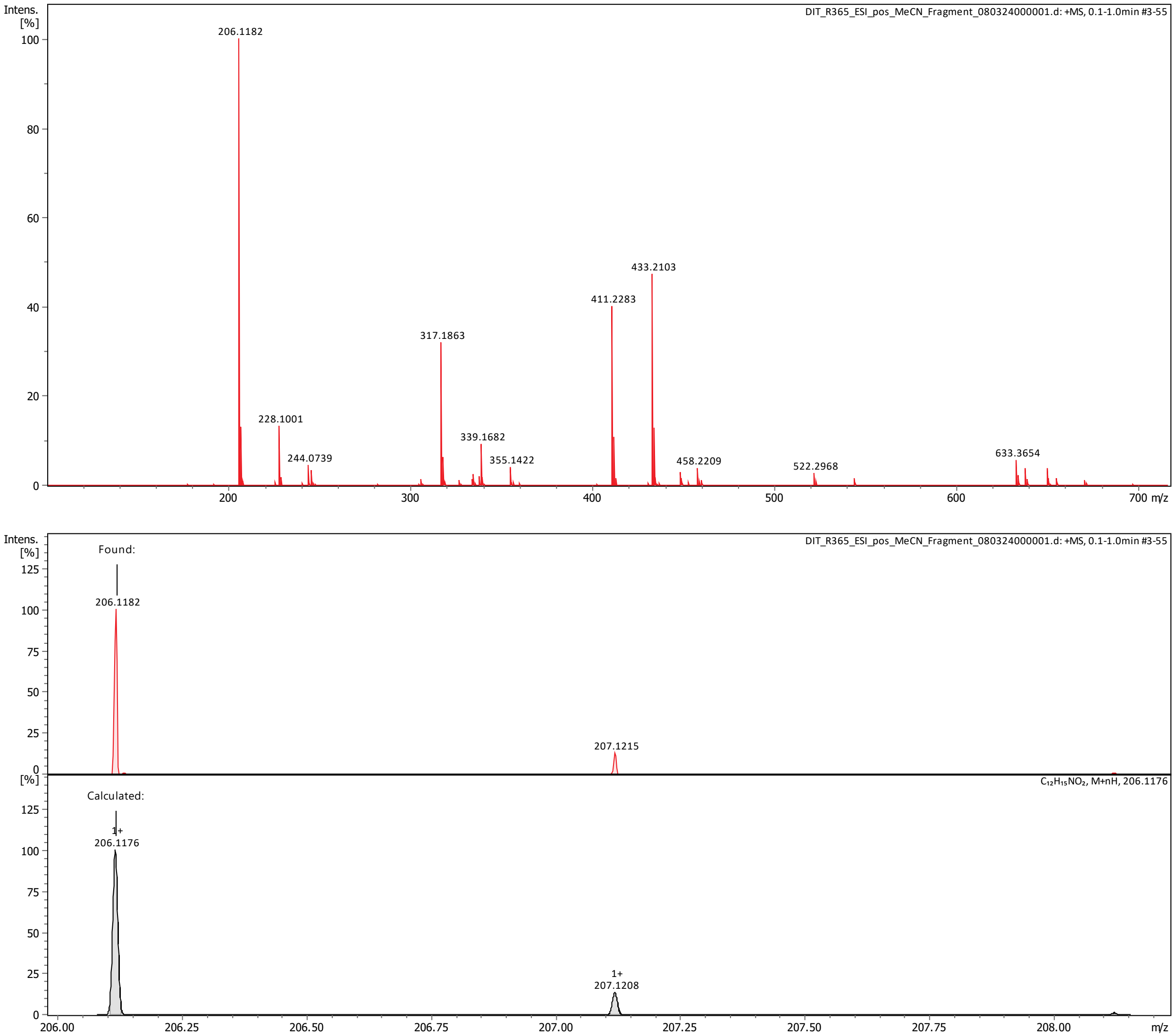

Intens. [%]
DIT_R365_ESI_pos_MeCN_Fragment_080324000001.d: +MS, 0.1-1.0min #3-55
206.1182
228.1001
244.0739
317.1863
339.1682
355.1422
411.2283
433.2103
458.2209
522.2968
633.3654
m/z
Found:
206.1182
207.1215
Calculated:
$C_{12}H_{15}NO_2$, M+nH, 206.1176
1+
206.1176
1+
207.1208

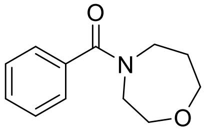

S3

IR

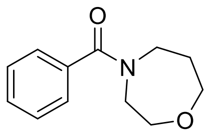

S3

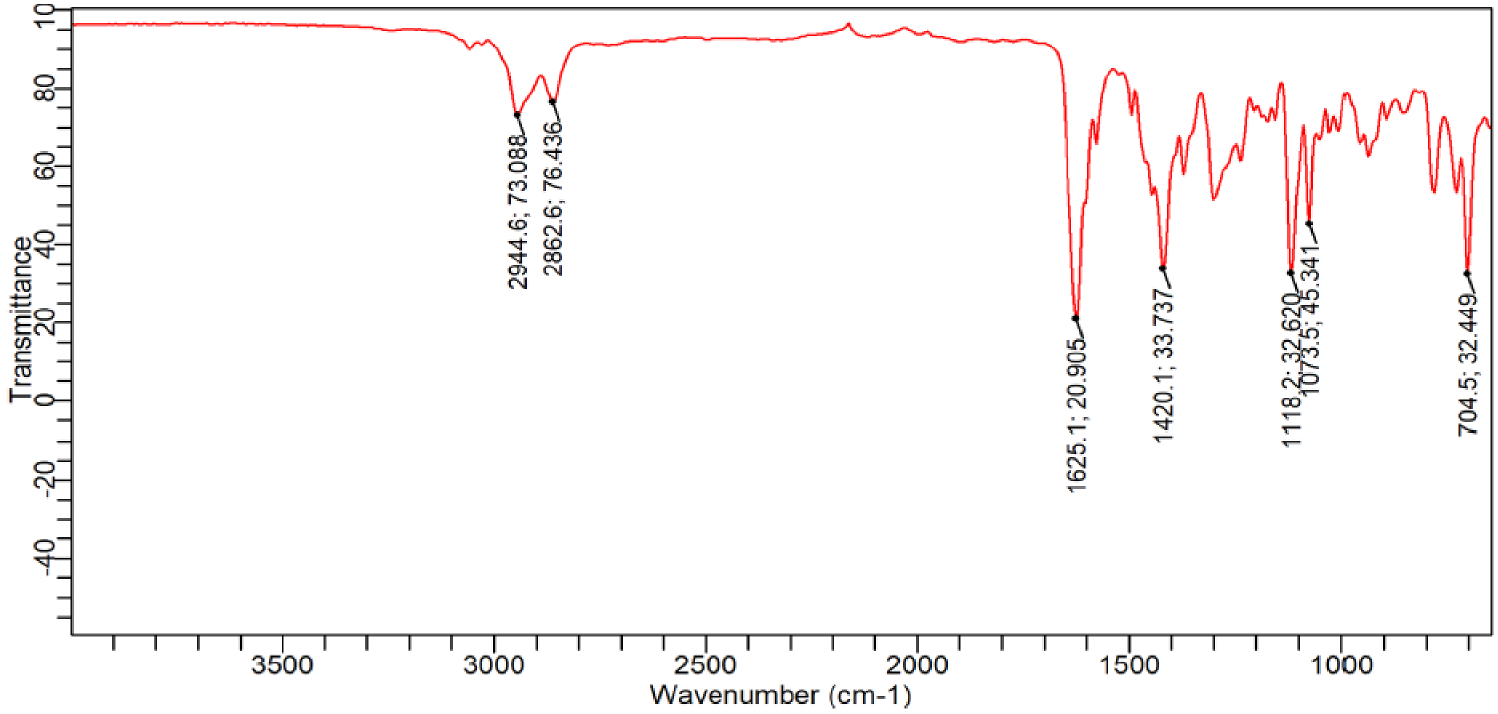

Transmittance
Wavenumber (cm-1)
2944.6; 73.088
2862.6; 76.436
1625.1; 20.905
1420.1; 33.737
1118.2; 32.620
1073.5; 45.341
704.5; 32.449

32 2-[4-(Morpholine-4-carbonyl)phenyl]-1-(1-piperidyl)ethanone (11)

^{1}H NMR

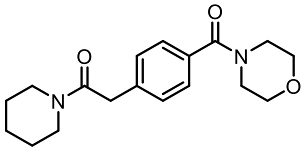

11

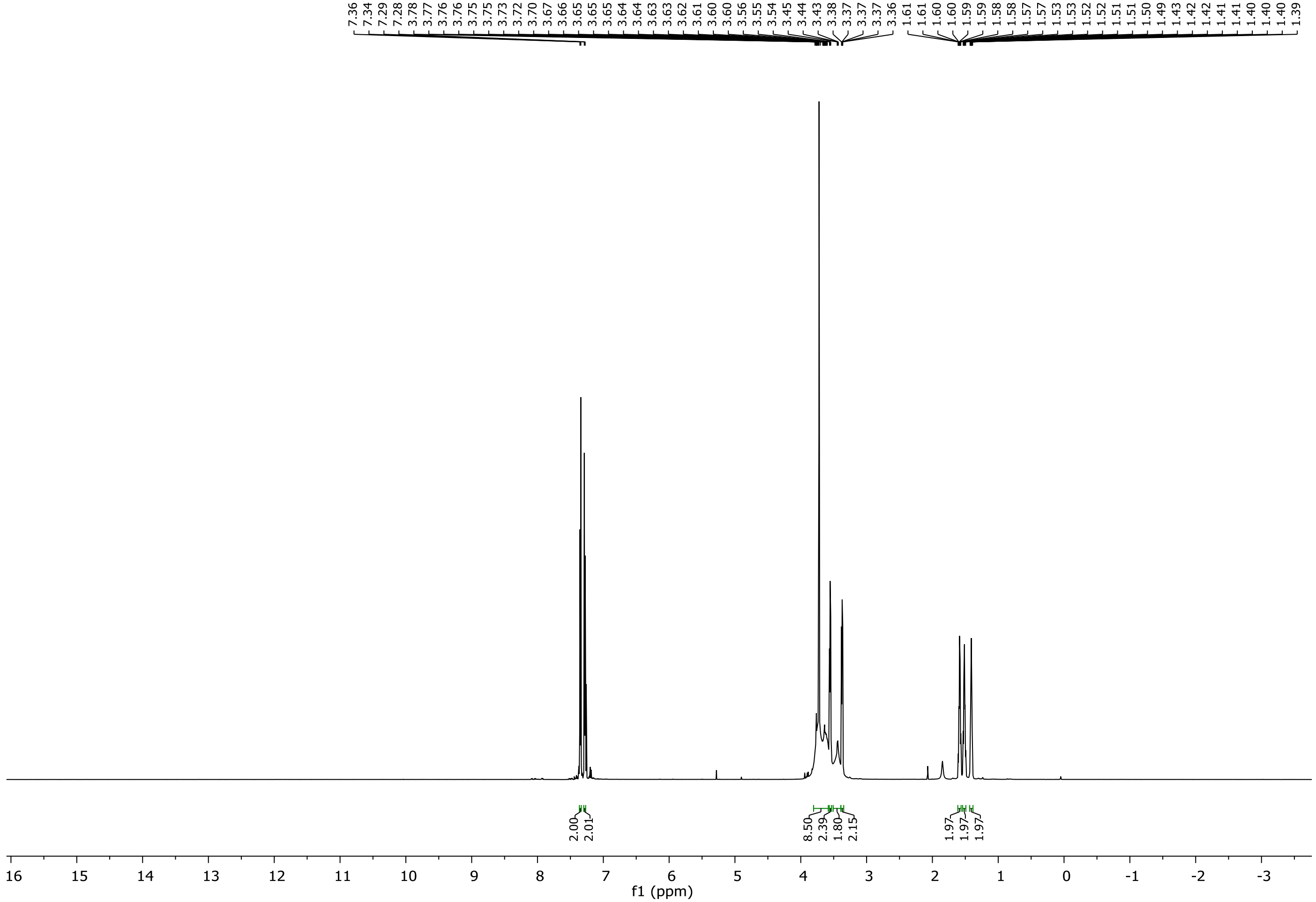

^{13}C NMR

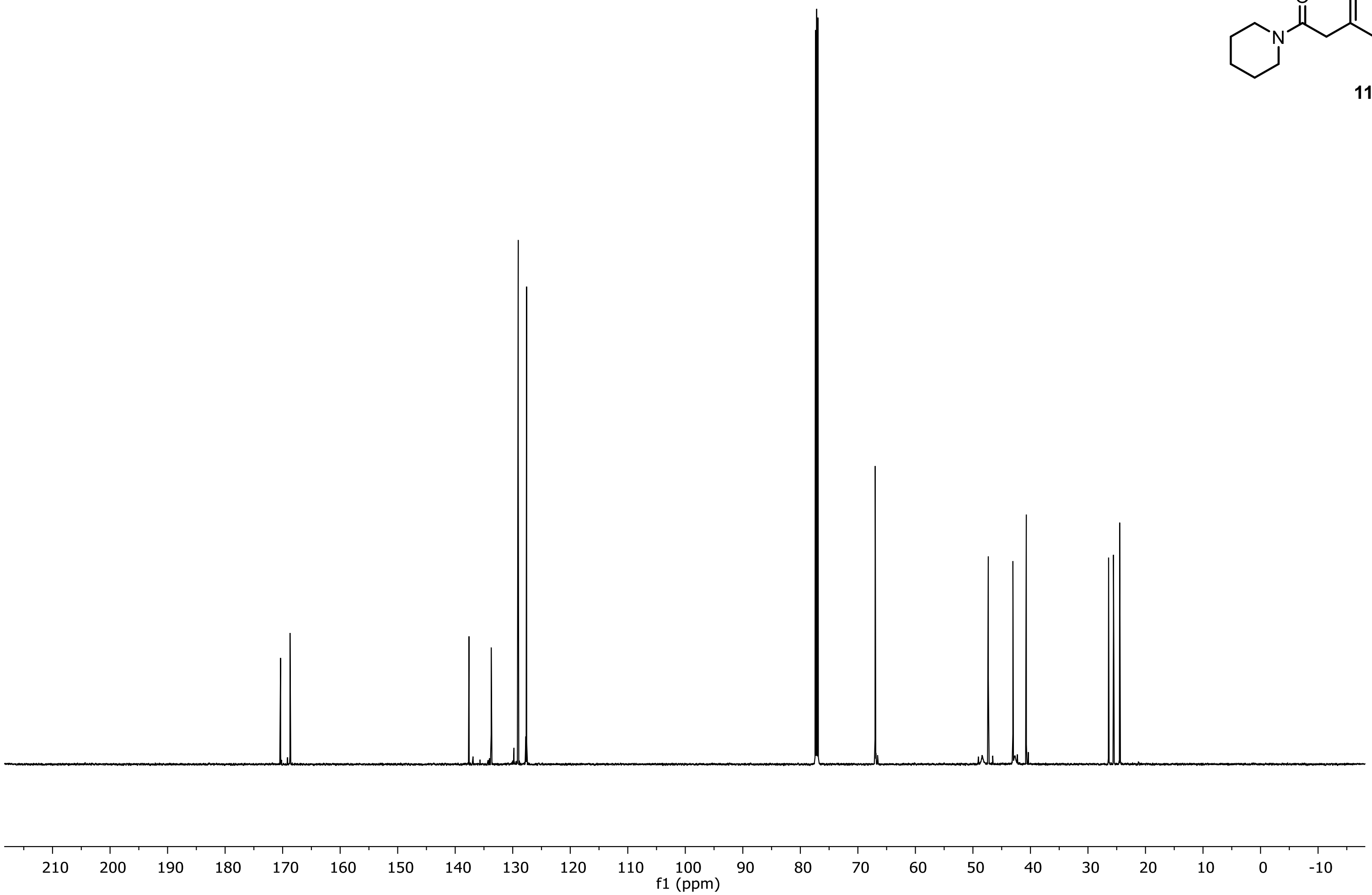
170.4
168.7
137.6
133.7
129.0
127.6
67.0
48.4
47.3
46.6
43.0
40.7
26.4
25.6
24.5
210 200 190 180 170 160 150 140 130 120 110 100 90 80 70 60 50 40 30 20 10 0 -10
f1 (ppm)

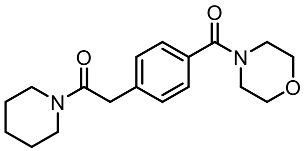

11

^{1}H, ^{1}H COSY

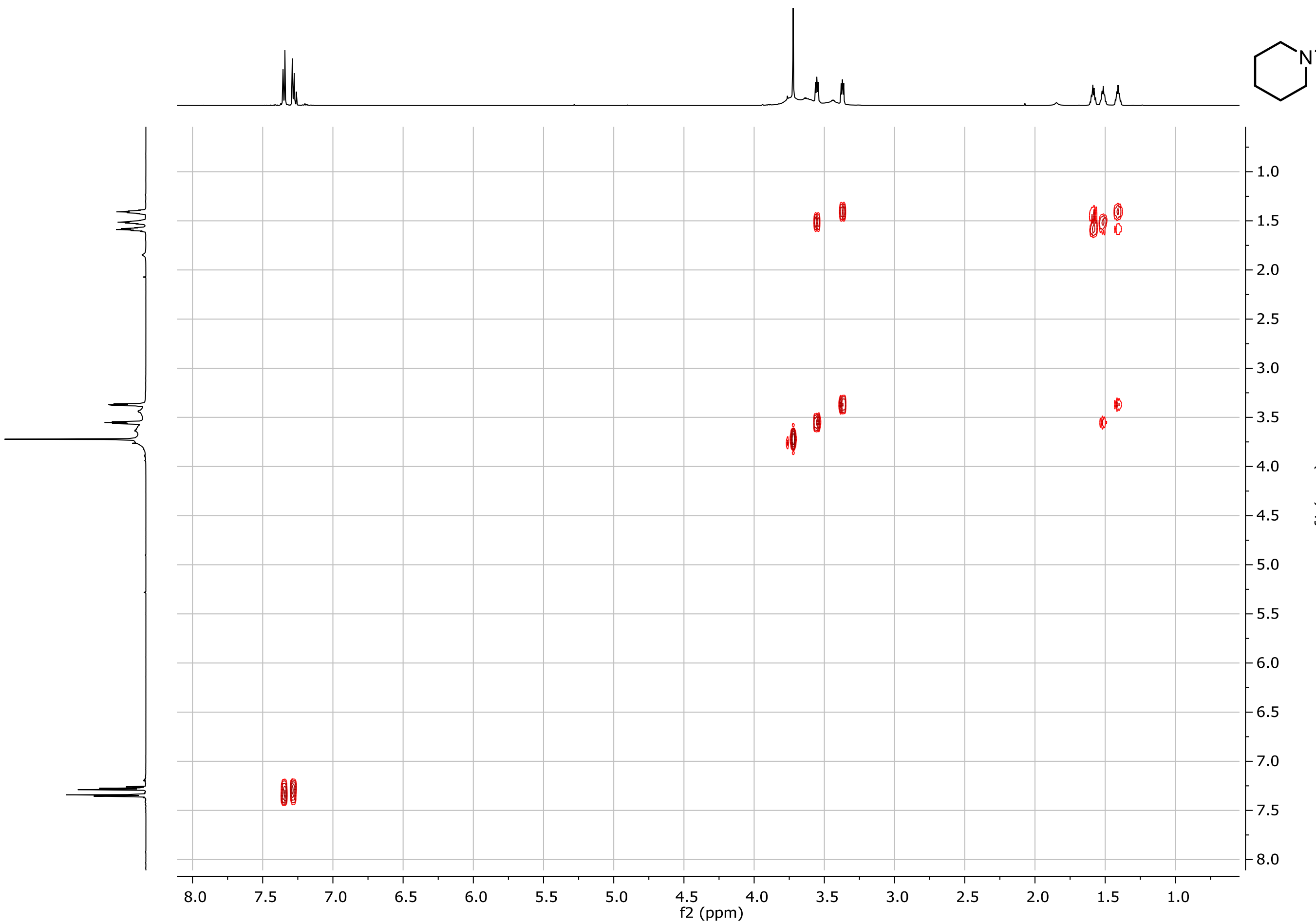

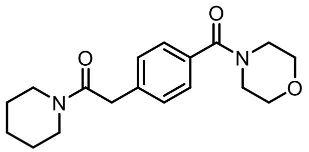

11

^{1}H, ^{13}C HMBC

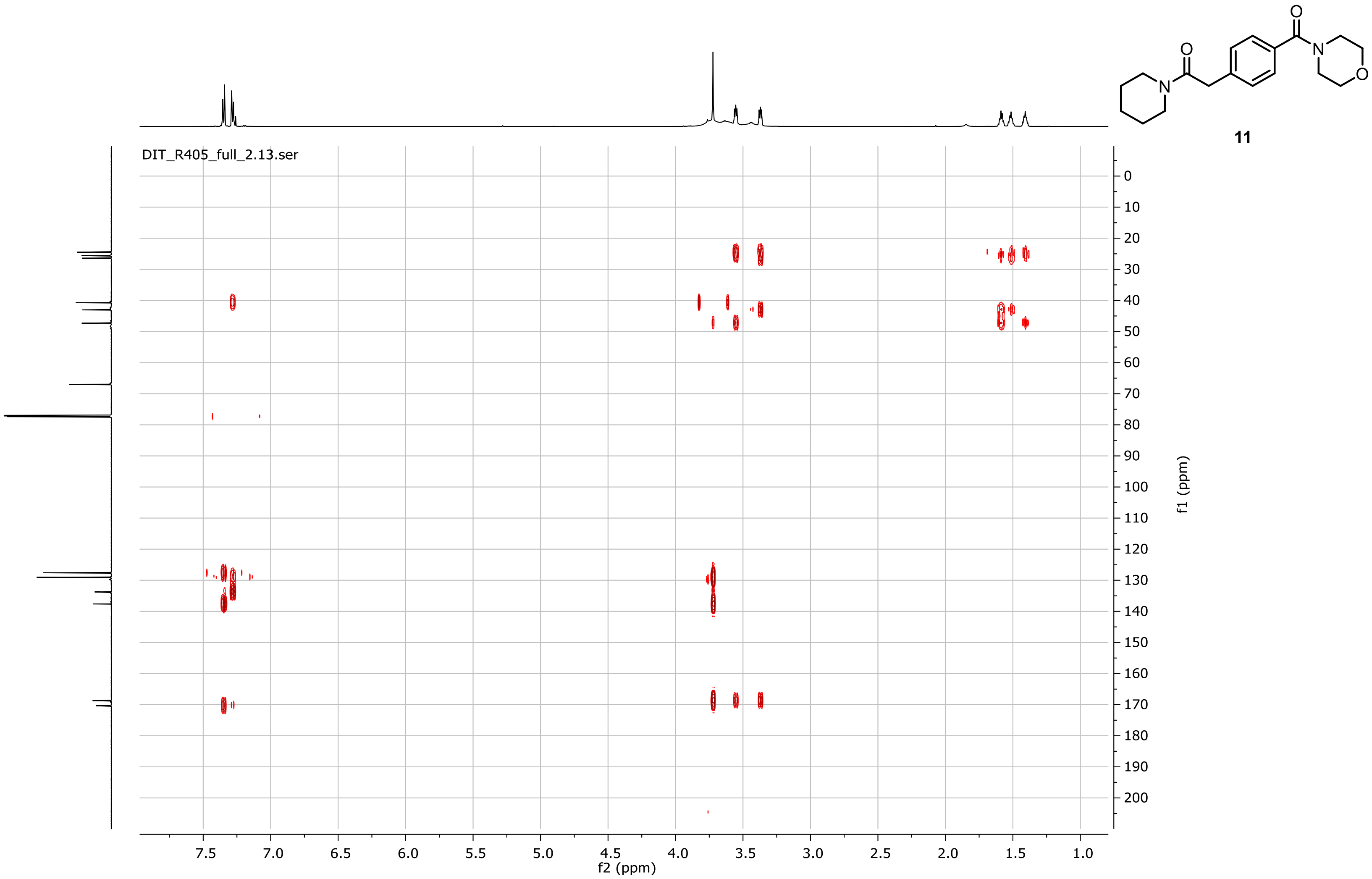

11

^{1}H, ^{13}C HSQC

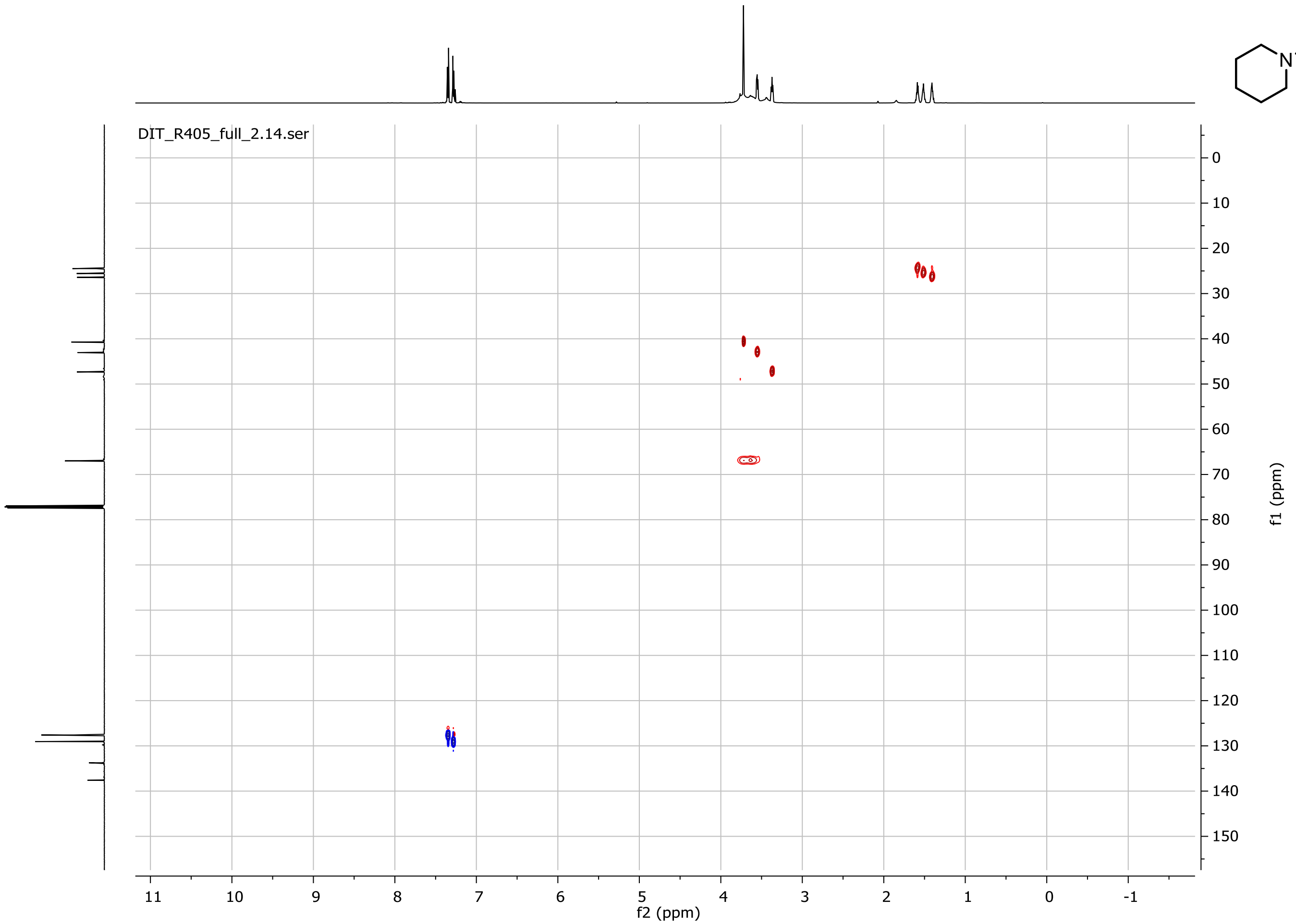

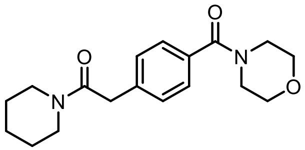

11

HRMS

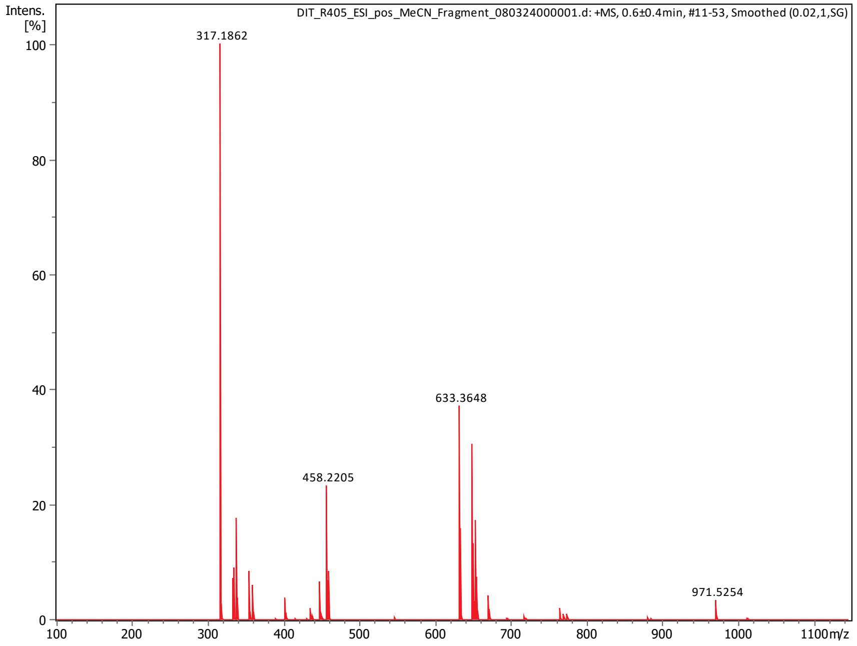

Intens. [%]
DIT_R405_ESI_pos_MeCN_Fragment_080324000001.d: +MS, 0.6±0.4min, #11-53, Smoothed (0.02,1,SG)
317.1862
633.3648
458.2205
971.5254
m/z

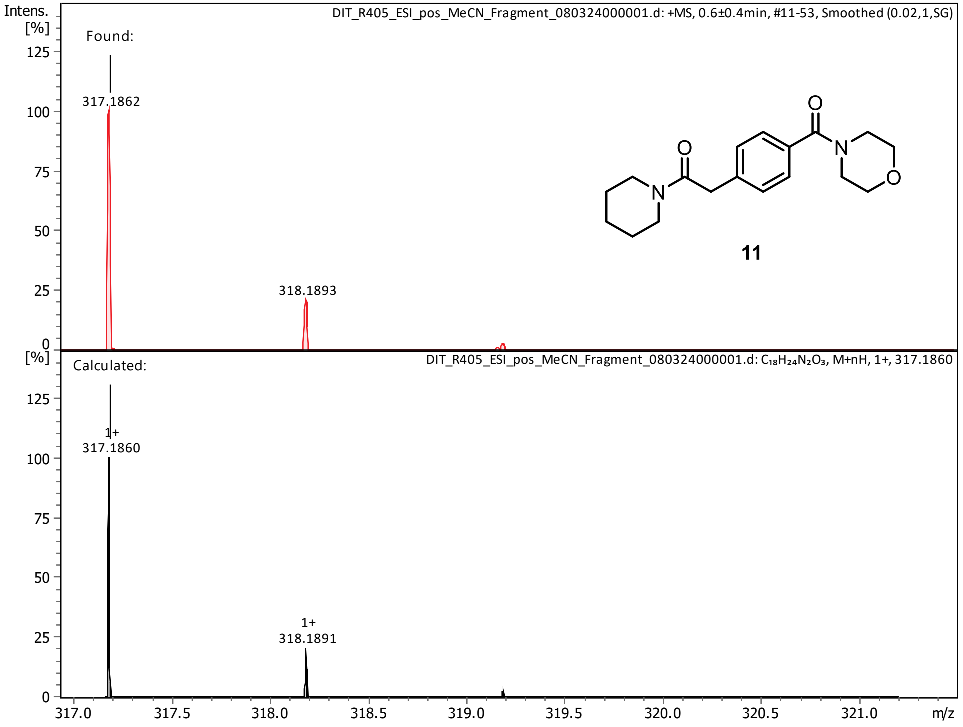

Intens. [%]
DIT_R405_ESI_pos_MeCN_Fragment_080324000001.d: +MS, 0.6±0.4min, #11-53, Smoothed (0.02,1,SG)
Found:
317.1862
318.1893
11
Calculated:
DIT_R405_ESI_pos_MeCN_Fragment_080324000001.d: $C_{18}H_{24}N_2O_3$, M+nH, 1+, 317.1860
1+
317.1860
1+
318.1891
m/z

IR

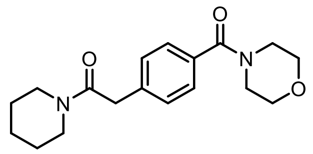

11

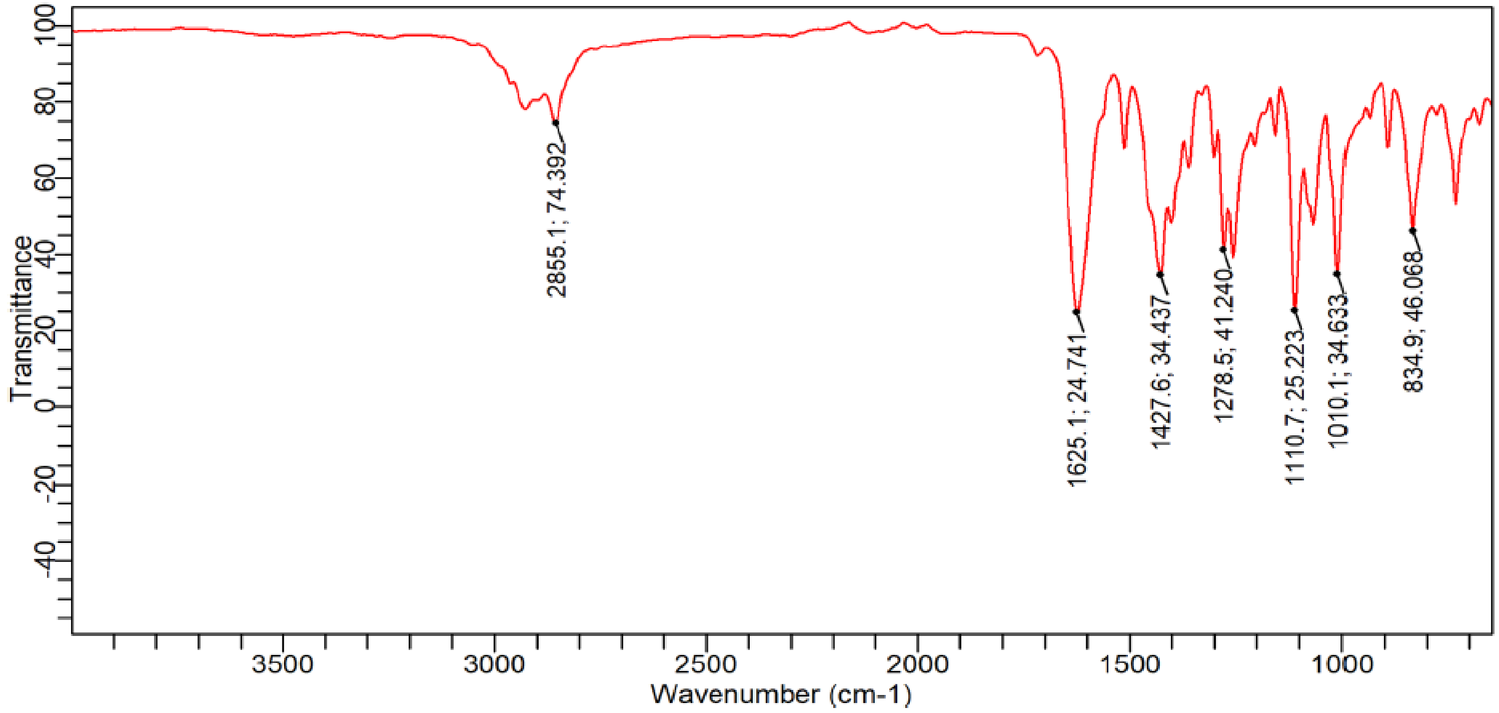
Transmittance
Wavenumber (cm-1)
2855.1; 74.392
1625.1; 24.741
1427.6; 34.437
1278.5; 41.240
1110.7; 25.223
1010.1; 34.633
834.9; 46.068

34 3-(4-Benzoylpiperazin-1-yl)-1-(1-piperidyl)propan-1-one (13)

^{1}H NMR

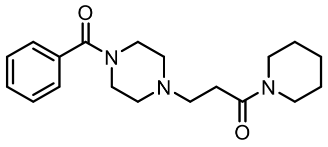

13

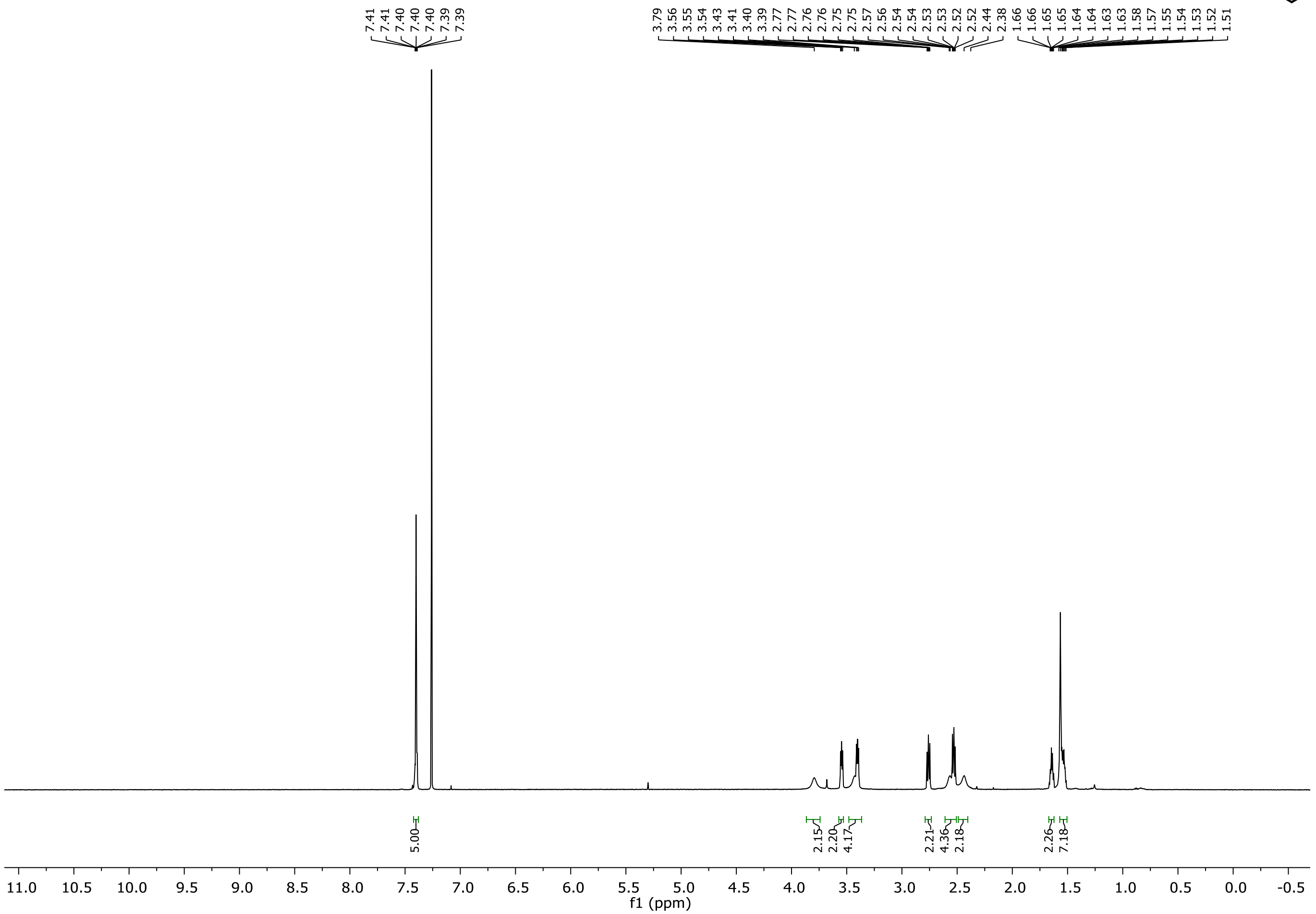

^{13}C NMR

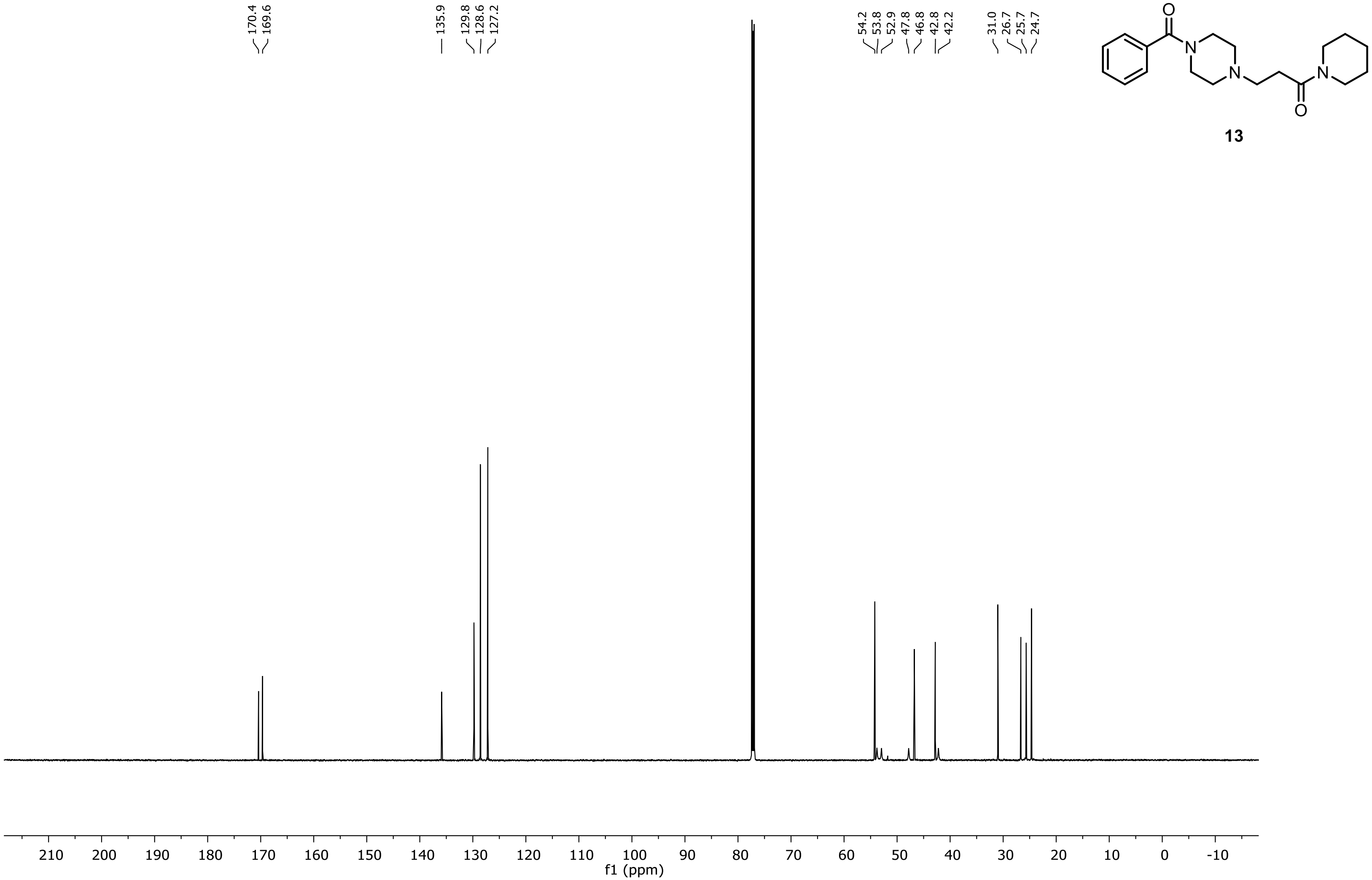
170.4
169.6
135.9
129.8
128.6
127.2
54.2
53.8
52.9
47.8
46.8
42.8
42.2
31.0
26.7
25.7
24.7
210 200 190 180 170 160 150 140 130 120 110 100 90 80 70 60 50 40 30 20 10 0 -10
f1 (ppm)

13

^{1}H, ^{1}H COSY

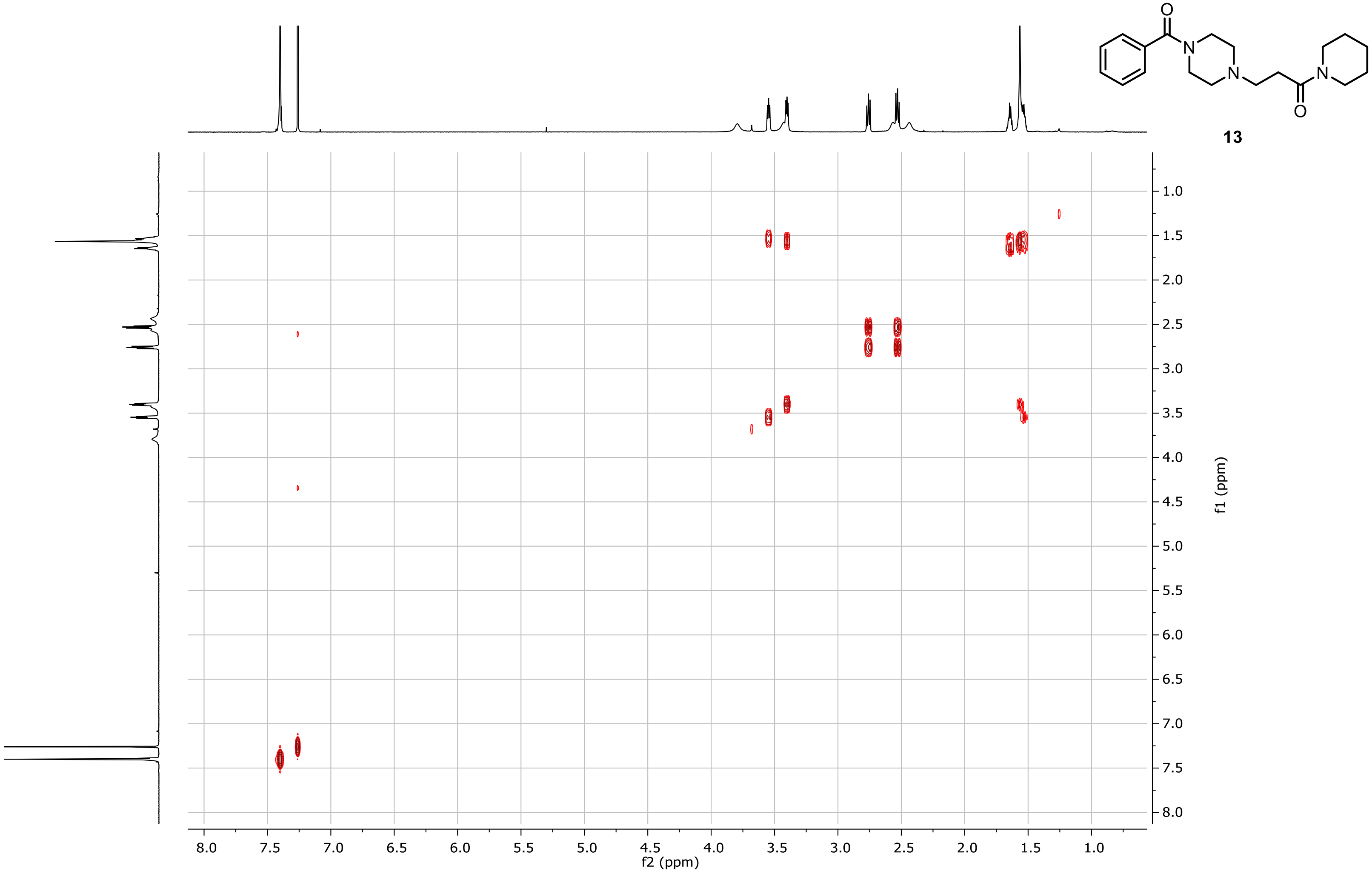

13

^{1}H, ^{13}C HMBC

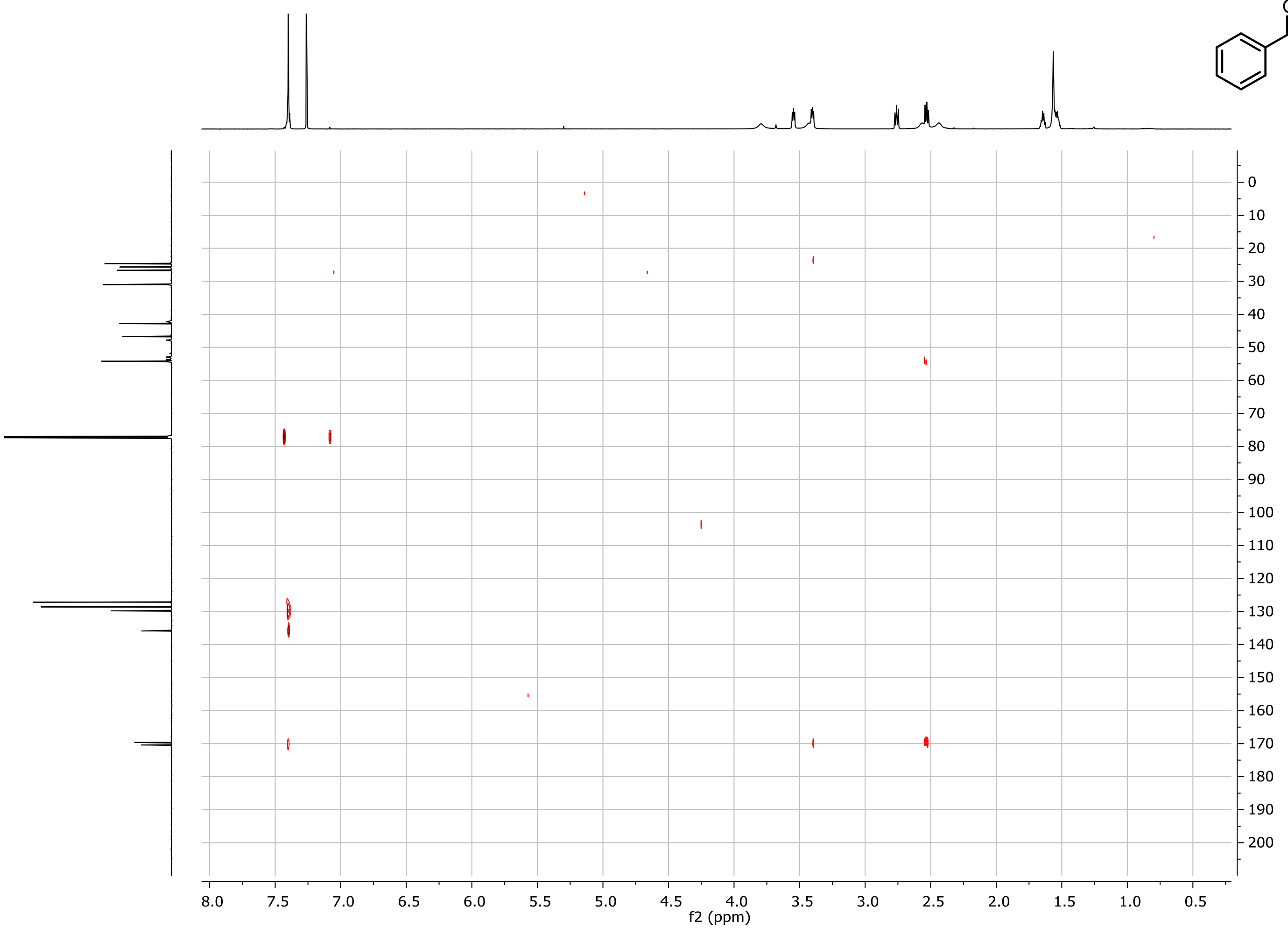

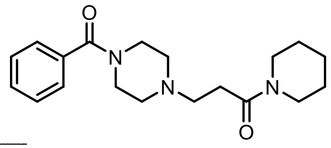

13

^{1}H, ^{13}C HSQC

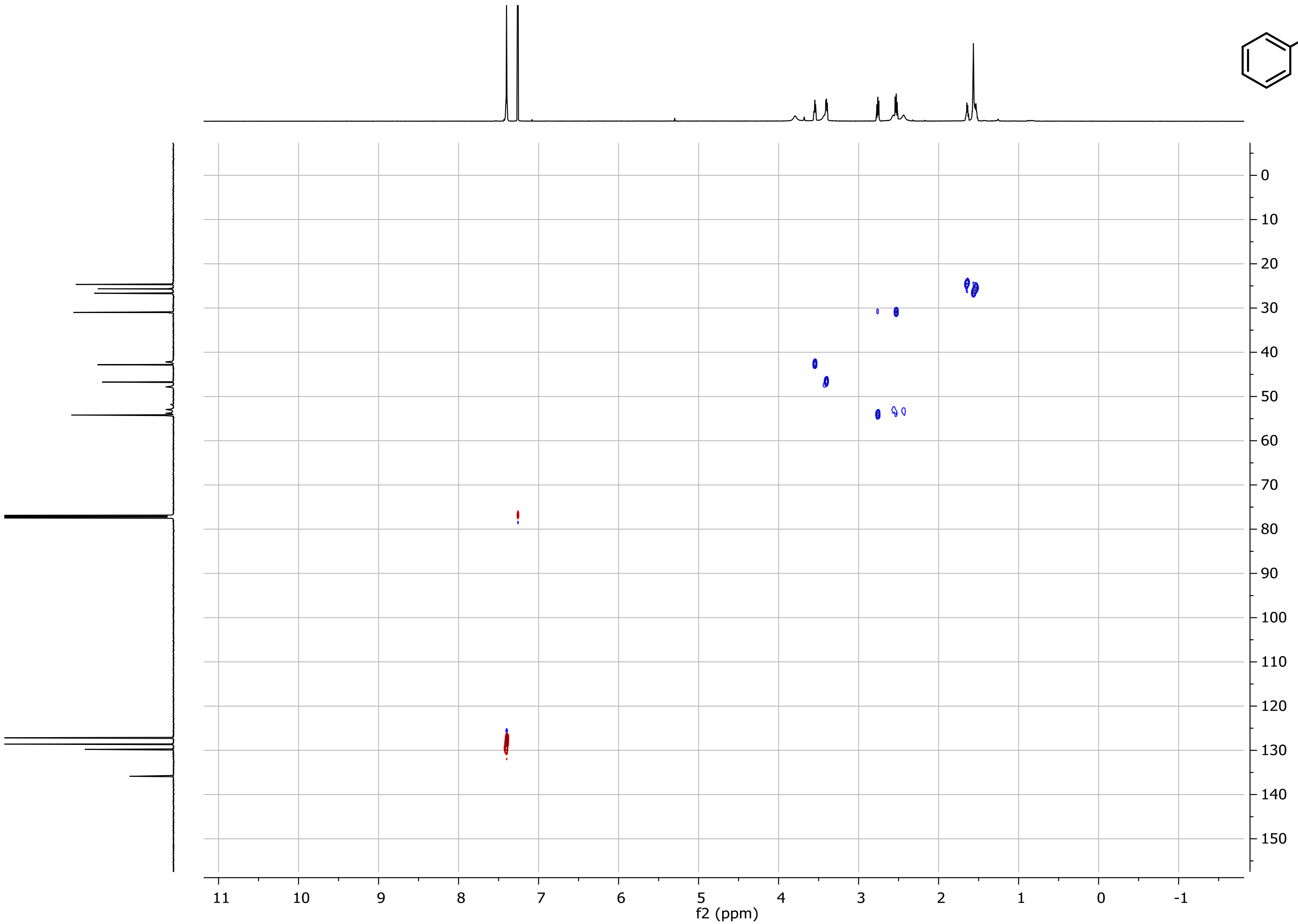

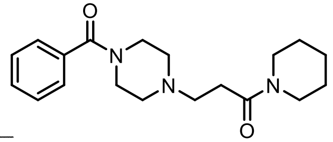

13

HRMS

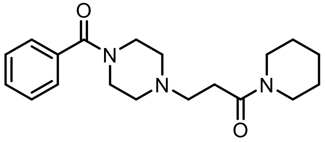

13

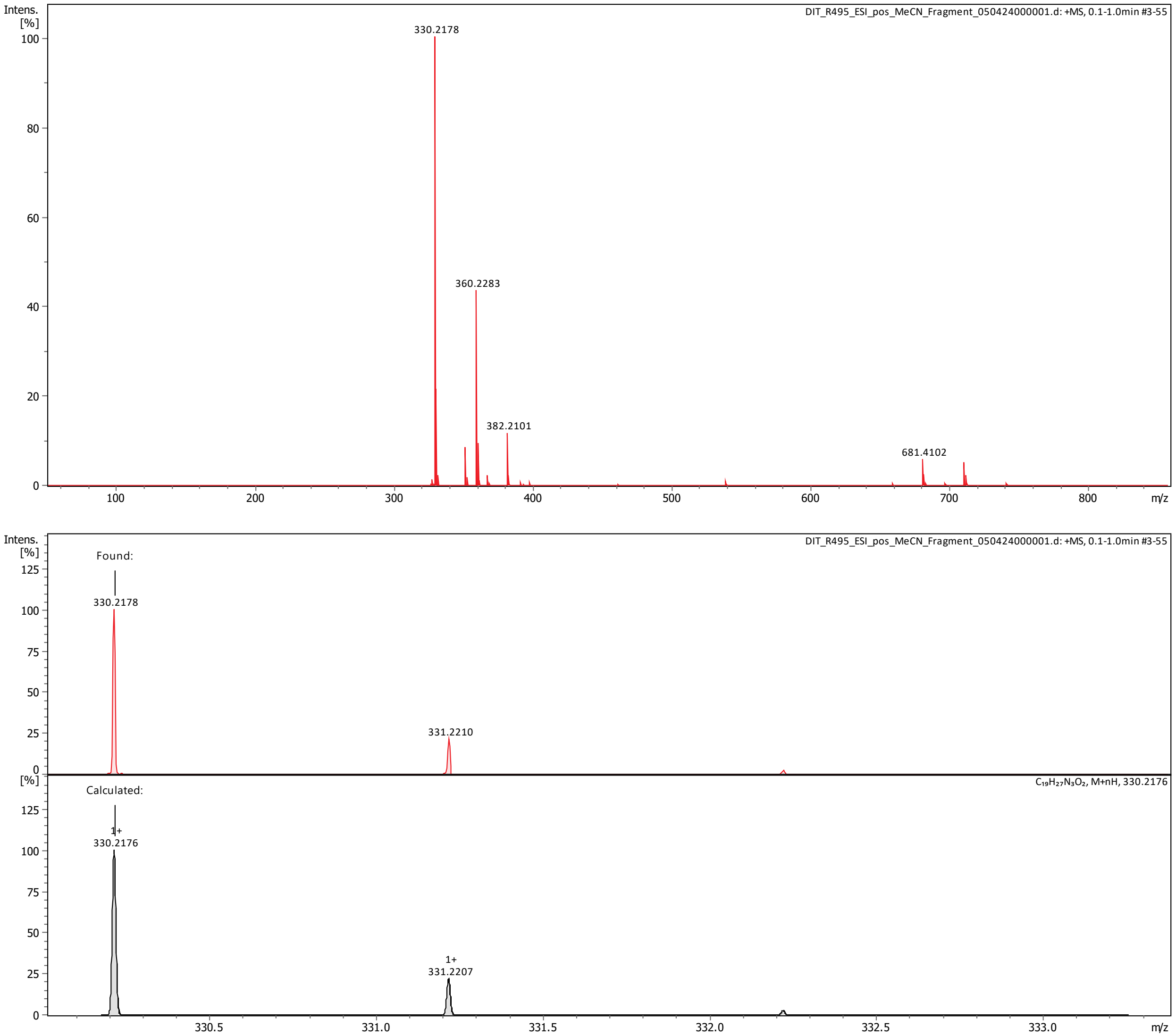

Intens. [%]
DIT_R495_ESI_pos_MeCN_Fragment_050424000001.d: +MS, 0.1-1.0min #3-55
330.2178
360.2283
382.2101
681.4102
m/z
Found:
330.2178
331.2210
Calculated:
$C_{19}H_{27}N_3O_2$, M+nH, 330.2176
1+
330.2176
1+
331.2207

IR

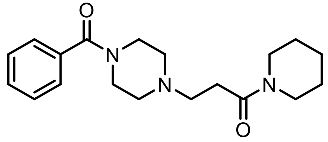

13

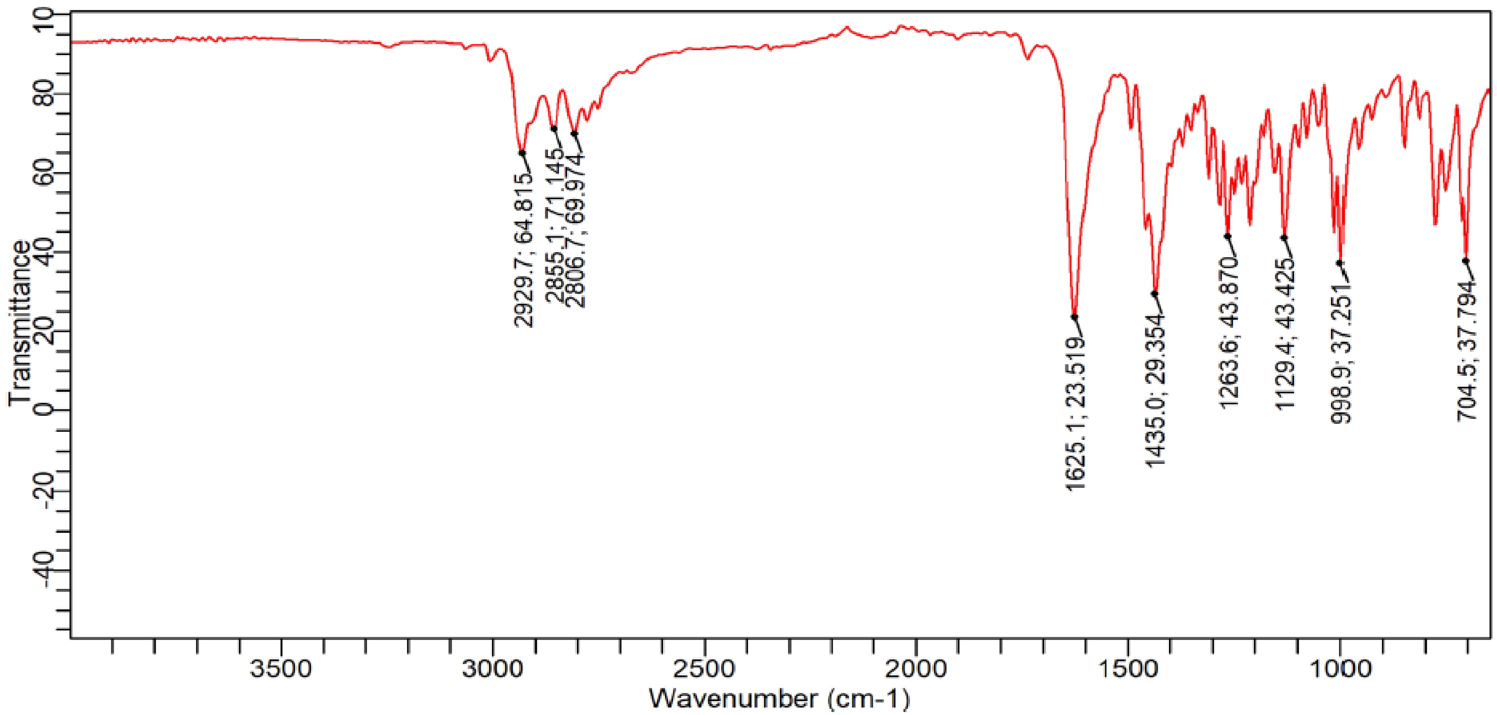
Transmittance
Wavenumber (cm-1)
2929.7; 64.815
2855.1; 71.145
2806.7; 69.974
1625.1; 23.519
1435.0; 29.354
1263.6; 43.870
1129.4; 43.425
998.9; 37.251
704.5; 37.794

35 (4-(Morpholine-4-carbonyl)phenyl)(piperidin-1-yl)methanone (16c)

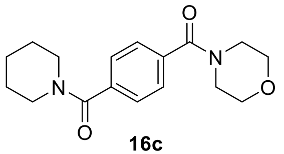

16c

H NMR

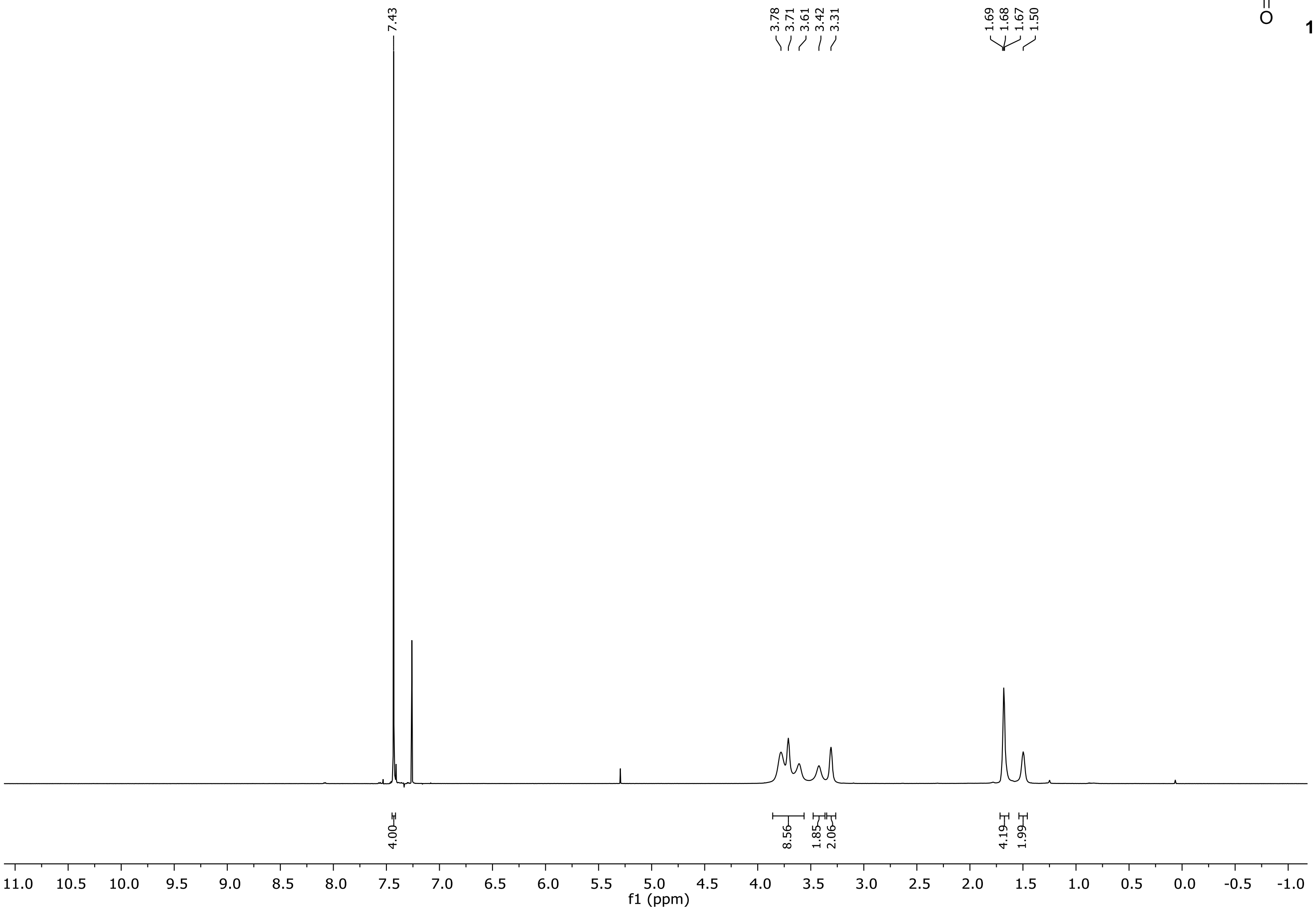

^{13}C NMR

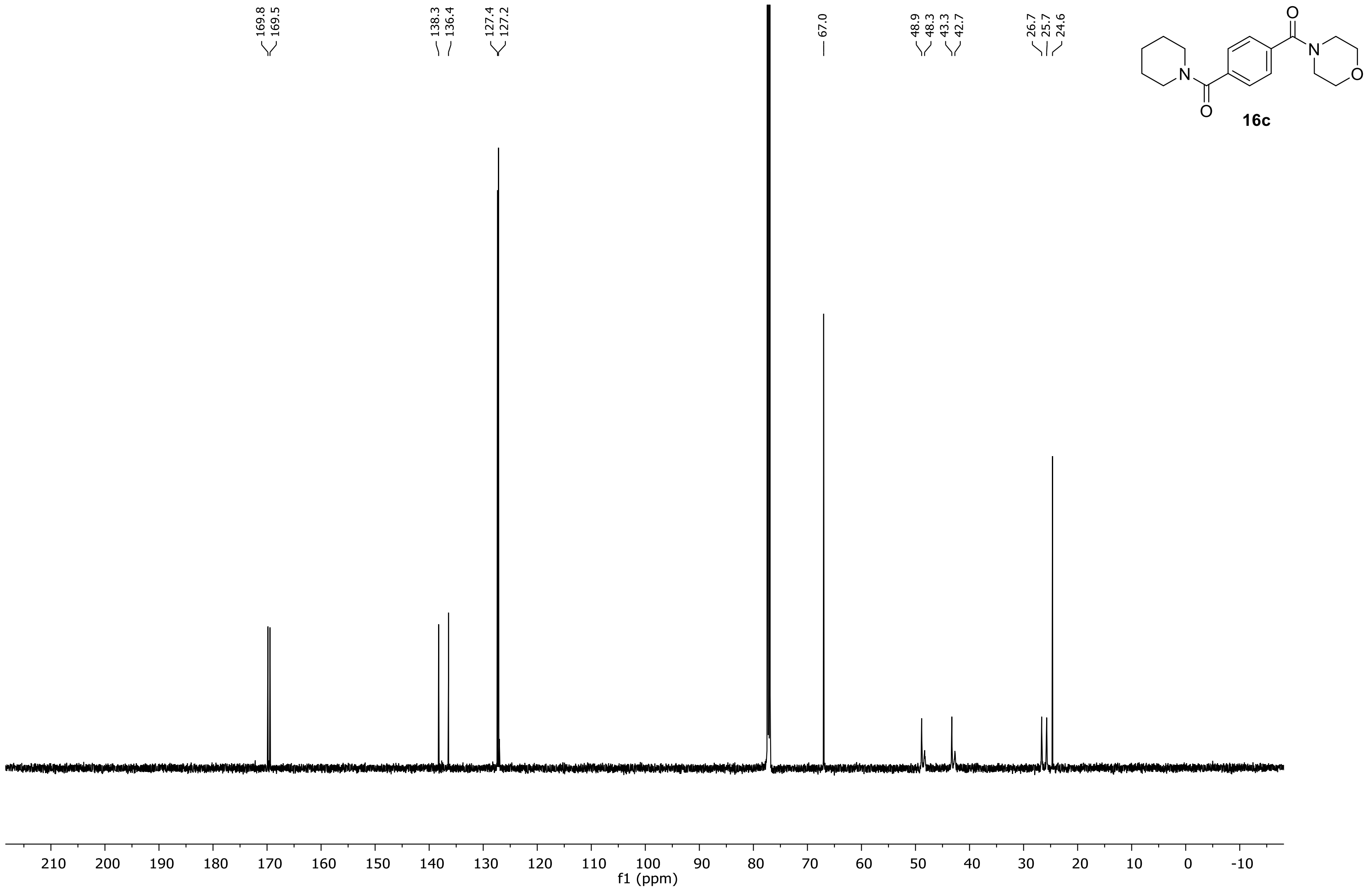
169.8
169.5
138.3
136.4
127.4
127.2
67.0
48.9
48.3
43.3
42.7
26.7
25.7
24.6
210 200 190 180 170 160 150 140 130 120 110 100 90 80 70 60 50 40 30 20 10 0 -10
f1 (ppm)
N
O
N
O
O

16c

^1H, ^1H COSY

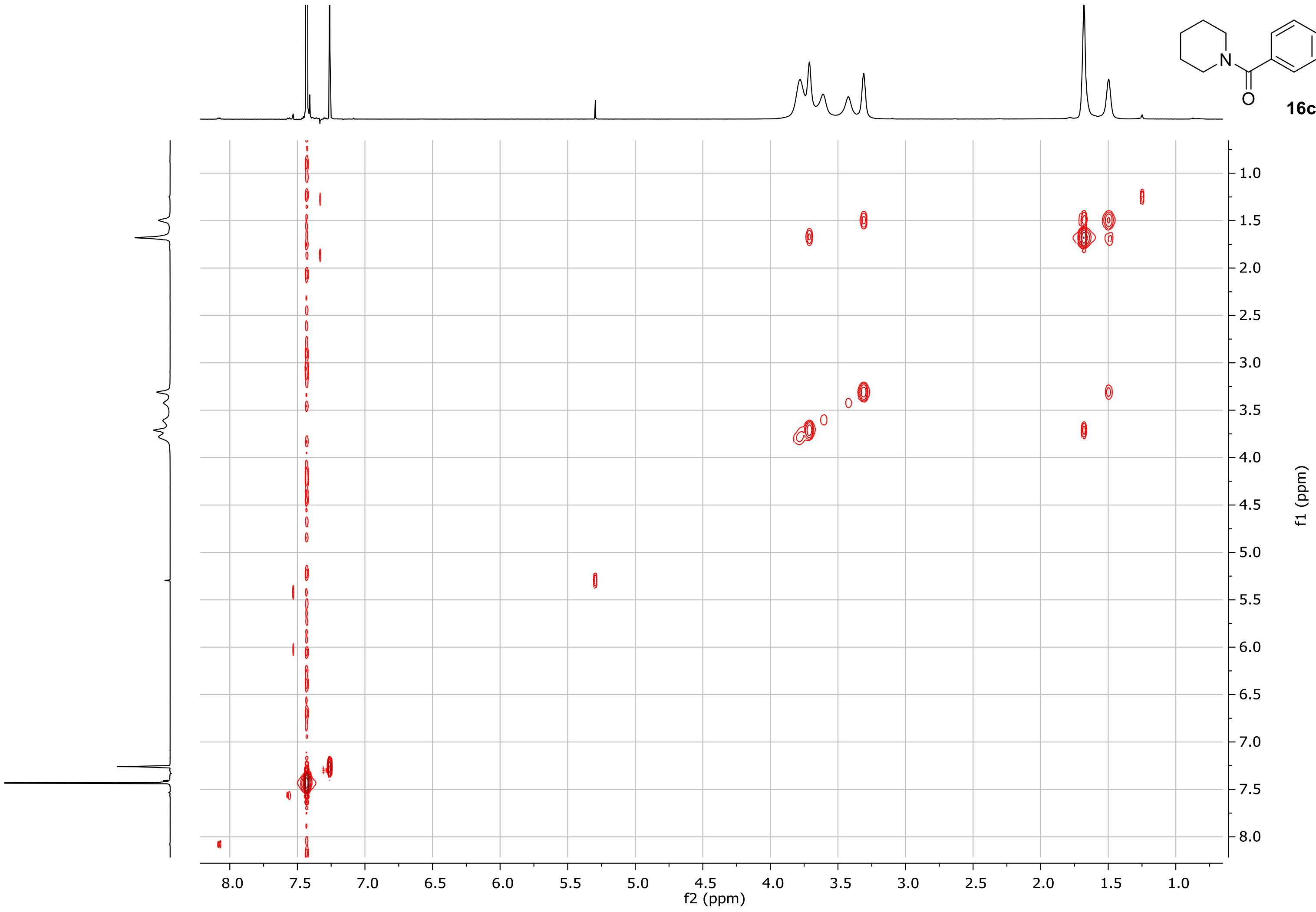

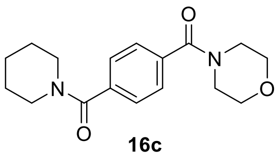

16c

^{1}H, ^{13}C HMBC

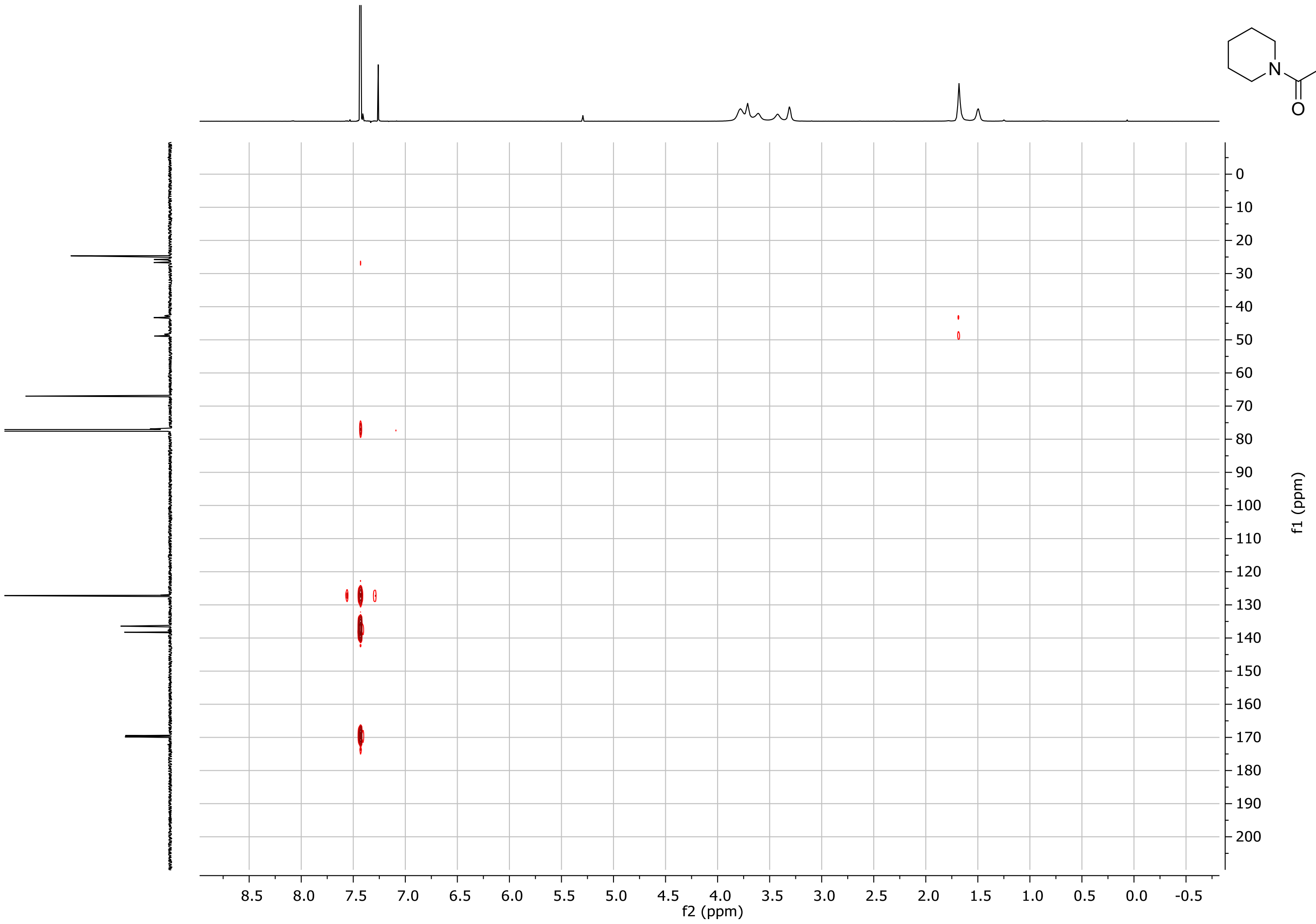

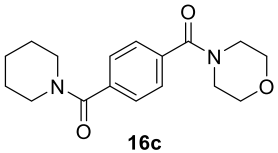

16c

^{1}H, ^{13}C HSQC

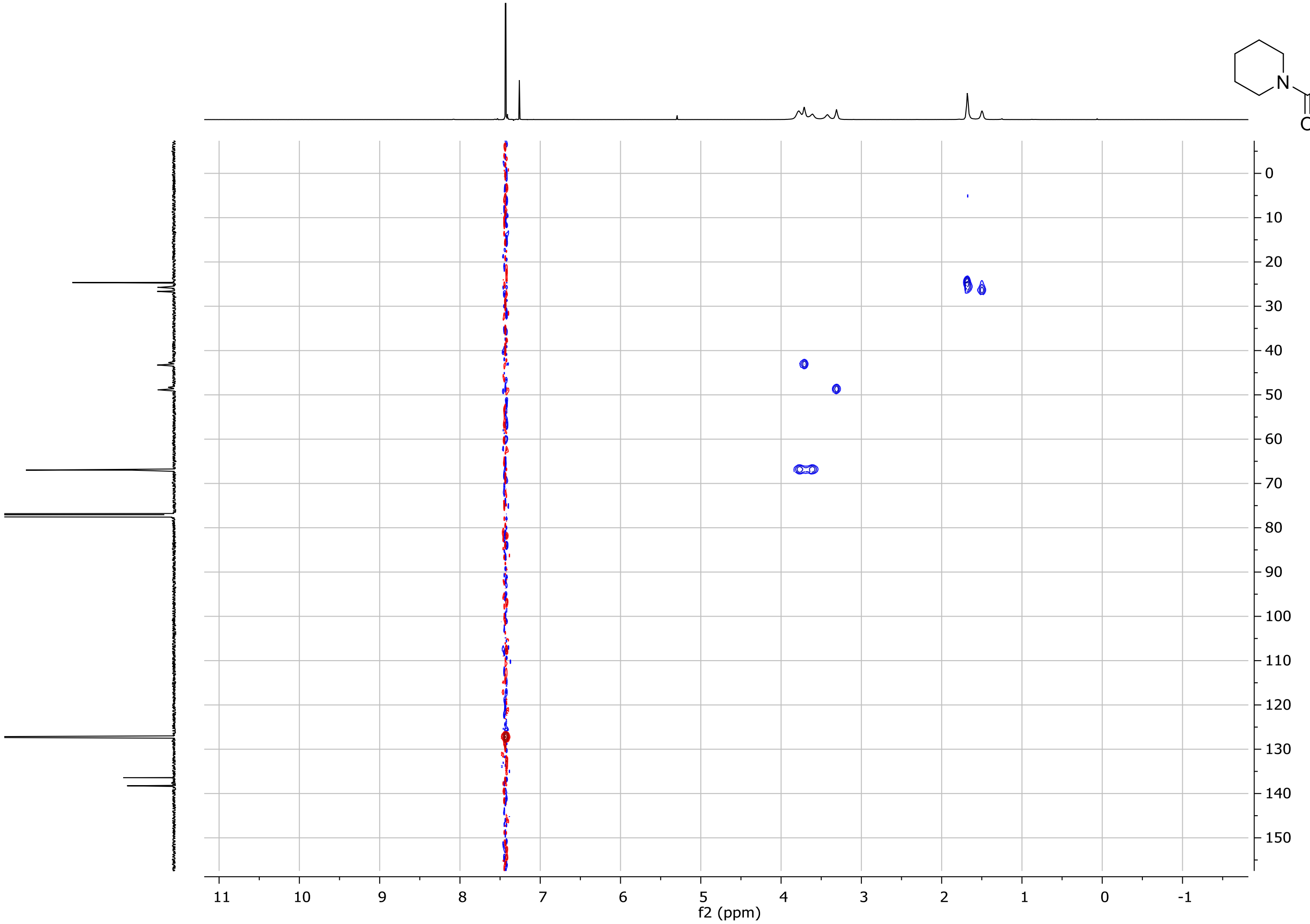

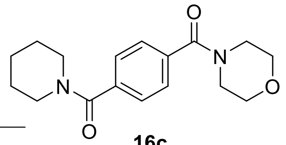

16c

HRMS

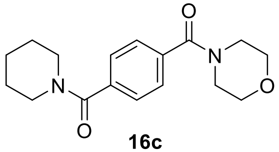

N
O
O
N
O

16c

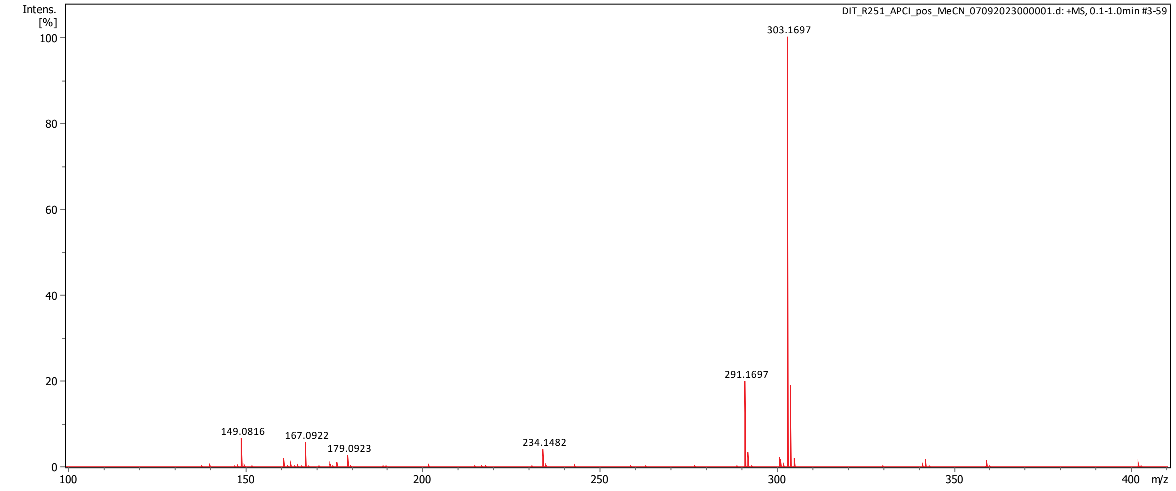

Intens.
[%]
DIT_R251_APCI_pos_MeCN_07092023000001.d: +MS, 0.1-1.0min #3-59
303.1697
291.1697
149.0816
167.0922
179.0923
234.1482
m/z

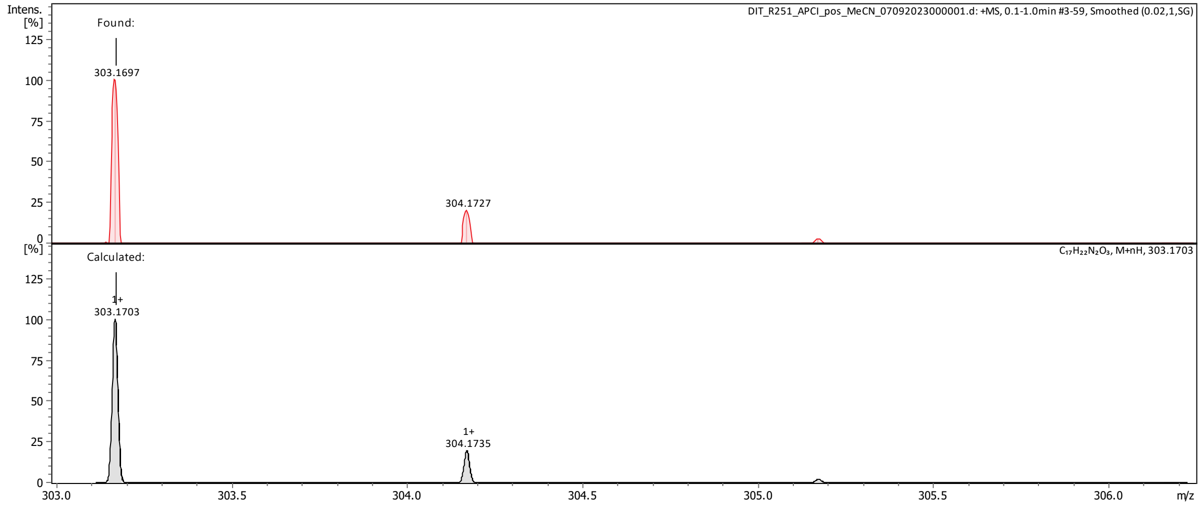

Intens.
[%]
Found:
303.1697
304.1727
DIT_R251_APCI_pos_MeCN_07092023000001.d: +MS, 0.1-1.0min #3-59, Smoothed (0.02,1,SG)
Calculated:
1+
303.1703
1+
304.1735
C17H22N2O3, M+nH, 303.1703
m/z

IR

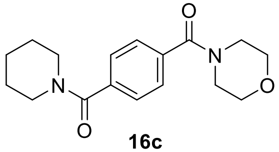

16c

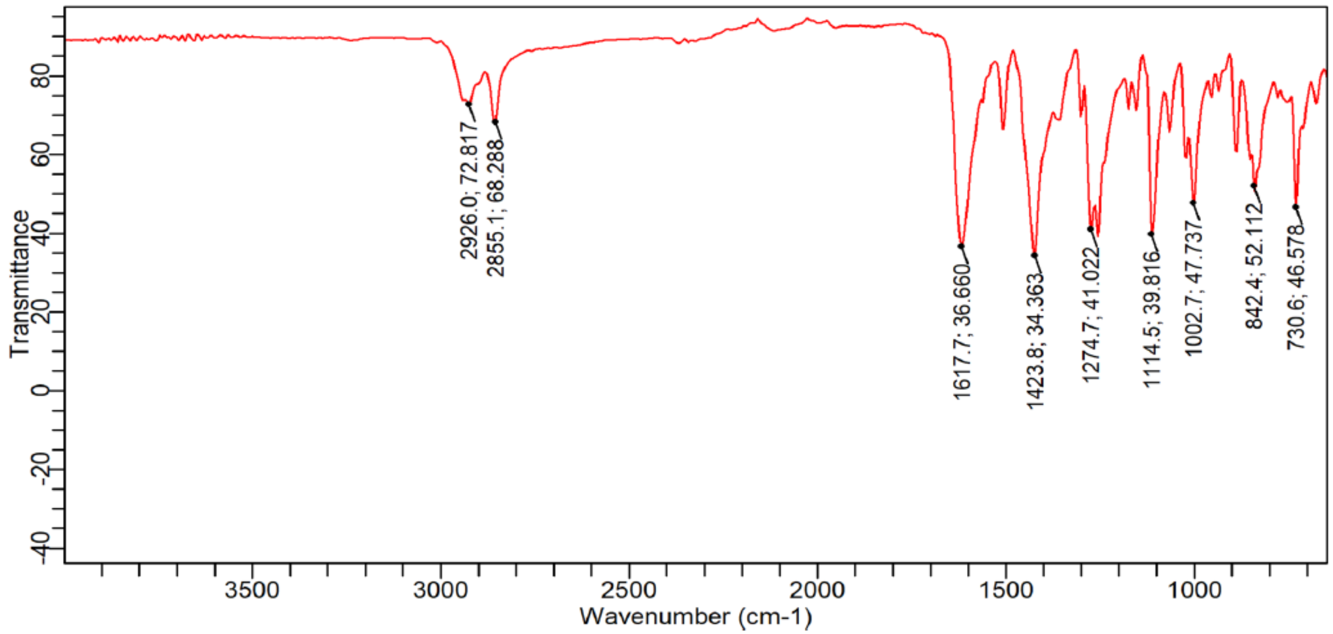

Transmittance
Wavenumber (cm-1)
2926.0; 72.817
2855.1; 68.288
1617.7; 36.660
1423.8; 34.363
1274.7; 41.022
1114.5; 39.816
1002.7; 47.737
842.4; 52.112
730.6; 46.578

36 *N,N*-Diethyl-4-(morpholine-4-carbonyl)benzamide (16a)

^{1}H NMR

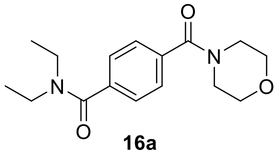

16a

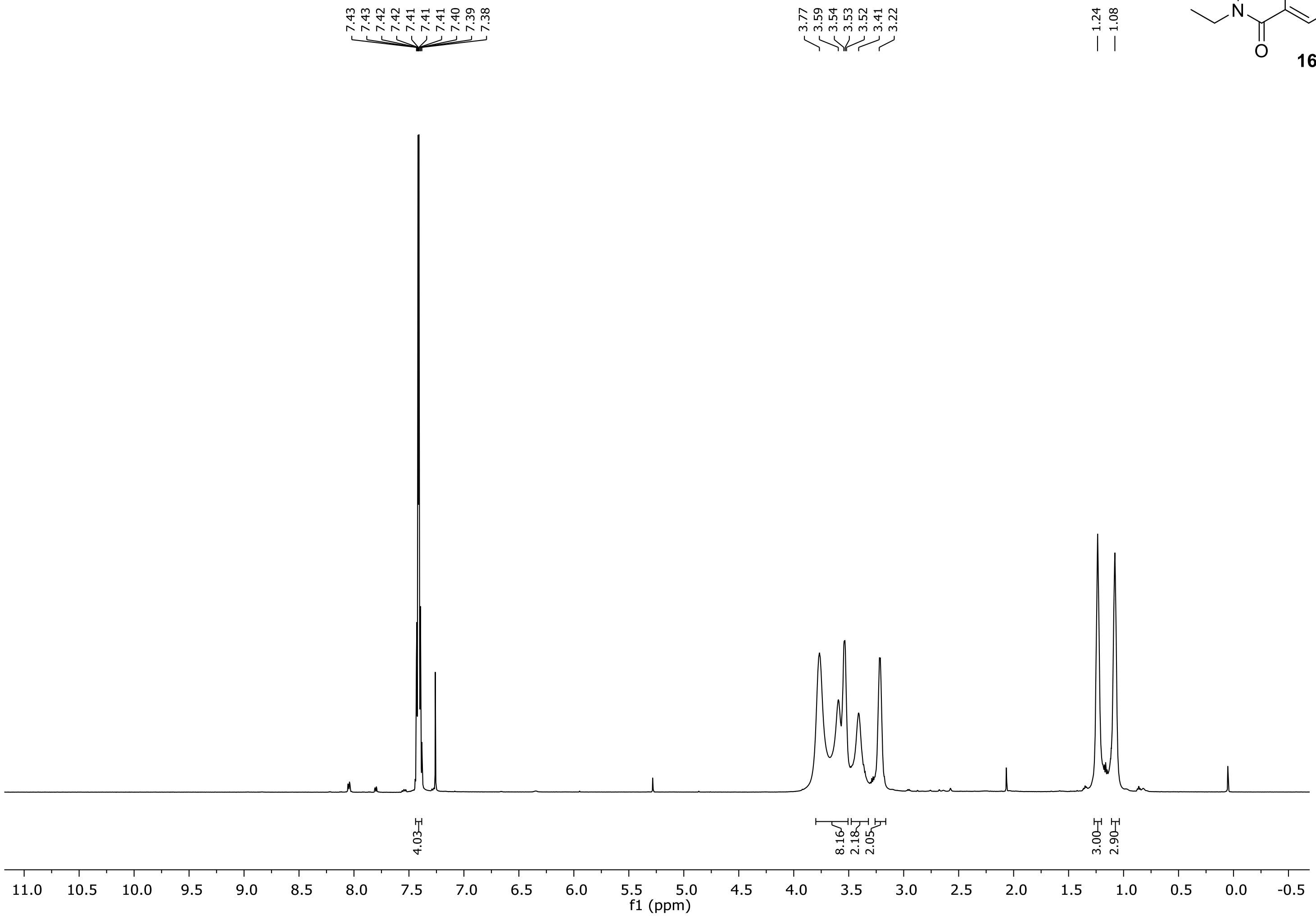

^{13}C NMR

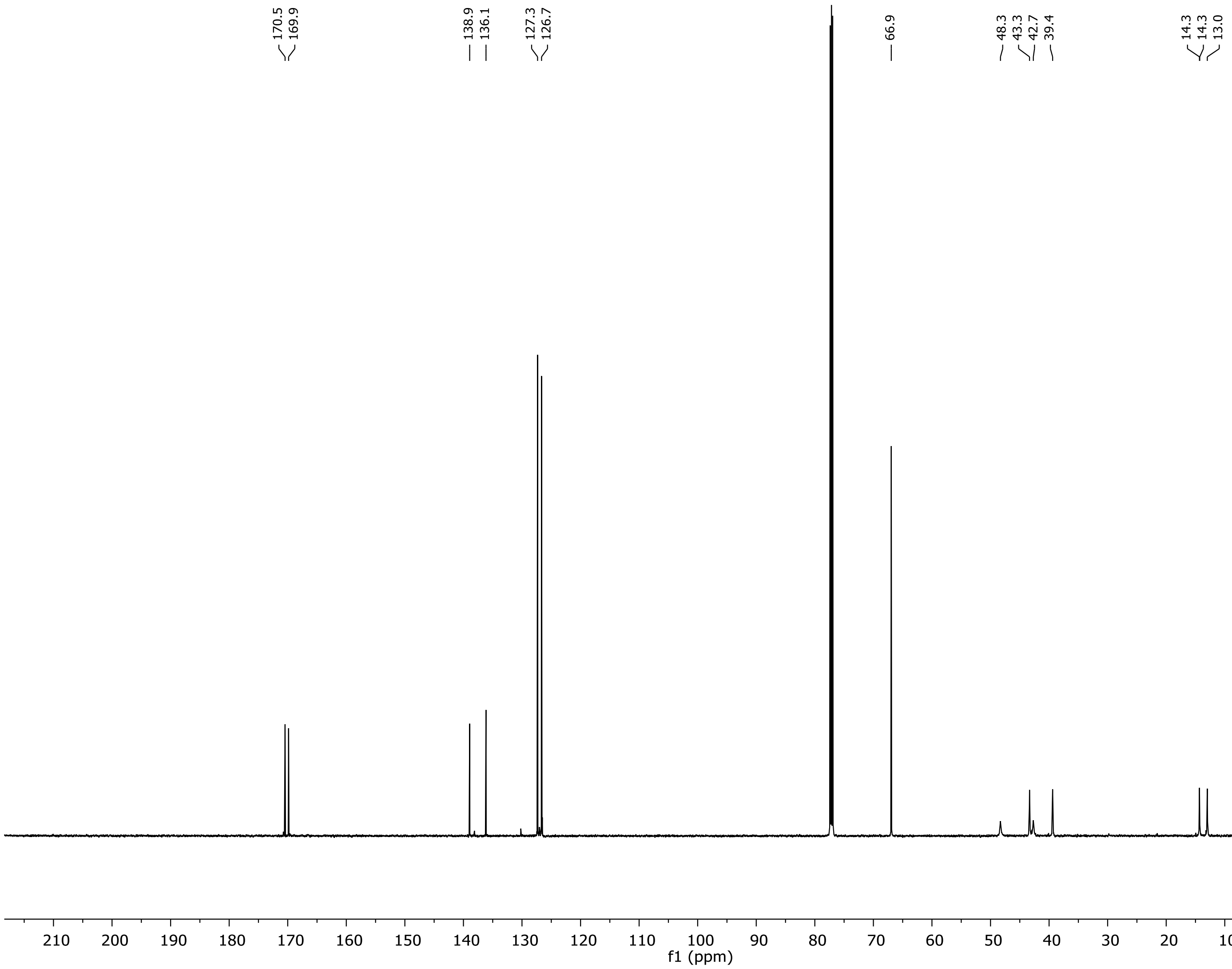
170.5
169.9
138.9
136.1
127.3
126.7
66.9
48.3
43.3
42.7
39.4
14.3
14.3
13.0
210 200 190 180 170 160 150 140 130 120 110 100 90 80 70 60 50 40 30 20 10 0 -10
f1 (ppm)

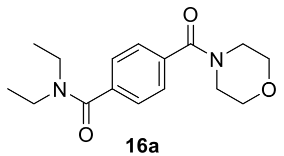
O
N
O
N
O

16a

^{1}H, ^{1}H COSY

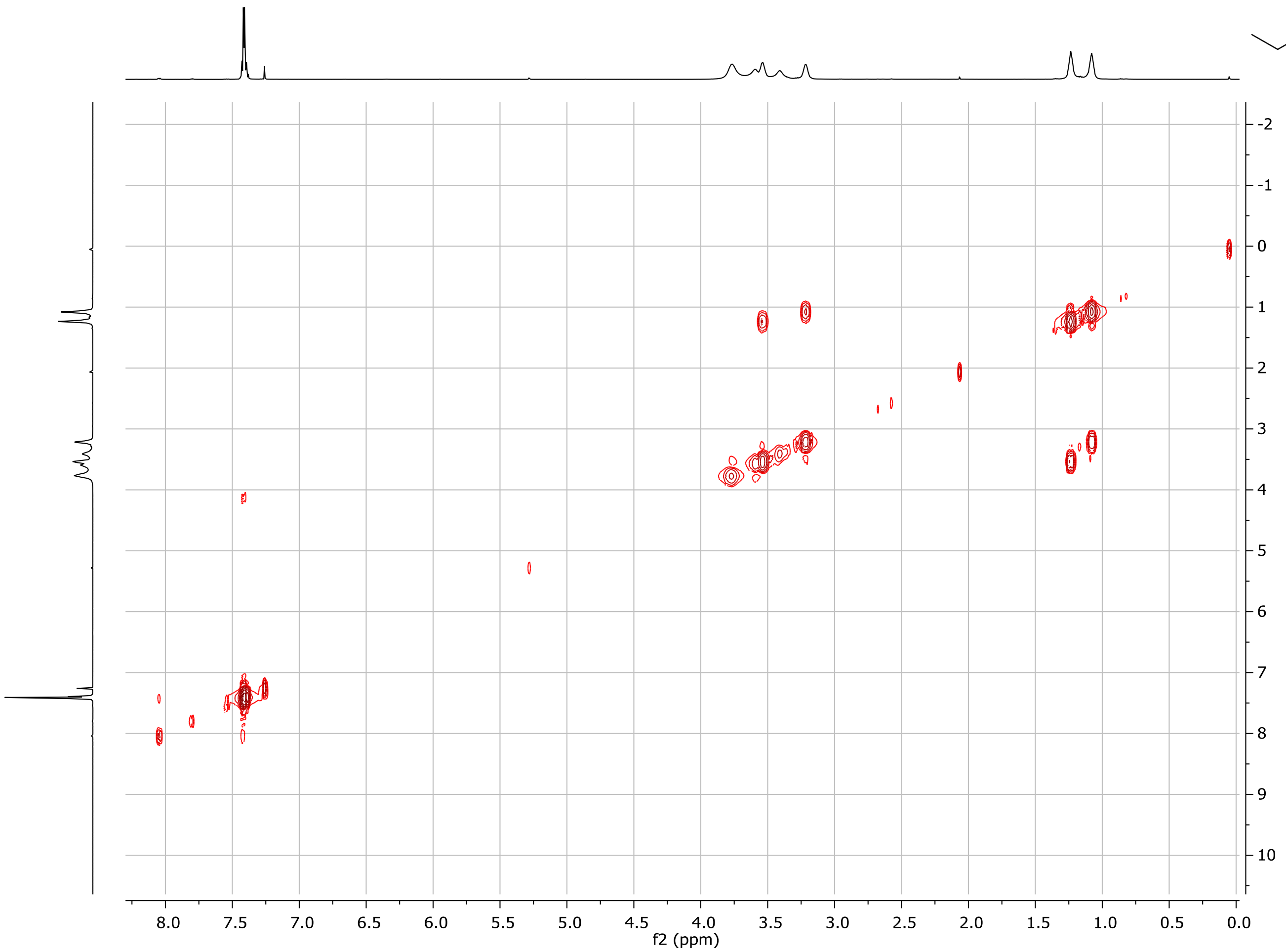

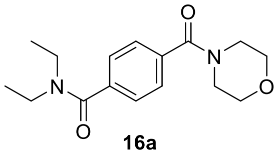

16a

^{1}H, ^{13}C HMBC

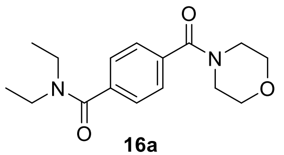

16a

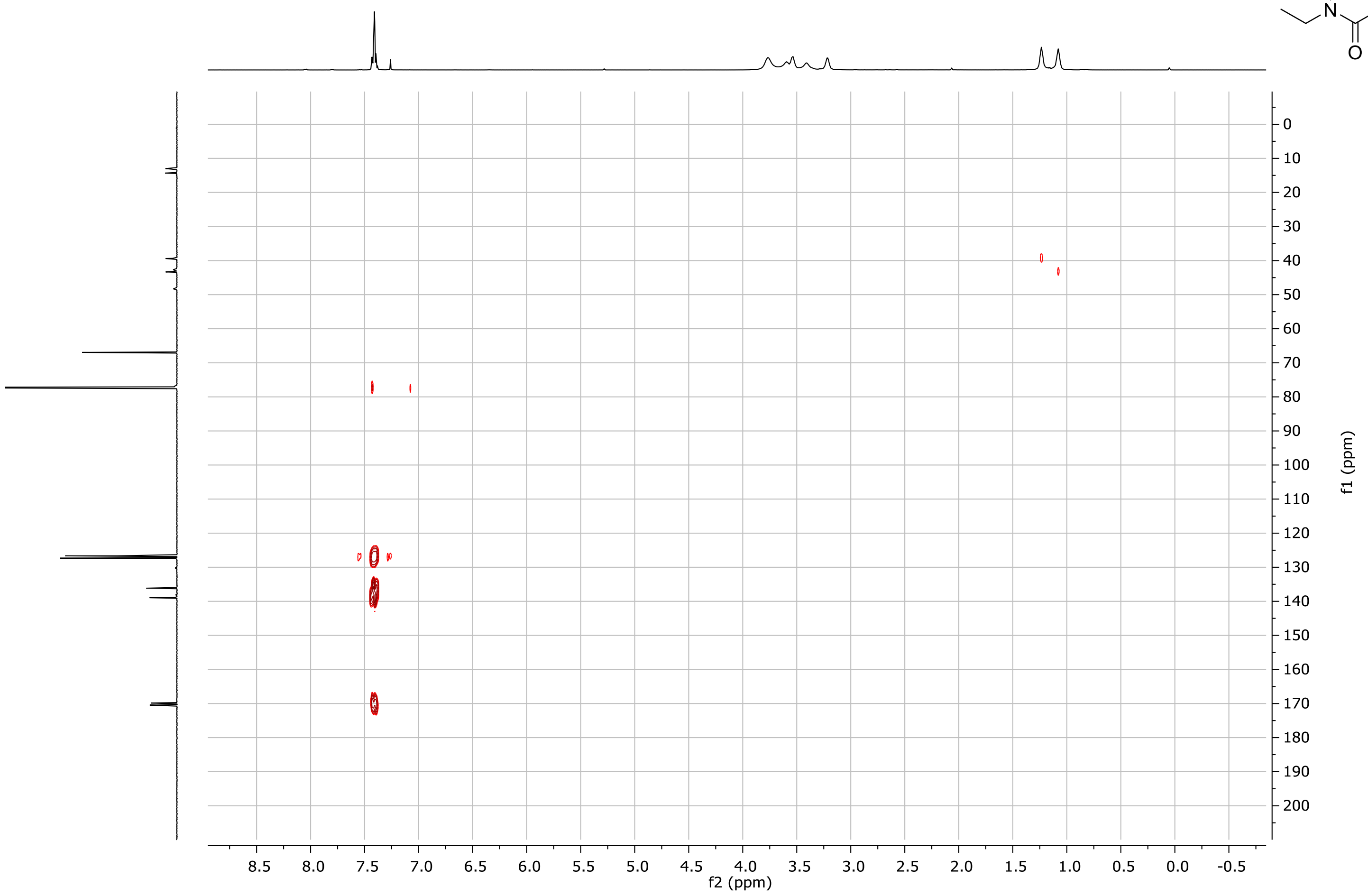

^1H, ^{13}C HSQC

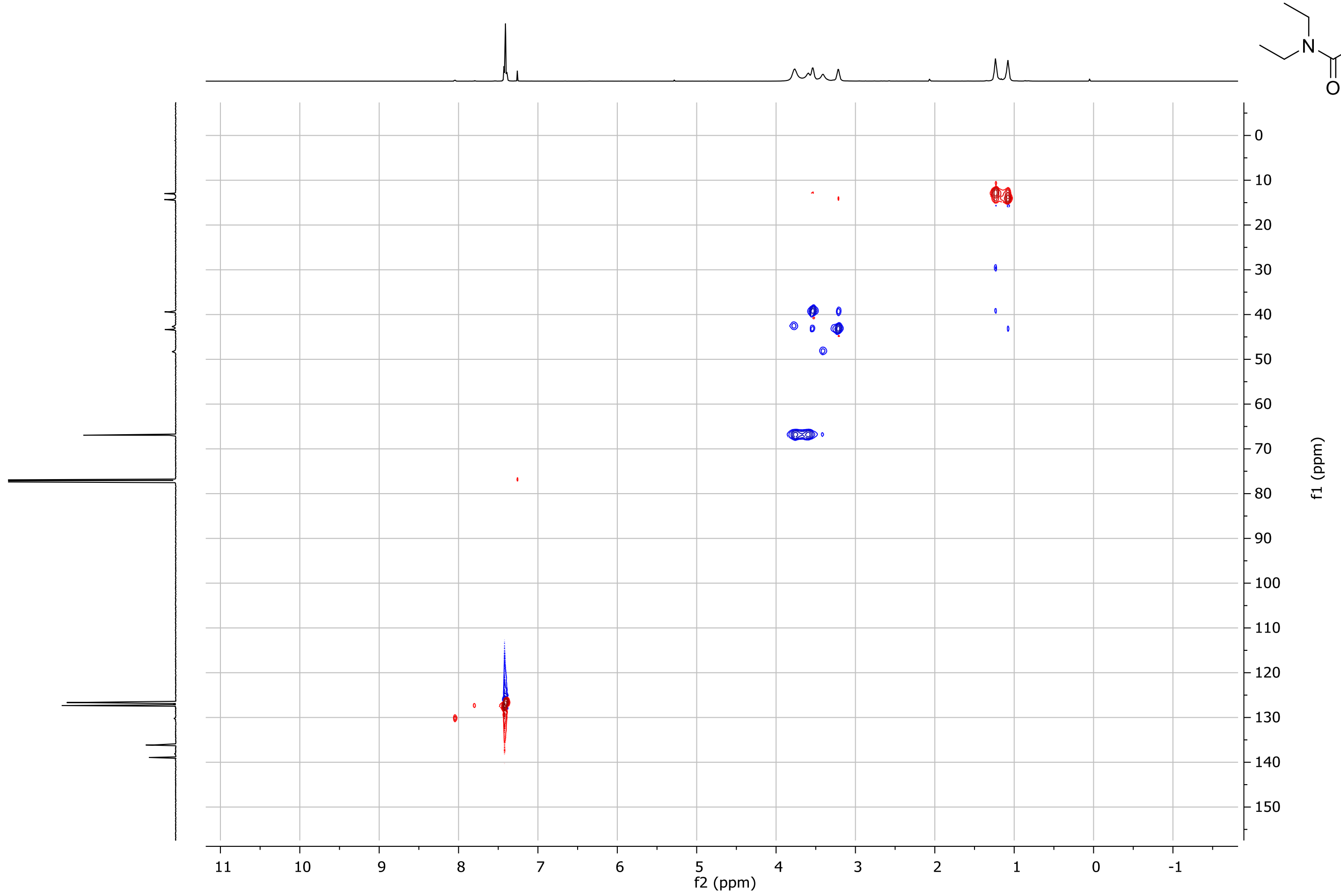

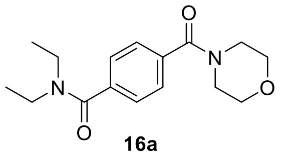

16a

HRMS

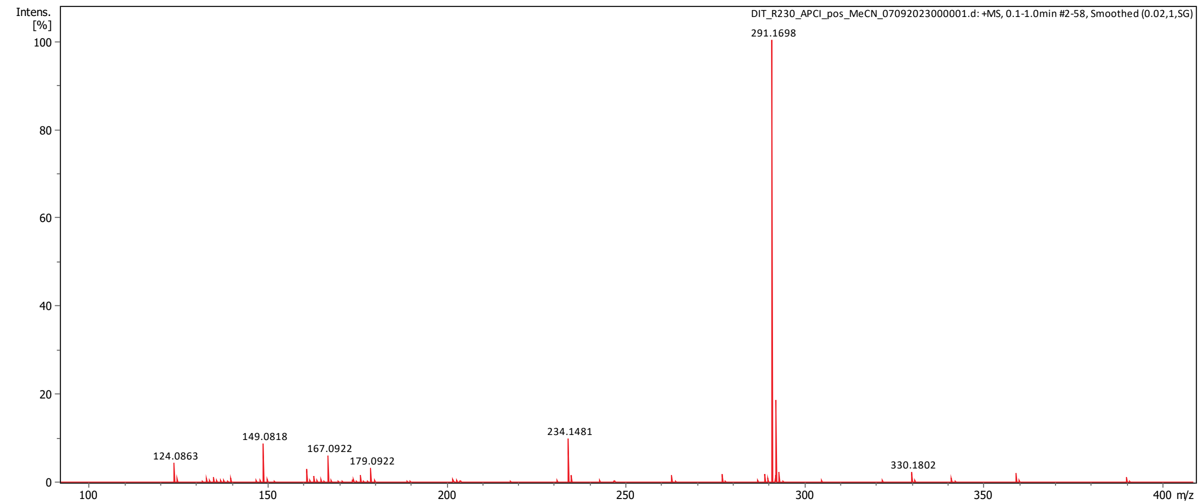

Intens. [%]
DIT_R230_APCI_pos_MeCN_07092023000001.d: +MS, 0.1-1.0min #2-58, Smoothed (0.02,1,SG)
291.1698
234.1481
149.0818
167.0922
124.0863
179.0922
330.1802
m/z

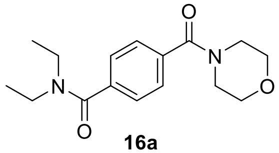

16a

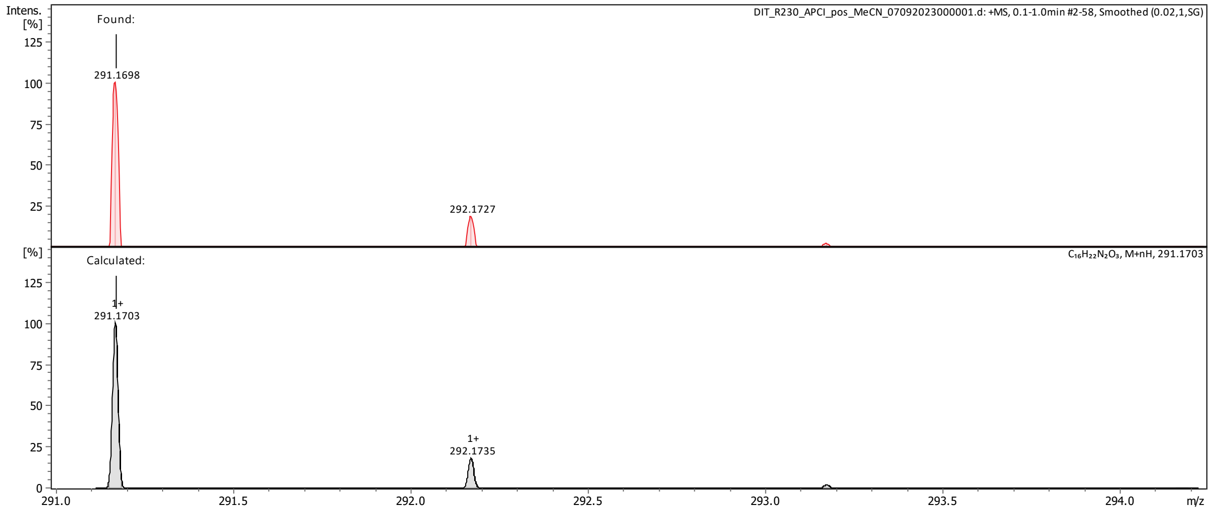

Intens. [%]
Found:
291.1698
292.1727
DIT_R230_APCI_pos_MeCN_07092023000001.d: +MS, 0.1-1.0min #2-58, Smoothed (0.02,1,SG)
Calculated:
1+
291.1703
1+
292.1735
C16H22N2O3, M+nH, 291.1703
m/z

IR

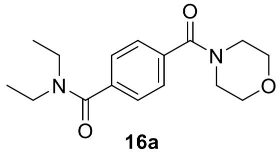

16a

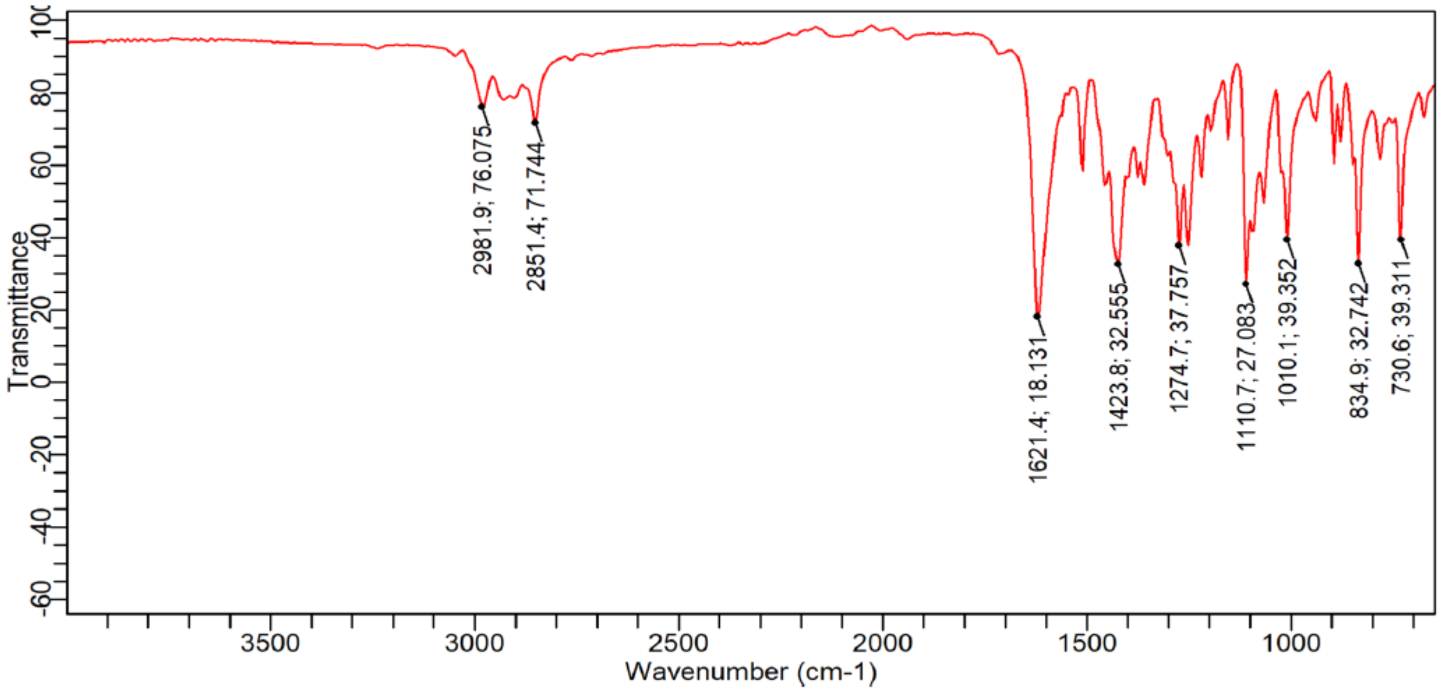

Transmittance
100
80
60
40
20
0
-20
-40
-60
2981.9; 76.075
2851.4; 71.744
1621.4; 18.131
1423.8; 32.555
1274.7; 37.757
1110.7; 27.083
1010.1; 39.352
834.9; 32.742
730.6; 39.311
3500
3000
2500
2000
1500
1000
Wavenumber (cm-1)

37 *N*-(2-methoxyethyl)-*N*-methyl-4-(morpholine-4-carbonyl)benzamide (16b)

^{1}H NMR

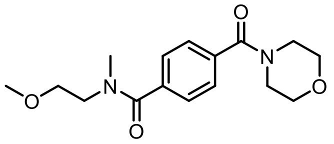

16b

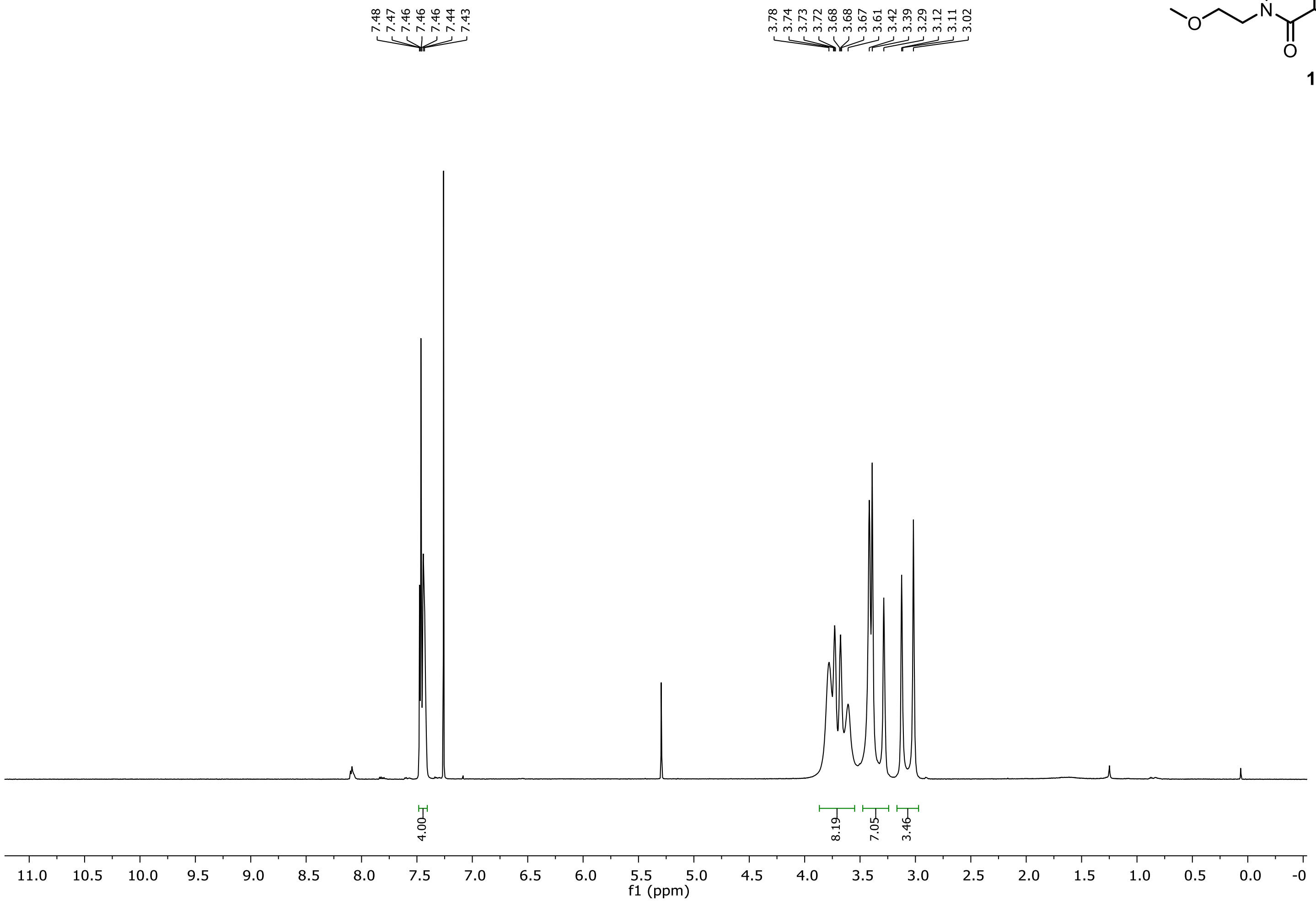

^{13}C NMR

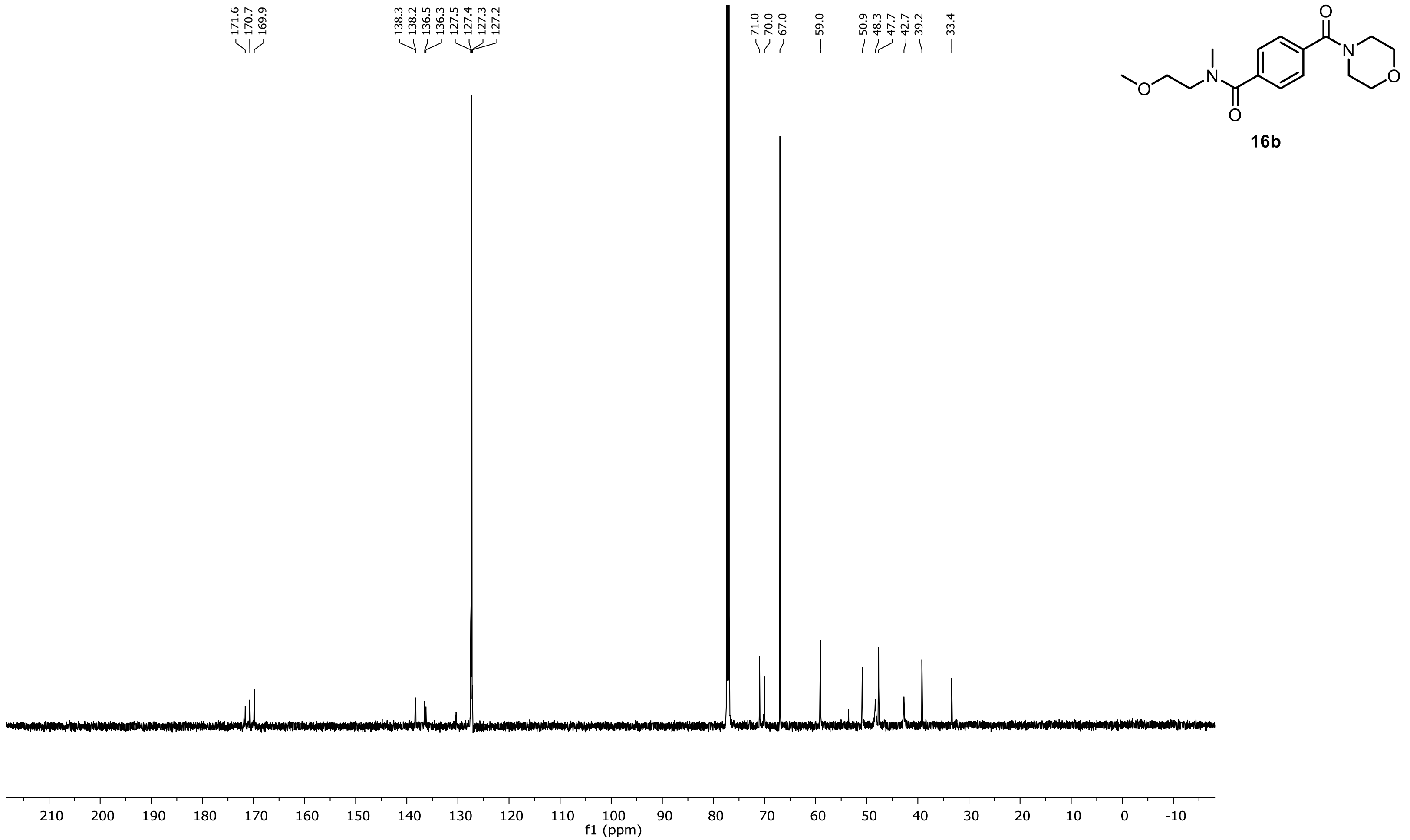
171.6
170.7
169.9
138.3
138.2
136.5
136.3
127.5
127.4
127.3
127.2
71.0
70.0
67.0
59.0
50.9
48.3
47.7
42.7
39.2
33.4
210 200 190 180 170 160 150 140 130 120 110 100 90 80 70 60 50 40 30 20 10 0 -10
f1 (ppm)

16b

$^{1}H, ^{1}H$ COSY

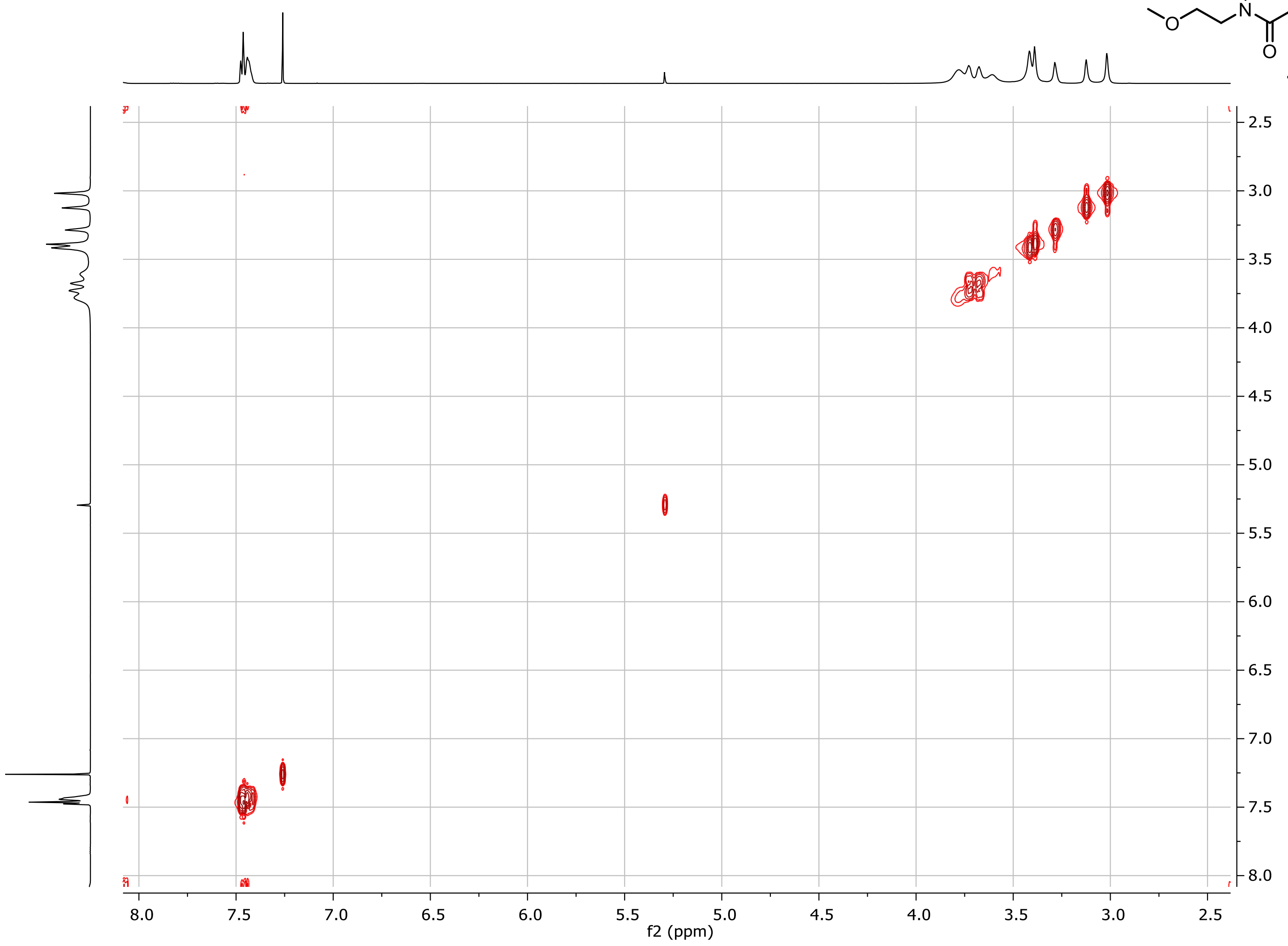

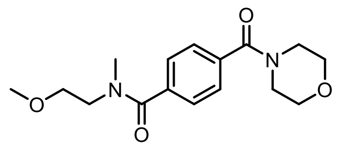

16b

^1H, ^{13}C HMBC

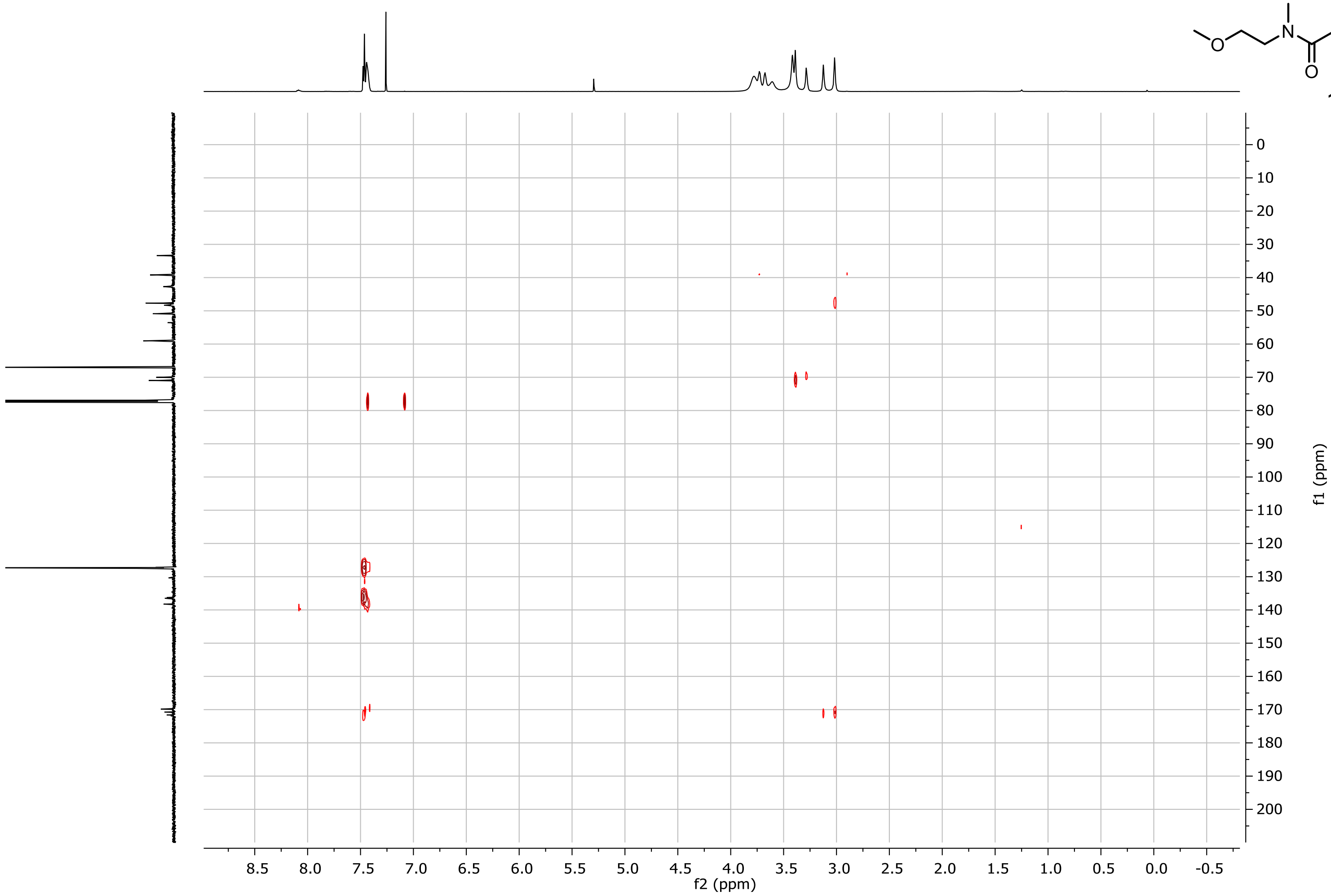

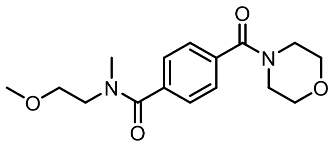

16b

^{1}H, ^{13}C HSQC

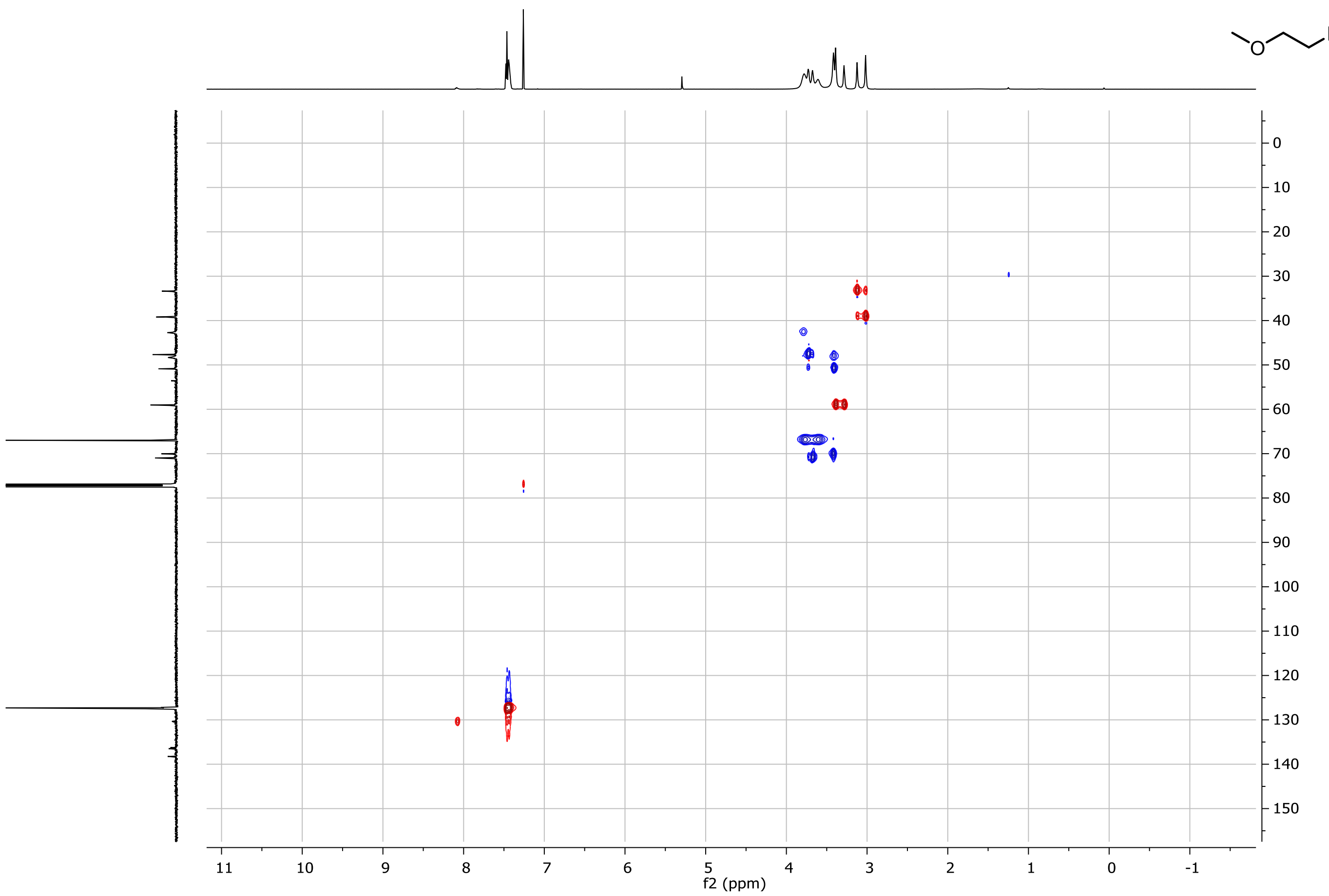

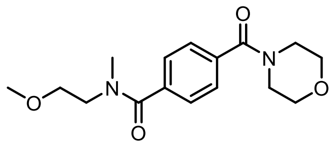

16b

HRMS

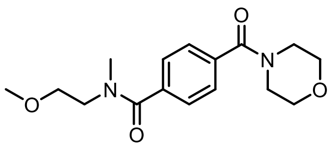

16b

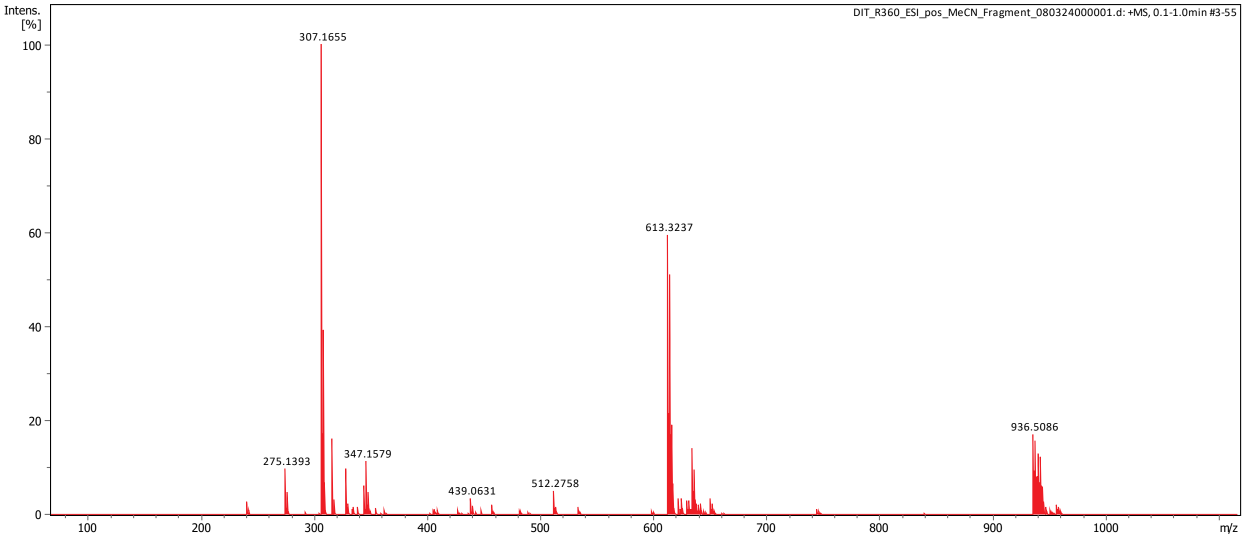

Intens. [%]
DIT_R360_ESI_pos_MeCN_Fragment_080324000001.d: +MS, 0.1-1.0min #3-55
307.1655
275.1393
347.1579
439.0631
512.2758
613.3237
936.5086
m/z

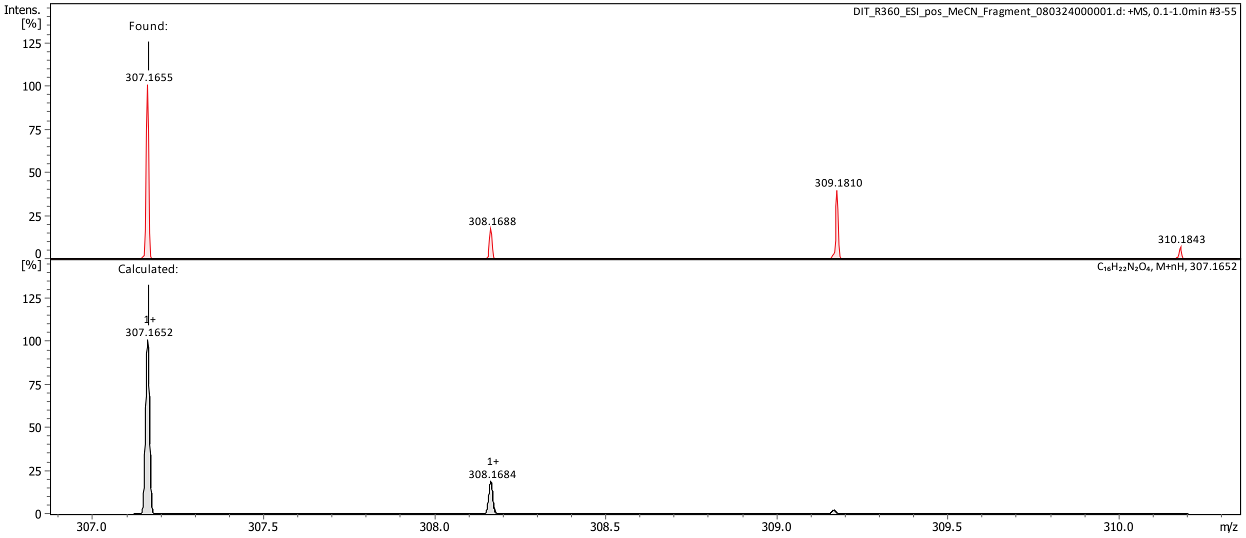

Intens. [%]
DIT_R360_ESI_pos_MeCN_Fragment_080324000001.d: +MS, 0.1-1.0min #3-55
Found:
307.1655
308.1688
309.1810
310.1843
Calculated:
$C_{16}H_{22}N_2O_4$, M+nH, 307.1652
1+ 307.1652
1+ 308.1684
m/z

IR

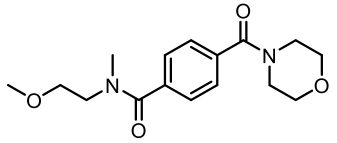

16b

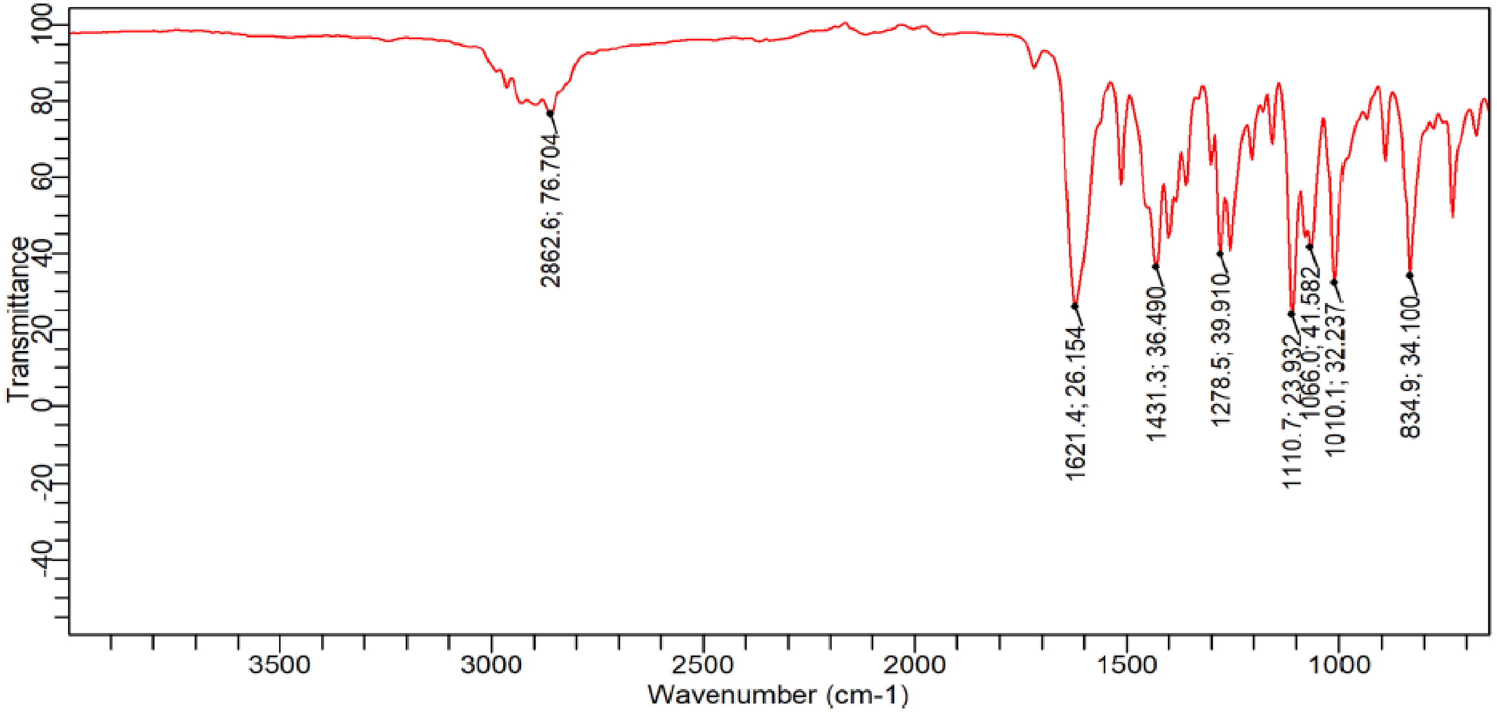

Transmittance
Wavenumber (cm-1)
100
80
60
40
20
0
-20
-40
3500
3000
2500
2000
1500
1000
2862.6; 76.704
1621.4; 26.154
1431.3; 36.490
1278.5; 39.910
1110.7; 23.932
1066.0; 41.582
1010.1; 32.237
834.9; 34.100

38 2-[Ethoxycarbonyl(methyl)amino]acetic acid (S24)

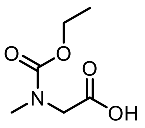

S24

^{1}H NMR

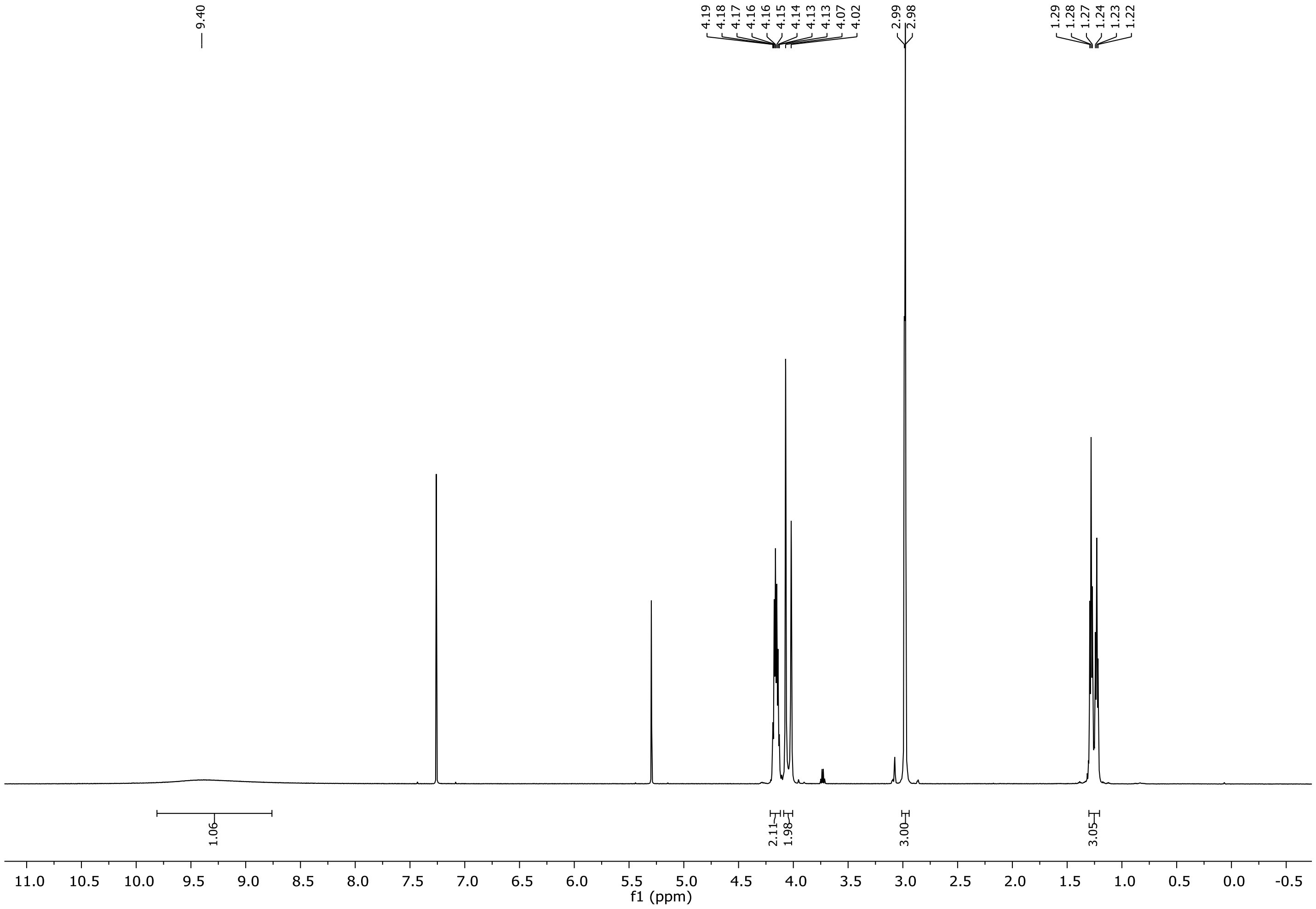

^{13}C NMR

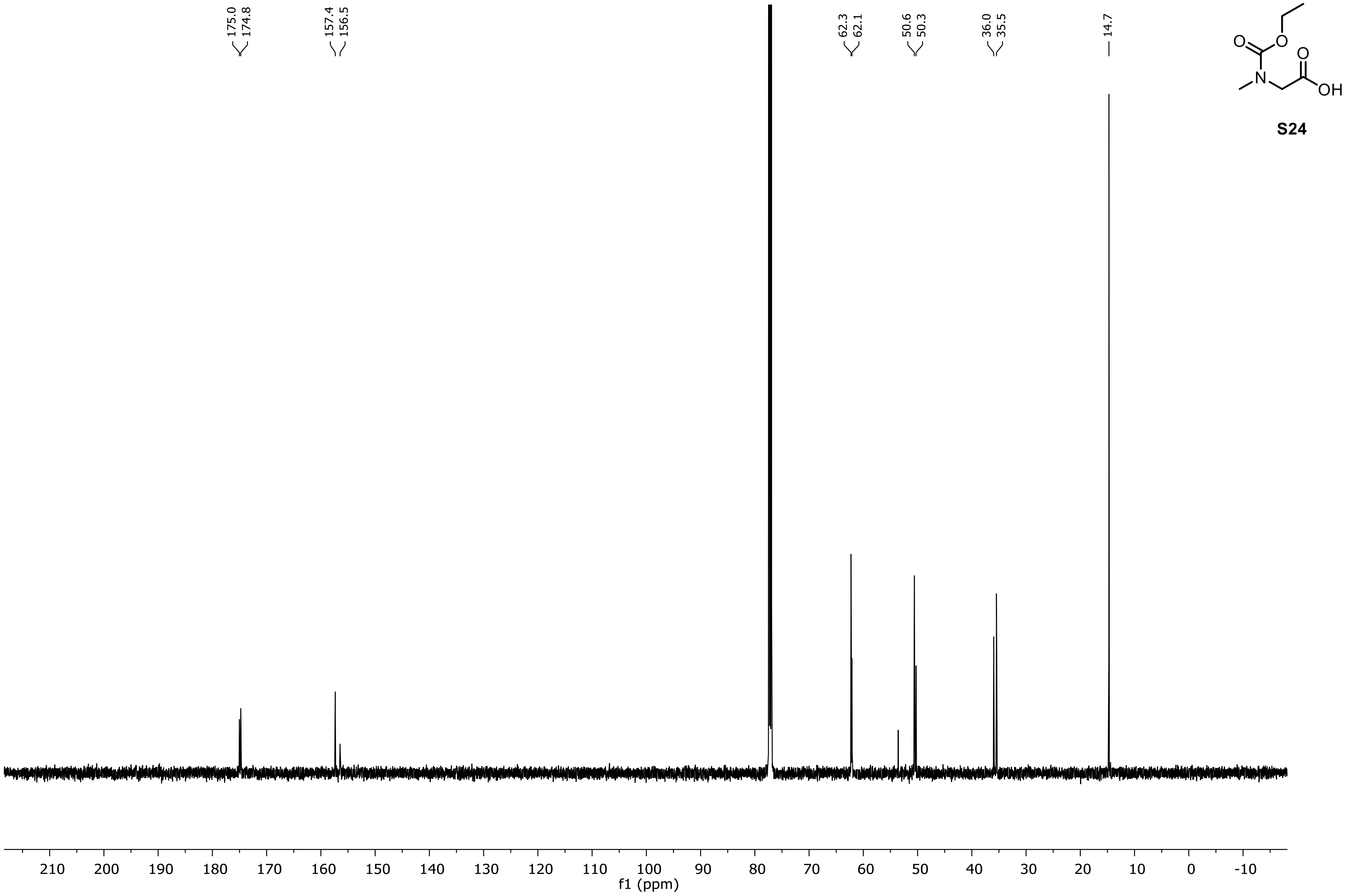
175.0
174.8
157.4
156.5
62.3
62.1
50.6
50.3
36.0
35.5
14.7
210 200 190 180 170 160 150 140 130 120 110 100 90 80 70 60 50 40 30 20 10 0 -10
f1 (ppm)
O
O
O
N
OH

S24

^{1}H, ^{1}H COSY

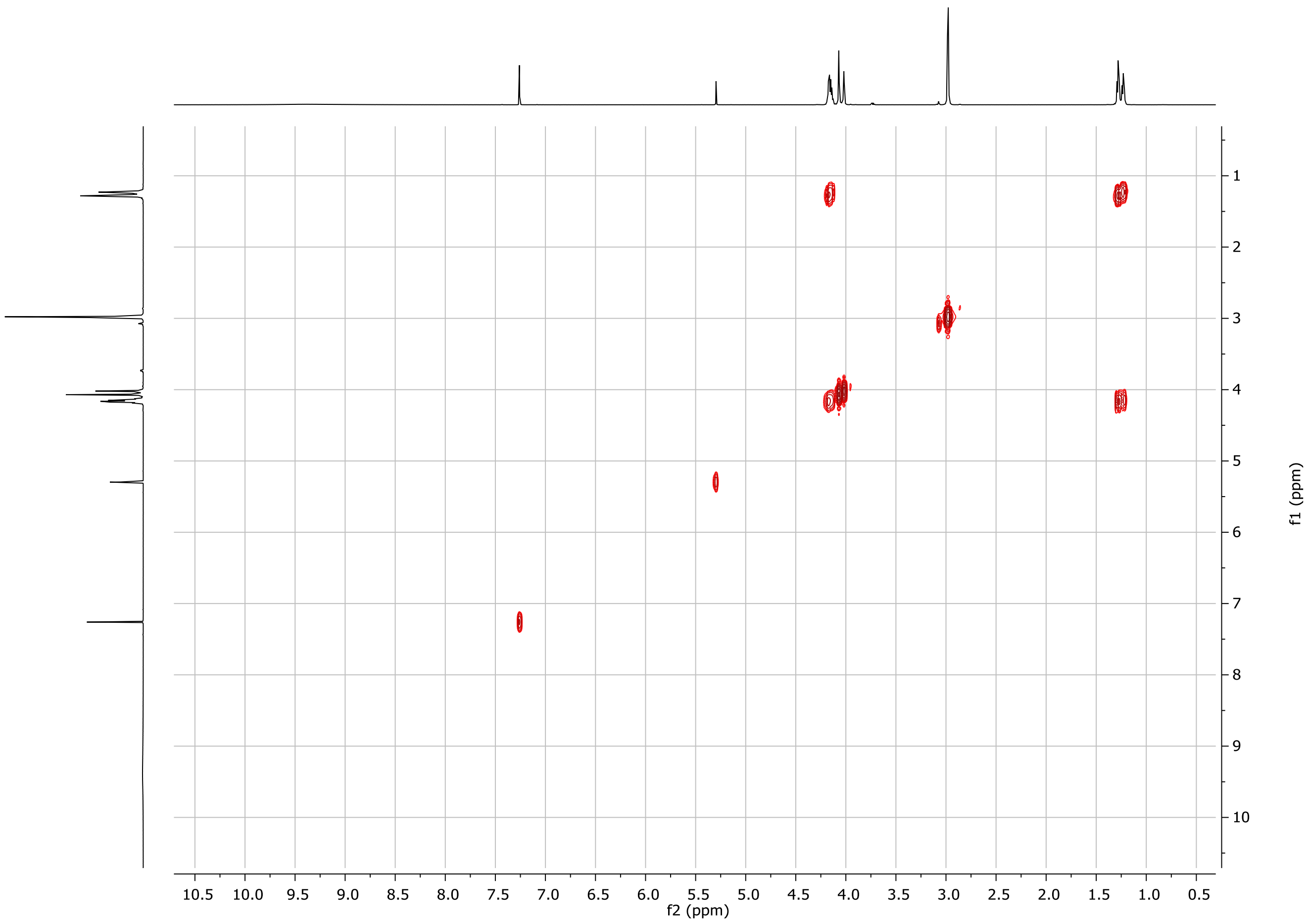

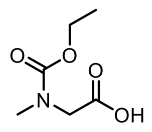

S24

^1H, ^{13}C HMBC

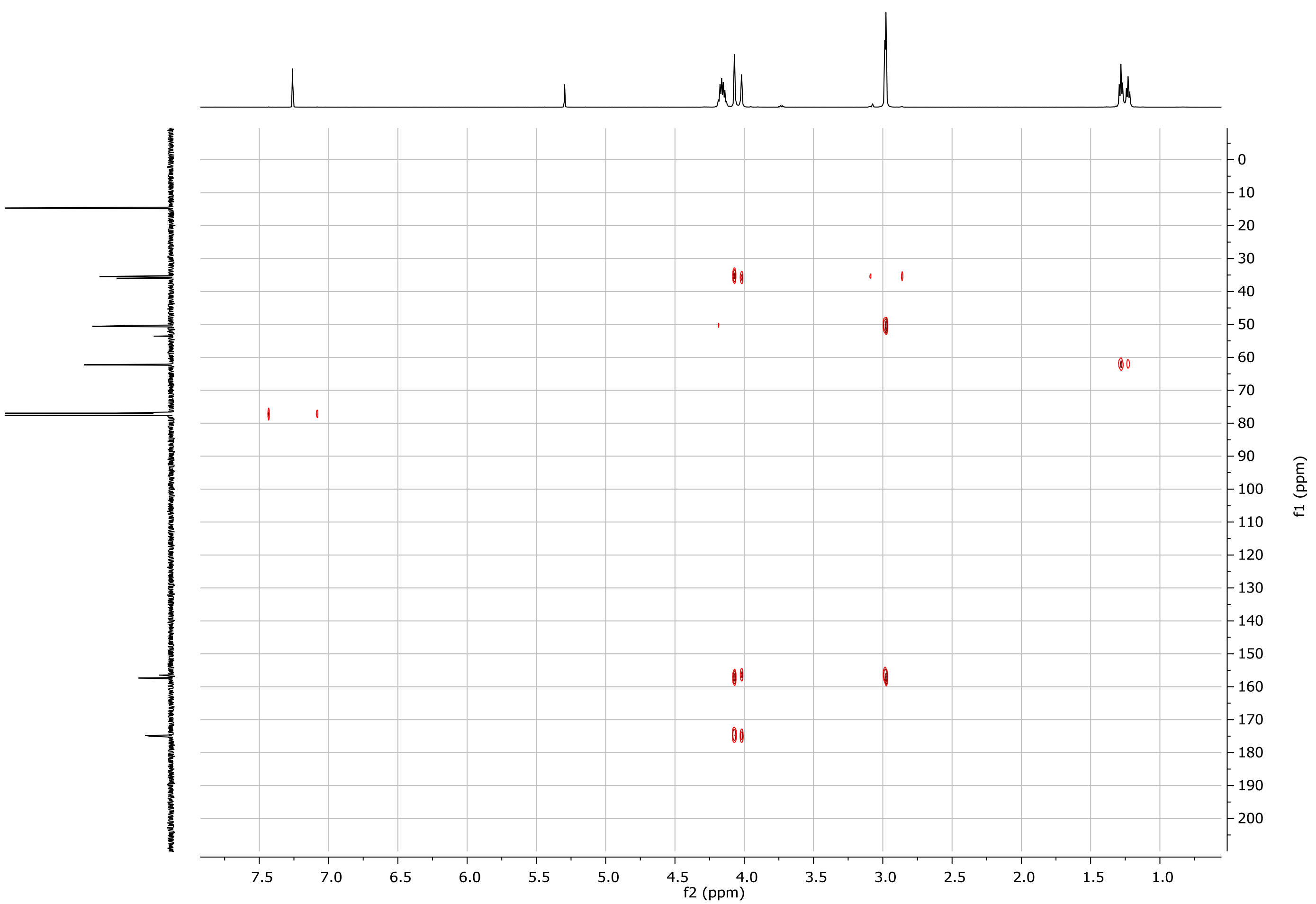

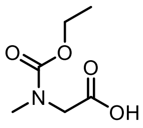

S24

^{1}H, ^{13}C HSQC

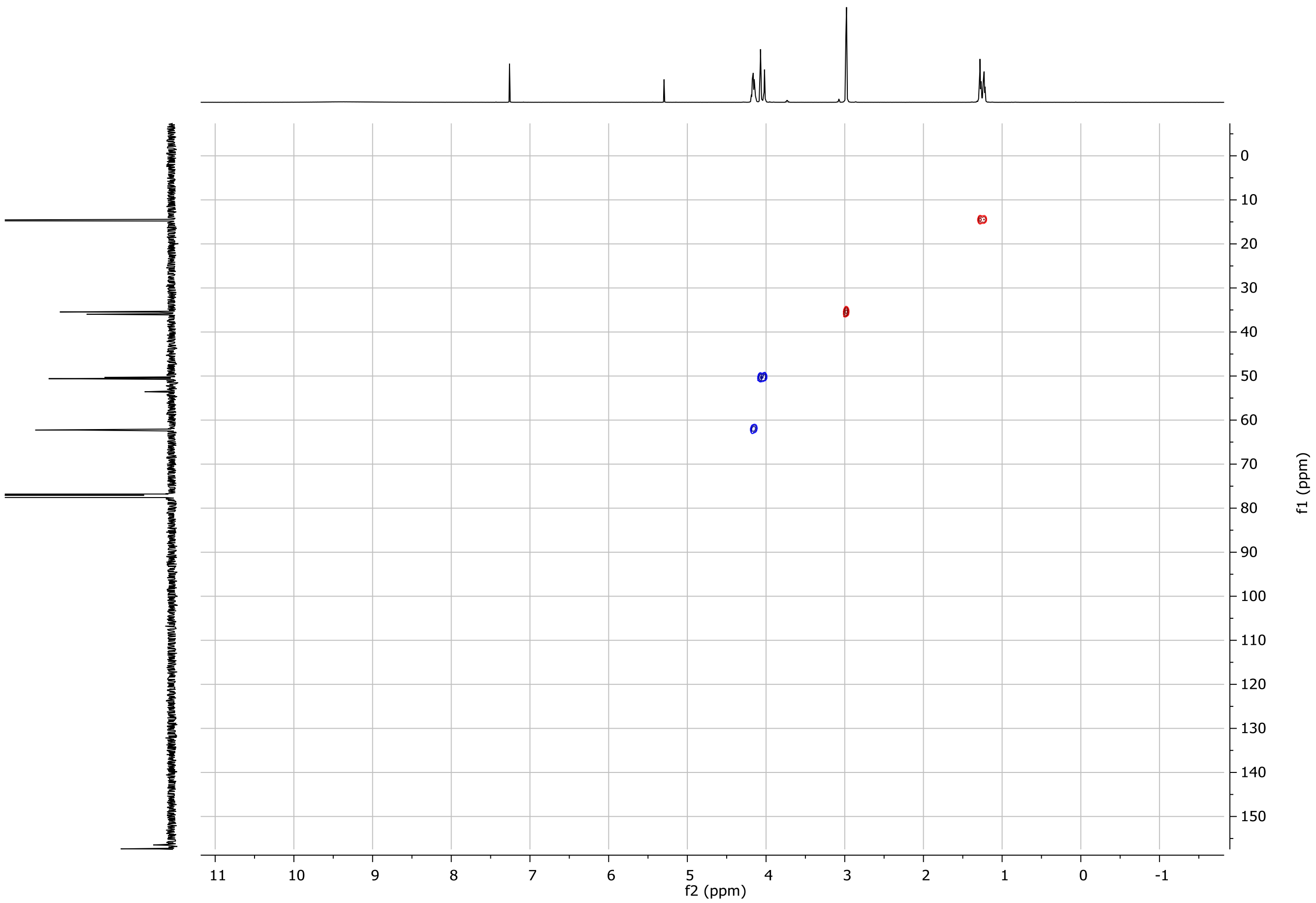

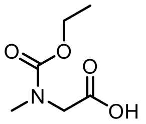

S24

HRMS

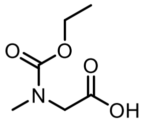

S24

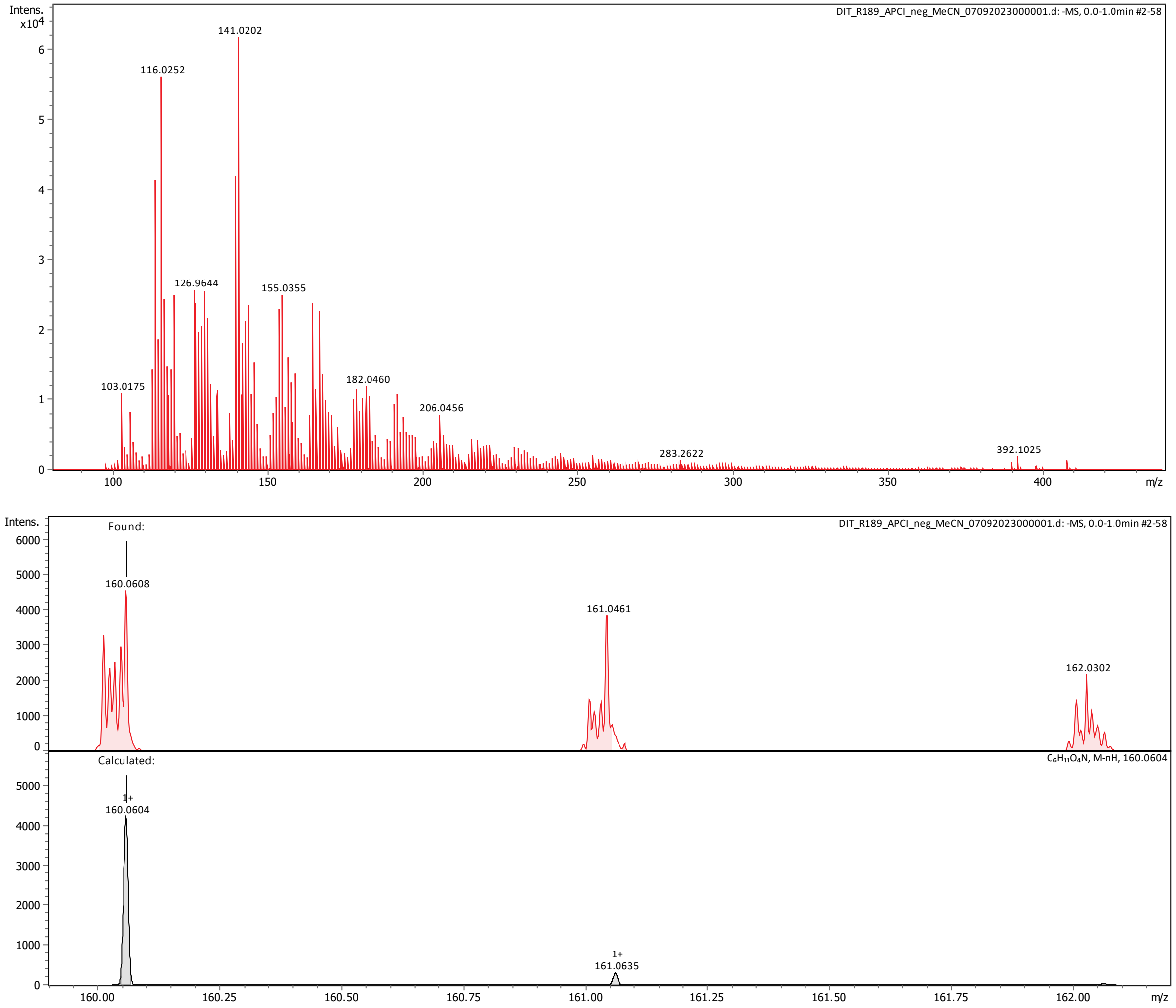

Intens. x10^4
DIT_R189_APCI_neg_MeCN_07092023000001.d: -MS, 0.0-1.0min #2-58
141.0202
116.0252
126.9644
155.0355
103.0175
182.0460
206.0456
283.2622
392.1025
m/z
Intens.
Found:
160.0608
161.0461
162.0302
Calculated:
C6H11O4N, M-nH, 160.0604
1+
160.0604
1+
161.0635
m/z

IR

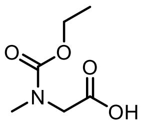

S24

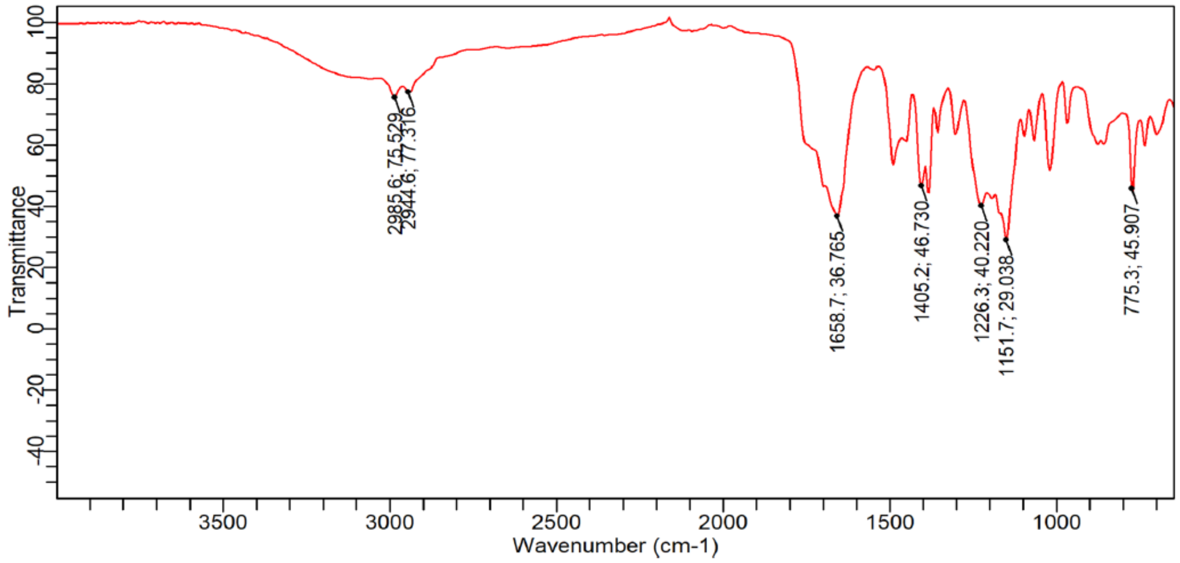
Transmittance
Wavenumber (cm-1)
2985.6; 75.529
2944.6; 77.316
1658.7; 36.765
1405.2; 46.730
1226.3; 40.220
1151.7; 29.038
775.3; 45.907

39 Ethyl methyl(2-morpholino-2-oxoethyl)carbamate (S25)

^{1}H NMR

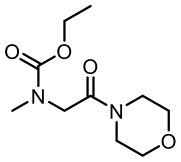

S25

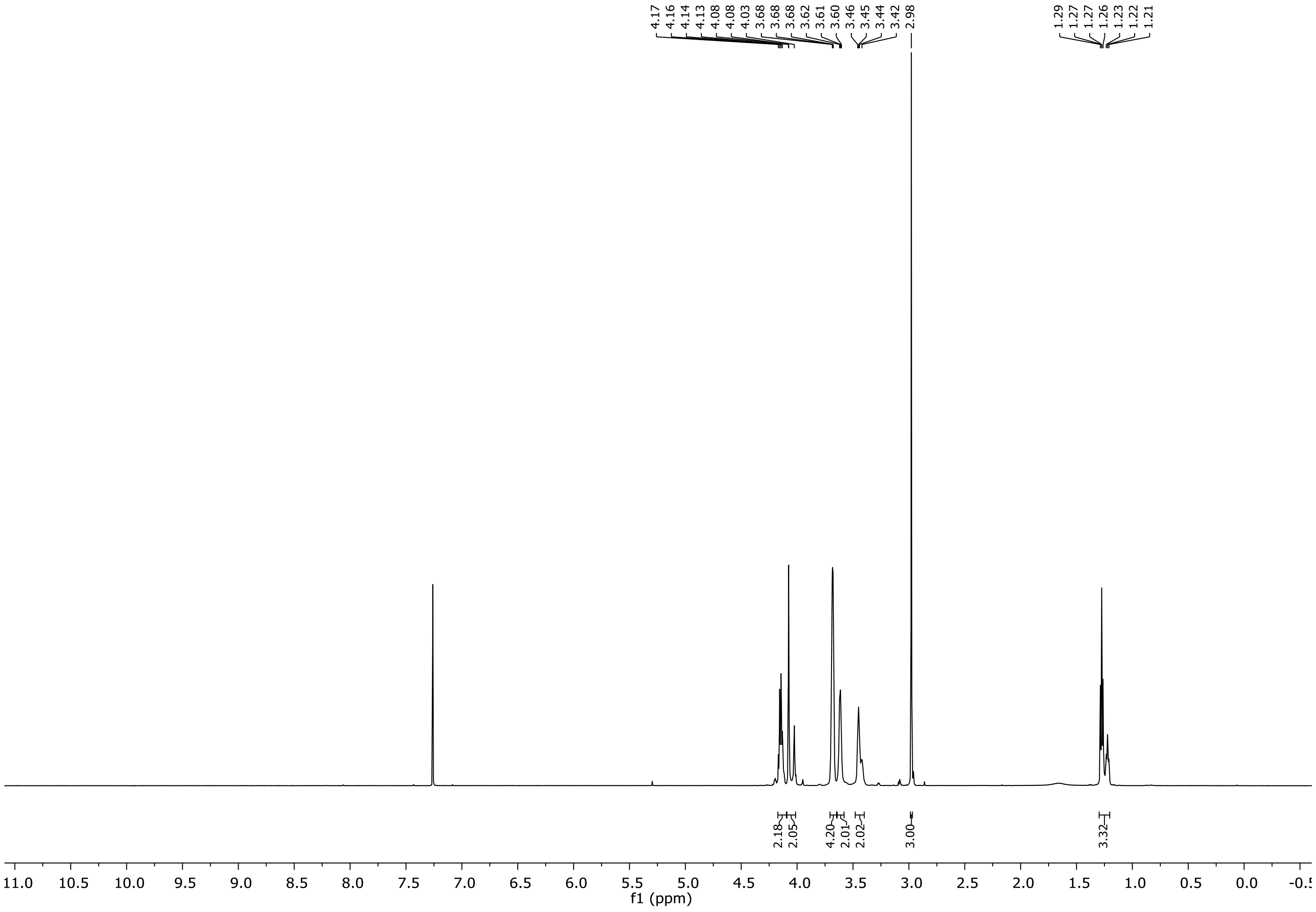

^{13}C NMR

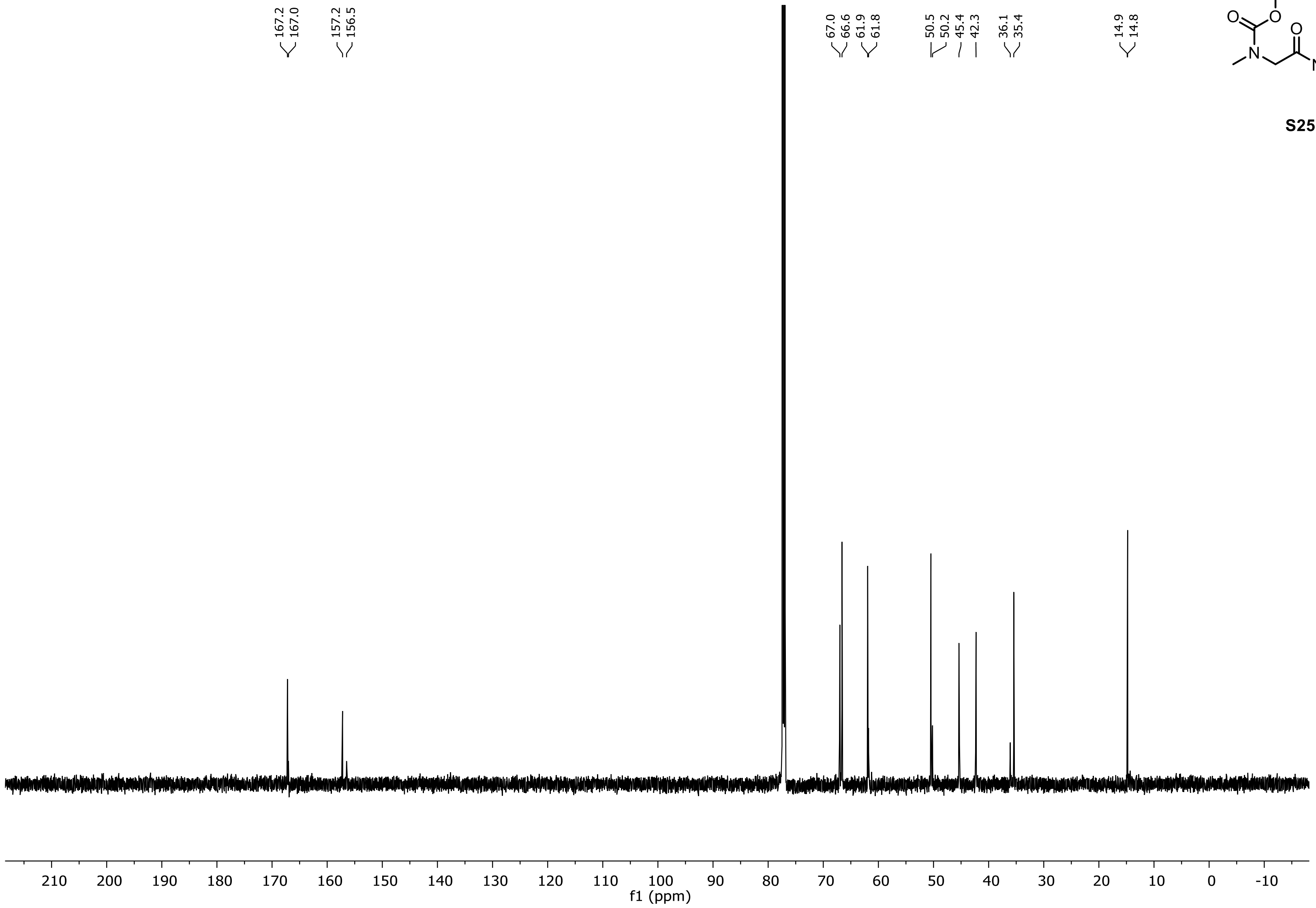
167.2
167.0
157.2
156.5
67.0
66.6
61.9
61.8
50.5
50.2
45.4
42.3
36.1
35.4
14.9
14.8
210 200 190 180 170 160 150 140 130 120 110 100 90 80 70 60 50 40 30 20 10 0 -10
f1 (ppm)

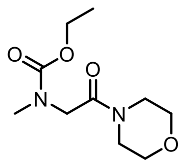

S25

^1H, ^1H COSY

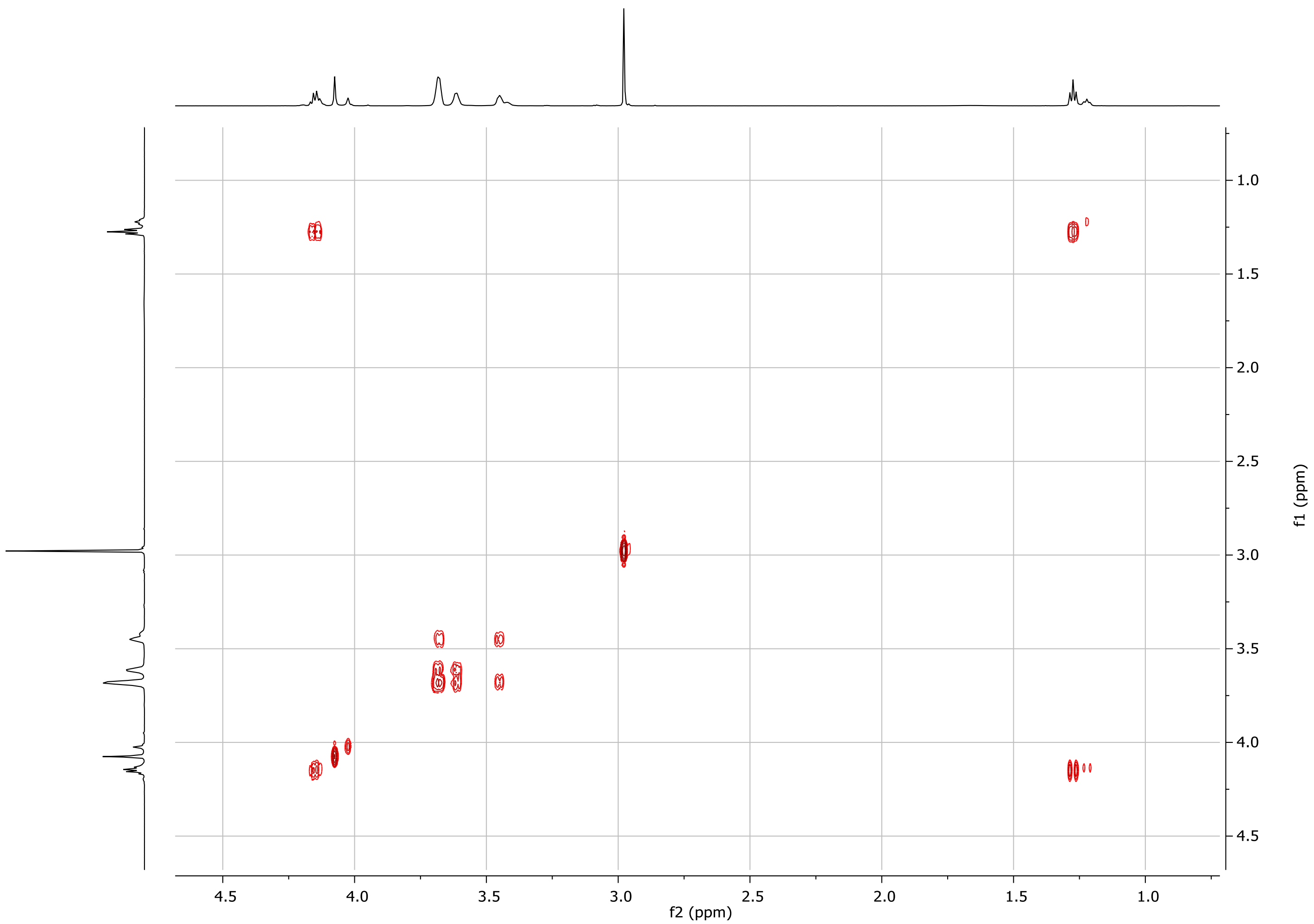

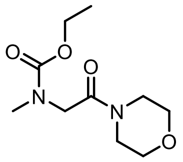

S25

^{1}H, ^{13}C HMBC

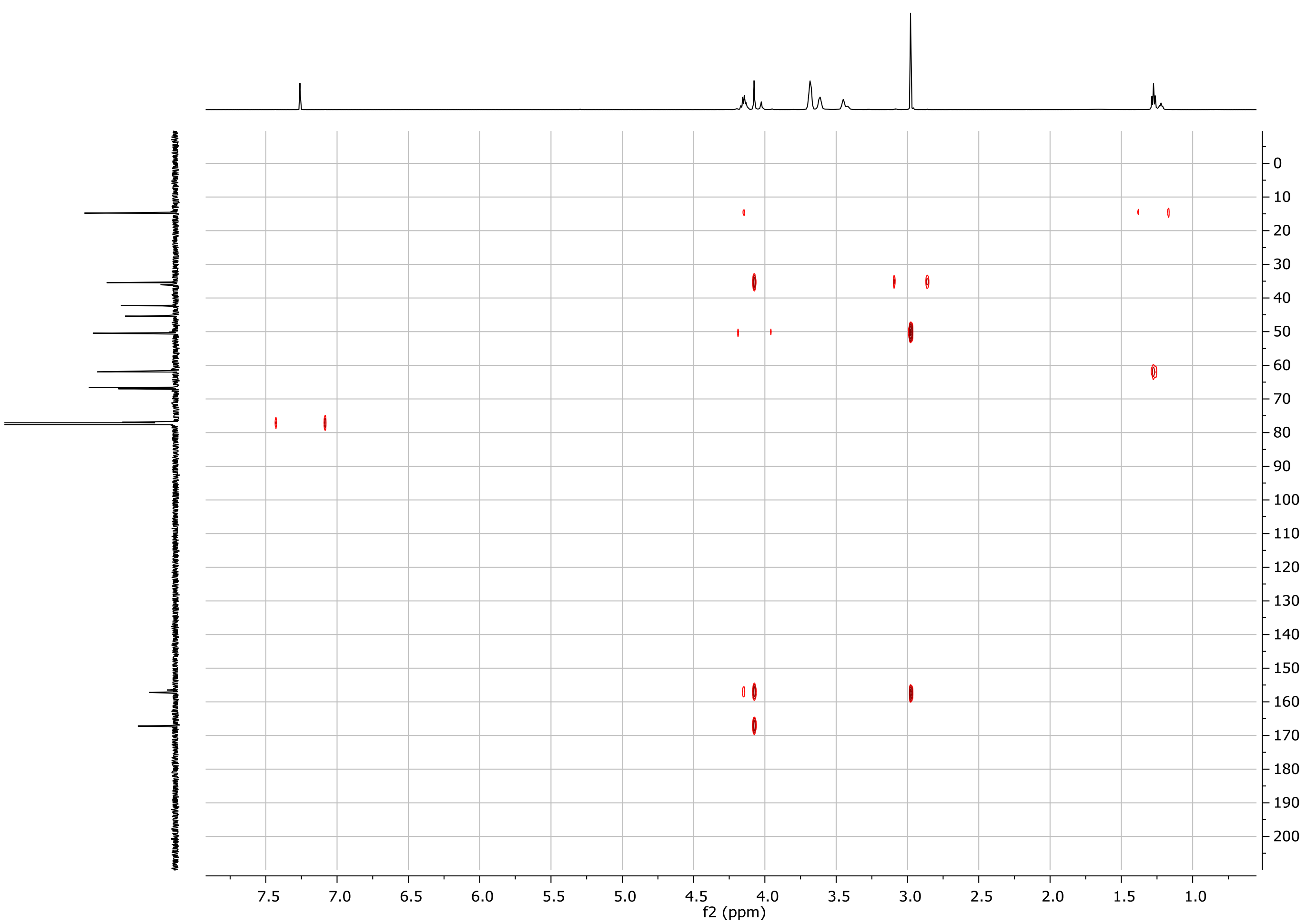

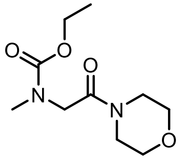

S25

^1H, ^{13}C HSQC

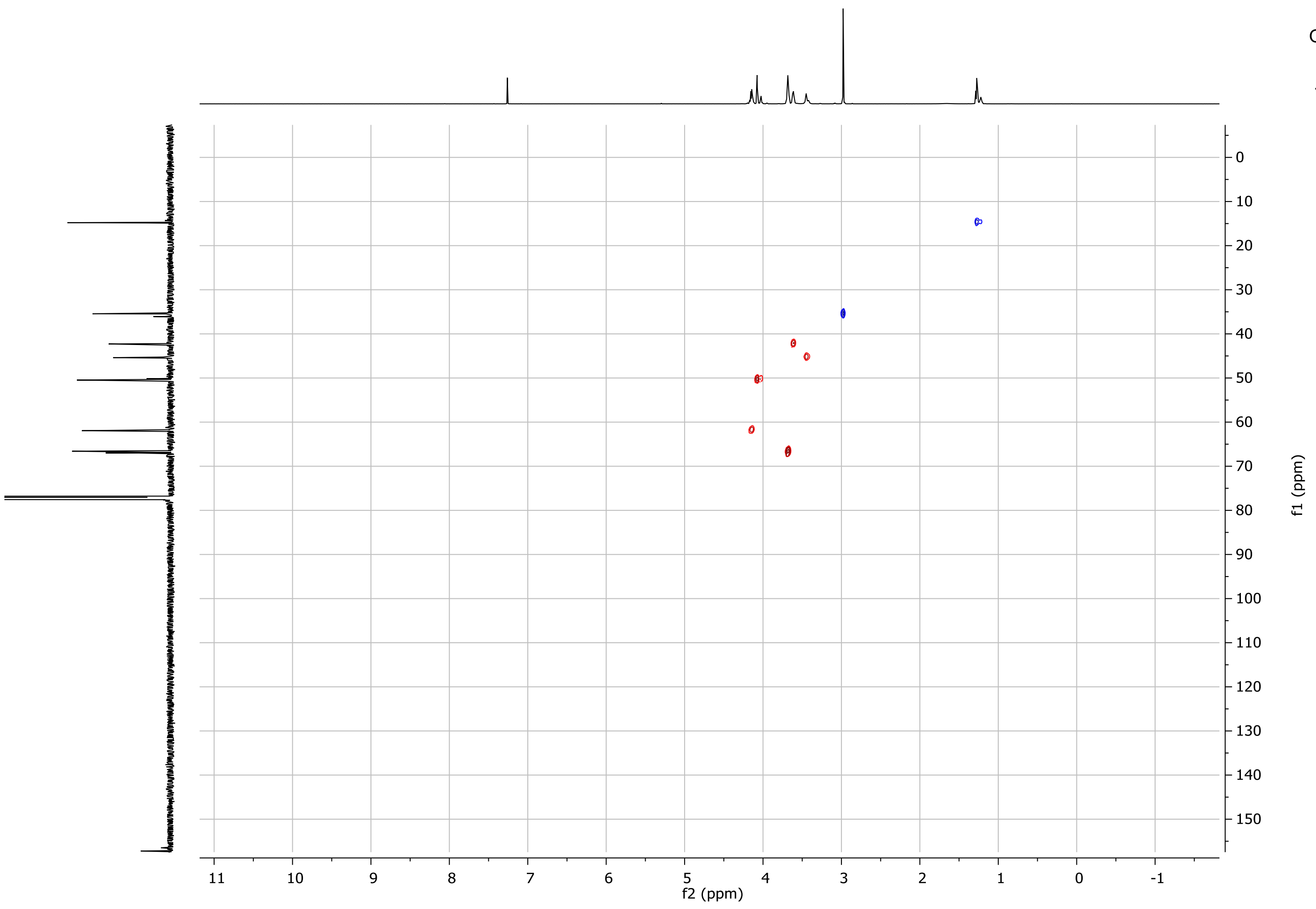

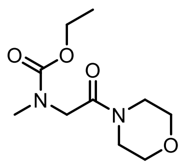

S25

HRMS

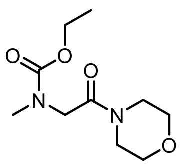

S25

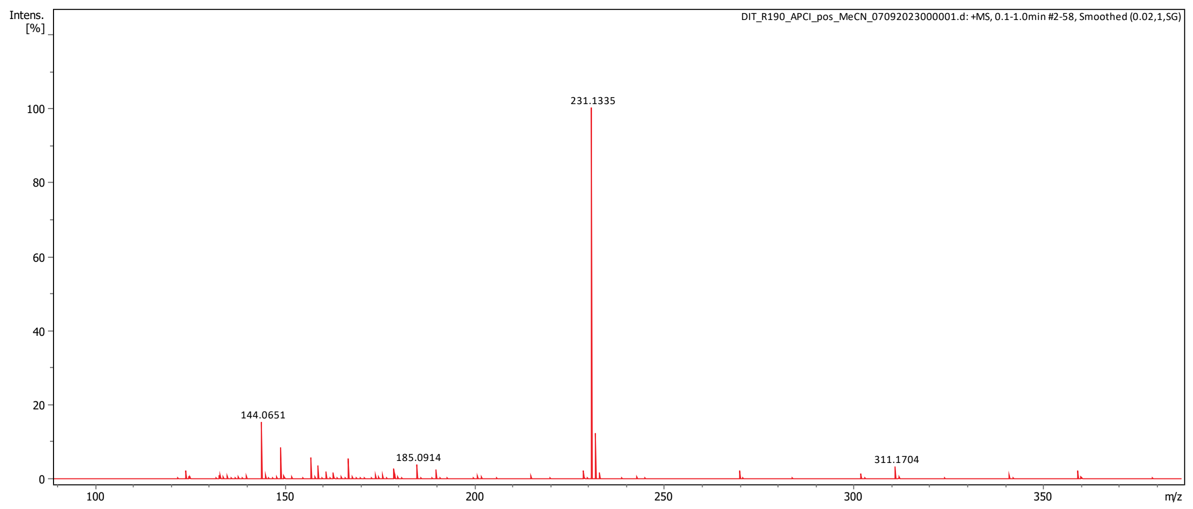

Intens. [%]
DIT_R190_APCI_pos_MeCN_07092023000001.d: +MS, 0.1-1.0min #2-58, Smoothed (0.02,1,SG)
231.1335
144.0651
185.0914
311.1704
m/z

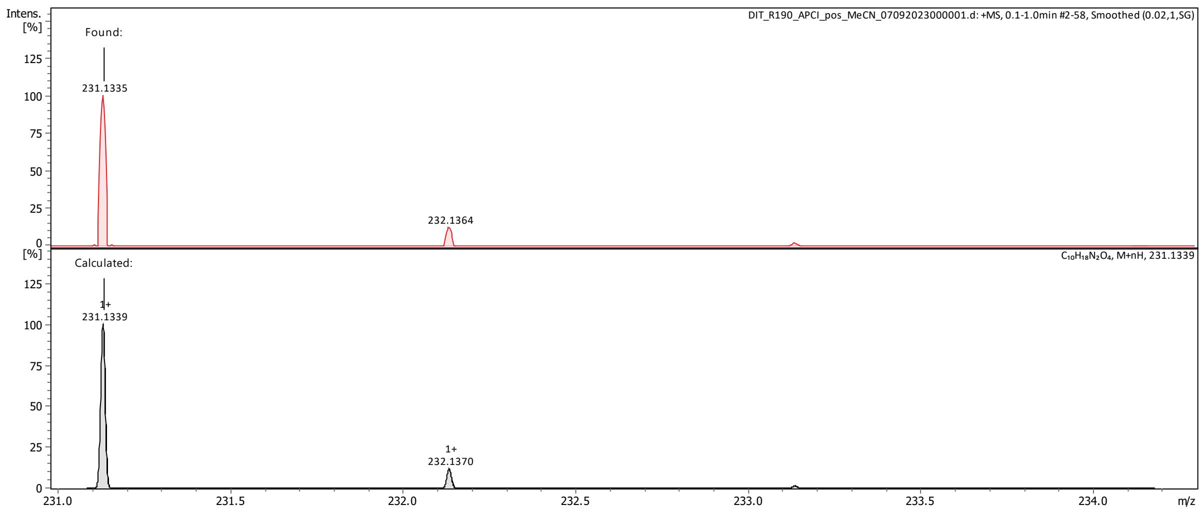

Intens. [%]
DIT_R190_APCI_pos_MeCN_07092023000001.d: +MS, 0.1-1.0min #2-58, Smoothed (0.02,1,SG)
Found:
231.1335
232.1364
Calculated:
$C_{10}H_{18}N_2O_4$, M+nH, 231.1339
1+
231.1339
1+
232.1370
m/z

IR

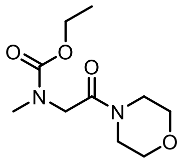

S25

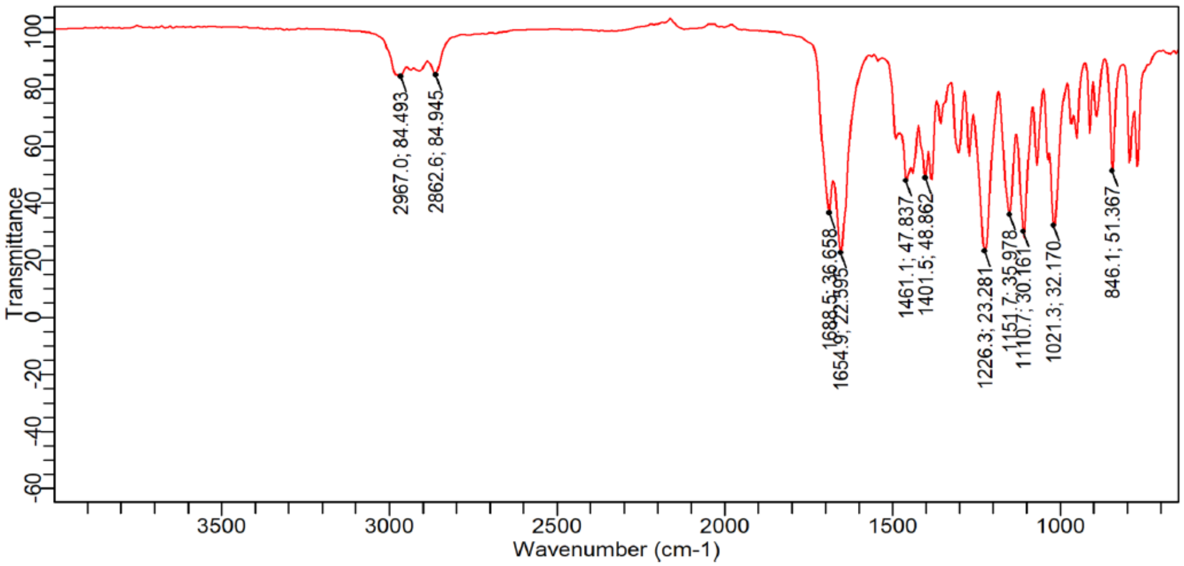
Transmittance
Wavenumber (cm-1)
100
80
60
40
20
0
-20
-40
-60
3500
3000
2500
2000
1500
1000
2967.0; 84.493
2862.6; 84.945
1688.5; 36.658
1654.9; 22.595
1461.1; 47.837
1401.5; 48.862
1226.3; 23.281
1151.7; 35.978
1110.7; 30.161
1021.3; 32.170
846.1; 51.367

40 Morpholino(phenyl-d$_5$)methanone (S26)

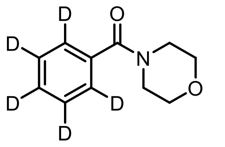

S26

^{1}H NMR

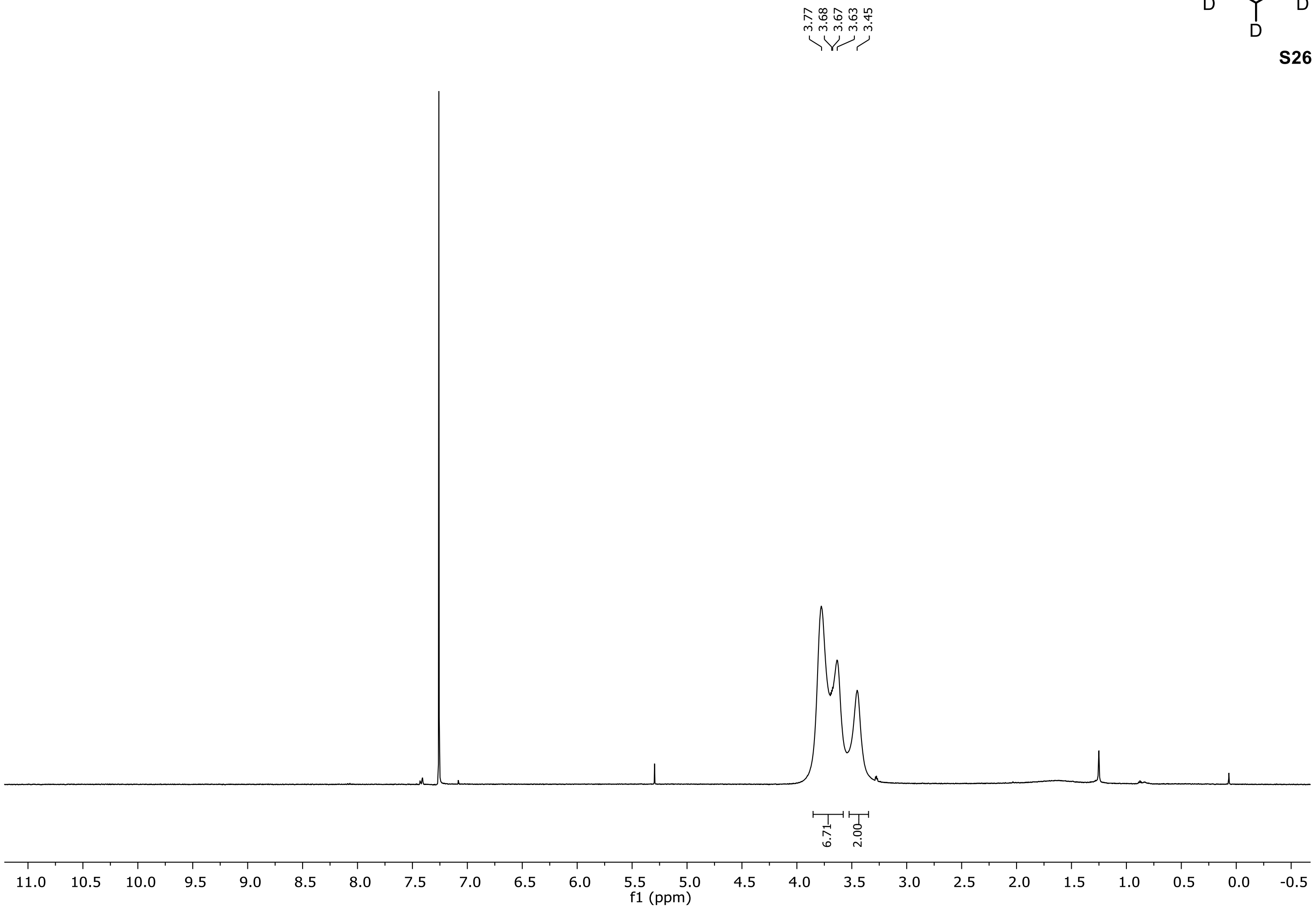

^{2}H NMR

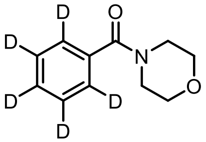

S26

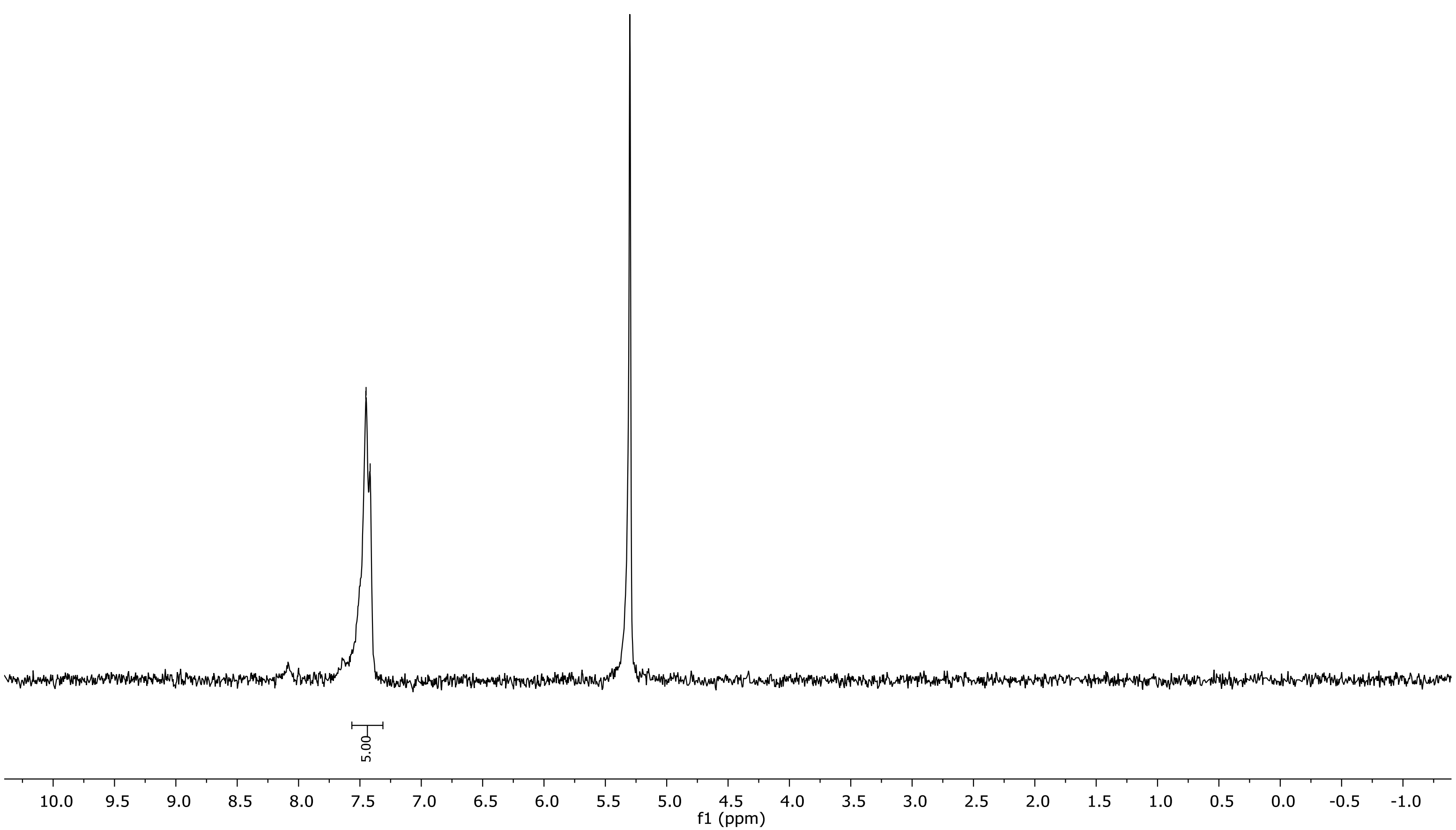

7.45
7.45
7.42
5.00
10.0
9.5
9.0
8.5
8.0
7.5
7.0
6.5
6.0
5.5
5.0
4.5
4.0
3.5
3.0
2.5
2.0
1.5
1.0
0.5
0.0
-0.5
-1.0
f1 (ppm)

^{13}C NMR

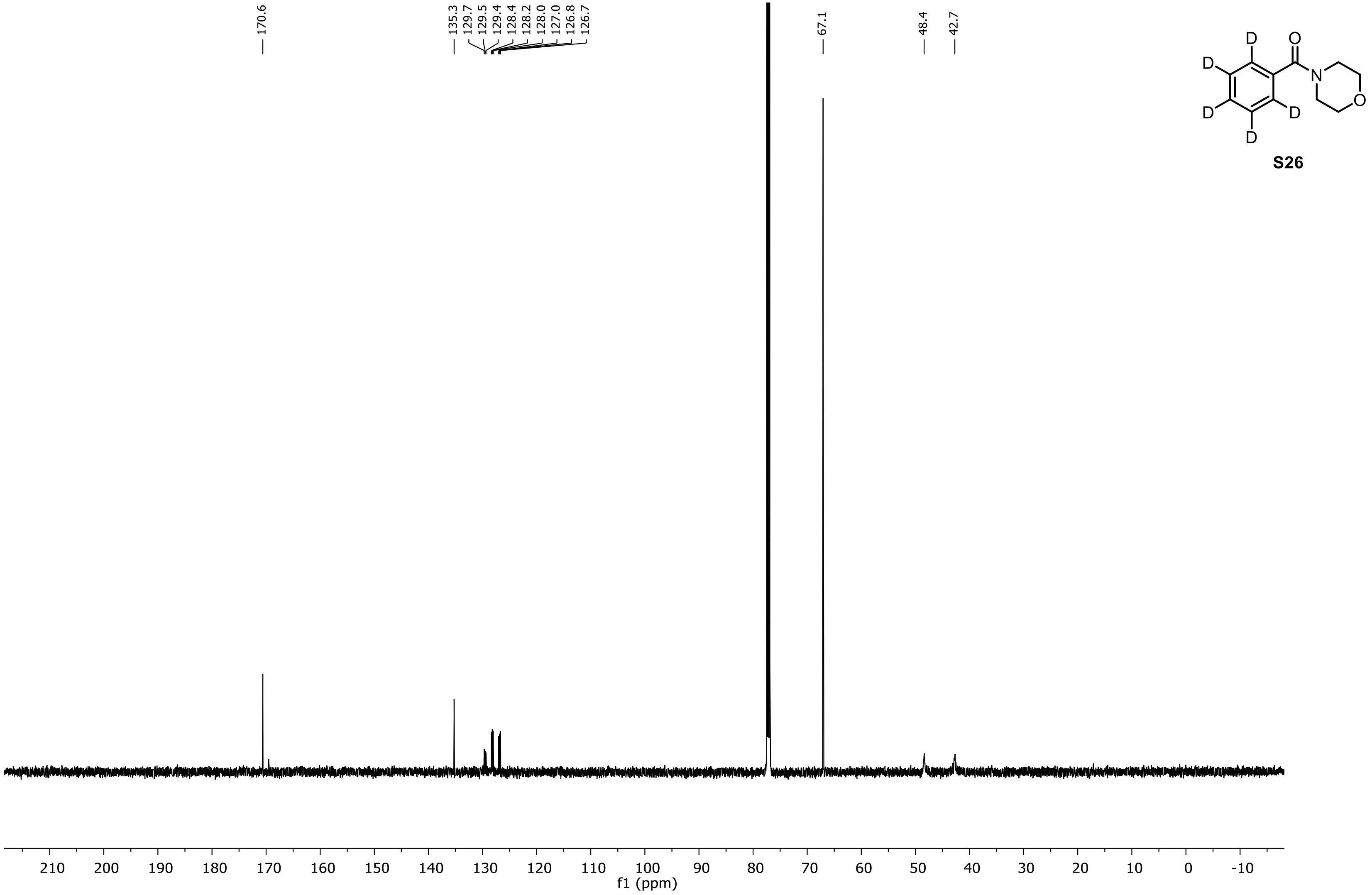
170.6
135.3
129.7
129.5
129.4
128.4
128.2
128.0
127.0
126.8
126.7
67.1
48.4
42.7
210
200
190
180
170
160
150
140
130
120
110
100
90
80
70
60
50
40
30
20
10
0
-10
f1 (ppm)
D
O
D
N
D
O
D
D

S26

^1H, ^1H COSY

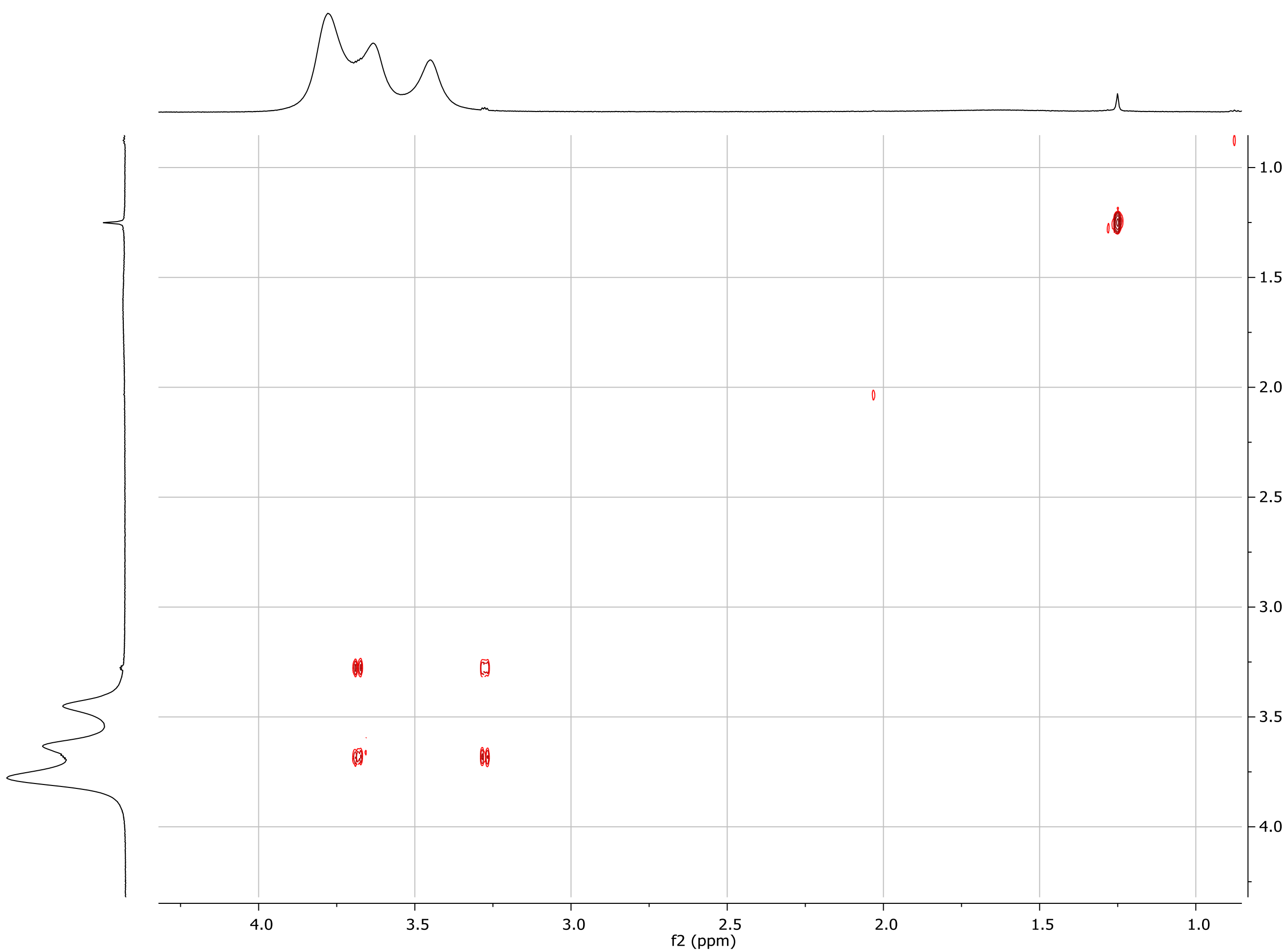

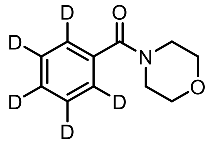

S26

^1H, ^{13}C HMBC

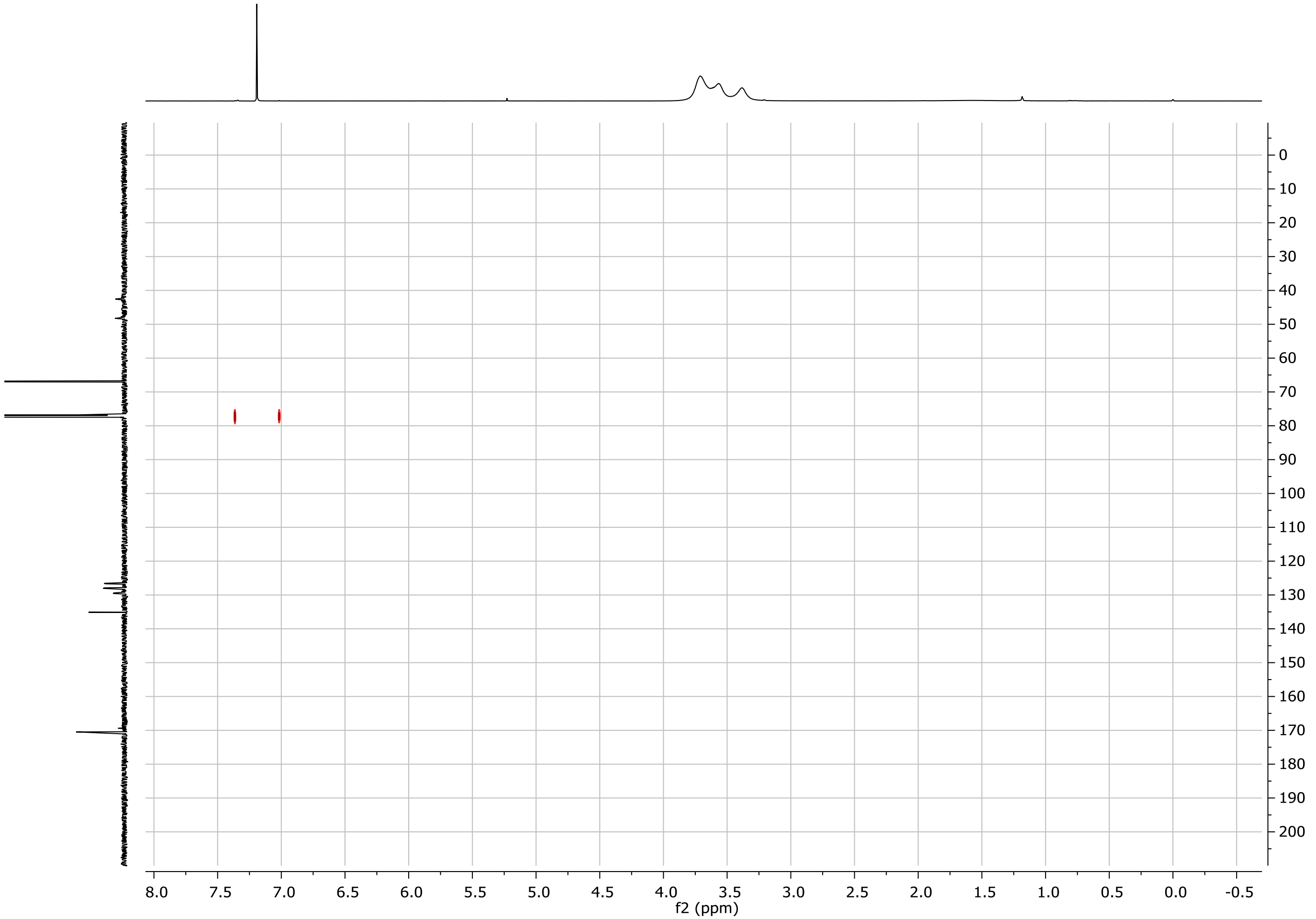

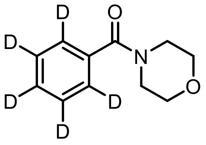

S26

^{1}H, ^{13}C HSQC

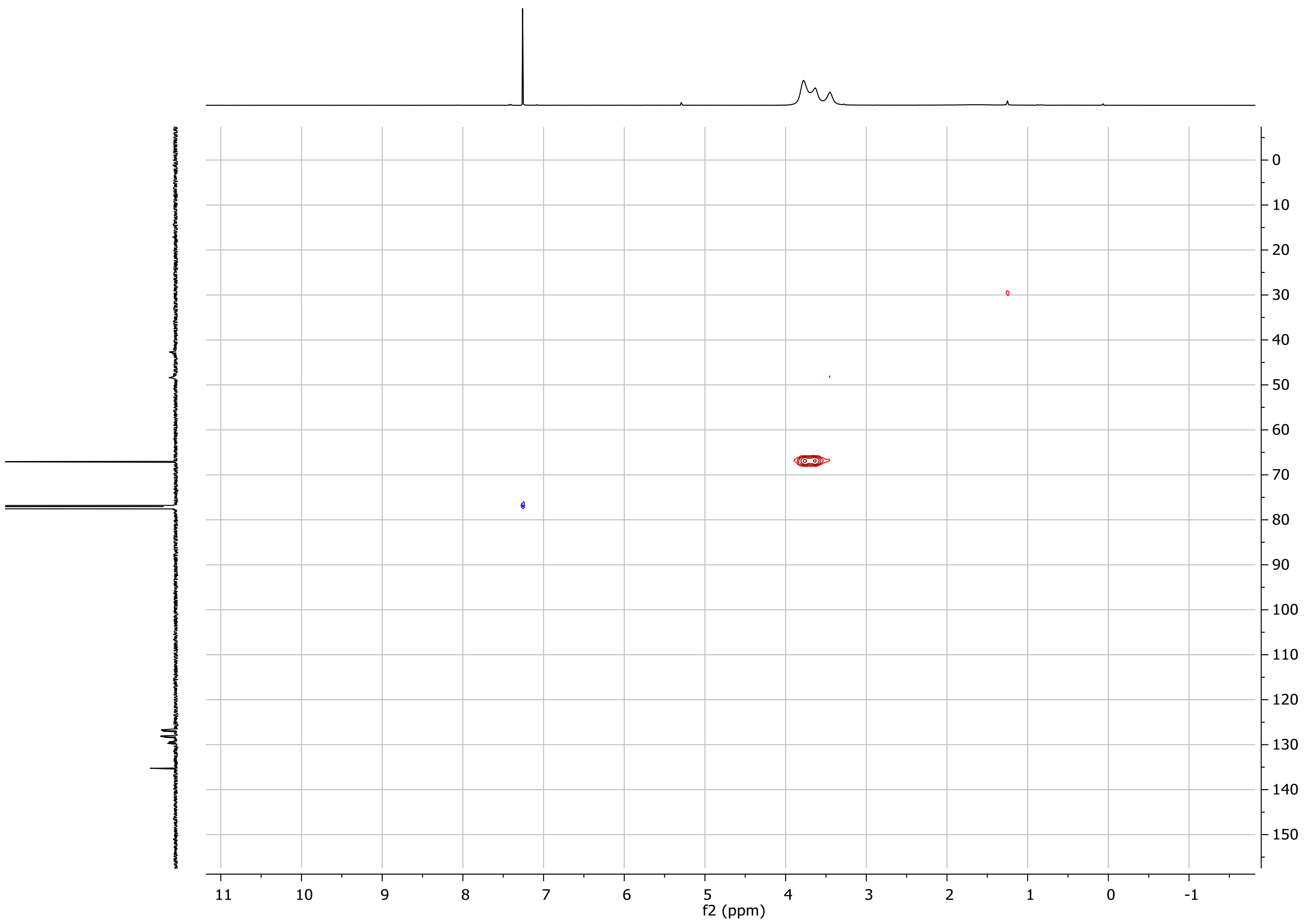

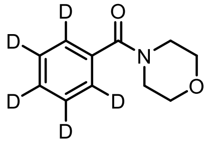

S26

HRMS

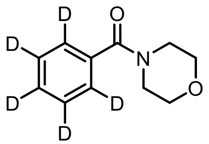

S26

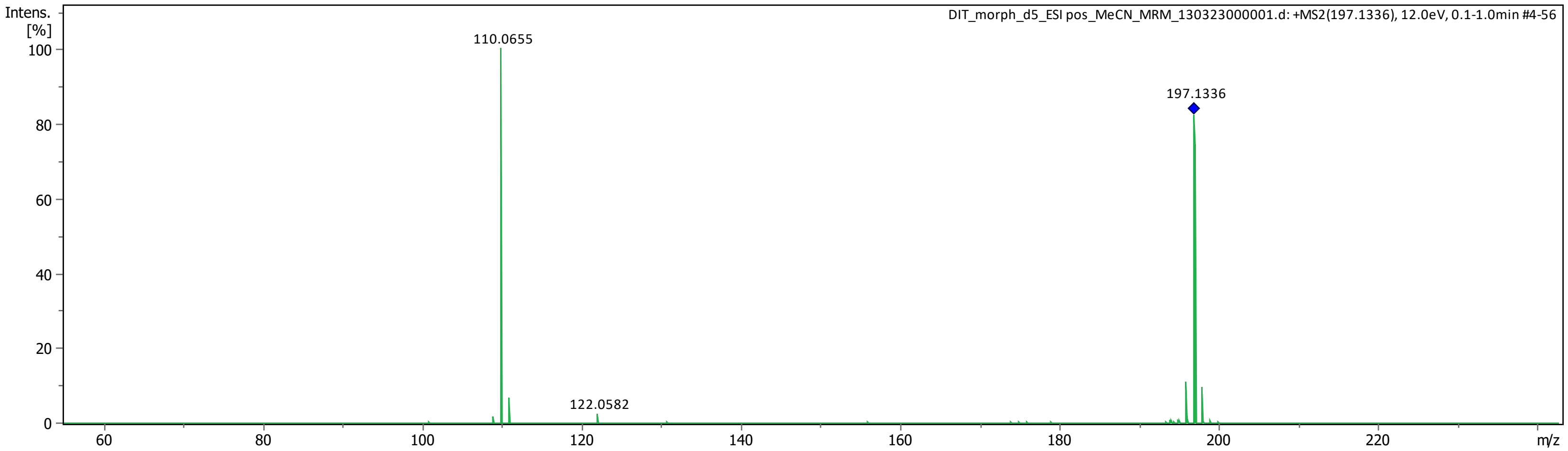
Intens.
[%]
DIT_morph_d5_ESI pos_MeCN_MRM_130323000001.d: +MS2(197.1336), 12.0eV, 0.1-1.0min #4-56
110.0655
197.1336
122.0582
100
80
60
40
20
0
60
80
100
120
140
160
180
200
220
m/z

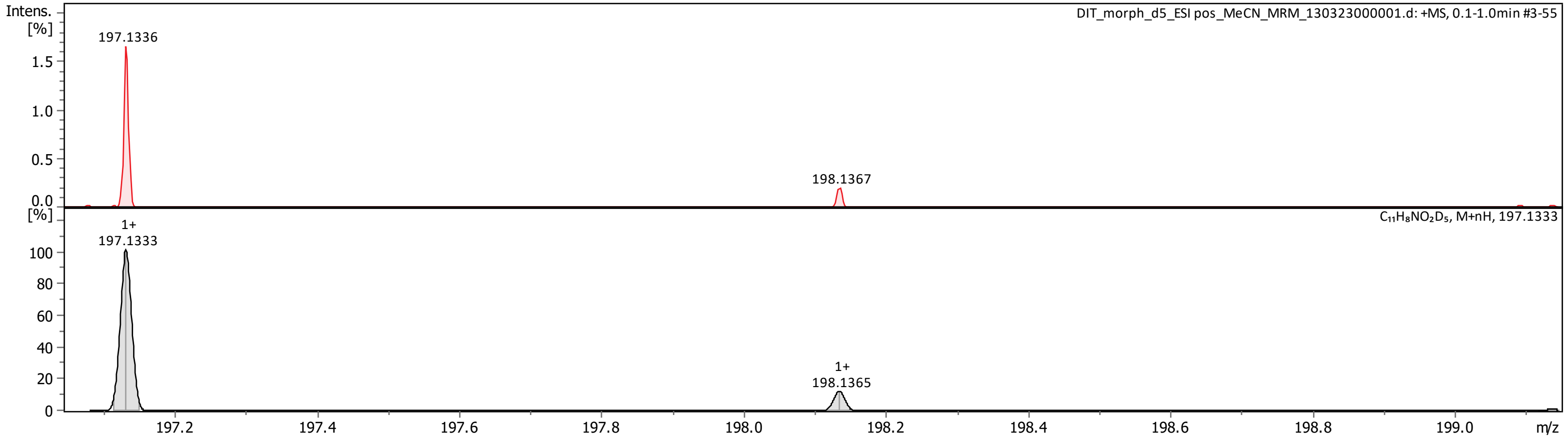
Intens.
[%]
DIT_morph_d5_ESI pos_MeCN_MRM_130323000001.d: +MS, 0.1-1.0min #3-55
197.1336
198.1367
1.5
1.0
0.5
0.0
[%]
$C_{11}H_8NO_2D_5$, M+nH, 197.1333
1+
197.1333
1+
198.1365
100
80
60
40
20
0
197.2
197.4
197.6
197.8
198.0
198.2
198.4
198.6
198.8
199.0
m/z

IR

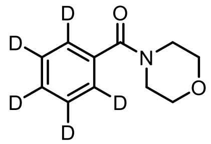

D
D
D
D
D
O
N
O

S26

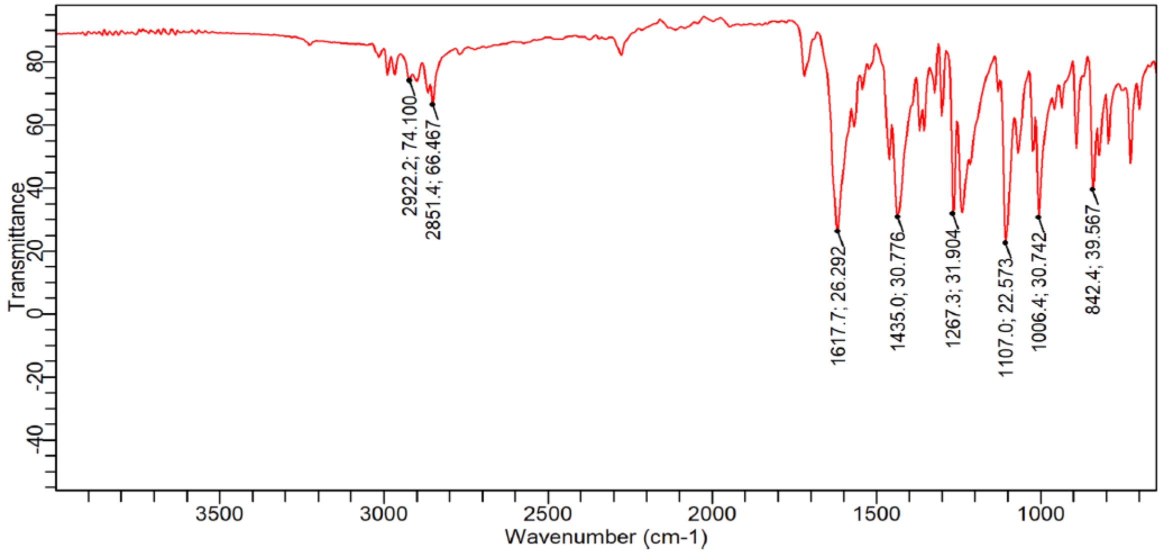

Transmittance
Wavenumber (cm-1)
2922.2; 74.100
2851.4; 66.467
1617.7; 26.292
1435.0; 30.776
1267.3; 31.904
1107.0; 22.573
1006.4; 30.742
842.4; 39.567

41 2-Methylene-4-morpholino-1-(1-piperidyl)butane-1,4-dione (S30)

^{1}H NMR

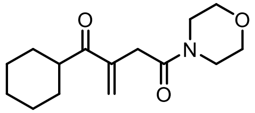

S30

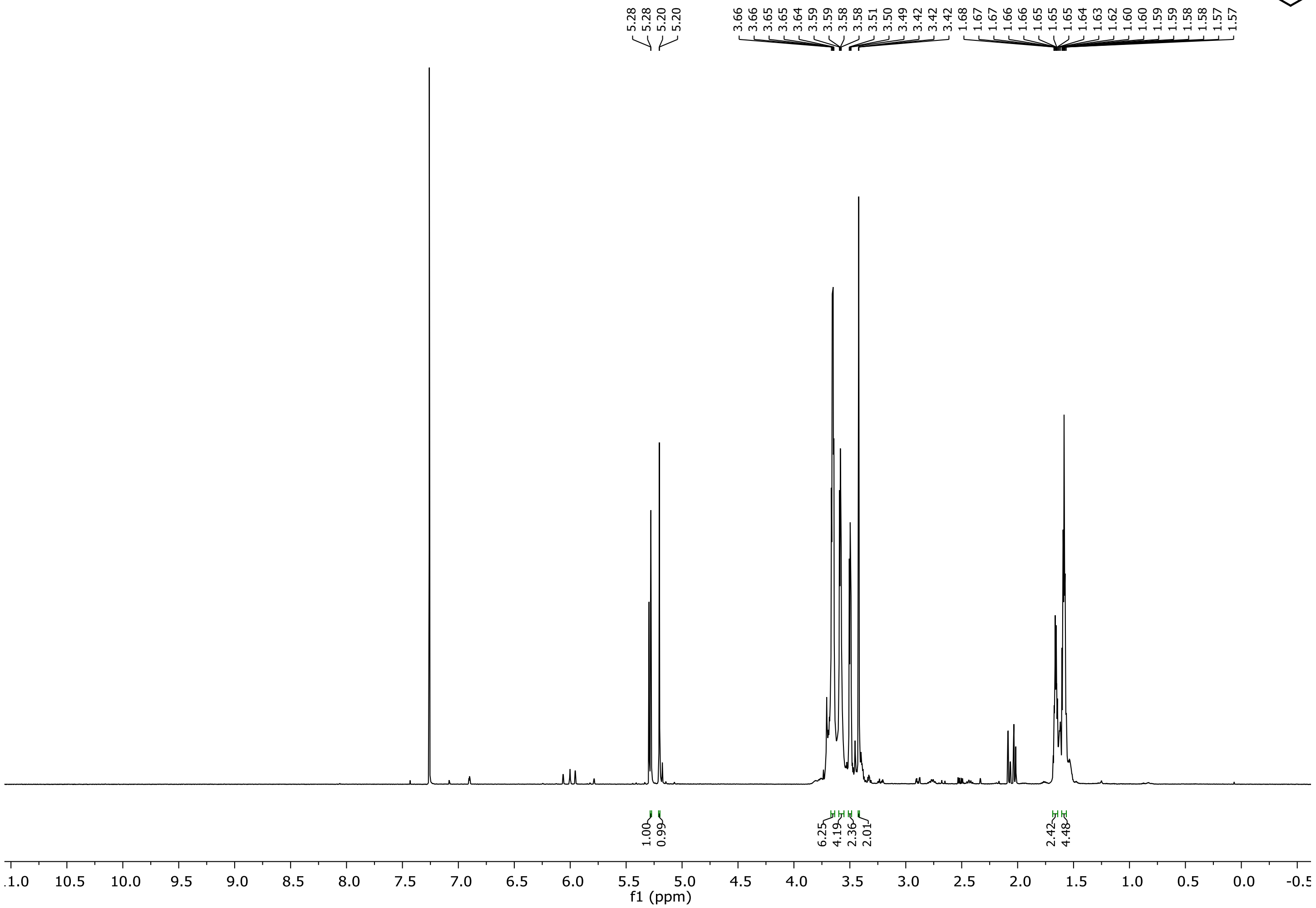

^{13}C NMR

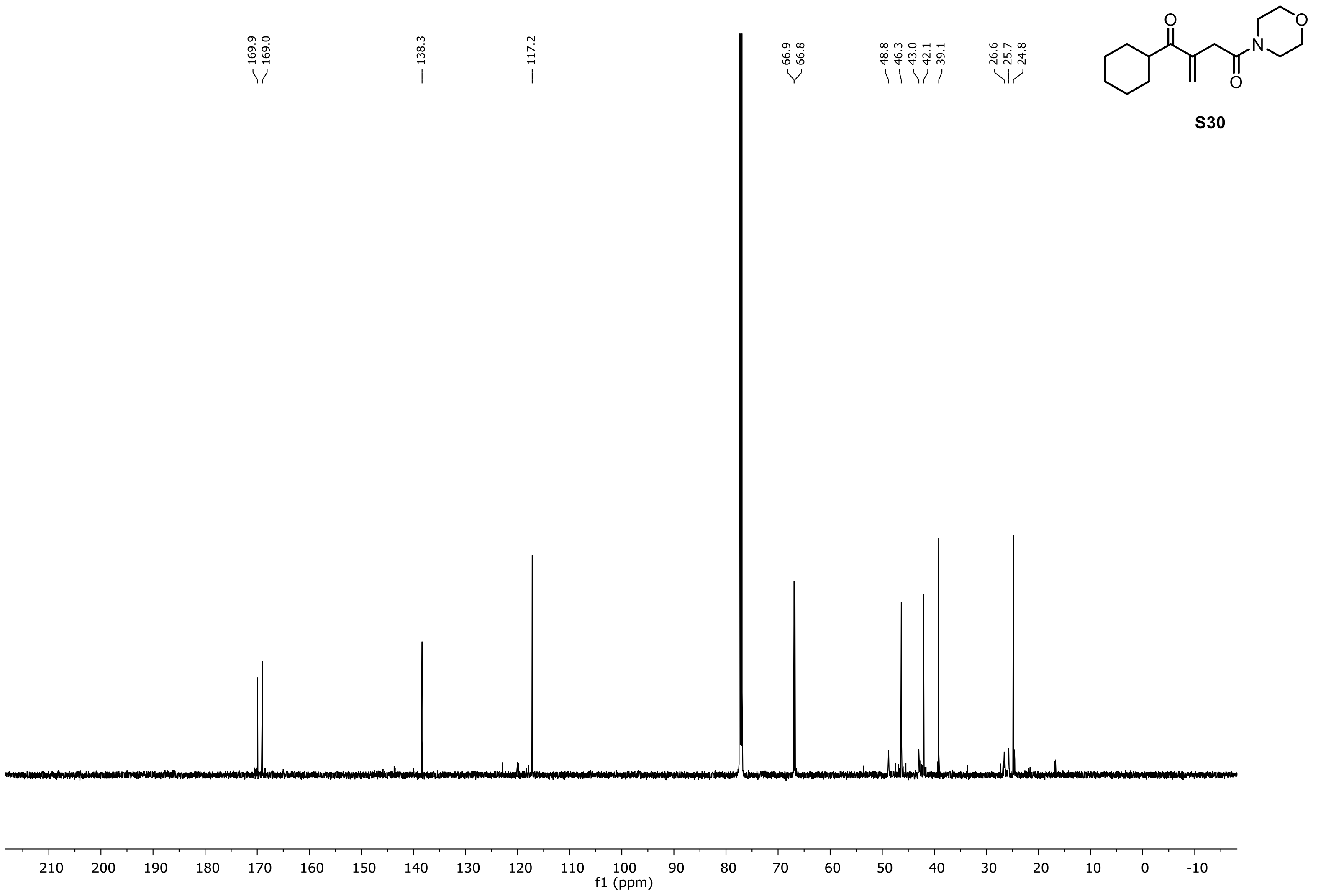
169.9
169.0
138.3
117.2
66.9
66.8
48.8
46.3
43.0
42.1
39.1
26.6
25.7
24.8
210 200 190 180 170 160 150 140 130 120 110 100 90 80 70 60 50 40 30 20 10 0 -10
f1 (ppm)
O
O
N
O

S30

^{1}H, ^{1}H COSY

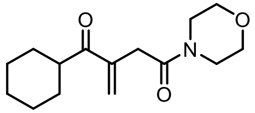

S30

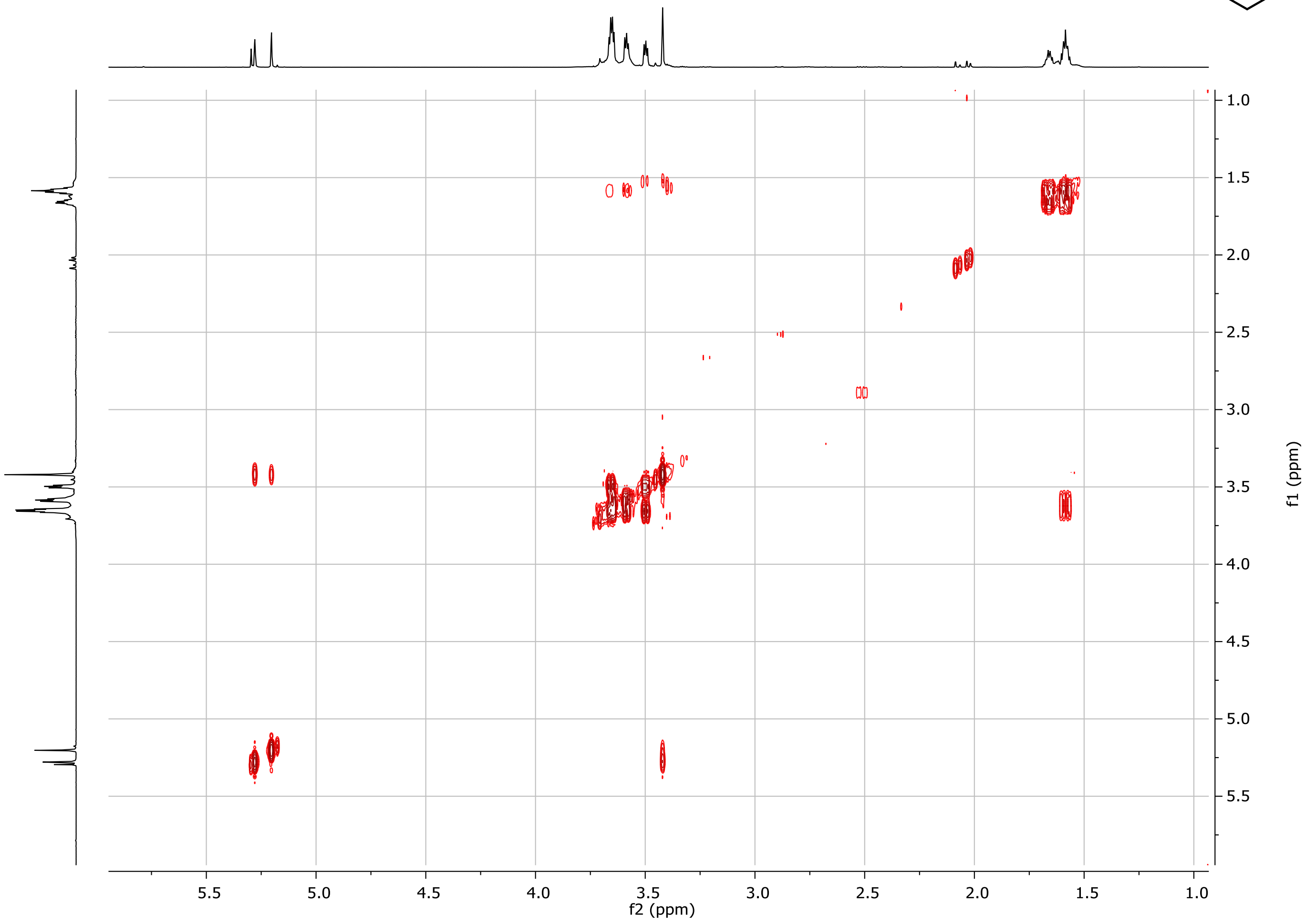

^1H, ^{13}C HMBC

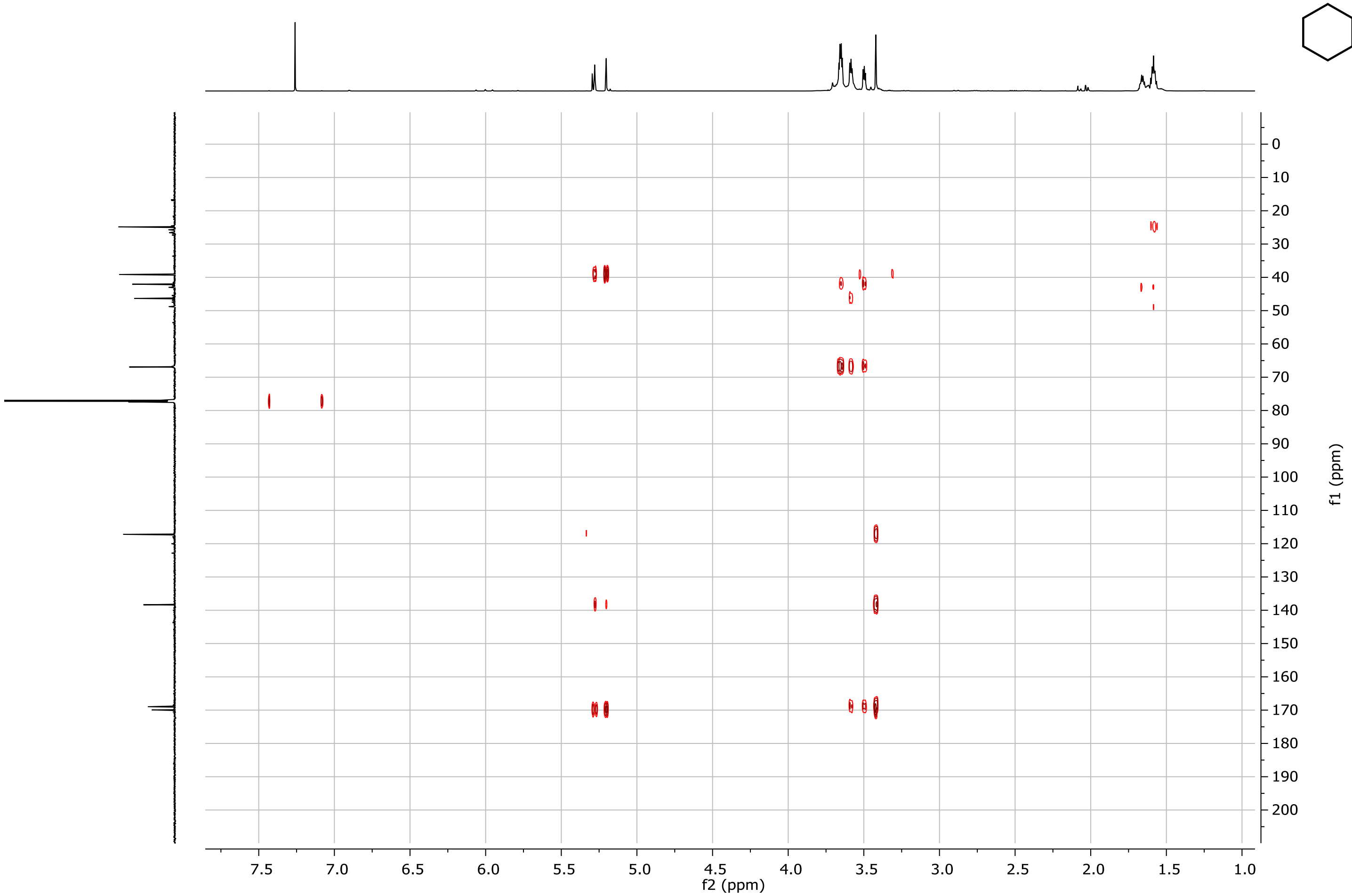

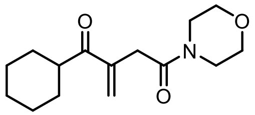

S30

^1H, ^{13}C HSQC

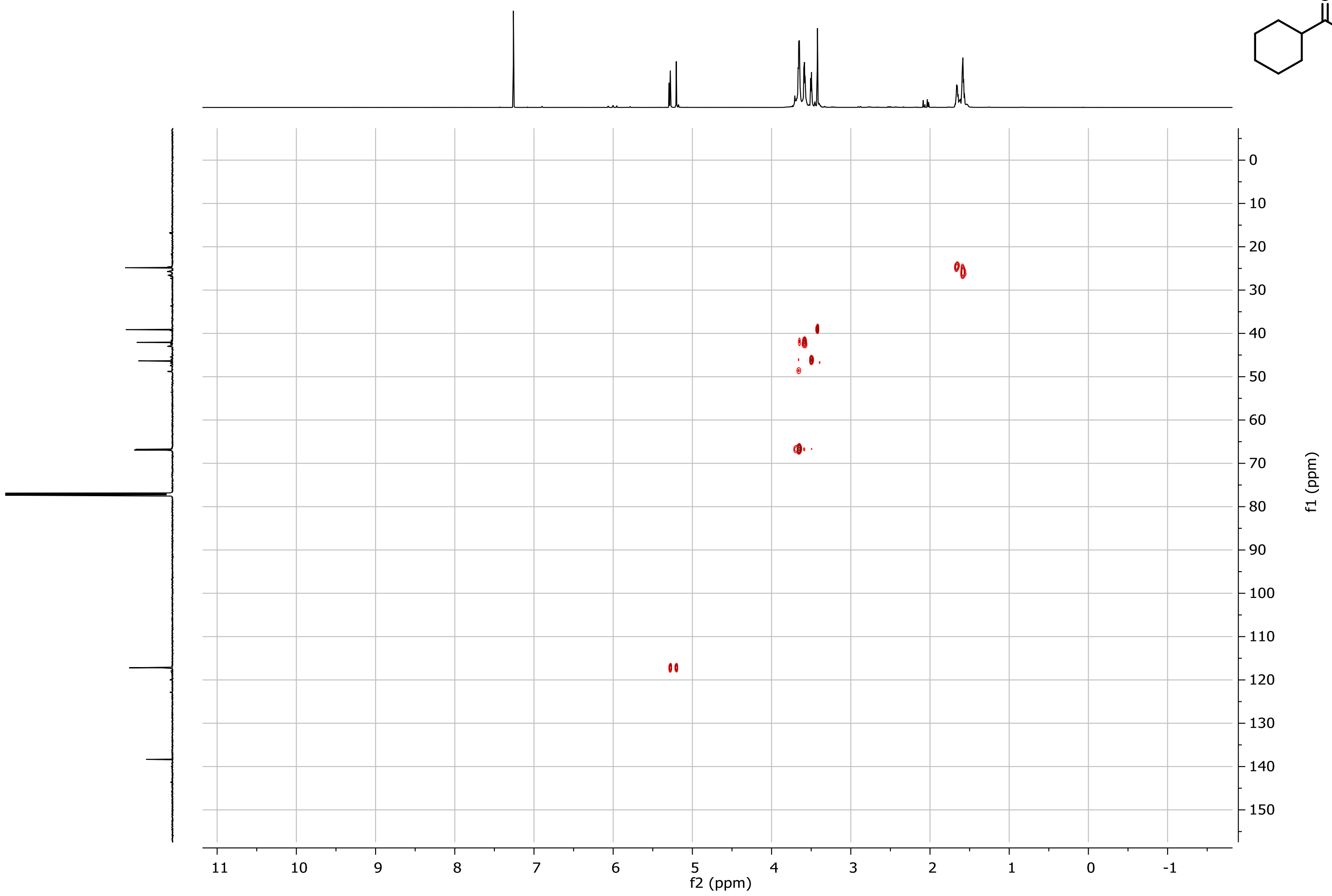

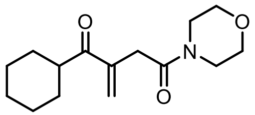

S30

HRMS

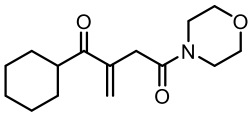

S30

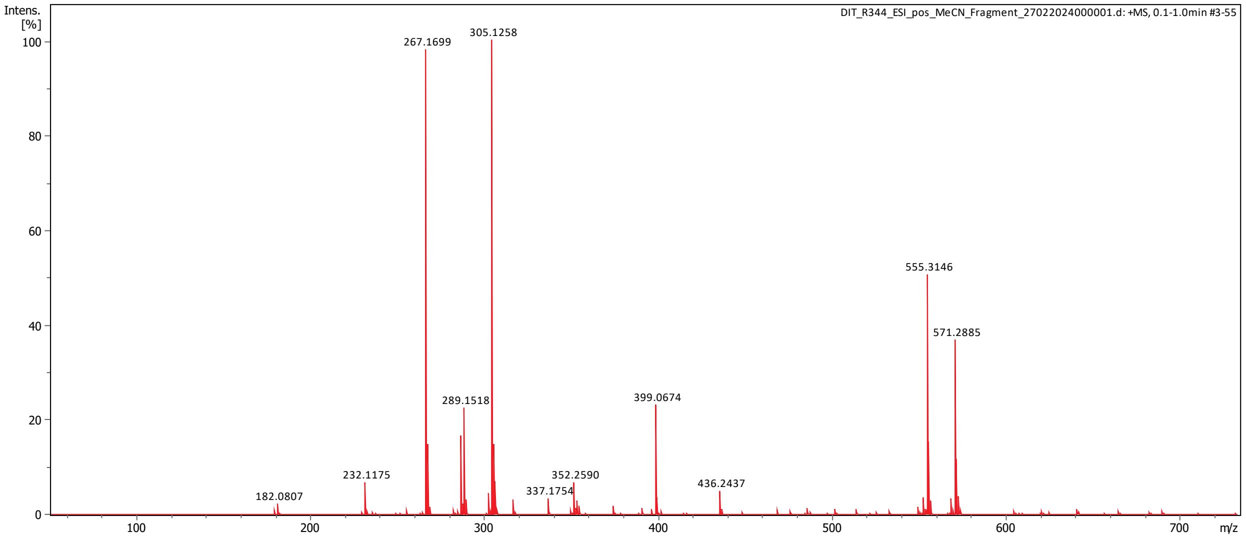

Intens. [%]
DIT_R344_ESI_pos_MeCN_Fragment_27022024000001.d: +MS, 0.1-1.0min #3-55
267.1699
305.1258
289.1518
399.0674
555.3146
571.2885
232.1175
182.0807
337.1754
352.2590
436.2437
m/z

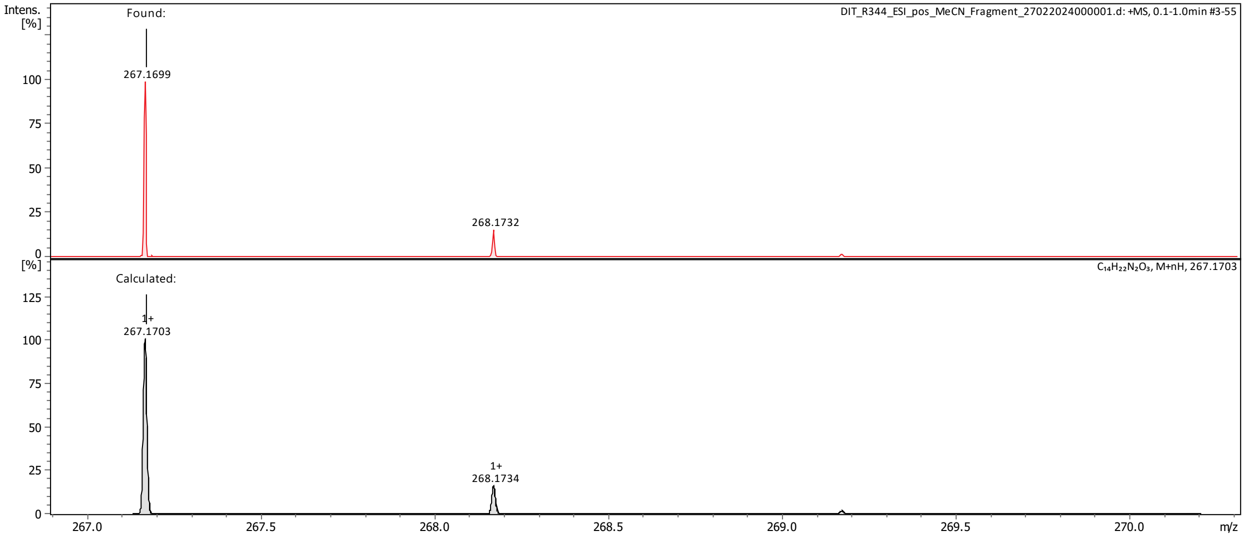

Intens. [%]
Found:
DIT_R344_ESI_pos_MeCN_Fragment_27022024000001.d: +MS, 0.1-1.0min #3-55
267.1699
268.1732
Calculated:
C14H22N2O3, M+nH, 267.1703
1+
267.1703
1+
268.1734
m/z

IR

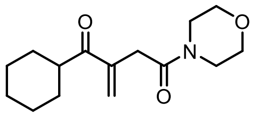
O
O
N
O

S30

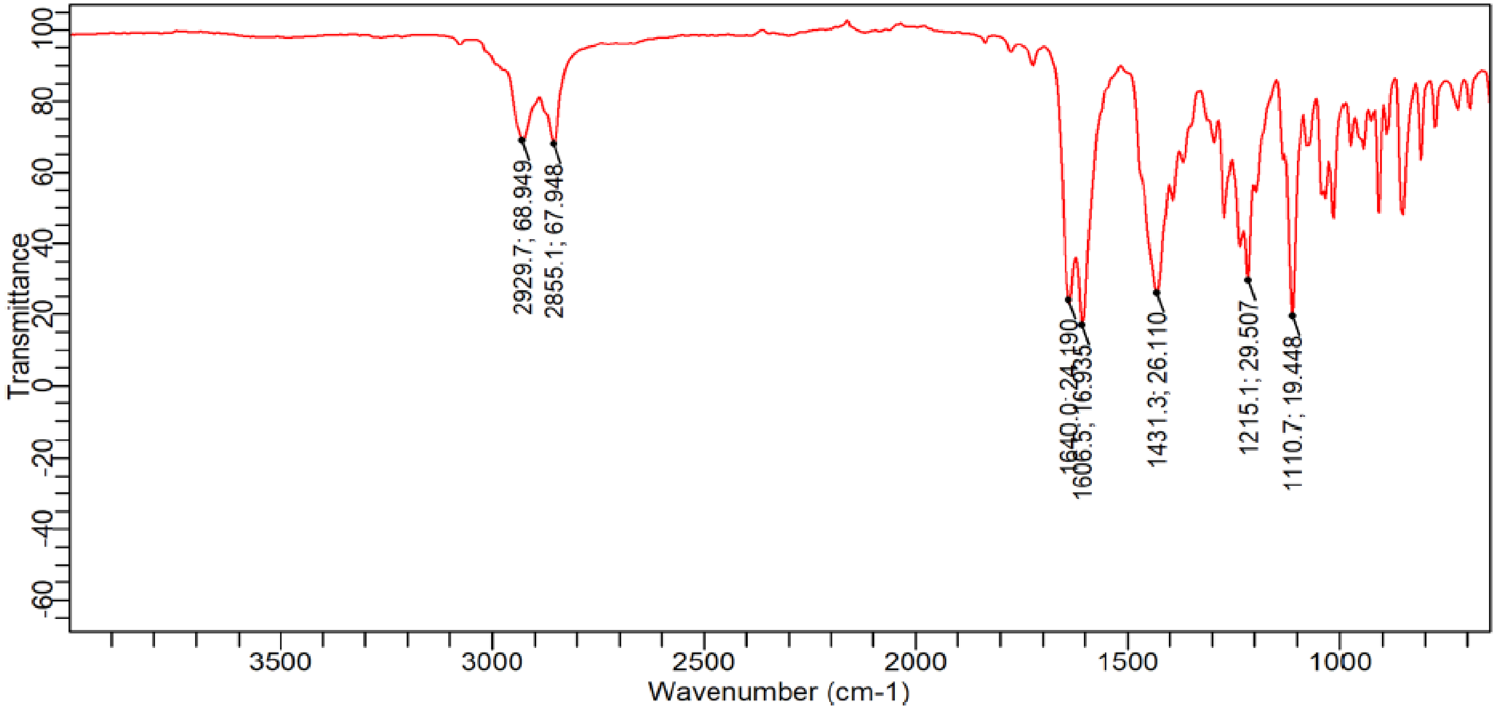
Transmittance
100
80
60
40
20
0
-20
-40
-60
3500
3000
2500
2000
1500
1000
Wavenumber (cm-1)
2929.7; 68.949
2855.1; 67.948
1640.0; 24.190
1606.5; 16.935
1431.3; 26.110
1215.1; 29.507
1110.7; 19.448

42 2-Methylene-1-morpholino-4-(1-piperidyl)butane-1,4-dione (S33)

^{1}H NMR

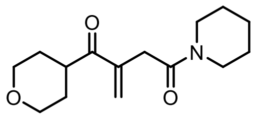

S33

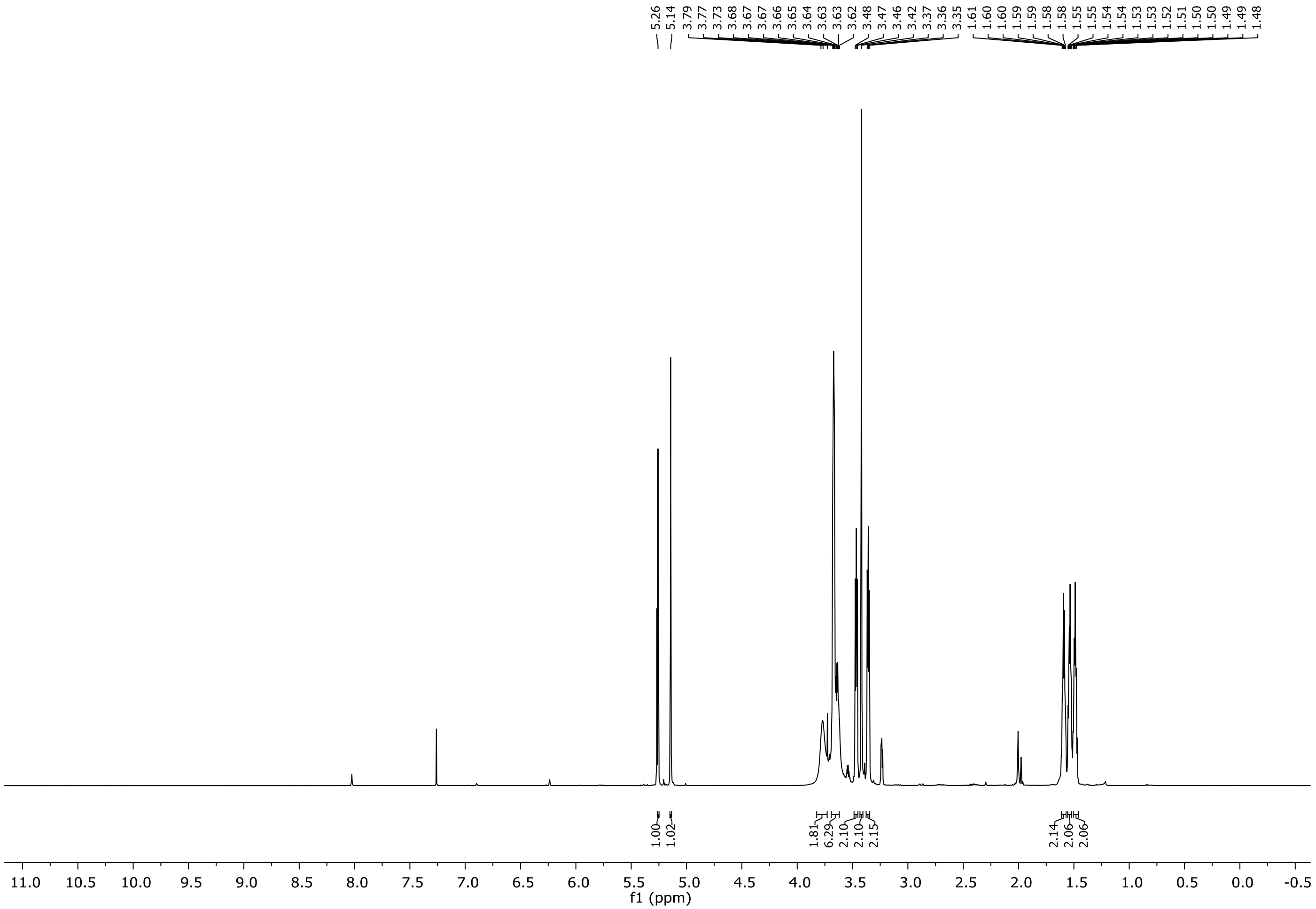

^{13}C NMR

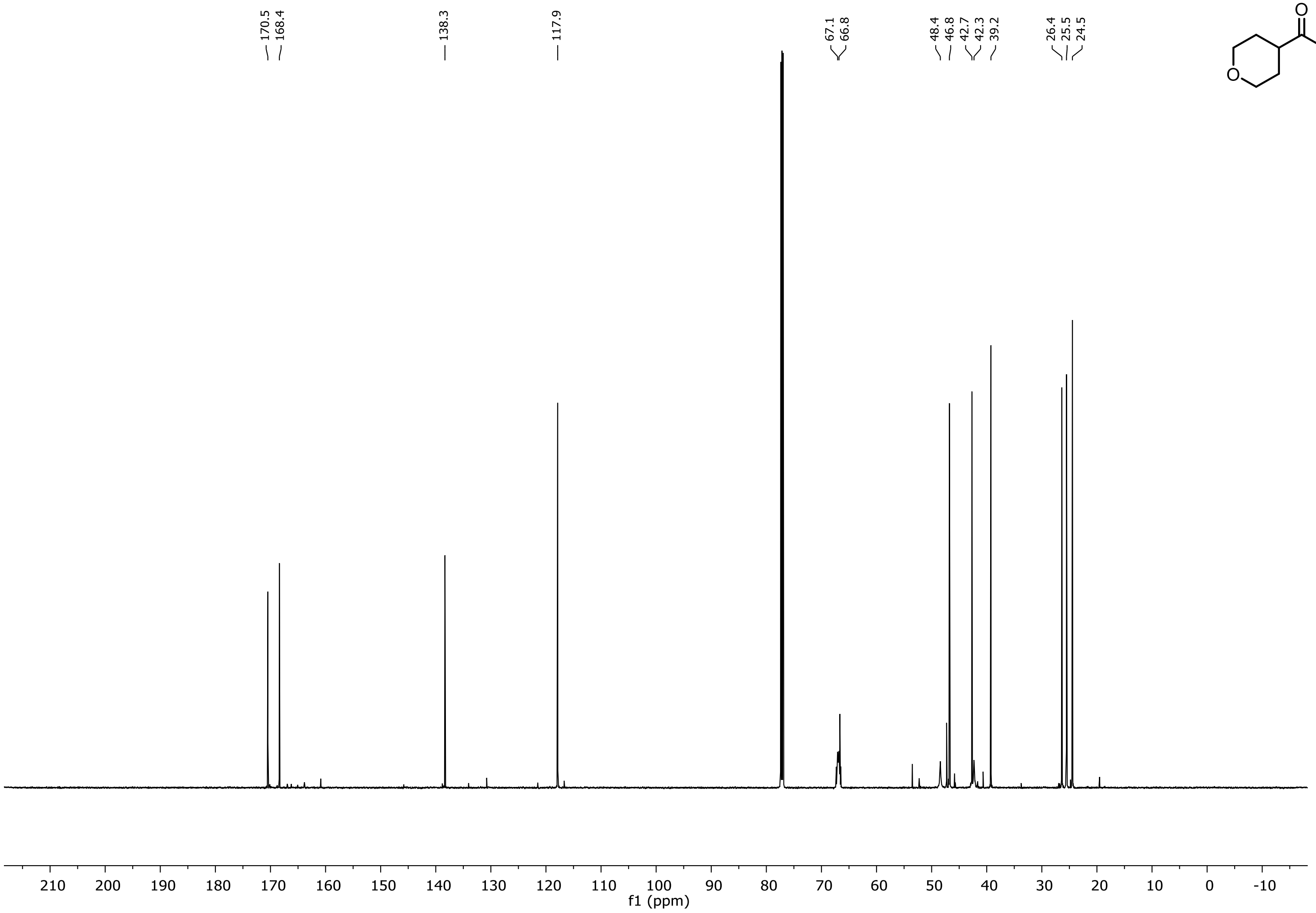
170.5
168.4
138.3
117.9
67.1
66.8
48.4
46.8
42.7
42.3
39.2
26.4
25.5
24.5
210
200
190
180
170
160
150
140
130
120
110
100
90
80
70
60
50
40
30
20
10
0
-10
f1 (ppm)

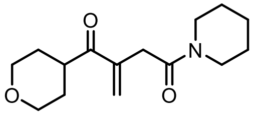
O
O
N
O

S33

^{1}H, ^{1}H COSY

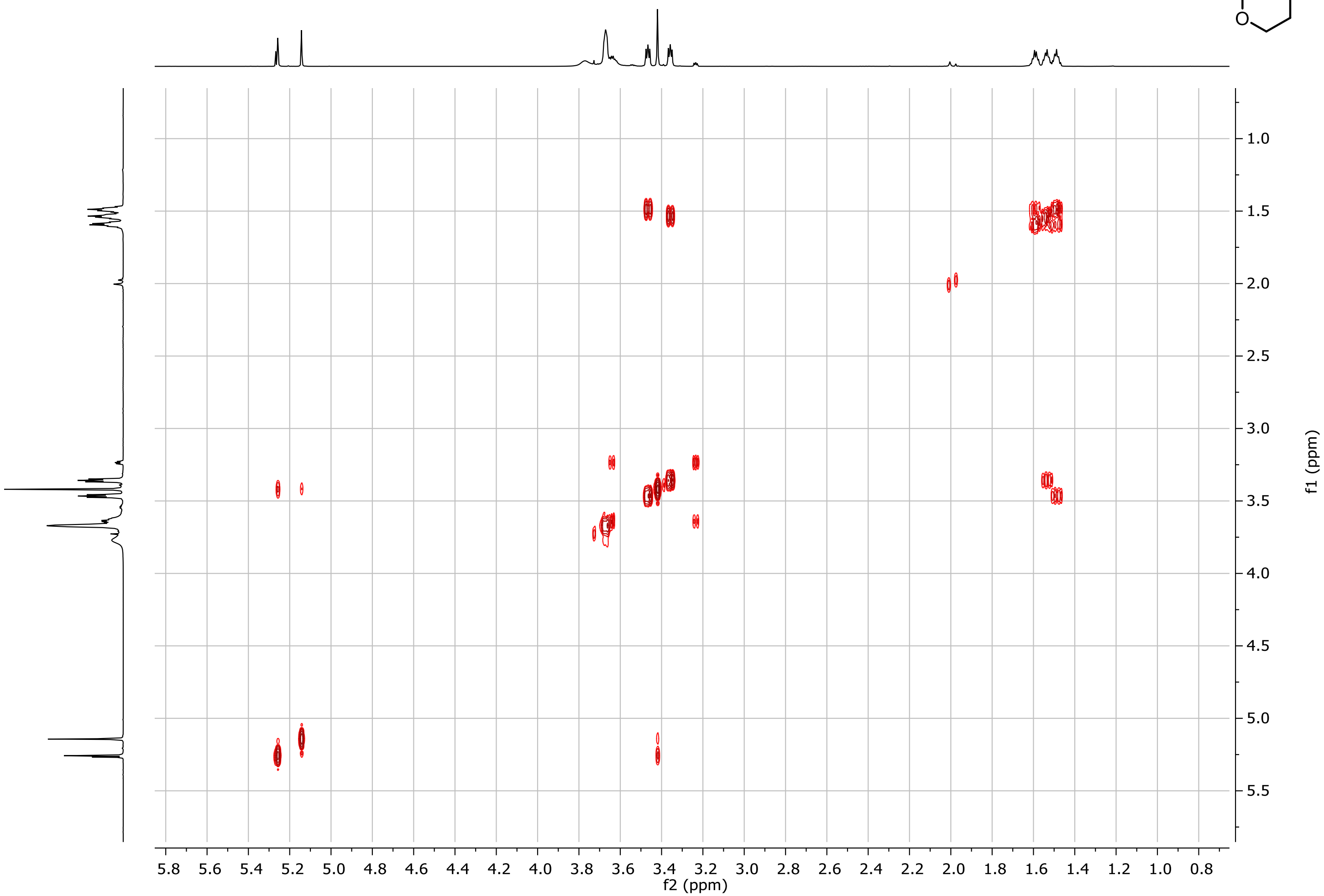

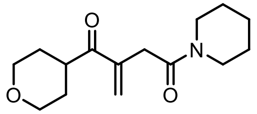

S33

^{1}H, ^{13}C HMBC

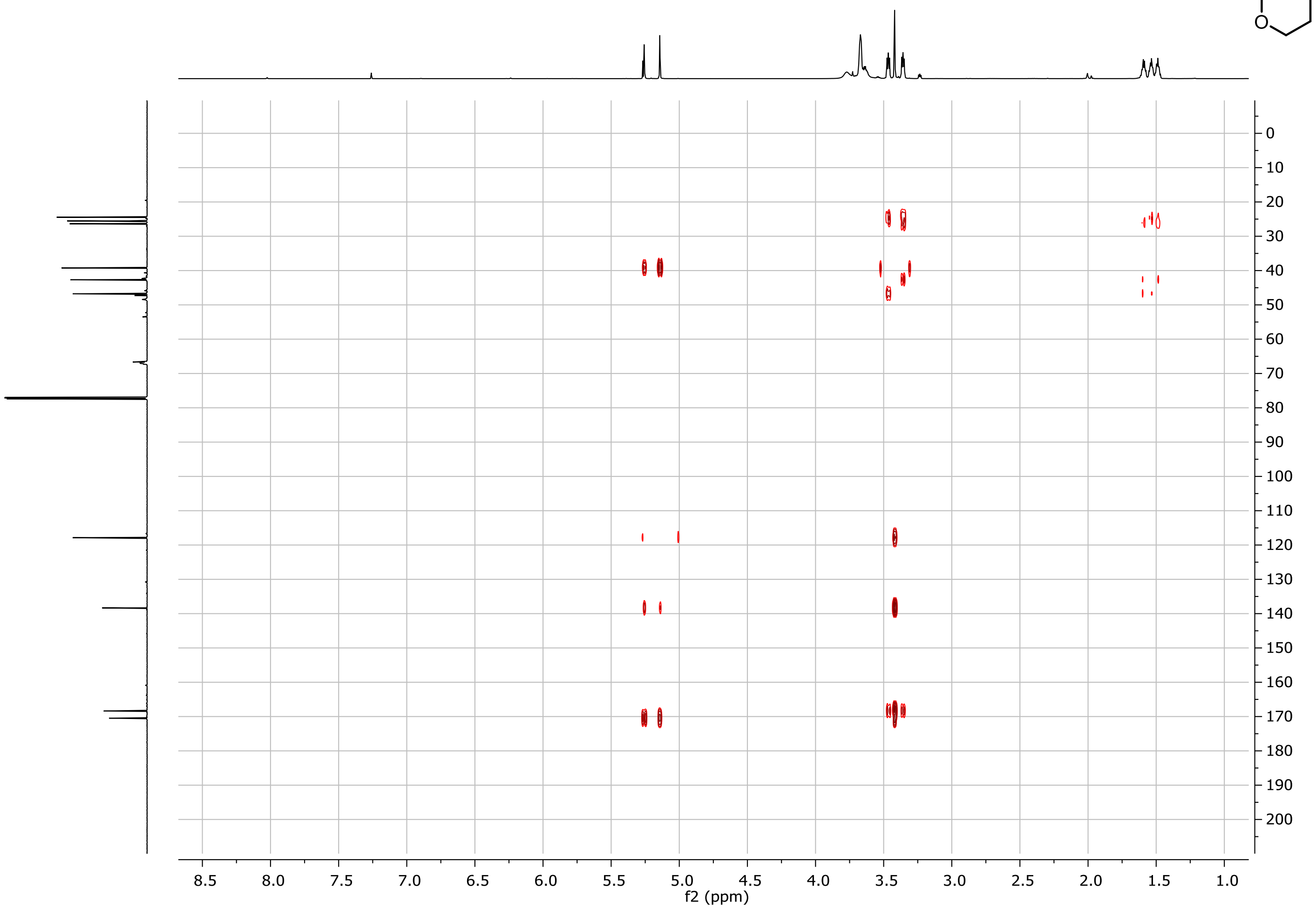

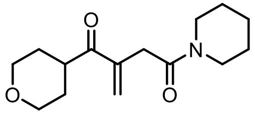

S33

^{1}H, ^{13}C HSQC

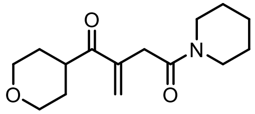

S33

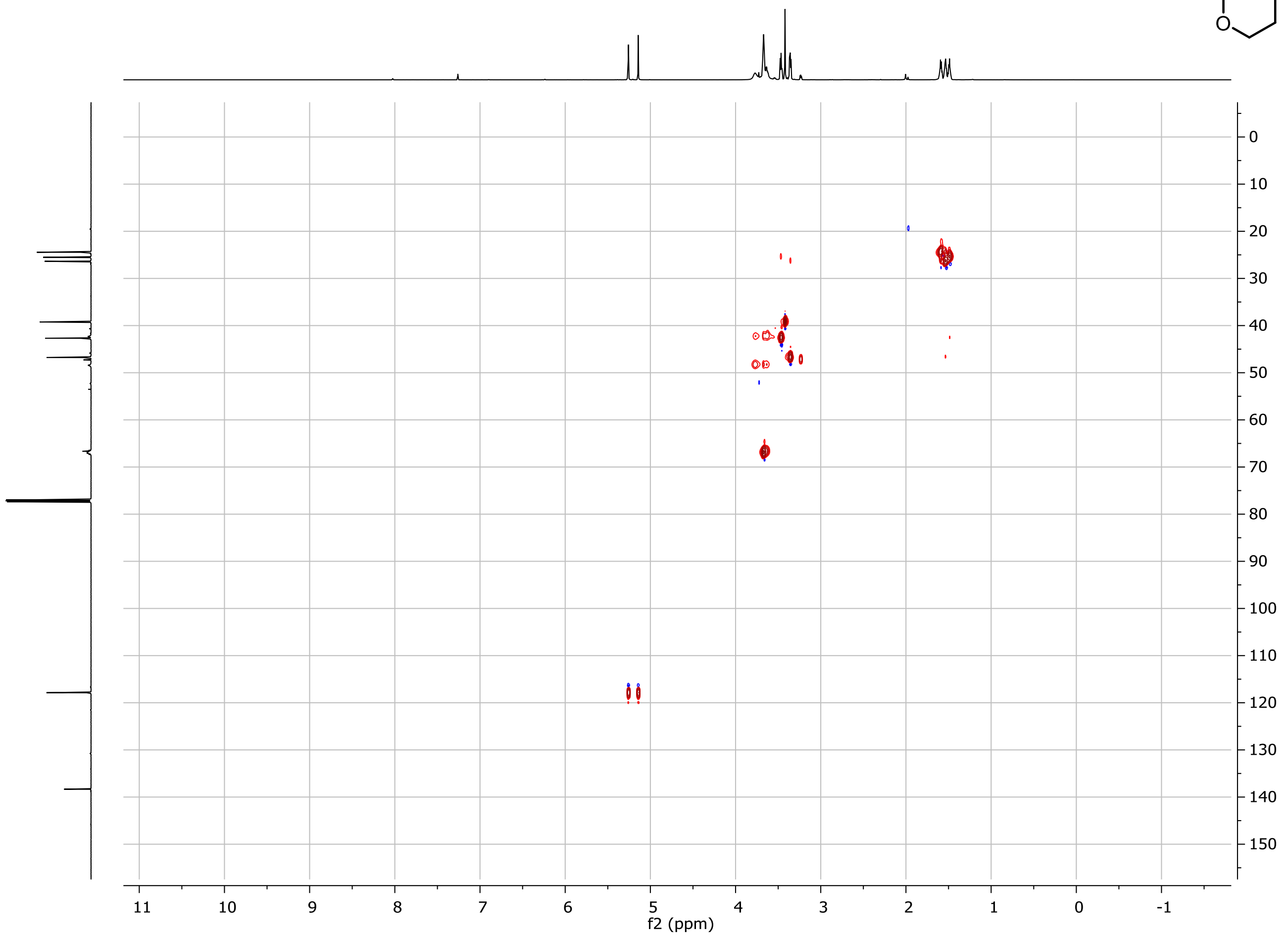

HRMS

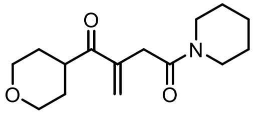

S33

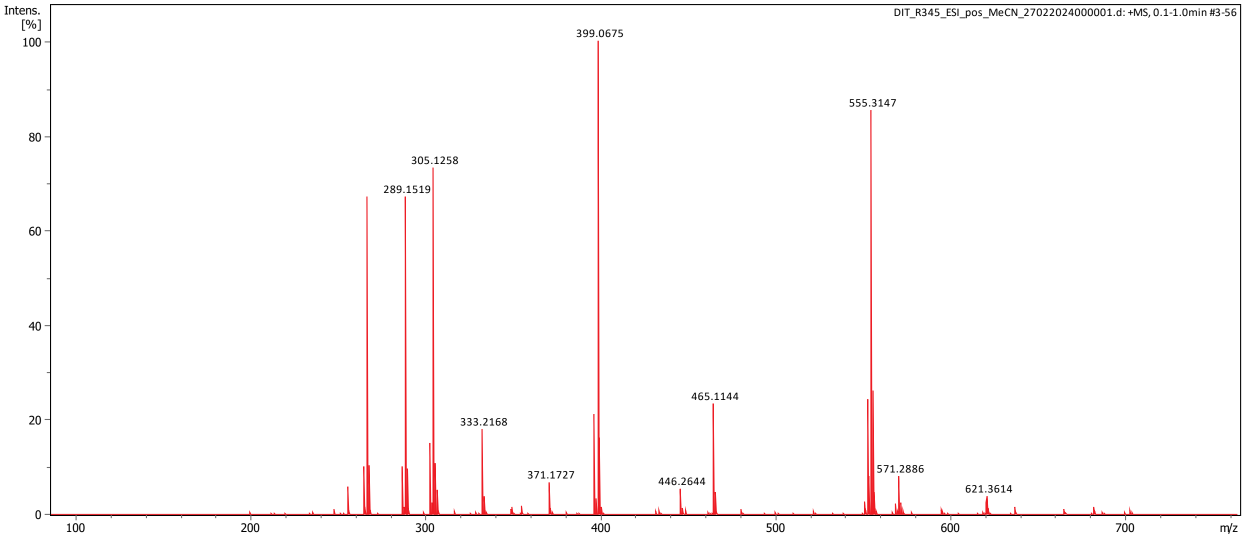

Intens.
[%]
DIT_R345_ESI_pos_MeCN_27022024000001.d: +MS, 0.1-1.0min #3-56
399.0675
555.3147
305.1258
289.1519
465.1144
333.2168
371.1727
446.2644
571.2886
621.3614
m/z

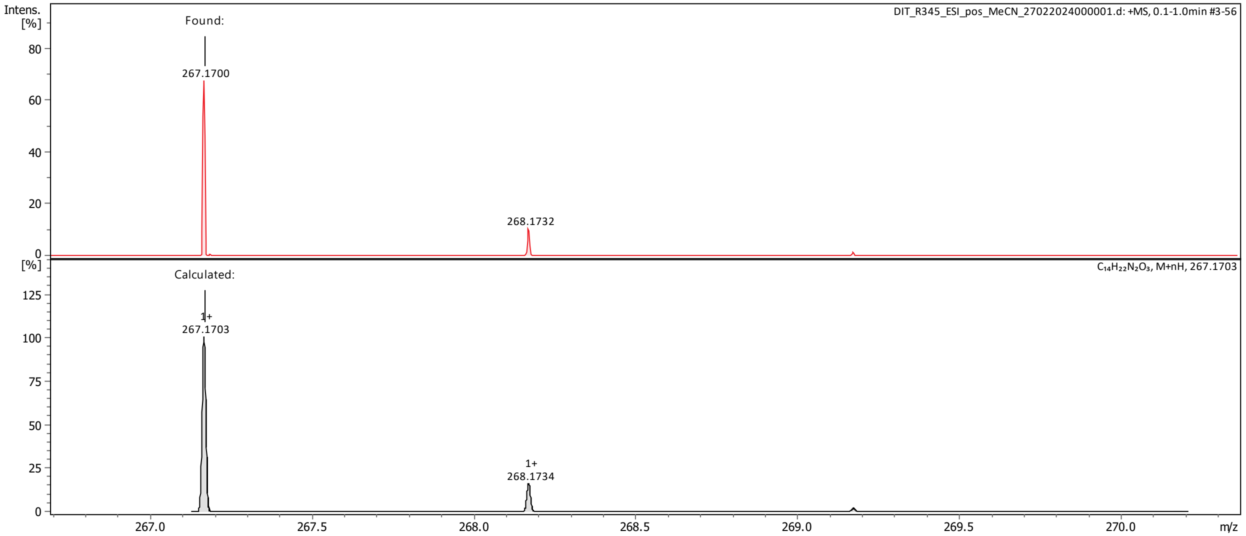

Intens.
[%]
DIT_R345_ESI_pos_MeCN_27022024000001.d: +MS, 0.1-1.0min #3-56
Found:
267.1700
268.1732
Calculated:
C14H22N2O3, M+nH, 267.1703
1+
267.1703
1+
268.1734
m/z

IR

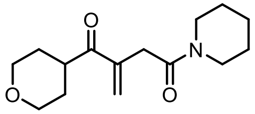

S33

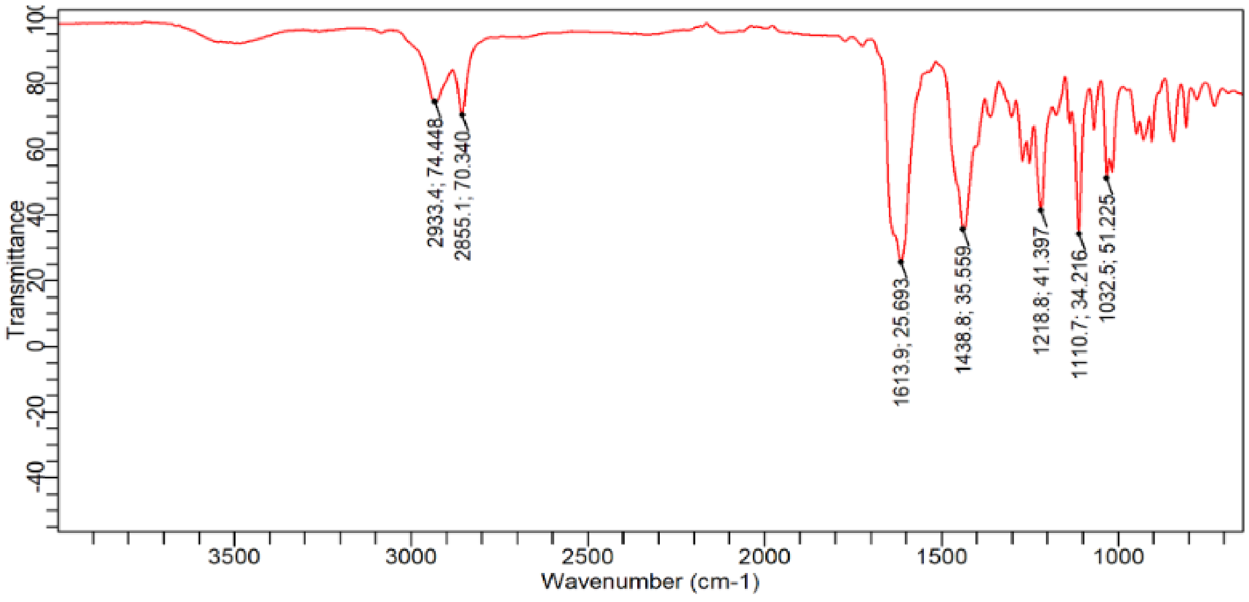
Transmittance
Wavenumber (cm-1)
2933.4; 74.448
2855.1; 70.340
1613.9; 25.693
1438.8; 35.559
1218.8; 41.397
1110.7; 34.216
1032.5; 51.225

43 1-Morpholino-10-(1-piperidyl)decane-1,10-dione (S35)

^{1}H NMR

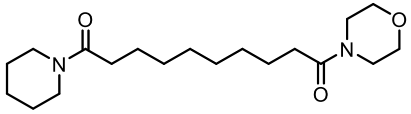

S35

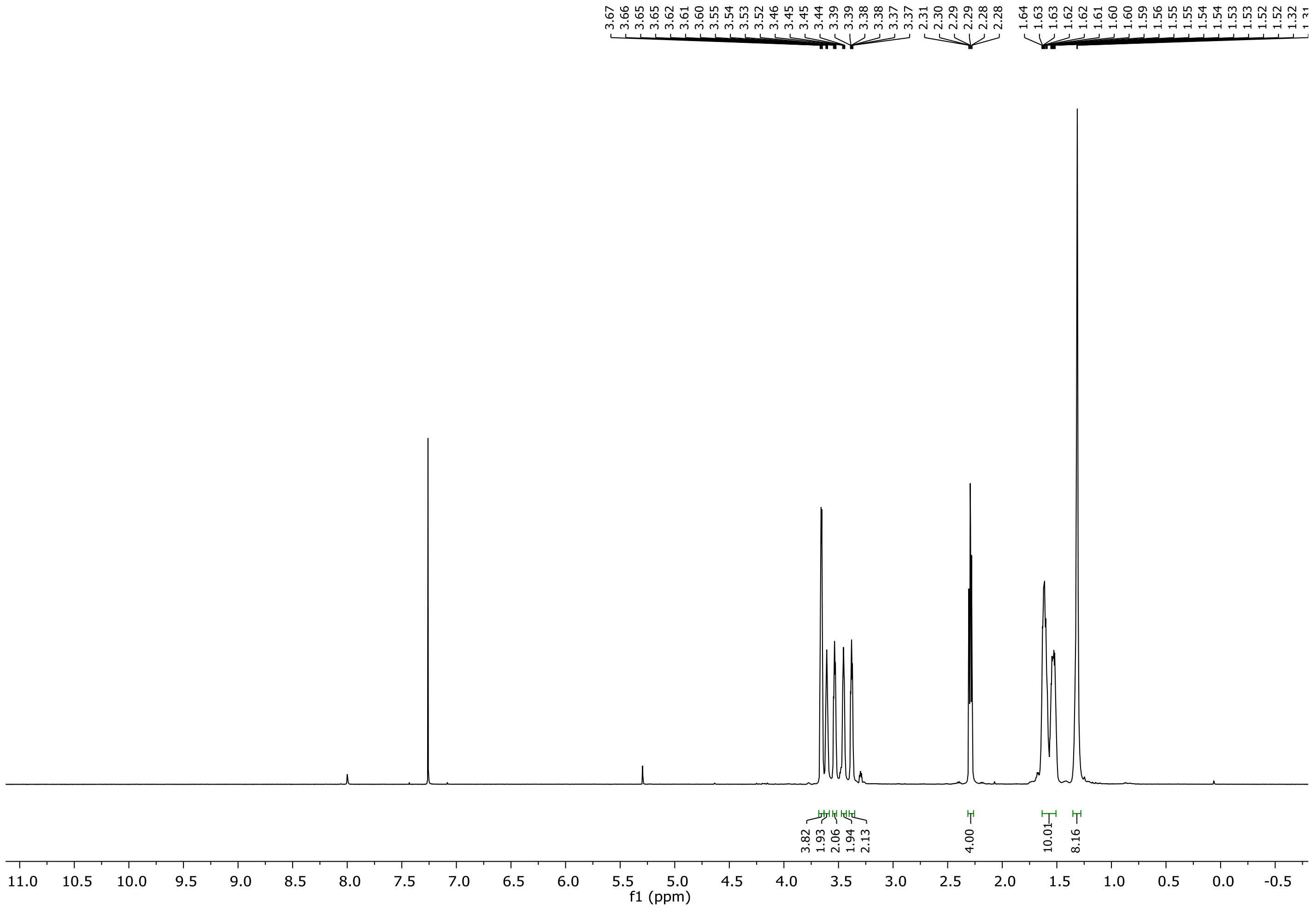

^{13}C NMR

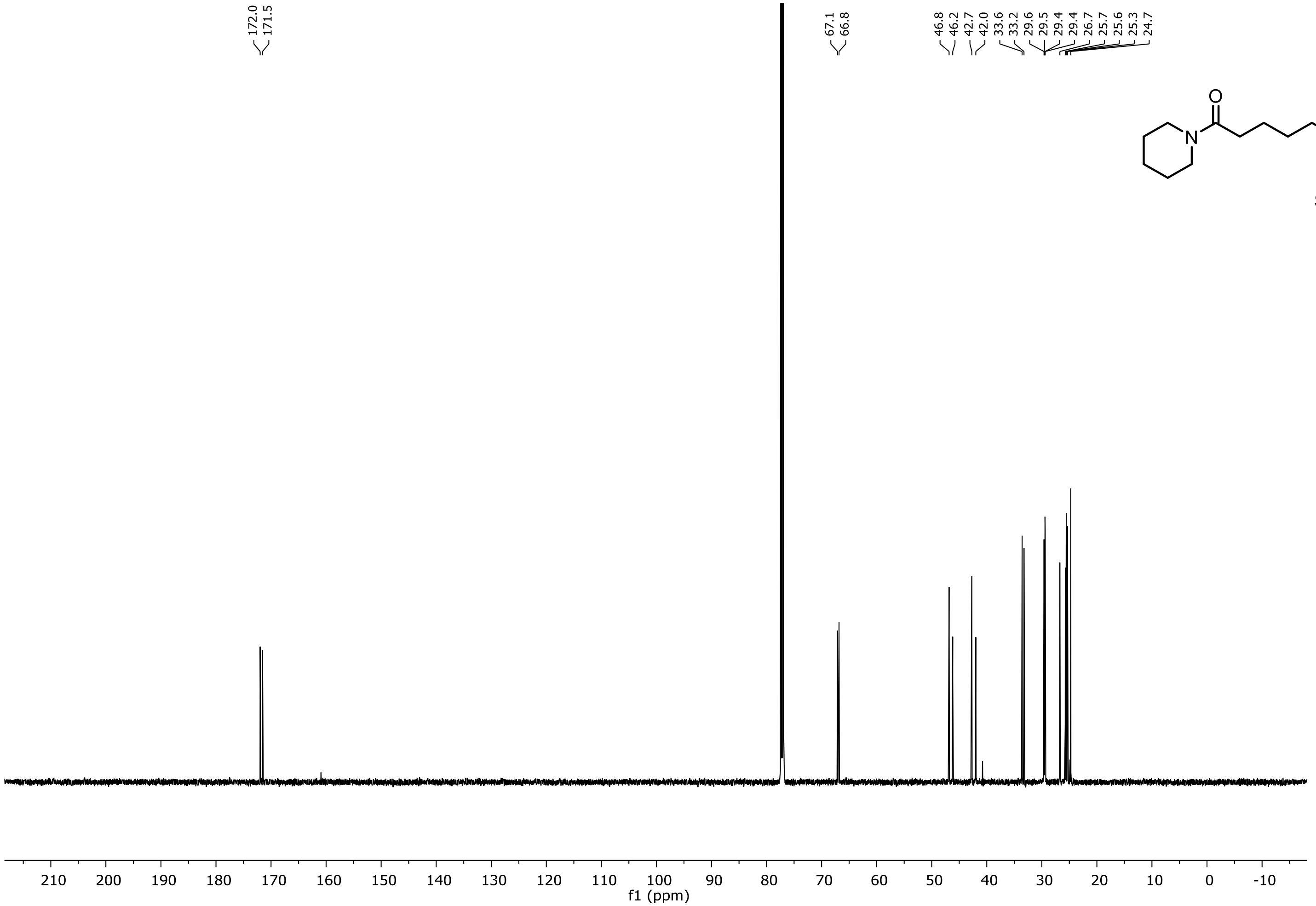
172.0
171.5
67.1
66.8
46.8
46.2
42.7
42.0
33.6
33.2
29.6
29.5
29.4
29.4
26.7
25.7
25.6
25.3
24.7
210 200 190 180 170 160 150 140 130 120 110 100 90 80 70 60 50 40 30 20 10 0 -10
f1 (ppm)

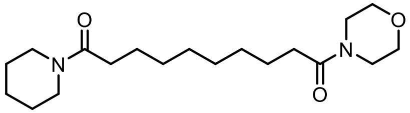
O
N
O
N
O

S35

^{1}H, ^{1}H COSY

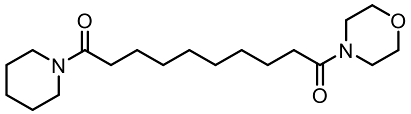

O
N
O
N
O
S35

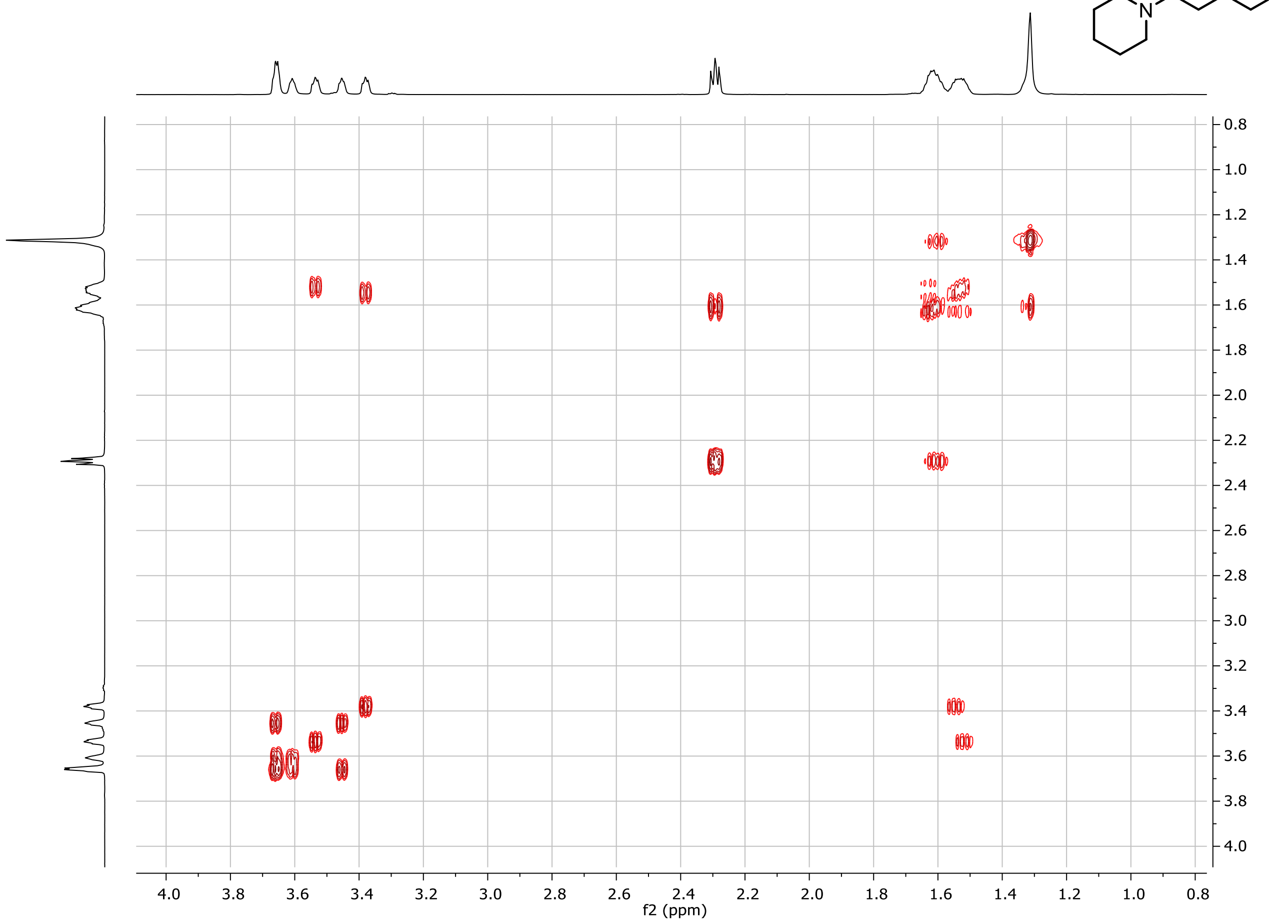

0.8
1.0
1.2
1.4
1.6
1.8
2.0
2.2
2.4
2.6
2.8
3.0
3.2
3.4
3.6
3.8
4.0
f1 (ppm)
4.0
3.8
3.6
3.4
3.2
3.0
2.8
2.6
2.4
2.2
2.0
1.8
1.6
1.4
1.2
1.0
0.8
f2 (ppm)

^{1}H, ^{13}C HMBC

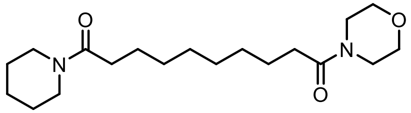

S35

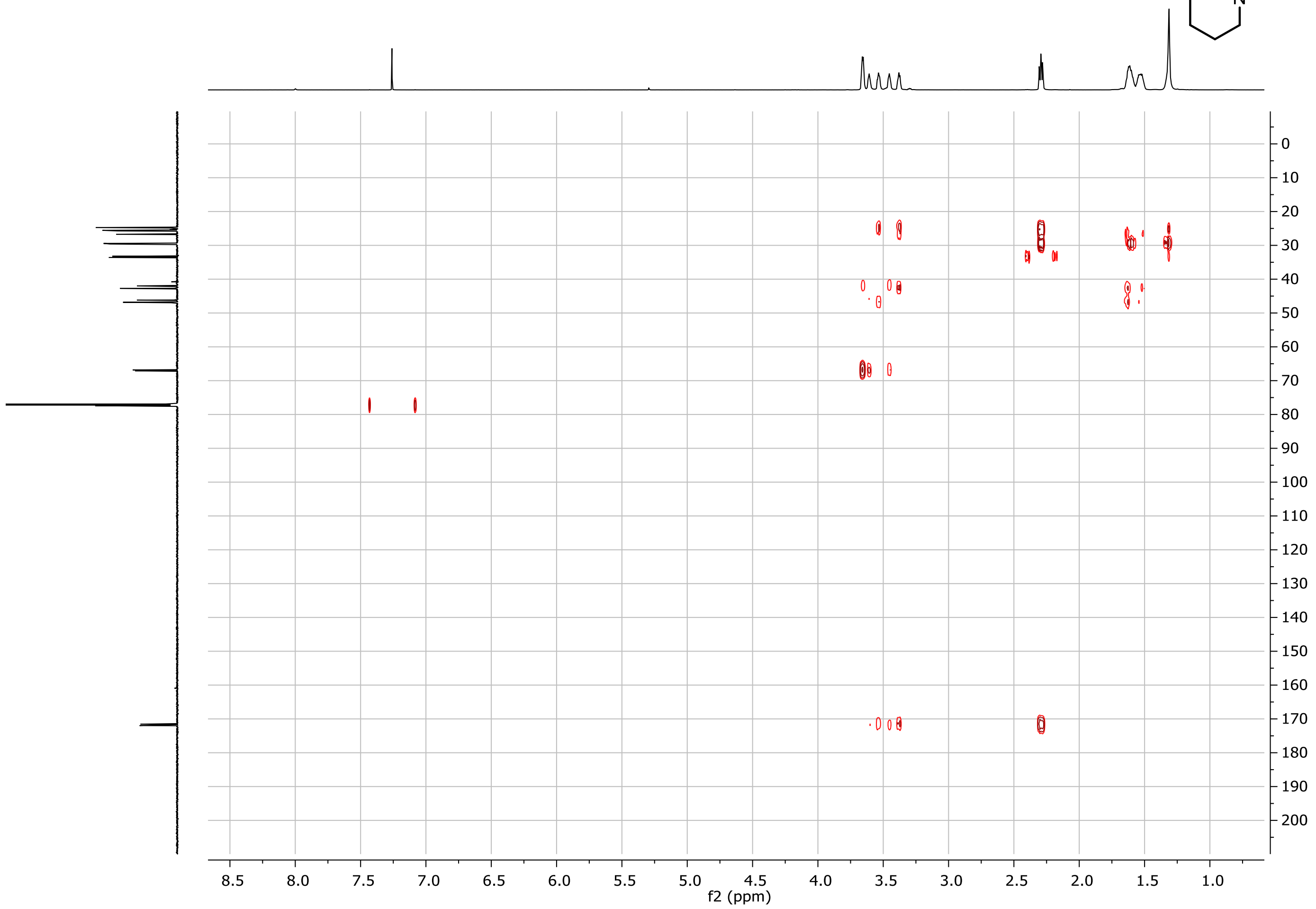

^{1}H, ^{13}C HSQC

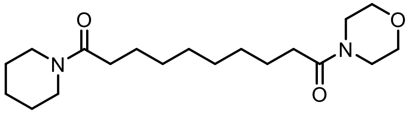

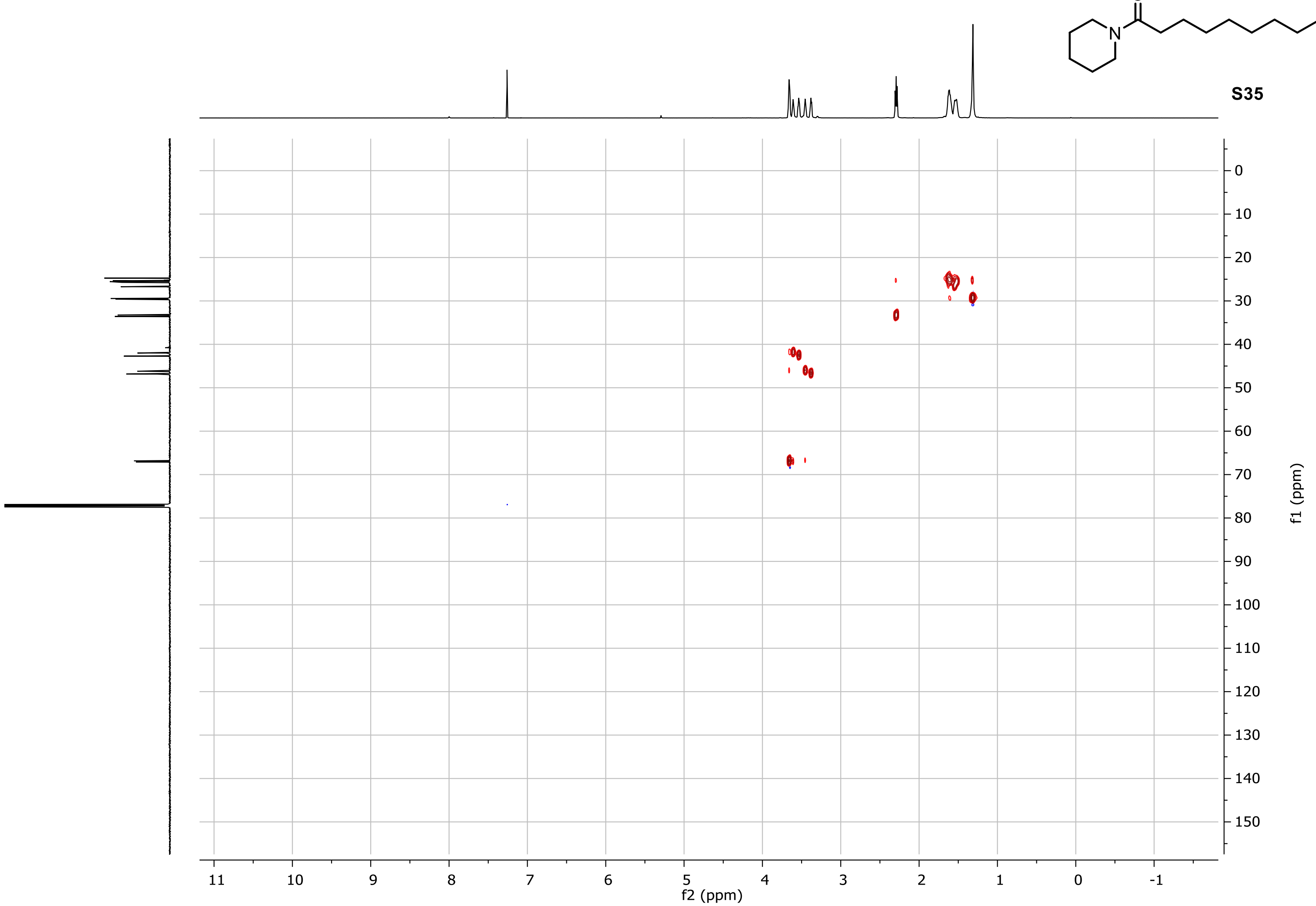

HRMS

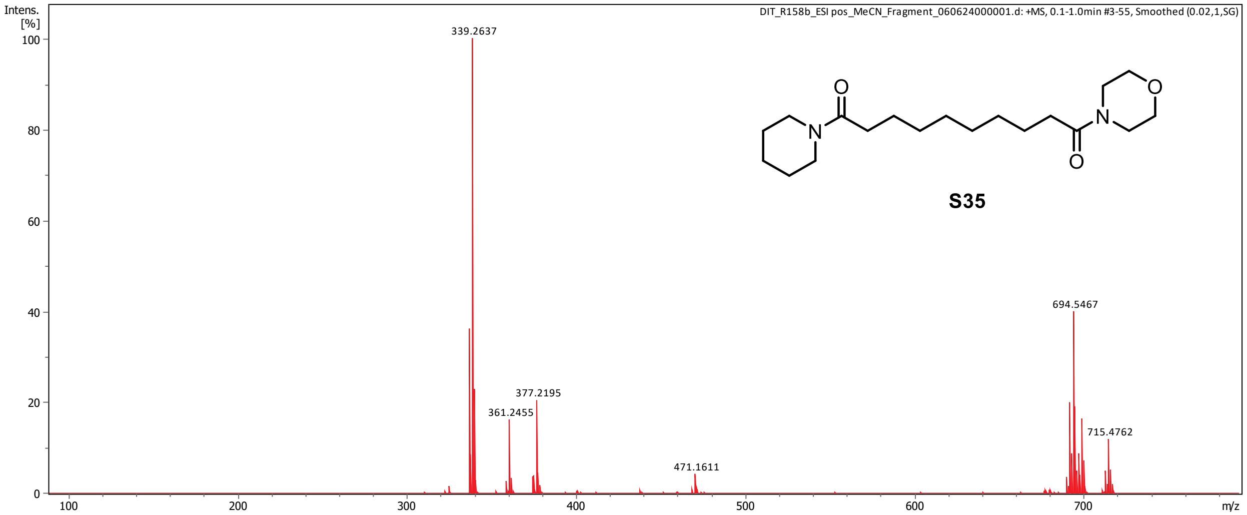

DIT_R158b_ESI pos_MeCN_Fragment_060624000001.d: +MS, 0.1-1.0min #3-55, Smoothed (0.02,1,SG)
Intens. [%]
339.2637
377.2195
361.2455
471.1611
694.5467
715.4762
S35
m/z

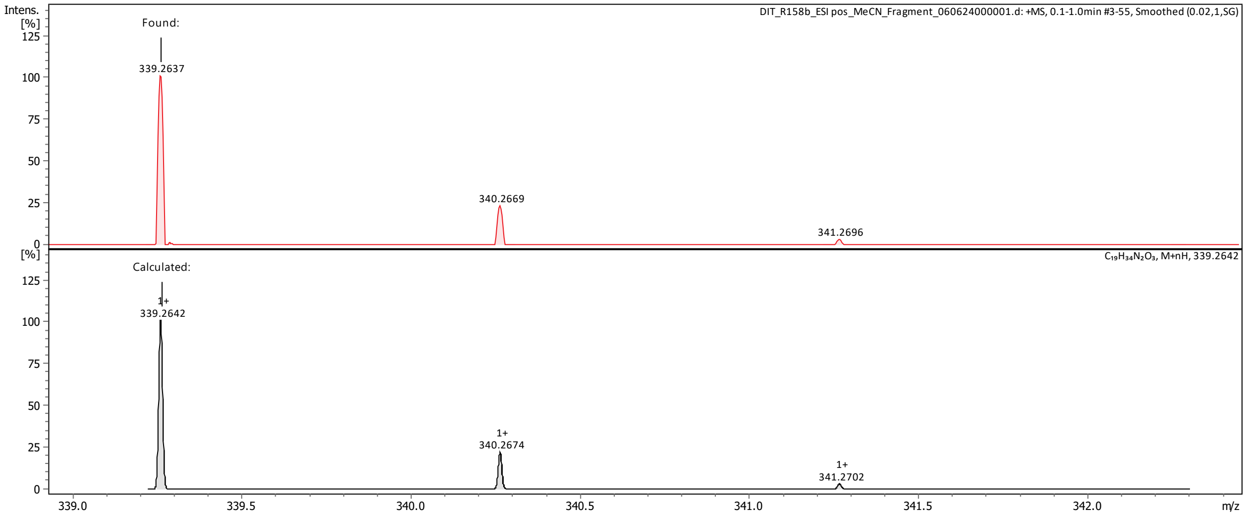

DIT_R158b_ESI pos_MeCN_Fragment_060624000001.d: +MS, 0.1-1.0min #3-55, Smoothed (0.02,1,SG)
Intens. [%]
Found:
339.2637
340.2669
341.2696
Calculated:
$C_{19}H_{34}N_2O_3$, M+nH, 339.2642
1+ 339.2642
1+ 340.2674
1+ 341.2702
m/z

IR

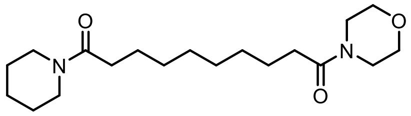

S35

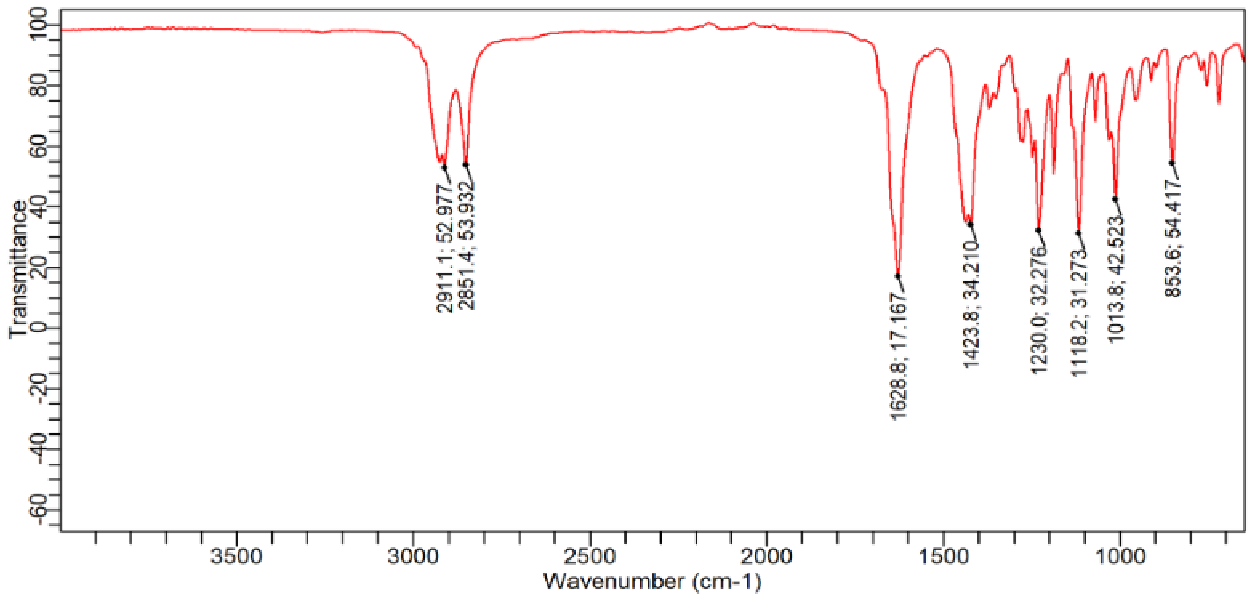
Transmittance
Wavenumber (cm-1)
2911.1; 52.977
2851.4; 53.932
1628.8; 17.167
1423.8; 34.210
1230.0; 32.276
1118.2; 31.273
1013.8; 42.523
853.6; 54.417

44 [4-(Hydroxymethyl)phenyl]-(1-piperidyl)methanone (S36)

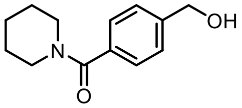

S36

^{1}H NMR

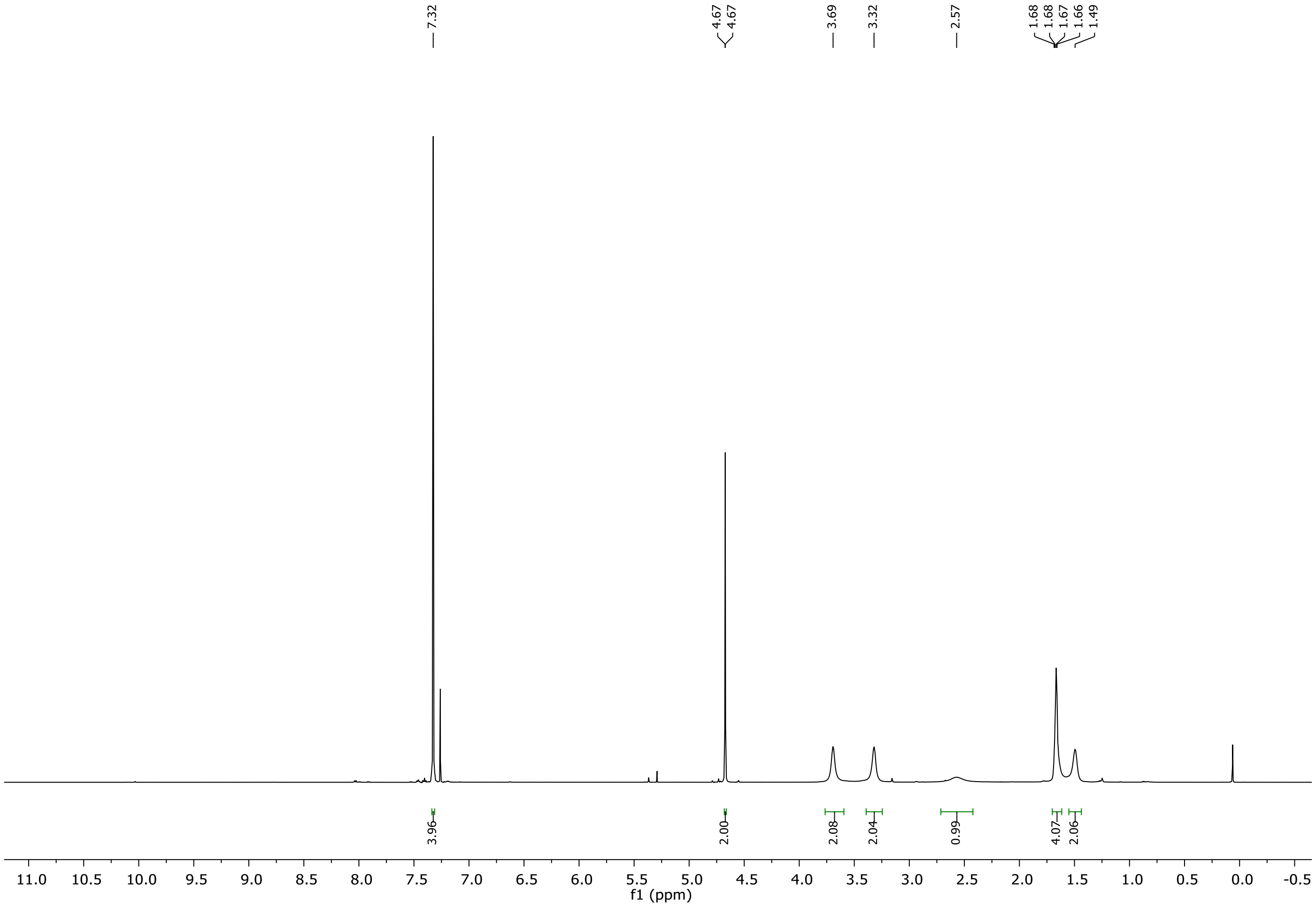

^{13}C NMR

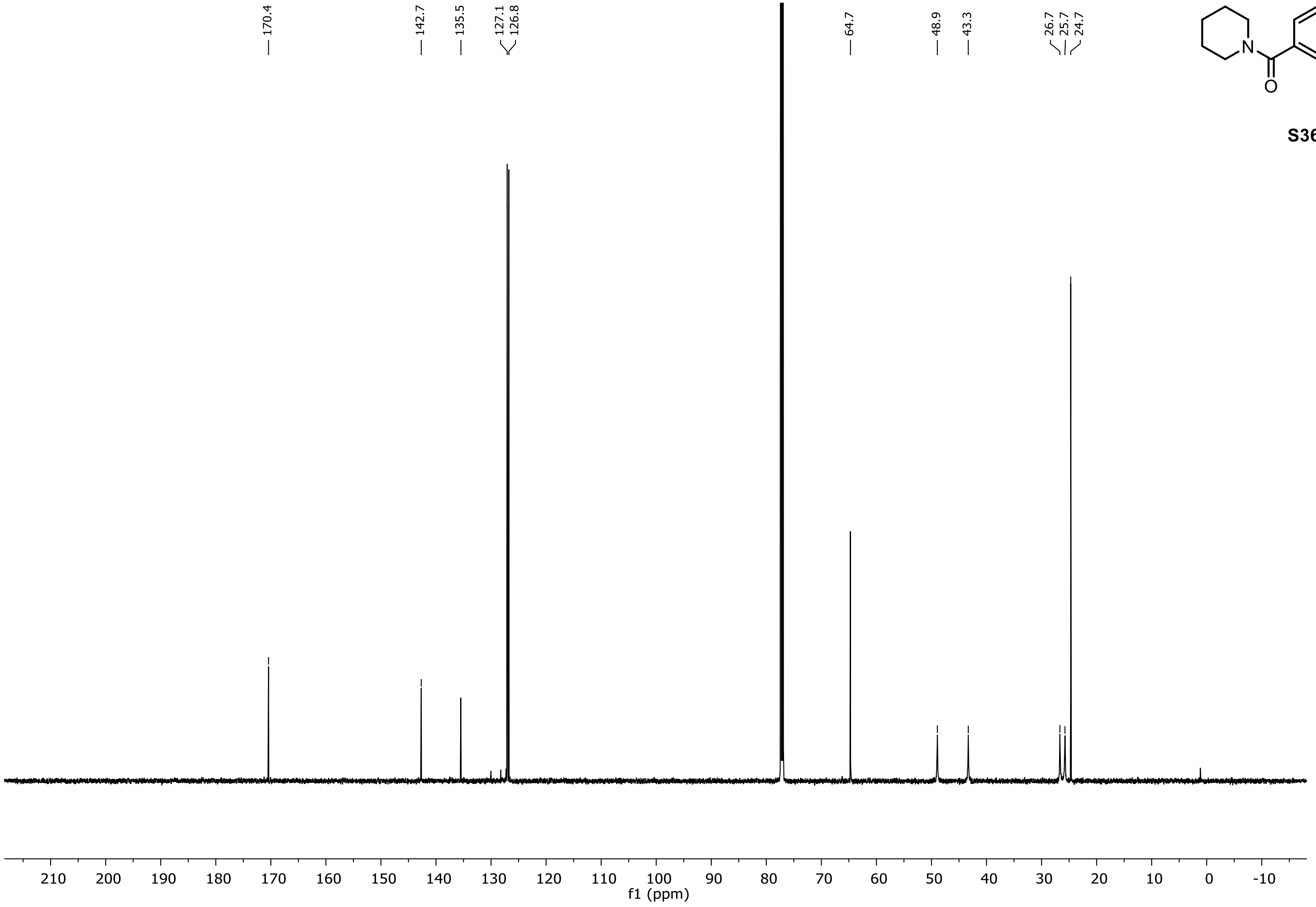
170.4
142.7
135.5
127.1
126.8
64.7
48.9
43.3
26.7
25.7
24.7
210 200 190 180 170 160 150 140 130 120 110 100 90 80 70 60 50 40 30 20 10 0 -10
f1 (ppm)

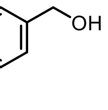
N
O
OH

S36

^1H, ^1H COSY

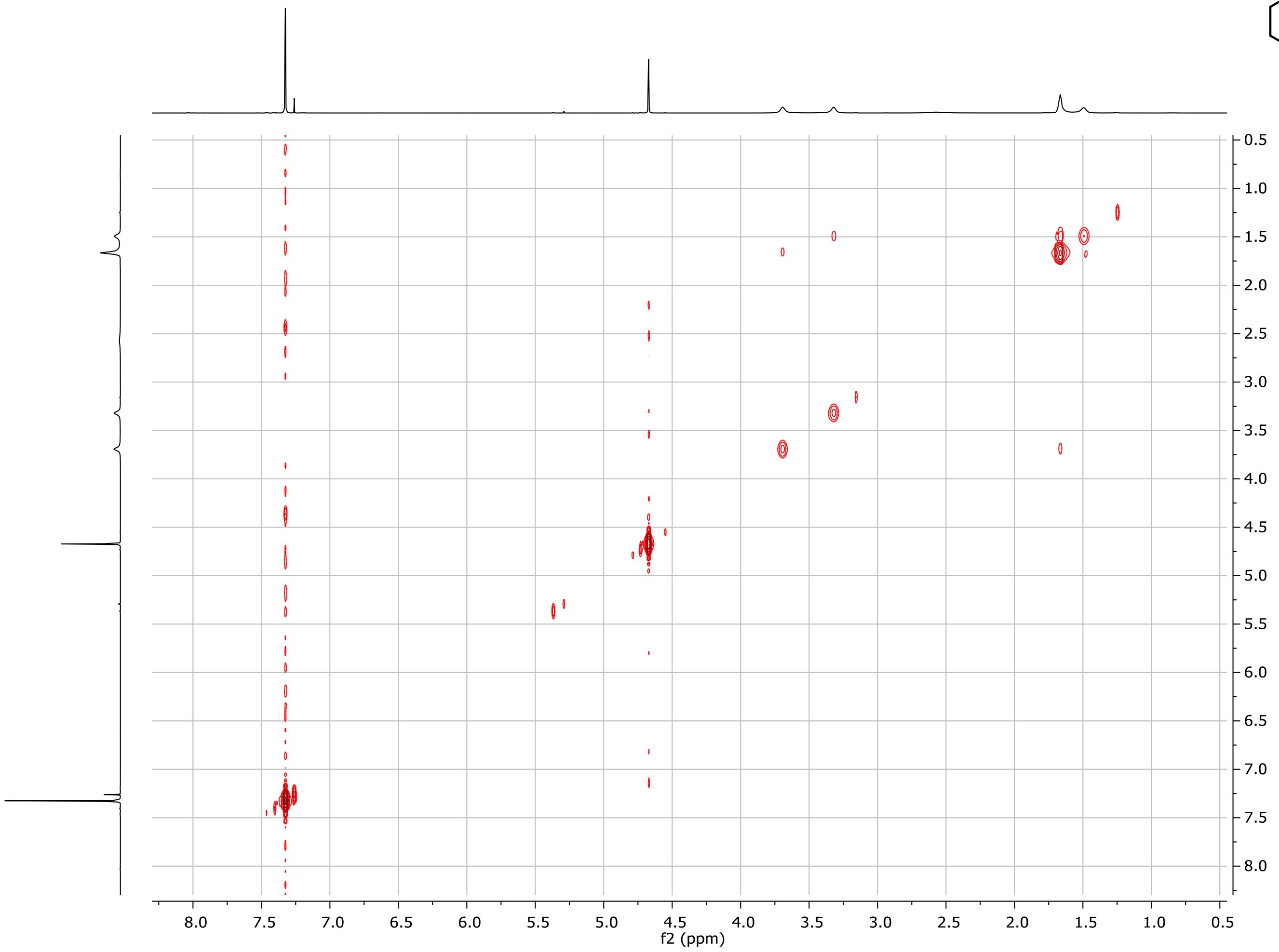

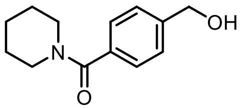

S36

^1H, ^{13}C HMBC

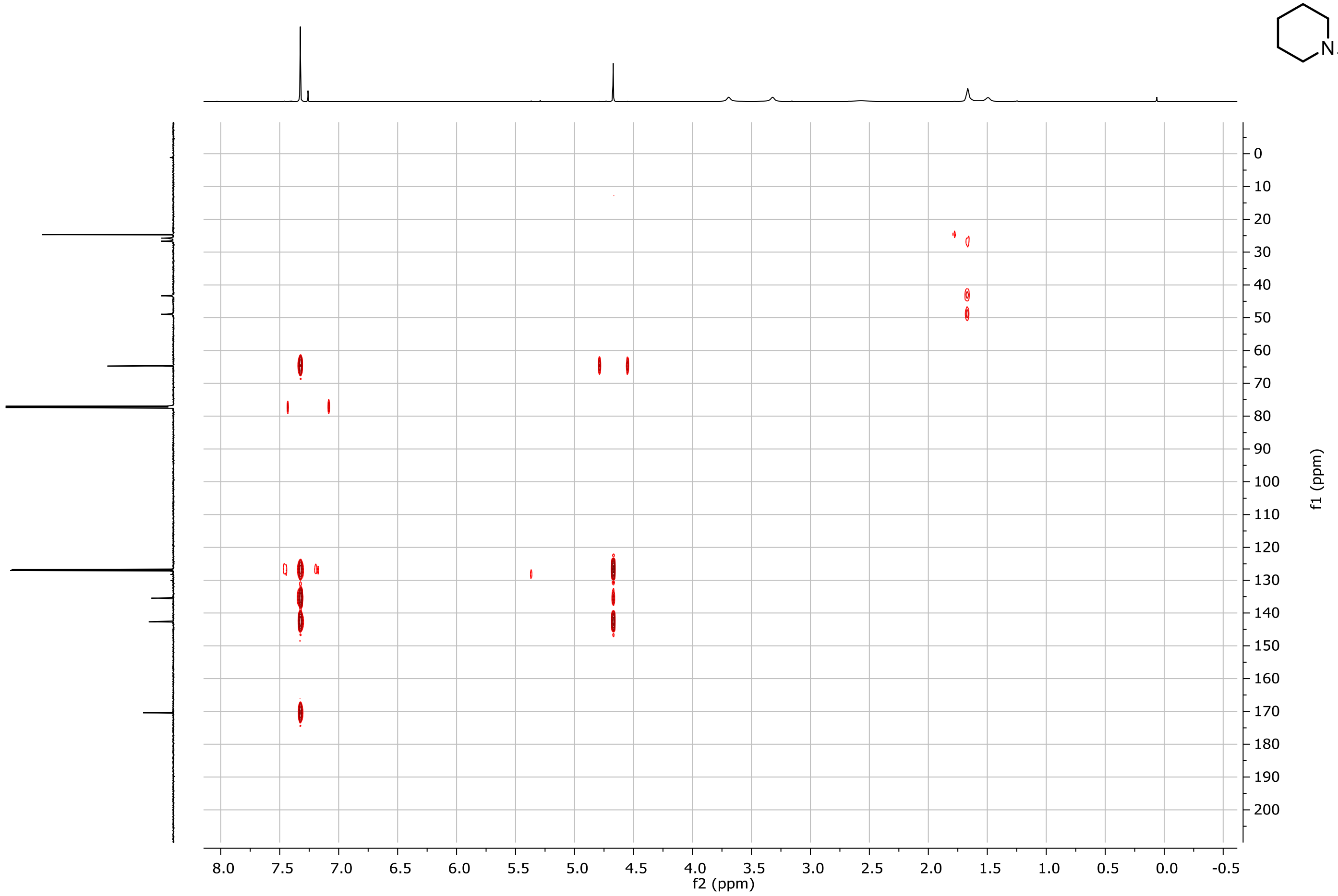

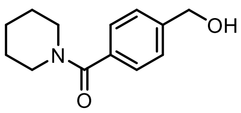

S36

^{1}H, ^{13}C HSQC

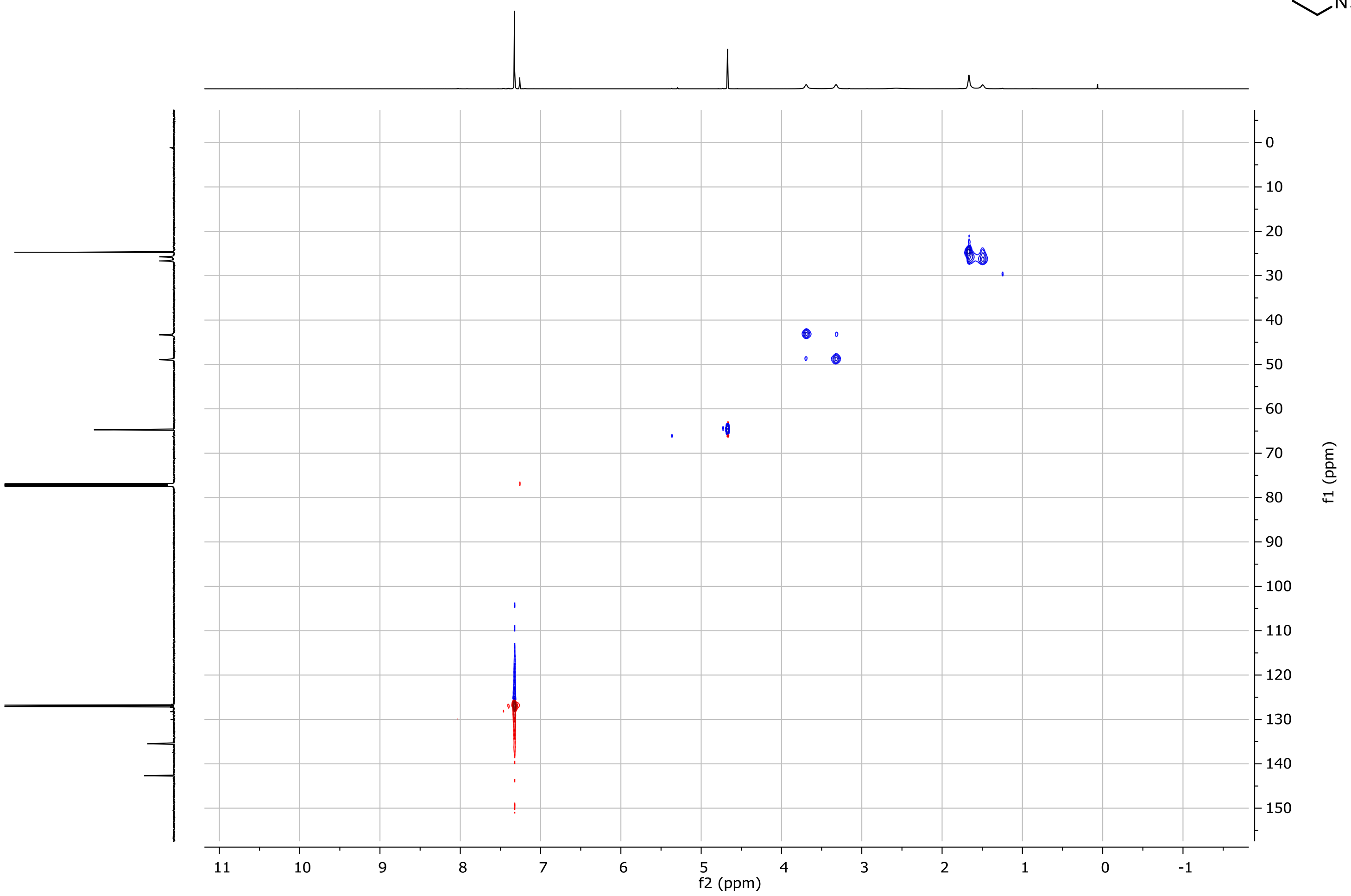

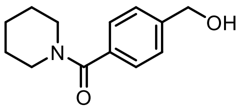

S36

HRMS

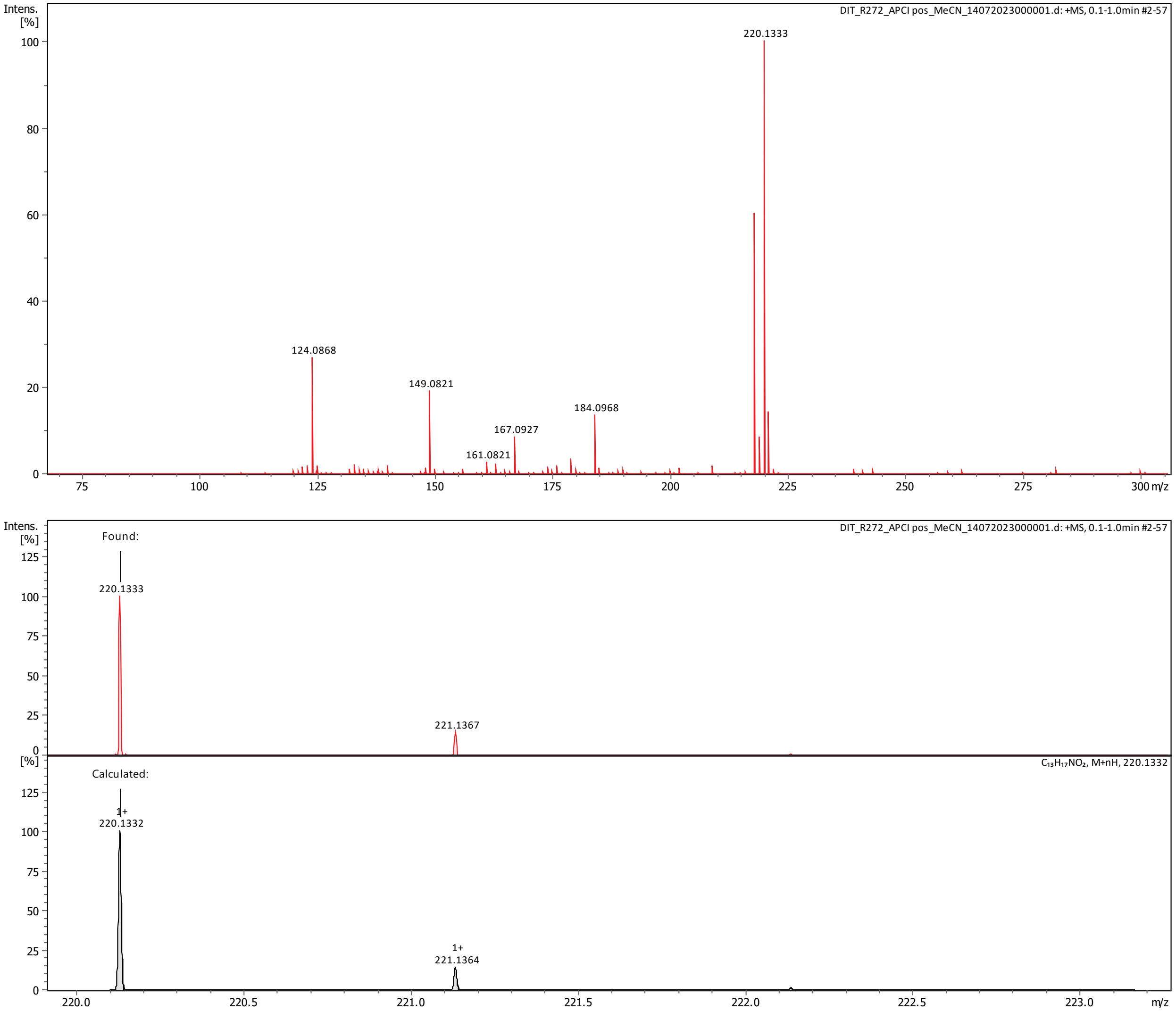

Intens. [%]
DIT_R272_APCI pos_MeCN_14072023000001.d: +MS, 0.1-1.0min #2-57
220.1333
124.0868
149.0821
184.0968
167.0927
161.0821
m/z
Found:
220.1333
221.1367
Calculated:
C₁₃H₁₇NO₂, M+nH, 220.1332
1+
220.1332
1+
221.1364

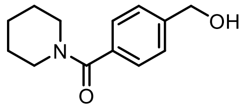

S36

IR

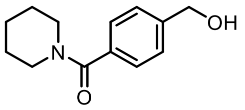

N
O
OH

S36

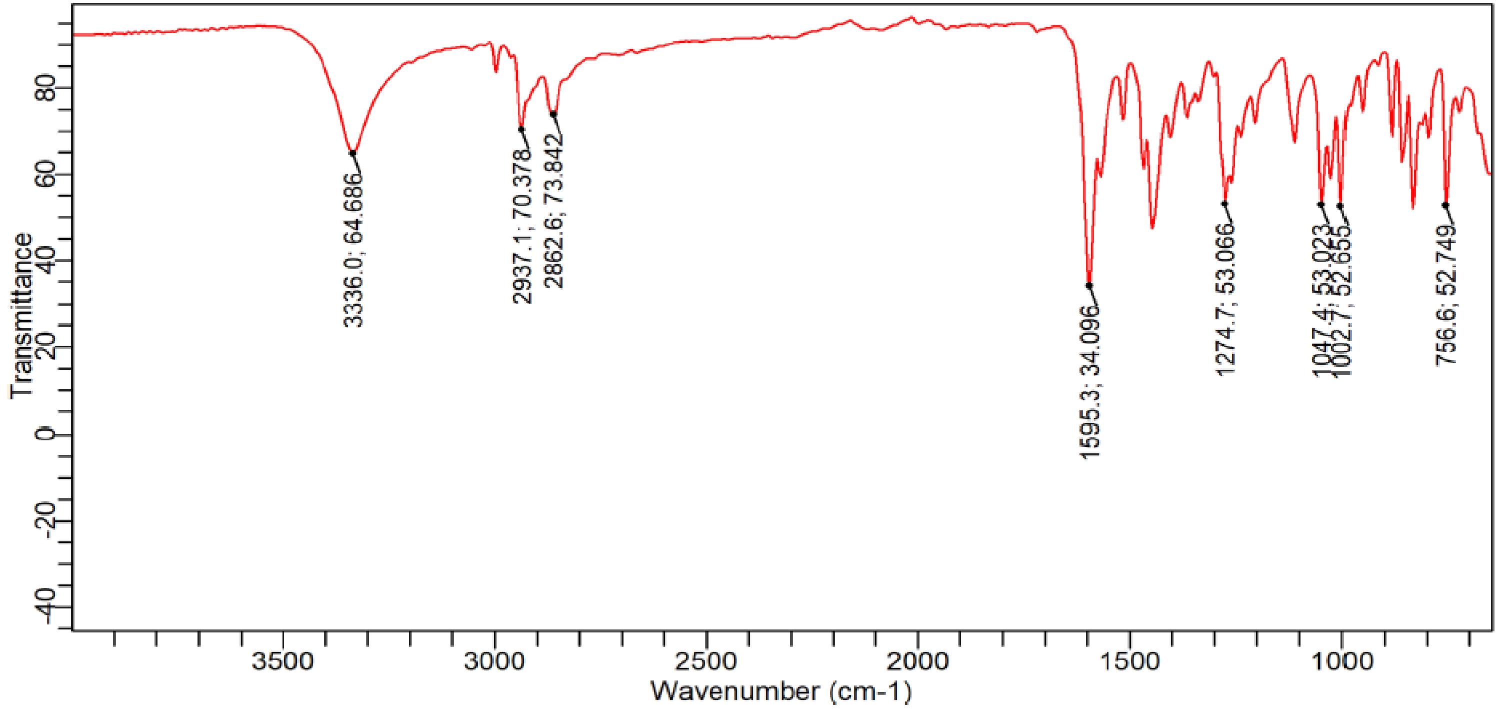

Transmittance
Wavenumber (cm-1)
3336.0; 64.686
2937.1; 70.378
2862.6; 73.842
1595.3; 34.096
1274.7; 53.066
1047.4; 53.023
1002.7; 52.655
756.6; 52.749

45 [4-(Hydroxymethyl)phenyl]-morpholino-methanone (S37)

^{1}H NMR

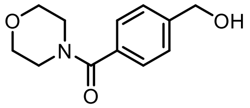

S37

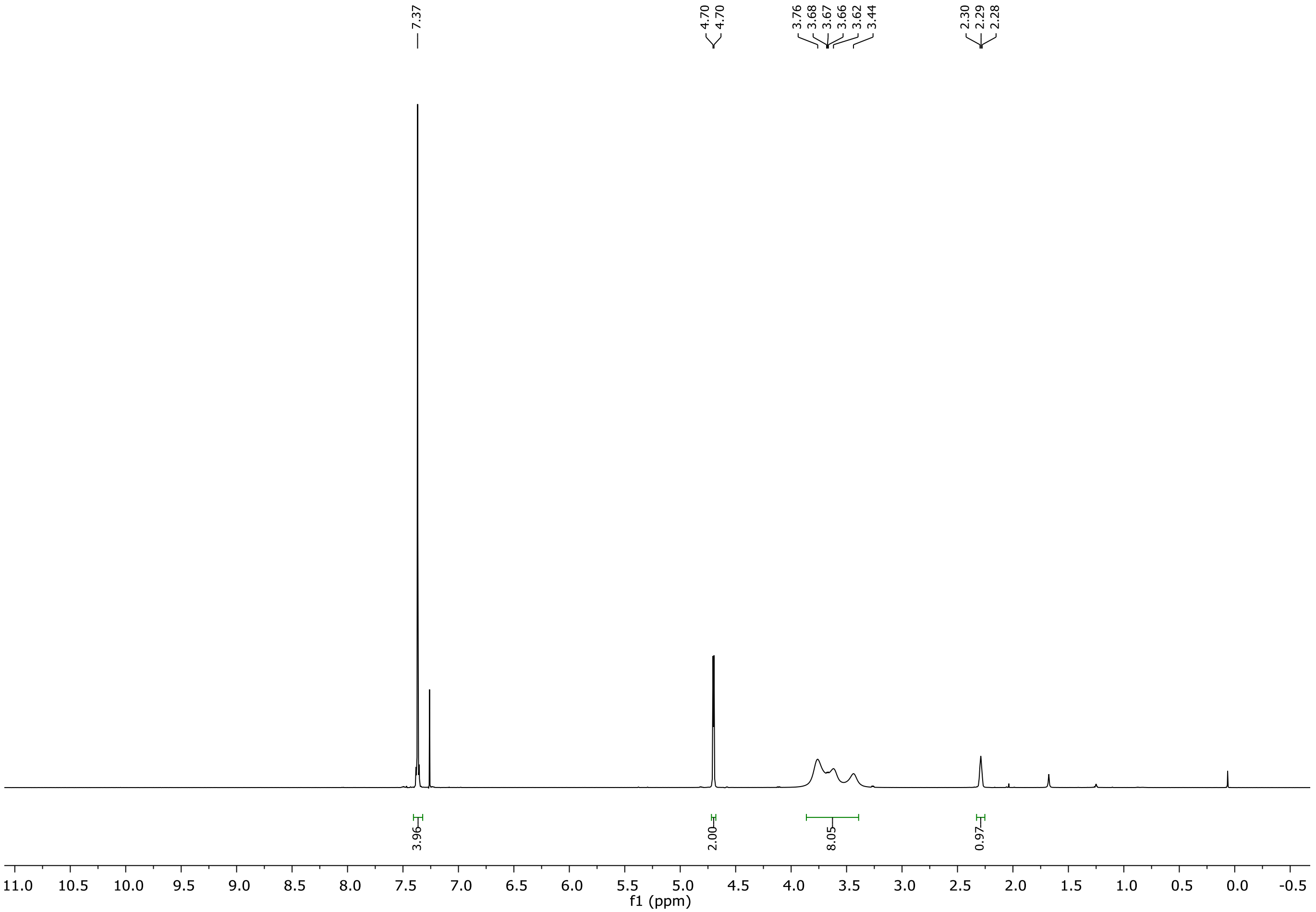

^{13}C NMR

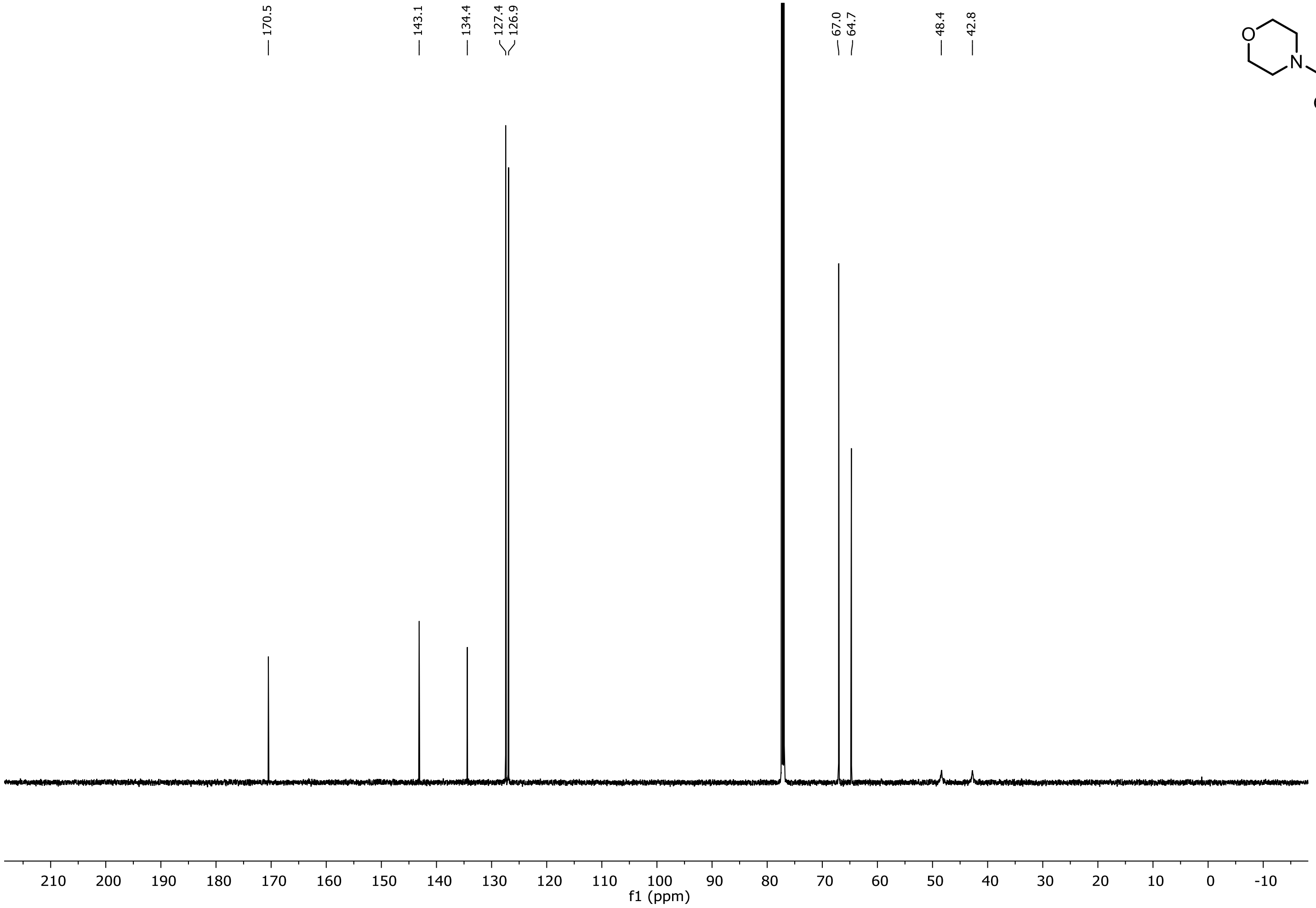

170.5
143.1
134.4
127.4
126.9
67.0
64.7
48.4
42.8
210 200 190 180 170 160 150 140 130 120 110 100 90 80 70 60 50 40 30 20 10 0 -10
f1 (ppm)

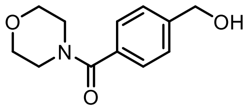

O
N
O
OH

S37

^{1}H, ^{1}H COSY

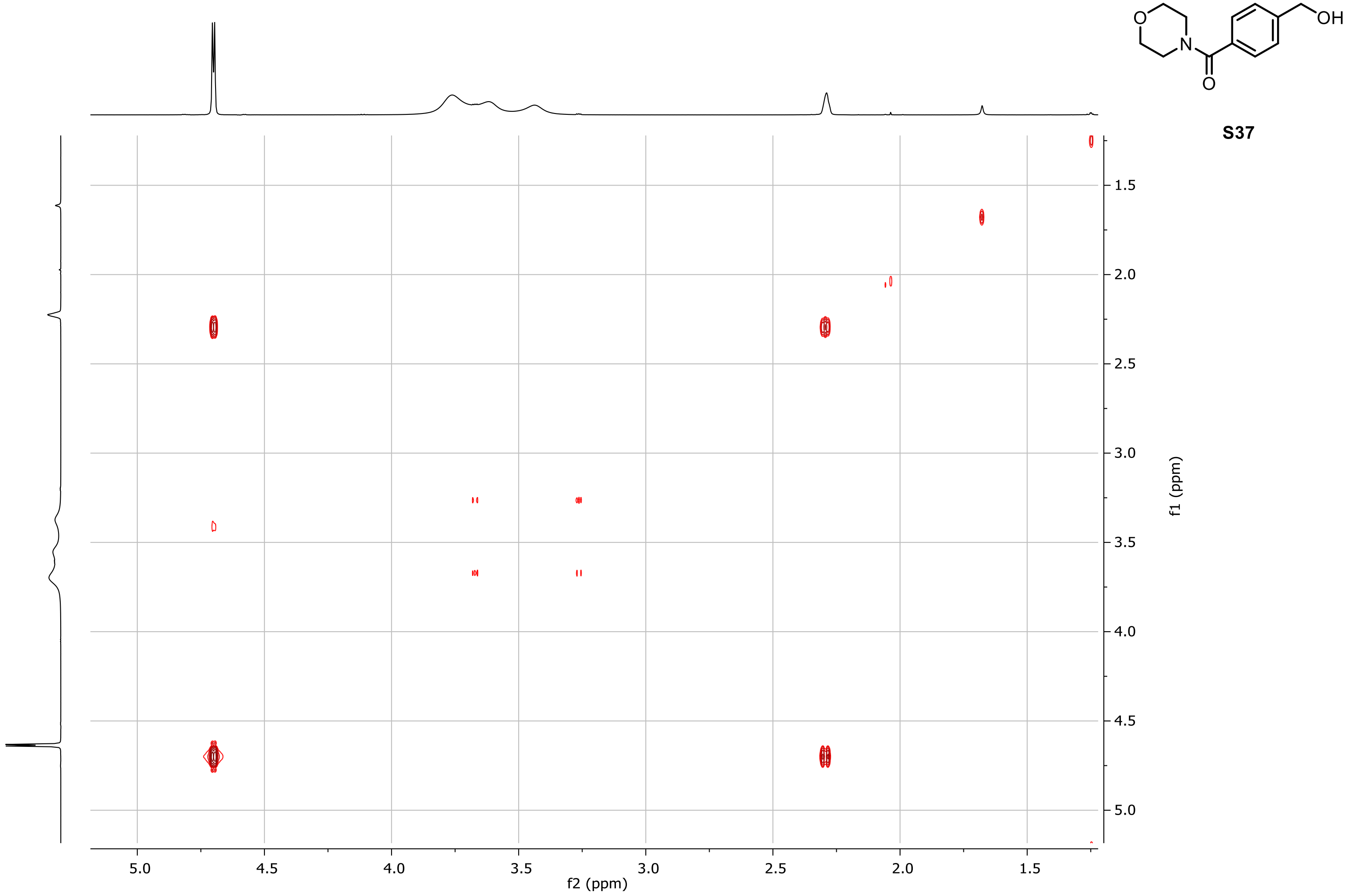

S37

^{1}H, ^{13}C HMBC

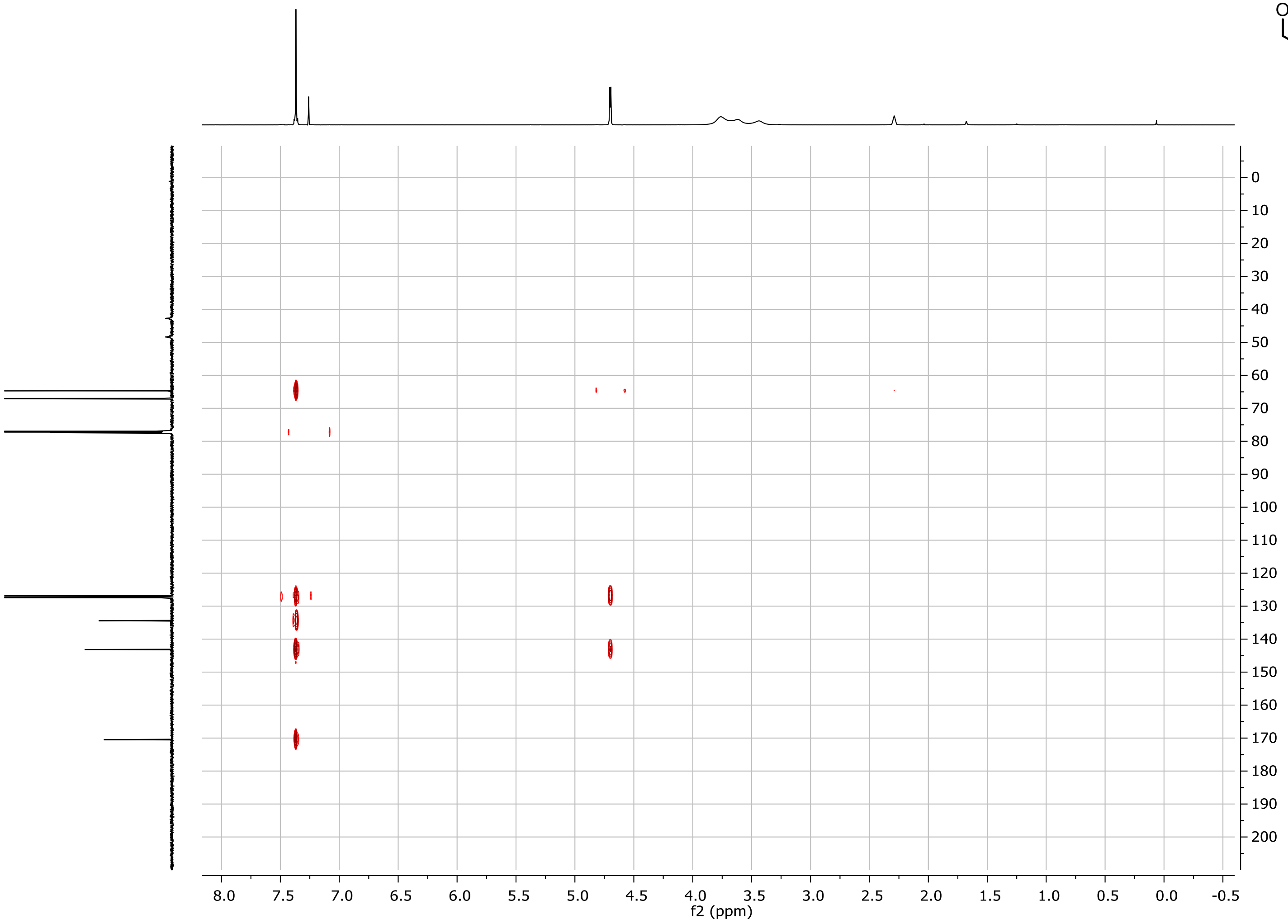

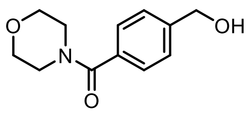

S37

^{1}H, ^{13}C HSQC

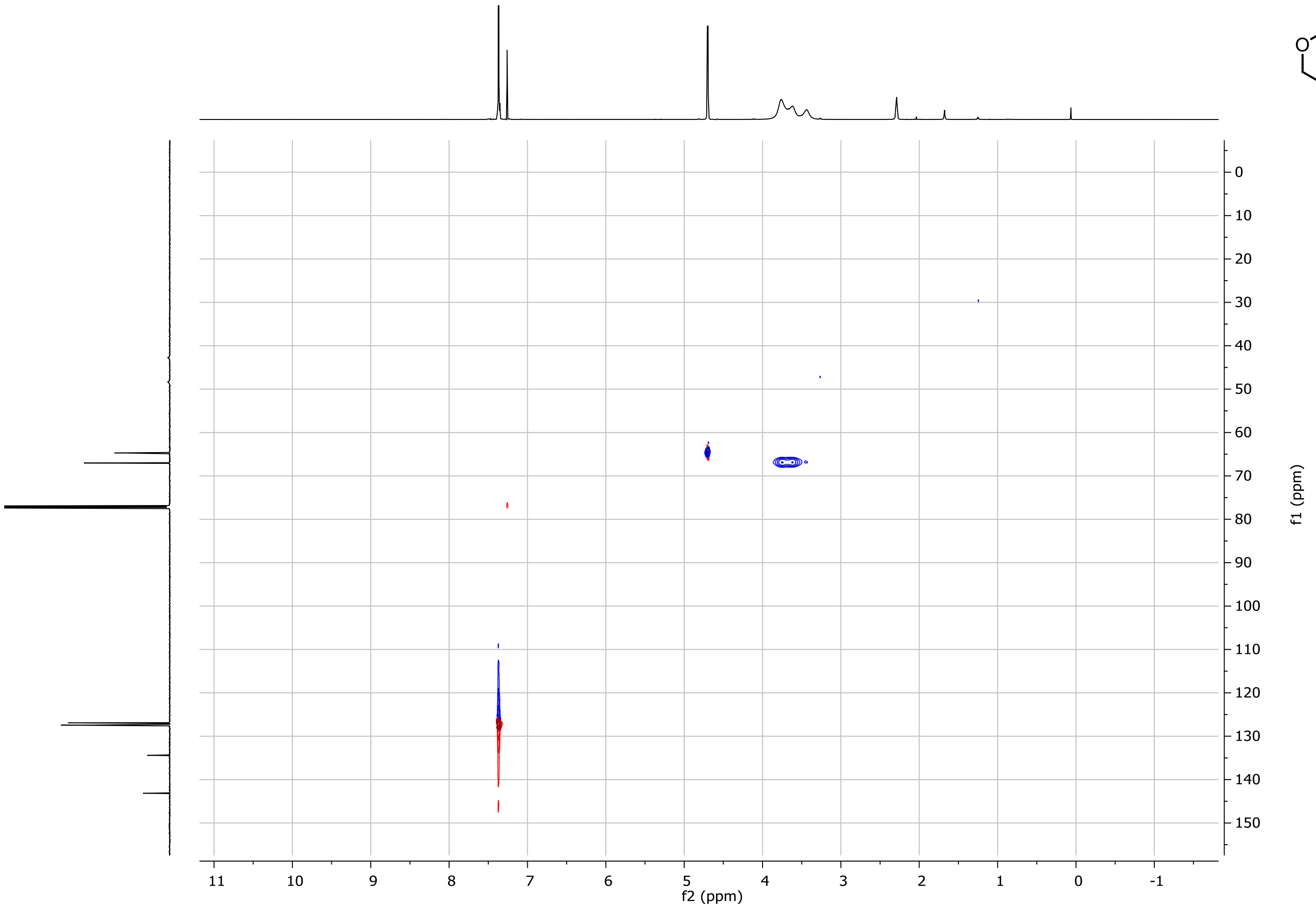

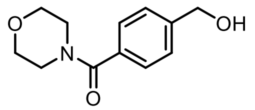

S37

HRMS

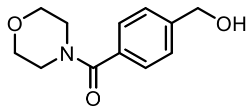

S37

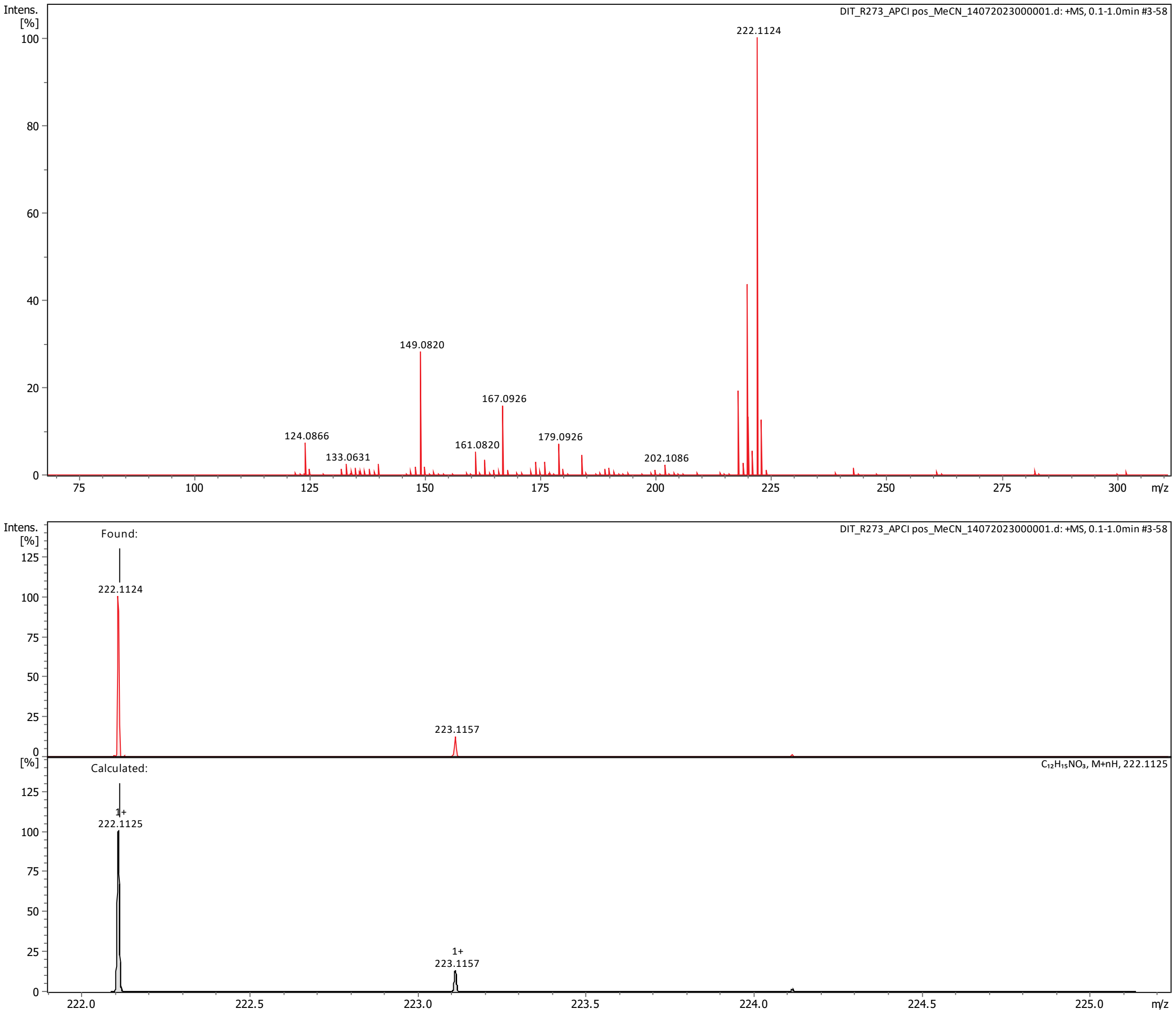

Intens. [%]
DIT_R273_APCI pos_MeCN_14072023000001.d: +MS, 0.1-1.0min #3-58
222.1124
149.0820
167.0926
124.0866
161.0820
179.0926
133.0631
202.1086
m/z
Found:
222.1124
223.1157
Calculated:
$C_{12}H_{15}NO_3$, M+nH, 222.1125
1+
222.1125
1+
223.1157

IR

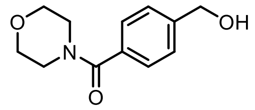

O
N
OH
O

S37

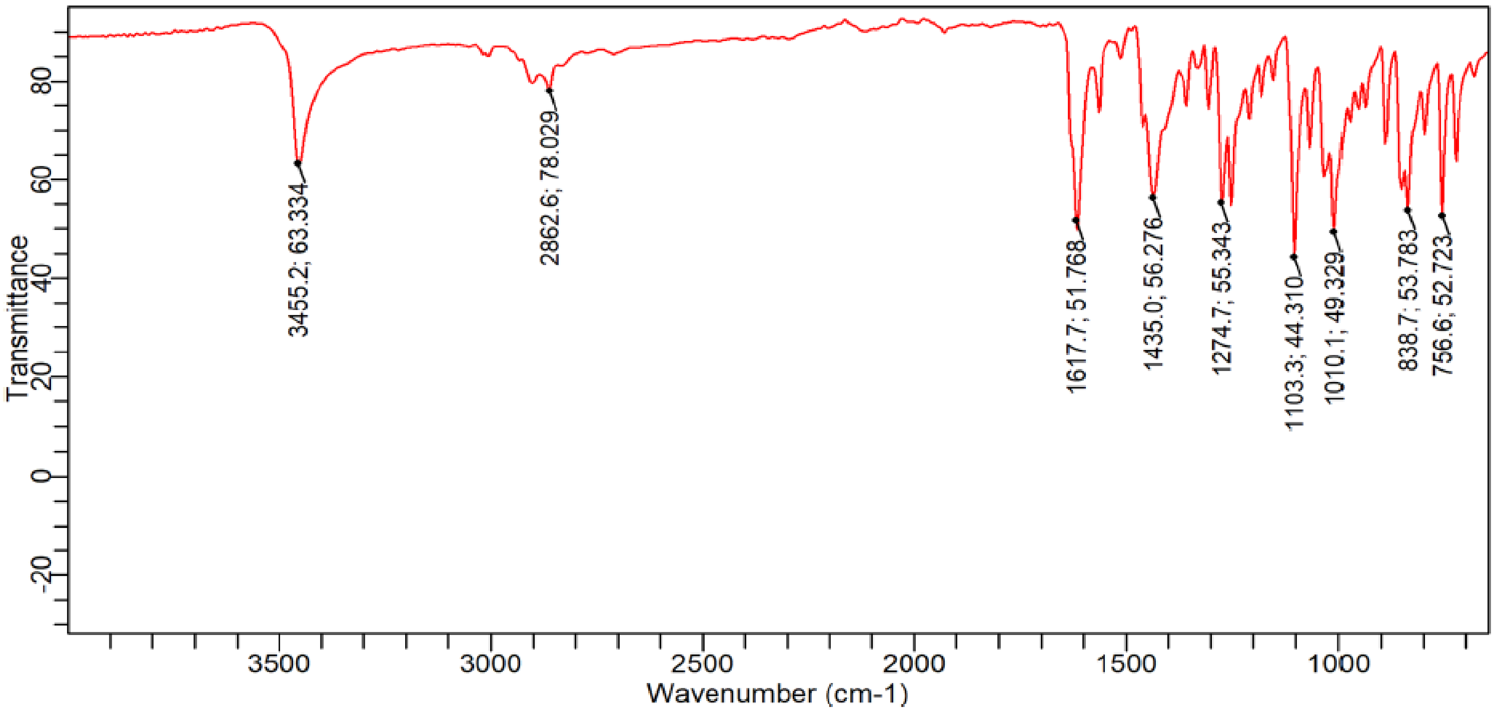

3455.2; 63.334
2862.6; 78.029
1617.7; 51.768
1435.0; 56.276
1274.7; 55.343
1103.3; 44.310
1010.1; 49.329
838.7; 53.783
756.6; 52.723
Transmittance
80
60
40
20
0
-20
3500
3000
2500
2000
1500
1000
Wavenumber (cm-1)

46 2-[2-(Piperidine-1-carbonyl)phenyl]acetic acid (S93)

^{1}H NMR

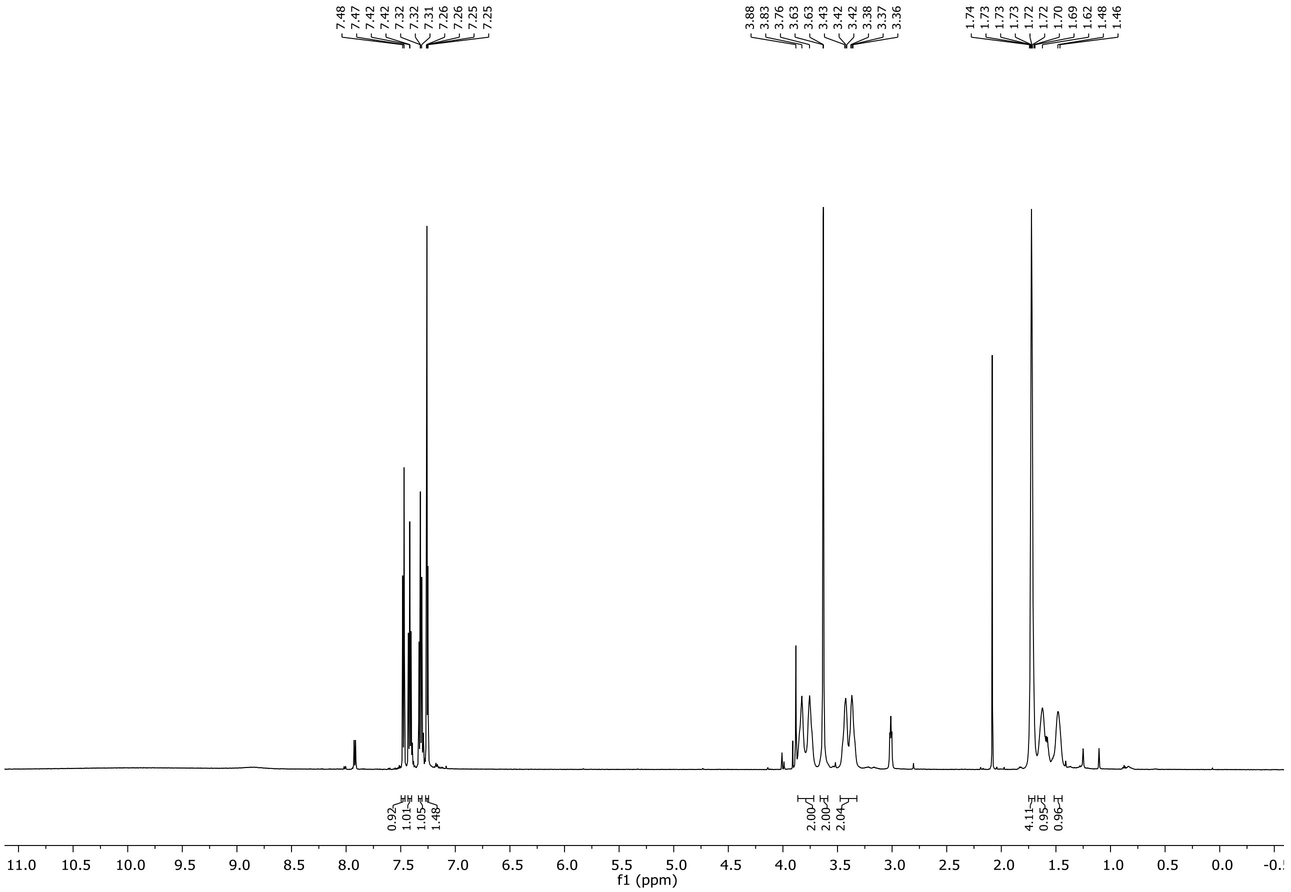

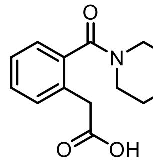

S93

^{13}C NMR

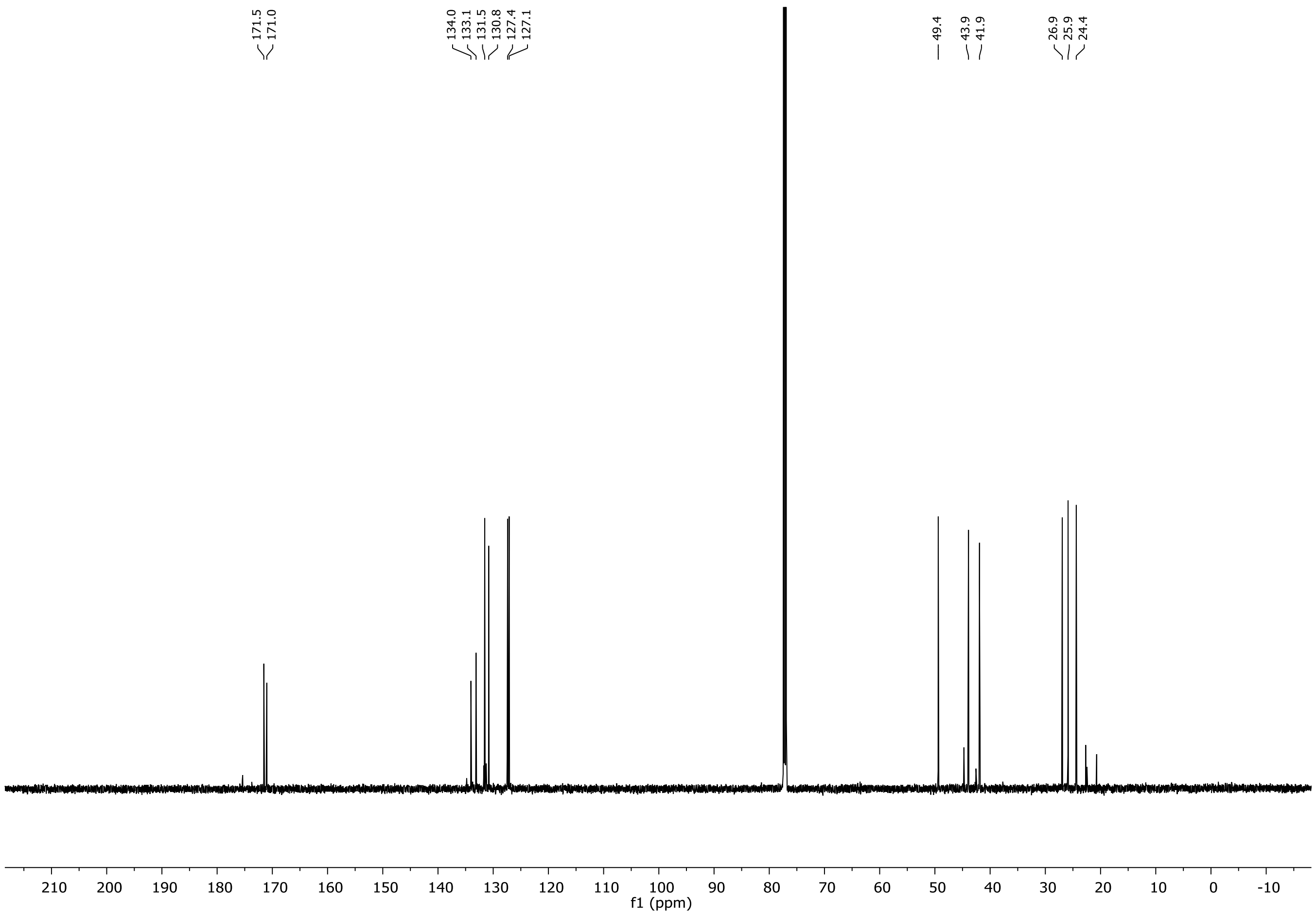

171.5
171.0
134.0
133.1
131.5
130.8
127.4
127.1
49.4
43.9
41.9
26.9
25.9
24.4
210
200
190
180
170
160
150
140
130
120
110
100
90
80
70
60
50
40
30
20
10
0
-10
f1 (ppm)

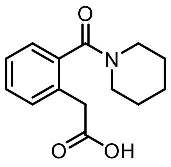

O
N
O
OH

S93

^1H, ^1H COSY

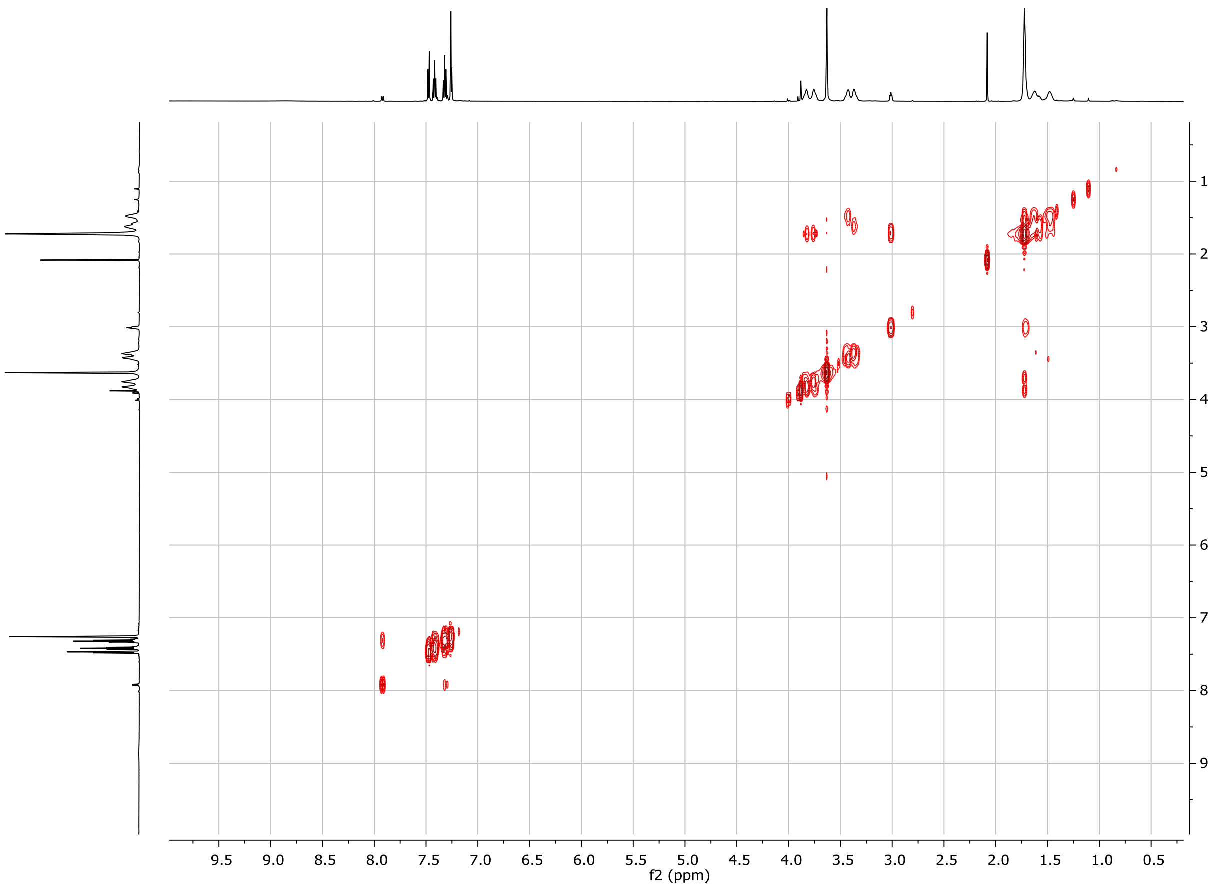

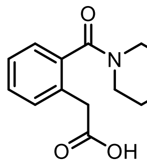

S93

^1H, ^{13}C HMBC

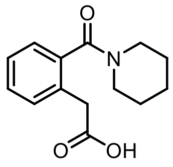

S93

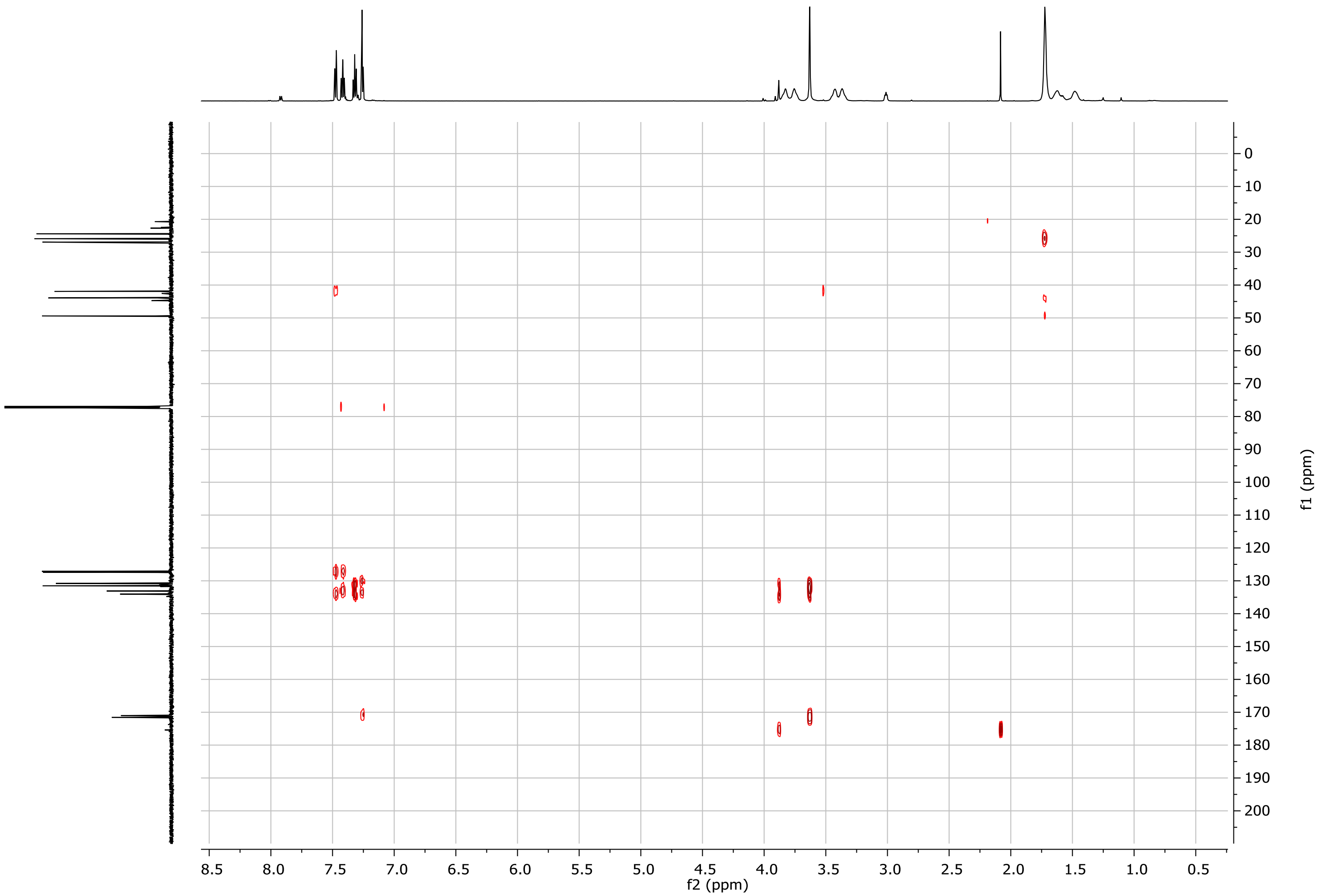

^{1}H, ^{13}C HSQC

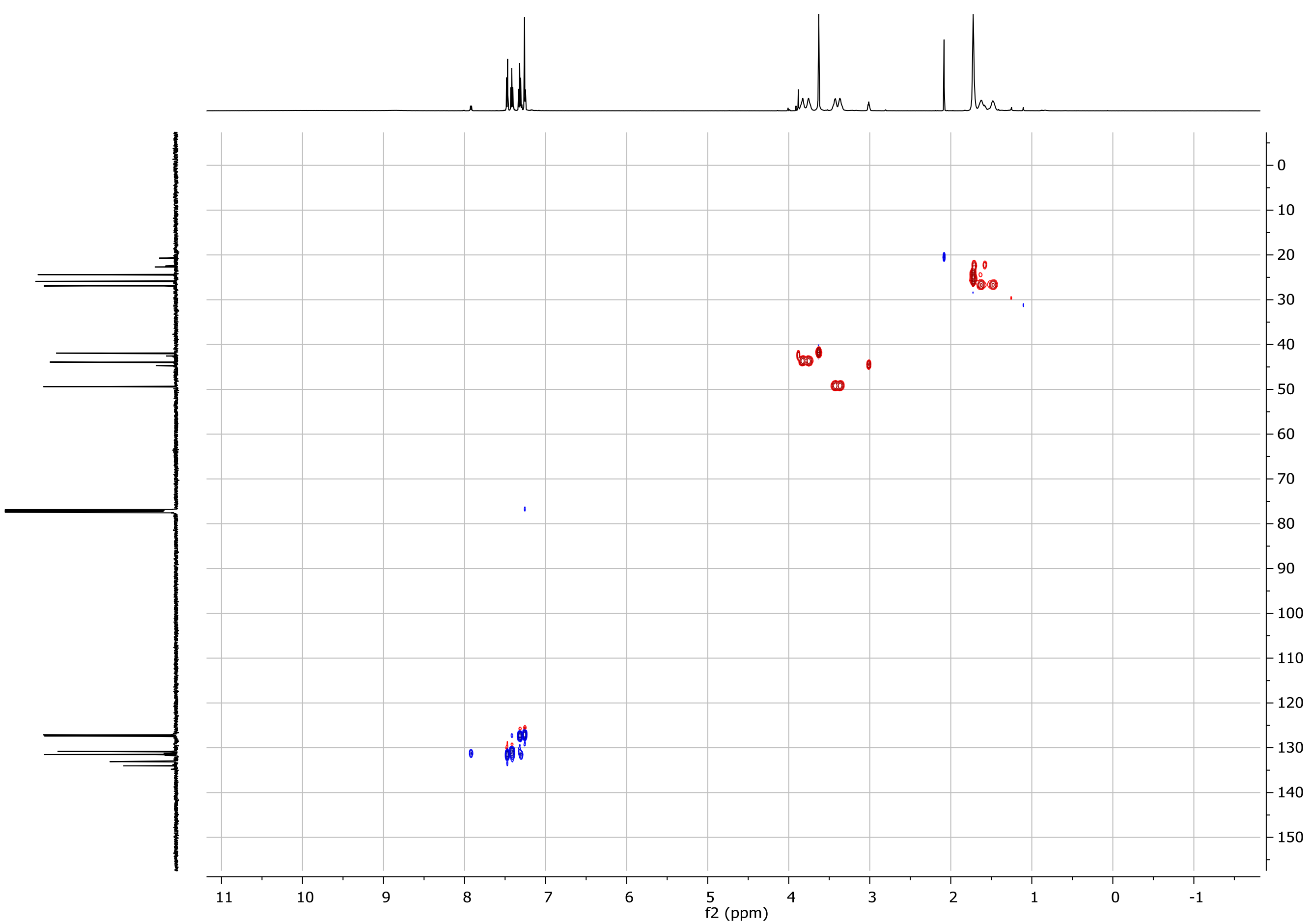

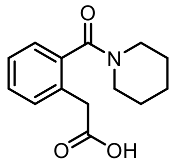

S93

HRMS

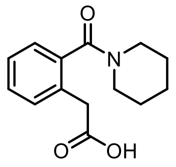

S93

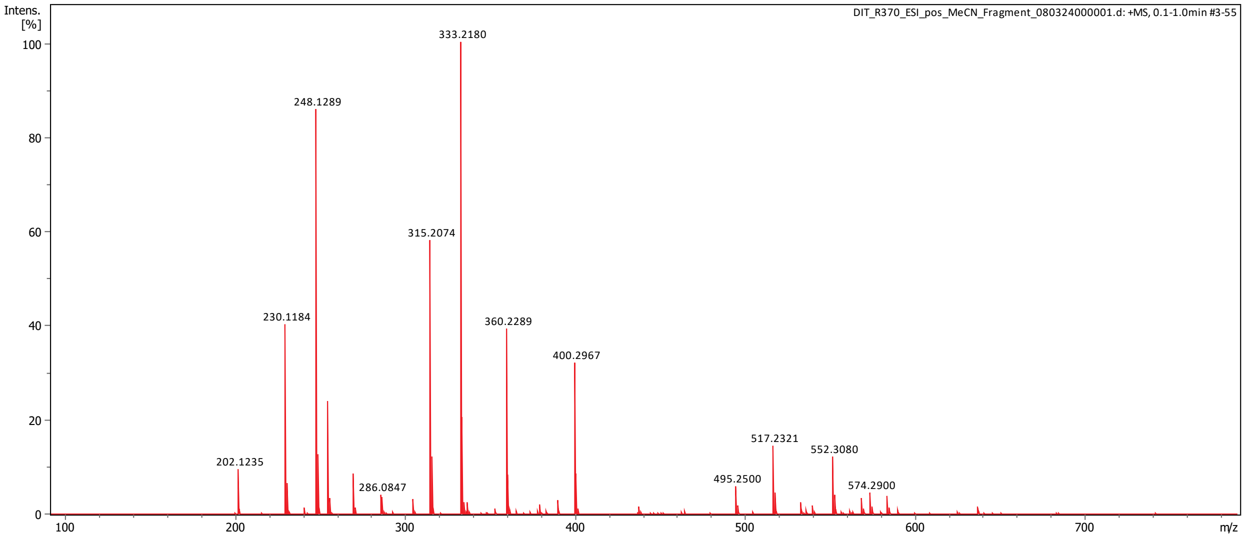
Intens. [%]
DIT_R370_ESI_pos_MeCN_Fragment_080324000001.d: +MS, 0.1-1.0min #3-55
333.2180
248.1289
315.2074
230.1184
360.2289
400.2967
202.1235
286.0847
495.2500
517.2321
552.3080
574.2900
m/z

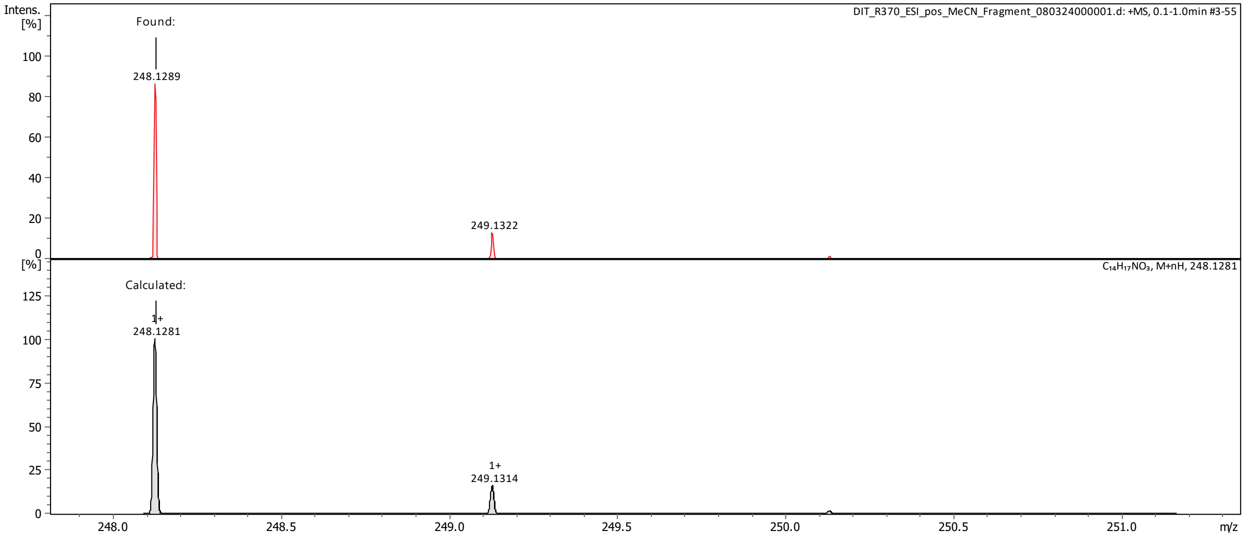
Intens. [%]
DIT_R370_ESI_pos_MeCN_Fragment_080324000001.d: +MS, 0.1-1.0min #3-55
Found:
248.1289
249.1322
$C_{14}H_{17}NO_3$, M+nH, 248.1281
Calculated:
1+
248.1281
1+
249.1314
m/z

IR

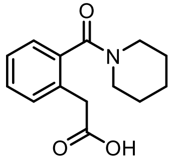
O
N
O
OH

S93

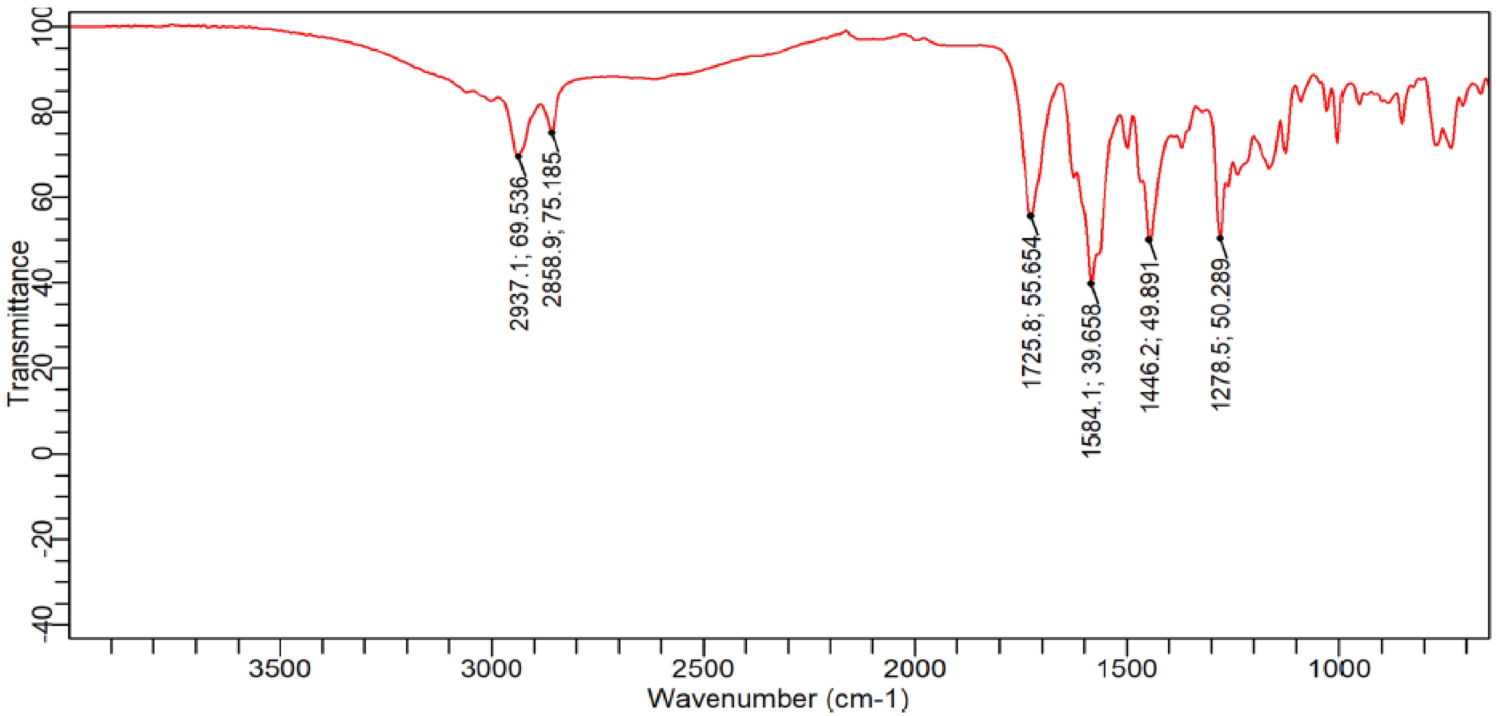
Transmittance
Wavenumber (cm-1)
2937.1; 69.536
2858.9; 75.185
1725.8; 55.654
1584.1; 39.658
1446.2; 49.891
1278.5; 50.289

47 1-Morpholino-2-[2-(piperidine-1-carbonyl)phenyl]ethanone (S94)

^{1}H NMR

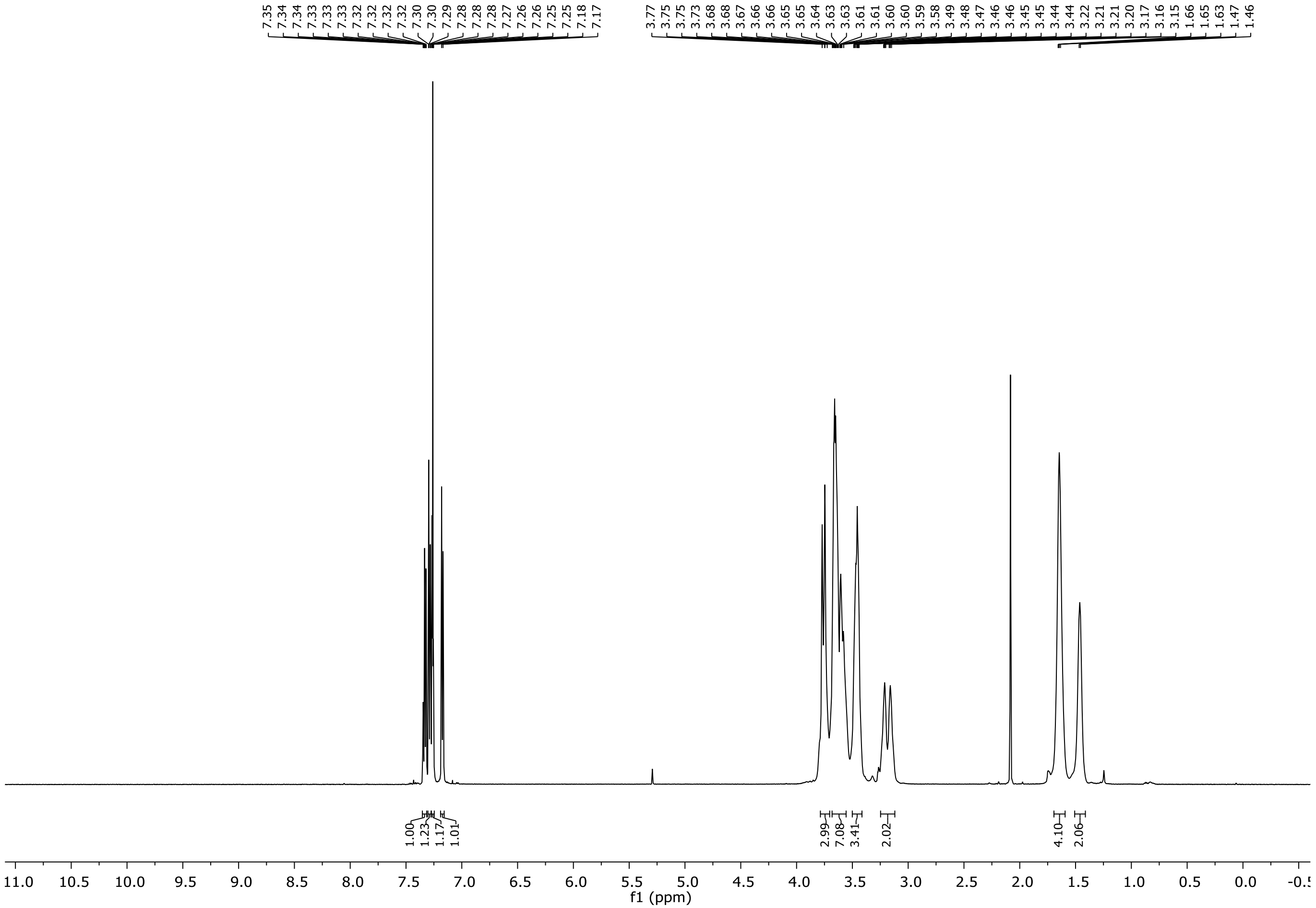

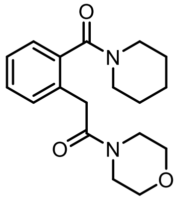

S94

^{13}C NMR

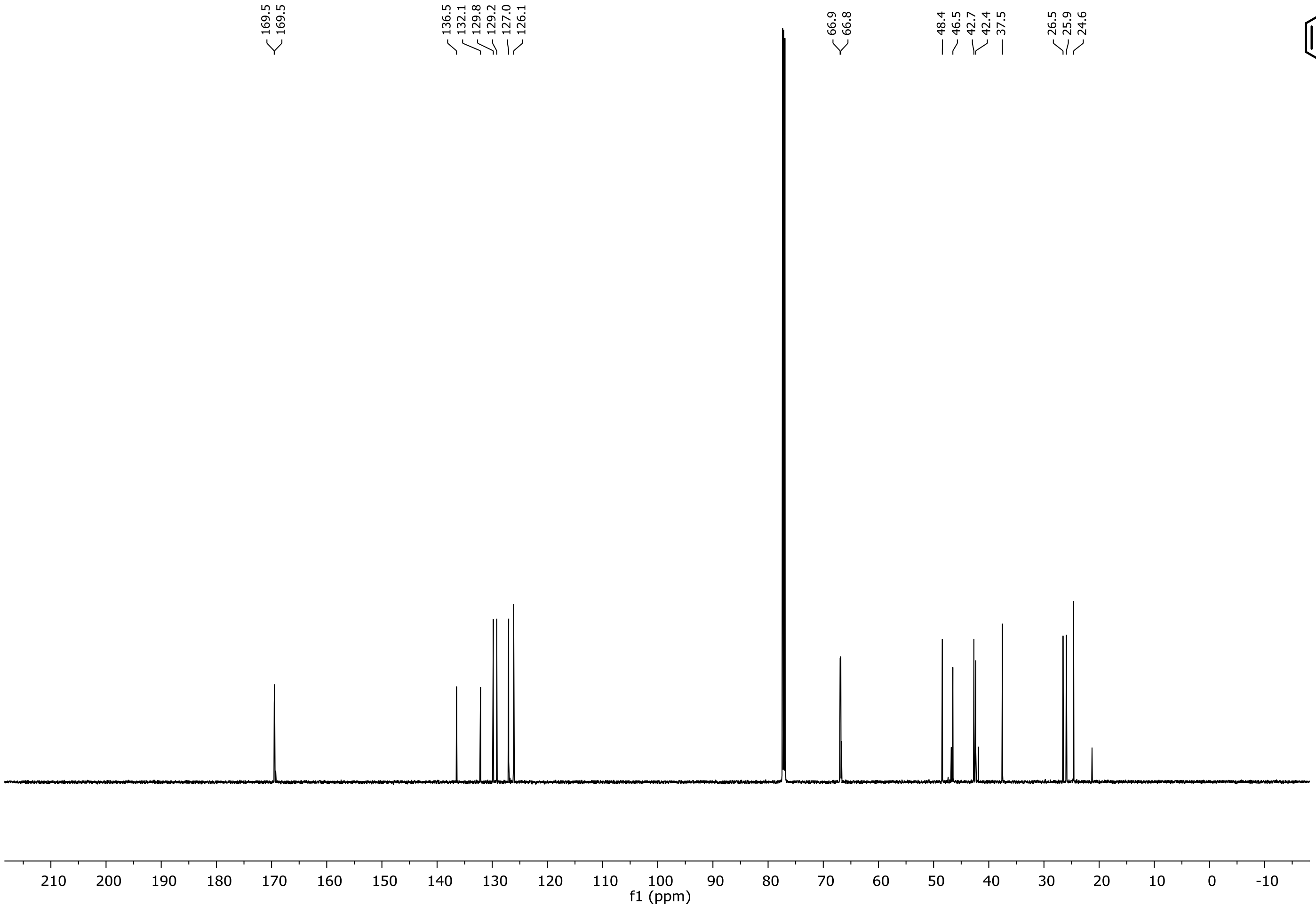
169.5
169.5
136.5
132.1
129.8
129.2
127.0
126.1
66.9
66.8
48.4
46.5
42.7
42.4
37.5
26.5
25.9
24.6
210
200
190
180
170
160
150
140
130
120
110
100
90
80
70
60
50
40
30
20
10
0
-10
f1 (ppm)

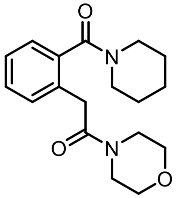
O
N
O
N
O

S94

^{1}H, ^{1}H COSY

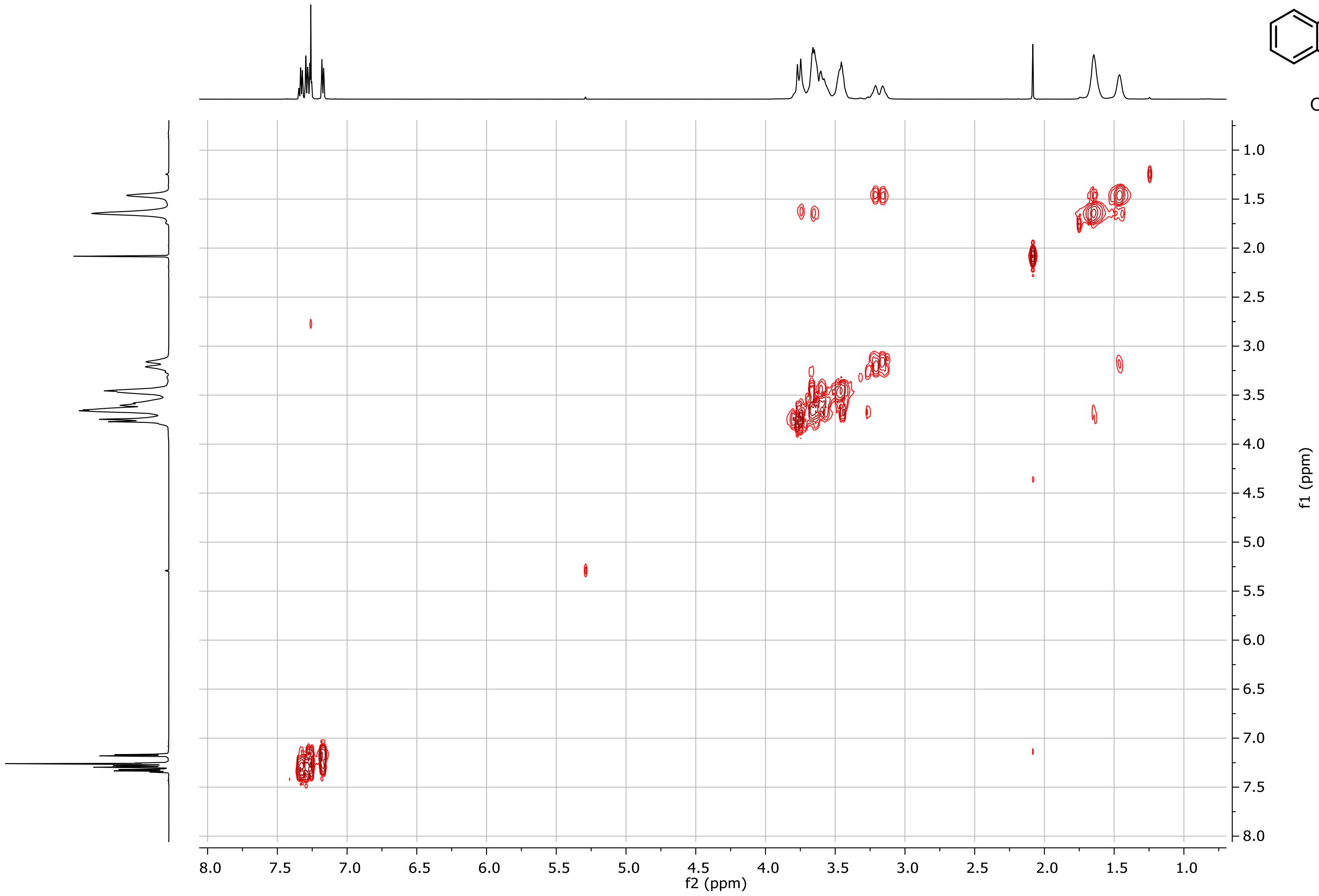

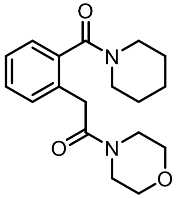

S94

^{1}H, ^{13}C HMBC

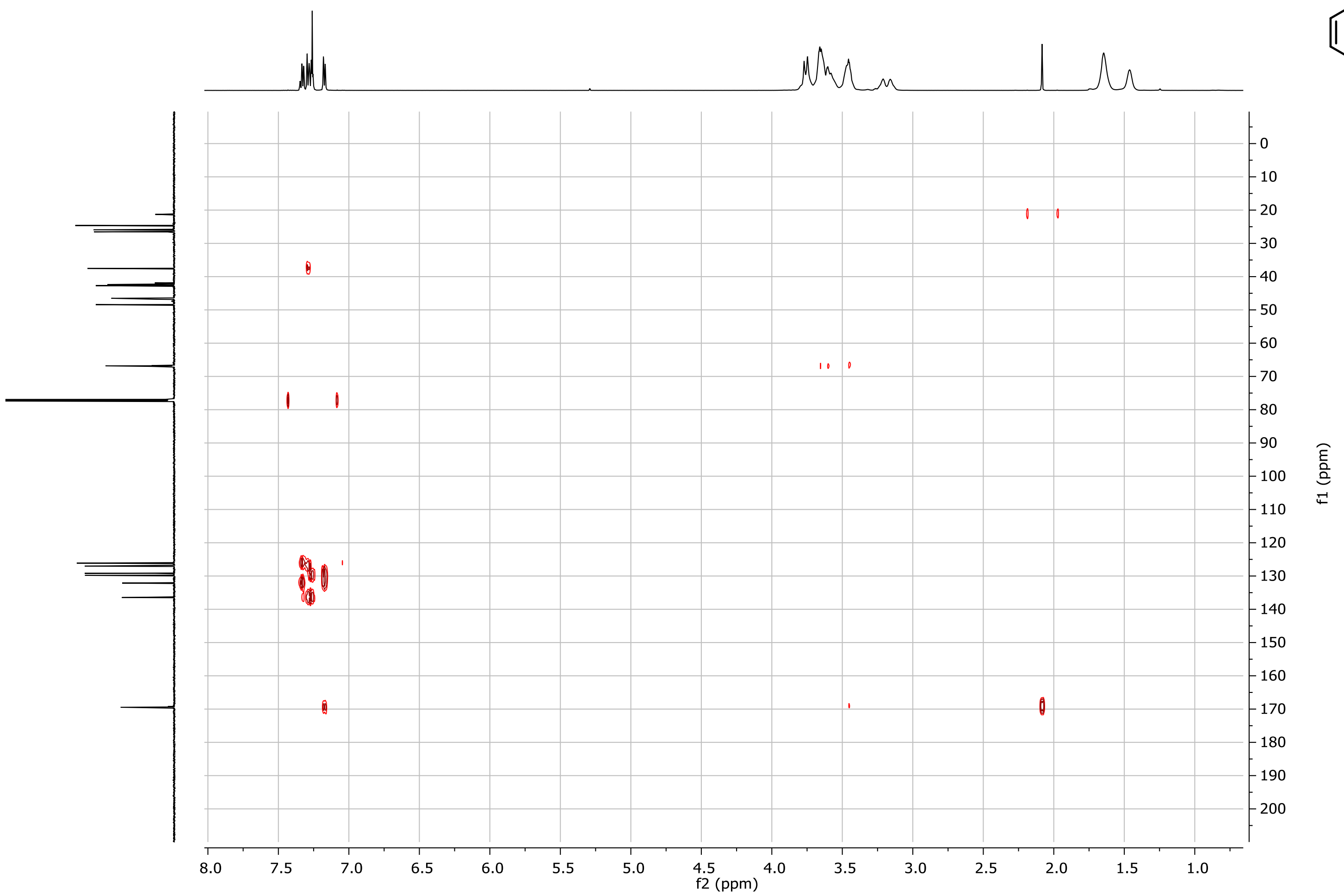

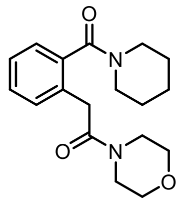

S94

^{1}H, ^{13}C HSQC

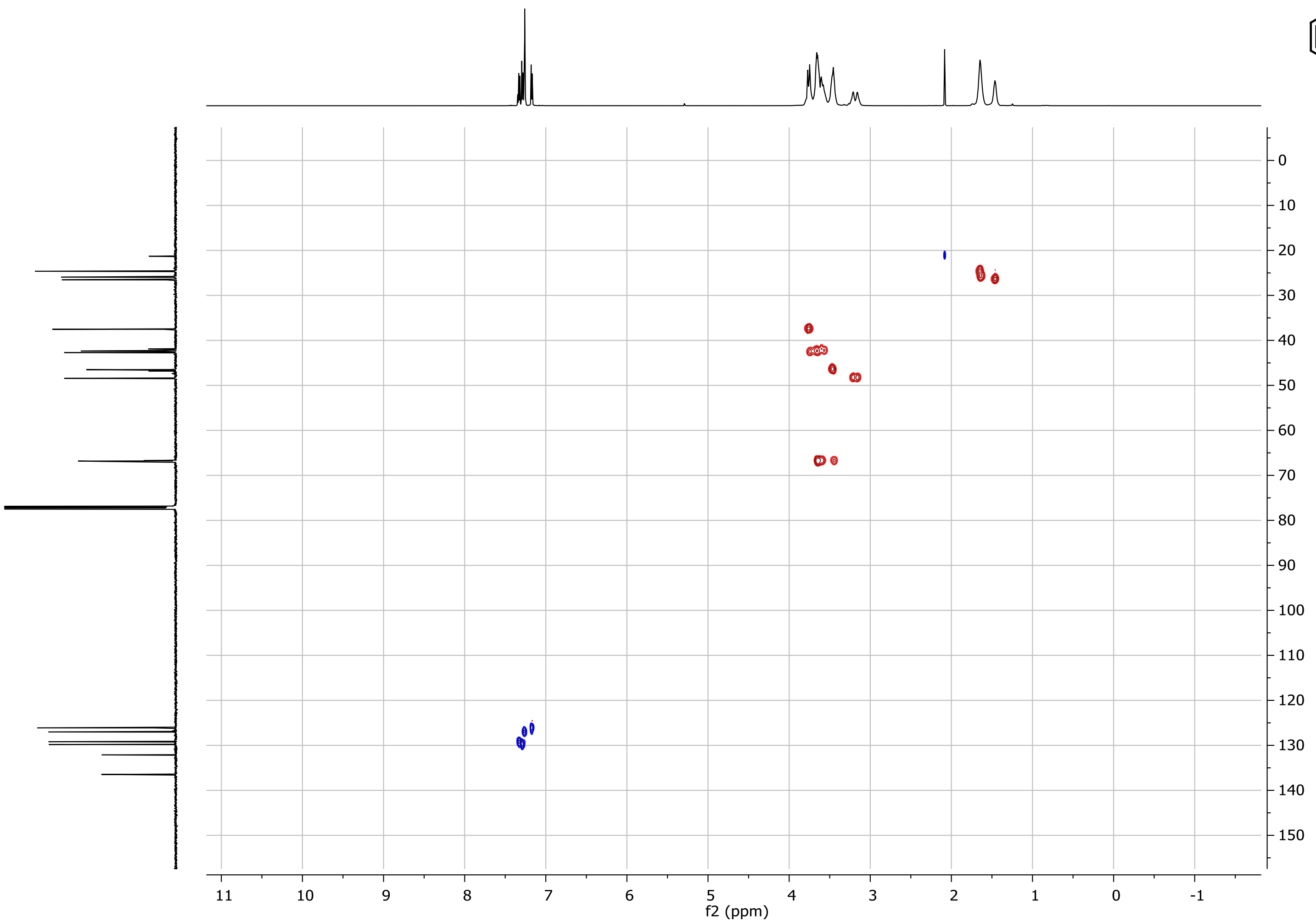

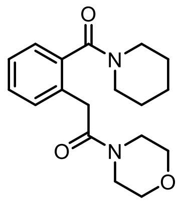

S94

HRMS

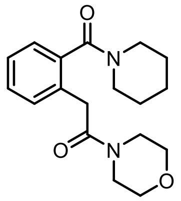

S94

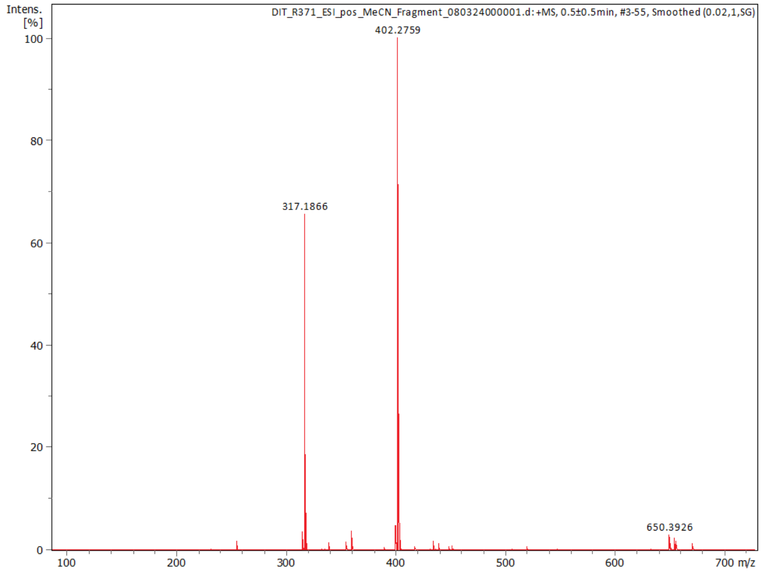

Intens. [%]
DIT_R371_ESI_pos_MeCN_Fragment_080324000001.d: +MS, 0.5±0.5min, #3-55, Smoothed (0.02,1,SG)
402.2759
317.1866
650.3926
m/z

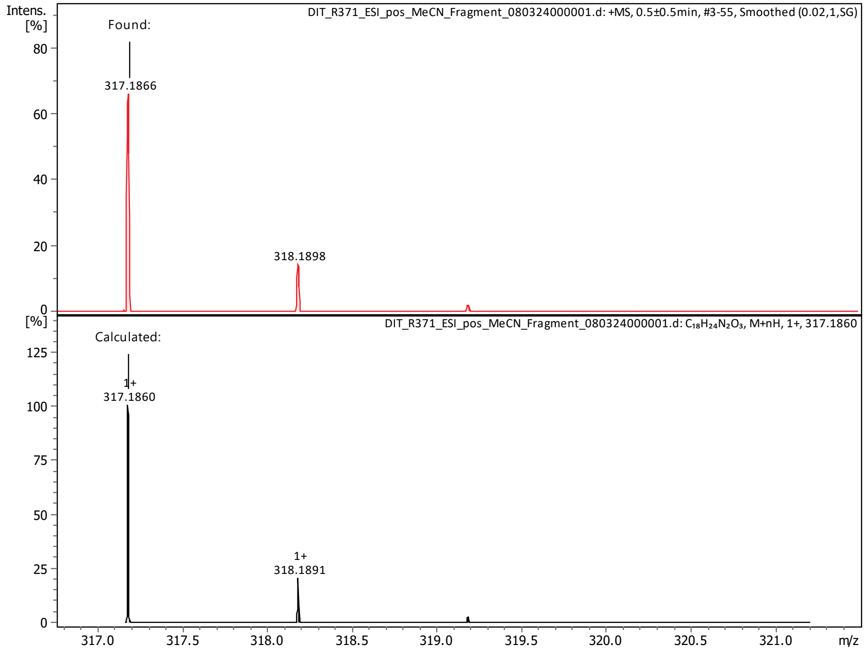

Intens. [%]
DIT_R371_ESI_pos_MeCN_Fragment_080324000001.d: +MS, 0.5±0.5min, #3-55, Smoothed (0.02,1,SG)
Found:
317.1866
318.1898
DIT_R371_ESI_pos_MeCN_Fragment_080324000001.d: C18H24N2O3, M+nH, 1+, 317.1860
Calculated:
1+
317.1860
1+
318.1891
m/z

IR

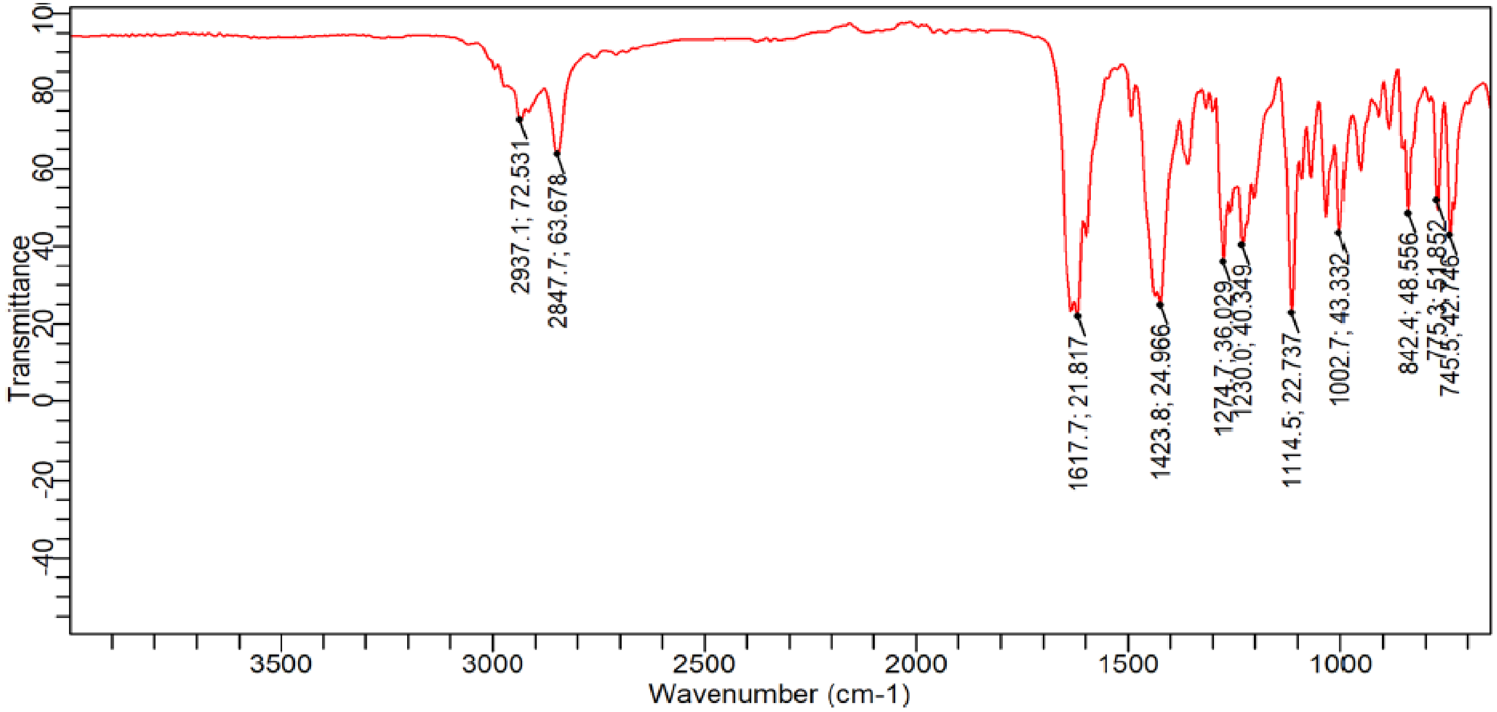

Transmittance
Wavenumber (cm-1)
2937.1; 72.531
2847.7; 63.678
1617.7; 21.817
1423.8; 24.966
1274.7; 36.029
1230.0; 40.349
1114.5; 22.737
1002.7; 43.332
842.4; 48.556
775.3; 51.852
745.5; 42.746

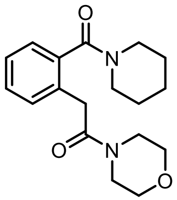

S94

48 1-Morpholino-4-[4-(piperidine-1-carbonyl)phenyl]butan-1-one (S97)

^{1}H NMR

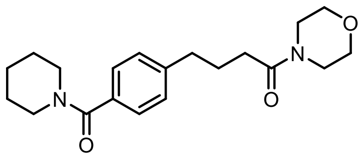

S97

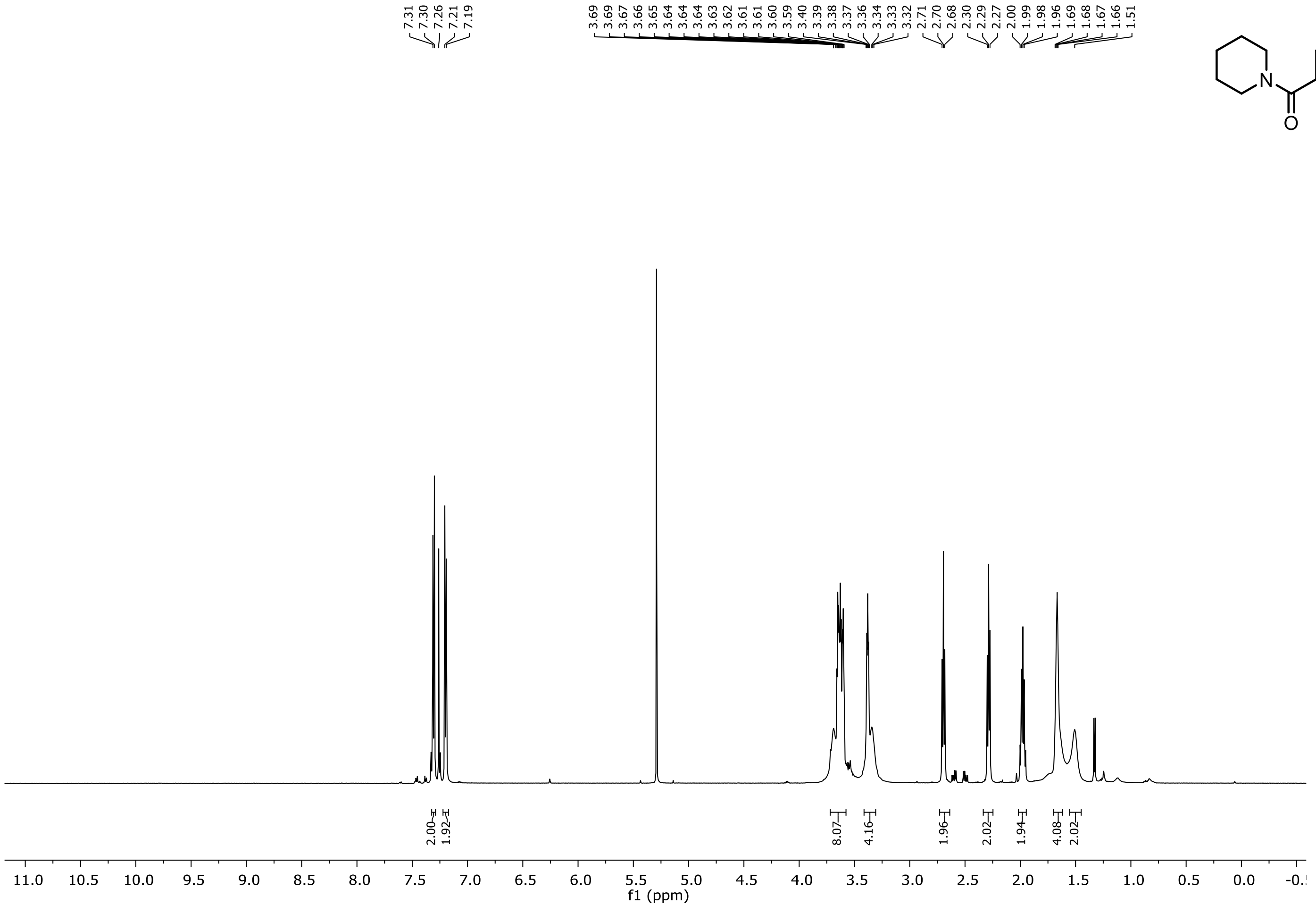

^{13}C NMR

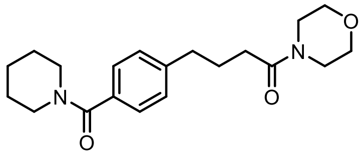

S97

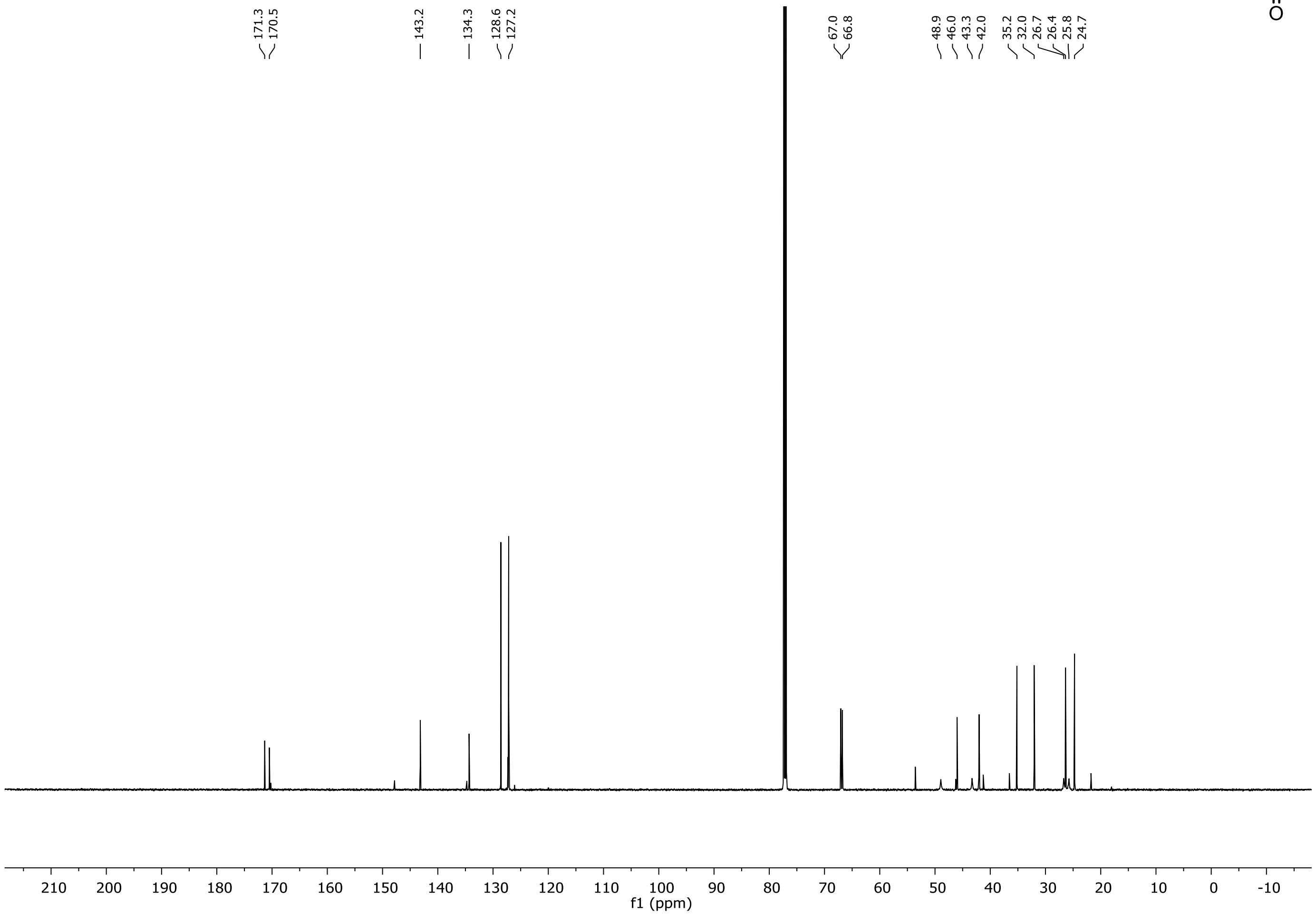
171.3
170.5
143.2
134.3
128.6
127.2
67.0
66.8
48.9
46.0
43.3
42.0
35.2
32.0
26.7
26.4
25.8
24.7
210 200 190 180 170 160 150 140 130 120 110 100 90 80 70 60 50 40 30 20 10 0 -10
f1 (ppm)

^{1}H, ^{1}H COSY

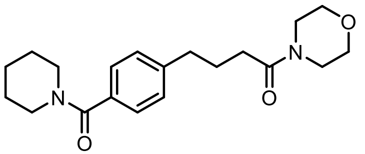

S97

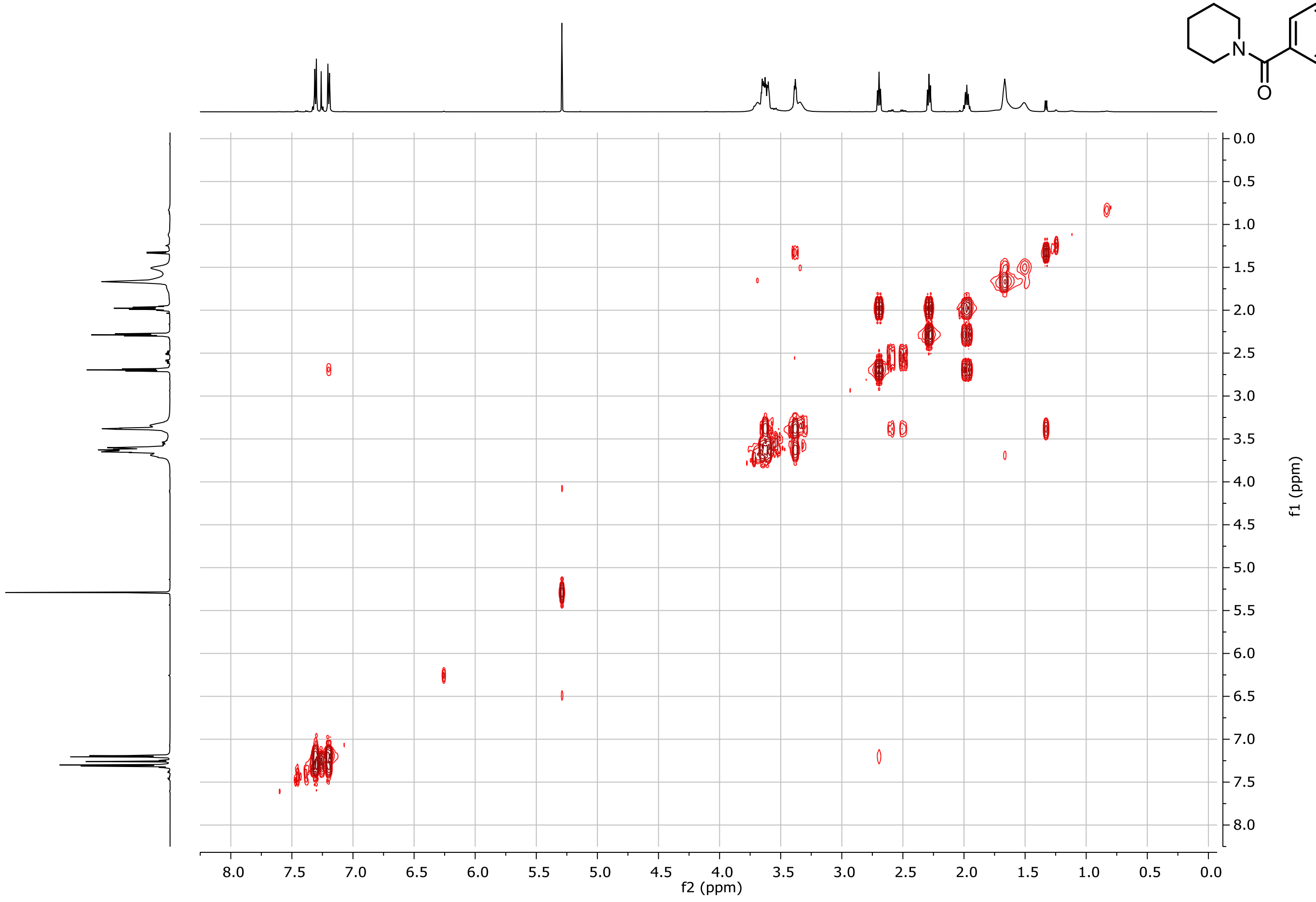

^{1}H, ^{13}C HMBC

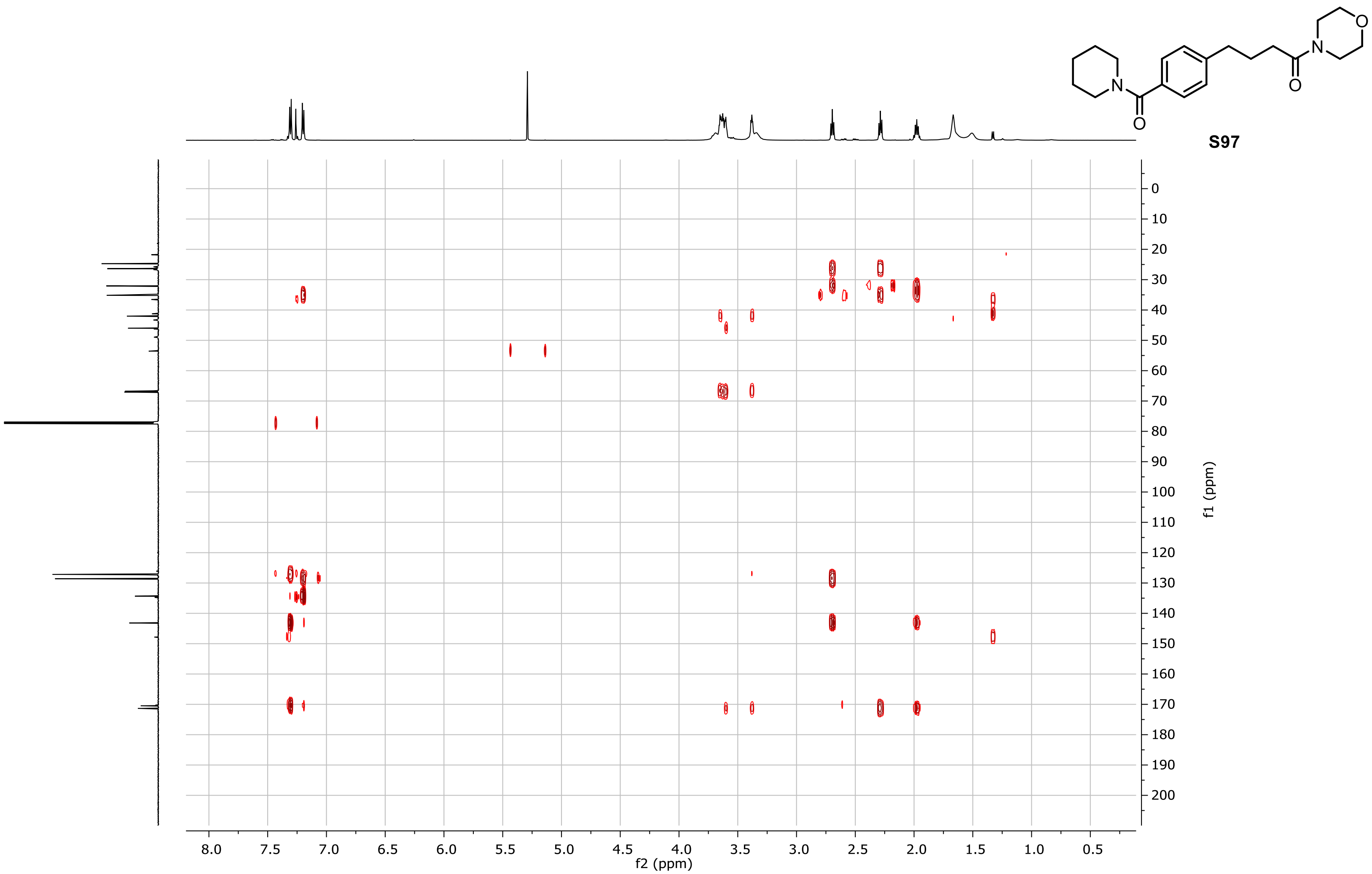

S97

^{1}H, ^{13}C HSQC

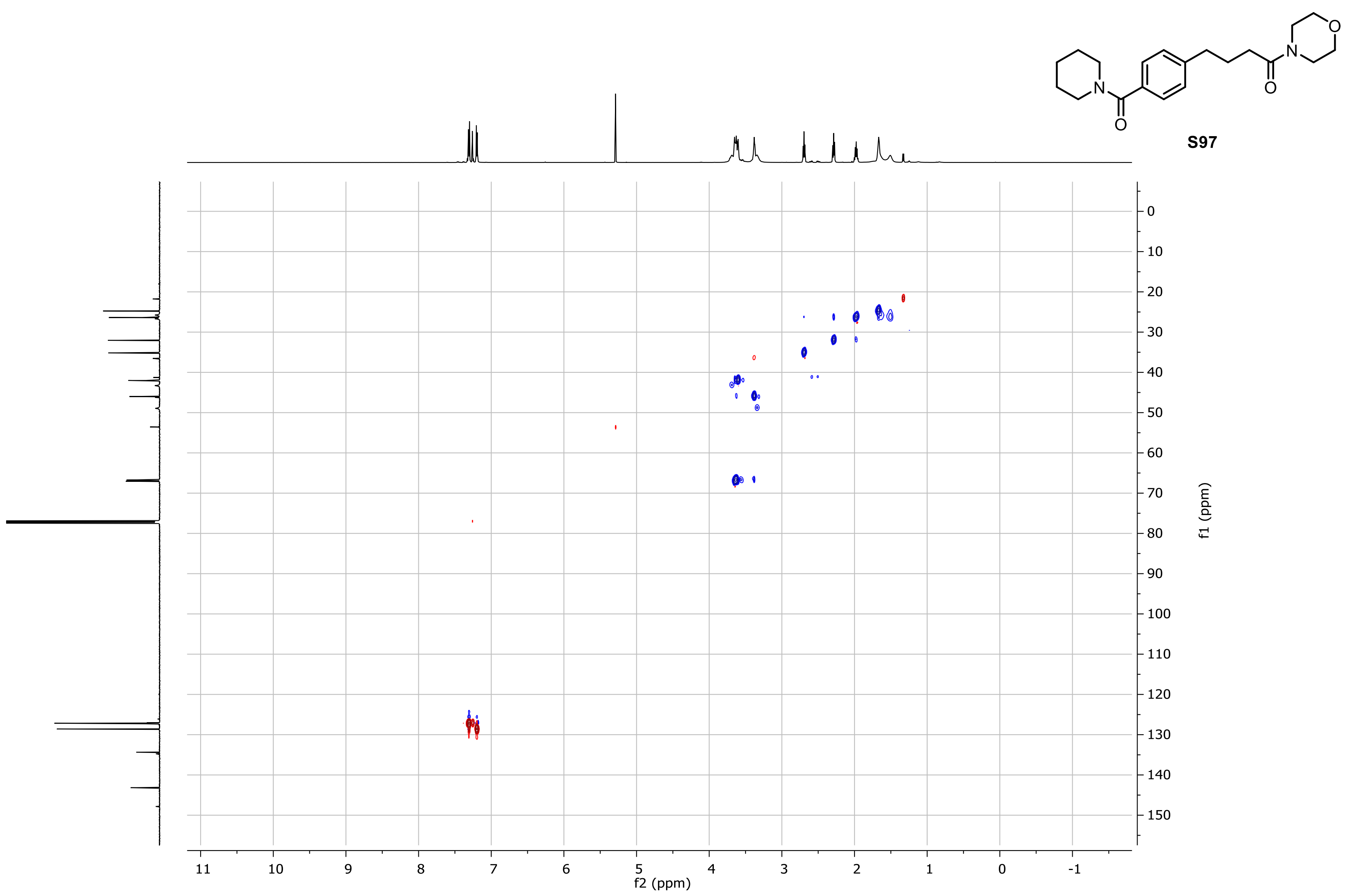

S97

GC-MS

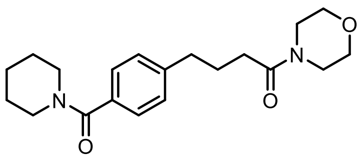

S97

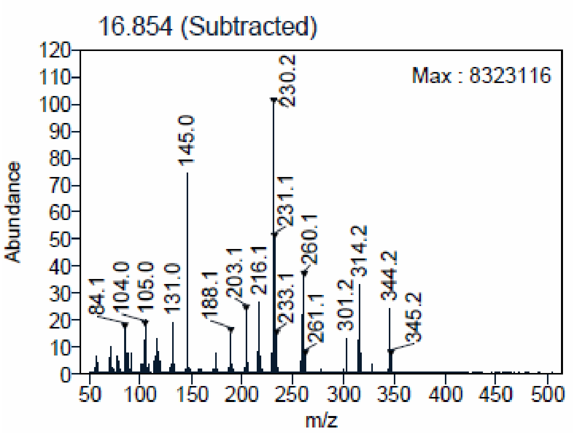
16.854 (Subtracted)
Max : 8323116
Abundance
m/z
84.1
104.0
105.0
131.0
145.0
188.1
203.1
216.1
230.2
231.1
233.1
260.1
261.1
301.2
314.2
344.2
345.2

IR

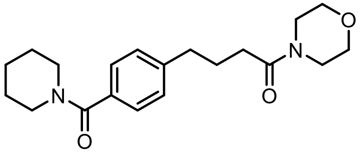

S97

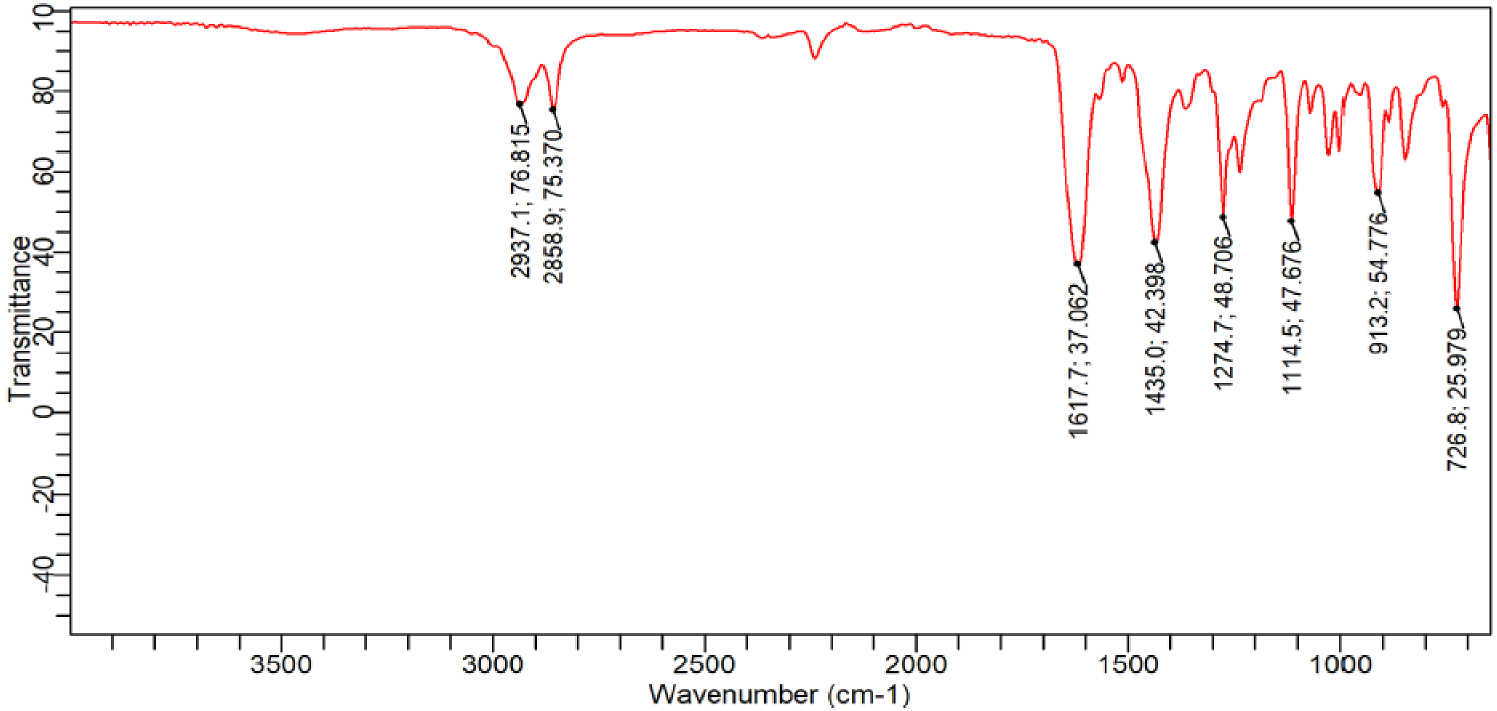
Transmittance
Wavenumber (cm-1)
2937.1; 76.815
2858.9; 75.370
1617.7; 37.062
1435.0; 42.398
1274.7; 48.706
1114.5; 47.676
913.2; 54.776
726.8; 25.979

49 1-Cyclopropyl-6-fluoro-7-(4-methylpiperazin-1-yl)-3-(morpholine-4-carbonyl)quinolin-4-one (S100)

^{1}H NMR

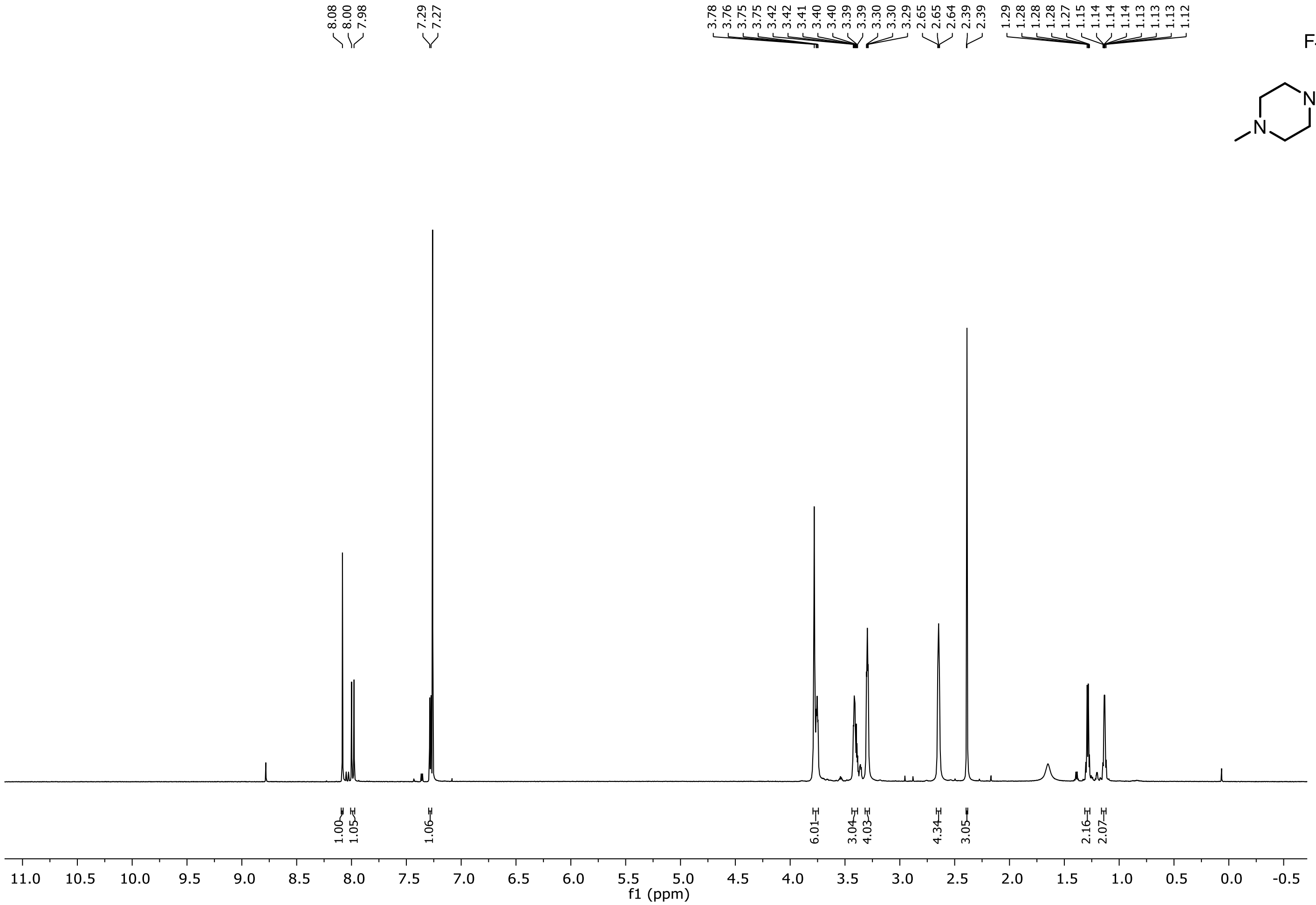

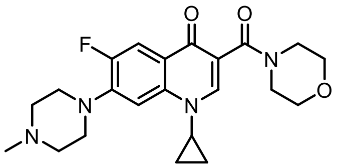

S100

^{13}C NMR

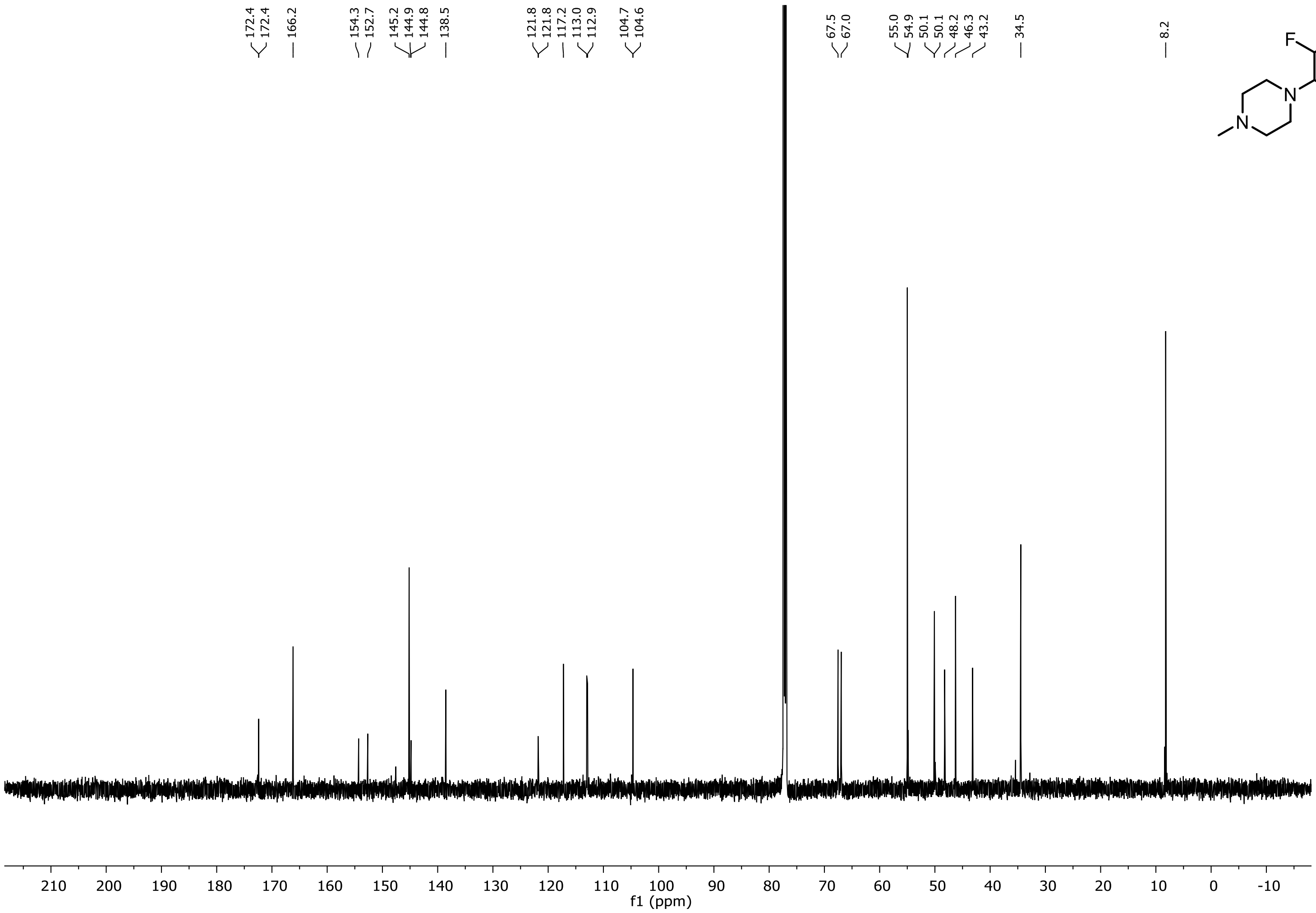
172.4
172.4
166.2
154.3
152.7
145.2
144.9
144.8
138.5
121.8
121.8
117.2
113.0
112.9
104.7
104.6
67.5
67.0
55.0
54.9
50.1
50.1
48.2
46.3
43.2
34.5
8.2
210 200 190 180 170 160 150 140 130 120 110 100 90 80 70 60 50 40 30 20 10 0 -10
f1 (ppm)

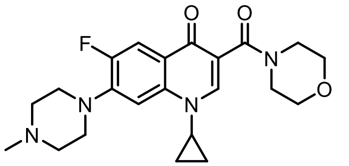
F
O
O
N
N
N
N
O

S100

^{19}F NMR

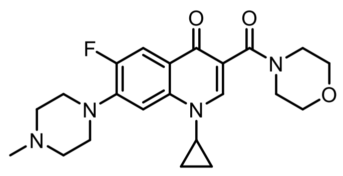

F
O
O
N
N
N
N
O

S100

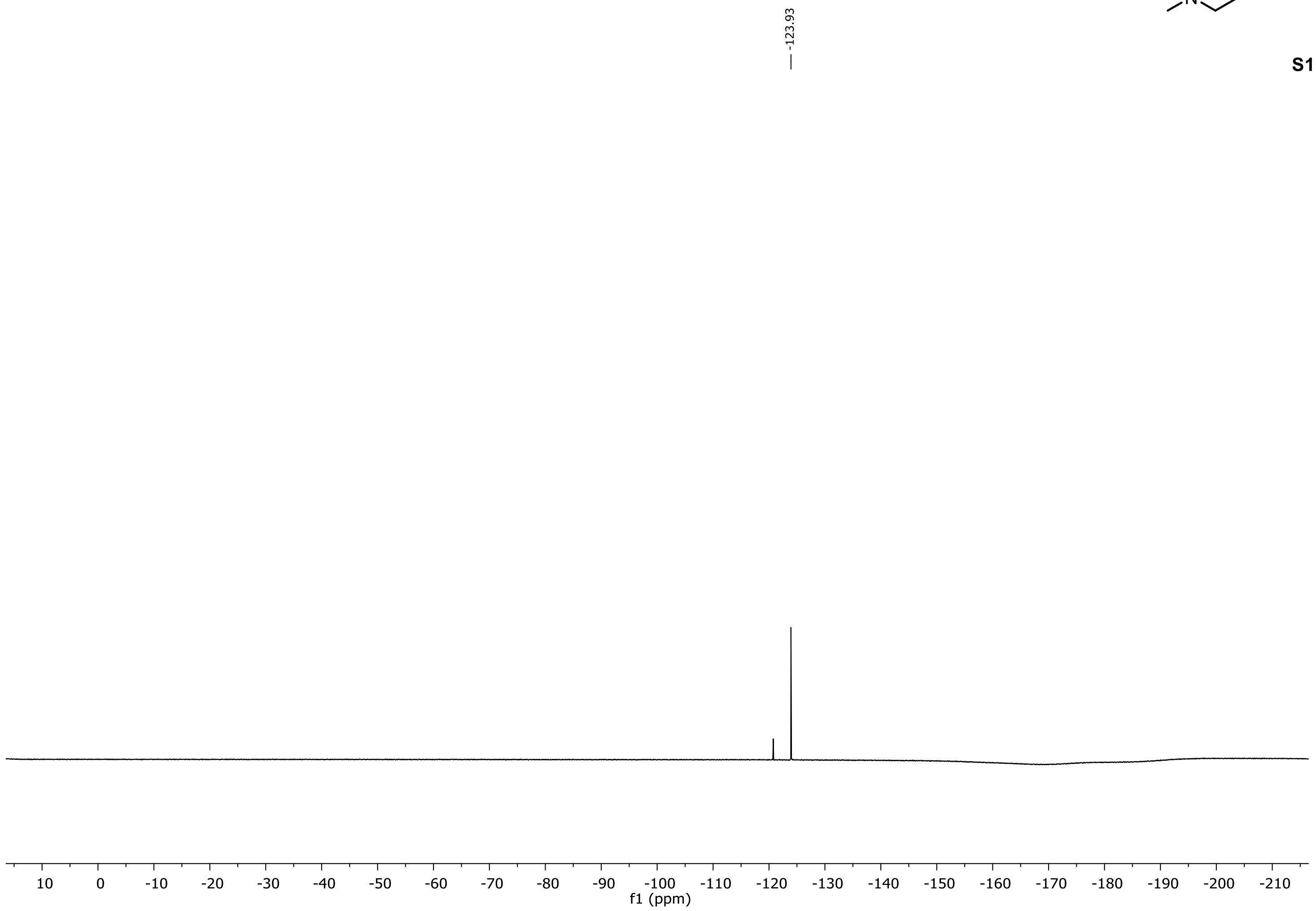

-123.93
10
0
-10
-20
-30
-40
-50
-60
-70
-80
-90
-100
-110
-120
-130
-140
-150
-160
-170
-180
-190
-200
-210
f1 (ppm)

^{1}H, ^{1}H COSY

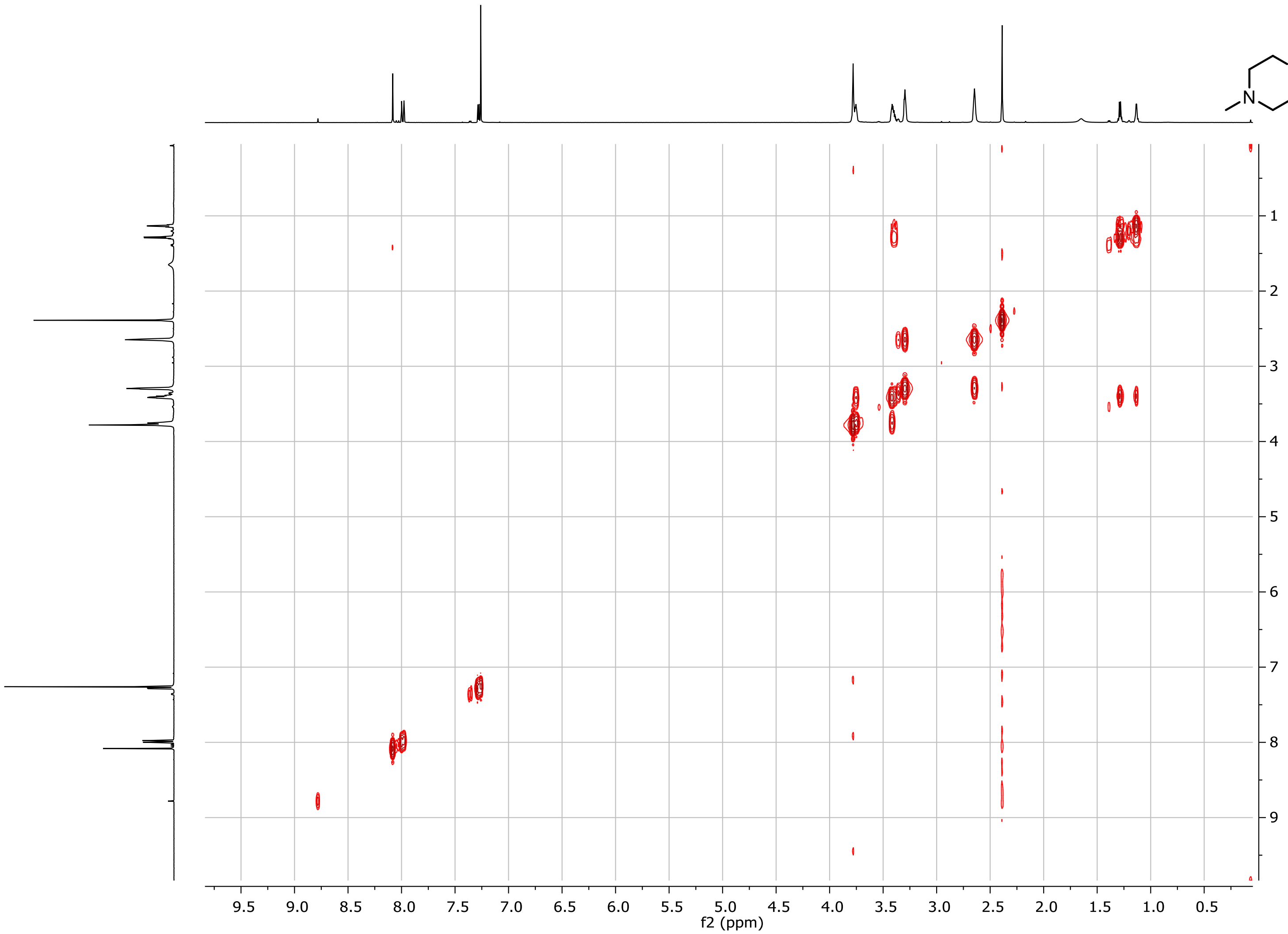

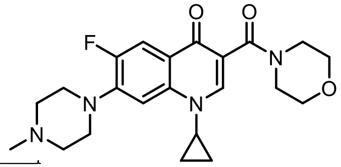

S100

^{1}H, ^{13}C HMBC

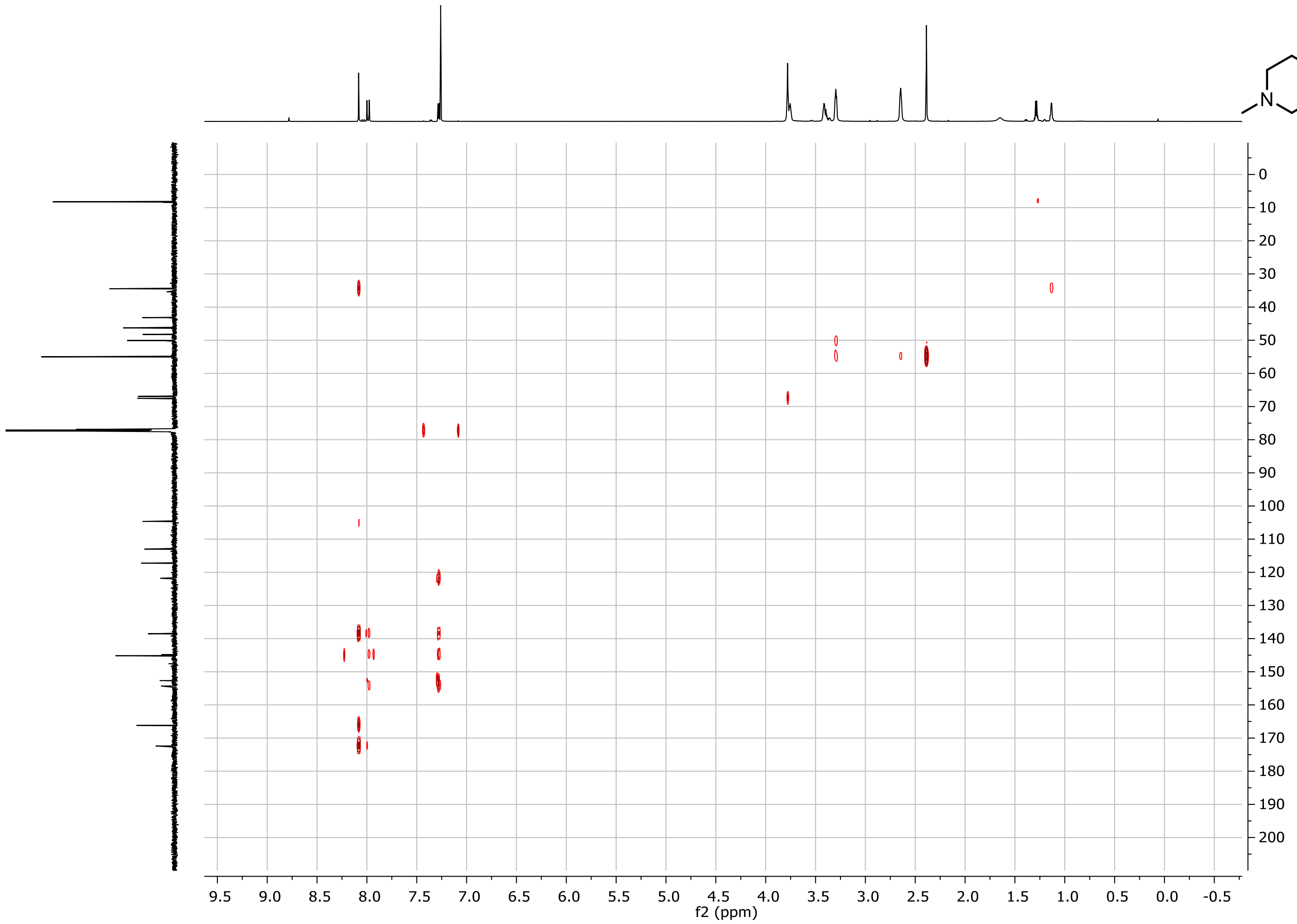

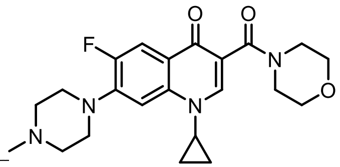

S100

^{1}H, ^{13}C HSQC

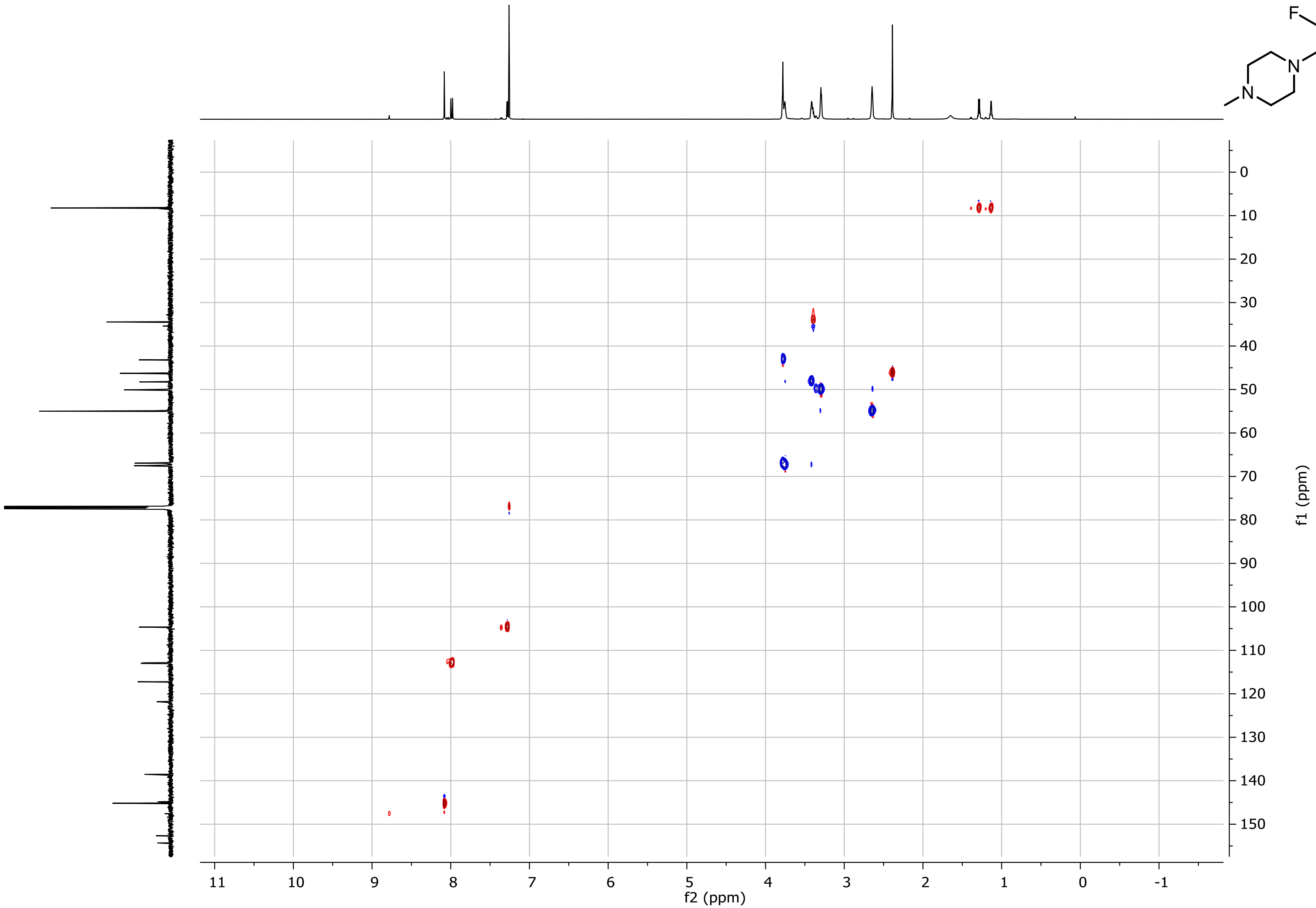

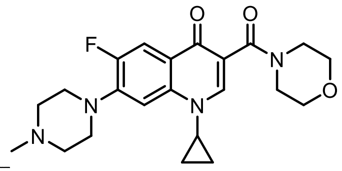

S100

HRMS

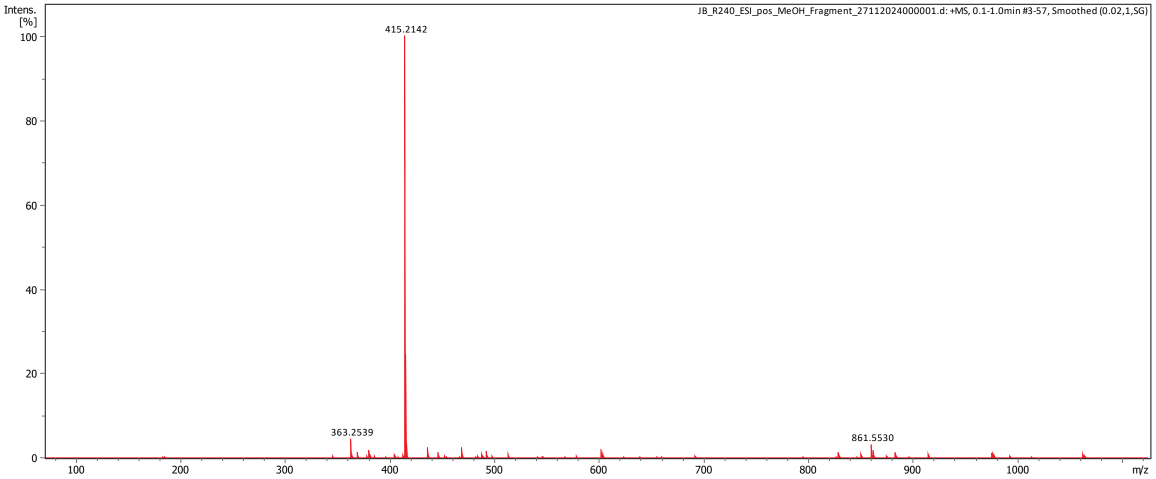

Intens. [%]
JB_R240_ESI_pos_MeOH_Fragment_27112024000001.d: +MS, 0.1-1.0min #3-57, Smoothed (0.02,1,SG)
415.2142
363.2539
861.5530
m/z

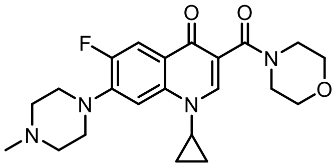

S100

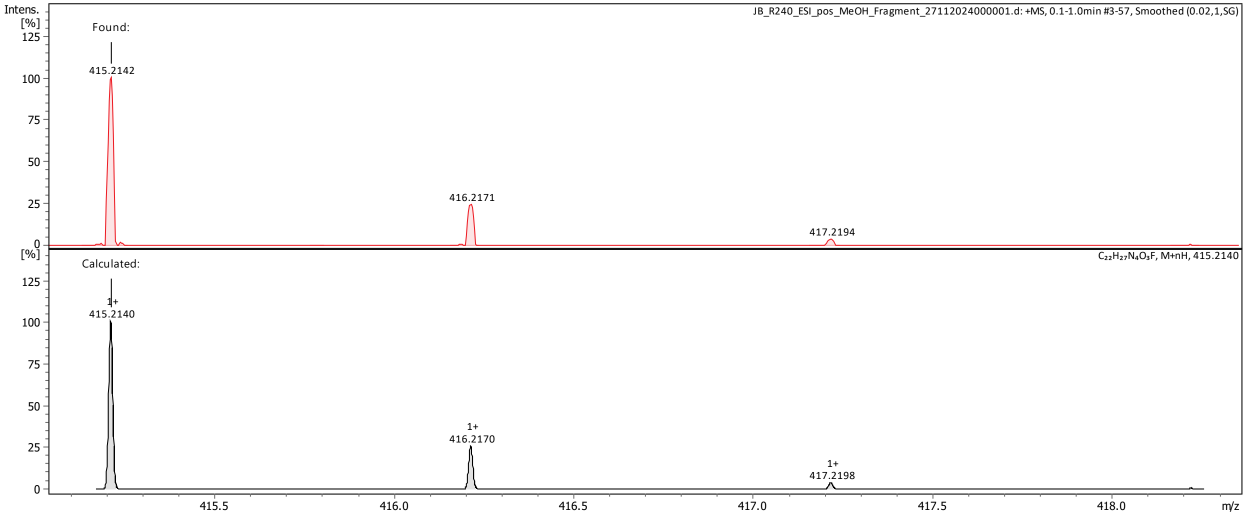

Intens. [%]
JB_R240_ESI_pos_MeOH_Fragment_27112024000001.d: +MS, 0.1-1.0min #3-57, Smoothed (0.02,1,SG)
Found:
415.2142
416.2171
417.2194
Calculated:
C₂₂H₂₇N₄O₃F, M+nH, 415.2140
1+ 415.2140
1+ 416.2170
1+ 417.2198
m/z

IR

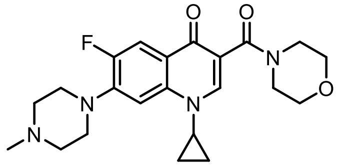

O
O
F
N
N
N
O
N

S100

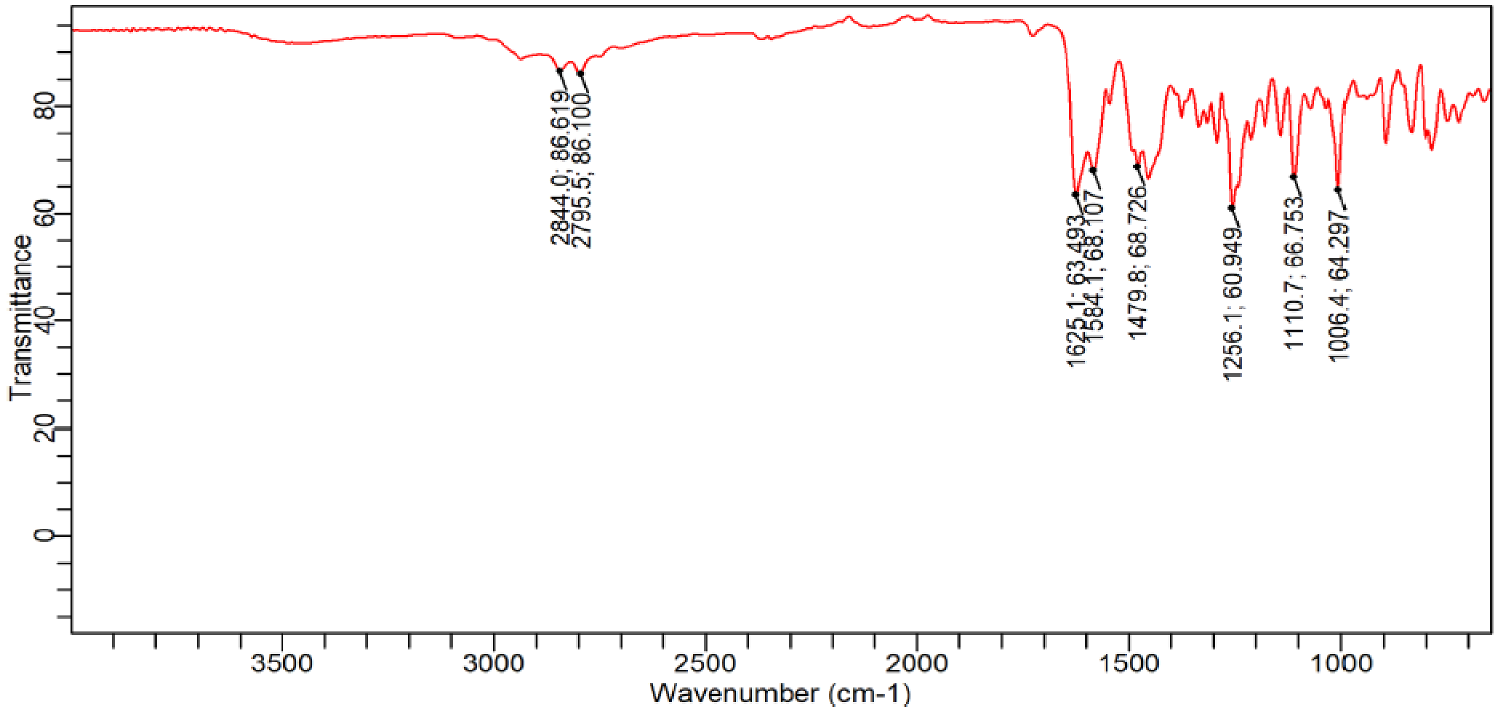

2844.0; 86.619
2795.5; 86.100
1625.1; 63.493
1584.1; 68.107
1479.8; 68.726
1256.1; 60.949
1110.7; 66.753
1006.4; 64.297
Transmittance
80
60
40
20
0
3500
3000
2500
2000
1500
1000
Wavenumber (cm-1)

50 7-[4-(4-Chlorobenzoyl)piperazin-1-yl]-1-cyclopropyl-6-fluoro-3-(piperidine-1-carbonyl)quinolin-4-one (S102)

^{1}H NMR

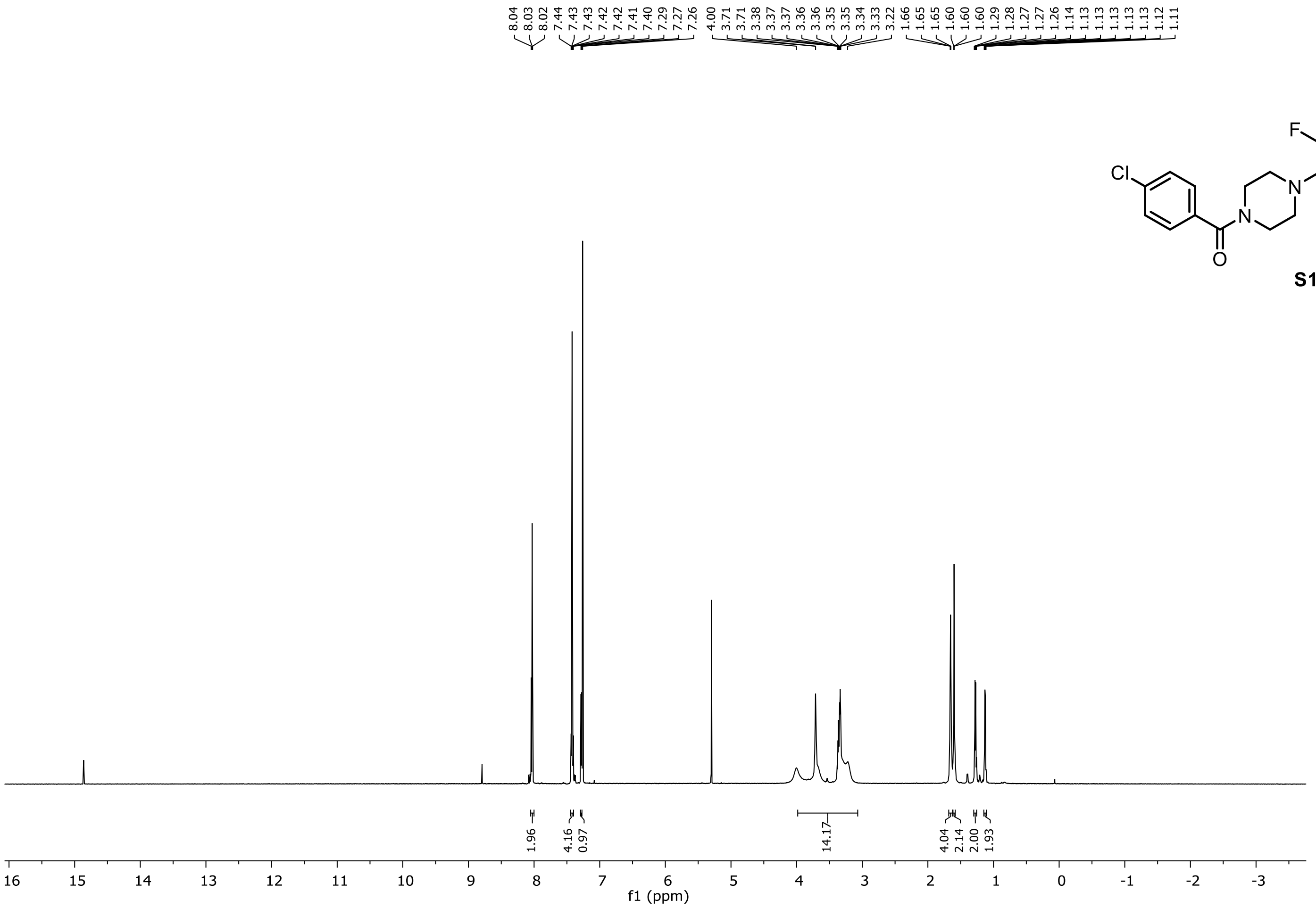

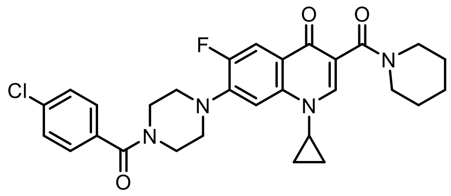

S102

^{13}C NMR

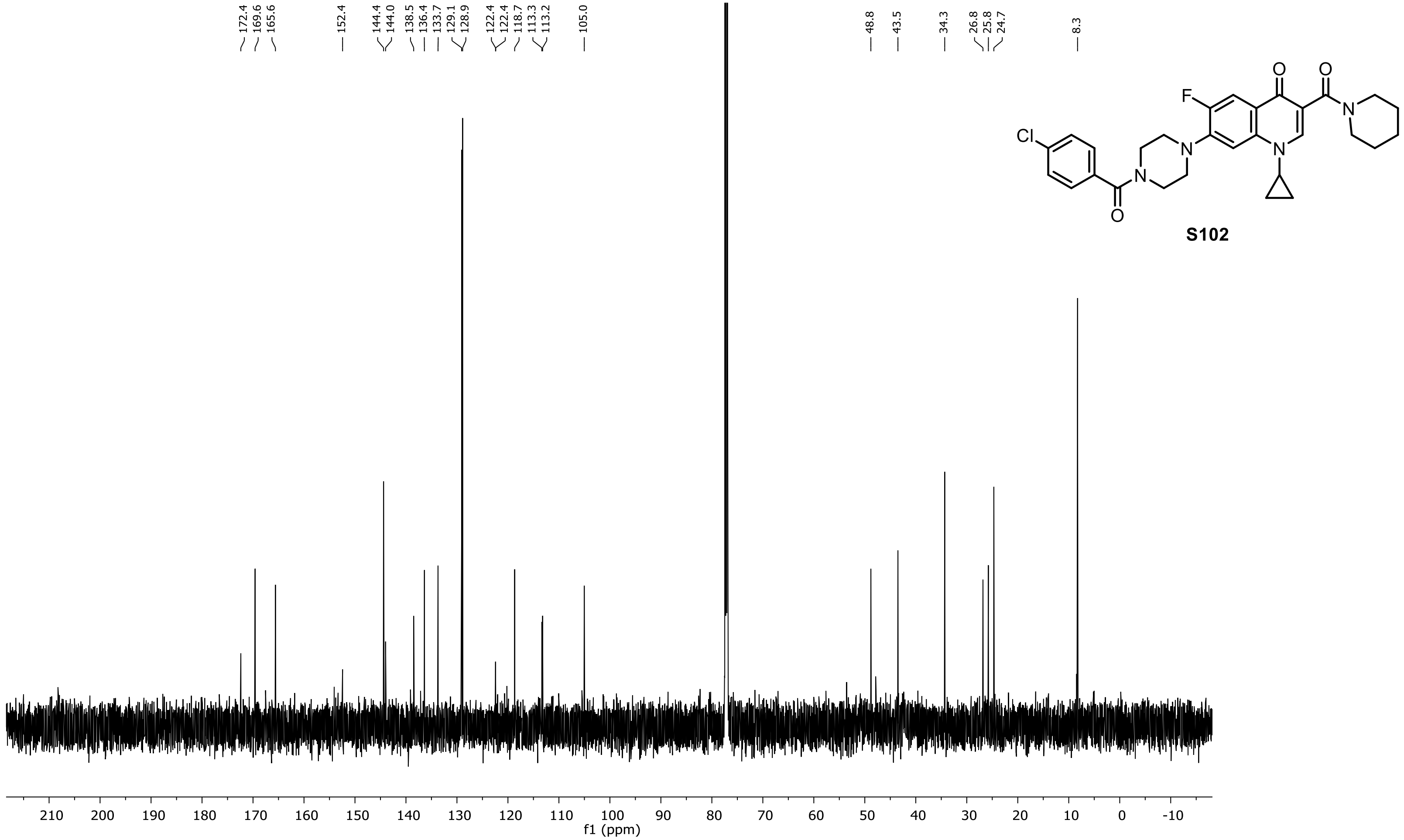
172.4
169.6
165.6
152.4
144.4
144.0
138.5
136.4
133.7
129.1
128.9
122.4
122.4
118.7
113.3
113.2
105.0
48.8
43.5
34.3
26.8
25.8
24.7
8.3
210 200 190 180 170 160 150 140 130 120 110 100 90 80 70 60 50 40 30 20 10 0 -10
f1 (ppm)
Cl
F
O
O
N
N
N
N
O

S102

^{19}F NMR

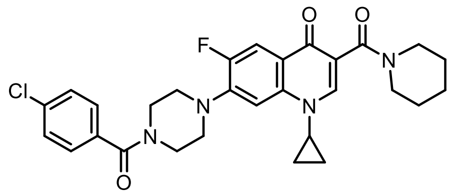

Cl
F
O
O
N
N
N
N
O

S102

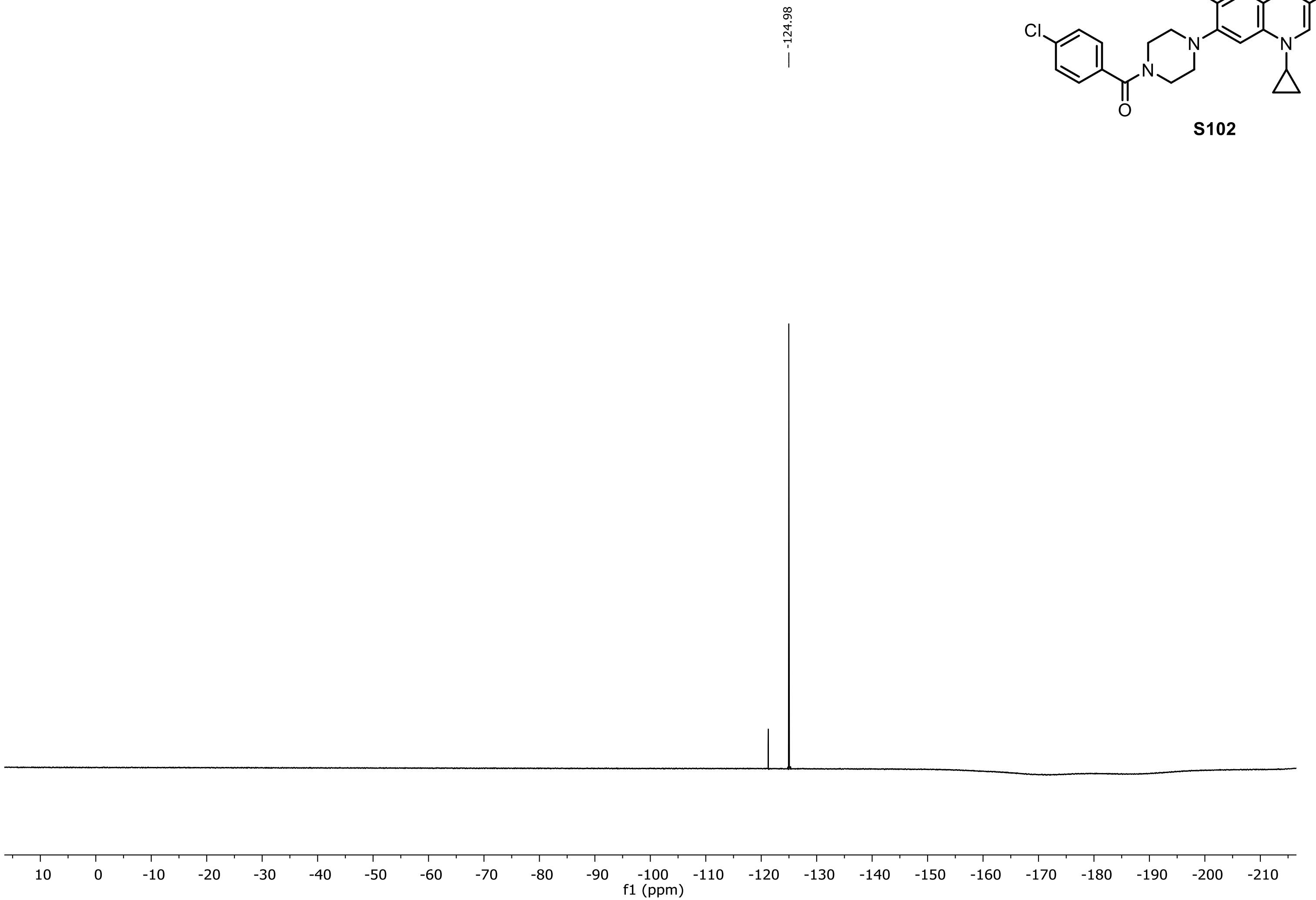

-124.98
10
0
-10
-20
-30
-40
-50
-60
-70
-80
-90
-100
-110
-120
-130
-140
-150
-160
-170
-180
-190
-200
-210
f1 (ppm)

^{1}H, ^{1}H COSY

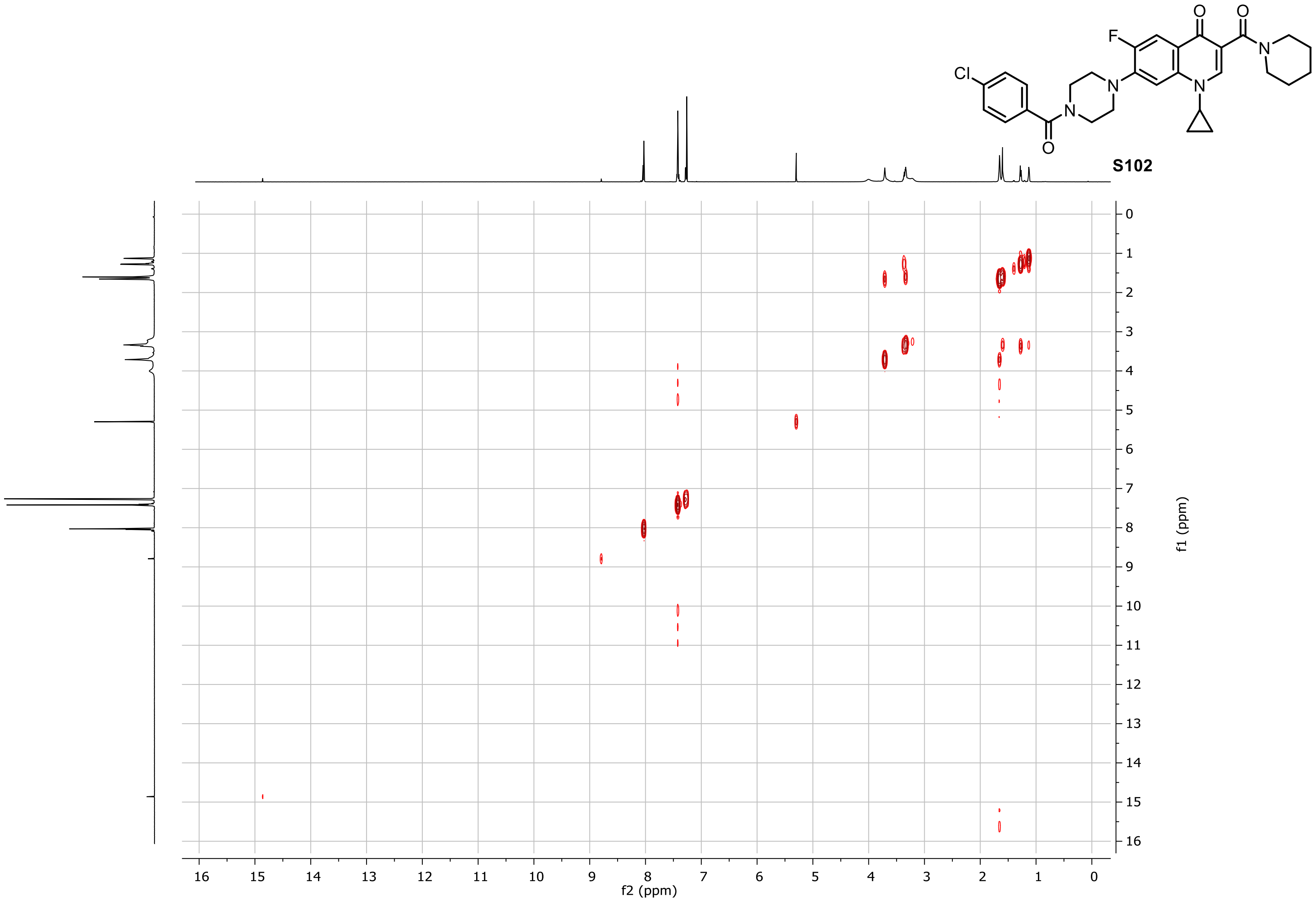

S102

^1H, ^{13}C HMBC

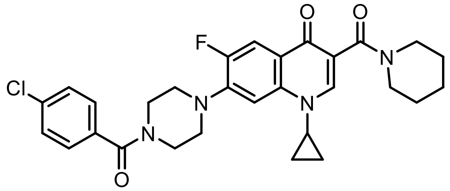

S102

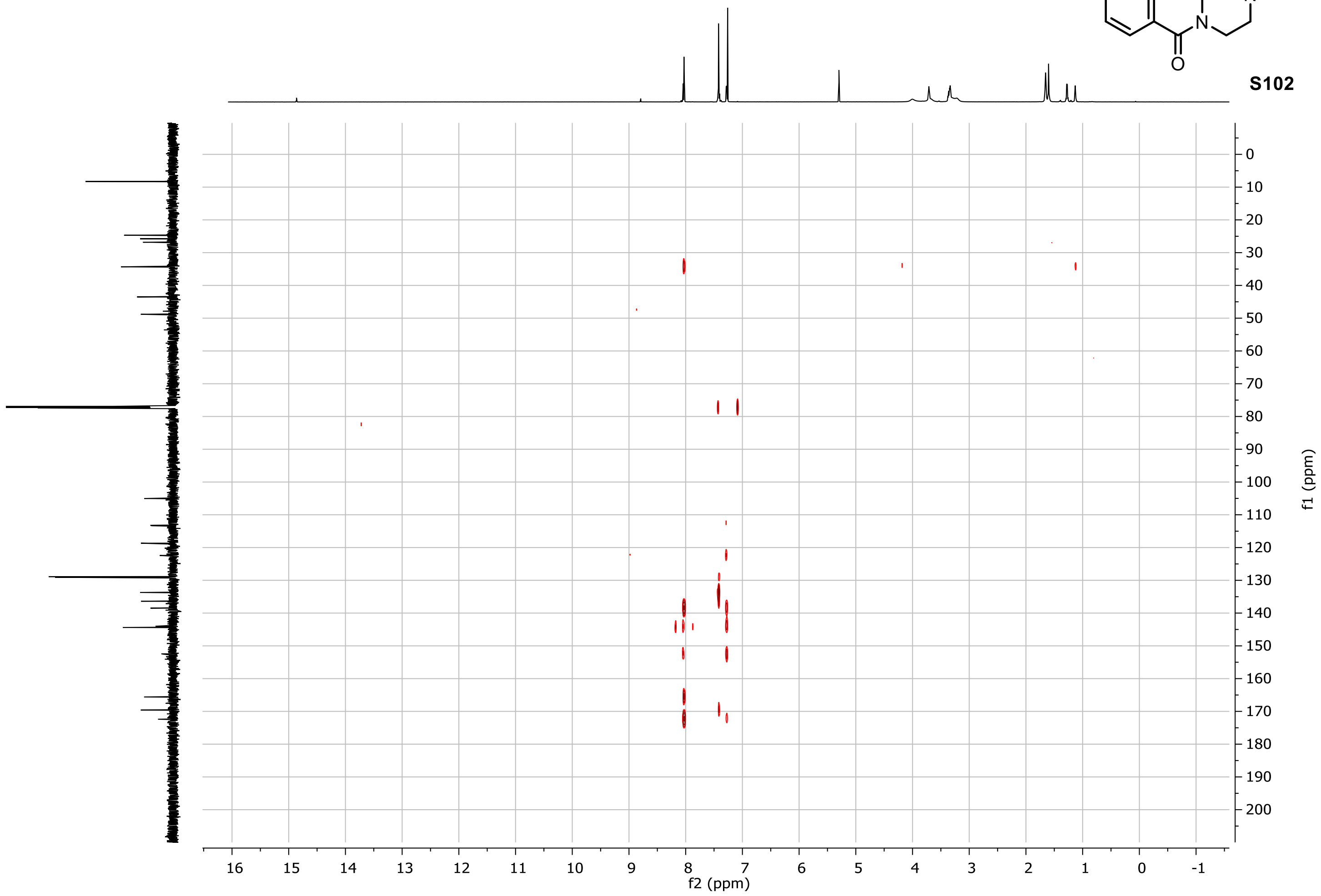

^{1}H, ^{13}C HSQC

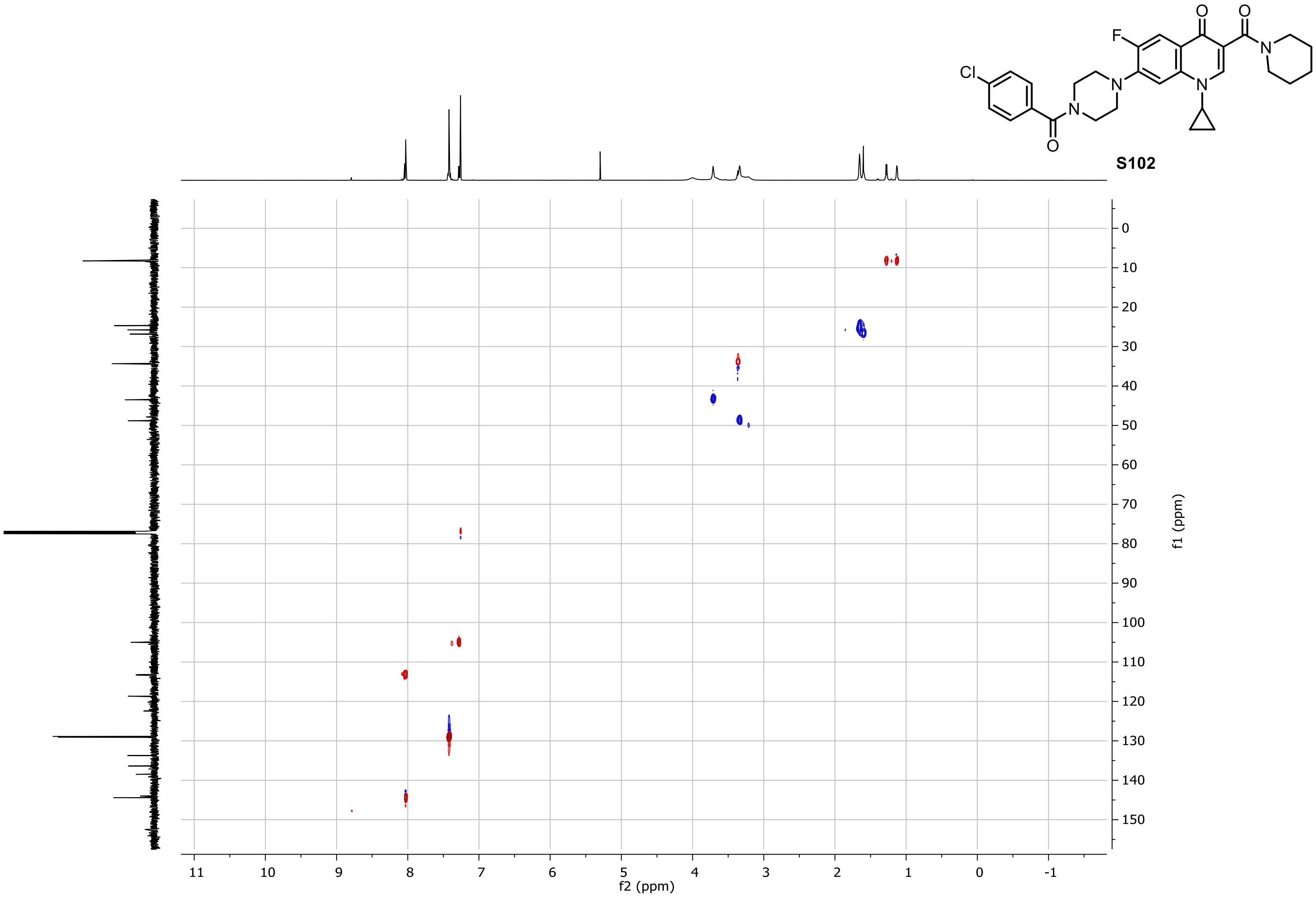

Cl
F
O
O
N
N
N
N
O
S102
0
10
20
30
40
50
60
70
80
90
100
110
120
130
140
150
f1 (ppm)
11
10
9
8
7
6
5
4
3
2
1
0
-1
f2 (ppm)

IR

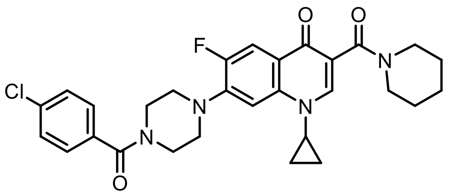

Cl
F
O
O
N
N
N
N
O

S102

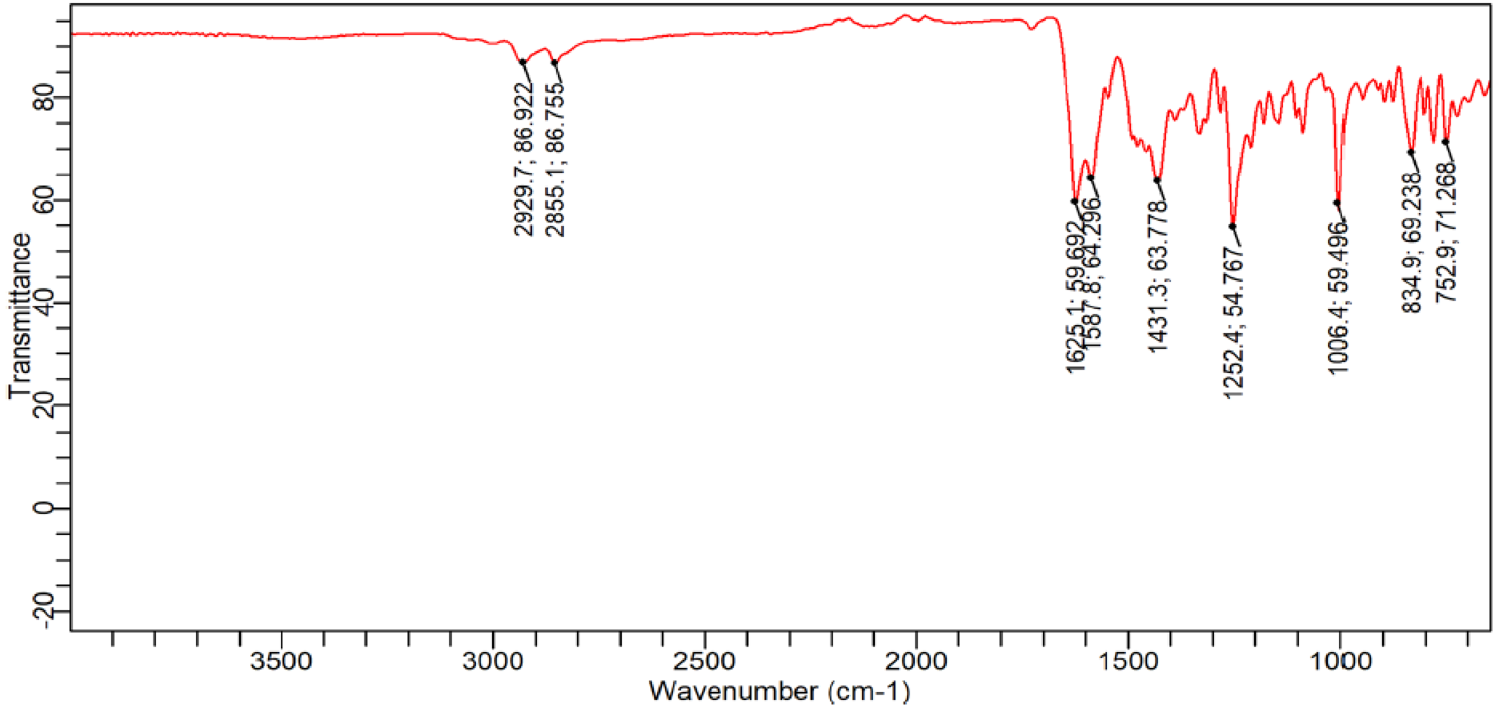

Transmittance
Wavenumber (cm-1)
2929.7; 86.922
2855.1; 86.755
1625.1; 59.692
1587.8; 64.296
1431.3; 63.778
1252.4; 54.767
1006.4; 59.496
834.9; 69.238
752.9; 71.268

51 Benzyl alcohol from phenyl(thiomorpholino)methanone (2g)

^{1}H NMR

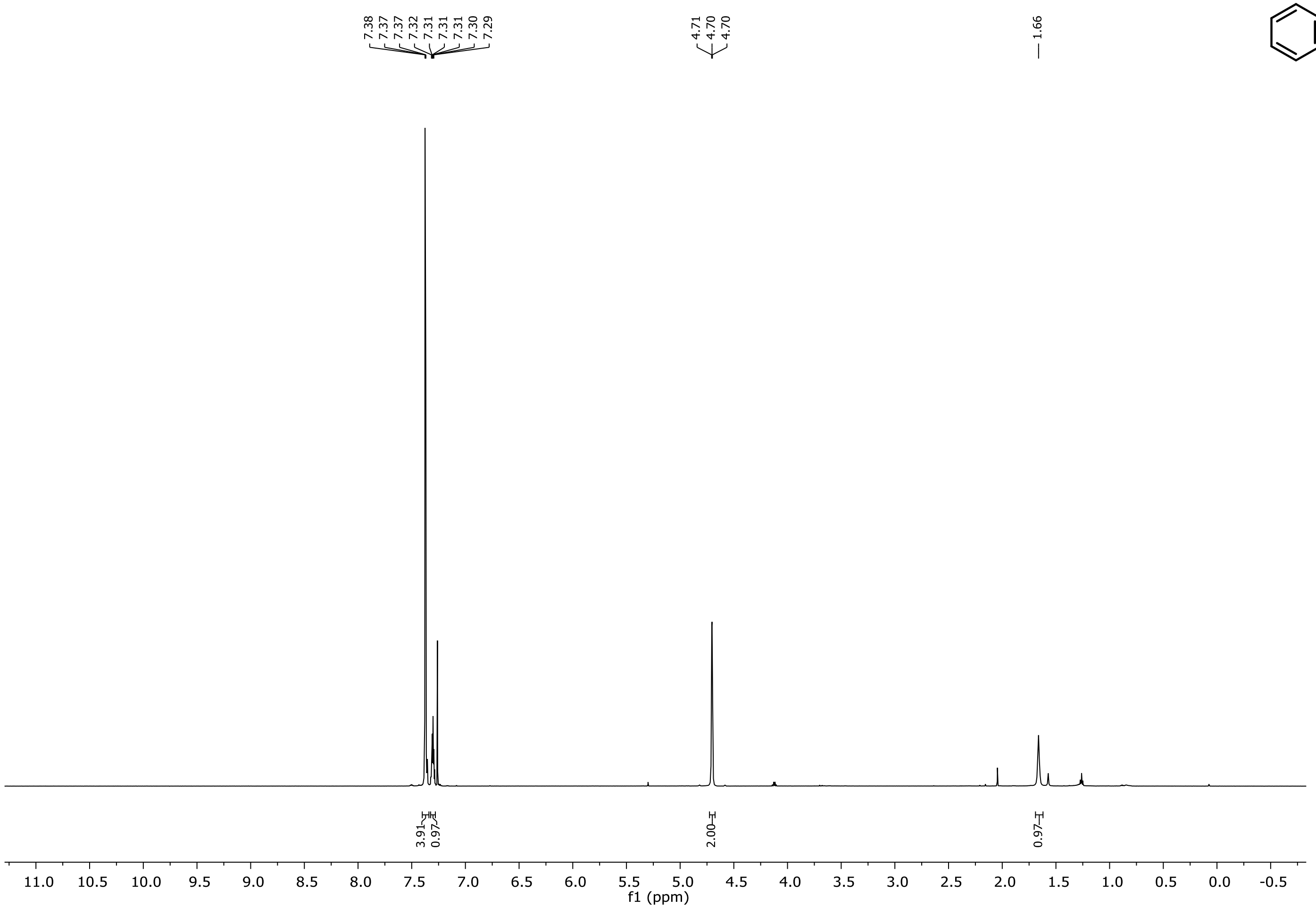

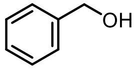

2g

^{13}C NMR

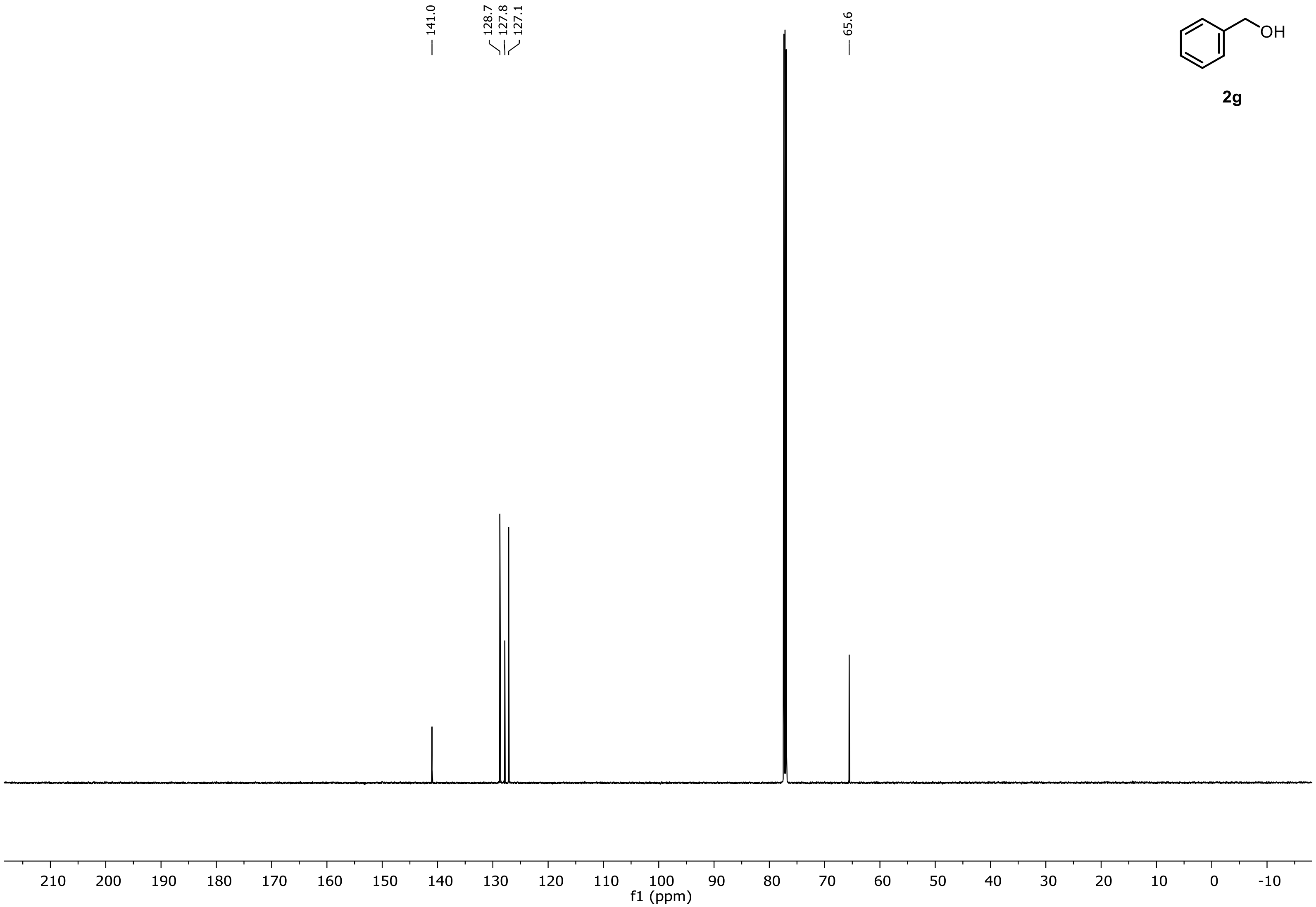
141.0
128.7
127.8
127.1
65.6
OH
210
200
190
180
170
160
150
140
130
120
110
100
90
80
70
60
50
40
30
20
10
0
-10
f1 (ppm)

2g

^1H, ^1H COSY

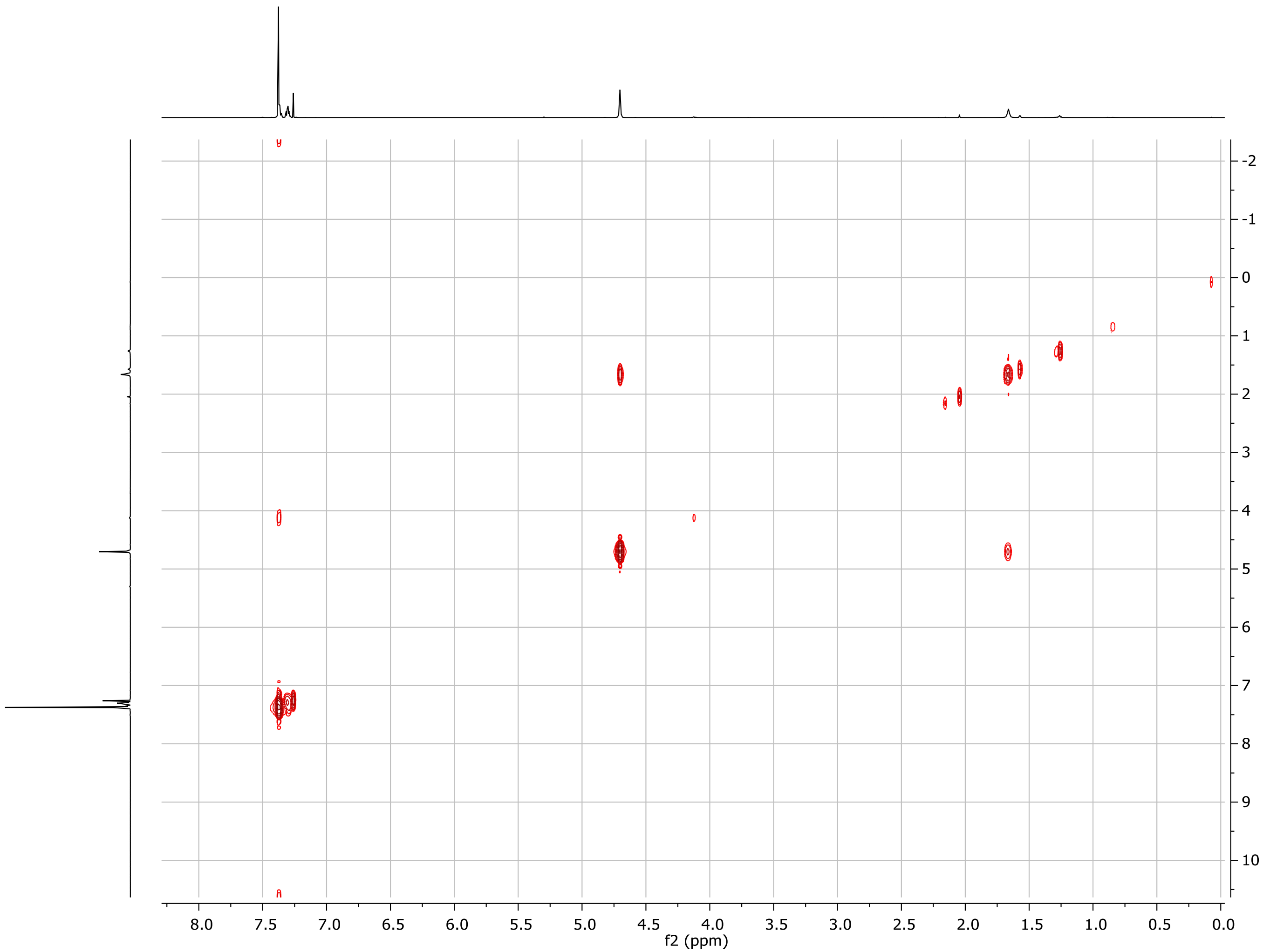

8.0
7.5
7.0
6.5
6.0
5.5
5.0
4.5
4.0
3.5
3.0
2.5
2.0
1.5
1.0
0.5
0.0
f2 (ppm)
-2
-1
0
1
2
3
4
5
6
7
8
9
10
f1 (ppm)

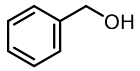

OH

2g

^{1}H, ^{13}C HMBC

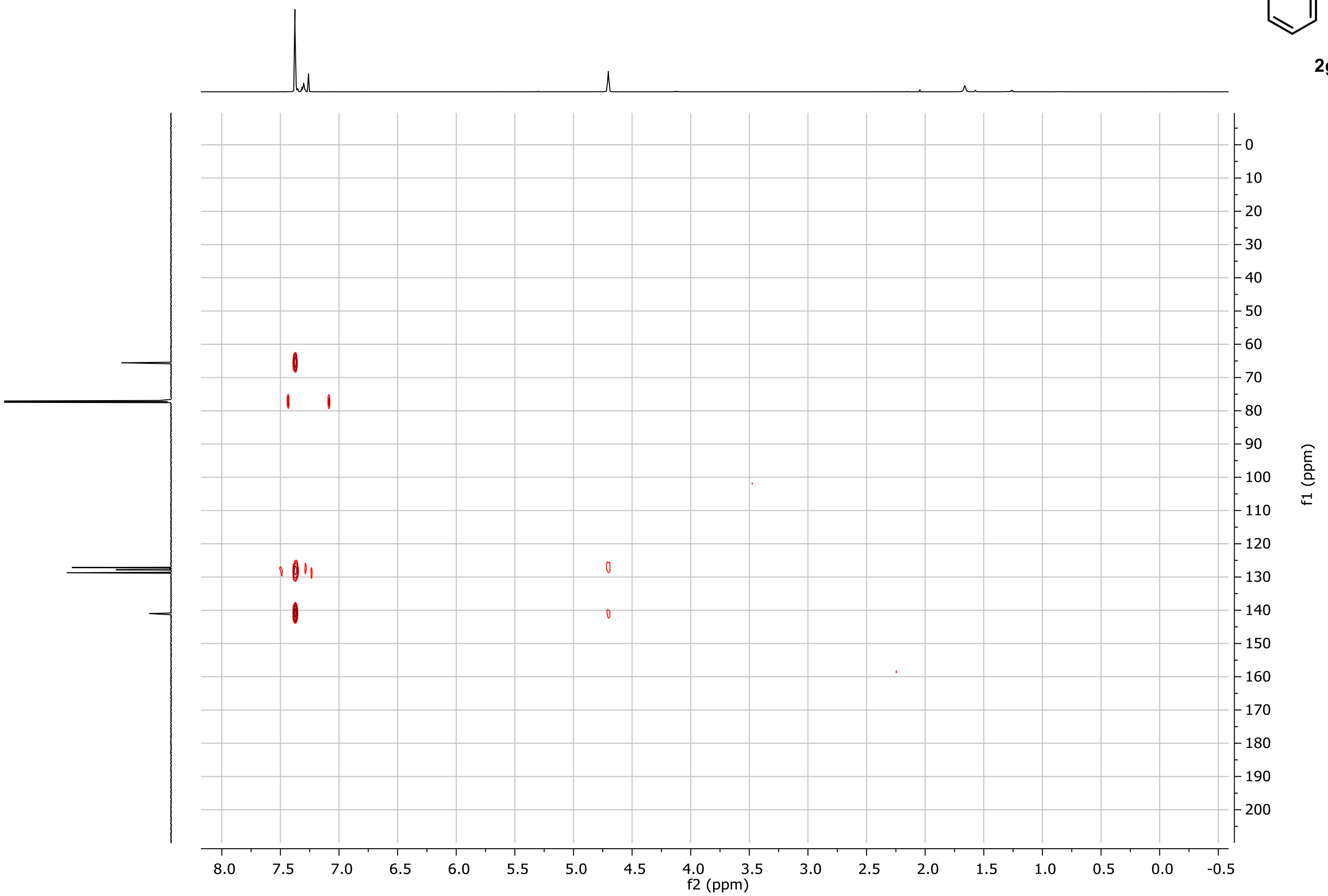

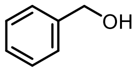

2g

^{1}H, ^{13}C HSQC

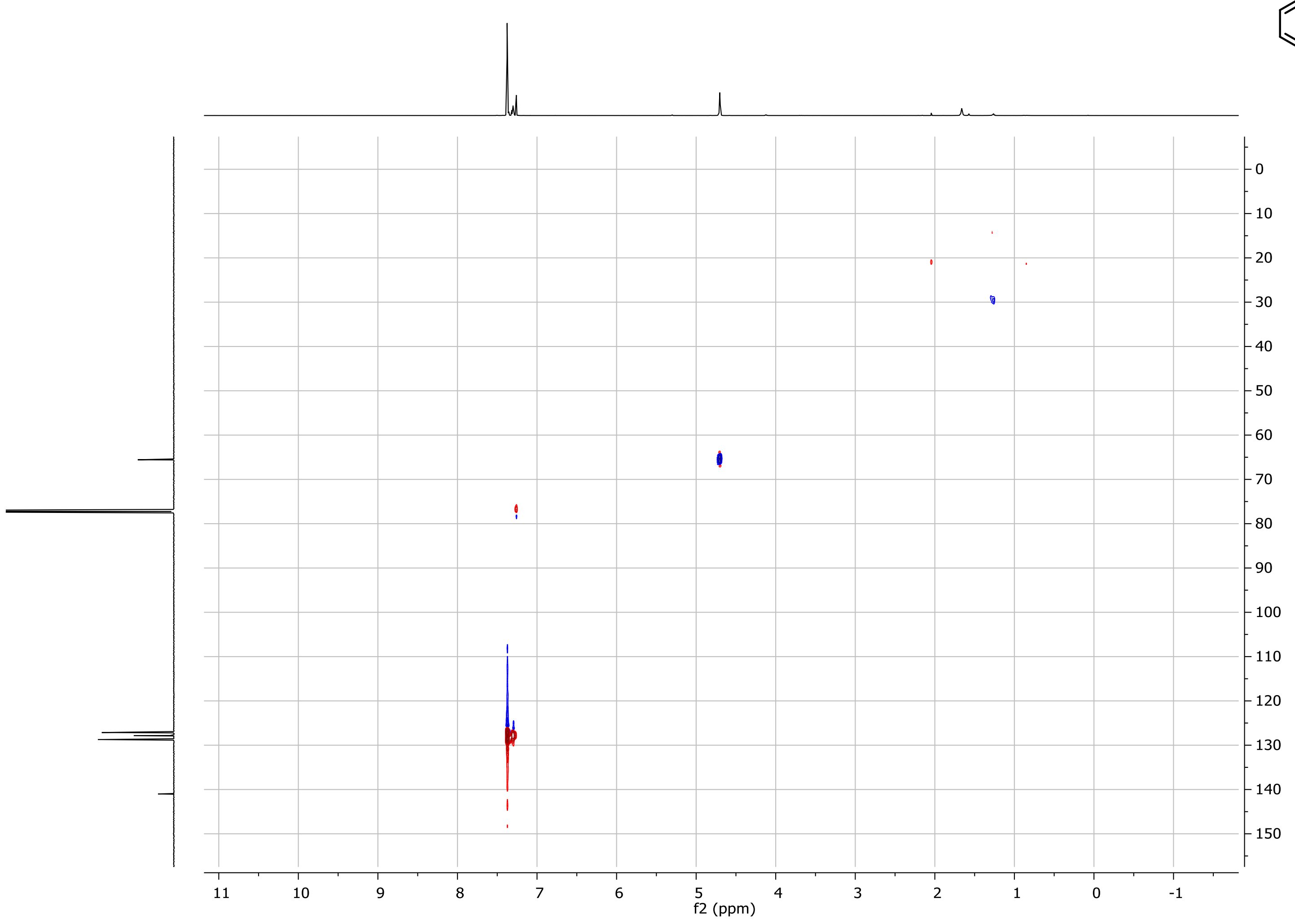

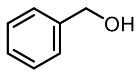

2g

HRMS

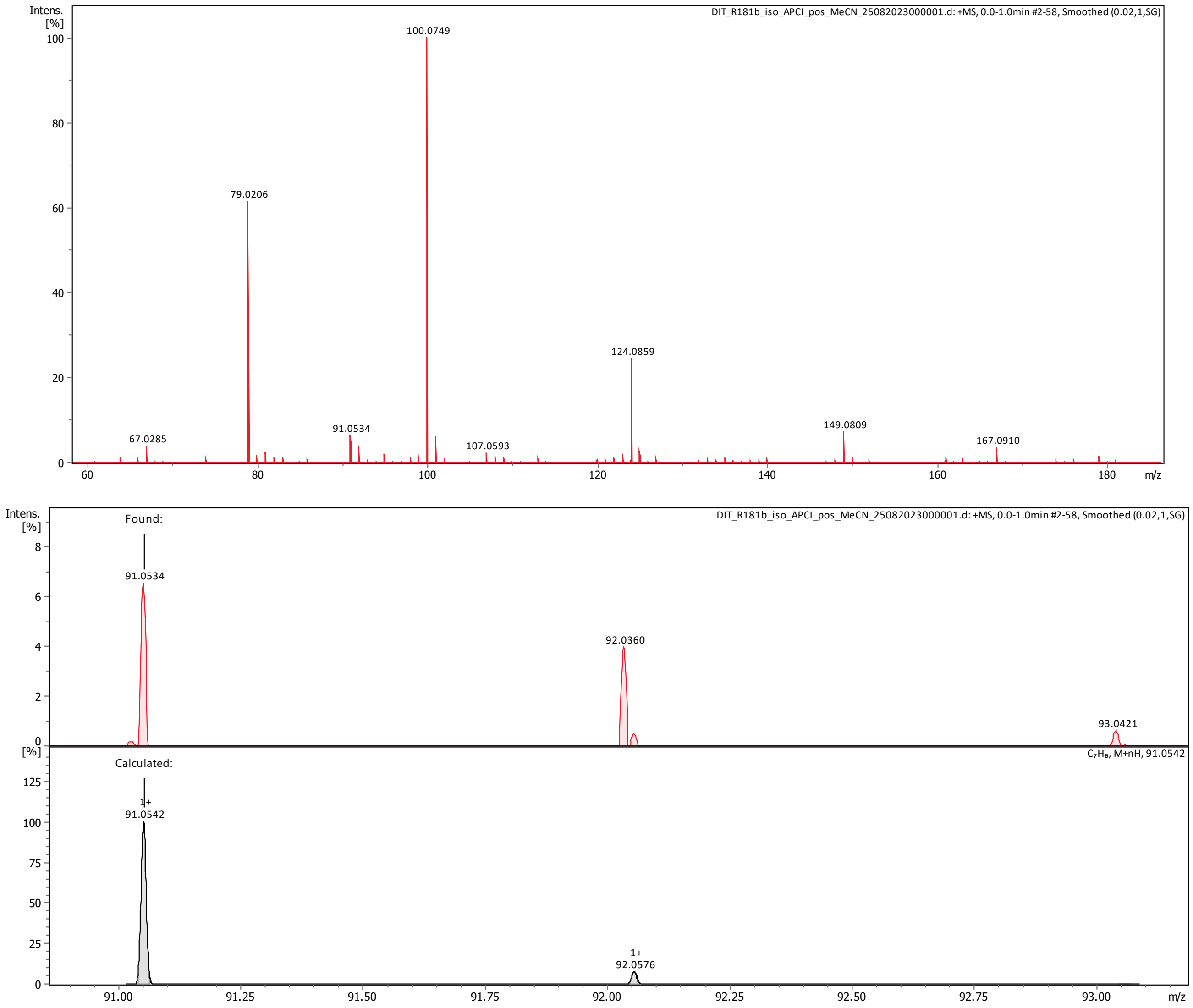

Intens. [%]
DIT_R181b_iso_APCI_pos_MeCN_25082023000001.d: +MS, 0.0-1.0min #2-58, Smoothed (0.02,1,SG)
100.0749
79.0206
124.0859
149.0809
167.0910
107.0593
91.0534
67.0285
m/z
Found:
91.0534
92.0360
93.0421
Calculated:
C7H6, M+nH, 91.0542
1+
91.0542
1+
92.0576

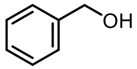

OH

2g

52 Benzyl alcohol from 2-oxa-6-azaspiro[3.3]hept-6-ylphenylmethanone (2h)

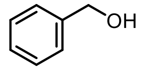

2h

^{1}H NMR

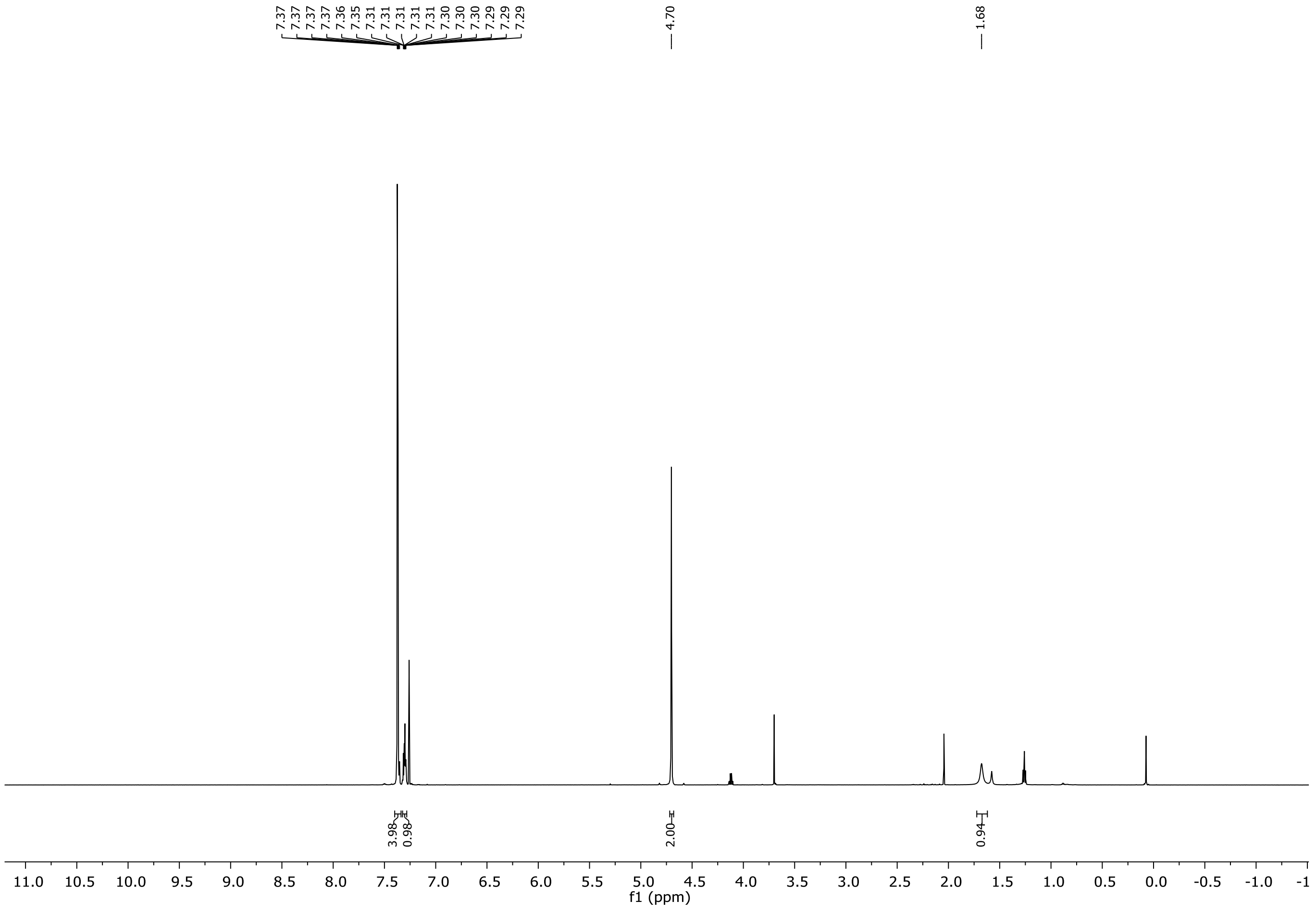

^{13}C NMR

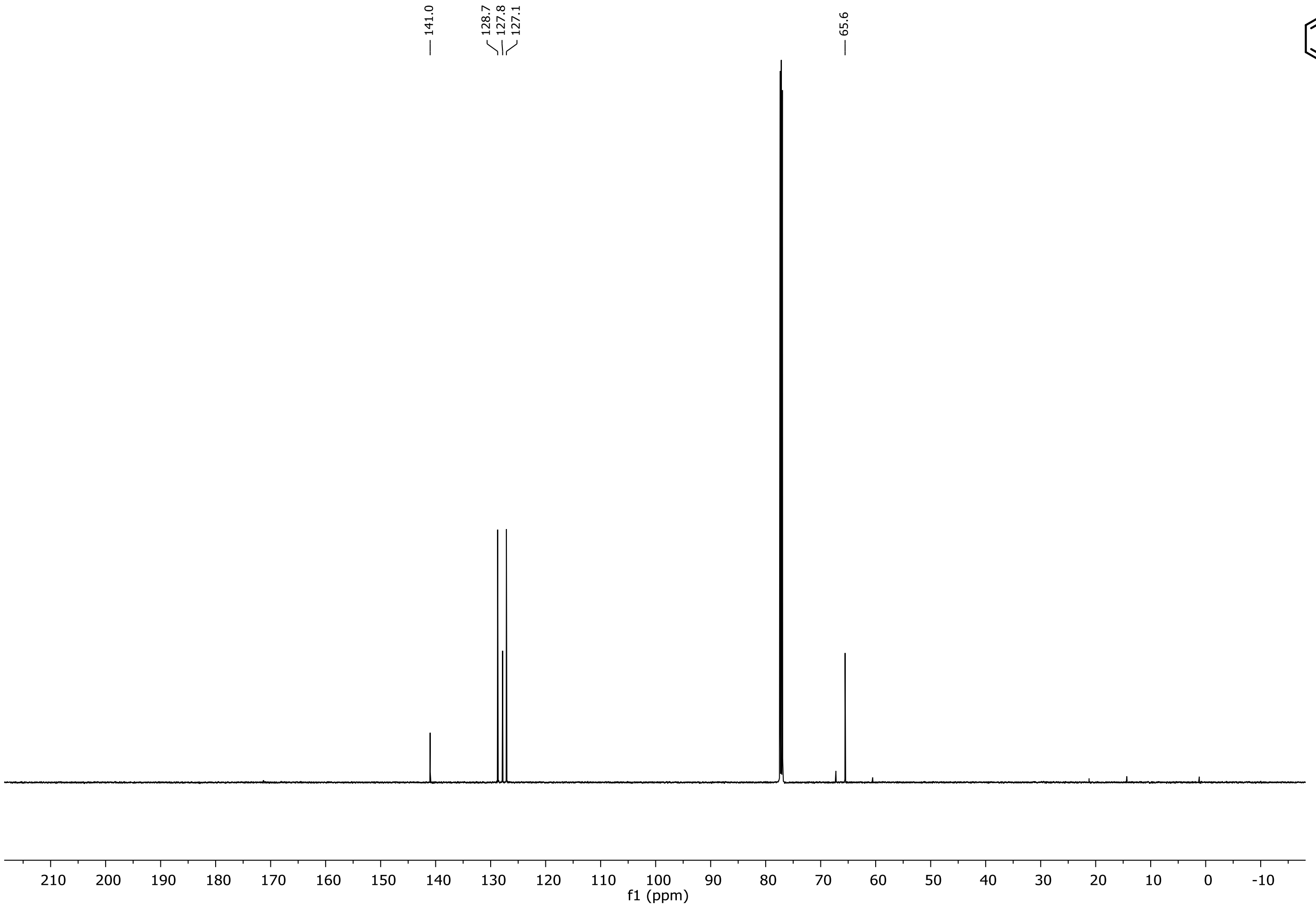
141.0
128.7
127.8
127.1
65.6
210
200
190
180
170
160
150
140
130
120
110
100
90
80
70
60
50
40
30
20
10
0
-10
f1 (ppm)

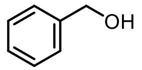
OH

2h

^{1}H, ^{1}H COSY

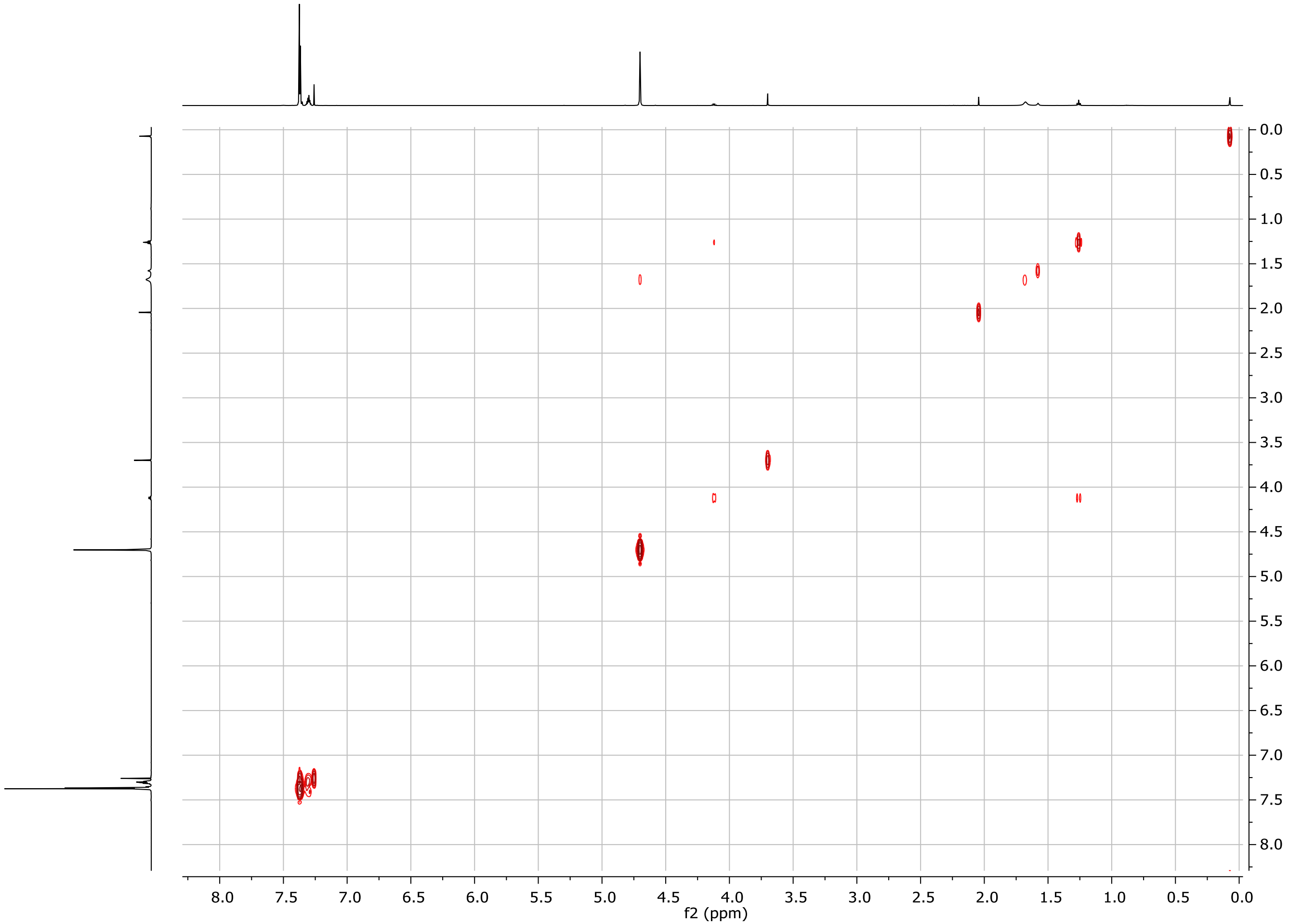

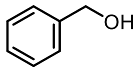

2h

^1H, ^{13}C HMBC

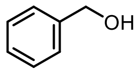

2h

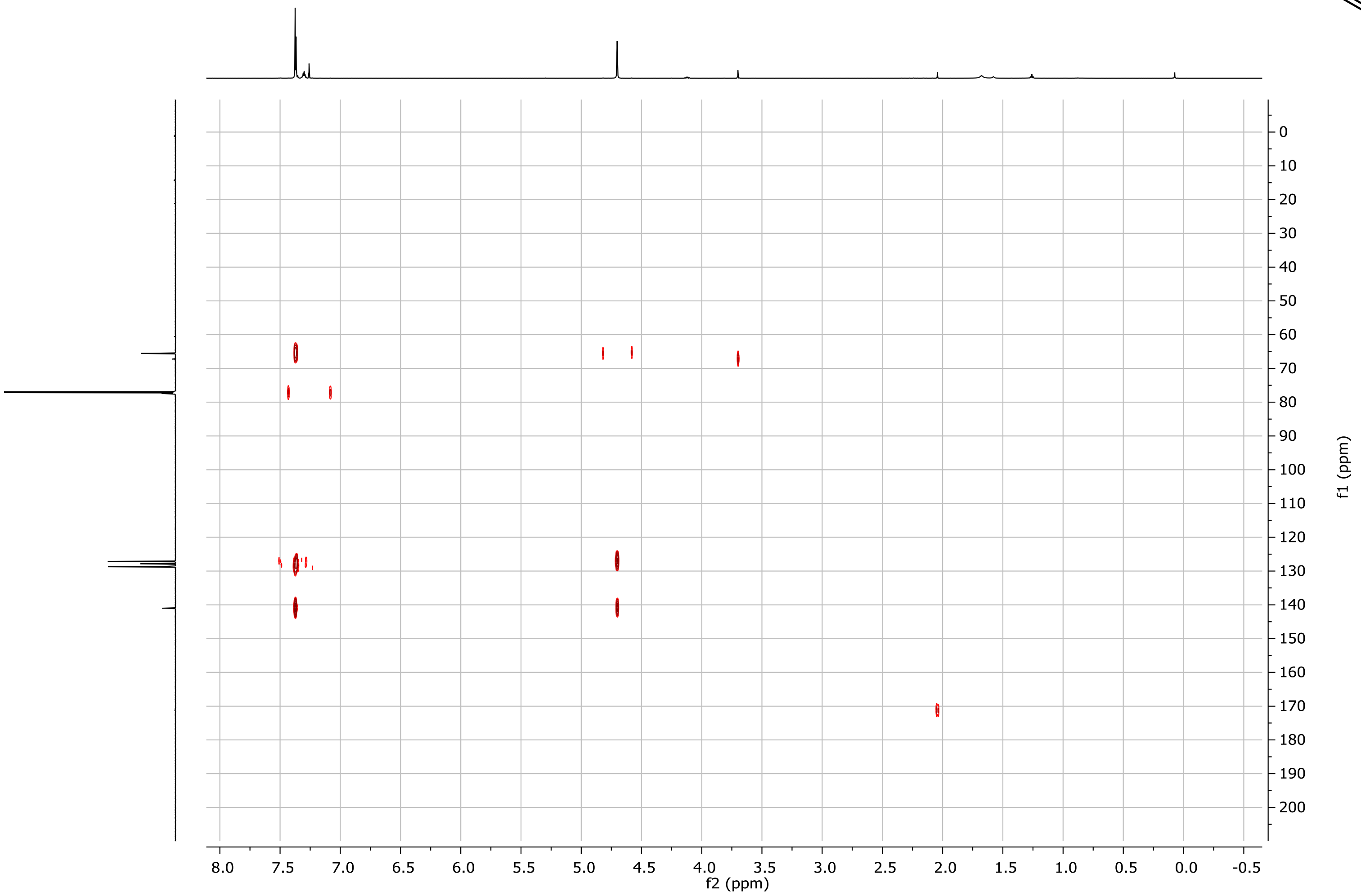

^{1}H, ^{13}C HSQC

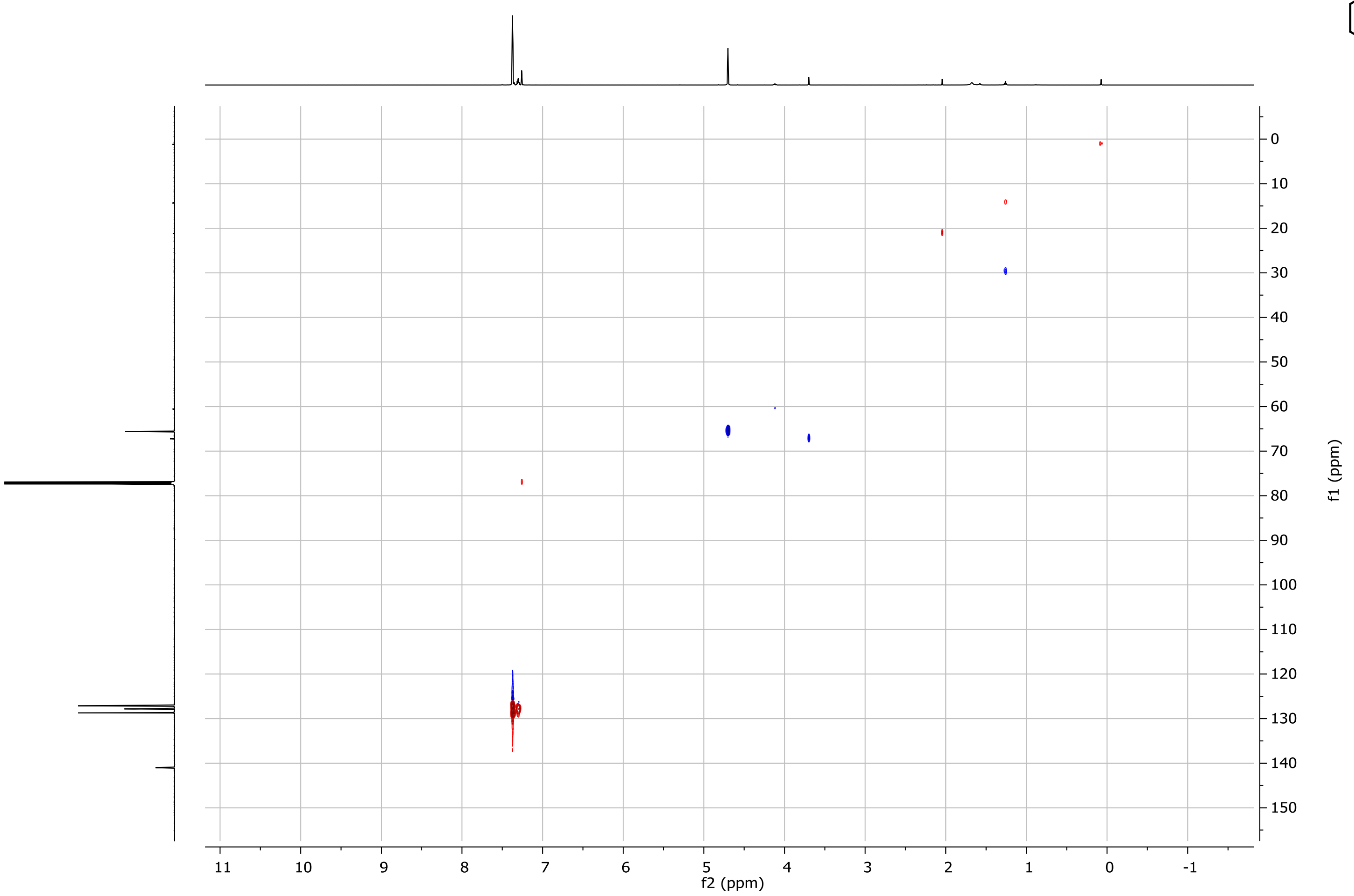

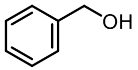

2h

53 Benzyl alcohol from phenyl(4-phenylpiperazin-1-yl)methanone (2i)

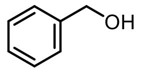

2i

^{1}H NMR

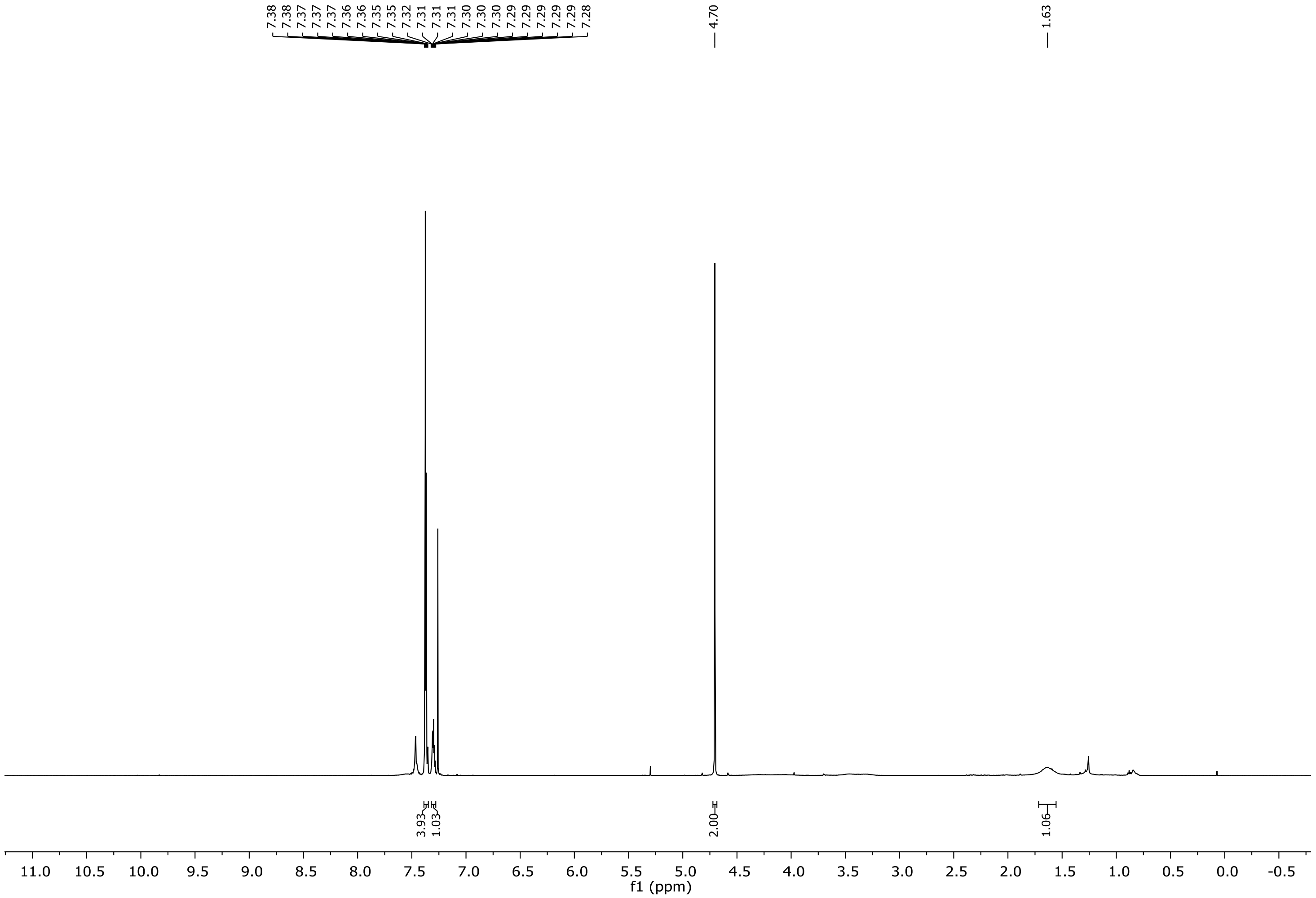

^{13}C NMR

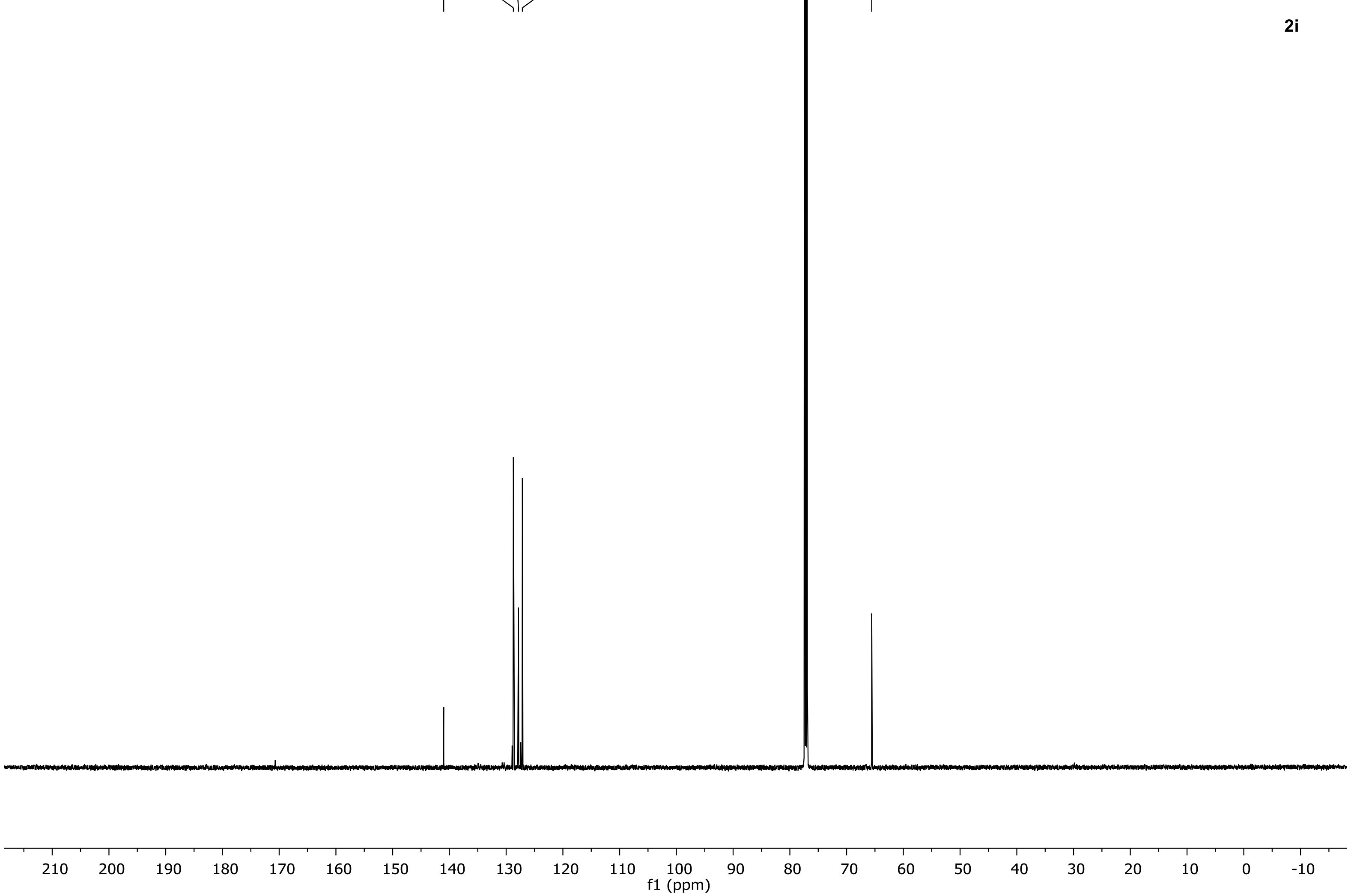

141.0
128.7
127.8
127.1
65.6
210
200
190
180
170
160
150
140
130
120
110
100
90
80
70
60
50
40
30
20
10
0
-10
f1 (ppm)

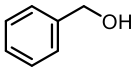

OH

2i

^{1}H, ^{1}H COSY

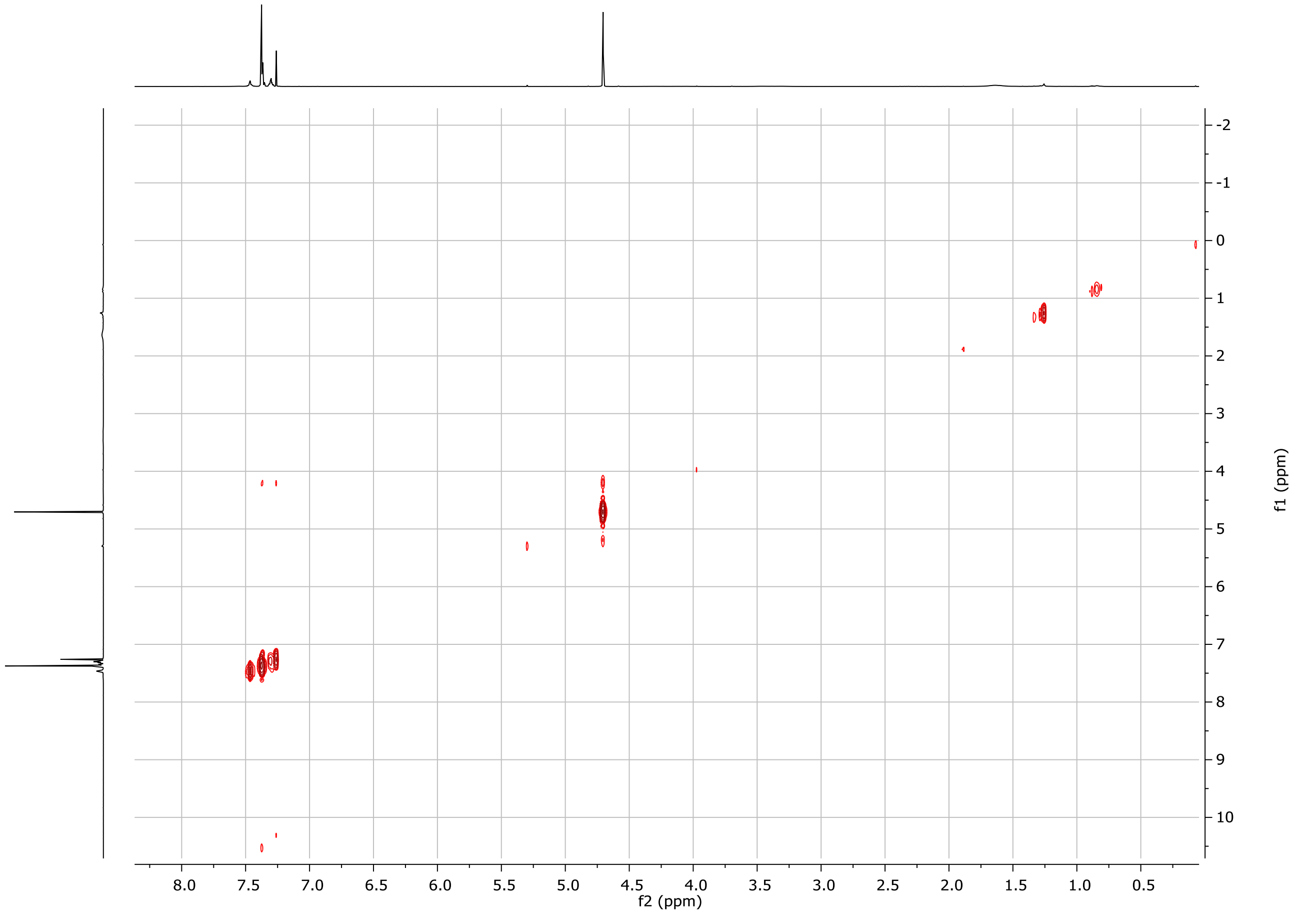

8.0
7.5
7.0
6.5
6.0
5.5
5.0
4.5
4.0
3.5
3.0
2.5
2.0
1.5
1.0
0.5
f2 (ppm)
-2
-1
0
1
2
3
4
5
6
7
8
9
10
f1 (ppm)

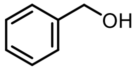

OH

2i

^{1}H, ^{13}C HMBC

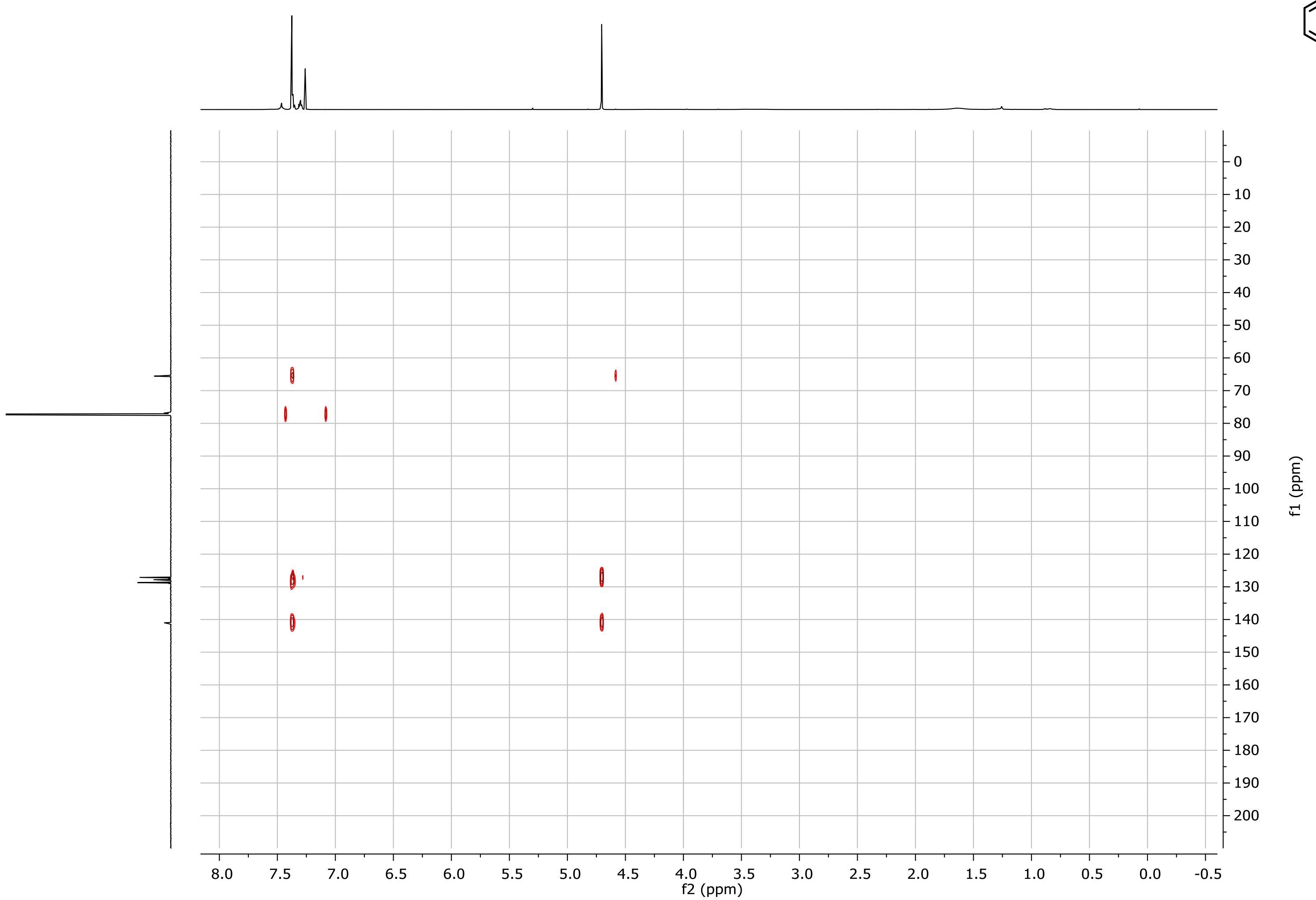

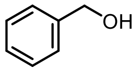

2i

^{1}H, ^{13}C HSQC

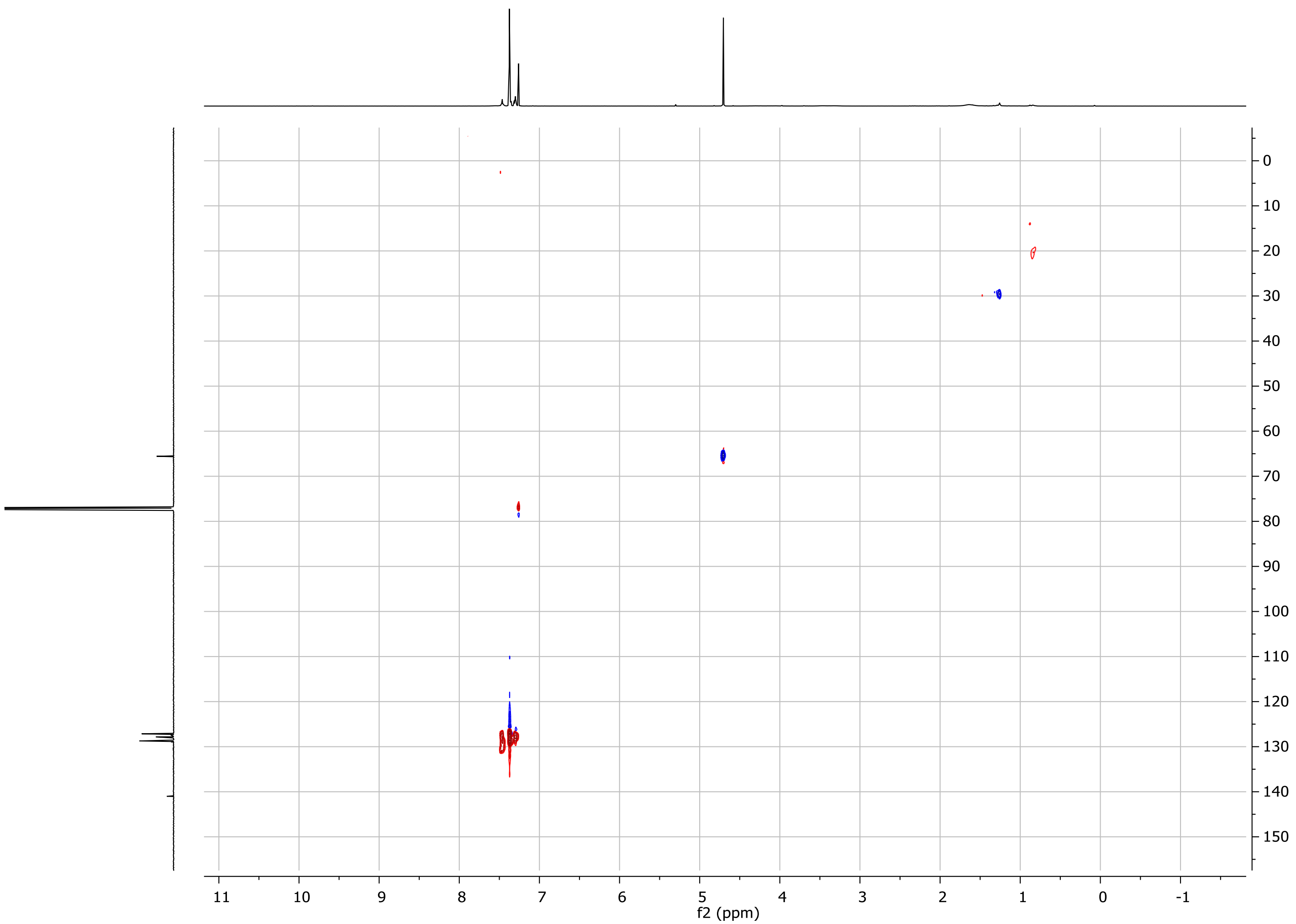

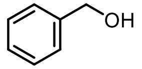

2i

1-Phenylpiperazine (8i)

^{1}H NMR

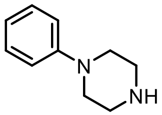

8i

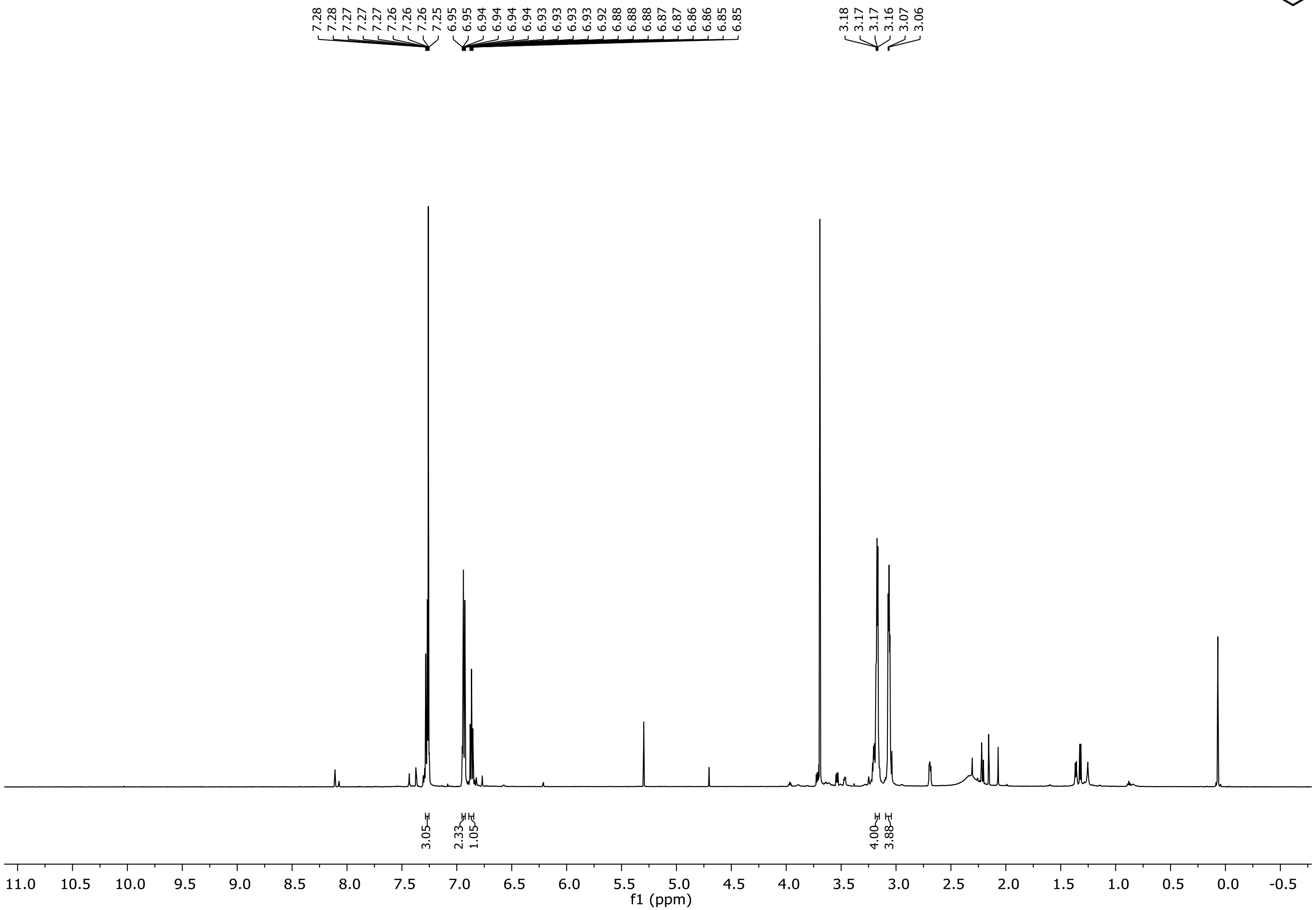

^{13}C NMR

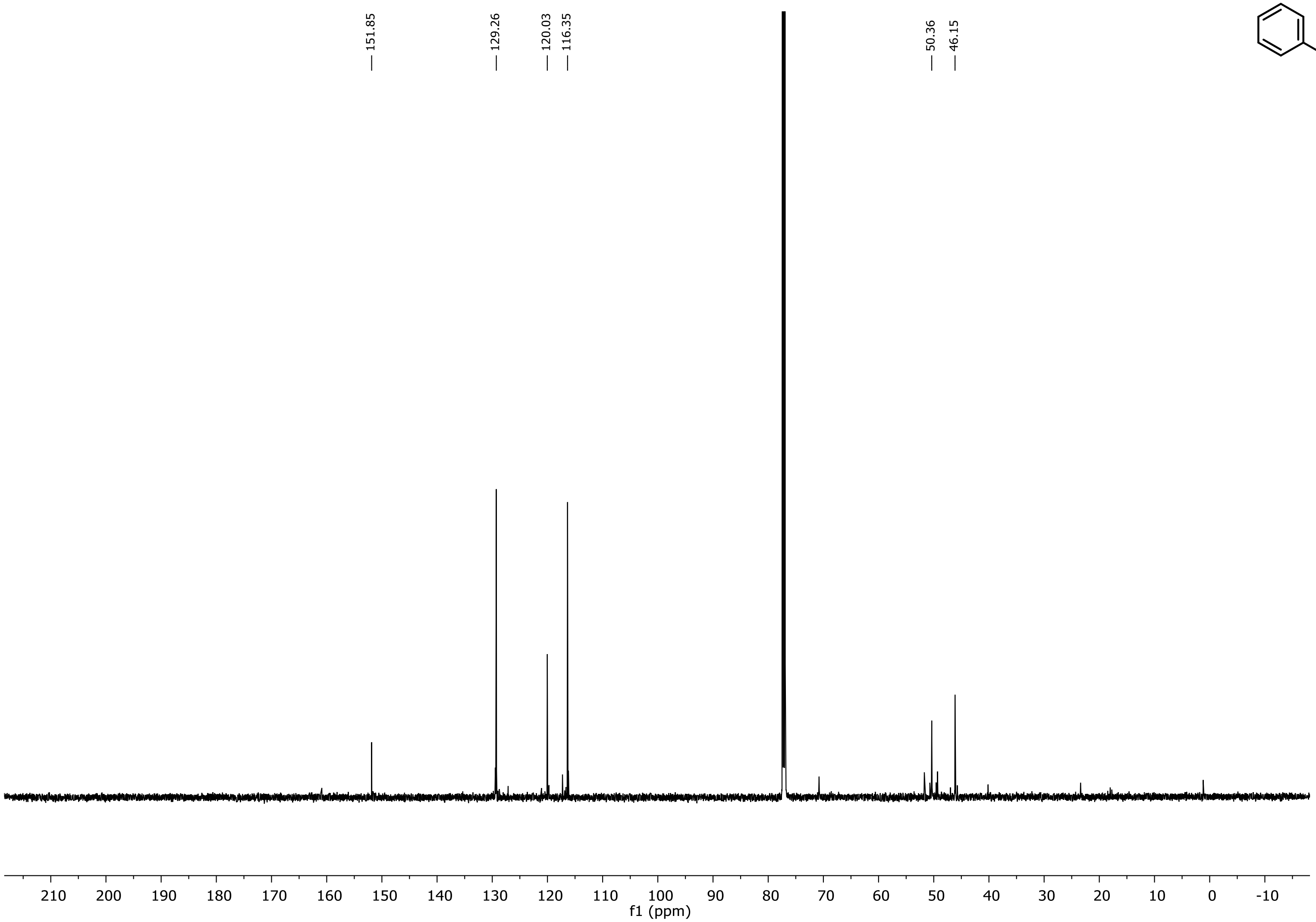
151.85
129.26
120.03
116.35
50.36
46.15
210 200 190 180 170 160 150 140 130 120 110 100 90 80 70 60 50 40 30 20 10 0 -10
f1 (ppm)

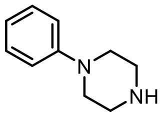
N
NH

8i

^1H, ^1H COSY

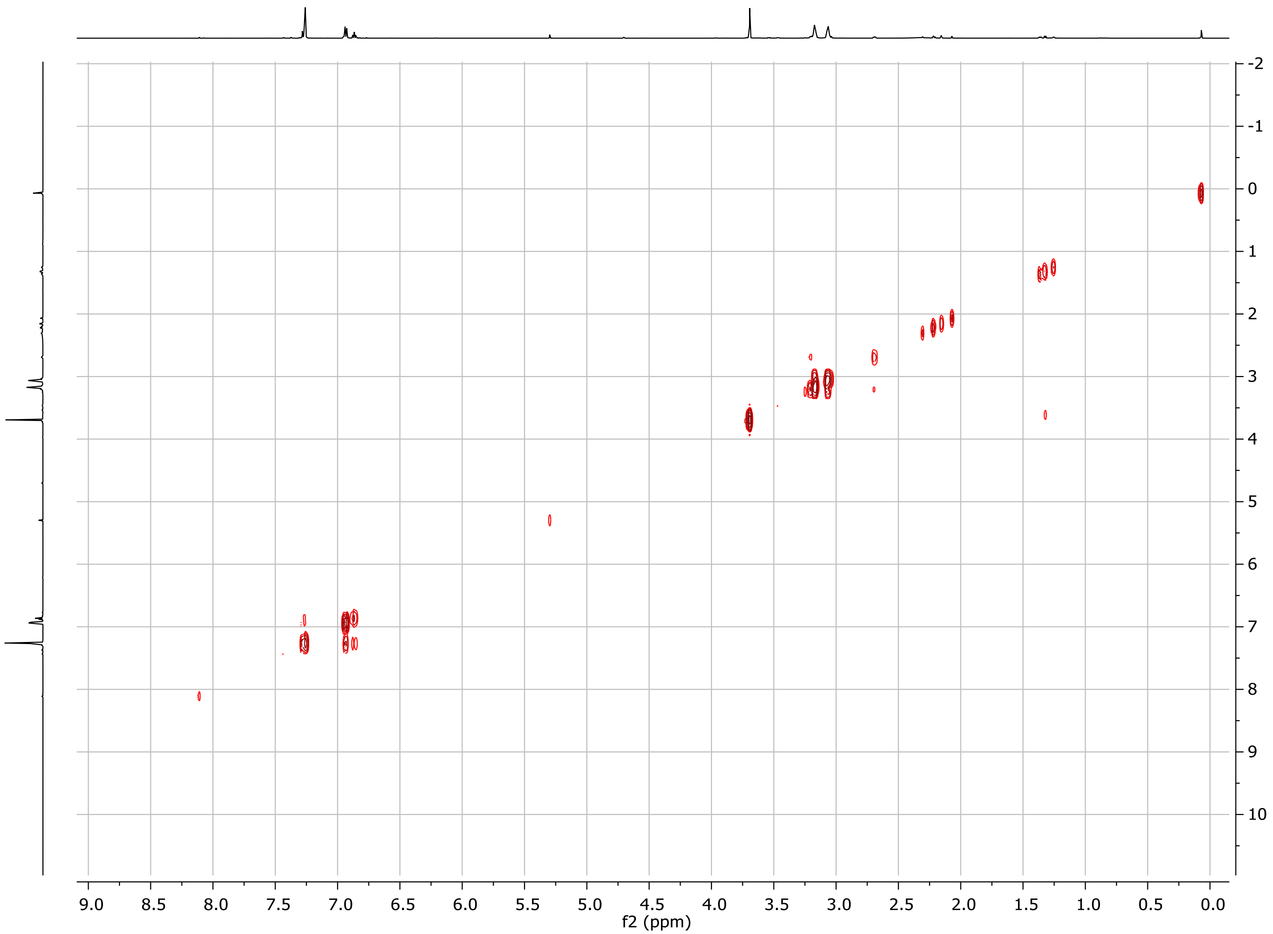

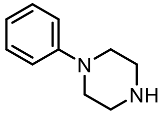

8i

^{1}H, ^{13}C HMBC

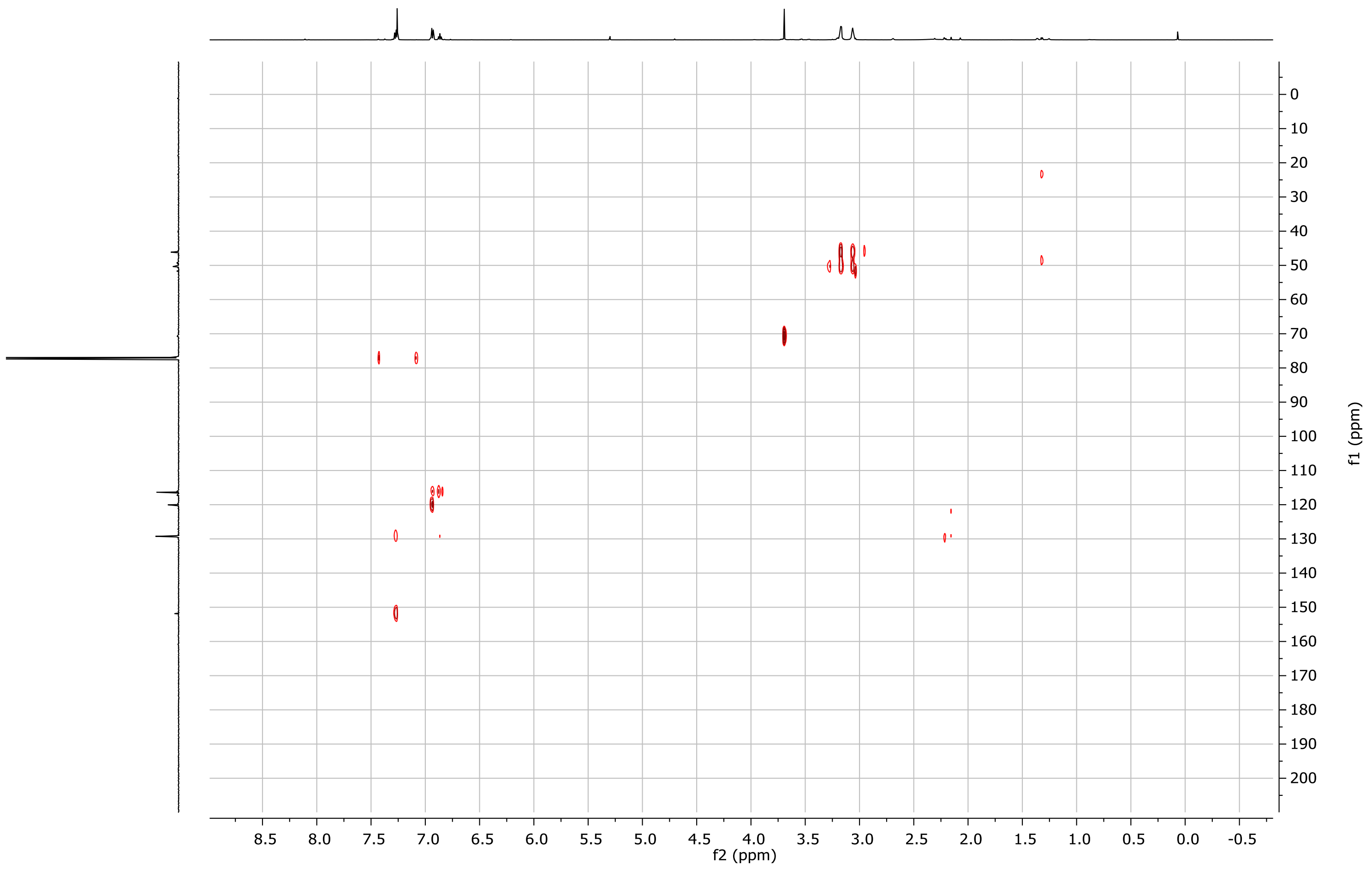

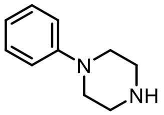

8i

^{1}H, ^{13}C HSQC

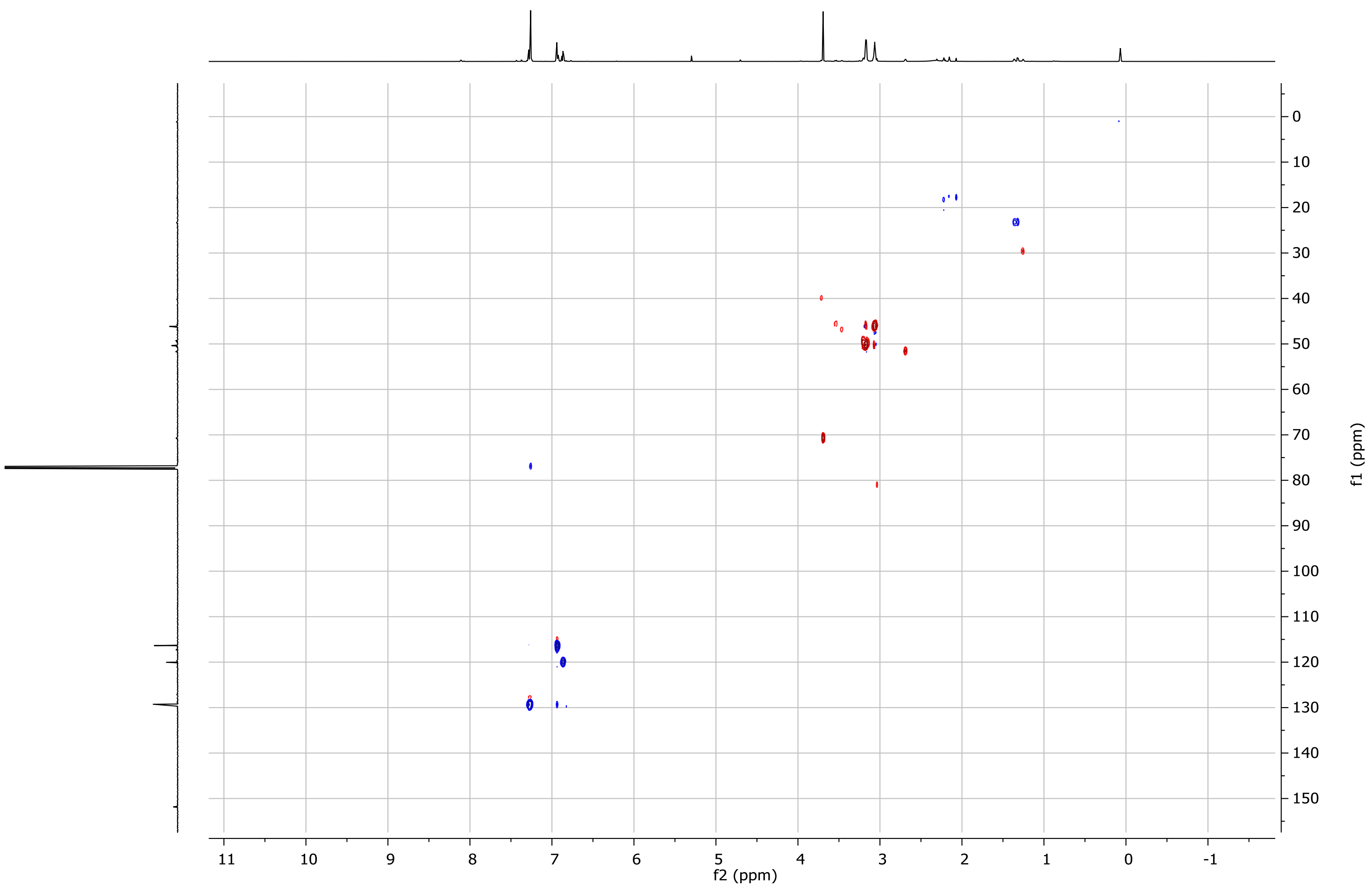

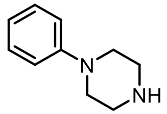

8i

HRMS

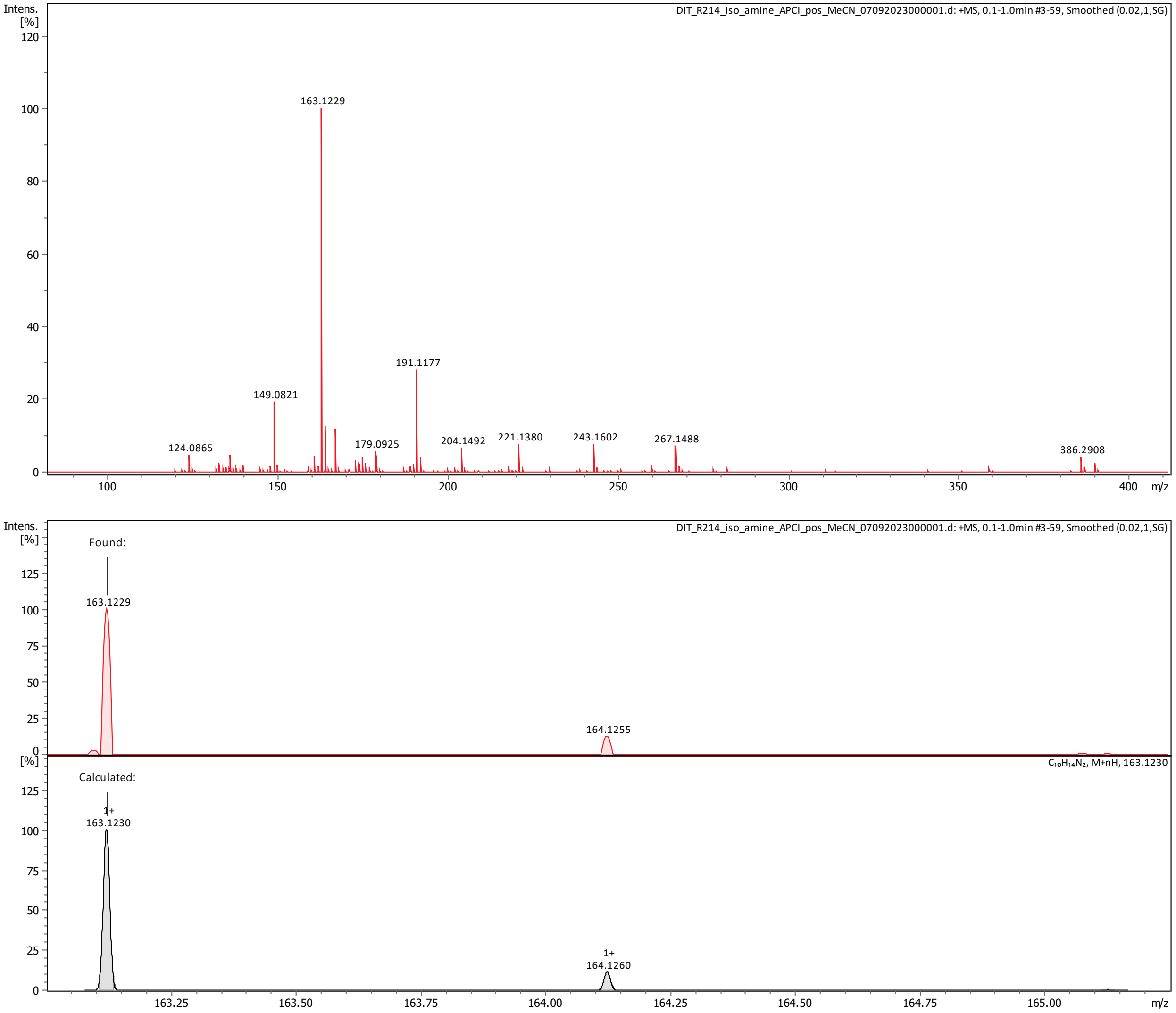

DIT_R214_iso_amine_APCI_pos_MeCN_07092023000001.d: +MS, 0.1-1.0min #3-59, Smoothed (0.02,1,SG)
Intens. [%]
163.1229
191.1177
149.0821
124.0865
179.0925
204.1492
221.1380
243.1602
267.1488
386.2908
m/z
Found:
163.1229
164.1255
Calculated:
1+ 163.1230
1+ 164.1260
C10H14N2, M+nH, 163.1230

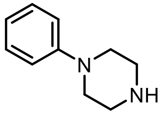

N
NH

8i

54 Benzyl alcohol from morpholino(phenyl)methanone (2j)

^{1}H NMR

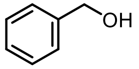

2j

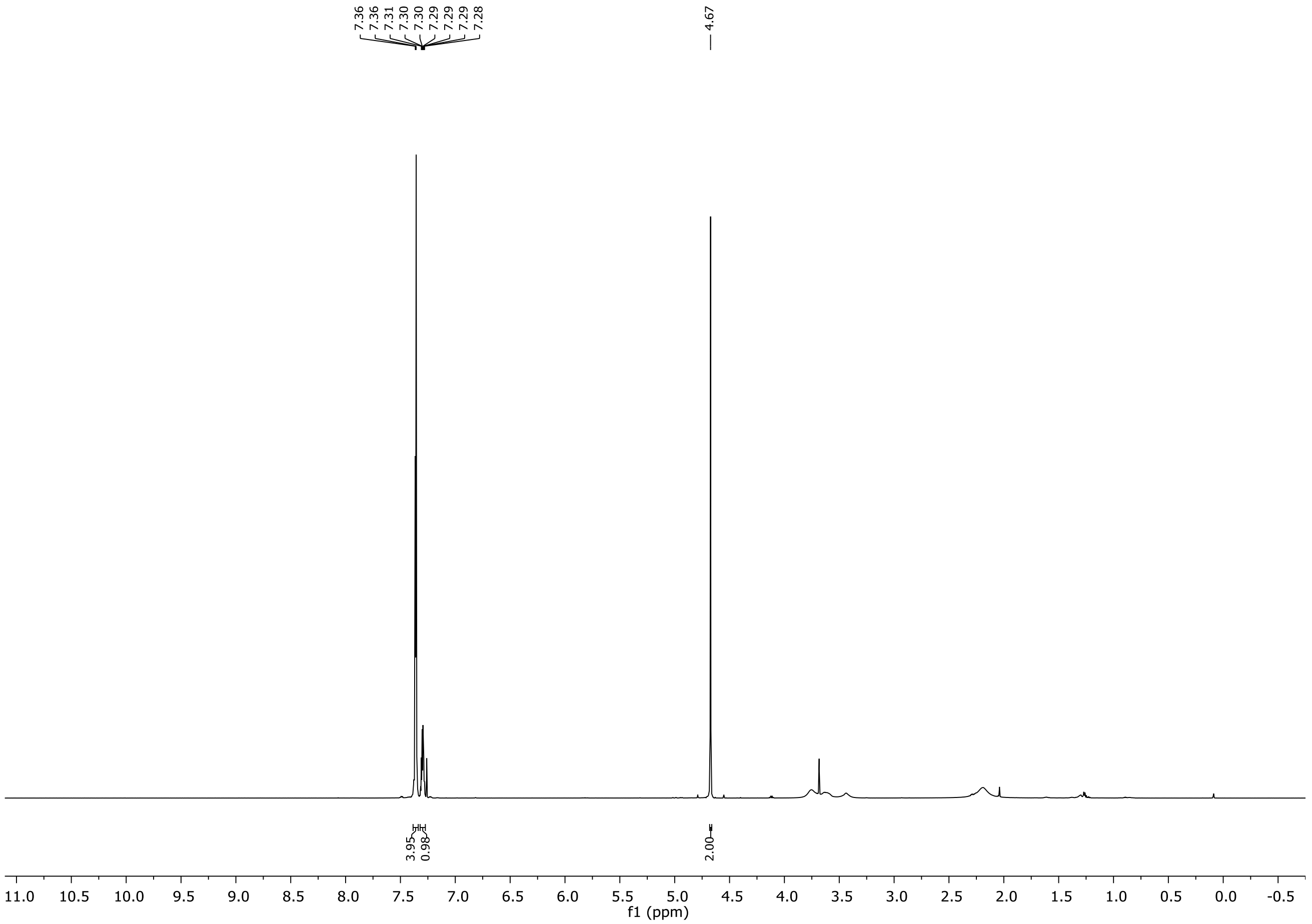

^{13}C NMR

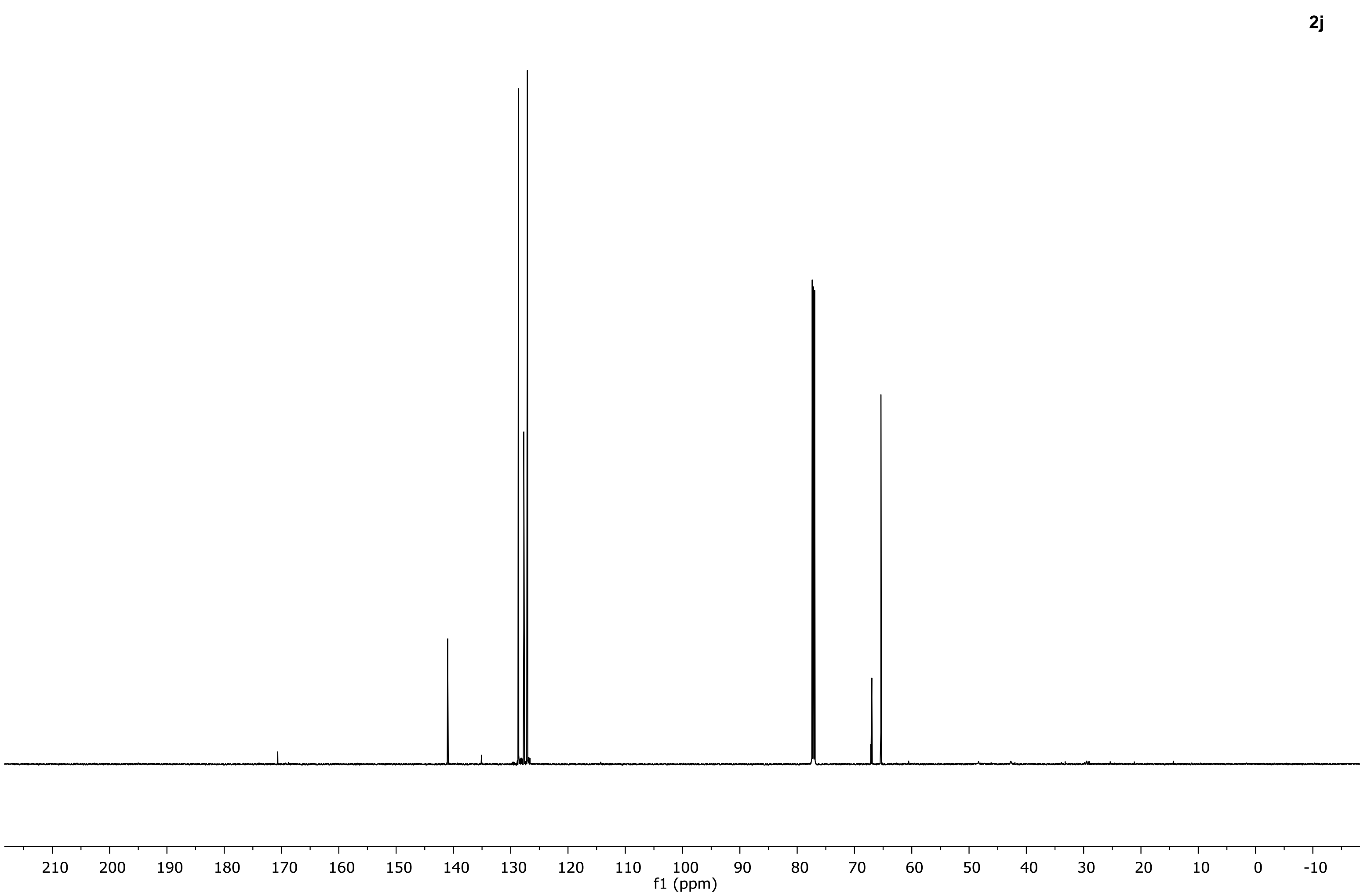

141.0
128.6
127.7
127.1
65.4
210 200 190 180 170 160 150 140 130 120 110 100 90 80 70 60 50 40 30 20 10 0 -10
f1 (ppm)

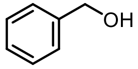

OH

2j

$^1H, ^1H$ COSY

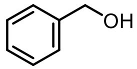

2j

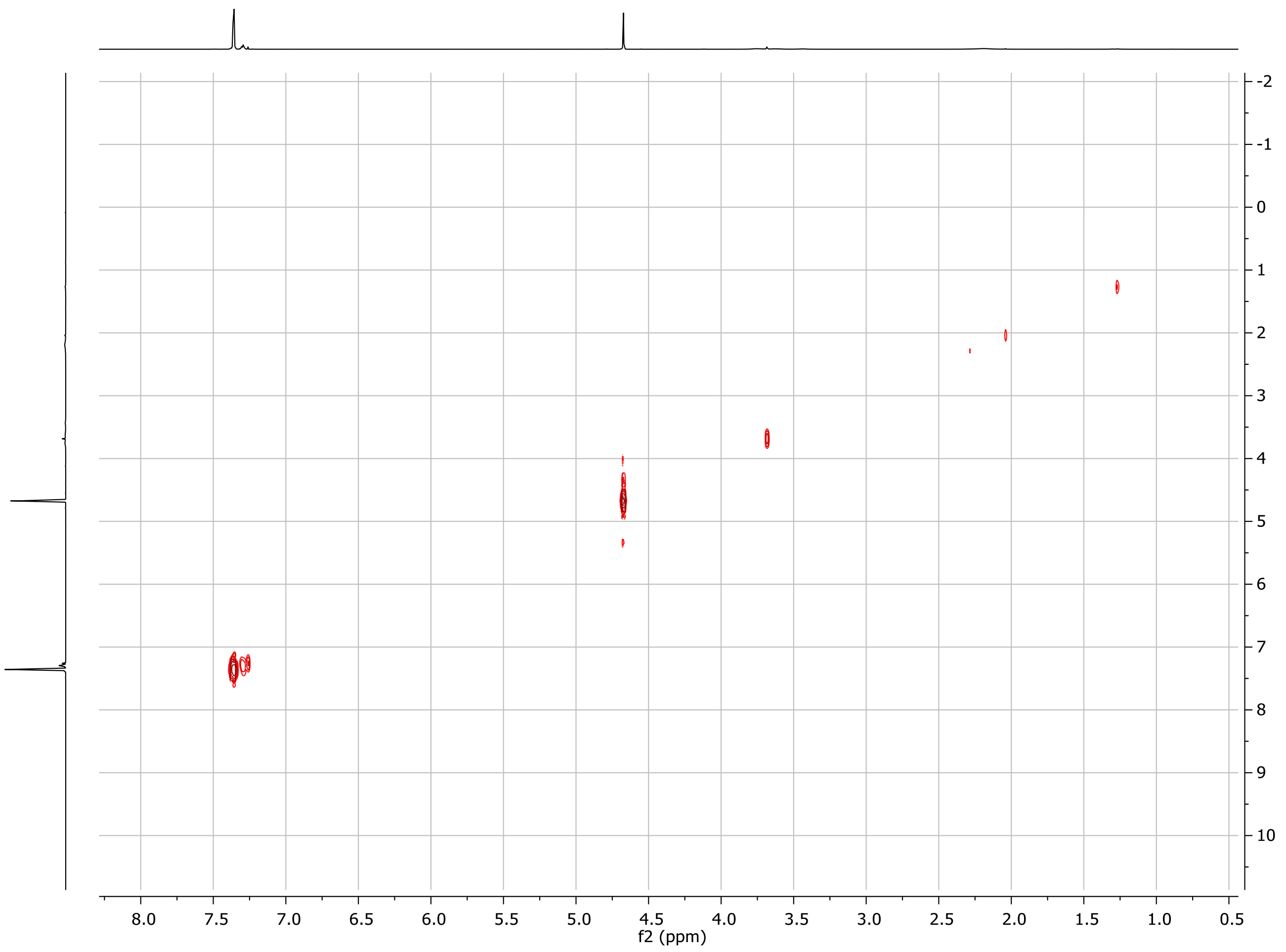

8.0
7.5
7.0
6.5
6.0
5.5
5.0
4.5
4.0
3.5
3.0
2.5
2.0
1.5
1.0
0.5
f2 (ppm)
-2
-1
0
1
2
3
4
5
6
7
8
9
10
f1 (ppm)

^{1}H, ^{13}C HMBC

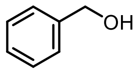

2j

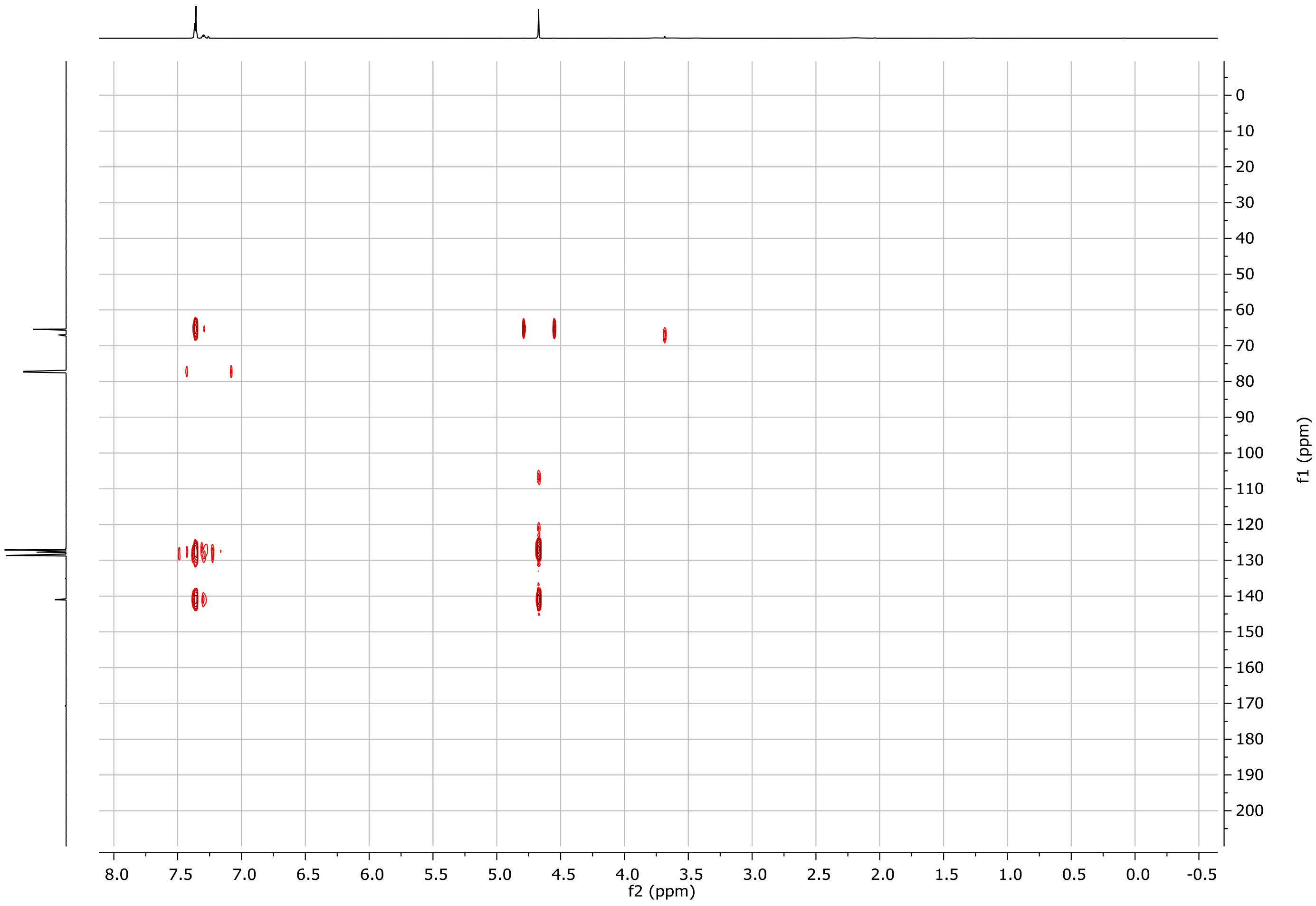

^{1}H, ^{13}C HSQC

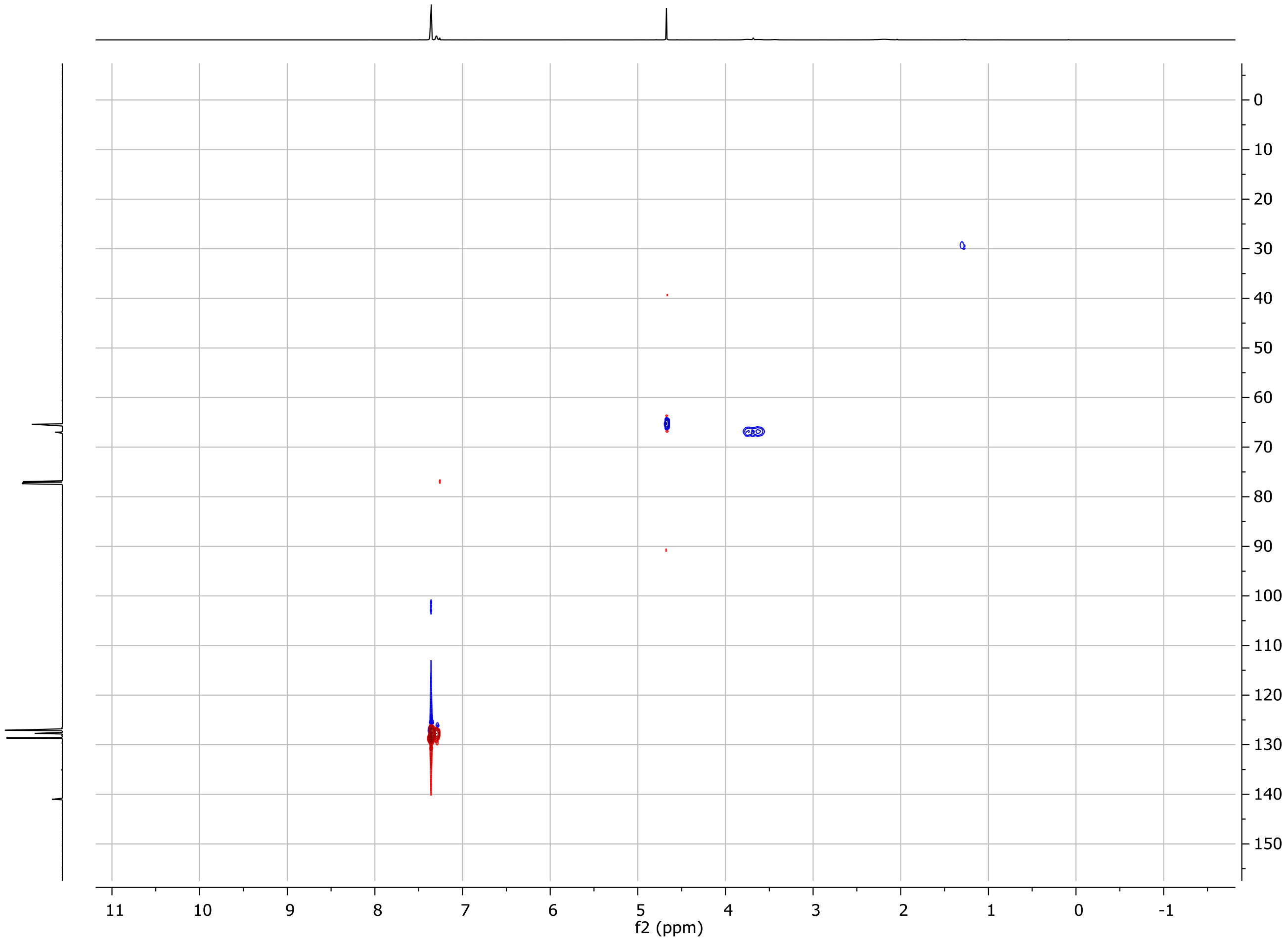

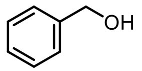

2j

HRMS

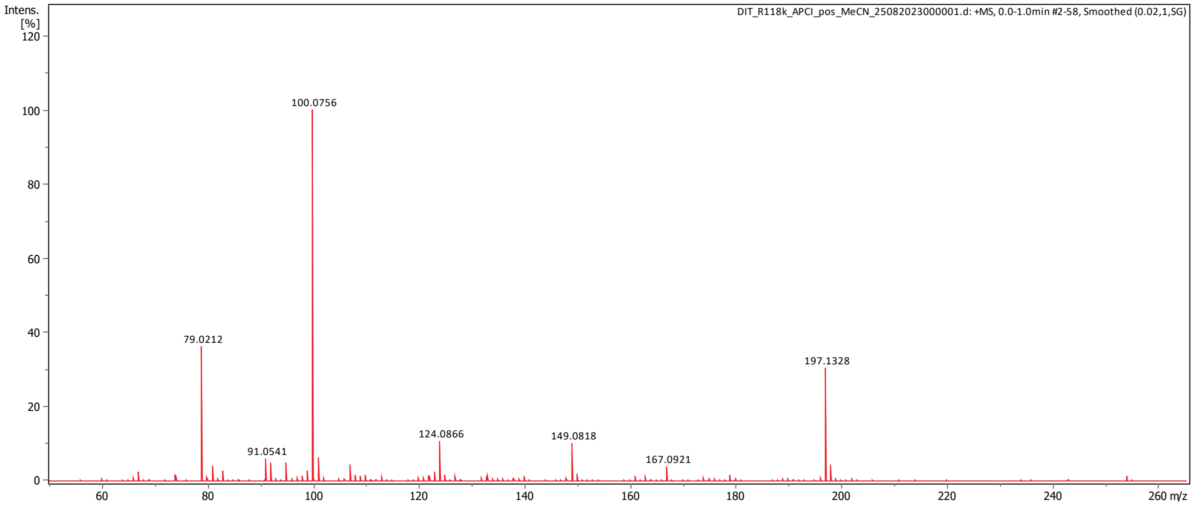

Intens. [%]
DIT_R118k_APCI_pos_MeCN_25082023000001.d: +MS, 0.0-1.0min #2-58, Smoothed (0.02,1,SG)
100.0756
79.0212
91.0541
124.0866
149.0818
167.0921
197.1328
m/z

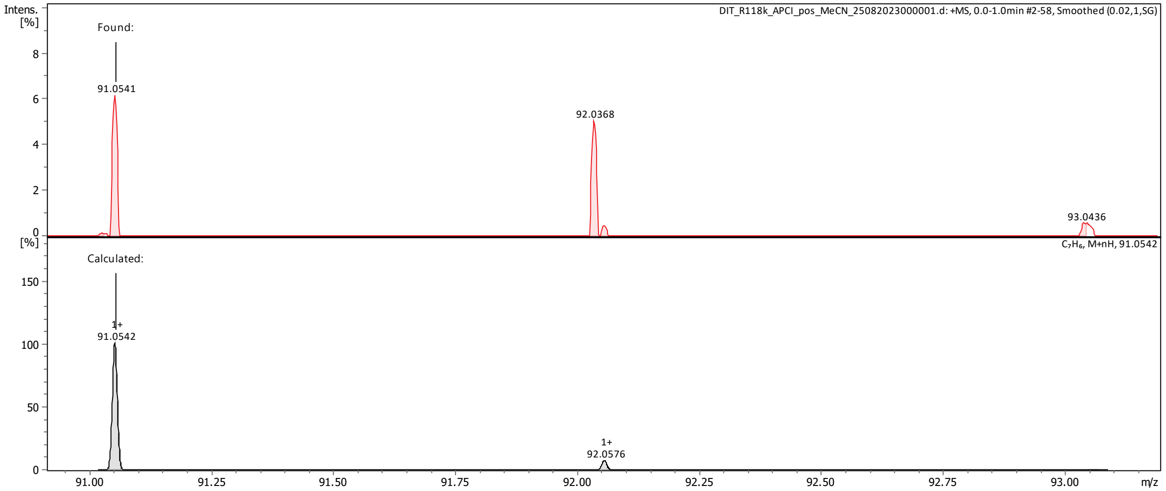

Intens. [%]
DIT_R118k_APCI_pos_MeCN_25082023000001.d: +MS, 0.0-1.0min #2-58, Smoothed (0.02,1,SG)
Found:
91.0541
92.0368
93.0436
C7H6, M+nH, 91.0542
Calculated:
1+
91.0542
1+
92.0576
m/z

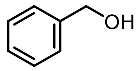

2j

55 *p*-Tolylmethanol (2m)

^{1}H NMR

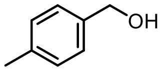

2m

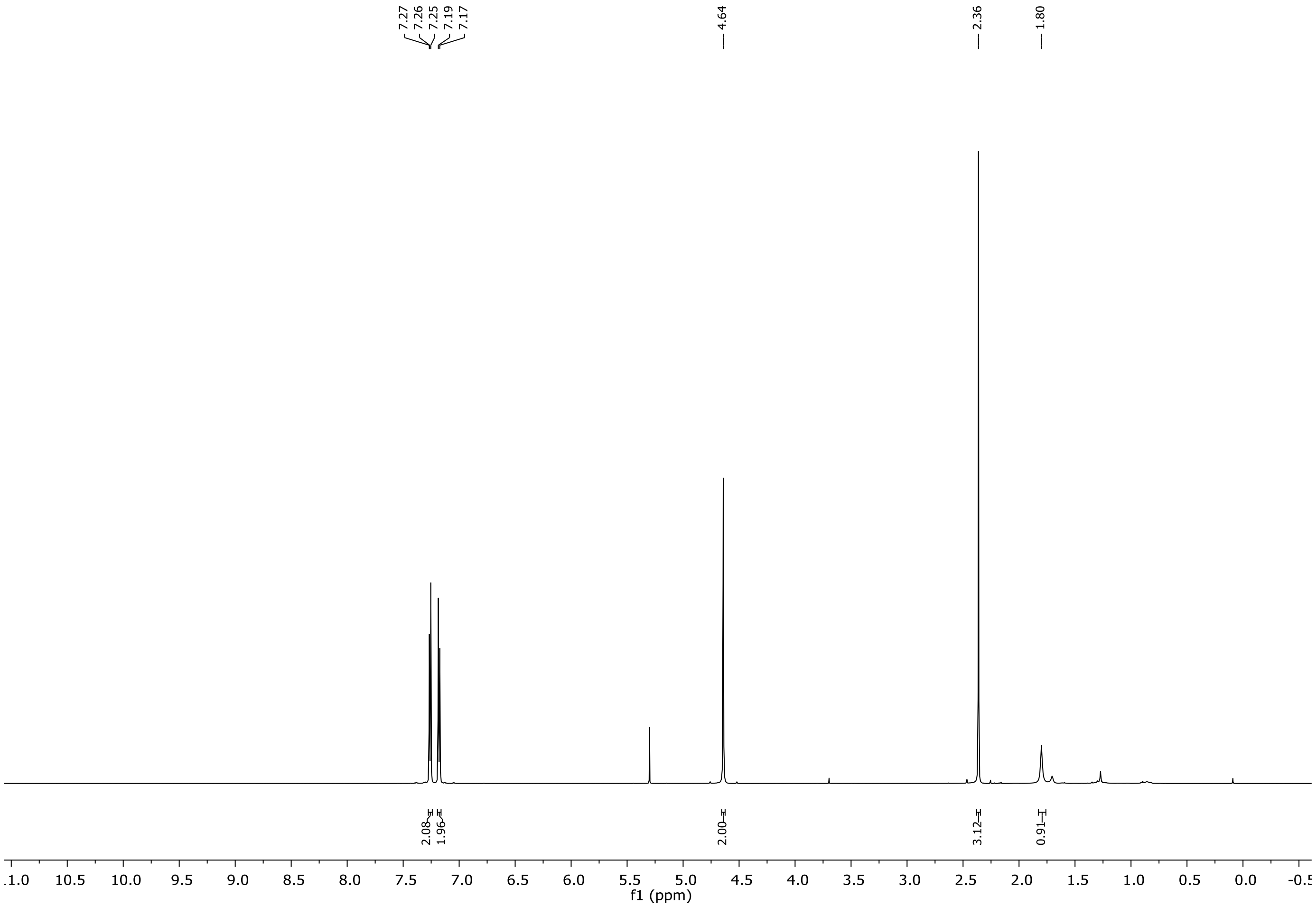

^{13}C NMR

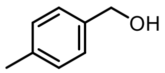

OH

2m

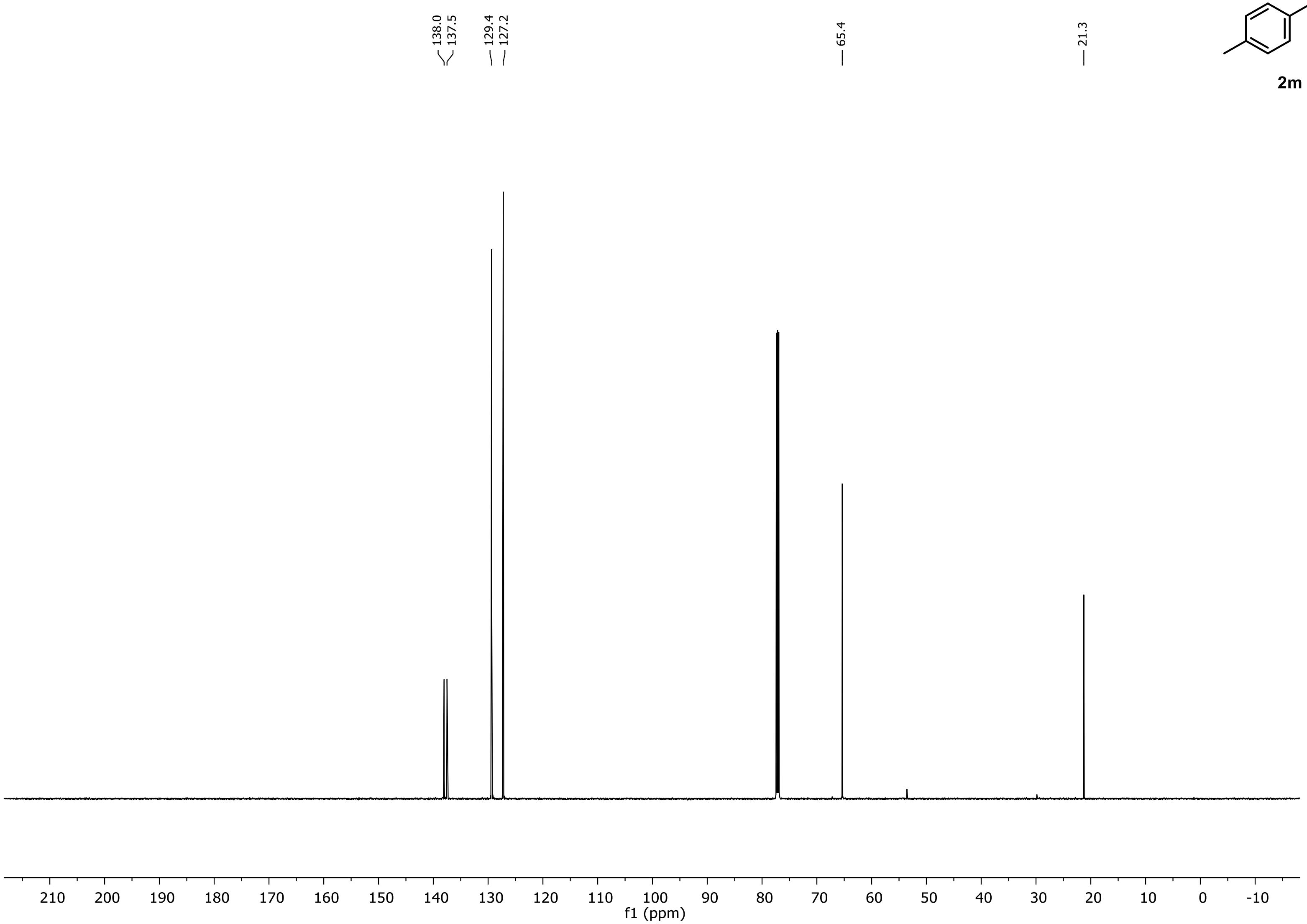

138.0
137.5
129.4
127.2
65.4
21.3
210 200 190 180 170 160 150 140 130 120 110 100 90 80 70 60 50 40 30 20 10 0 -10
f1 (ppm)

^1H, ^1H COSY

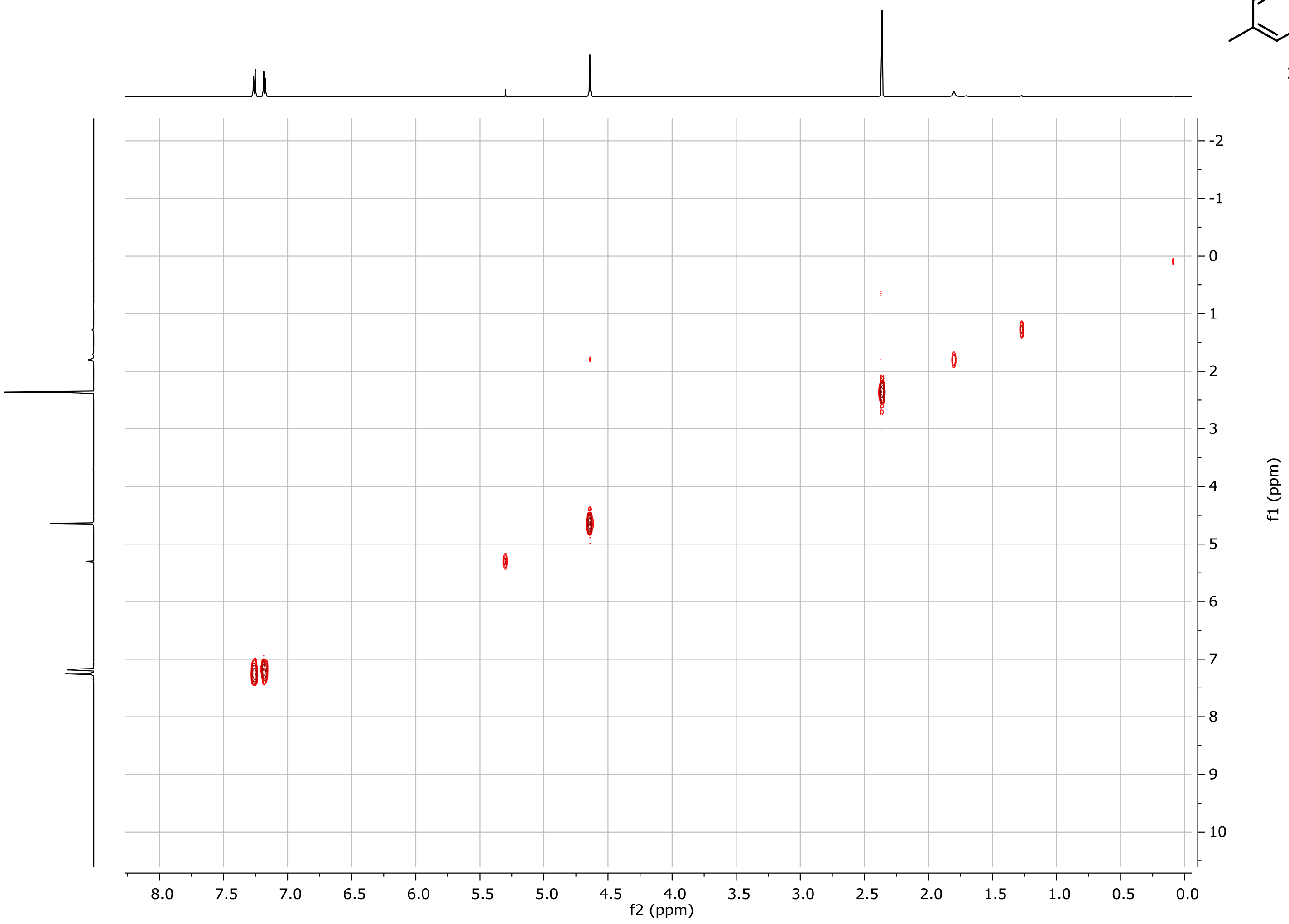

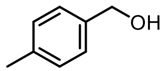

2m

^{1}H, ^{13}C HMBC

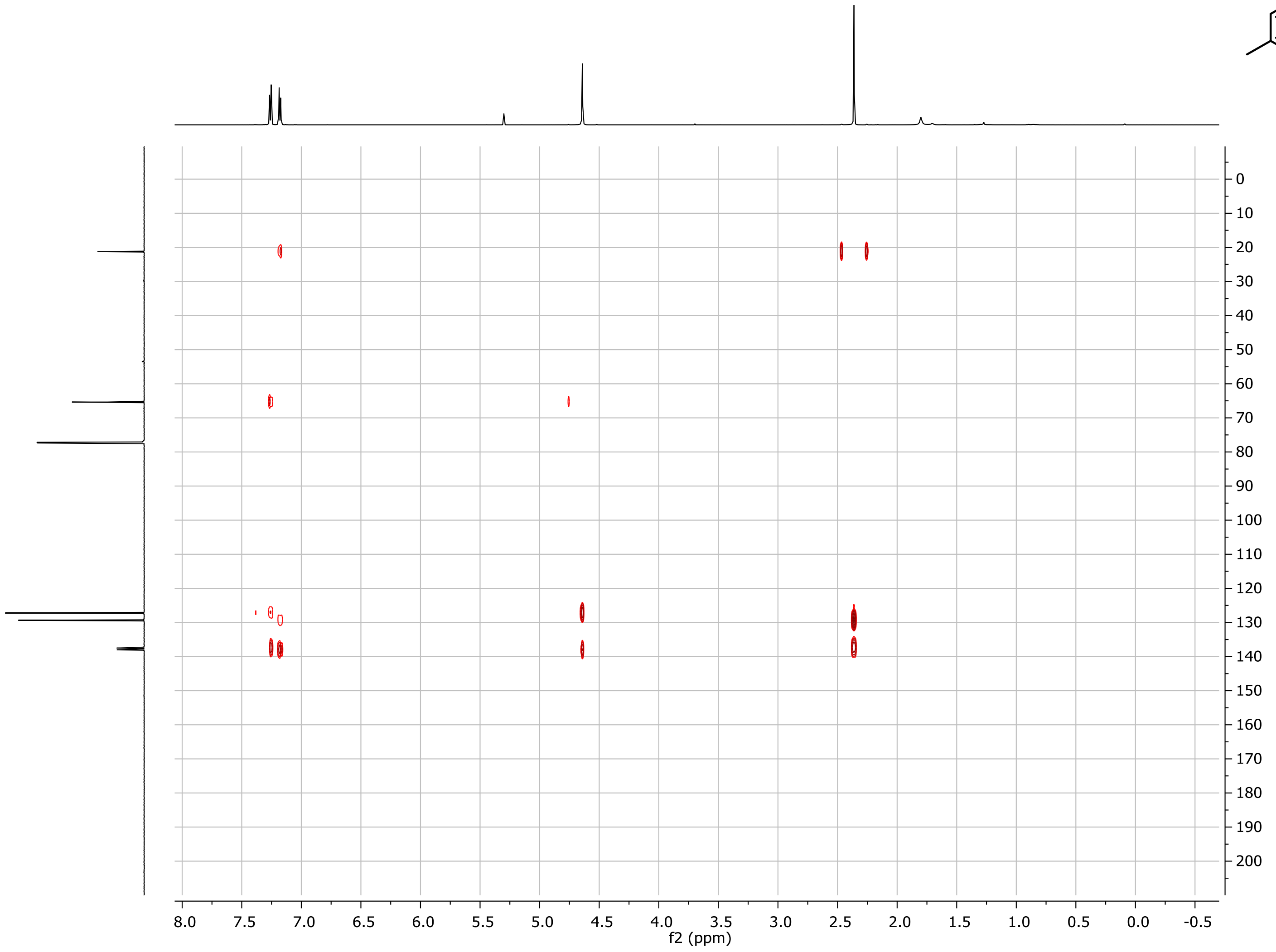

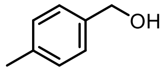

2m

^{1}H, ^{13}C HSQC

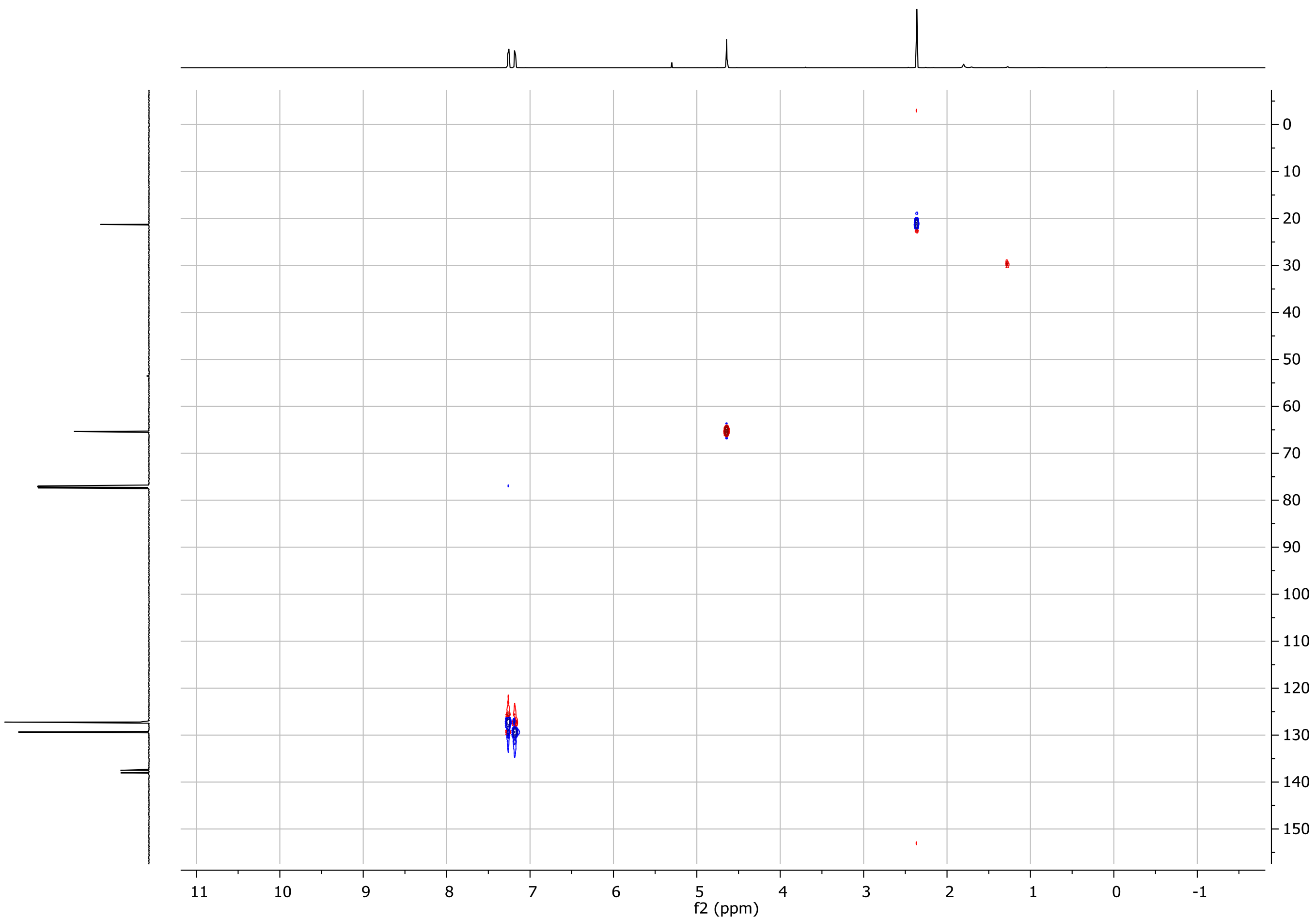

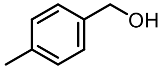

2m

HRMS

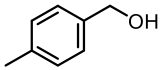

OH

2m

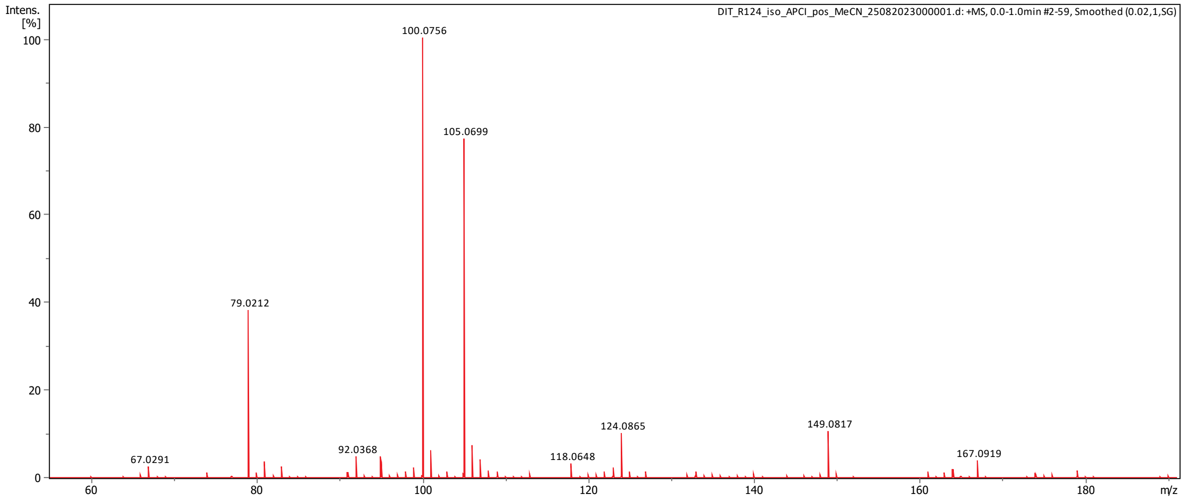

Intens.
[%]
DIT_R124_iso_APCI_pos_MeCN_25082023000001.d: +MS, 0.0-1.0min #2-59, Smoothed (0.02,1,SG)
100.0756
105.0699
79.0212
92.0368
67.0291
118.0648
124.0865
149.0817
167.0919
100
80
60
40
20
0
60
80
100
120
140
160
180
m/z

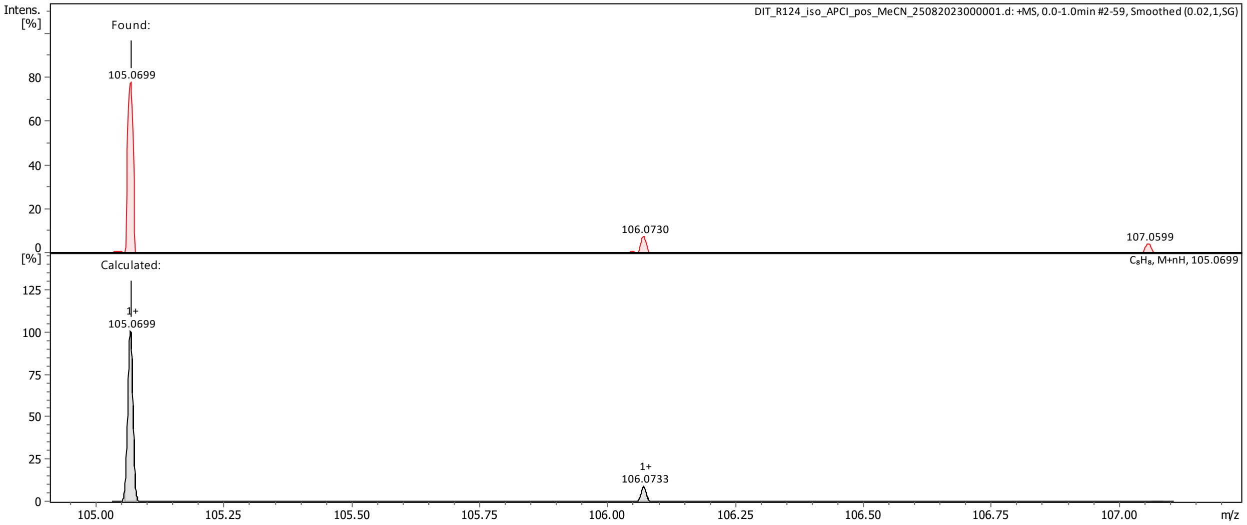

Intens.
[%]
DIT_R124_iso_APCI_pos_MeCN_25082023000001.d: +MS, 0.0-1.0min #2-59, Smoothed (0.02,1,SG)
Found:
105.0699
106.0730
107.0599
80
60
40
20
0
[%]
Calculated:
C_8H_8, M+nH, 105.0699
1+
105.0699
1+
106.0733
125
100
75
50
25
0
105.00
105.25
105.50
105.75
106.00
106.25
106.50
106.75
107.00
m/z

56 Cyclohexylmethanol (2n)

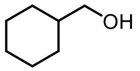

2n

^{1}H NMR

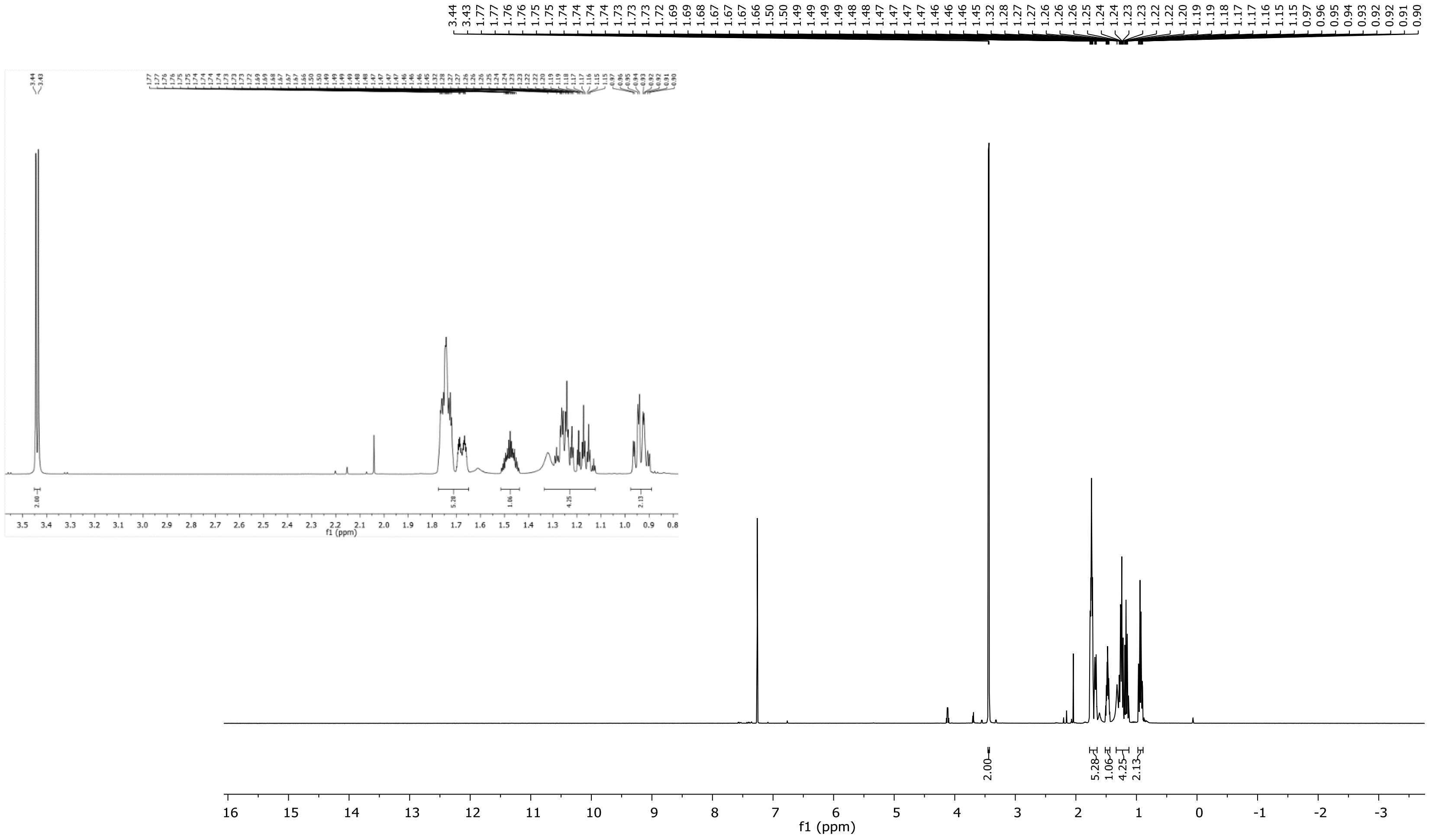

^{13}C NMR

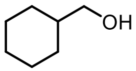
OH

2n

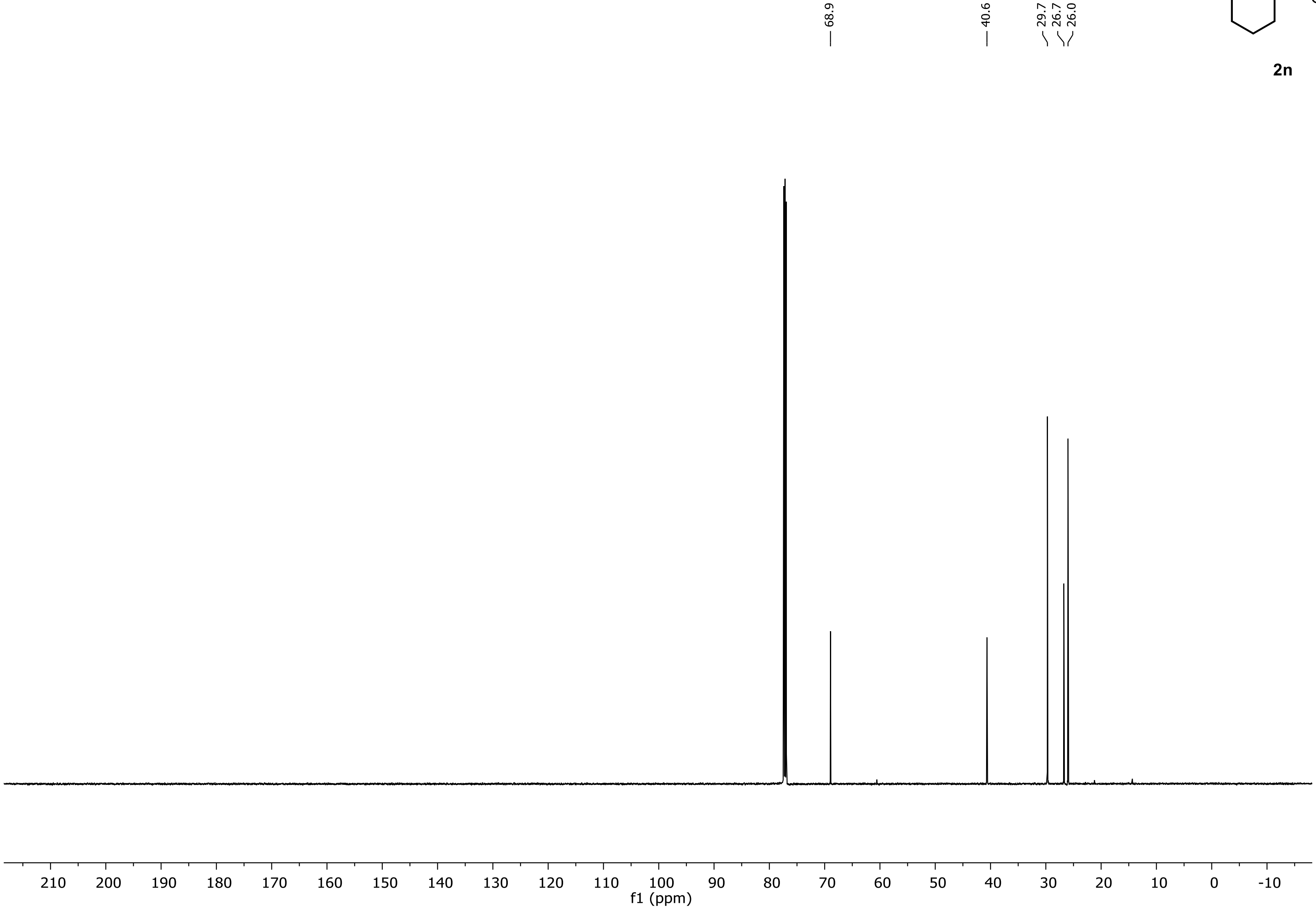
68.9
40.6
29.7
26.7
26.0
210
200
190
180
170
160
150
140
130
120
110
100
90
80
70
60
50
40
30
20
10
0
-10
f1 (ppm)

^{1}H, ^{1}H COSY

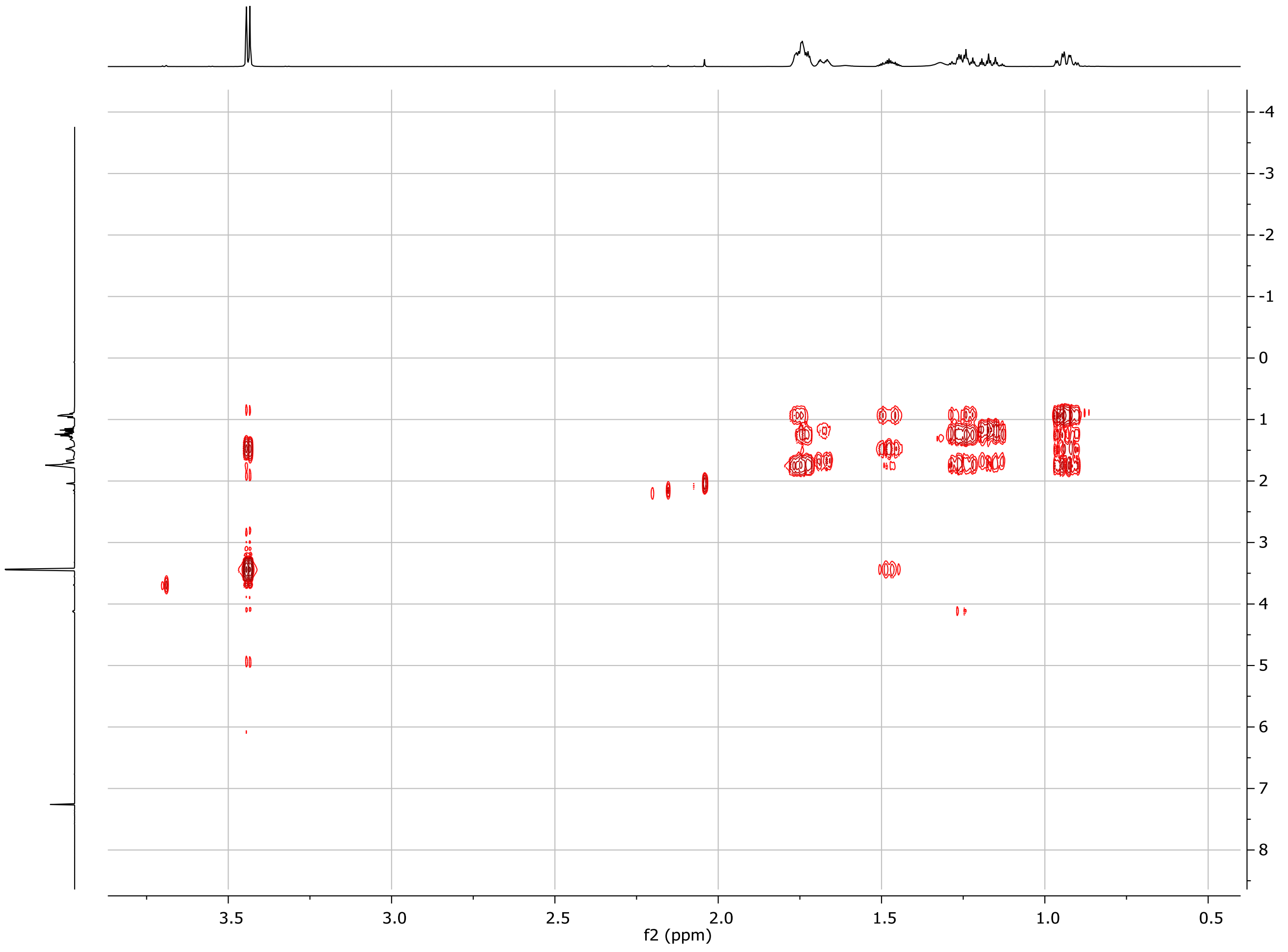

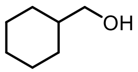

2n

^1H, ^{13}C HMBC

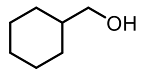

2n

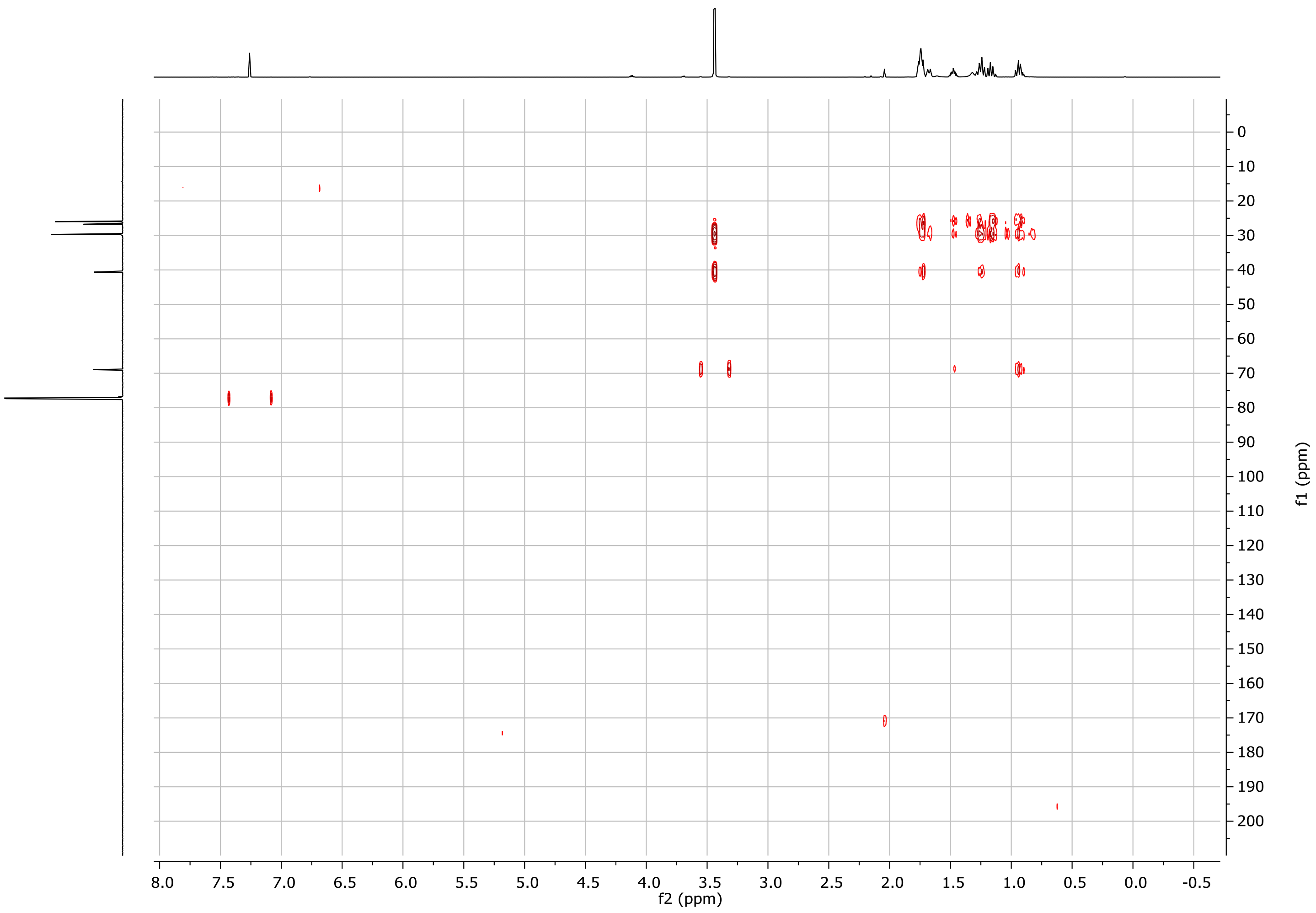

^{1}H, ^{13}C HSQC

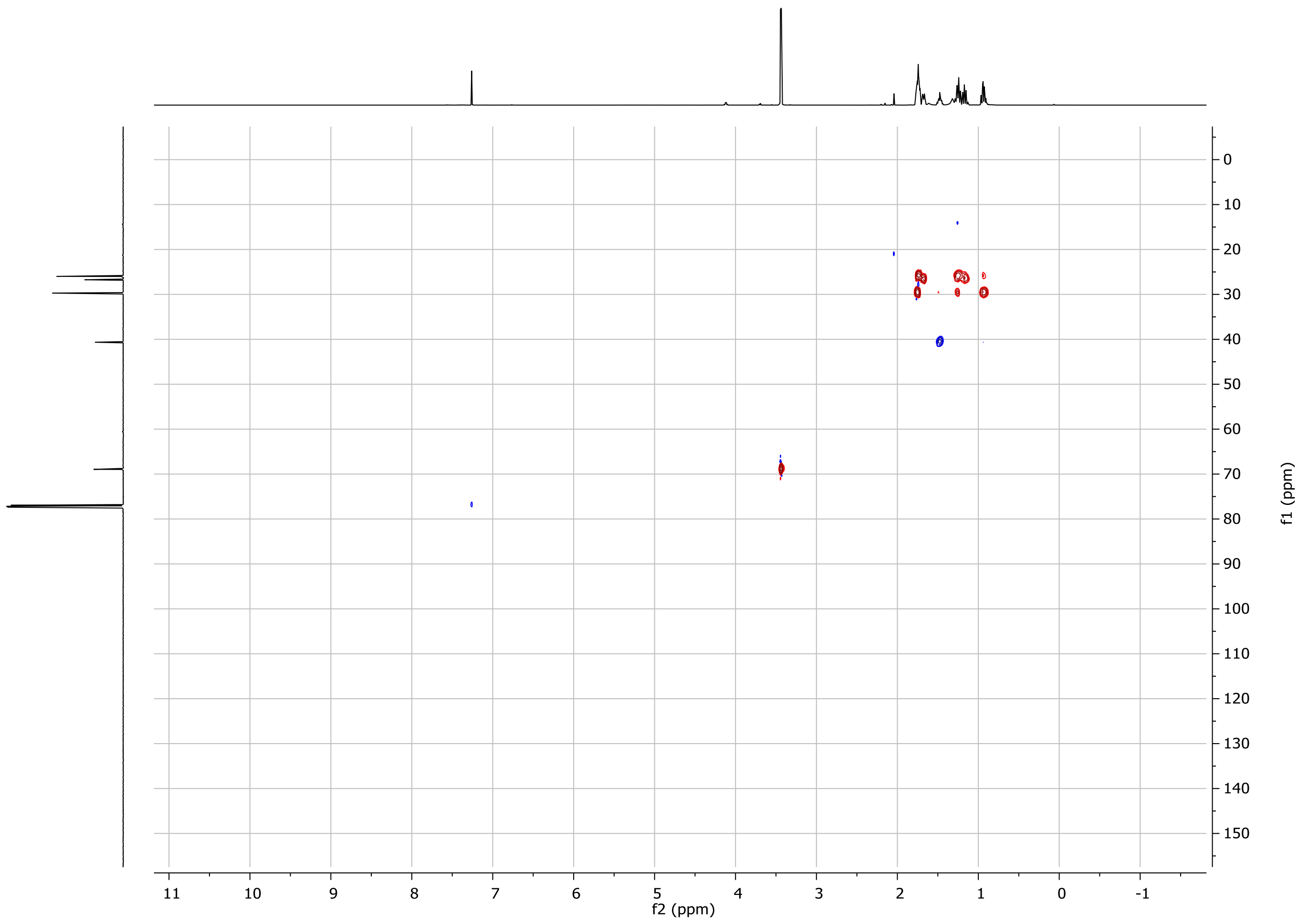

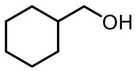

2n

HRMS

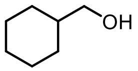

2n

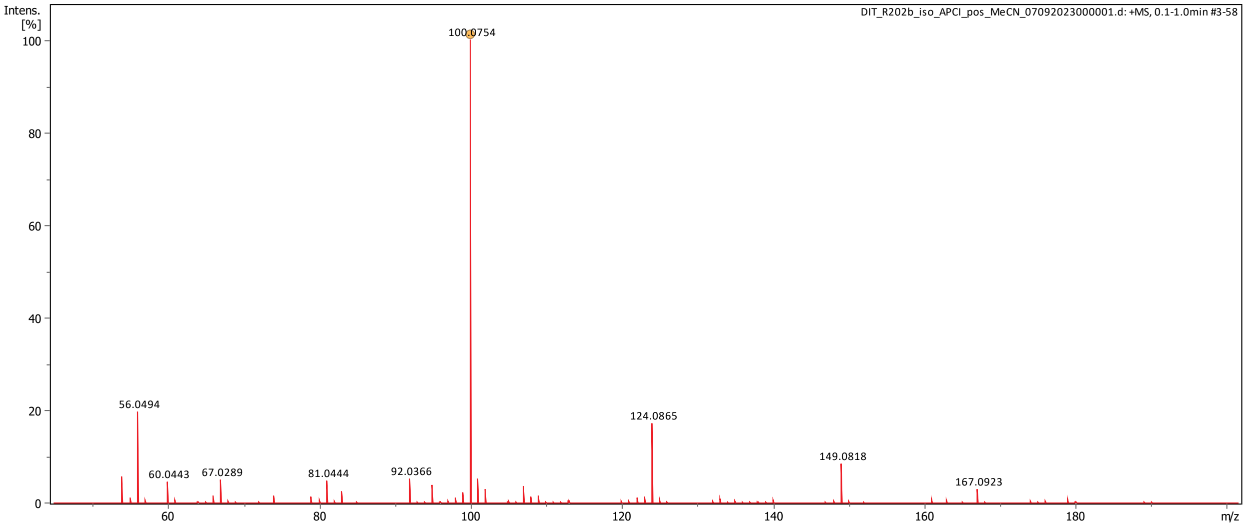
DIT_R202b_iso_APCI_pos_MeCN_07092023000001.d: +MS, 0.1-1.0min #3-58
Intens. [%]
100.0754
56.0494
124.0865
149.0818
167.0923
60.0443
67.0289
81.0444
92.0366
m/z

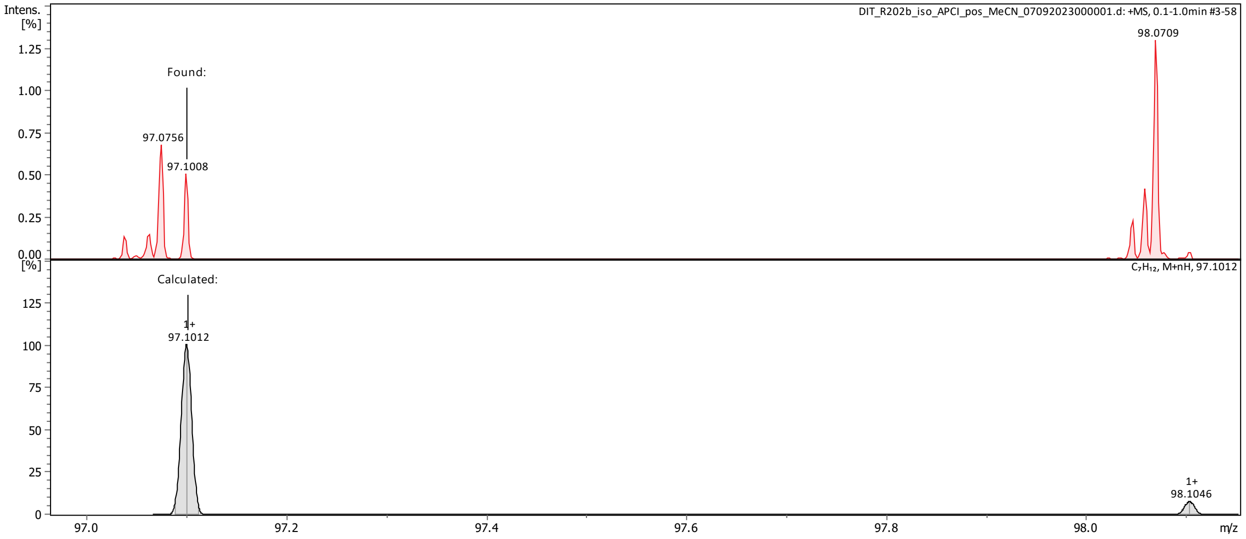
DIT_R202b_iso_APCI_pos_MeCN_07092023000001.d: +MS, 0.1-1.0min #3-58
Intens. [%]
Found:
97.0756
97.1008
98.0709
Calculated:
C_7H_{12}, M+nH, 97.1012
1+
97.1012
1+
98.1046
m/z

57 *m*-Tolylmethanol (2o)

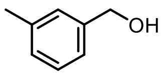

2o

^{1}H NMR

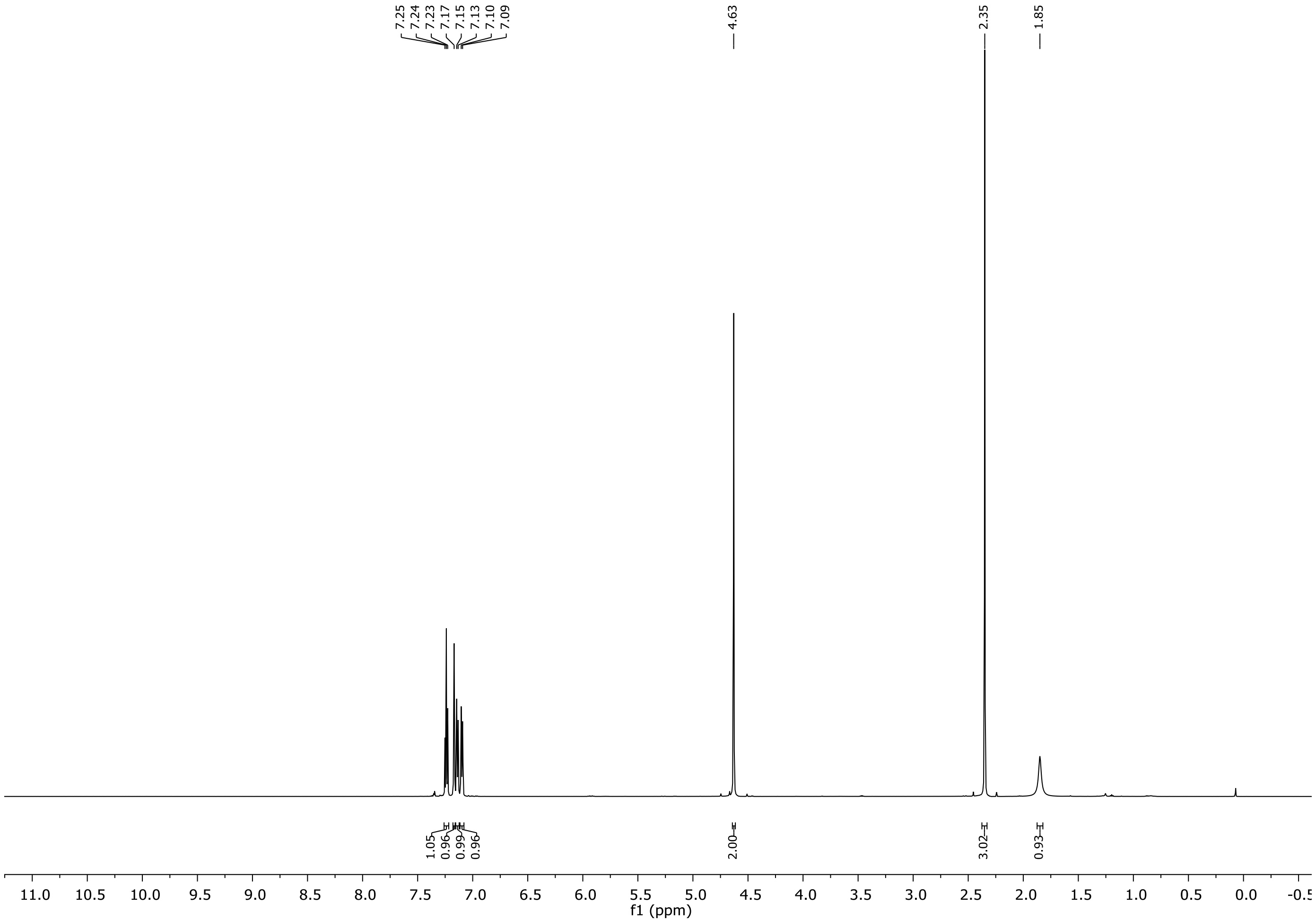

^{13}C NMR

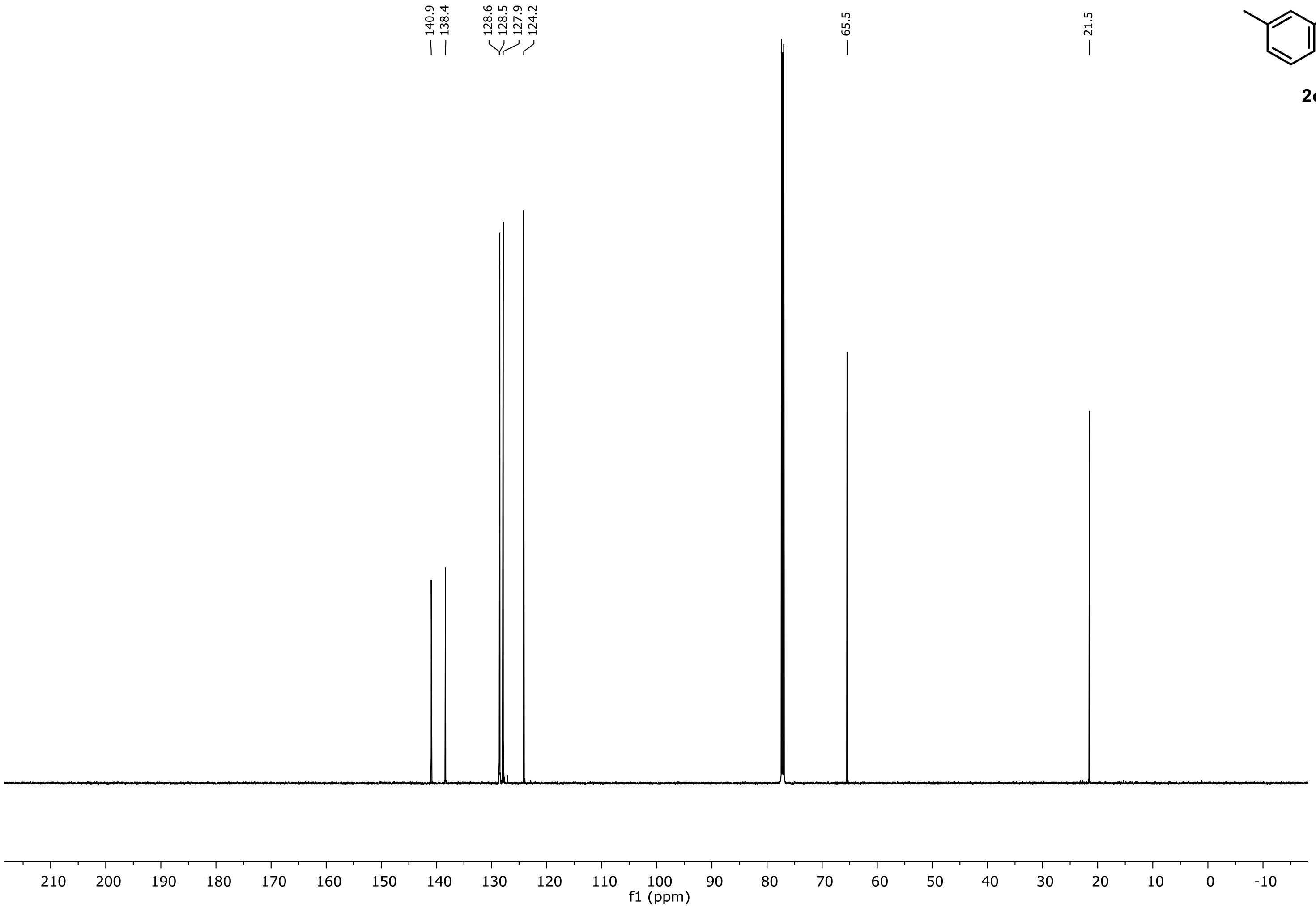
140.9
138.4
128.6
128.5
127.9
124.2
65.5
21.5
210 200 190 180 170 160 150 140 130 120 110 100 90 80 70 60 50 40 30 20 10 0 -10
f1 (ppm)

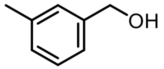
OH

2o

^1H, ^1H COSY

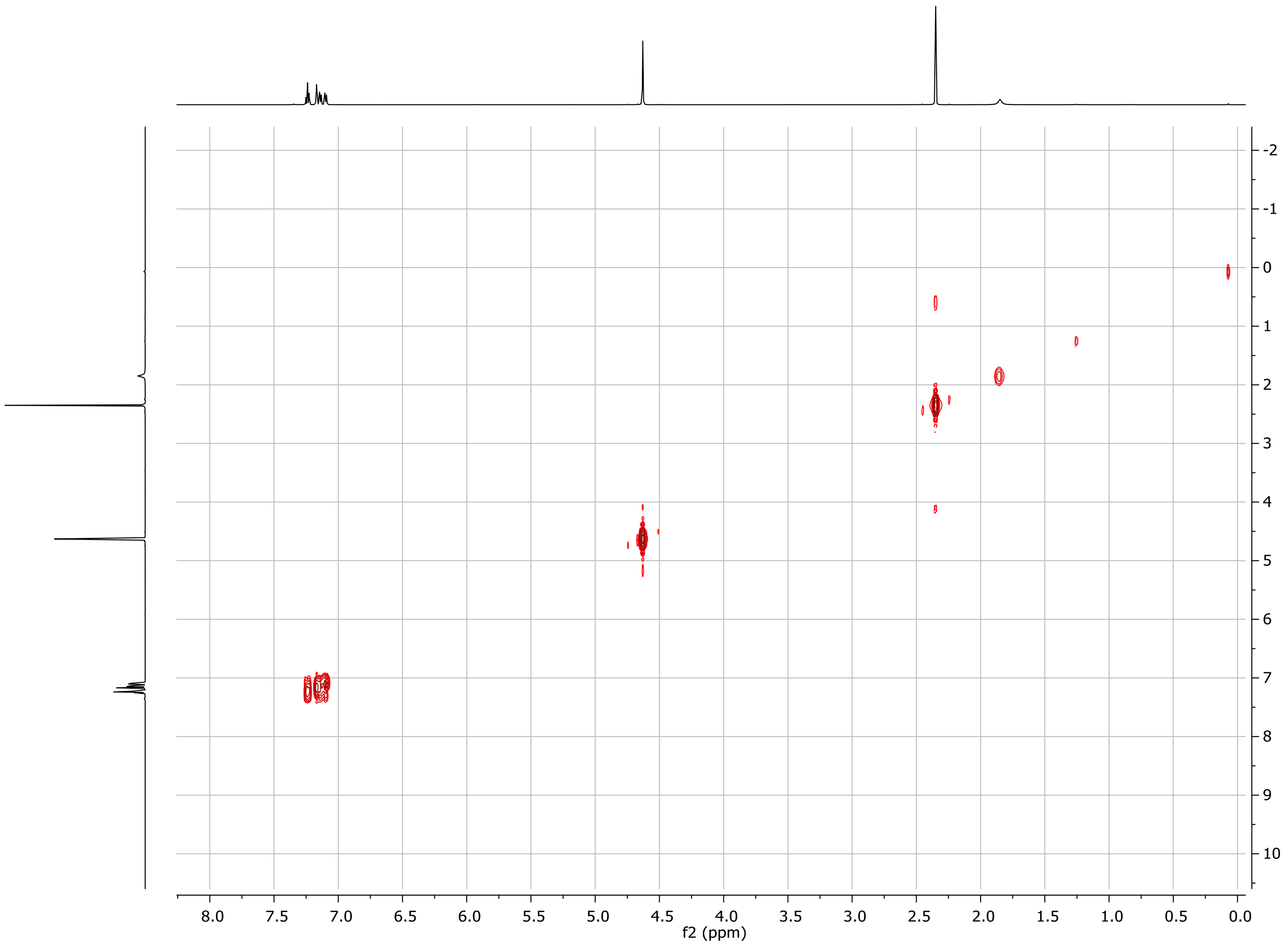

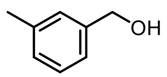

2o

^{1}H, ^{13}C HMBC

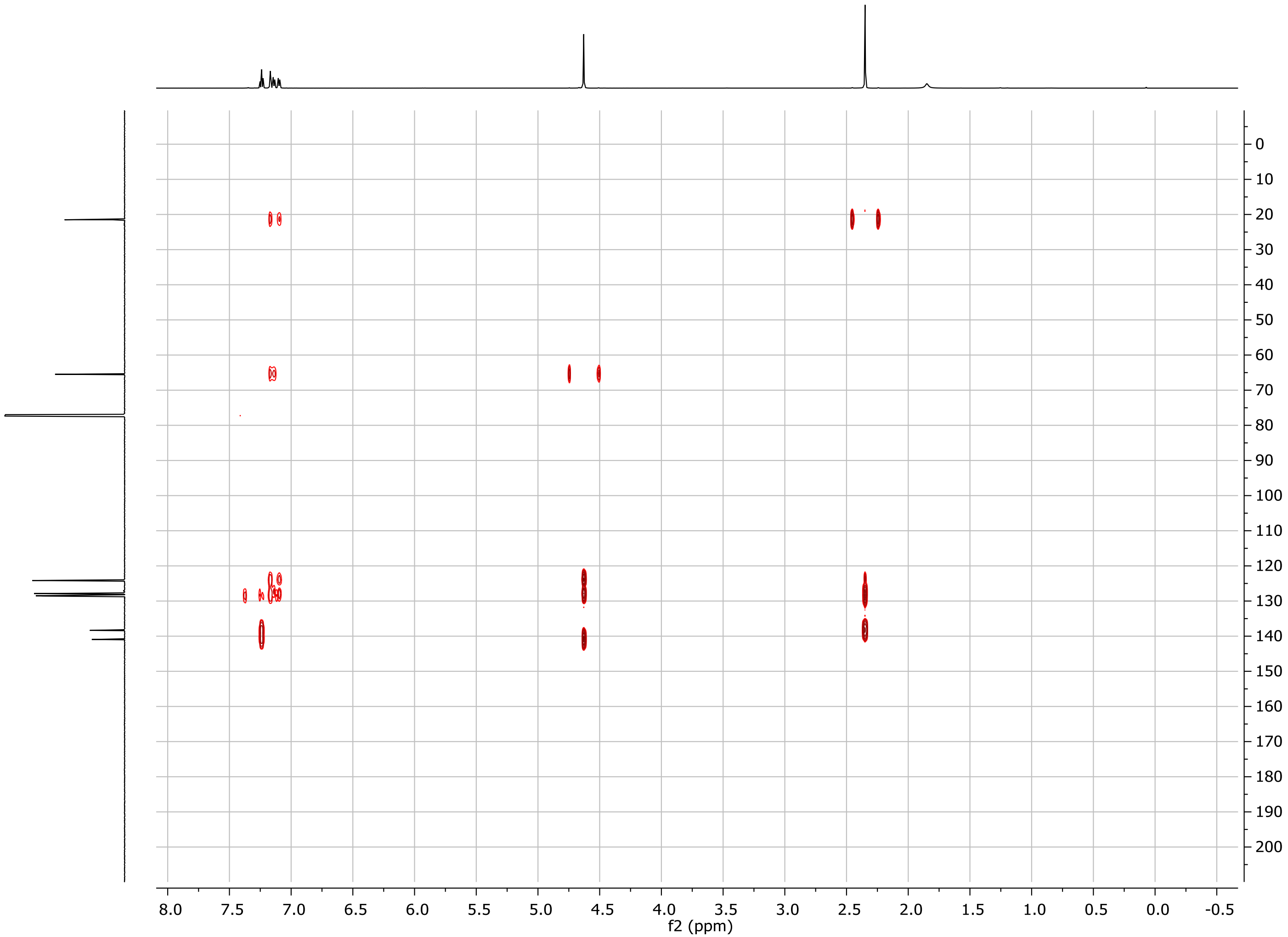

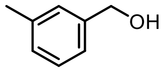

2o

^1H, ^{13}C HSQC

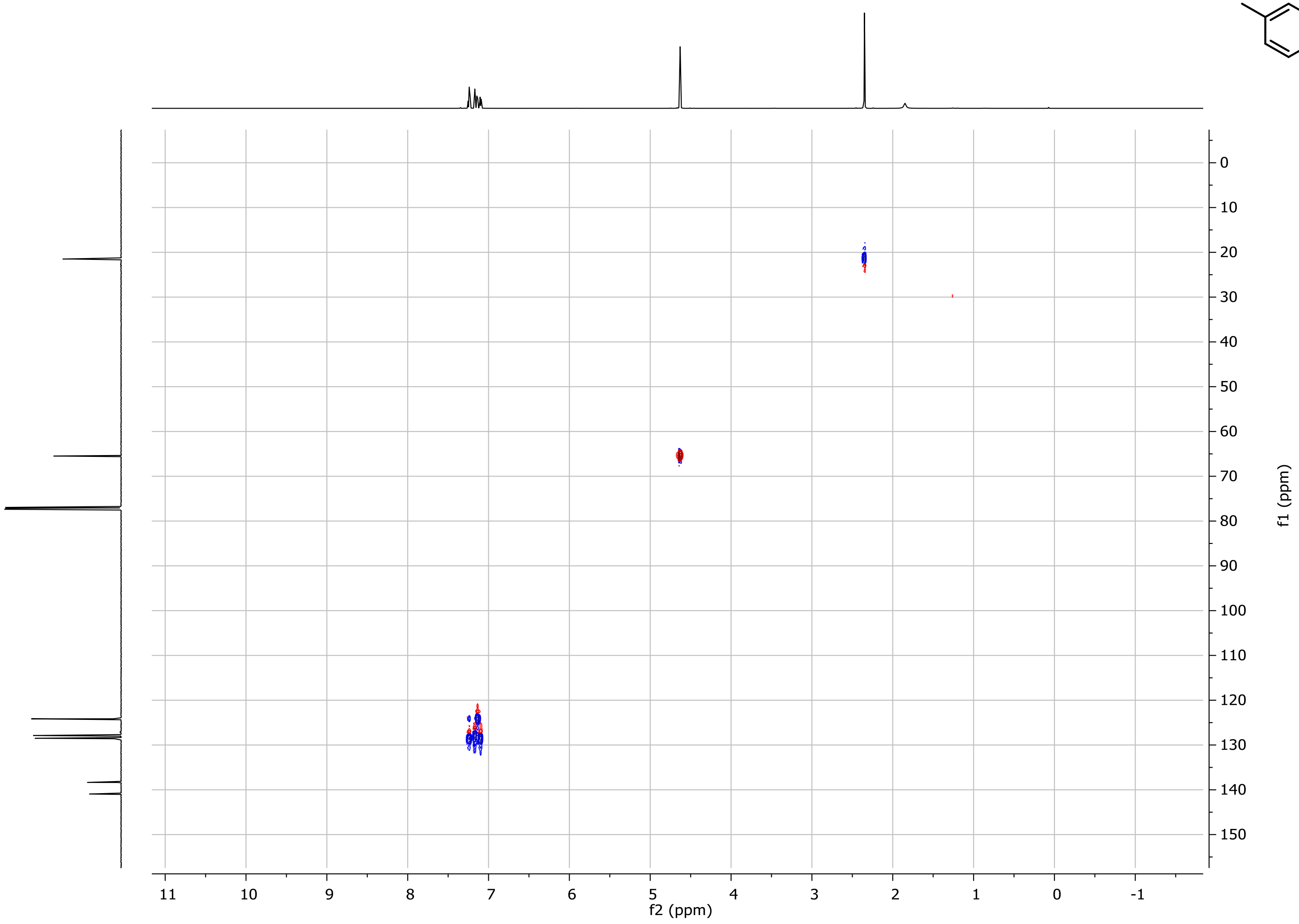

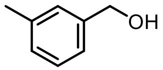

2o

HRMS

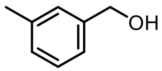
OH

2o

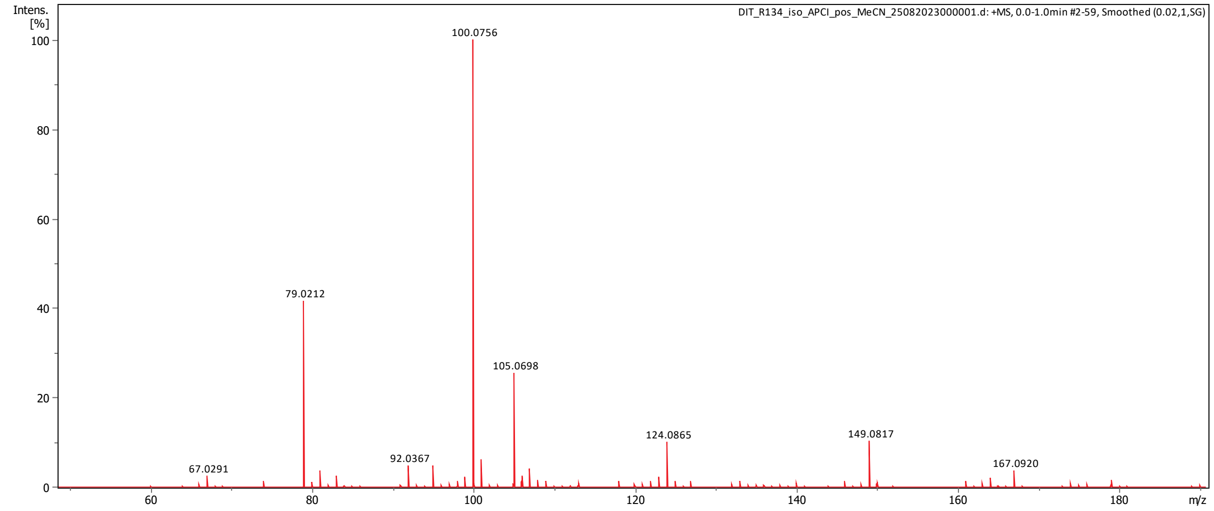
Intens.
[%]
DIT_R134_iso_APCI_pos_MeCN_25082023000001.d: +MS, 0.0-1.0min #2-59, Smoothed (0.02,1,SG)
100.0756
79.0212
105.0698
124.0865
149.0817
92.0367
67.0291
167.0920
m/z

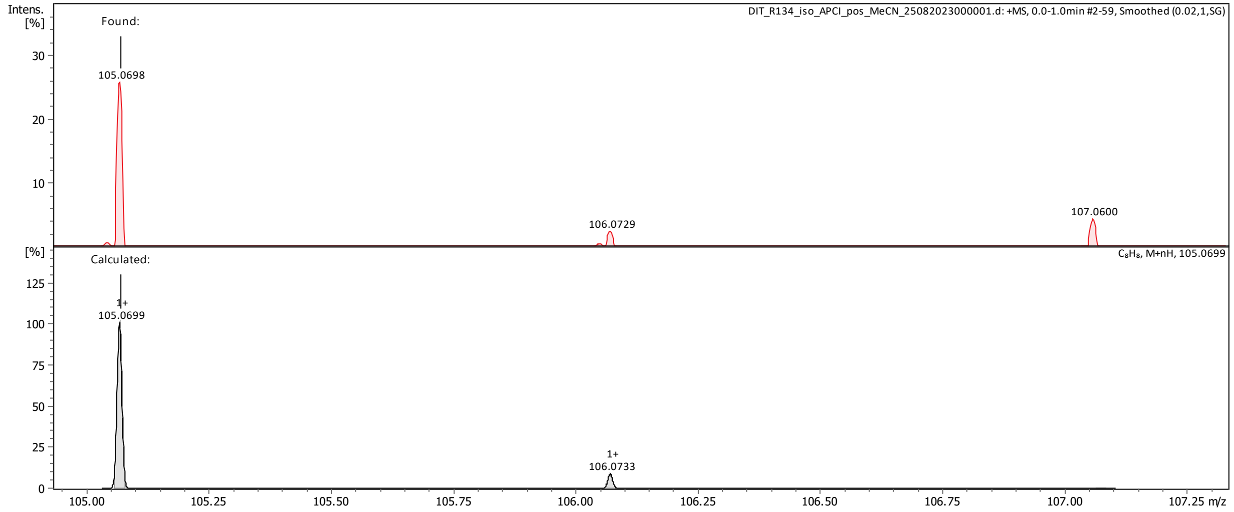
Intens.
[%]
Found:
DIT_R134_iso_APCI_pos_MeCN_25082023000001.d: +MS, 0.0-1.0min #2-59, Smoothed (0.02,1,SG)
105.0698
106.0729
107.0600
[%]
Calculated:
C8H8, M+nH, 105.0699
1+
105.0699
1+
106.0733
m/z

58 *o*-Tolylmethanol (2p)

^{1}H NMR

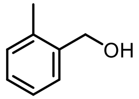

1p

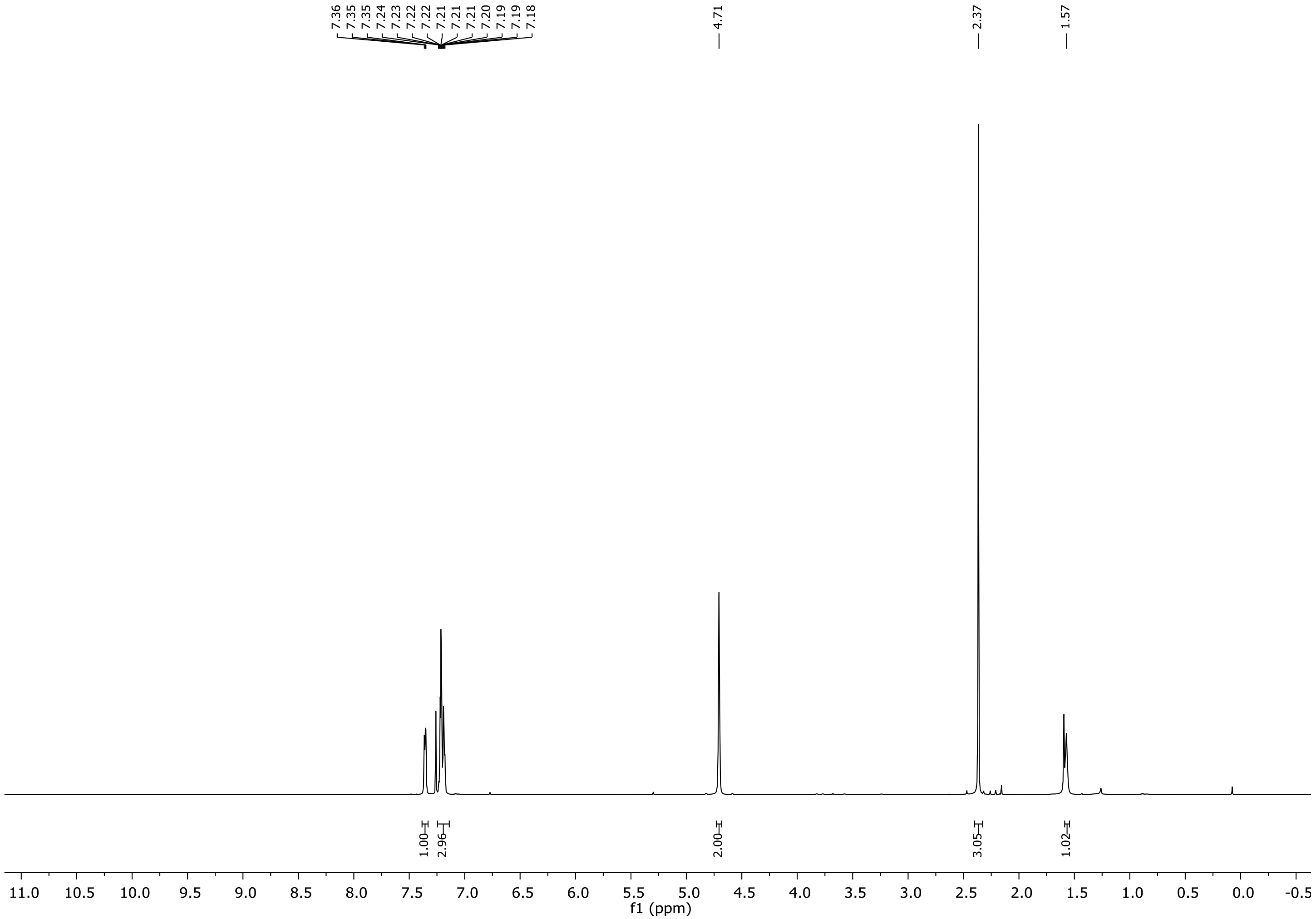

^{13}C NMR

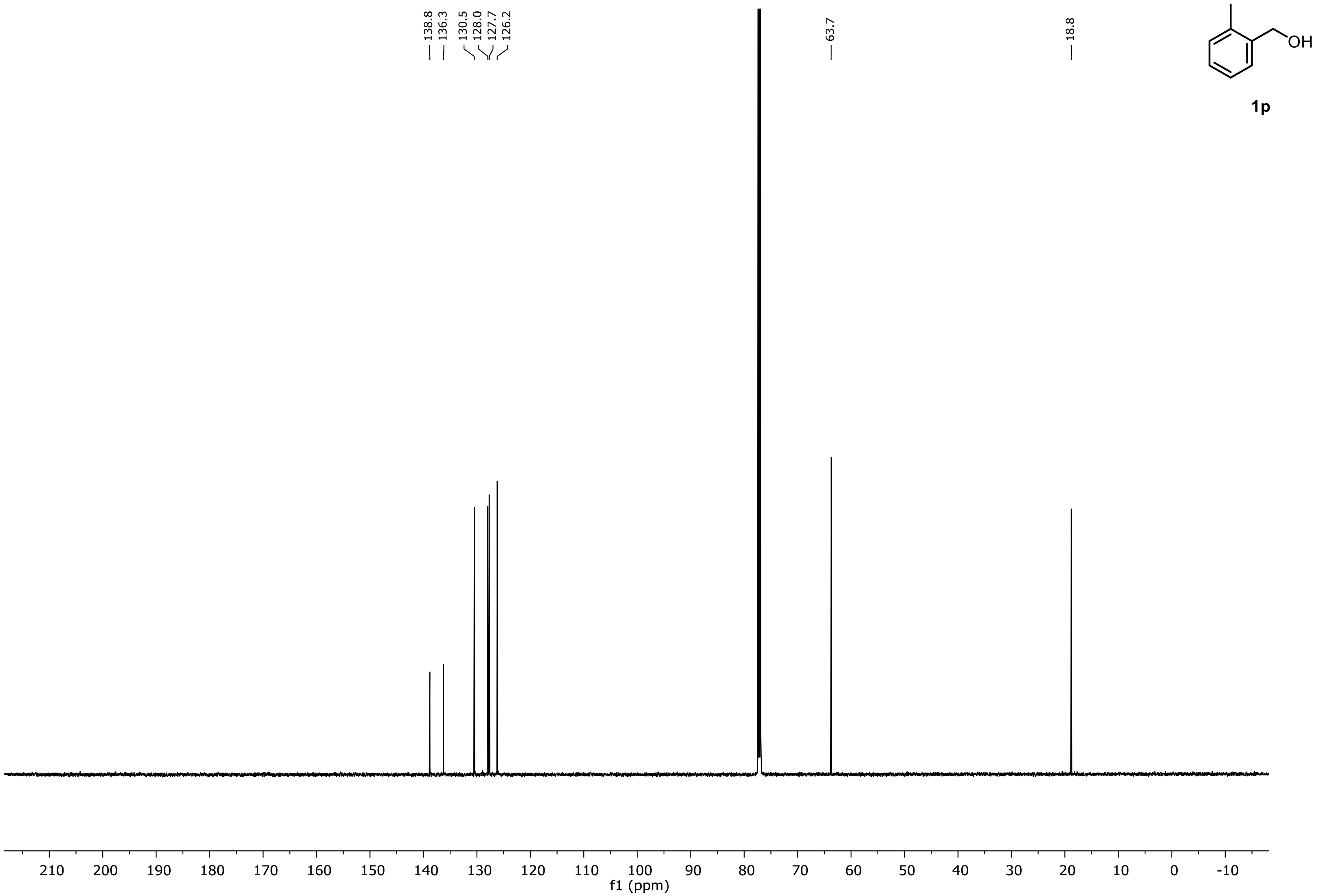
138.8
136.3
130.5
128.0
127.7
126.2
63.7
18.8
OH
210 200 190 180 170 160 150 140 130 120 110 100 90 80 70 60 50 40 30 20 10 0 -10
f1 (ppm)

1p

^{1}H, ^{1}H COSY

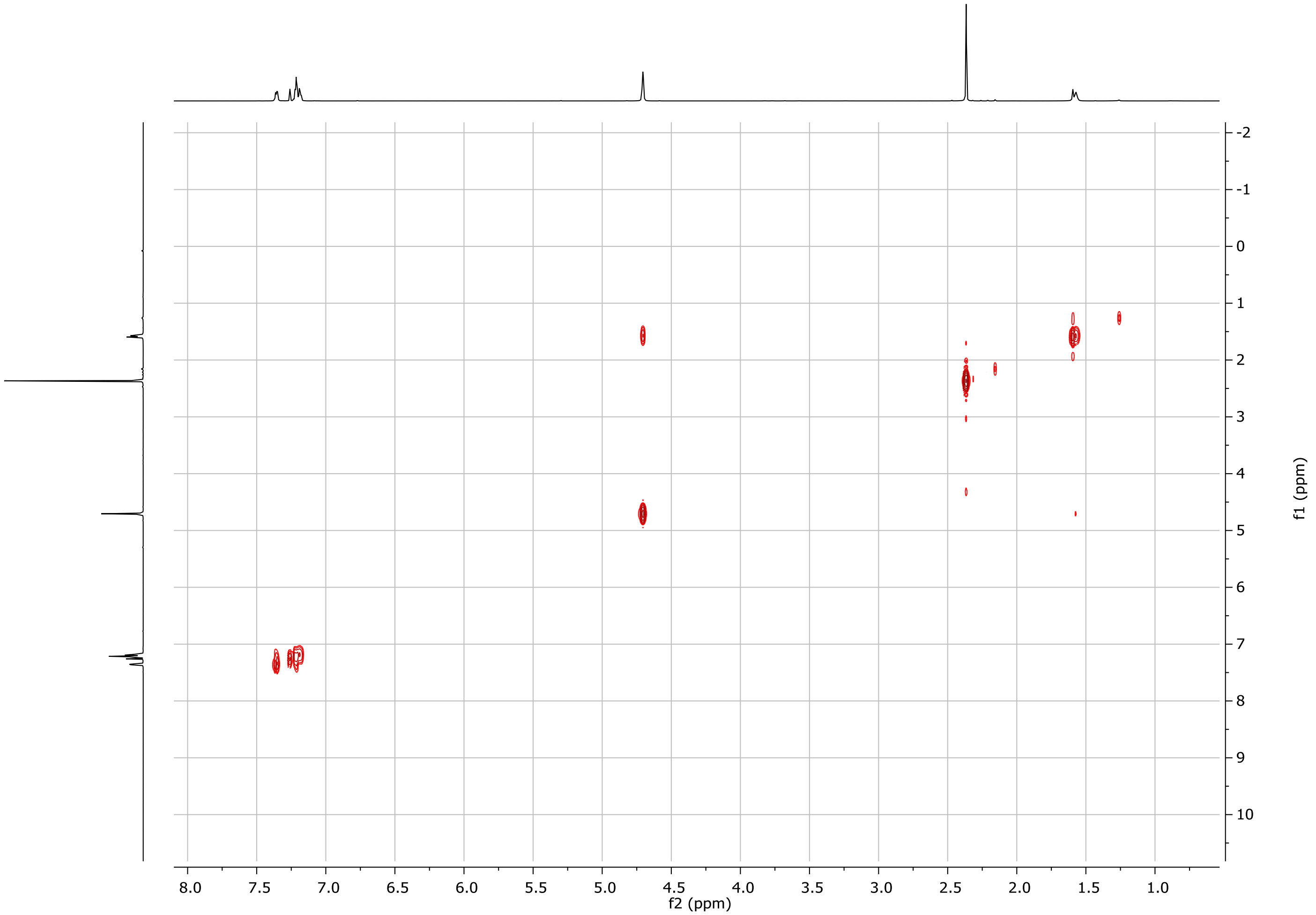
8.0 7.5 7.0 6.5 6.0 5.5 5.0 4.5 4.0 3.5 3.0 2.5 2.0 1.5 1.0
f2 (ppm)
-2 -1 0 1 2 3 4 5 6 7 8 9 10
f1 (ppm)

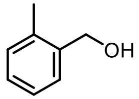
OH

1p

^{1}H, ^{13}C HMBC

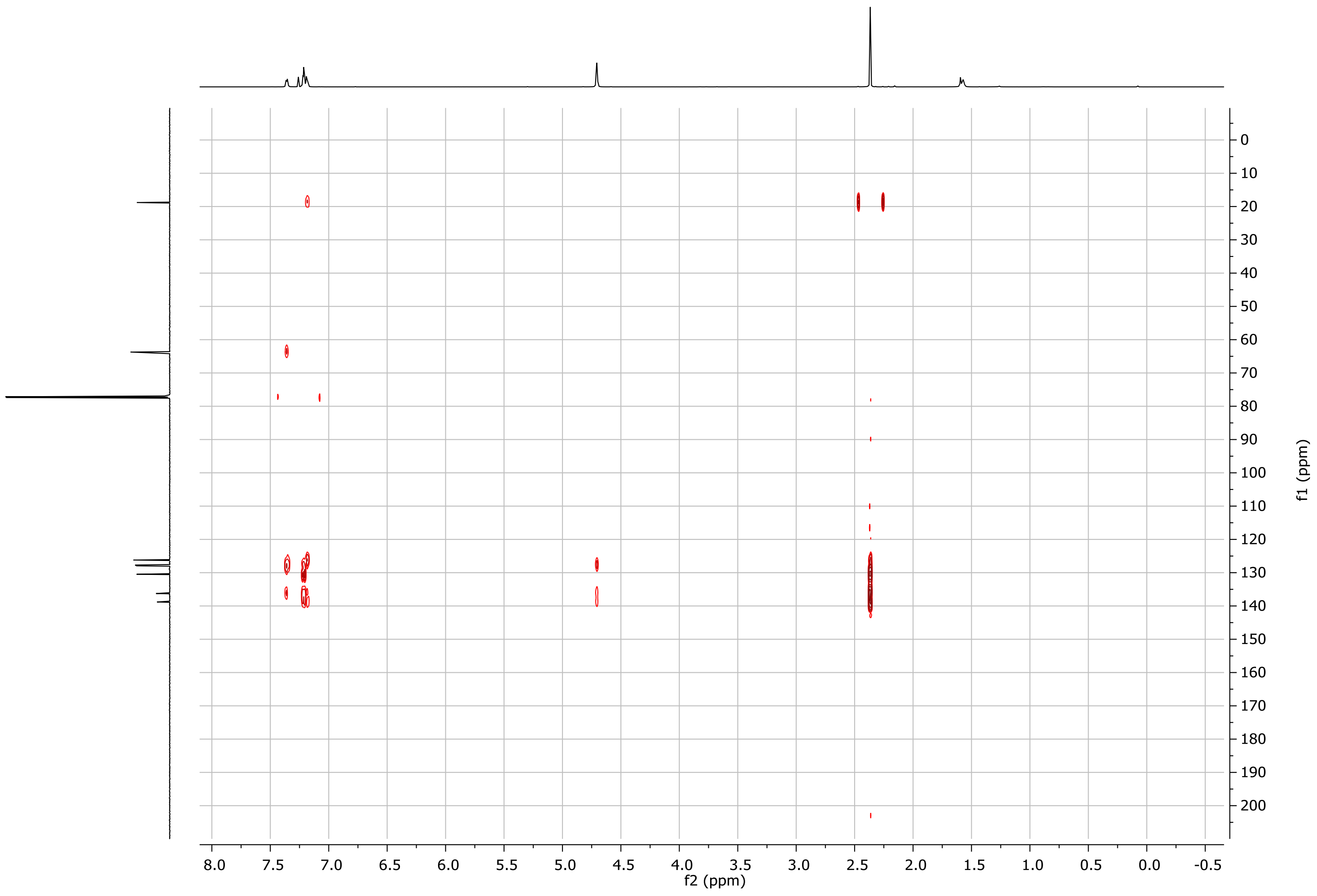

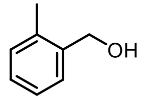

1p

^{1}H, ^{13}C HSQC

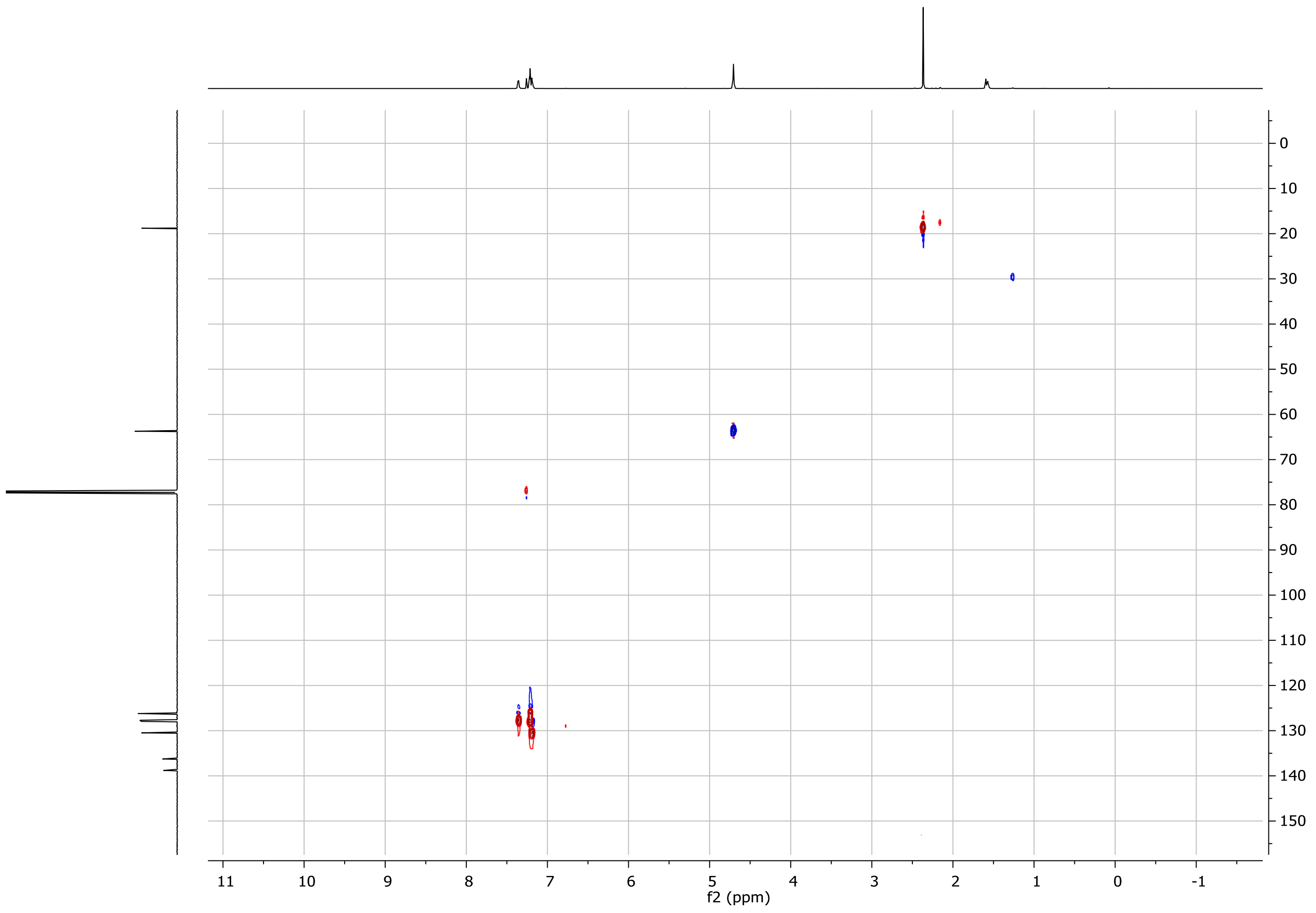

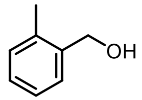

1p

HRMS

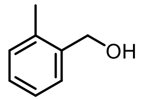
OH

1p

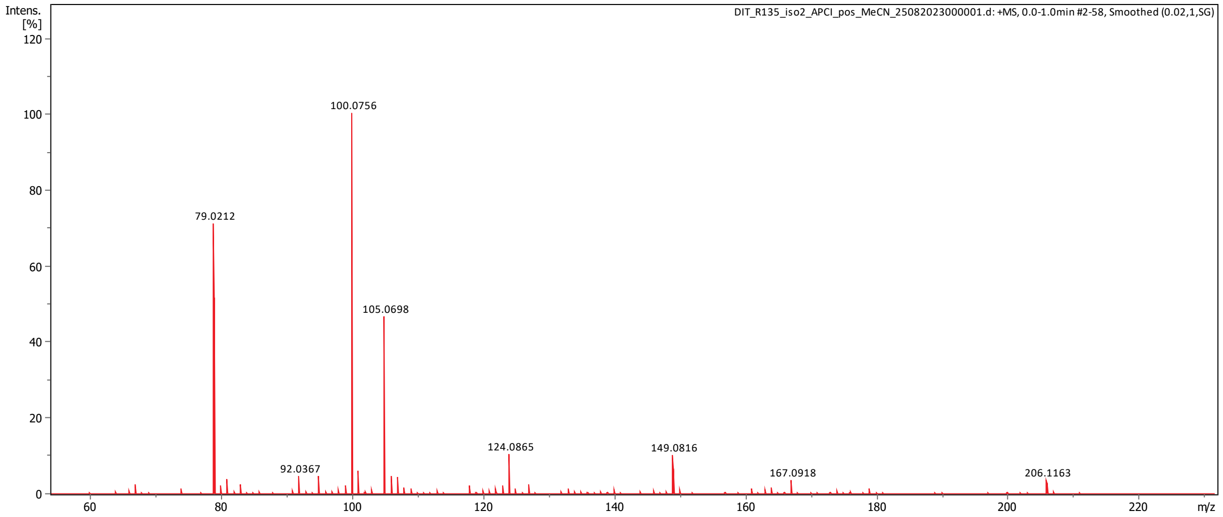
Intens.
[%]
DIT_R135_iso2_APCI_pos_MeCN_25082023000001.d: +MS, 0.0-1.0min #2-58, Smoothed (0.02,1,SG)
100.0756
79.0212
105.0698
92.0367
124.0865
149.0816
167.0918
206.1163
m/z

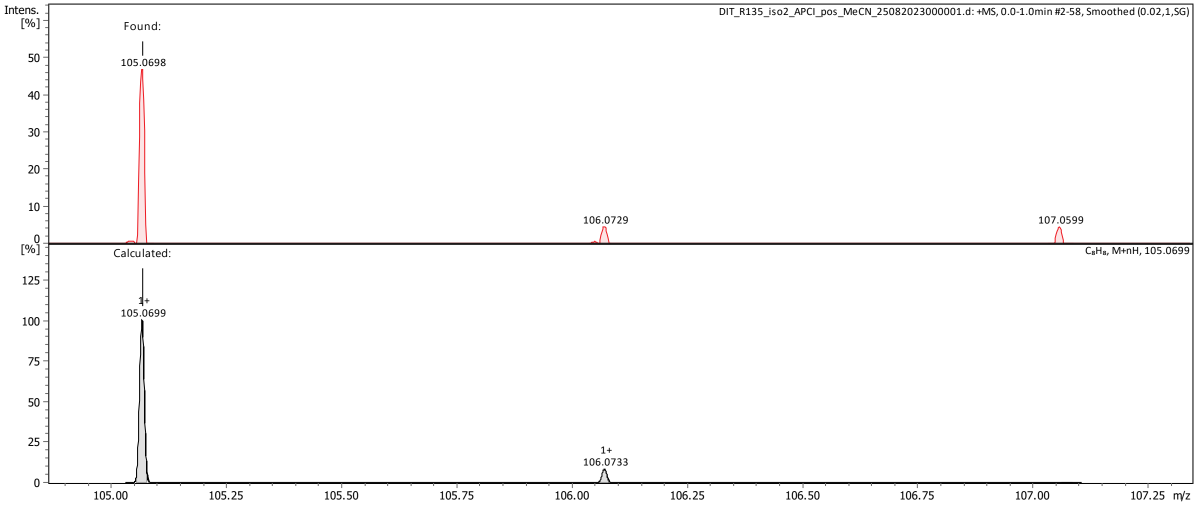
Intens.
[%]
DIT_R135_iso2_APCI_pos_MeCN_25082023000001.d: +MS, 0.0-1.0min #2-58, Smoothed (0.02,1,SG)
Found:
105.0698
106.0729
107.0599
Calculated:
C₈H₉, M+nH, 105.0699
1+
105.0699
1+
106.0733
m/z

59 4-(Trifluoromethyl)phenyl)methanol (2q)

^{1}H NMR

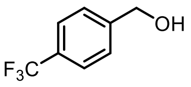

2q

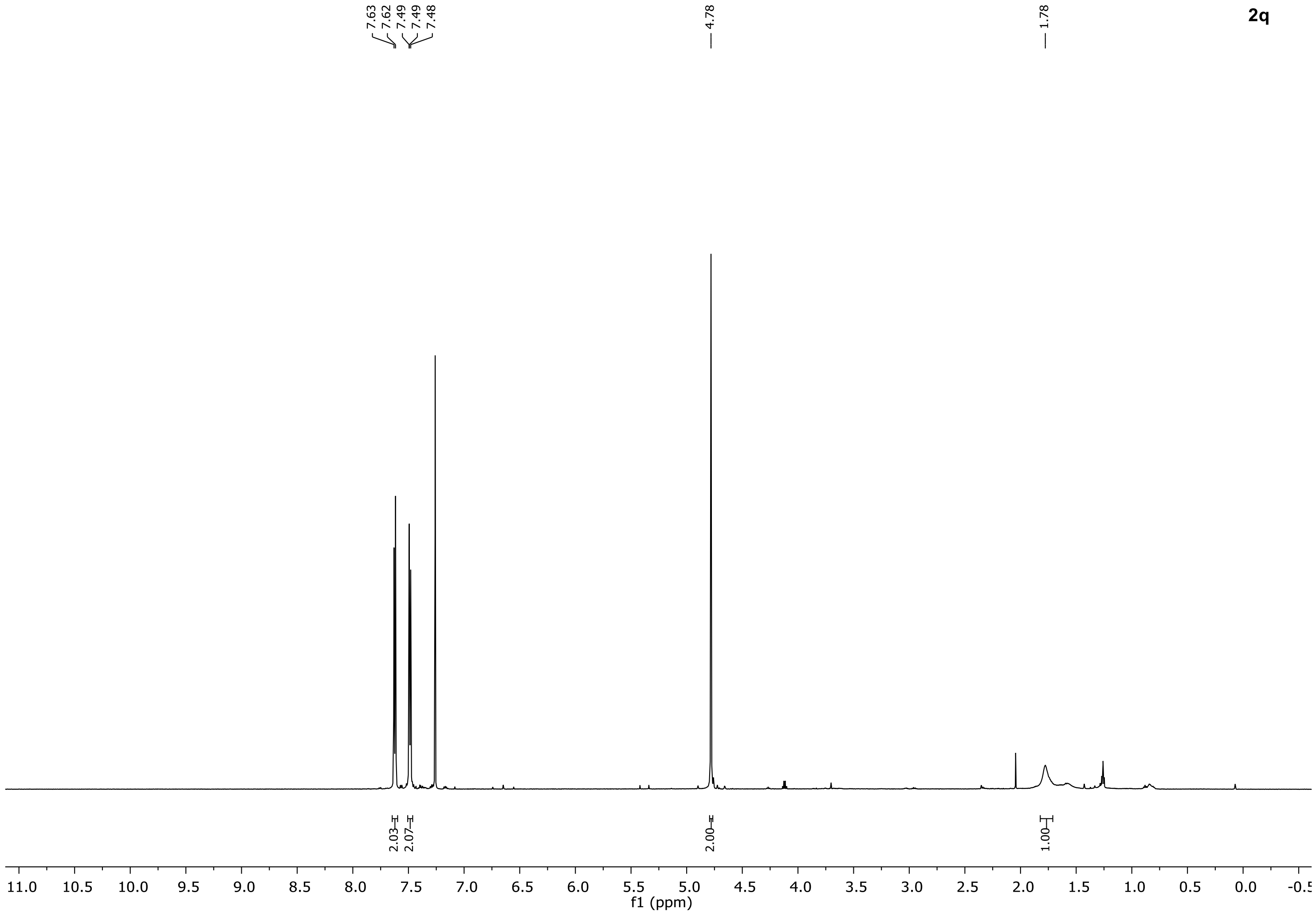

^{13}C NMR

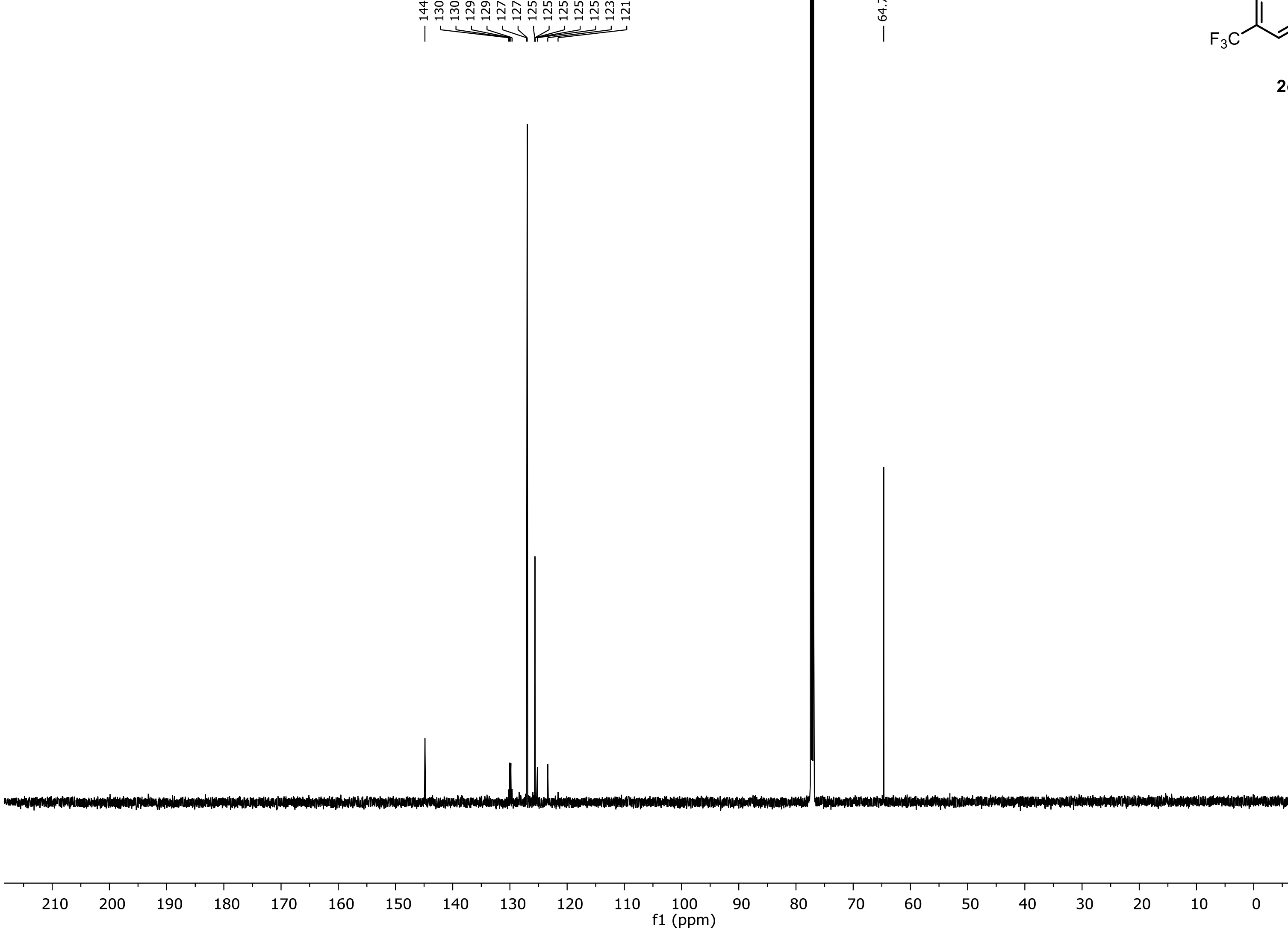
144.9
130.3
130.0
129.8
129.6
127.1
127.0
125.7
125.6
125.6
125.6
125.2
123.4
121.6
64.7
210 200 190 180 170 160 150 140 130 120 110 100 90 80 70 60 50 40 30 20 10 0 -10
f1 (ppm)

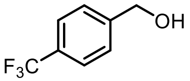
OH
F3C

2q

^{19}F NMR

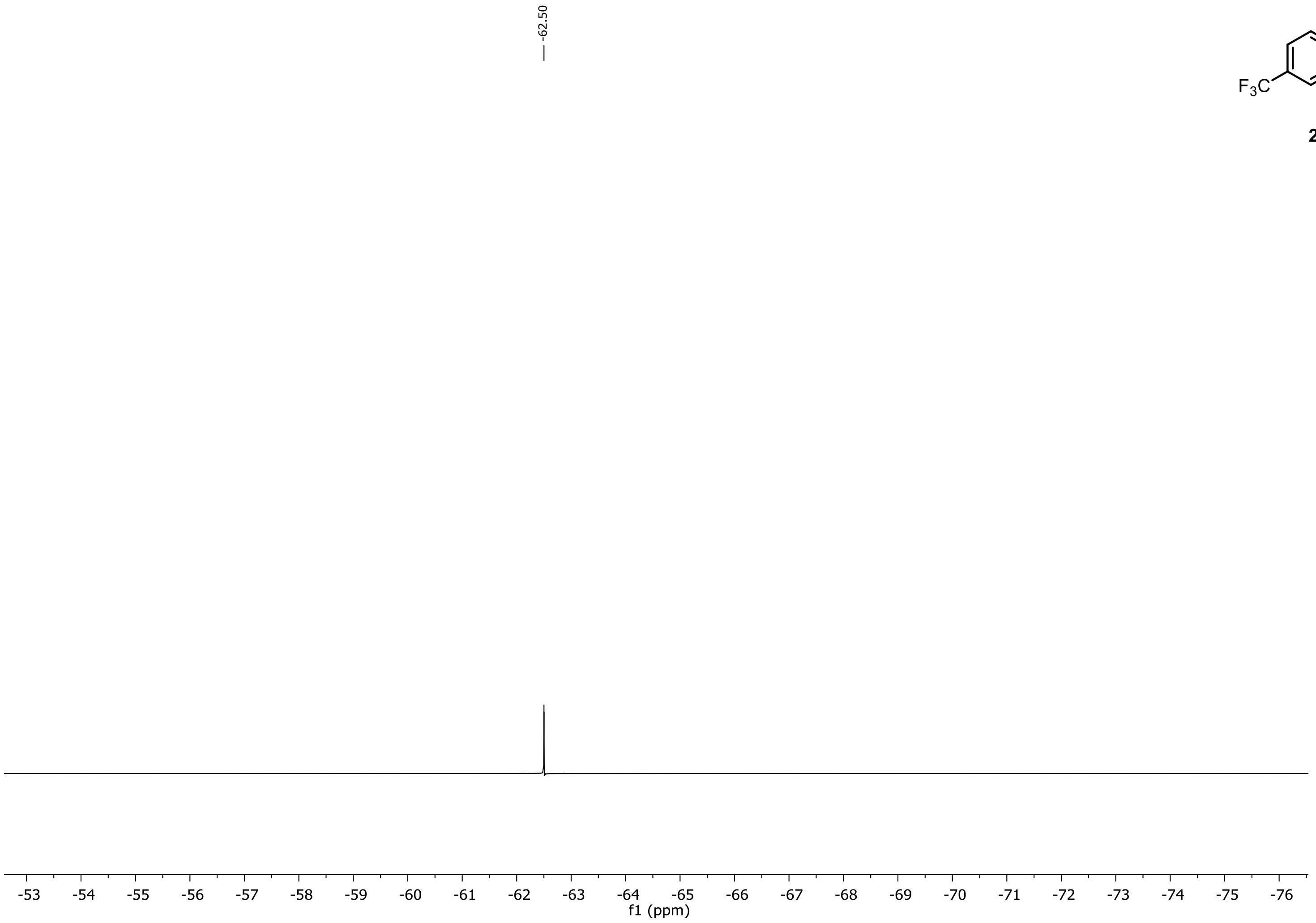
-62.50
-53
-54
-55
-56
-57
-58
-59
-60
-61
-62
-63
-64
-65
-66
-67
-68
-69
-70
-71
-72
-73
-74
-75
-76
f1 (ppm)

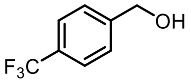
OH
F3C

2q

^{1}H, ^{1}H COSY

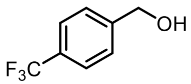

2q

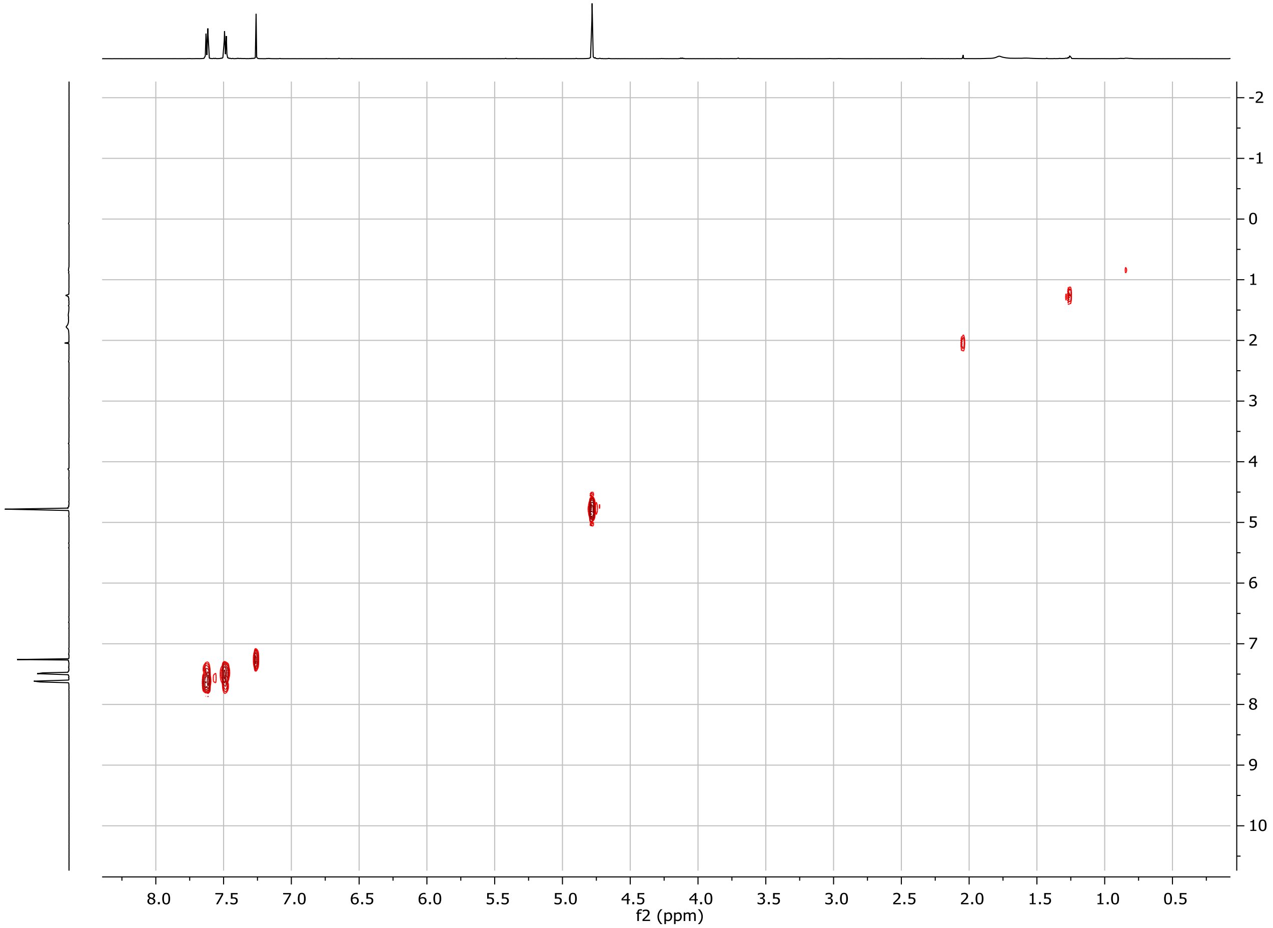

^{1}H, ^{13}C HMBC

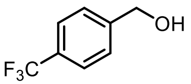

2q

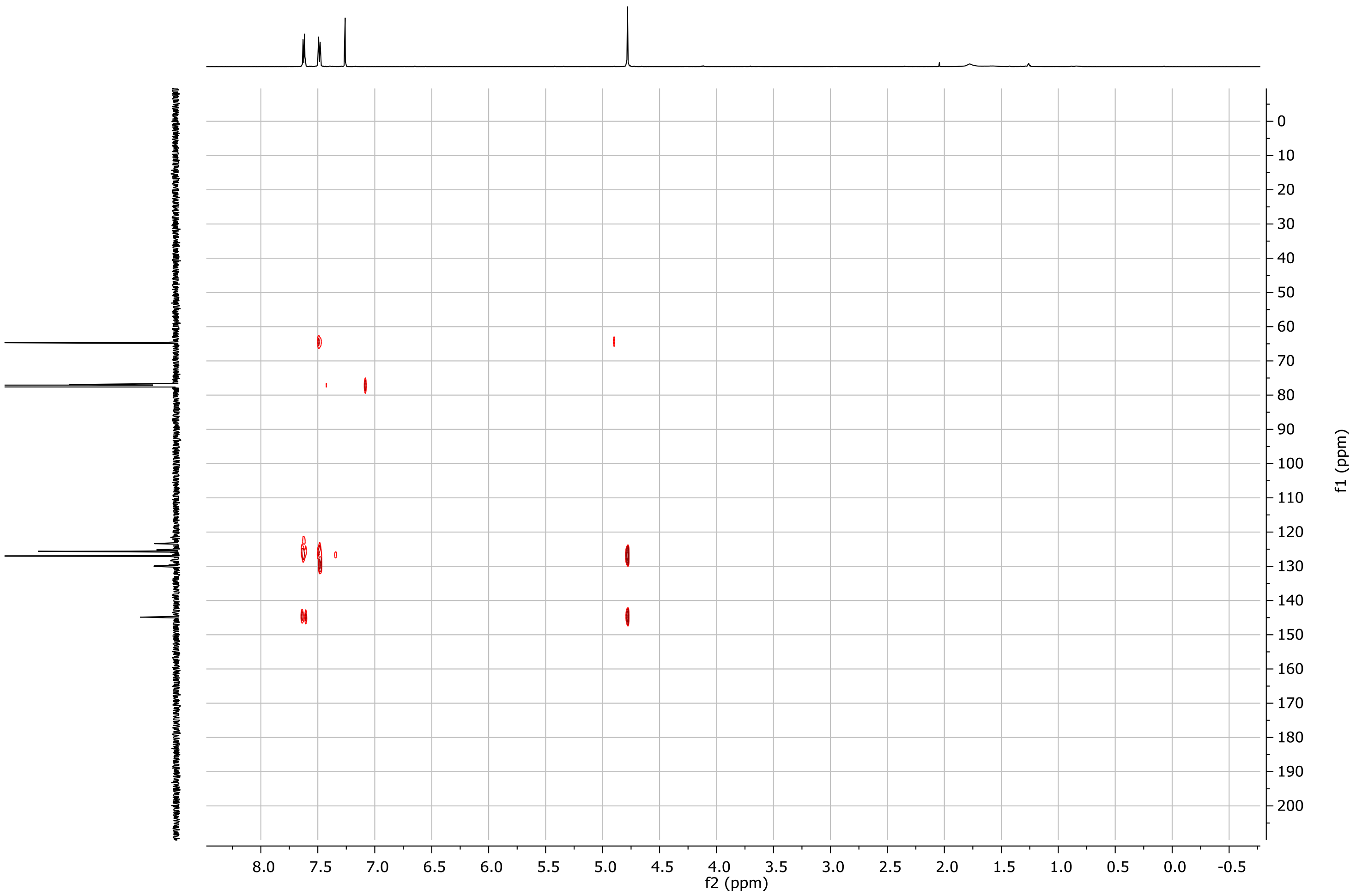

^{1}H, ^{13}C HSQC

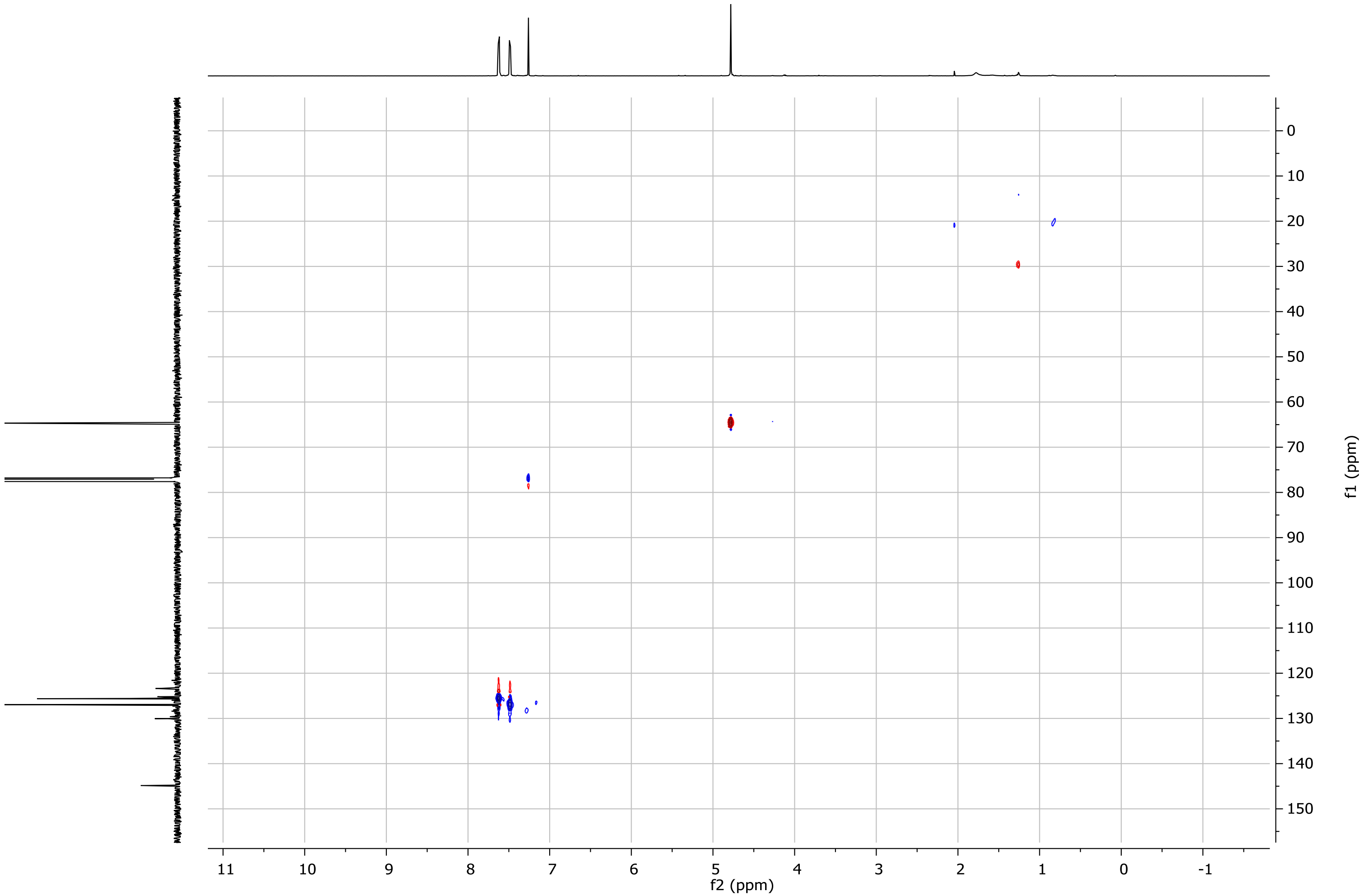

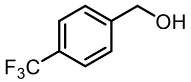

2q

HRMS

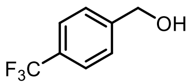

2q

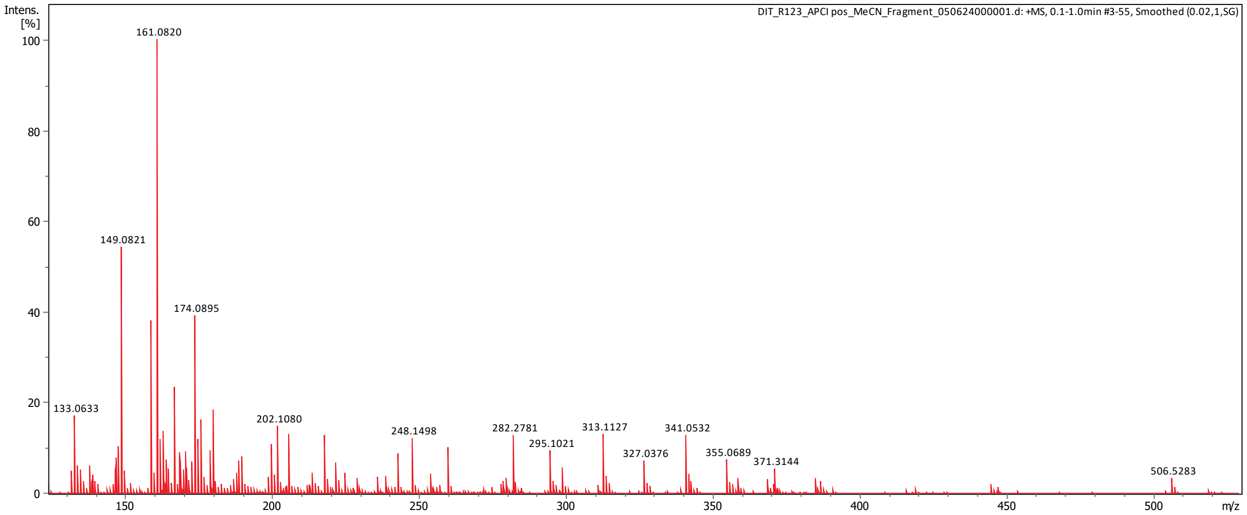

Intens. [%]
DIT_R123_APCI pos_MeCN_Fragment_050624000001.d: +MS, 0.1-1.0min #3-55, Smoothed (0.02,1,SG)
161.0820
149.0821
174.0895
133.0633
202.1080
248.1498
282.2781
295.1021
313.1127
327.0376
341.0532
355.0689
371.3144
506.5283
m/z

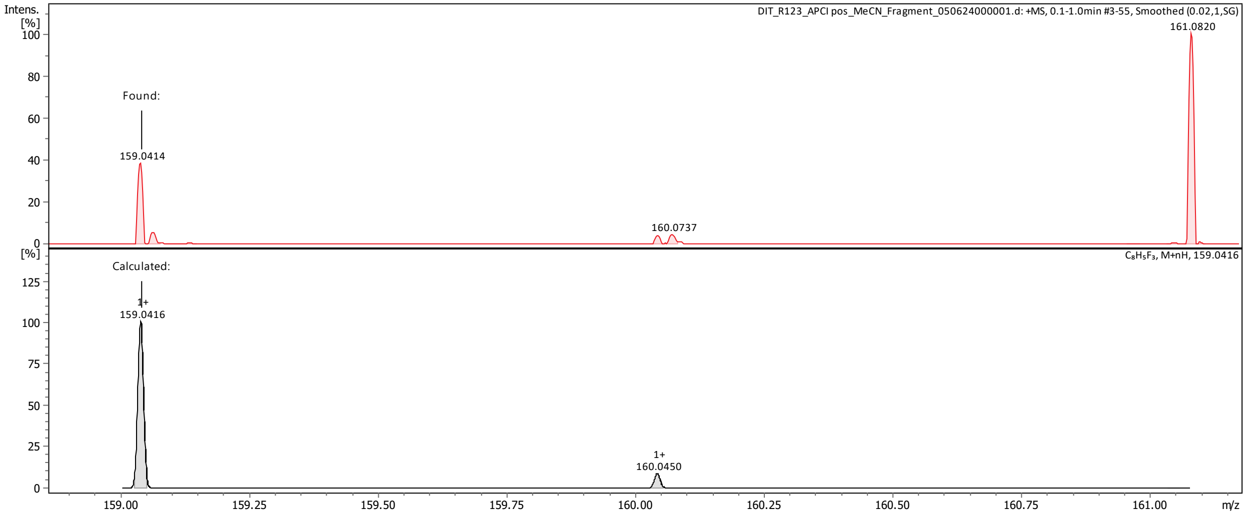

Intens. [%]
DIT_R123_APCI pos_MeCN_Fragment_050624000001.d: +MS, 0.1-1.0min #3-55, Smoothed (0.02,1,SG)
161.0820
Found:
159.0414
160.0737
Calculated:
C8H5F3, M+nH, 159.0416
1+
159.0416
1+
160.0450
m/z

60 (4-Methoxyphenyl)methanol (2r)

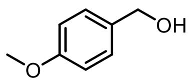

2r

^{1}H NMR

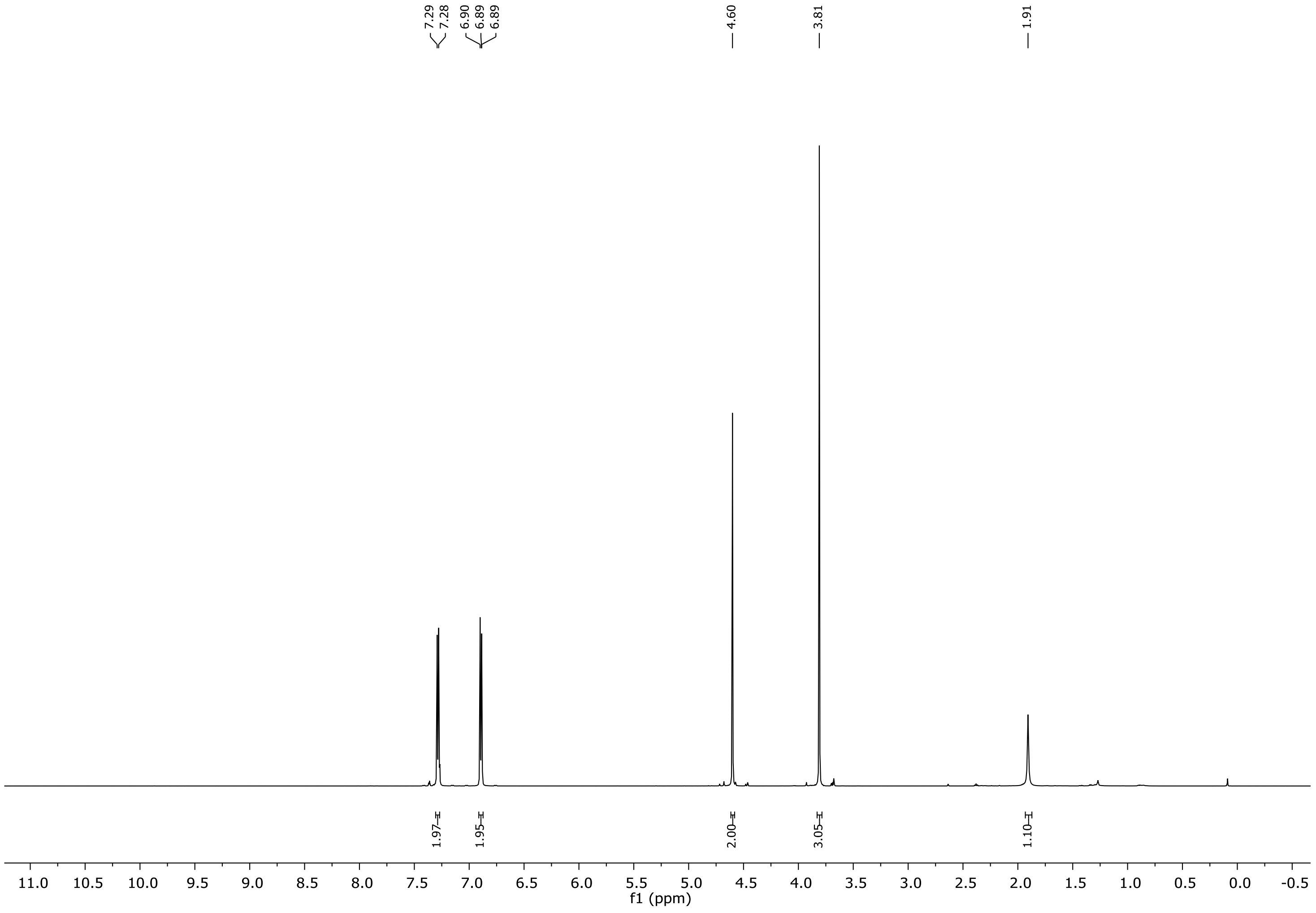

^{13}C NMR

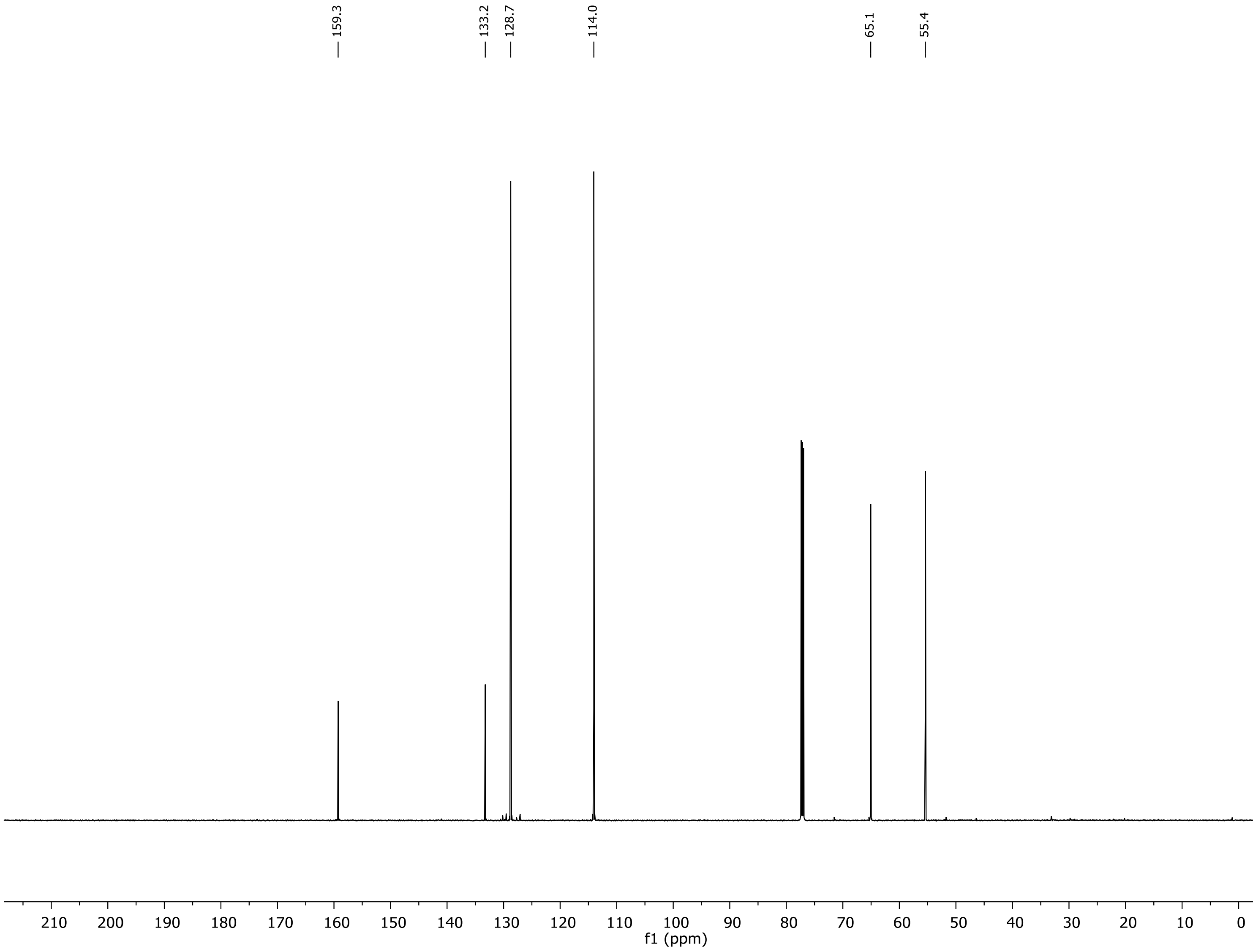
159.3
133.2
128.7
114.0
65.1
55.4
210 200 190 180 170 160 150 140 130 120 110 100 90 80 70 60 50 40 30 20 10 0 -10
f1 (ppm)

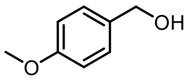
O
OH

2r

^{1}H, ^{1}H COSY

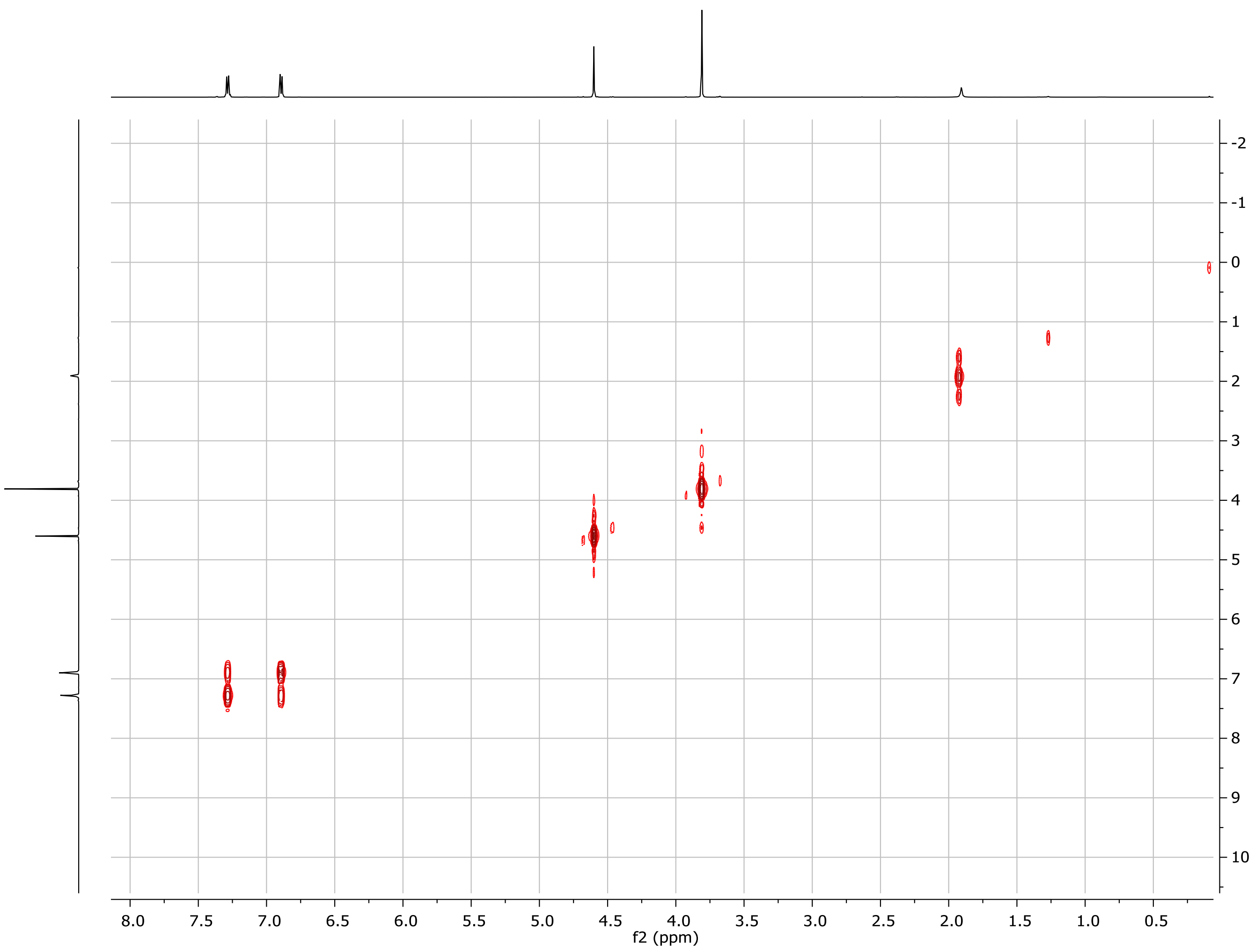

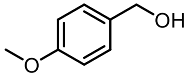

2r

^{1}H, ^{13}C HMBC

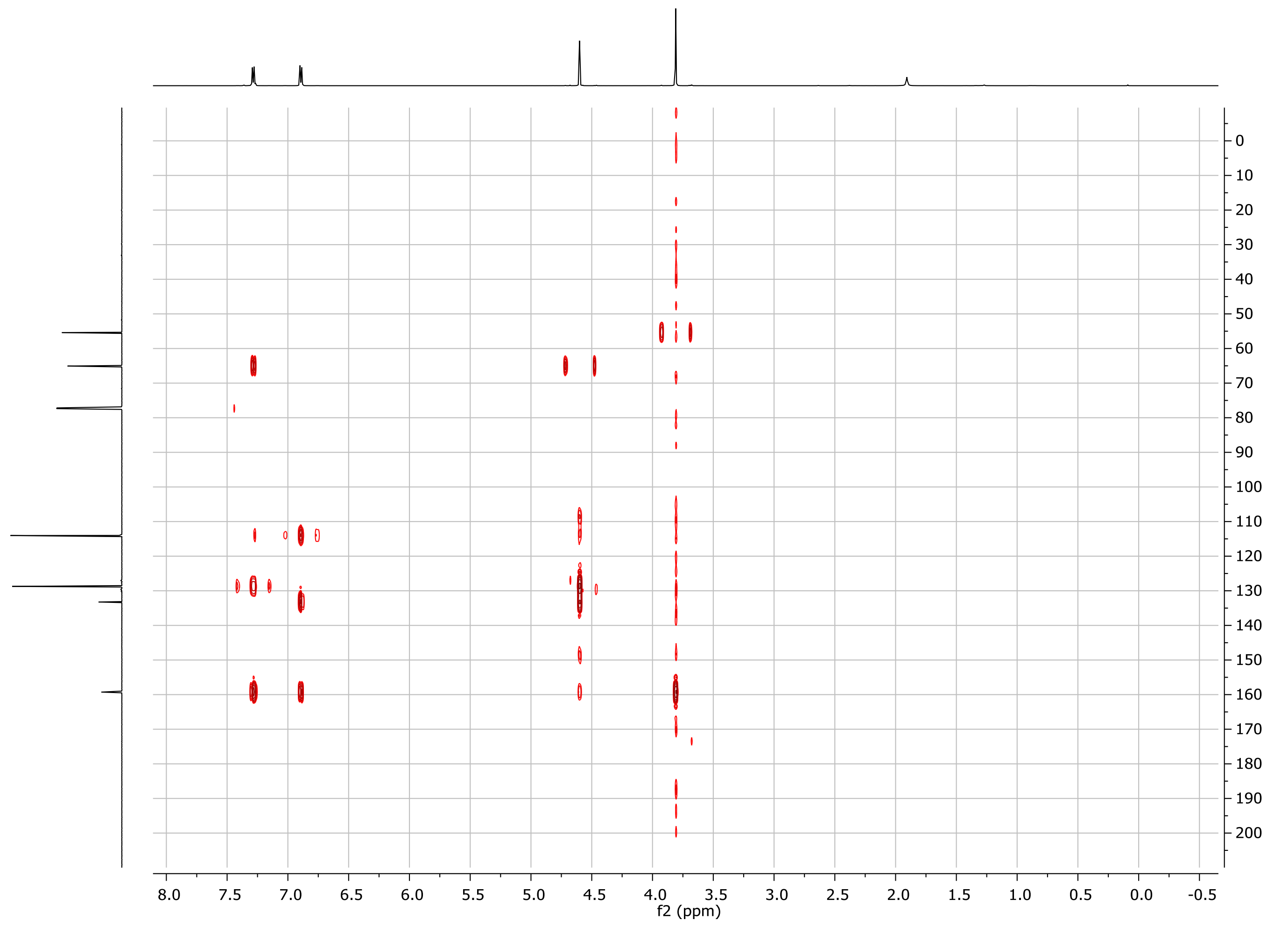

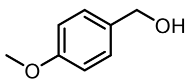

2r

^{1}H, ^{13}C HSQC

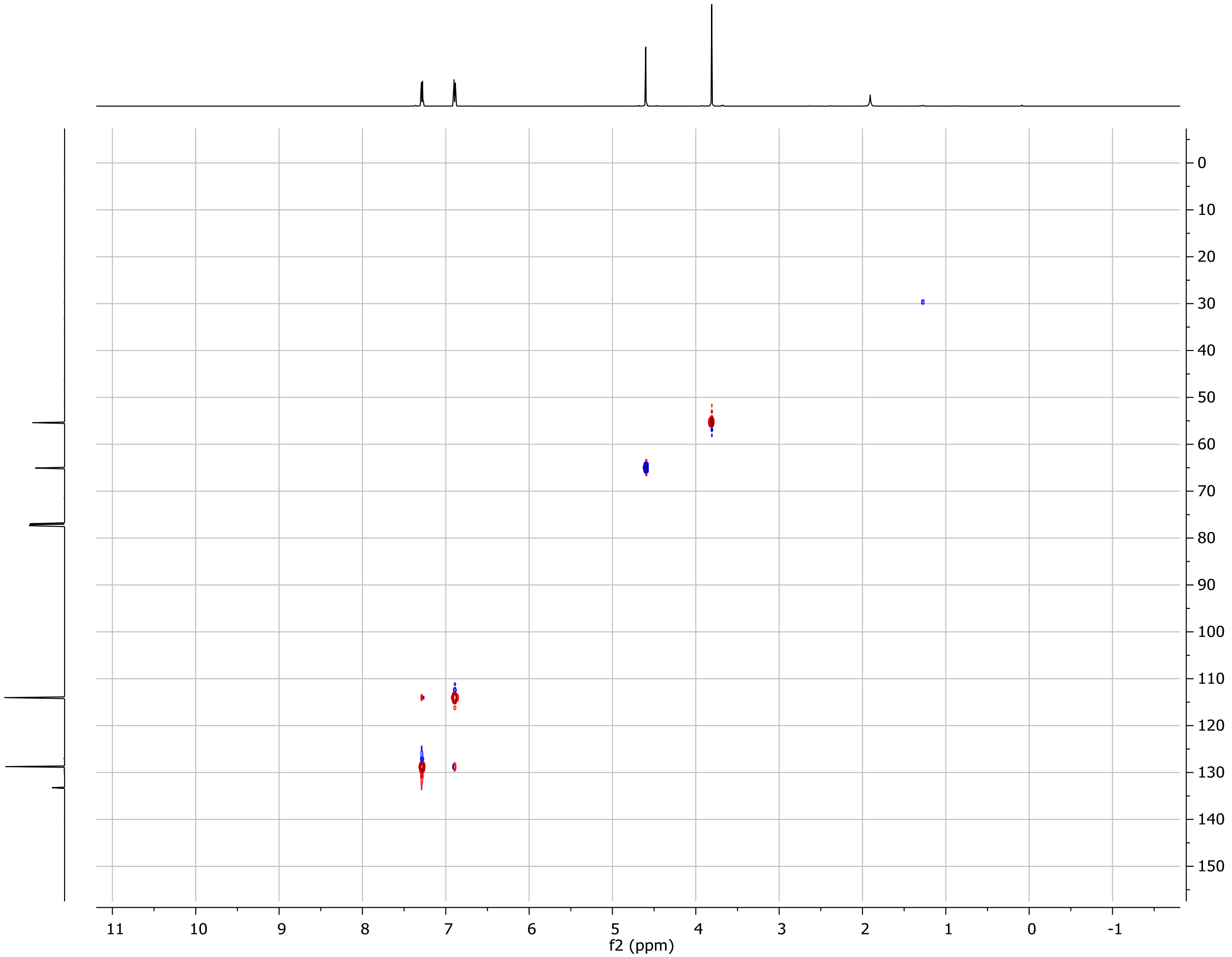

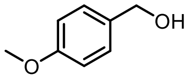

2r

HRMS

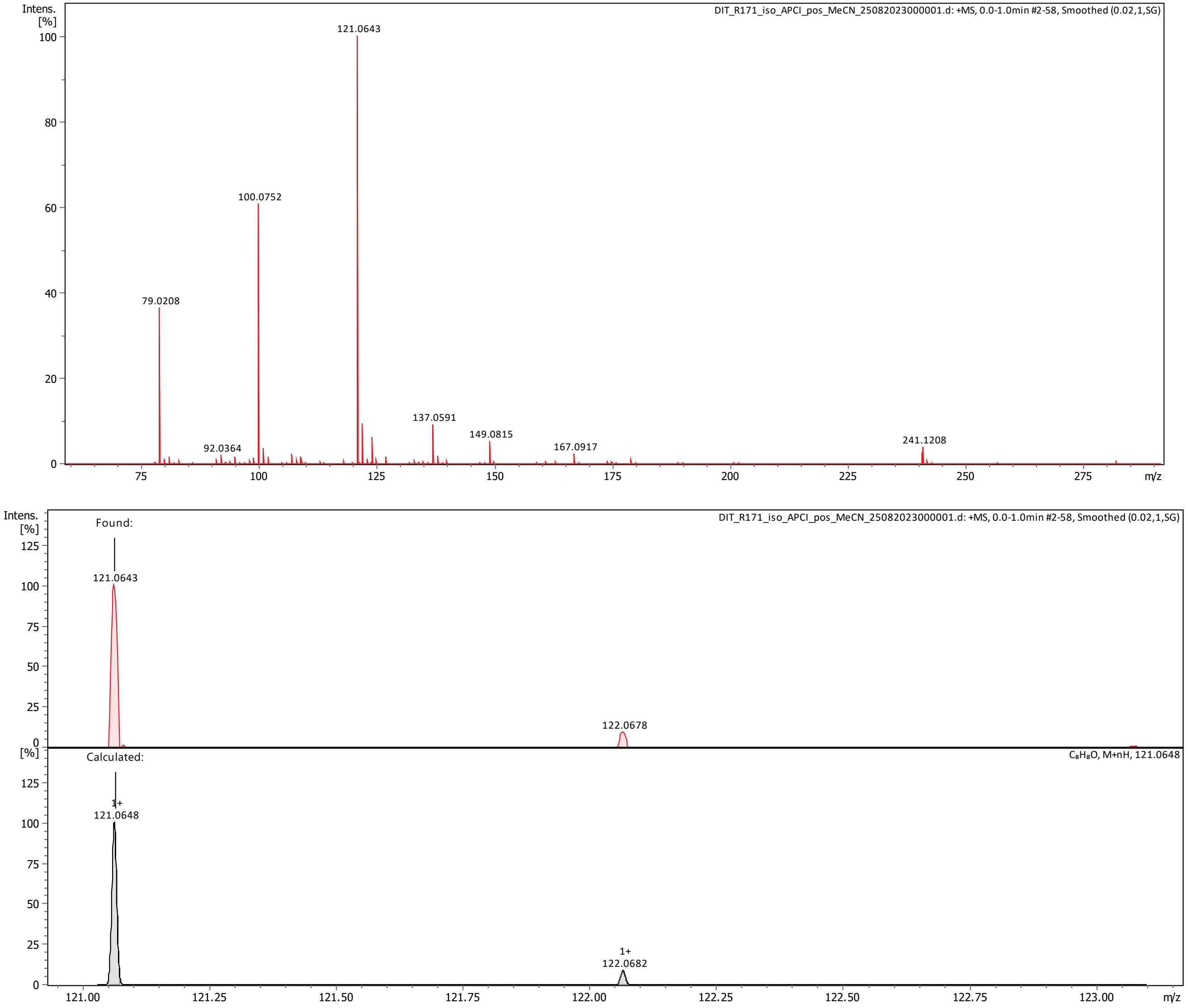

Intens. [%]
DIT_R171_iso_APCI_pos_MeCN_25082023000001.d: +MS, 0.0-1.0min #2-58, Smoothed (0.02,1,SG)
121.0643
100.0752
79.0208
92.0364
137.0591
149.0815
167.0917
241.1208
m/z
Found:
121.0643
122.0678
Calculated:
C8H8O, M+nH, 121.0648
1+
121.0648
1+
122.0682

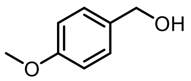

OH
O

2r

61 (4-Chlorophenyl)methanol (2s)

^{1}H NMR

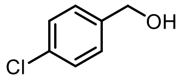

2s

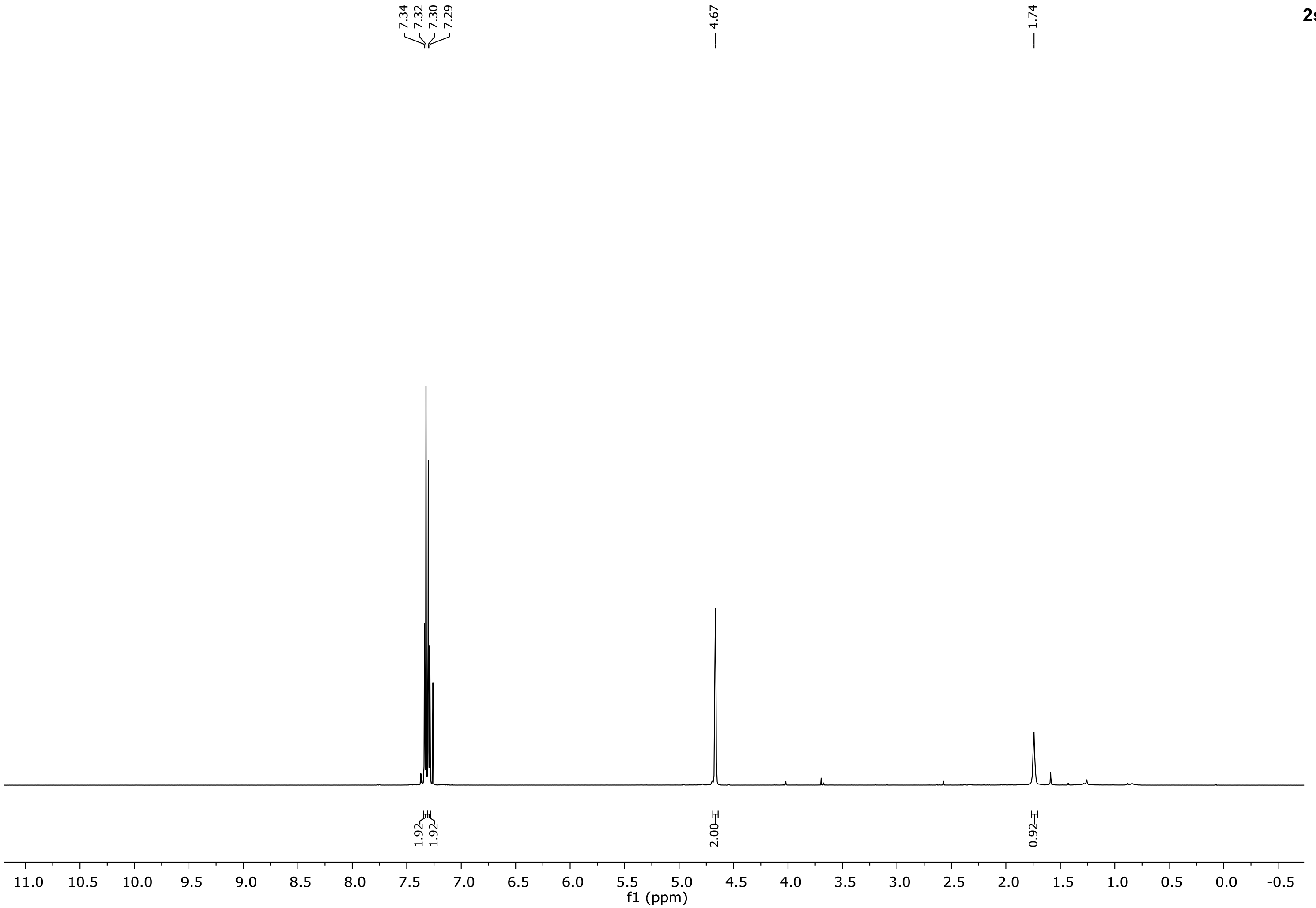

^{13}C NMR

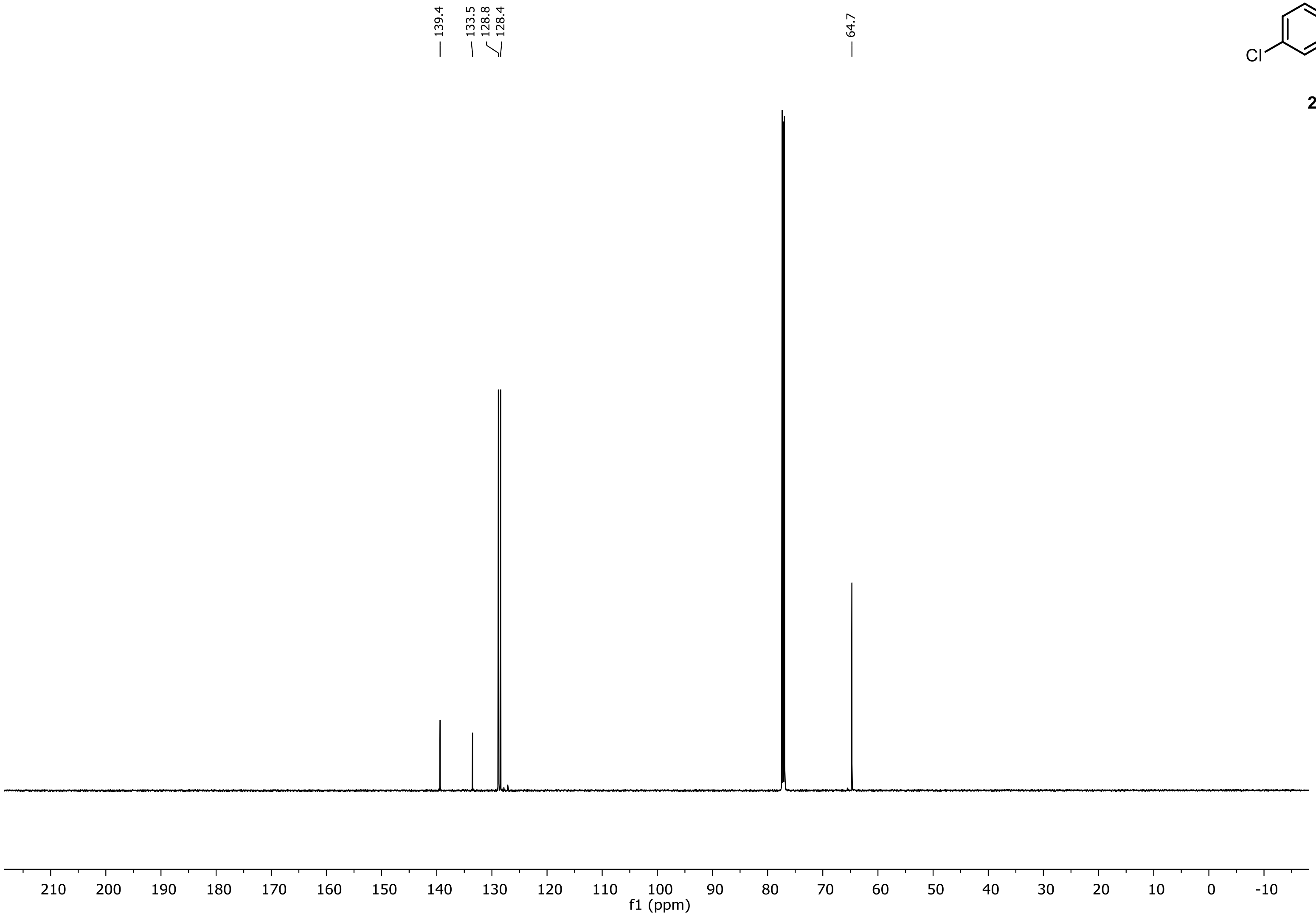

139.4
133.5
128.8
128.4
64.7
210
200
190
180
170
160
150
140
130
120
110
100
90
80
70
60
50
40
30
20
10
0
-10
f1 (ppm)

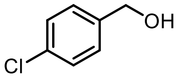

OH
Cl

2s

^{1}H, ^{1}H COSY

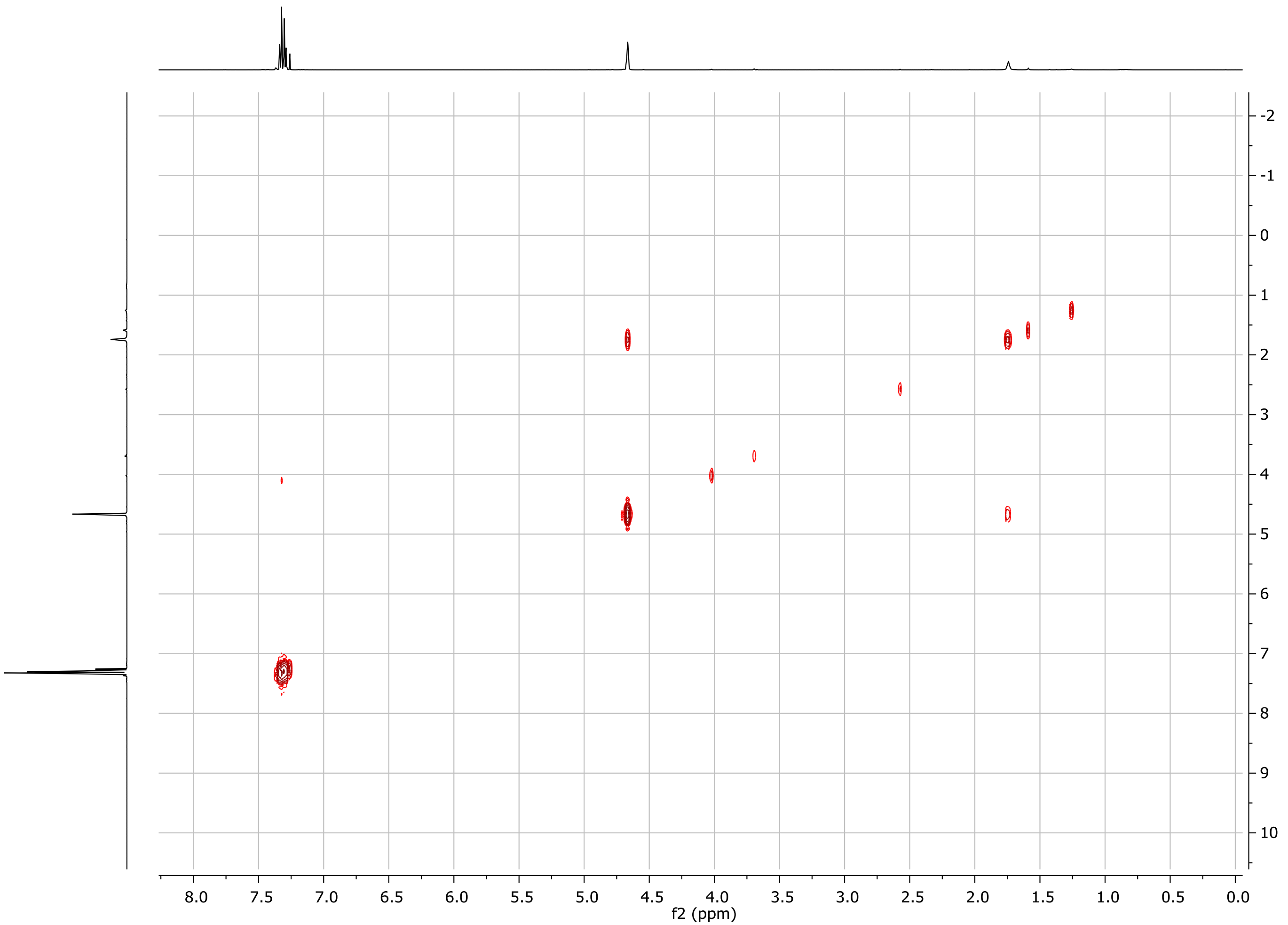
f2 (ppm)
f1 (ppm)
8.0
7.5
7.0
6.5
6.0
5.5
5.0
4.5
4.0
3.5
3.0
2.5
2.0
1.5
1.0
0.5
0.0
-2
-1
0
1
2
3
4
5
6
7
8
9
10

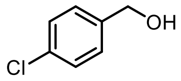
Cl
OH

2s

^1H, ^{13}C HMBC

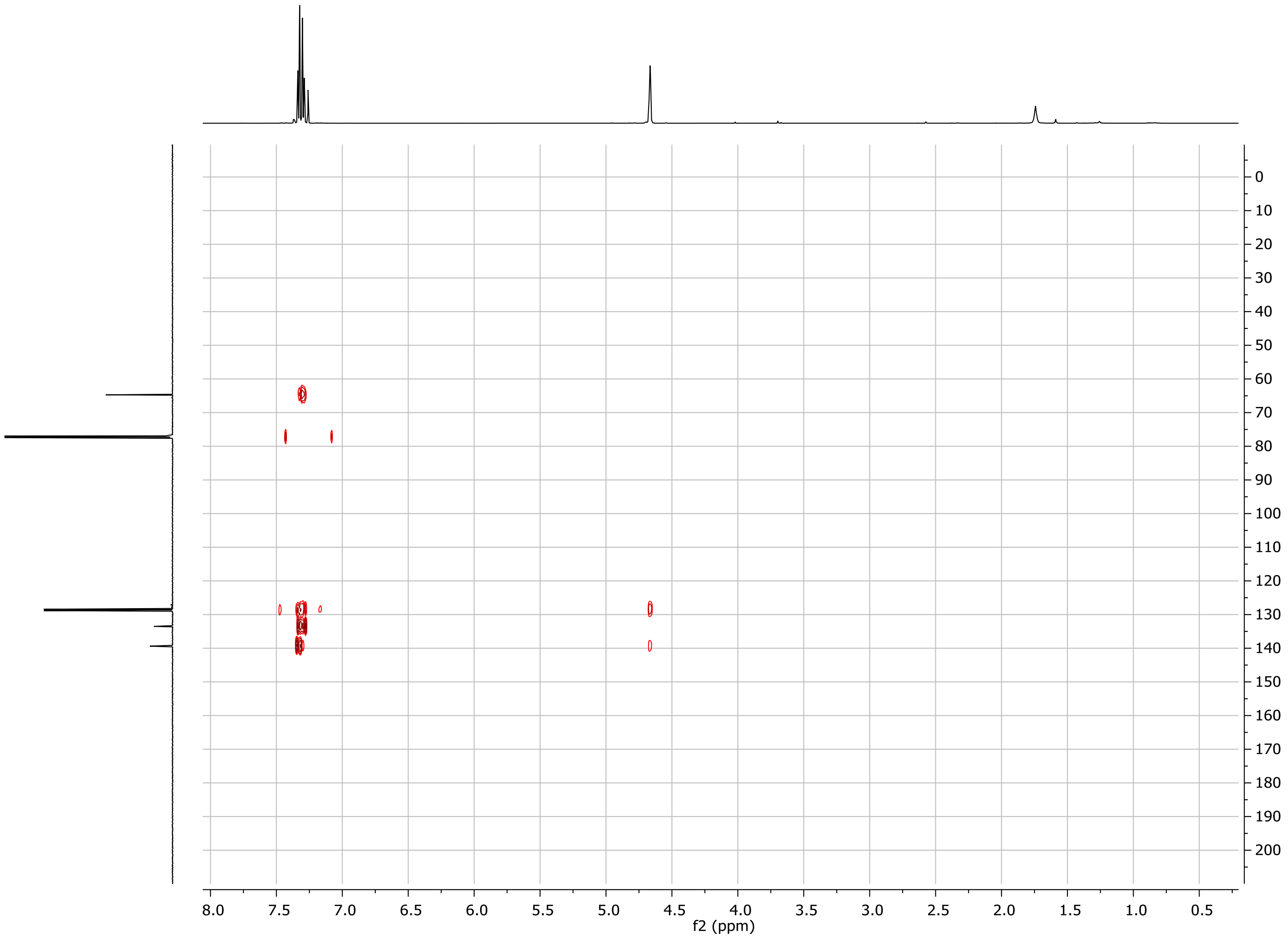

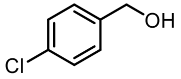

2s

^{1}H, ^{13}C HSQC

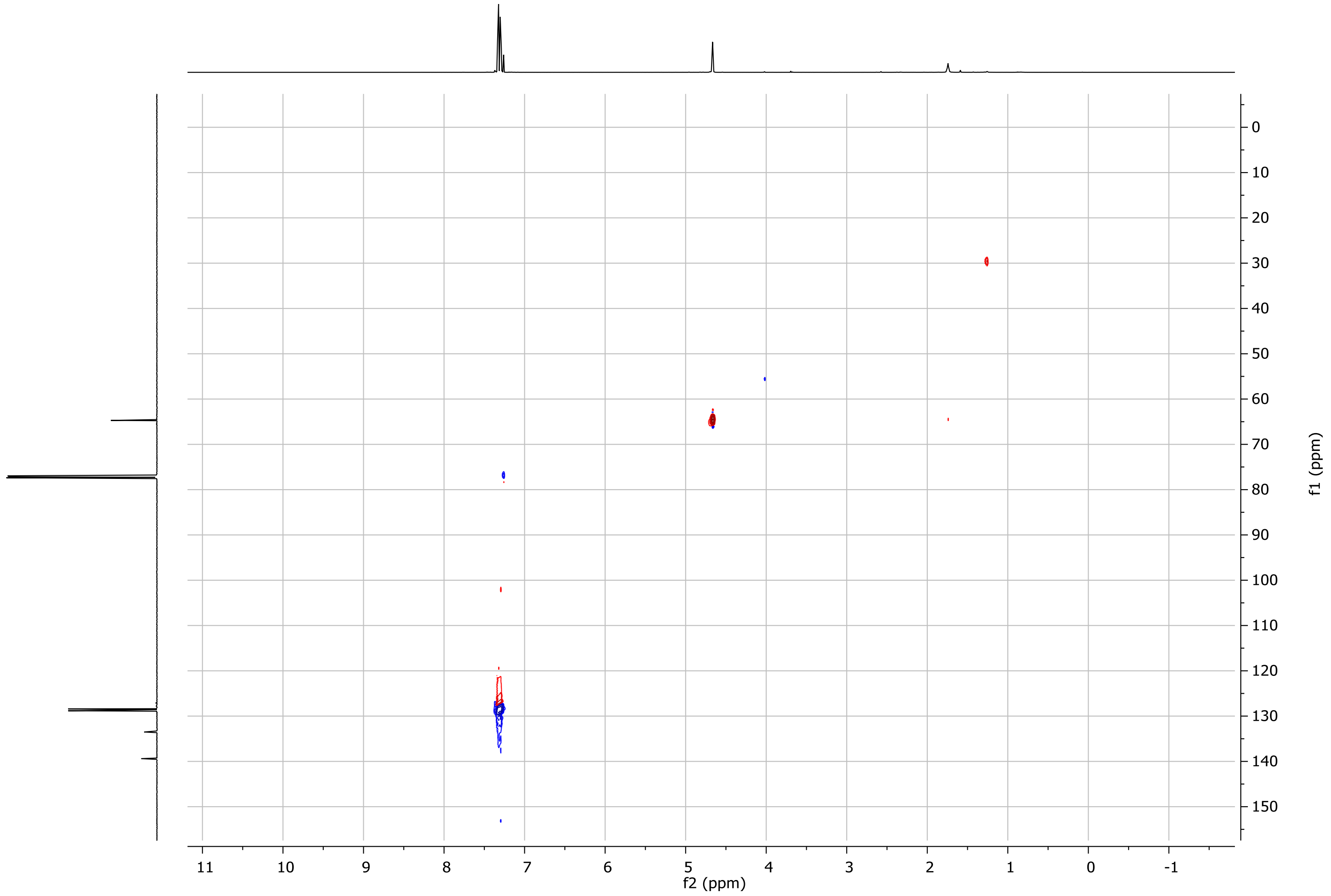

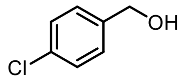

2s

HRMS

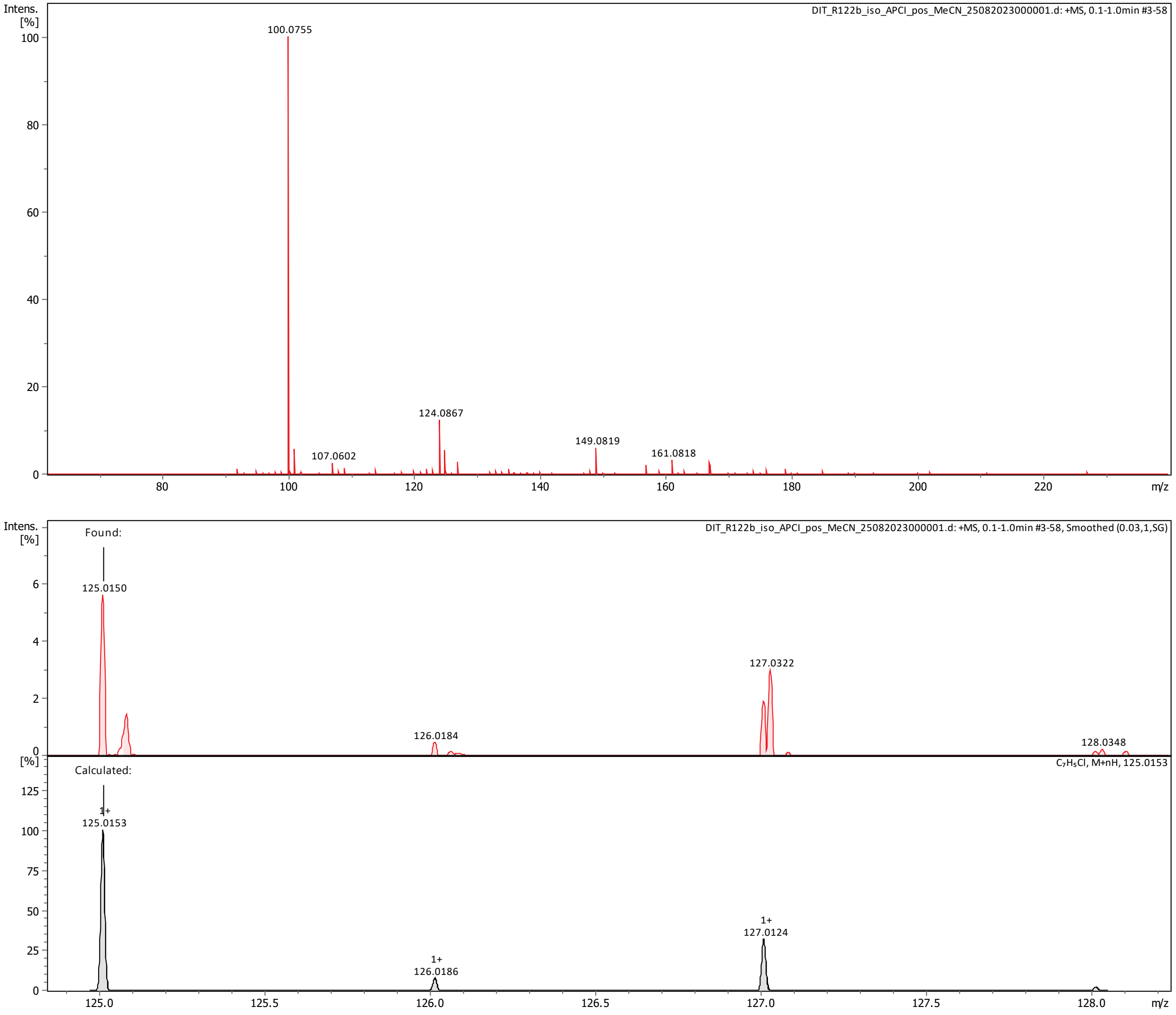

Intens. [%]
DIT_R122b_iso_APCI_pos_MeCN_25082023000001.d: +MS, 0.1-1.0min #3-58
100.0755
124.0867
107.0602
149.0819
161.0818
m/z
Found:
DIT_R122b_iso_APCI_pos_MeCN_25082023000001.d: +MS, 0.1-1.0min #3-58, Smoothed (0.03,1,SG)
125.0150
126.0184
127.0322
128.0348
Calculated:
C₇H₆Cl, M+nH, 125.0153
1+
125.0153
1+
126.0186
1+
127.0124

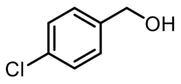

Cl
OH

2s

62 (4-(4,4,5,5-Tetramethyl-1,3,2-dioxaborolan-2-yl)phenyl)methanol (2t)

^{1}H NMR

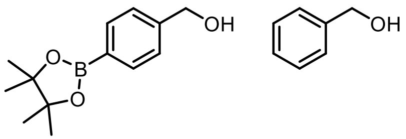

2t **2t'**

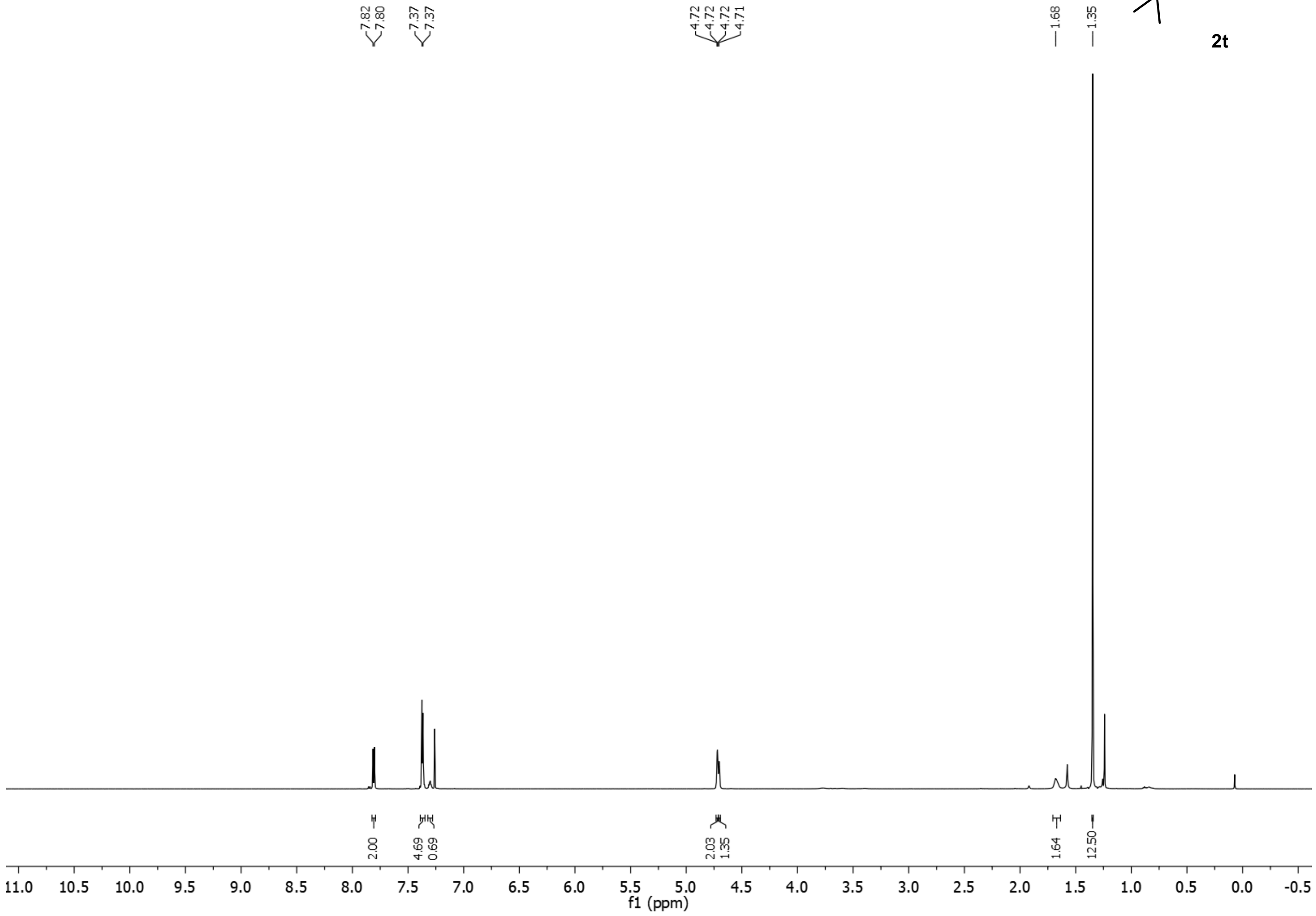

^{13}C NMR

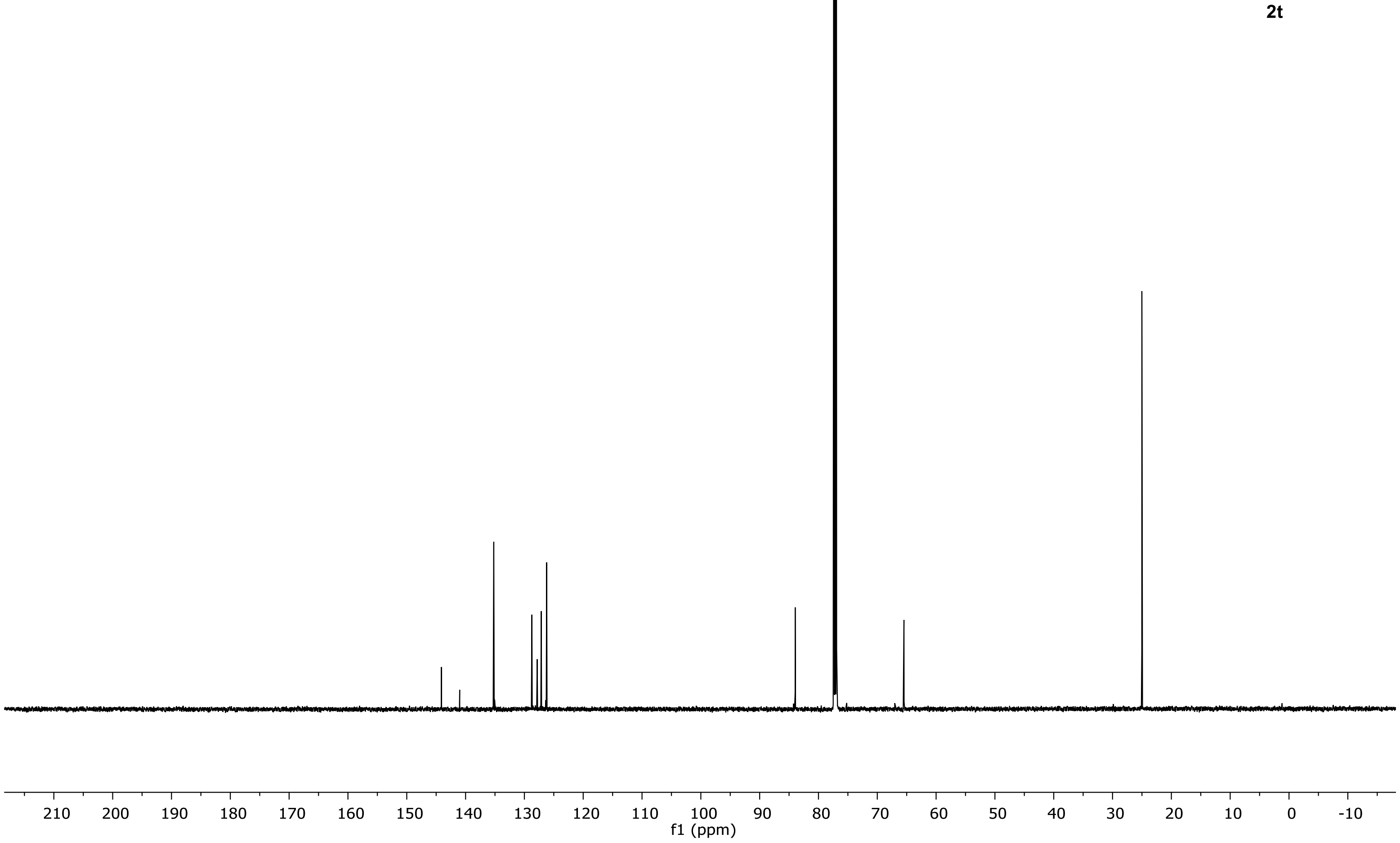
144.1
141.0
135.2
135.1
128.7
127.8
127.1
126.4
126.2
84.0
65.5
25.0
210 200 190 180 170 160 150 140 130 120 110 100 90 80 70 60 50 40 30 20 10 0 -10
f1 (ppm)

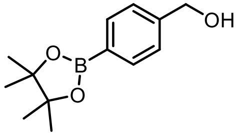
O
B
O
OH

2t

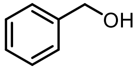
OH

2t'

^{11}B NMR

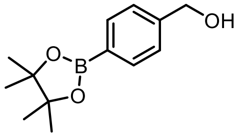

2t

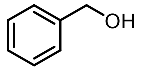

2t'

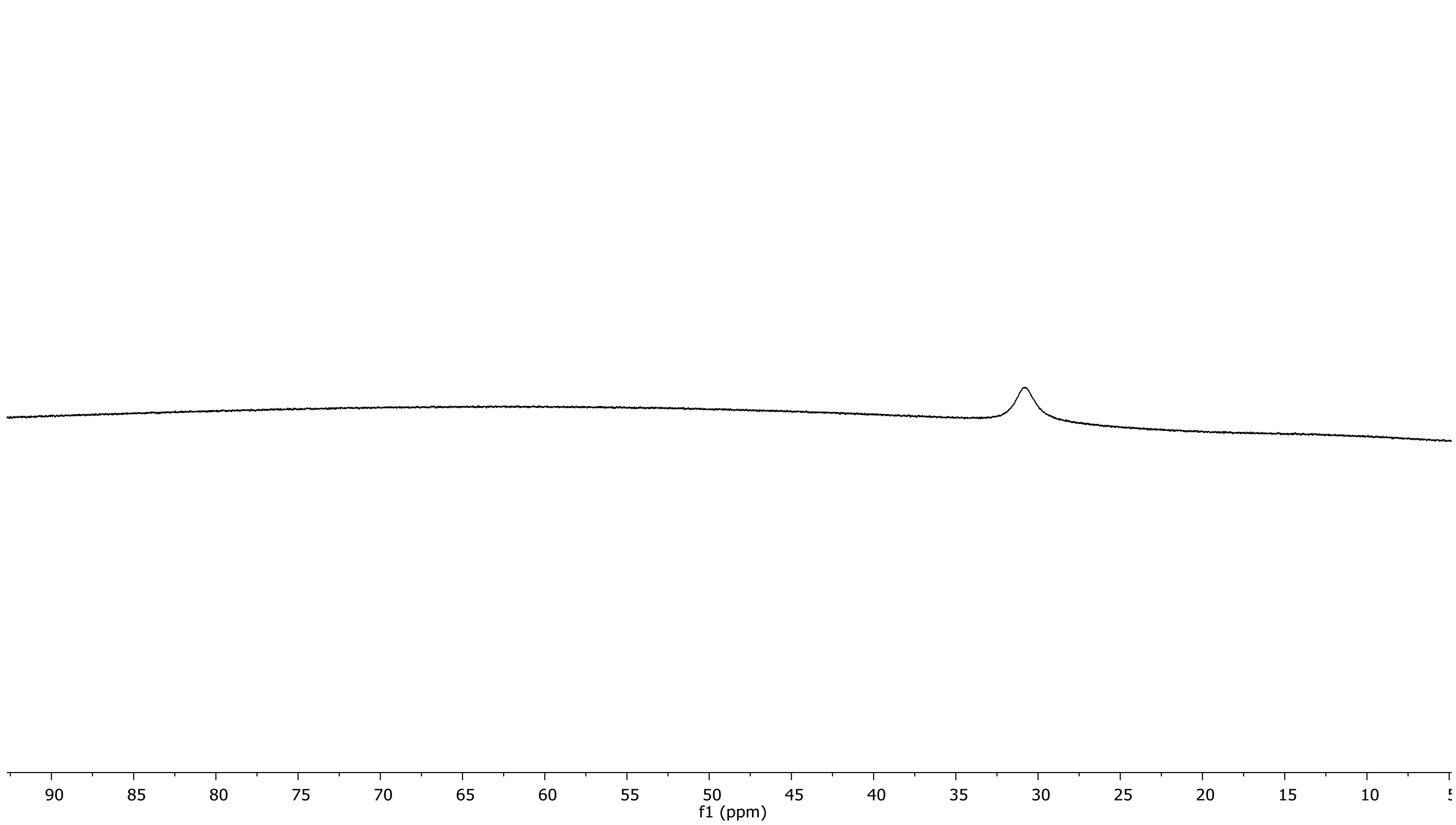

^{1}H, ^{1}H COSY

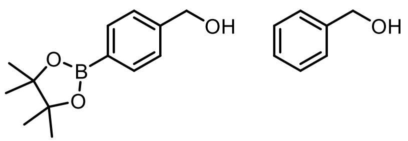

2t

2t'

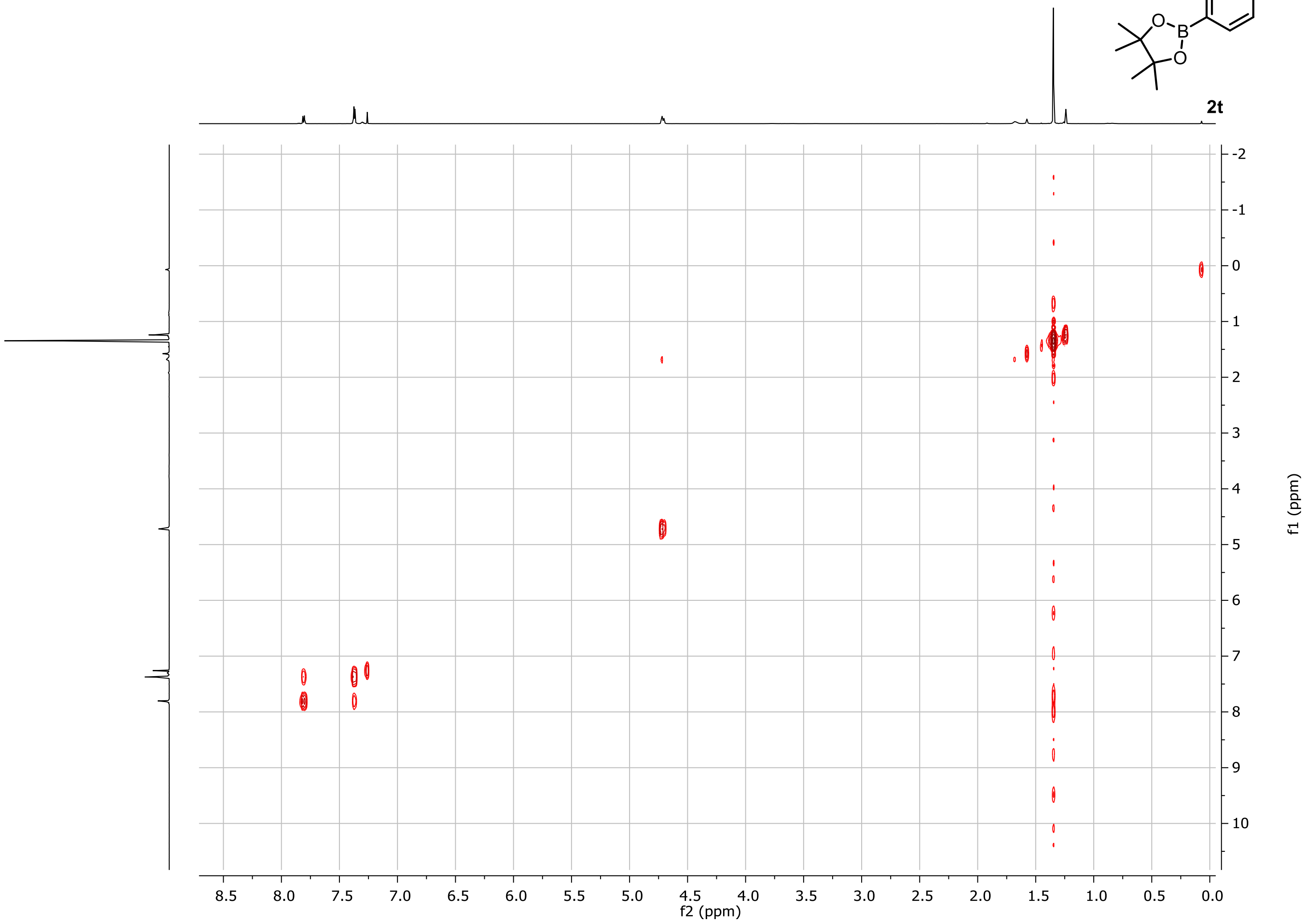

^{1}H, ^{13}C HMBC

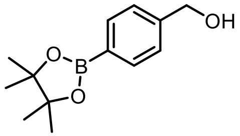

2t

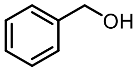

2t'

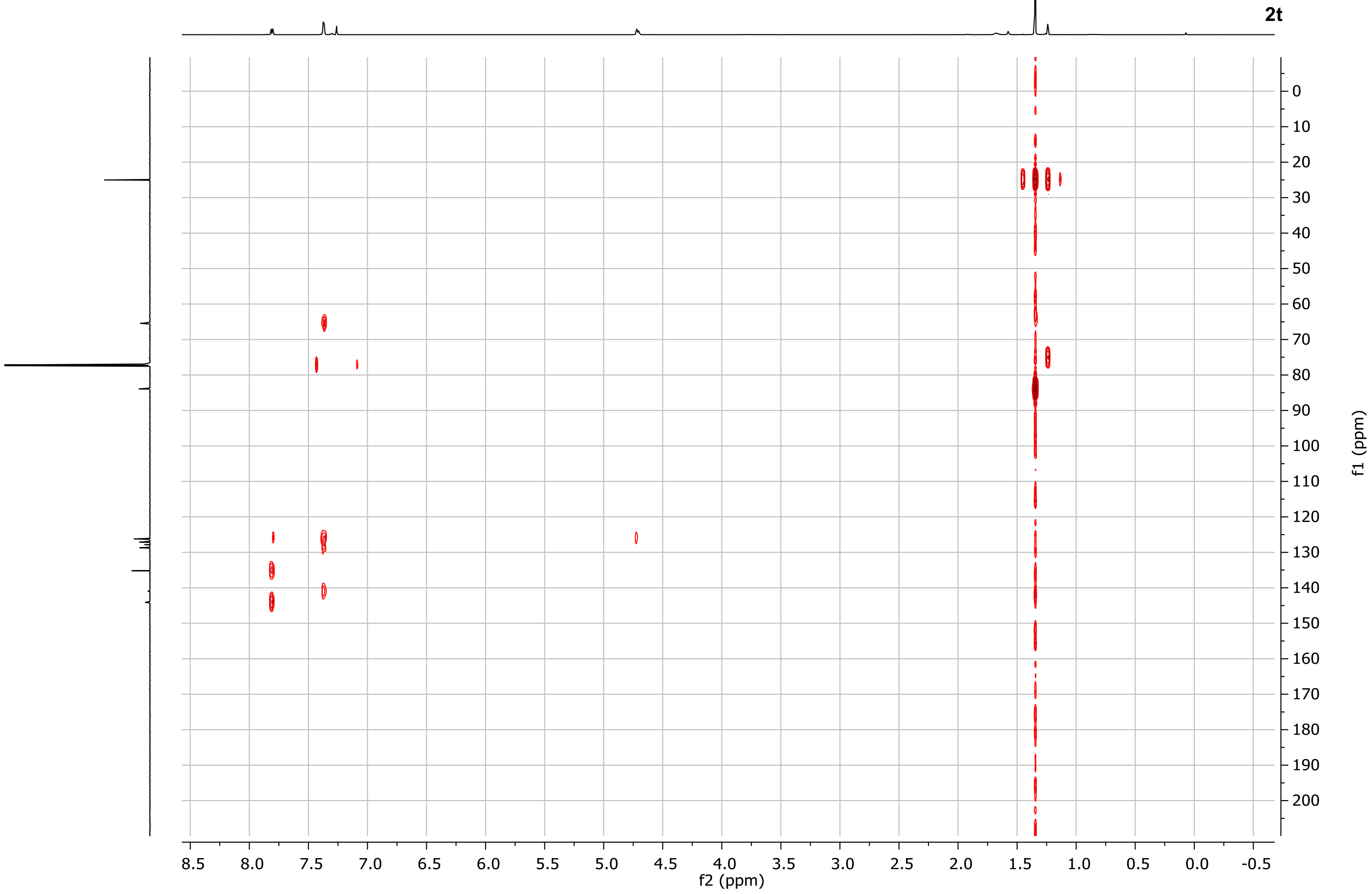

^{1}H, ^{13}C HSQC

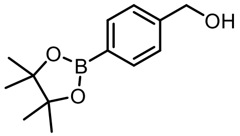

2t

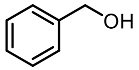

2t'

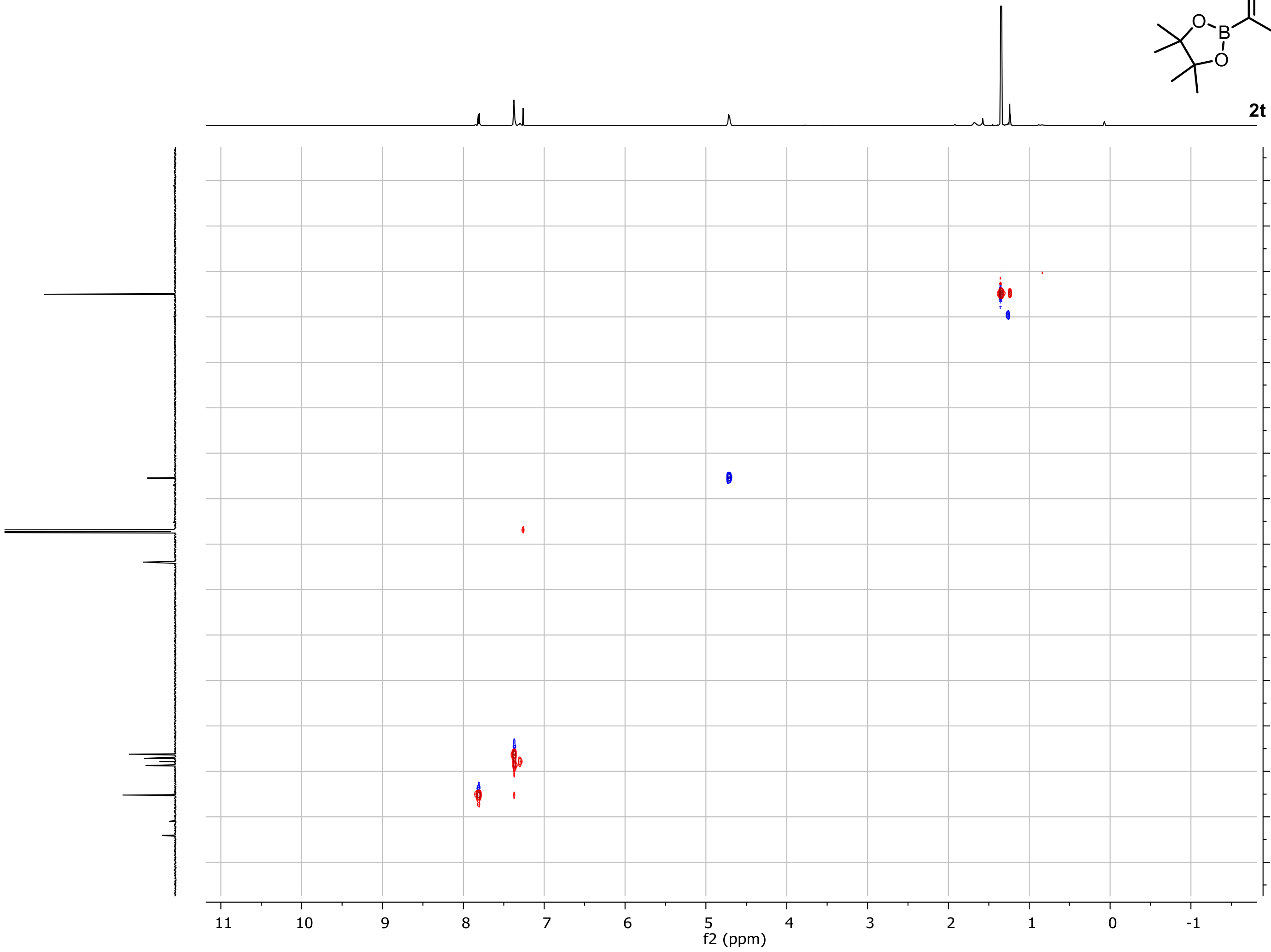

HRMS

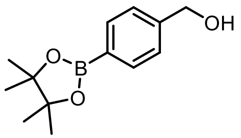

2t

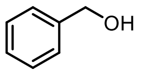

2t'

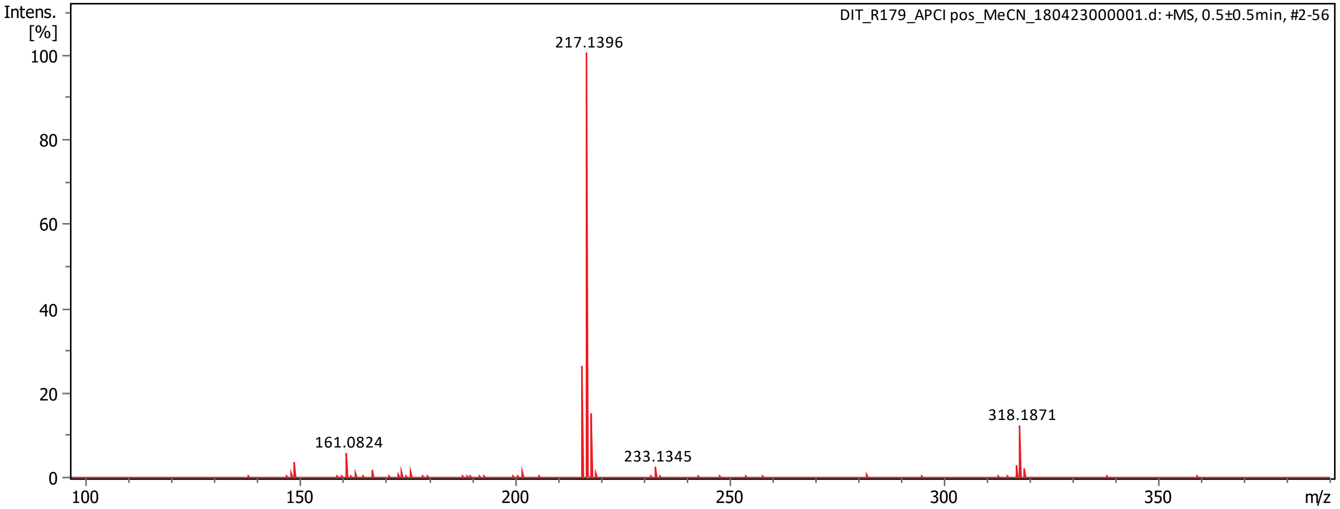

Intens. [%]
DIT_R179_APCI pos_MeCN_180423000001.d: +MS, 0.5±0.5min, #2-56
217.1396
161.0824
233.1345
318.1871
m/z

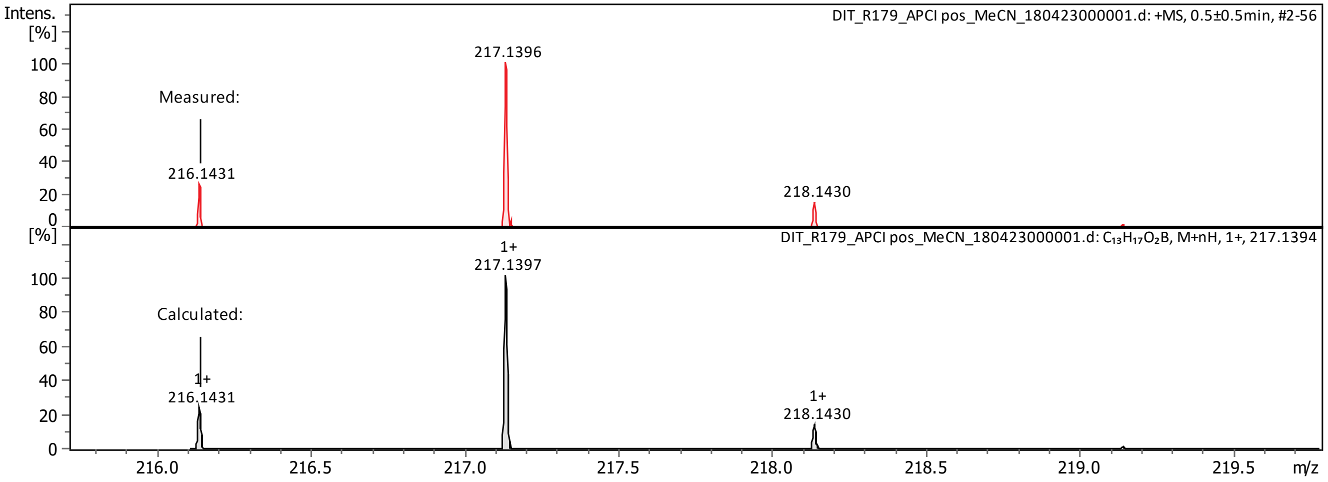

Intens. [%]
DIT_R179_APCI pos_MeCN_180423000001.d: +MS, 0.5±0.5min, #2-56
Measured:
216.1431
217.1396
218.1430
DIT_R179_APCI pos_MeCN_180423000001.d: C13H17O2B, M+nH, 1+, 217.1394
Calculated:
1+ 216.1431
1+ 217.1397
1+ 218.1430
m/z

63 Furan-2-ylmethanol (2u)

^{1}H NMR

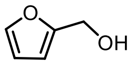

2u

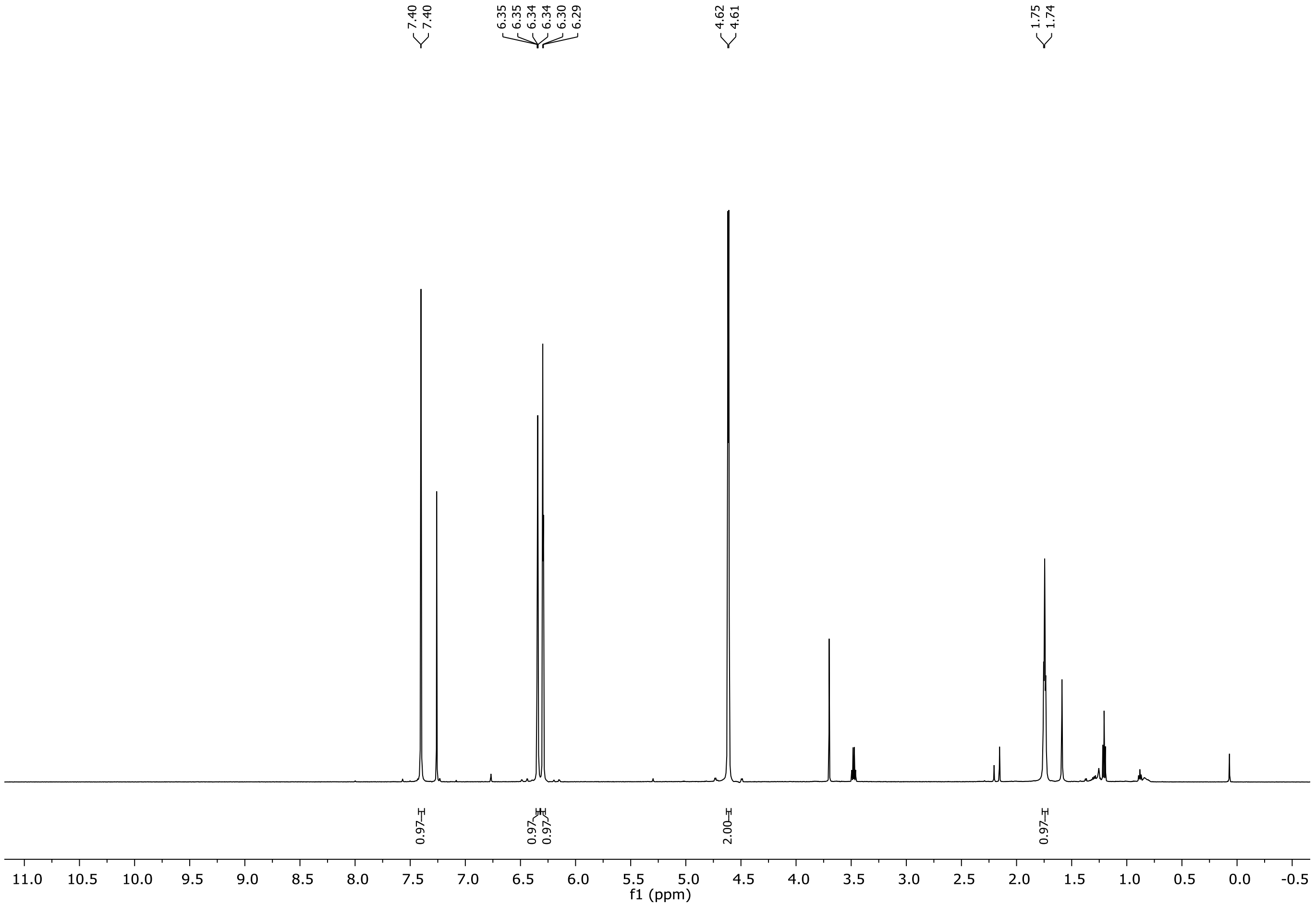

^{13}C NMR

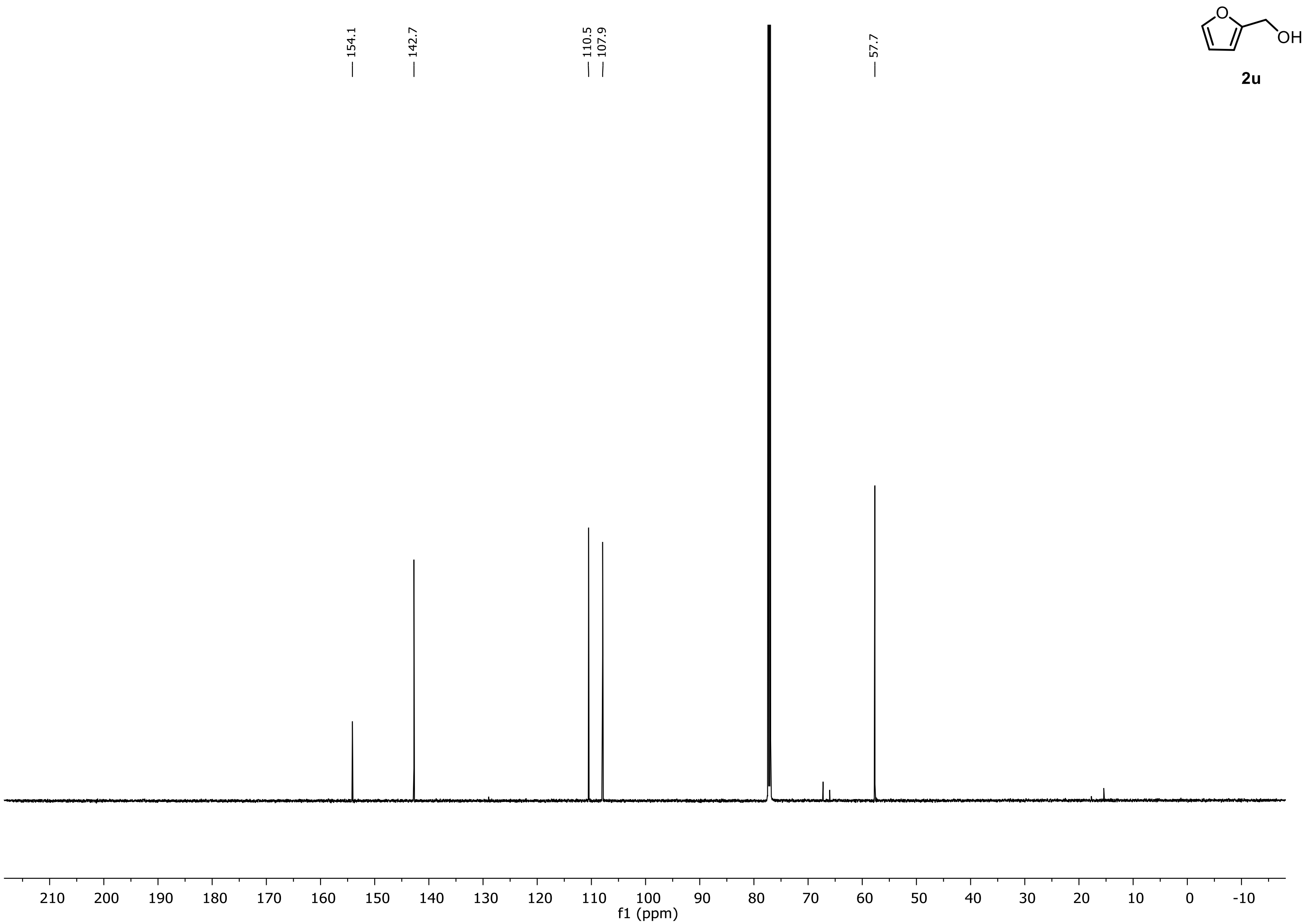

154.1
142.7
110.5
107.9
57.7
OH
O
2u
210
200
190
180
170
160
150
140
130
120
110
100
90
80
70
60
50
40
30
20
10
0
-10
f1 (ppm)

^{1}H, ^{1}H COSY

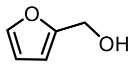

2u

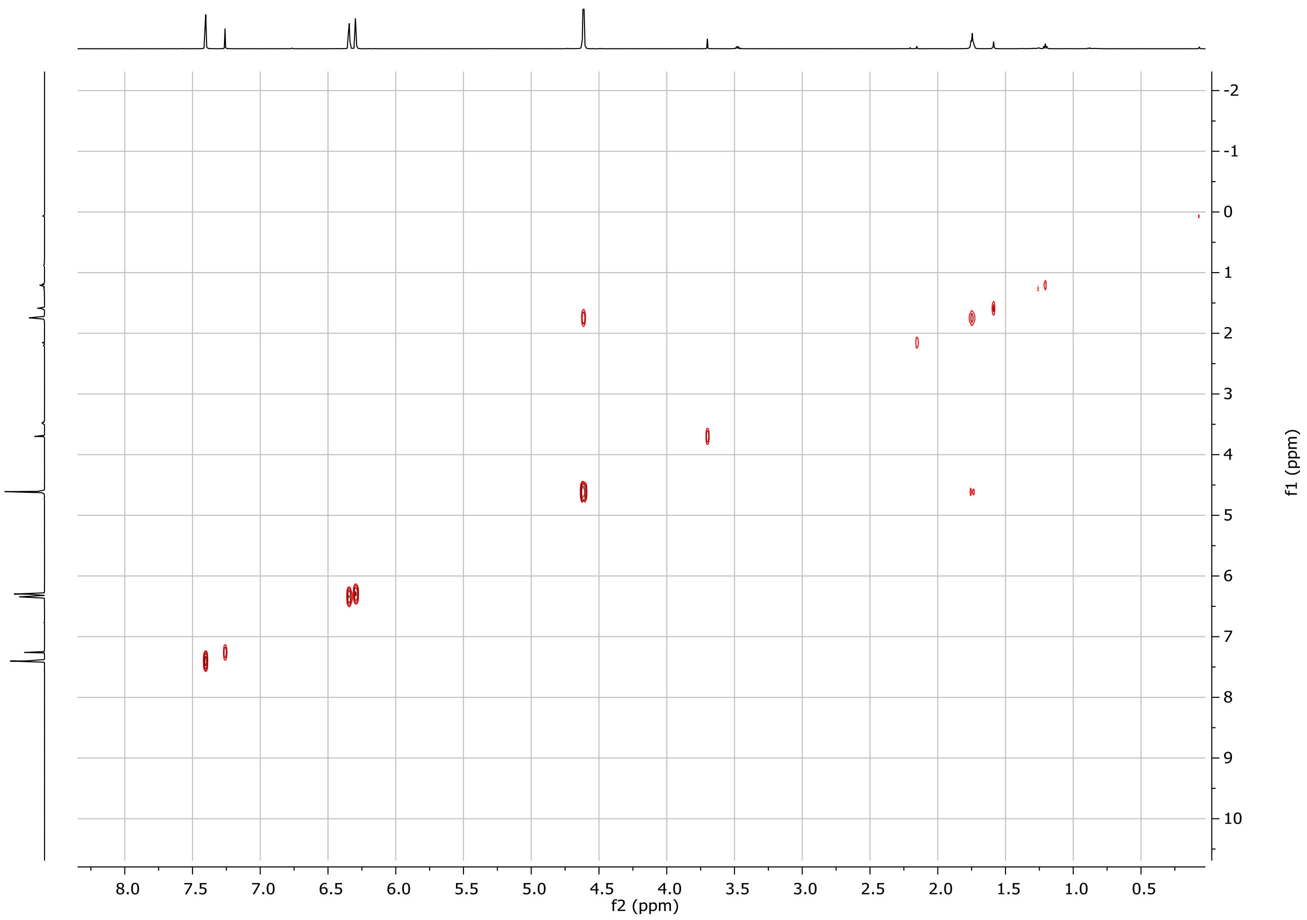

8.0
7.5
7.0
6.5
6.0
5.5
5.0
4.5
4.0
3.5
3.0
2.5
2.0
1.5
1.0
0.5
f2 (ppm)
-2
-1
0
1
2
3
4
5
6
7
8
9
10
f1 (ppm)

^1H, ^{13}C HMBC

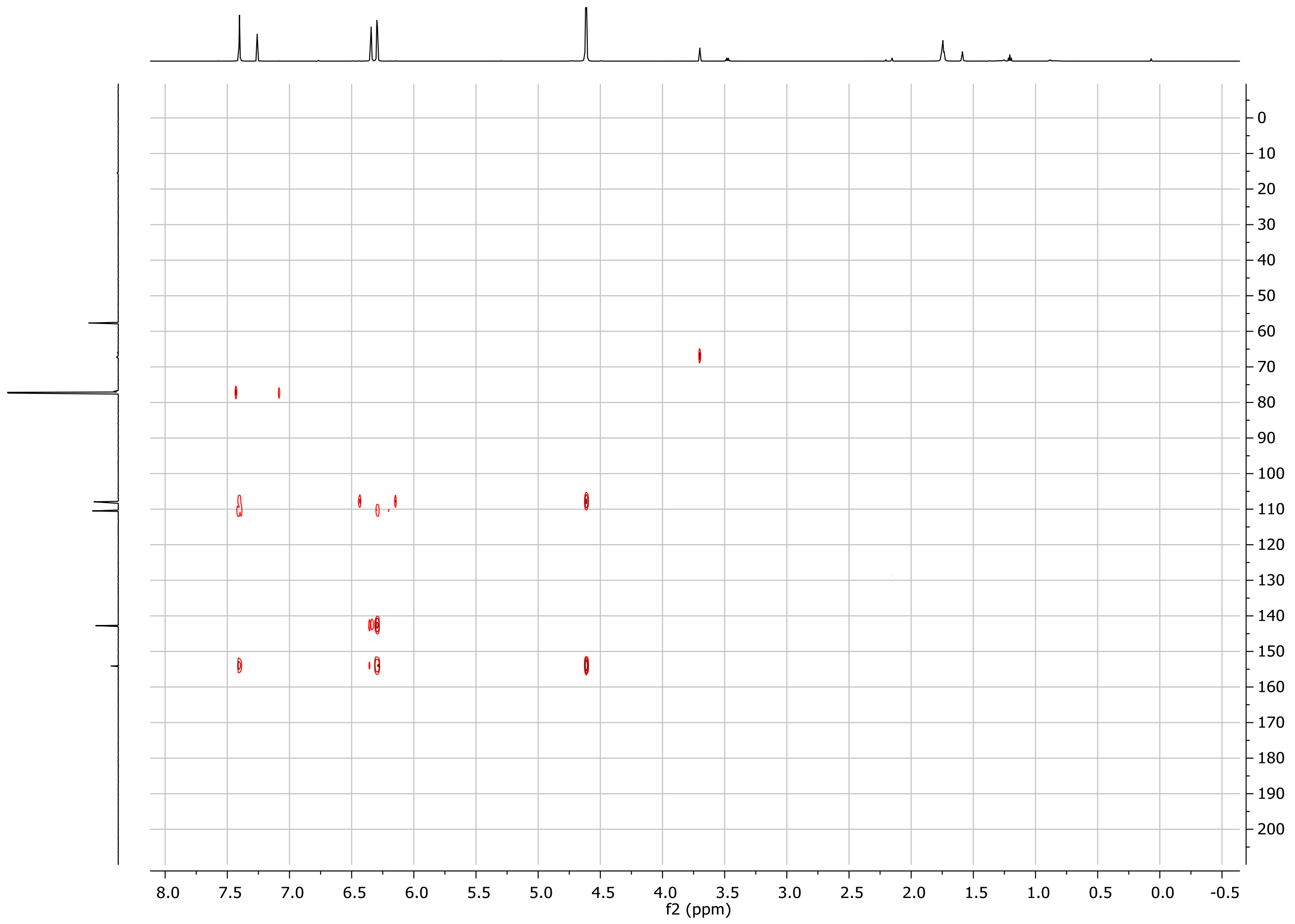

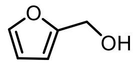

2u

^{1}H, ^{13}C HSQC

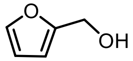

2u

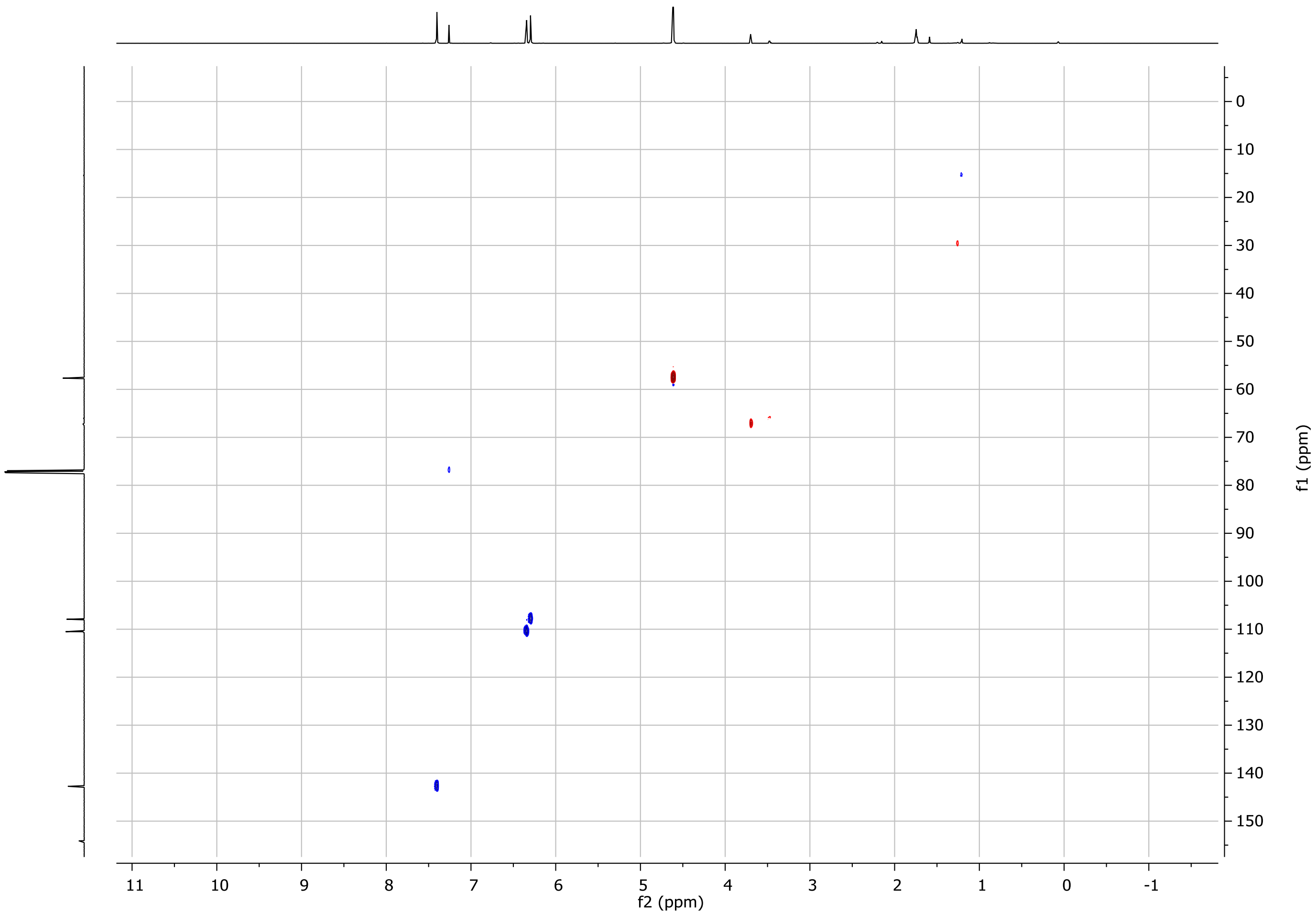

HRMS

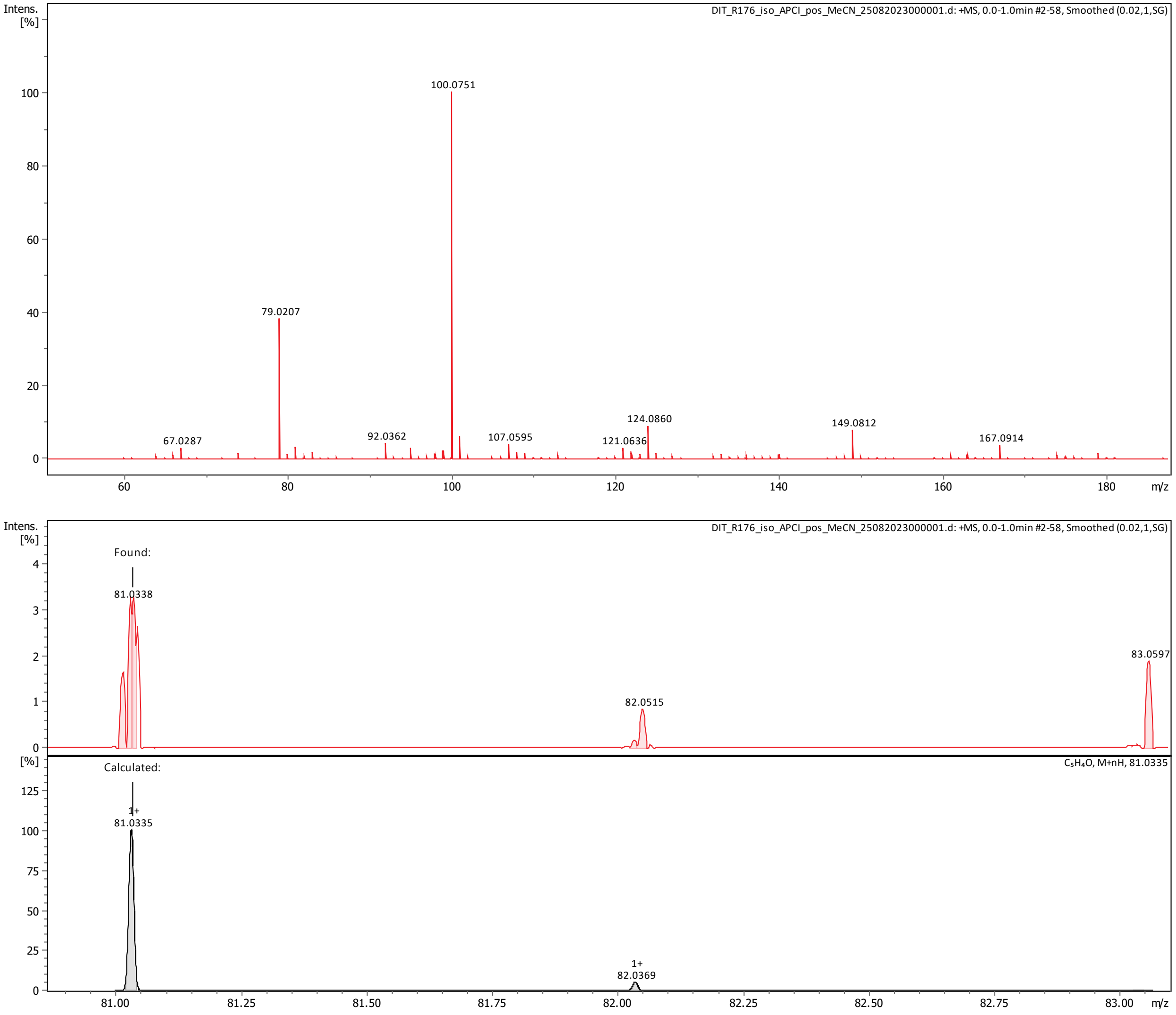

Intens. [%]
DIT_R176_iso_APCI_pos_MeCN_25082023000001.d: +MS, 0.0-1.0min #2-58, Smoothed (0.02,1,SG)
100.0751
79.0207
67.0287
92.0362
107.0595
121.0636
124.0860
149.0812
167.0914
m/z
Found:
81.0338
82.0515
83.0597
Calculated:
C_5H_4O, M+nH, 81.0335
1+
81.0335
1+
82.0369

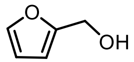

2u

64 Thiophen-2-ylmethanol (2v)

^{1}H NMR

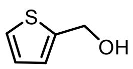

2v

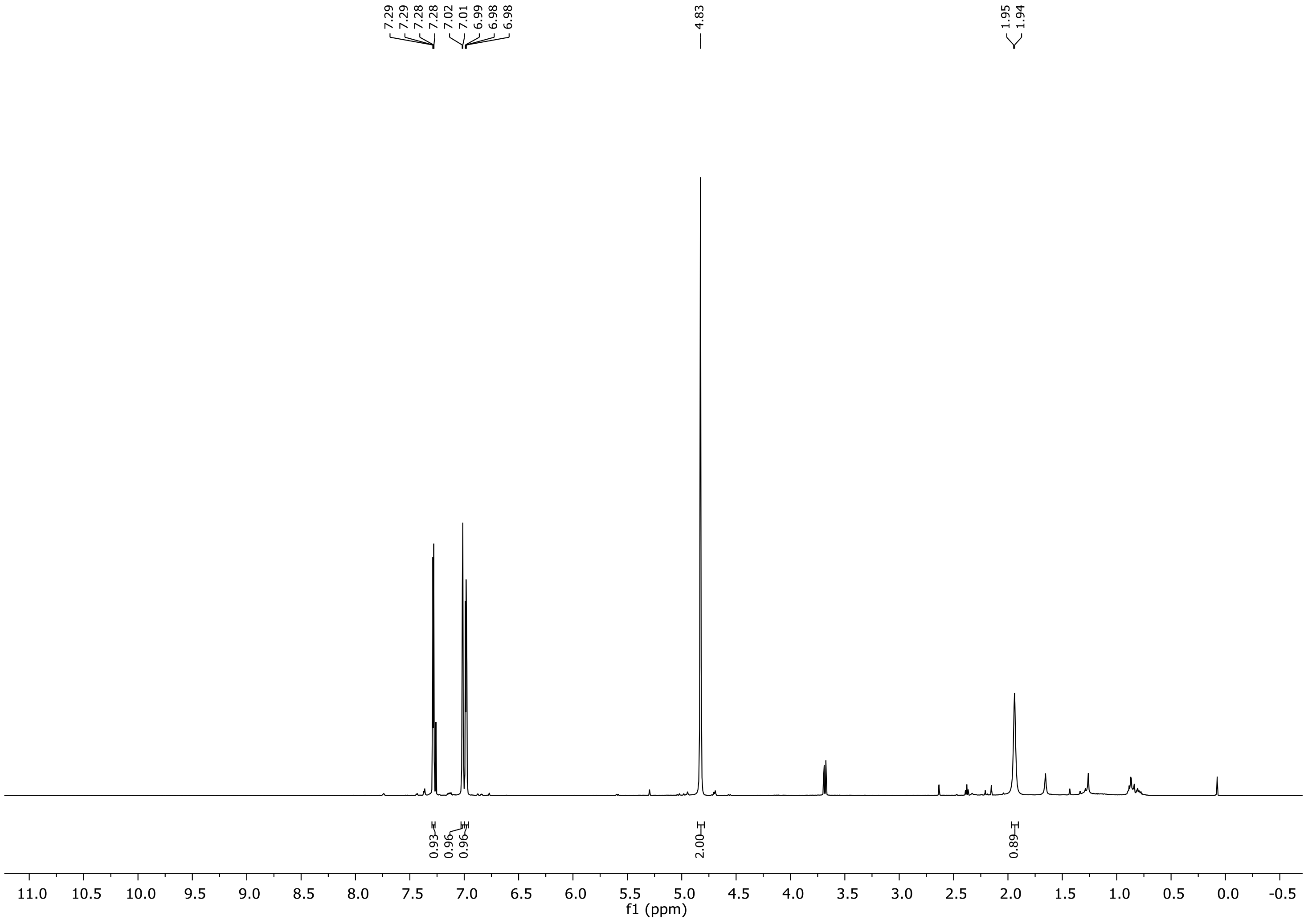

^{13}C NMR

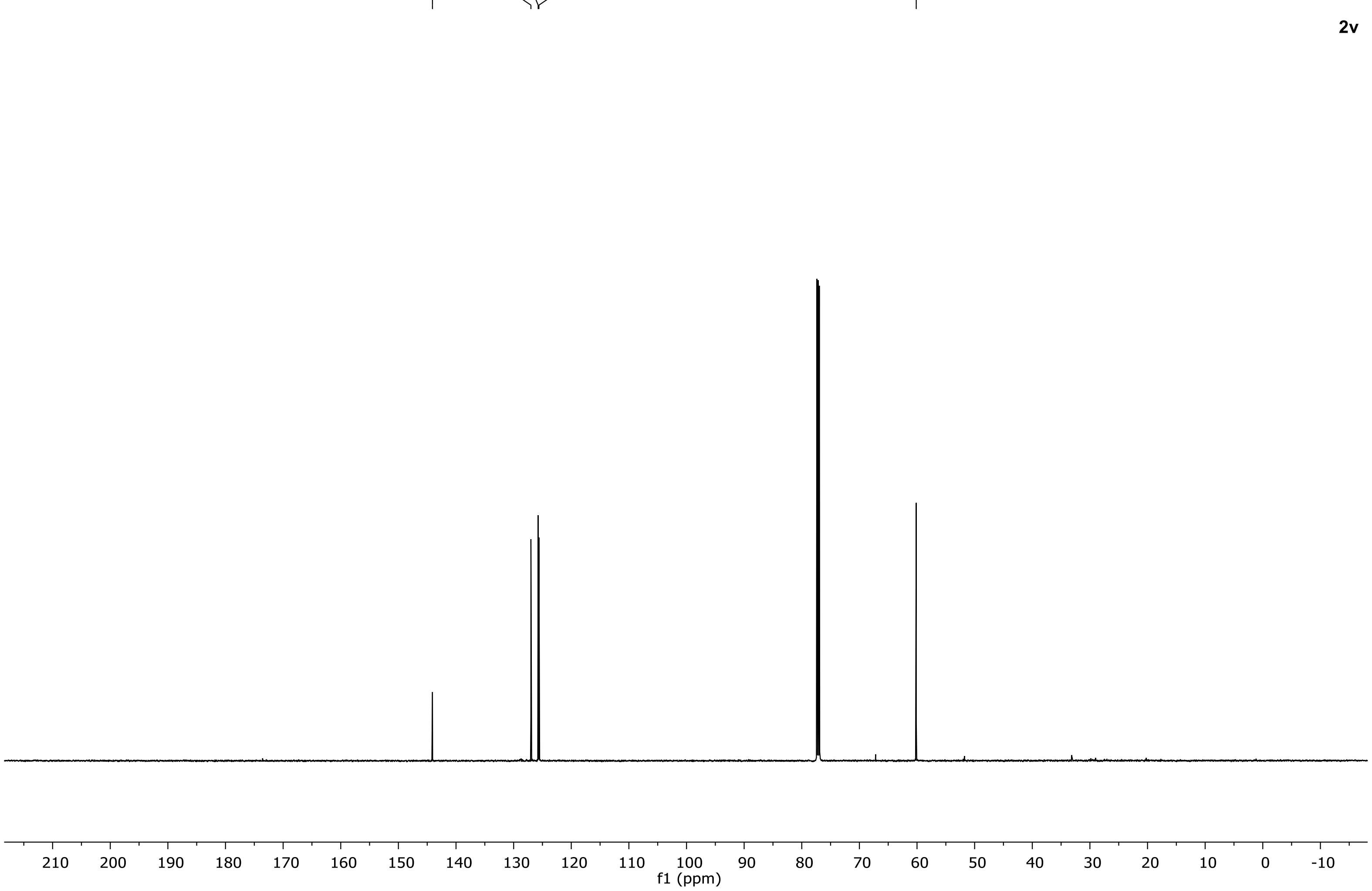

144.1
127.0
125.7
125.6
60.1
210 200 190 180 170 160 150 140 130 120 110 100 90 80 70 60 50 40 30 20 10 0 -10
f1 (ppm)

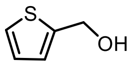

S
OH

2v

^1H, ^1H COSY

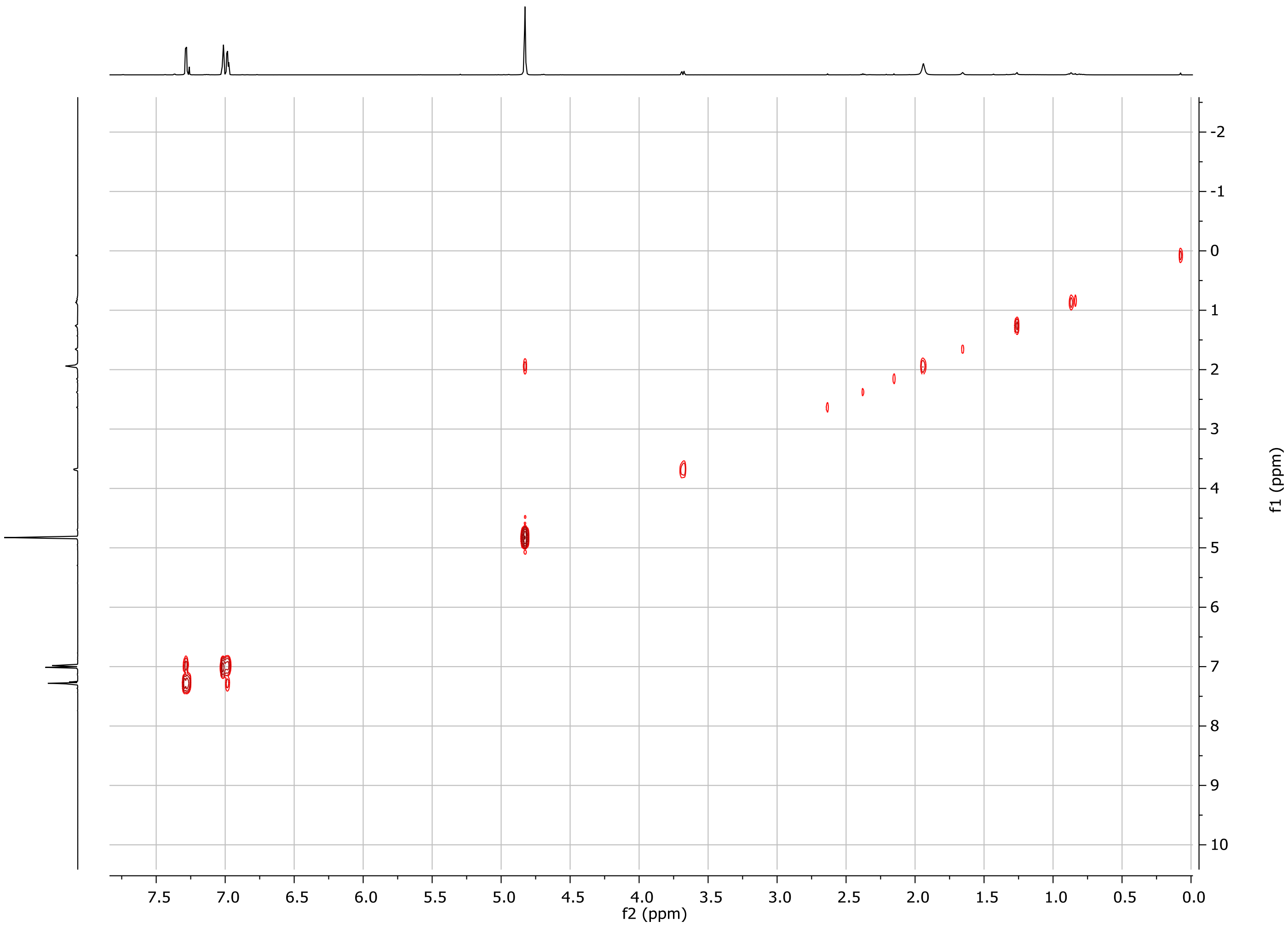

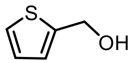

2v

^{1}H, ^{13}C HMBC

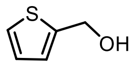

2v

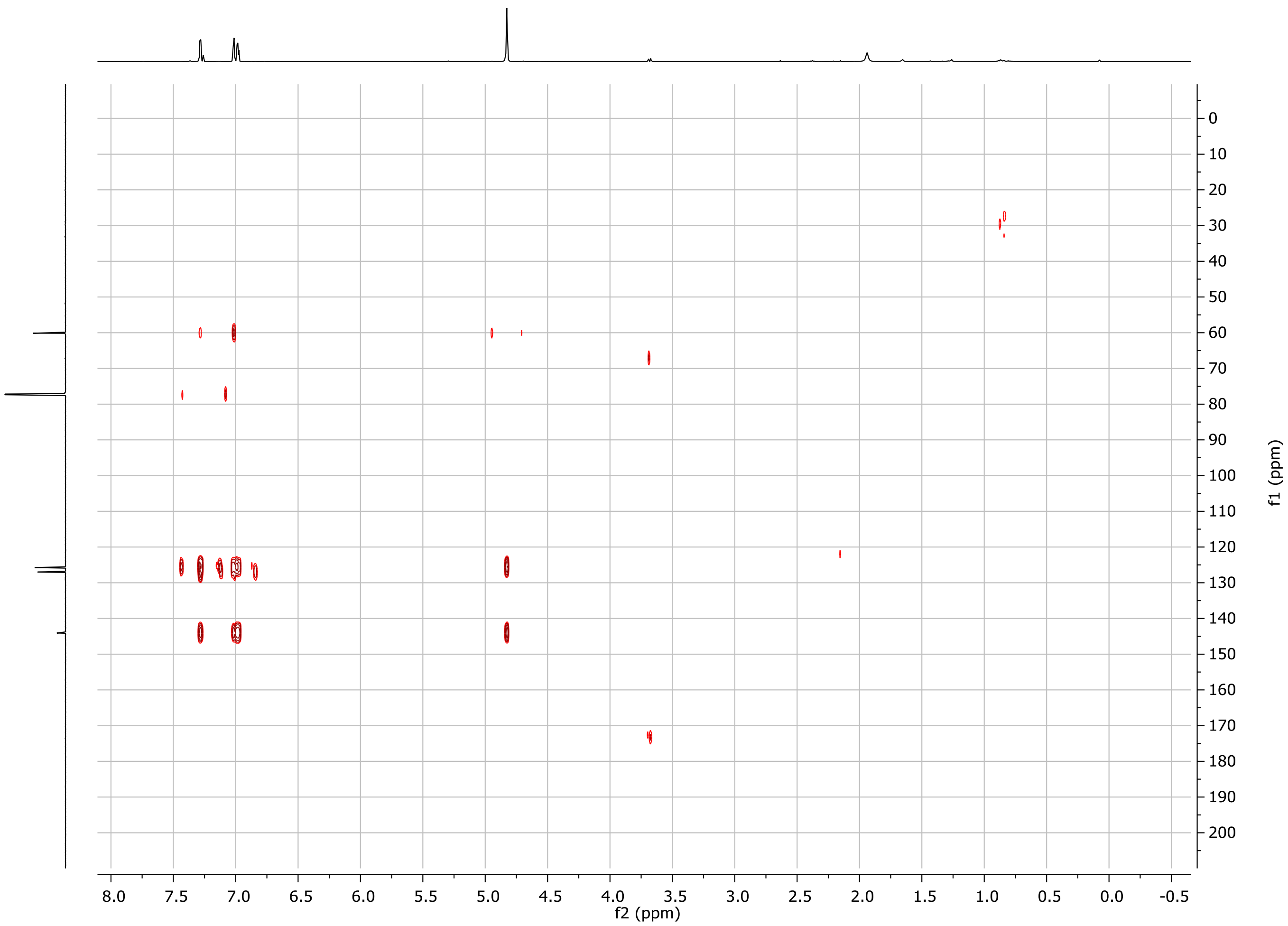

^{1}H, ^{13}C HSQC

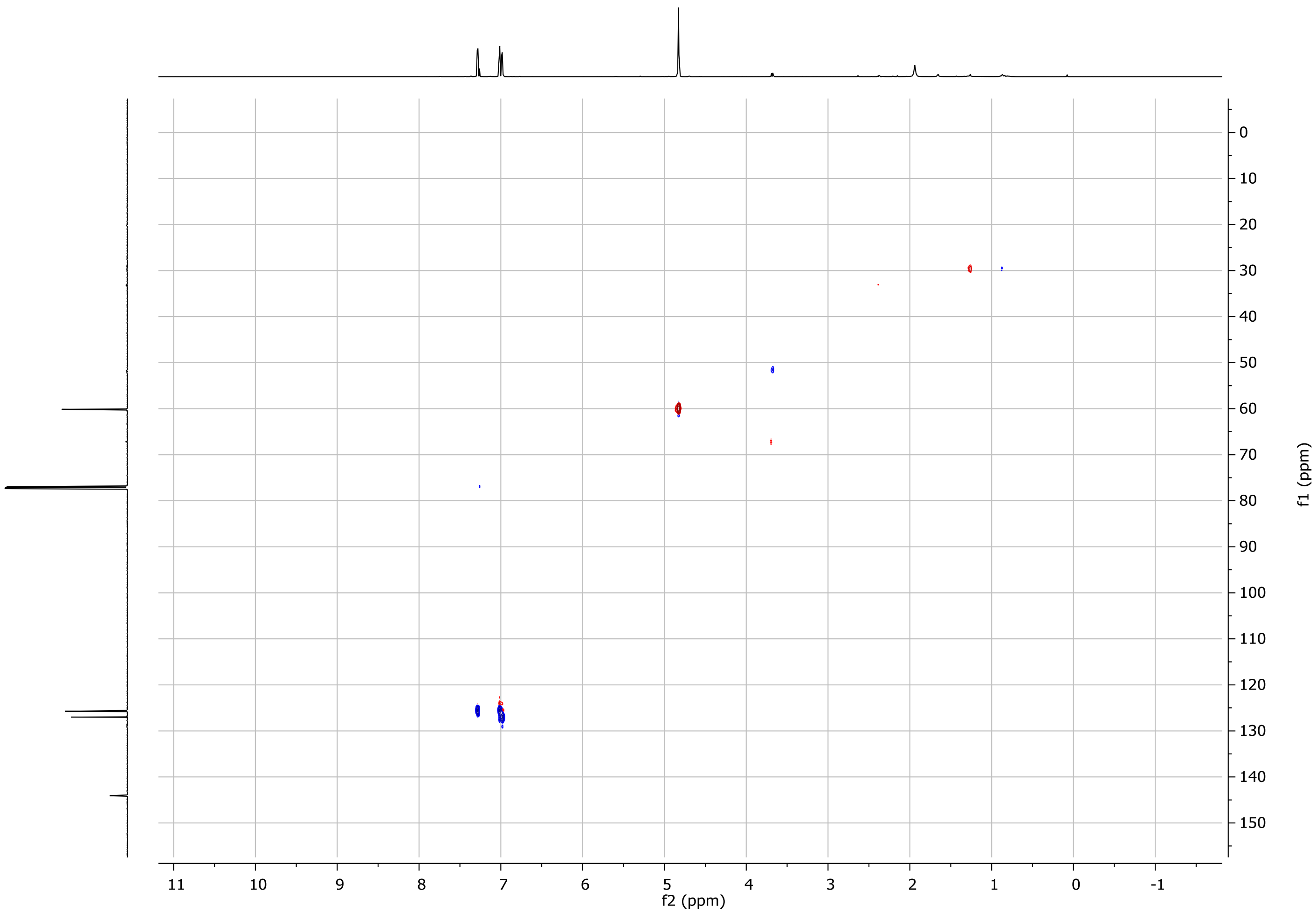

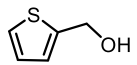

2v

HRMS

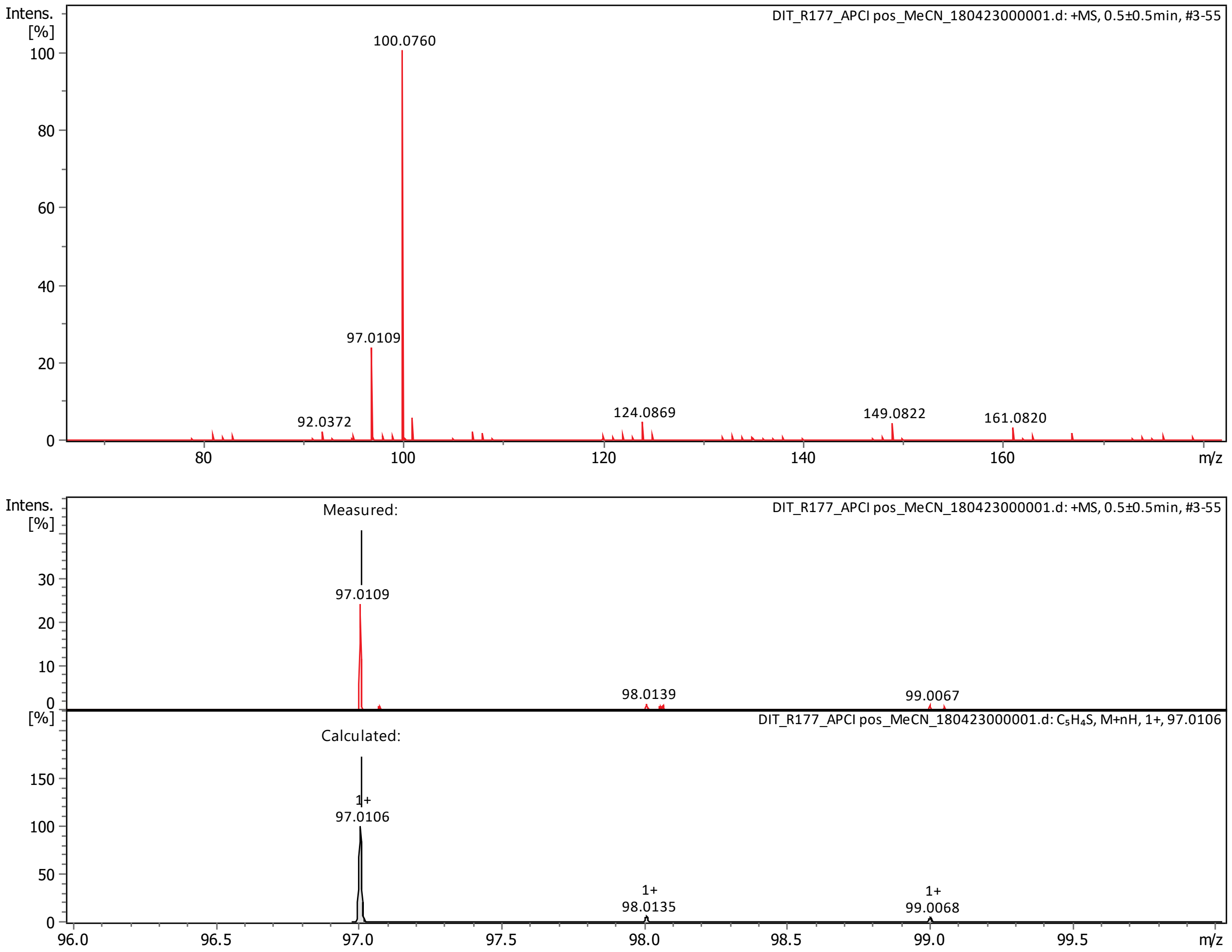

Intens. [%]
DIT_R177_APCI pos_MeCN_180423000001.d: +MS, 0.5±0.5min, #3-55
100.0760
97.0109
92.0372
124.0869
149.0822
161.0820
m/z
Measured:
97.0109
98.0139
99.0067
DIT_R177_APCI pos_MeCN_180423000001.d: C₅H₄S, M+nH, 1+, 97.0106
Calculated:
1+
97.0106
1+
98.0135
1+
99.0068

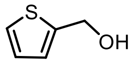

S
OH

2v

65 (5-((Benzyloxy)methyl)furan-2-yl)methanol (2w)

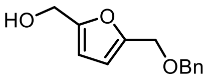

2w

^{1}H NMR

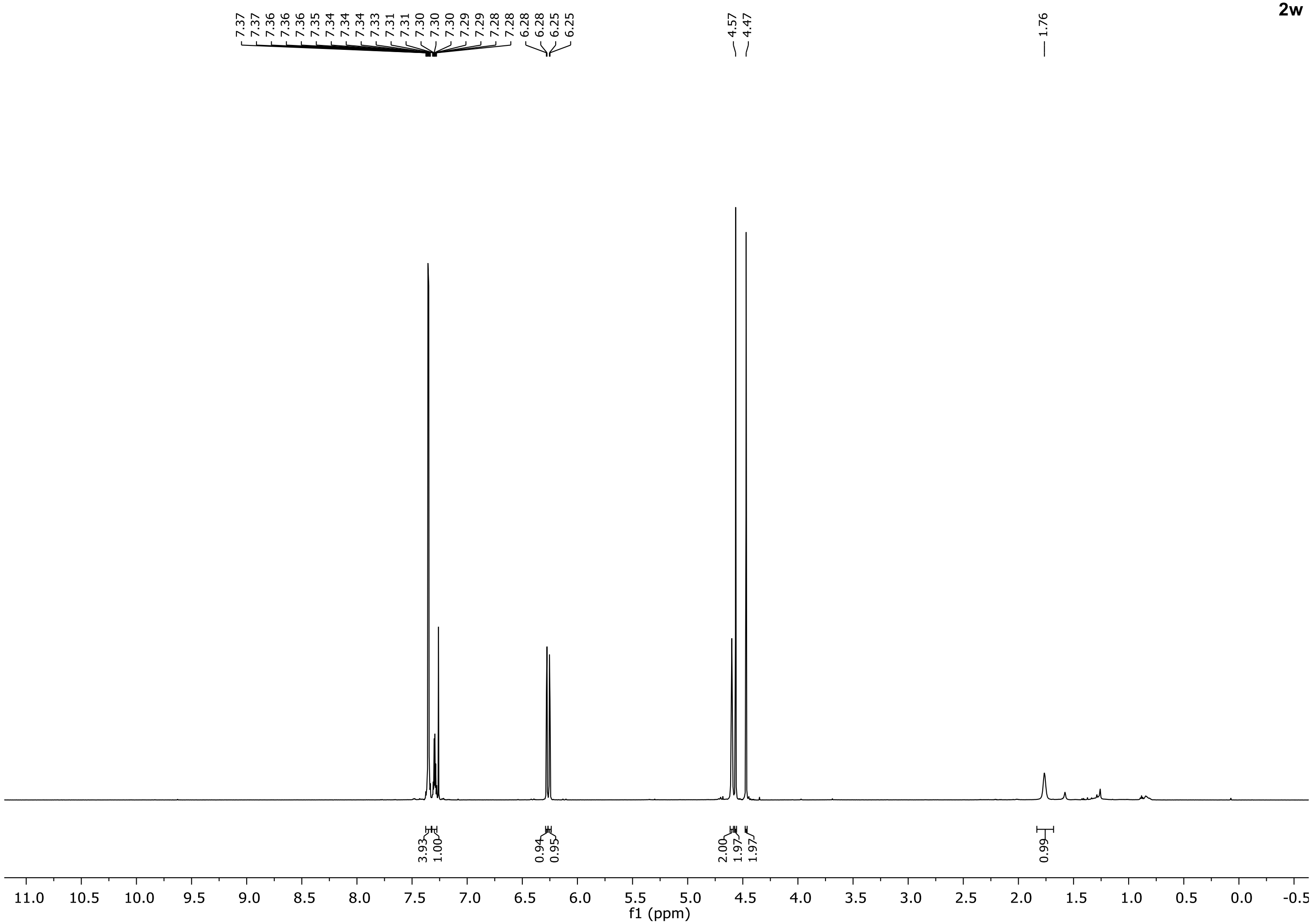

^{13}C NMR

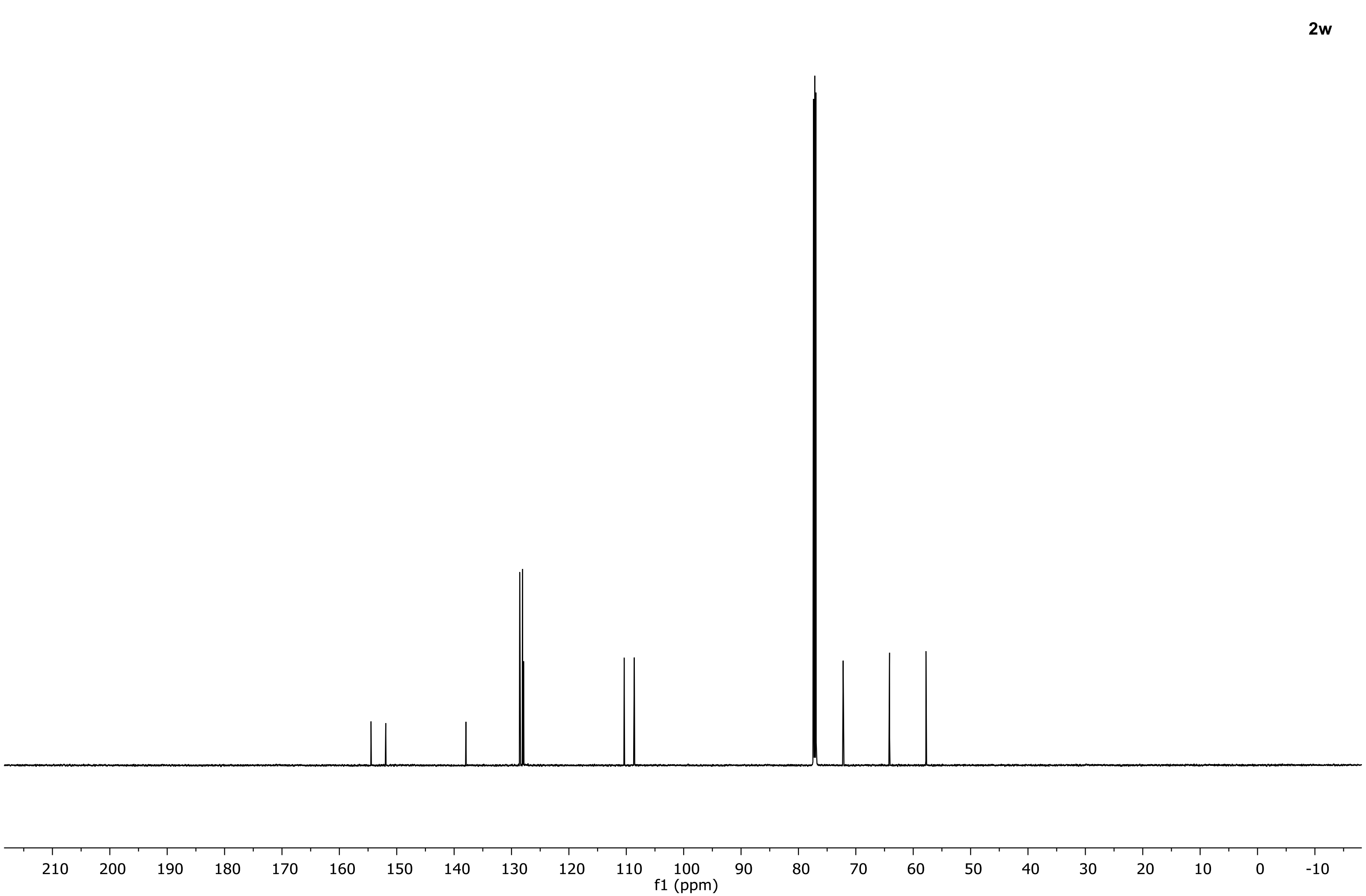
154.5
151.9
137.9
128.6
128.1
127.9
110.4
108.6
77.4
72.2
64.1
57.8
210 200 190 180 170 160 150 140 130 120 110 100 90 80 70 60 50 40 30 20 10 0 -10
f1 (ppm)

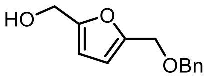
HO
O
OBn

2w

^{1}H, ^{1}H COSY

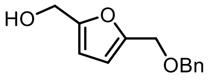

2w

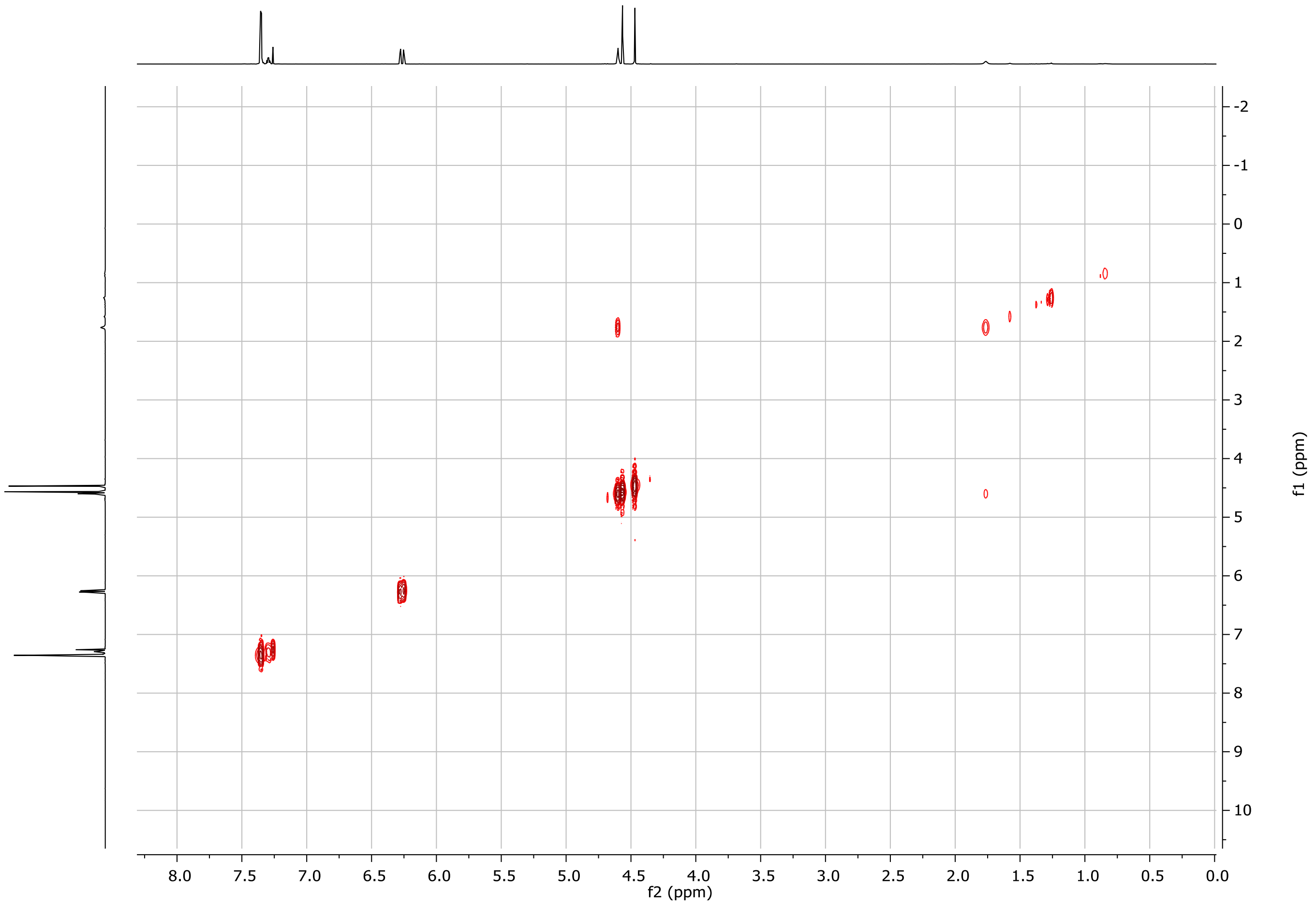

^{1}H, ^{13}C HMBC

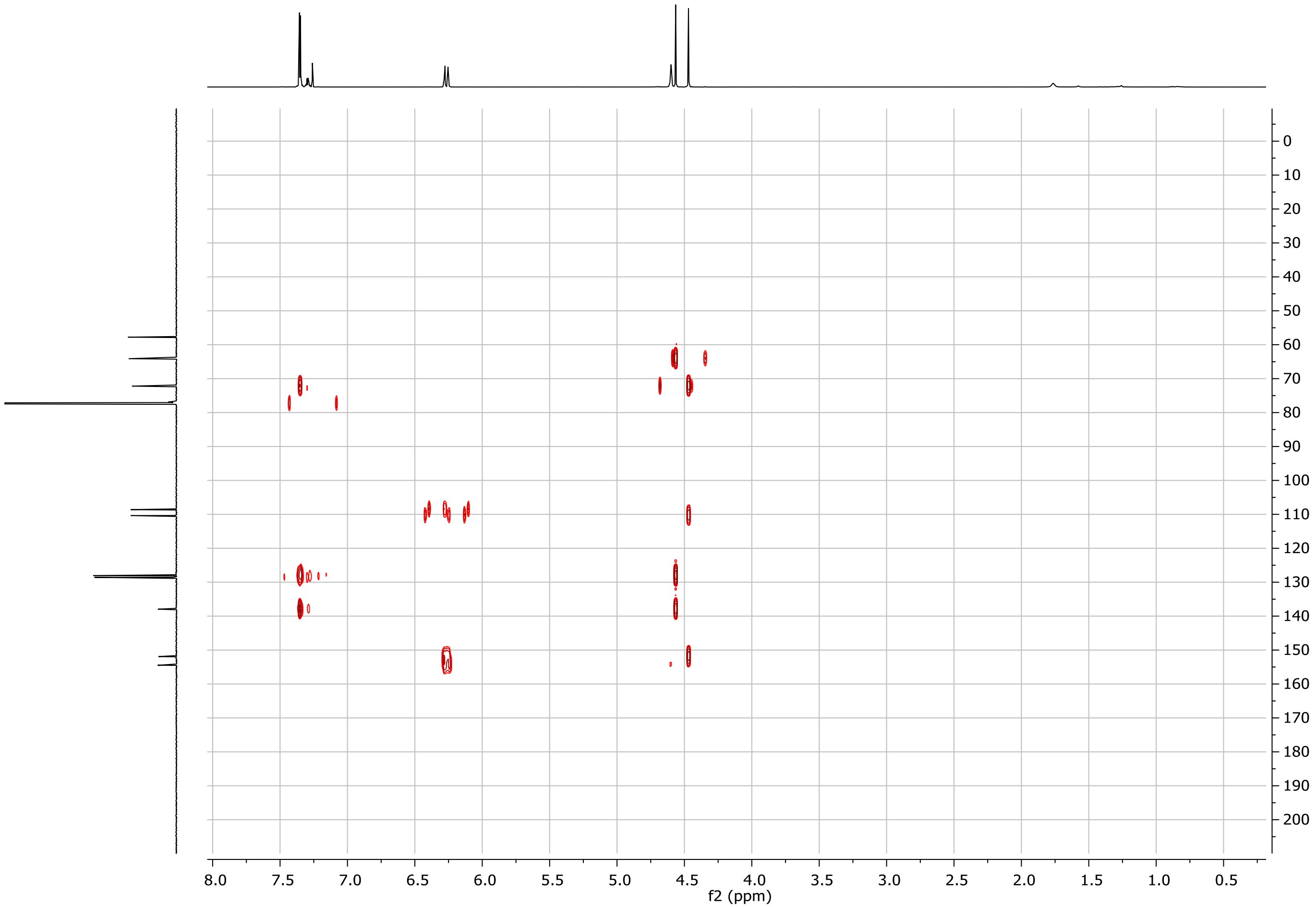

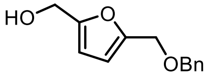

2w

^{1}H, ^{13}C HSQC

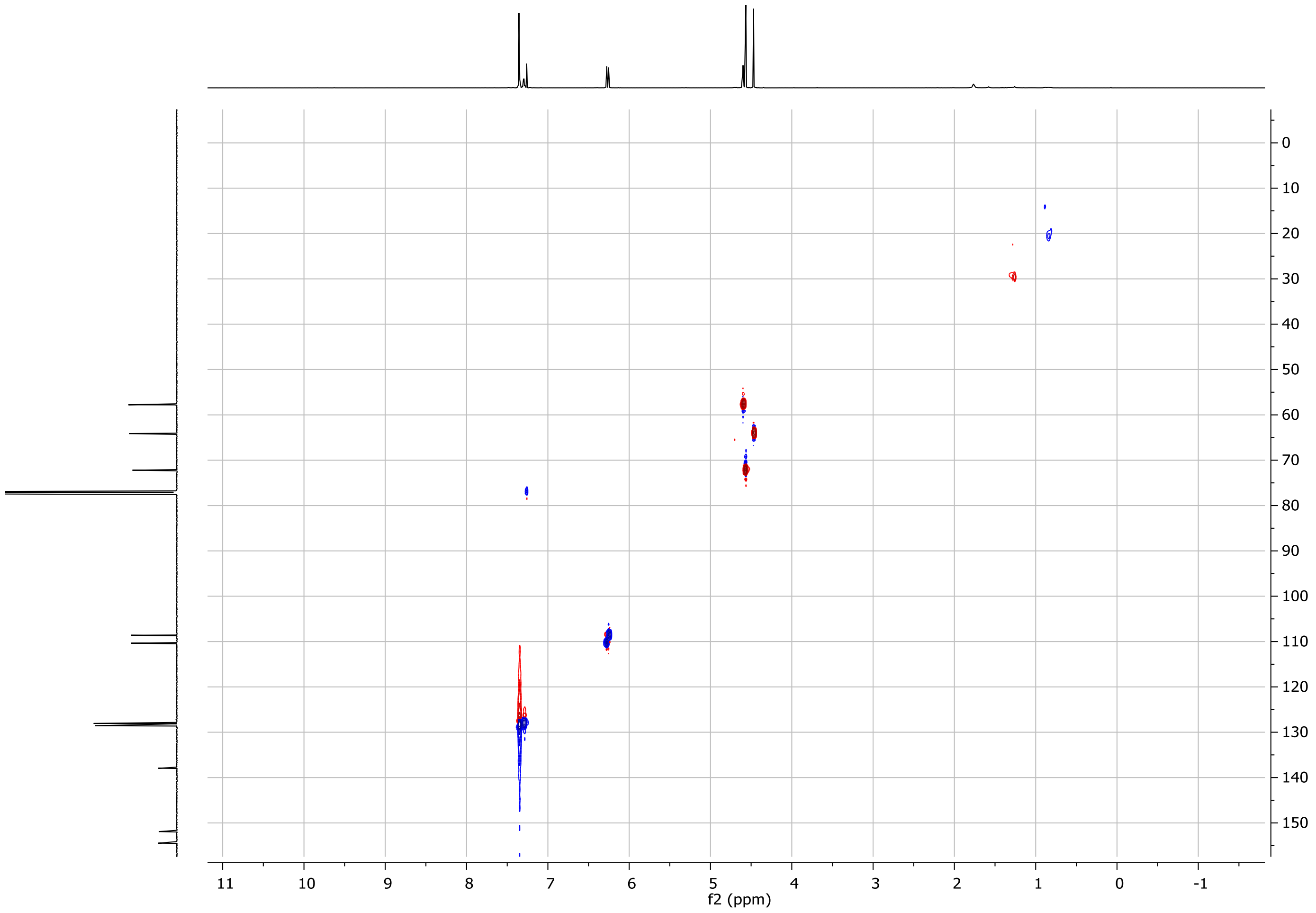

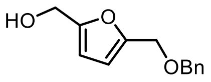

2w

HRMS

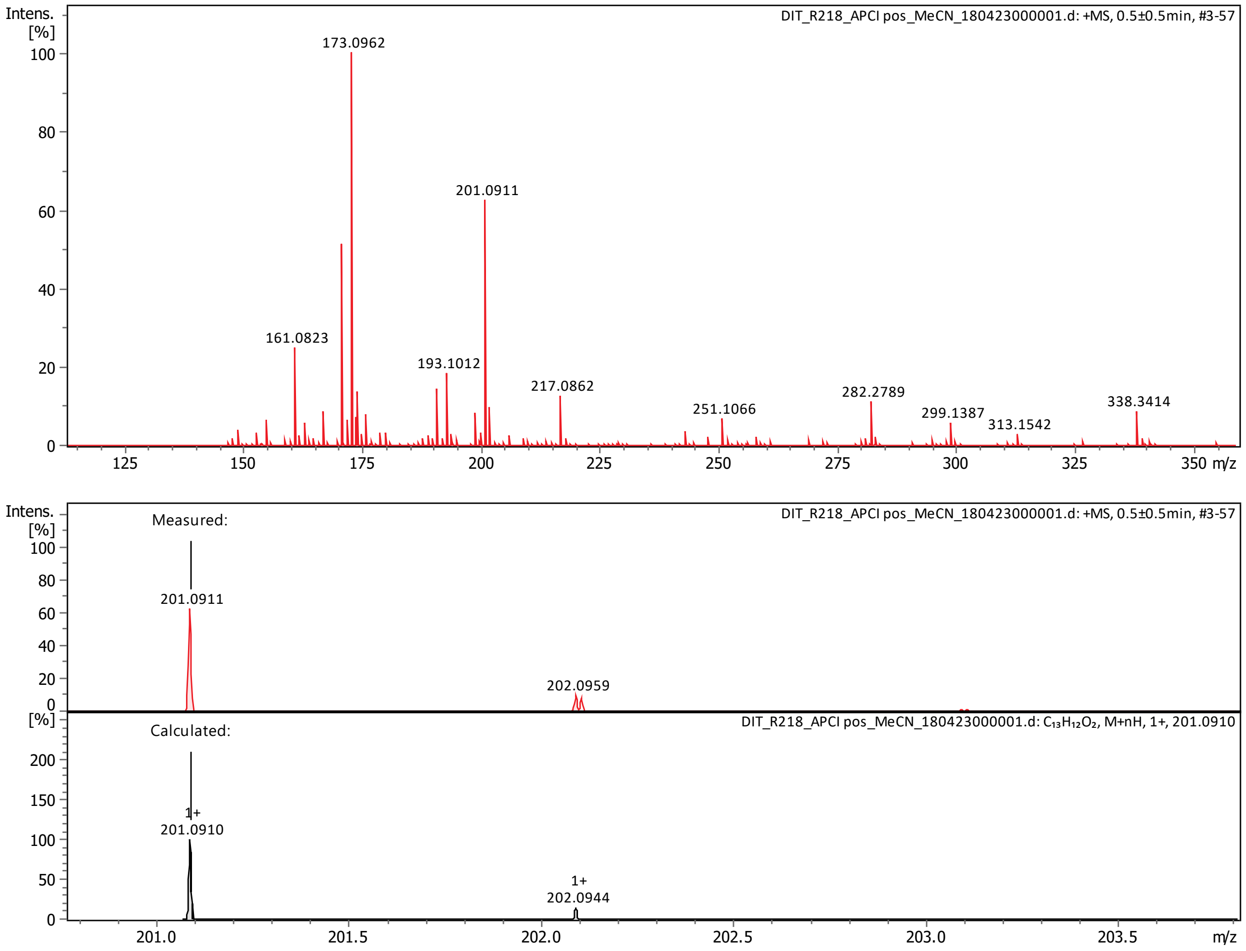

DIT_R218_APCI pos_MeCN_180423000001.d: +MS, 0.5±0.5min, #3-57
173.0962
201.0911
161.0823
193.1012
217.0862
251.1066
282.2789
299.1387
313.1542
338.3414
Measured:
201.0911
202.0959
Calculated:
DIT_R218_APCI pos_MeCN_180423000001.d: $C_{13}H_{12}O_2$, M+nH, 1+, 201.0910
1+
201.0910
1+
202.0944

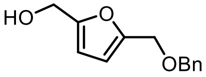

HO
O
OBn

2w

66 4-Phenylbutan-1-ol (2x)

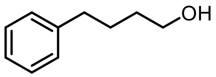

2x

^{1}H NMR

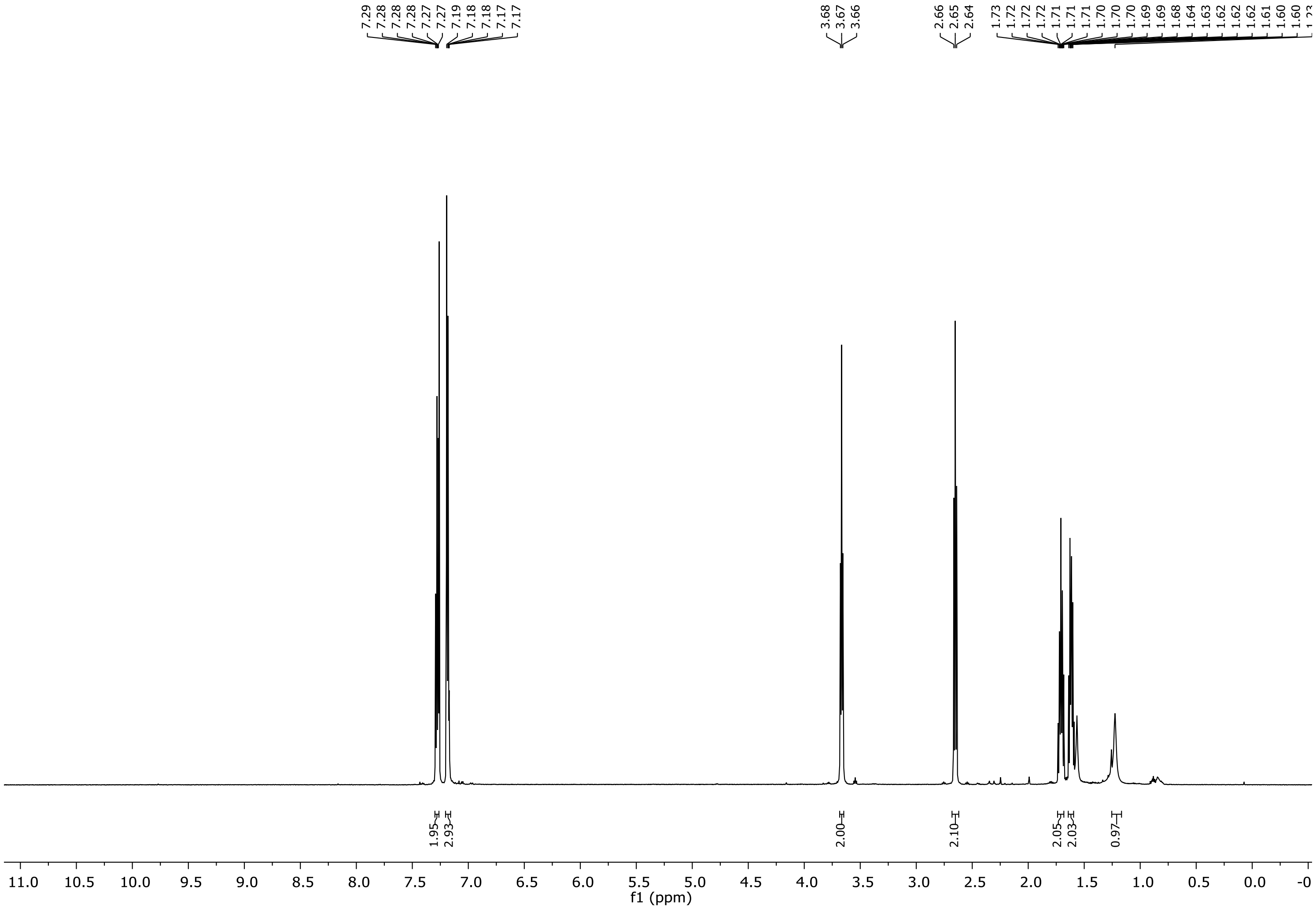

^{13}C NMR

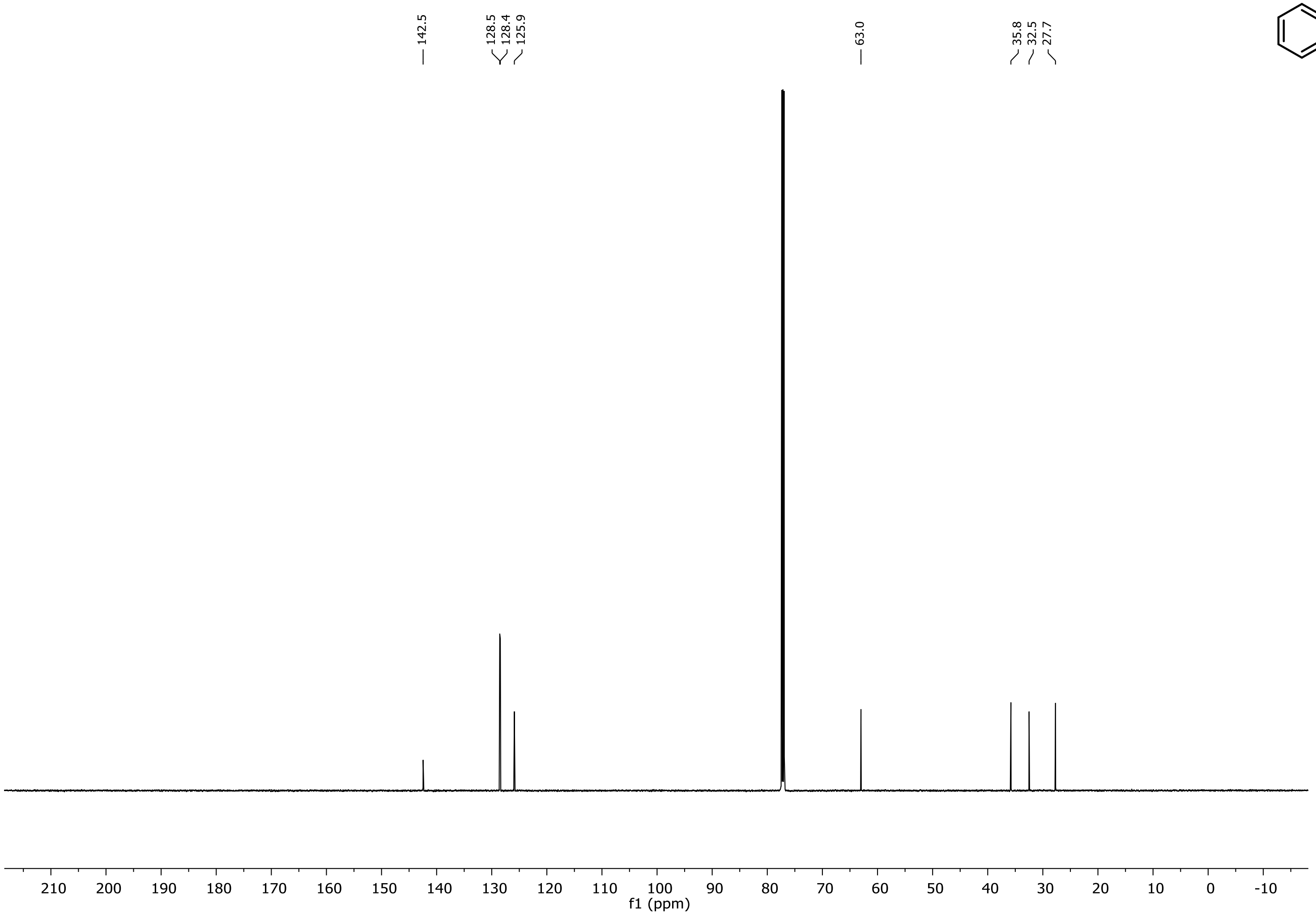
142.5
128.5
128.4
125.9
63.0
35.8
32.5
27.7
210 200 190 180 170 160 150 140 130 120 110 100 90 80 70 60 50 40 30 20 10 0 -10
f1 (ppm)

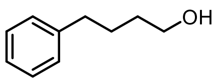
OH

2x

^{1}H, ^{1}H COSY

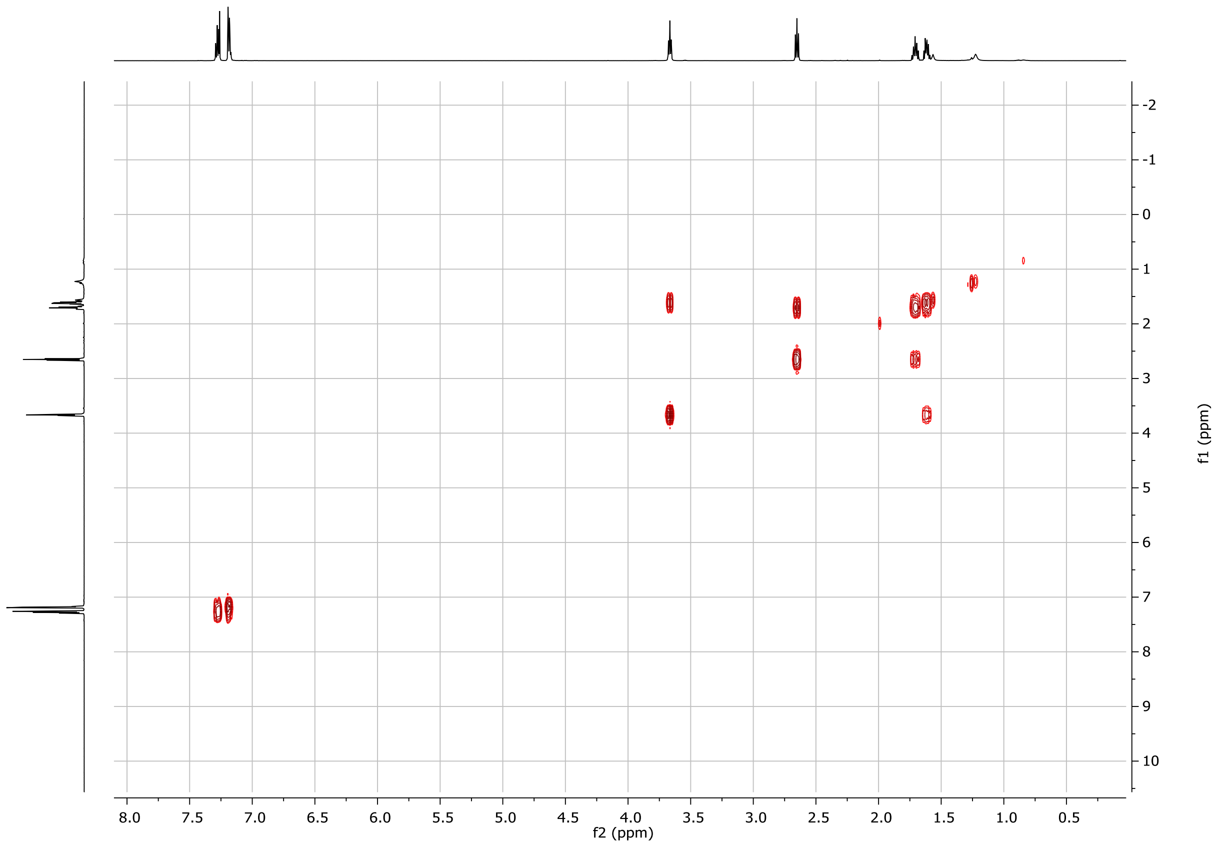

8.0
7.5
7.0
6.5
6.0
5.5
5.0
4.5
4.0
3.5
3.0
2.5
2.0
1.5
1.0
0.5
f2 (ppm)
-2
-1
0
1
2
3
4
5
6
7
8
9
10
f1 (ppm)

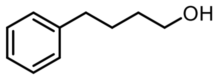

OH

2x

^{1}H, ^{13}C HMBC

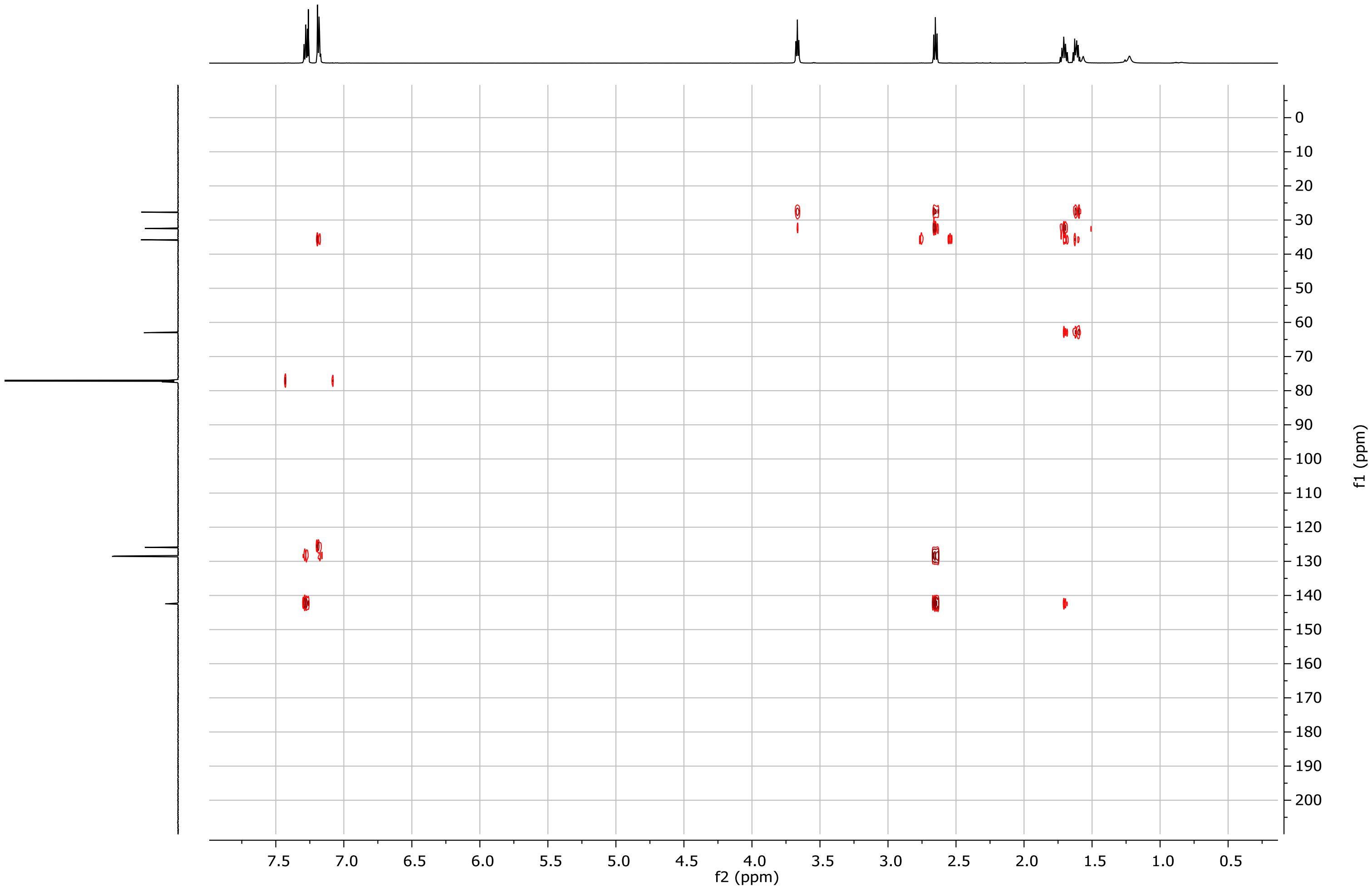

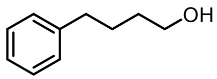

2x

^1H, ^{13}C HSQC

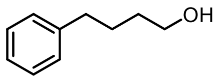

2x

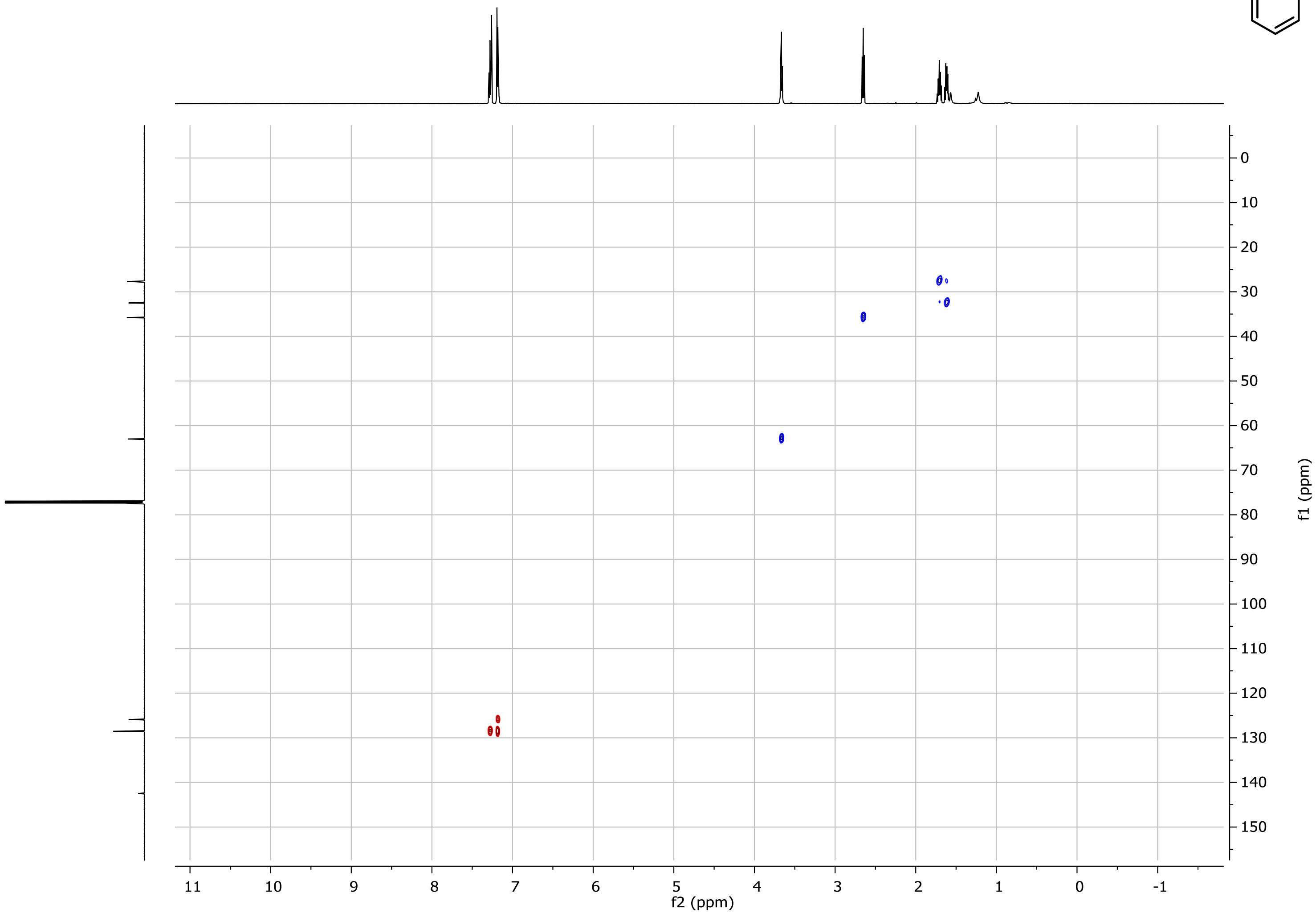

HRMS

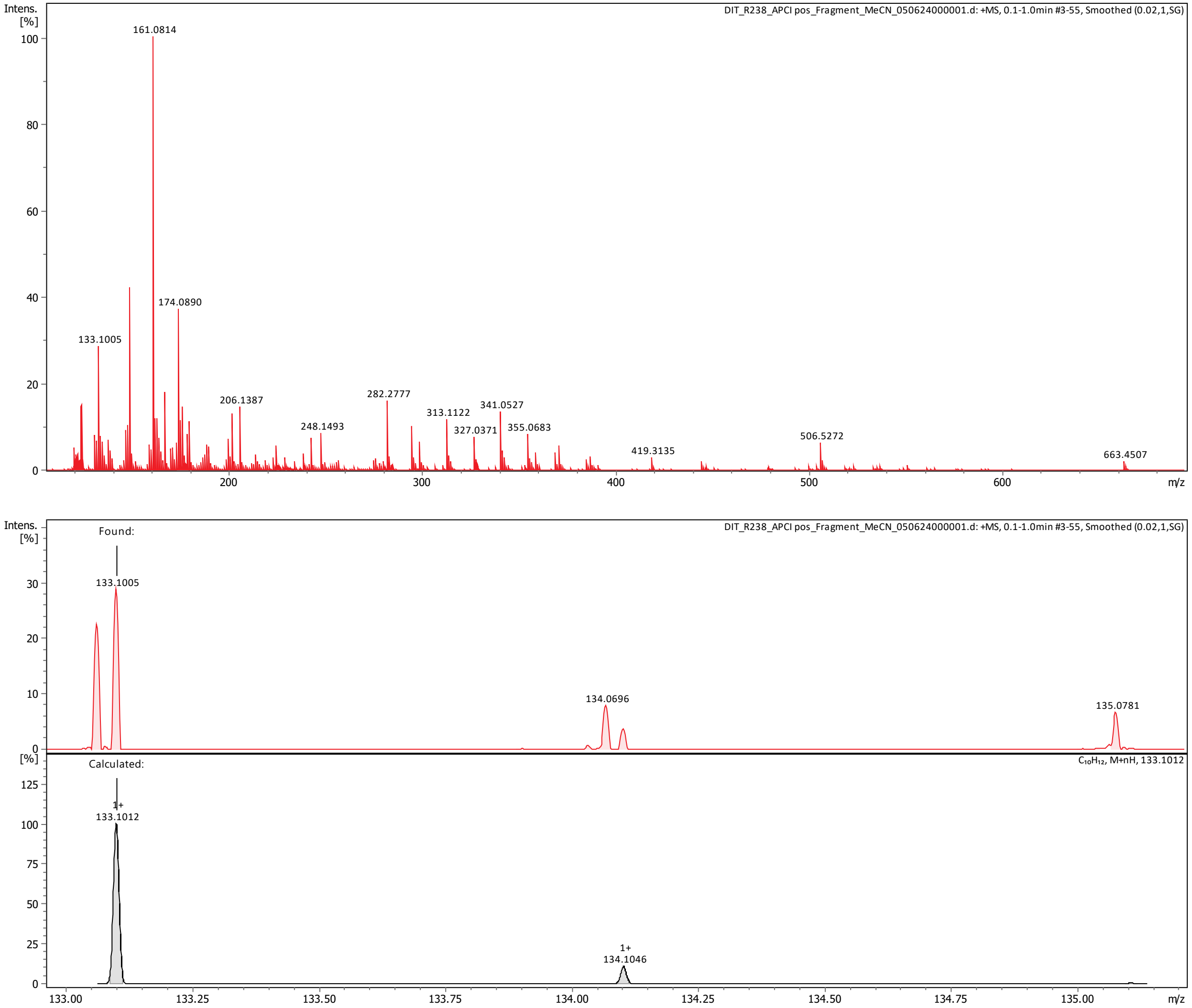

Intens. [%]
DIT_R238_APCI pos_Fragment_MeCN_050624000001.d: +MS, 0.1-1.0min #3-55, Smoothed (0.02,1,SG)
161.0814
174.0890
133.1005
206.1387
248.1493
282.2777
313.1122
327.0371
341.0527
355.0683
419.3135
506.5272
663.4507
m/z
Found:
133.1005
134.0696
135.0781
Calculated:
C₁₀H₁₂, M+nH, 133.1012
1+
133.1012
1+
134.1046

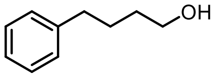

OH

2x

67 Undec-10-en-1-ol (2y)

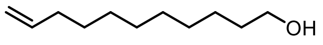

2y

^{1}H NMR

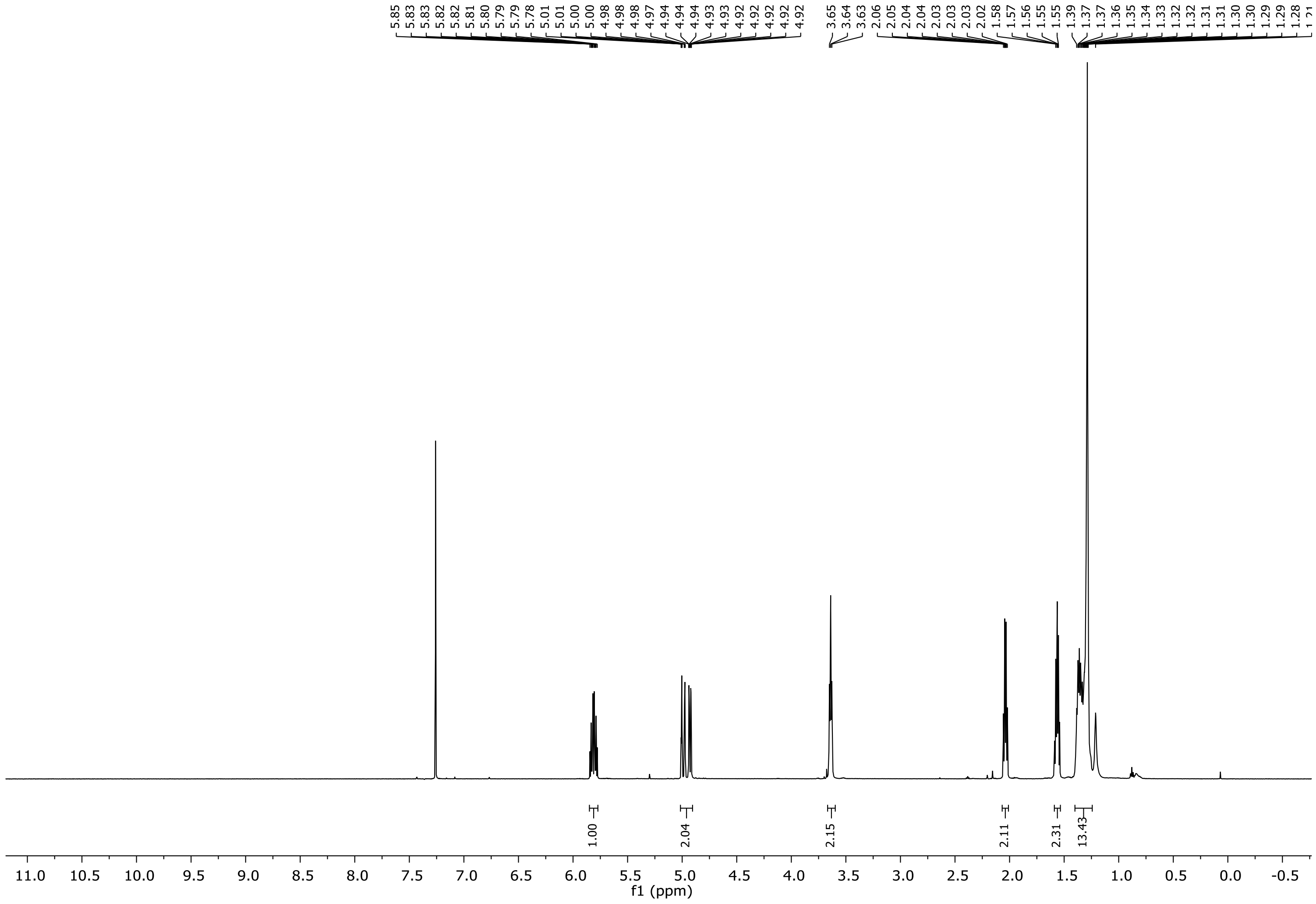

^{13}C NMR

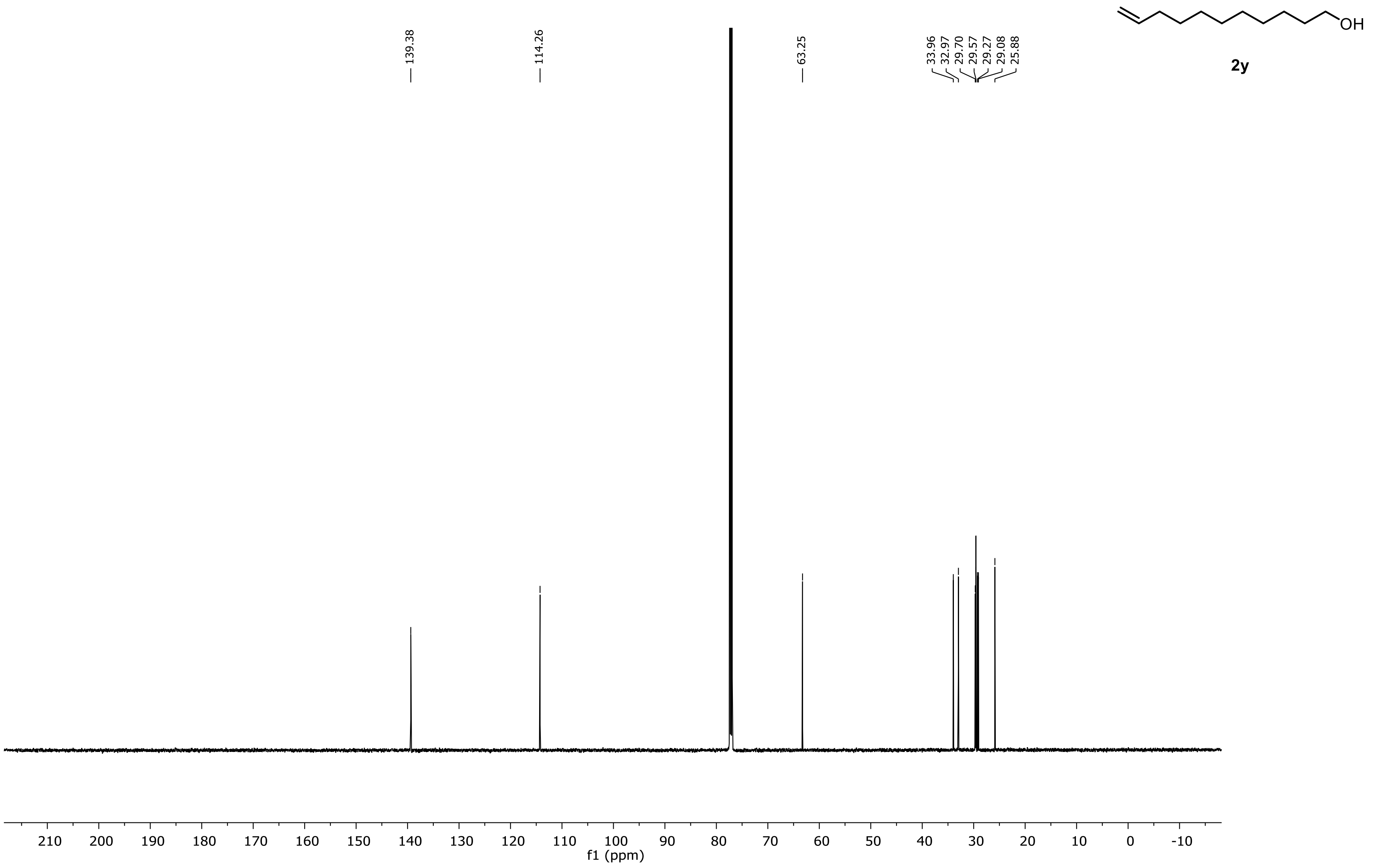

139.38
114.26
63.25
33.96
32.97
29.70
29.57
29.27
29.08
25.88
OH
2y
210 200 190 180 170 160 150 140 130 120 110 100 90 80 70 60 50 40 30 20 10 0 -10
f1 (ppm)

^1H, ^1H COSY

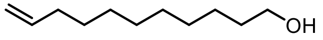

2y

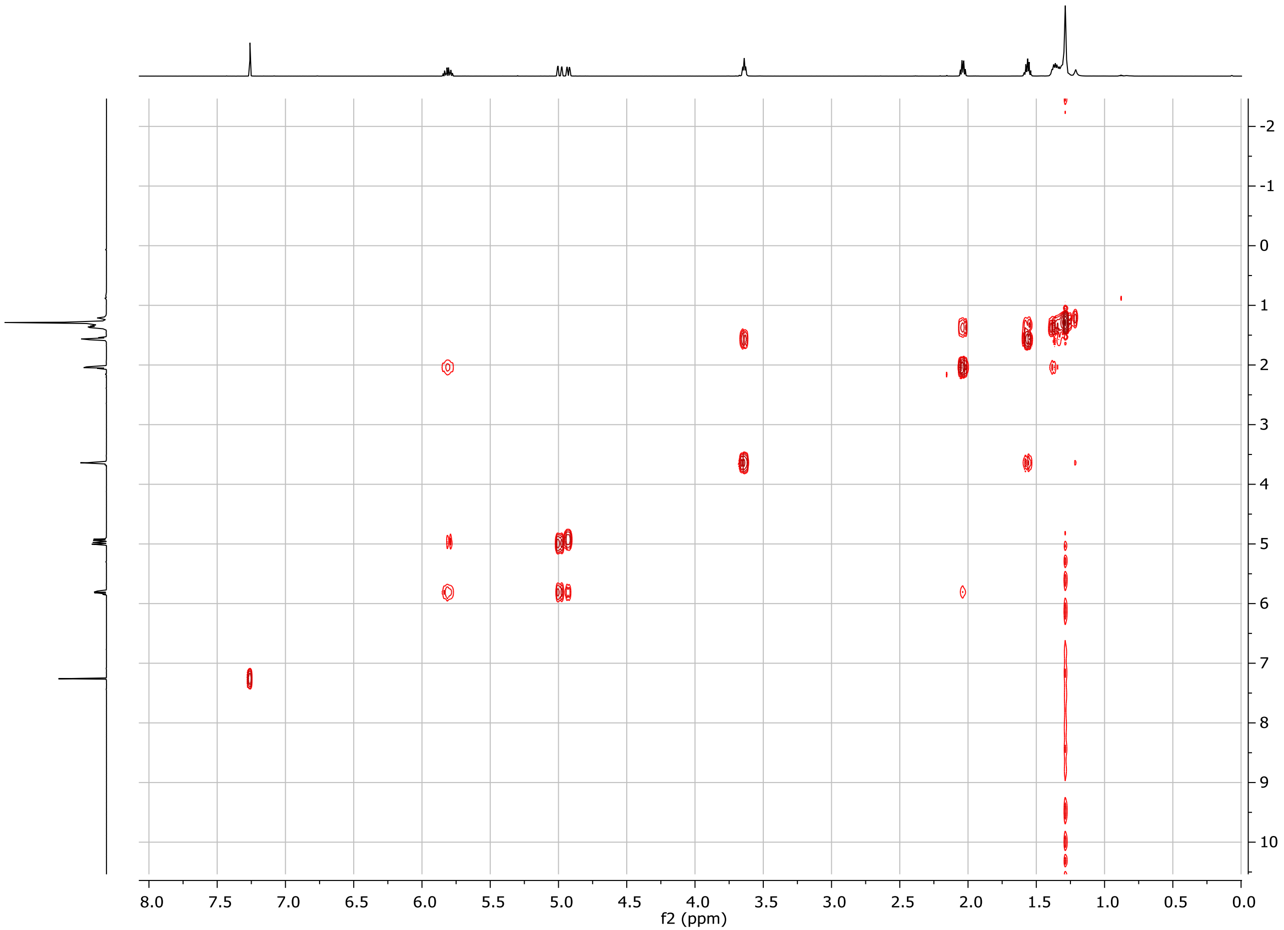

^{1}H, ^{13}C HMBC

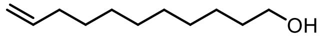

2y

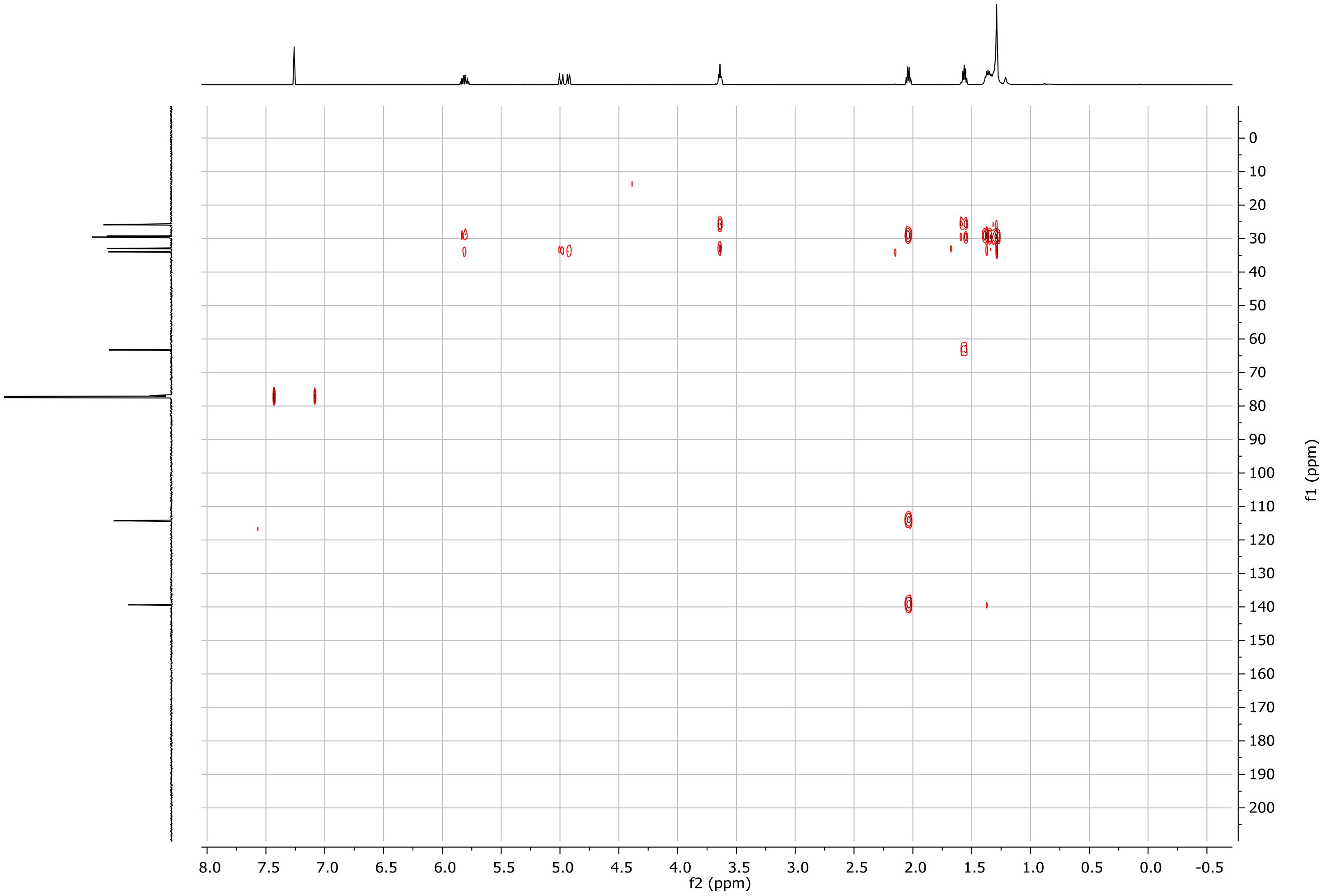

^{1}H, ^{13}C HSQC

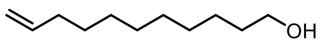

2y

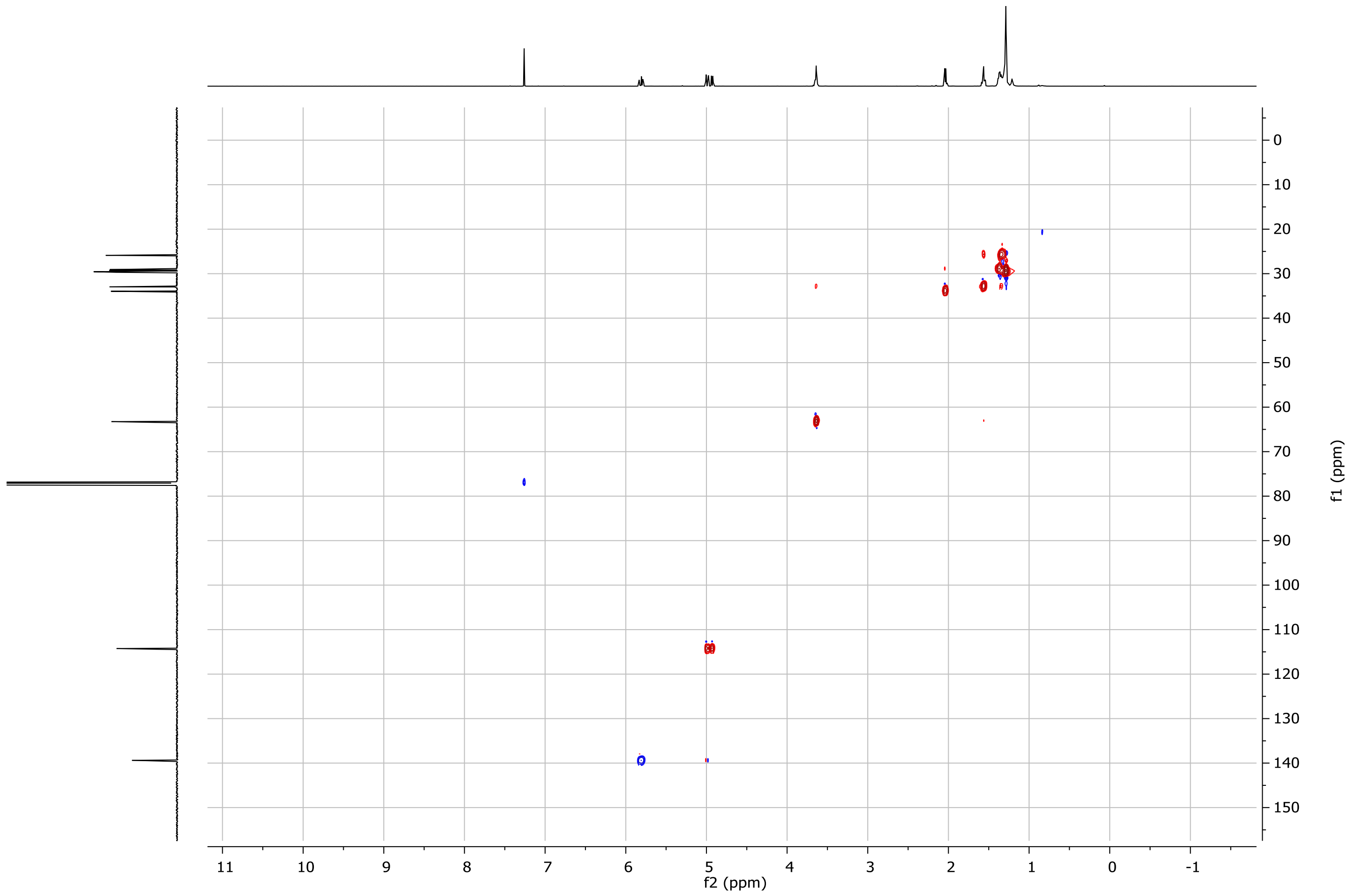

HRMS

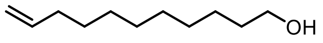

2y

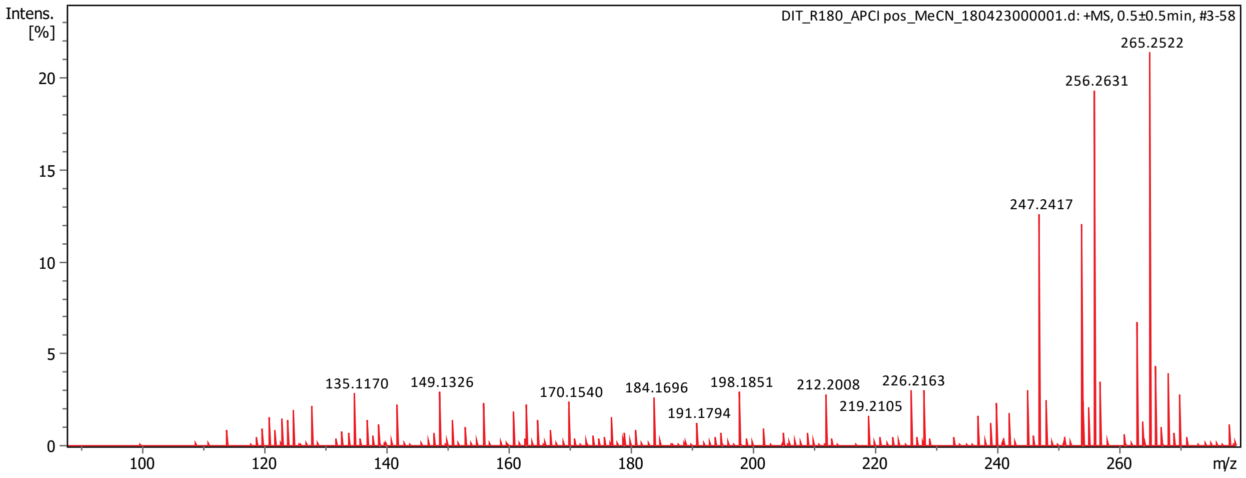

Intens. [%]
DIT_R180_APCI pos_MeCN_180423000001.d: +MS, 0.5±0.5min, #3-58
265.2522
256.2631
247.2417
135.1170
149.1326
170.1540
184.1696
191.1794
198.1851
212.2008
219.2105
226.2163
m/z

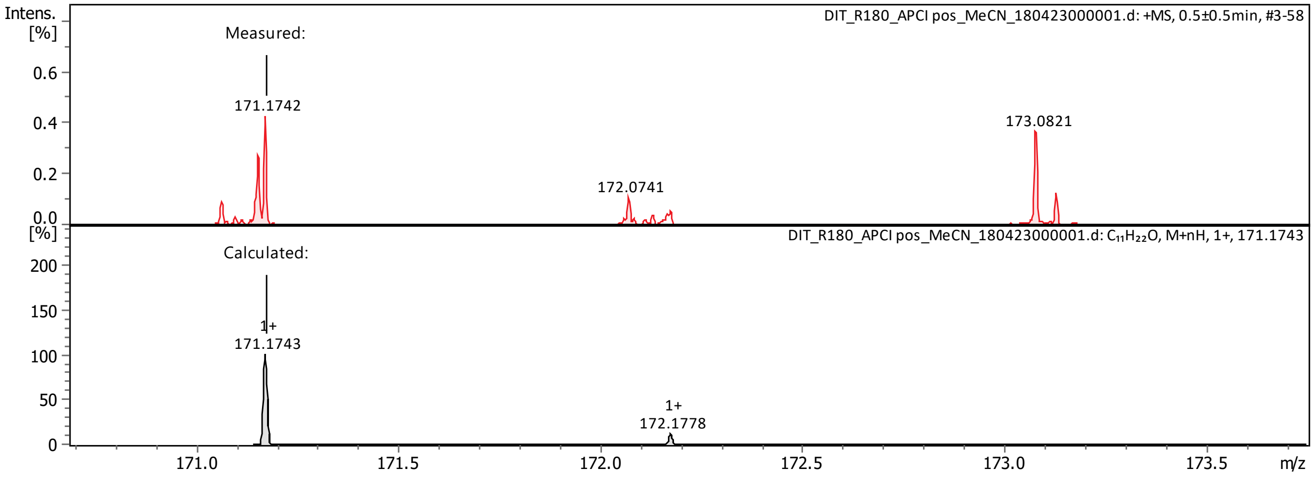

Intens. [%]
DIT_R180_APCI pos_MeCN_180423000001.d: +MS, 0.5±0.5min, #3-58
Measured:
171.1742
172.0741
173.0821
DIT_R180_APCI pos_MeCN_180423000001.d: C₁₁H₂₂O, M+nH, 1+, 171.1743
Calculated:
1+
171.1743
1+
172.1778
m/z

68 Decan-1-ol (2z)

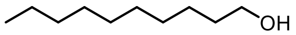

2z

^{1}H NMR

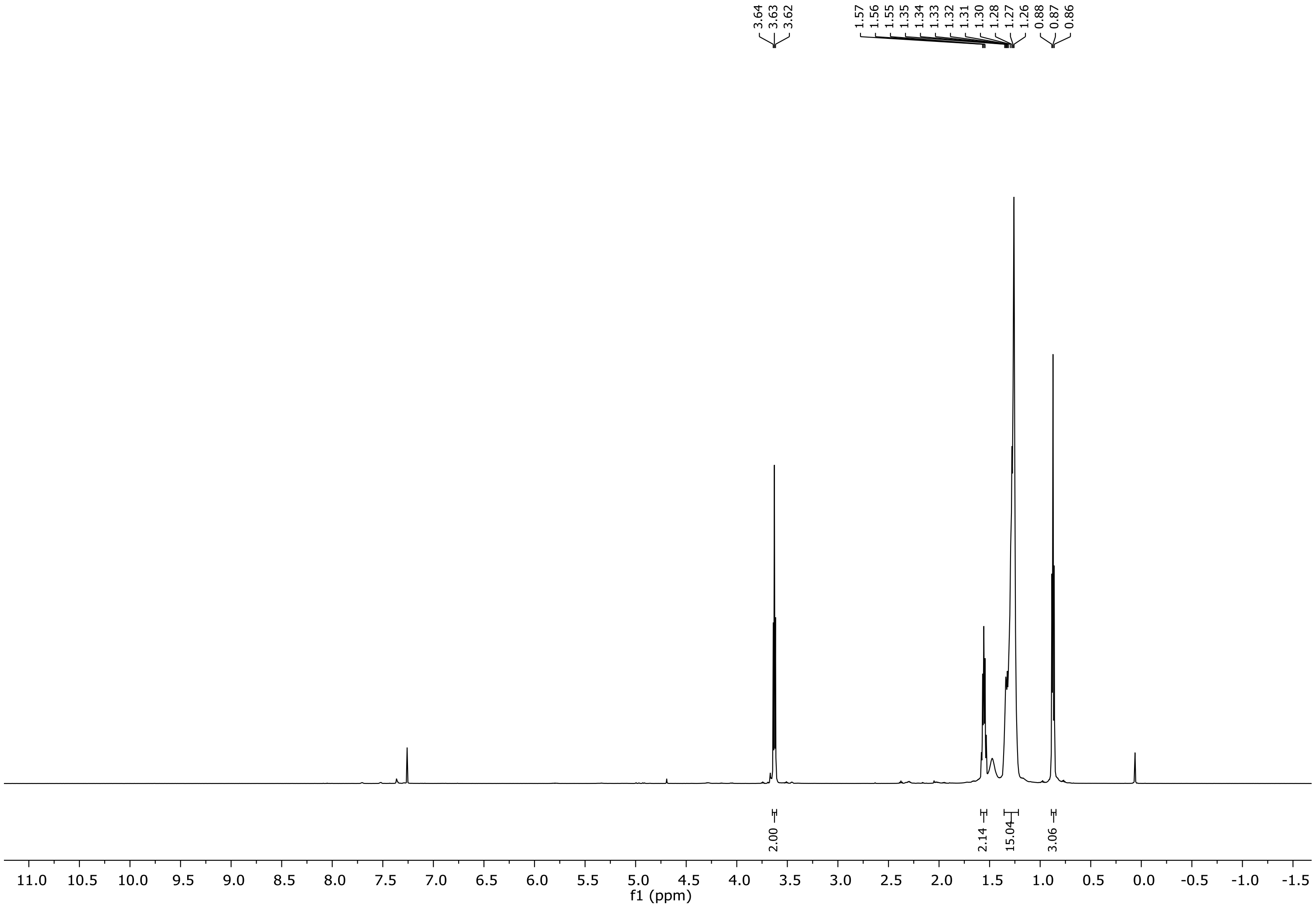

^{13}C NMR

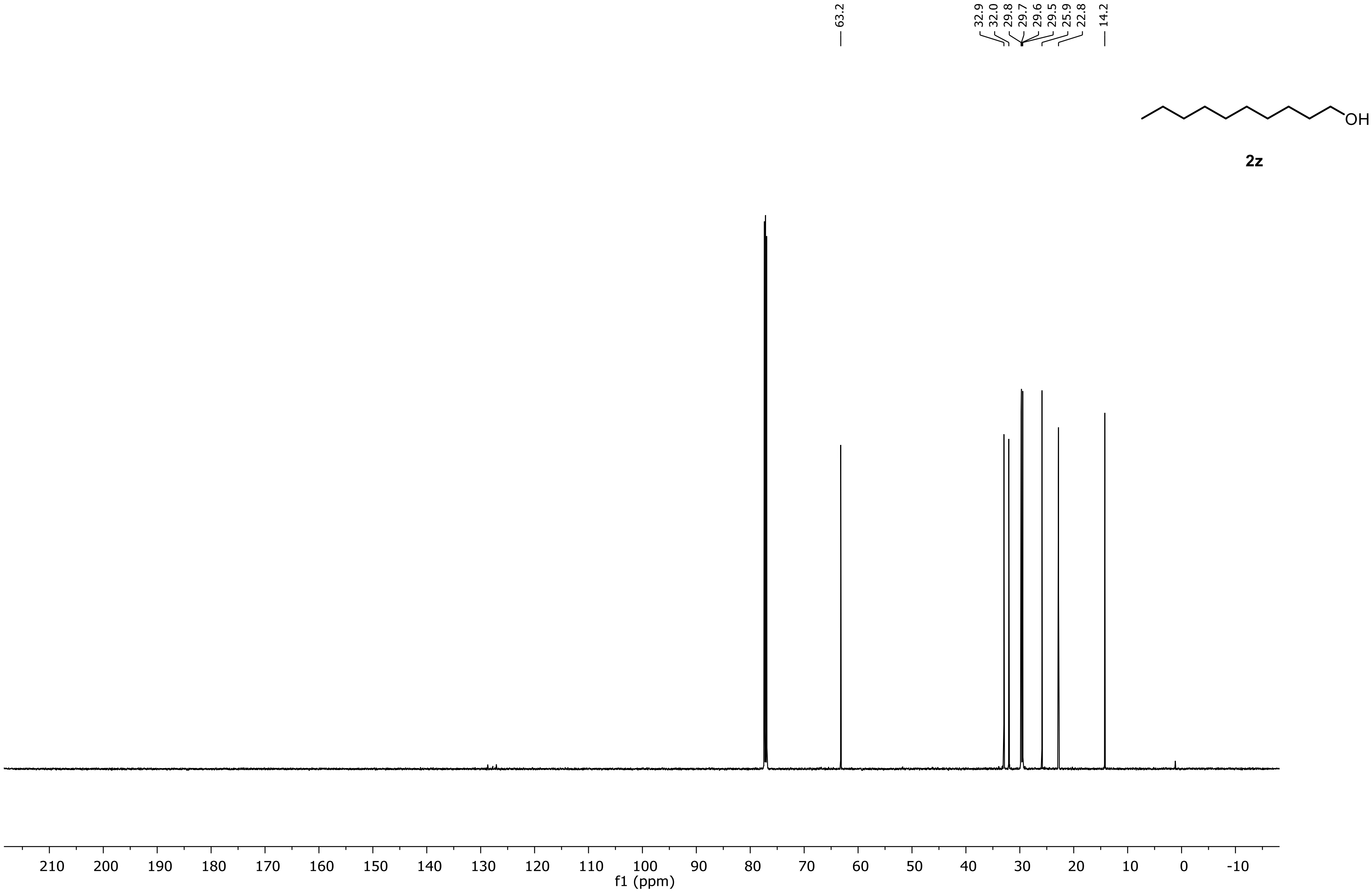
63.2
32.9
32.0
29.8
29.7
29.6
29.5
25.9
22.8
14.2
OH
2z
210
200
190
180
170
160
150
140
130
120
110
100
90
80
70
60
50
40
30
20
10
0
-10
f1 (ppm)

^{1}H, ^{1}H COSY

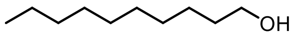

2z

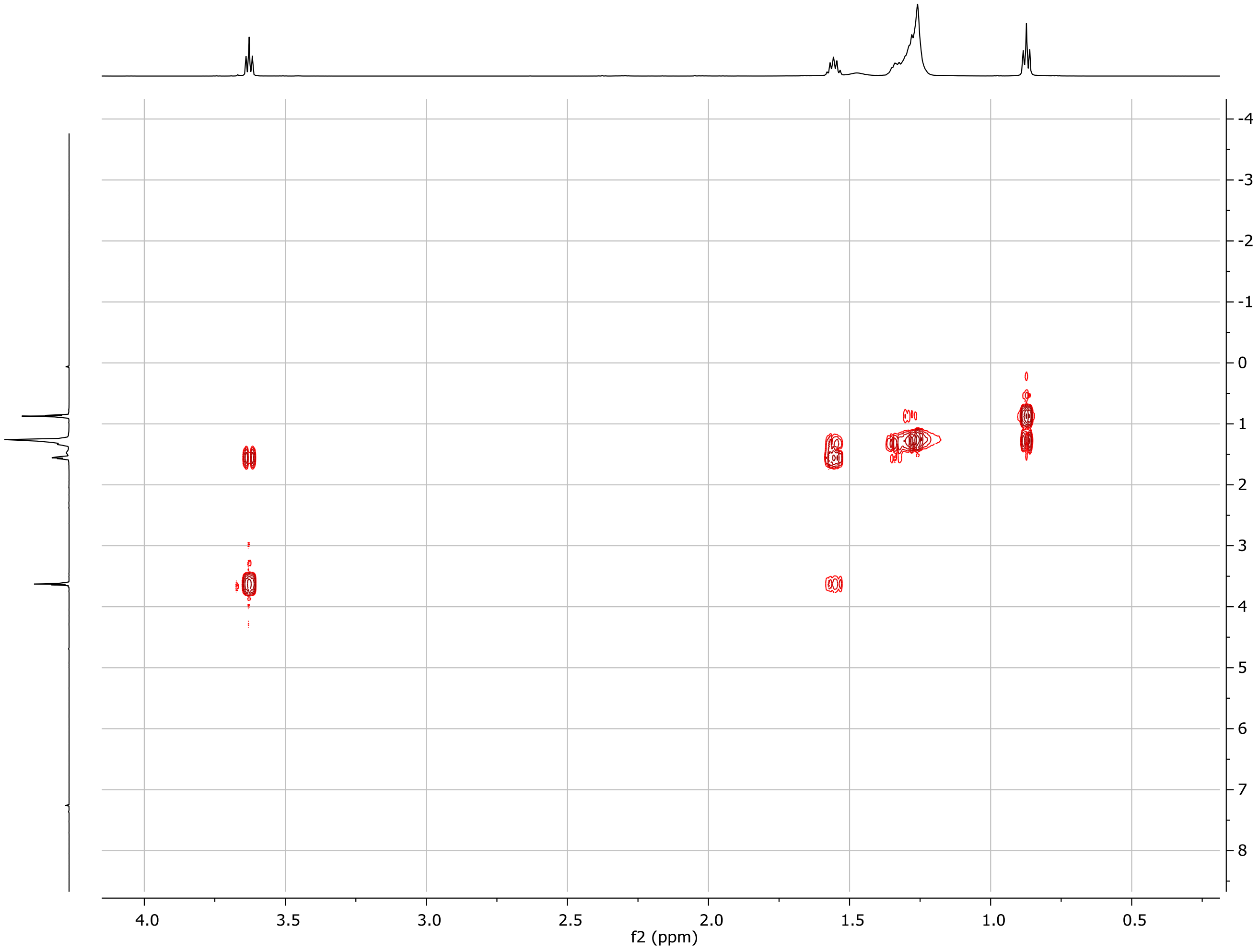

^{1}H, ^{13}C HMBC

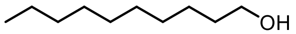

2z

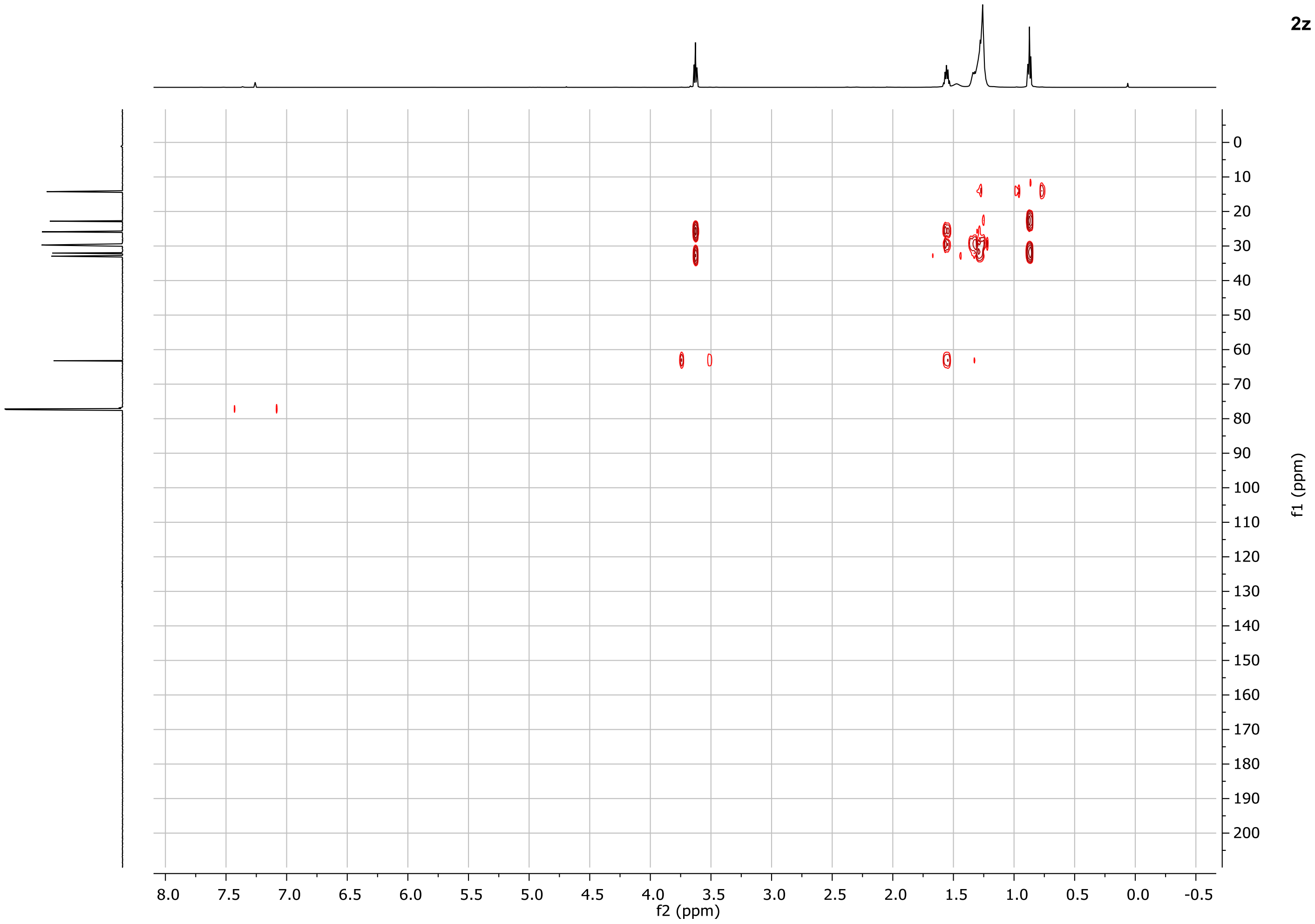

^{1}H, ^{13}C HSQC

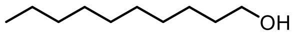

2z

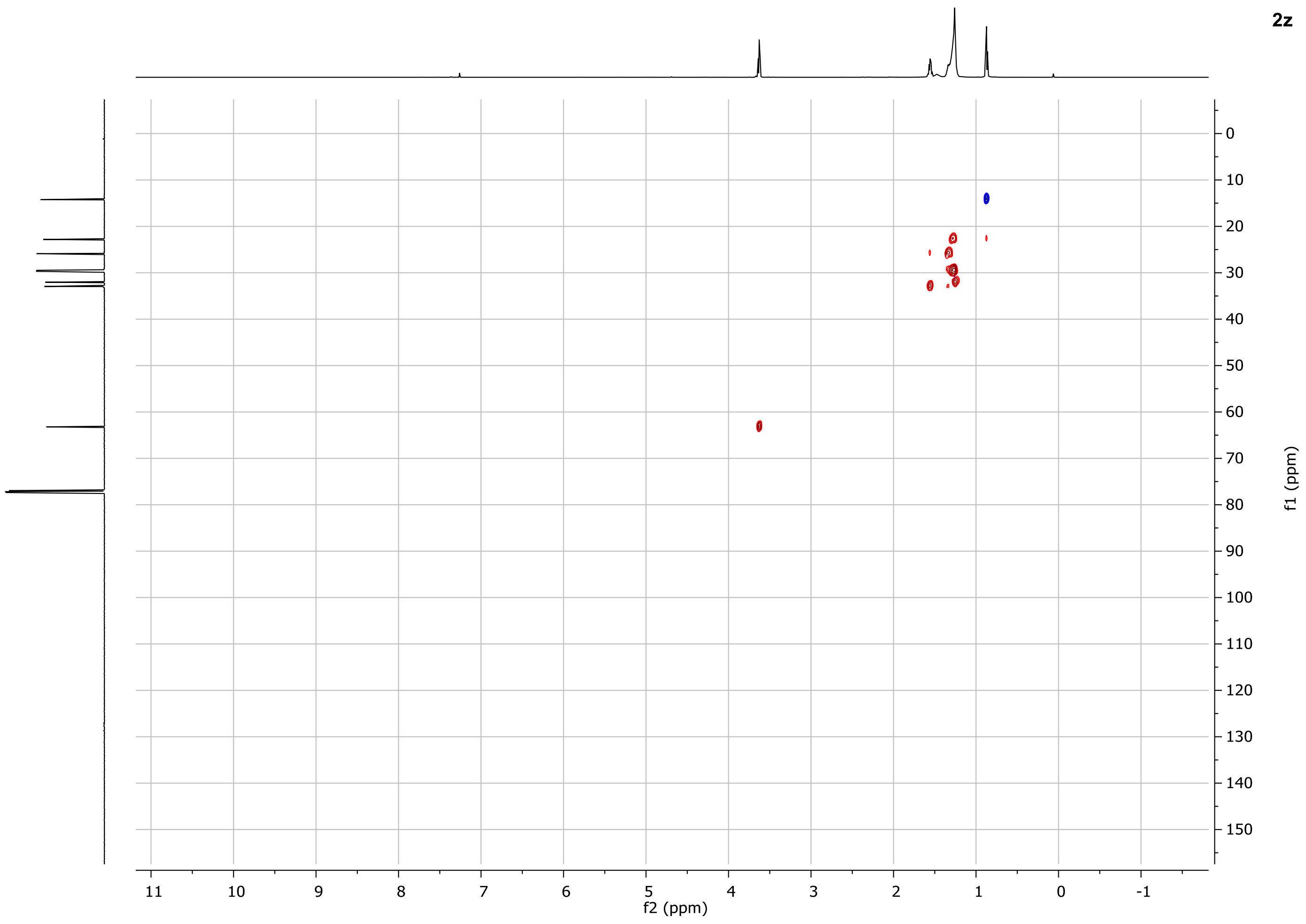

HRMS

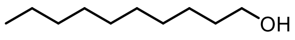

OH

2z

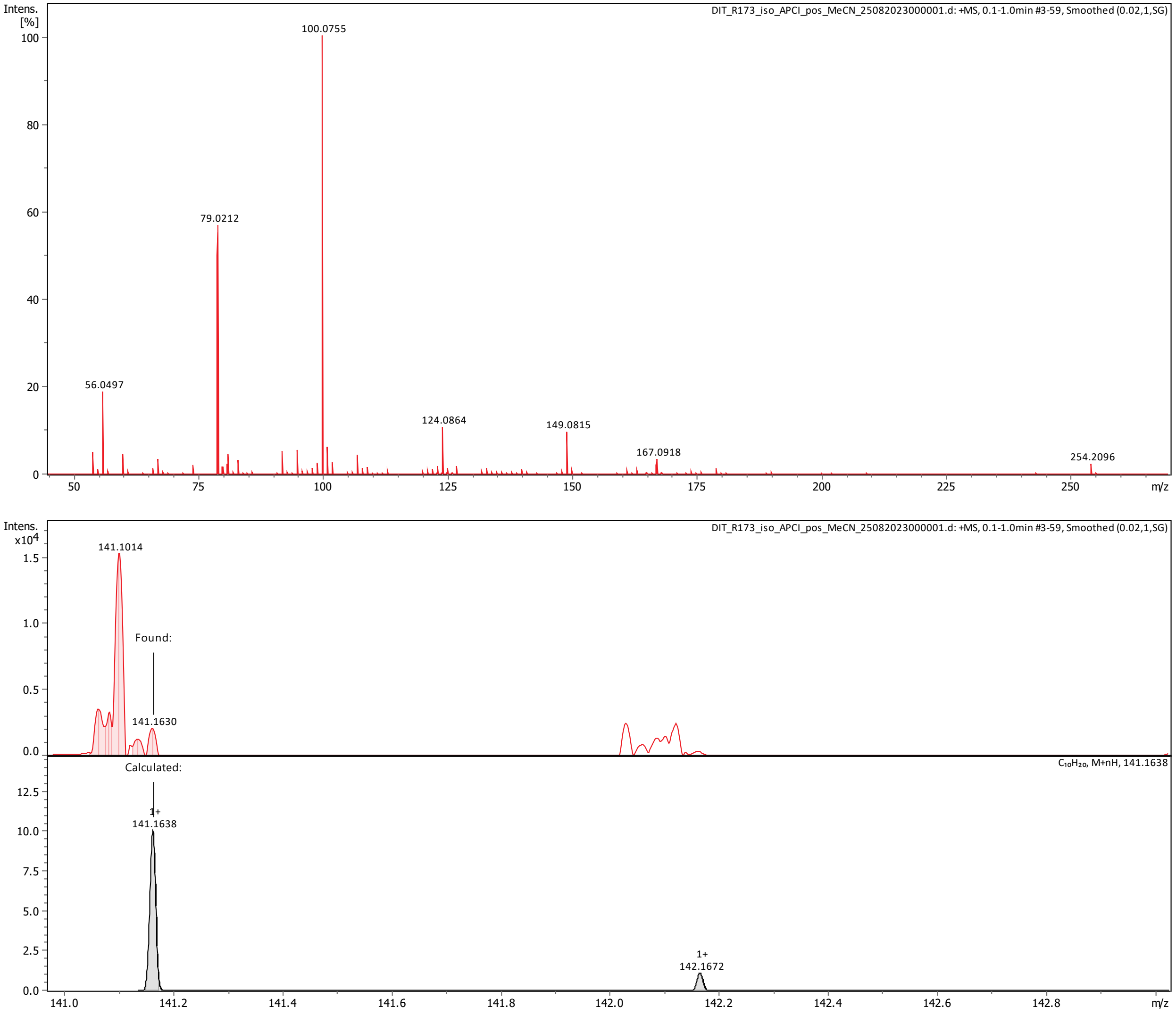

Intens. [%]
DIT_R173_iso_APCI_pos_MeCN_25082023000001.d: +MS, 0.1-1.0min #3-59, Smoothed (0.02,1,SG)
100.0755
79.0212
56.0497
124.0864
149.0815
167.0918
254.2096
m/z
Intens. x10^4
141.1014
Found:
141.1630
Calculated:
C10H20, M+nH, 141.1638
1+
141.1638
1+
142.1672
m/z

69 (4-Styrylphenyl)methanol (2aa)

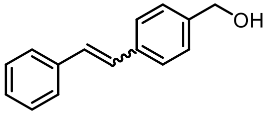

2aa

^{1}H NMR

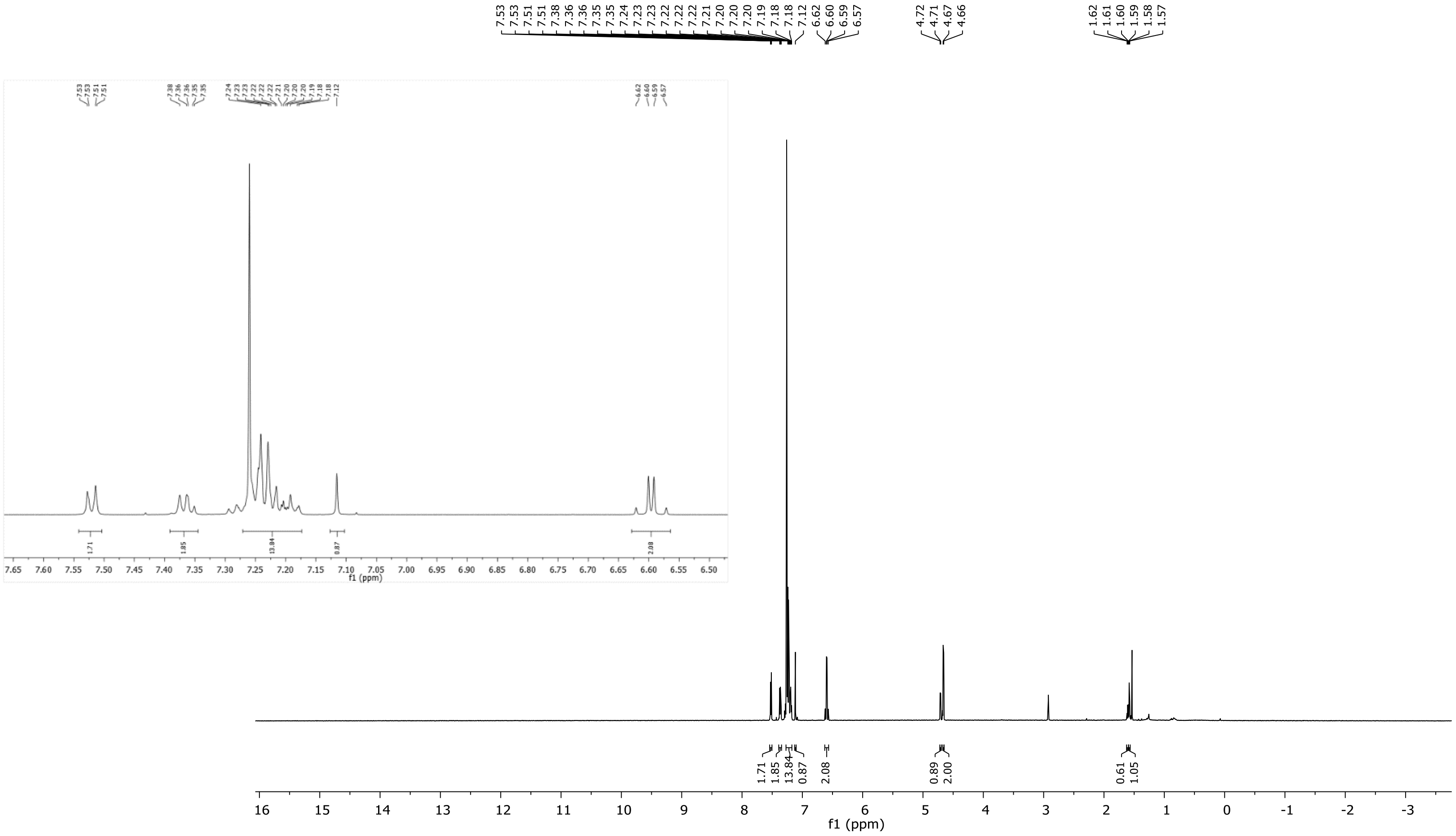

^{13}C NMR

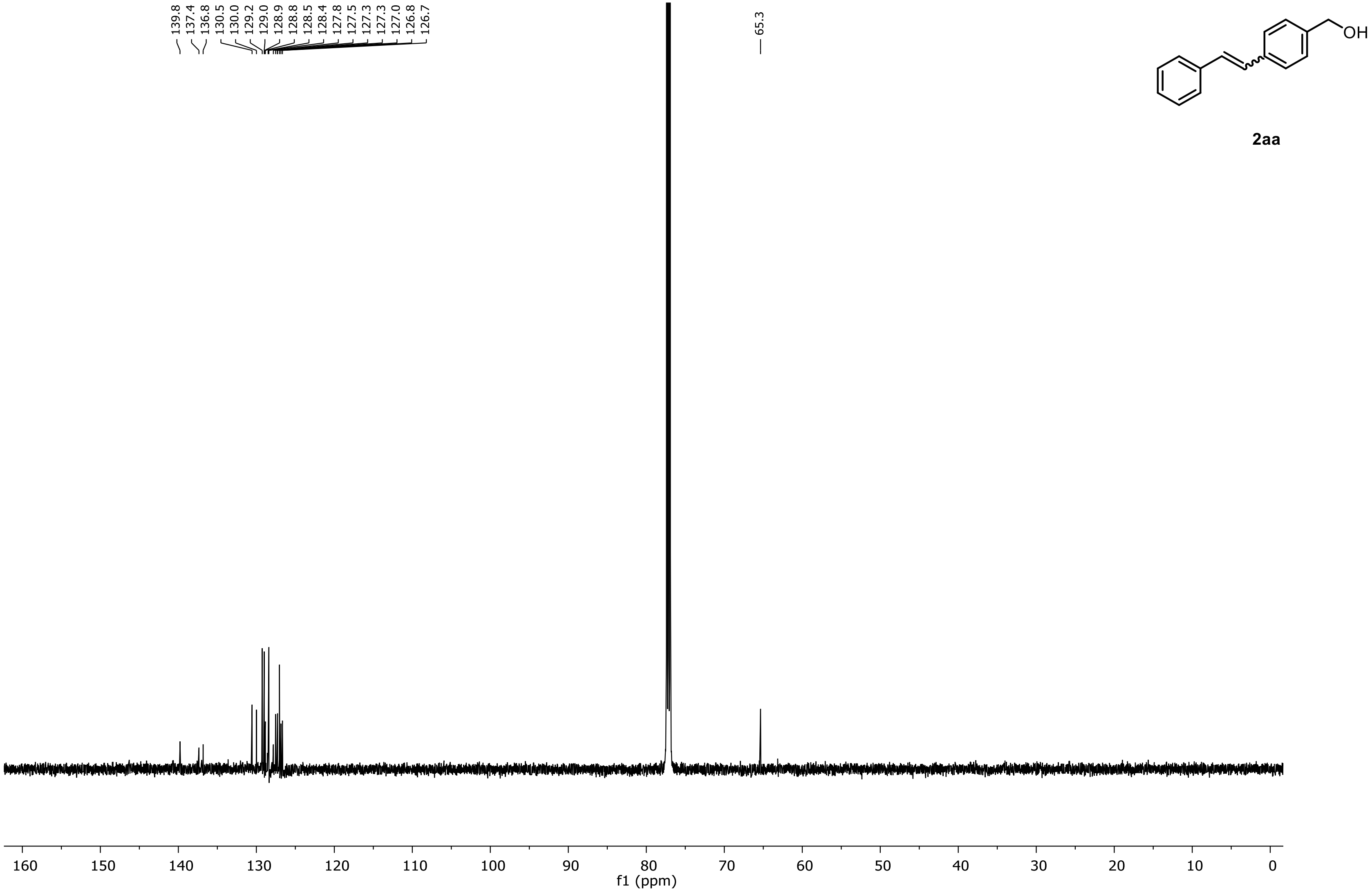

139.8
137.4
136.8
130.5
130.0
129.2
129.0
128.9
128.8
128.8
128.5
128.4
127.8
127.5
127.3
127.3
127.0
126.8
126.7
65.3
160
150
140
130
120
110
100
90
80
70
60
50
40
30
20
10
0
f1 (ppm)
OH

2aa

^{1}H, ^{1}H COSY

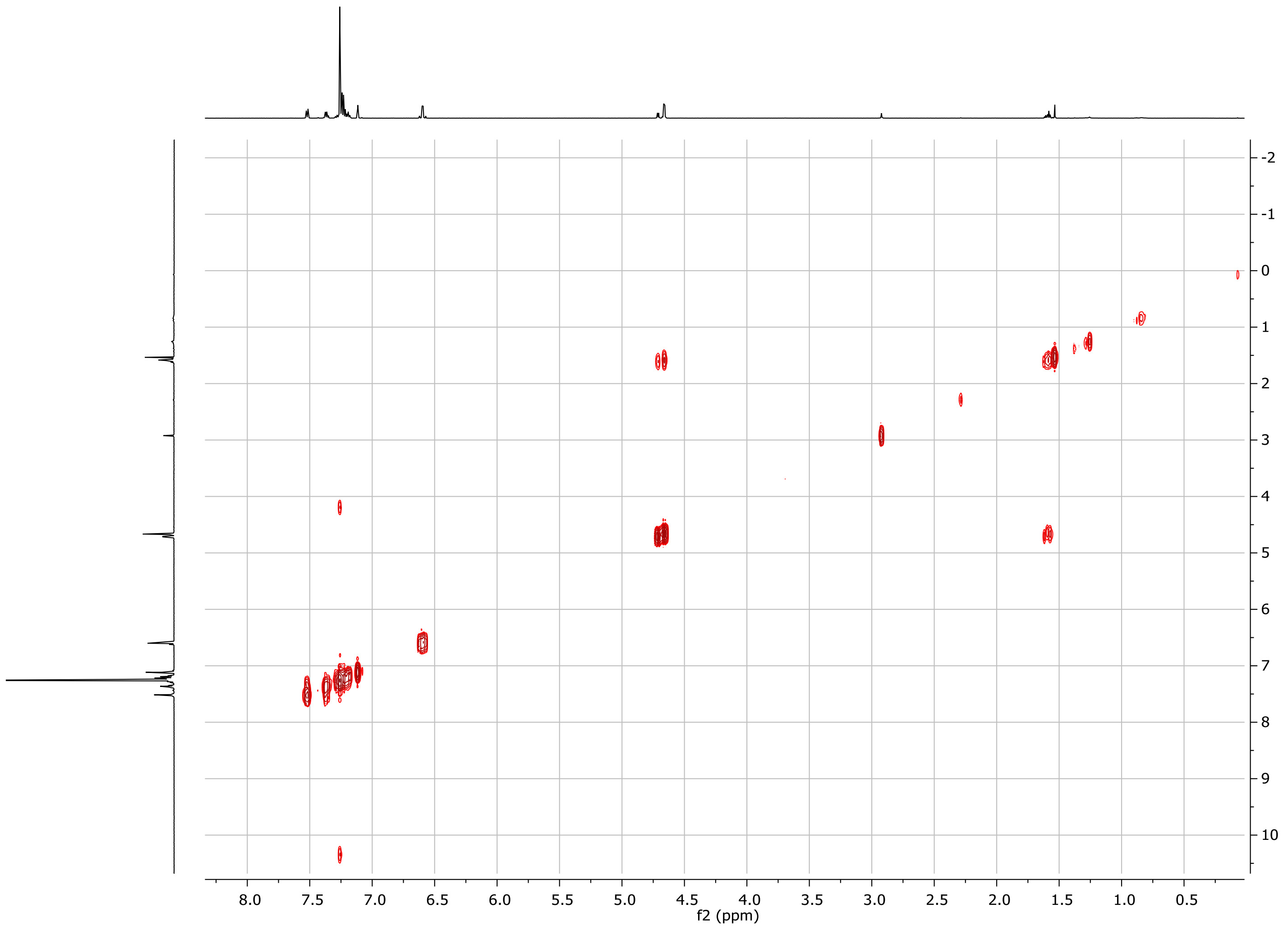

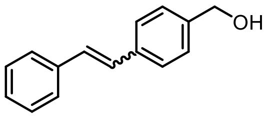

2aa

^{1}H, ^{13}C HMBC

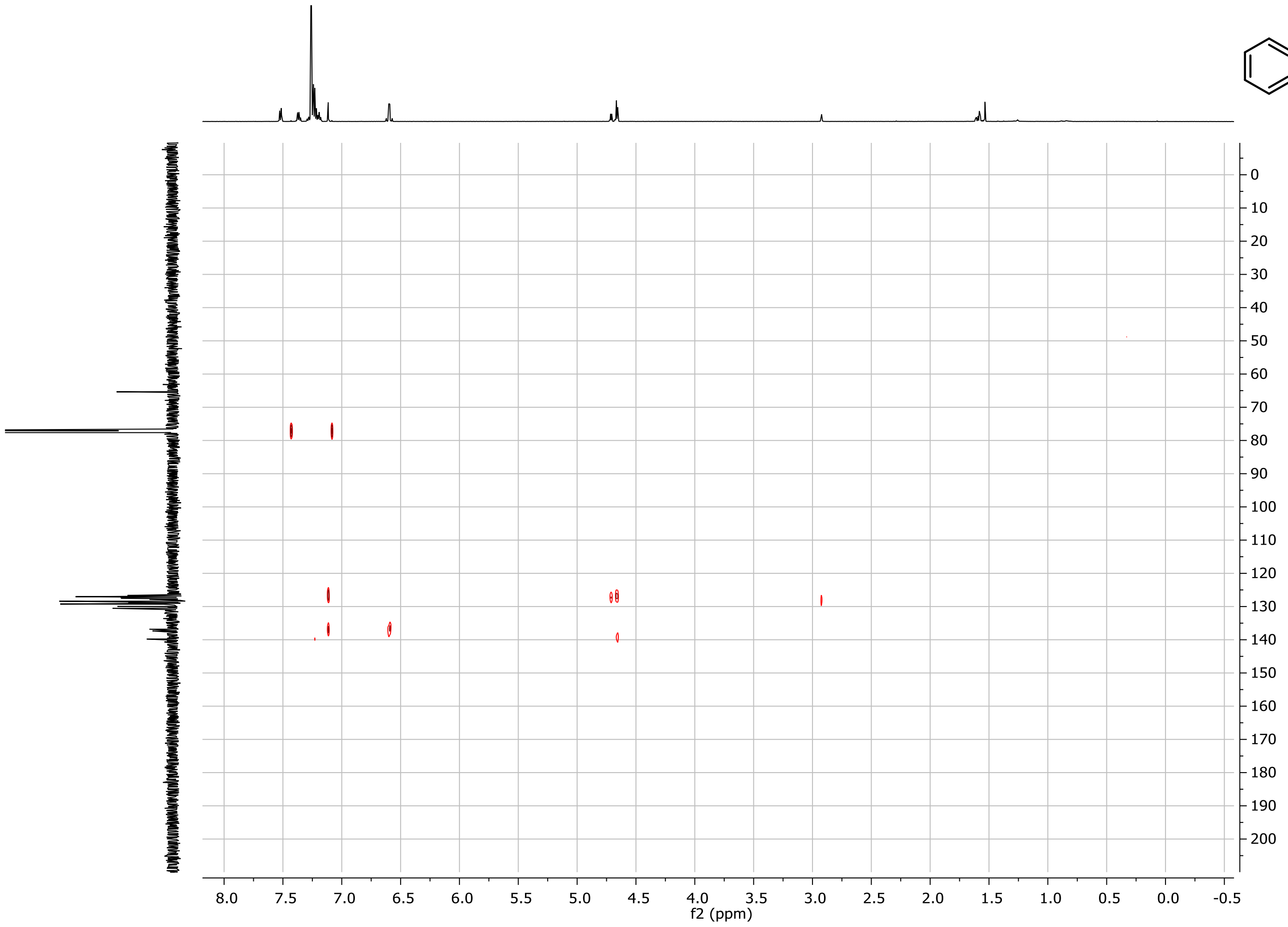

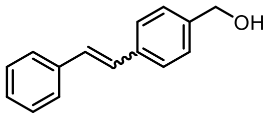

2aa

^{1}H, ^{13}C HSQC

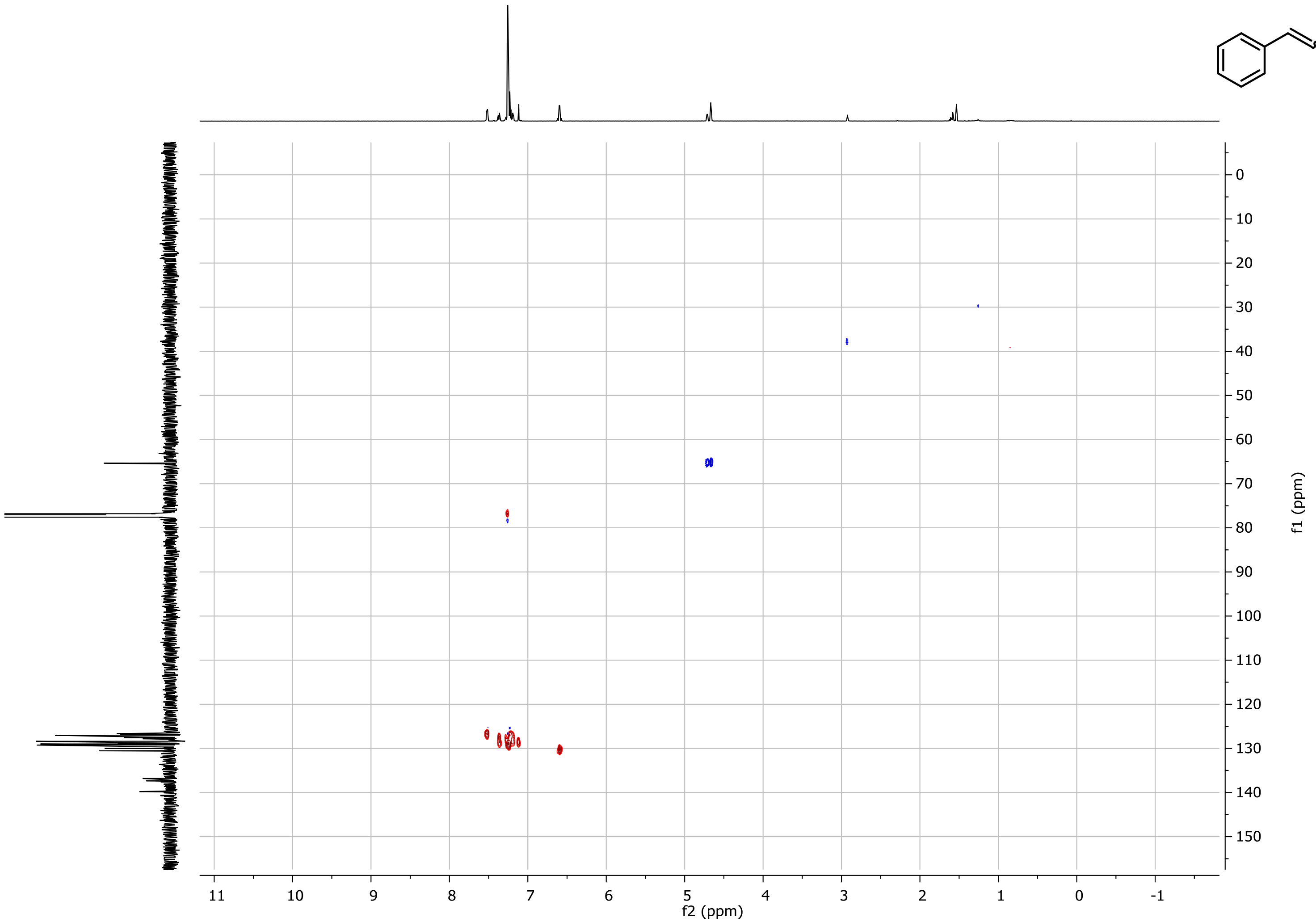

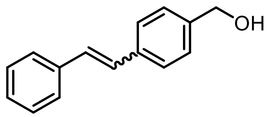

2aa

HRMS

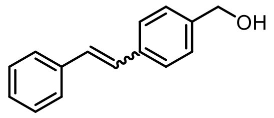
OH

2aa

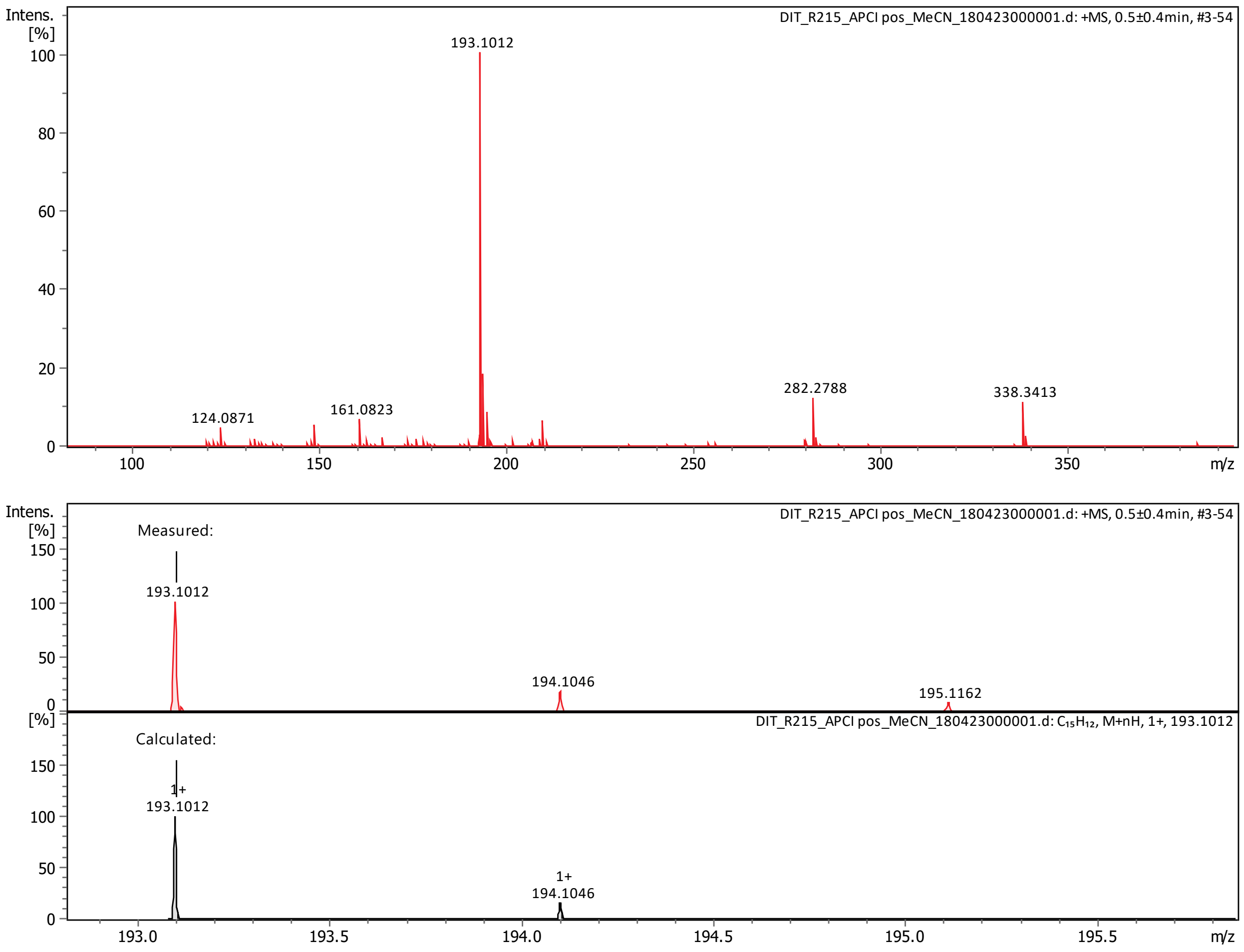
Intens.
[%]
DIT_R215_APCI pos_MeCN_180423000001.d: +MS, 0.5±0.4min, #3-54
193.1012
124.0871
161.0823
282.2788
338.3413
m/z
Measured:
193.1012
194.1046
195.1162
Calculated:
DIT_R215_APCI pos_MeCN_180423000001.d: C15H12, M+nH, 1+, 193.1012
1+
193.1012
1+
194.1046

70 Benzyl alcohol from (4-methylpiperazin-1-yl)(phenyl)methanone (2ab)

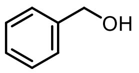

2ab

^{1}H NMR

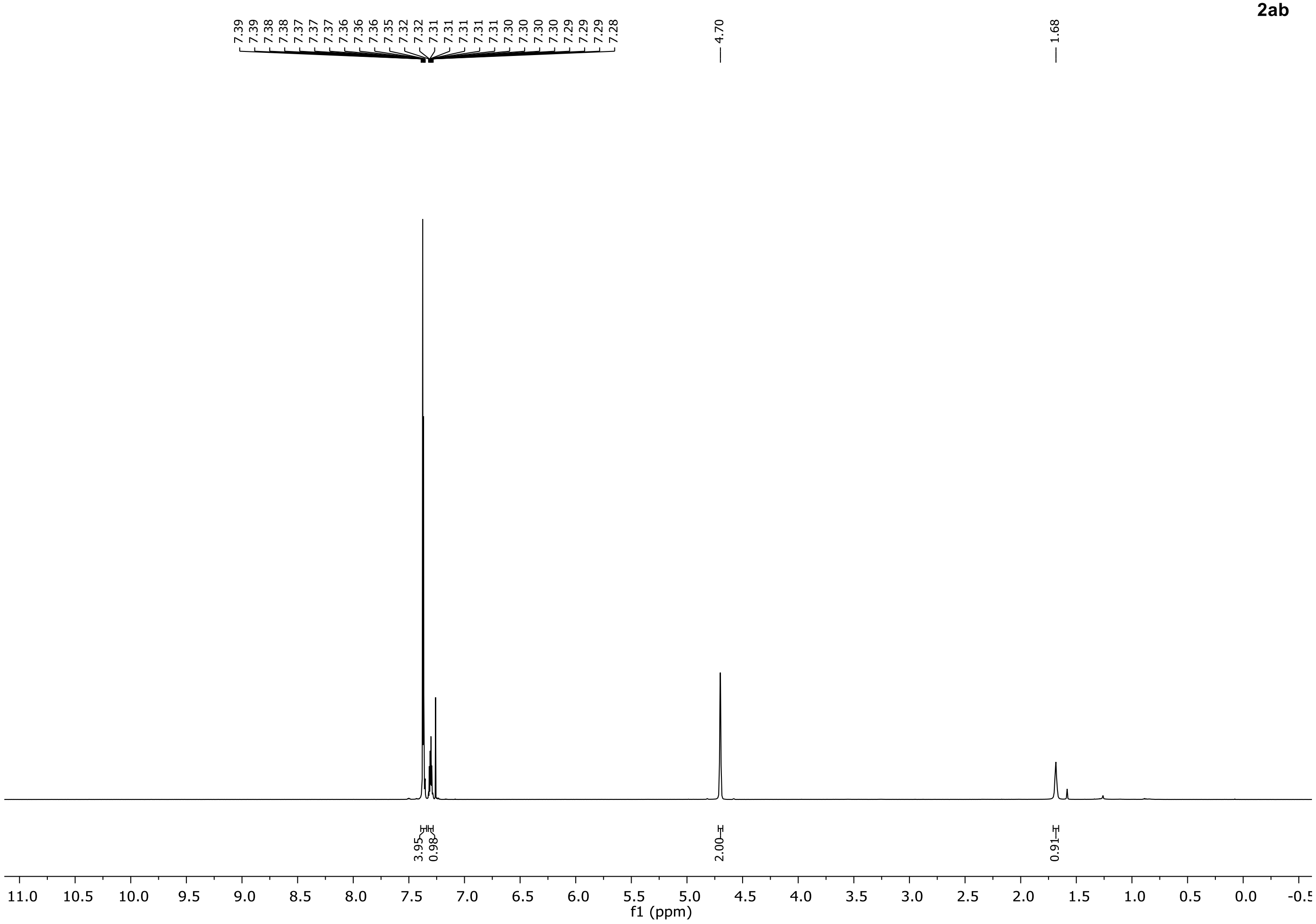

^{13}C NMR

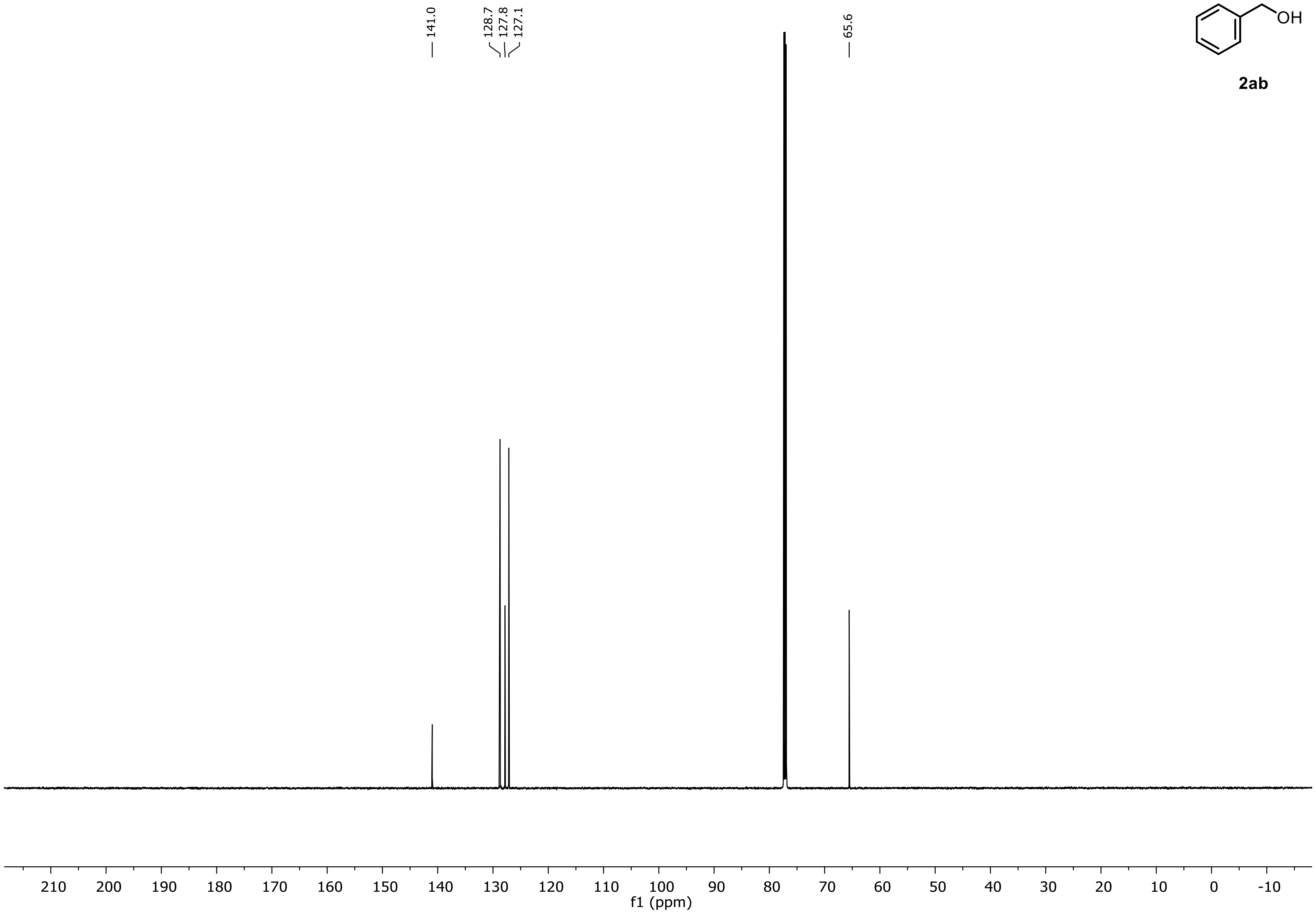
141.0
128.7
127.8
127.1
65.6
210 200 190 180 170 160 150 140 130 120 110 100 90 80 70 60 50 40 30 20 10 0 -10
f1 (ppm)
OH

2ab

^1H, ^1H COSY

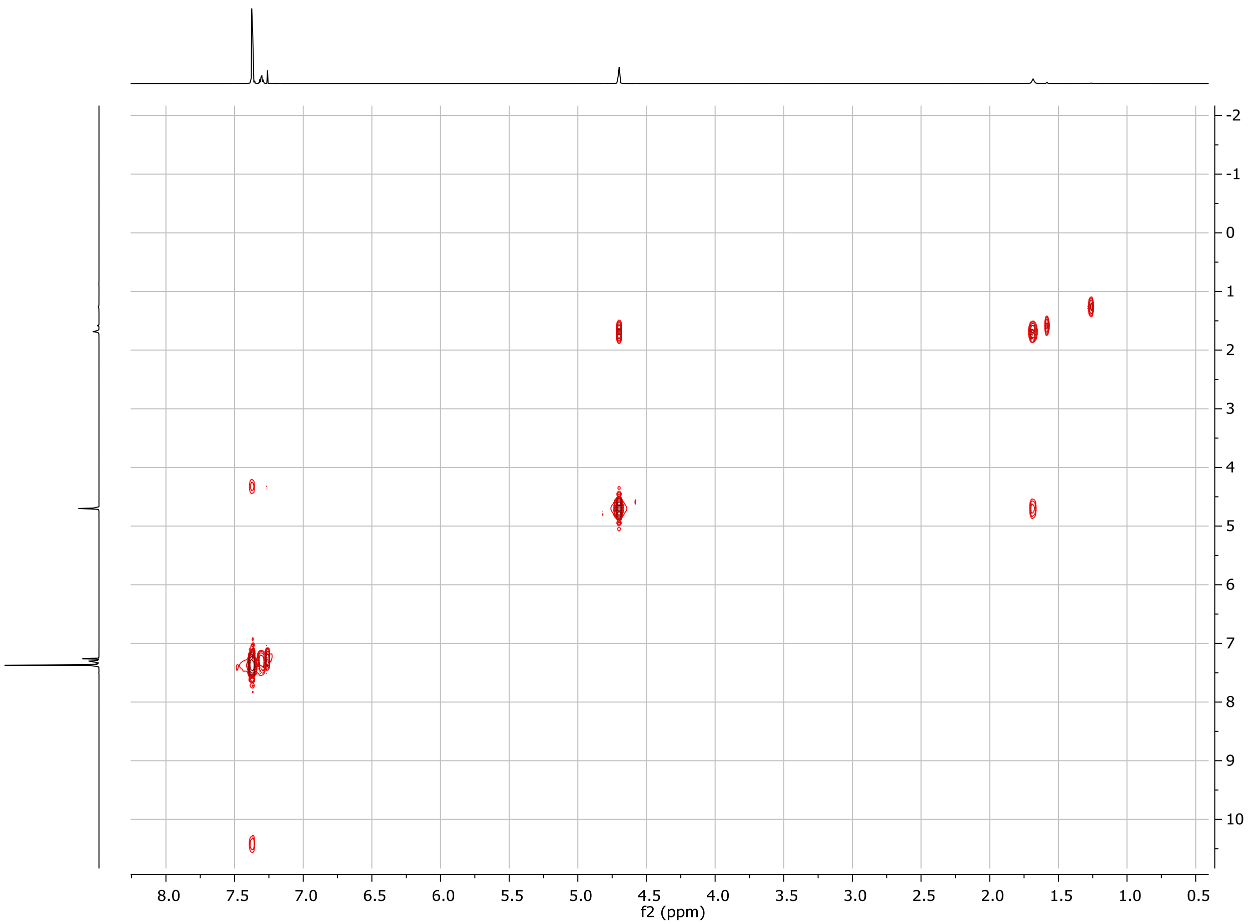

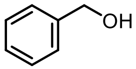

2ab

^{1}H, ^{13}C HMBC

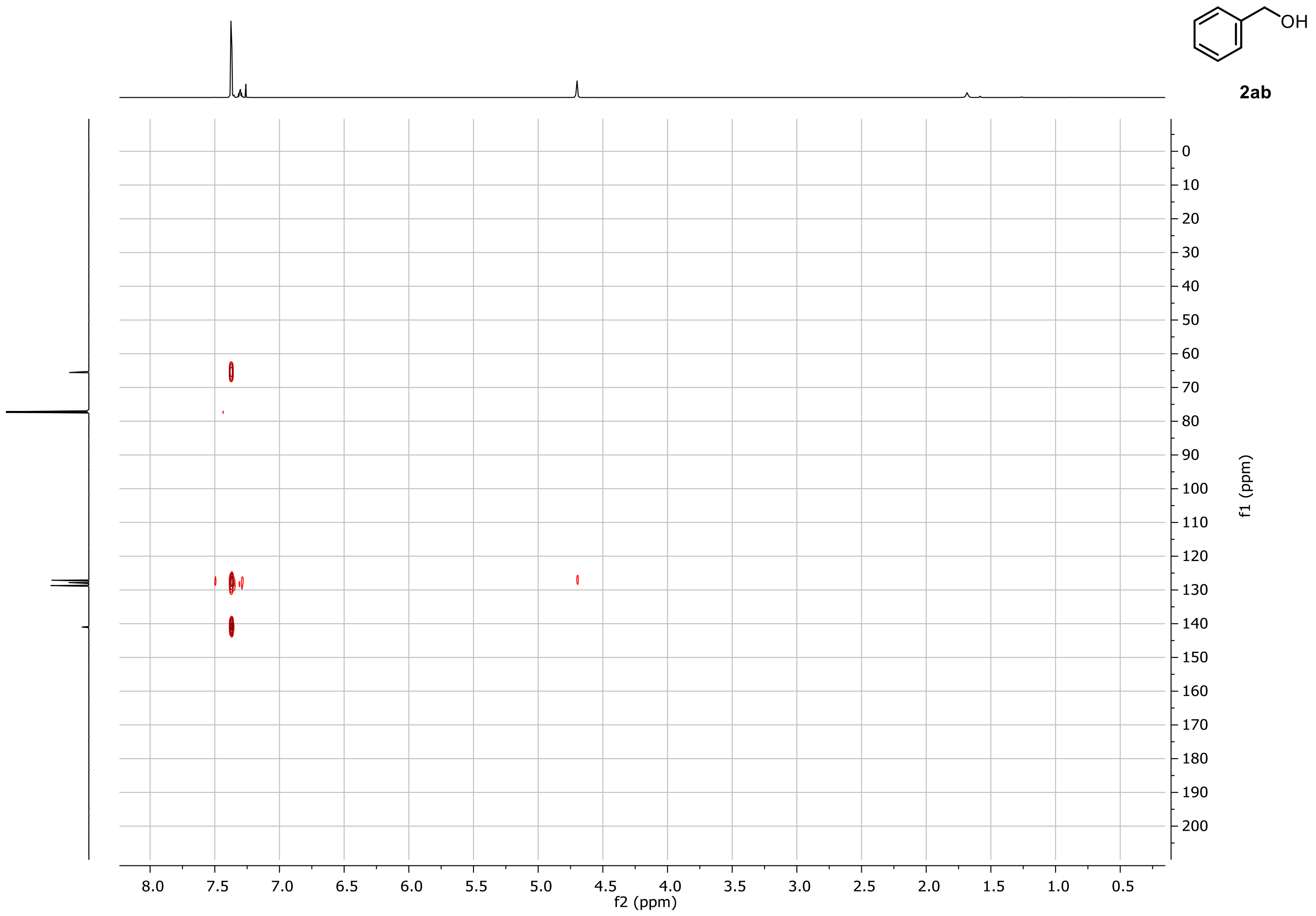

2ab

^{1}H, ^{13}C HSQC

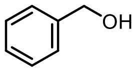

2ab

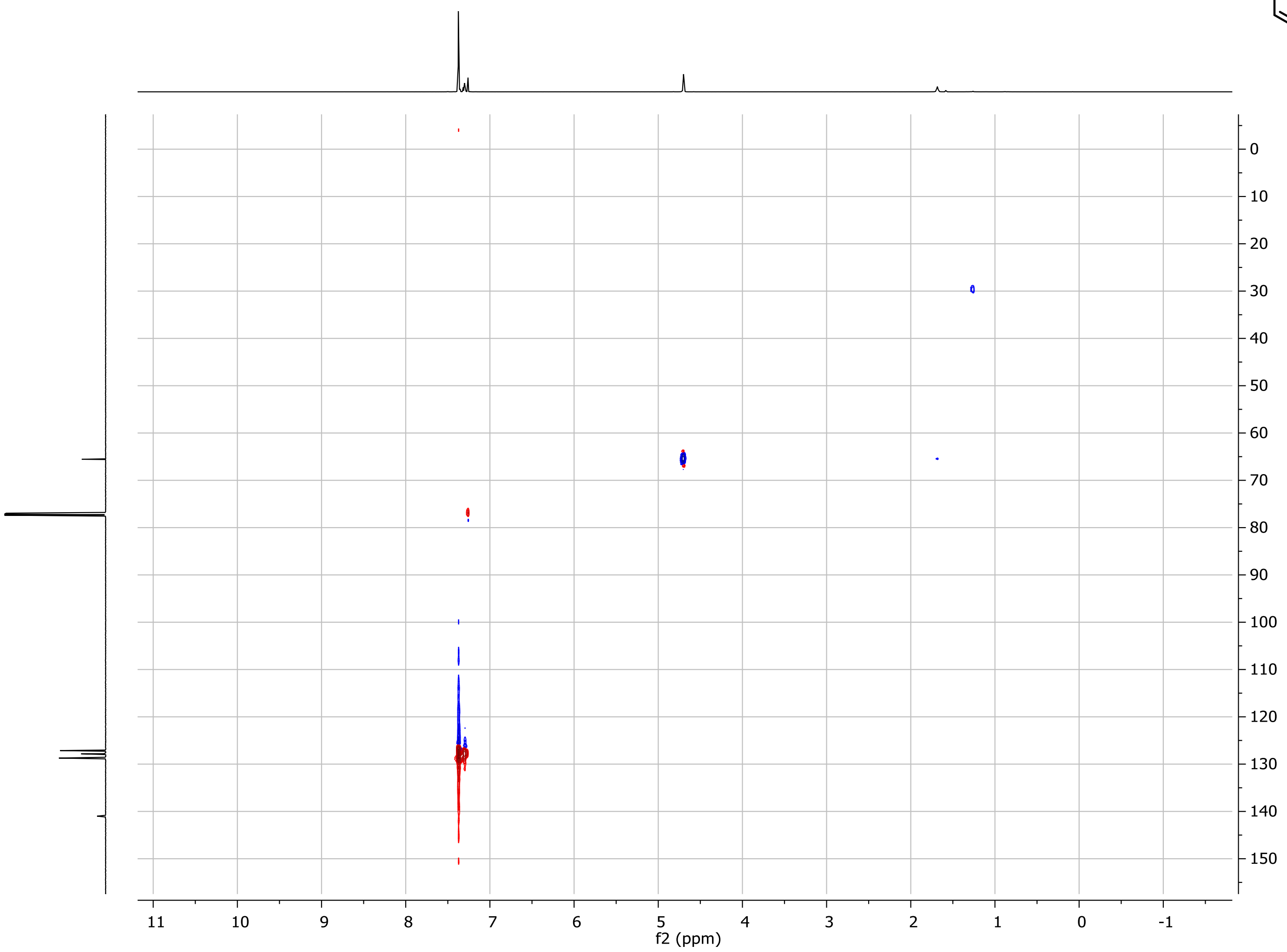

HRMS

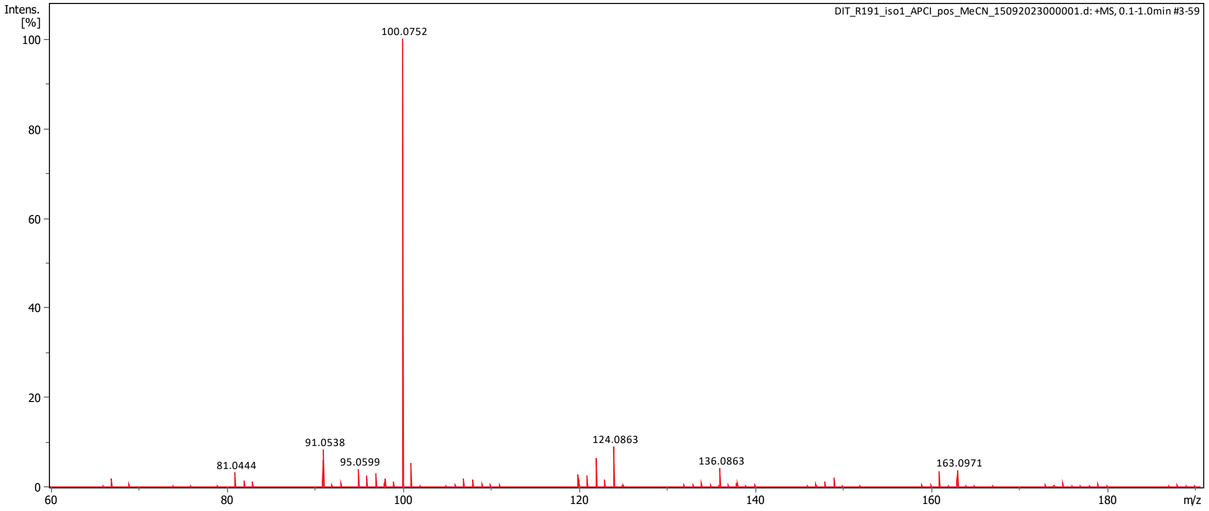
Intens.
[%]
DIT_R191_iso1_APCI_pos_MeCN_15092023000001.d: +MS, 0.1-1.0min #3-59
100.0752
91.0538
81.0444
95.0599
124.0863
136.0863
163.0971
100
80
60
40
20
0
60
80
100
120
140
160
180
m/z

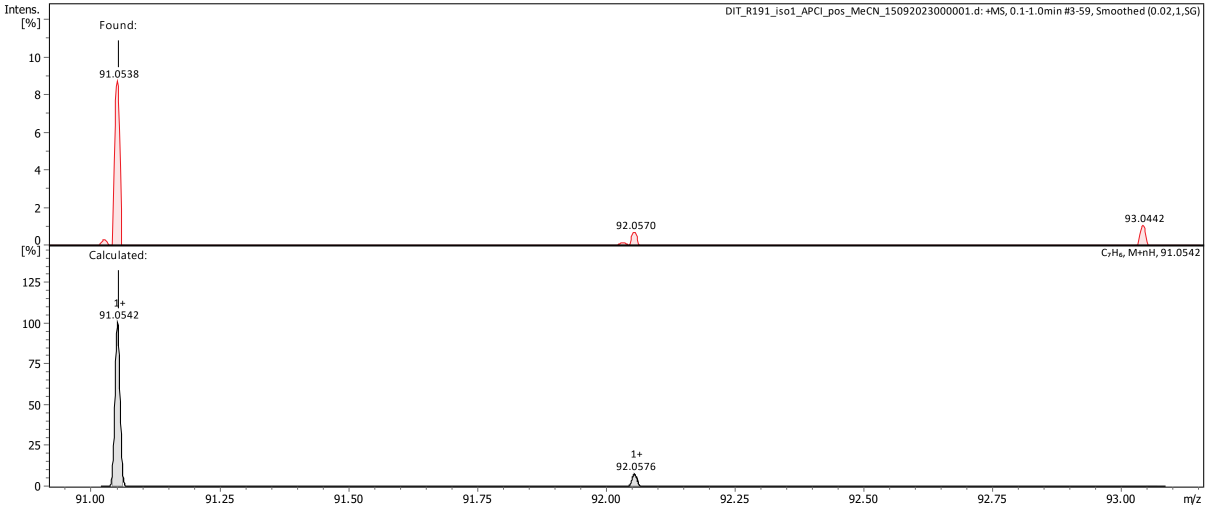
Intens.
[%]
DIT_R191_iso1_APCI_pos_MeCN_15092023000001.d: +MS, 0.1-1.0min #3-59, Smoothed (0.02,1,SG)
Found:
91.0538
92.0570
93.0442
Calculated:
C_7H_6, M+nH, 91.0542
1+
91.0542
1+
92.0576
91.00
91.25
91.50
91.75
92.00
92.25
92.50
92.75
93.00
m/z

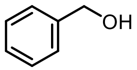
OH

2ab

71 Benzyl alcohol from (4-ethylpiperazin-1-yl)(phenyl)methanone (2ac)

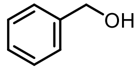

2ac

^{1}H NMR

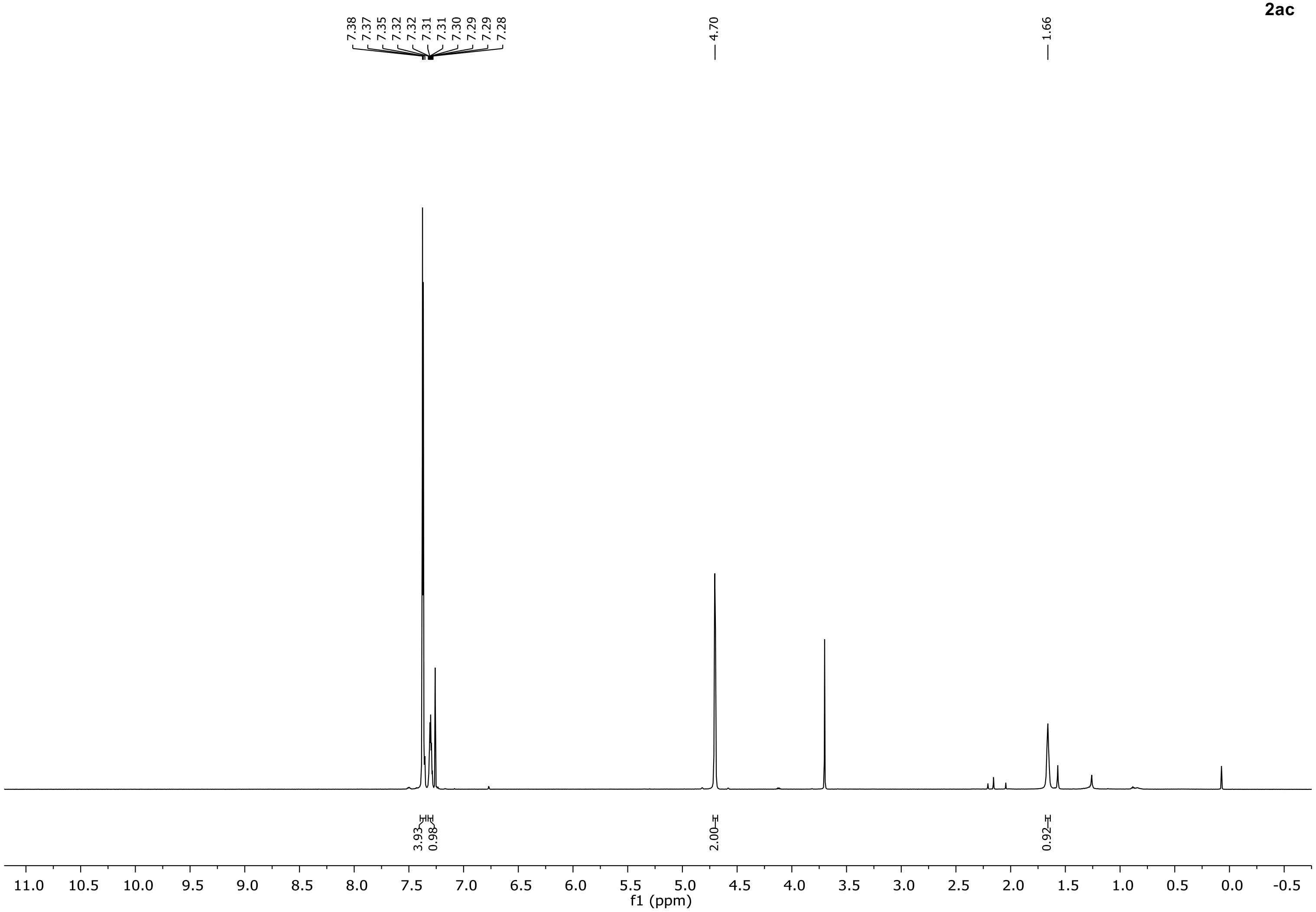

^{13}C NMR

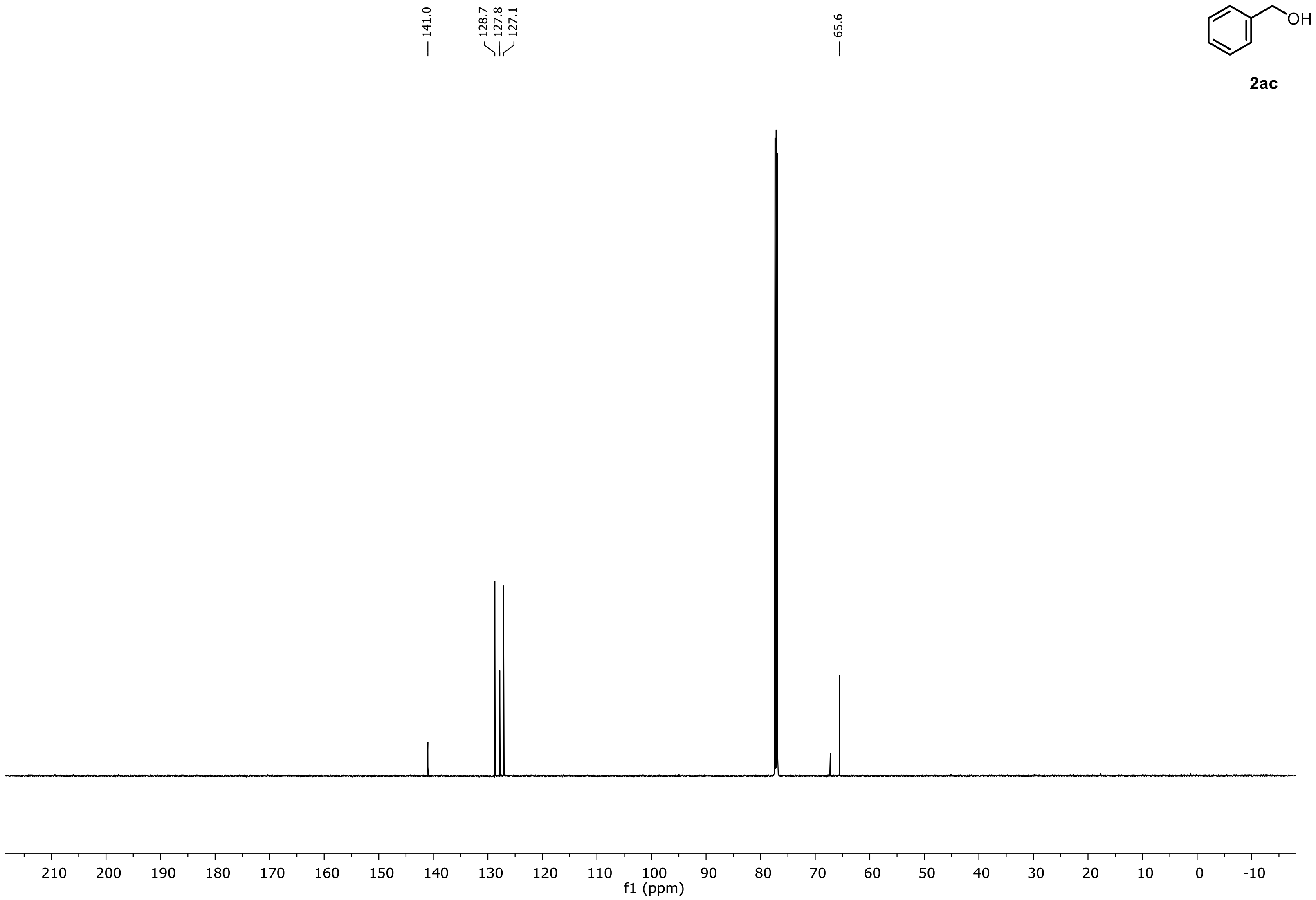

141.0
128.7
127.8
127.1
65.6
210
200
190
180
170
160
150
140
130
120
110
100
90
80
70
60
50
40
30
20
10
0
-10
f1 (ppm)
OH

2ac

^{1}H, ^{1}H COSY

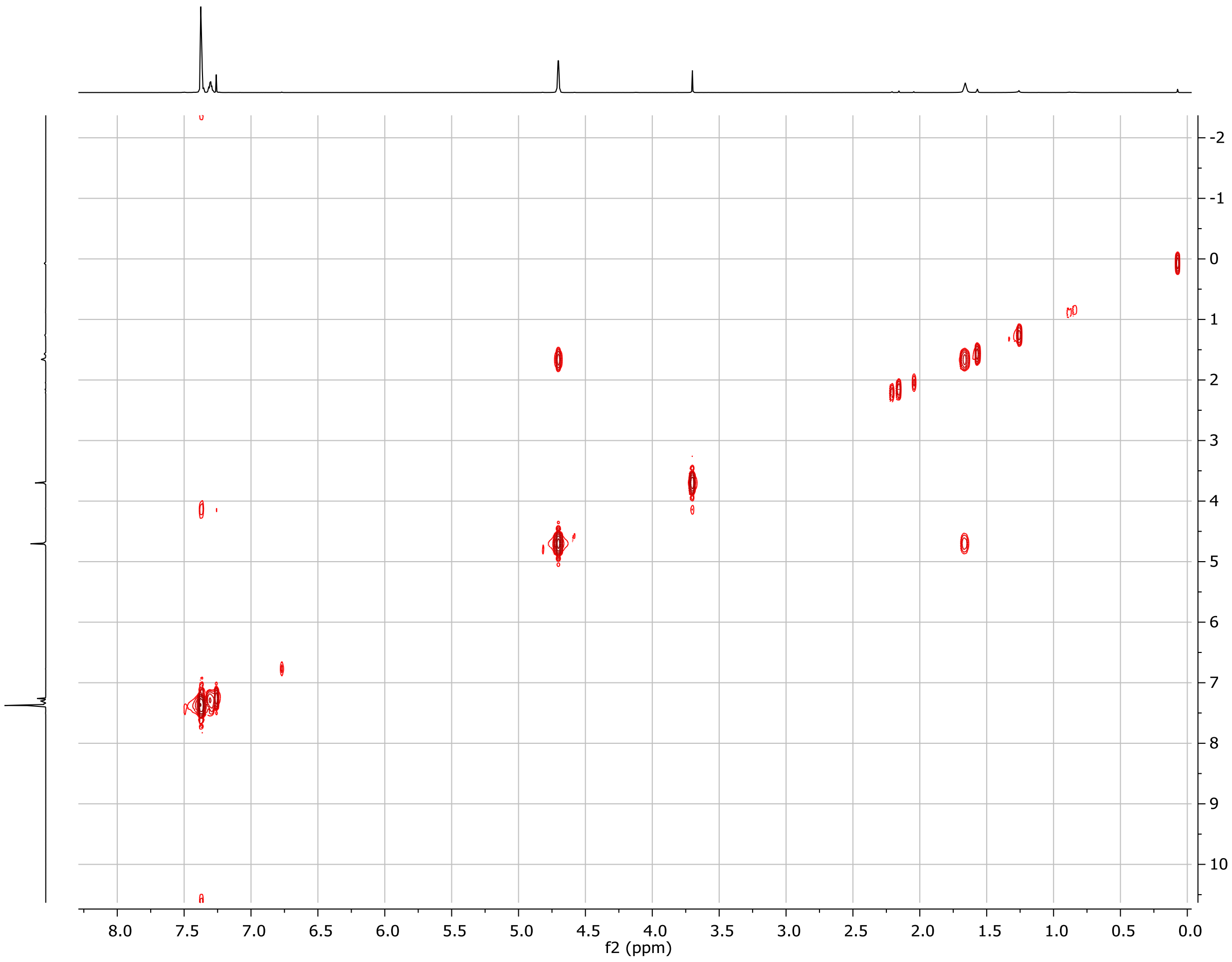

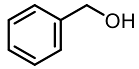

2ac

^{1}H, ^{13}C HMBC

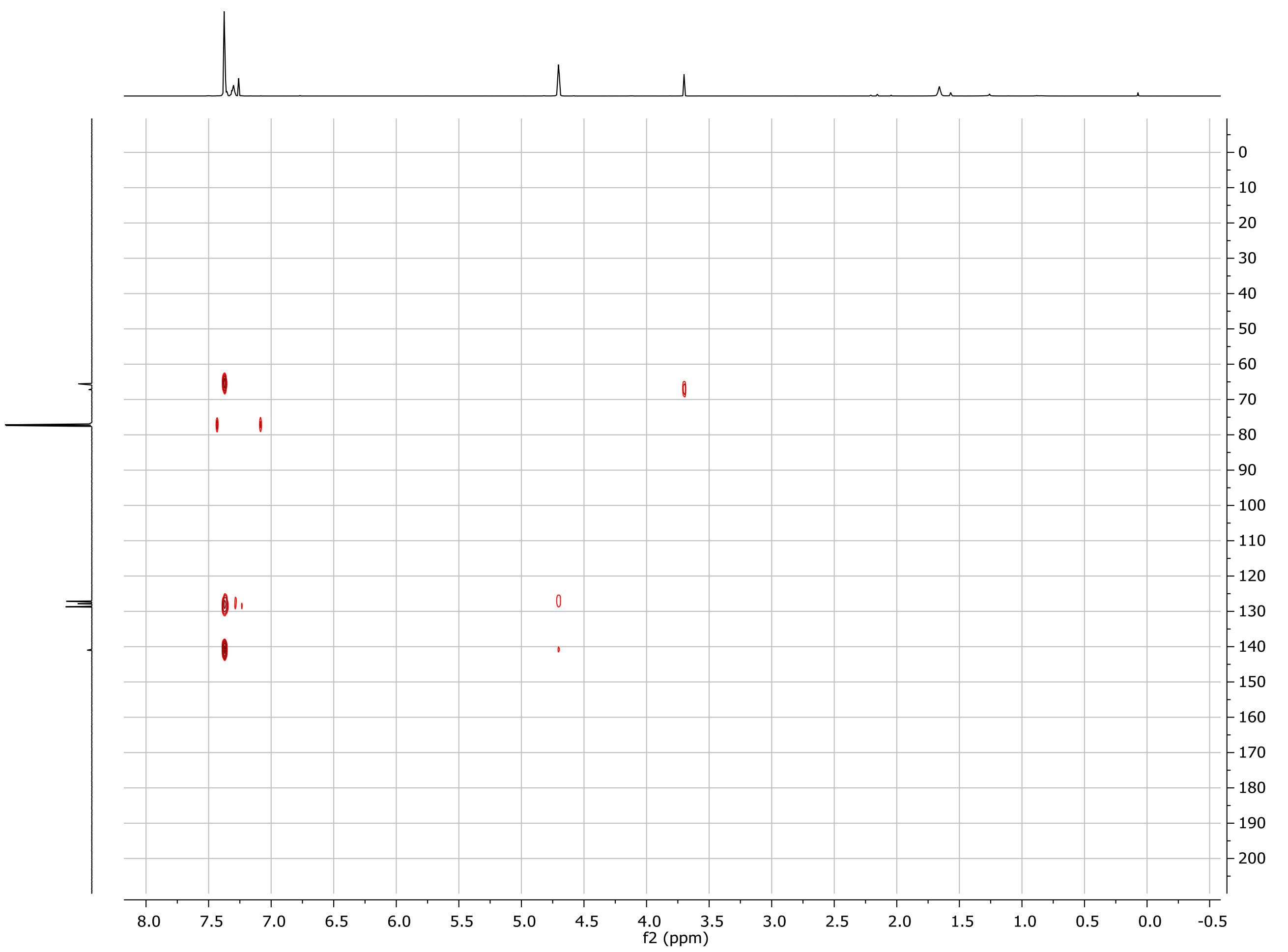

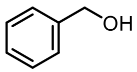

2ac

^1H, ^{13}C HSQC

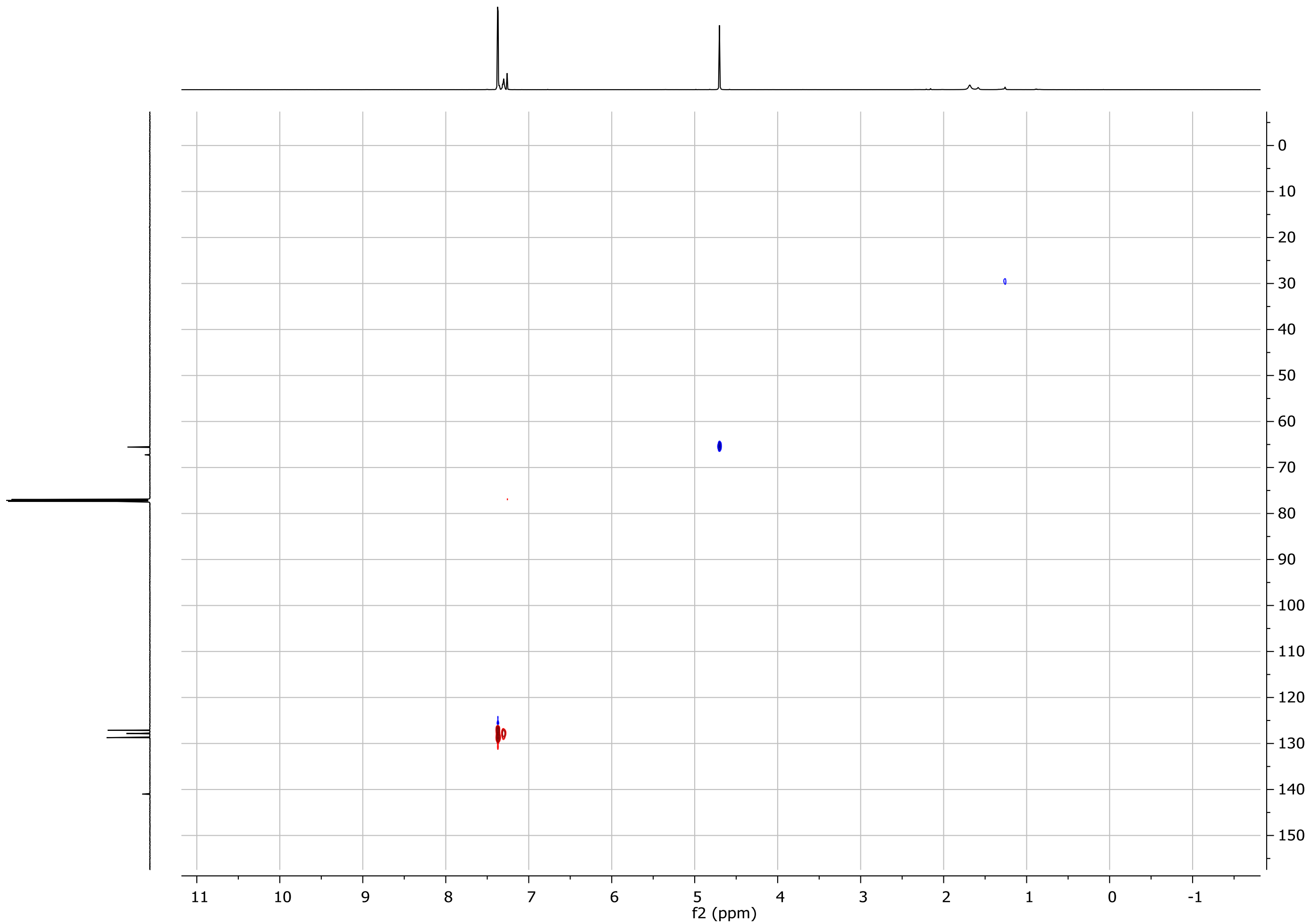

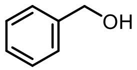

2ac

72 Benzyl alcohol from (4-isopropylpiperazin-1-yl)(phenyl)methanone (2ad)

^{1}H NMR

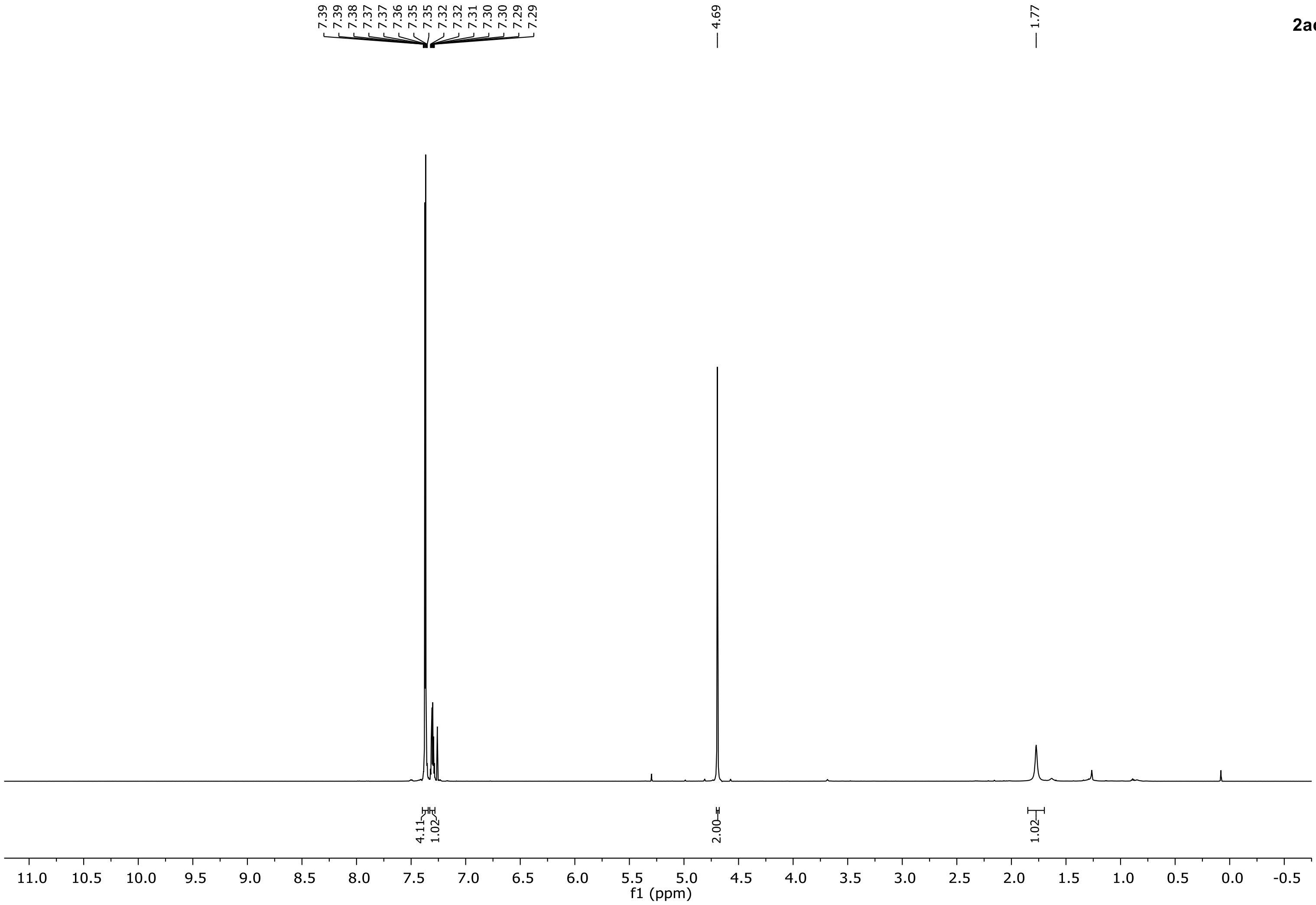

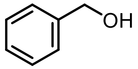

2ad

^{13}C NMR

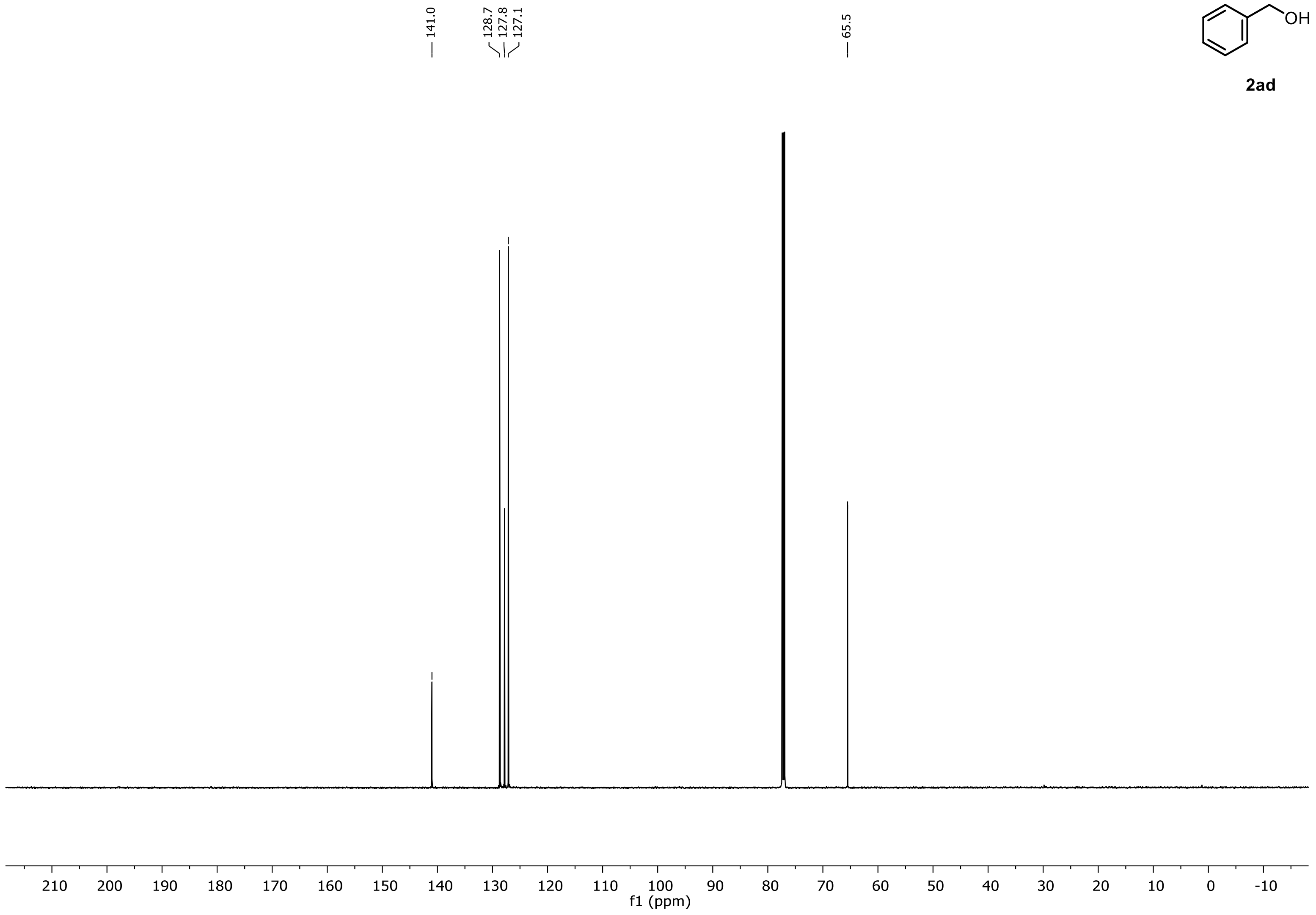

141.0
128.7
127.8
127.1
65.5
OH
2ad
210
200
190
180
170
160
150
140
130
120
110
100
90
80
70
60
50
40
30
20
10
0
-10
f1 (ppm)

^1H, ^1H COSY

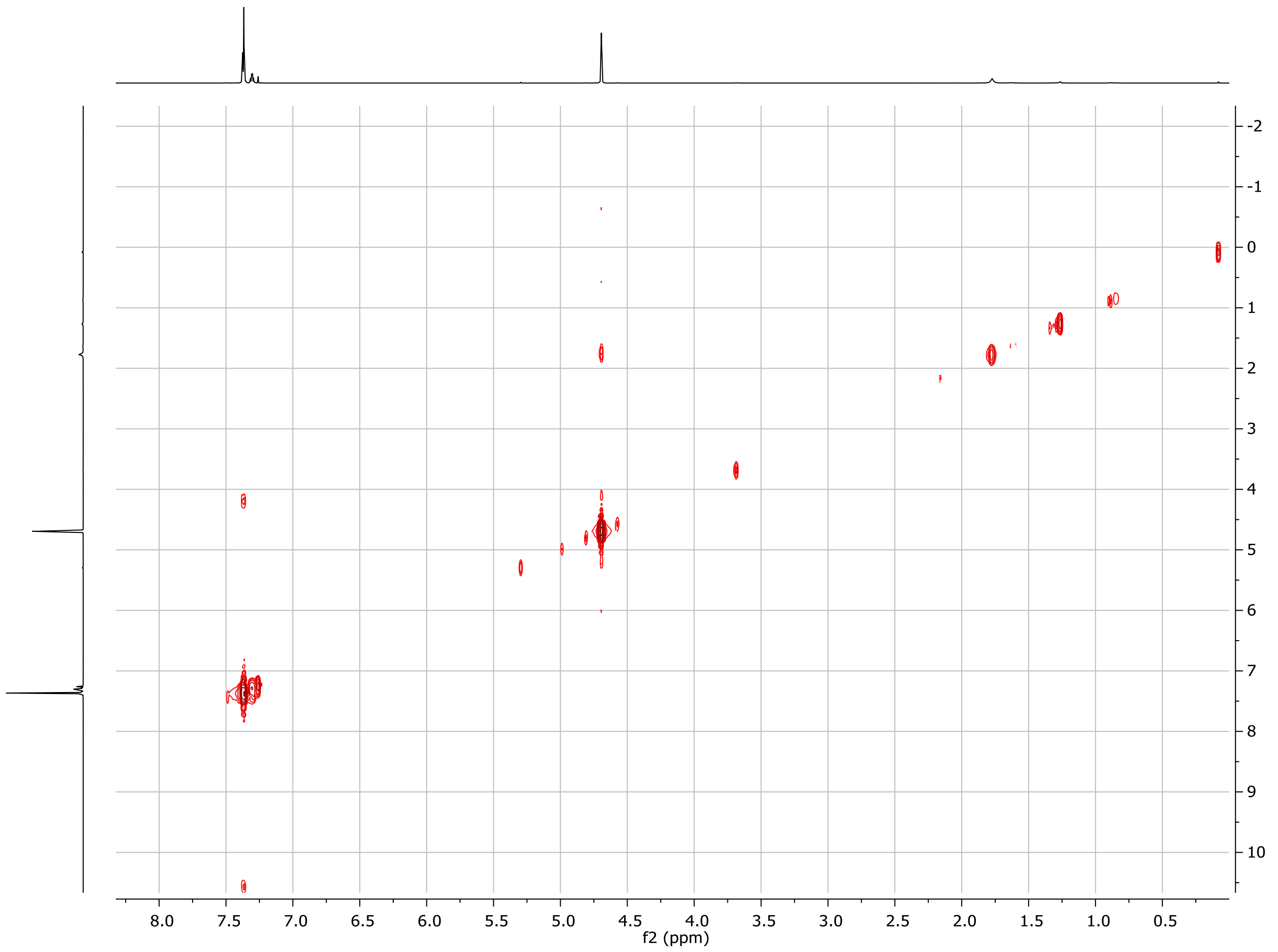

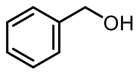

2ad

^{1}H, ^{13}C HMBC

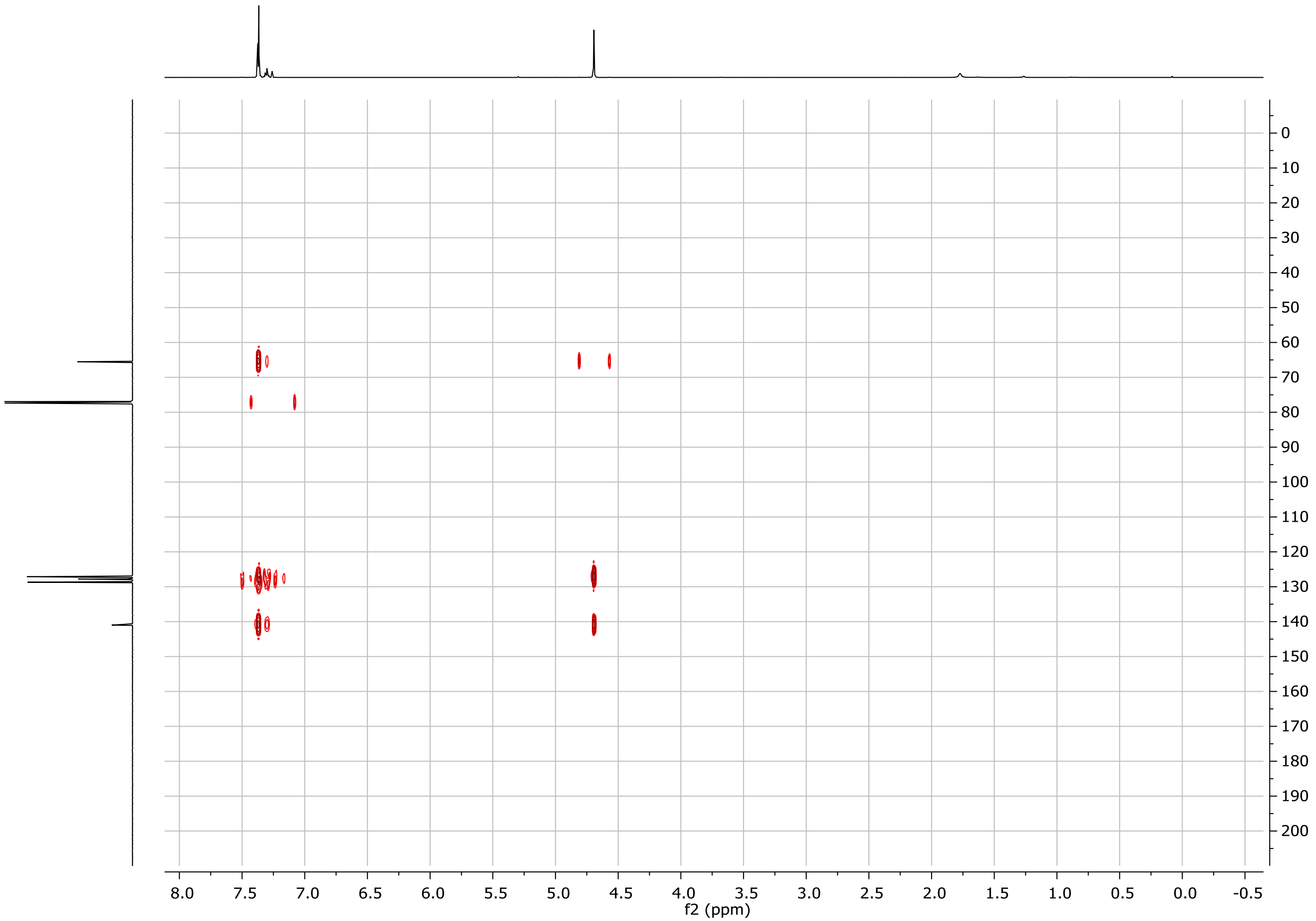

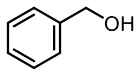

2ad

^{1}H, ^{13}C HSQC

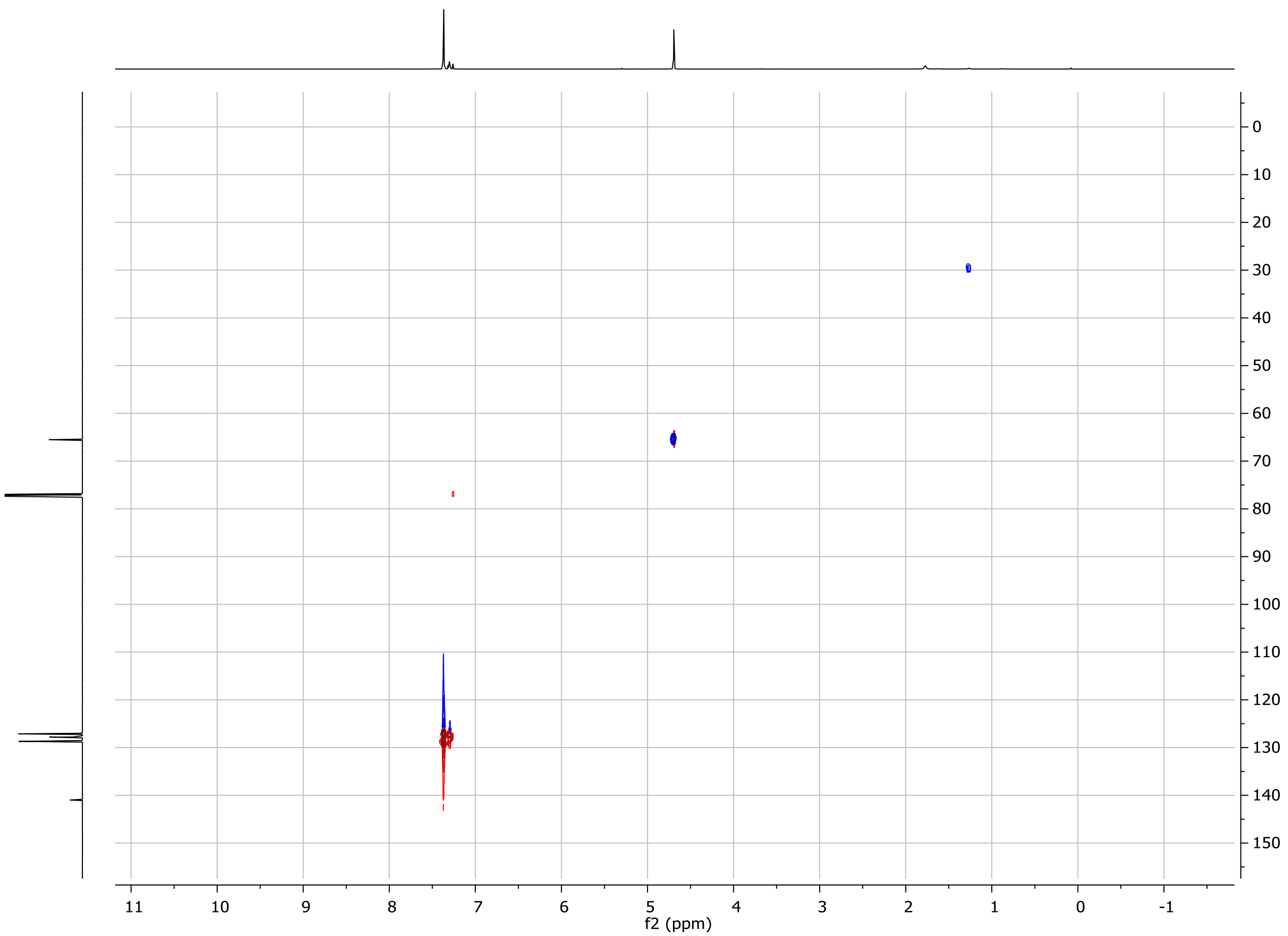

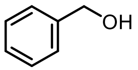

2ad

73 Benzyl alcohol from phenyl(4-(pyridin-2-yl)piperazin-1-yl)methanone (2ae)

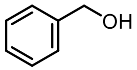

2ae

^{1}H NMR

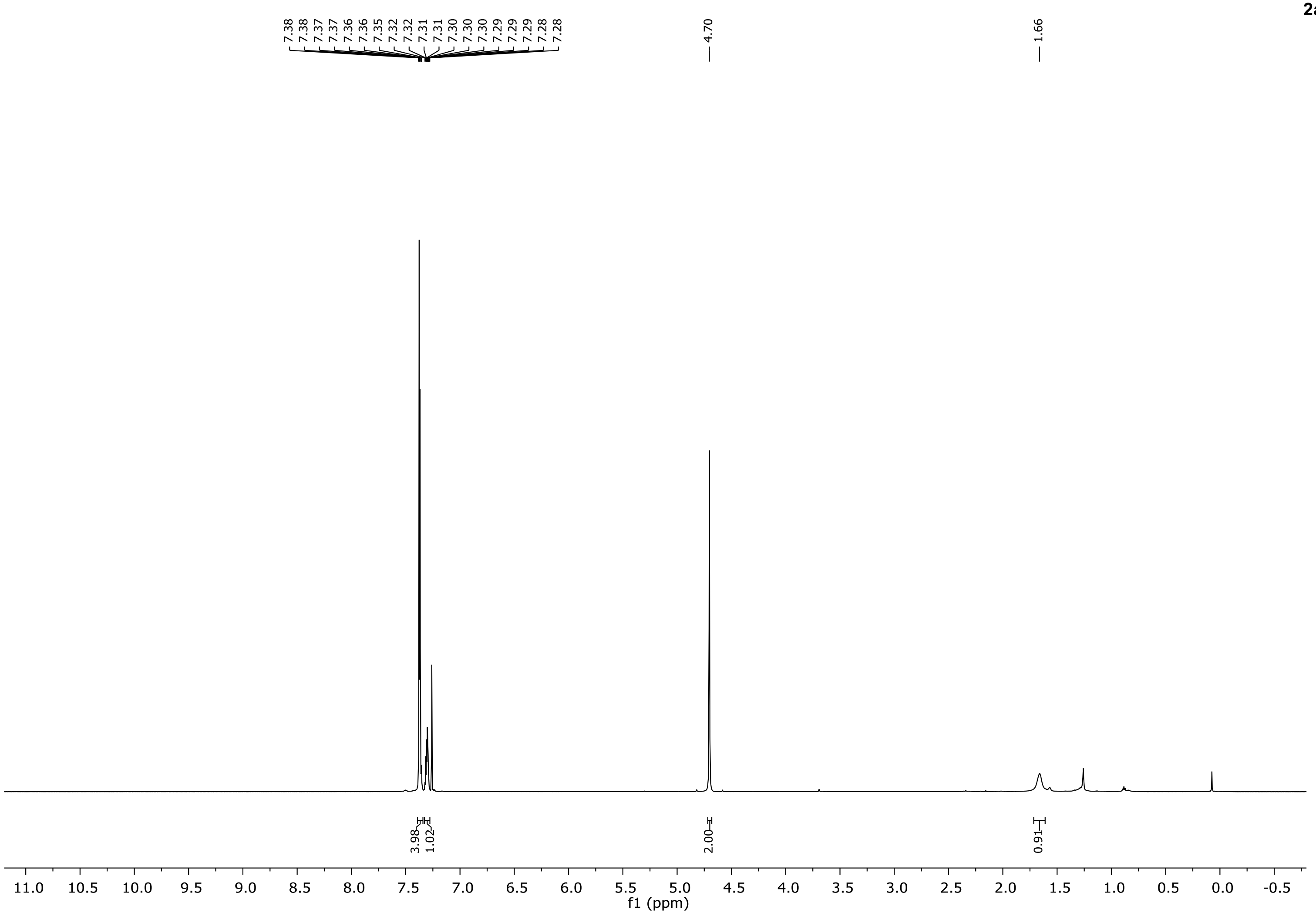

^{13}C NMR

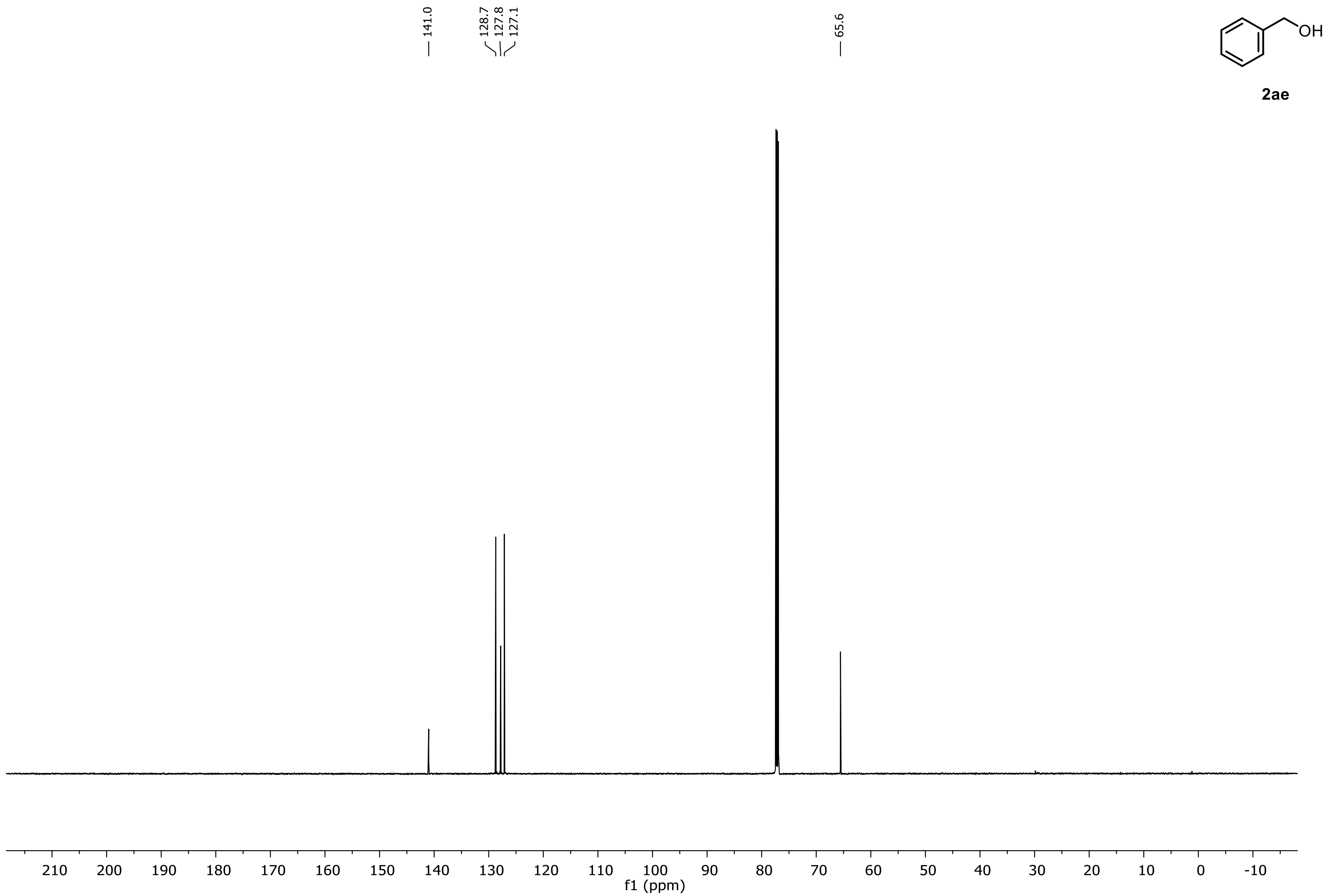

141.0
128.7
127.8
127.1
65.6
210
200
190
180
170
160
150
140
130
120
110
100
90
80
70
60
50
40
30
20
10
0
-10
f1 (ppm)
OH

2ae

^{1}H, ^{1}H COSY

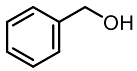

2ae

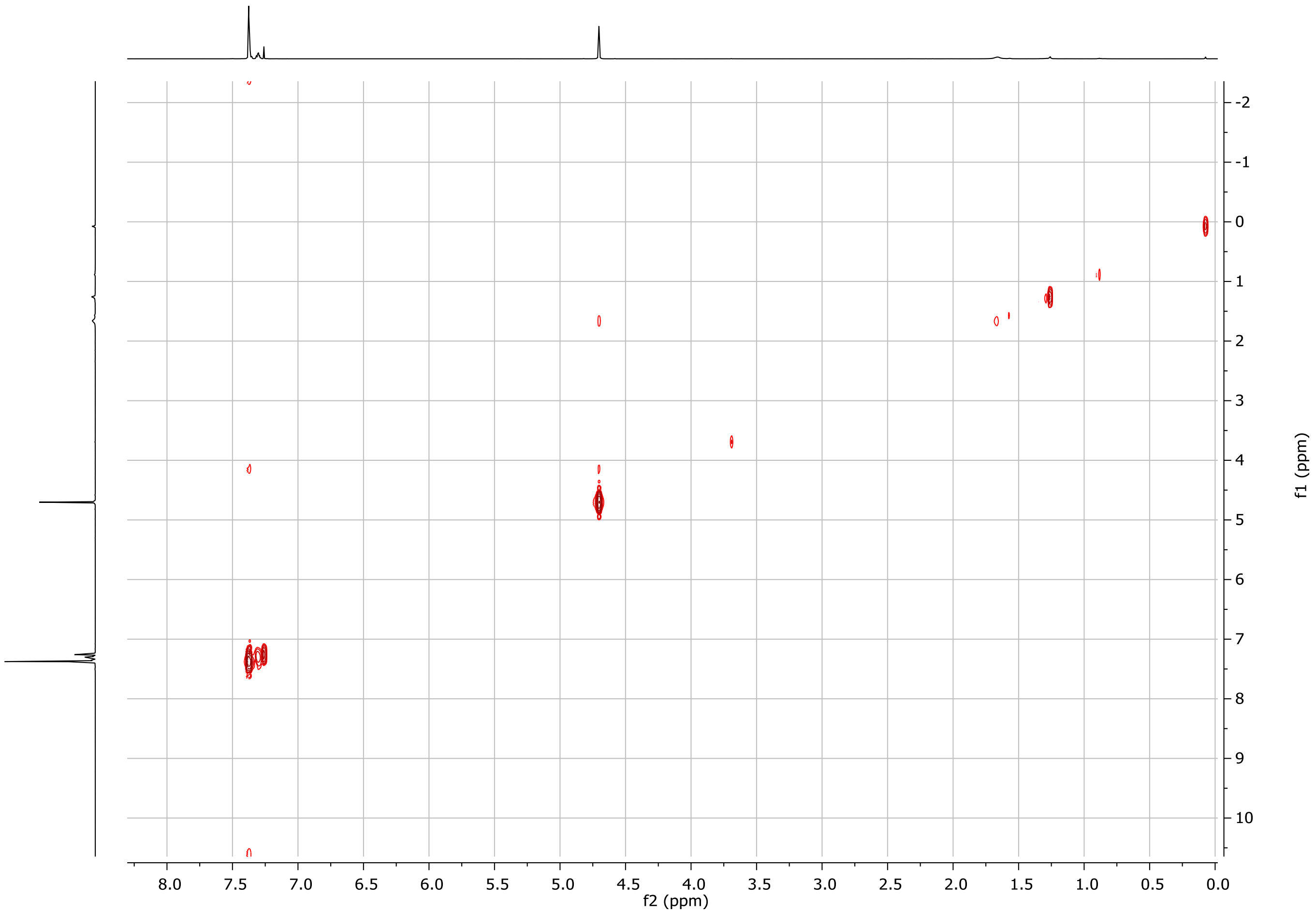

^{1}H, ^{13}C HMBC

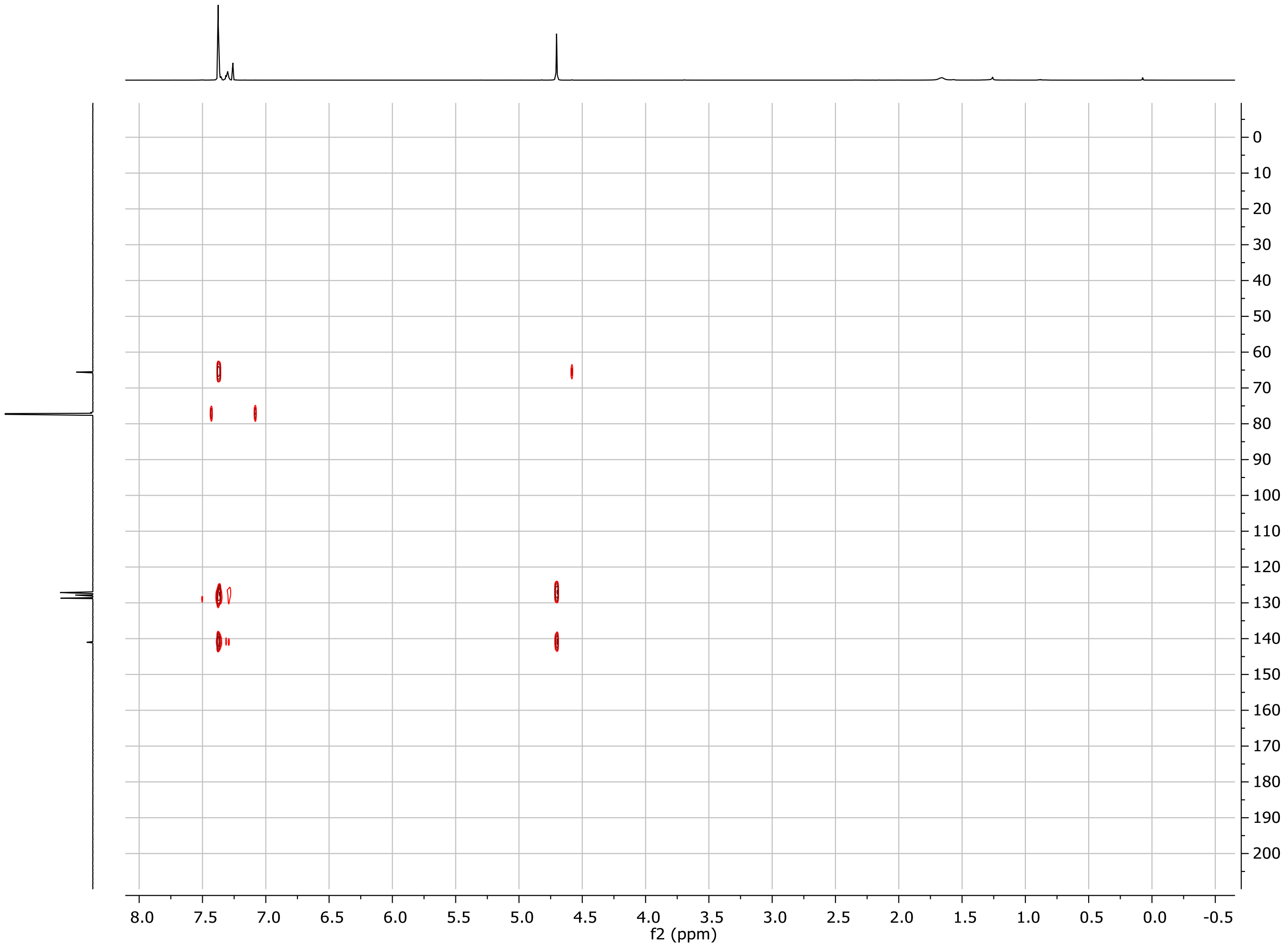

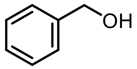

2ae

^{1}H, ^{13}C HSQC

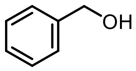

2ae

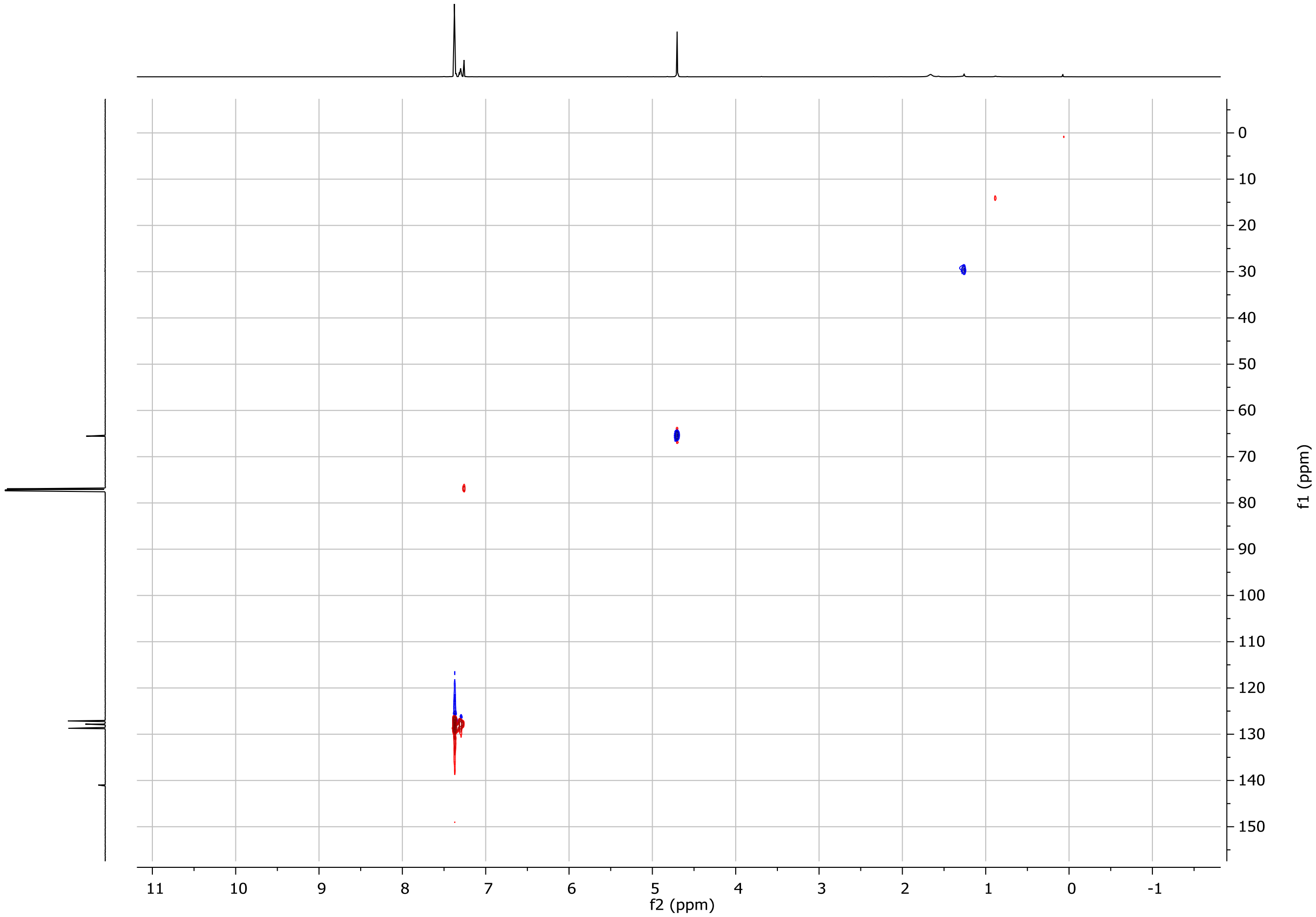

74 2-[4-(Hydroxymethyl)phenyl]-1-(1-piperidyl)ethanone (12)

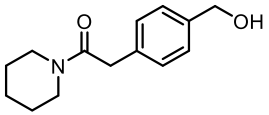

12

^{1}H NMR

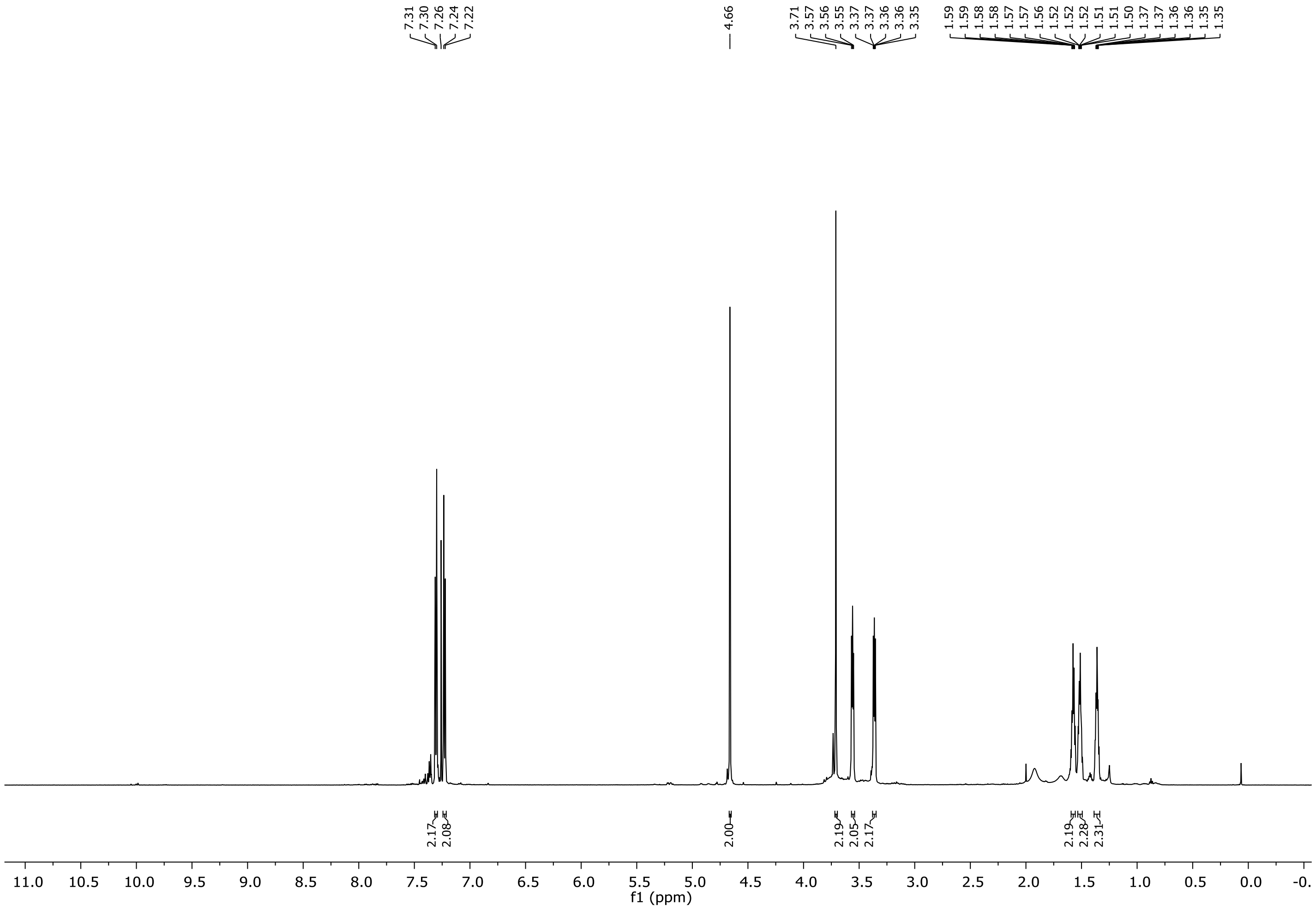

^{13}C NMR

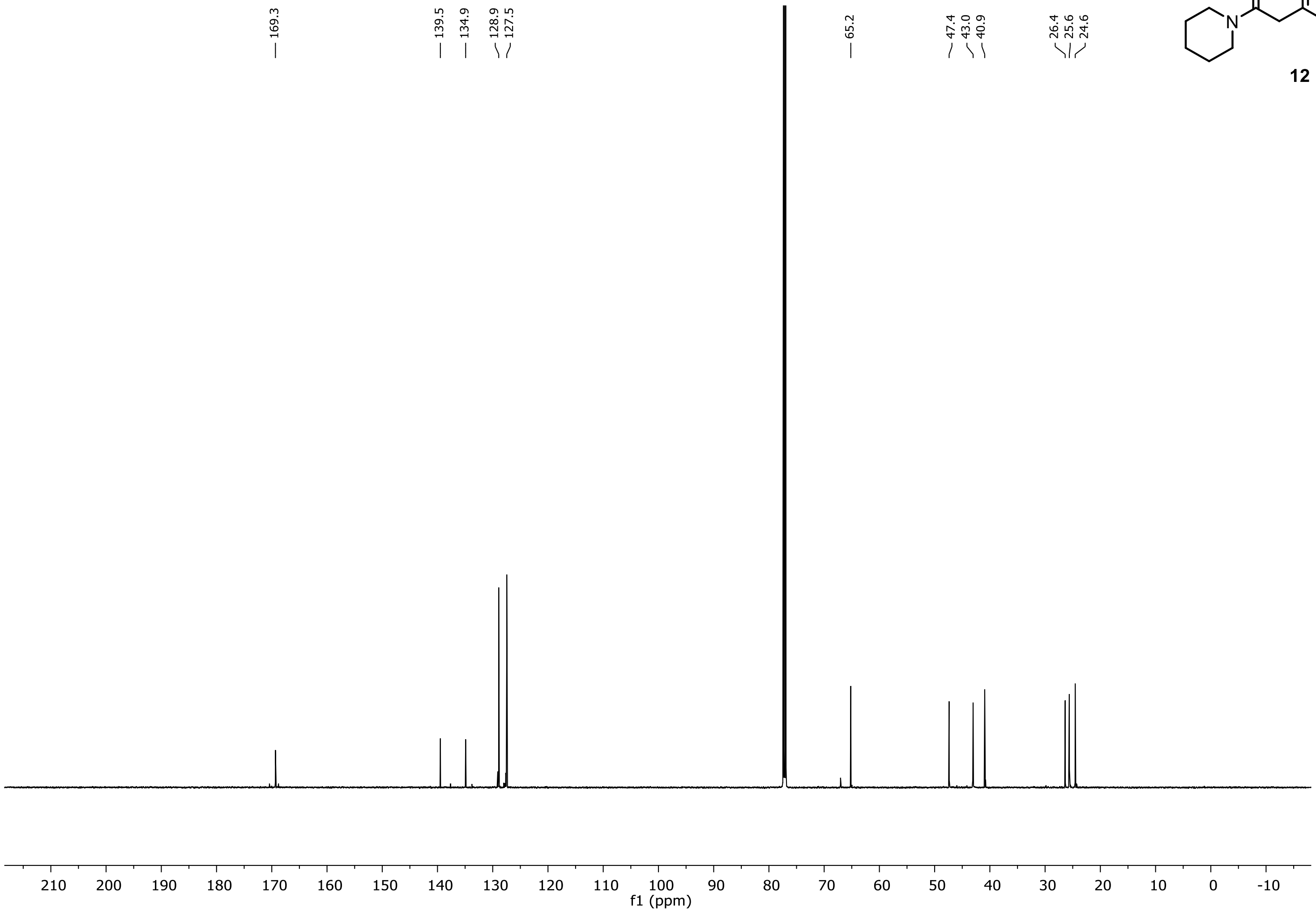

169.3
139.5
134.9
128.9
127.5
65.2
47.4
43.0
40.9
26.4
25.6
24.6
210 200 190 180 170 160 150 140 130 120 110 100 90 80 70 60 50 40 30 20 10 0 -10
f1 (ppm)

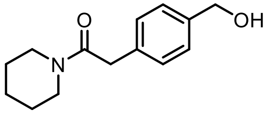

O
N
OH

12

^{1}H, ^{1}H COSY

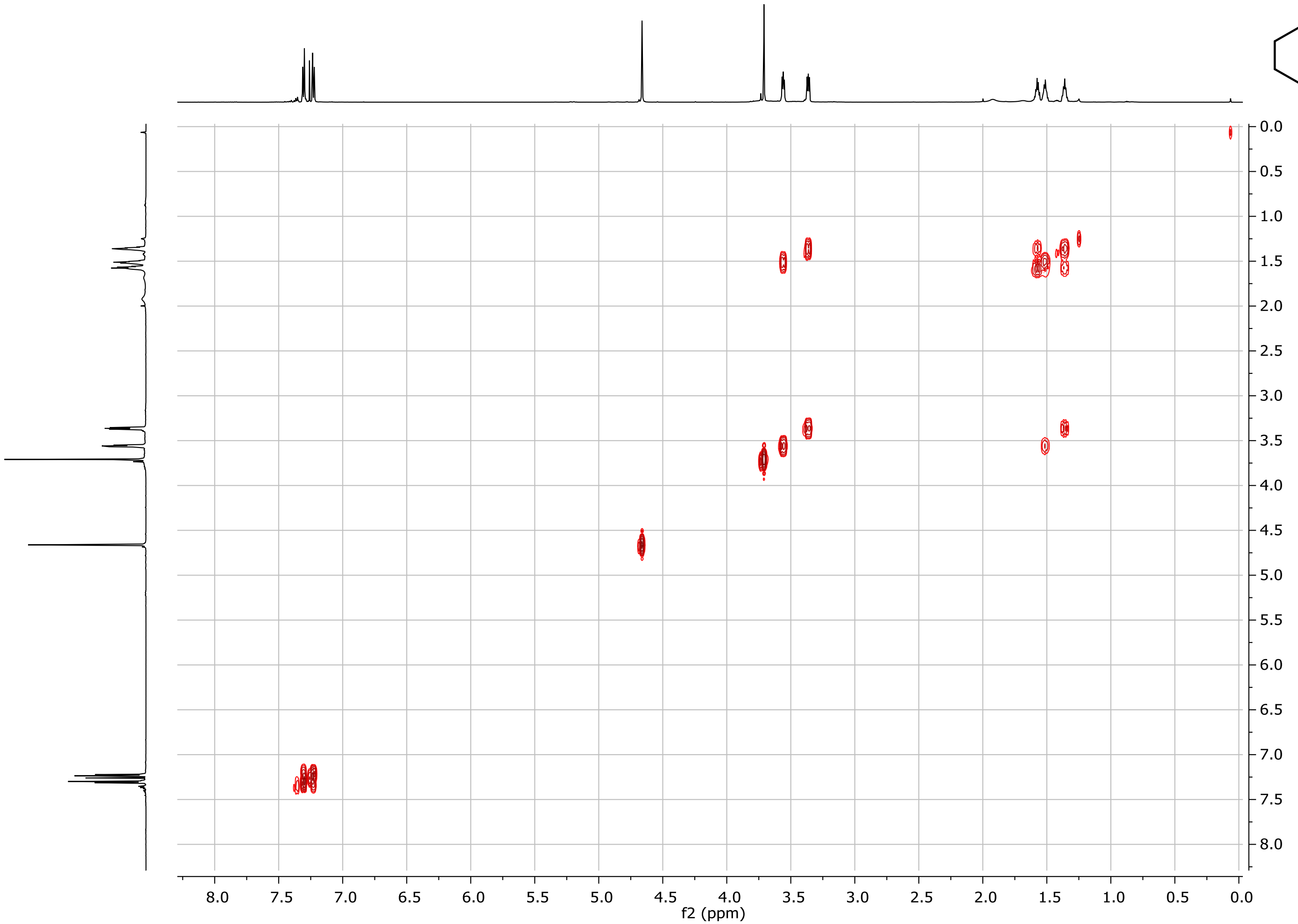

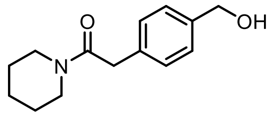

12

^{1}H, ^{13}C HMBC

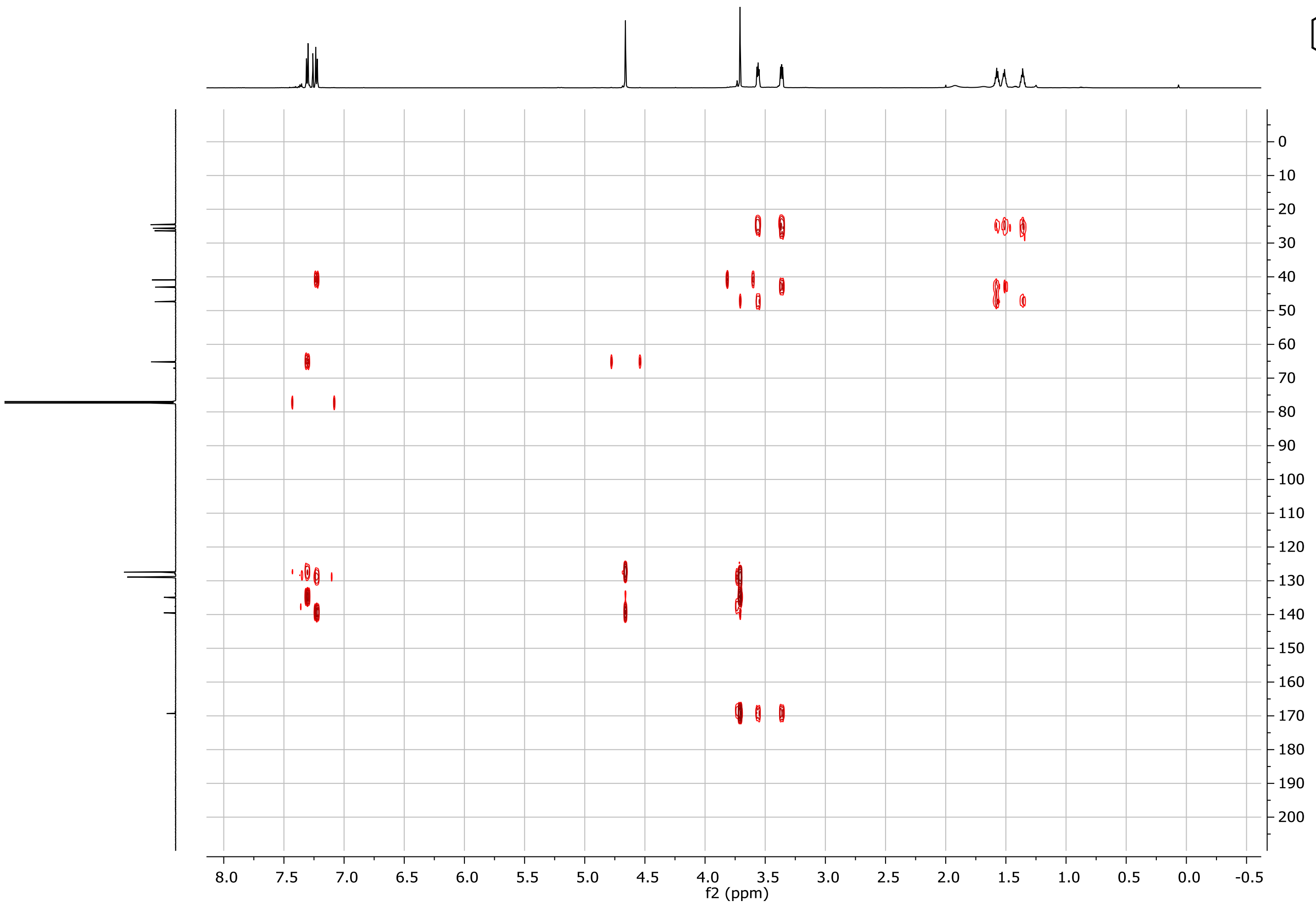

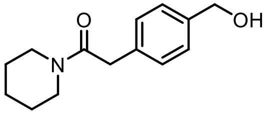

12

^1H, ^{13}C HSQC

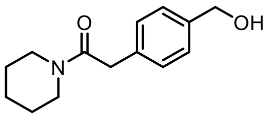

12

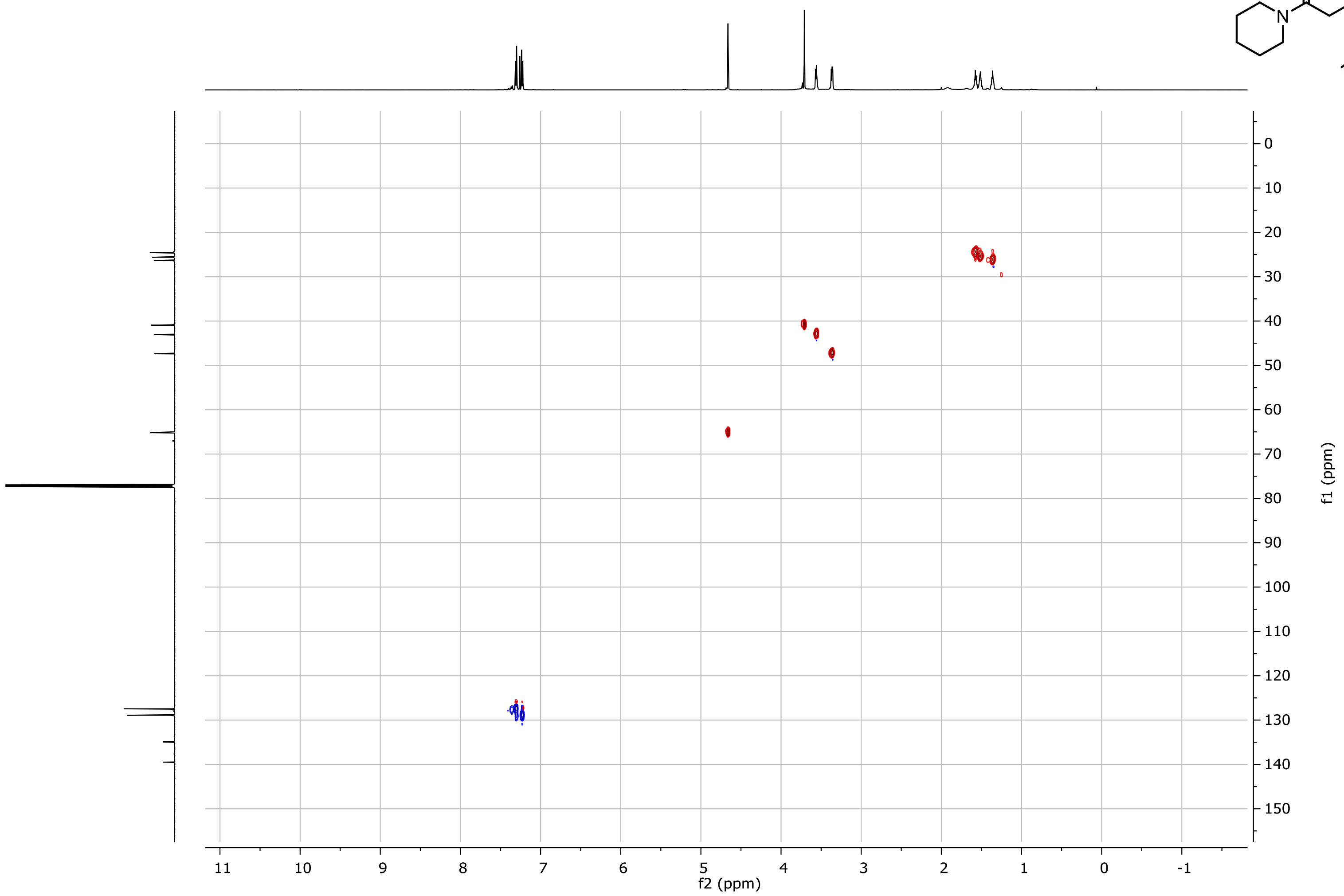

HRMS

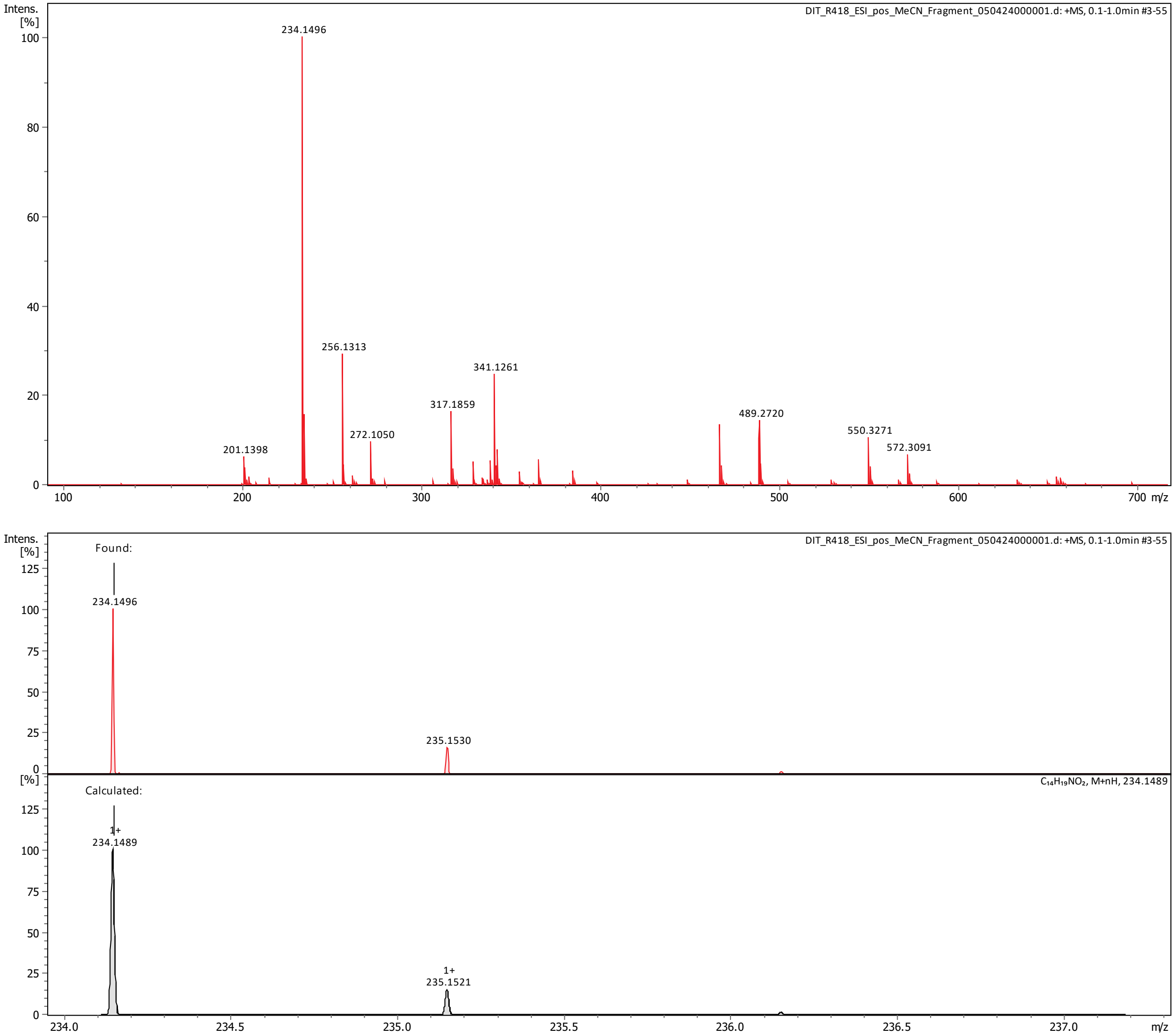

Intens. [%]
DIT_R418_ESI_pos_MeCN_Fragment_050424000001.d: +MS, 0.1-1.0min #3-55
234.1496
256.1313
341.1261
317.1859
272.1050
201.1398
489.2720
550.3271
572.3091
m/z
Found:
234.1496
235.1530
Calculated:
$C_{14}H_{19}NO_2$, M+nH, 234.1489
1+
234.1489
1+
235.1521

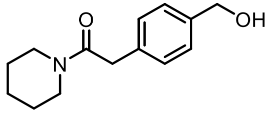

O
N
OH

12

IR

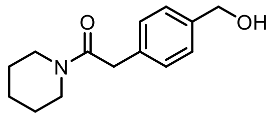

O
N
OH

12

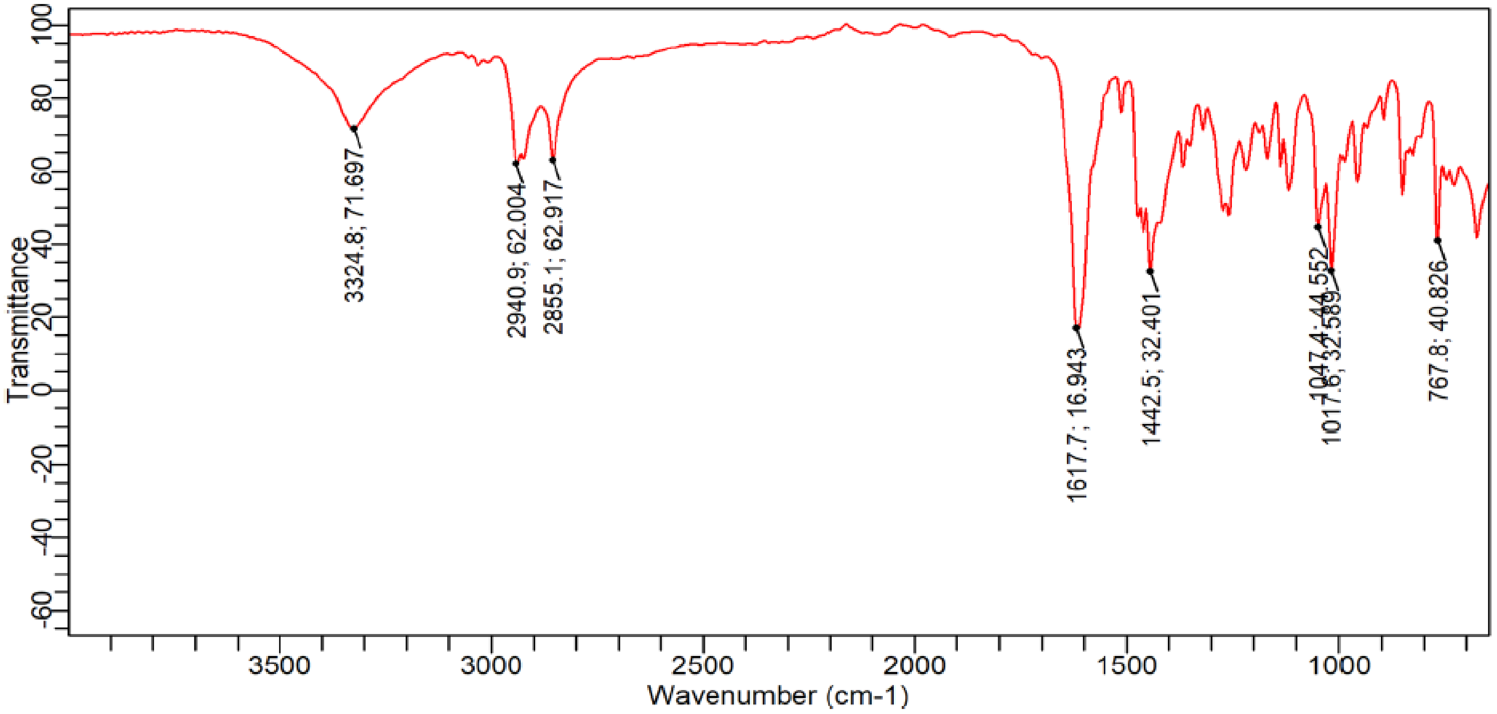

Transmittance
Wavenumber (cm-1)
3324.8; 71.697
2940.9; 62.004
2855.1; 62.917
1617.7; 16.943
1442.5; 32.401
1047.4; 44.552
1017.6; 32.589
767.8; 40.826

75 Benzyl alcohol from 3-(4-benzoylpiperazin-1-yl)-1-(1-piperidyl)propan-1-one (14)

^{1}H NMR

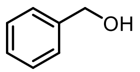

14

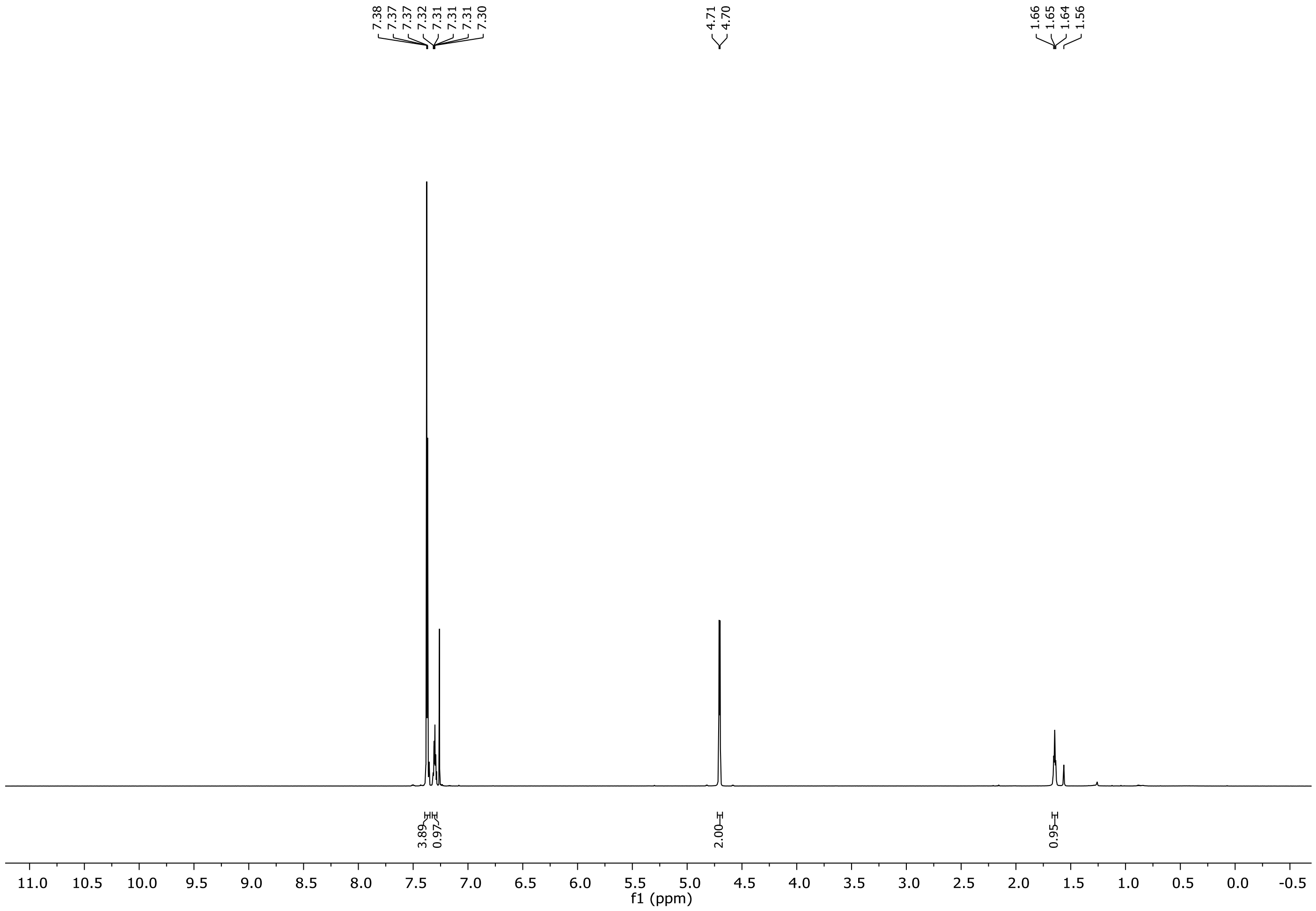

^{13}C NMR

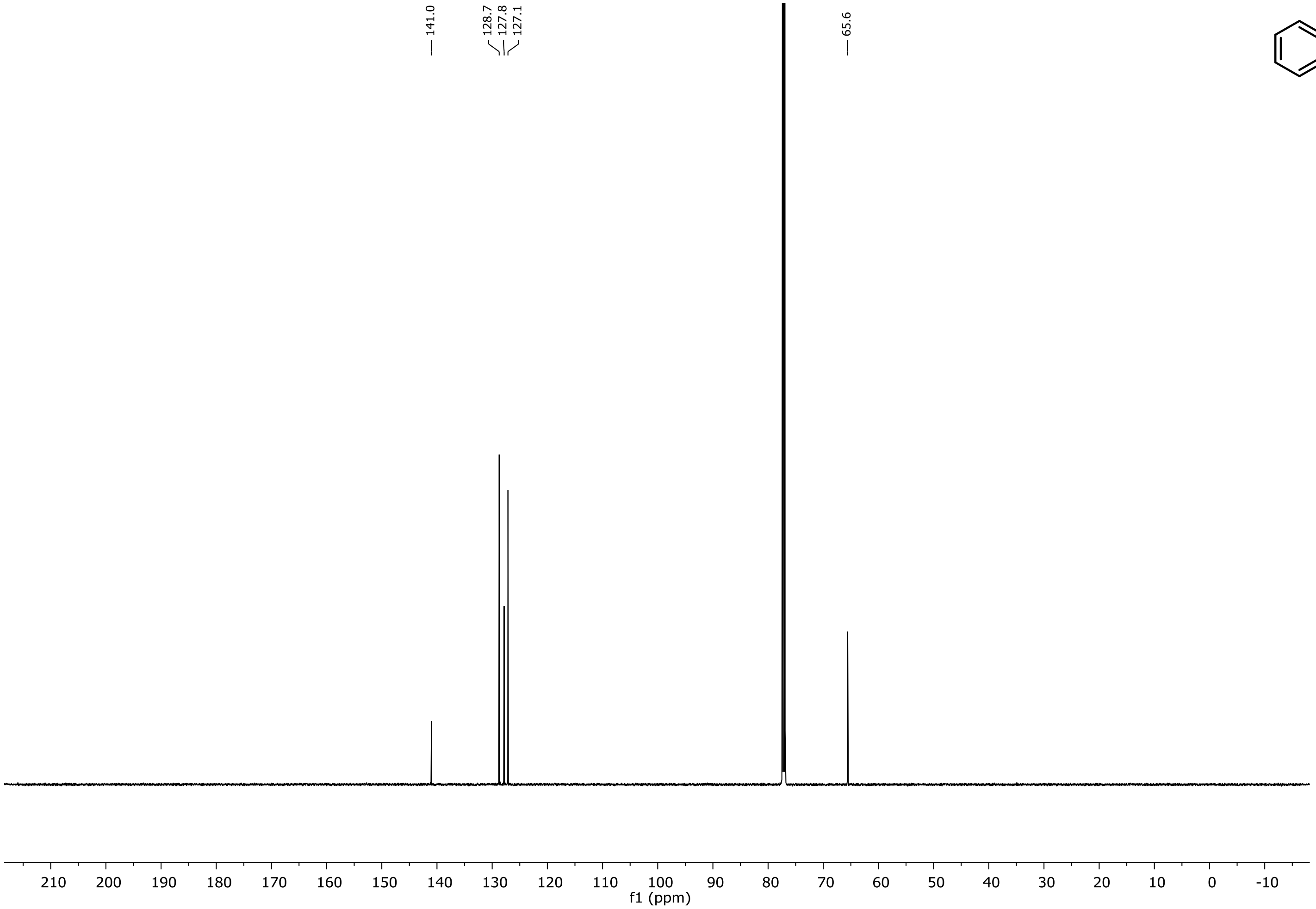
141.0
128.7
127.8
127.1
65.6
210
200
190
180
170
160
150
140
130
120
110
100
90
80
70
60
50
40
30
20
10
0
-10
f1 (ppm)

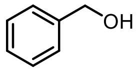
OH

14

^1H, ^1H COSY

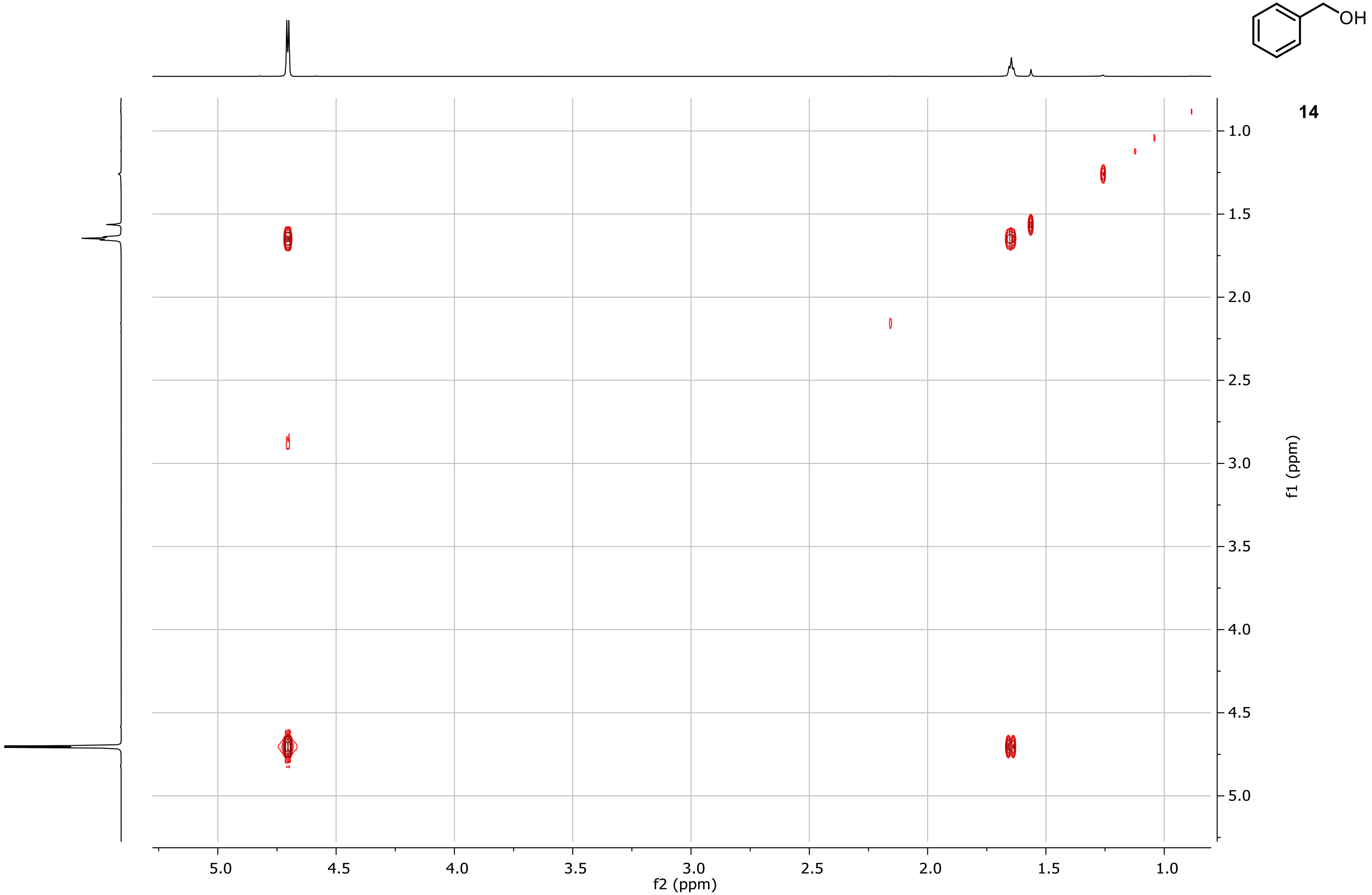

14

^{1}H, ^{13}C HMBC

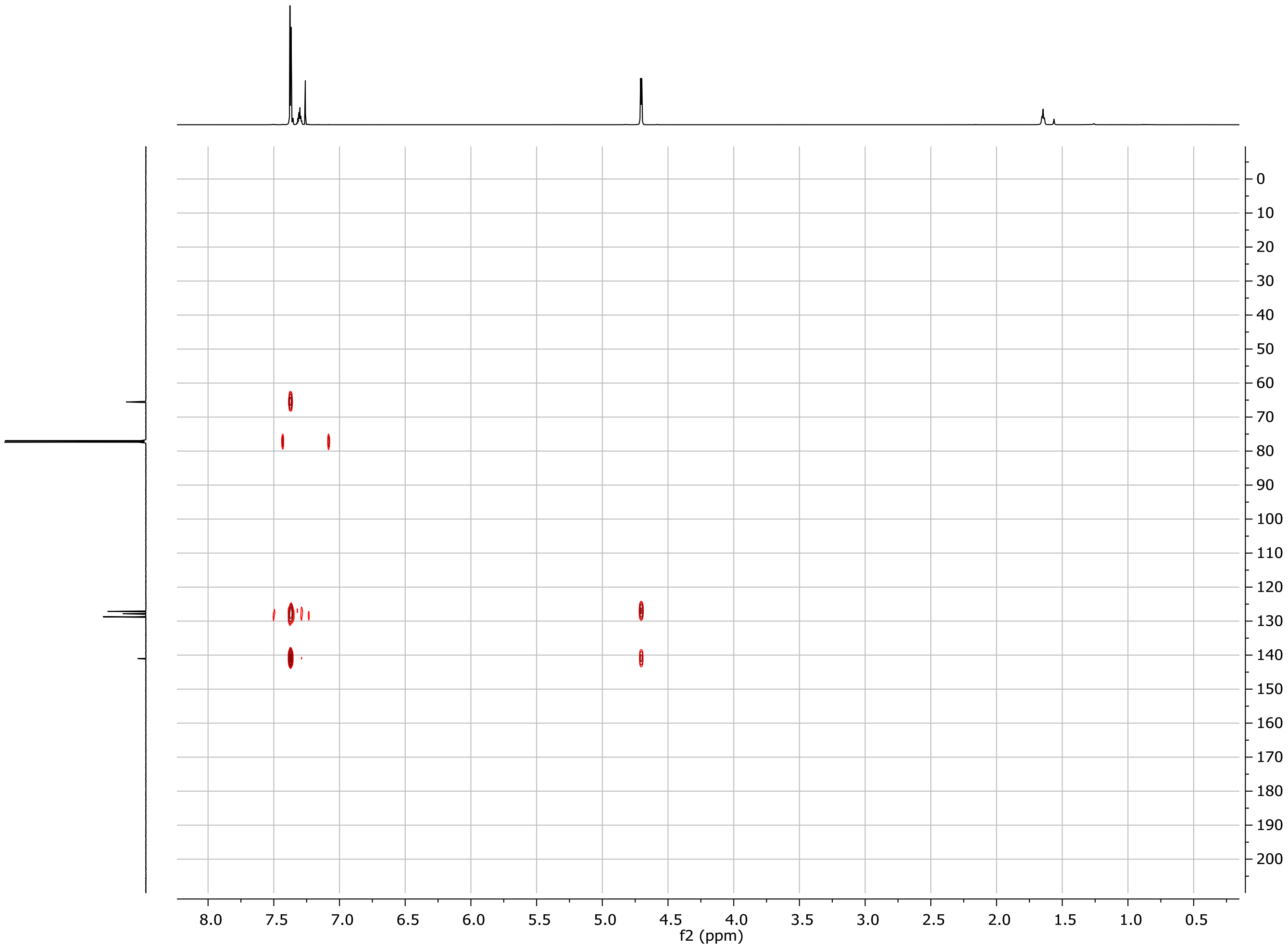

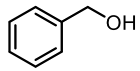

14

^{1}H, ^{13}C HSQC

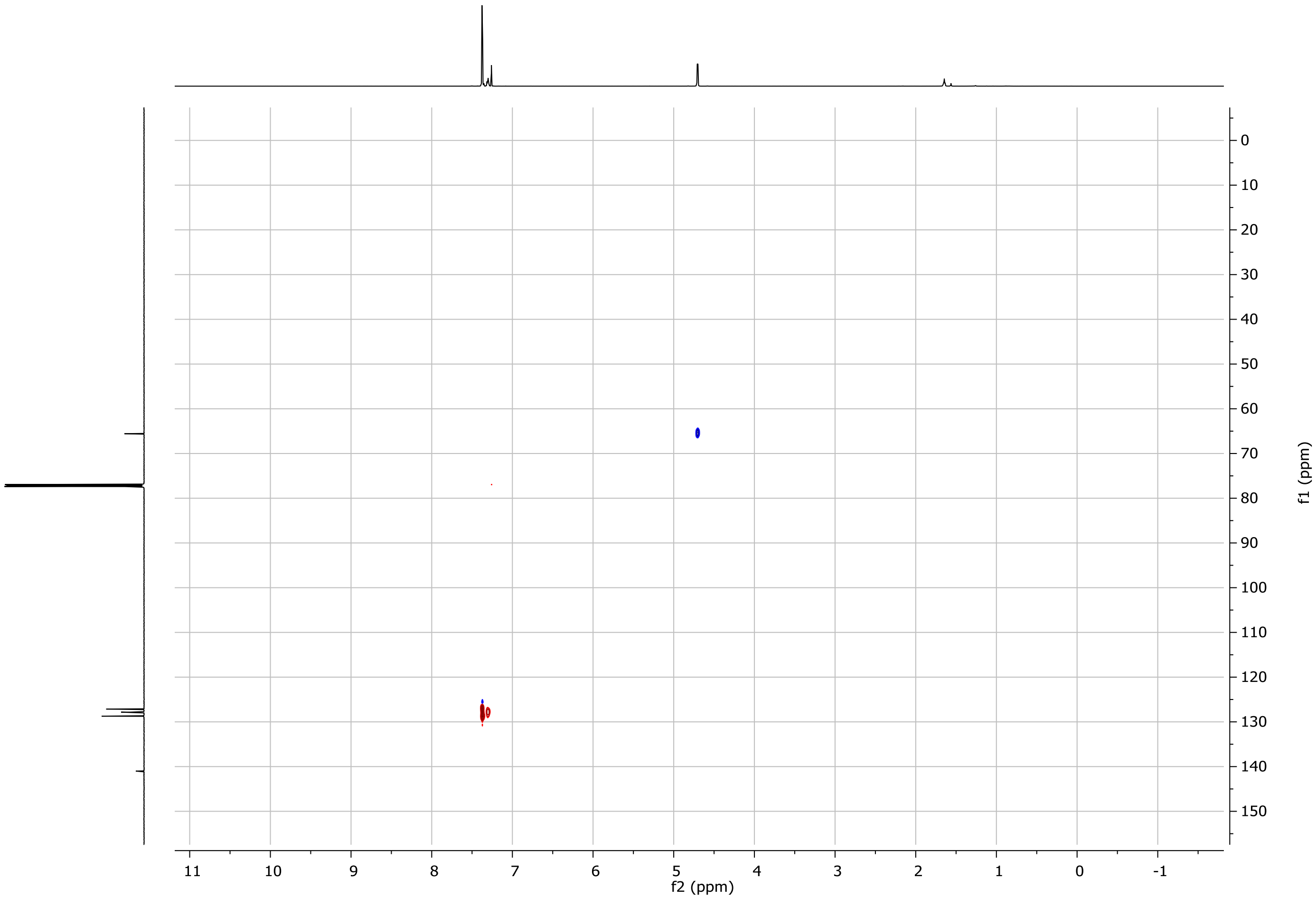

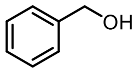

14

HRMS

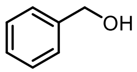

14

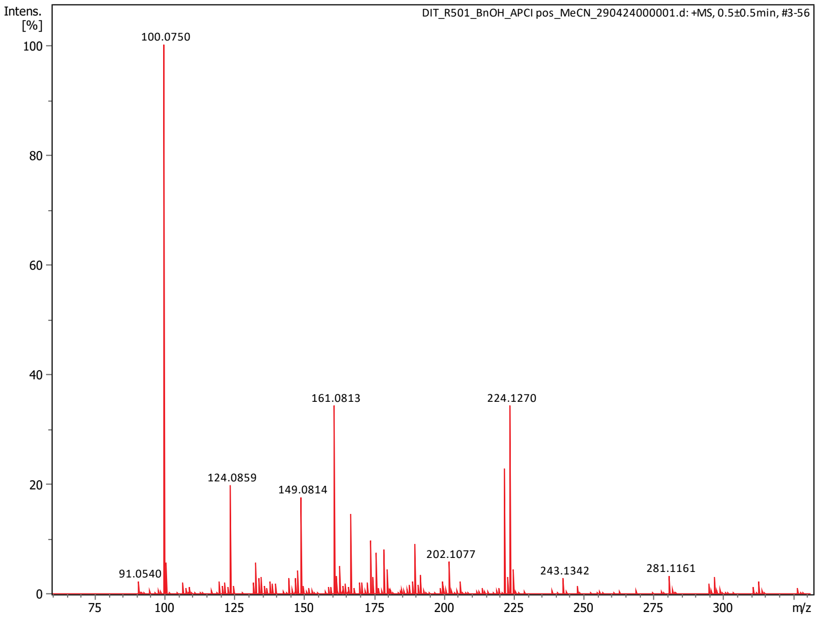
Intens. [%]
DIT_R501_BnOH_APCI pos_MeCN_290424000001.d: +MS, 0.5±0.5min, #3-56
100.0750
124.0859
149.0814
161.0813
224.1270
202.1077
243.1342
281.1161
91.0540
m/z

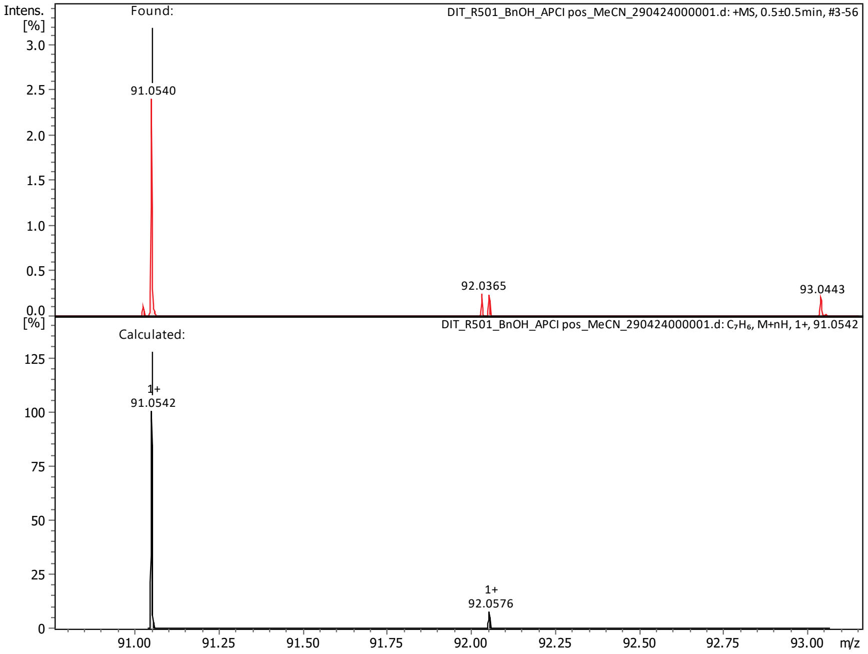
Intens. [%]
Found:
DIT_R501_BnOH_APCI pos_MeCN_290424000001.d: +MS, 0.5±0.5min, #3-56
91.0540
92.0365
93.0443
Calculated:
DIT_R501_BnOH_APCI pos_MeCN_290424000001.d: C7H6, M+nH, 1+, 91.0542
1+ 91.0542
1+ 92.0576
m/z

76 3-Piperazin-1-yl-1-(1-piperidyl)propan-1-one (15)

^{1}H NMR

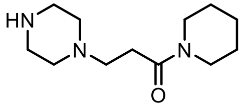

15

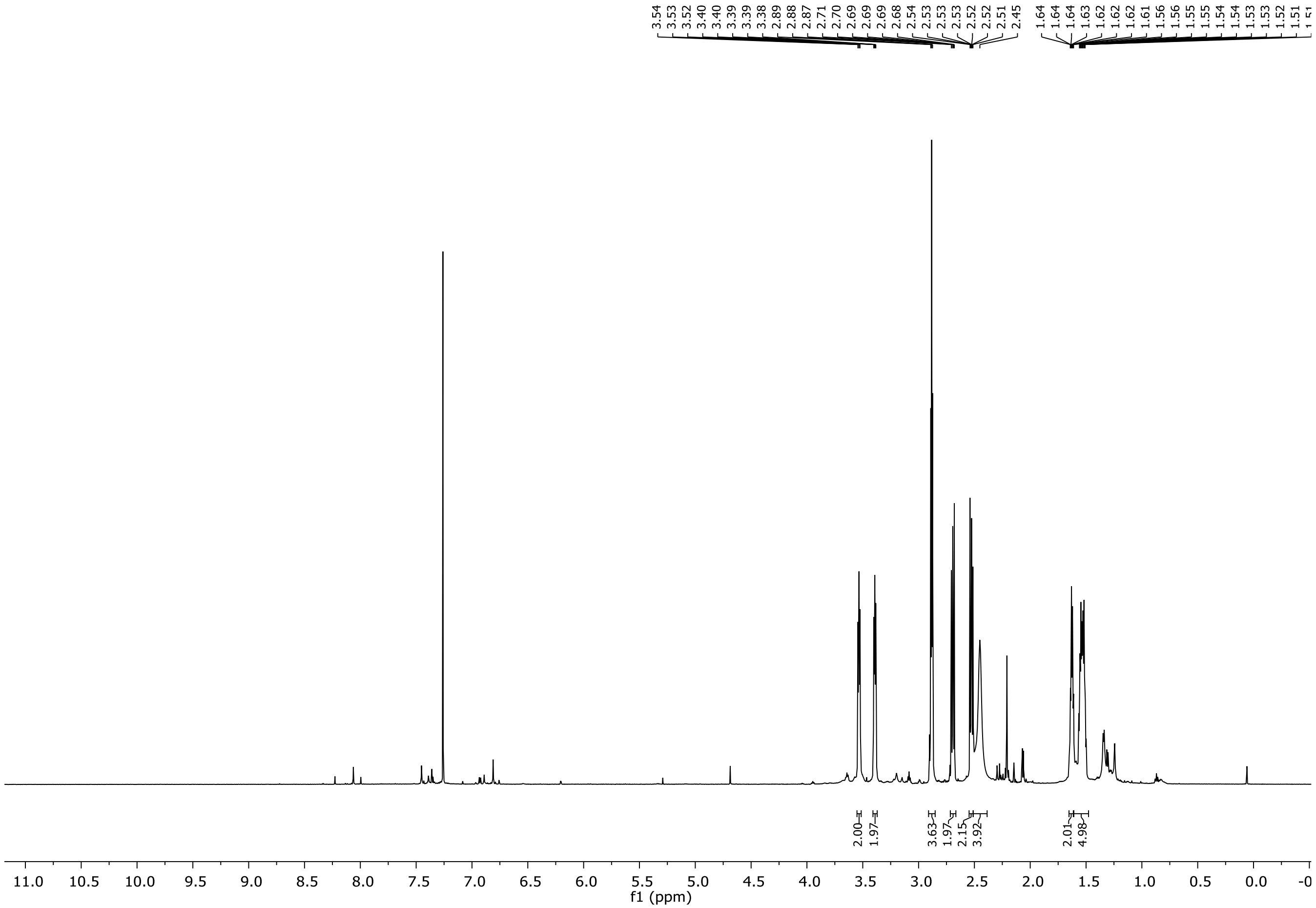

^{13}C NMR

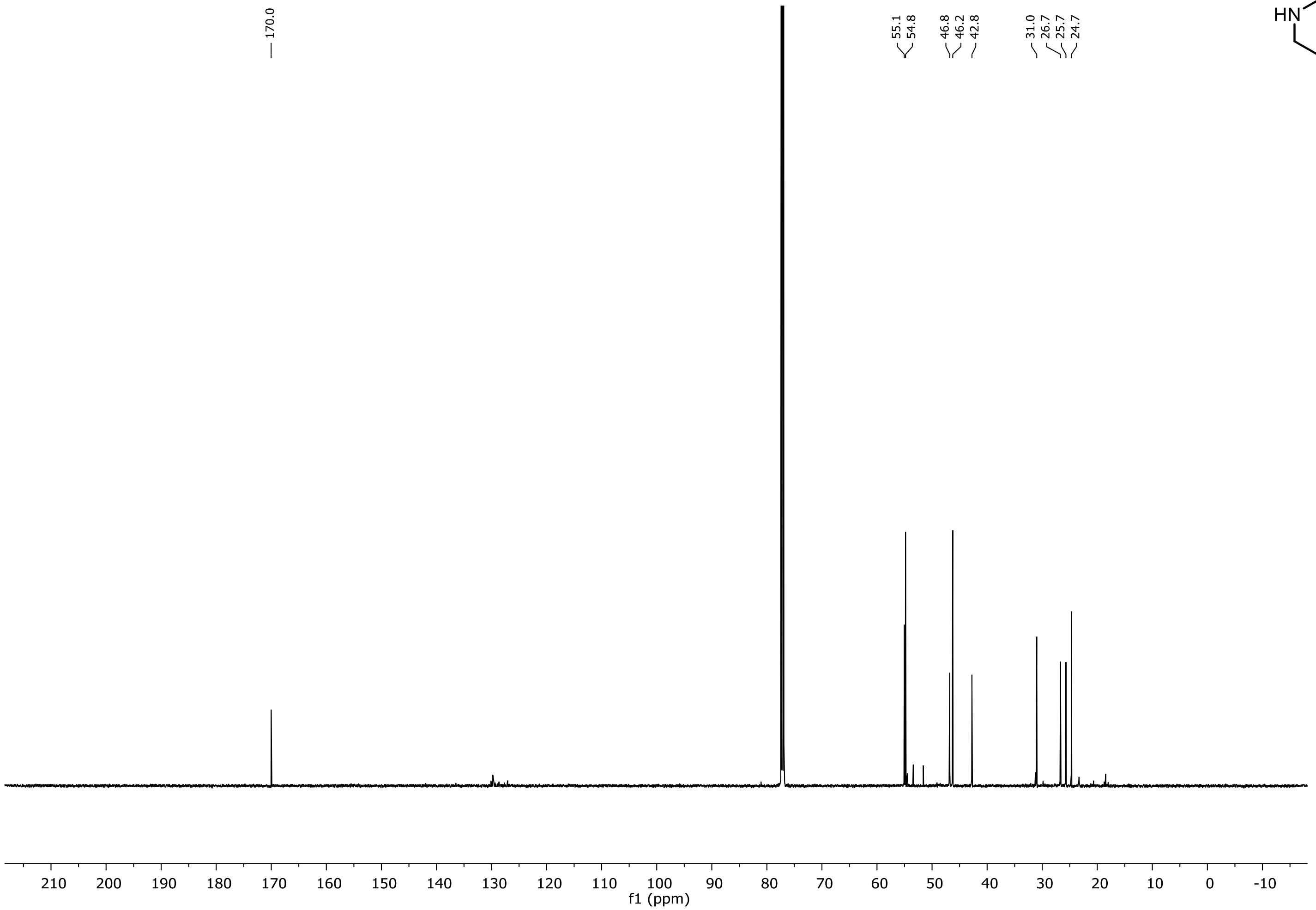
170.0
55.1
54.8
46.8
46.2
42.8
31.0
26.7
25.7
24.7
210 200 190 180 170 160 150 140 130 120 110 100 90 80 70 60 50 40 30 20 10 0 -10
f1 (ppm)

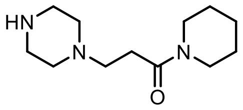
HN
N
N
O

15

^{1}H, ^{1}H COSY

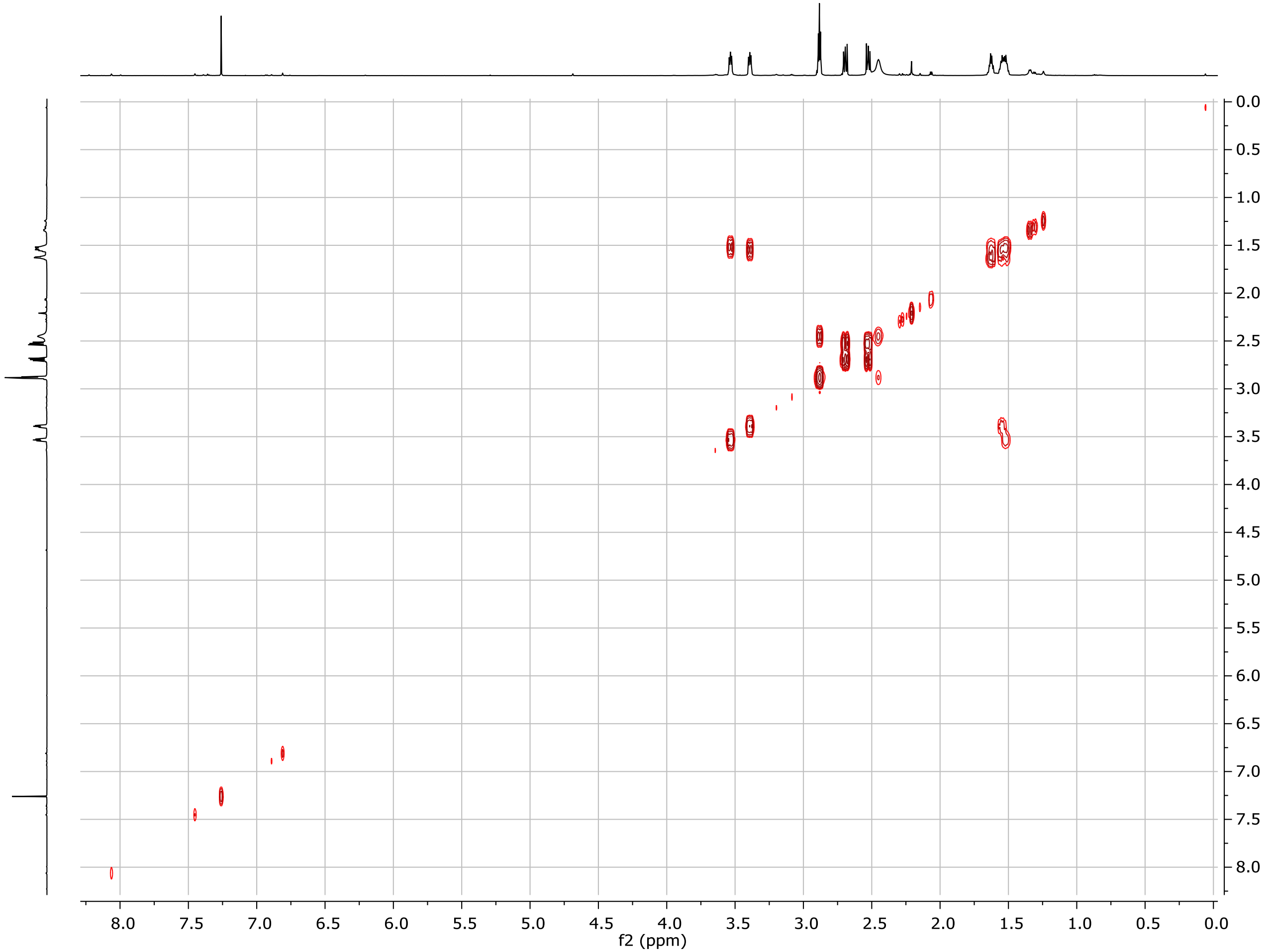

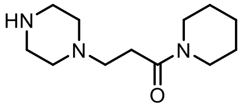

15

^{1}H, ^{13}C HMBC

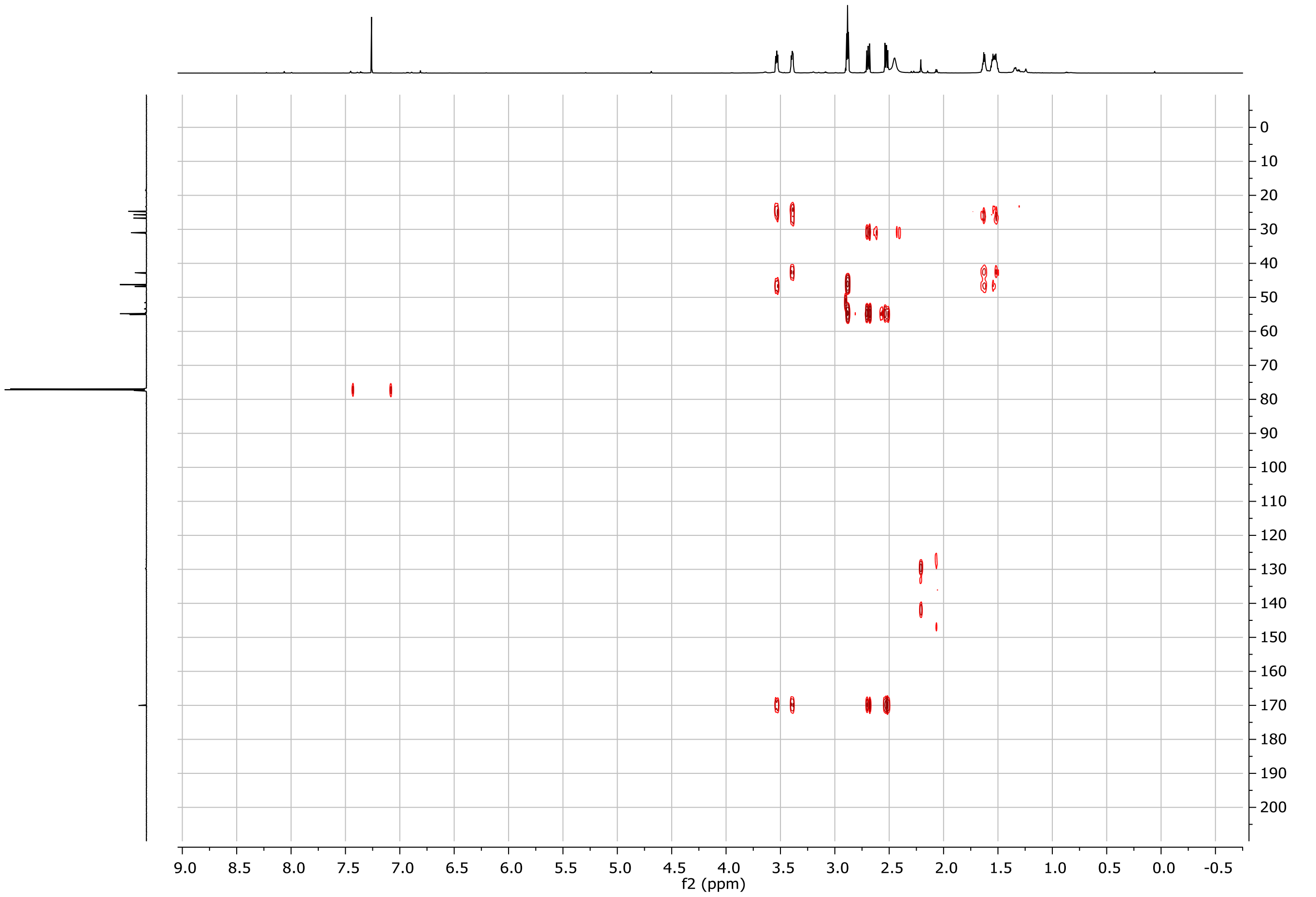

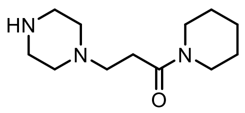

15

^{1}H, ^{13}C HSQC

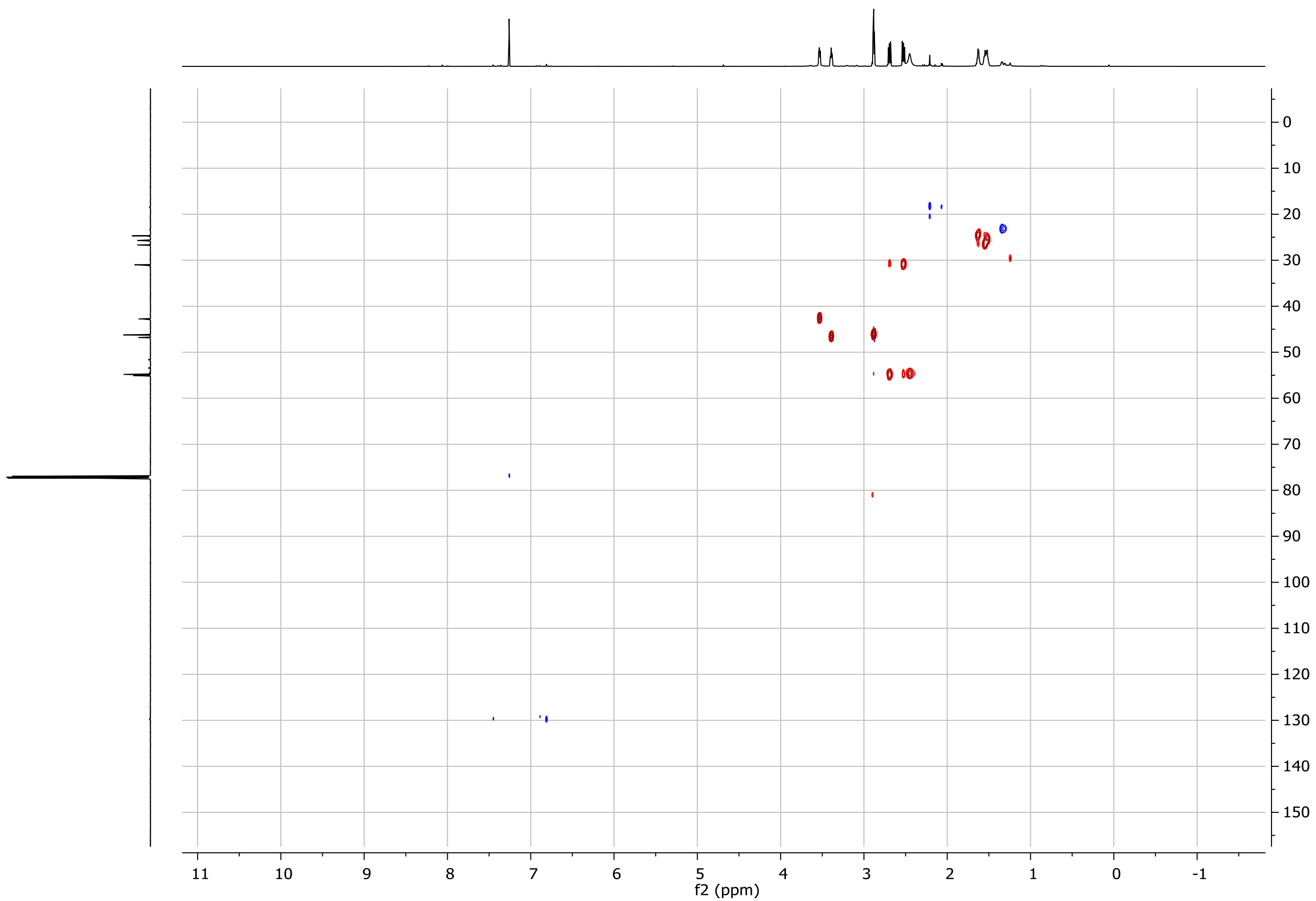

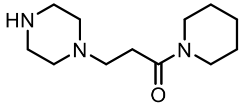

15

HRMS

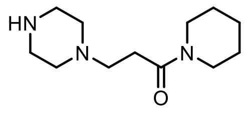

15

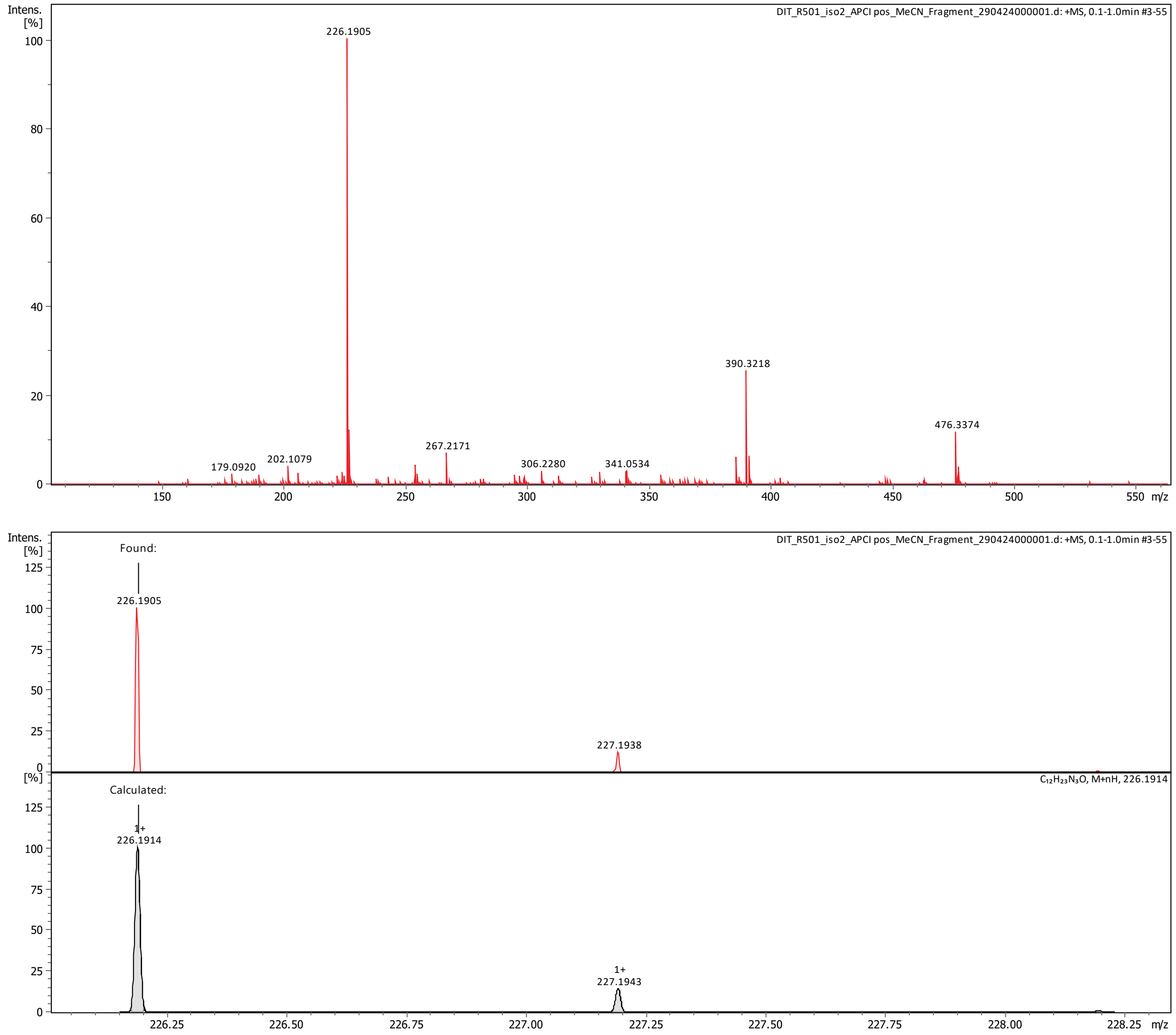

Intens. [%]
DIT_R501_iso2_APCI pos_MeCN_Fragment_290424000001.d: +MS, 0.1-1.0min #3-55
226.1905
390.3218
476.3374
267.2171
179.0920
202.1079
306.2280
341.0534
m/z
Found:
226.1905
227.1938
Calculated:
$C_{12}H_{23}N_3O$, M+nH, 226.1914
1+
226.1914
1+
227.1943

IR

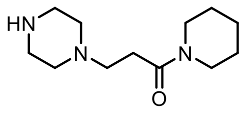

15

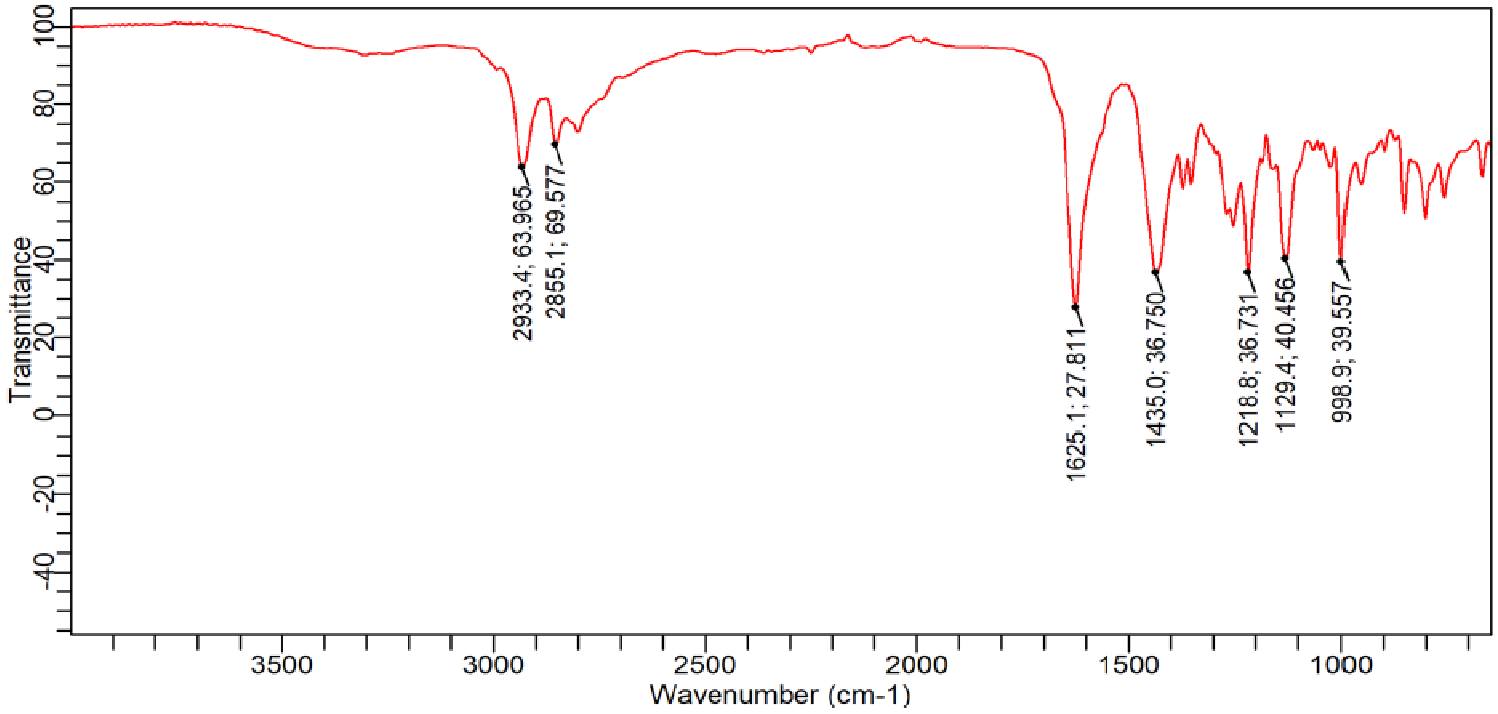
Transmittance
Wavenumber (cm-1)
2933.4; 63.965
2855.1; 69.577
1625.1; 27.811
1435.0; 36.750
1218.8; 36.731
1129.4; 40.456
998.9; 39.557

77 *N,N*-diethyl-4-(hydroxymethyl)benzamide (17a)

^{1}H NMR

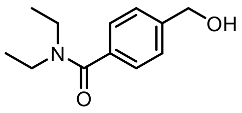

17a

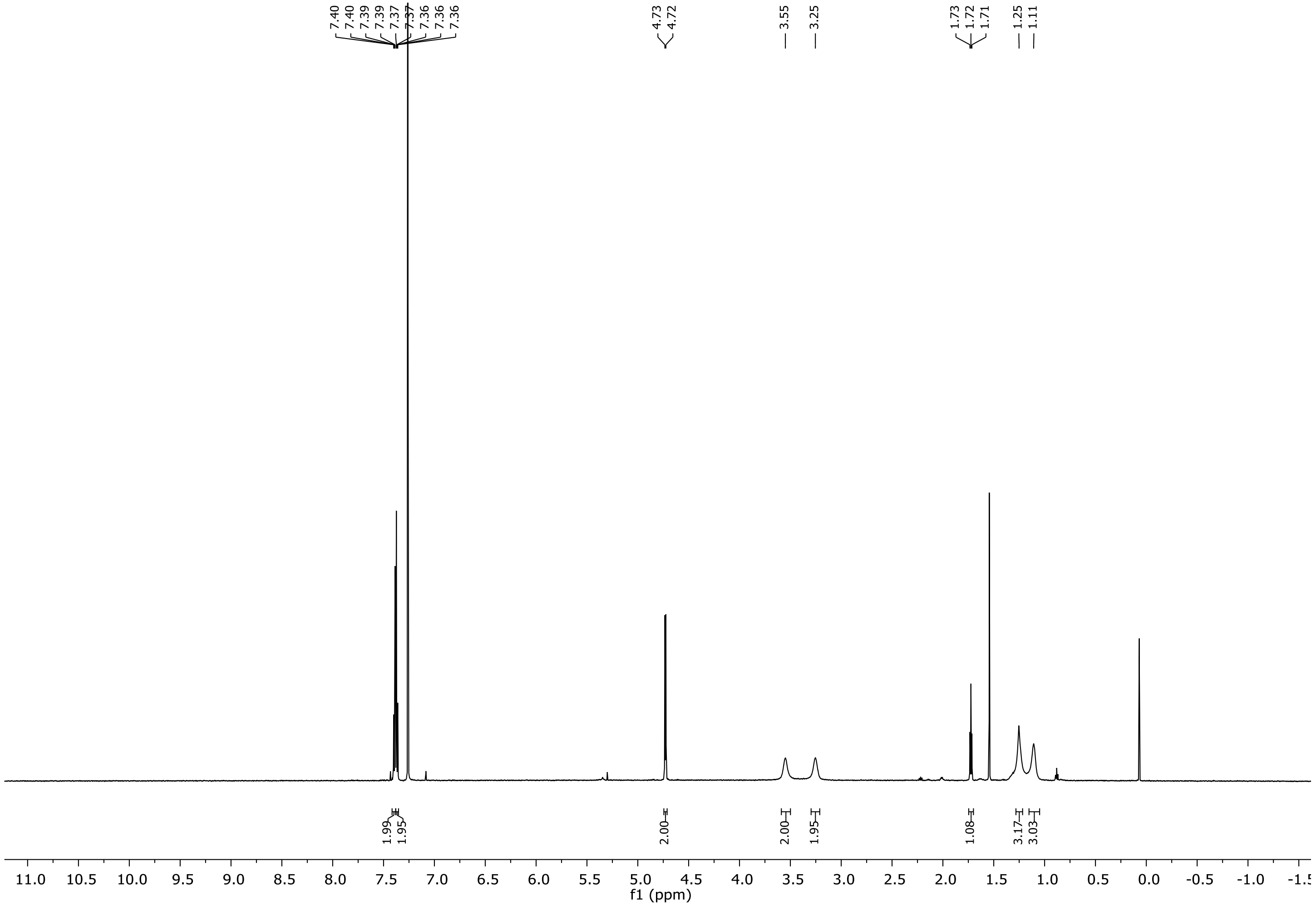

^{13}C NMR

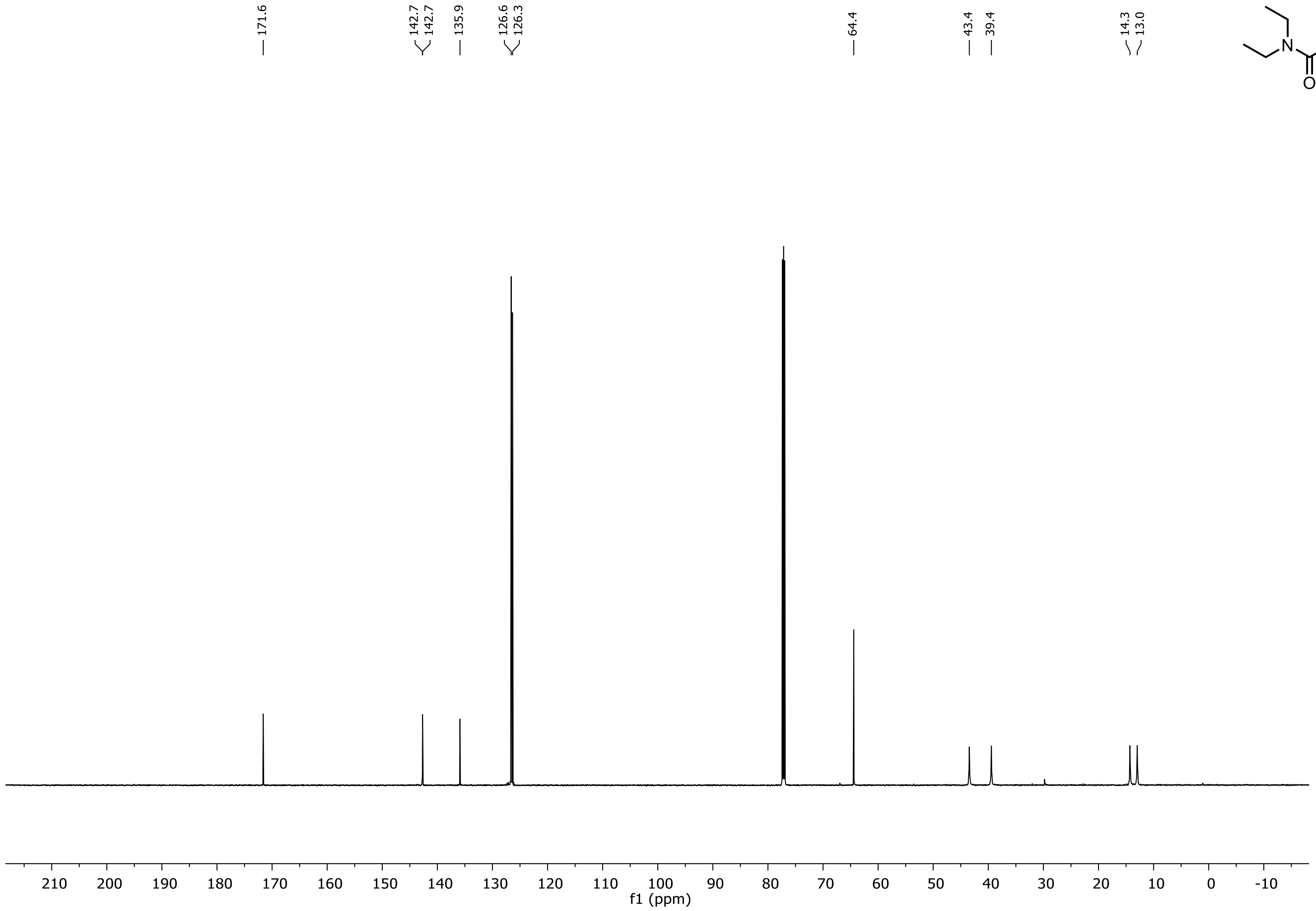
171.6
142.7
142.7
135.9
126.6
126.3
64.4
43.4
39.4
14.3
13.0
210 200 190 180 170 160 150 140 130 120 110 100 90 80 70 60 50 40 30 20 10 0 -10
f1 (ppm)

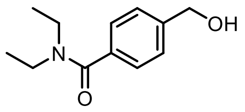
N
O
OH

17a

^{1}H, ^{1}H COSY

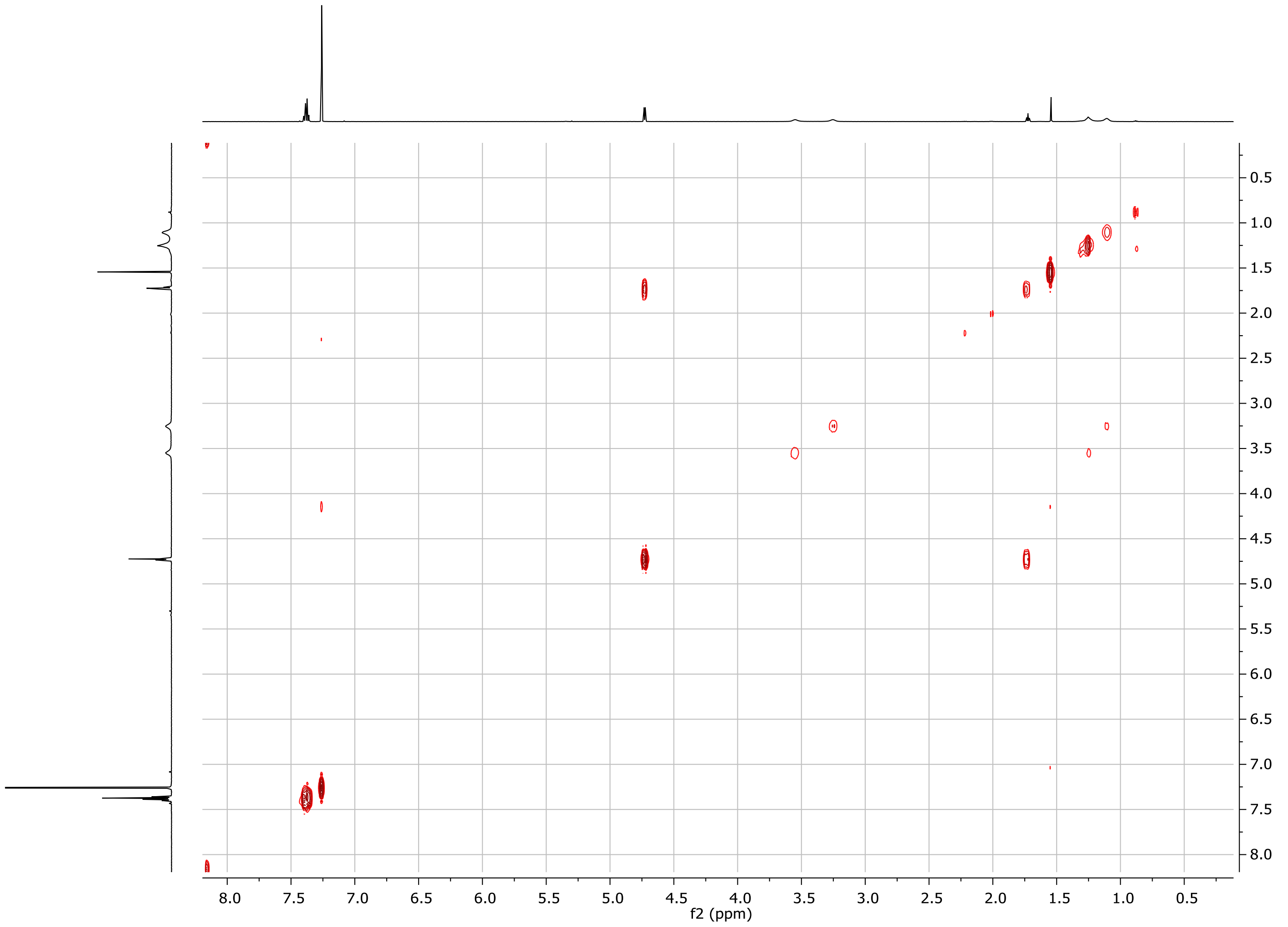

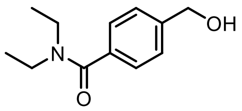

17a

^1H, ^{13}C HMBC

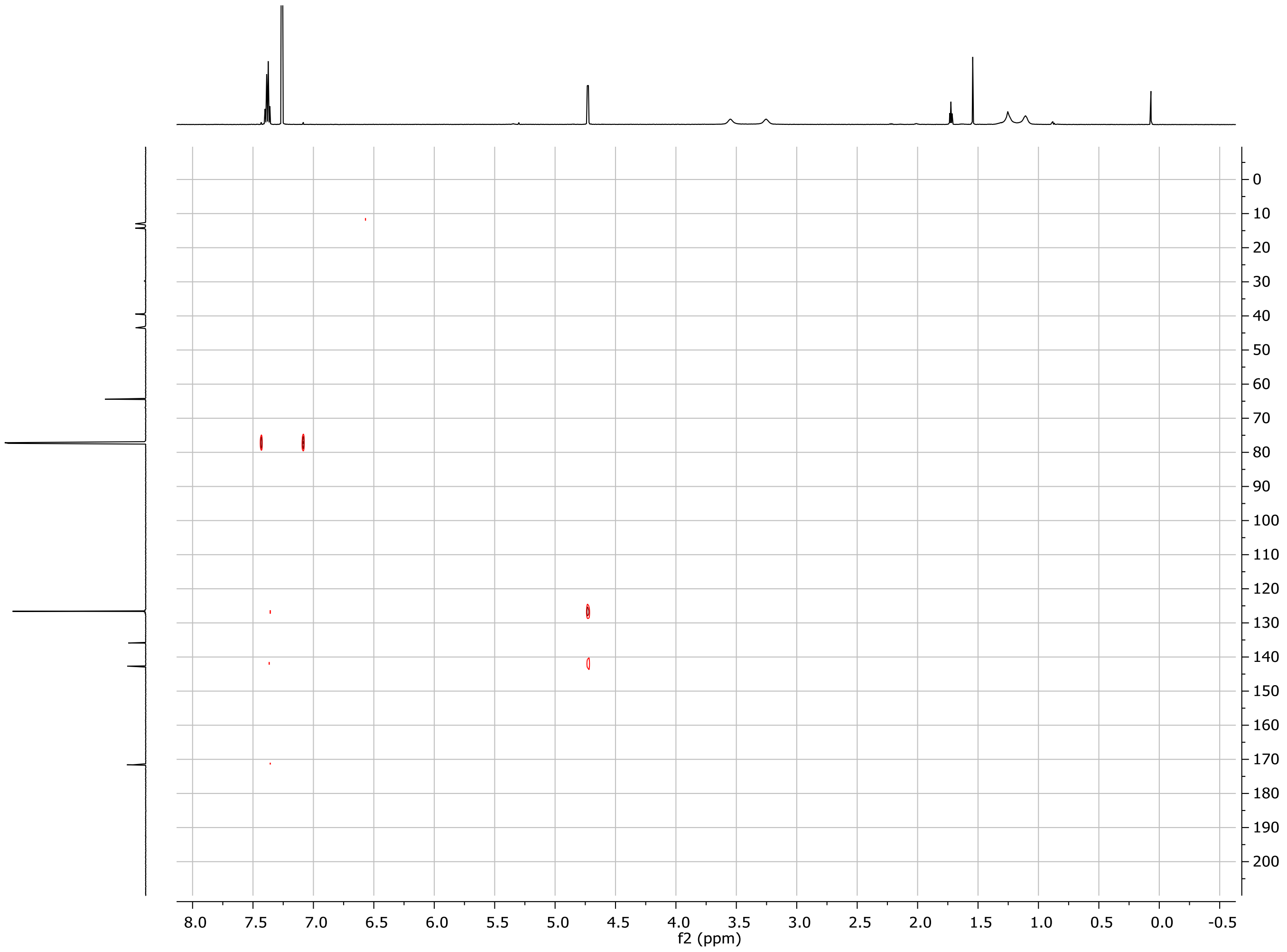

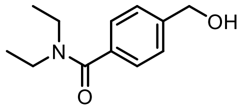

17a

^{1}H, ^{13}C HSQC

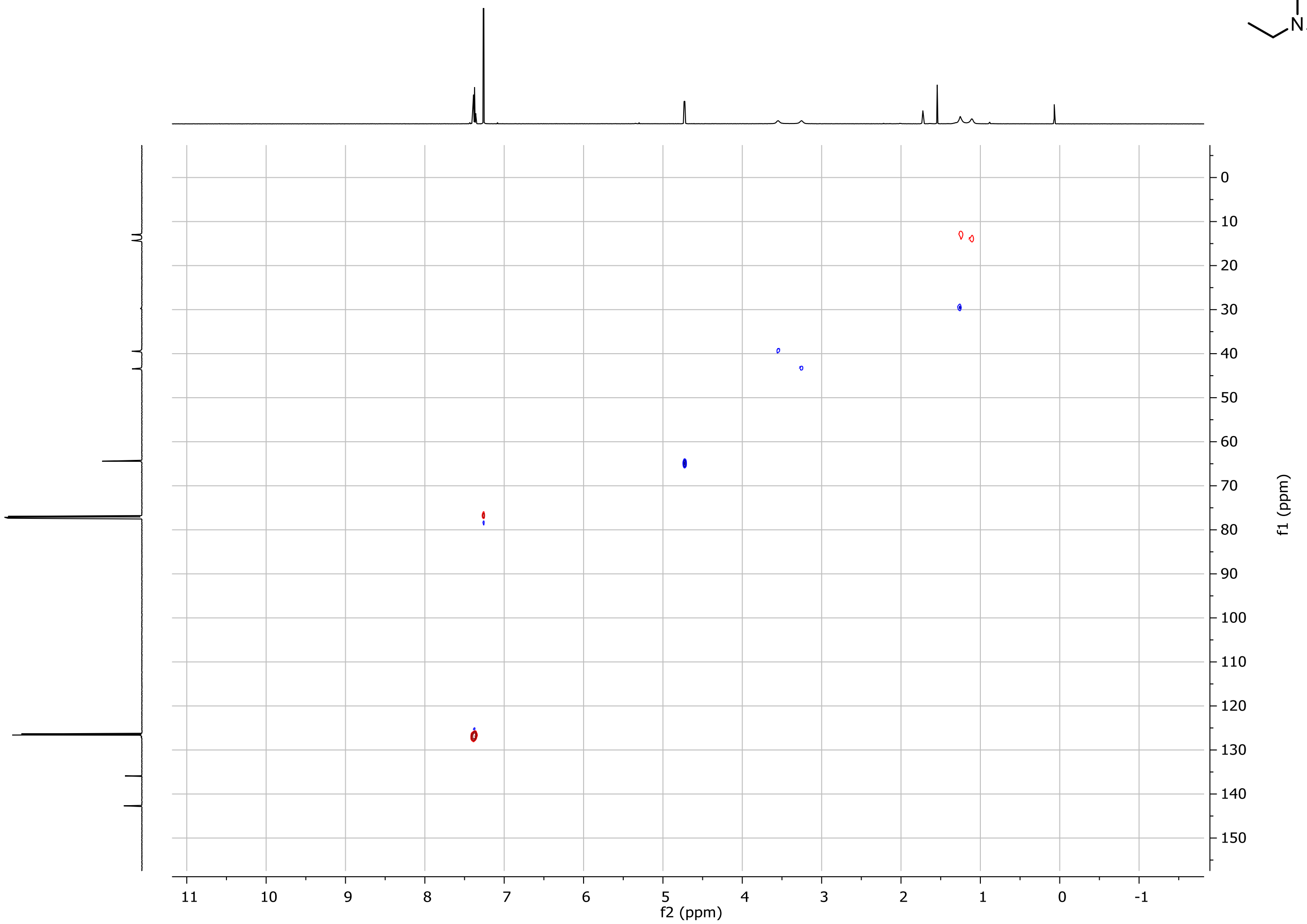

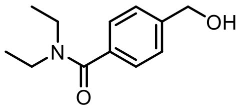

17a

HRMS

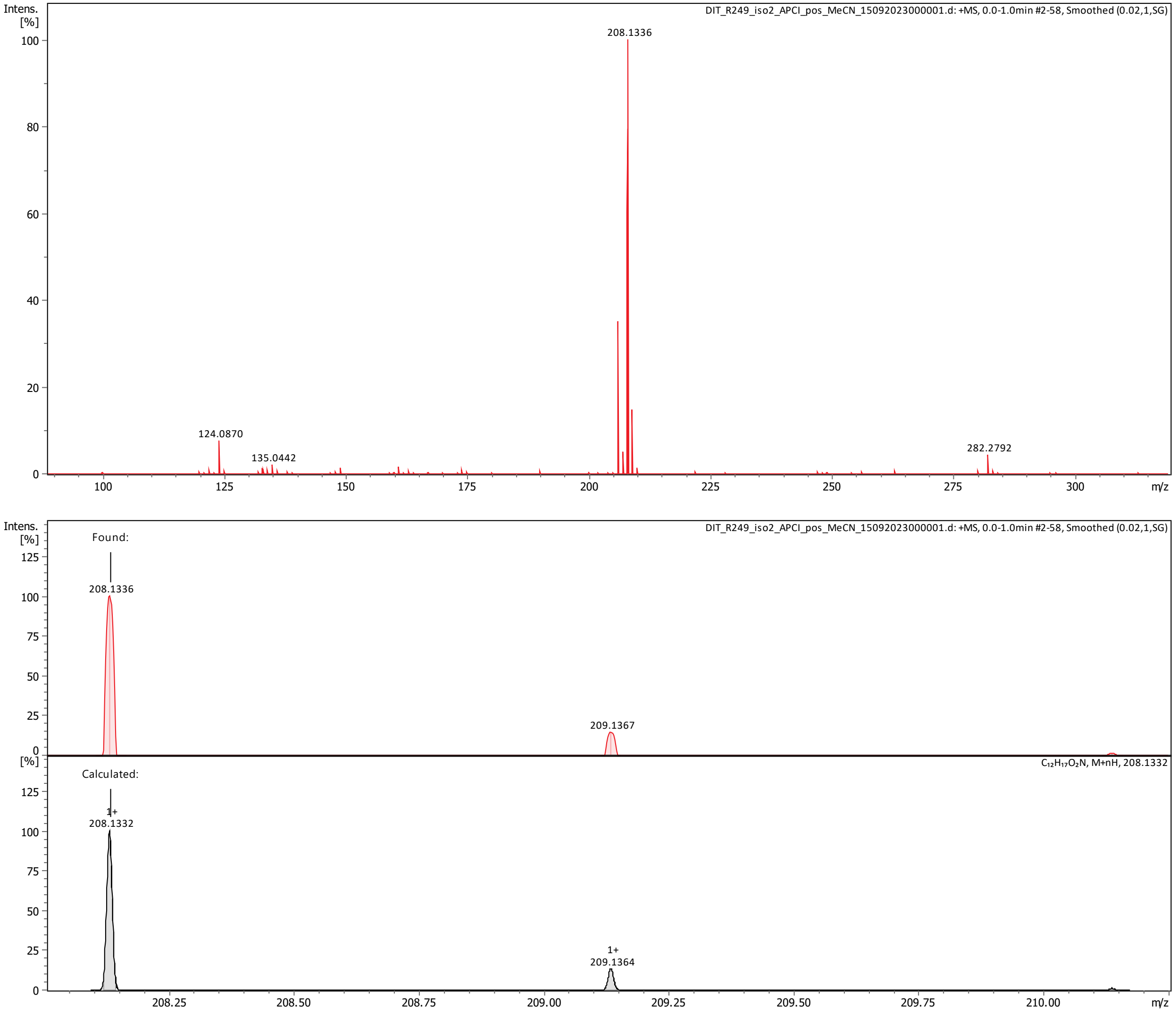

DIT_R249_iso2_APCI_pos_MeCN_15092023000001.d: +MS, 0.0-1.0min #2-58, Smoothed (0.02,1,SG)
Intens. [%]
208.1336
124.0870
135.0442
282.2792
m/z
Found:
208.1336
209.1367
Calculated:
$C_{12}H_{17}O_2N$, M+nH, 208.1332
1+
208.1332
1+
209.1364

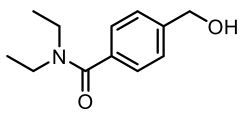

N
O
OH

17a

78 4-(Hydroxymethyl)-*N*-(2-methoxyethyl)-*N*-methyl-benzamide (17b)

^{1}H NMR

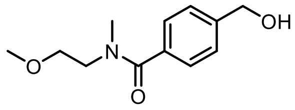

17b

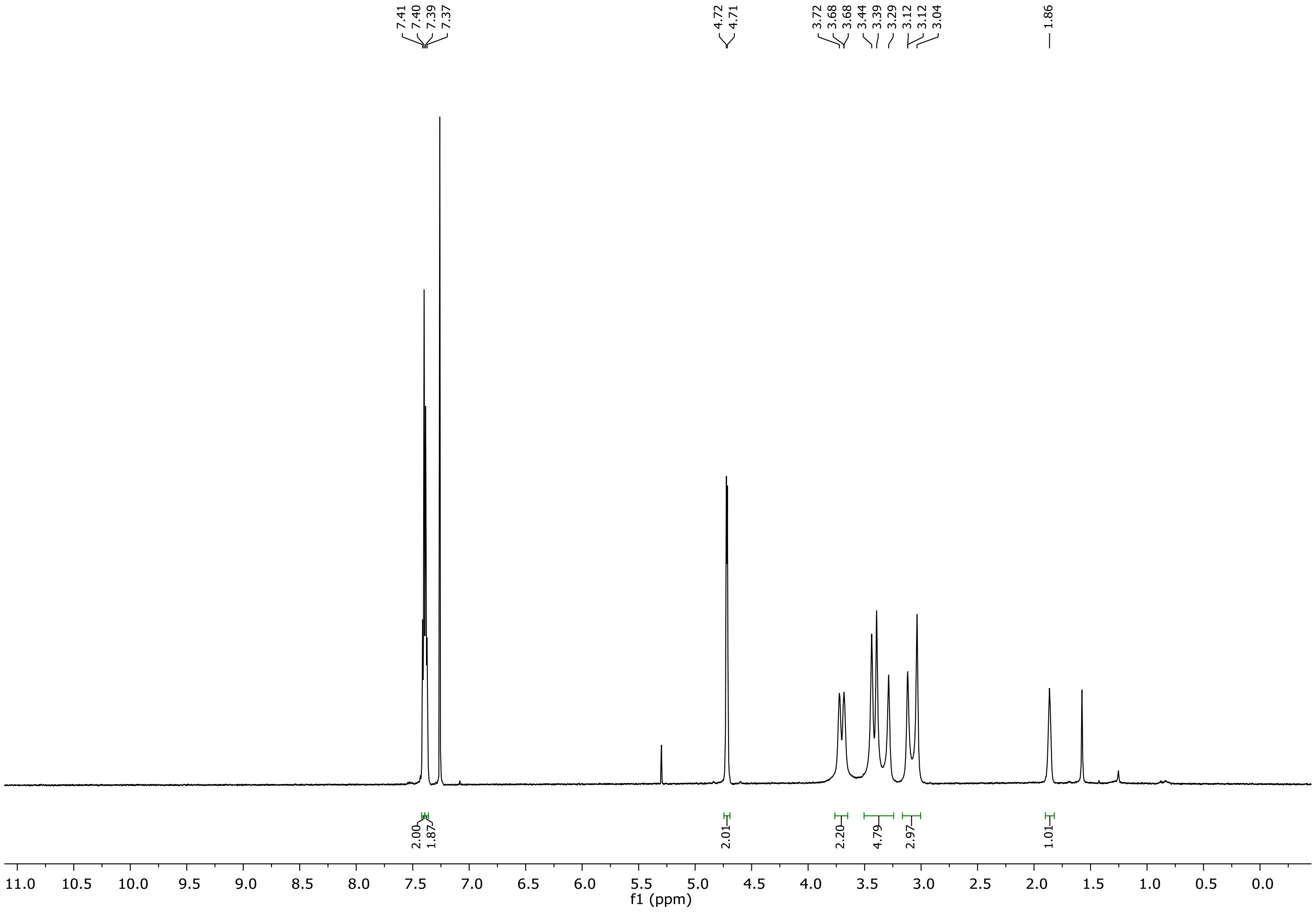

^{13}C NMR

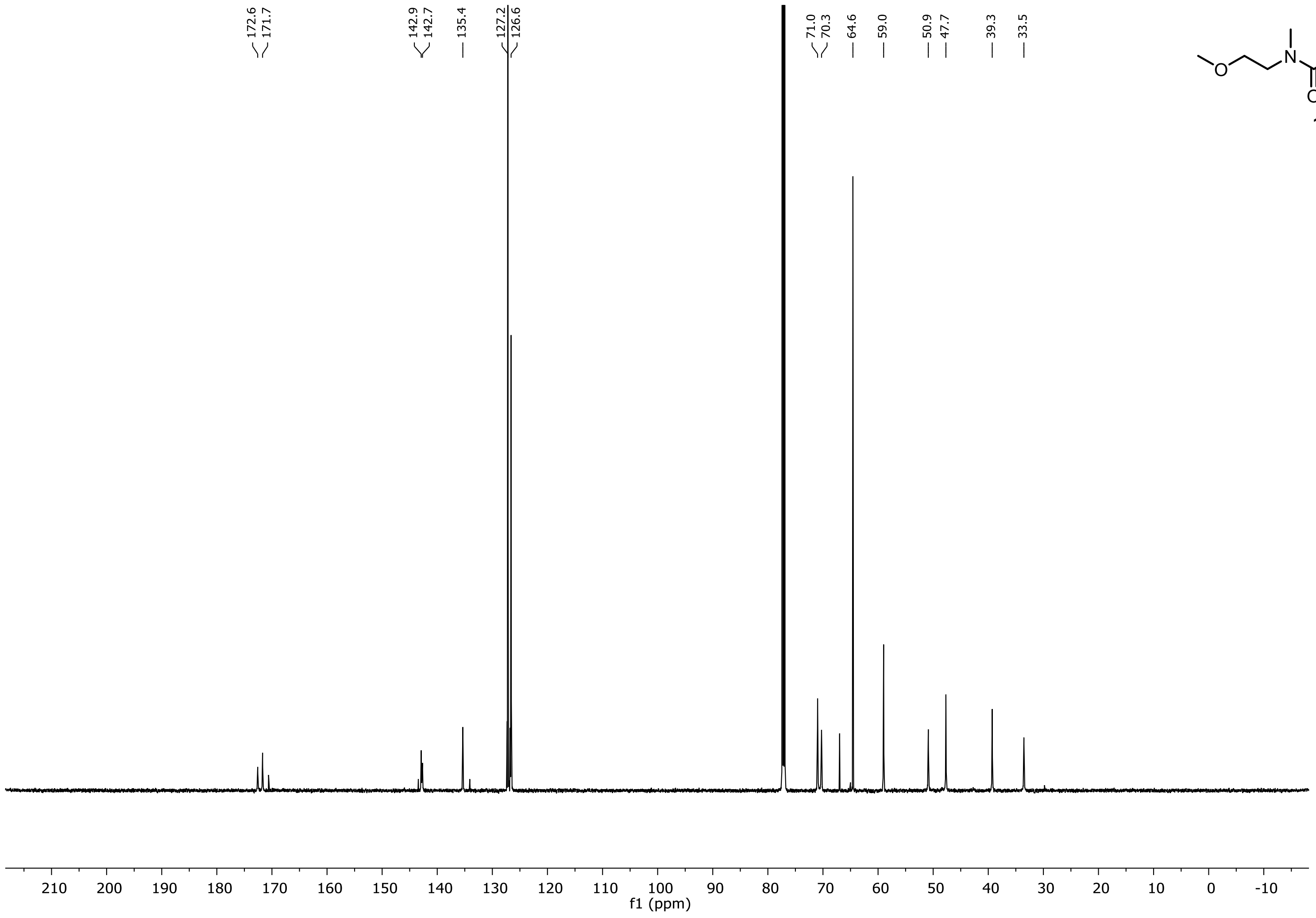

172.6
171.7
142.9
142.7
135.4
127.2
126.6
71.0
70.3
64.6
59.0
50.9
47.7
39.3
33.5
210 200 190 180 170 160 150 140 130 120 110 100 90 80 70 60 50 40 30 20 10 0 -10
f1 (ppm)

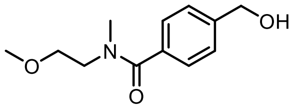

O
N
O
OH

17b

^{1}H, ^{1}H COSY

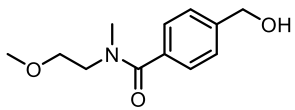

17b

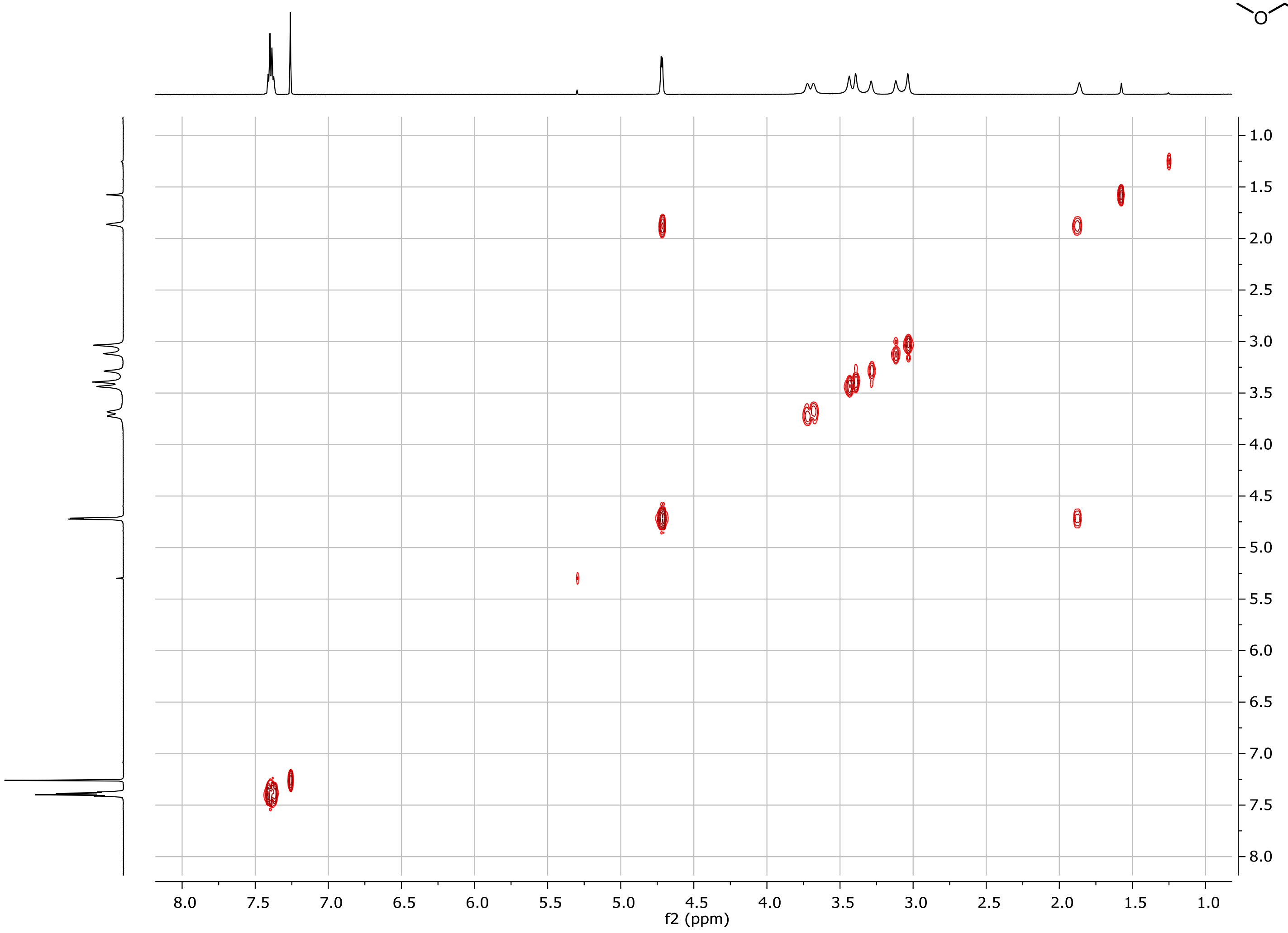

^{1}H, ^{13}C HMBC

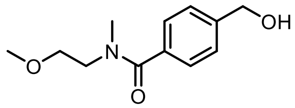

17b

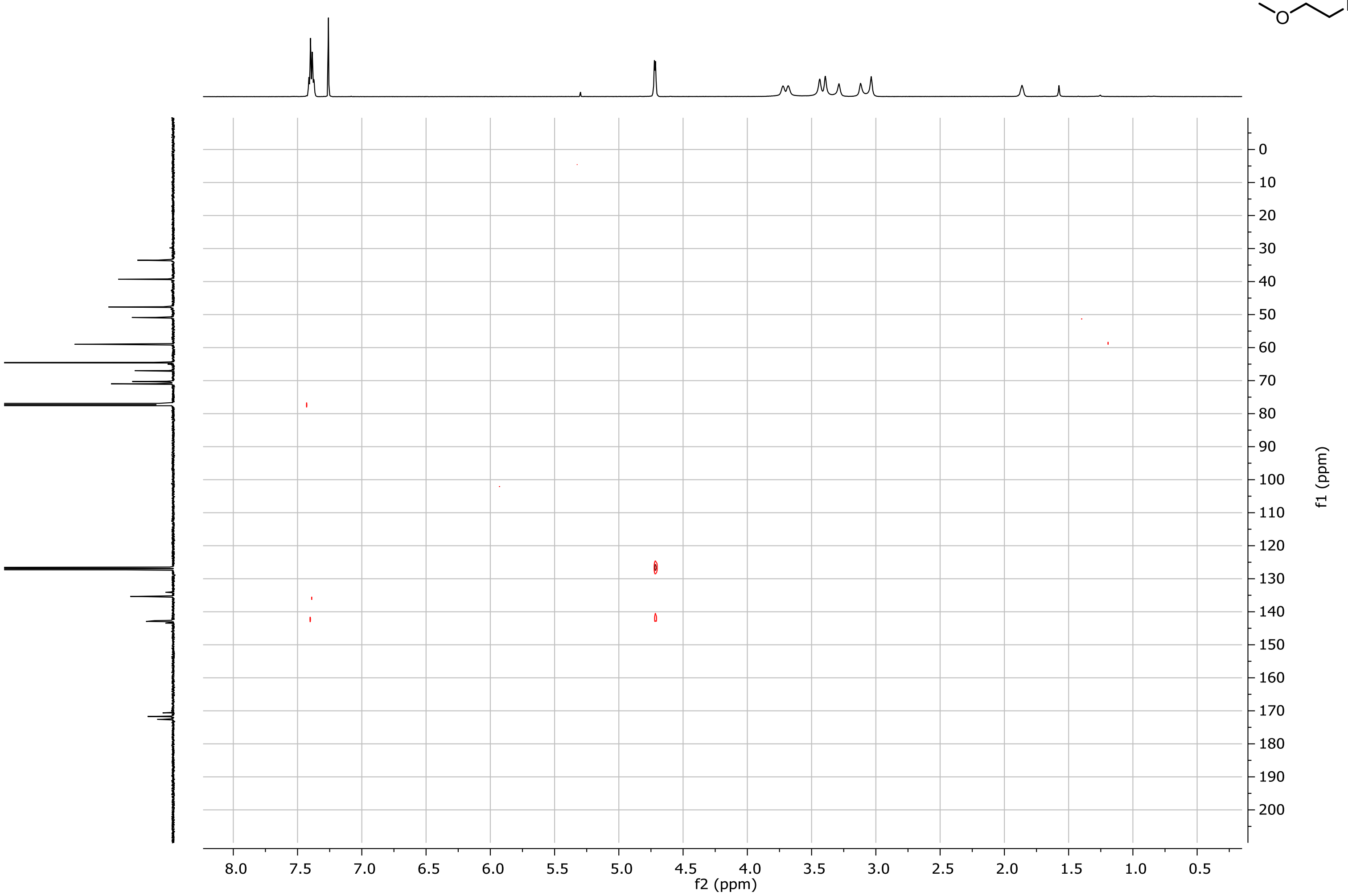

^{1}H, ^{13}C HSQC

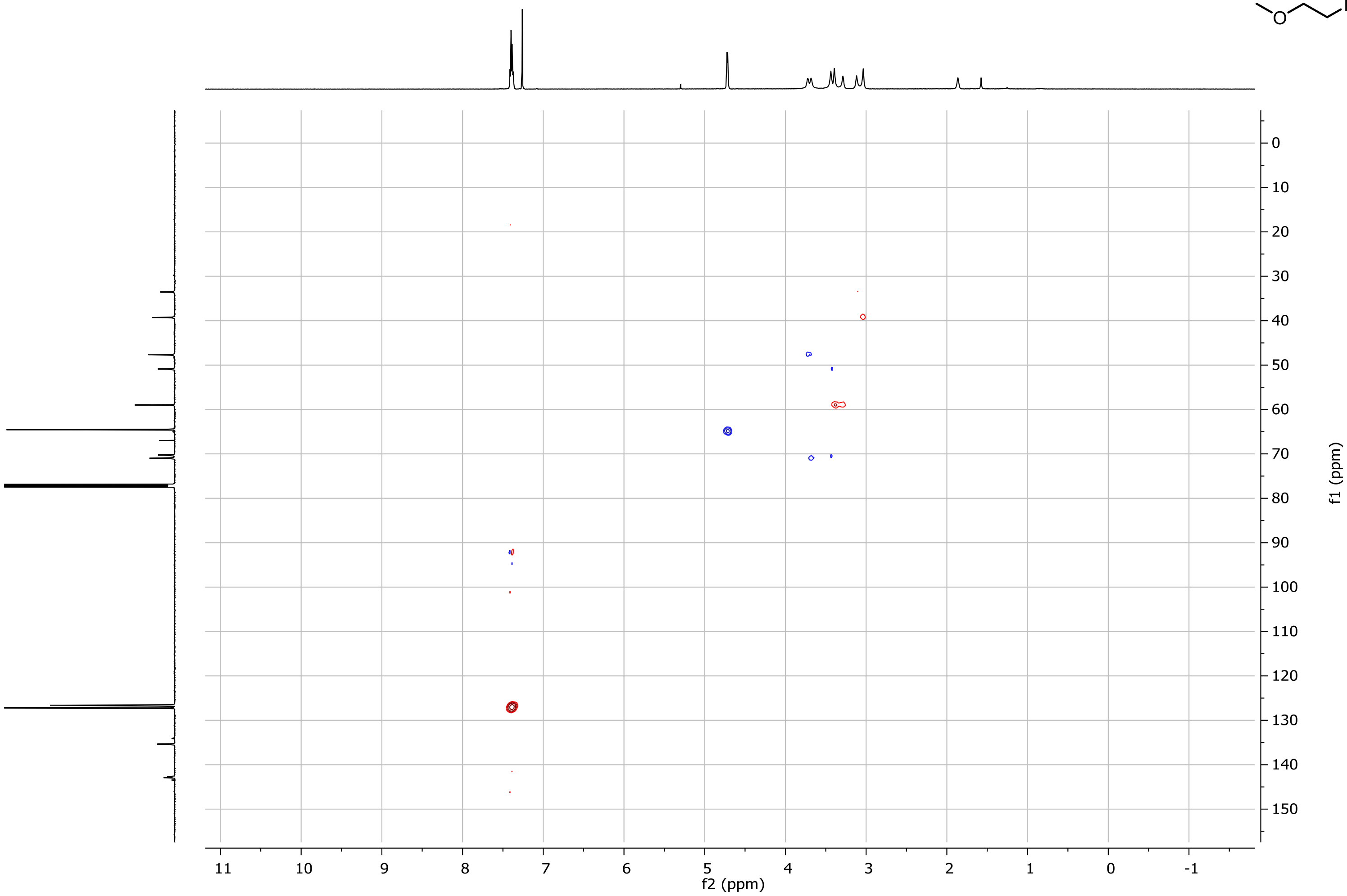

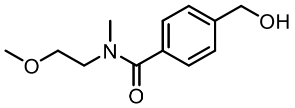

17b

HRMS

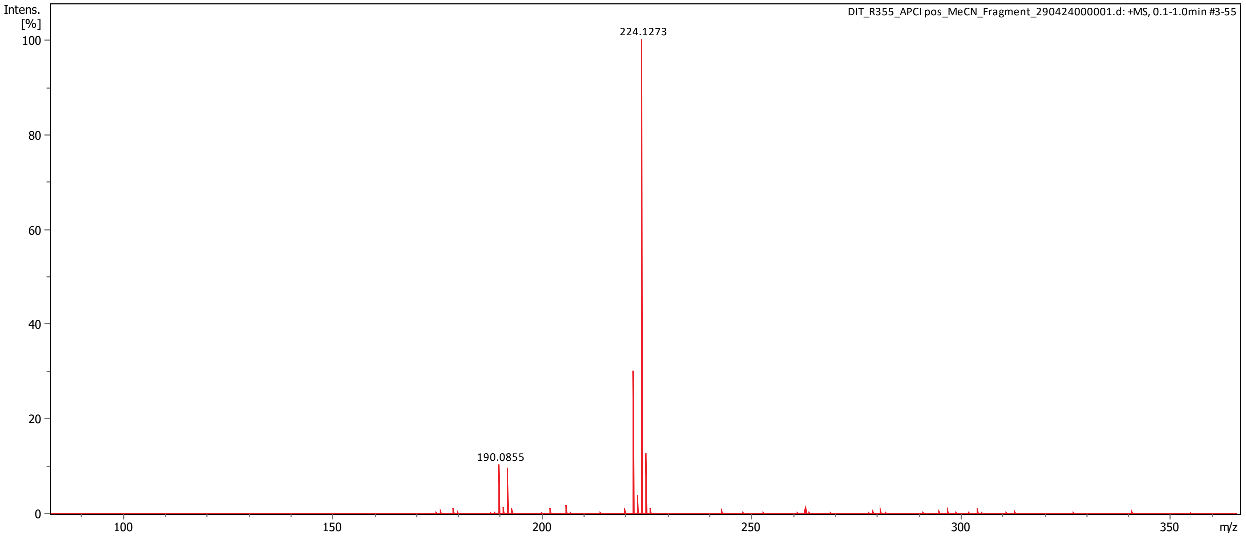

Intens. [%]
DIT_R355_APCI pos_MeCN_Fragment_290424000001.d: +MS, 0.1-1.0min #3-55
224.1273
190.0855
m/z

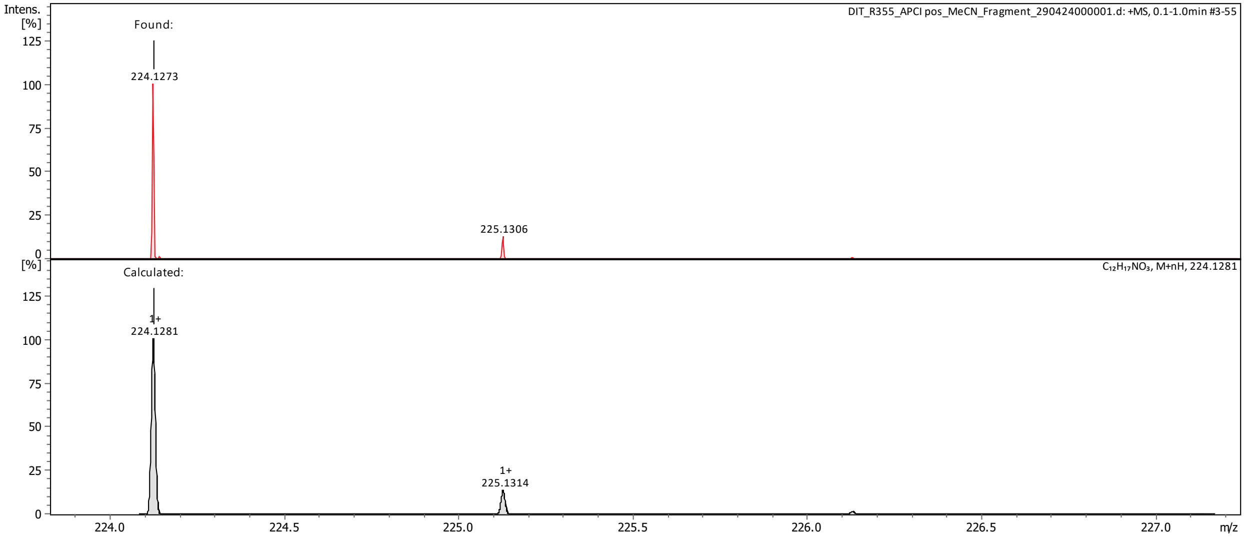

Intens. [%]
Found:
224.1273
225.1306
DIT_R355_APCI pos_MeCN_Fragment_290424000001.d: +MS, 0.1-1.0min #3-55
Calculated:
1+ 224.1281
1+ 225.1314
C12H17NO3, M+nH, 224.1281
m/z

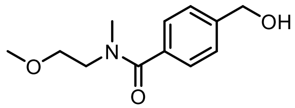

O
N
O
OH

17b

IR

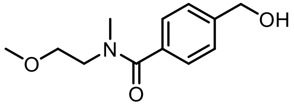

17b

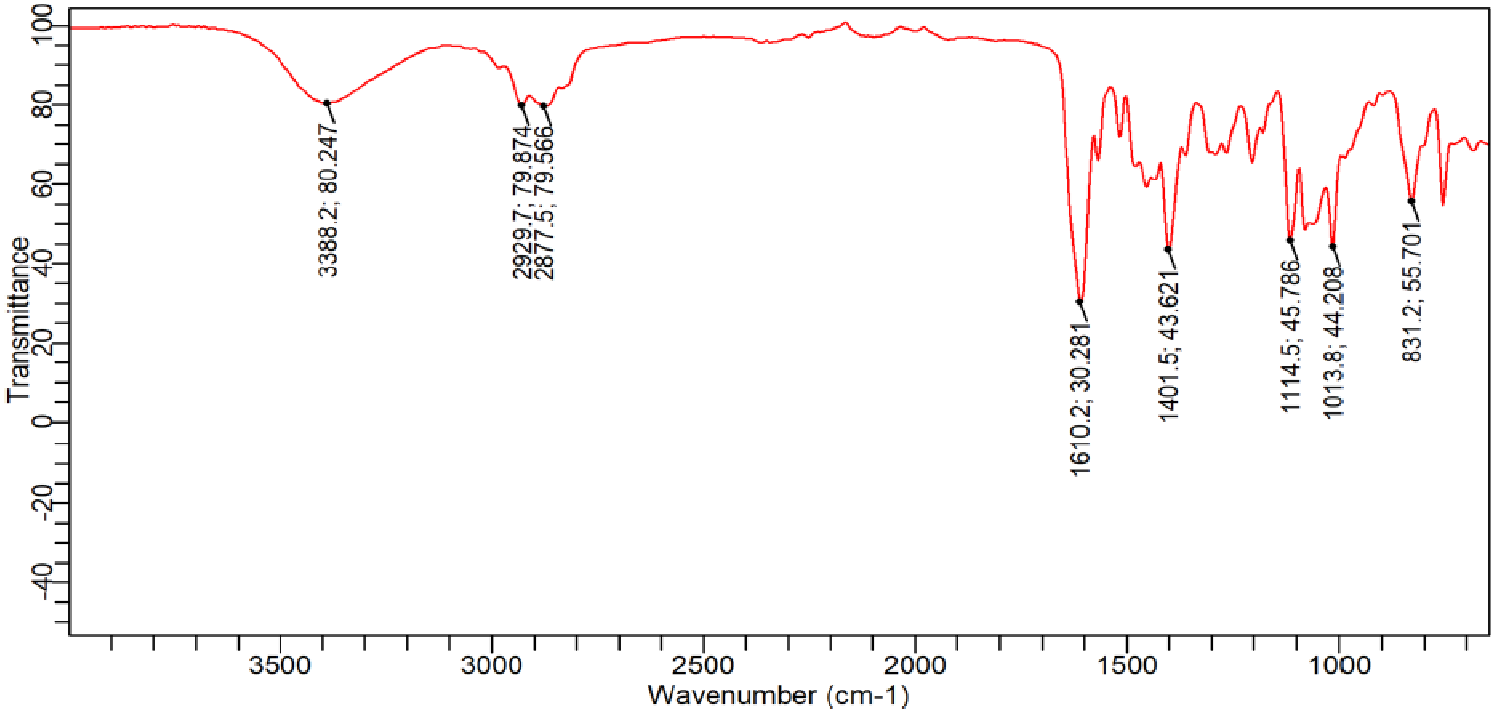

Transmittance
Wavenumber (cm-1)
3388.2; 80.247
2929.7; 79.874
2877.5; 79.566
1610.2; 30.281
1401.5; 43.621
1114.5; 45.786
1013.8; 44.208
831.2; 55.701

79 (4-(Hydroxymethyl)phenyl)(piperidin-1-yl)methanone (17c)

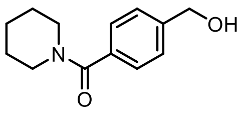

17c

^{1}H NMR

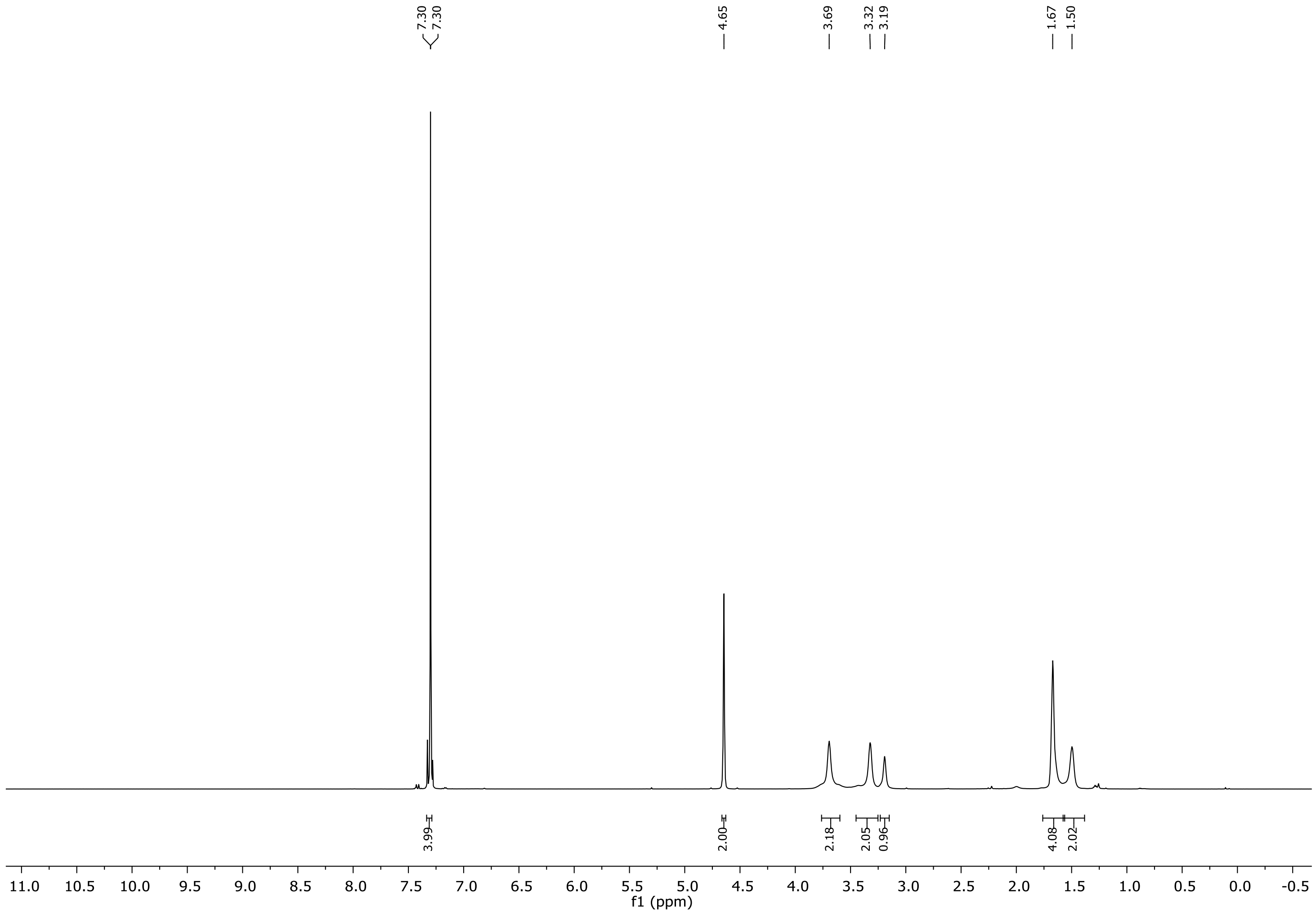

^{13}C NMR

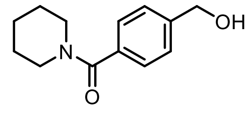
OH
N
O

17c

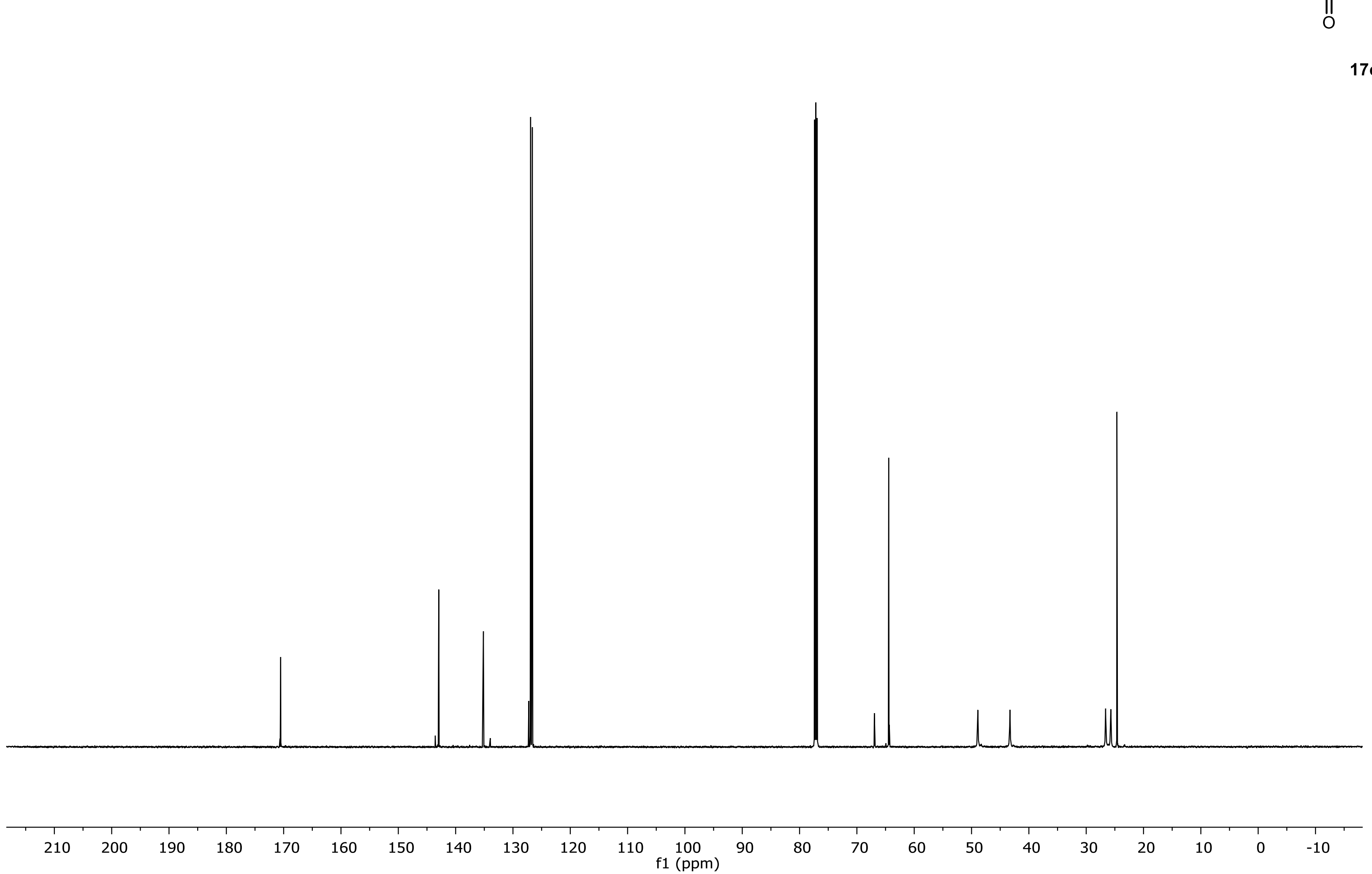
170.5
142.9
135.2
126.9
126.6
64.5
48.9
43.3
26.6
25.7
24.6
210 200 190 180 170 160 150 140 130 120 110 100 90 80 70 60 50 40 30 20 10 0 -10
f1 (ppm)

^{1}H, ^{1}H COSY

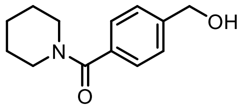

17c

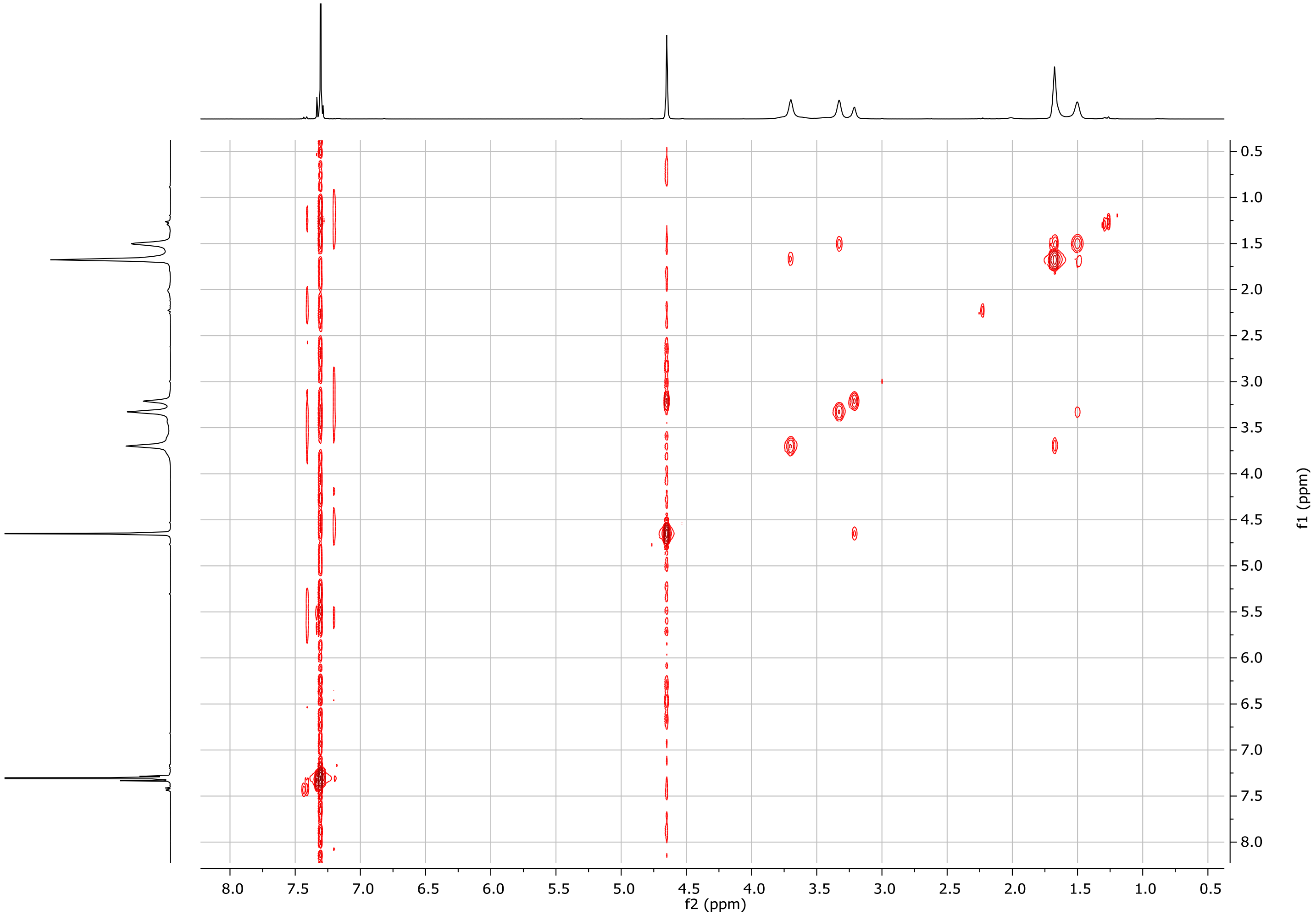

^{1}H, ^{13}C HMBC

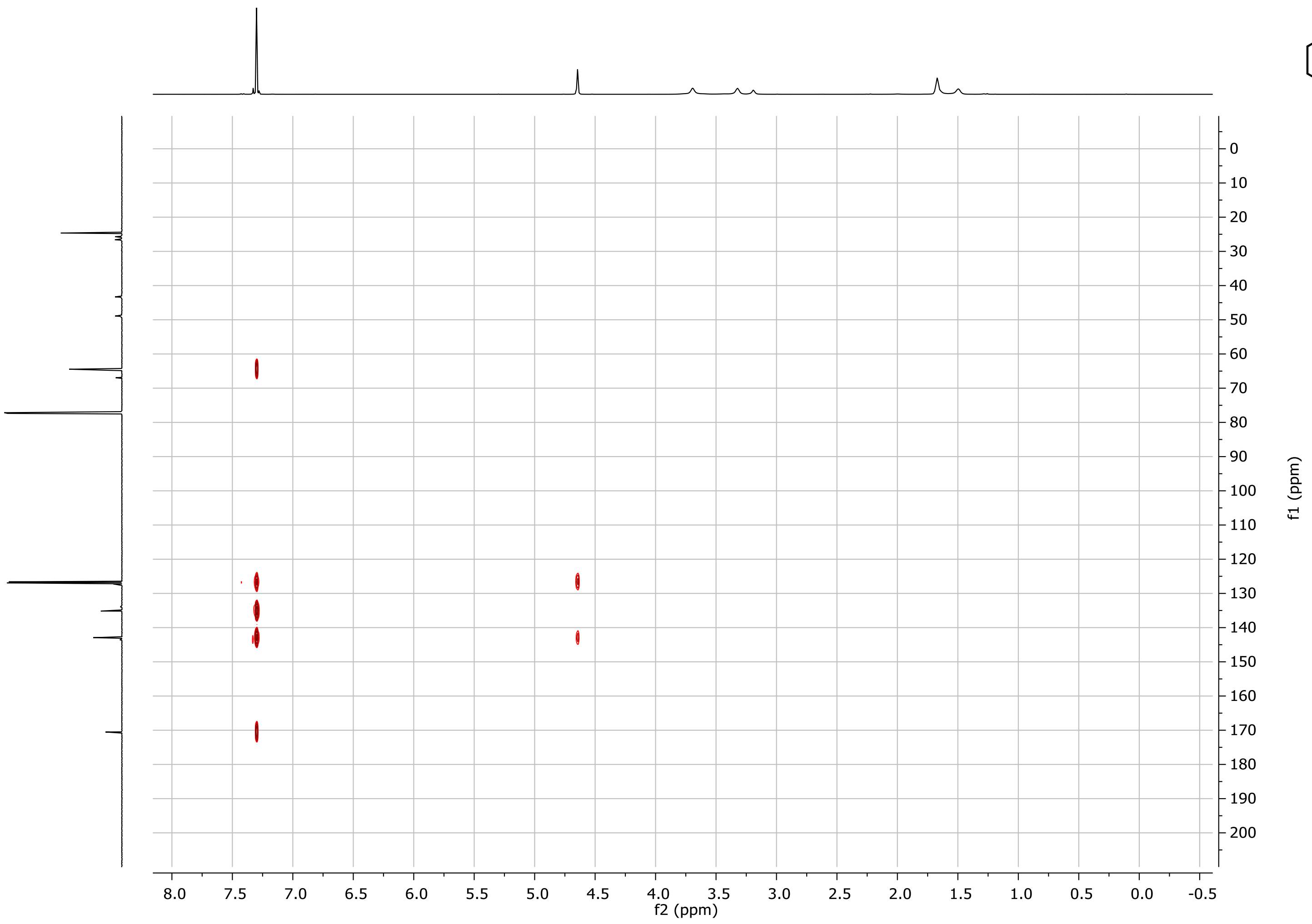

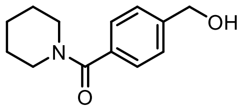

17c

^{1}H, ^{13}C HSQC

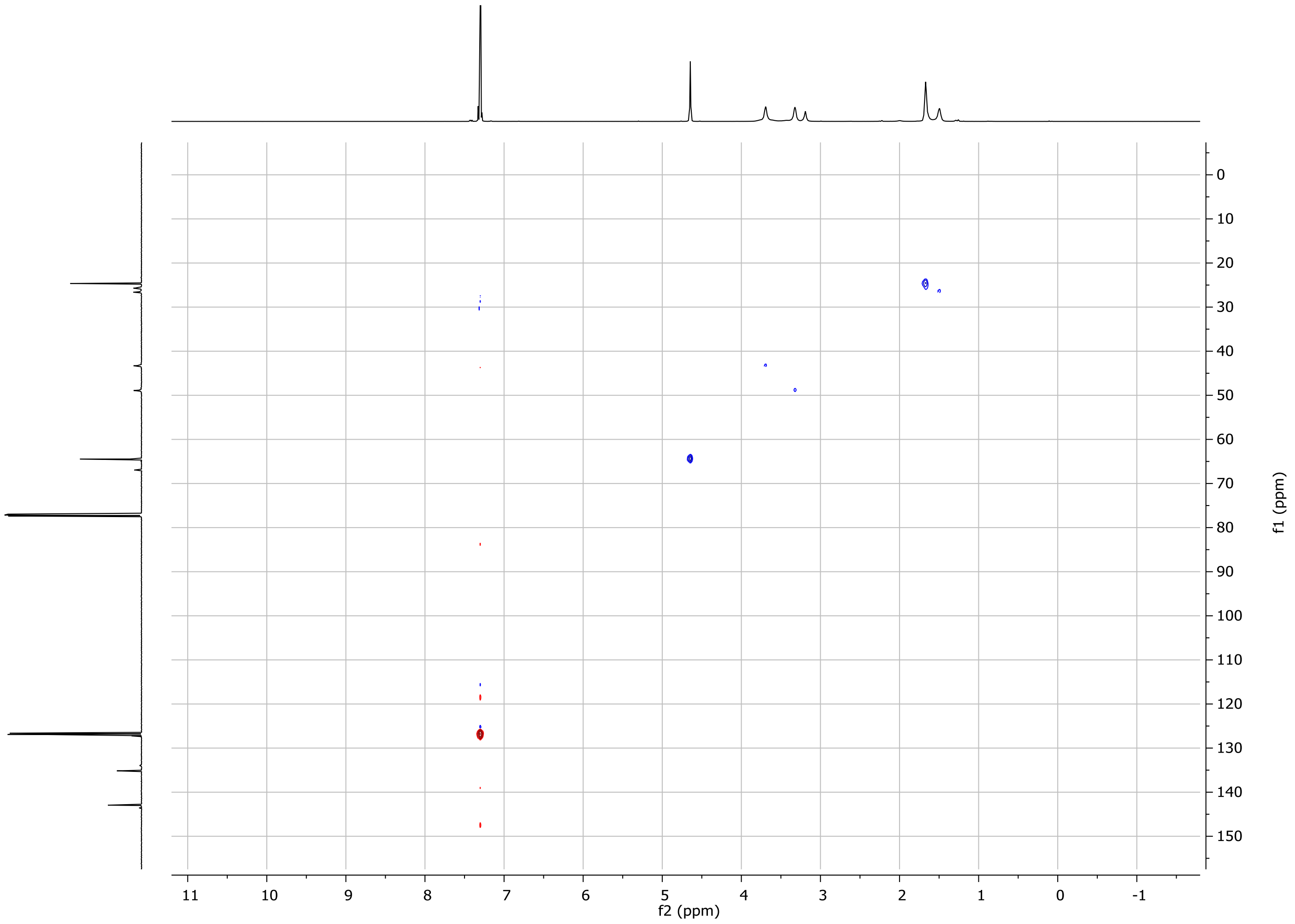

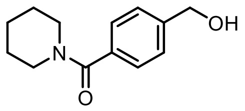

17c

HRMS

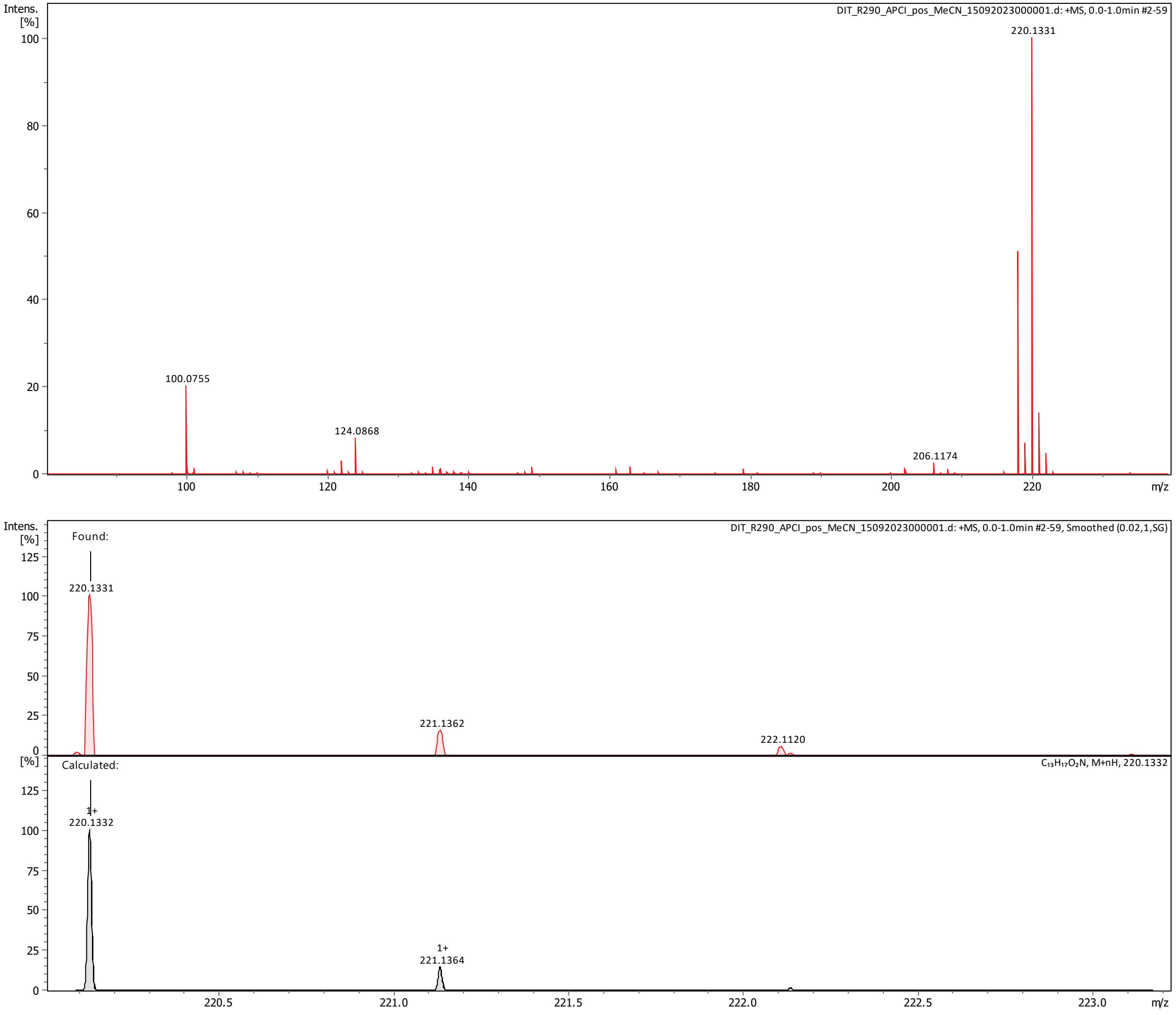
Intens. [%]
DIT_R290_APCI_pos_MeCN_15092023000001.d: +MS, 0.0-1.0min #2-59
220.1331
100.0755
124.0868
206.1174
m/z
Found:
DIT_R290_APCI_pos_MeCN_15092023000001.d: +MS, 0.0-1.0min #2-59, Smoothed (0.02,1,SG)
220.1331
221.1362
222.1120
Calculated:
$C_{13}H_{17}O_2N$, M+nH, 220.1332
1+
220.1332
1+
221.1364

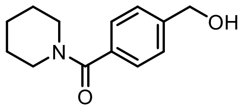
N
OH
O

17c

IR

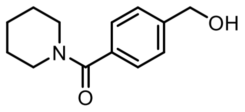

N
O
OH

17c

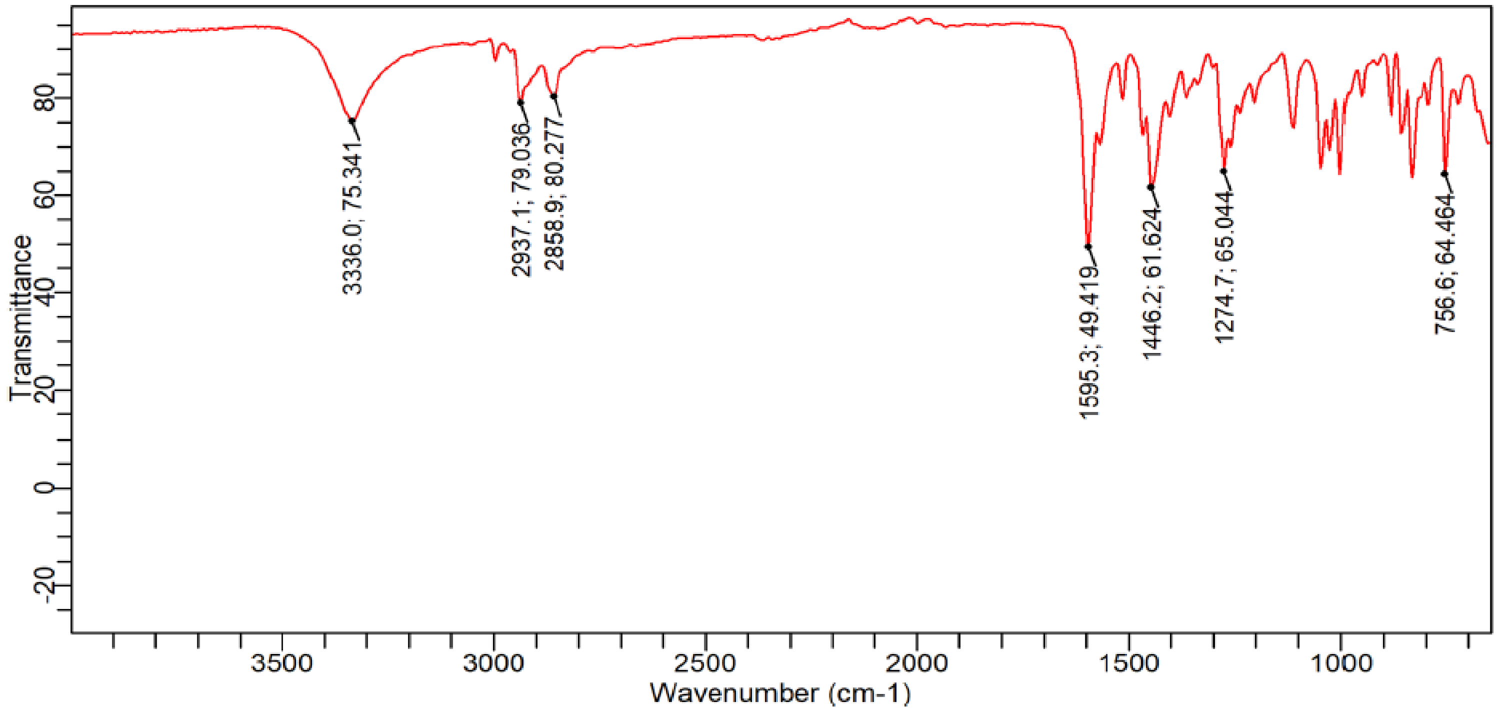

Transmittance
80
60
40
20
0
-20
3336.0; 75.341
2937.1; 79.036
2858.9; 80.277
1595.3; 49.419
1446.2; 61.624
1274.7; 65.044
756.6; 64.464
3500
3000
2500
2000
1500
1000
Wavenumber (cm-1)

80 (2-Methylphenyl)-1-piperidinylmethanone (S103)

^{1}H NMR

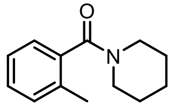

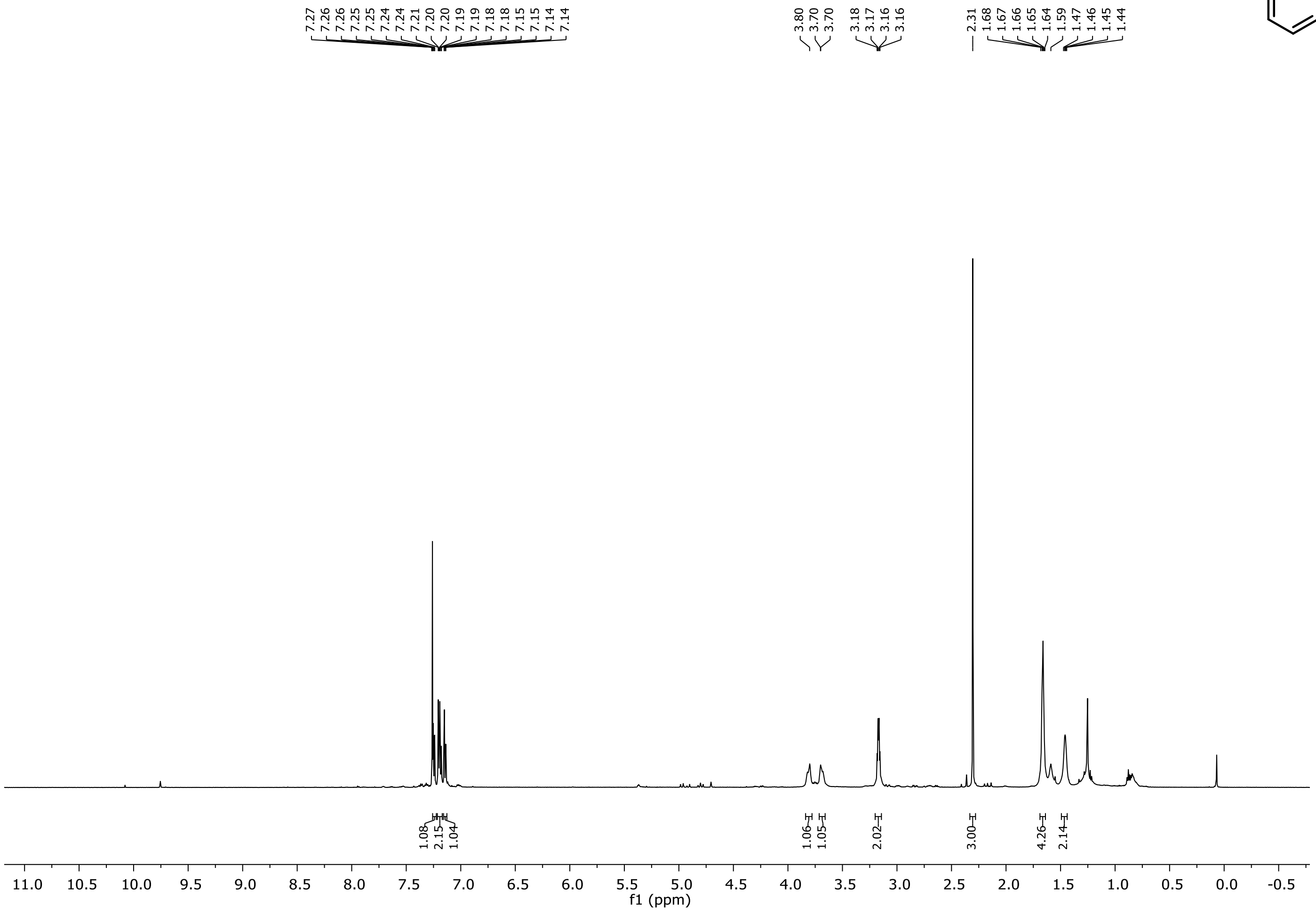

^{13}C NMR

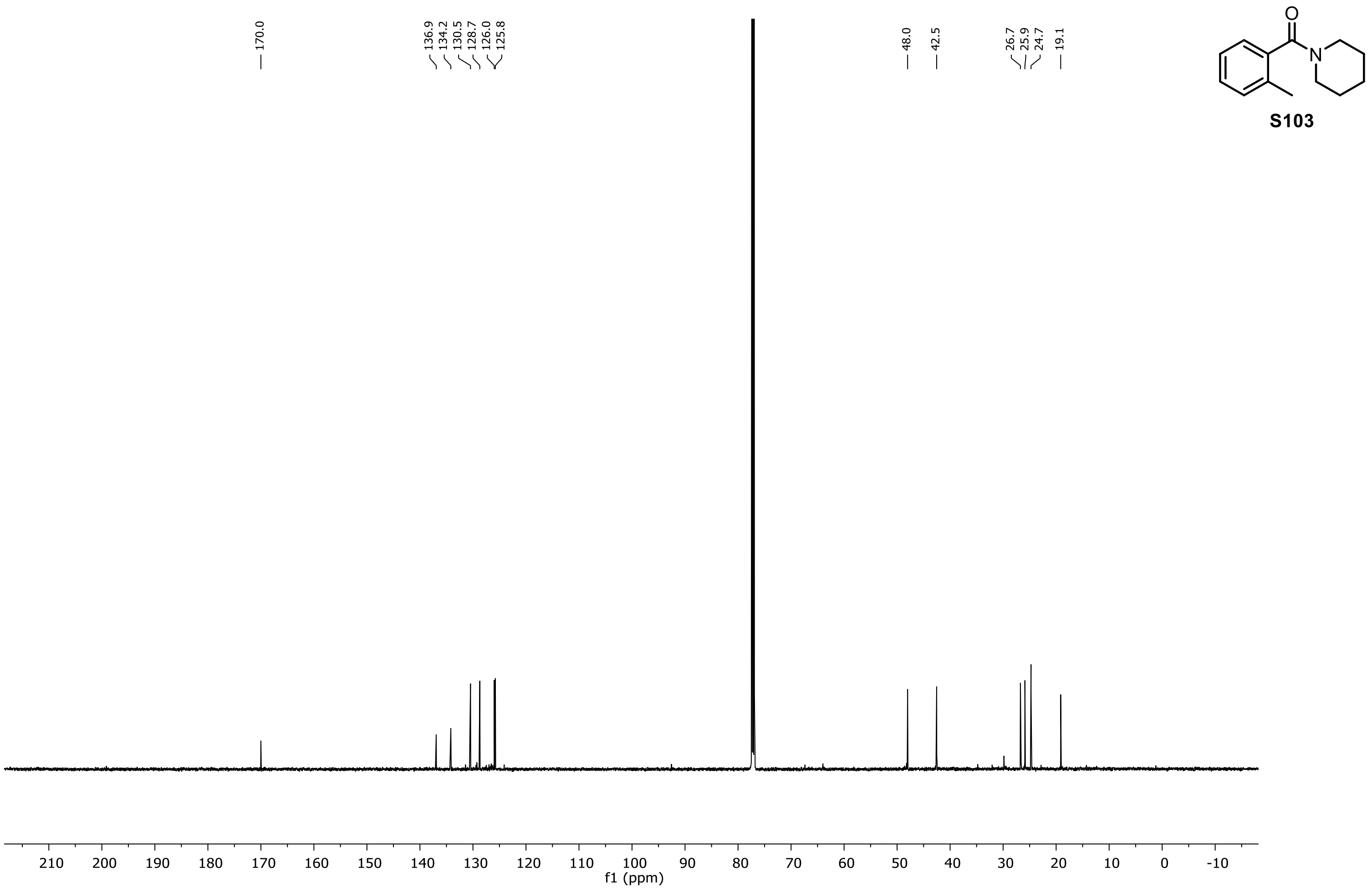

170.0
136.9
134.2
130.5
128.7
126.0
125.8
48.0
42.5
26.7
25.9
24.7
19.1
210 200 190 180 170 160 150 140 130 120 110 100 90 80 70 60 50 40 30 20 10 0 -10
f1 (ppm)
O
N

S103

¹H, ¹H COSY

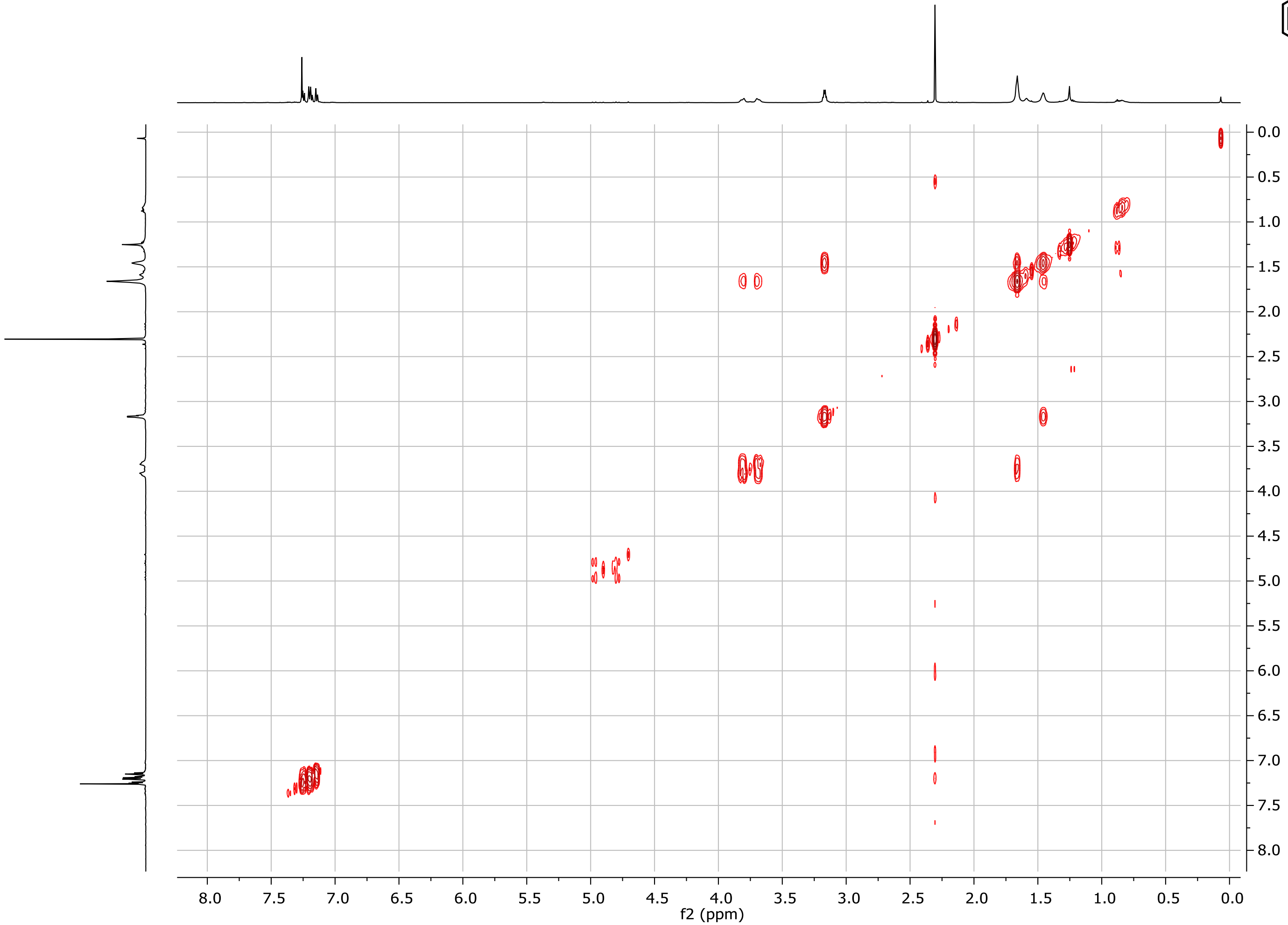

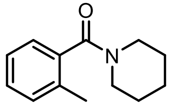

S103

^{1}H, ^{13}C HMBC

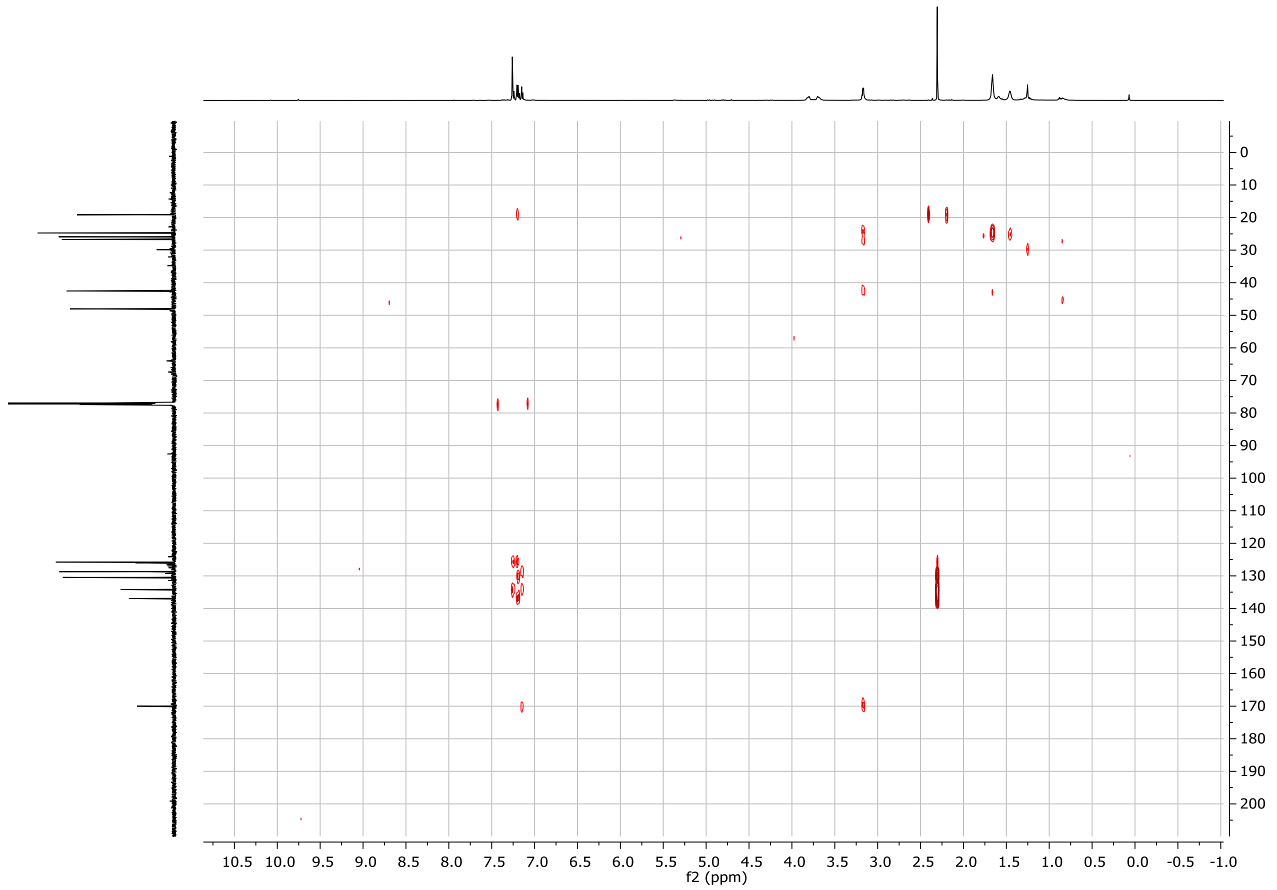

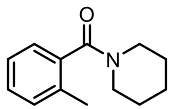

S103

^1H, ^{13}C HSQC

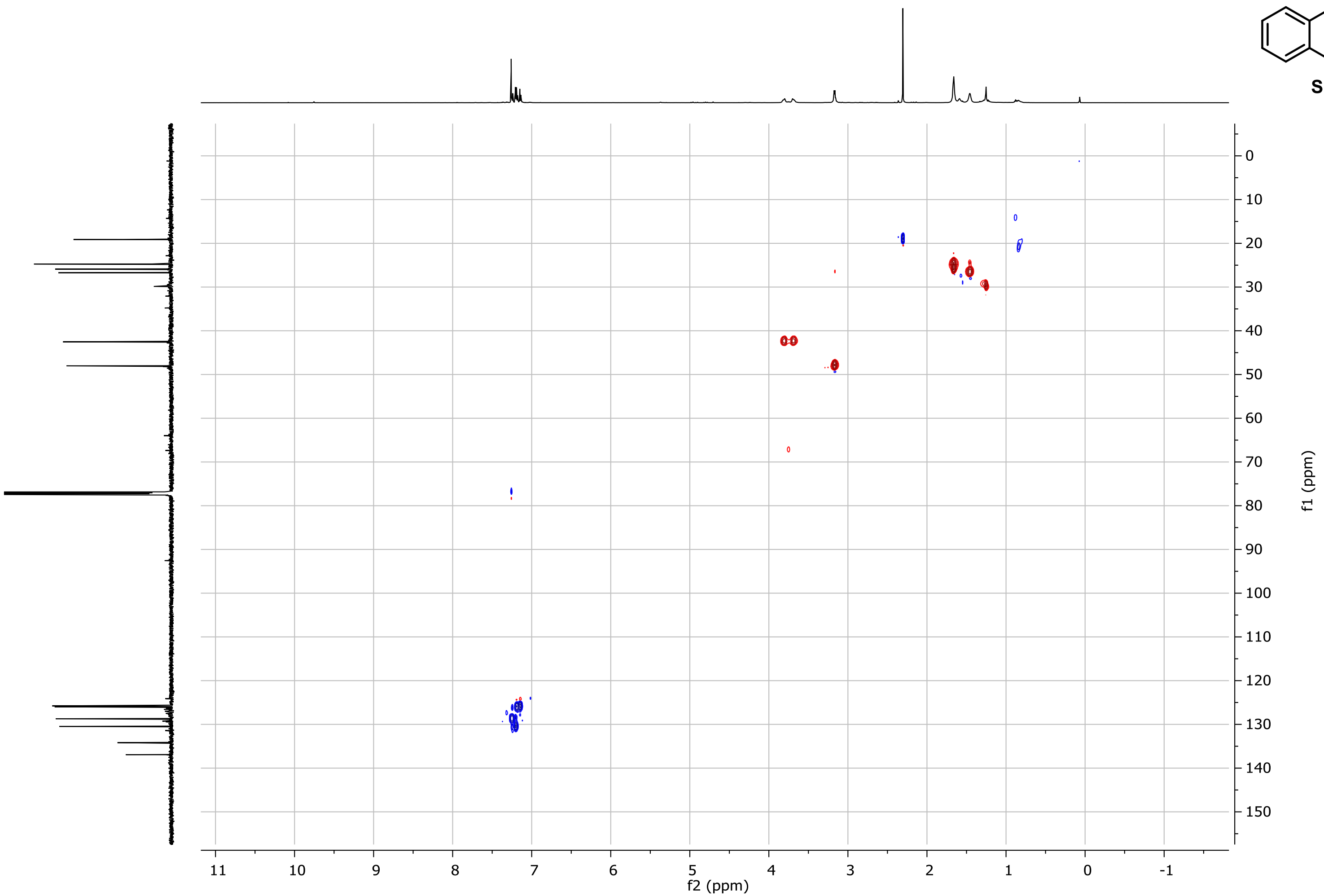

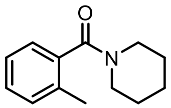

S103

GC-MS

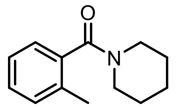

S103

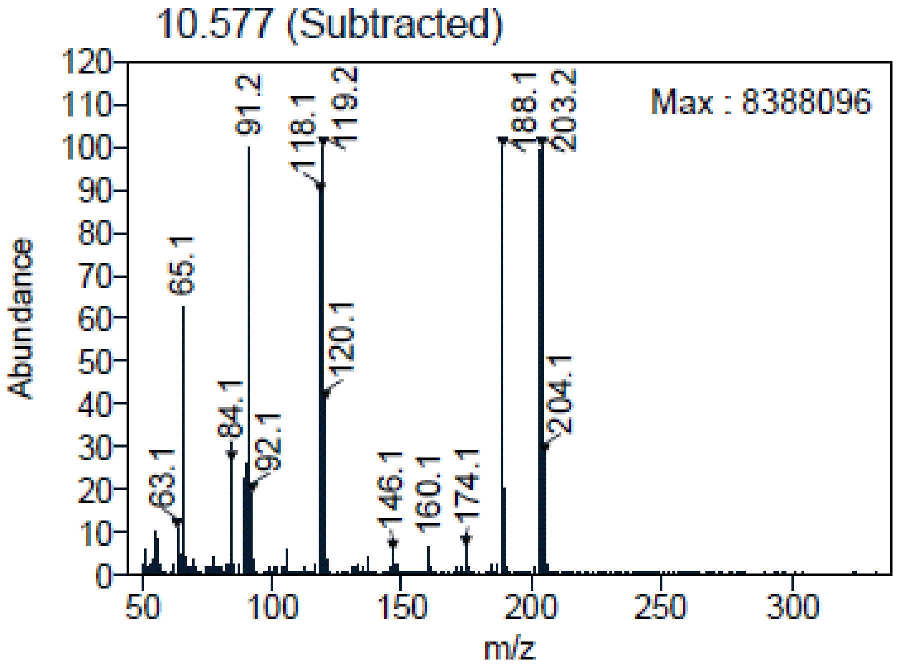
10.577 (Subtracted)
Max : 8388096
Abundance
m/z
65.1
63.1
84.1
91.2
92.1
118.1
119.2
120.1
146.1
160.1
174.1
188.1
203.2
204.1
120
110
100
90
80
70
60
50
40
30
20
10
0
50
100
150
200
250
300

81 (4-Chlorophenyl)methanol (2s) from 7-[4-(4-chlorobenzoyl)piperazin-1-yl]-1-cyclopropyl-6-fluoro-3-(piperidine-1-carbonyl)quinolin-4-one (XX)

^{1}H NMR

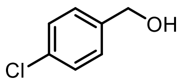

2s

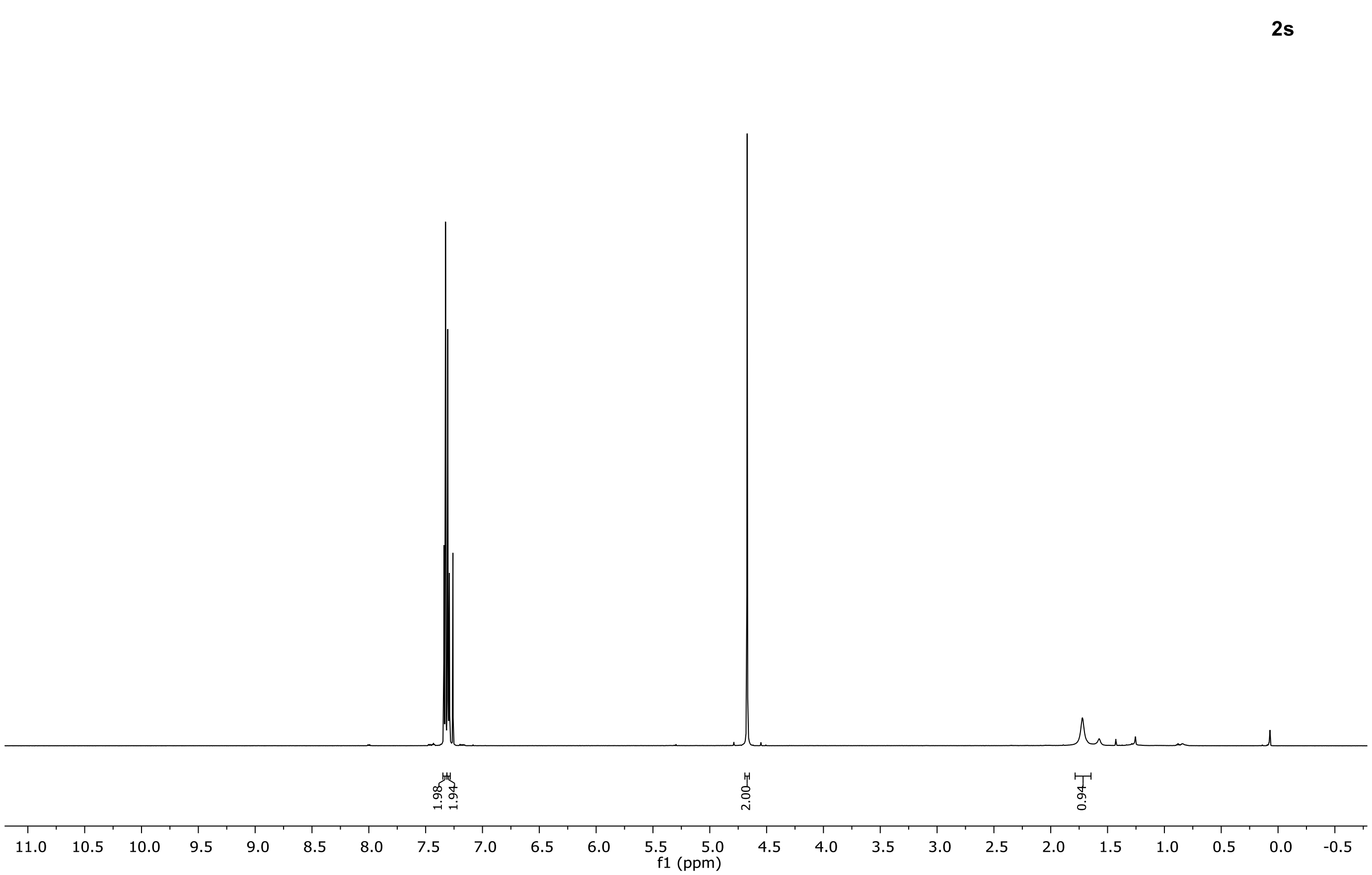

^{13}C NMR

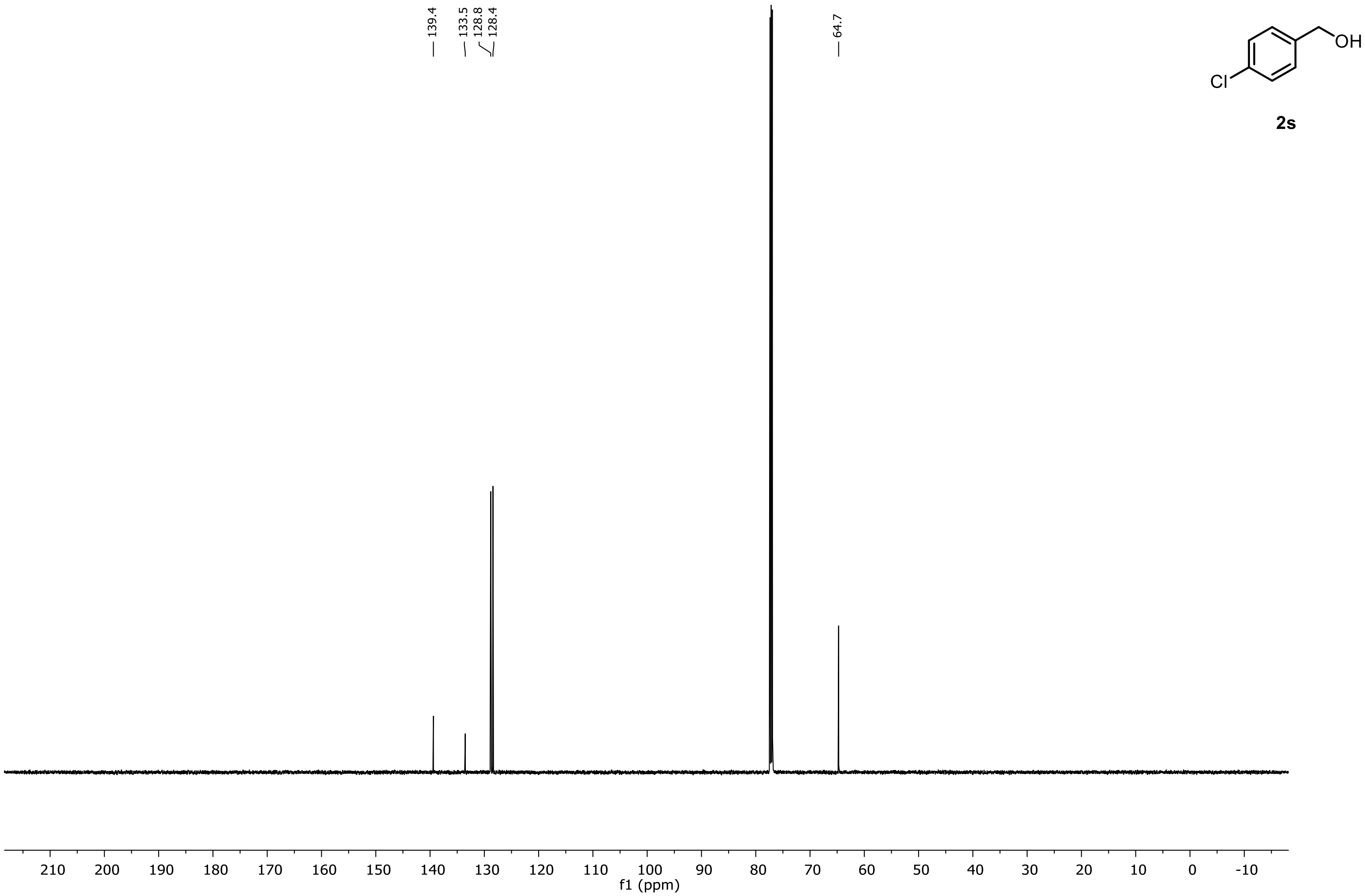
139.4
133.5
128.8
128.4
64.7
OH
Cl
210 200 190 180 170 160 150 140 130 120 110 100 90 80 70 60 50 40 30 20 10 0 -10
f1 (ppm)

2s

^{1}H, ^{1}H COSY

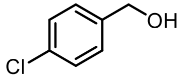
Cl
OH

2s

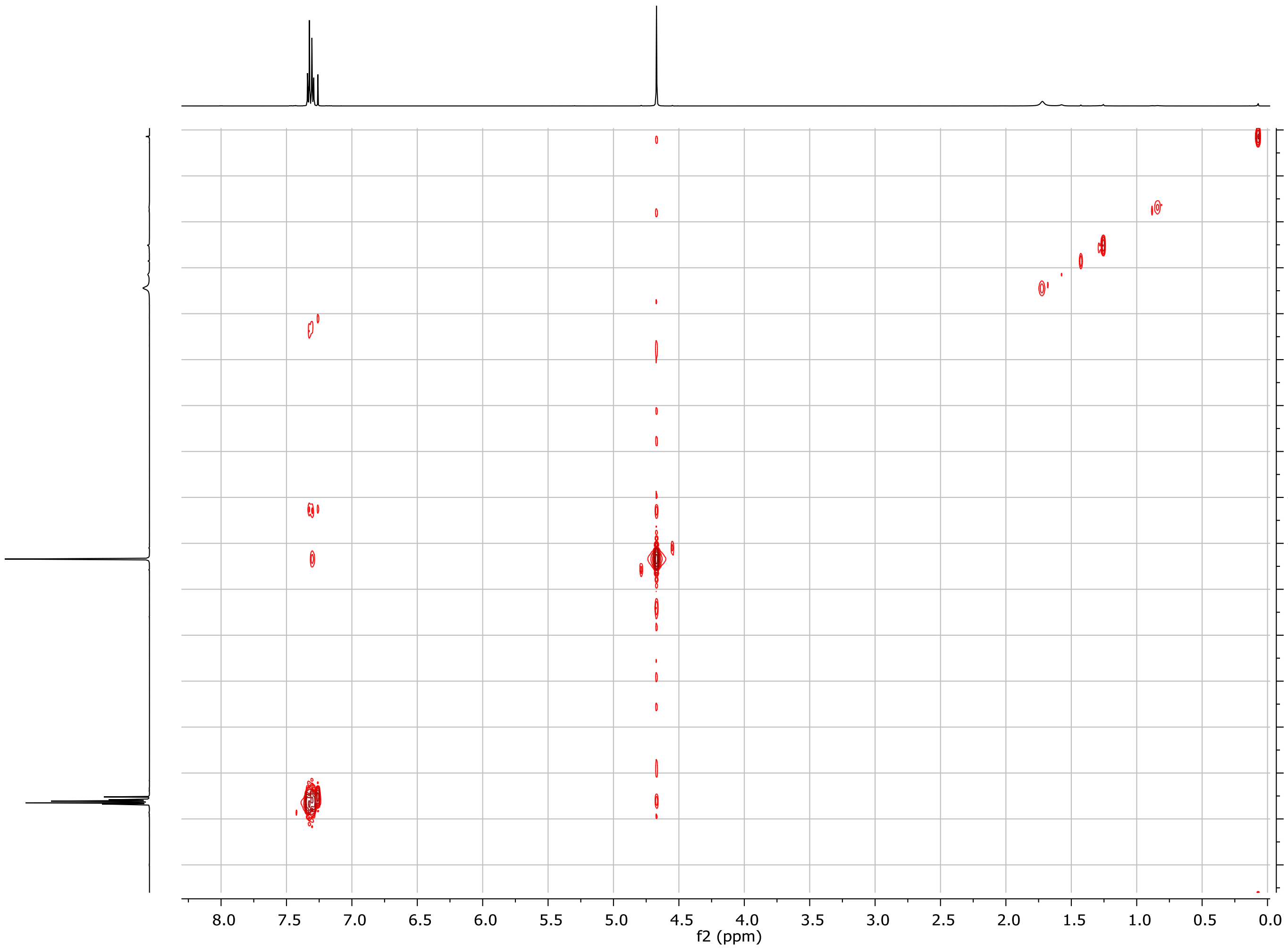
8.0 7.5 7.0 6.5 6.0 5.5 5.0 4.5 4.0 3.5 3.0 2.5 2.0 1.5 1.0 0.5 0.0
f2 (ppm)
0.0 0.5 1.0 1.5 2.0 2.5 3.0 3.5 4.0 4.5 5.0 5.5 6.0 6.5 7.0 7.5 8.0
f1 (ppm)

^{1}H, ^{13}C HMBC

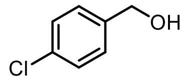

2s

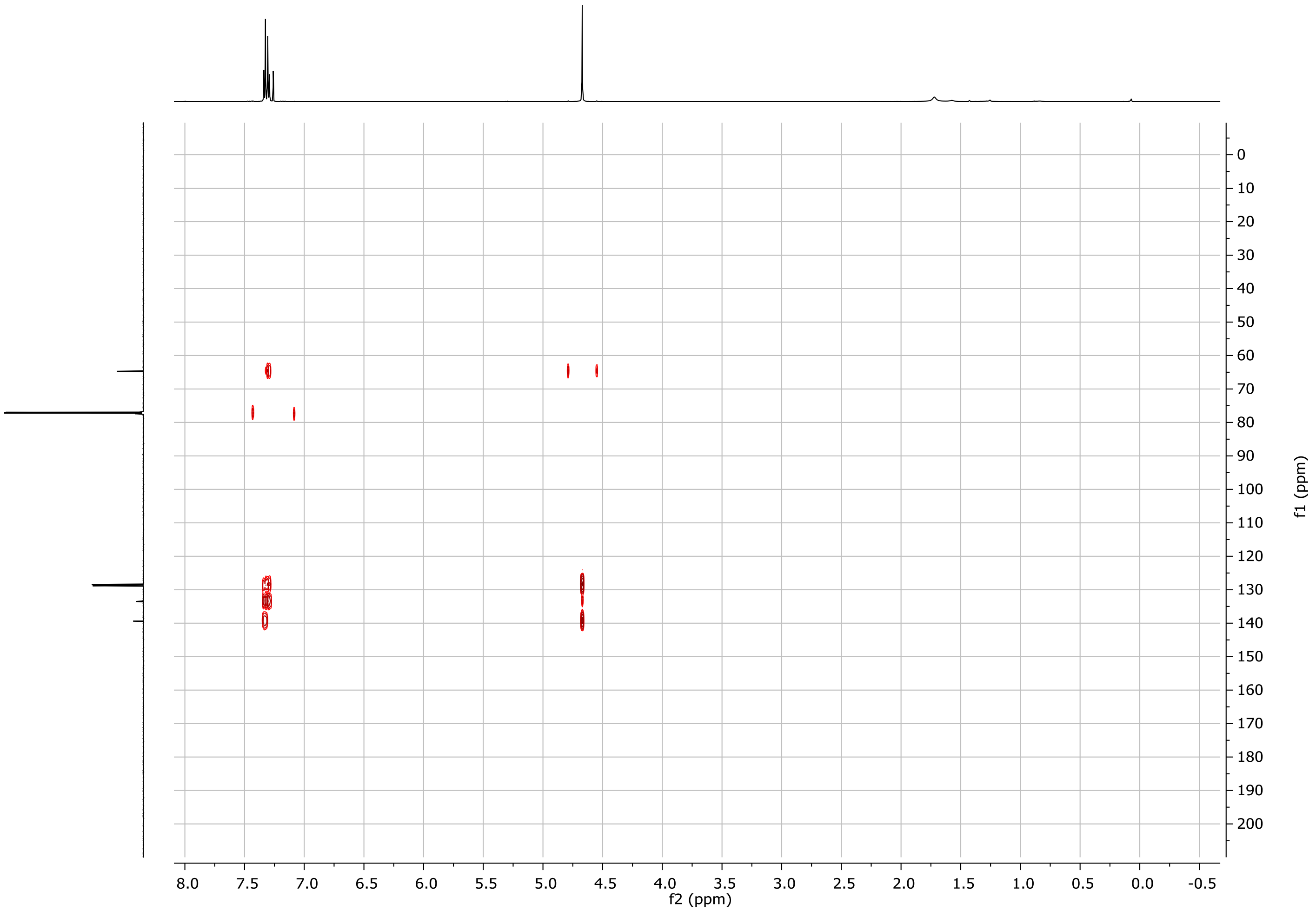

^1H, ^{13}C HSQC

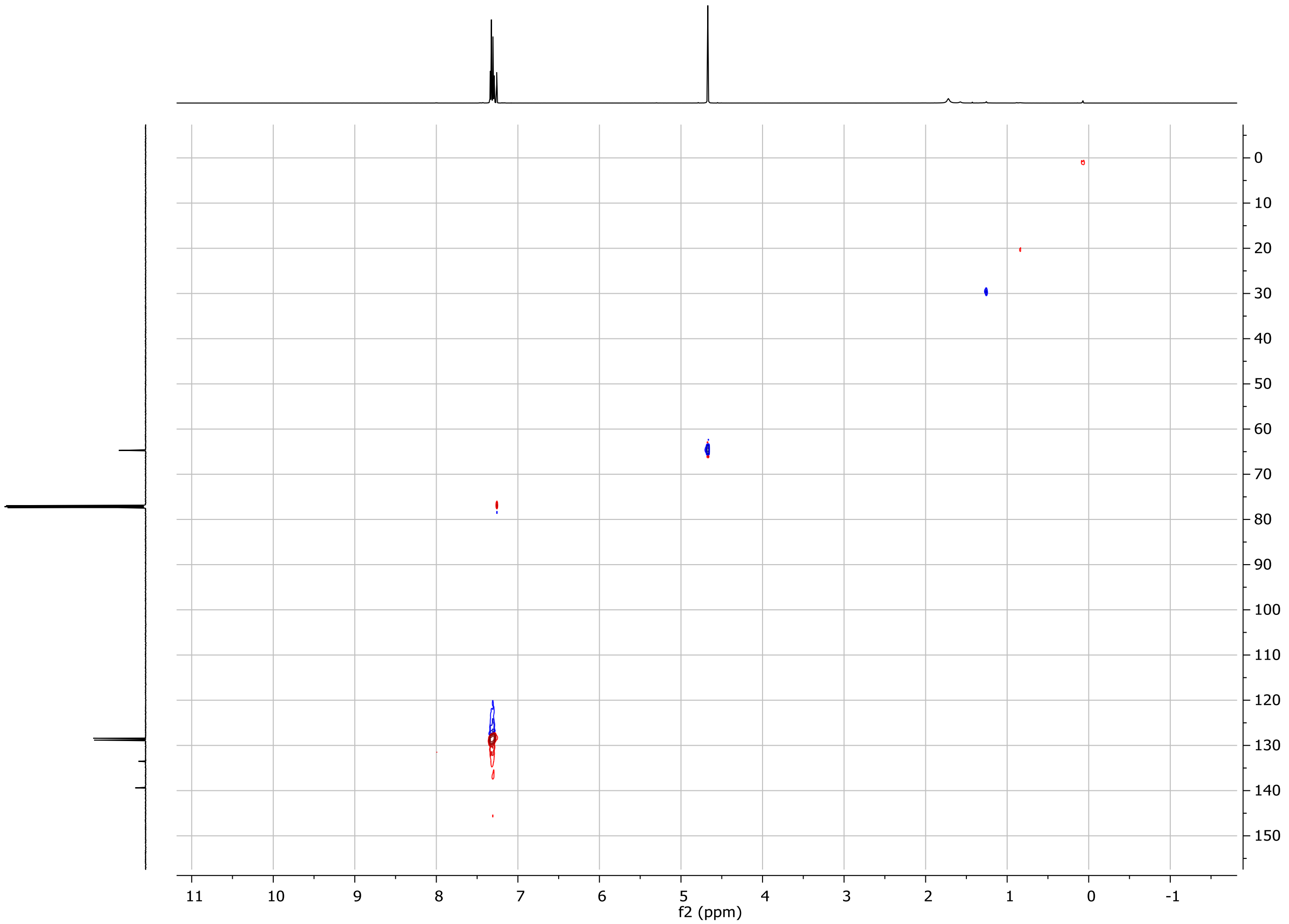

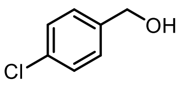

2s

82 7-[4-(4-Chlorobenzoyl)piperazin-1-yl]-1-cyclopropyl-6-fluoro-3-(piperidine-1-carbonyl)-2,3-dihydroquinolin-4-one (S104)

^{1}H NMR

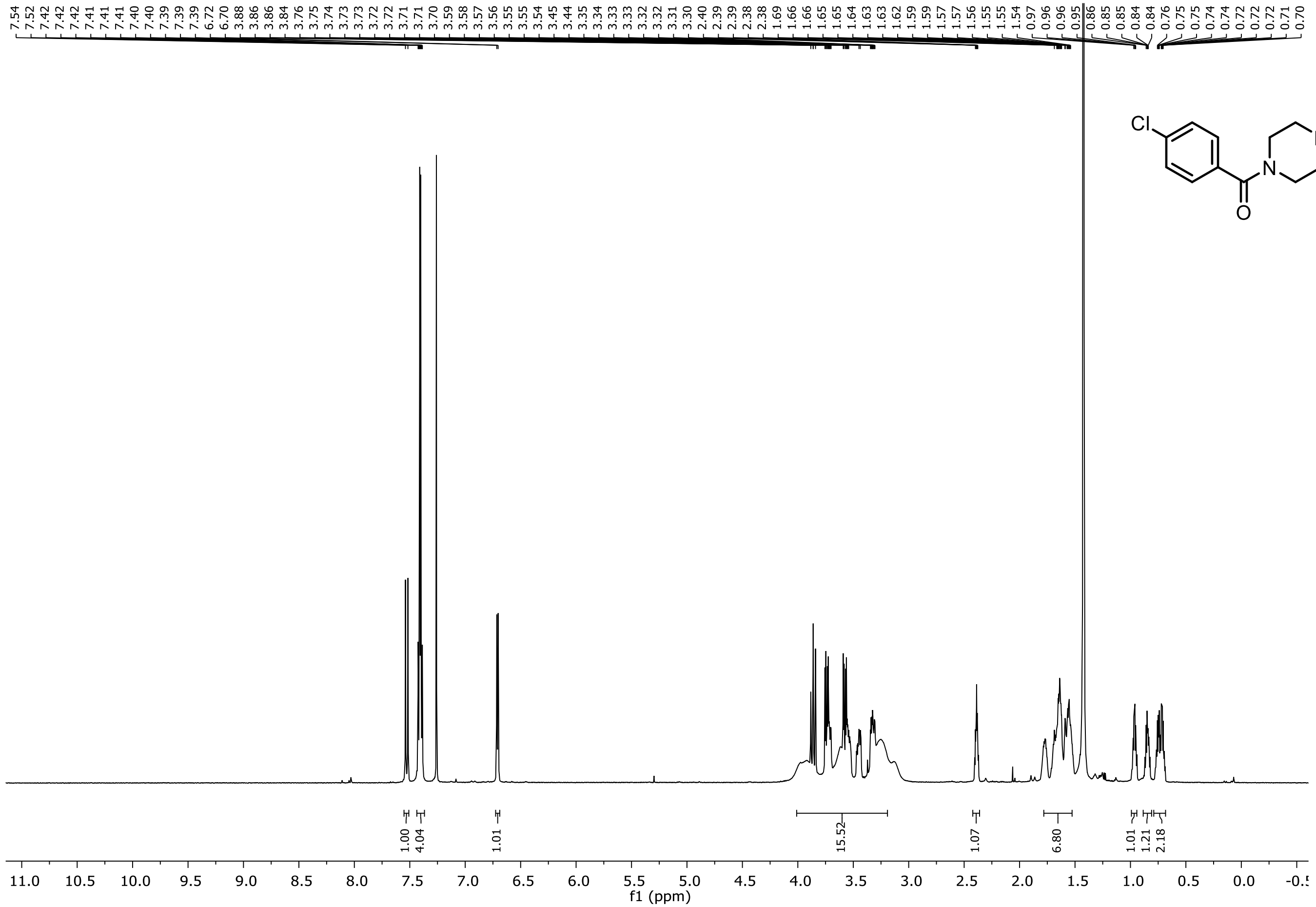

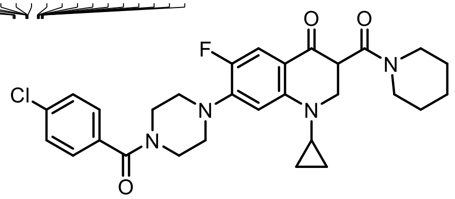

S104

^{13}C NMR

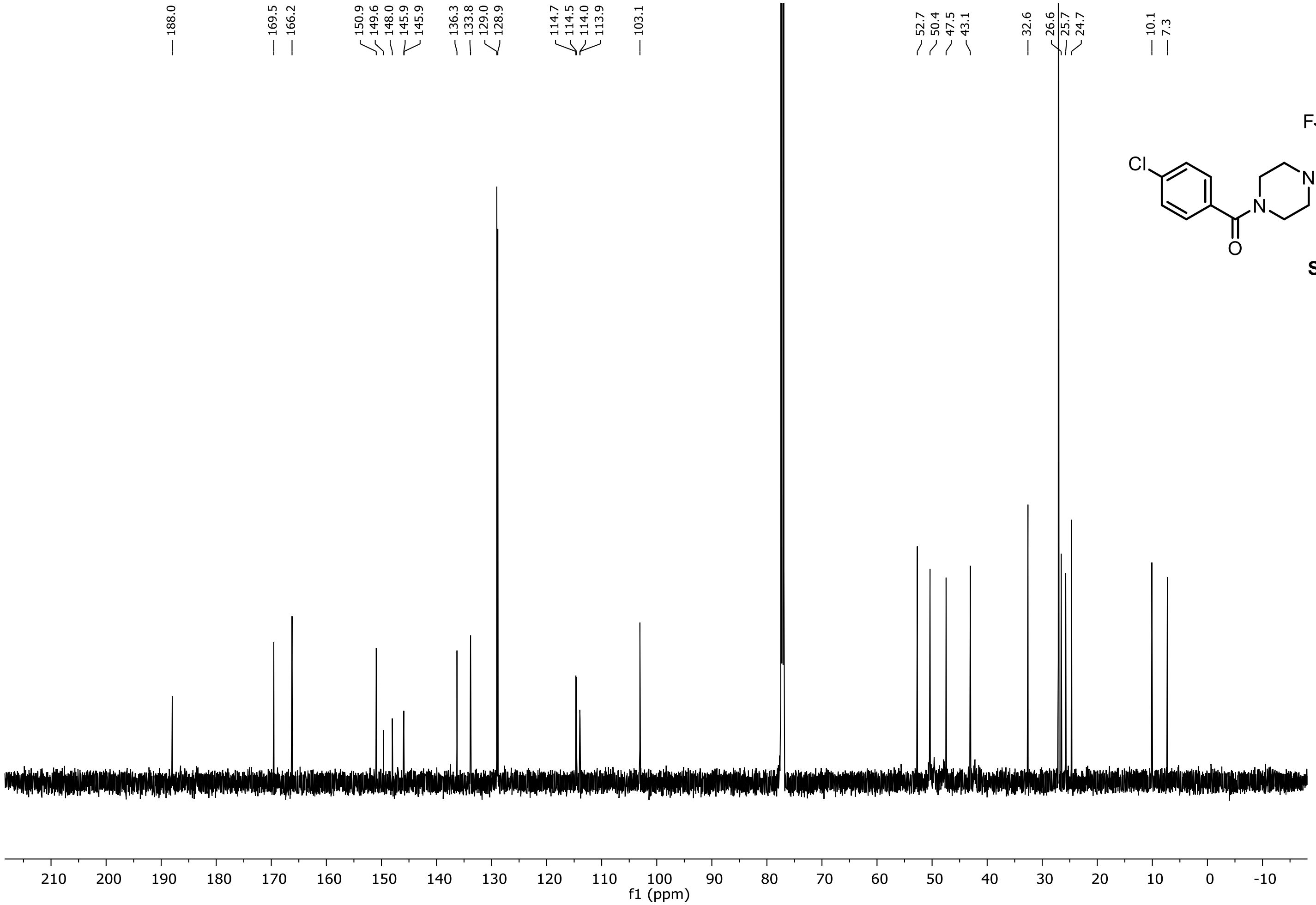
188.0
169.5
166.2
150.9
149.6
148.0
145.9
145.9
136.3
133.8
129.0
128.9
114.7
114.5
114.0
113.9
103.1
52.7
50.4
47.5
43.1
32.6
26.6
25.7
24.7
10.1
7.3
210 200 190 180 170 160 150 140 130 120 110 100 90 80 70 60 50 40 30 20 10 0 -10
f1 (ppm)

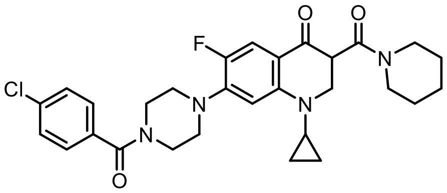
F
O
O
N
Cl
N
N
N
O

S104

^{19}F NMR

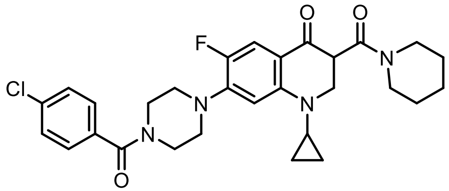

F
Cl
N
N
N
O
O
O
N

S104

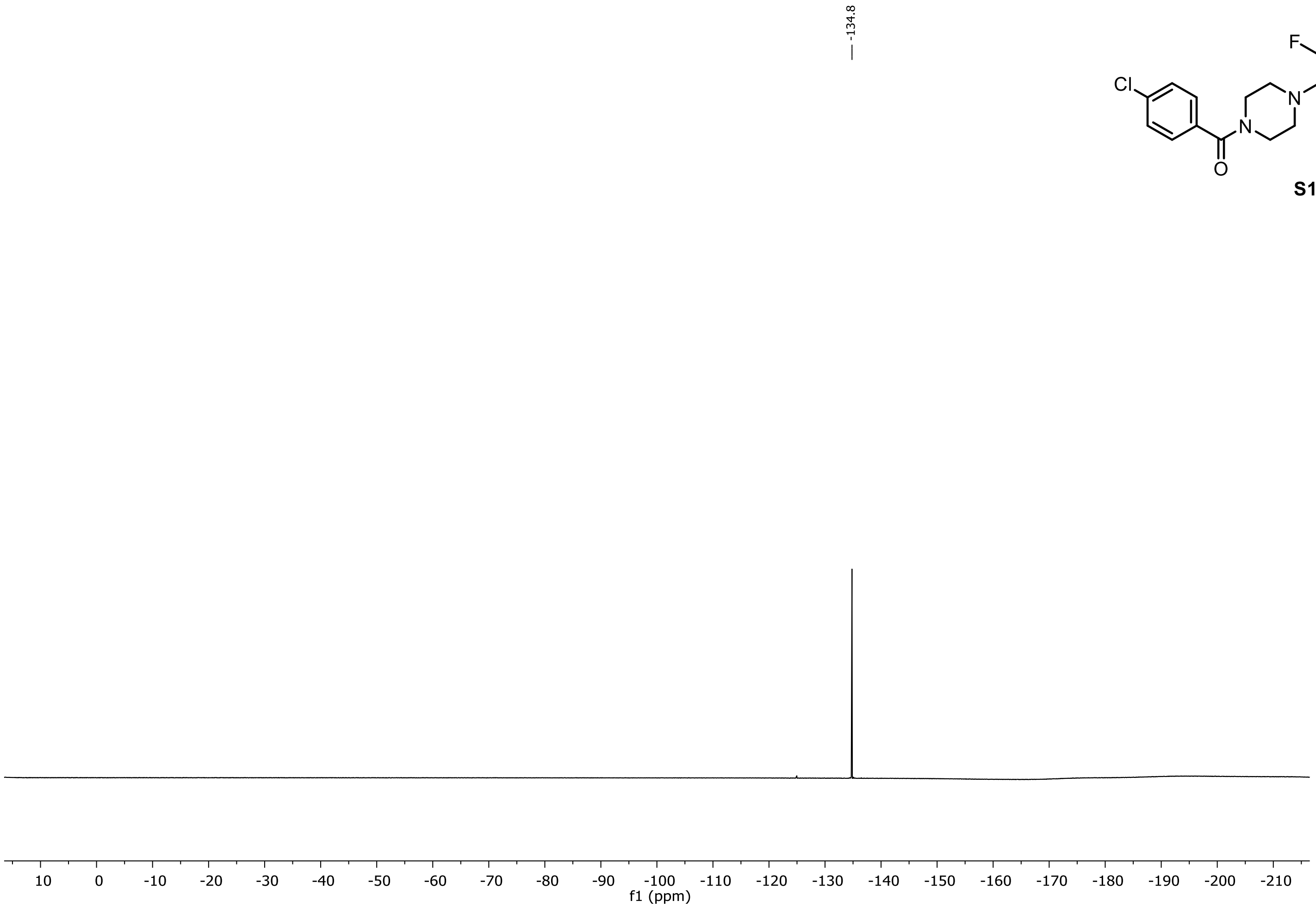

-134.8
10
0
-10
-20
-30
-40
-50
-60
-70
-80
-90
-100
-110
-120
-130
-140
-150
-160
-170
-180
-190
-200
-210
f1 (ppm)

^{1}H, ^{1}H COSY

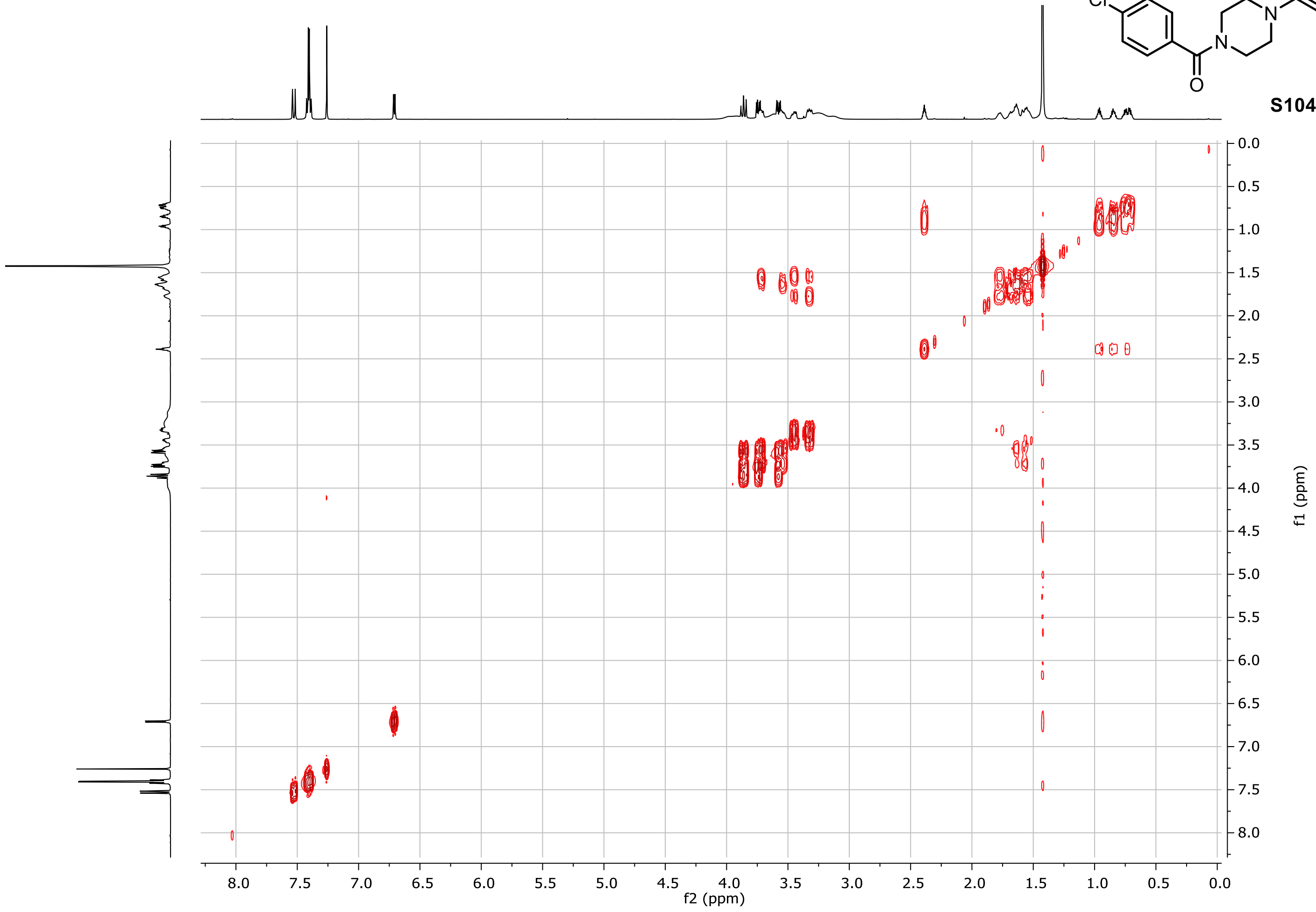

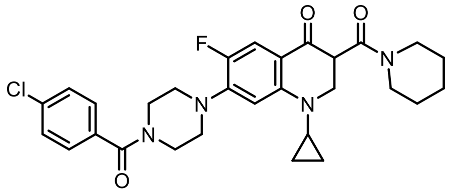

^{1}H, ^{13}C HMBC

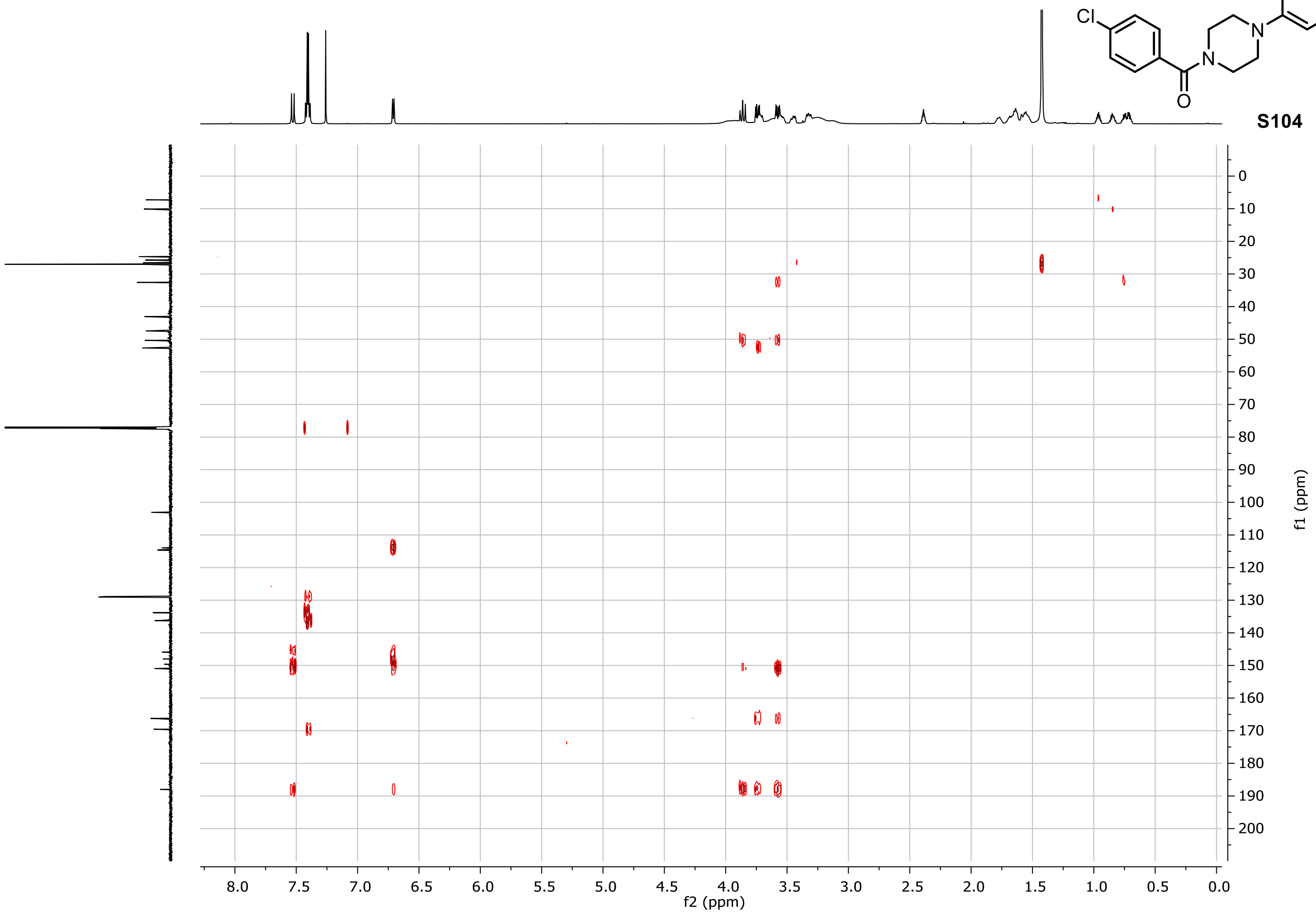

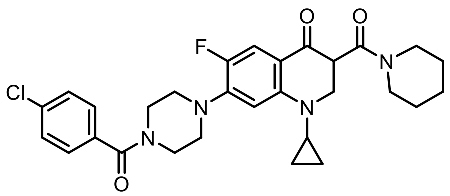

S104

^{1}H, ^{13}C HSQC

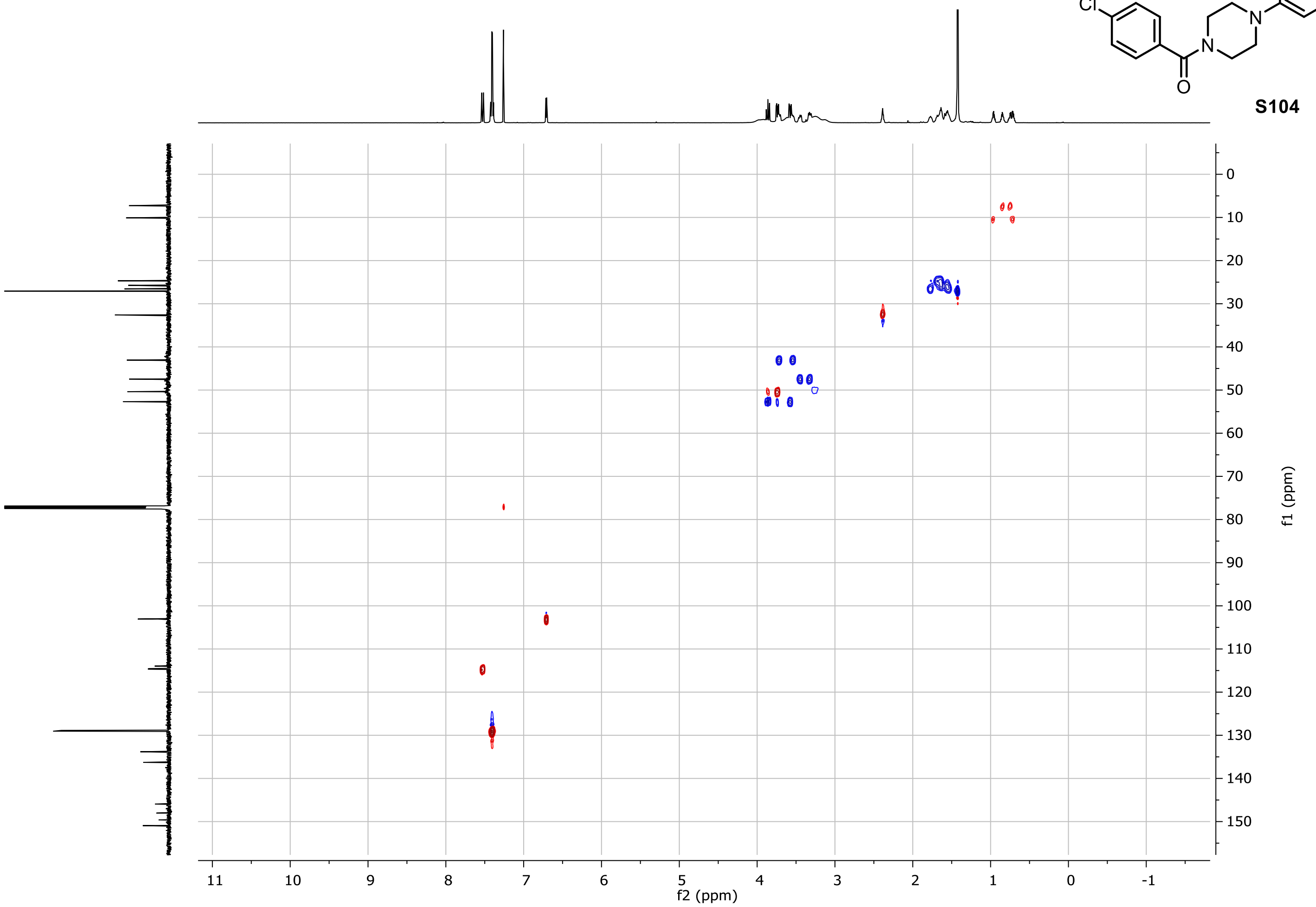

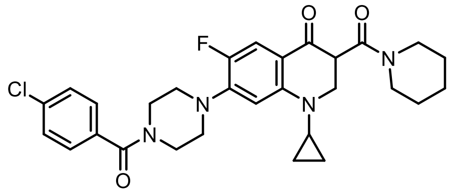

S104

IR

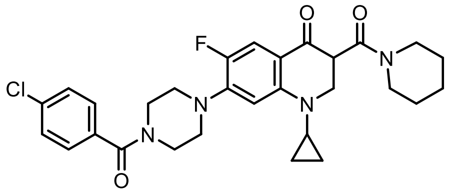

S104

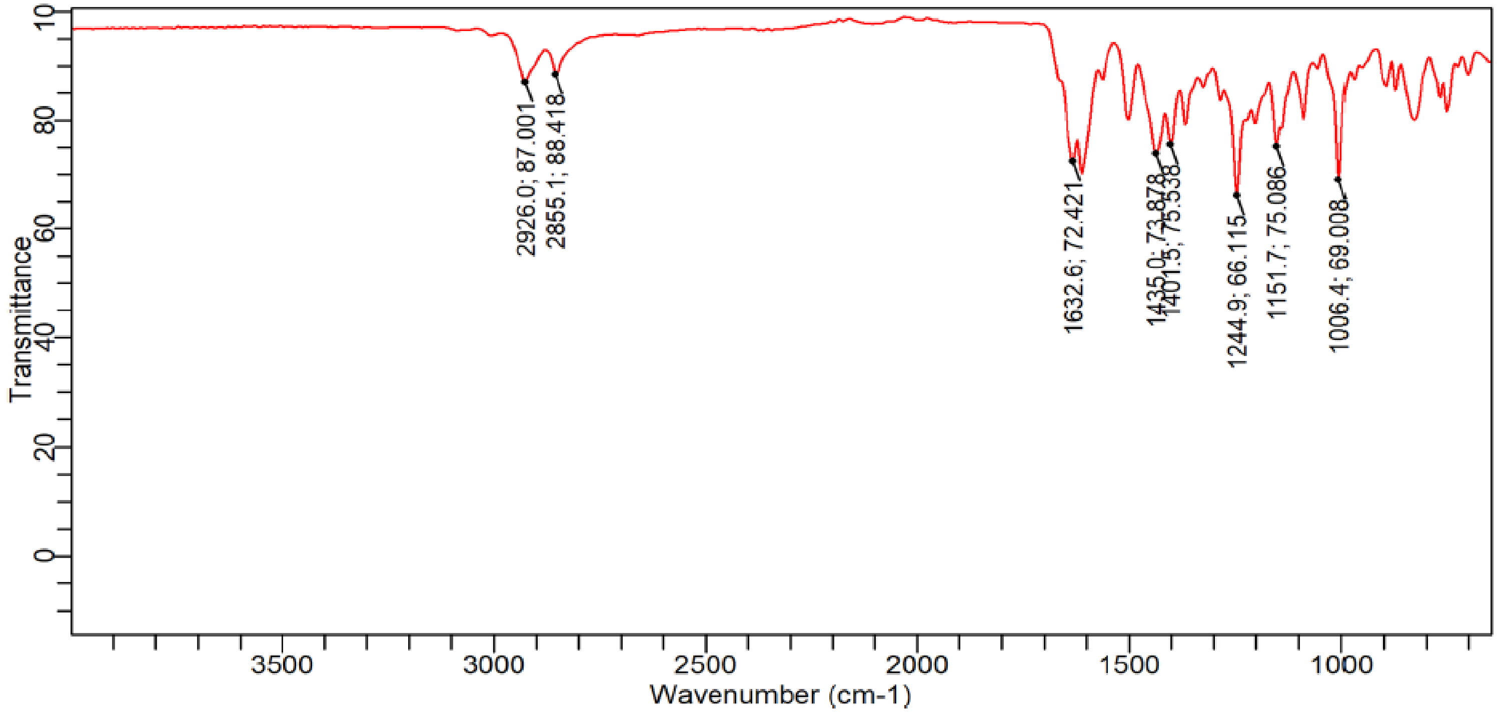

Transmittance
Wavenumber (cm-1)
2926.0; 87.001
2855.1; 88.418
1632.6; 72.421
1435.0; 73.878
1401.5; 75.538
1244.9; 66.115
1151.7; 75.086
1006.4; 69.008

83 Gram-Scale reaction-(4-Chlorophenyl)methanol (2s)

^{1}H NMR

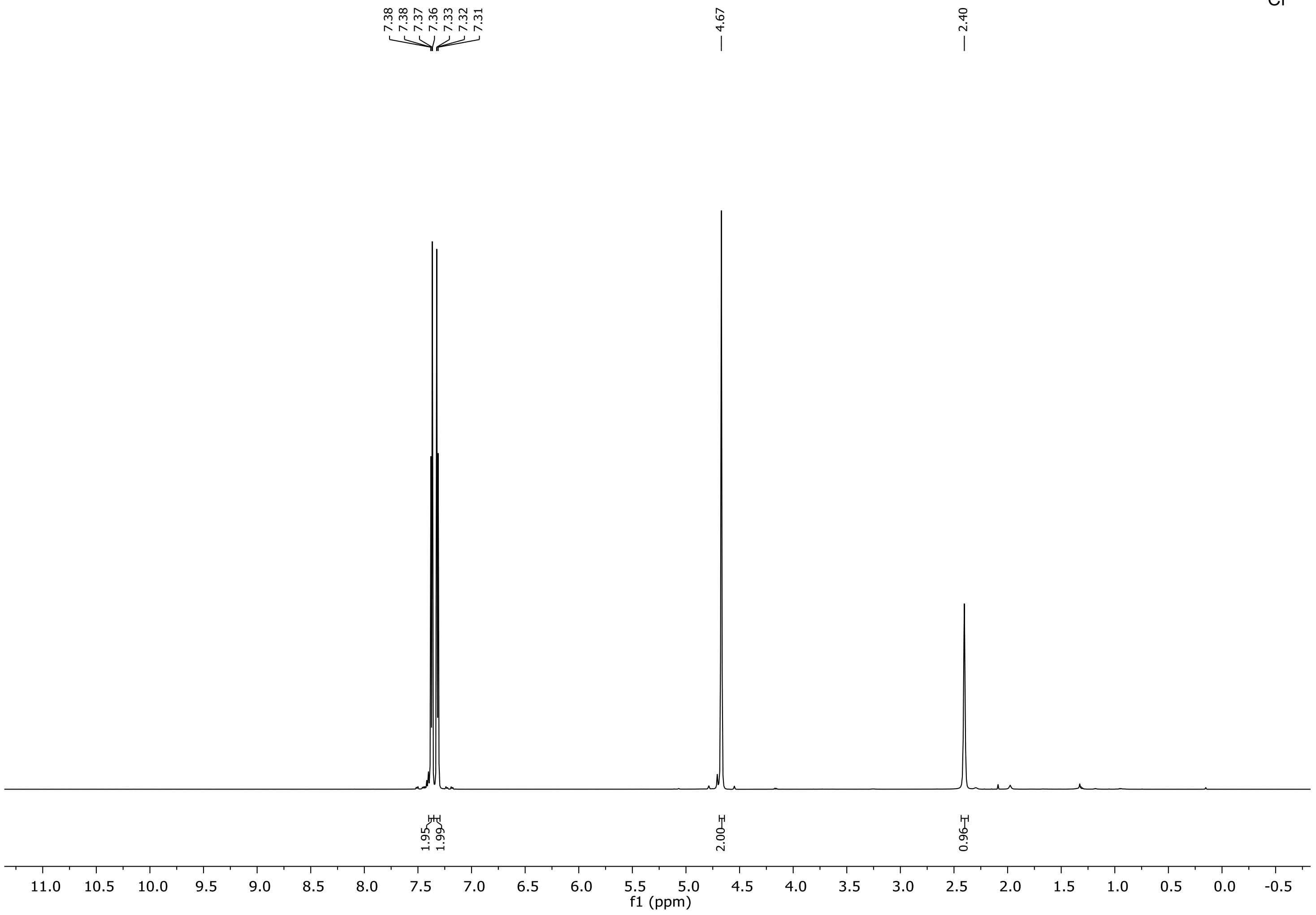

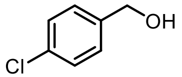

2s

^{13}C NMR

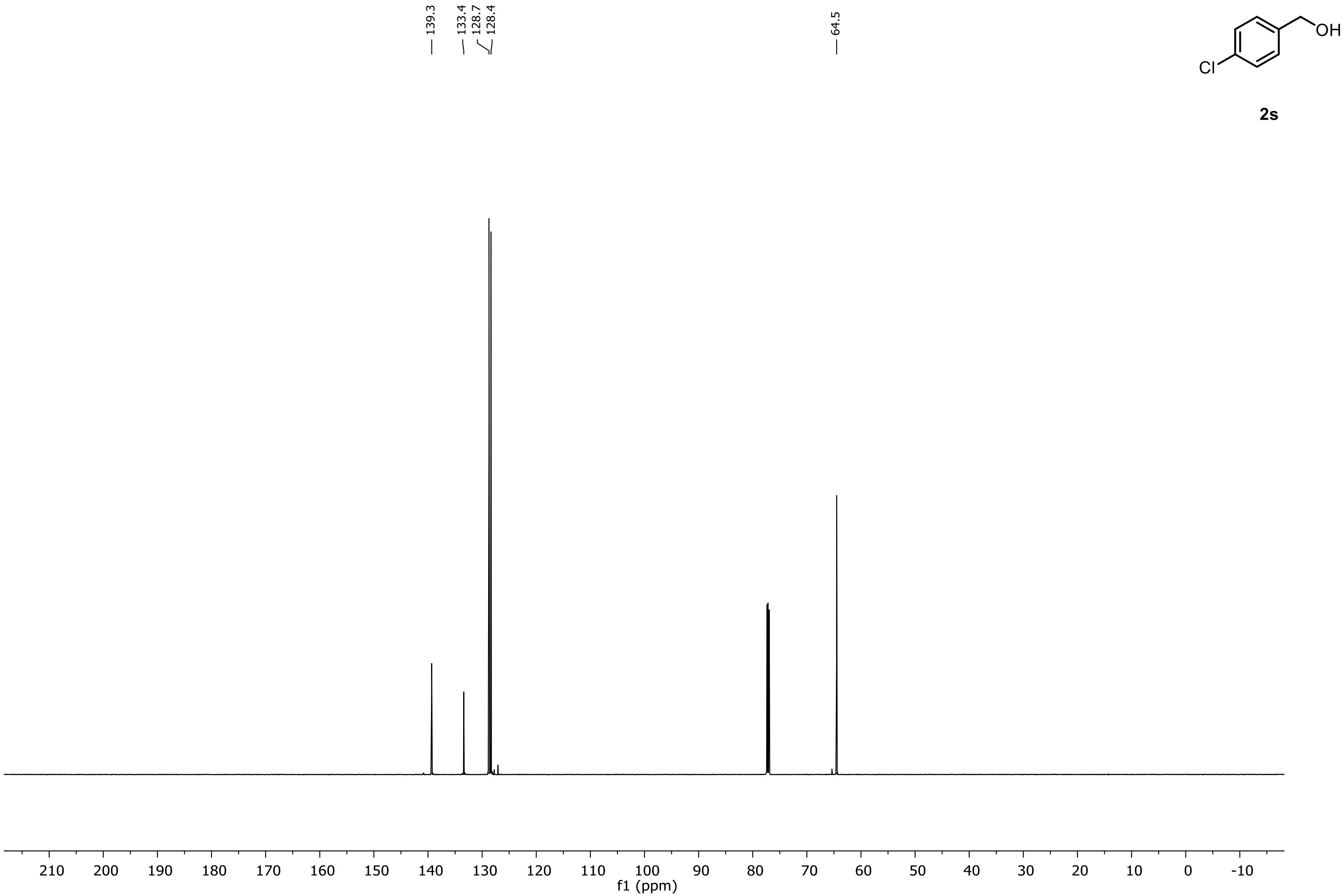

139.3
133.4
128.7
128.4
64.5
210 200 190 180 170 160 150 140 130 120 110 100 90 80 70 60 50 40 30 20 10 0 -10
f1 (ppm)
OH
Cl

2s

$^{1}H, {}^{1}H$ COSY

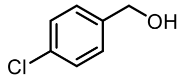

2s

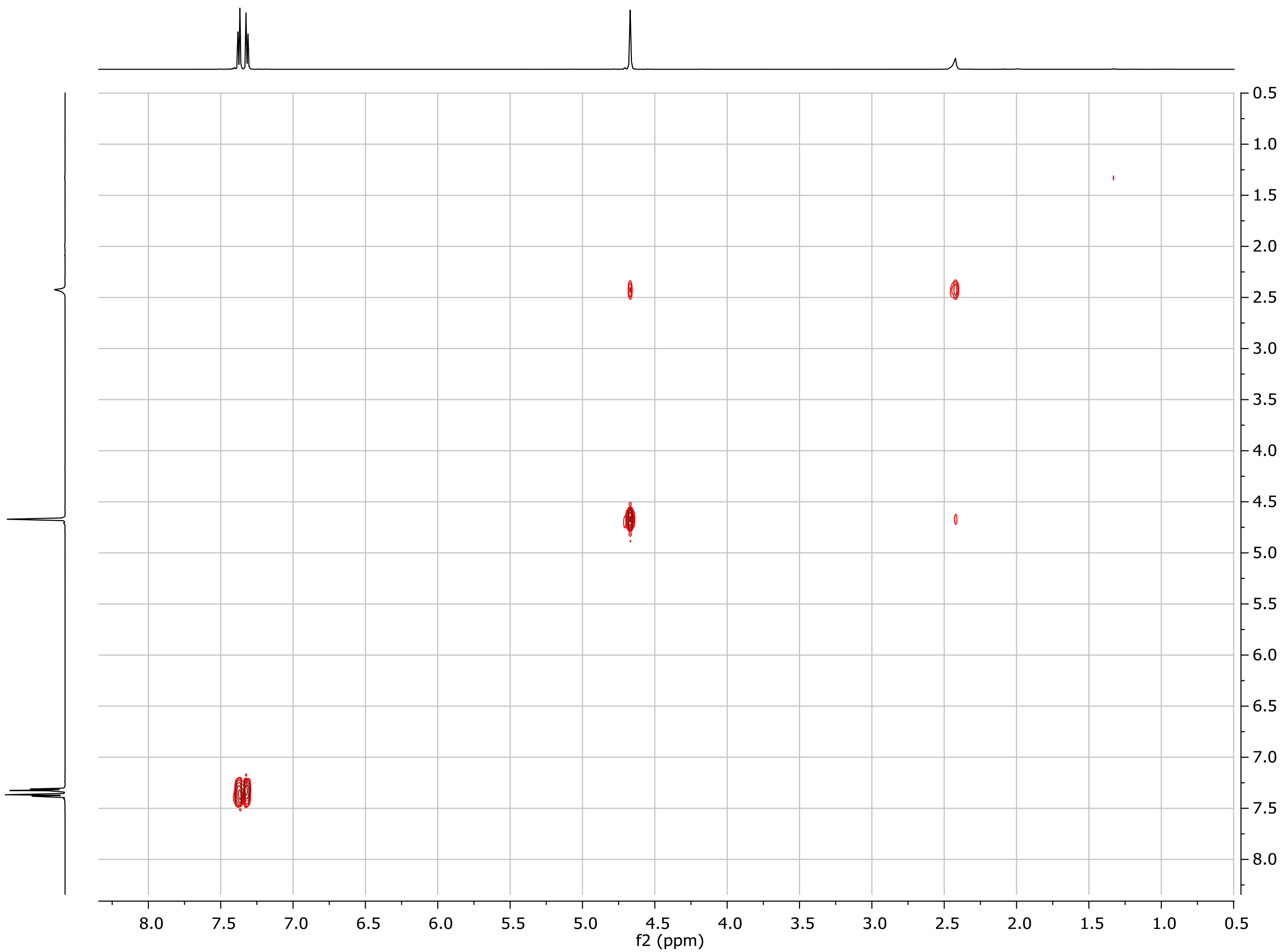

^1H, ^{13}C HMBC

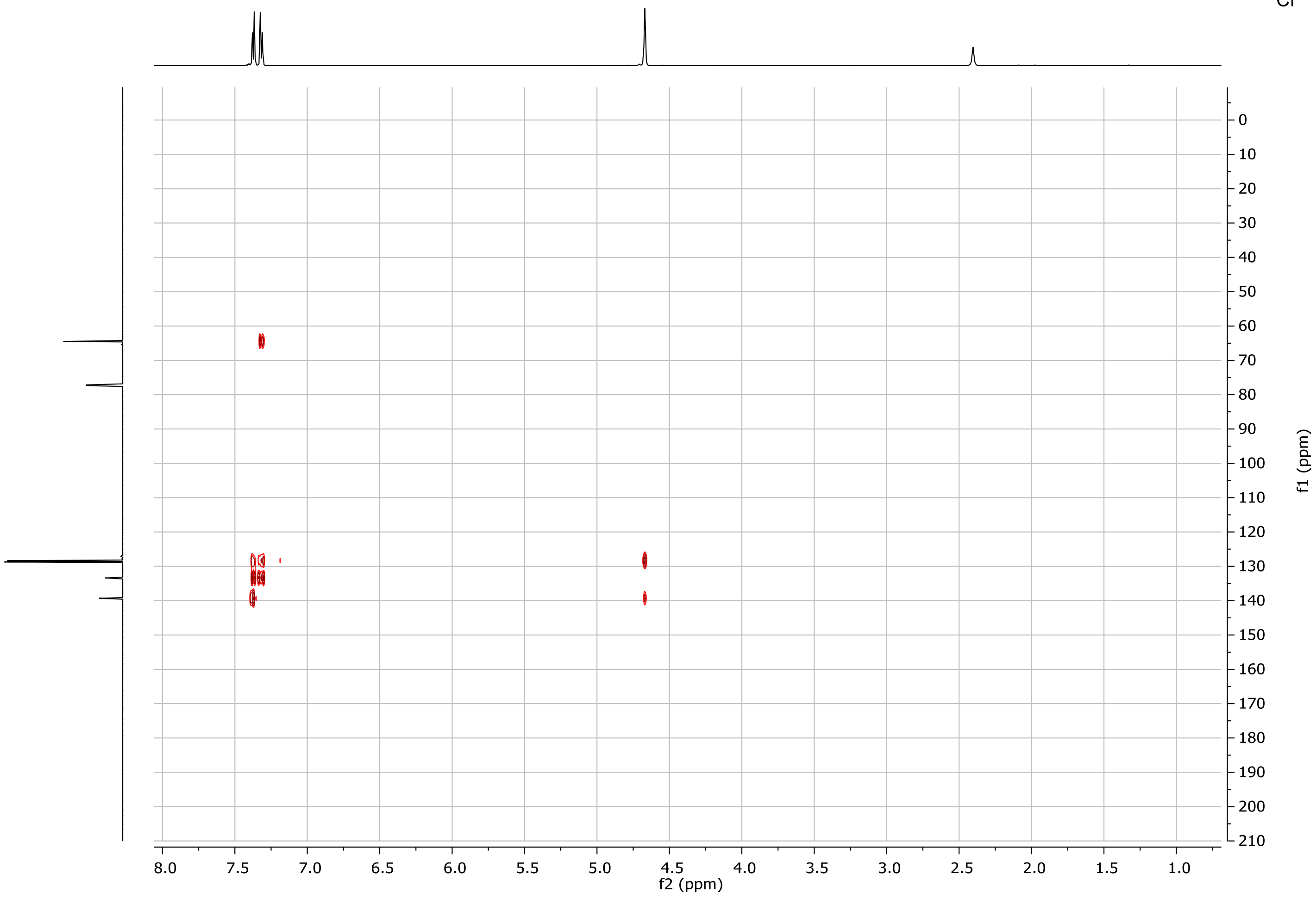

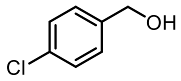

2s

^{1}H, ^{13}C HSQC

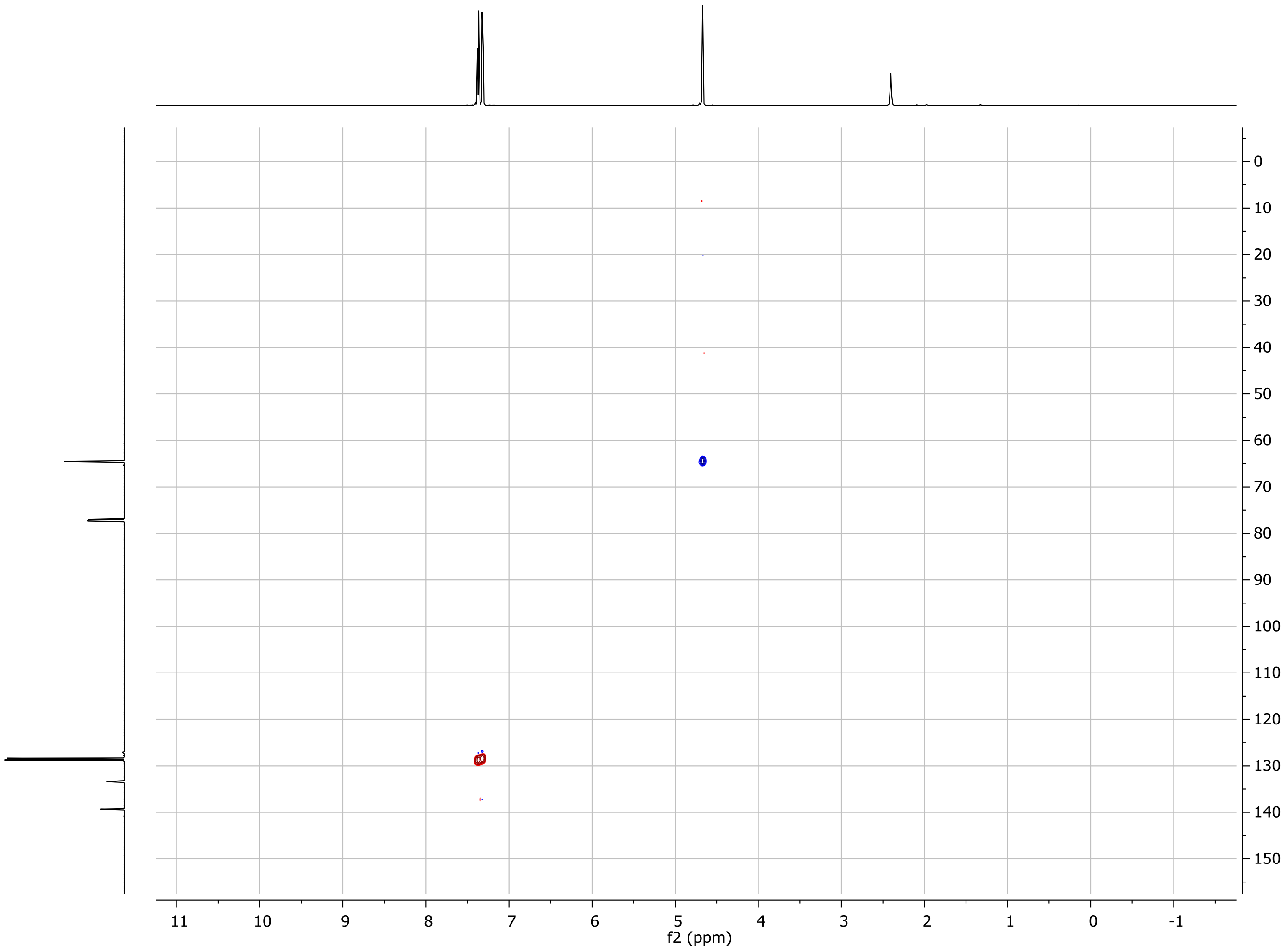

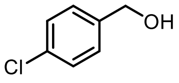

2s

HRMS

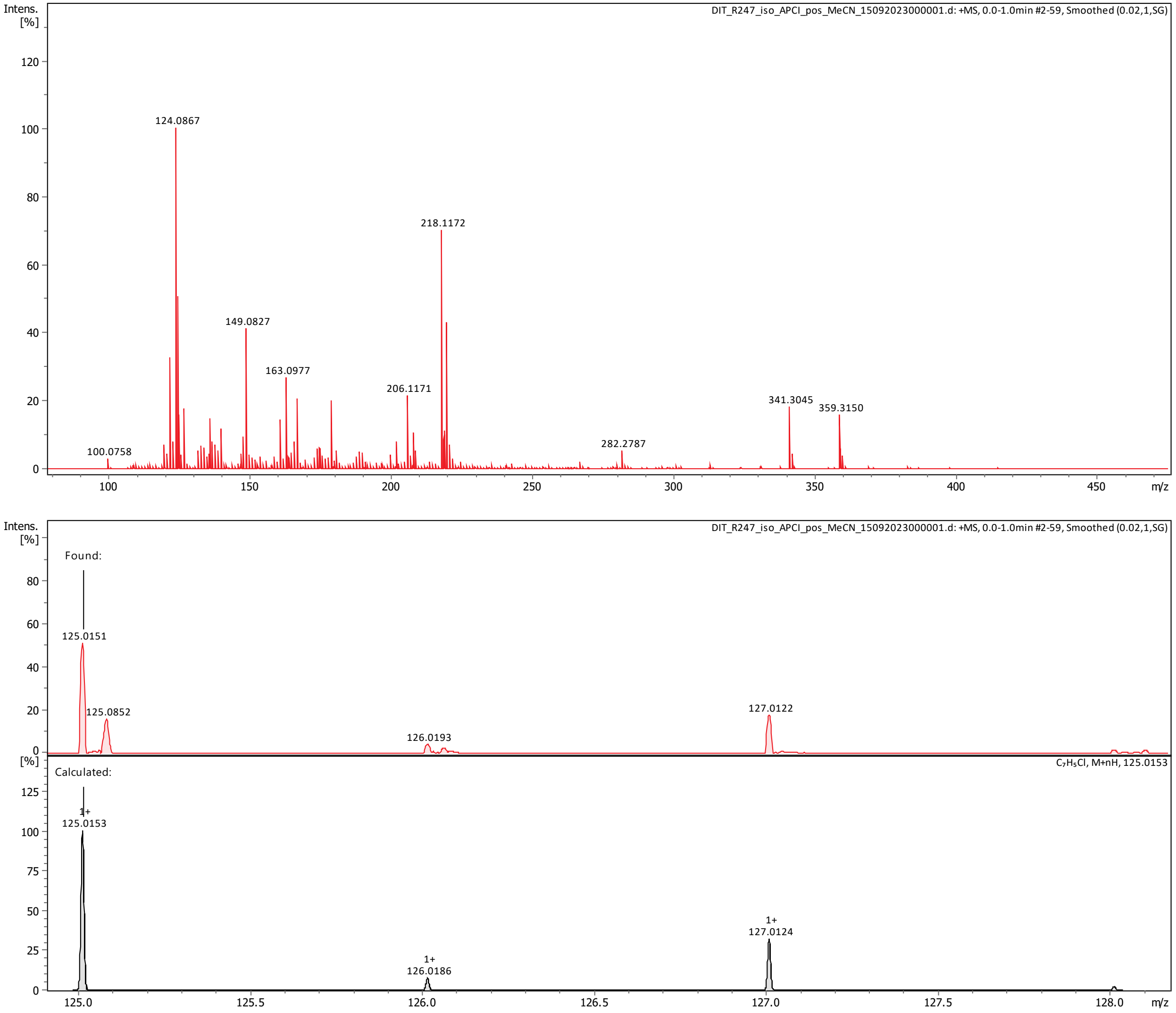

DIT_R247_iso_APCI_pos_MeCN_15092023000001.d: +MS, 0.0-1.0min #2-59, Smoothed (0.02,1,SG)
124.0867
218.1172
149.0827
163.0977
206.1171
341.3045
359.3150
282.2787
100.0758
Found:
125.0151
125.0852
126.0193
127.0122
Calculated:
C₇H₅Cl, M+nH, 125.0153
1+ 125.0153
1+ 126.0186
1+ 127.0124

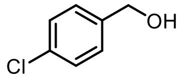

OH
Cl

2s

84 *p*-Tolylmethan-d_2-ol (d_2-2m)

^{1}H NMR

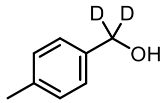

d_2-2m

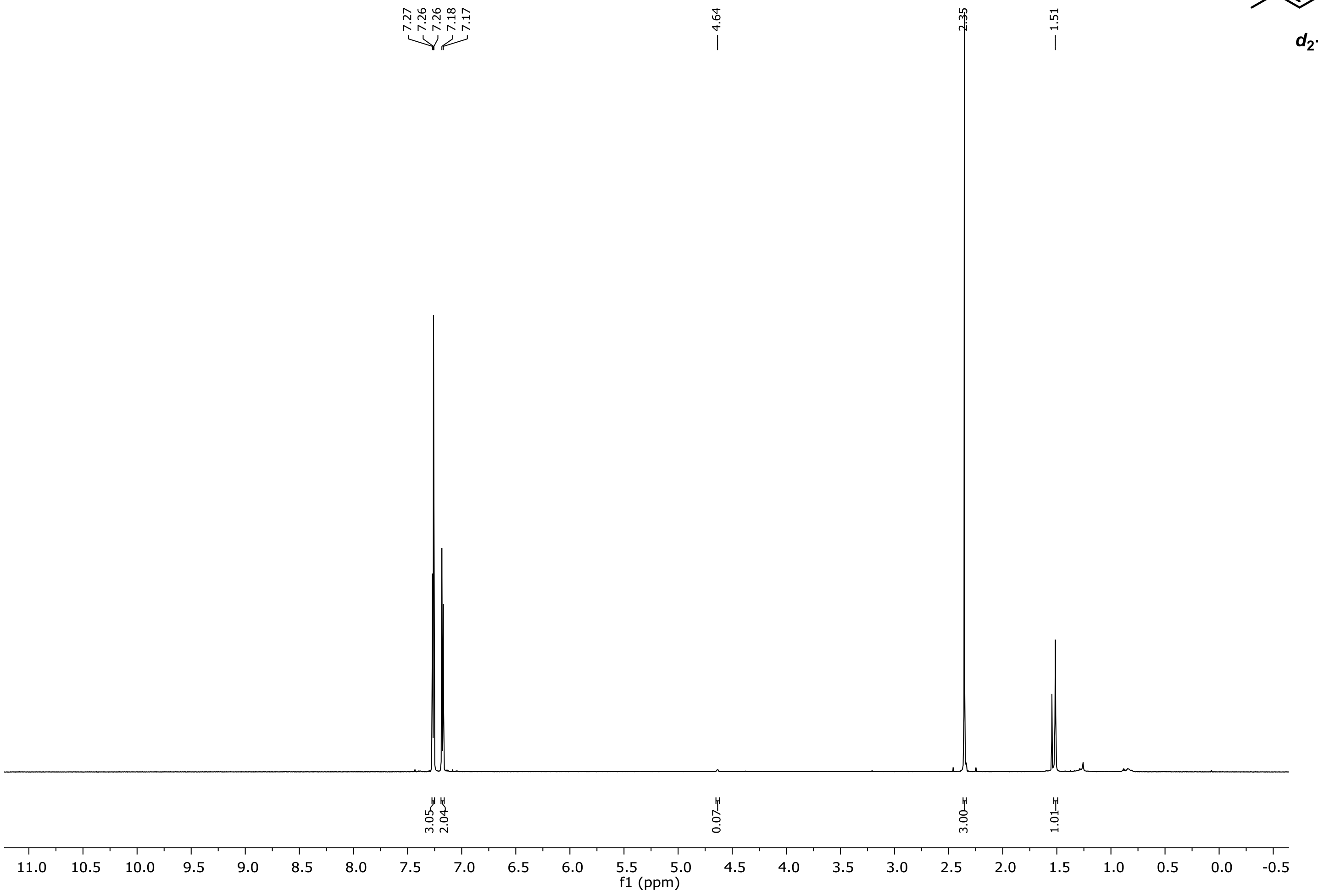

^{2}H NMR

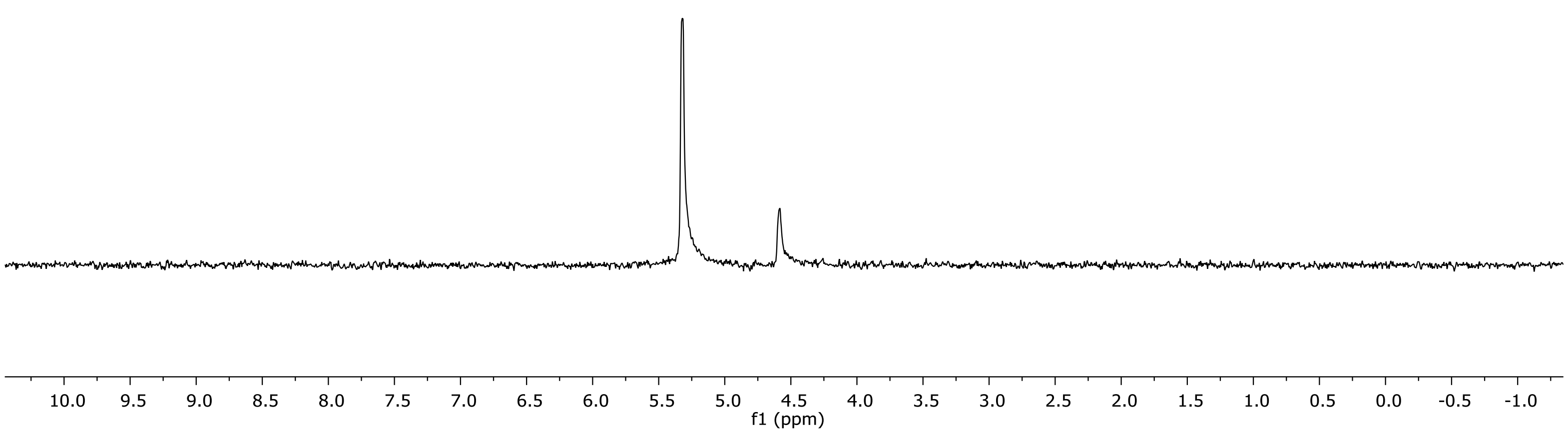
4.59
10.0
9.5
9.0
8.5
8.0
7.5
7.0
6.5
6.0
5.5
5.0
4.5
4.0
3.5
3.0
2.5
2.0
1.5
1.0
0.5
0.0
-0.5
-1.0
f1 (ppm)

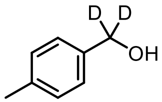
D
D
OH

***d*$_2$-2m**

^{13}C NMR

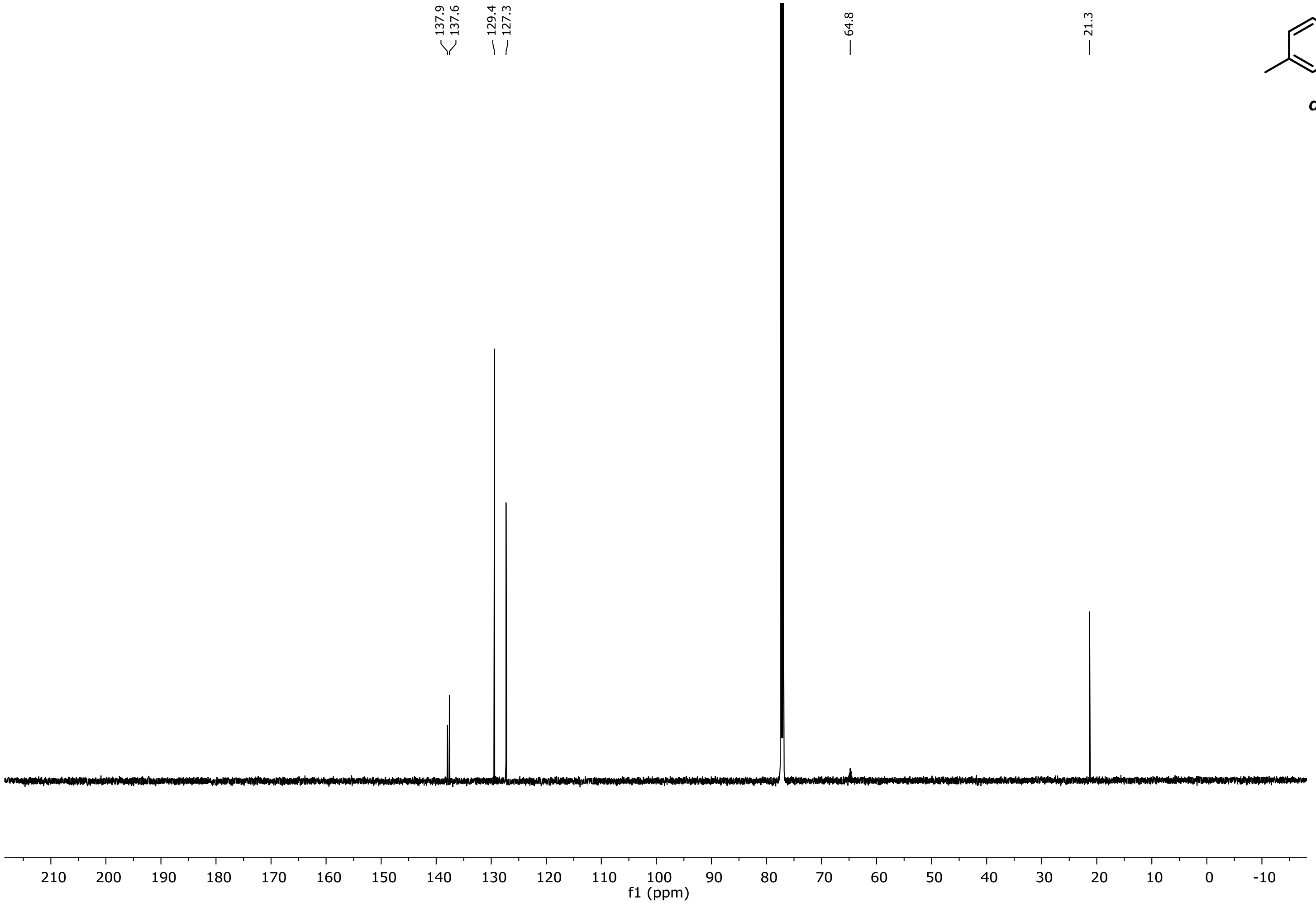

137.9
137.6
129.4
127.3
64.8
21.3
210 200 190 180 170 160 150 140 130 120 110 100 90 80 70 60 50 40 30 20 10 0 -10
f1 (ppm)

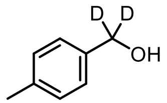

D D
OH

***d*$_2$-2m**

^{1}H, ^{1}H COSY

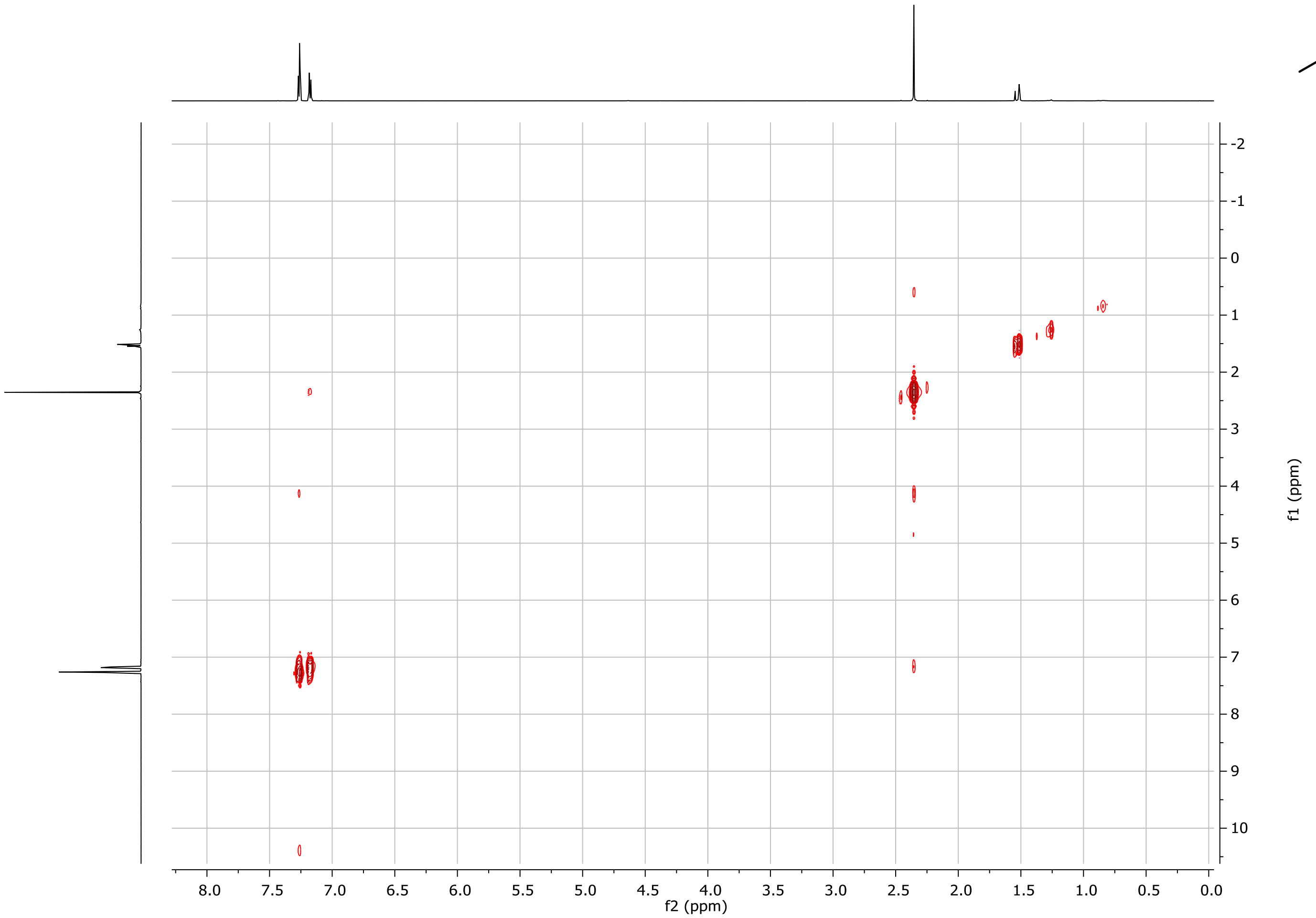

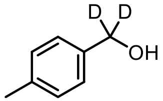

d$_2$-**2m**

^{1}H, ^{13}C HMBC

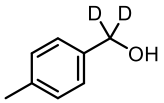

***d_2*-2m**

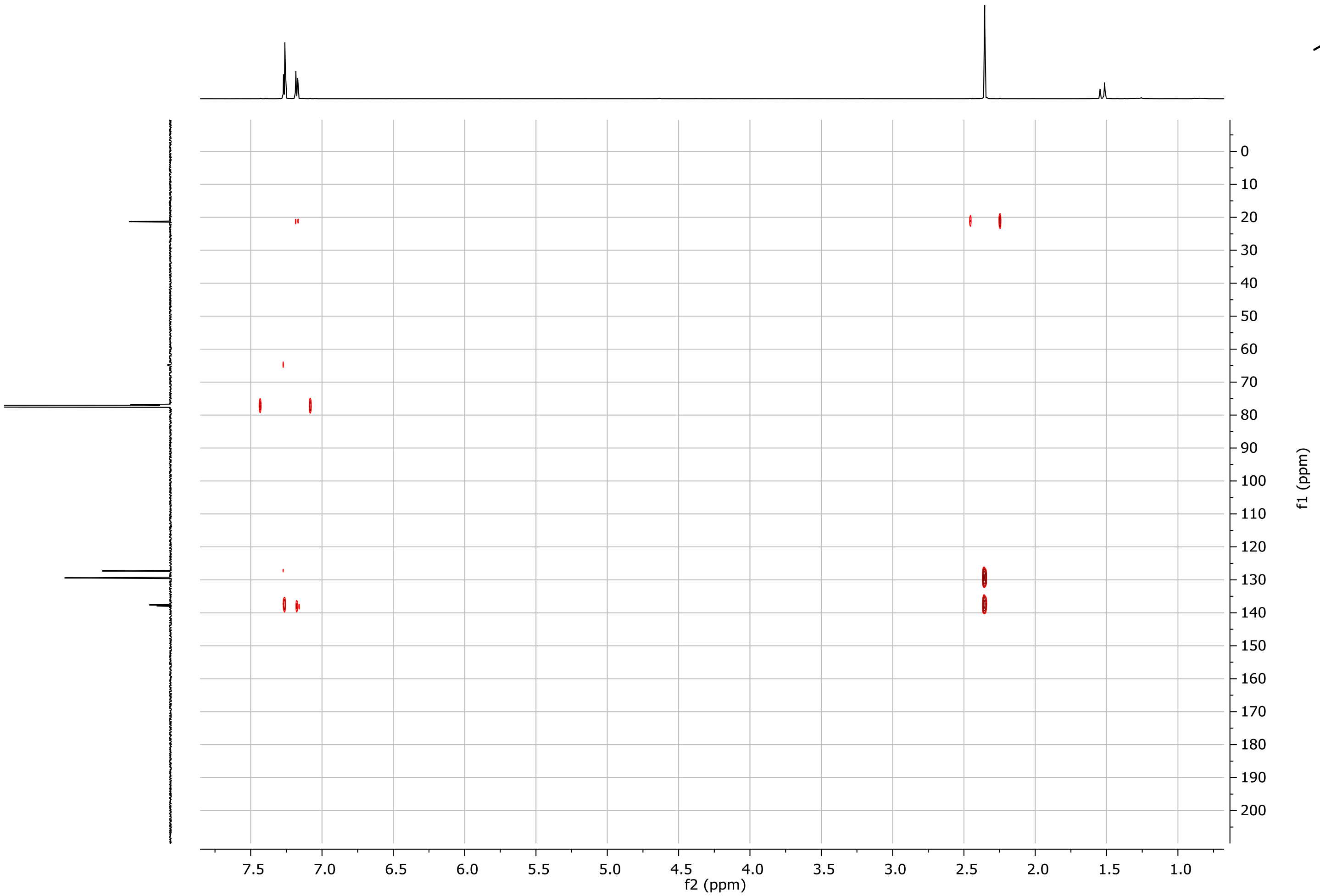

^{1}H, ^{13}C HSQC

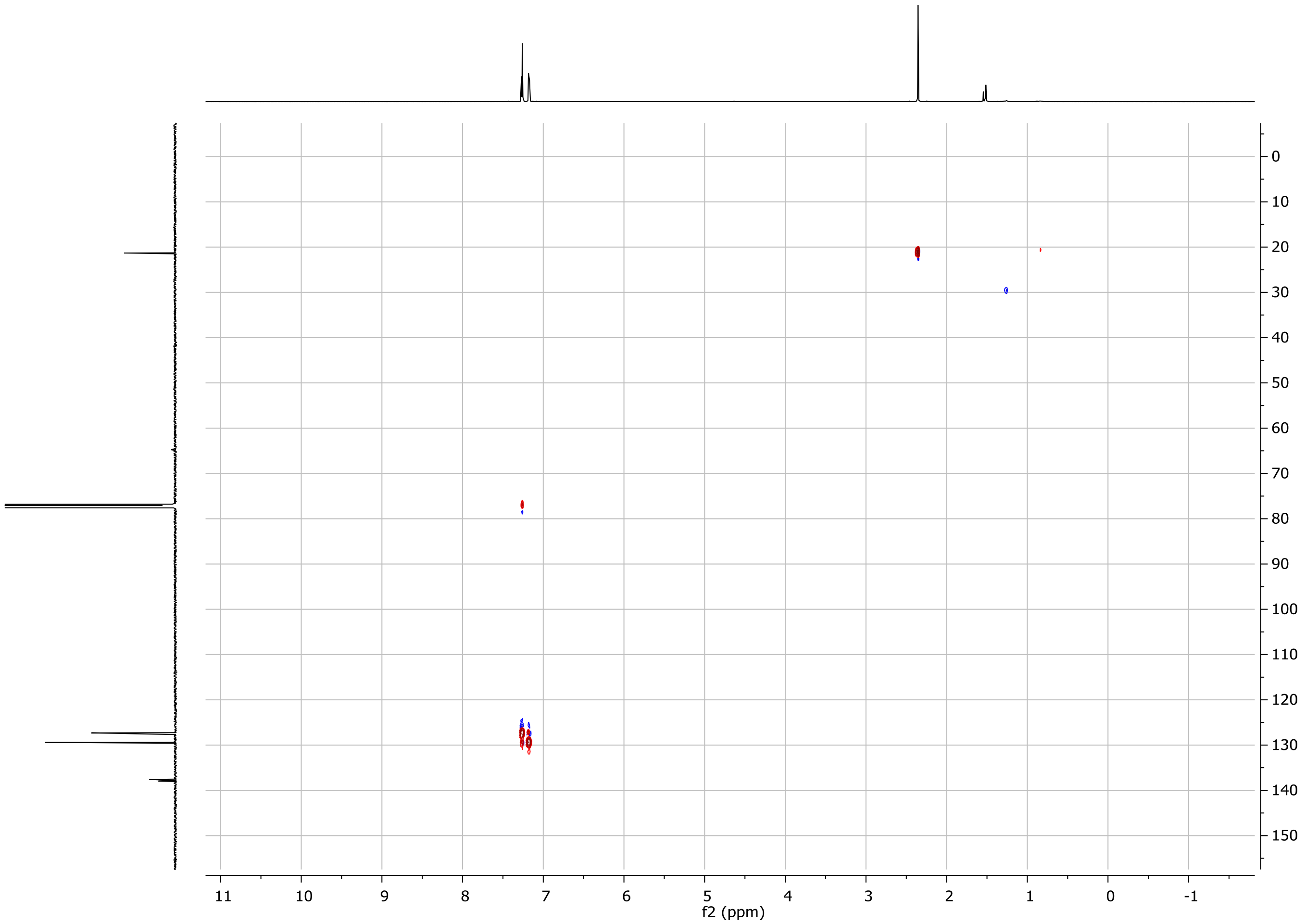

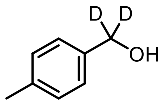

***d*$_2$-2m**

HRMS

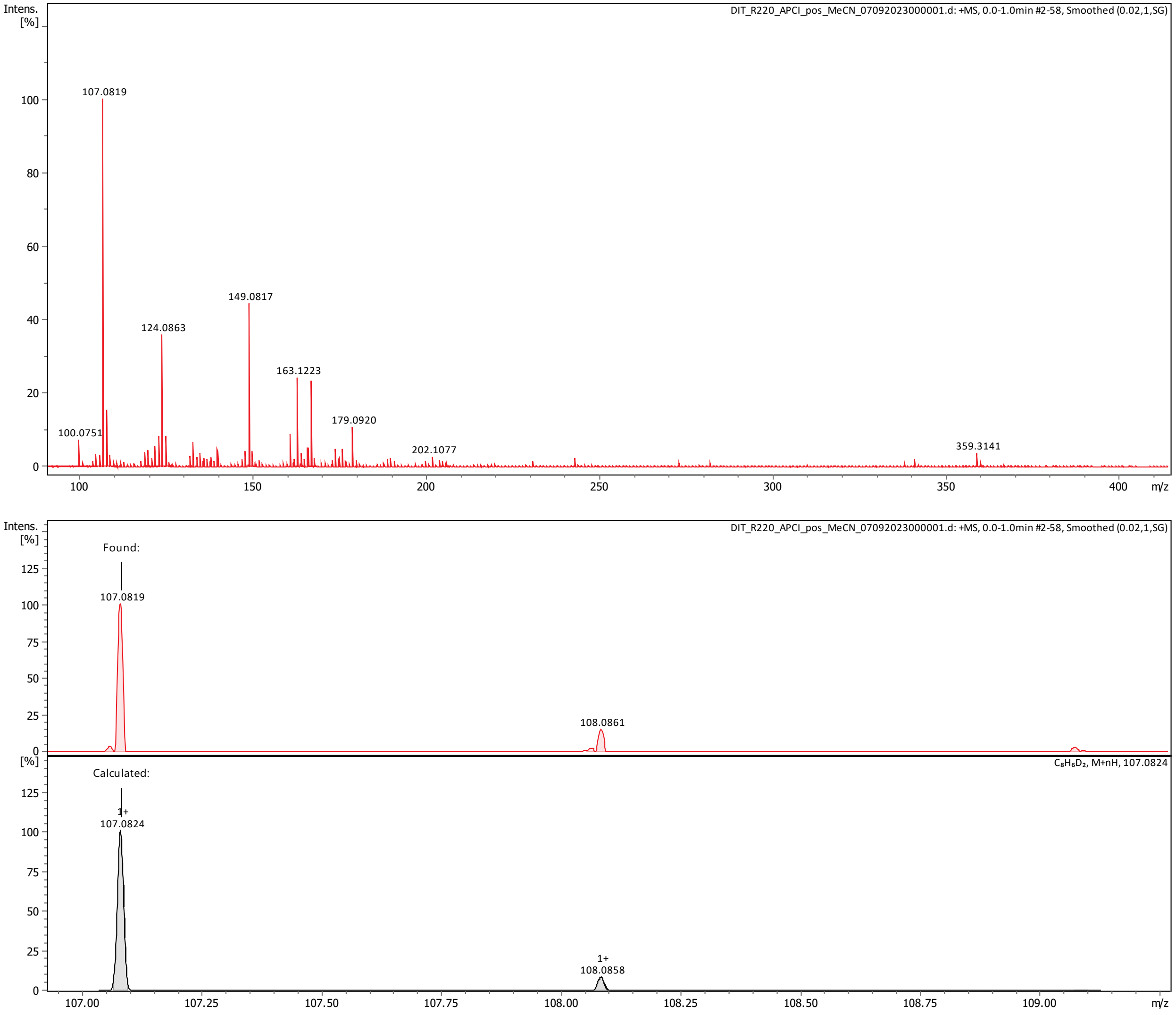

Intens. [%]
DIT_R220_APCI_pos_MeCN_07092023000001.d: +MS, 0.0-1.0min #2-58, Smoothed (0.02,1,SG)
107.0819
124.0863
149.0817
163.1223
179.0920
100.0751
202.1077
359.3141
m/z
Found:
107.0819
108.0861
Calculated:
1+
107.0824
1+
108.0858
$C_8H_6D_2$, M+nH, 107.0824

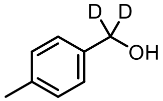

***d*$_2$-2m**

85 Cyclohexylmethan-d_3-ol (d_3-2n)

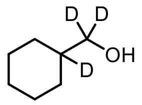

d_3-2n

^{1}H NMR

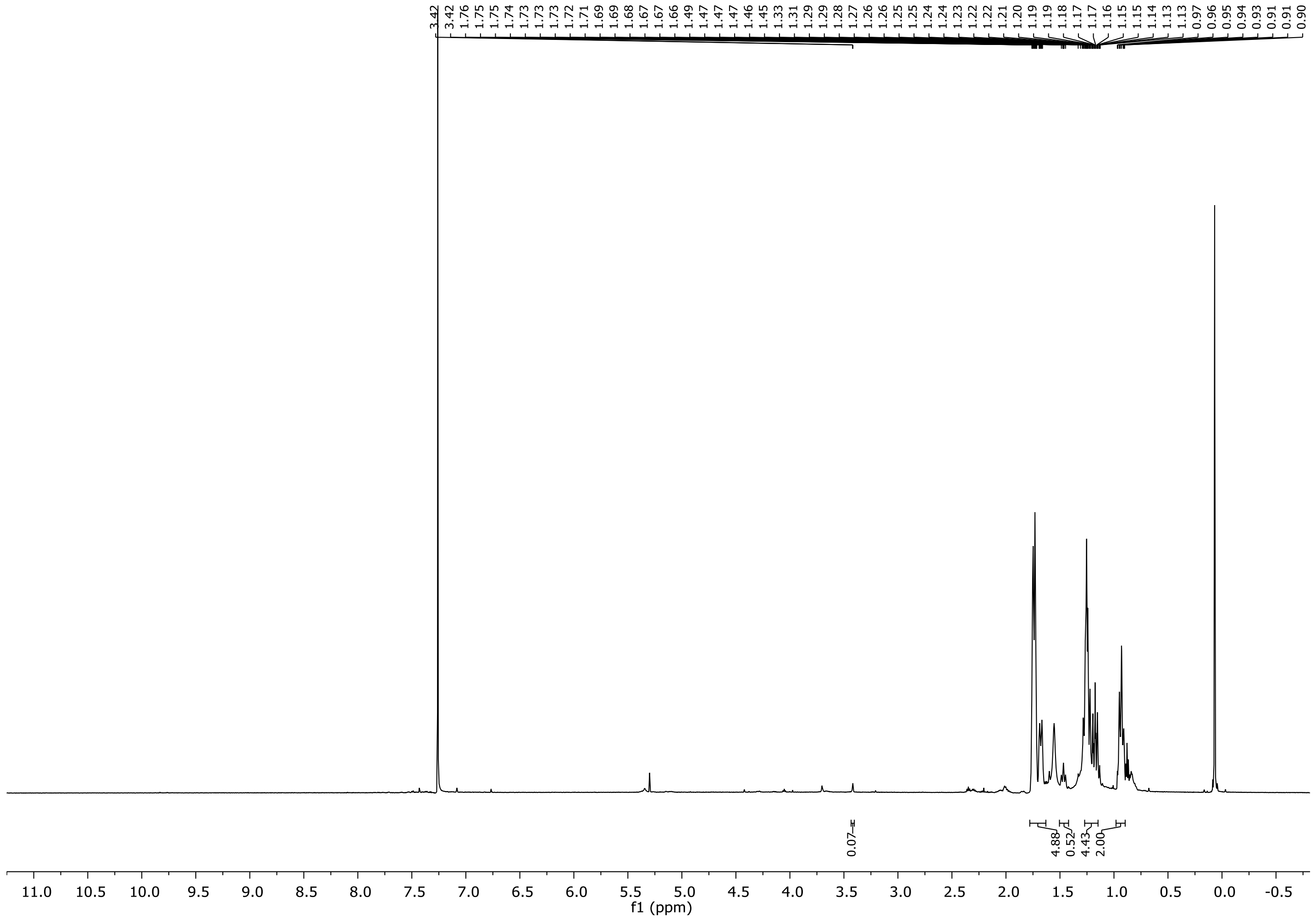

^{2}H NMR

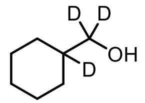

d_3-2n

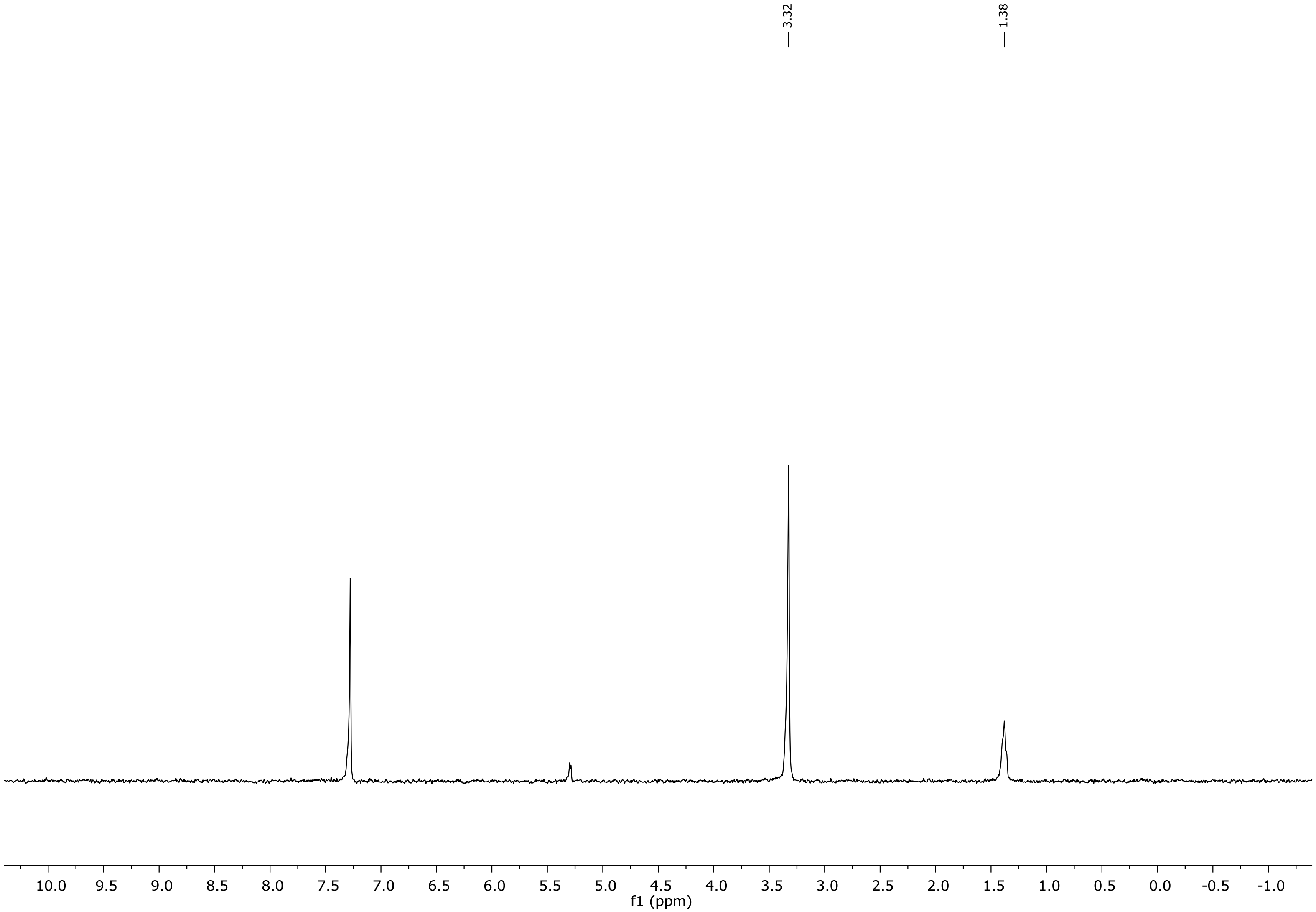
3.32
1.38
10.0 9.5 9.0 8.5 8.0 7.5 7.0 6.5 6.0 5.5 5.0 4.5 4.0 3.5 3.0 2.5 2.0 1.5 1.0 0.5 0.0 -0.5 -1.0
f1 (ppm)

^{13}C NMR

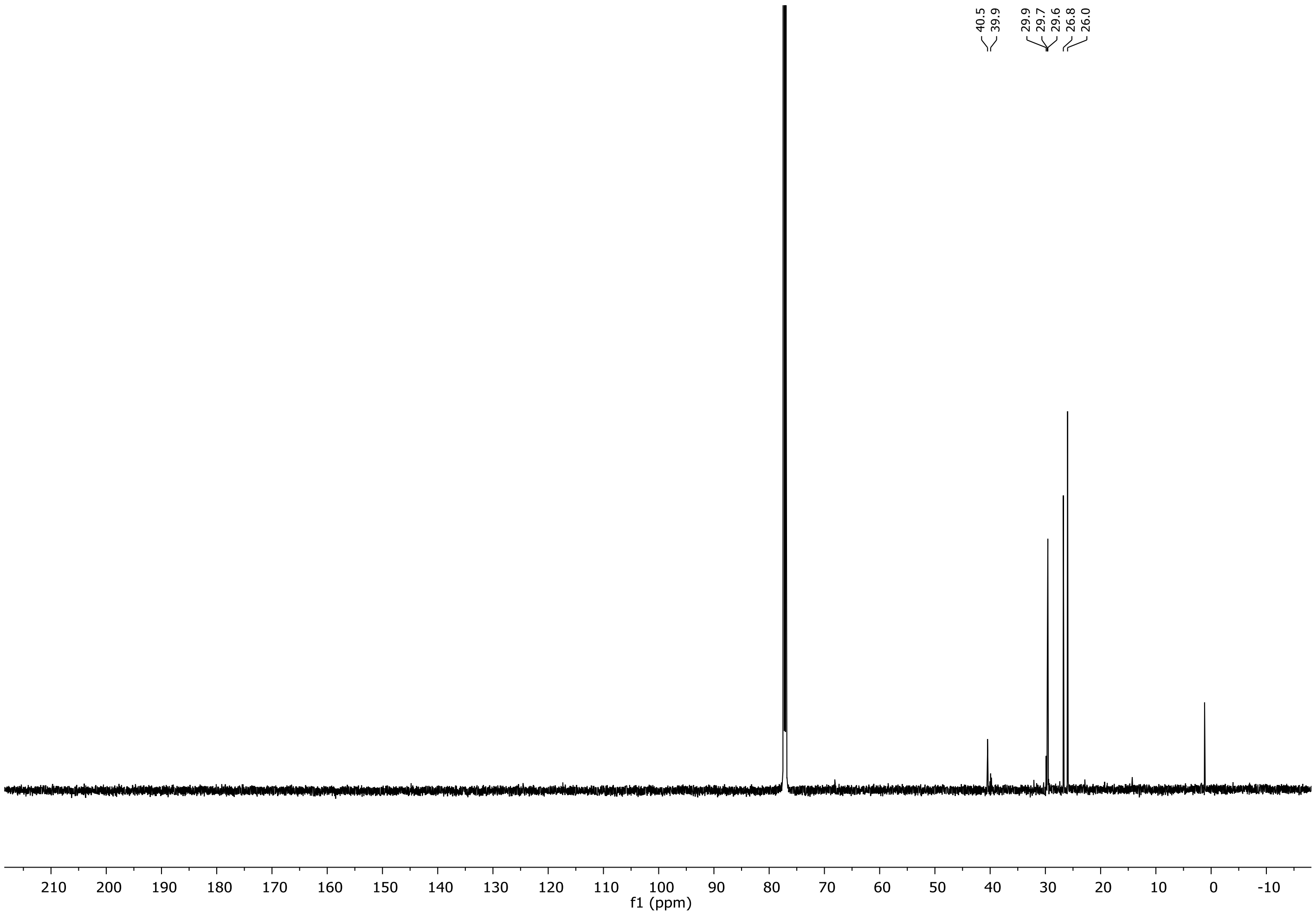

40.5
39.9
29.9
29.7
29.6
26.8
26.0
210
200
190
180
170
160
150
140
130
120
110
100
90
80
70
60
50
40
30
20
10
0
-10
f1 (ppm)

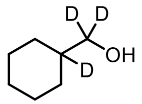

D
D
OH
D

***d*$_3$-2n**

^{1}H, ^{1}H COSY

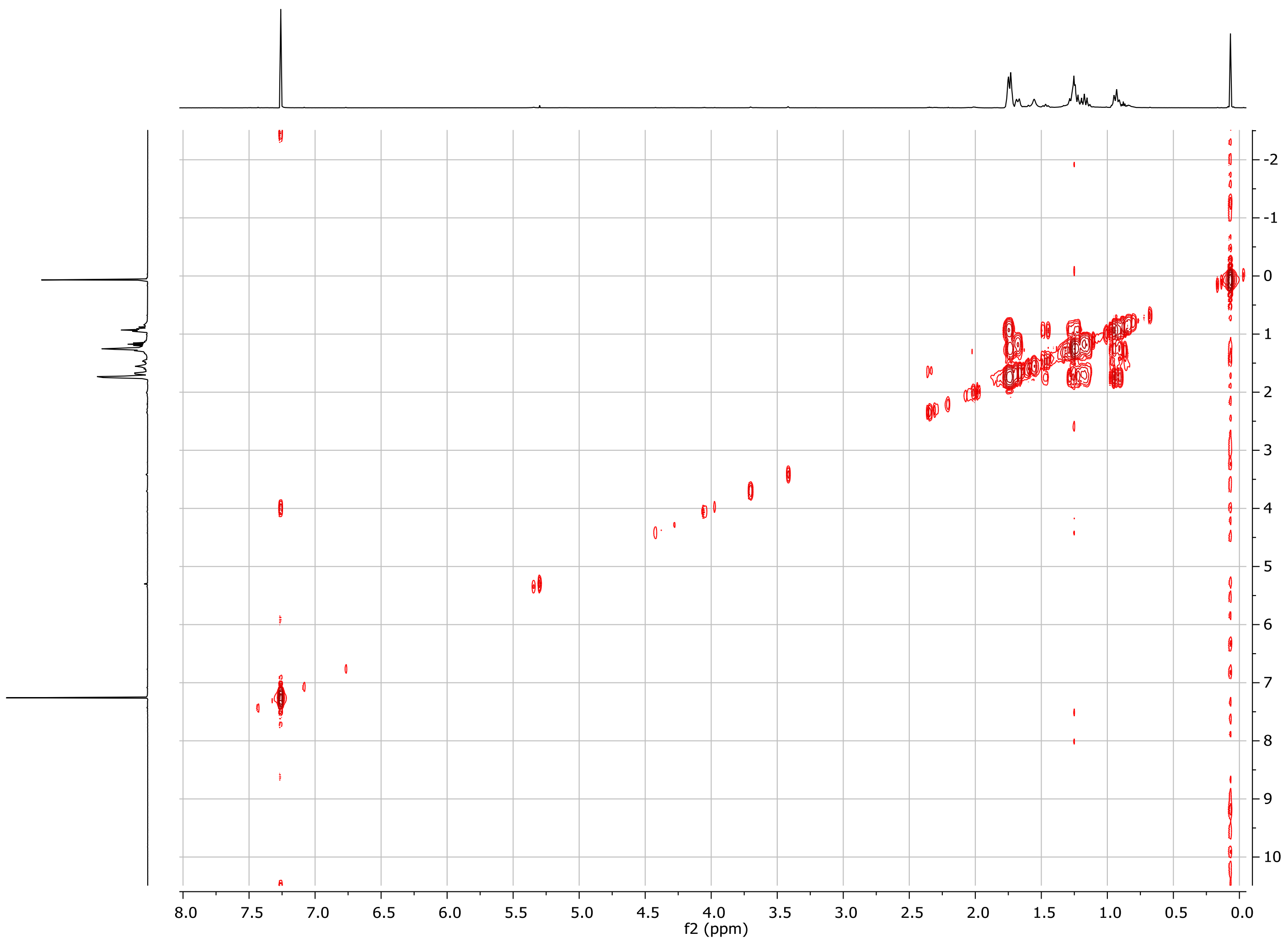

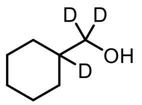

d_3-**2n**

^{1}H, ^{13}C HMBC

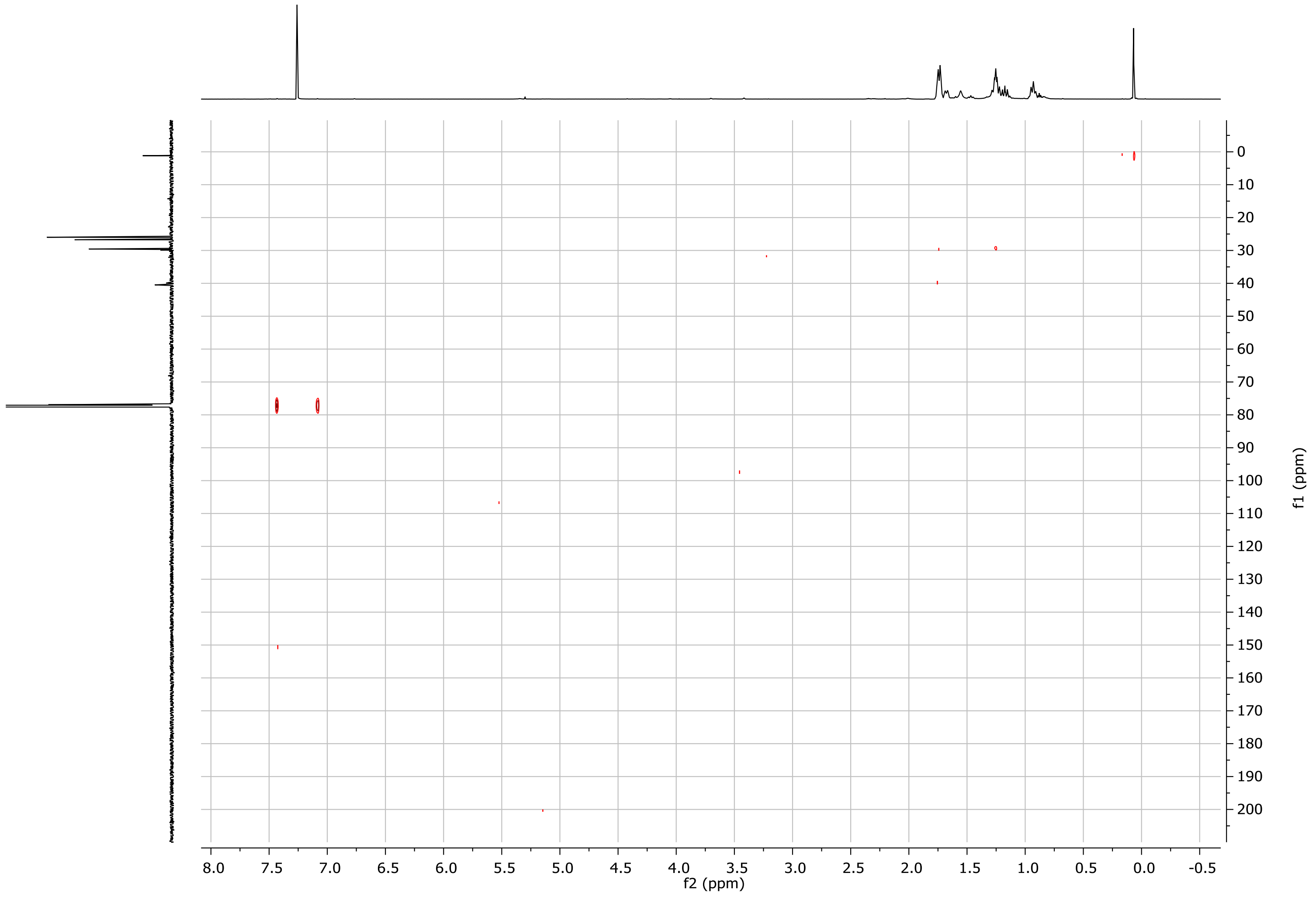

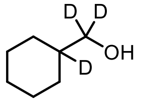

d_3-2n

^{1}H, ^{13}C HSQC

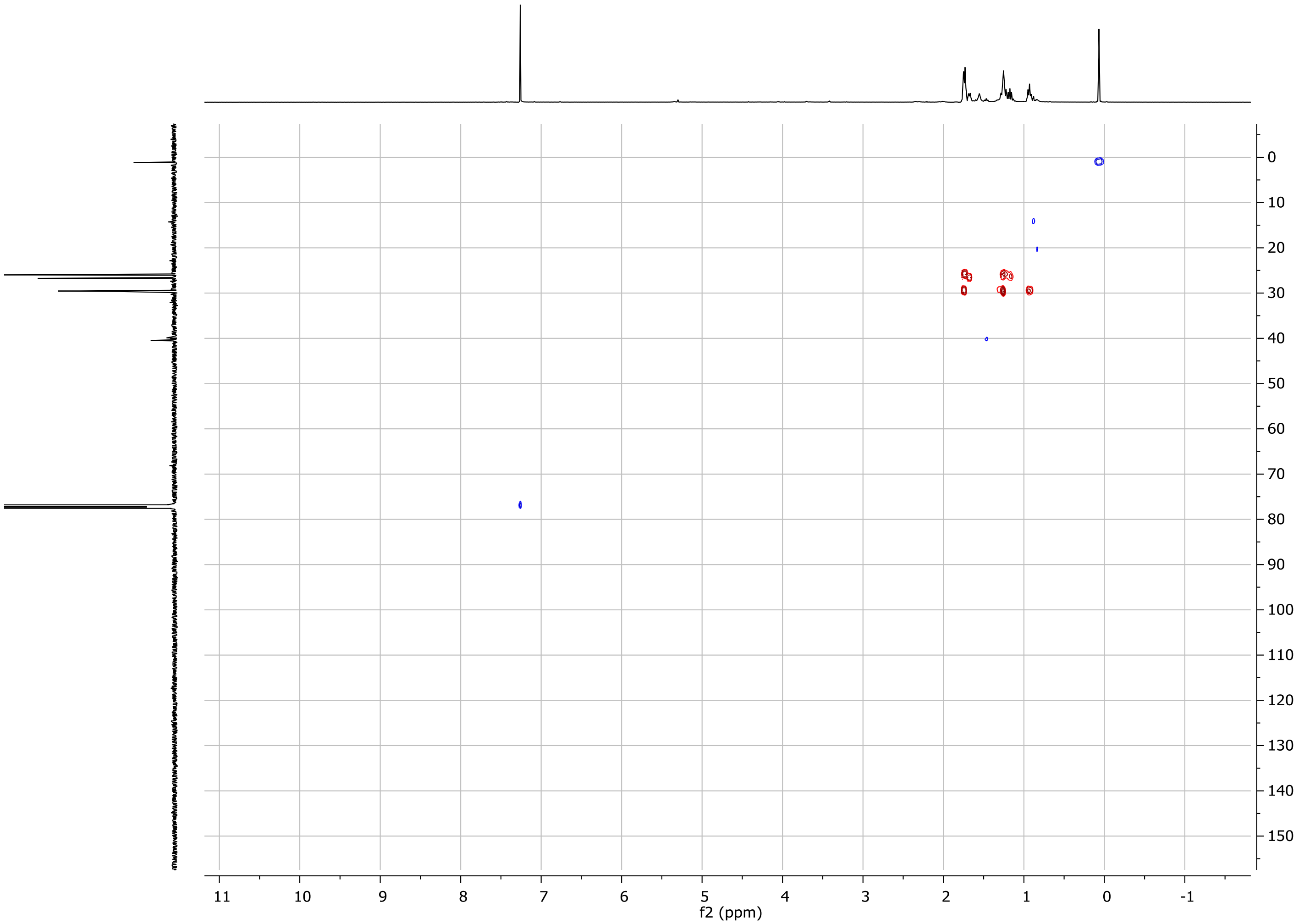

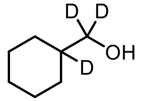

d$_3$-**2n**

HRMS

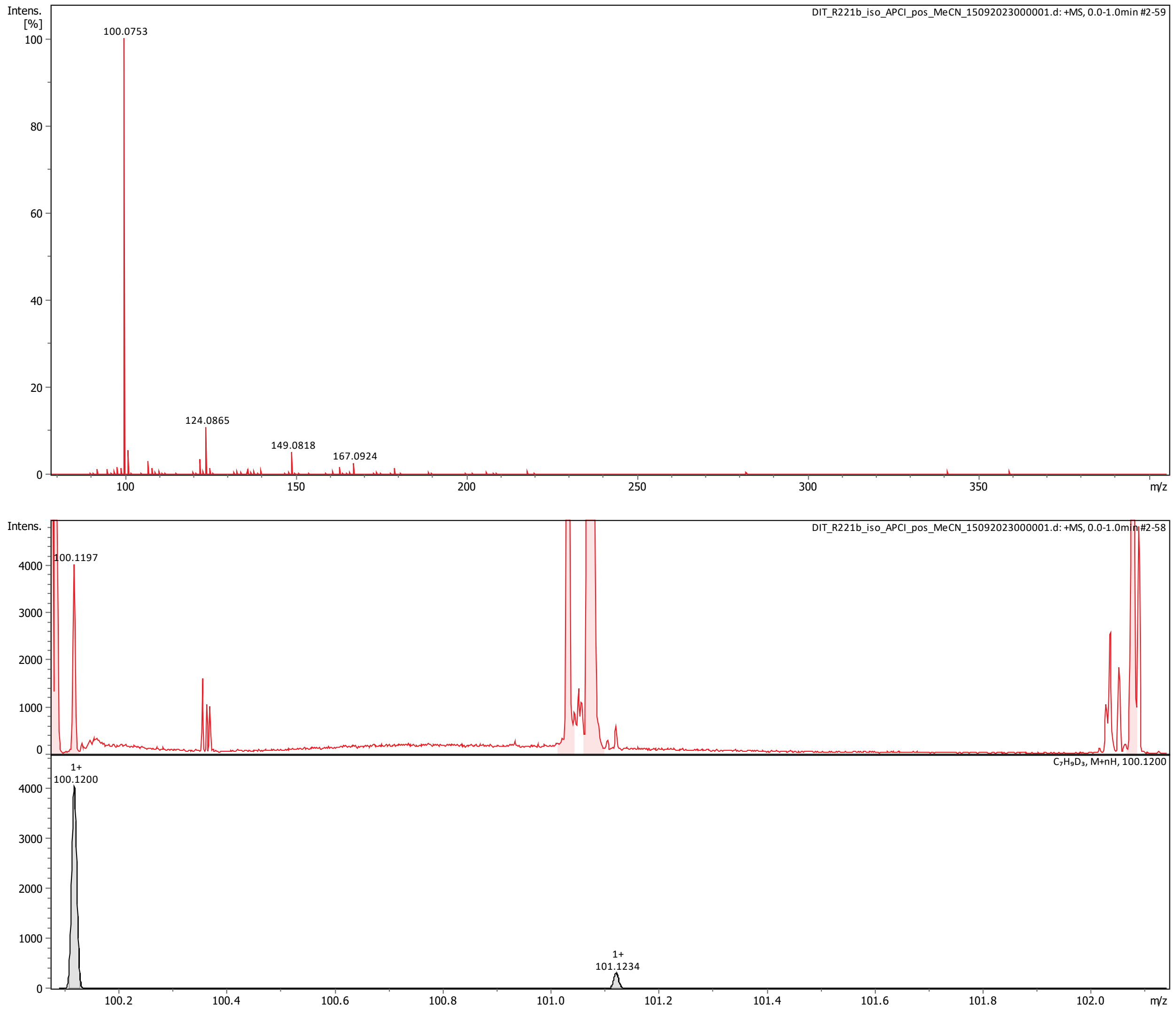

Intens.
[%]
DIT_R221b_iso_APCI_pos_MeCN_15092023000001.d: +MS, 0.0-1.0min #2-59
100.0753
124.0865
149.0818
167.0924
m/z
Intens.
DIT_R221b_iso_APCI_pos_MeCN_15092023000001.d: +MS, 0.0-1.0min #2-58
100.1197
C7H9D3, M+nH, 100.1200
1+
100.1200
1+
101.1234
m/z

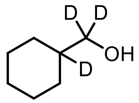

D
D
OH
D

d$_3$-**2n**

86 Methyl 4-(methylcarbamoyl)benzoate (S73)

^{1}H NMR

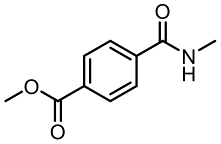

S73

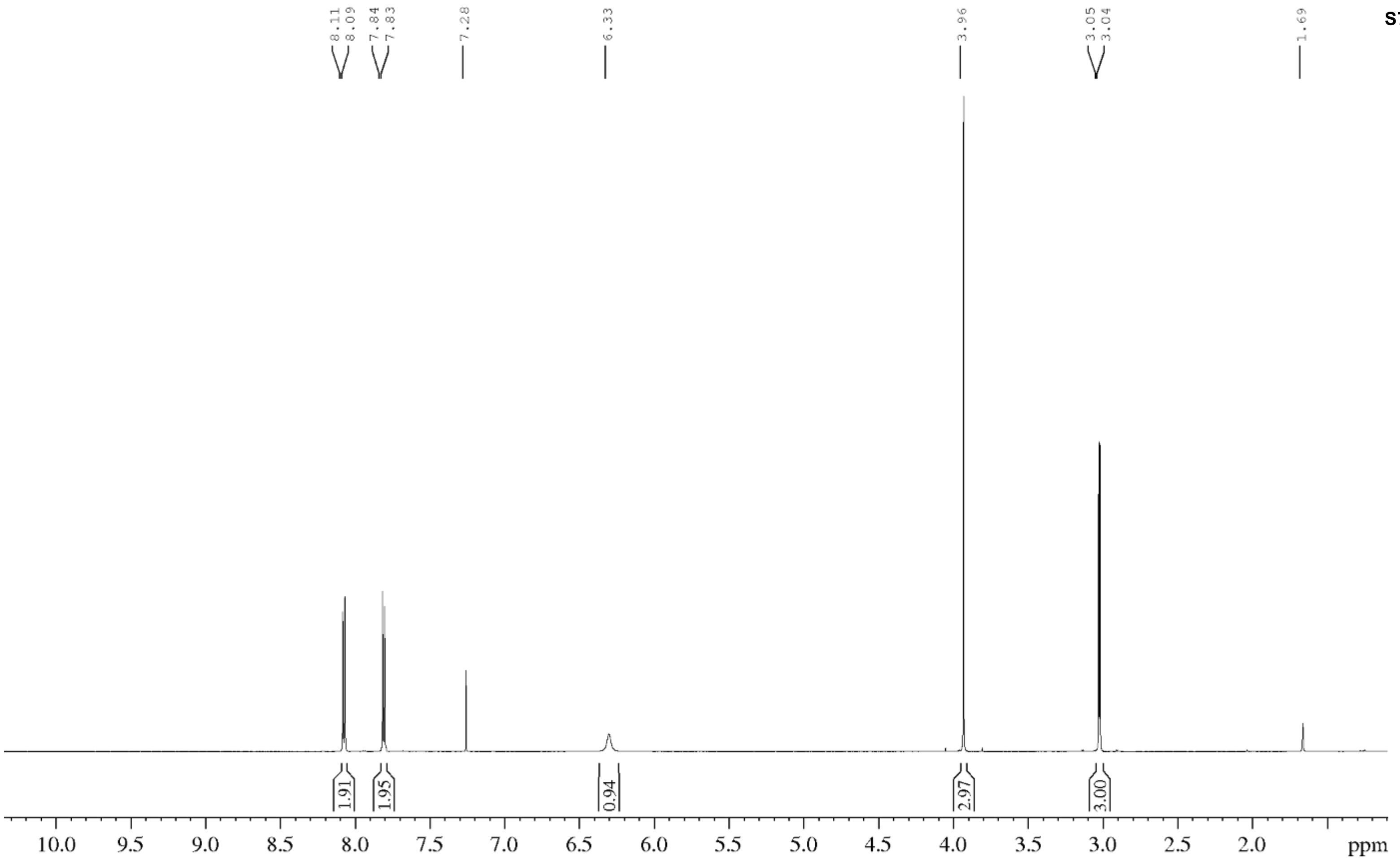

^{13}C NMR

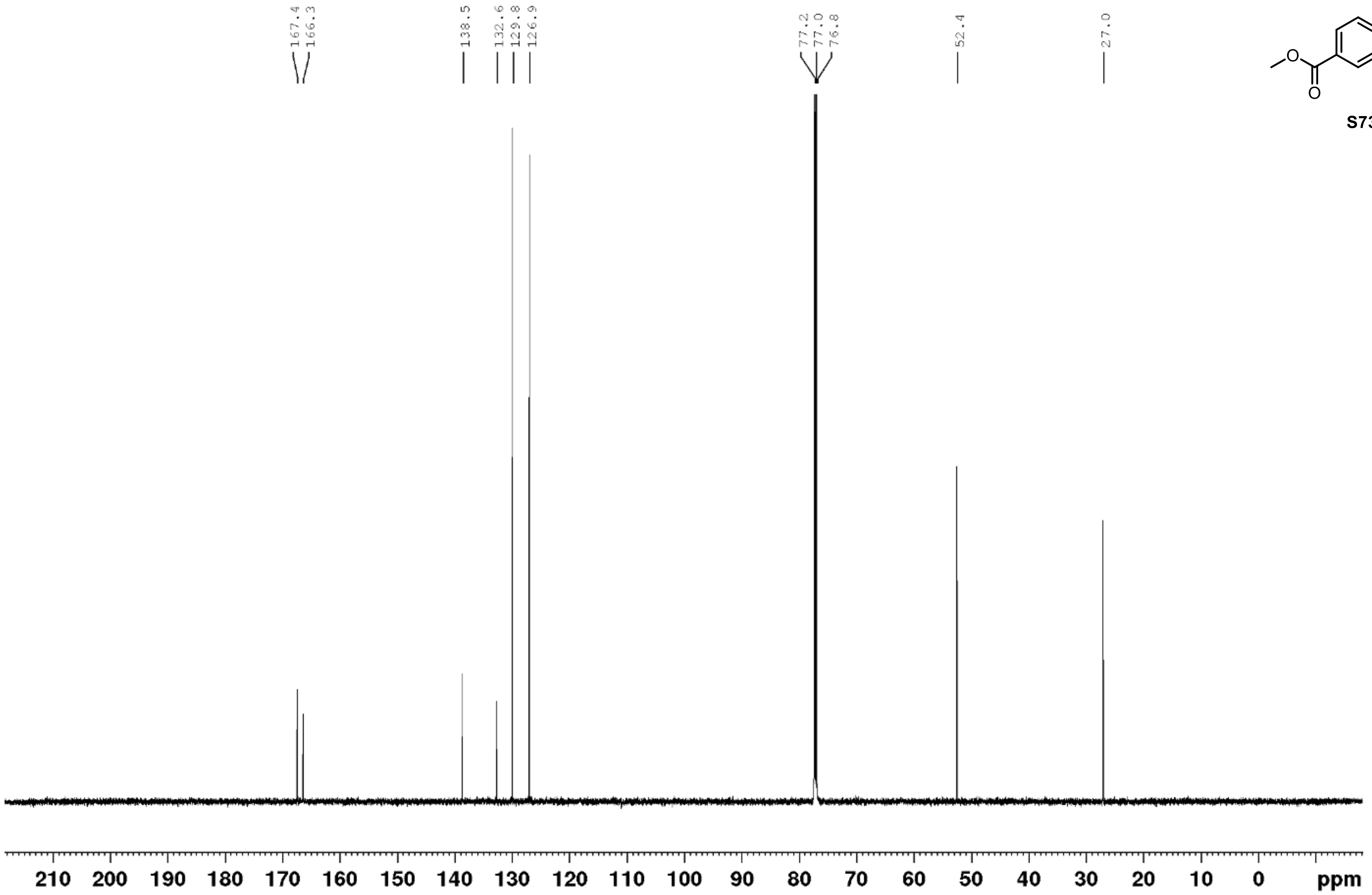
167.4
166.3
138.5
132.6
129.8
126.9
77.2
77.0
76.8
52.4
27.0
210 200 190 180 170 160 150 140 130 120 110 100 90 80 70 60 50 40 30 20 10 0 ppm

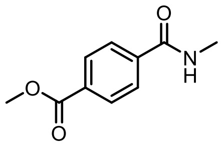
O
O
O
N
H

S73

^{1}H, ^{1}H COSY

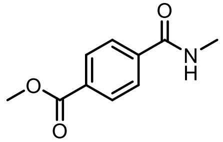

S73

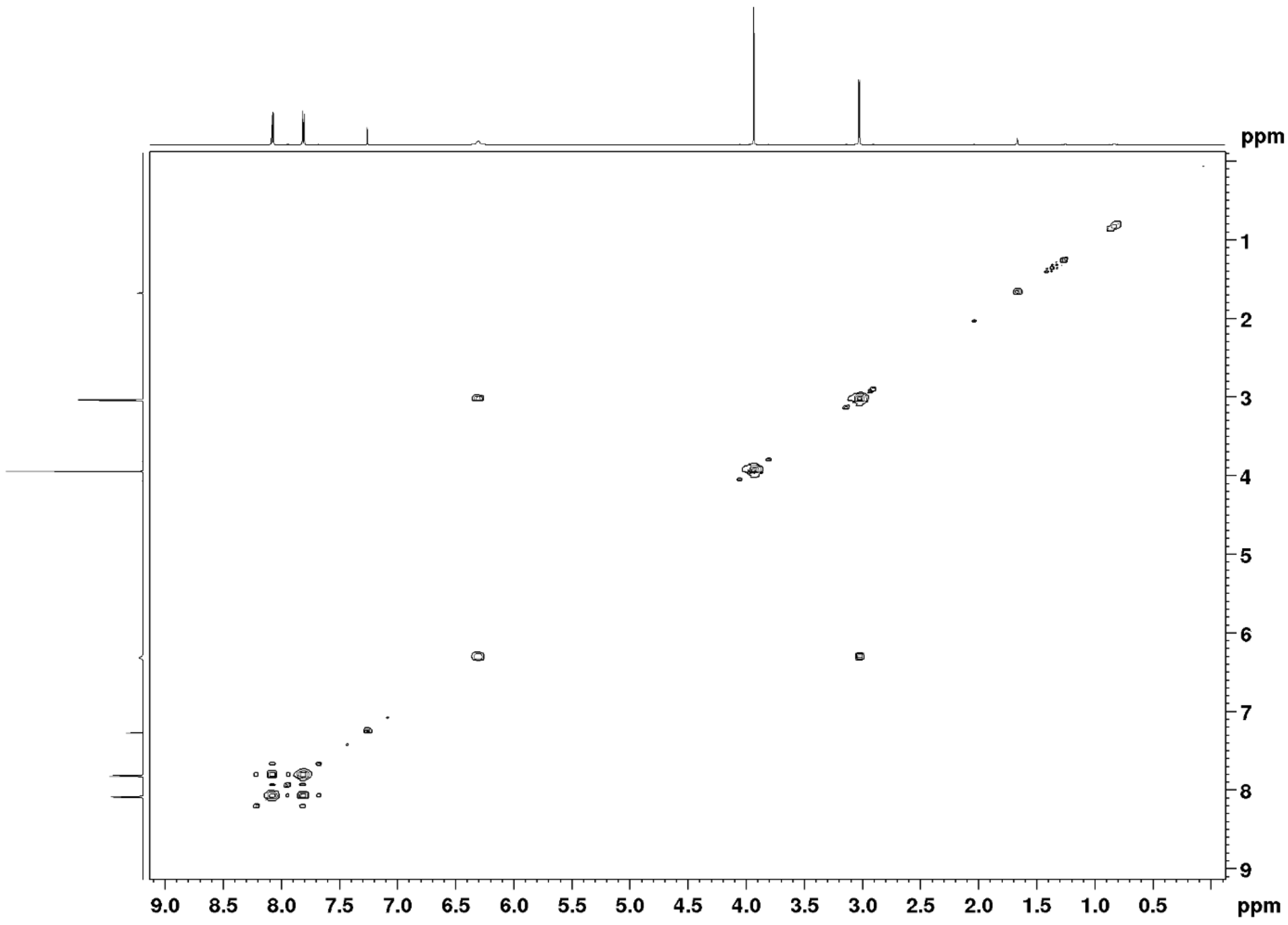

^{1}H, ^{13}C HMBC

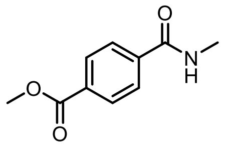

S73

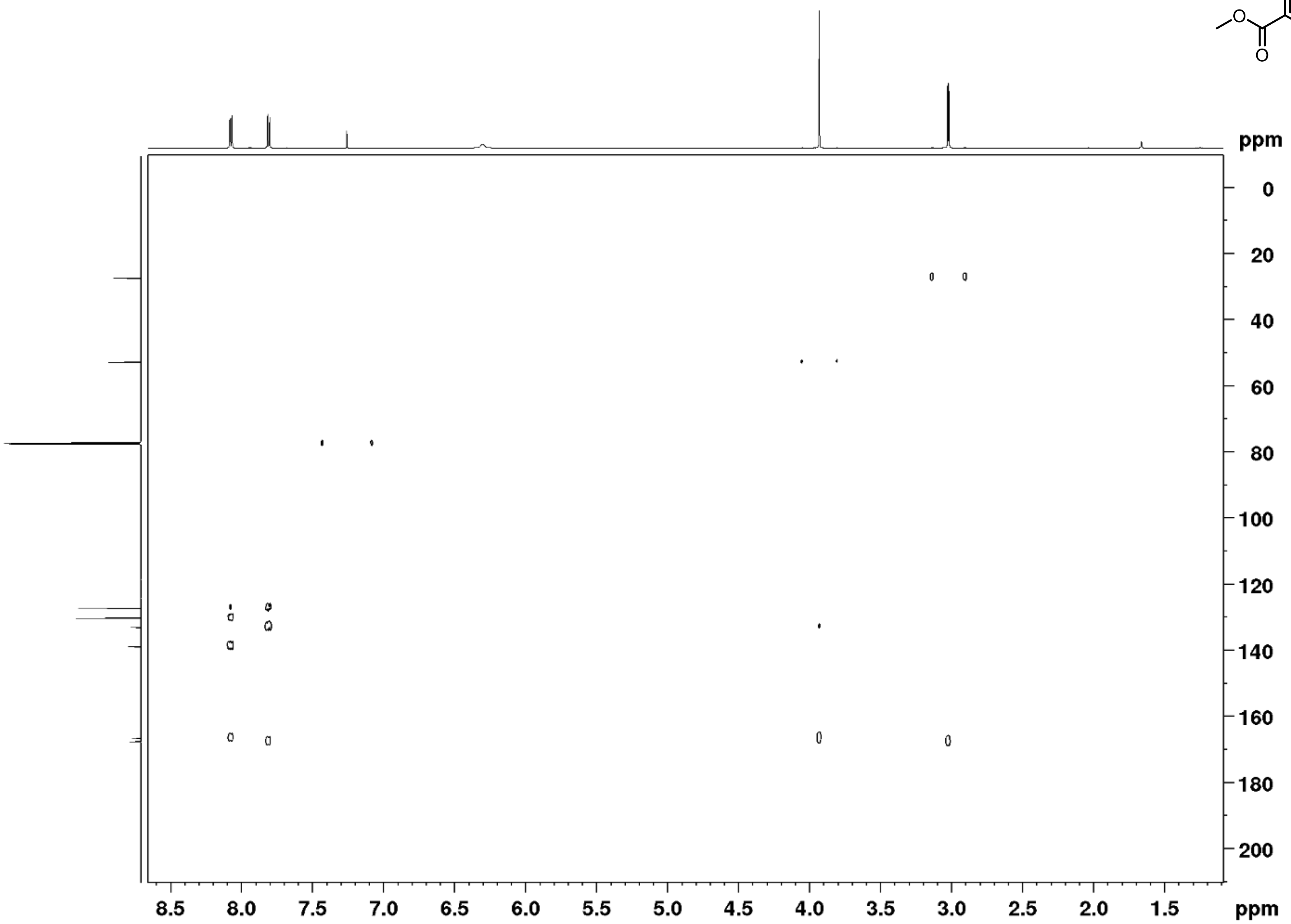

^{1}H, ^{13}C HSQC

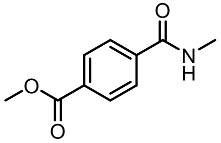

S73

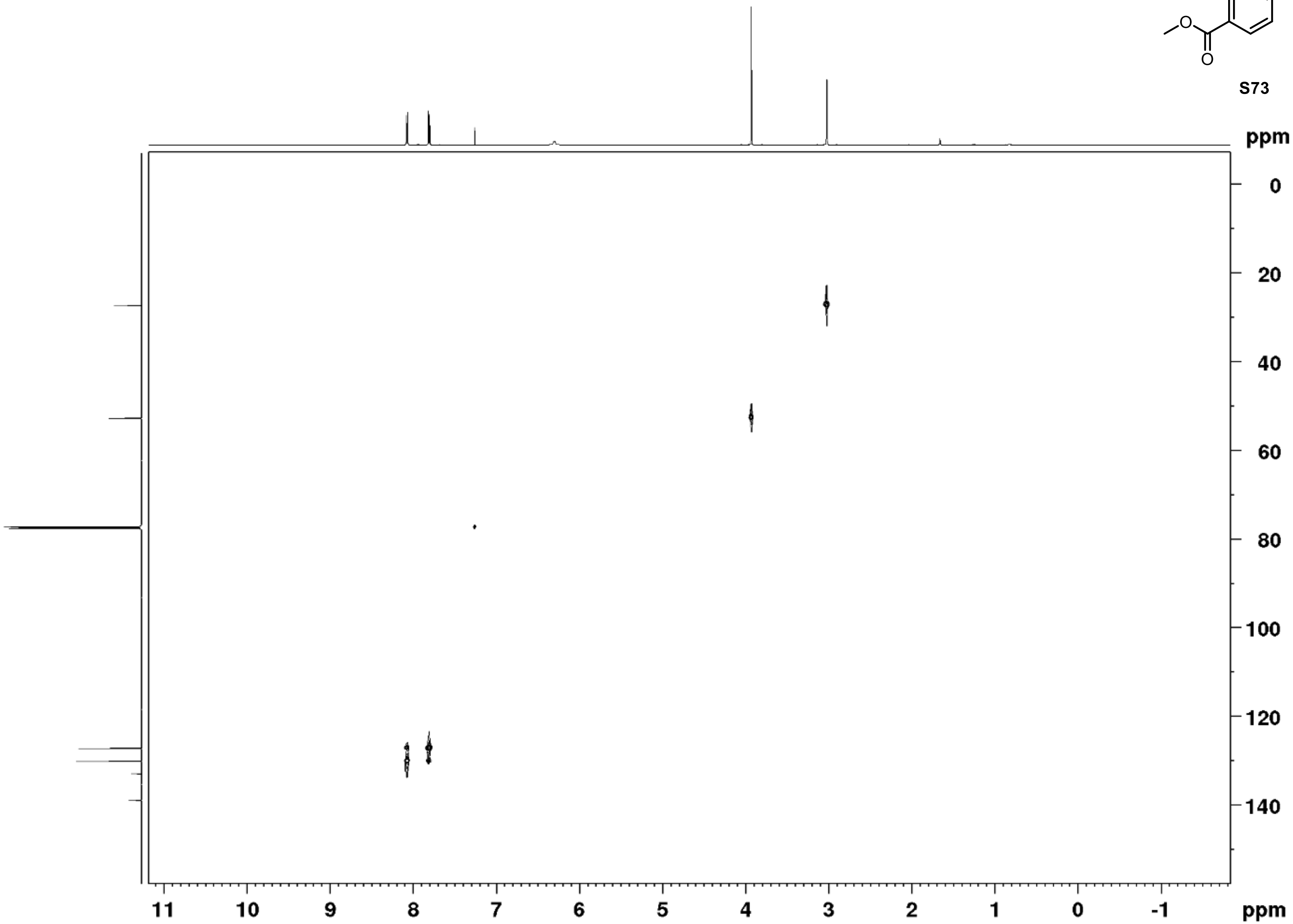

HRMS

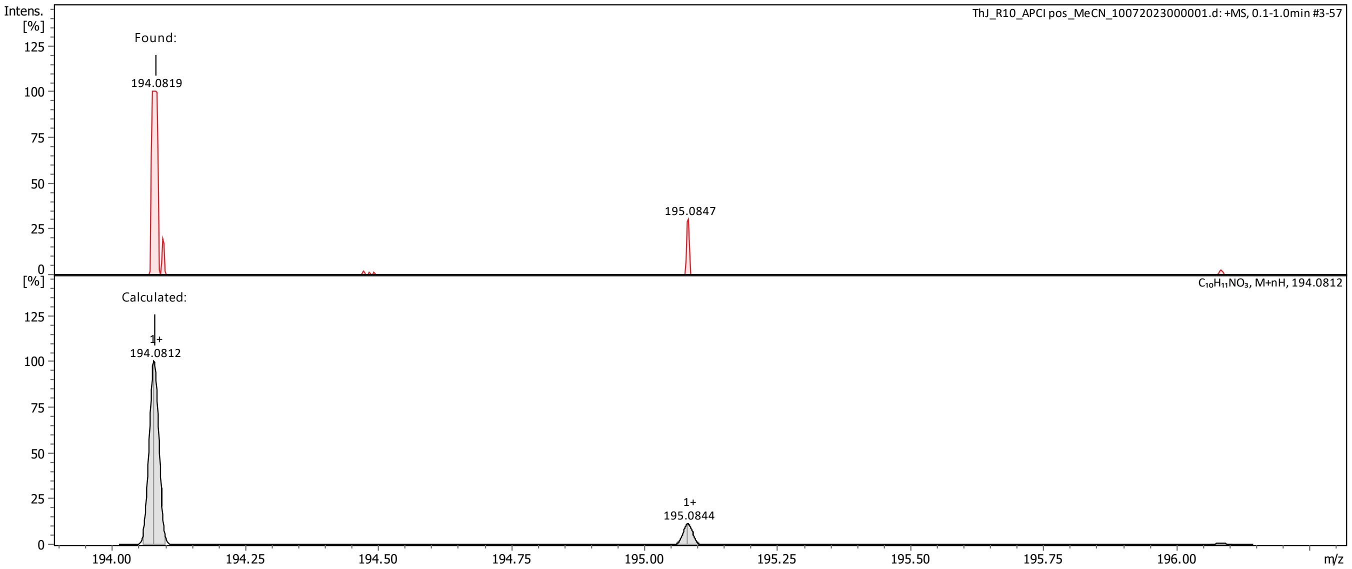

Intens. [%]
ThJ_R10_APCI pos_MeCN_10072023000001.d: +MS, 0.1-1.0min #3-57
Found:
194.0819
195.0847
C10H11NO3, M+nH, 194.0812
Calculated:
1+
194.0812
1+
195.0844
194.00
194.25
194.50
194.75
195.00
195.25
195.50
195.75
196.00
m/z

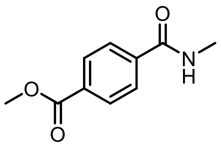

S73

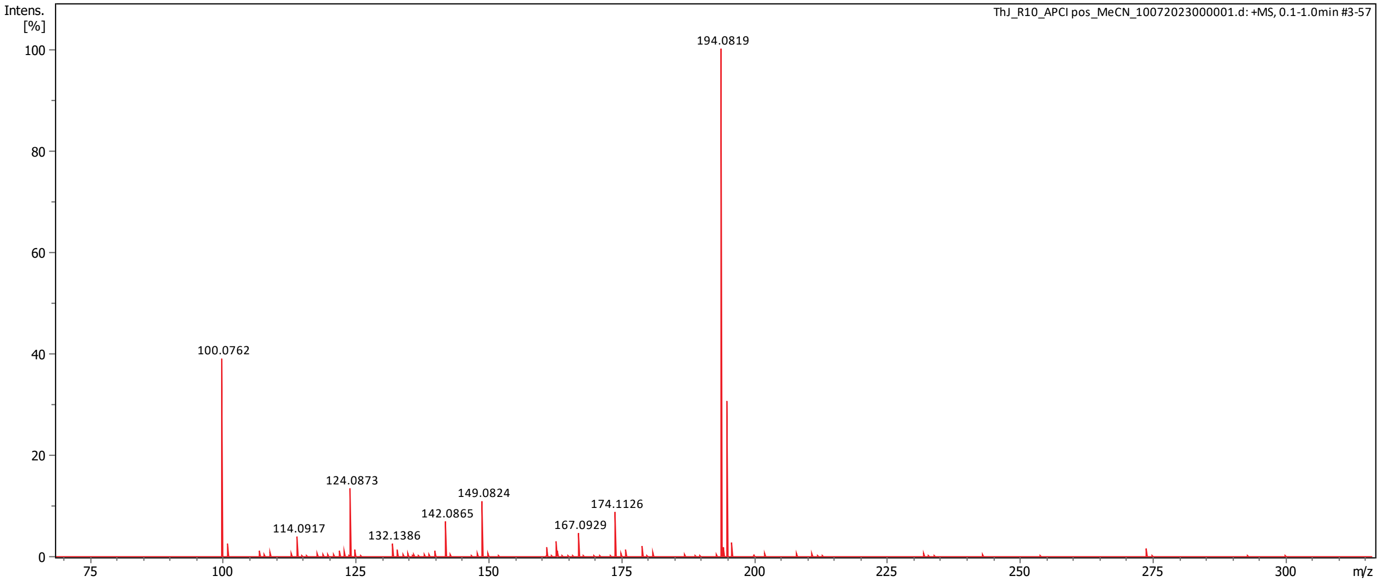

Intens. [%]
ThJ_R10_APCI pos_MeCN_10072023000001.d: +MS, 0.1-1.0min #3-57
194.0819
100.0762
124.0873
114.0917
132.1386
142.0865
149.0824
167.0929
174.1126
m/z

IR

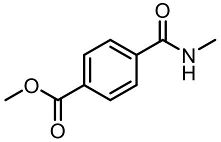

S73

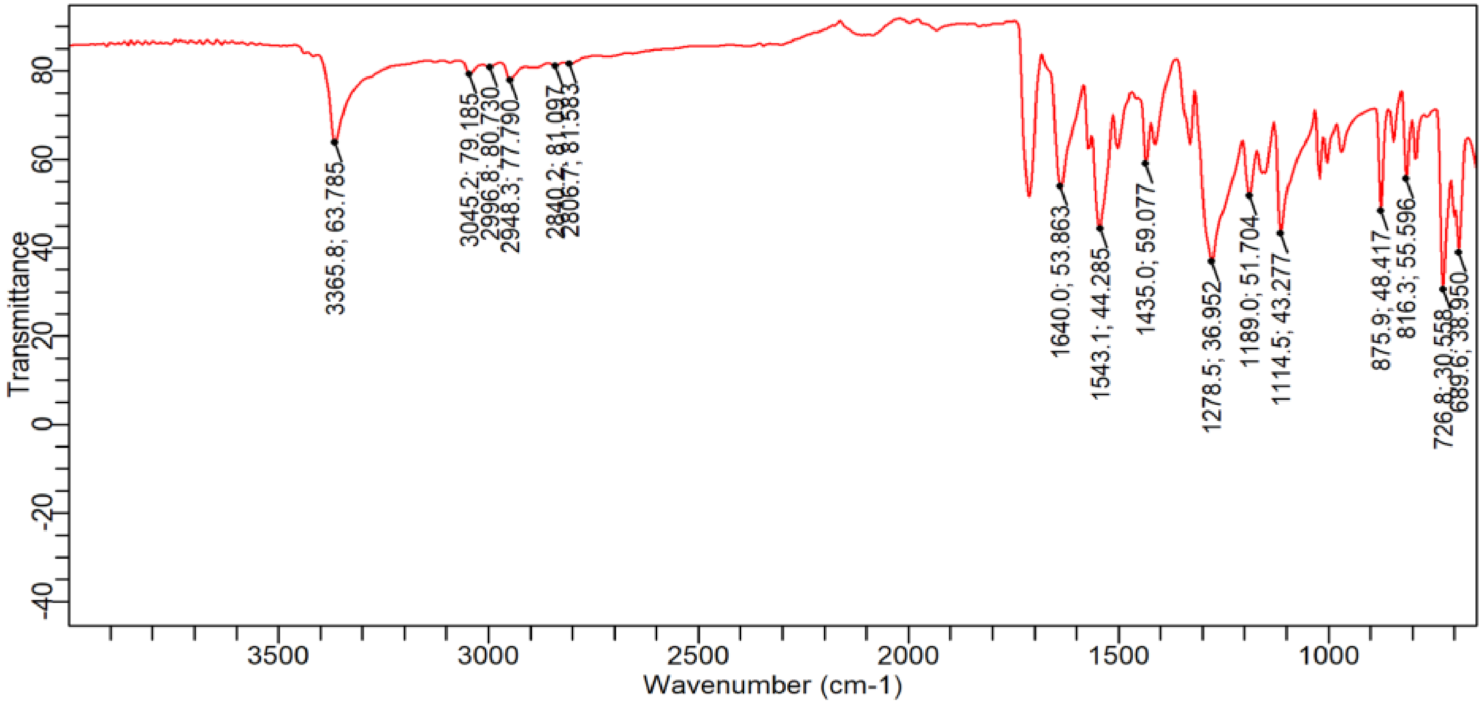
3365.8; 63.785
3045.2; 79.185
2996.8; 80.730
2948.3; 77.790
2840.2; 81.097
2806.7; 81.583
1640.0; 53.863
1543.1; 44.285
1435.0; 59.077
1278.5; 36.952
1189.0; 51.704
1114.5; 43.277
875.9; 48.417
816.3; 55.596
726.8; 30.558
689.6; 38.950
Transmittance
Wavenumber (cm-1)

87 (4-((Methylamino)methyl)phenyl)methanol (S74)

^{1}H NMR

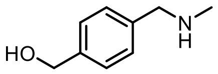

S74

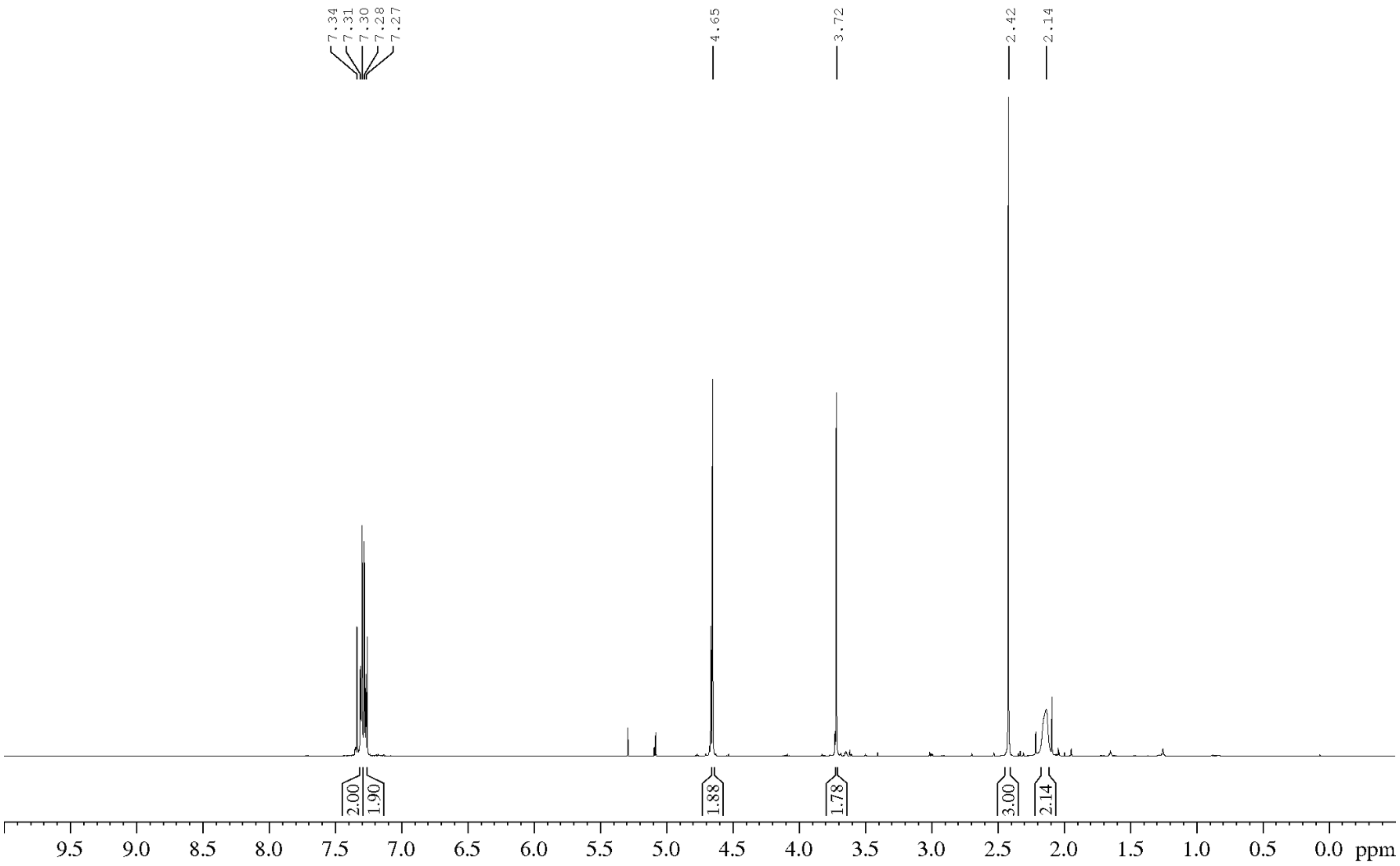

^{13}C NMR

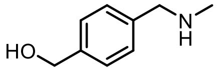

S74

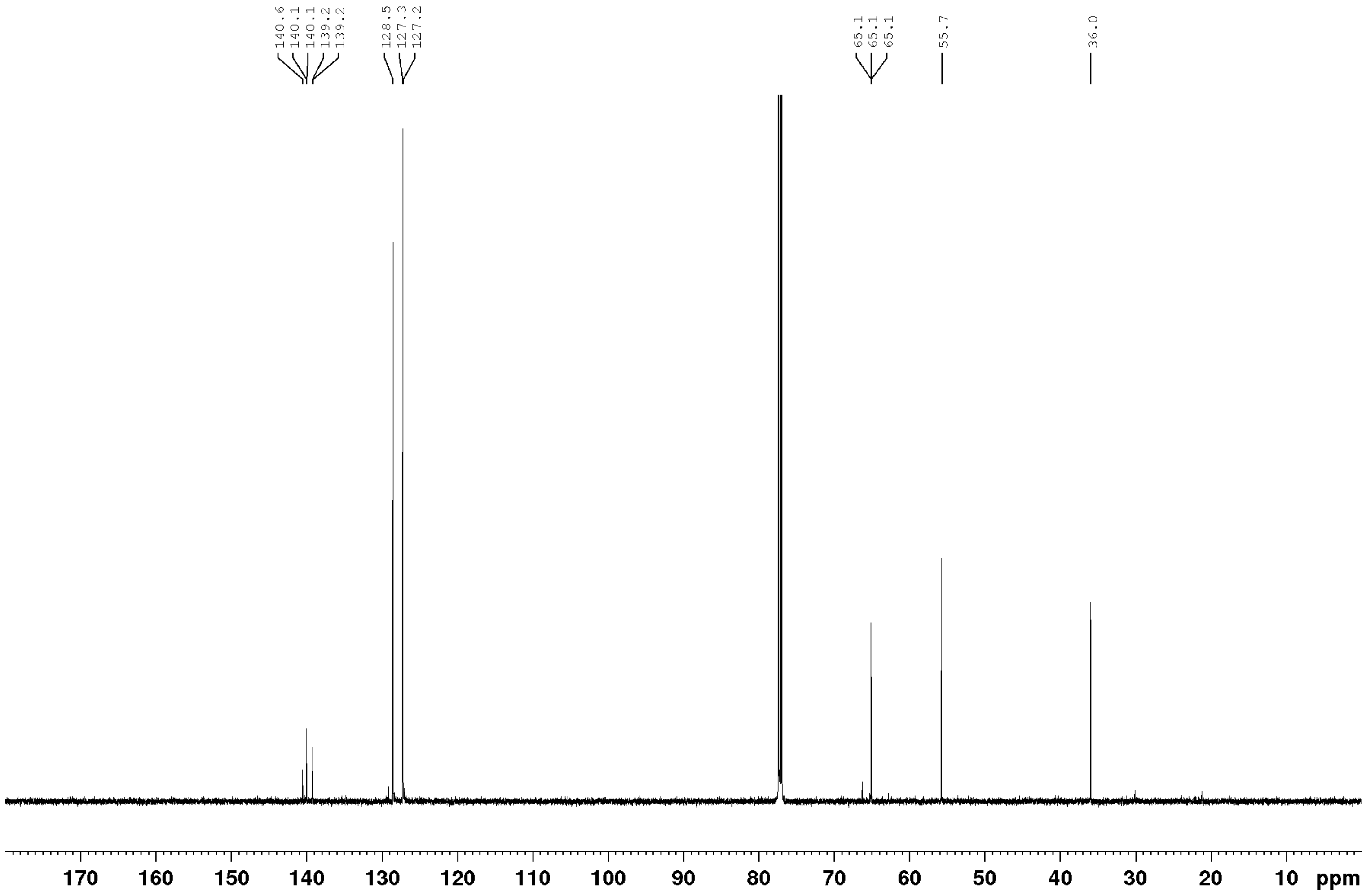

140.6
140.1
140.1
139.2
139.2
128.5
127.3
127.2
65.1
65.1
65.1
55.7
36.0
170 160 150 140 130 120 110 100 90 80 70 60 50 40 30 20 10 ppm

^{1}H, ^{1}H COSY

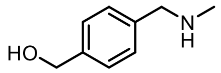

S74

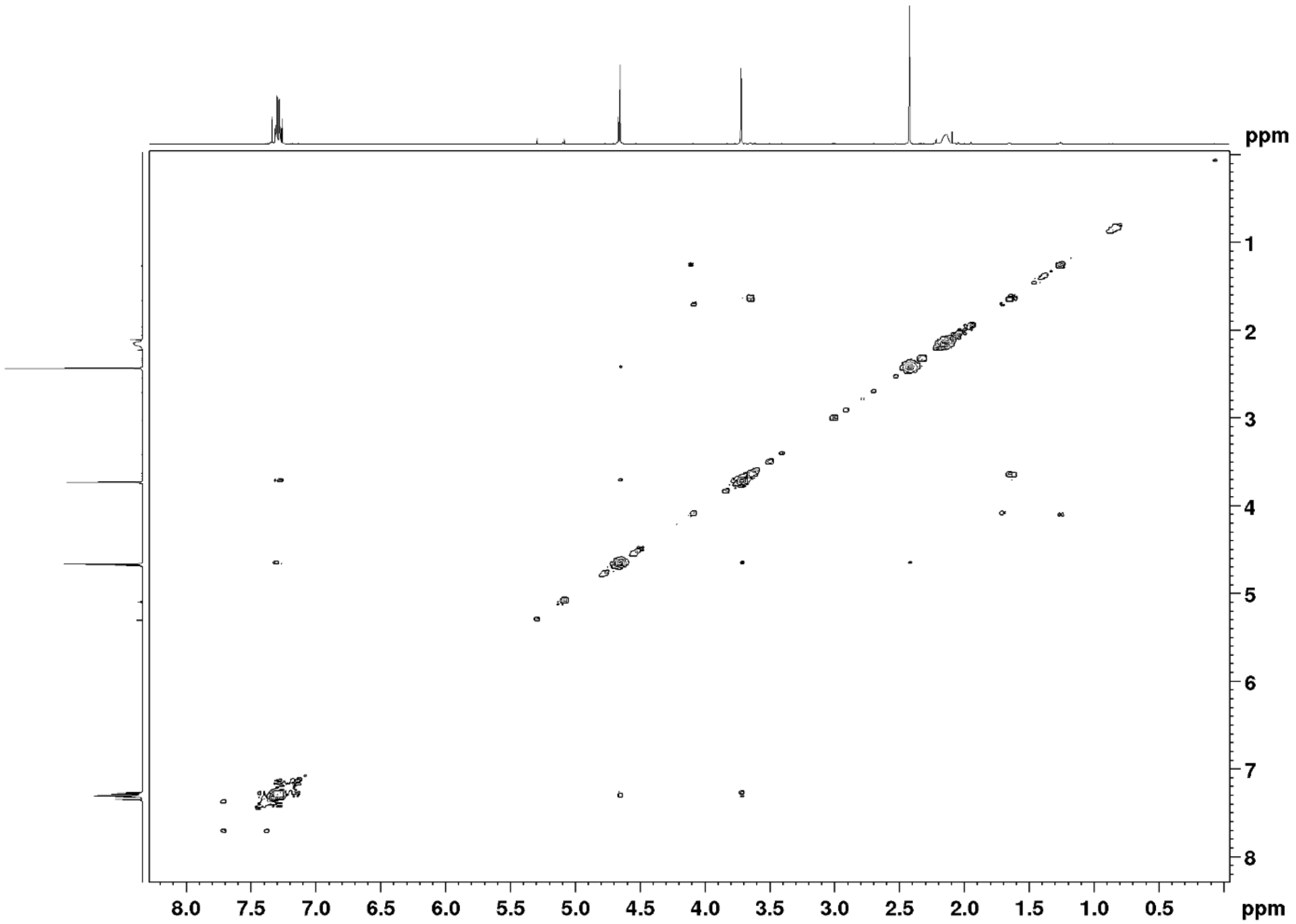

^{1}H, ^{13}C HMBC

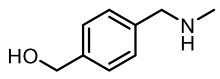

S74

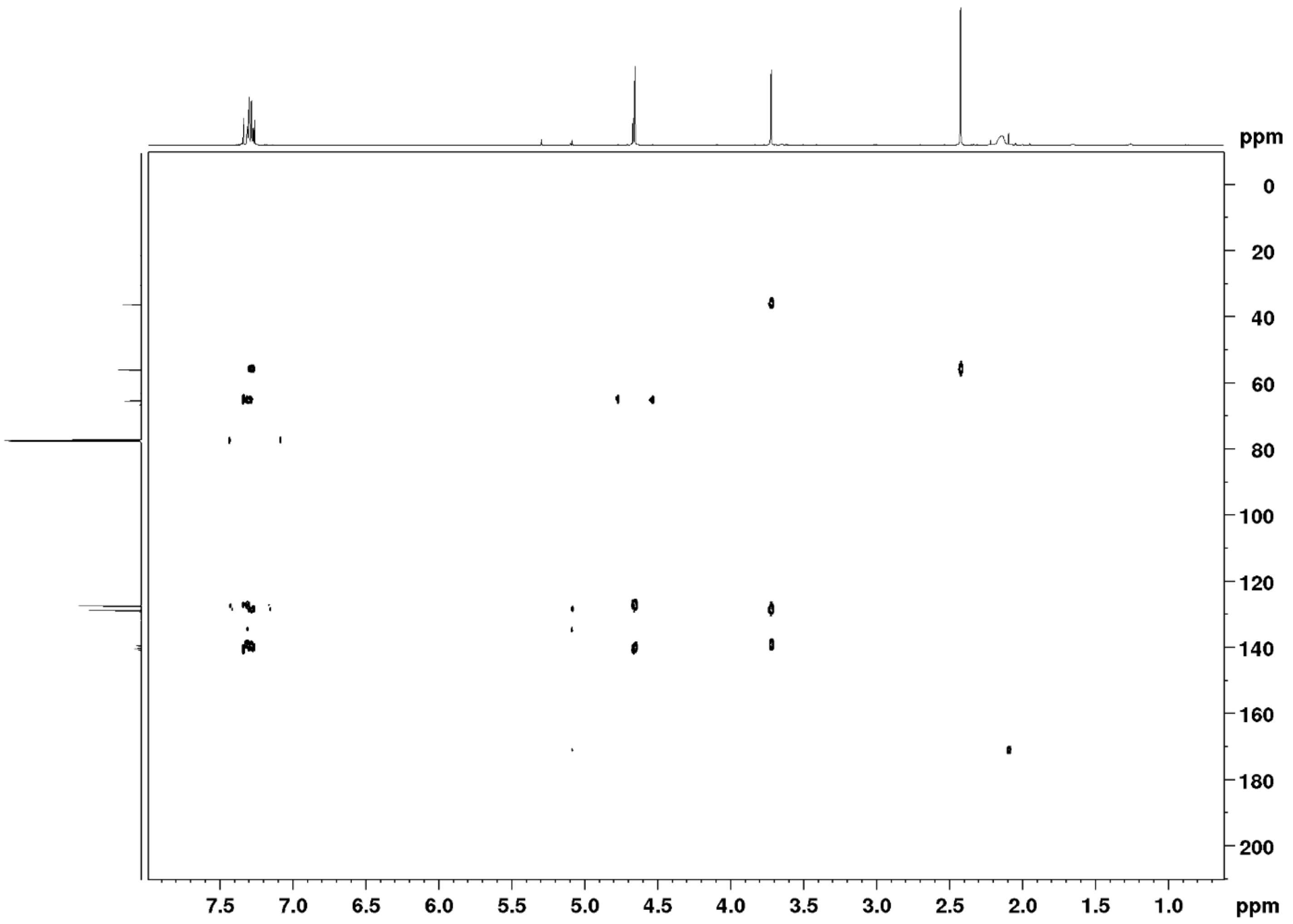

^{1}H, ^{13}C HSQC

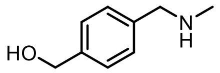

S74

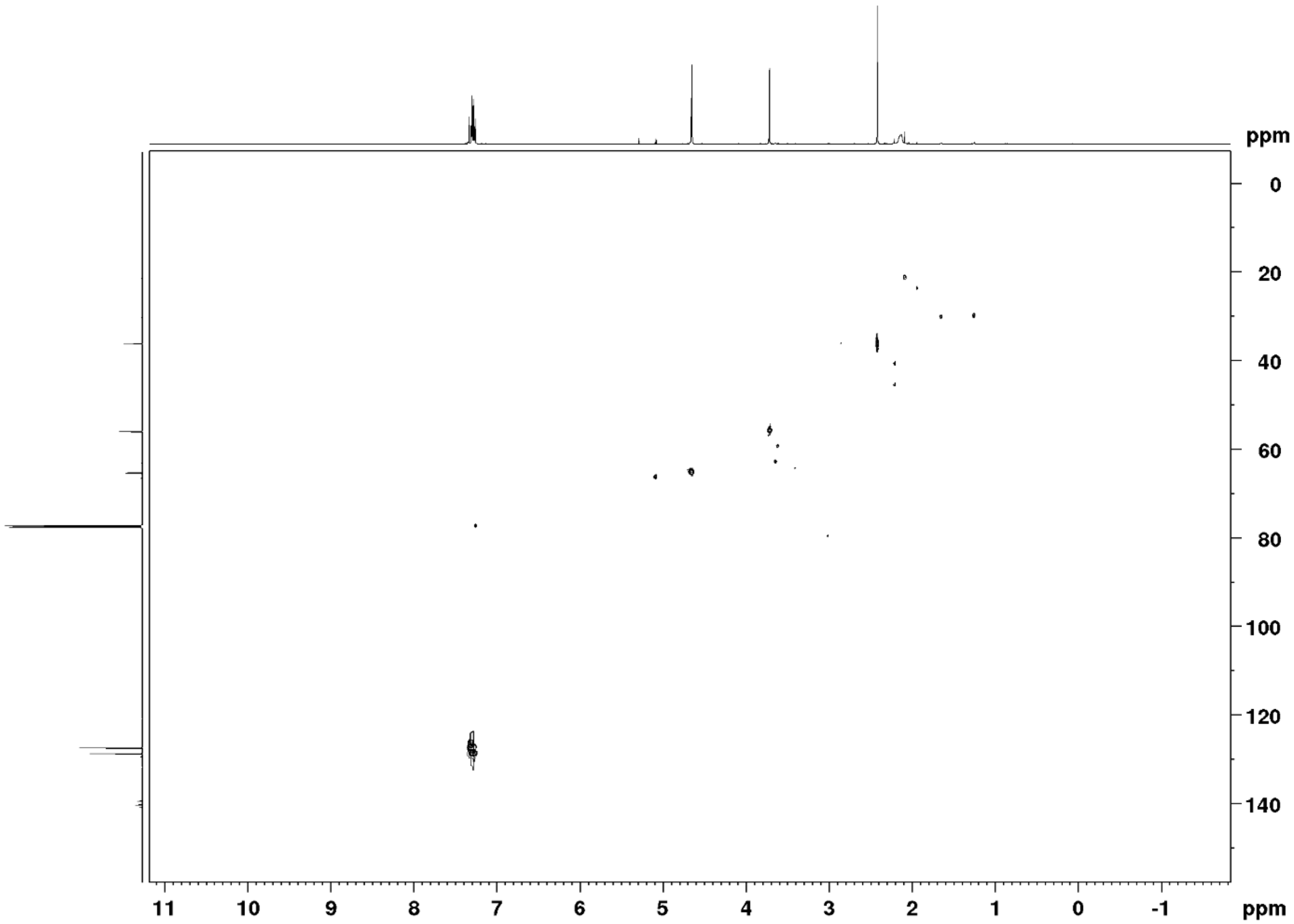

HRMS

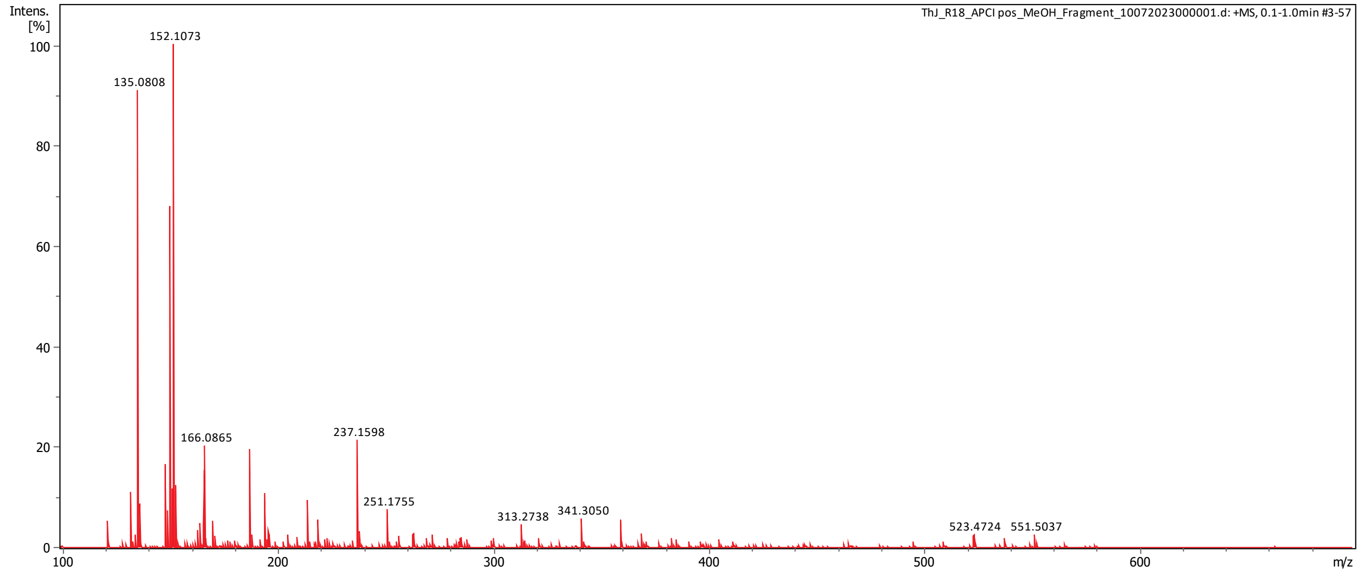
ThJ_R18_APCI pos_MeOH_Fragment_10072023000001.d: +MS, 0.1-1.0min #3-57
Intens. [%]
152.1073
135.0808
166.0865
237.1598
251.1755
313.2738
341.3050
523.4724
551.5037
m/z

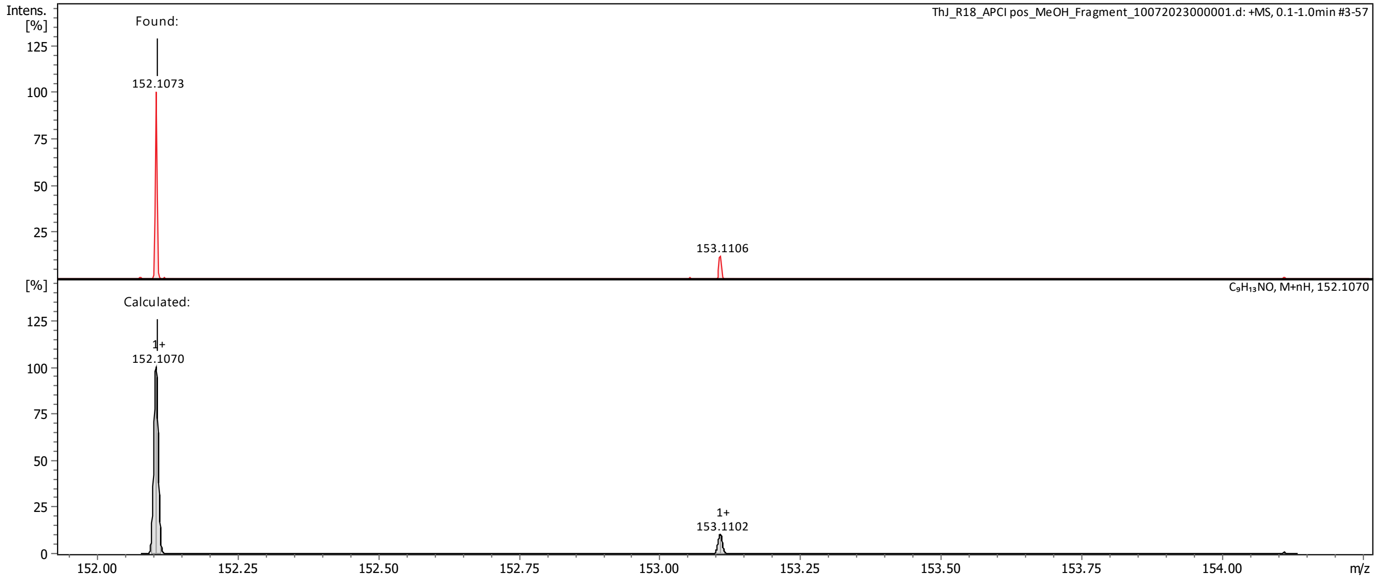
ThJ_R18_APCI pos_MeOH_Fragment_10072023000001.d: +MS, 0.1-1.0min #3-57
Intens. [%]
Found:
152.1073
153.1106
Calculated:
C9H13NO, M+nH, 152.1070
1+
152.1070
1+
153.1102
m/z

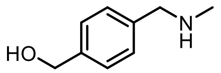
HO
N
H

S74

IR

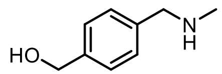

S74

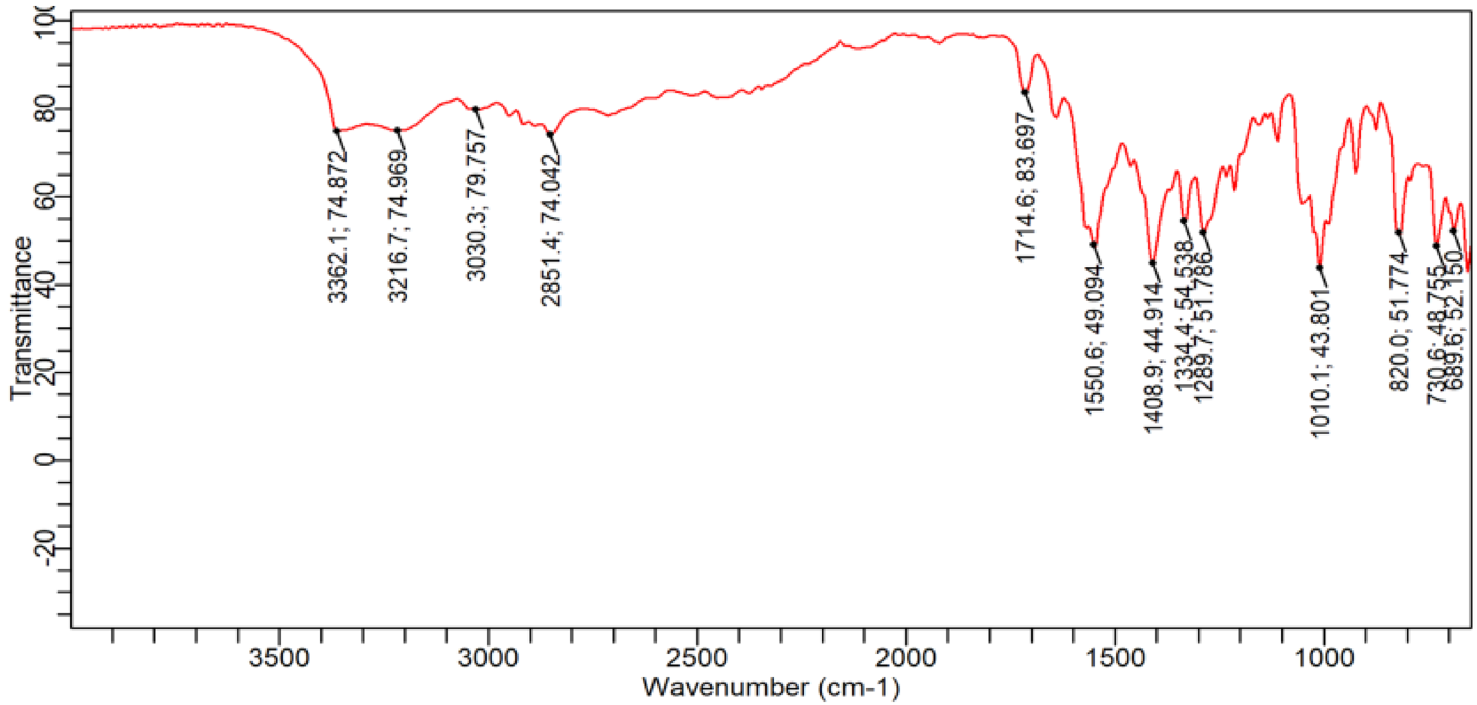

Transmittance
Wavenumber (cm-1)
3362.1; 74.872
3216.7; 74.969
3030.3; 79.757
2851.4; 74.042
1714.6; 83.697
1550.6; 49.094
1408.9; 44.914
1334.4; 54.538
1289.7; 51.786
1010.1; 43.801
820.0; 51.774
730.6; 48.755
689.6; 52.150

88 1-(4-(Bromomethyl)phenyl)-*N*-methylmethanamine hydrobromide (*S*79)

^{1}H NMR

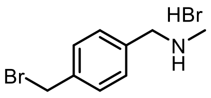

S79

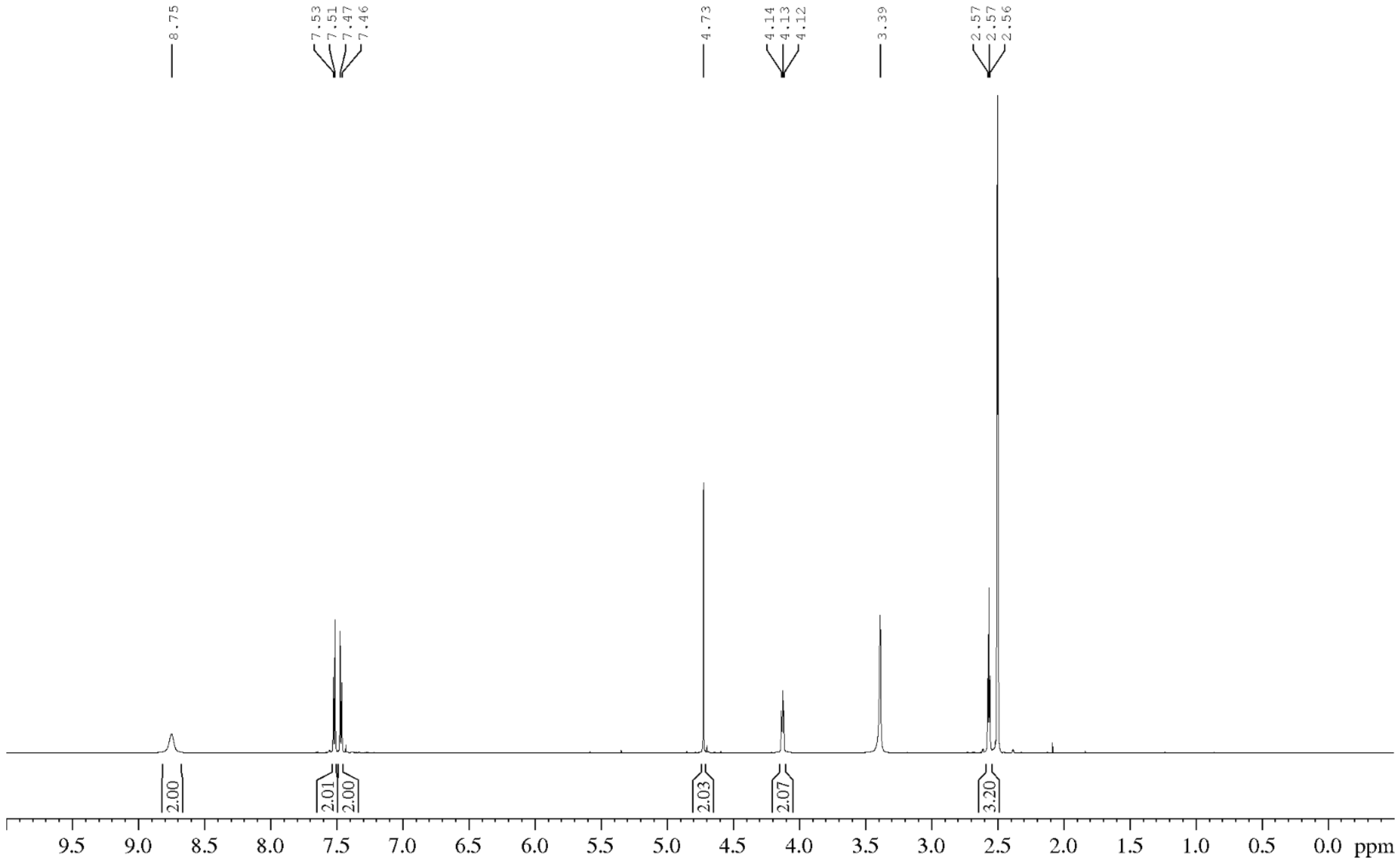

^{13}C NMR

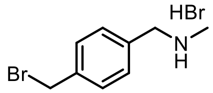

S79

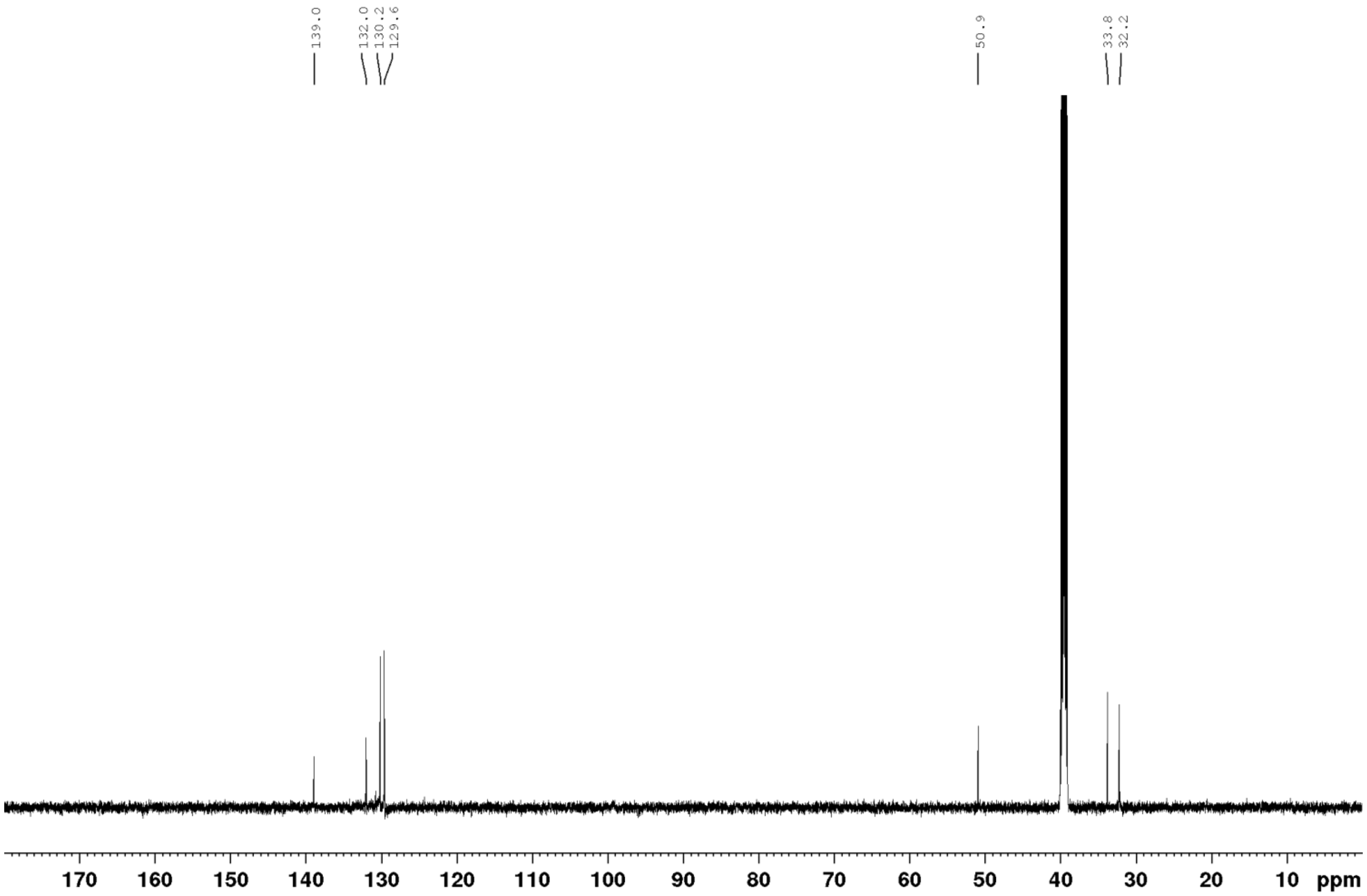

139.0
132.0
130.2
129.6
50.9
33.8
32.2
170
160
150
140
130
120
110
100
90
80
70
60
50
40
30
20
10
ppm

^{1}H, ^{1}H COSY

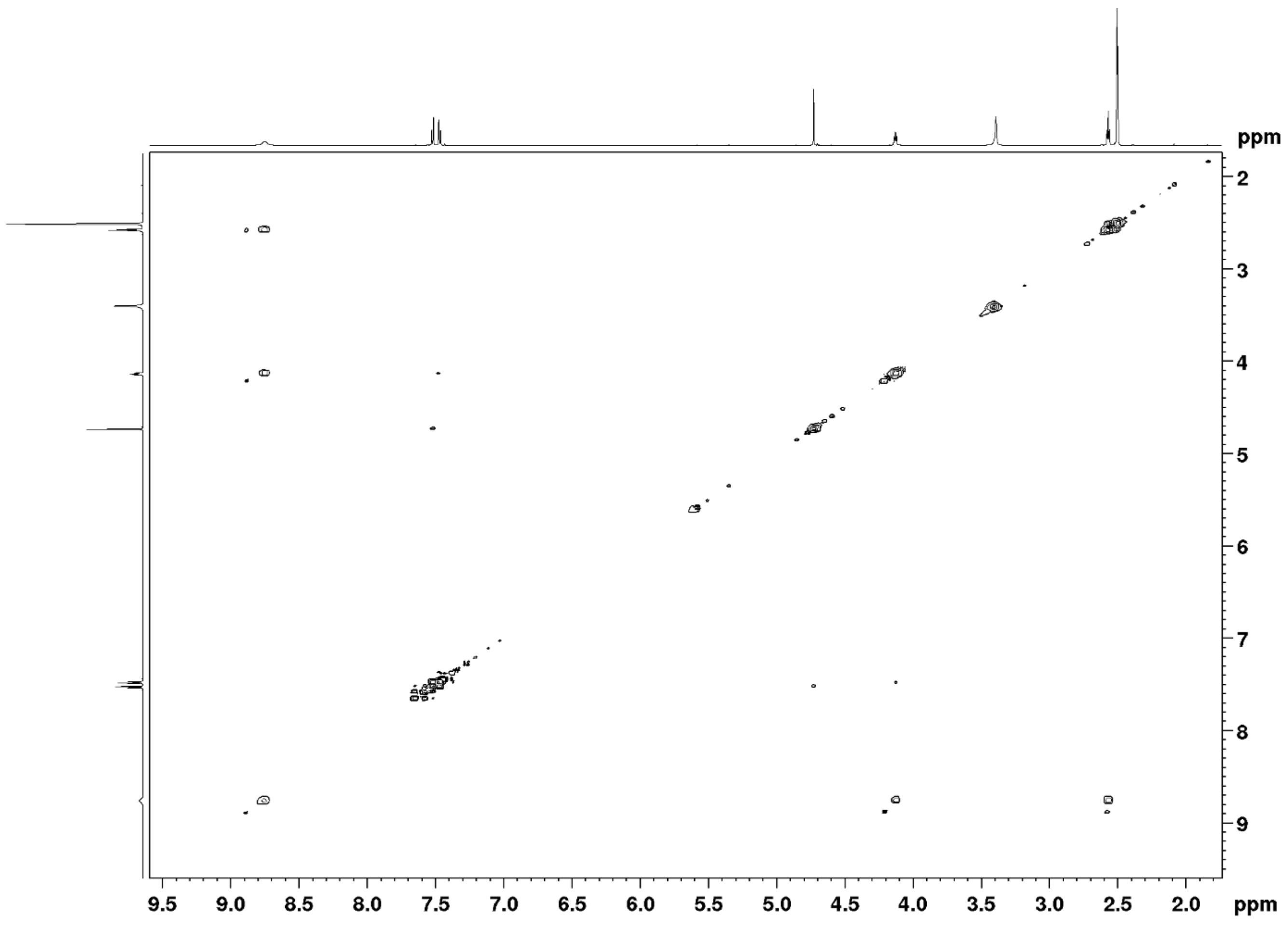

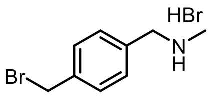

S79

^{1}H, ^{13}C HMBC

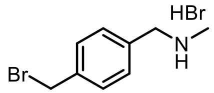

S79

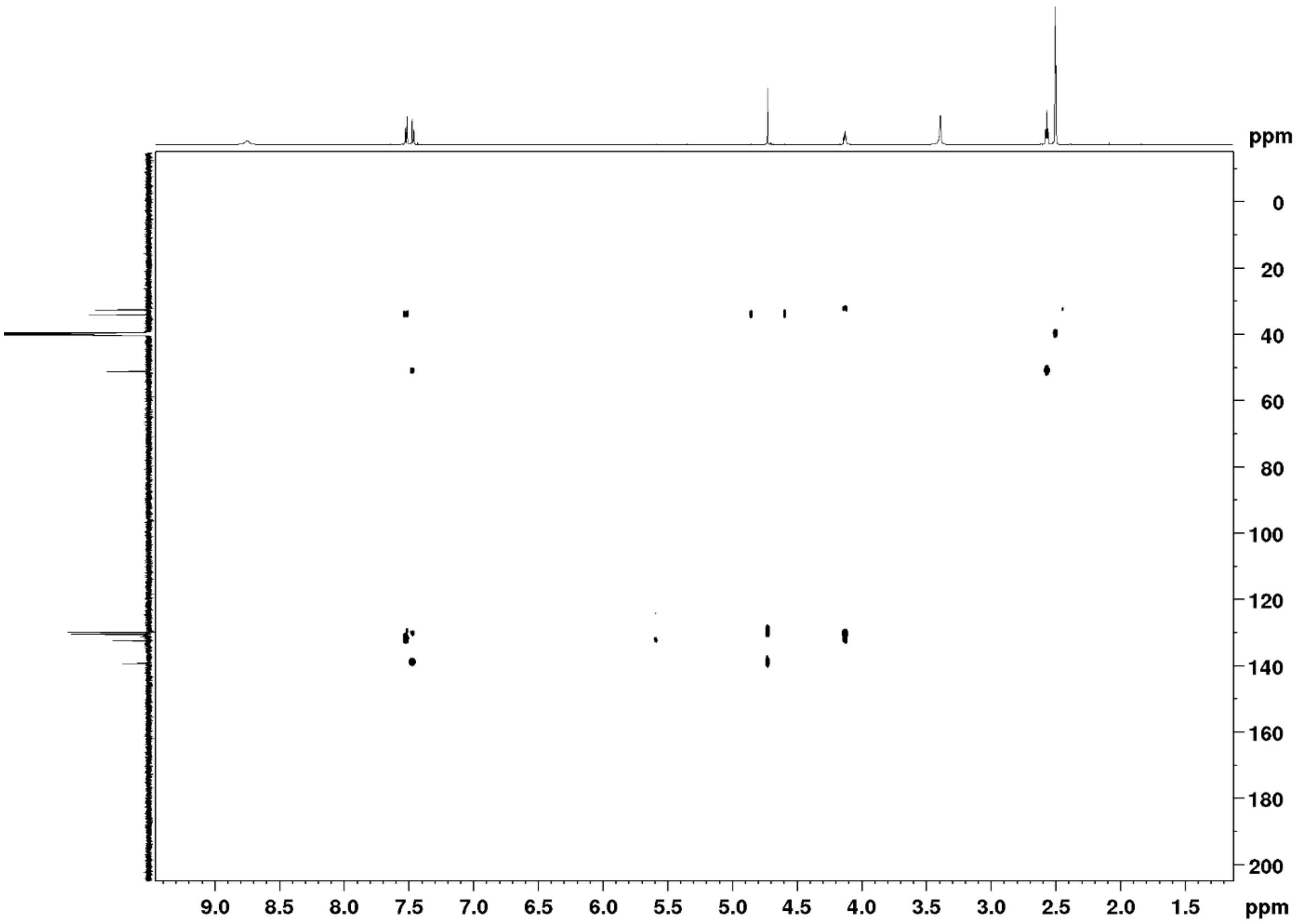

^{1}H, ^{13}C HSQC

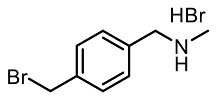

S79

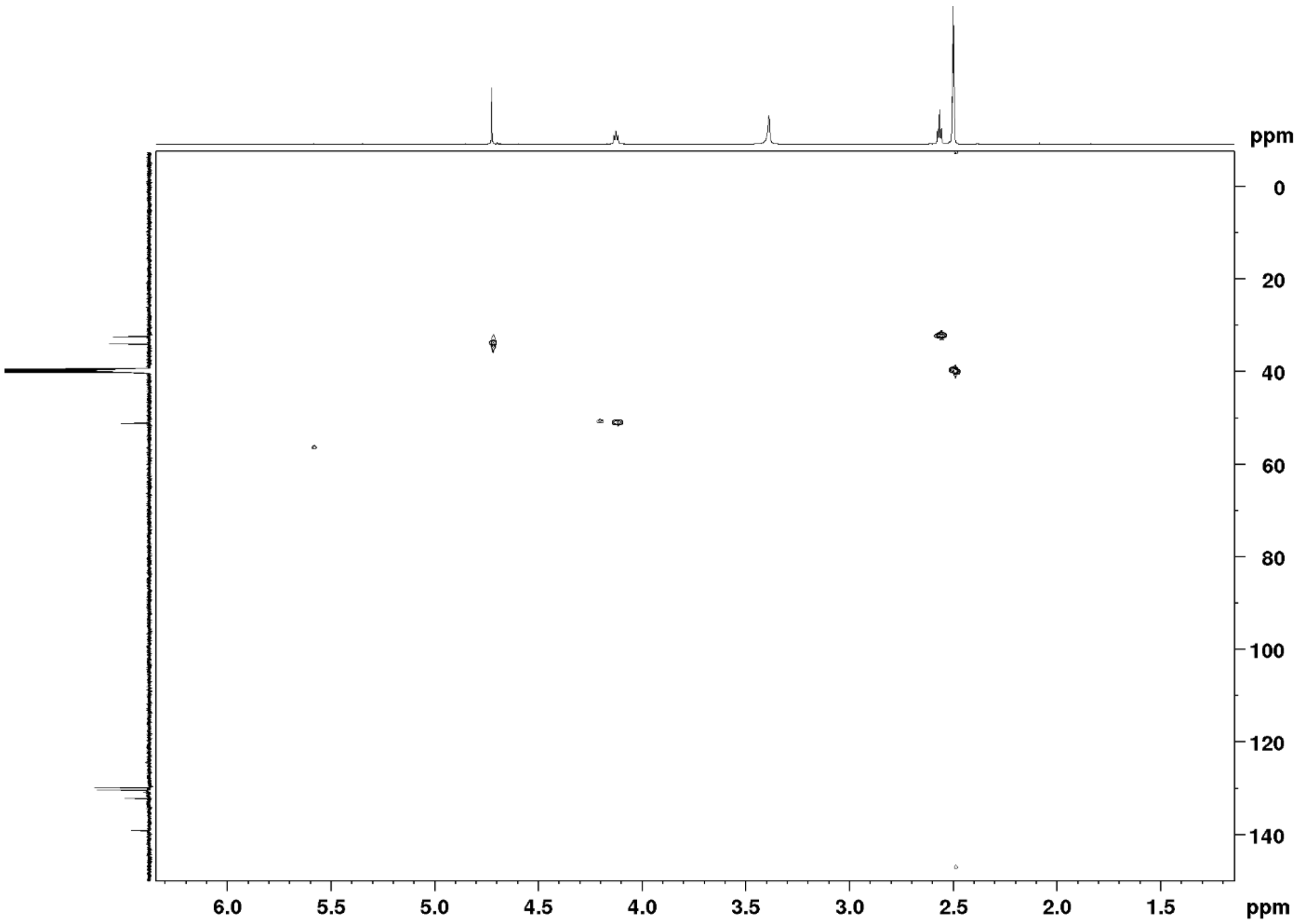

HRMS

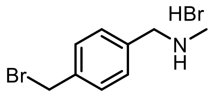
HBr
Br
N
H

S79

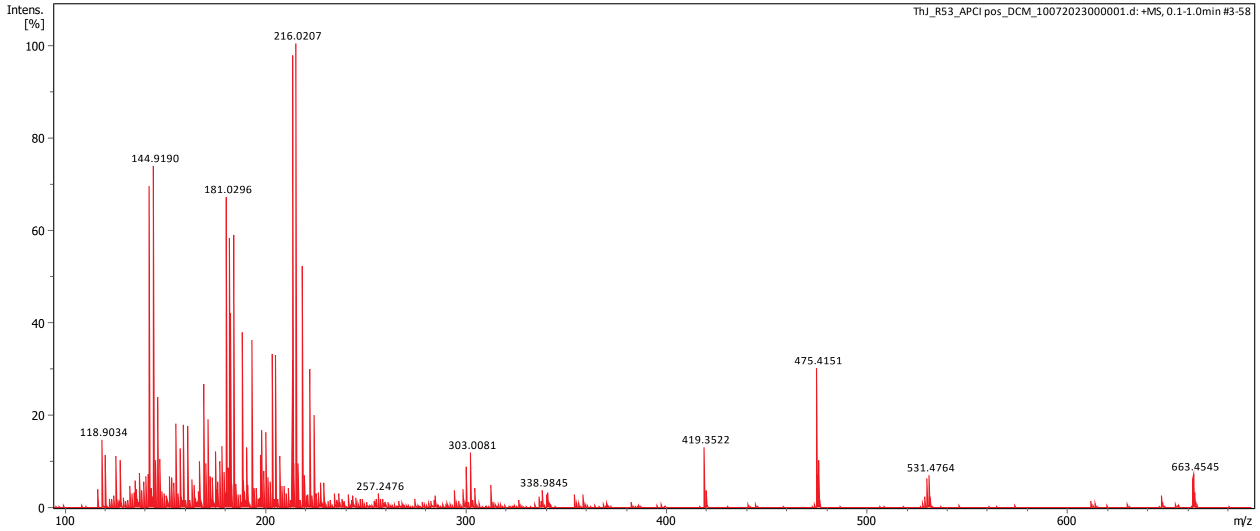
Intens.
[%]
ThJ_R53_APCI pos_DCM_10072023000001.d: +MS, 0.1-1.0min #3-58
216.0207
144.9190
181.0296
475.4151
118.9034
303.0081
257.2476
338.9845
419.3522
531.4764
663.4545
m/z

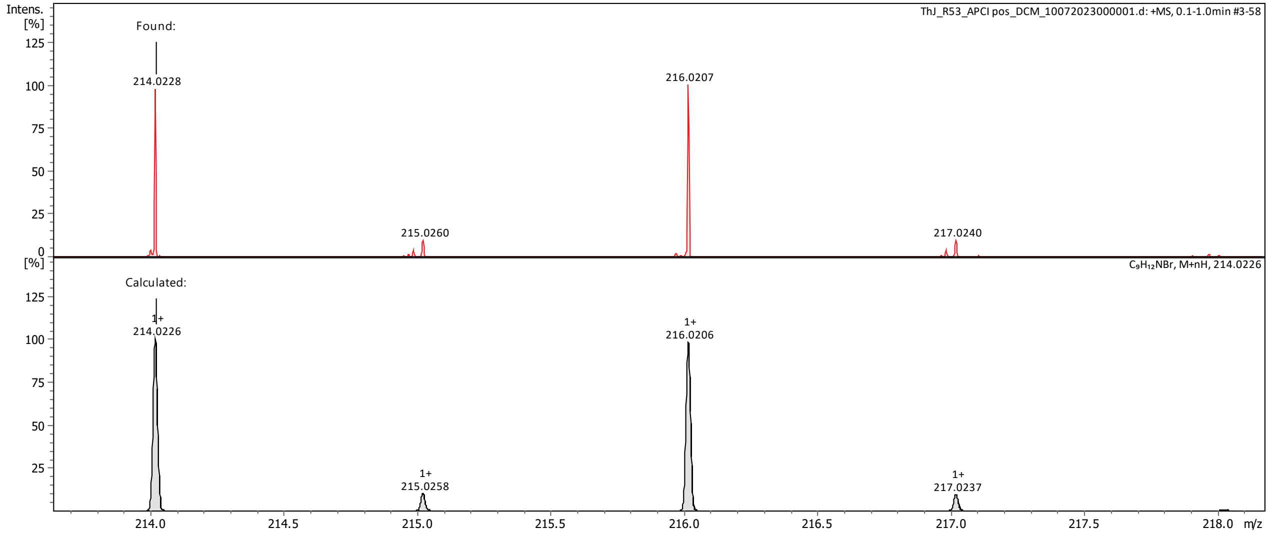
Intens.
[%]
ThJ_R53_APCI pos_DCM_10072023000001.d: +MS, 0.1-1.0min #3-58
Found:
214.0228
216.0207
215.0260
217.0240
$C_9H_{12}NBr$, M+nH, 214.0226
Calculated:
1+
214.0226
1+
216.0206
1+
215.0258
1+
217.0237
m/z

IR

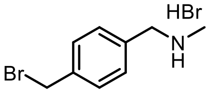
HBr
N
H
Br

S79

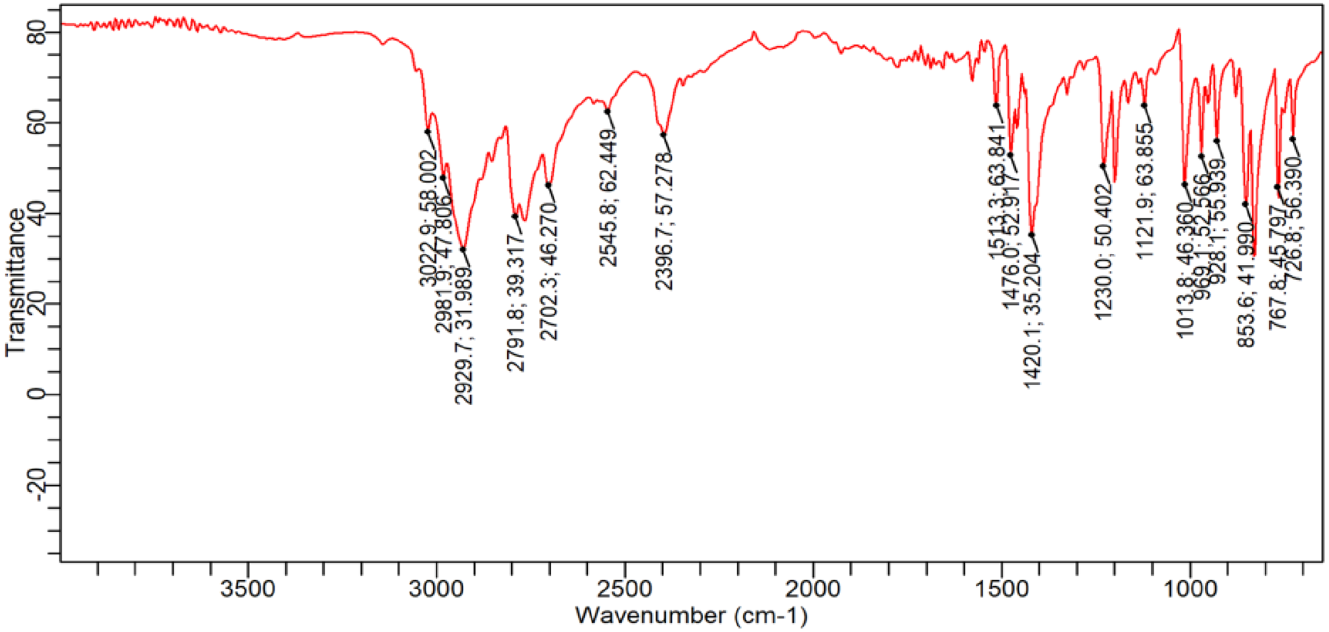
Transmittance
Wavenumber (cm-1)
3022.9; 58.002
2981.9; 47.806
2929.7; 31.989
2791.8; 39.317
2702.3; 46.270
2545.8; 62.449
2396.7; 57.278
1513.3; 63.841
1476.0; 52.917
1420.1; 35.204
1230.0; 50.402
1121.9; 63.855
1013.8; 46.360
969.1; 52.566
928.1; 55.939
853.6; 41.990
767.8; 45.797
726.8; 56.390

89 1-Mesityl-3-(4-((methylamino)methyl)benzyl)-1*H*-imidazol-3-ium bromide hydrobromide (S80)

^{1}H NMR

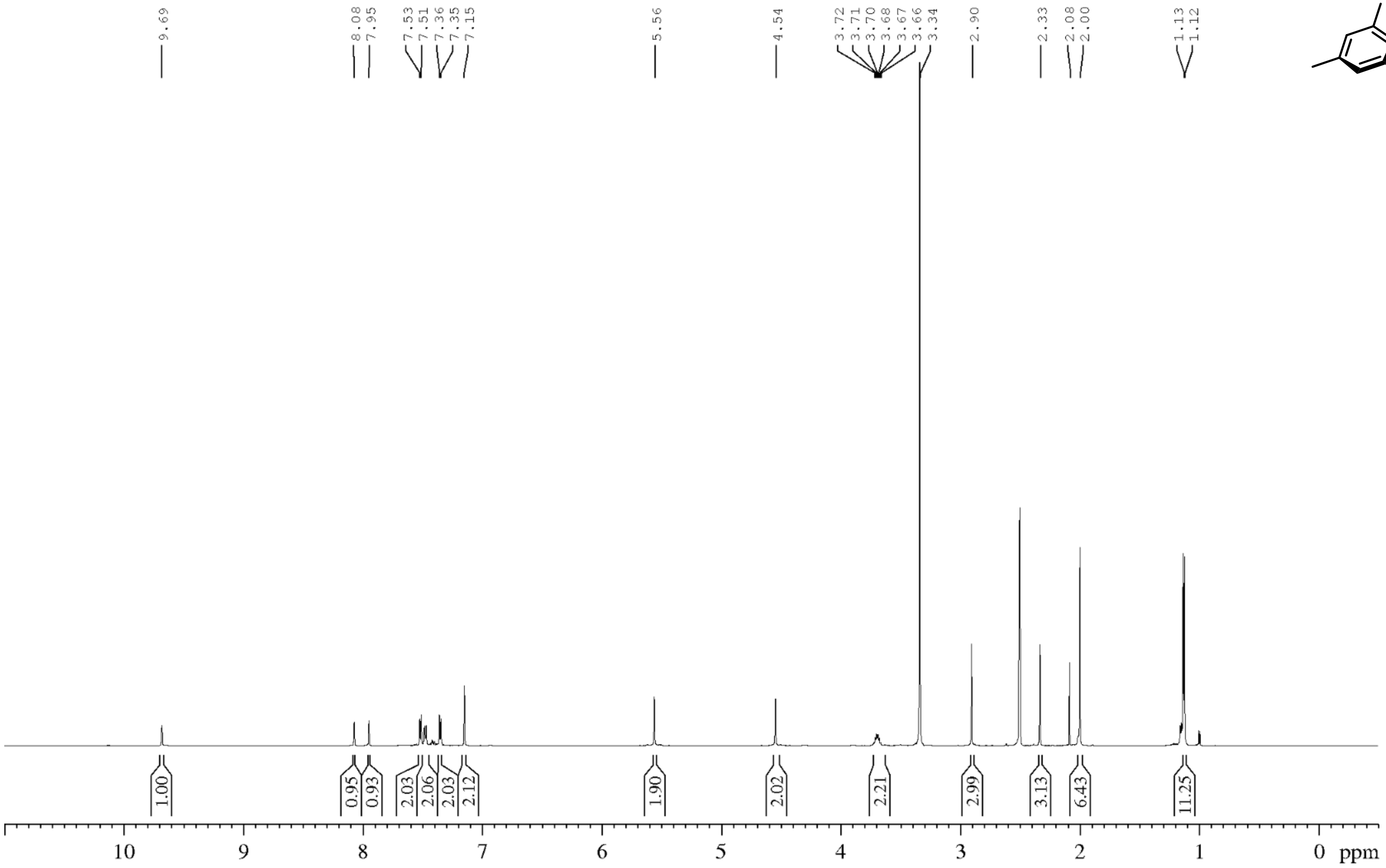

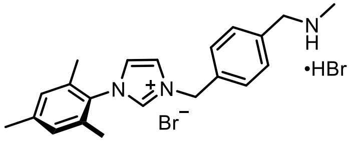

S80

^{13}C NMR

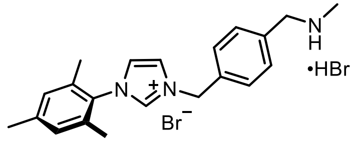

S80

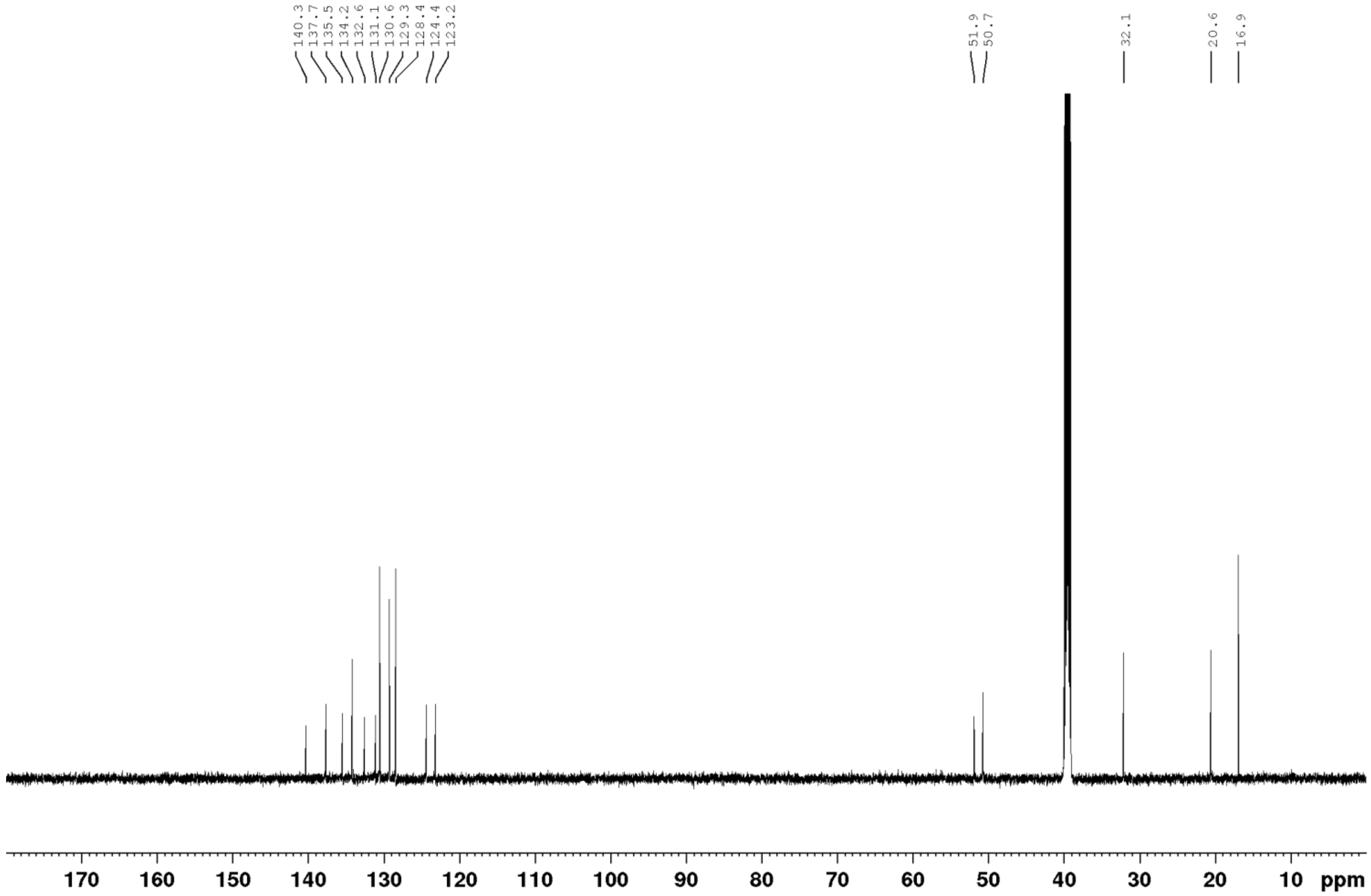
140.3
137.7
135.5
134.2
132.6
131.1
130.6
129.3
128.4
124.4
123.2
51.9
50.7
32.1
20.6
16.9
170 160 150 140 130 120 110 100 90 80 70 60 50 40 30 20 10 ppm

^1H, ^1H COSY

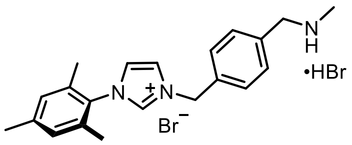

S80

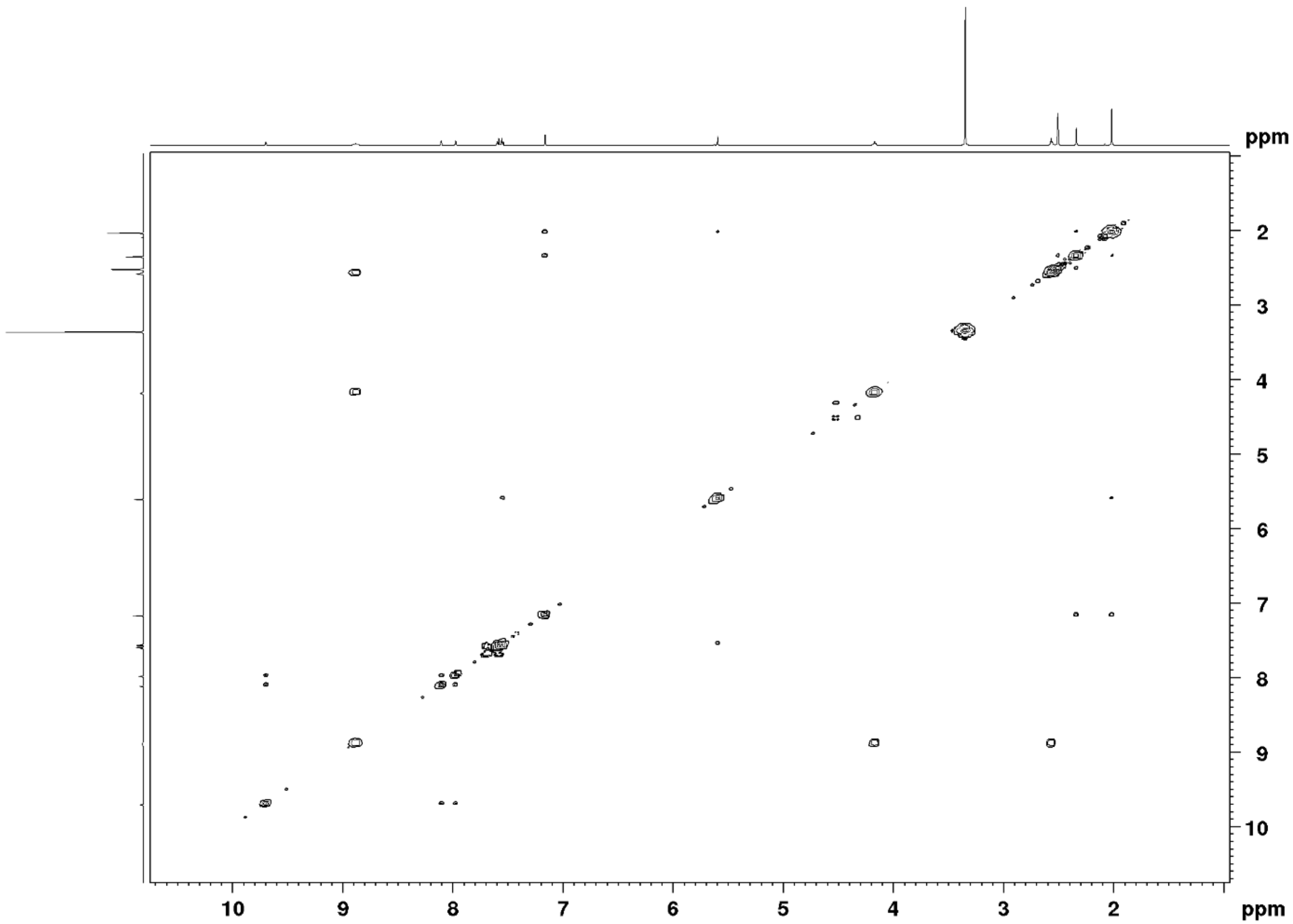

^{1}H, ^{13}C HMBC

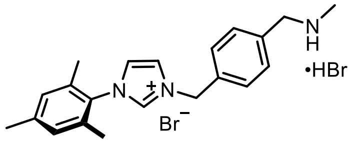

S80

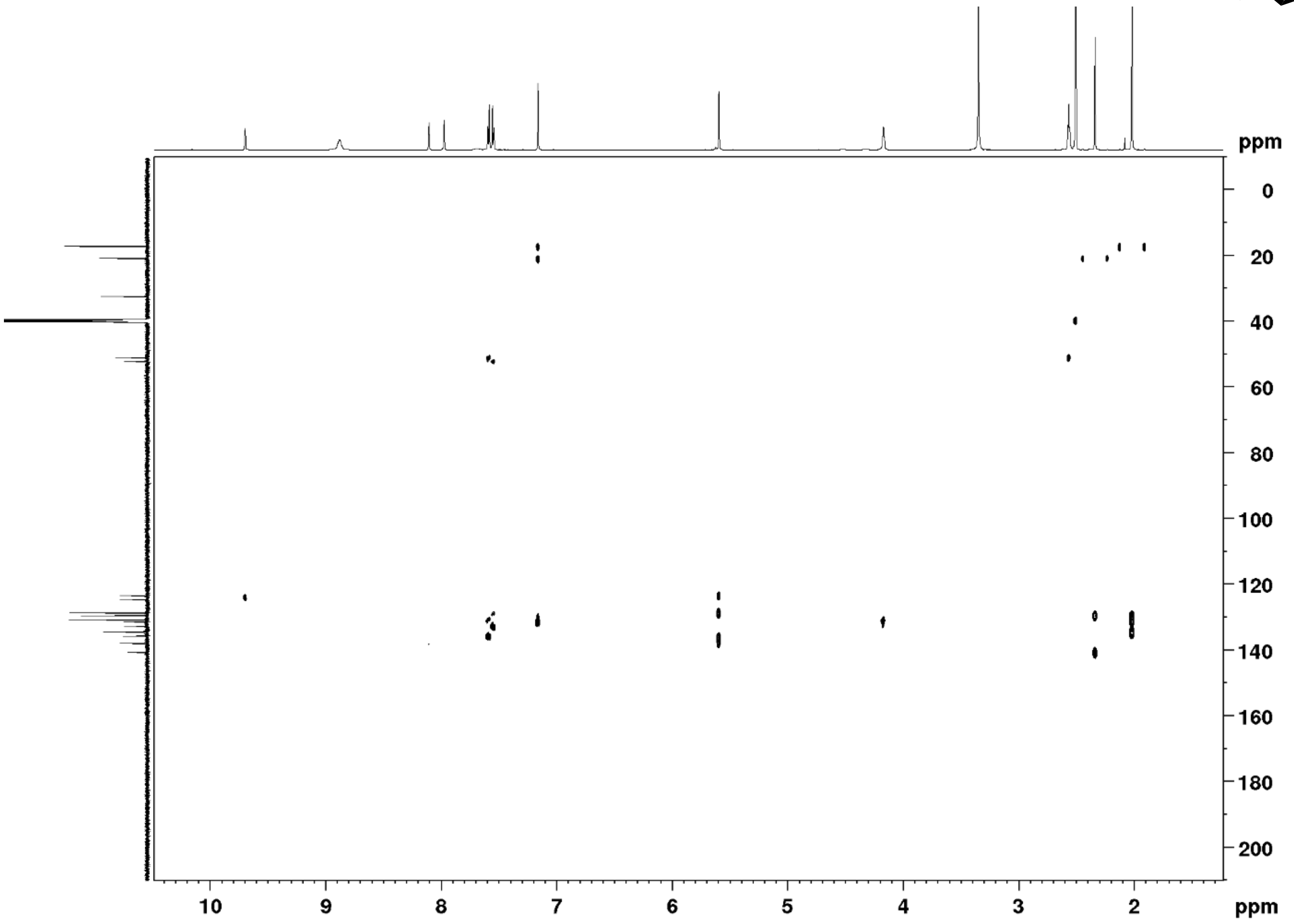

^{1}H, ^{13}C HSQC

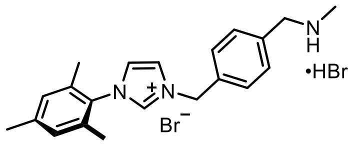

S80

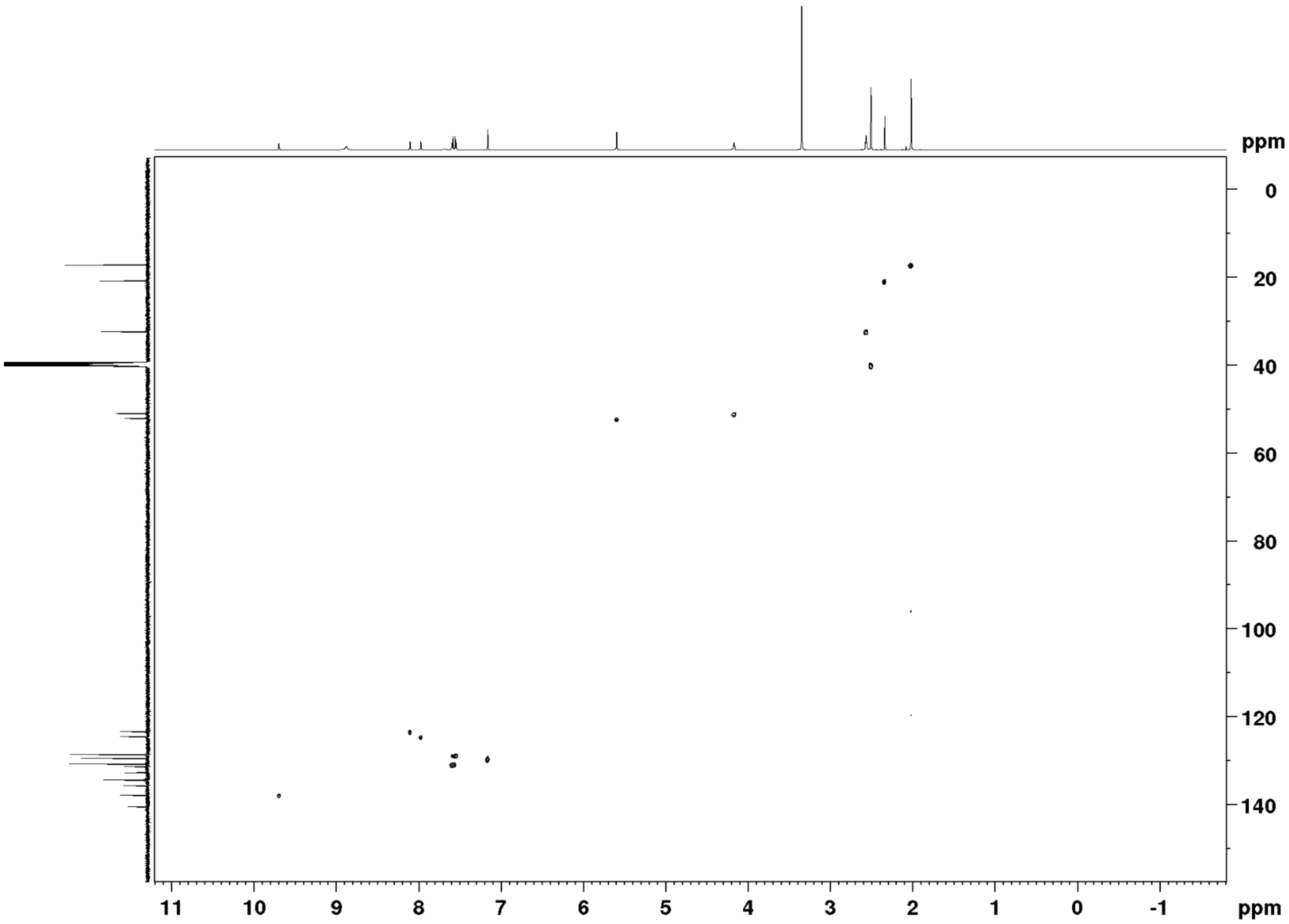

HRMS

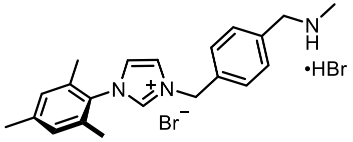
N
H
•HBr
N
+
N
Br⁻

S80

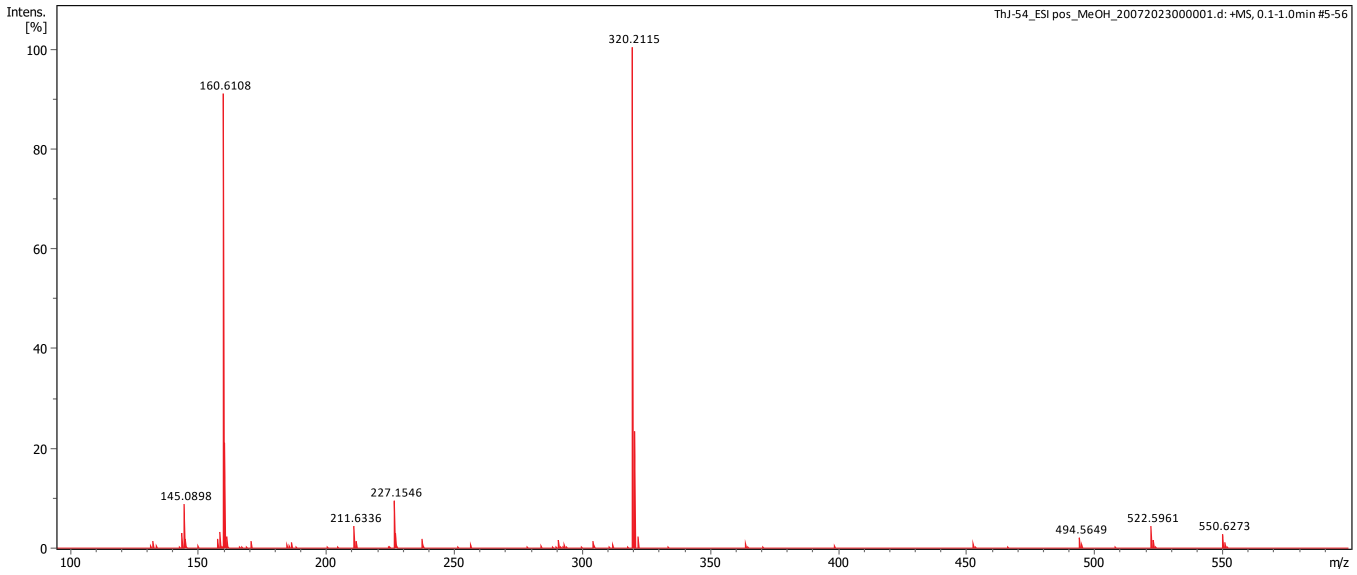
Intens.
[%]
ThJ-54_ESI pos_MeOH_20072023000001.d: +MS, 0.1-1.0min #5-56
320.2115
160.6108
145.0898
211.6336
227.1546
494.5649
522.5961
550.6273
m/z

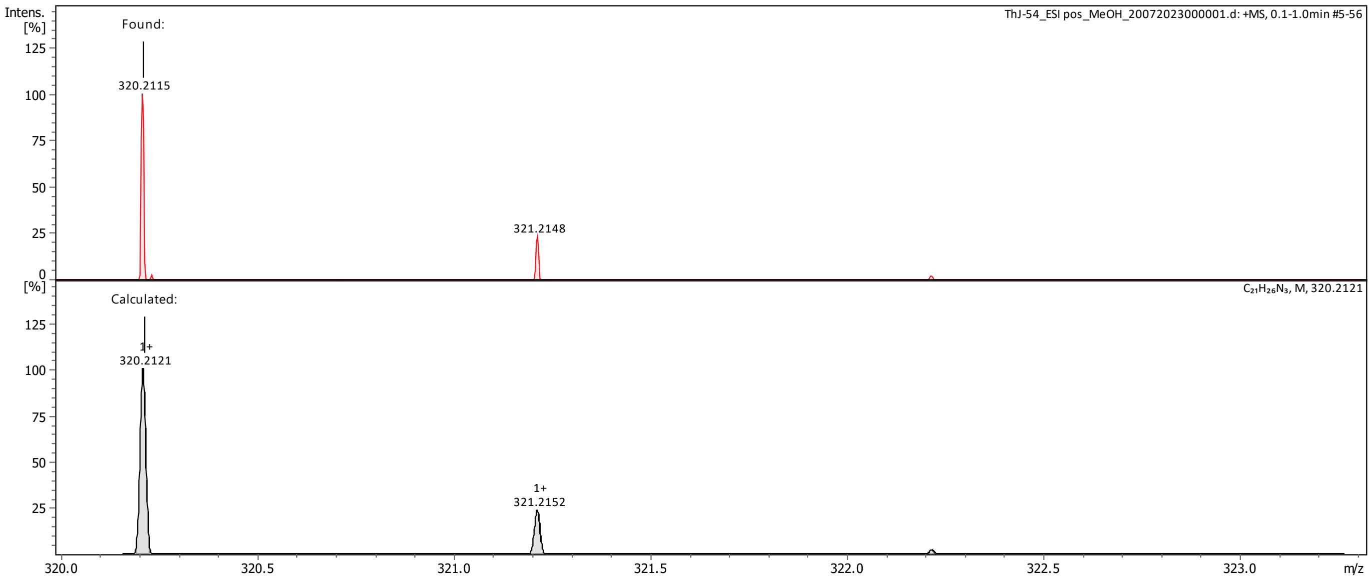
Intens.
[%]
Found:
ThJ-54_ESI pos_MeOH_20072023000001.d: +MS, 0.1-1.0min #5-56
320.2115
321.2148
Calculated:
C21H26N3, M, 320.2121
1+
320.2121
1+
321.2152
m/z

IR

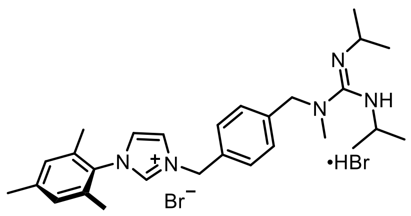
N
N
Br
•HBr
N
N
NH

S80

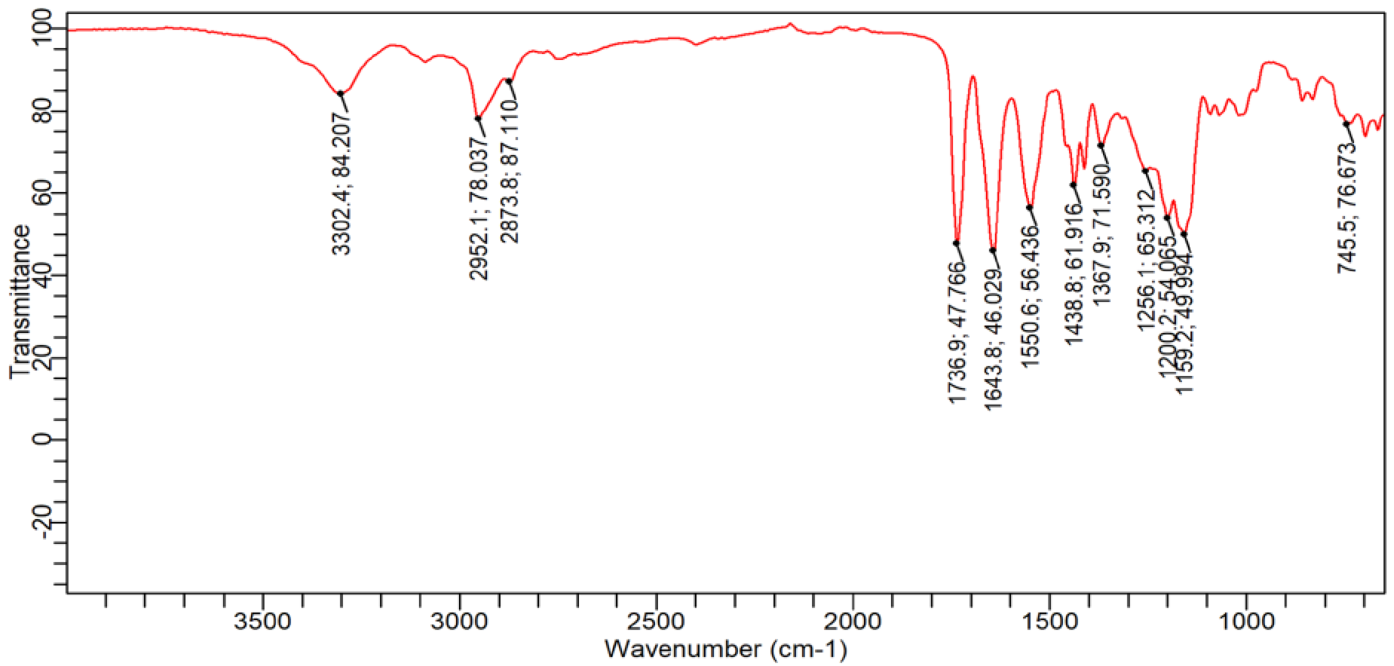
Transmittance
Wavenumber (cm-1)
3302.4; 84.207
2952.1; 78.037
2873.8; 87.110
1736.9; 47.766
1643.8; 46.029
1550.6; 56.436
1438.8; 61.916
1367.9; 71.590
1256.1; 65.312
1200.2; 54.065
1159.2; 49.994
745.5; 76.673

90 (*E*)-3-(4-((2,3-Diisopropyl-1-methylguanidino)methyl)benzyl)-1-mesityl-1*H*-imidazol-3-ium bromide hydrobromide (S81)

^{1}H NMR

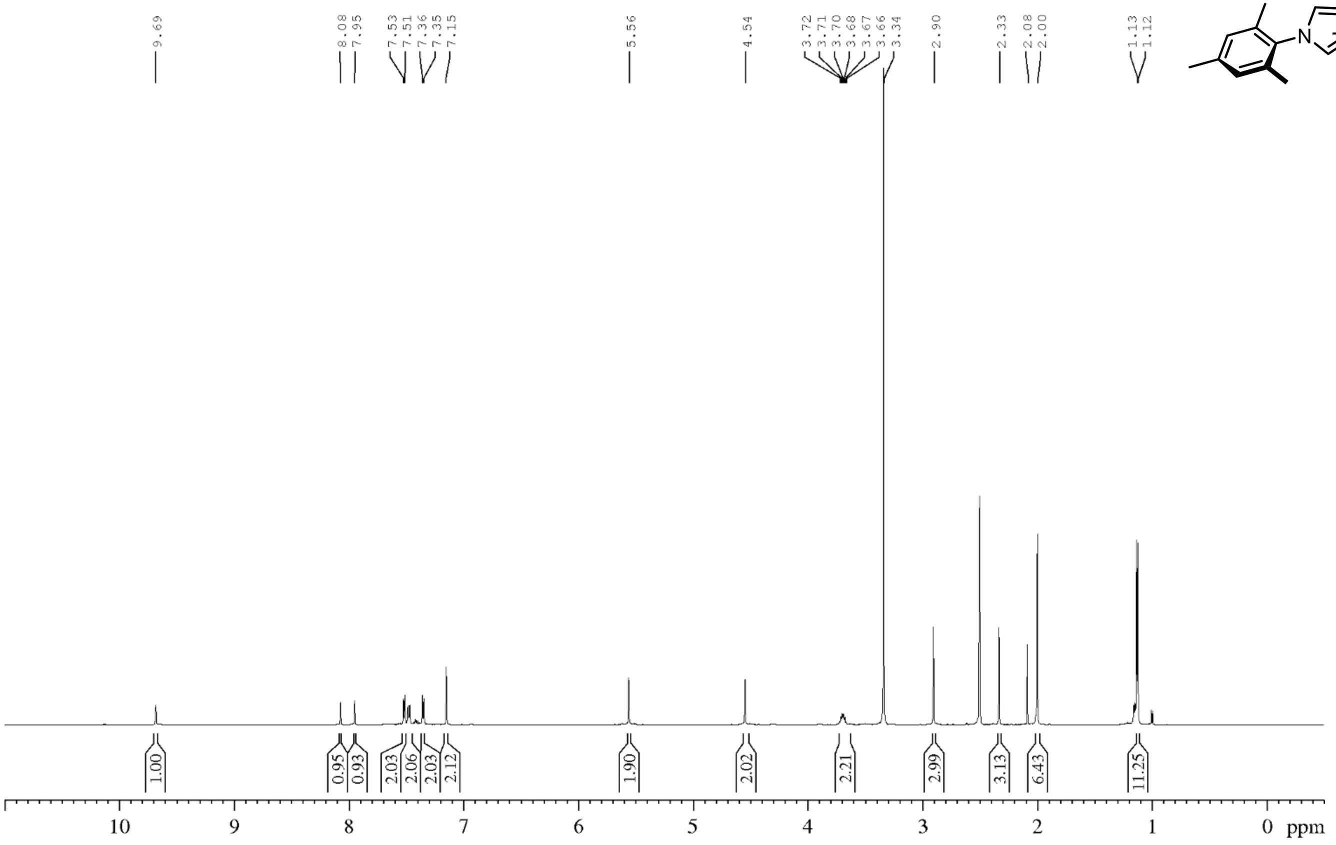

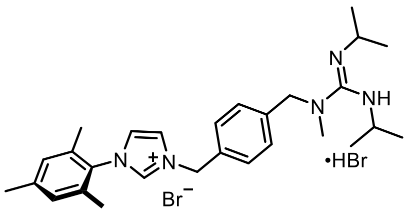

S81

^{13}C NMR

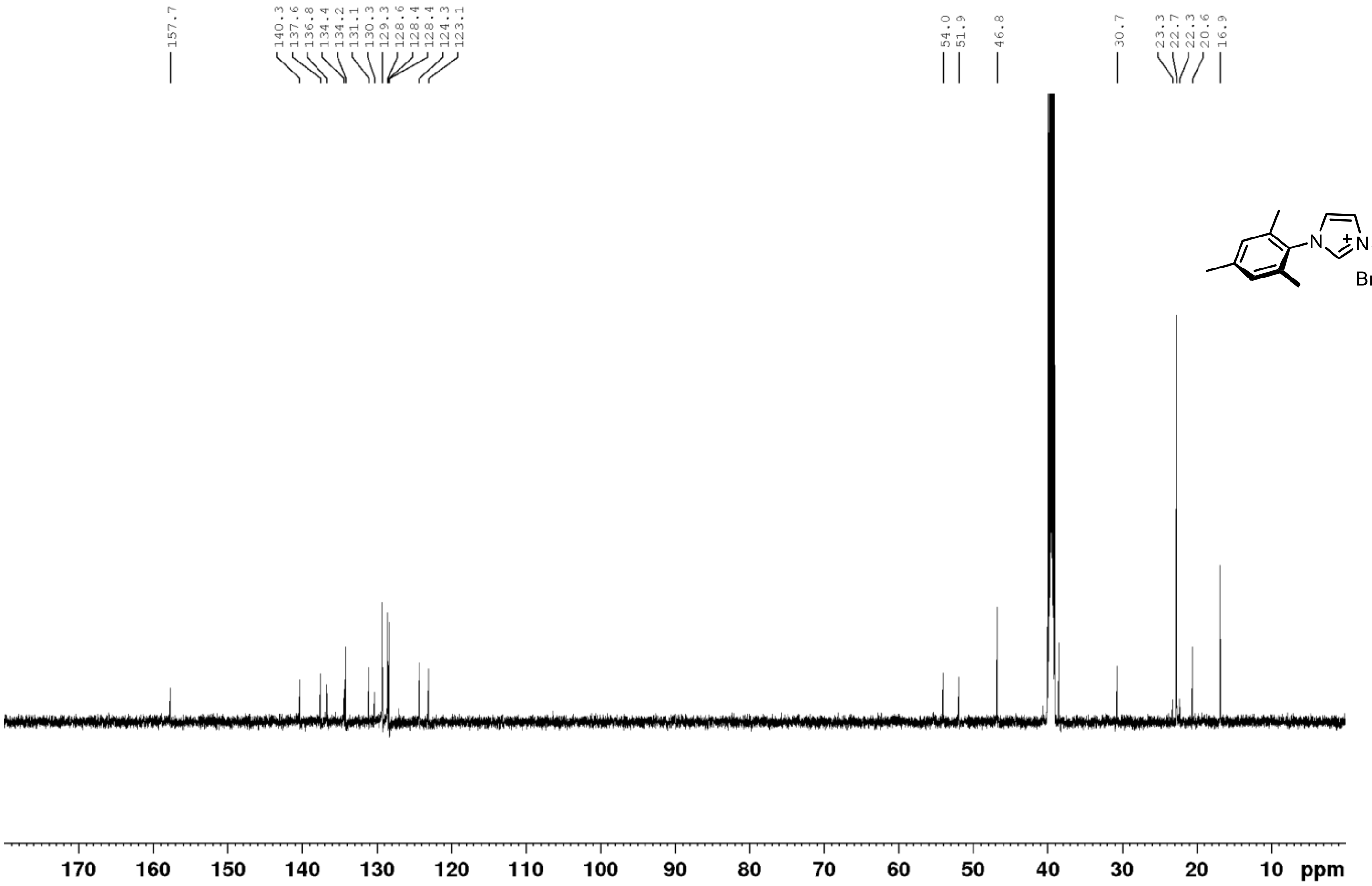
157.7
140.3
137.6
136.8
134.4
134.2
131.1
130.3
129.3
128.6
128.4
128.4
124.3
123.1
54.0
51.9
46.8
30.7
23.3
22.7
22.3
20.6
16.9
170 160 150 140 130 120 110 100 90 80 70 60 50 40 30 20 10 ppm

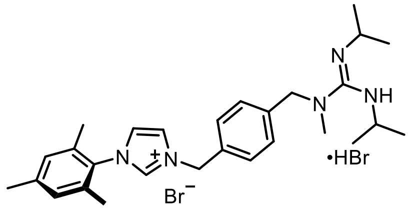
N
NH
N
N
N
Br⁻
•HBr

S81

^{1}H, ^{1}H COSY

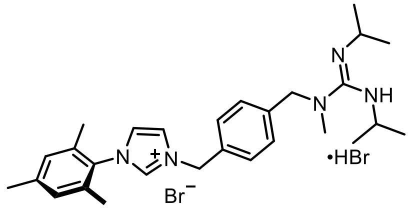

S81

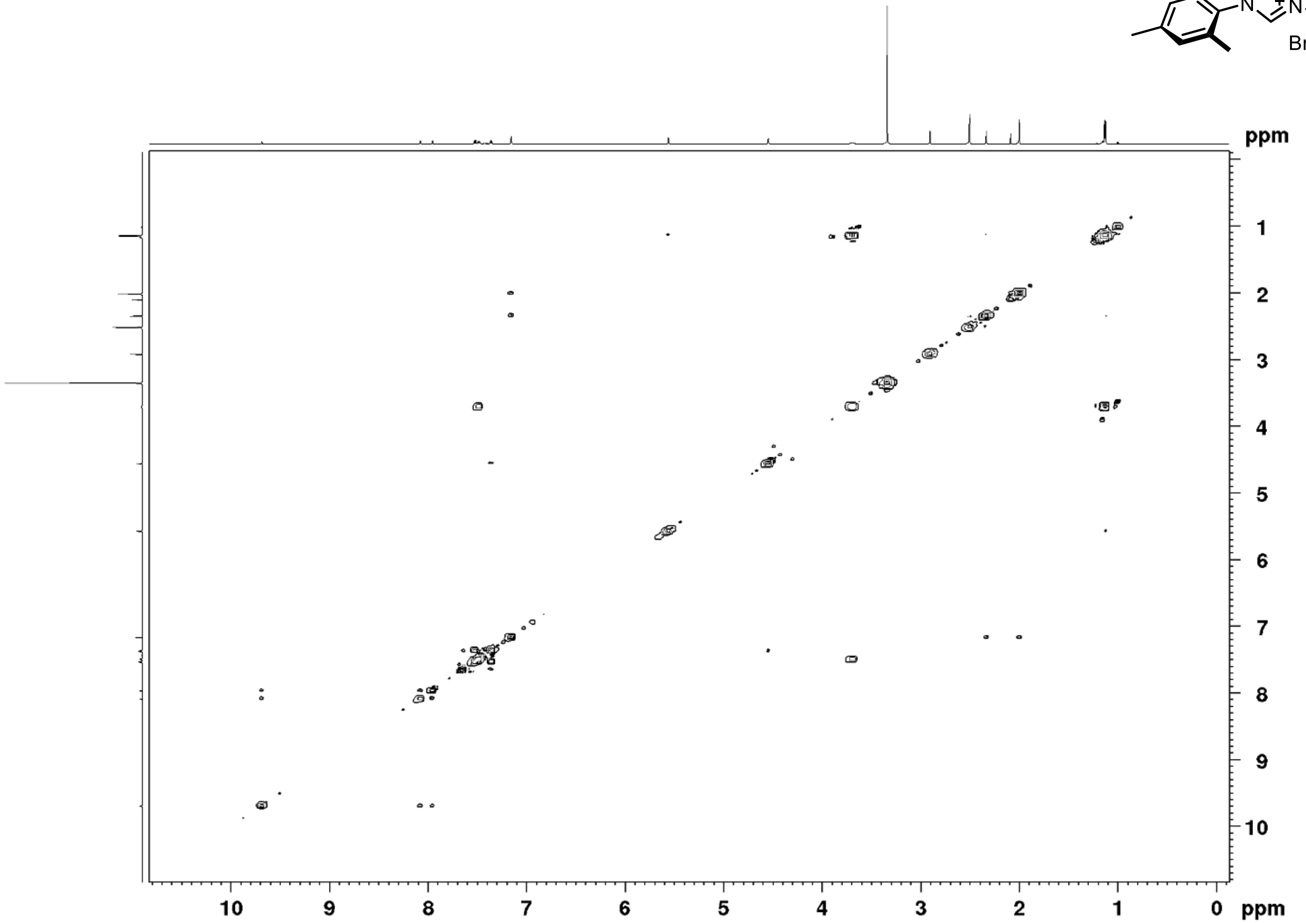

^{1}H, ^{13}C HMBC

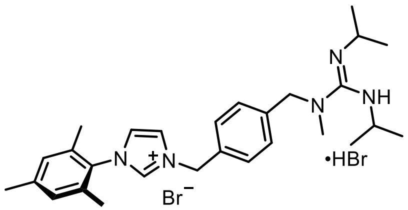

S81

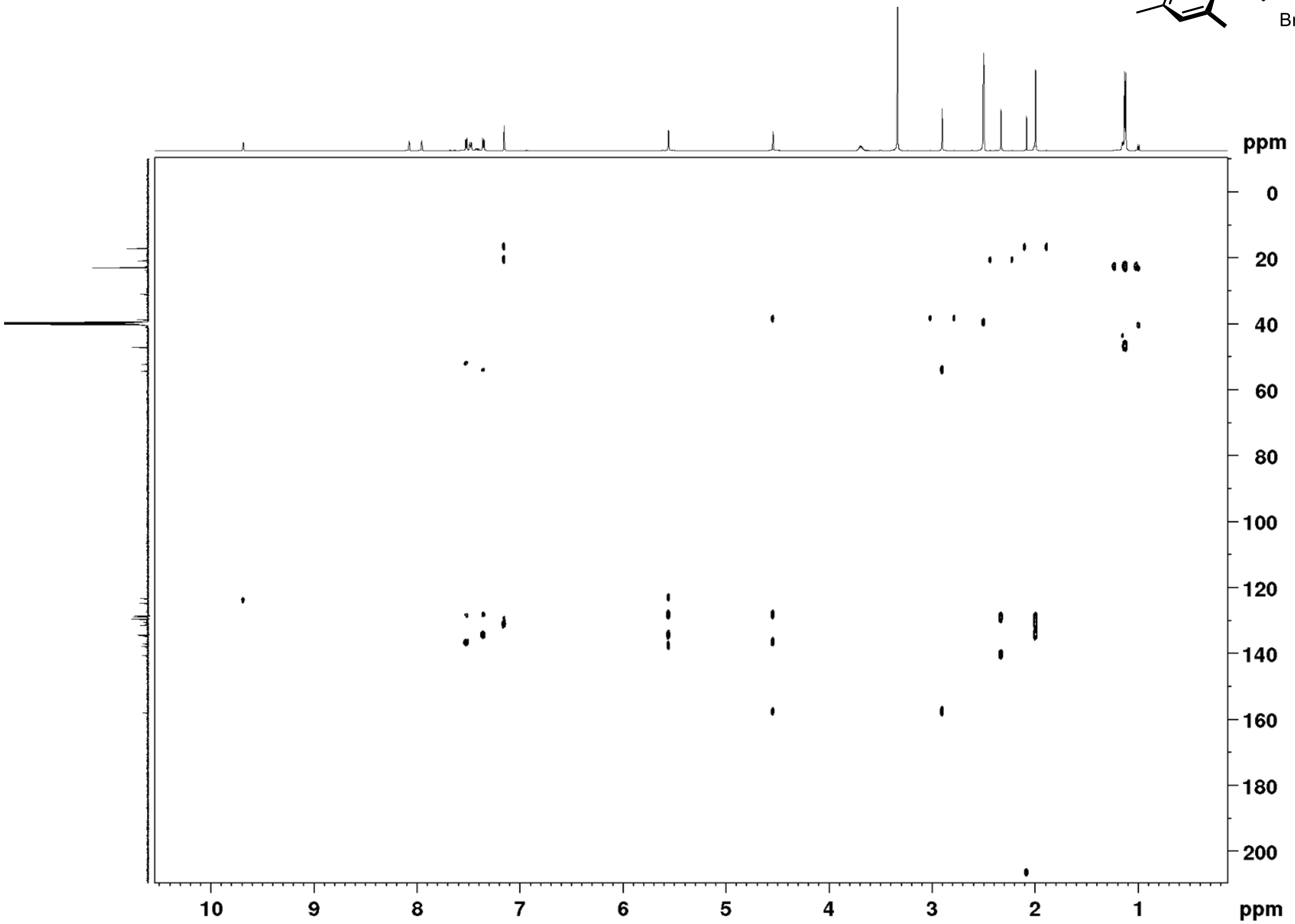

^{1}H, ^{13}C HSQC

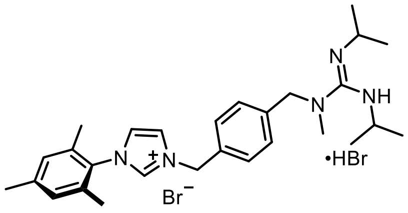

S81

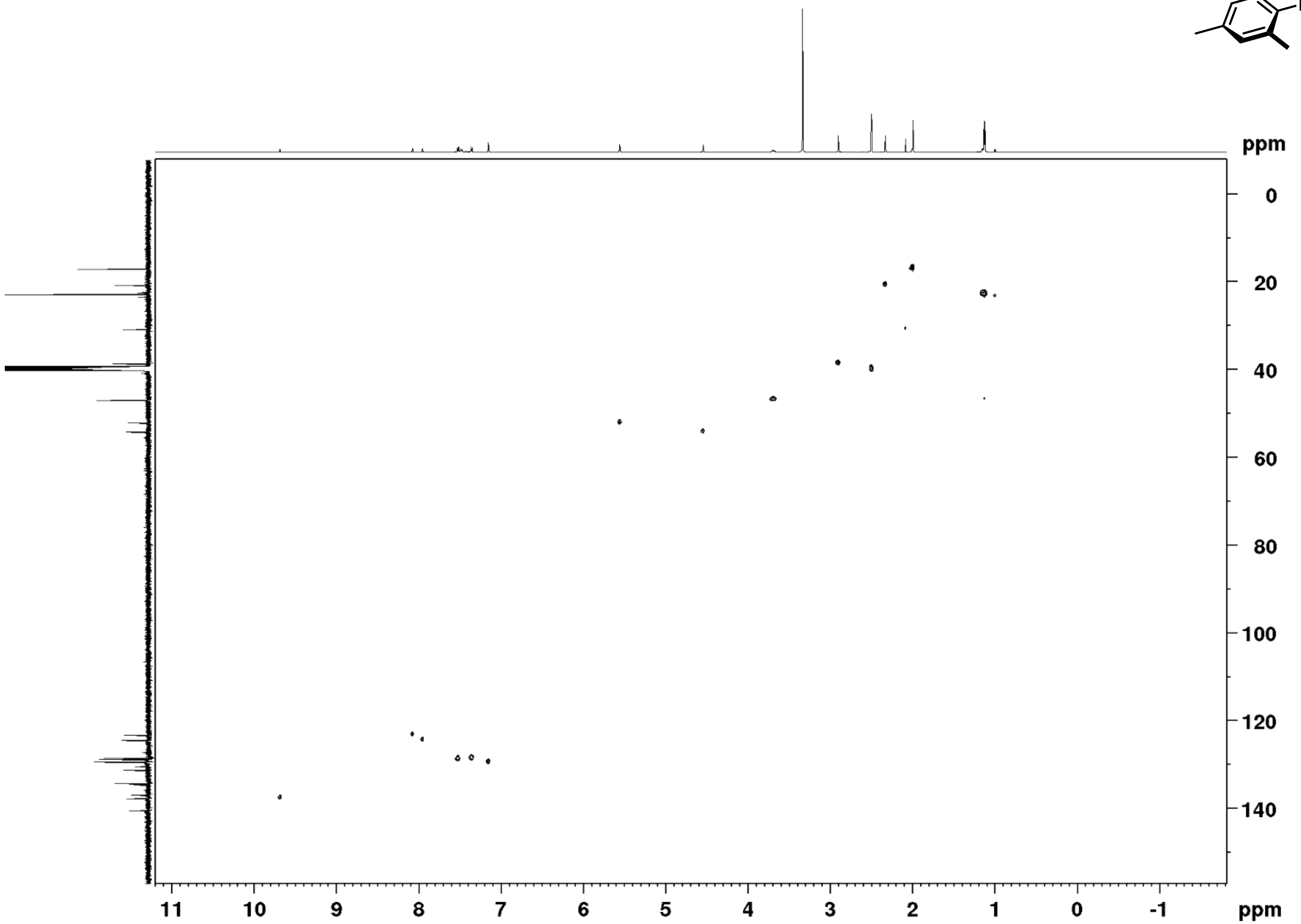

HRMS

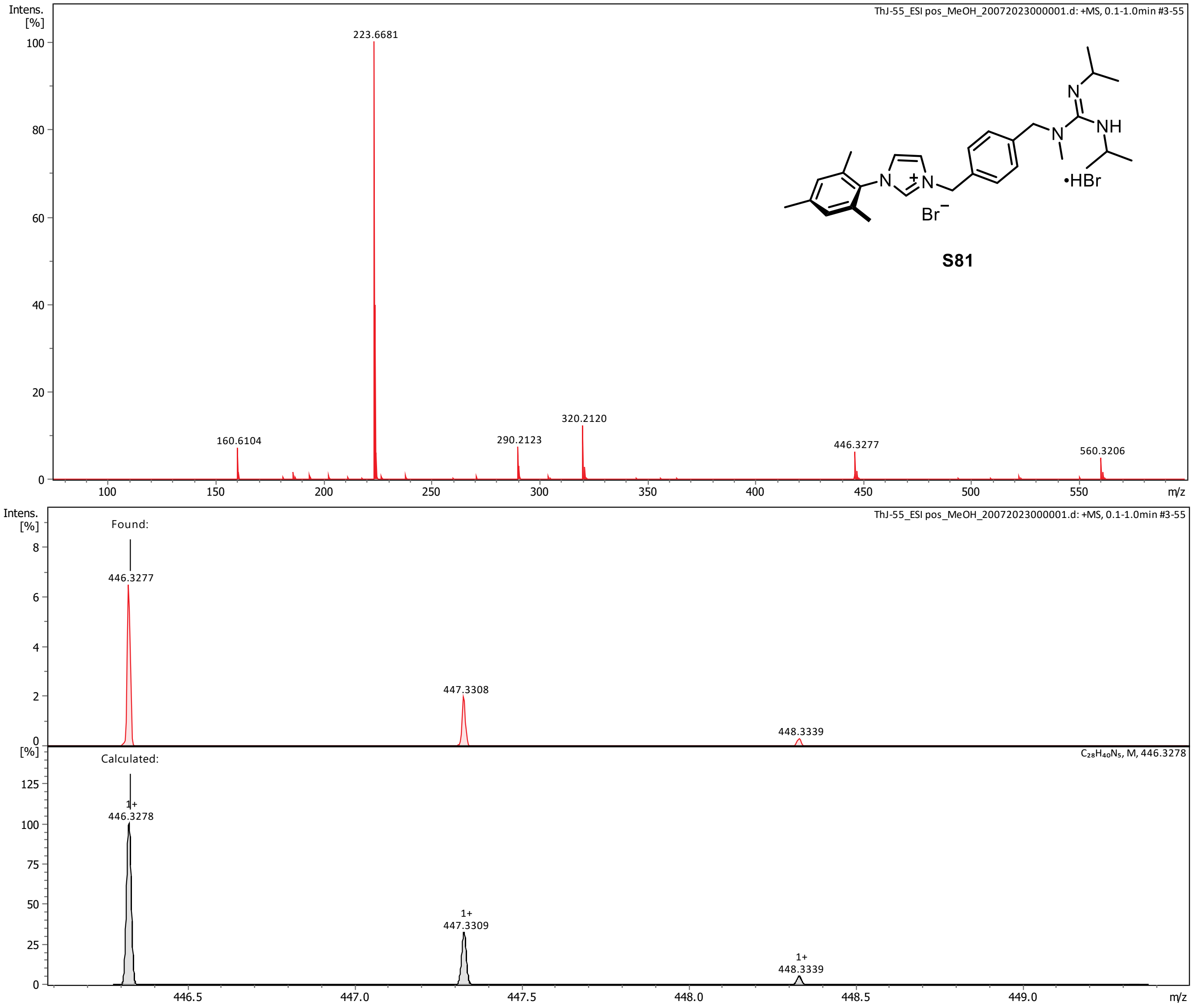

ThJ-55_ESI pos_MeOH_20072023000001.d: +MS, 0.1-1.0min #3-55
Intens. [%]
223.6681
160.6104
290.2123
320.2120
446.3277
560.3206
m/z
Found:
446.3277
447.3308
448.3339
Calculated:
C28H40N5, M, 446.3278
1+ 446.3278
1+ 447.3309
1+ 448.3339
•HBr
Br
S81

91 (*E*)-(1-(4-((2,3-Diisopropyl-1-methylguanidino)methyl)benzyl)-3-mesityl-1,3-dihydro-2*H*-imidazol-2-ylidene)copper(I) chloride (6)

^{1}H NMR

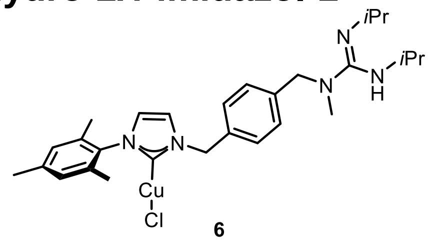

6

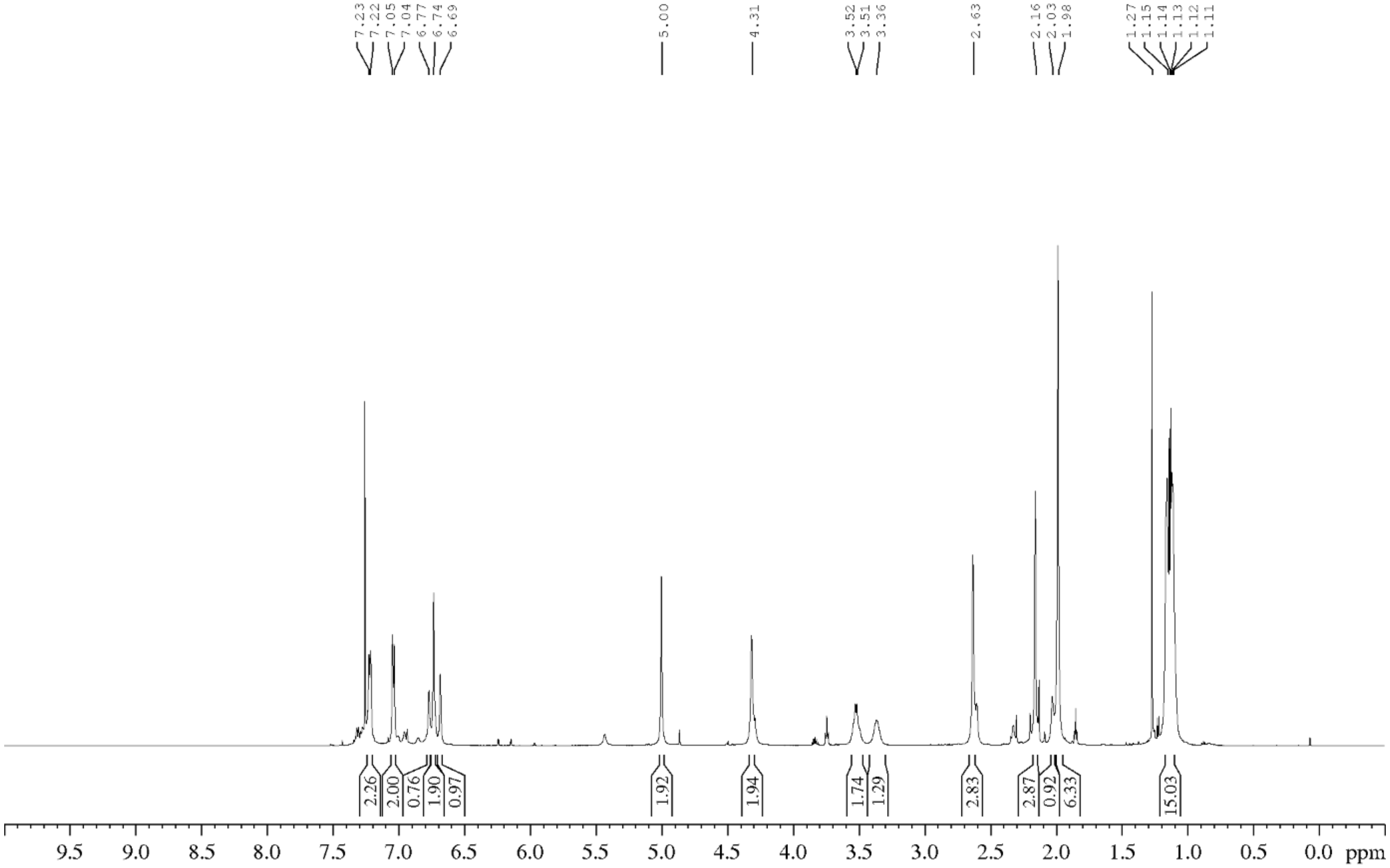

^{13}C NMR

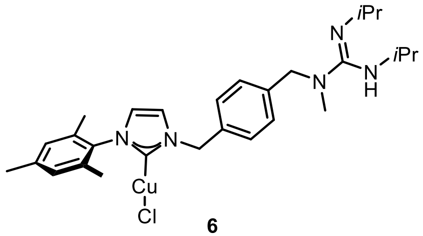

iPr
N
N
H
iPr
N
N
N
Cu
Cl

6

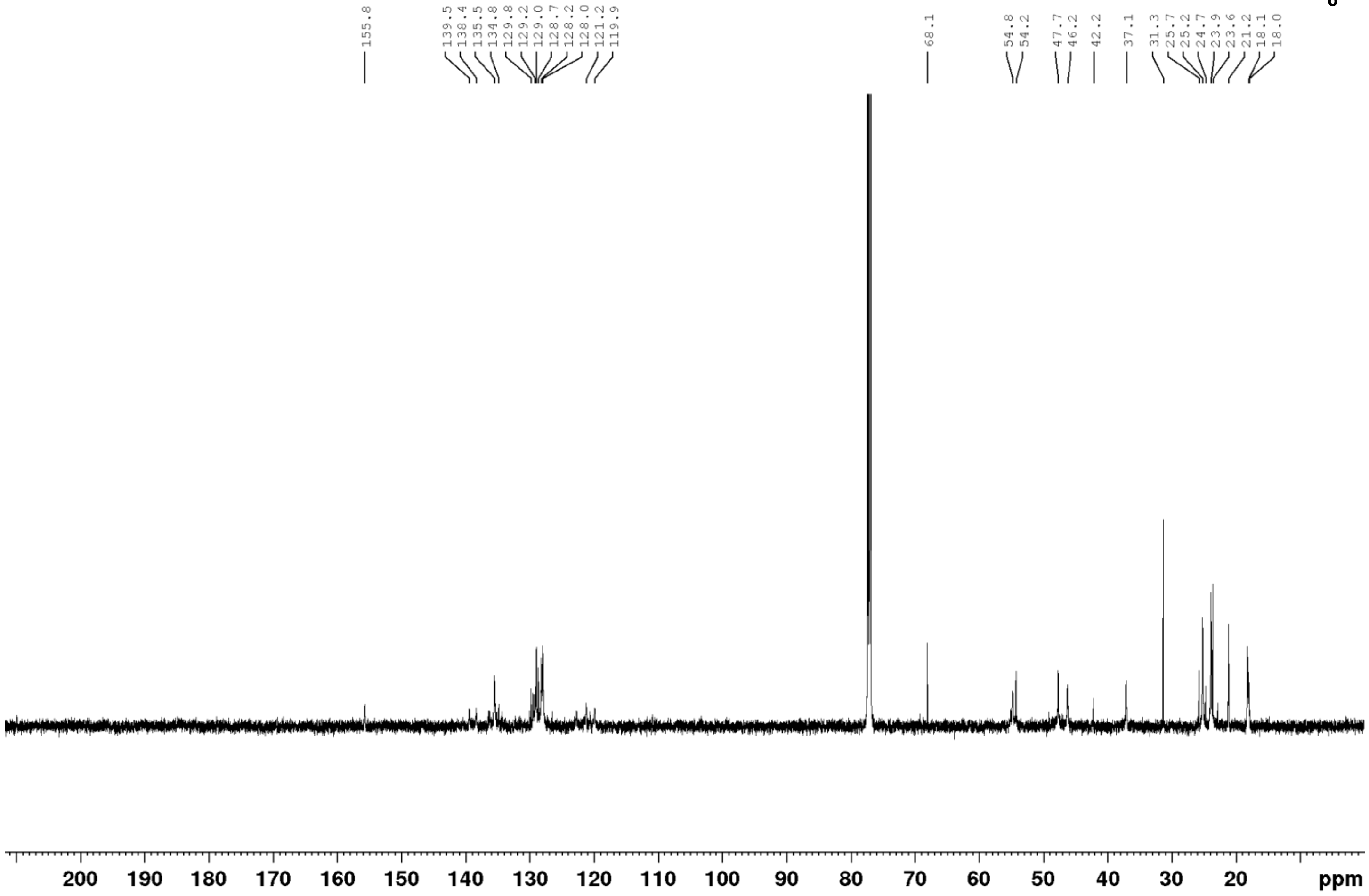

155.8
139.5
138.4
135.5
134.8
129.8
129.2
129.0
128.7
128.2
128.0
121.2
119.9
68.1
54.8
54.2
47.7
46.2
42.2
37.1
31.3
25.7
25.2
24.7
23.9
23.6
21.2
18.1
18.0
200 190 180 170 160 150 140 130 120 110 100 90 80 70 60 50 40 30 20 ppm

^{1}H, ^{1}H COSY

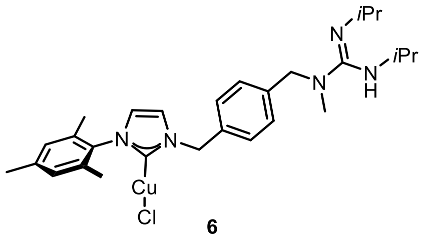

iPr
N
N
NH
H
iPr
N
N
Cu
Cl

6

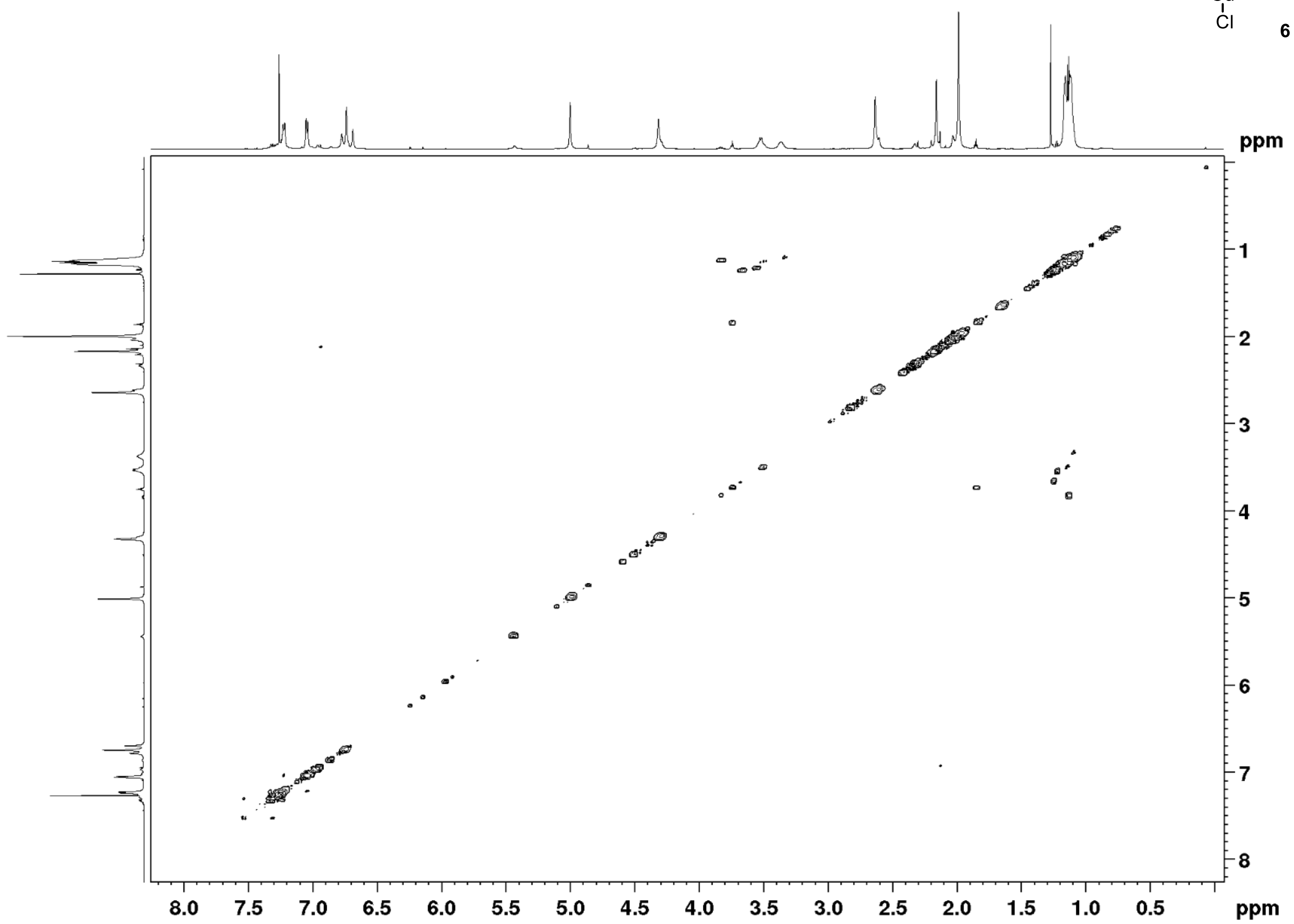

ppm
8.0
7.5
7.0
6.5
6.0
5.5
5.0
4.5
4.0
3.5
3.0
2.5
2.0
1.5
1.0
0.5
ppm
1
2
3
4
5
6
7
8

^1H, ^{13}C HMBC

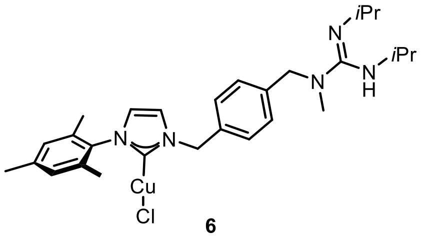

6

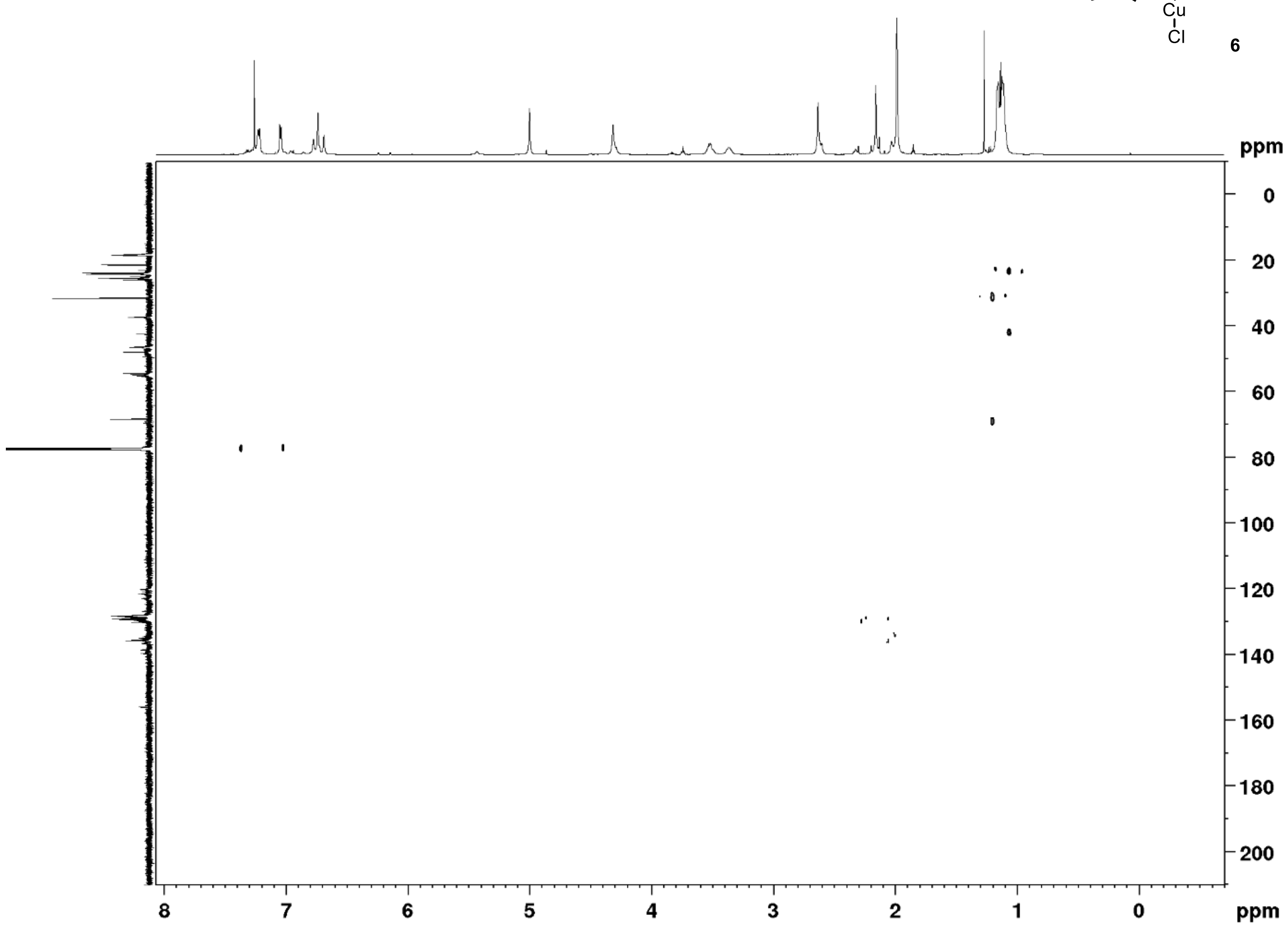

^{1}H, ^{13}C HSQC

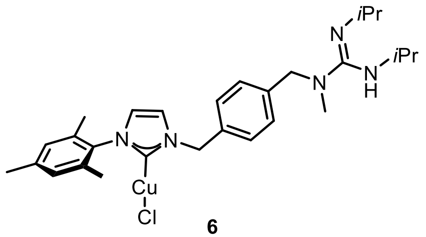
N
iPr
NH
iPr
N
N
N
Cu
Cl

6

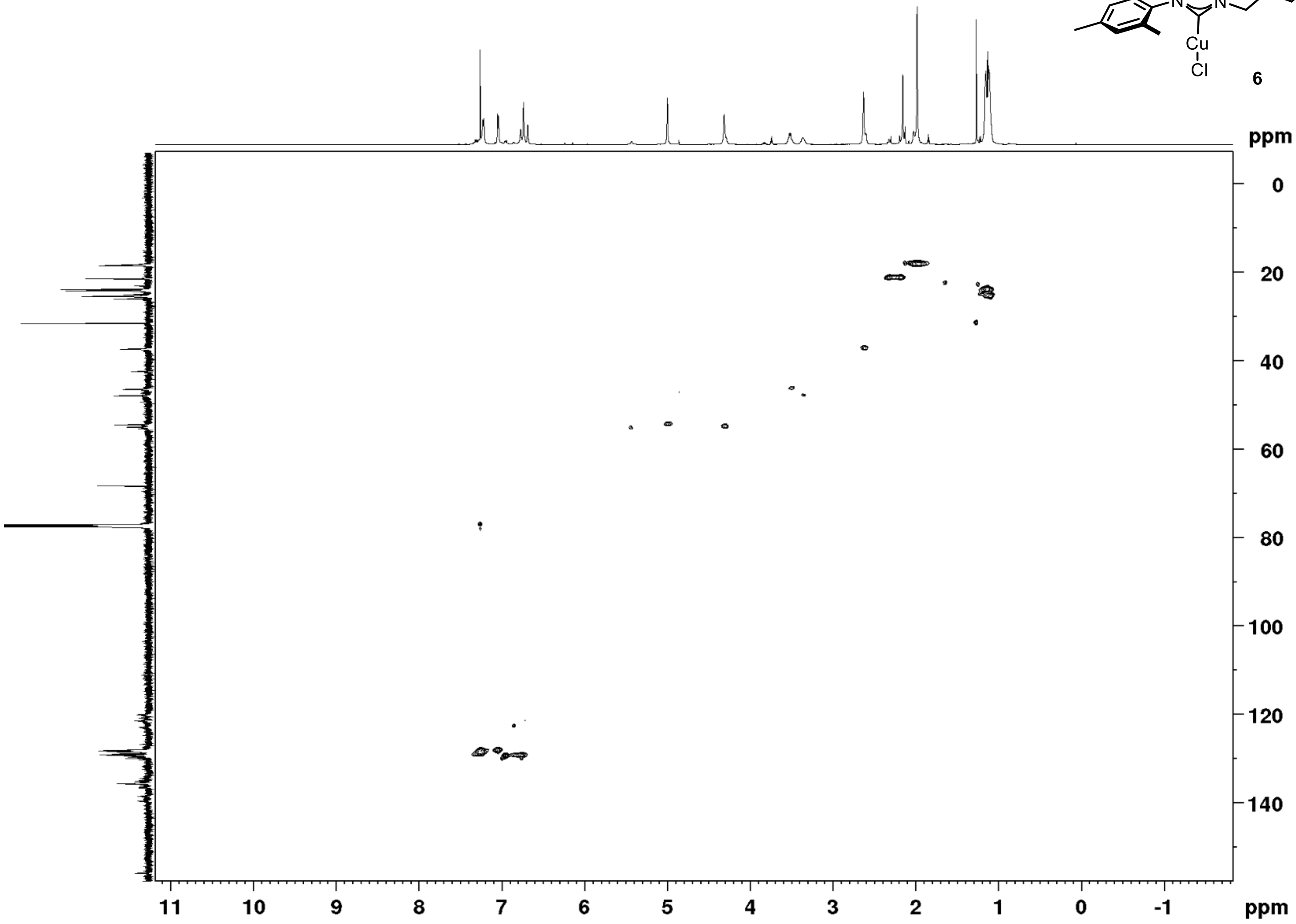
ppm
0
20
40
60
80
100
120
140
11 10 9 8 7 6 5 4 3 2 1 0 -1
ppm

HRMS

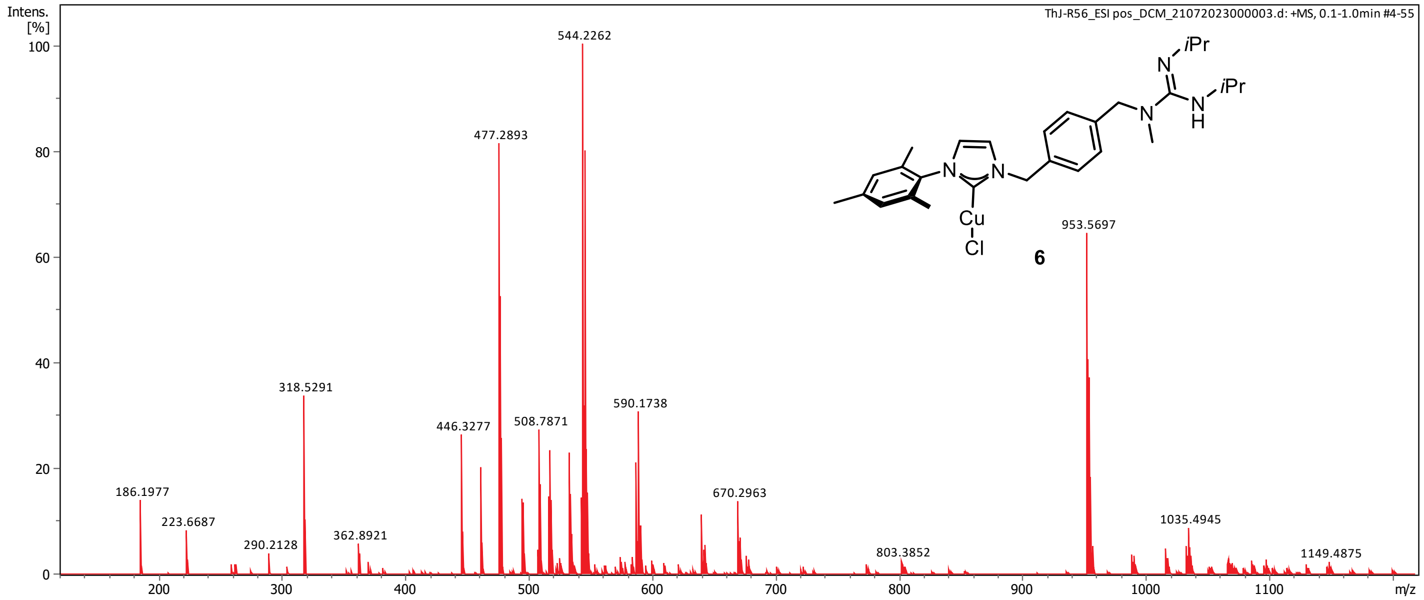

Intens. [%]
ThJ-R56_ESI pos_DCM_21072023000003.d: +MS, 0.1-1.0min #4-55
544.2262
477.2893
953.5697
318.5291
446.3277
508.7871
590.1738
670.2963
186.1977
223.6687
290.2128
362.8921
803.3852
1035.4945
1149.4875
6
m/z

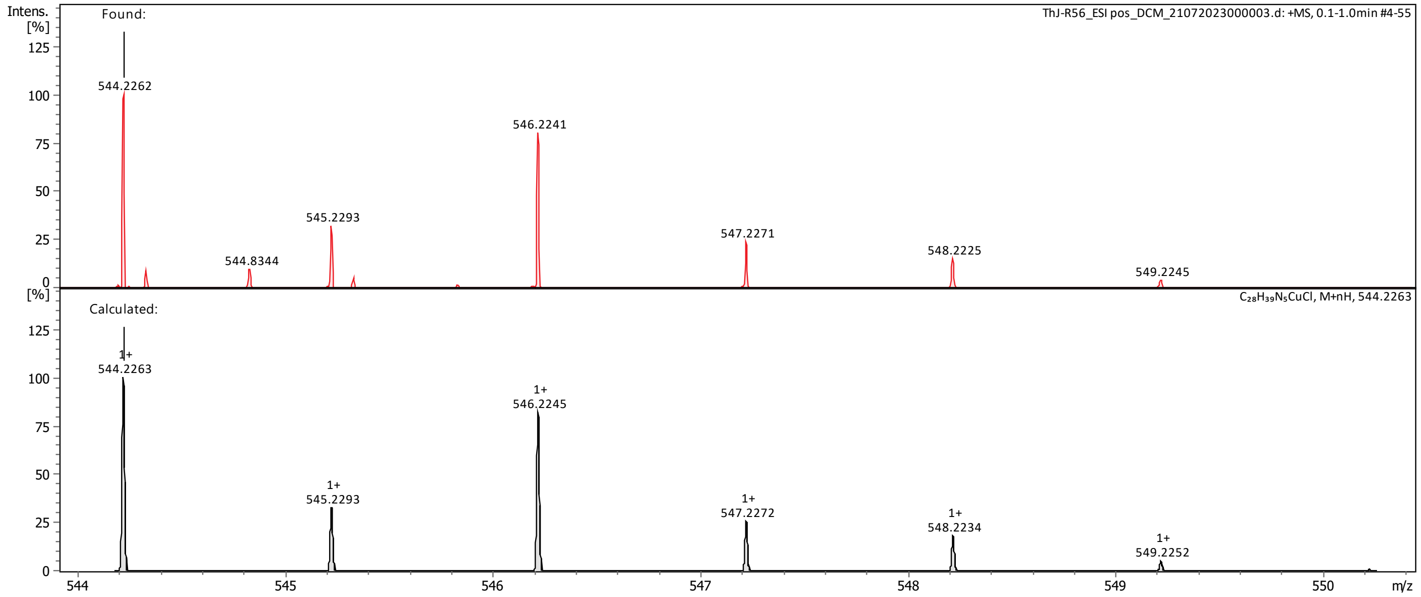

Intens. [%]
Found:
ThJ-R56_ESI pos_DCM_21072023000003.d: +MS, 0.1-1.0min #4-55
544.2262
546.2241
545.2293
544.8344
547.2271
548.2225
549.2245
Calculated:
C28H39N5CuCl, M+nH, 544.2263
1+ 544.2263
1+ 546.2245
1+ 545.2293
1+ 547.2272
1+ 548.2234
1+ 549.2252
m/z

92 Methyl 6-(methylamino)-6-oxohexanoate (S83)

^{1}H NMR

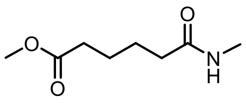

S83

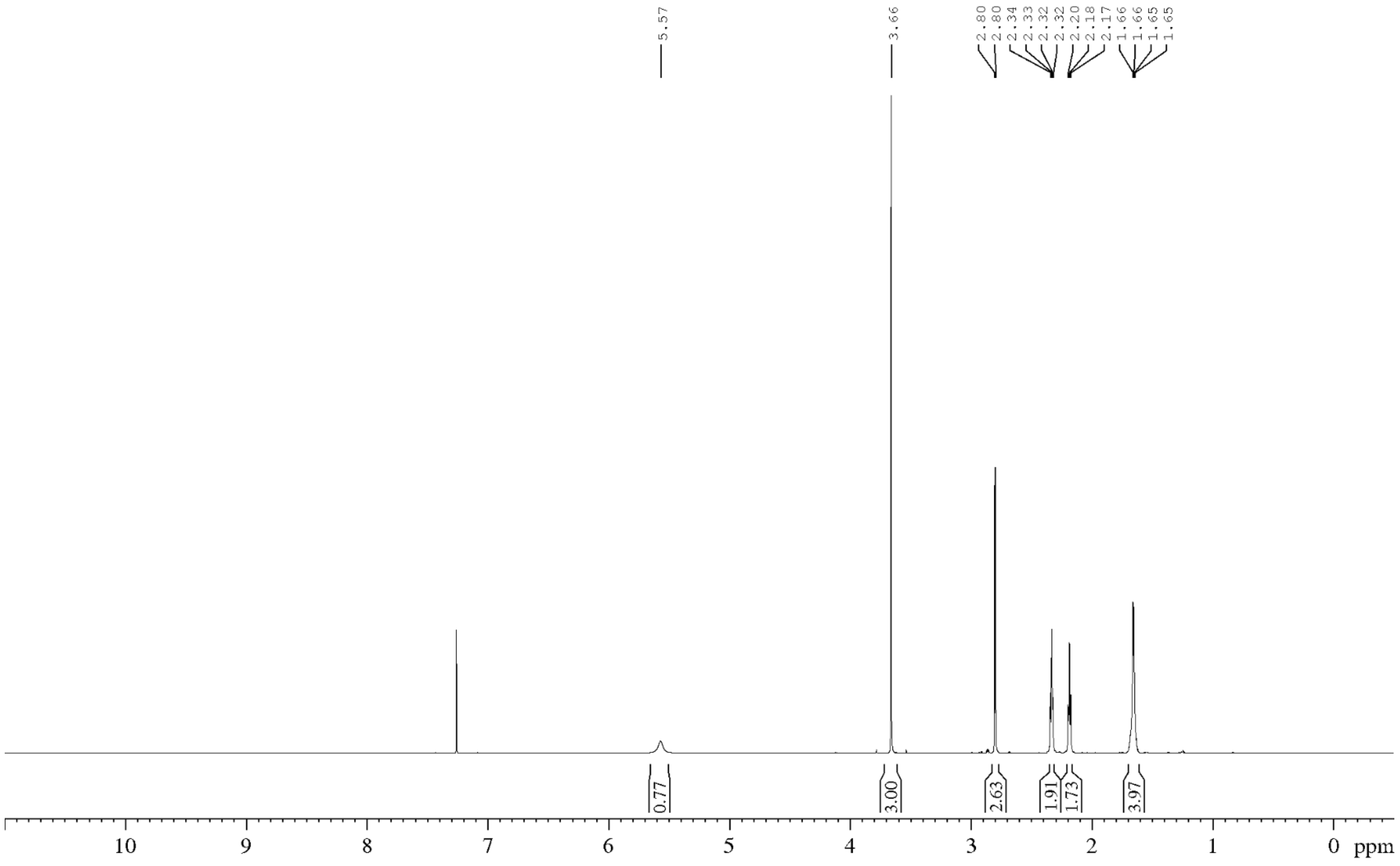

^{13}C NMR

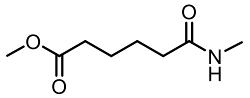

S83

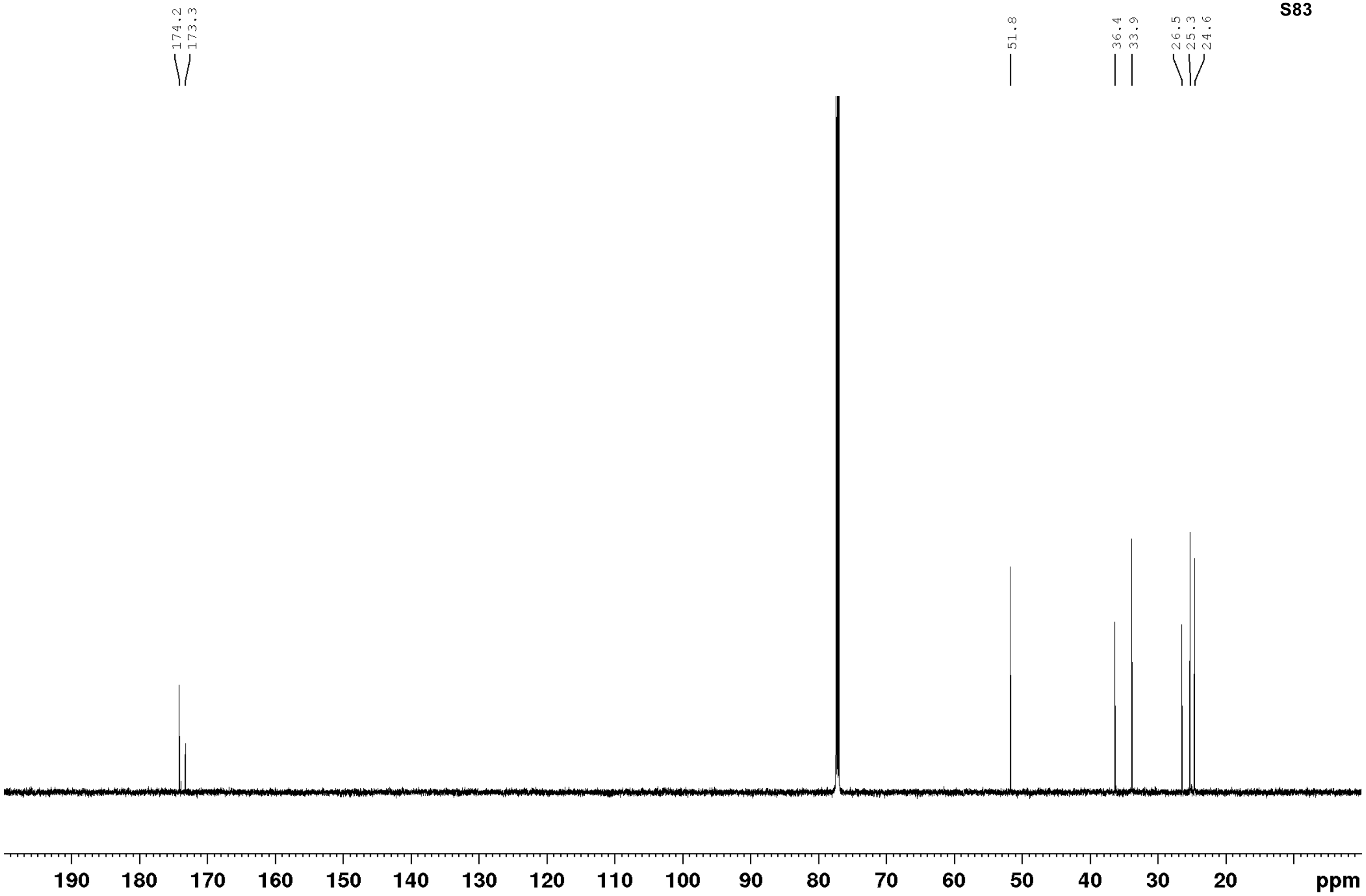
174.2
173.3
51.8
36.4
33.9
26.5
25.3
24.6
190
180
170
160
150
140
130
120
110
100
90
80
70
60
50
40
30
20
ppm

^{1}H, ^{1}H COSY

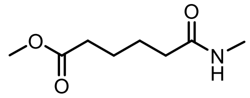

S83

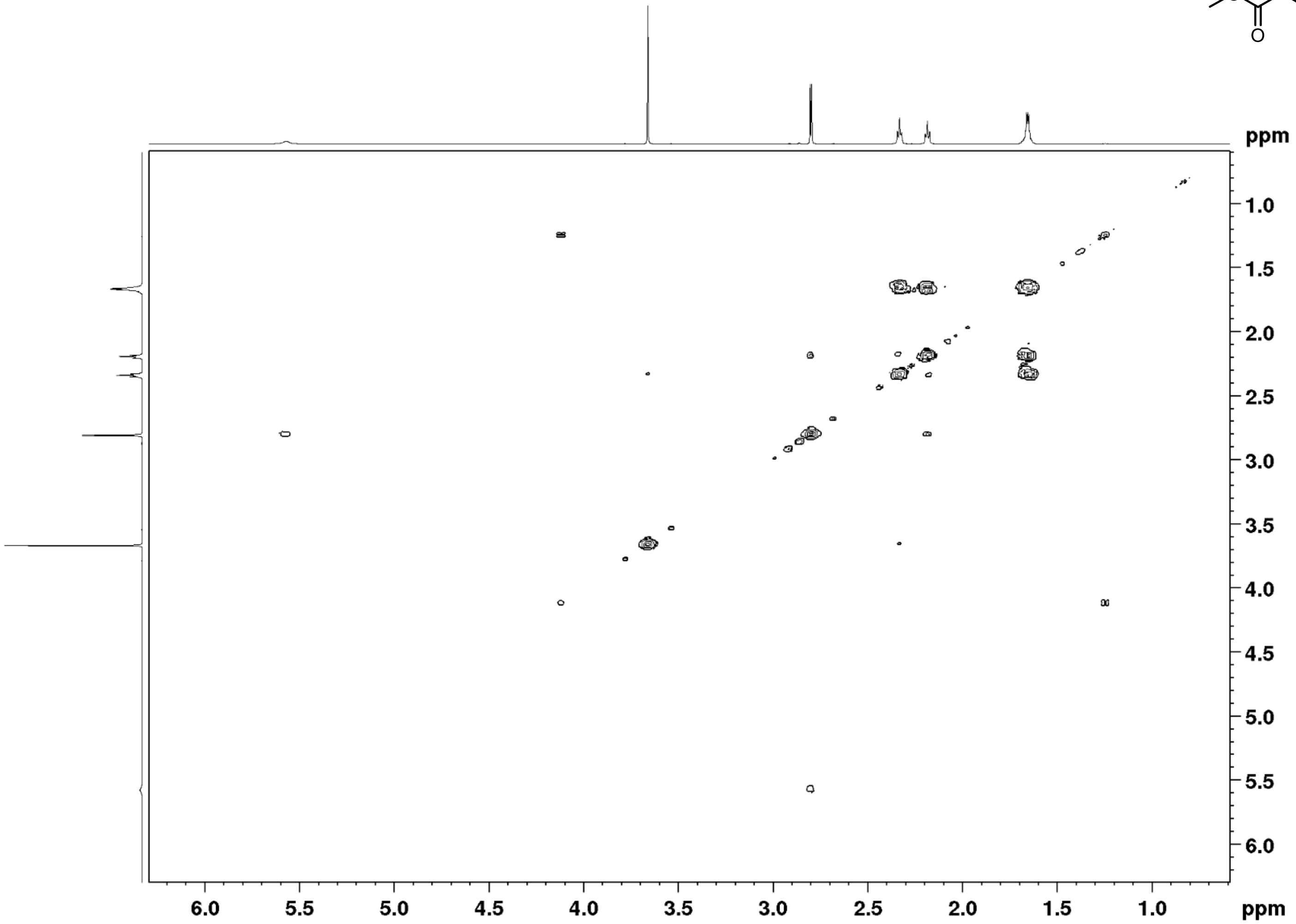

^{1}H, ^{13}C HMBC

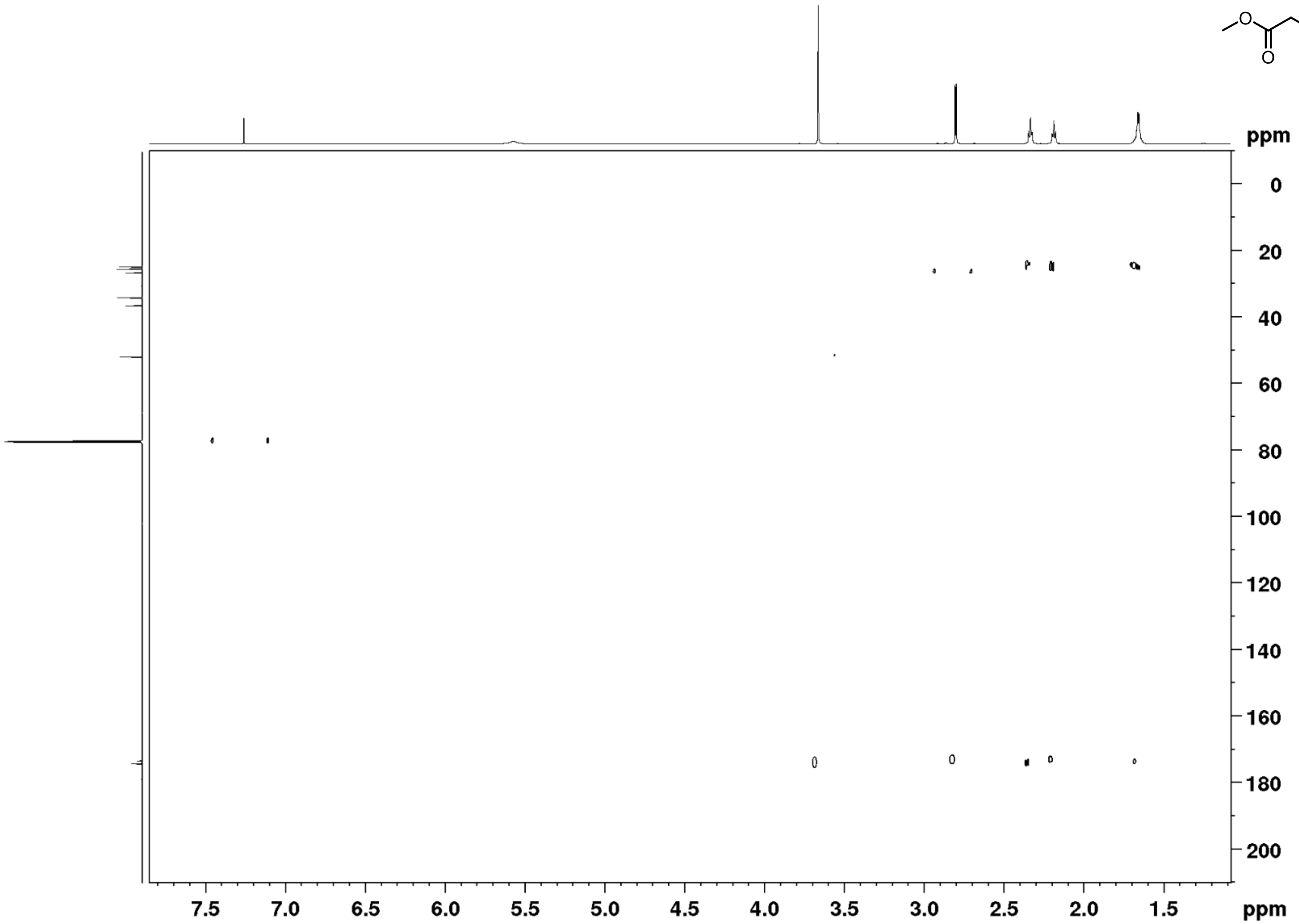

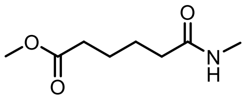

S83

^{1}H, ^{13}C HSQC

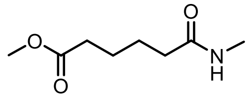

S83

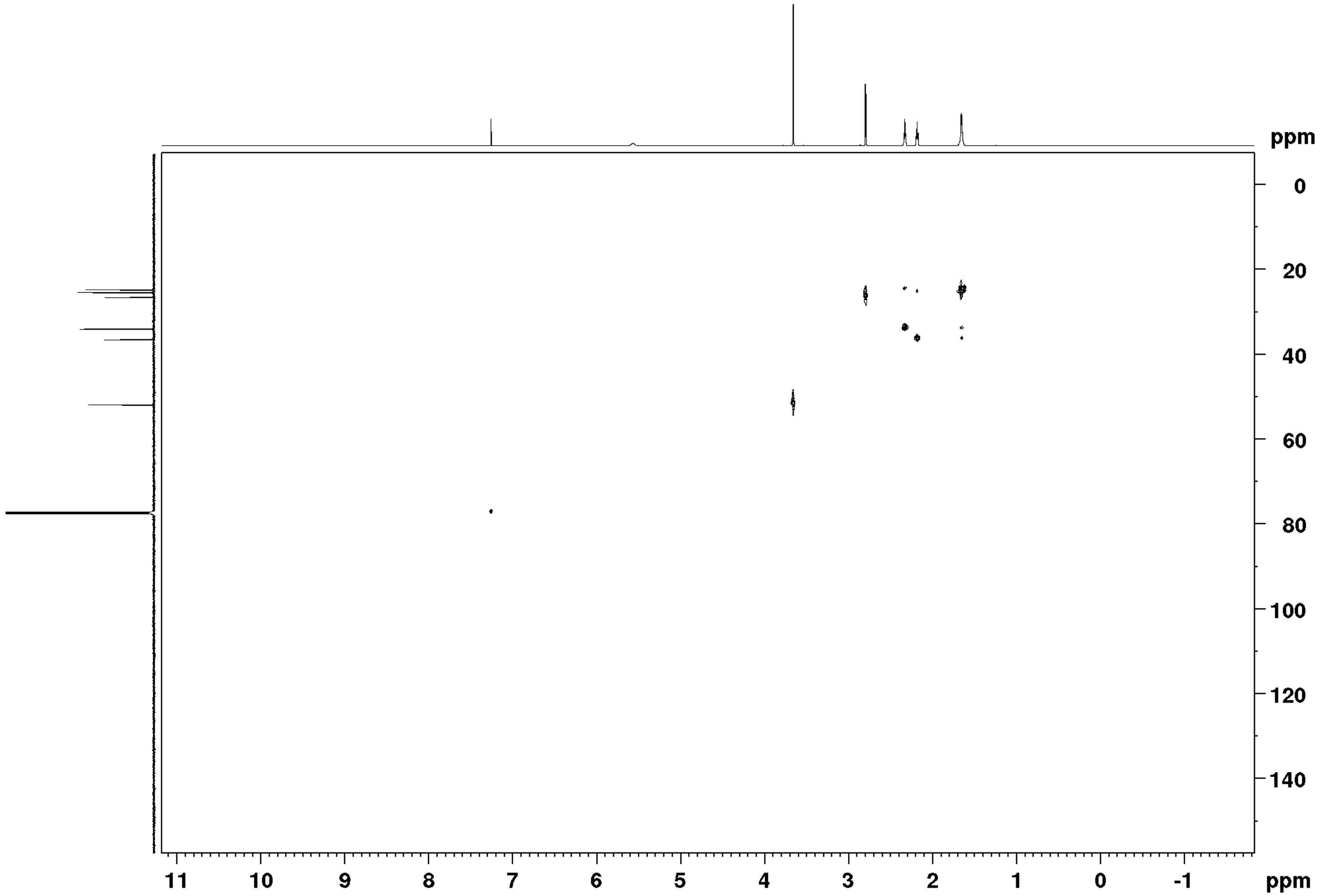

HRMS

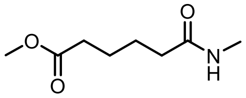

S83

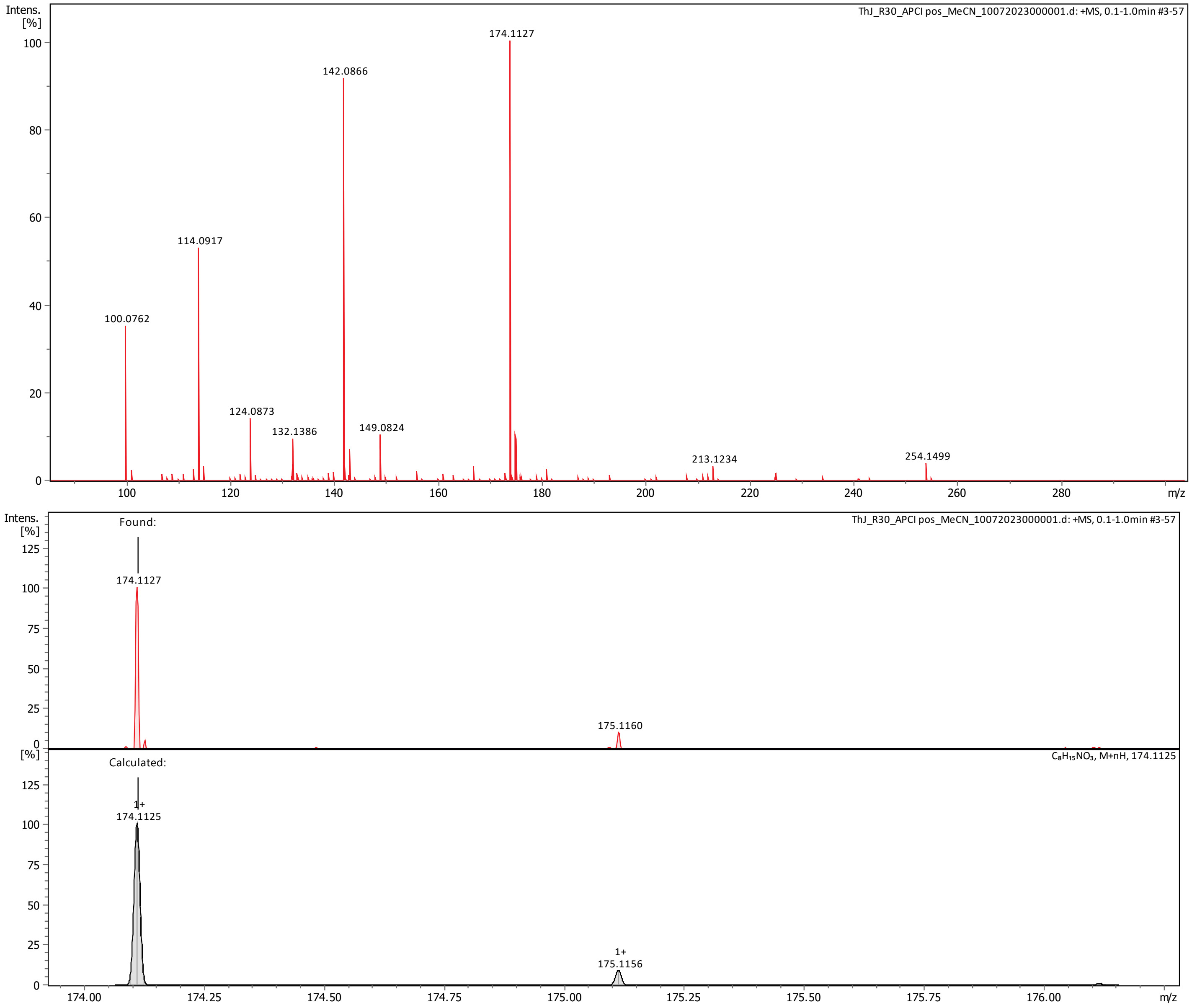

Intens. [%]
ThJ_R30_APCI pos_MeCN_10072023000001.d: +MS, 0.1-1.0min #3-57
174.1127
142.0866
114.0917
100.0762
124.0873
132.1386
149.0824
213.1234
254.1499
m/z
Found:
174.1127
175.1160
Calculated:
$C_8H_{15}NO_3$, M+nH, 174.1125
1+
174.1125
1+
175.1156

IR

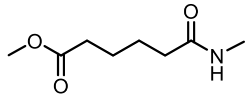
O
O
O
N
H

S83

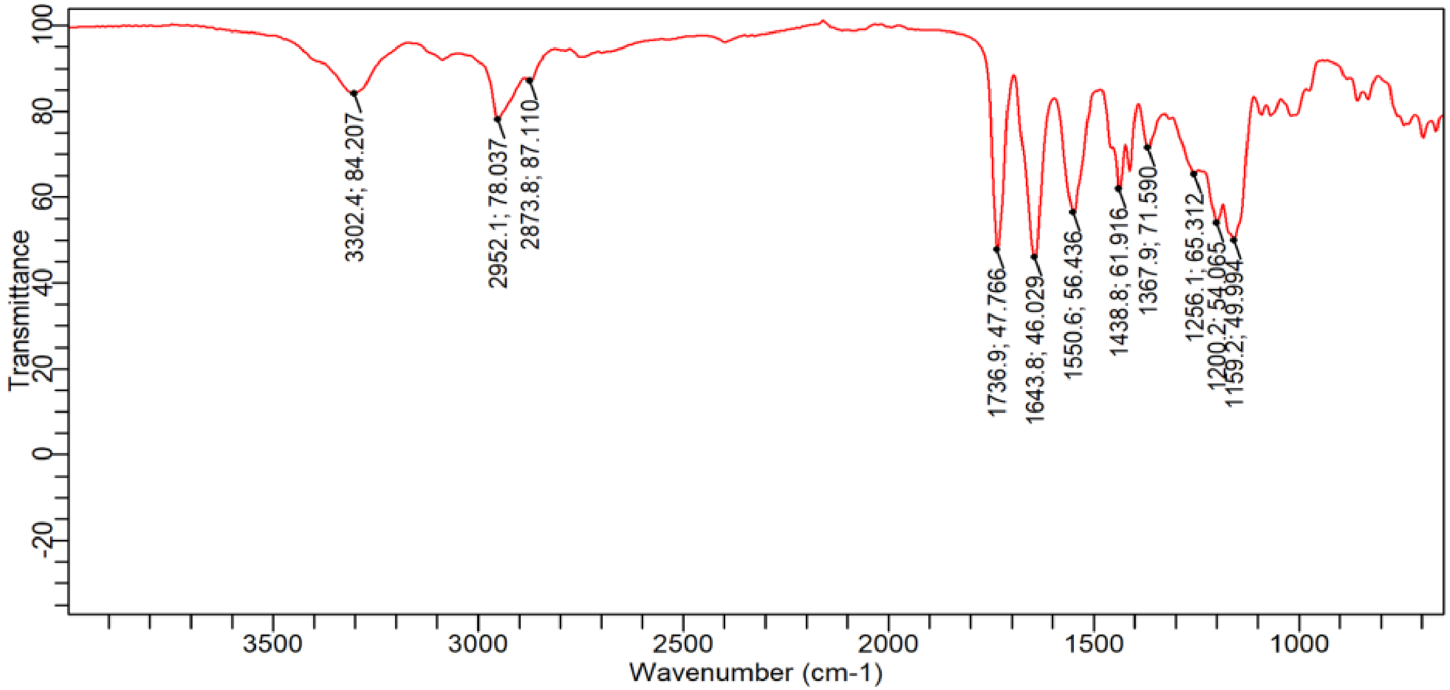
Transmittance
Wavenumber (cm-1)
3302.4; 84.207
2952.1; 78.037
2873.8; 87.110
1736.9; 47.766
1643.8; 46.029
1550.6; 56.436
1438.8; 61.916
1367.9; 71.590
1256.1; 65.312
1200.2; 54.065
1159.2; 49.994

93 6-(Methylamino)hexan-1-ol (S84)

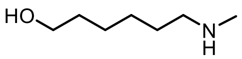

S84

^1H NMR

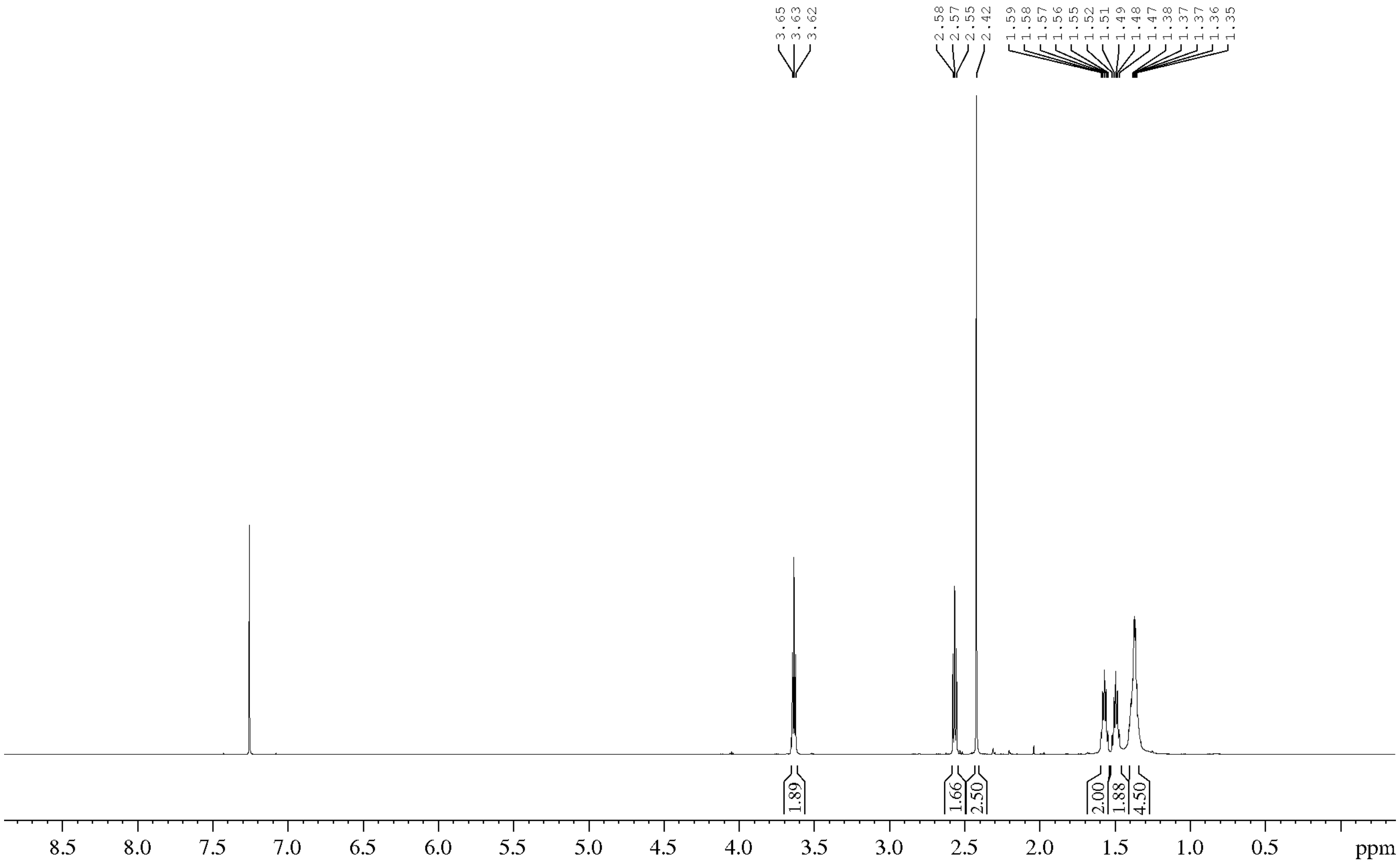

^{13}C NMR

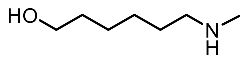

S84

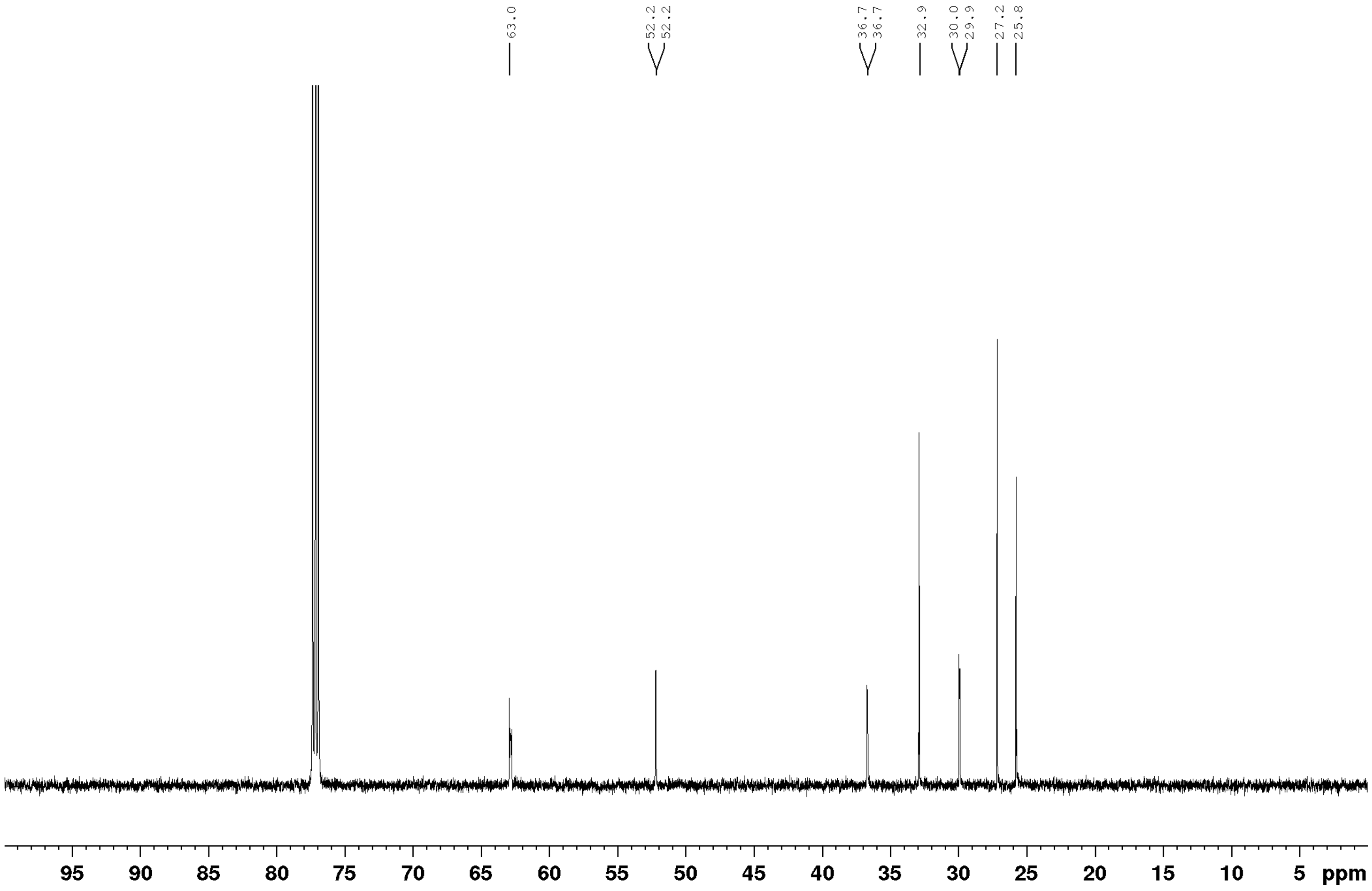
63.0
52.2
52.2
36.7
36.7
32.9
30.0
29.9
27.2
25.8
95 90 85 80 75 70 65 60 55 50 45 40 35 30 25 20 15 10 5 ppm

^1H, ^1H COSY

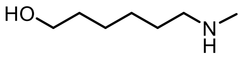

S84

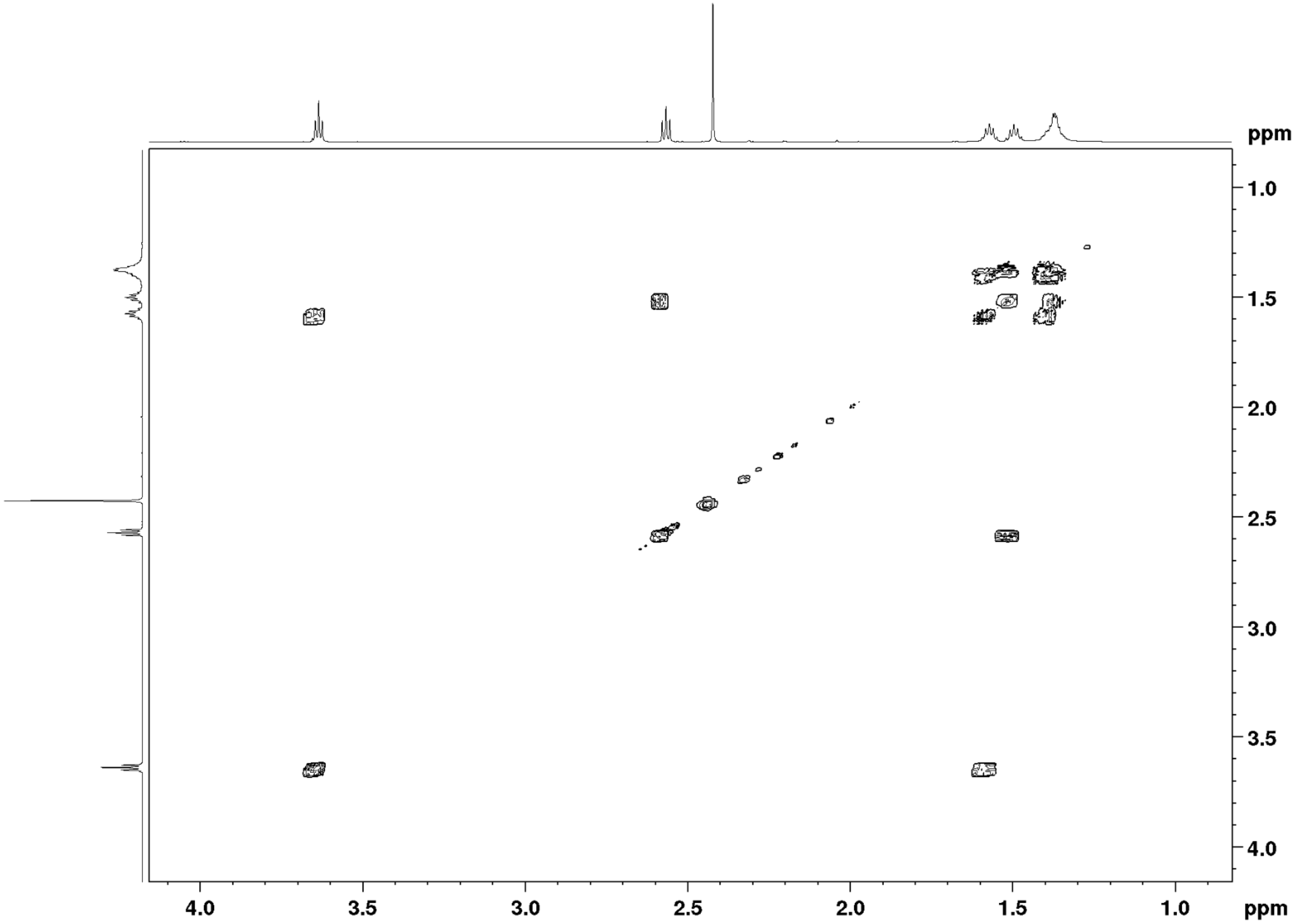

^{1}H, ^{13}C HMBC

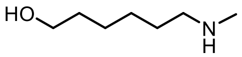

S84

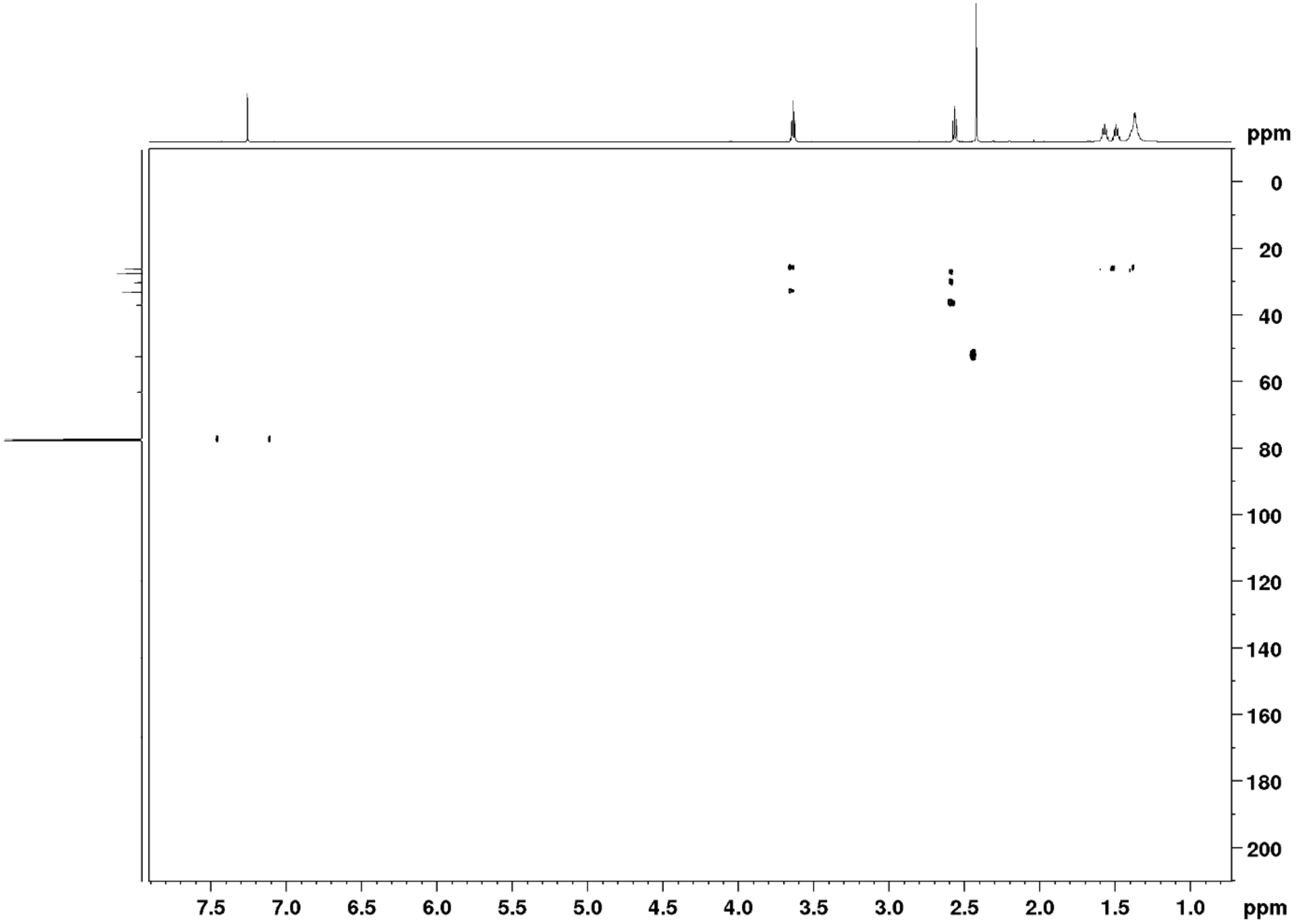

^{1}H, ^{13}C HSQC

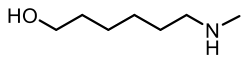

S84

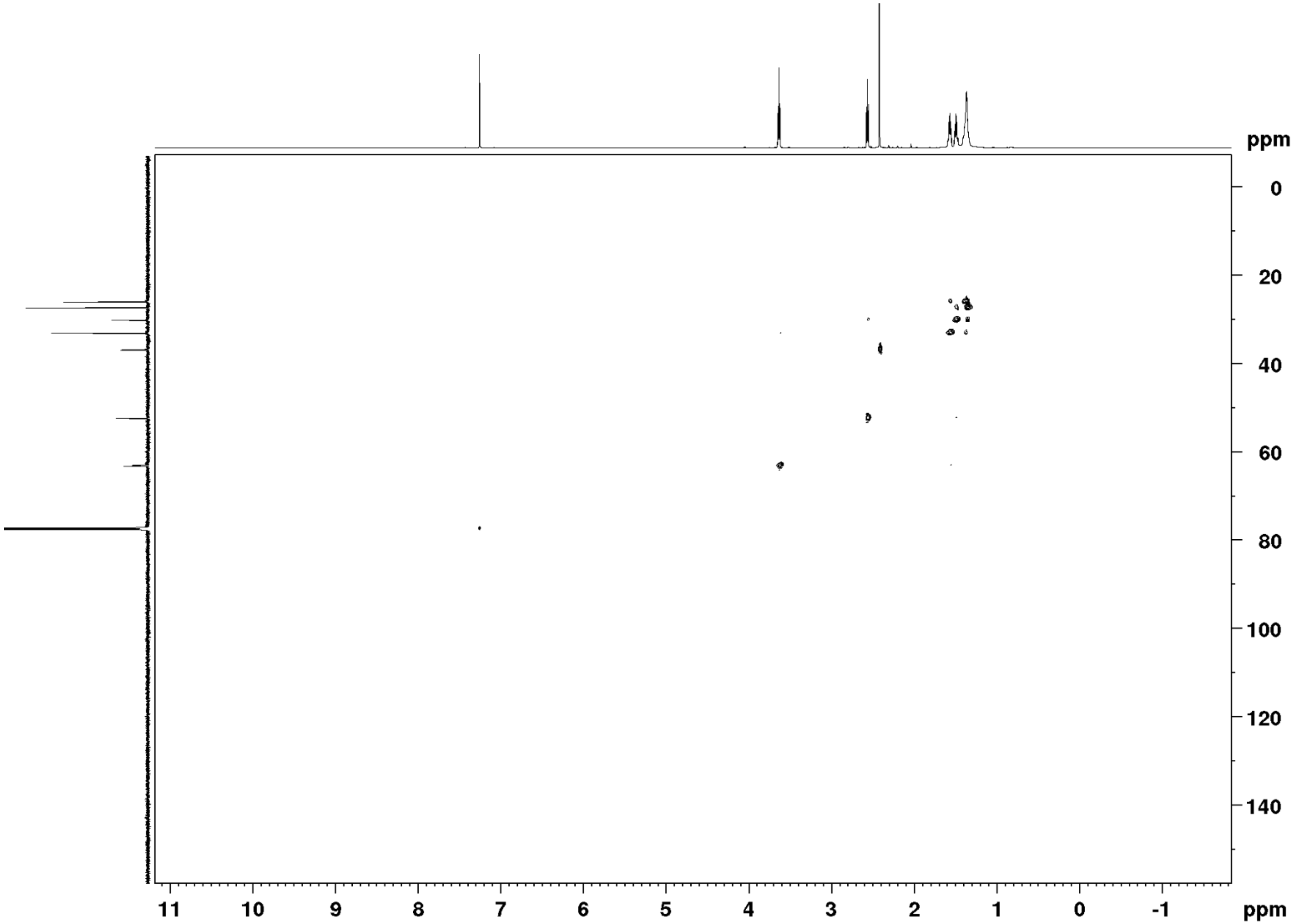

HRMS

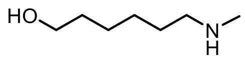

S84

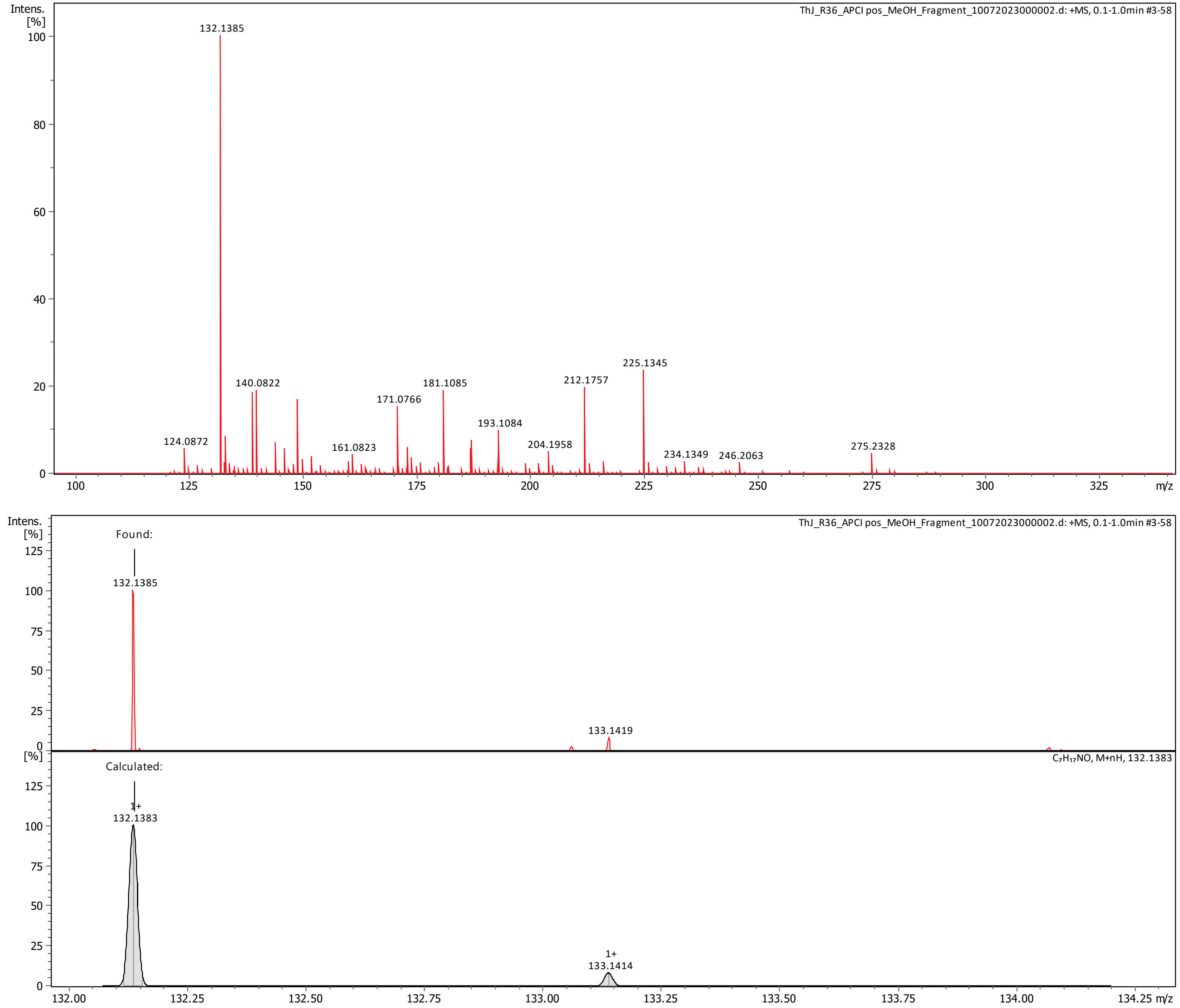

Intens. [%]
ThJ_R36_APCI pos_MeOH_Fragment_10072023000002.d: +MS, 0.1-1.0min #3-58
132.1385
140.0822
124.0872
161.0823
171.0766
181.1085
193.1084
204.1958
212.1757
225.1345
234.1349
246.2063
275.2328
m/z
Found:
132.1385
133.1419
Calculated:
$C_7H_{17}NO$, M+nH, 132.1383
1+
132.1383
1+
133.1414

IR

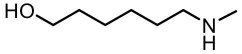
HO
N
H

S84

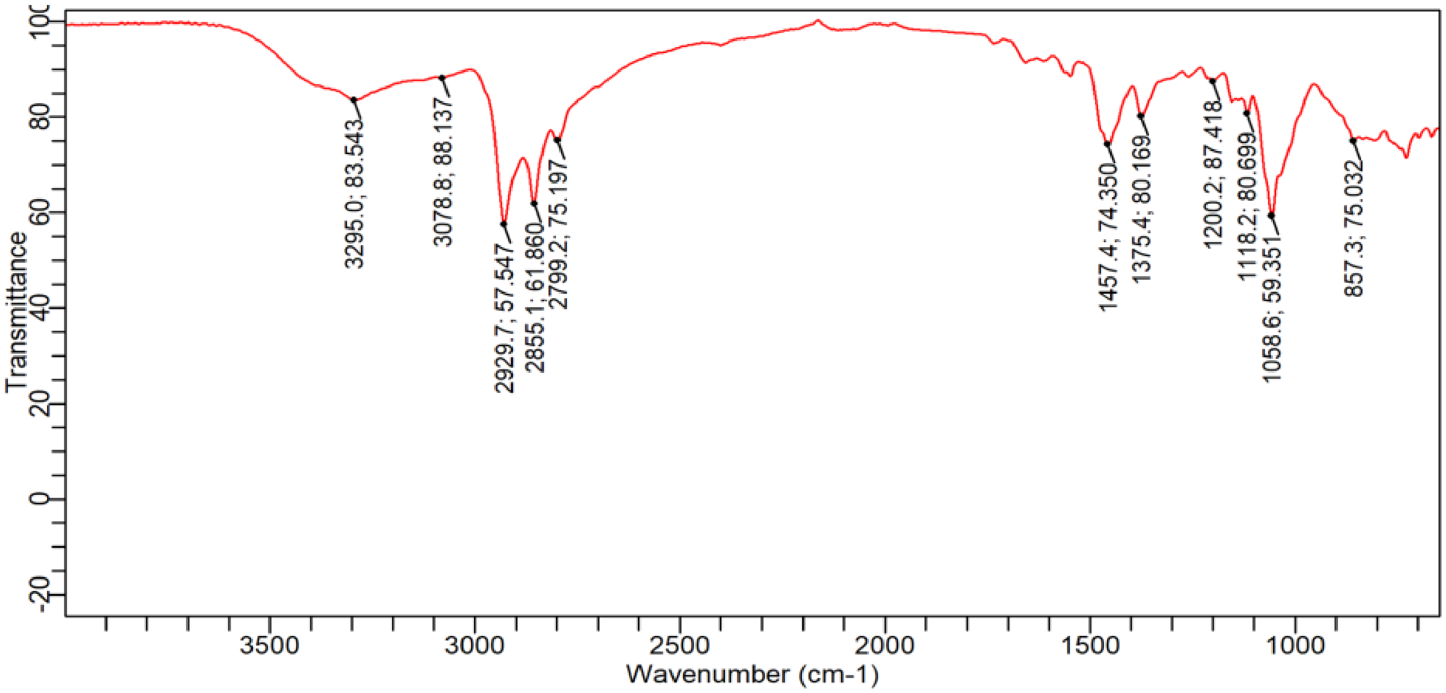
Transmittance
Wavenumber (cm-1)
3295.0; 83.543
3078.8; 88.137
2929.7; 57.547
2855.1; 61.860
2799.2; 75.197
1457.4; 74.350
1375.4; 80.169
1200.2; 87.418
1118.2; 80.699
1058.6; 59.351
857.3; 75.032
3500
3000
2500
2000
1500
1000
100
80
60
40
20
0
-20

94 6-Bromo-*N*-methylhexan-1-amine hydrobromide (S85)

^{1}H NMR

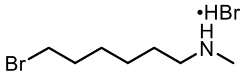

S85

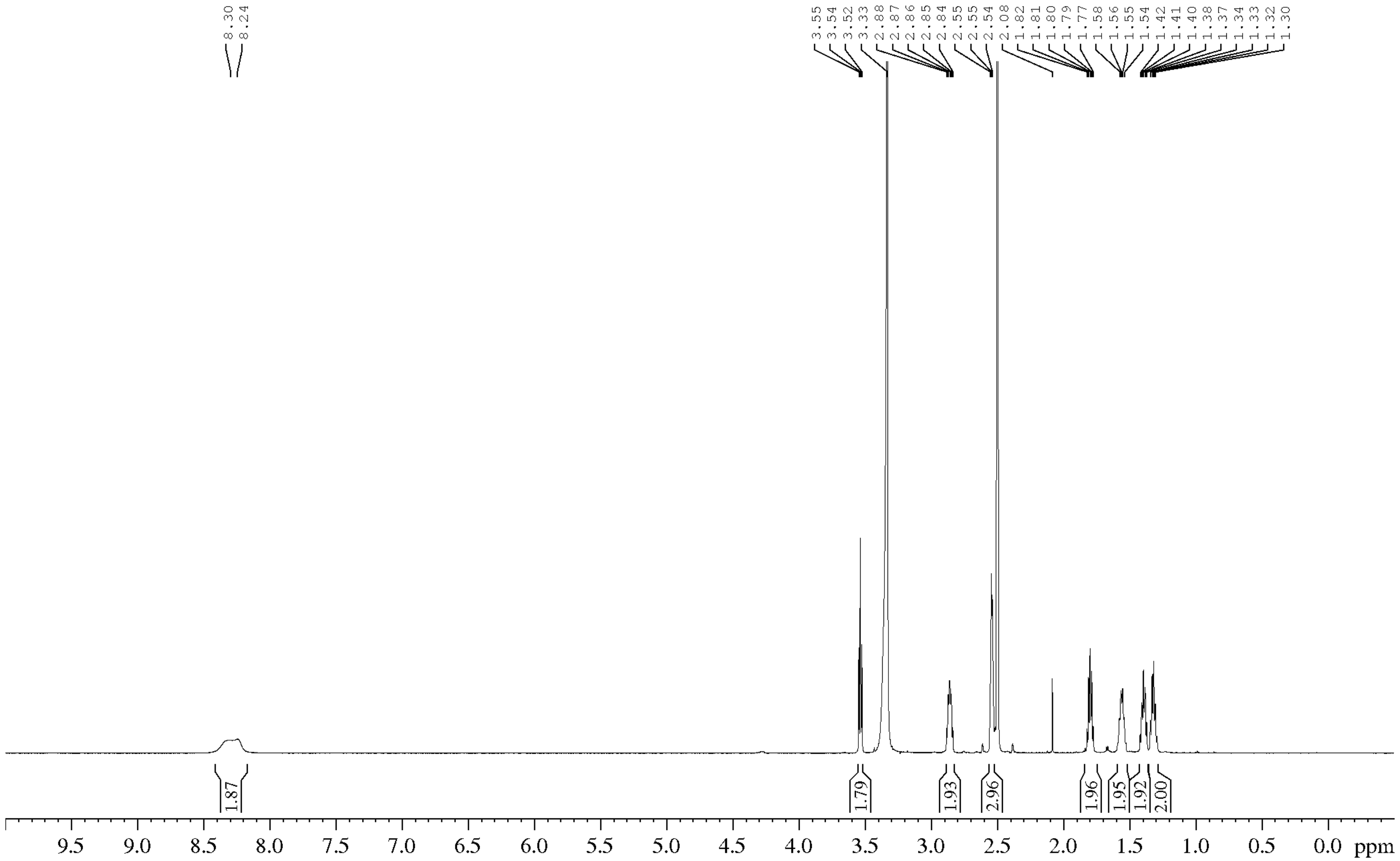

^{13}C NMR

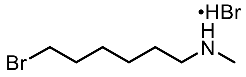
•HBr
H
N
Br

S85

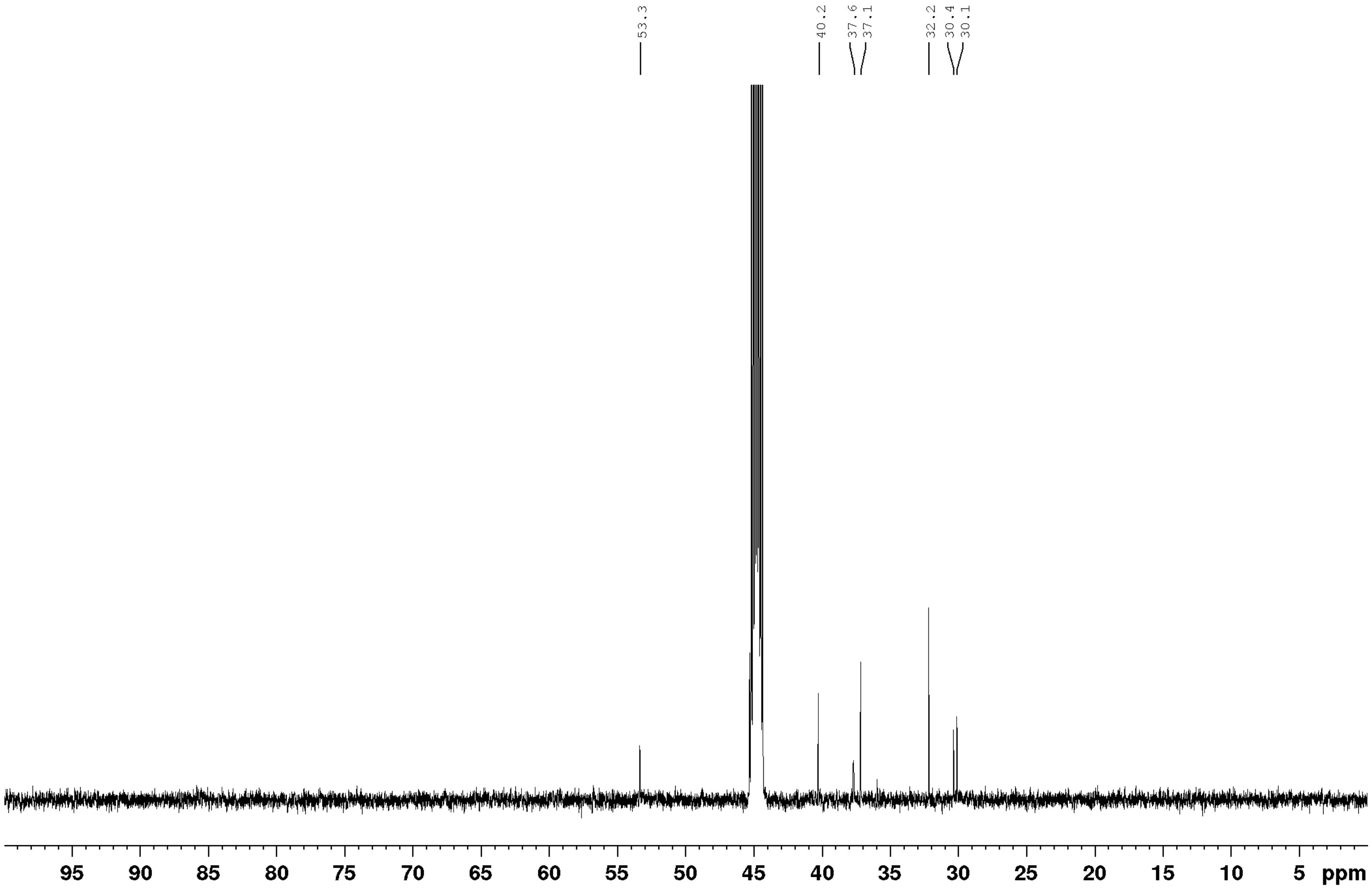
53.3
40.2
37.6
37.1
32.2
30.4
30.1
95 90 85 80 75 70 65 60 55 50 45 40 35 30 25 20 15 10 5 ppm

^{1}H, ^{1}H COSY

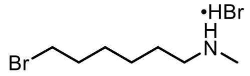

S85

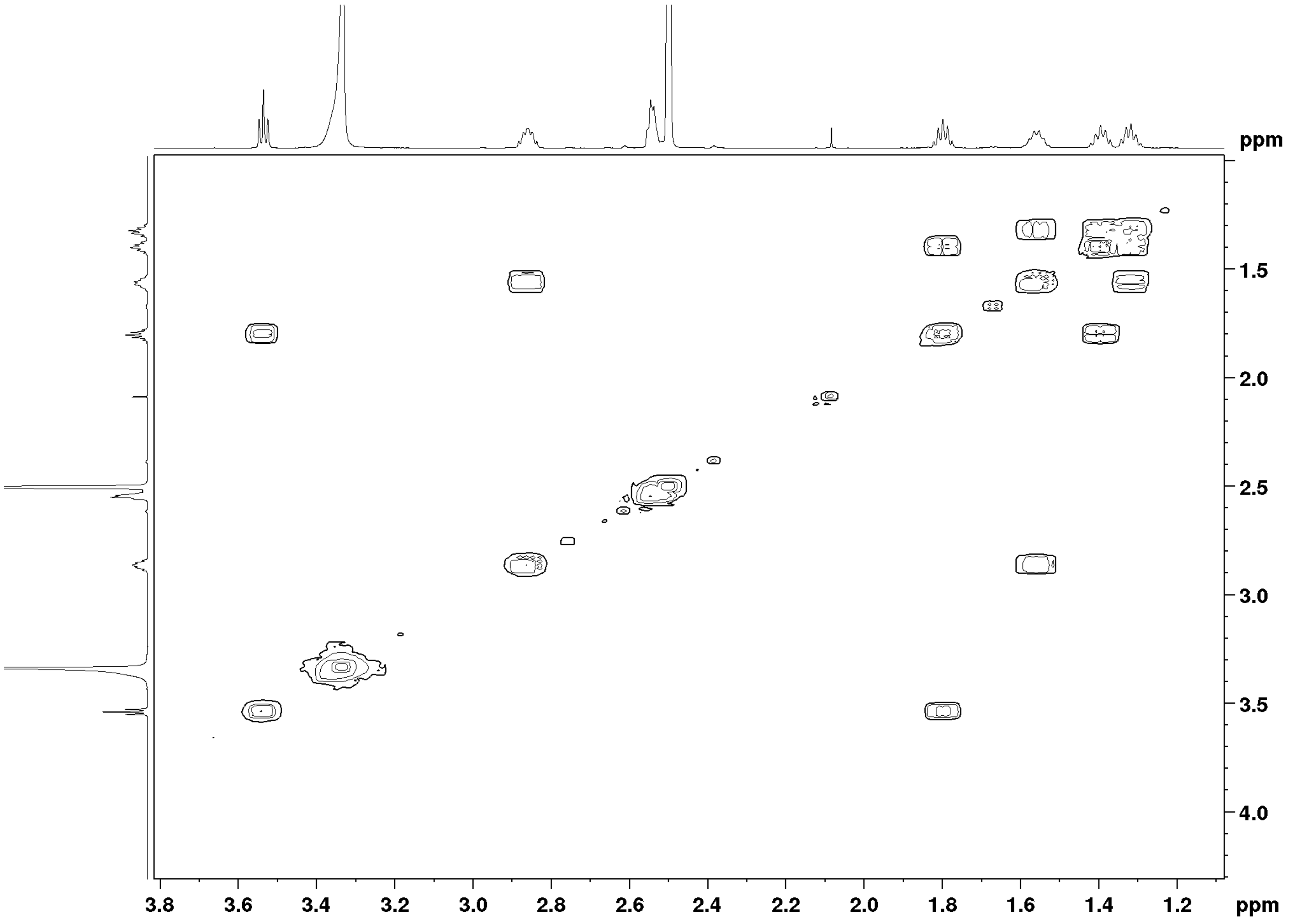

^{1}H, ^{13}C HMBC

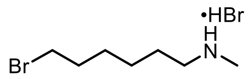

S85

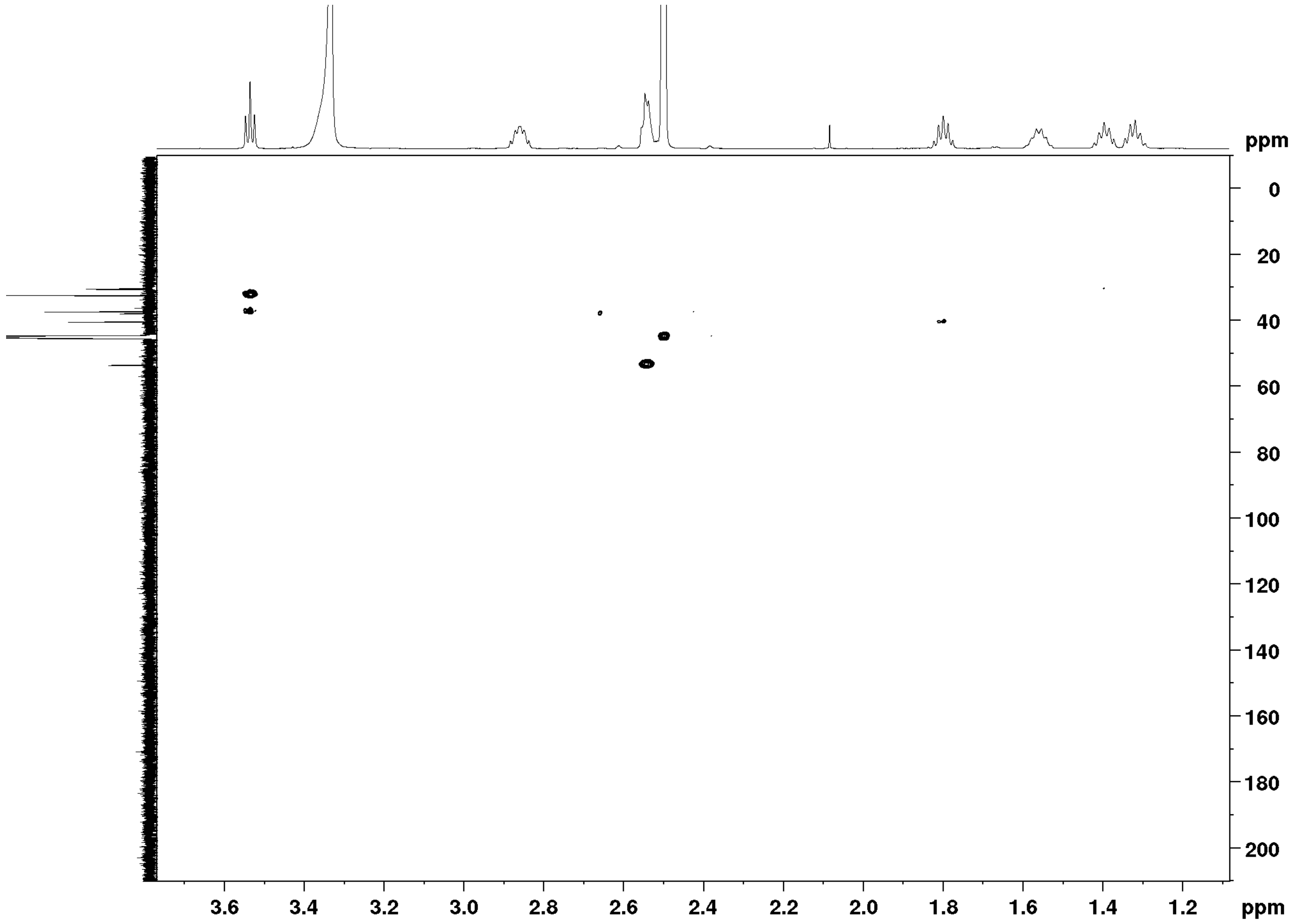

^{1}H, ^{13}C HSQC

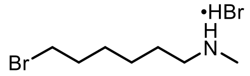

S85

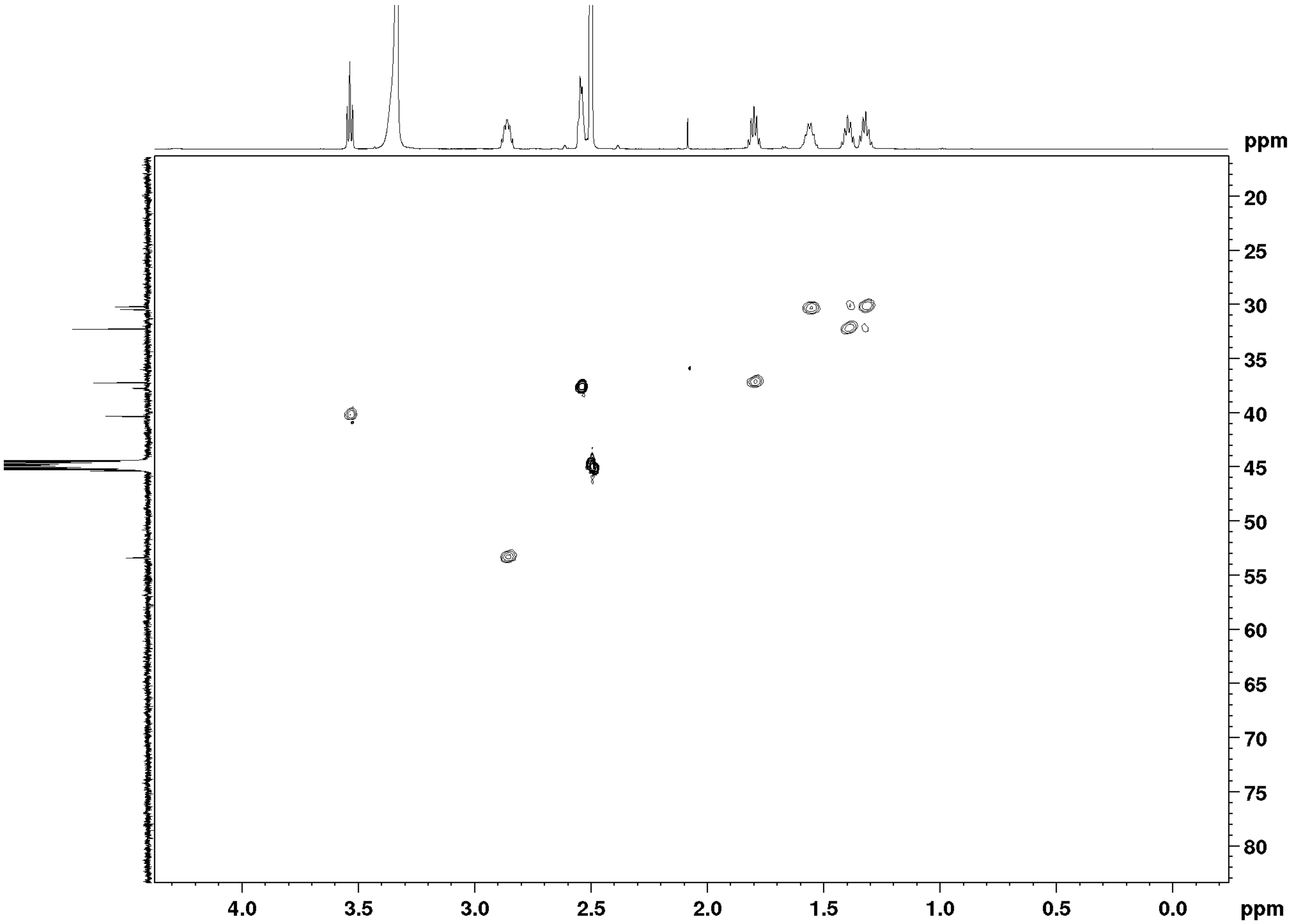

HRMS

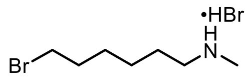

S85

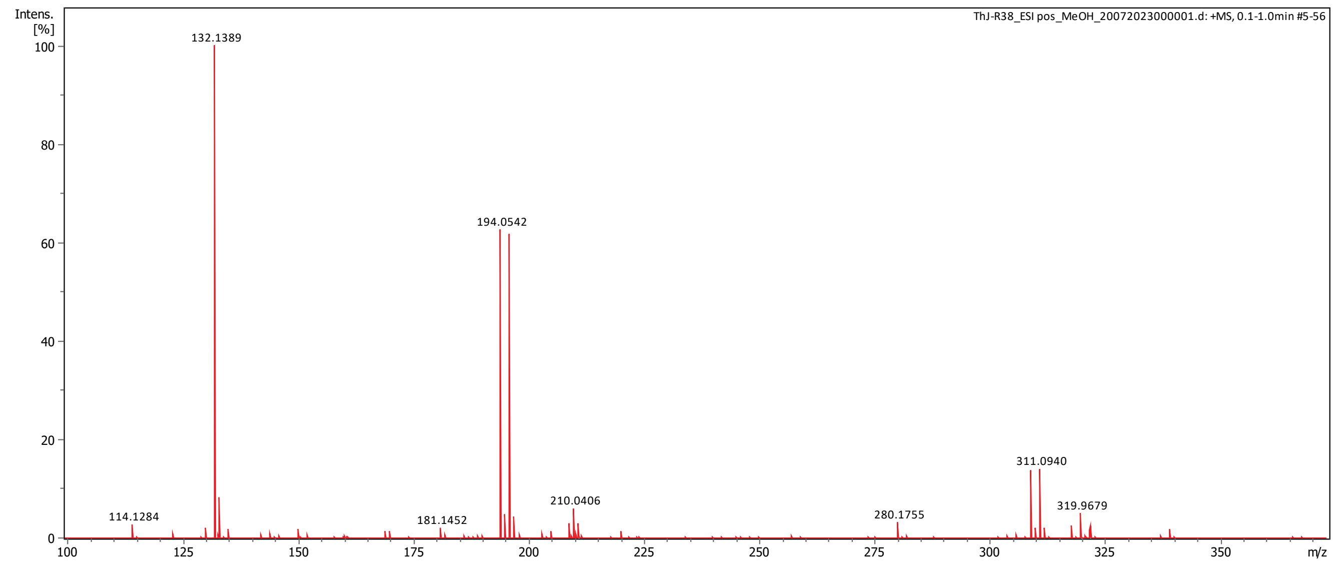
Intens. [%]
ThJ-R38_ESI pos_MeOH_20072023000001.d: +MS, 0.1-1.0min #5-56
132.1389
194.0542
210.0406
311.0940
319.9679
280.1755
181.1452
114.1284
m/z

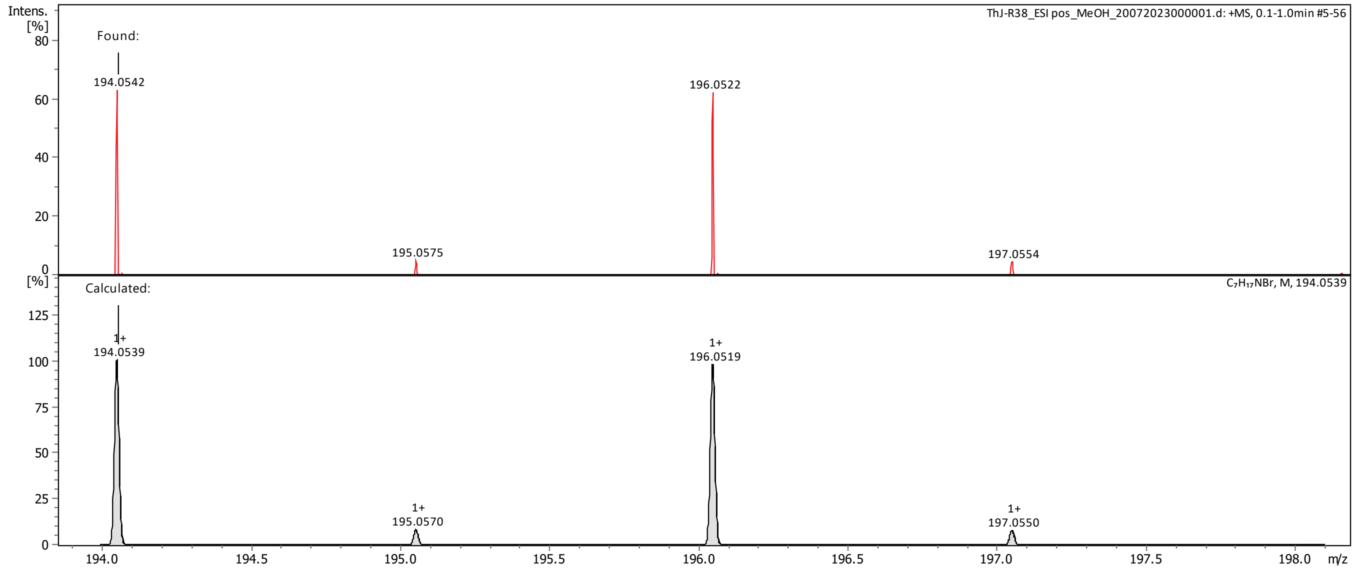
Intens. [%]
ThJ-R38_ESI pos_MeOH_20072023000001.d: +MS, 0.1-1.0min #5-56
Found:
194.0542
196.0522
195.0575
197.0554
Calculated:
$C_7H_{17}NBr$, M, 194.0539
1+ 194.0539
1+ 196.0519
1+ 195.0570
1+ 197.0550
m/z

IR

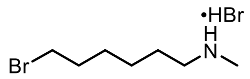
•HBr
H
N
Br

S85

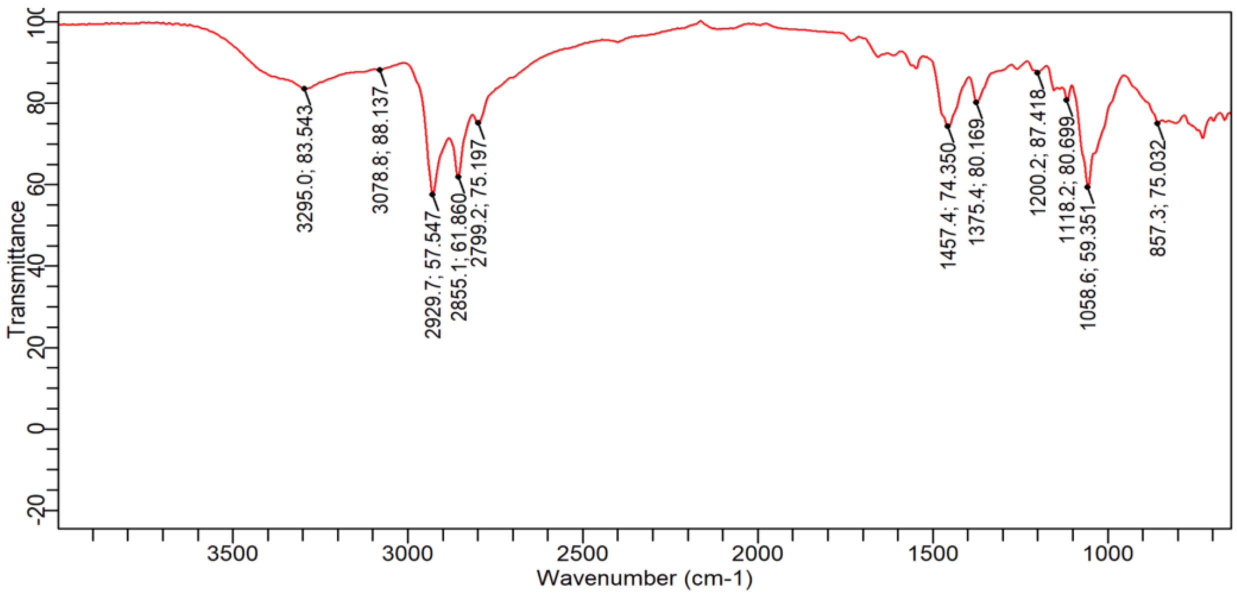
Transmittance
Wavenumber (cm-1)
3295.0; 83.543
3078.8; 88.137
2929.7; 57.547
2855.1; 61.860
2799.2; 75.197
1457.4; 74.350
1375.4; 80.169
1200.2; 87.418
1118.2; 80.699
1058.6; 59.351
857.3; 75.032

95 1-Mesityl-3-(6-(methylamino)hexyl)-1*H*-imidazol-3-ium bromide hydrobromide (S86)

^{1}H NMR

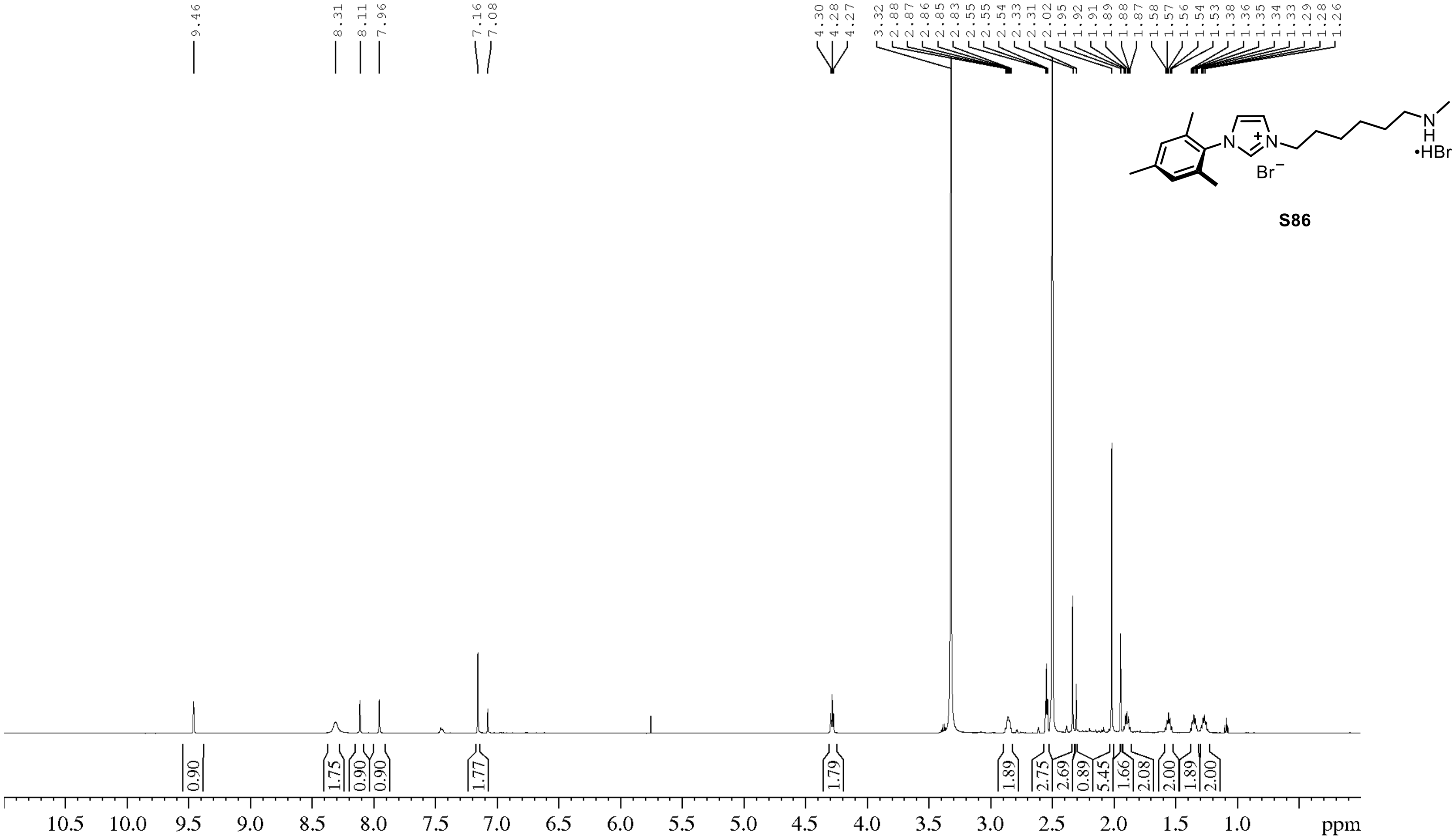

^{13}C NMR

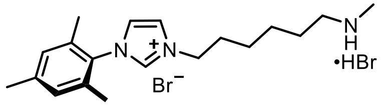
N
N
H
•HBr
Br

S86

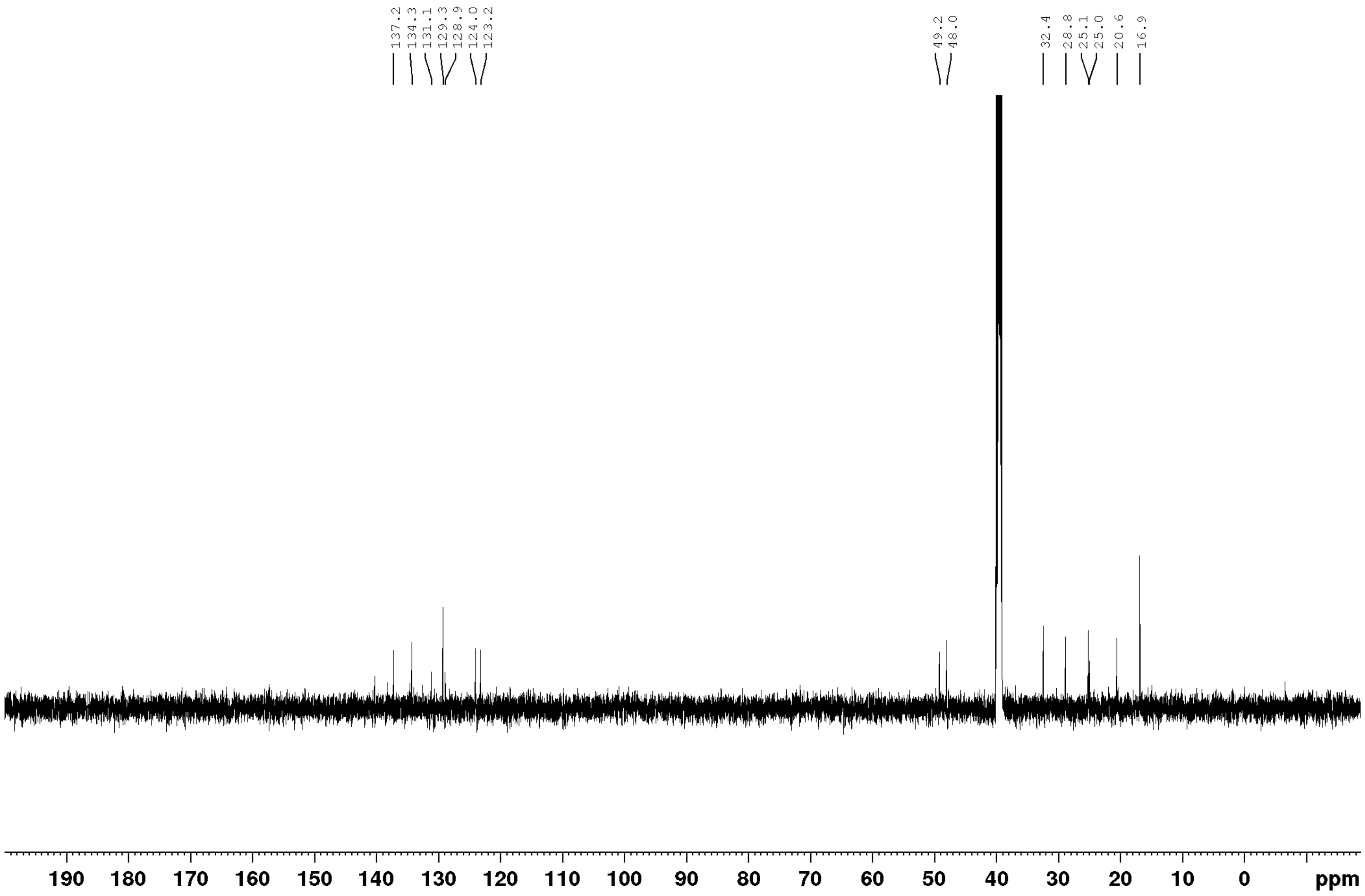
137.2
134.3
131.1
129.3
128.9
124.0
123.2
49.2
48.0
32.4
28.8
25.1
25.0
20.6
16.9
190
180
170
160
150
140
130
120
110
100
90
80
70
60
50
40
30
20
10
0
ppm

^{1}H, ^{1}H COSY

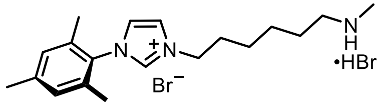

S86

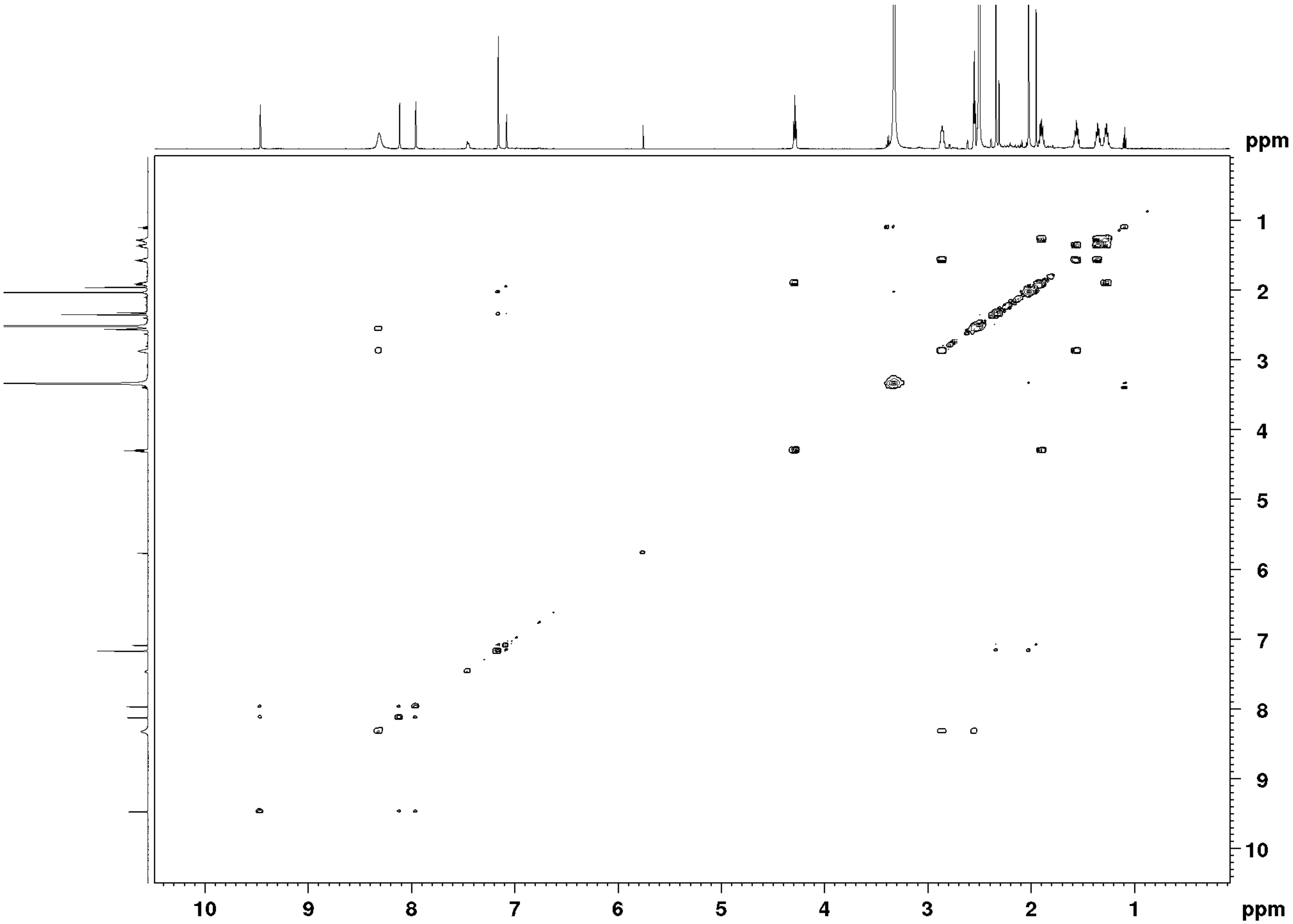

^{1}H, ^{13}C HMBC

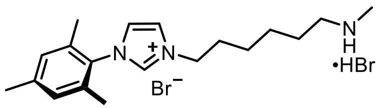

S86

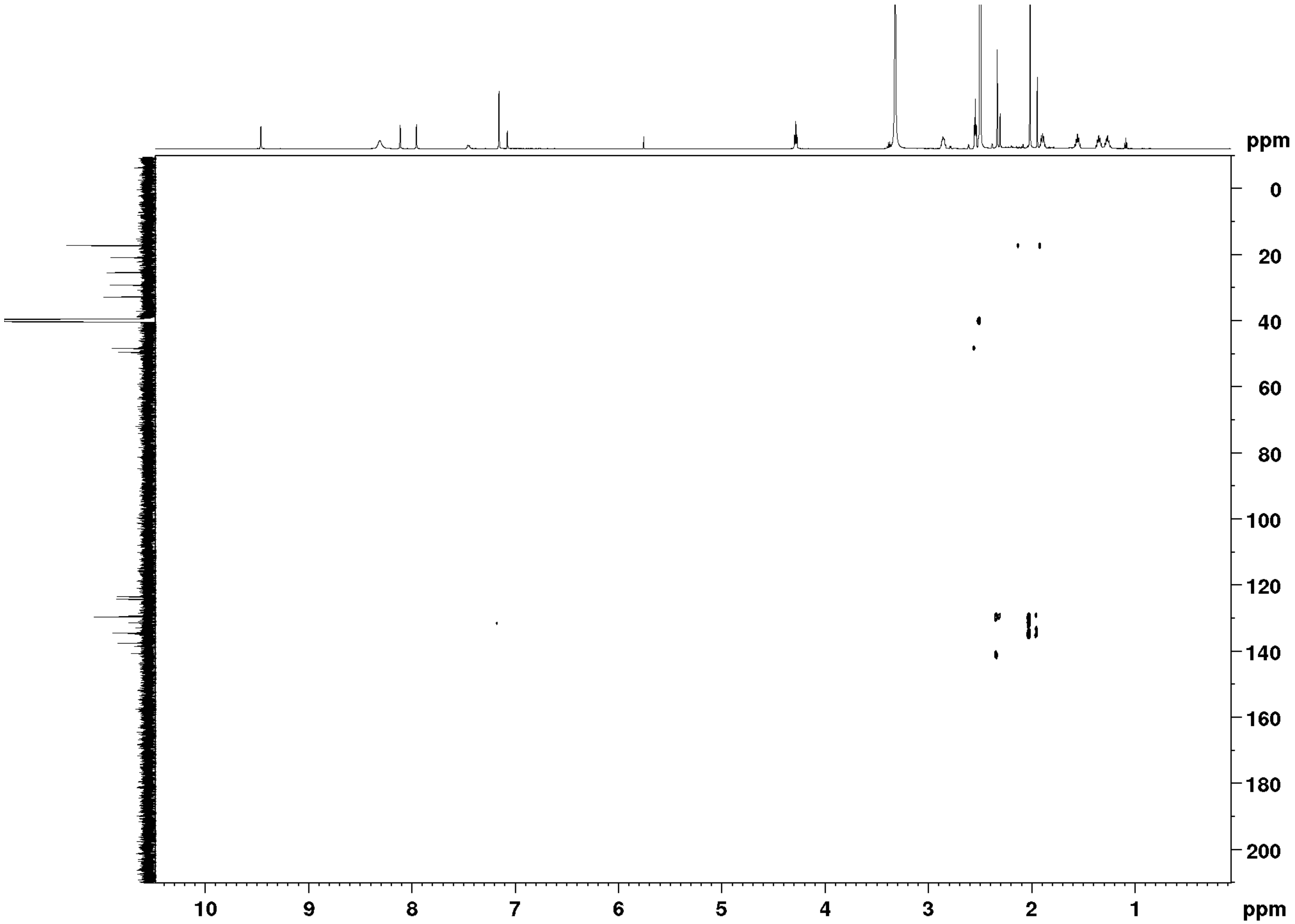

^{1}H, ^{13}C HSQC

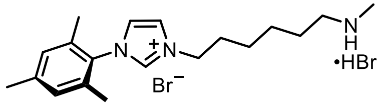

S86

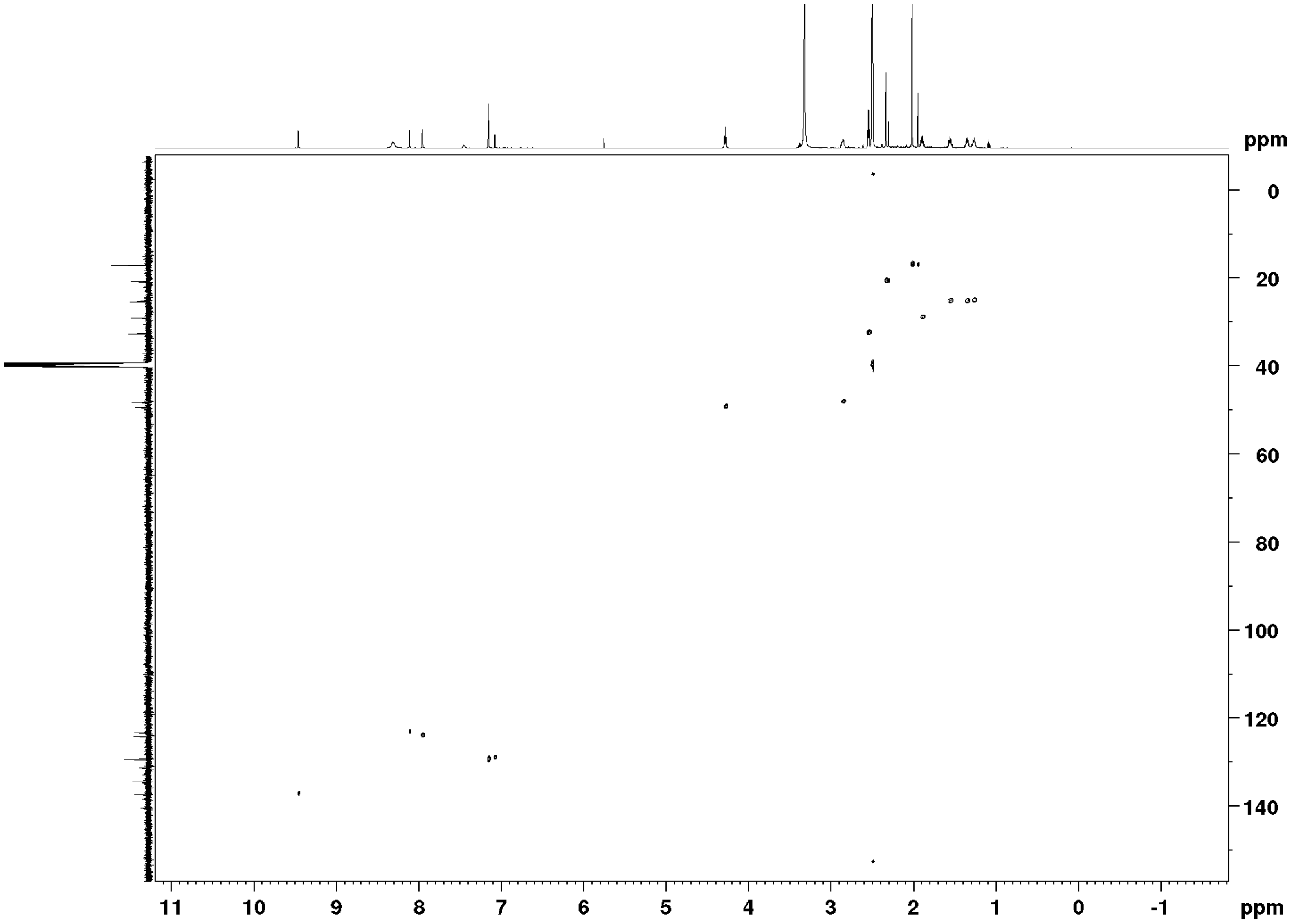

HRMS

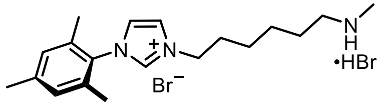

S86

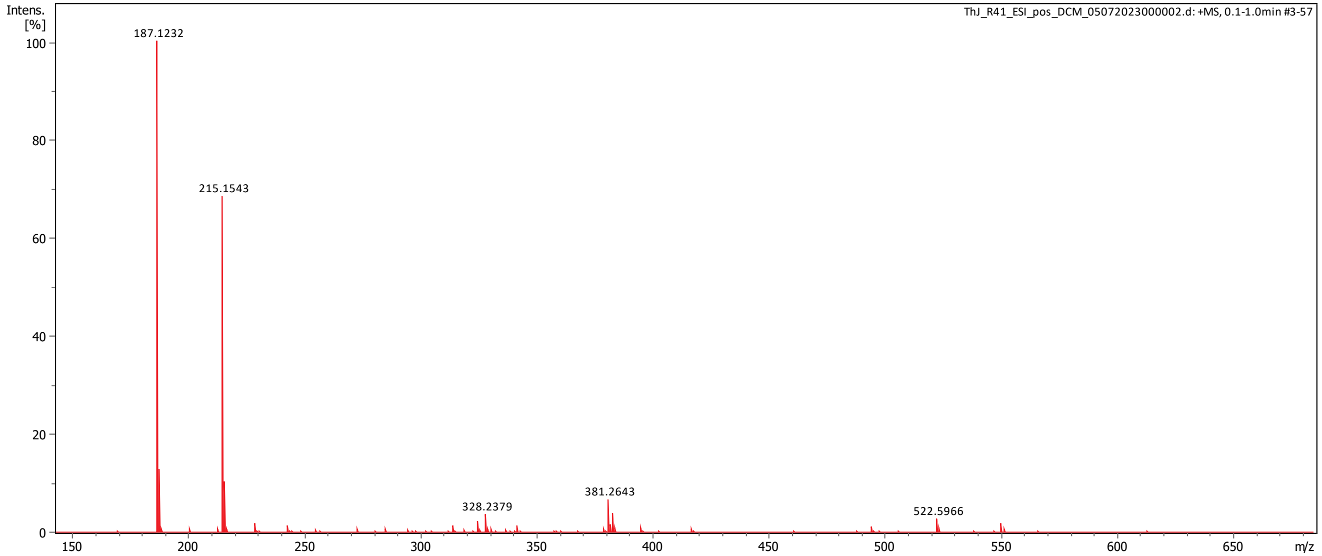

Intens. [%]
ThJ_R41_ESI_pos_DCM_05072023000002.d: +MS, 0.1-1.0min #3-57
187.1232
215.1543
328.2379
381.2643
522.5966
m/z

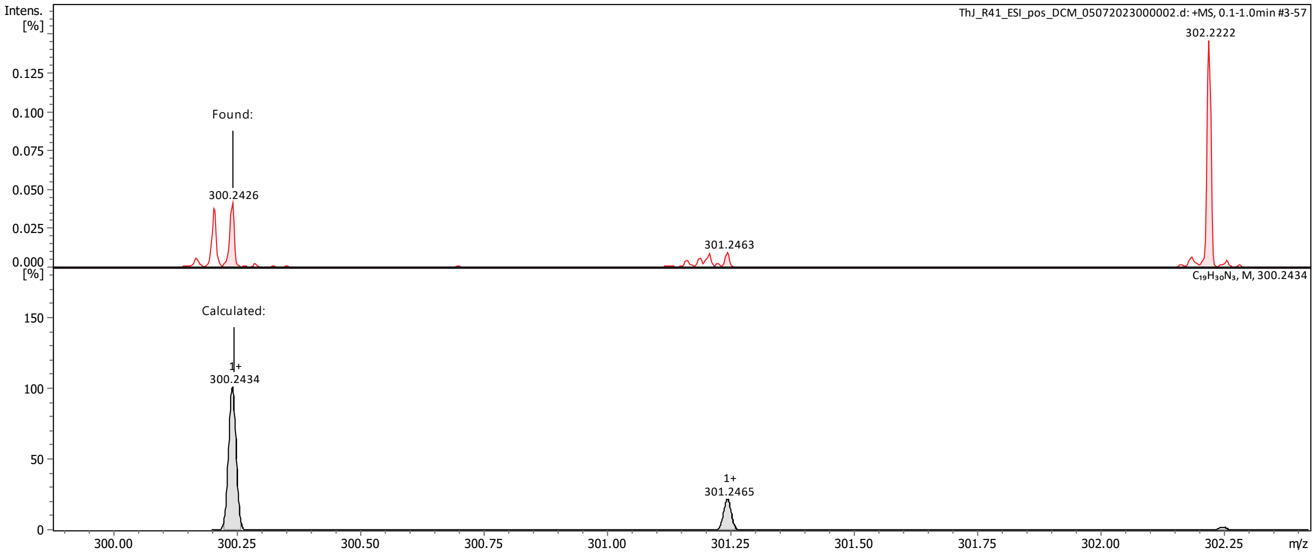

Intens. [%]
ThJ_R41_ESI_pos_DCM_05072023000002.d: +MS, 0.1-1.0min #3-57
302.2222
Found:
300.2426
301.2463
$C_{19}H_{30}N_3$, M, 300.2434
Calculated:
1+
300.2434
1+
301.2465
m/z

IR

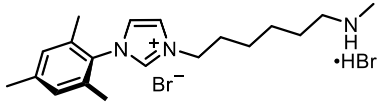
N
N
Br
N
H
•HBr

S86

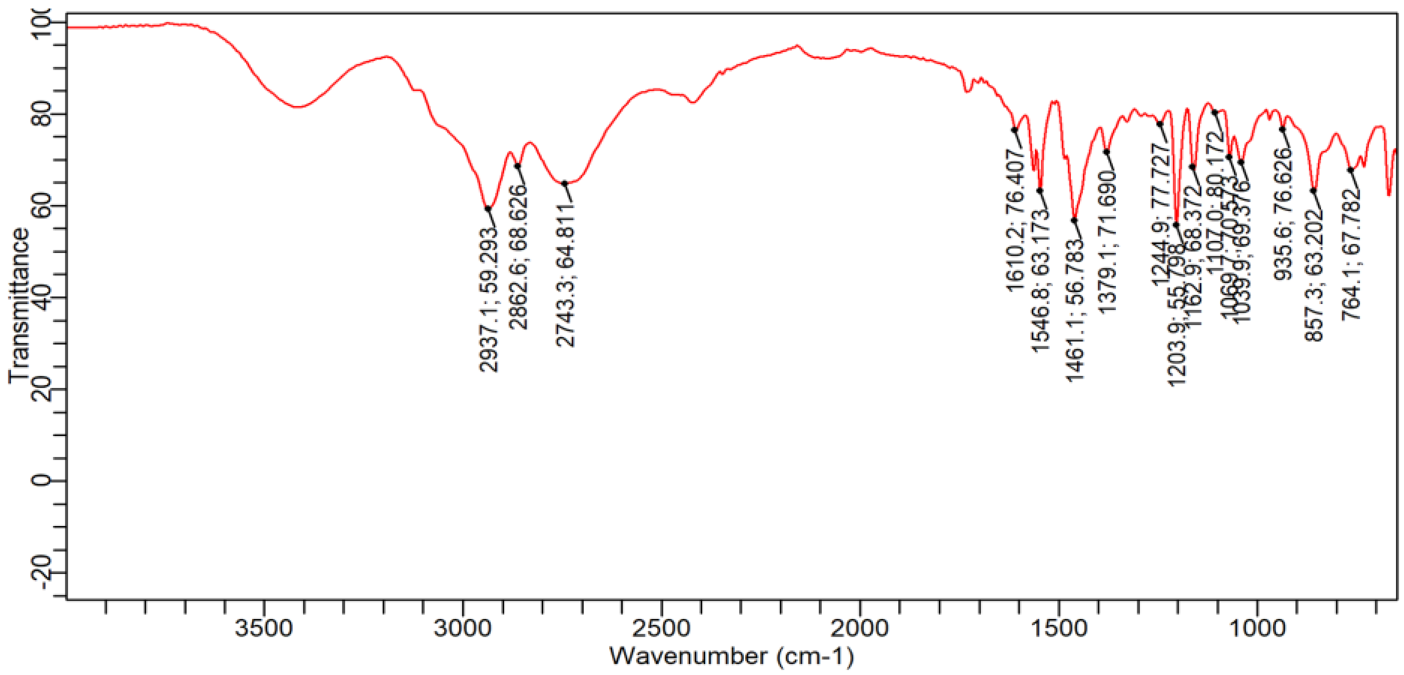
Transmittance
Wavenumber (cm-1)
2937.1; 59.293
2862.6; 68.626
2743.3; 64.811
1610.2; 76.407
1546.8; 63.173
1461.1; 56.783
1379.1; 71.690
1244.9; 77.727
935.6; 76.626
857.3; 63.202
764.1; 67.782

96 (*E*)-3-(6-(2,3-Diisopropyl-1-methylguanidino)hexyl)-1-mesityl-*1H*-imidazol-3-ium bromide hydrobromide (S87)

^{1}H NMR

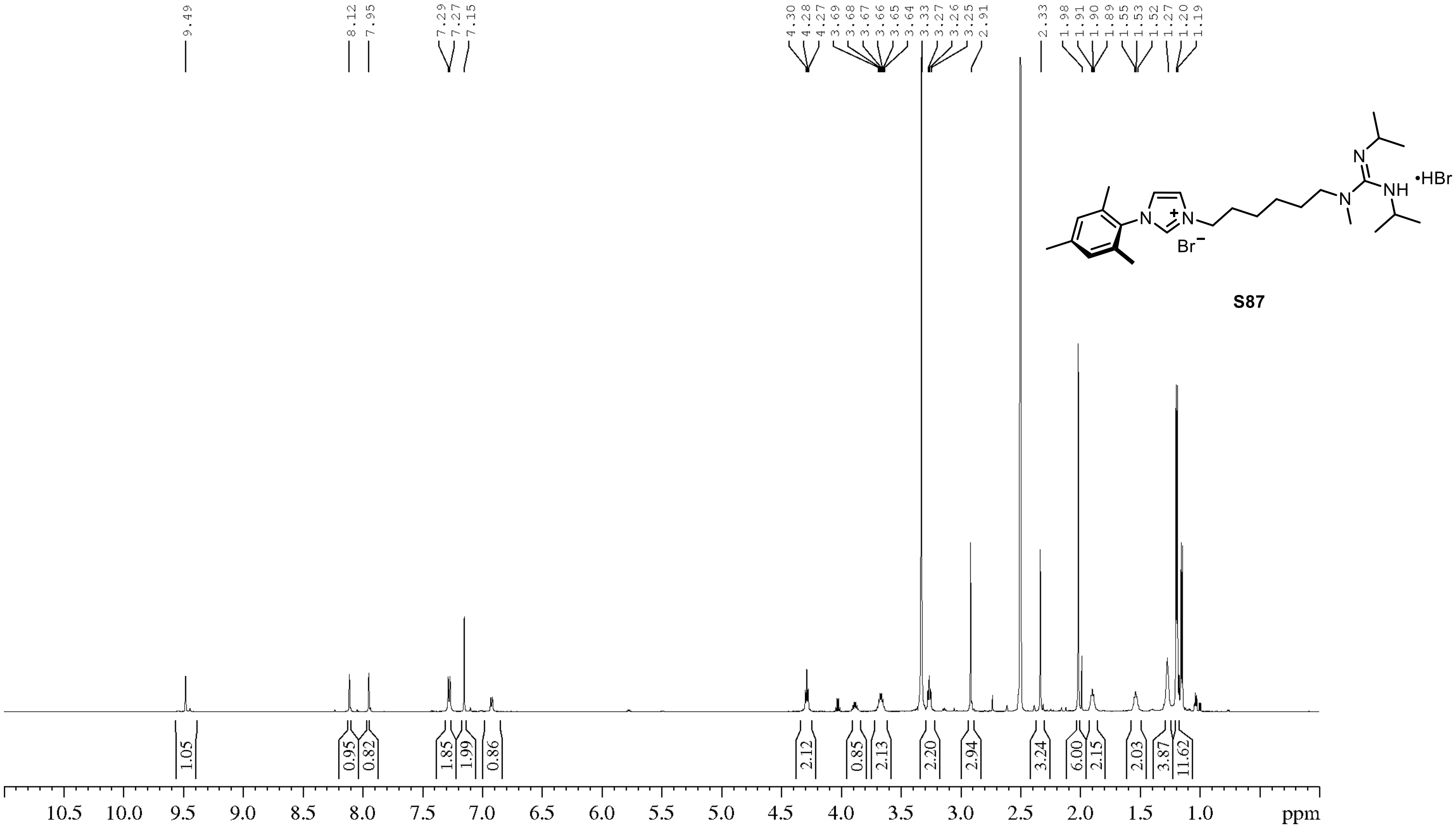

S87

^{13}C NMR

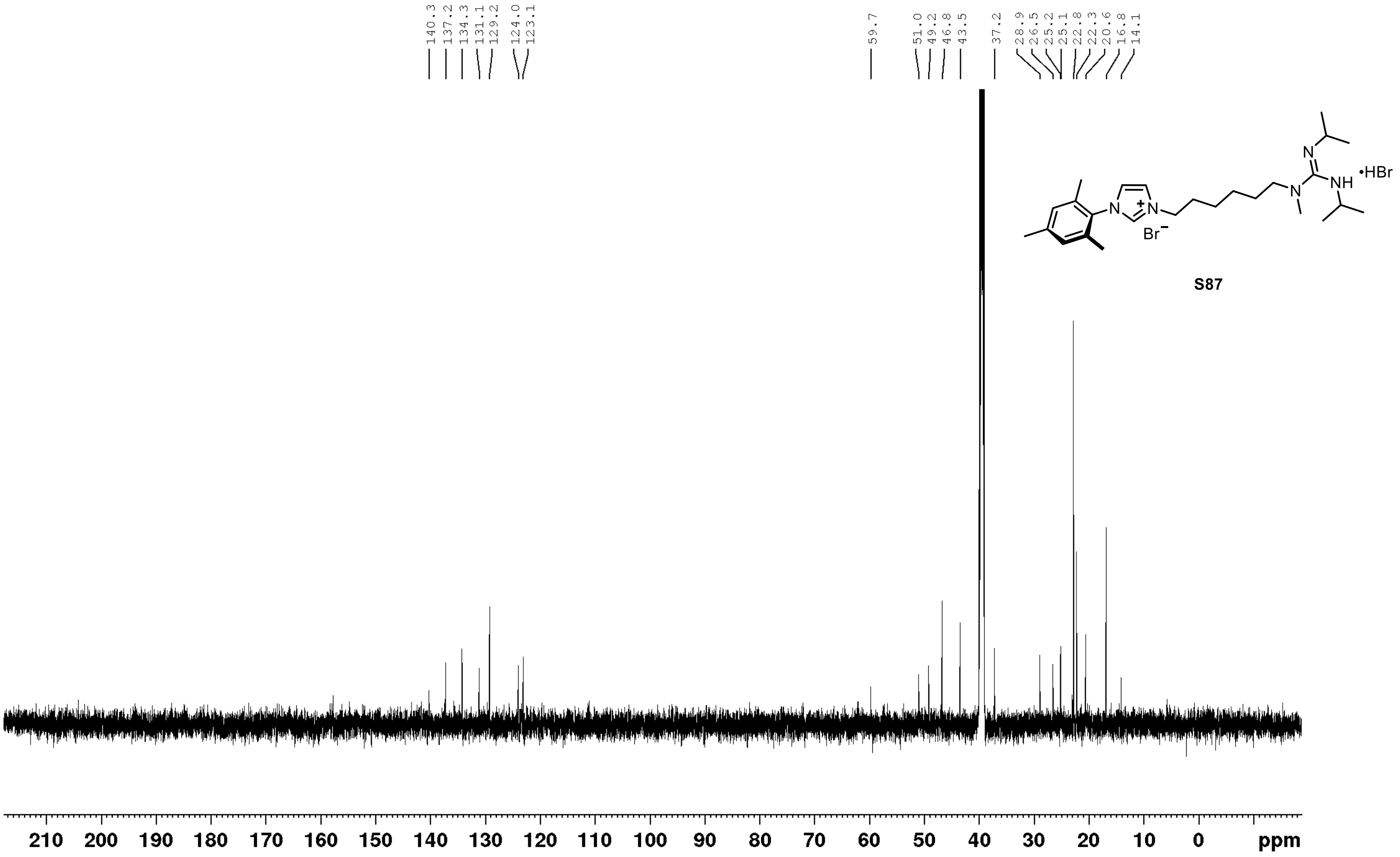
140.3
137.2
134.3
131.1
129.2
124.0
123.1
59.7
51.0
49.2
46.8
43.5
37.2
28.9
26.5
25.2
25.1
22.8
22.3
20.6
16.8
14.1
N
NH
•HBr
N
N
N
Br−
S87
210
200
190
180
170
160
150
140
130
120
110
100
90
80
70
60
50
40
30
20
10
0
ppm

^{1}H, ^{1}H COSY

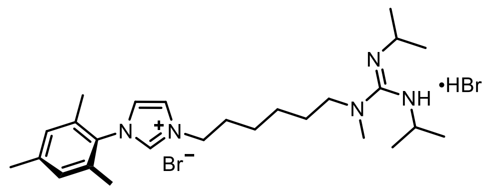

S87

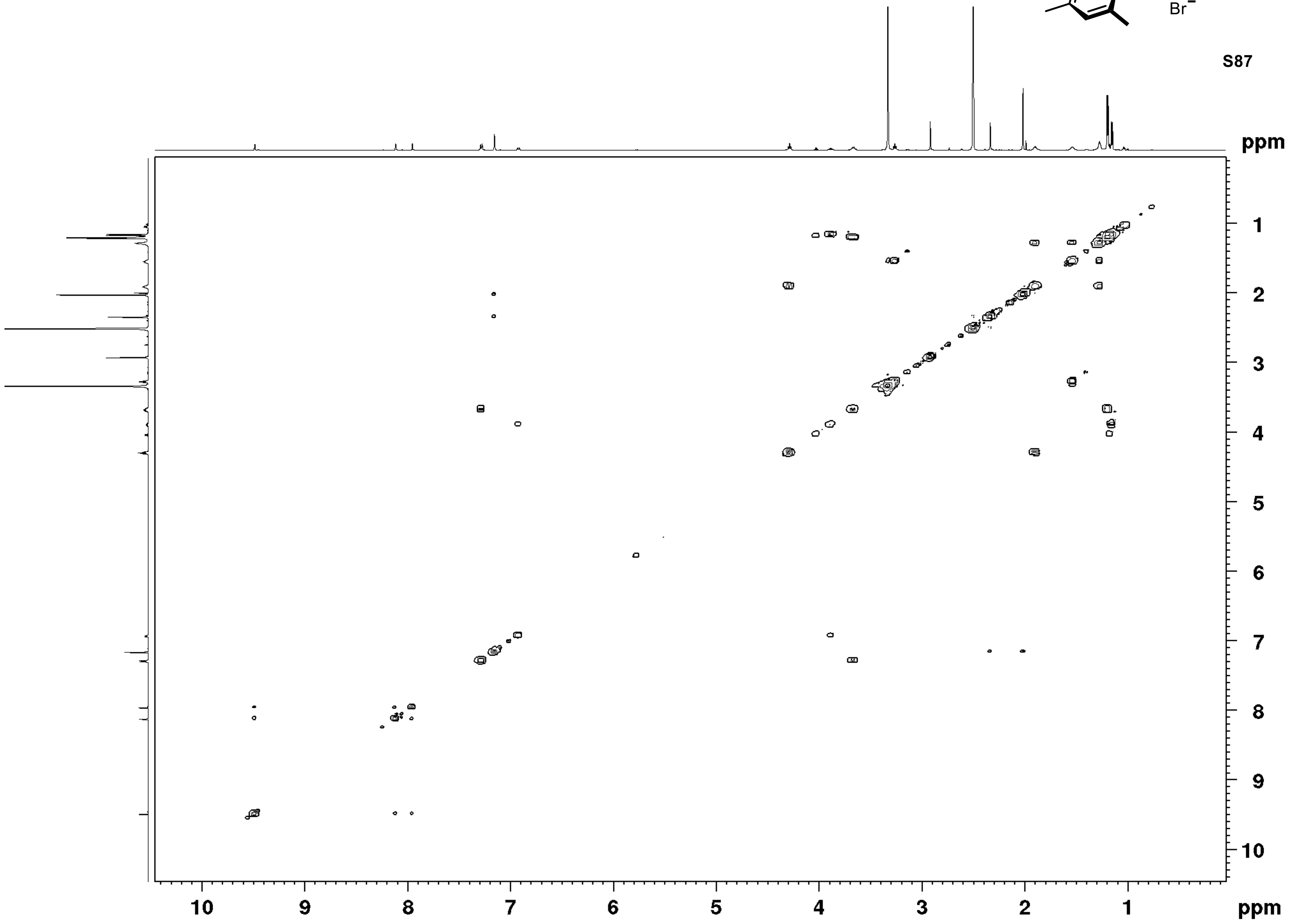

^{1}H, ^{13}C HMBC

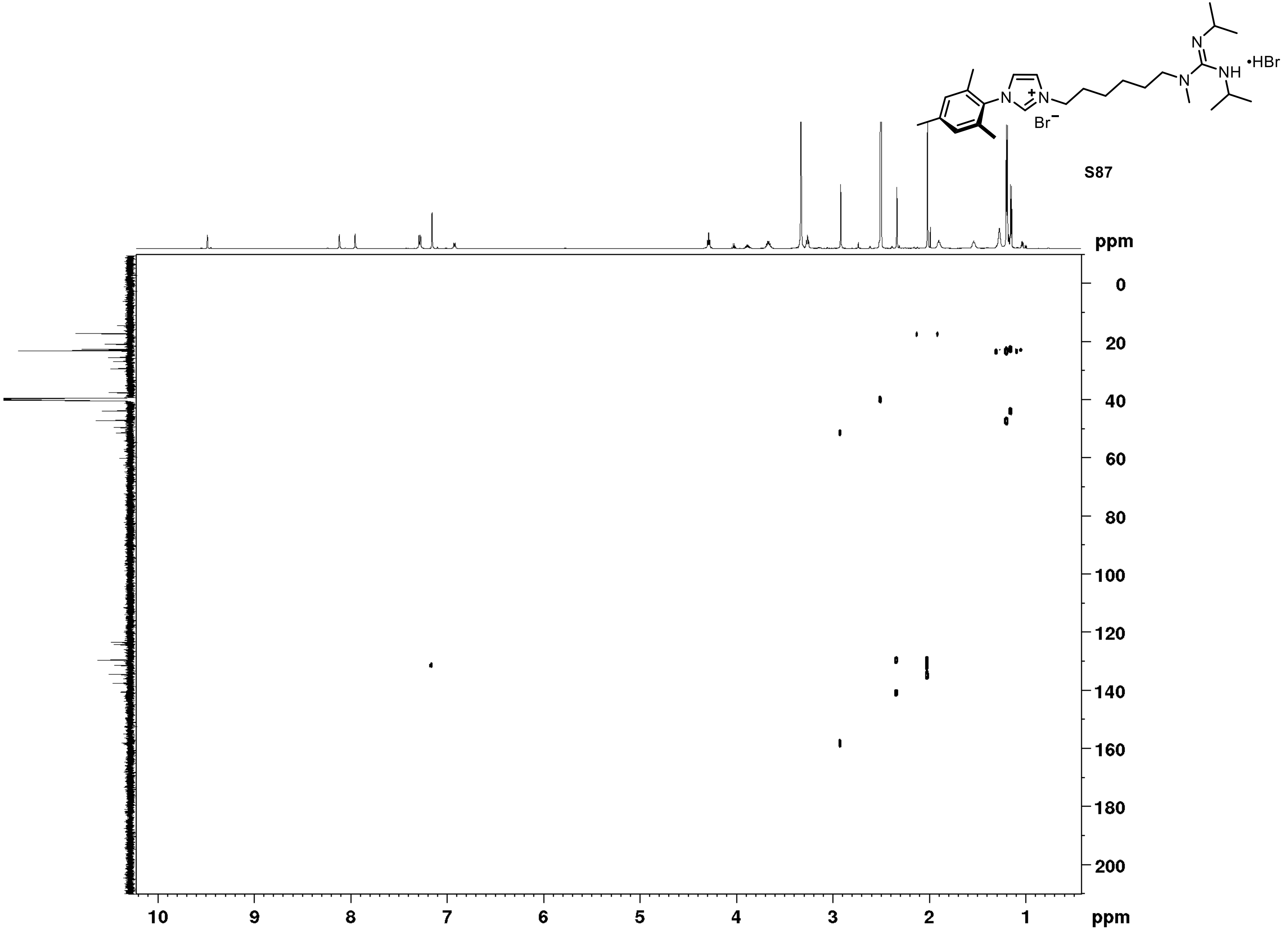

N
NH
•HBr
N
N
N
Br
S87
ppm
0
20
40
60
80
100
120
140
160
180
200
10
9
8
7
6
5
4
3
2
1
ppm

^{1}H, ^{13}C HSQC

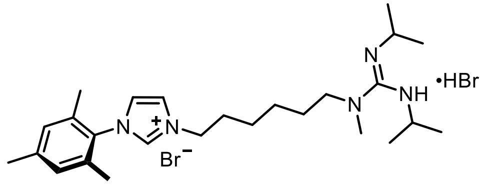

S87

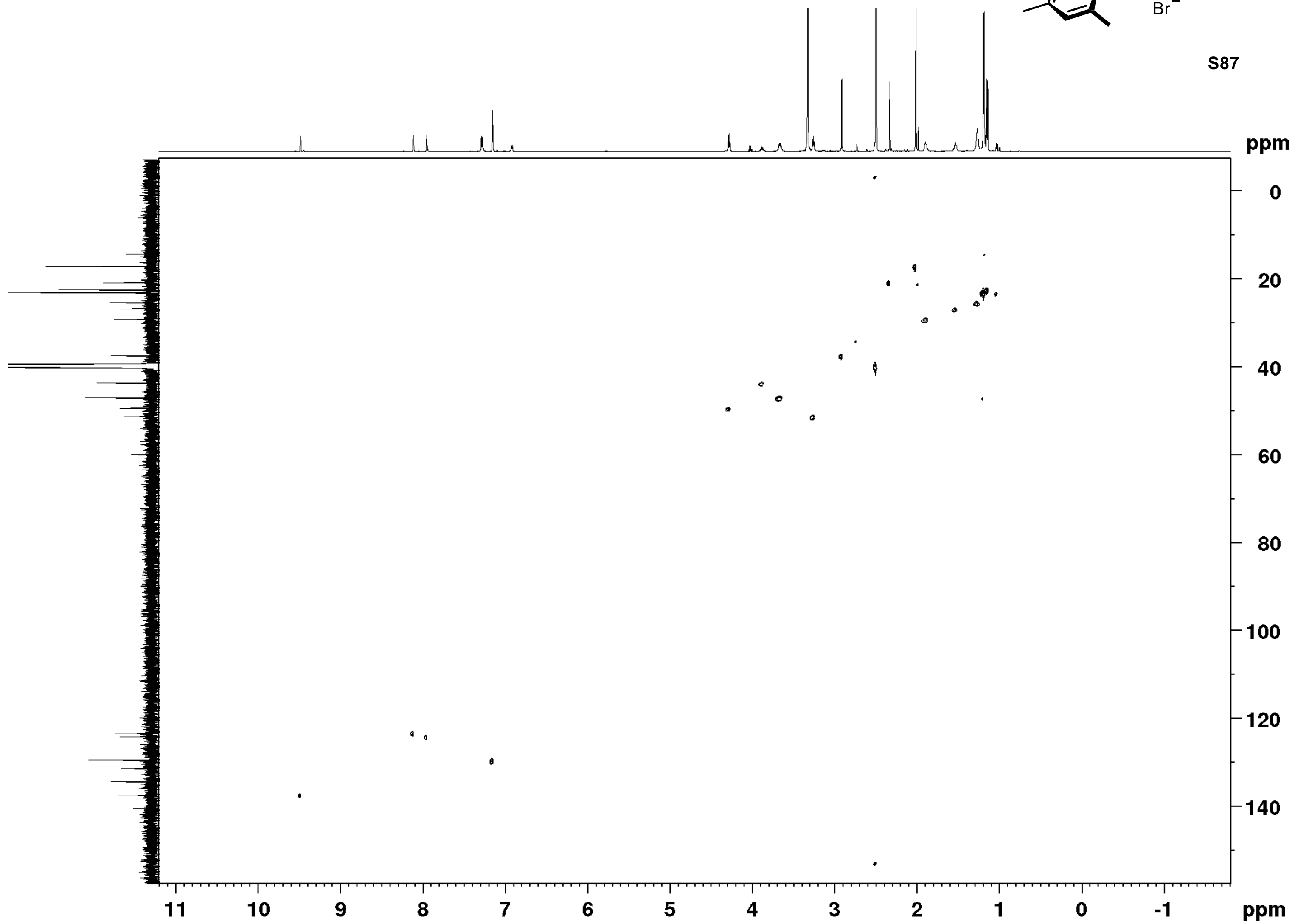

HRMS

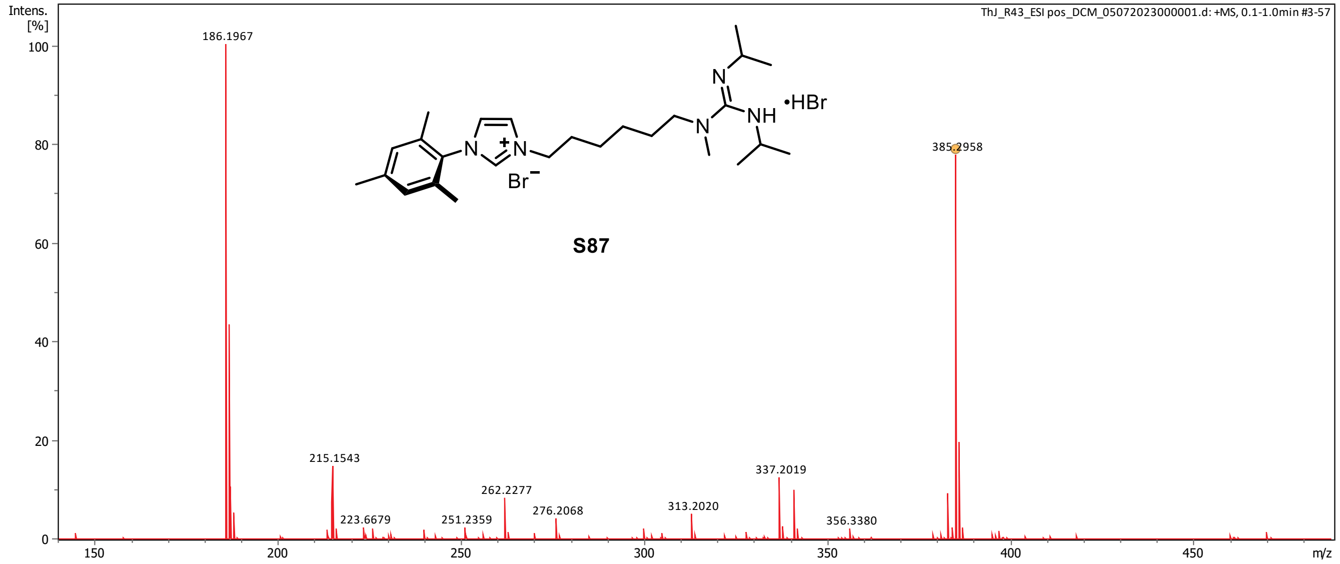
Intens. [%]
ThJ_R43_ESI pos_DCM_05072023000001.d: +MS, 0.1-1.0min #3-57
186.1967
215.1543
223.6679
251.2359
262.2277
276.2068
313.2020
337.2019
356.3380
385.2958
•HBr
Br−
S87
m/z

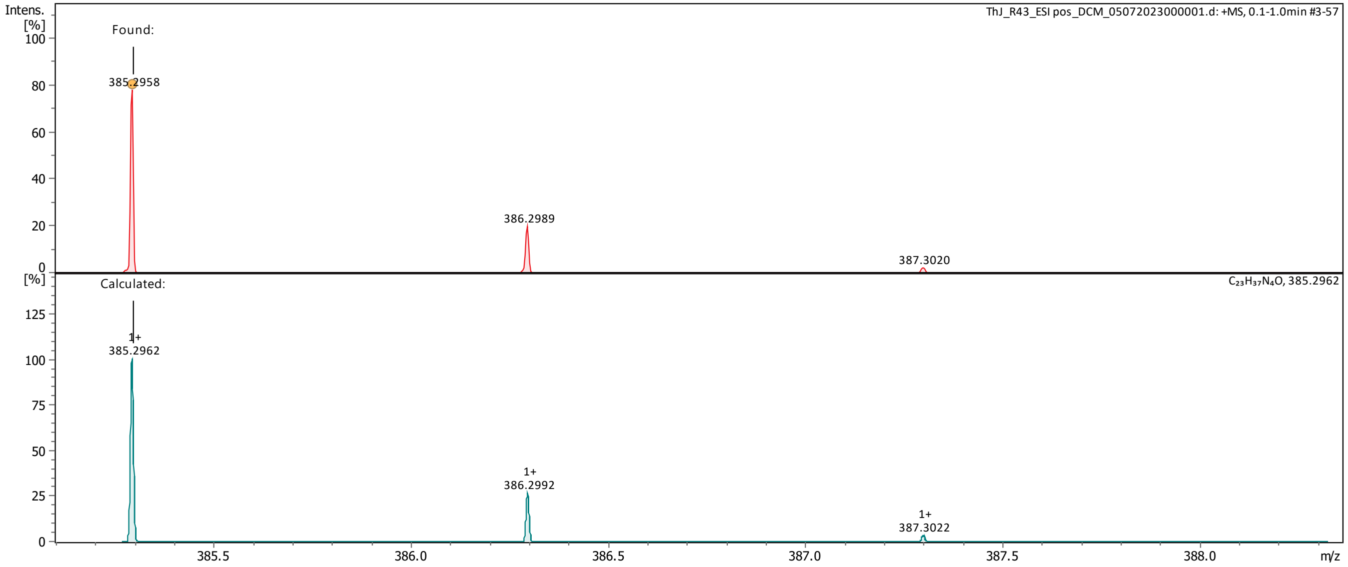
Intens. [%]
ThJ_R43_ESI pos_DCM_05072023000001.d: +MS, 0.1-1.0min #3-57
Found:
385.2958
386.2989
387.3020
Calculated:
C23H37N4O, 385.2962
1+
385.2962
1+
386.2992
1+
387.3022
m/z

IR

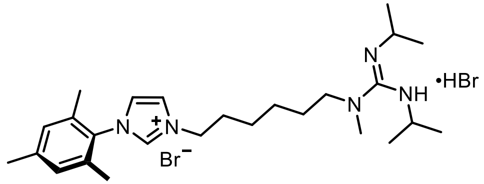

•HBr
Br−

S87

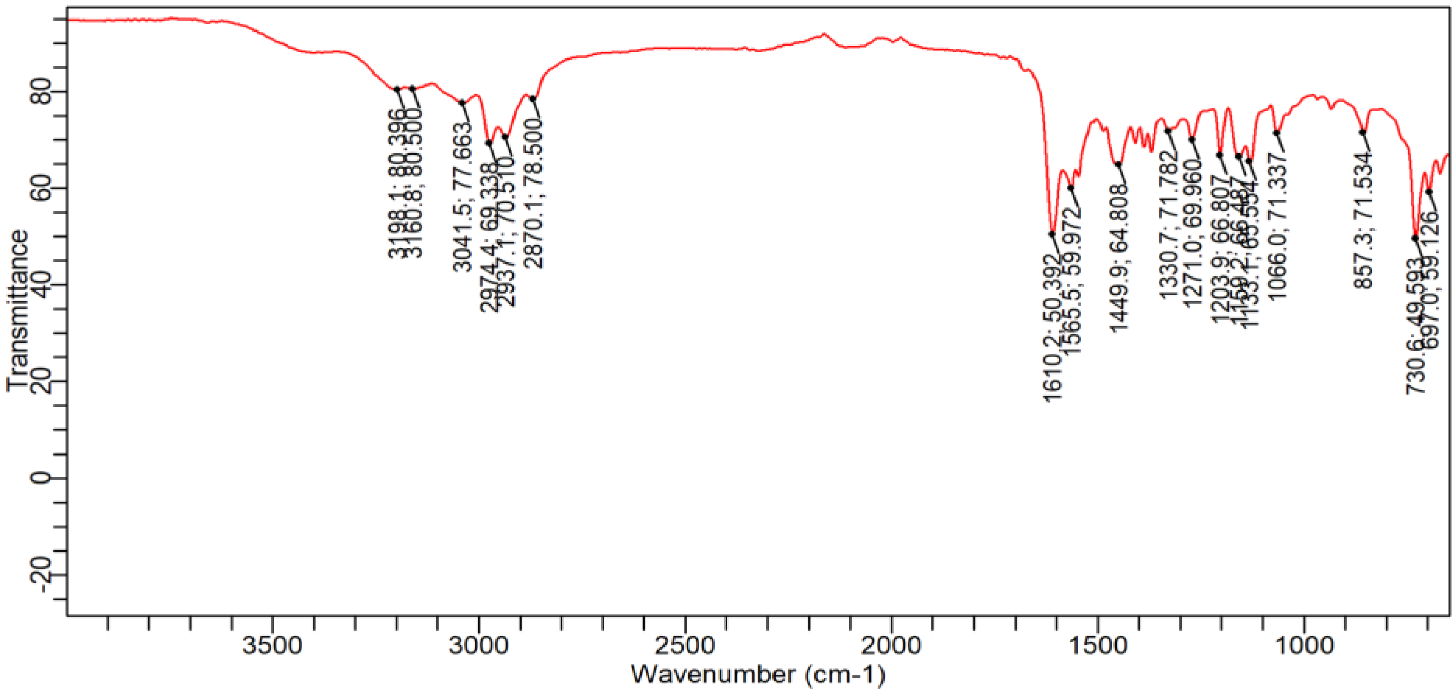

Transmittance
Wavenumber (cm-1)
3198.1; 80.396
3160.8; 80.500
3041.5; 77.663
2974.4; 69.338
2937.1; 70.510
2870.1; 78.500
1610.2; 50.392
1565.5; 59.972
1449.9; 64.808
1330.7; 71.782
1271.0; 69.960
1203.9; 66.807
1066.0; 71.337
857.3; 71.534
730.6; 49.593
697.0; 59.126

97 (*E*)-(1-(6-(2,3-Diisopropyl-1-methylguanidino)hexyl)-3-mesityl-1, 3-dihydro-2*H*-imidazol-2-ylidene)copper(I) chloride (7)

^{1}H NMR

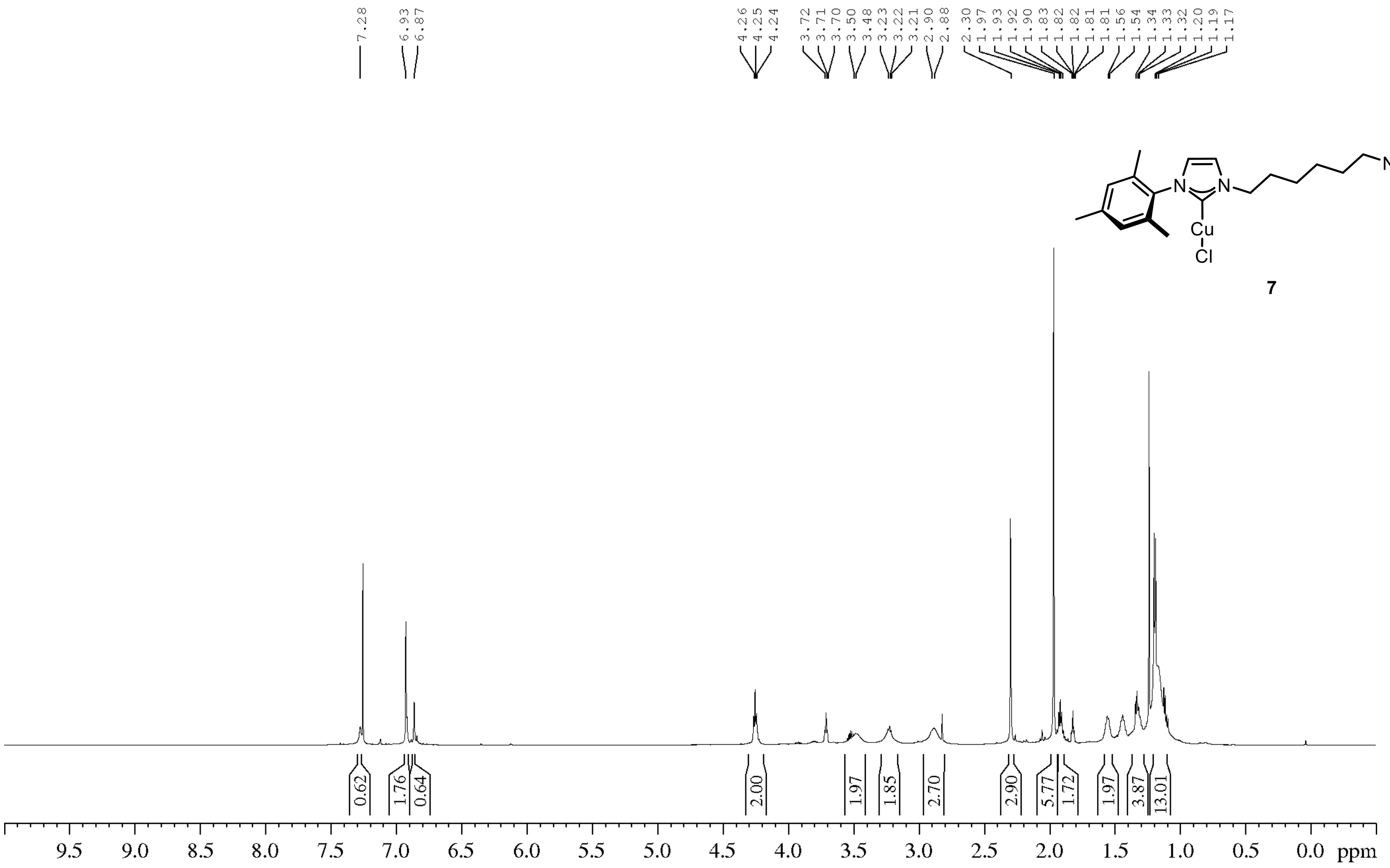

^{13}C NMR

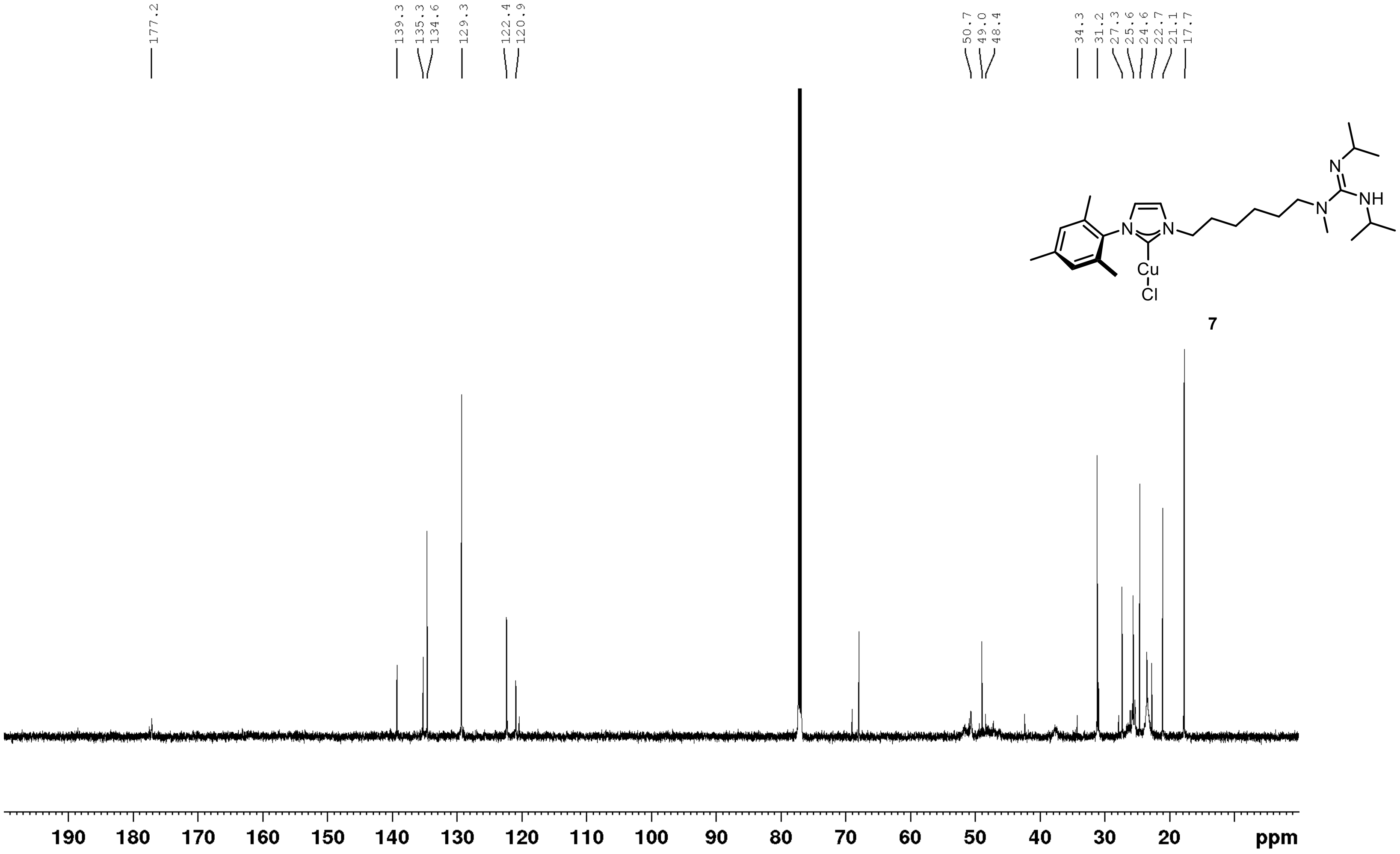
177.2
139.3
135.3
134.6
129.3
122.4
120.9
50.7
49.0
48.4
34.3
31.2
27.3
25.6
24.6
22.7
21.1
17.7
N
N
Cu
Cl
N
N
NH
7
190 180 170 160 150 140 130 120 110 100 90 80 70 60 50 40 30 20 ppm

^{1}H, ^{1}H COSY

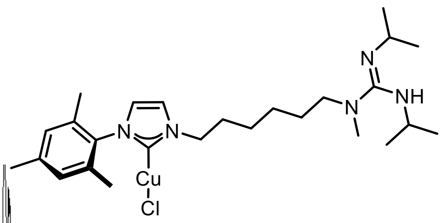

7

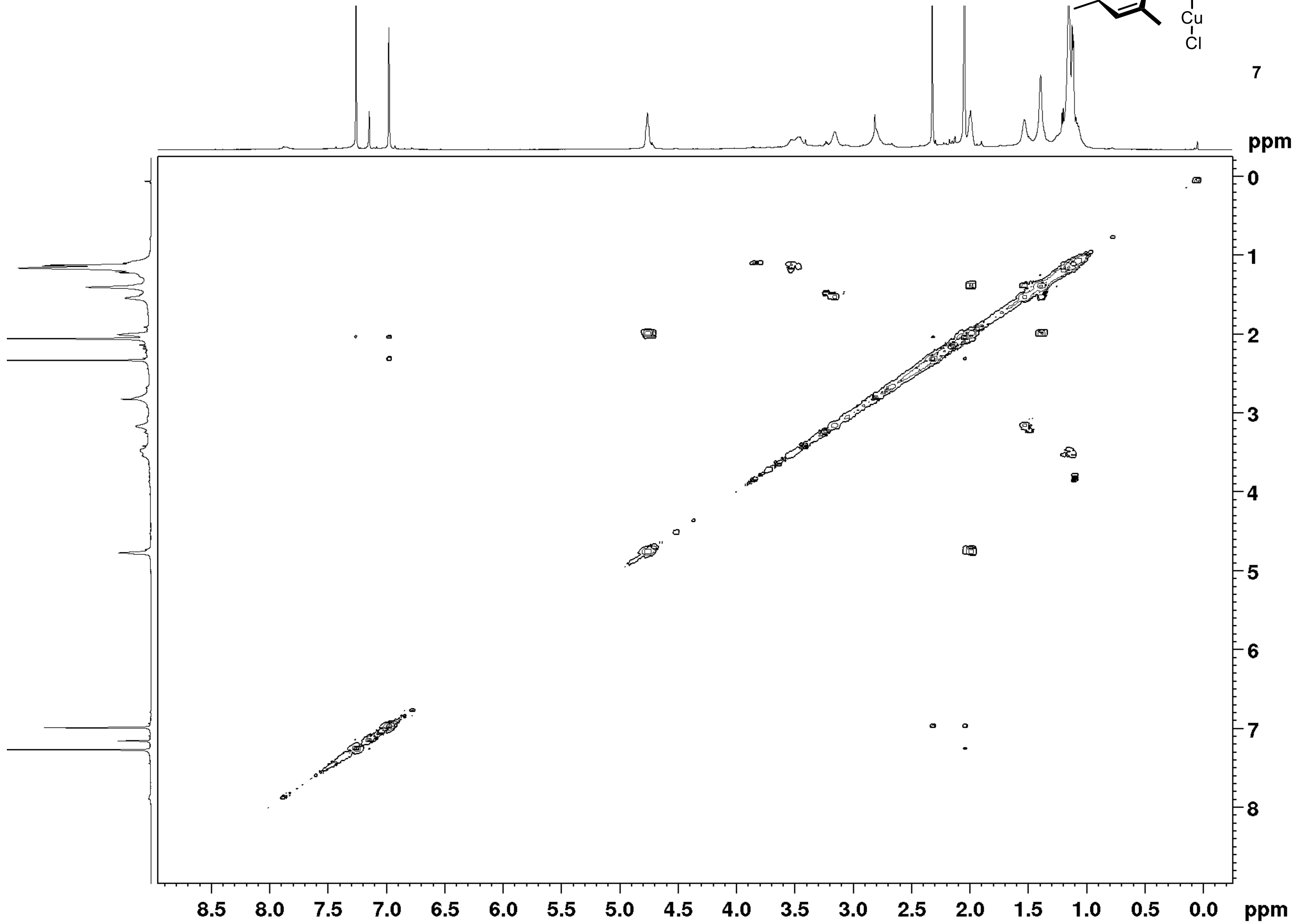
ppm
0
1
2
3
4
5
6
7
8
8.5
8.0
7.5
7.0
6.5
6.0
5.5
5.0
4.5
4.0
3.5
3.0
2.5
2.0
1.5
1.0
0.5
0.0
ppm

^{1}H, ^{13}C HMBC

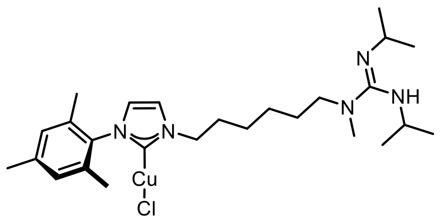

N
NH
N
N
N
Cu
Cl

7

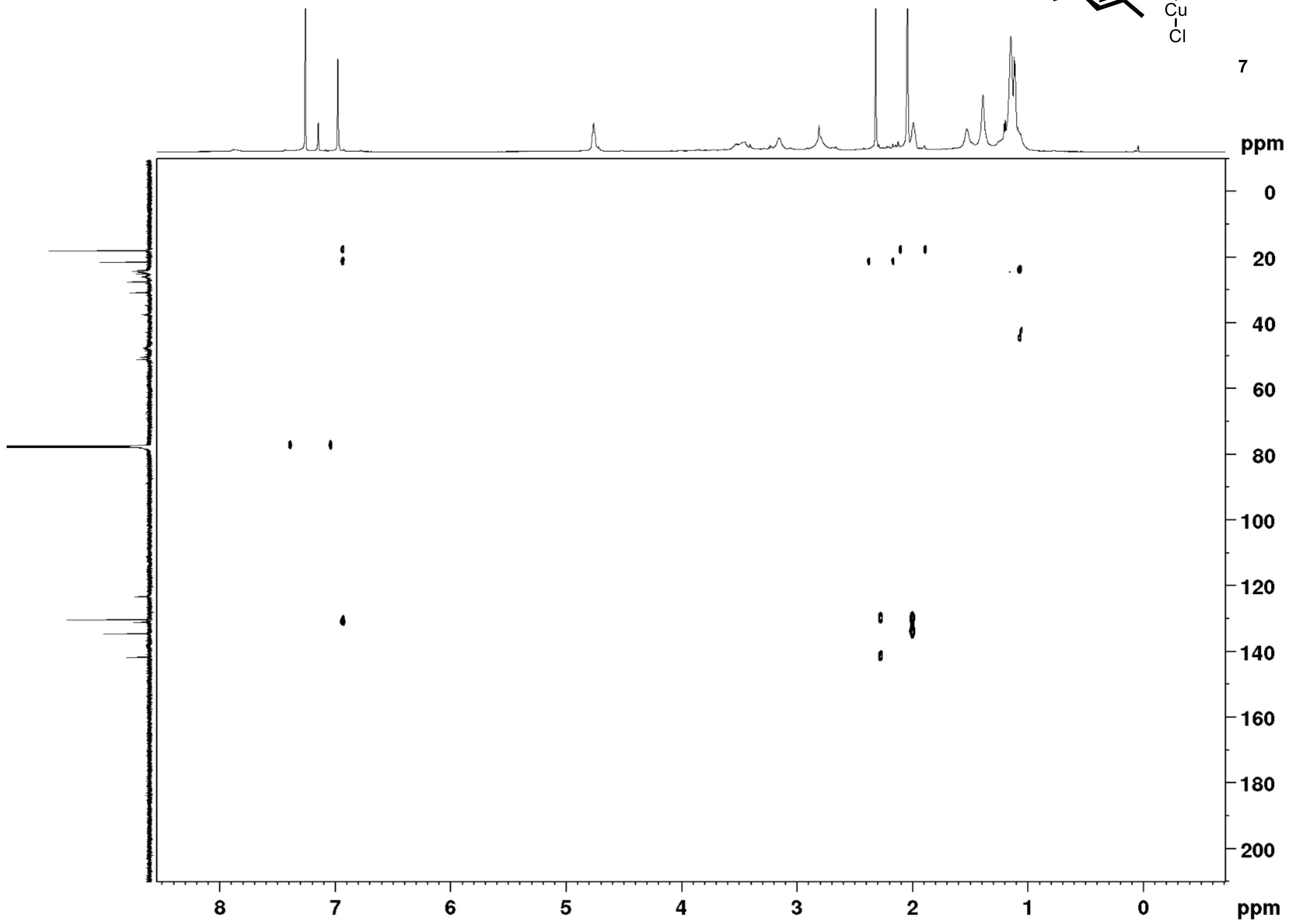

ppm
0
20
40
60
80
100
120
140
160
180
200
8
7
6
5
4
3
2
1
0
ppm

^{1}H, ^{13}C HSQC

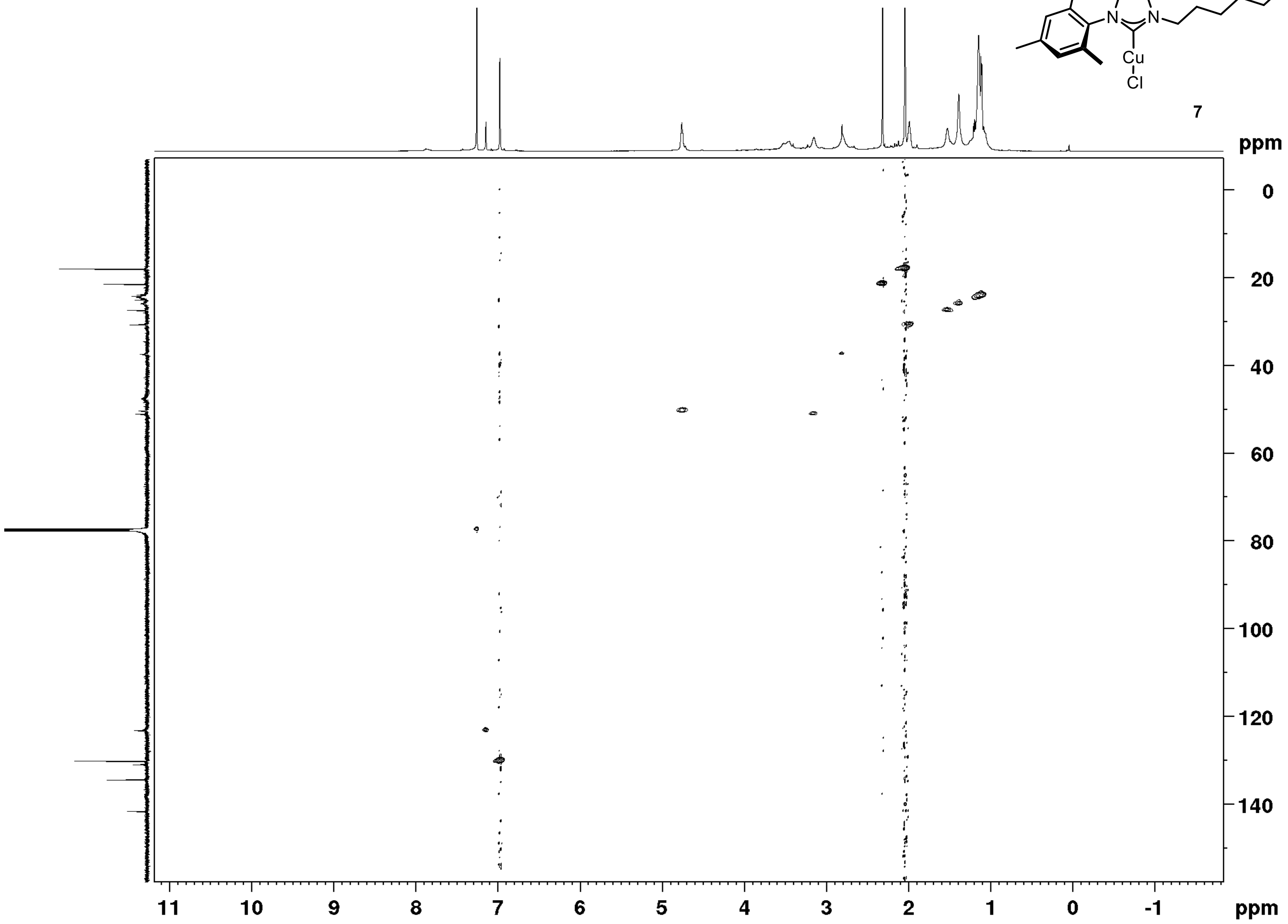

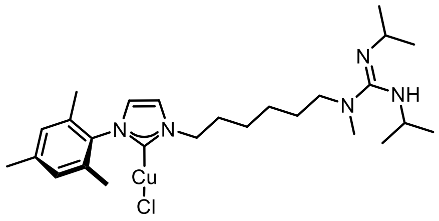

7

HRMS

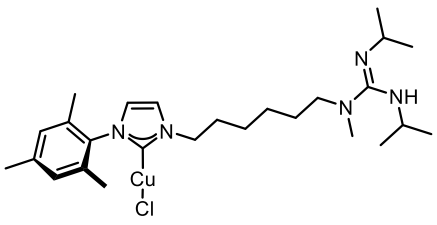
N
NH
N
N
N
Cu
Cl

7

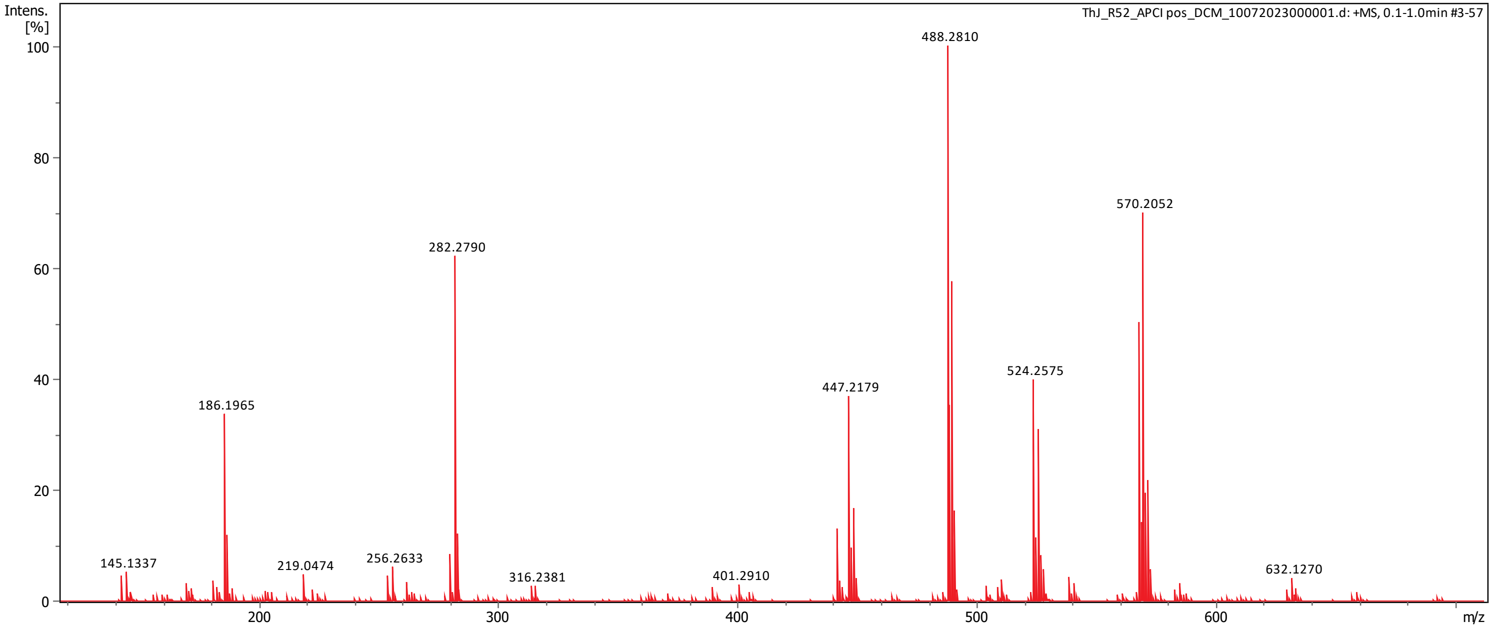
Intens.
[%]
ThJ_R52_APCI pos_DCM_10072023000001.d: +MS, 0.1-1.0min #3-57
100
80
60
40
20
0
488.2810
570.2052
282.2790
524.2575
447.2179
186.1965
145.1337
219.0474
256.2633
316.2381
401.2910
632.1270
200
300
400
500
600
m/z
